# Heterogeneity of Circulating Tumor Cell Neoplastic Subpopulations Outlined by Single-Cell Transcriptomics

**DOI:** 10.3390/cancers13194885

**Published:** 2021-09-29

**Authors:** Christine M. Pauken, Shelby Ray Kenney, Kathryn J. Brayer, Yan Guo, Ursa A. Brown-Glaberman, Dario Marchetti

**Affiliations:** 1 Division of Molecular Medicine, Department of Internal Medicine, University of New Mexico Health Sciences Center, Albuquerque, NM 87131, USA; cpauken@salud.unm.edu (C.M.P.); Ray.kenney@gmail.com (S.R.K.); kbrayer@salud.unm.edu (K.J.B.); yaguo@salud.unm.edu (Y.G.); 2 Division of Hematology/Oncology, University of New Mexico Comprehensive Cancer Center, Albuquerque, NM 87120, USA; ubrown-glaberman@salud.unm.edu

**Keywords:** circulating tumor cells (CTCs), metastatic breast cancer (mBC), lineage negative/lineage-positive (Lin−/Lin+) cell populations, CTC plasticity, epithelial cell adhesion molecule/cytokeratins (EpCAM/CKs), RareCyte, single-cell transcriptomics, 10x Genomics Chromium

## Abstract

**Simple Summary:**

Over 12% of women in the United States will be diagnosed with breast cancer in their lifetime. The overall 5-year survival rate for breast cancer is 90%, but the 5-year survival rate for women diagnosed with metastatic breast cancer is 28.1%. This study aims to characterize the cancerous cells that have left the primary tumor site and entered the blood, known as circulating tumor cells (CTCs). These cells could adhere to a site distant from the tumor and initiate metastasis. CTCs in breast cancer patients’ blood samples were enumerated and imaged. Cells from the blood were collected, RNA extracted, and the gene expression patterns of CTCs and other cell populations in the blood were investigated at the population and single cell level. This is a crucial step in characterizing CTCs as seeds of metastasis in breast cancer and for developing methods of detection to intercept metastasis before it localizes to distant regions of the body.

**Abstract:**

Fatal metastasis occurs when circulating tumor cells (CTCs) disperse through the blood to initiate a new tumor at specific sites distant from the primary tumor. CTCs have been classically defined as nucleated cells positive for epithelial cell adhesion molecule and select cytokeratins (EpCAM/CK/DAPI), while negative for the common lymphocyte marker CD45. The enumeration of CTCs allows an estimation of the overall metastatic burden in breast cancer patients, but challenges regarding CTC heterogeneity and metastatic propensities persist, and their decryption could improve therapies. CTCs from metastatic breast cancer (mBC) patients were captured using the RareCyte^TM^ Cytefinder II platform. The Lin− and Lin+ (CD45+) cell populations isolated from the blood of three of these mBC patients were analyzed by single-cell transcriptomic methods, which identified a variety of immune cell populations and a cluster of cells with a distinct gene expression signature, which includes both cells expressing EpCAM/CK (“classic” CTCs) and cells possessing an array of genes not previously associated with CTCs. This study put forward notions that the identification of these genes and their interactions will promote novel areas of analysis by dissecting properties underlying CTC survival, proliferation, and interaction with circulatory immune cells. It improves upon capabilities to measure and interfere with CTCs for impactful therapeutic interventions.

## 1. Introduction

Breast cancer is the most common cancer in women, accounting for 15% of all new cancer cases in the US. The 5-year survival rate for patients with localized breast cancer is 90%; however, this survival rate drops to 28.1% in patients diagnosed with metastatic breast cancer (mBC) [1]. Primary breast tumors are typed according to hormonal receptor status (estrogen receptor (ER), progesterone receptor (PR), and Erb-B2 receptor tyrosine kinase 2 (ERBB2 or HER2) amplification); and in some cases, the presence of specific mutations or changes in gene copy number also help to determine treatment. However, endocrine therapy fails to induce a response in up to 30% of patients with hormone receptor-positive mBC, and essentially all patients will eventually become refractory to endocrine therapy.

As the primary tumor grows, heterogeneous cell subpopulations with distinct gene expression signatures and biological characteristics arise. Some of these characteristics may modulate cellular adhesion and migration away from the tumor, as either a single cell or a cluster of cells. By intravasating into the bloodstream, cancer cells disperse throughout the body and can initiate a cascade of events, leading to fatal metastasis [2,3,4,5,6,7]. Extensive data obtained from clinical and preclinical models and cancer types have demonstrated the relevance of blood-borne cancer dissemination, and asserted that cell dissemination occurs early during tumor development [8,9]. Notably, circulating tumor cells (CTCs) directly captured from the peripheral blood of patients have emerged as a powerful biomarker for monitoring cancer progression [7,10,11,12]. Because CTCs express distinct and specific transcriptional profiles compared to primary or metastatic tumors, they offer the unique opportunity to analyze transcriptional information, which can be therapeutically useful [7,13,14,15]. Historically, CTCs have been defined as cells positive for epithelial-cell adhesion molecule (EpCAM), select cytokeratins (CK 8/18/19), and DAPI, while negative for the common lymphocytic marker CD45, which we term “classic” CTCs [13]. This definition has been applied by the US Food and Drug Administration (FDA)-cleared CellSearch^TM^ platform for the capture and enumeration of CTCs in clinical settings [16,17,18]. Quantification of CTCs has proven to be an independent prognostic indicator of progression-free/overall survival of metastatic cancer patients, including mBC [6,10,12]. Importantly, recent studies have identified new populations of breast cancer CTCs, which display phenotypic and gene expression profiles, which are distinct from “classic” CTCs [19,20]. However, populations of dedifferentiated CTCs, which are EpCAM-negative, CK-negative, or “stem like“ CTCs identified as CD44^high^/CD24^low^ cells, are excluded from CTC capturing by the CellSearch platform, but are considered to be intimately involved in metastatic [13,21,22] or tumor dormancy processes [23]. These concepts can be particularly relevant because transcriptional profiling performed on a CTC population does not recapitulate CTC transcriptional heterogeneity and plasticity [13]. This CTC plasticity can promote the abilities of some CTCs to survive in the circulation and to escape immune surveillance, causing chemo/radiotherapy resistance. Moreover, these characteristics can account for CTC adaptation to new microenvironments that CTCs encounter during the metastatic process and for minimal residual disease. 

While advances in CTC transcriptomic profiling have been significant, challenges remain. Most studies have been limited to the use of “classic” CTCs, with little attention directed towards the other CTC subsets. It is therefore critical to determine the continuum of circulating neoplastic cell subpopulations to better understand how these states may contribute to cancer progression and metastasis. We addressed these issues by devising an experimental strategy for capturing all CTCs, then performing not only standard RNA Sequencing (RNA-Seq), but also, notably, comprehensive and unbiased single-cell RNA sequencing analyses (scRNA-Seq; 10x Genomics Chromium) on blood samples collected from mBC patients. We report the complete interrogation of Lineage− and Lineage+ (Lin−/Lin+) cells [13] isolated from the blood of mBC patients by transcriptomic analyses at the single-cell level, revealing the presence of distinct circulating neoplastic cell populations. Moreover, we report the detection and classification of discrete circulatory Lin− cell subtypes whose gene expression patterns revise the classical model of CTCs, suggesting that the neoplastic cell capacity and metastatic competence ought to be ascertained across the entire spectrum of cell profile heterogeneity. 

## 2. Materials and Methods

### 2.1. Patients’ Blood Collection and Analyses

Patients diagnosed with metastatic breast cancer (mBC) undergoing active cancer treatment signed an informed consent to provide blood samples, per IRB-approved protocols (HRRC#15-507 and HRRC#19-513). The clinical parameters of each patient are shown in Table S1. Under strict aseptic conditions, peripheral blood samples (12–18 mL) were collected in sodium-EDTA tubes from mBC patients as part of their visits for routine monitoring appointments. Patients’ blood was collected at the times shown in Table S1. After collection, 7.5 mL of blood was transferred to an AccuCyte collection tube (RareCyte, Cat 24-1208-000, Seattle, WA, USA) for Cytefinder II^TM^ analyses, while the remainder of blood was processed to isolate peripheral blood mononuclear cells (PMBCs) for fluorescence activated cell sorting (FACS). RareCyte Cytefinder II analyses were performed on all patients and at indicated time points, allowing for longitudinal monitoring of CTC burden (detailed for each patient in Table S1). Patient samples 1, 2, and 3 were subjected to scRNA-Seq, while remaining patient samples were subjected to RNA-Seq.

### 2.2. Fluorescence Activated Cell Sorting (FACS)

FACS was performed to isolate Lineage-negative (Lin−) and Lineage-positive (Lin+) cell populations, as reported [13,23,24]. Briefly, red blood cells (RBCs) were lysed using RBC lysis buffer (BioLegend, Cat# 420302, San Diego, CA, USA), and washed twice using 1XPBS (VWR, Cat# E703-1L, Radnor, PA, USA), containing 5 mM EDTA (USB, Cat# 15694, Cleveland, OH, USA). The remaining peripheral blood mononuclear cells (PBMCs) were then counted using a Countess II automated cell counter (Thermo Fisher, Waltham, MA, USA). Cells were then blocked with Fc Block (BioLegend, Cat# 422302, San Diego, CA, USA), and labelled with FITC-conjugated human CD45, CD34, CD73, CD90, and CD105 (BioLegend, San Diego, CA, USA, Cat# 304038, 343504, 344016, 328108, and 323204, respectively) and Pacific Blue conjugated CD235 (BioLegend, San Diego, CA, USA, Cat# 306612). CD34 recognizes endothelial and human stem/pluripotent stem cells, CD73 and CD105 recognize endothelial cells, the CD73/CD90/CD105 triplet is the pathologically established biomarker for mesenchymal stem cells (CD90 also recognizes primitive stem cells). CD235 recognizes erythrocytes. Labelled cells were subsequently run on a Sony iCyt SY3200 cell sorter (San Jose, CA, USA). FITC-positive/DAPI-negative cells were collected into the Lin+ cell population, while FITC-negative/DAPI-negative cells were collected into the Lin− cell population. Cells labeled with CD235 (RBCs) or stained with DAPI (Thermo Fisher, Cat# D3571, Waltham, MA, USA) were sorted to waste. Accordingly, the FACS-selected Lin− cell population contained cells negative for above biomarkers, while the Lin+ fraction contained cells positive for these biomarkers [13,23,24]. 

### 2.3. RareCyte Cytefinder II™ Analysis

RareCyte CTC analyses were performed using protocols established by the manufacturer (RareCyte Inc., Seattle, WA, USA). This platform allows for the identification of CTCs and generates slides (8 per sample of 7.5 mL blood) to be used for CTC enumeration. Briefly, blood was centrifuged using AccuCyte sorting tubes to separate RBCs from nucleated cells. Further centrifugation collected the nucleated cell layer in a microcentrifuge tube containing cell isolation fluid (RareCyte, Seattle, WA, USA, 24-1090-002). Cells were suspended in AccuCyte CyteSpreader Transfer Fluid (RareCyte, Seattle, WA, USA, 42-1010-002), spread and dried on 8 slides, and the slides stored at −20 °C. Slides were then fixed, permeabilized, and stained using the Breast Cancer identification kit (RareCyte, Seattle, WA, USA, 0700-MA) which contains DAPI, which contains antibodies to human CD45 for the visualization of normal immune cells, and antibodies to human EpCAM and Pan-Cytokeratin for the detection of classical CTCs (EpCAM+/CK+/DAPI+ but CD45- cells). Slides were then imaged and analyzed using Cytefinder II software (RareCyte, Seattle, WA, USA). CTCs were visualized and enumerated by CyteMapper^TM^ software. Nuclei of CTCs are often larger than the nuclei of immune cells leading to a less intense DAPI staining. The number of CTCs is reported as CTCs/mL of blood.

### 2.4. RNA Isolation and Sequencing

RNA was isolated from 25–50 × 10^3^ cells from the Lin− and Lin+ fractions after FACS using a MicroRNA kit (Qiagen, Cat# 74004, Germantown, MD, USA). RNAs from matching Lin− and Lin+ patient samples were also compared with RNA from PBMC samples of normal healthy donors (negative controls). Synthesis of cDNA and library preparation were performed using the SMARTer Universal Low Input RNA kit for sequencing (Clontech, Cat# 634946, San Jose, CA, USA), and the Ion Plus Fragment Library kit (Thermo Fisher, Waltham, MA, USA, Cat# 4471252), as previously described [25,26,27]. Sequencing was performed using the Ion Proton S5/XL system (Thermo Fisher, Waltham, MA, USA) in the Analytical and Translational Genomics Shared Resource at the University of New Mexico Comprehensive Cancer Center.

### 2.5. RNA-Seq Analyses

Sequences were aligned using tmap (v5.10.11) to a BED file containing non-overlapping exons from UCSC genome hg38. Exon counts were calculated using HTSeq (v0.11.1) [28], and gene counts were generated by summing counts across exons. Samples were then normalized for library size using edgeR35 [29], and low expressing genes were excluded from the final analysis using a filtering threshold for at least 50 reads in a minimum of 3 samples. EdgeR and DESeq were used for principal component analysis [30]. EdgeR was also used for the differential expression (crosswise comparison of the three groups) using the glm method with an adjusted *p*-value of cutoff of 0.05 and requiring a minimum fold-change of 2. Differentially expressed genes were further analyzed using various R packages including clusterProfiler [31], topGO [32], GAGE [33], and pathview [34].

### 2.6. 10x Genomics Chromium Single-Cell RNA Sequencing

Following FACS, cell concentrations and viability were determined on the Cell Countess II and cell suspensions (Lin−/Lin+) were loaded into the Next GEM Chip G and Chromium Controller (10x Genomics, Pleasanton, CA, USA), per manufacturer’s protocol standardized for GEM formation and barcoding. The Chromium Next GEM Single Cell 3′ Reagent Kit v3.1 was used per the manufacturer’s protocol to complete first-strand and second strand cDNA synthesis. The same protocol was used to complete the 3′ Gene Expression Library. Library quality was assessed following cDNA synthesis and after completion of the 3′ Gene Expression Libraries on the Agilent Bioanalyzer employing a DNA High Sensitivity Chip (Agilent, Santa Clara, CA, USA). The KAPA Library Quantification Kit was used to determine final library concentrations. The 3′ libraries were sequenced by Illumina NovaSeq 6000 instrument on S4 Flow cells at the University of Colorado Anschutz Medical Campus’s Genomics Shared Resource Cancer Center.

### 2.7. Single-Cell RNA Transcriptome Analyses

Sequence data was demultiplexed and fastq files were generated using bcl2fastq (v2.20.0.422, Illumina, San Diego, CA, USA) with 37 parameter “--barcode-mismatches” set to 1. Fastq files were aligned and genes/cells were counted with Cell Ranger (v3.1.1, 10x Genomics) against the human reference genome (GRCh38) provided with CellRanger41 [35]. Cell Ranger-generated filtered files and Seurat (v3.2.3), an R package (v4.0.3), were both used for downstream analysis [36,37,38]. Cells expressing fewer than 200 genes, or more than 5000 genes and with mitochondrial gene expression content greater than 5%, were removed. Genes expressed in less than 10 cells were also removed. DoubletFinder was used to identify and remove doublet cells [39]. Data were transformed using SCTransform implemented in Seurat [40] with mitochondrial genes, number of features (nFeature_RNA), and differences between G2M and S cell cycle phase scores were regressed out. Data from all samples were integrated using Seurat’s standard integration workflow. Principle component (PC) analysis and an elbow plot were used to visualize the variance and select PCs encompassing ~80% of the variance prior to unsupervised clustering. Clusters were then determined using the FindNeighbors and FindClusters function with default parameters and a sufficiently high-resolution parameter to capture biological variability. Cell type prediction was performed using singleR (v1.4.0) against the human primary cell atlas database [41,42]. Differential gene expression was determined using the R package MAST [43] as implemented in Seurat. Significant genes were defined by average log fold-change ≥0.25, adjusted *p*-value ≤ 0.05, and expressed in >10% of the cells in the cluster. An additional “biomarker” level of stringency was defined as genes either having an average log fold-change ≥1.0 and in ≥30% of the cells and an adjusted *p*-value ≤ 0.01, or genes expressed in ≥90% of the cells in the cluster and ≤30% outside of the cluster. ClusterProfiler37 (v3.18.0) was used for pathway analysis, as previously reported [44].

## 3. Results

### 3.1. Isolation and Characterization of CTCs from mBC Patients by RareCyte Analyses

**Figure 1:**
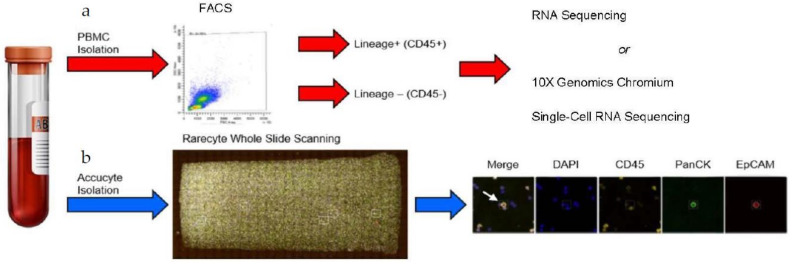
Flowchart depicting the strategy for selection and interrogation of Lin−/Lin+ cell populations from mBC patients. Patient blood samples were collected into sodium-EDTA tubes, and each sample was sub-divided. (**a**) One portion of the blood sample was processed for isolating Lin−/Lin+ cell populations via FACs, followed by RNA Sequencing or scRNA-Sequencing. See “Section 2” for details. (**b**) A 7.5 mL portion of the blood sample was transferred to an AccuCyte collection tube for RareCyte analysis.

The detection of CTCs as defined by the EpCAM+/CK+/DAPI+/CD45− expression pattern has been observed in the blood of metastatic cancer patients, including mBC, and their relevance affecting metastatic competence has been established [16,21,45,46,47,48]. However, the comprehensive and thorough portraits of CTC subsets detailing their distinct gene expression patterns at the single-cell transcriptomic level have not been reported to date. We have addressed this gap by devising a novel four-pronged workflow (Figure 1), which consists of: (1) using the RareCyte Cytefinder II platform to detect classically defined CTCs in blood of mBC patients; (2) isolating Lin−/Lin+ cell populations from blood of these patients by multi-parametric flow sorting; (3) evaluating populations of Lin−/Lin+ cells by RNA-Seq; and (4) comprehensively interrogating patient Lin−/Lin+ cell populations at the single-cell level using scRNA-Seq (10x Genomics Chromium). Thus, each blood sample had CTCs analyzed by two unique methods.

**Figure 2:**
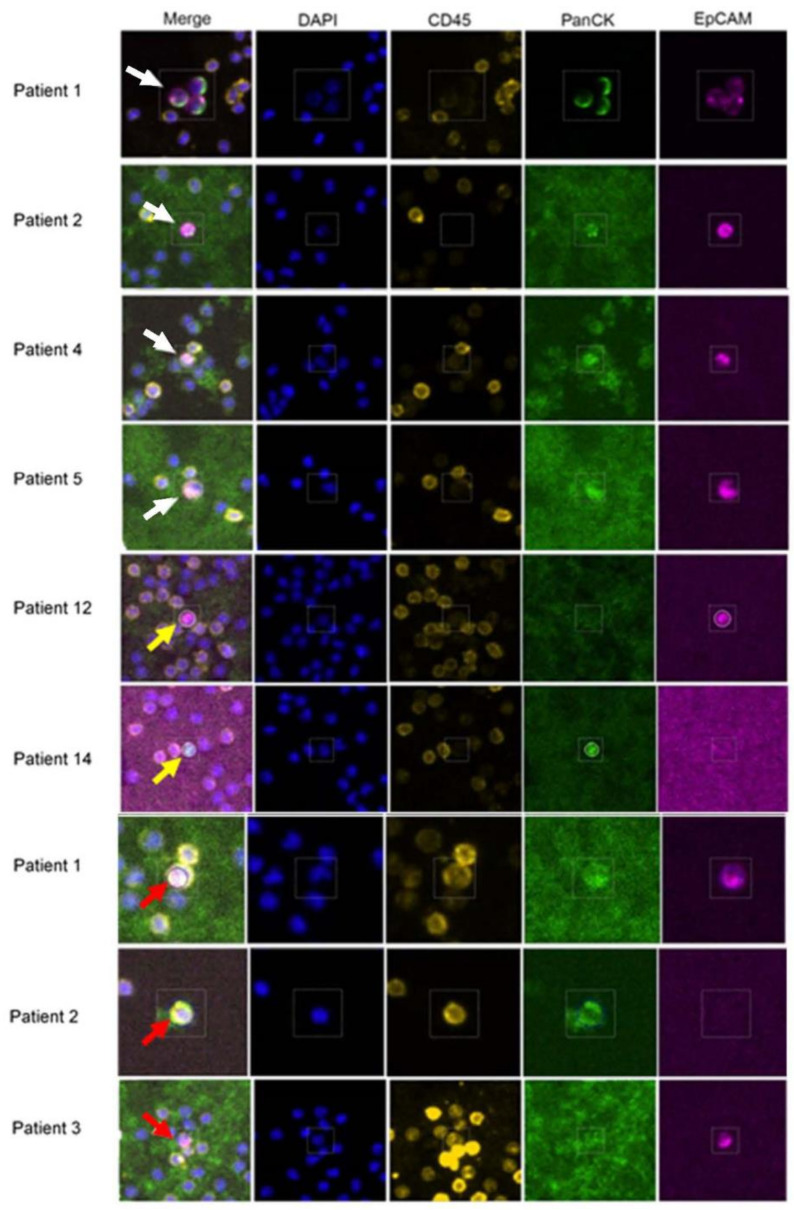
Immunofluorescence identification of CTCs from patients using the Cytefinder II platform. Cells were isolated from mBC blood samples as detailed in the methods. CTCs identified by EpCAM (magenta) and pan-cytokeratin (green) positive staining were visualized as single cells (patient 2), as associated with CD45+ cells (yellow) (patient 3, 4, 12, 14), or as multiple CTCs clustered together (patient 1, white arrow). CTCs marked with a yellow arrow are either CTCs that EpCAM+/CK− (patient 12) or EpCAM−/CK+ (patient 14). The CTCs marked with a red arrow are cells that are either EpCAM+/CK+/CD45+ (patient 1) or CK+/CD45+ (patient 2). Each CTC in a cluster was enumerated separately, and given a distinct cell identification number.

We implemented this strategy on a cohort of 21 mBC patients whose clinical parameters are listed in Table S1. The patients had various lengths of time as metastatic patients, including three who had surgery in the year preceding sample collection. The presence of classical CTCs was confirmed by the RareCyte Cytefinder II platform (Figure 1). The RareCyte platform uses blood from patients, removes most erythrocytes and platelets through centrifugation in the AccuCyte tube, spreads the remaining cells on eight slides, then identifies CTCs based on CD45−/EpCAM+/PanCK+/DAPI+ staining. This gentle, automated, and unbiased analysis of patient samples allows for the detection of single CTCs as well as clusters containing homogenous and/or heterogenous cell aggregates. Any immune cells within these clusters are labeled by CD45; however, associated platelet cells would not be labeled and have no DAPI signal. All patients involved in this study had detectable CTCs in one blood collection, at minimum (Table S1). Further, we performed multiple longitudinal blood collections from these patients to allow for the precise monitoring of patient overall tumor burden in relation to CTC numbers and CTC characteristics. Consistent with previous knowledge asserting that many factors can influence the number of CTCs in a liquid biopsy [17], we found variability in CTC numbers among patients and between each patient’s periodic collections (Table S1). CTCs were detected as single cells (Figure 2, patient 2), CTC clusters (Figure 2, patient 1), or as clusters with other cells (Figure 2, patients 3, 4, 12, 14). In many patients, we captured cells in which the expression of Pan-Cytokeratin (PanCK) (Figure 2, patient 12), or EpCAM (Figure 2, patient 14) was not detected. In rare cases, cells that were positive for all three antibodies (CD45+/EpCAM+/PanCK+/DAPI+ patient 1, red arrow), or CD45+/EpCAM-/PanCK+/DAPI+ (patient 1, red arrow), or CD45+/EpCAM+/PanCK-/DAPI+ (patient 1, red arrow) were identified. Total CTCs per patient analyzed are presented as number of cells/mL blood [18] (Table S1; the patient samples used for RNA-Seq and scRNA-Seq are italicized). The presence of CD45−/PanCK+/EpCAM− or CD45−/PanCK−/EpCAM+ is noted after the number of CTCs.

### 3.2. FACS Isolation of Patient Lin− and Lin+ Cell Populations and RNA-Seq Analyses

**Figure 3:**
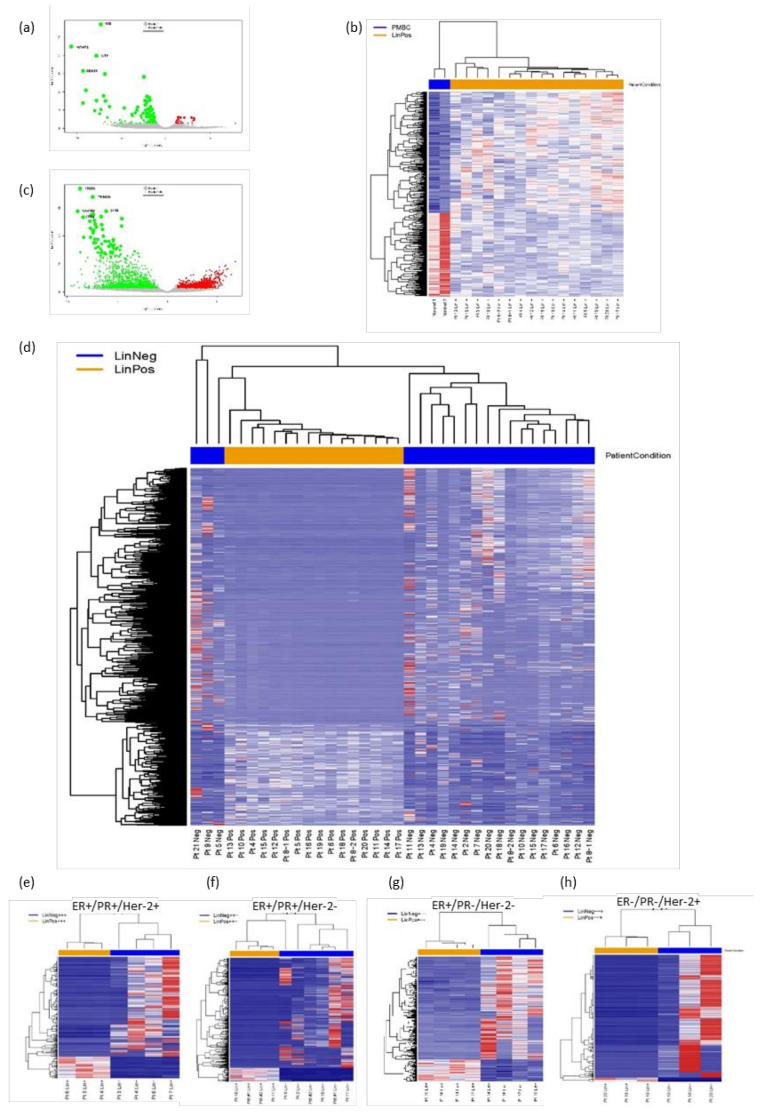
RNA-Seq of FACS Lin−/Lin+ cell populations. Blood was collected from normal human donors and 19 mBC patients. PBMCs were isolated and sorted as described, followed by RNA-Seq of collected cells. (**a**) A volcano graph showing genes with significant log2 fold change and −log10 (*p* value) for the normal PBMCs (green dots) in comparison with the Lin+ population from 15 mBC patients (red dots). (**b**) Heat maps of gene expression showing significant differences between normal human PBMCs (left/ blue) and Lin+ cells (right/ yellow) from patients. Patient 8 provided two samples 3 months apart. (**c**) A volcano graph showing genes with significant log2 fold change and −log10 (*p* value) for the Lin− populations (green dots) in comparison with the Lin+ population (red dots). (**d**) Heat maps of gene expression, indicating differences between the Lin+ (yellow) and Lin− (blue) populations from mBC patients. (**e**–**h**) Heat maps for patients grouped by hormone receptor status (shown above the map) (**e**) ER+/PR+/Her2+ (**f**) ER+/PR+/Her2− (**g**) ER+/PR−/Her2−, and (**h**) ER−/PR−/Her2+. Other subtypes had less than three patients in the overall set. All heat maps were done with unsupervised clustering.

Next, to detect gene expression differences between the immune cell population and the Lin− population containing the entirety of the CTCs, we performed FACS analyses of PBMCs to obtain Lin− and Lin+ cell populations, which were subsequently interrogated by RNA-Seq. Gene expression analysis detected 188 differentially expressed genes (DEGs) (log2FC > 1, *p* value ≤ 0.05) between PBMCs and Lin+ cells isolated from BC patients (Figure 3a). While overall similar, there was variability in gene expression levels between the Lin+ populations from patient samples analyzed as shown on the heat map (Figure 3b). Supplement File 1 (File S1) shows what genes are significant with large logFC, and a GSEA analysis of this data is shown on sheet 2 of File S1. Analyses of gene expression between Lin− and Lin+ cell populations demonstrates that the Lin− fraction had a number of genes with significantly increased expression levels (Figure 3c, green dots). These analyses demonstrated that there were a similar number of genes upregulated (1881) in the Lin+ cell populations as compared to those enhanced (1867) in the Lin− cell fraction. Furthermore, heat maps of gene expression showed differences between the Lin− and Lin+ populations, with a greater range of diversity between Lin− samples (Figure 3d; genes are listed in File S1, sheet 3). Some of the most significant differentially expressed genes in the Lin− cell population included CAVIN2, ITGB3, LY6G6F, TUBB1, LTBP1, and TRIM58. Some genes associated with epithelial cells such as EPCAM, TACSTD2, MUC2, KRT7, KRT8, KRT18, and KRT19 were detected in the Lin− DEG, while genes such as LTF and CTTN are specific to mammary tissue. Analysis of these genes showed enrichment of several pathways, including epithelial mesenchymal transition (EMT), apical junctions, estrogen response, and angiogenesis, as well as the KEGG pathways, ECM receptors interactions, and focal adhesion (File S1, sheet 4). Furthermore, GSEA analysis against the hallmark gene sets collection has a number of breast cancer associated gene sets being significantly enhanced (File S1, sheet 4). The Lin+ cell population was enriched in a wide variety of genes including PTPRC (CD45), CD3, CD4, CD8, CD27, and HEY1 indicative of an immune cell population. 

It may be that the Lin− fractions from patients with the same hormone receptor subtype have greater similarities than the entire compilation of Lin− fractions. Patients were grouped by hormone receptor subtypes (ER/PR/Her2), and heat maps were produced for those receptor subtypes for which there were more than three patient samples (Figure 3e–h). Even within a specific receptor subtype, significant differences between patients were detected. For example, some genes such as CAVIN2, CLU, ITGA2B, ITGB3, LTF, PTGS1, PTPRJ, LTBP1, TGFB1, and VNCL had enhanced expression in comparison to the Lin+ cell populations from the same patients in all groups, with many of these genes being involved in cell communication, adhesion, and migration. Thus, FACS separation into Lin+ and Lin− fractions, enriched for the epithelial/ mammary cells in the Lin− fraction.

### 3.3. Interrogation of the Lin− and Lin+ Populations by Single-Cell RNA Sequencing (scRNA-Seq)

**Figure 4:**
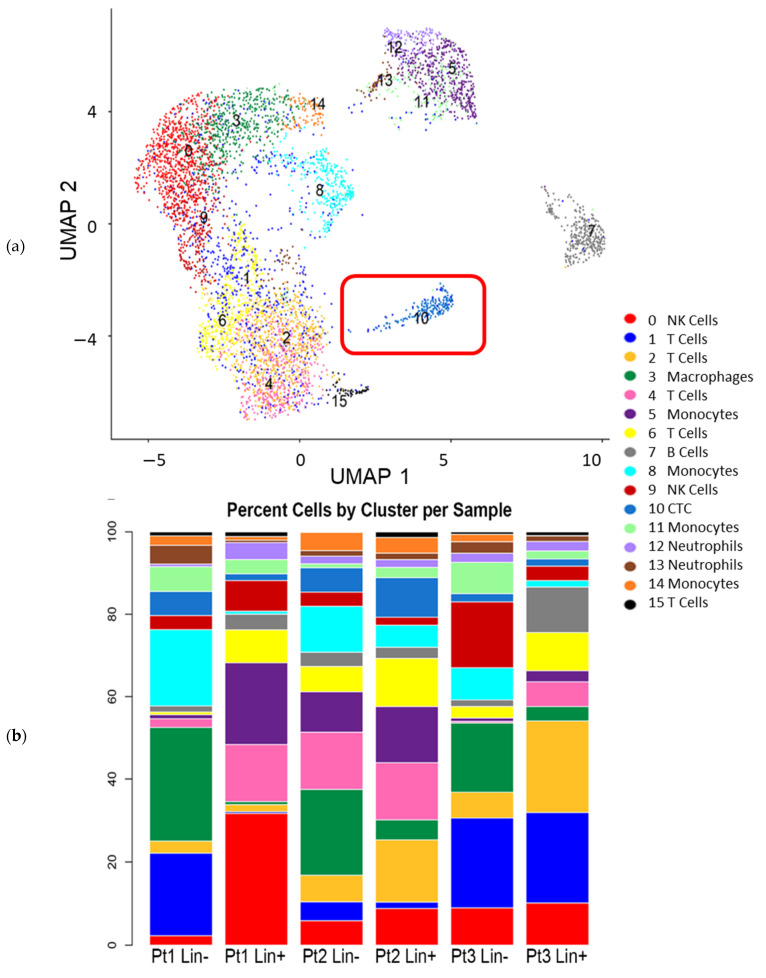
ScRNA-Seq analysis identified distinct cell populations in Lin− and Lin+ fractions. PBMCs from the three patients were sorted into Lin− and Lin+ fractions, and analyzed using Chromium single-cell transcriptomics platform (10x Genomics Chromium) (see “Materials and Methods” for details). (**a**) Cluster analysis of gene expression data of Lin− and Lin+ fractions from three mBC patients identified 16 distinct cell populations, which are displayed as UMAP plot. Cell type predictions are shown with clusters being identified as immune cells and cluster 10 (red box) identified as CTC/CSC cells (epithelial cells). (**b**) Stacked percentage bar graph showing the percentage of cell type per cluster and per sample analyzed with cluster identification being the same color as in (**a**).

The RNA-Seq data did not allow further characterization of changes of CTC gene expression within the Lin− cell population. Therefore, scRNA-Seq investigations were performed on Lin− and Lin+ cell populations from three mBC patients that had differing hormone receptor status and organ sites metastasis (Table S1). Immediately after sorting, cells from each Lin−/Lin+ fraction were loaded into the 10x Genomics Chromium system. After filtering for signal quality (between 200 and 5000 genes, less than 5% mitochondrial gene reads), a total of 5801 cells remained, ranging from 500 to 2115 cells per sample. Moreover, 10x Genomics Chromium clustering identified 16 distinct cell clusters in the integrated data (Figure 4). 

**Figure 5:**
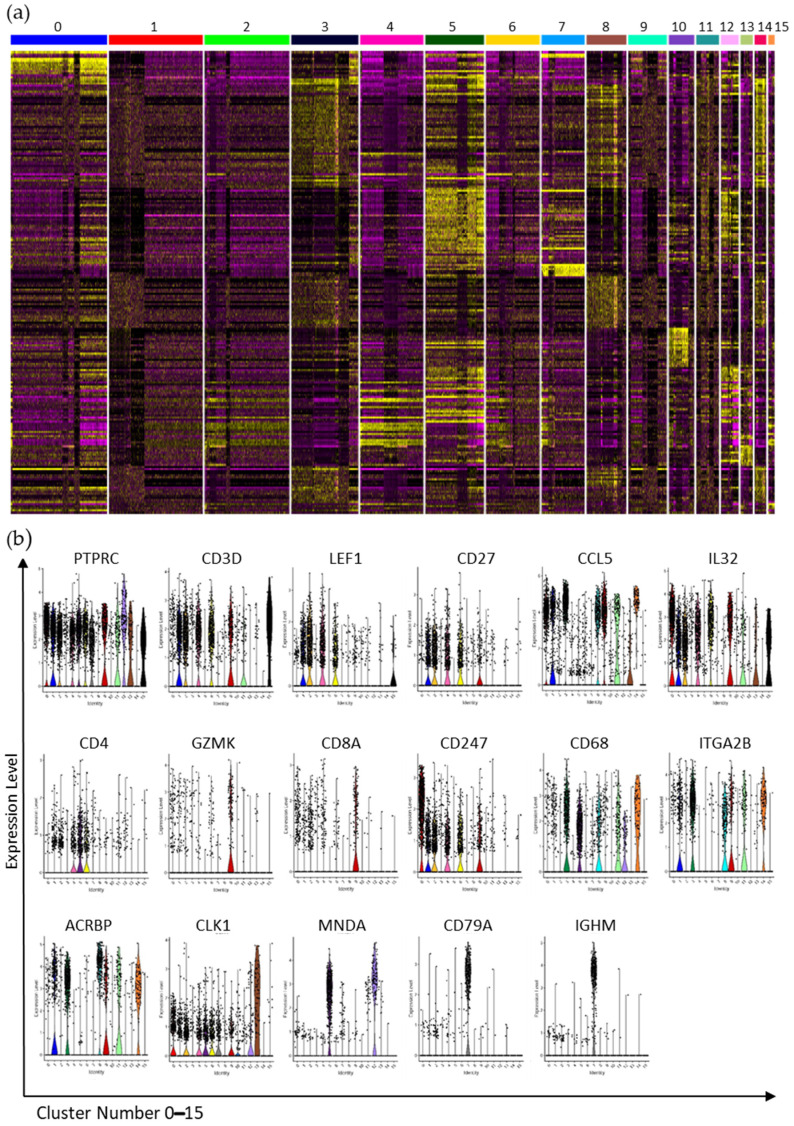
Gene expression of biomarkers in individual cells within each cluster for identification of genes of interest. (**a**) Heat map of gene expression in the 16 clusters determined by scRNA-Seq. Genes that were significantly expressed across samples and denoted as biomarkers were used for cluster identification. (**b**) Violin plots of some of the biomarker genes used for the identification of cell clusters.

Each cluster contained cells from every patient and fraction, although the percentage of cells in a particular cluster could vary between fractions (Figure 4b). For example, clusters 3, 8, 10, and 14 had a higher percentage of cells from Lin− than Lin+ cell populations while clusters 5 and 6 each contained a higher percentage of cells from Lin+ fractions. Distinct differences in percentages among patients were most striking for clusters 0, 1, 2, 4, 5 (Figure 4b). In particular, the Pt1 Lin+ sample contributed a high percentage of cells to cluster 0. Each cluster was compared to all other clusters to determine significantly different gene expression (*p* value ≤ 0.05), and this information was used to determine the cell type of each cluster. Biomarker status for any gene with an adjusted *p* value ≤ 0.01 in a cluster was based on one of two criteria: the first related to the extent of expression which required for the gene to be expressed in greater than 90% of the cells in that cluster, but in less than 30% of cells in all the other clusters. The second criteria centered on large fold-change (FC) in which the gene was expressed in more than 30% of the cells in that cluster with an average log2 fold change ≥1 for upregulated genes. Figure 5a shows the expression of the top 50 genes in all clusters and thus the uniqueness of each cluster. Figure 5b shows the violin plots of single genes across all clusters demonstrating that some genes are expressed to different extents in several clusters (e.g., PTPRC (CD45), CD3D, IL32, and CLK1), while some genes were highly expressed in one or two clusters (e.g., MNDA, CD79A, and IGHM).

Note that some genes such as IGHM and CD79A were primarily expressed in cluster 7 (Figure 5b). Conversely, other genes, such as CD3D, PTPRC, and TMA7, were expressed in a number of clusters (Figure 5b), but varied in both the percent of cells expressing that particular gene, and the level of expression in those cells. Notably, PTPRC, also known as CD45, and the primary determinant of FACS fractionation, was detectable in multiple clusters while genes corresponding to the other FACS antibodies were not detected (Figure 5b). PTPRC had varied levels of expression in the 16 clusters with highest levels in clusters 5, 9, and 12. Conversely, few cells expressed PTPRC in clusters 3, 8, 10, and 14 (Figure 5b). CD3D, a marker for T cells, was expressed in a high percentage of cells in clusters 1, 2, 4, 6, and 15, and in some cells of cluster 9 (Figure 5b). CD3E and CD3G were also expressed in similar patterns. LEF1, a transcription factor, and CD27, required for T cell maintenance, were expressed in clusters 1, 2, 4, and 6 with minimal expression of LEF1 in cluster 15 (Figure 5b). CD27 was also detected in cluster 9 while the cytokine CCL5 was expressed at high levels in clusters 0, 1, 3, 8, 9, 11, and in all cells of cluster 14, but it was not widely present in clusters 2, 4, 6, and 15. Clusters 0 and 6 were marked by high-expression levels of IL32 cytokine, with lower levels in clusters 1, 2, and 4, as well as in clusters 9 and 15. CD4 expression was most widely expressed in clusters 4, 5, 6, although not at very high levels. Activated T cells characterized by CD69 expression were primarily found in clusters 4 and 6, while cluster 14 was marked by the expression of SUB1. Altogether, 5 clusters, 1, 2, 4, 6, and 15 were considered T cell clusters, although some T cell gene markers were detectable in subsets of other scRNA-Seq clusters.

By decoding scRNA-Seq clusters beyond T cell subsets, we found that clusters 0 and 9 contained NK cells with high expression of NK genes such as CCL5 (Figure 5b), along with NKG7 and CTSW transcript detection in a high percent of cells in these clusters. Cluster 9 also expressed the NK marker GZMK in a high percentage of cells, with a subset of cells expressing CD8 (Figure 5b). Conversely, cluster 0 was more homogenous and unique in its expression of genes such as CD247 (also known as PDL1), NCAM, KLRC2, KLRD1 (all markers of NK cells), and TBX21 (a marker of T helper cells) (Figure 5b). Lastly, cluster 11 expressed CD68, CCL5, and several other transcripts found expressed extensively across multiple clusters. Cluster 5 was identified as a monocyte cell cluster with some T cells. Critical transcripts for its identification included CD4 (Figure 5b), and TYROBP (also in clusters 0 and 12), while MS4A7, CDKN1C, DEK, and MTDH transcripts were present uniquely in cluster 5. Clusters 12 and 13 were classified as neutrophils by CLK1 biomarker (Figure 5b), with CCNL1 and NAMPT significantly detected in all 3 clusters, while the presence of AIF1 and MNDA (Figure 5b), S100A8, and S100A9 was signifthat areicant in clusters 5 and 12, but not in cluster 13. However, cluster 13 included cell subsets expressing RUNX1 and TP53INP1, while cluster 12 possessed subsets of cells expressing FCGR3B and PTEN transcripts. Cluster 11 was considered a monocyte or dendritic cell cluster.

Further interrogation found that the B cell population comprised cluster 7, with many cells expressing various HLA-D genes as well as the CD19, CD37, and CD74 genes. Transcripts with a significant FC increase in cluster 7 included CD79A, CD79B, IGHM (Figure 5b), IGKC, and IGLC2. Cluster 1 expressed CD3D, CD3G, and IL7R suggesting a type of T cell. Over 50% of the cells in this cluster were from the Lin+ fraction (Pt3_LinP). Altogether, these data show that PTPRC-dominated clusters could be identified, although none of them was found to be homogeneous. Clusters 3, 8, 10, and 14 had few cells expressing PTPRC, and these clusters contained a higher-percentage of cells from Lin− samples. Cluster 3 had extensive expression of a number of “housekeeping genes” such as β-actin and HLA isoforms, but also ITG2B, TAGLN, CALM3, CCL5, TGFB1, and ITM2B, while less than 20% of the cells expressed PTPRC. We found that CDC37 gene expression was unique to cluster 3, while a subset of cells in this cluster expressed ARG2. Clusters 8 and 14 also expressed ITGA2B, although at a slightly lower levels in cluster 8 (Figure 5b). This cluster was marked by the extensive expression of ACRBP (Figure 5b) and NRGN, and high-level increases in MDM, ARG2, PTCRA, and NF2E gene levels, plus the uniquely expressed genes EGLN3, MEIS, and CTNNAL1, suggesting that cluster 8 is a monocyte/macrophage cluster. Further, all cells in cluster 14 expressed CCL5 (Figure 5b), along with high levels of ITGA2B, ACRBP, and CD68 (Figure 5b), suggesting a monocyte/macrophage identity. The expression of TGFβ1 in all 3 clusters, and the expression of ARG2 in cluster 8 and a subset of cluster 3 provided evidence that some of these macrophages belong to the M1 phenotype. Platelet specific genes were found in clusters 3, 5, 7, 9, and 12 suggesting there is a heterogeneity of platelet transcriptomes in response to the physiological conditions. GSEA analysis of the transcriptomic clusters shows a uniqueness in the molecular functions (GOMF), biological processes (GOBP), reactome pathways, oncogenic processes, immunological processes, and the TF regulatory processes that are enhanced in the various clusters (File S2). 

**Figure 6:**
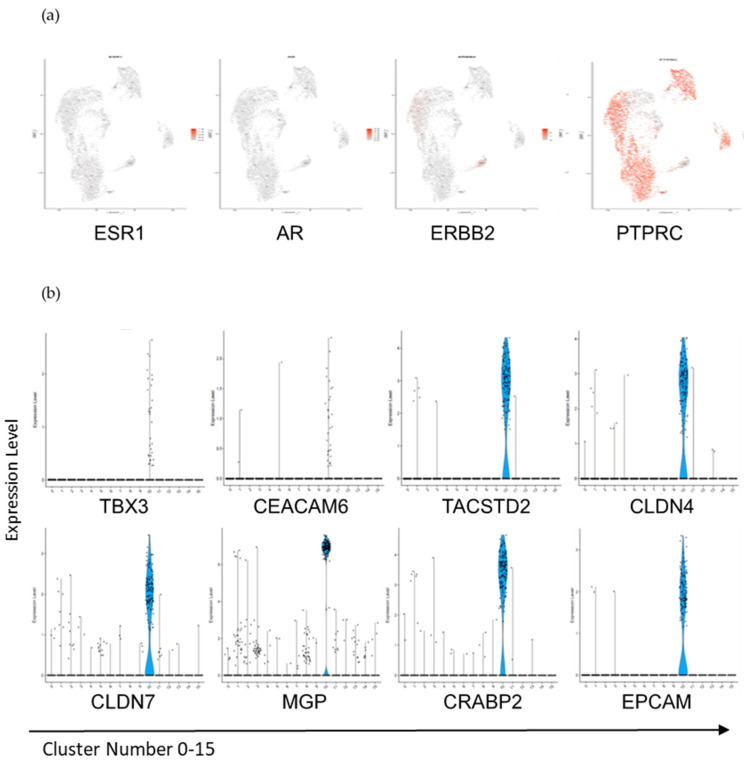
Significant gene expression within 10x Chromium cluster 10. (**a**) UMAPs displaying a differential gene expression of ER/PR/HER2 (ERBB2) and EpCAM identifying cluster 10 as non-immune cells. Few cells expressing these hormone-receptor genes were detected in cluster 10, however they were also expressed in other clusters. The scale of expression levels is shown for each gene. (**b**) Violin plots displaying genes of interest, which were significantly upregulated in cluster 10 when compared to other clusters. Claudin 4 (CLDN4) and Claudin 7 (CLDN7) while (TACSTD2), TBX3, and CEACAM6 were significantly expressed in cluster 10 (CTCs) with a minimal expression in the other clusters. Matrix Gla protein (MGP) and cellular retinoic acid binding protein 2 (CRABP2) were highly upregulated in cluster 10, with low-level expression in remaining clusters.

Cluster 10 then appears as a cluster of cells whose transcriptomic profiles are not associated with immune cells. Cluster 10 consisted of 201 cells from both the Lin− and Lin+ cell populations from each patient, with over 1800 genes that were highly significant (*p* value ≤ 0.01) (File S3). Genes known to be associated with breast tissue [15,19,22], such as ESR, AR, and ERBB2 (Figure 6a) were detected at low levels in clusters 10 as well as in other clusters. Genes associated with epithelial cells such as KRT8, 18, 19, and 7; as well as TBX3, TACSTD2, EPCAM, CLDN4, CLDN7, CEACAM6, and MGP (Figure 6b) were highly specific to cluster 10. Many of these genes were expressed in less than 75% of the cells in the cluster; however, those genes expressed in a subset of cells in cluster 10 were either undetected or expressed in fewer than 5% of cells in the other clusters, suggesting the presence of a significant phenotypic spectrum of cells in cluster 10. Most surprisingly, the cells from the Lin+ population in cluster 10 expressed PTPRC and some of these cells also expressed EPCAM, TACSTD2, or some of the CKs.

Keratins (KRTs, gene code for CKs) are intermediate filament proteins commonly found in epithelial cells, and the presence of CK 7, 8, 18, and 19 are often used in breast cancer diagnosis as well as key biomarkers in the detection of CTCs [17]. Notably, KRT7, 8, and 19 were expressed in 70% or more in cells of cluster 10, and in less than 3% of all other cells (File S3). The biomarker KRT18 was expressed in 79% of cells in cluster 10, and was detected in less than 20% of cells in cluster 8. Another keratin, KRT10, was found to be present in some of the cells of clusters 0, 10, and 12, and in a few cells of clusters 8 and 3. Moreover, EPCAM, a key biomarker defining classical CTCs [17], was also found to be unique to cluster 10, and expressed in 54% of cells, while its paralog, TACSTD2, was detected in 70% of these cells. EPCAM is known to interact with claudins 3, 4, and 7 (Figure 6), and tetraspanins, such as TSPAN1; genes that were extensively expressed in cluster 10 and known to be involved in cell adhesion events [2,3]. Other significant genes encoding proteins involved in cell adhesion were expressed in cluster 10, and included MGP, PERP, RAB25, DSP, EMP2, PHLDA2, ERBB2, CEACAM6, and CTNND1 (File S3). However, cadherins and integrins were not expressed in a significant percentage of cells of cluster 10, setting forward the notion that only a subset of cells of this important cluster has the ability to fully adhere to distant organ sites as disseminated cancer cells. Several genes encoded proteins implicated in the response to calcium levels (TACSTD2, SRI, S100A16, S100A14, and CRACR2B). The most significant signaling proteins were encoded by genes such as CRABP2 (Figure 6b), CAMK2N1, PLK2, RHOV, SPINT1, RALA, and RERG (File S3). Additionally, some genes coding for proteins known to be responsive to estrogen were detected, including CITED1, CITED4, GATA3, KRT19, CCND1, and IGFBP2, however ESR1 and ESR2 were each only detected once in cluster 10. Critical cell proliferation genes, e.g., CCND1, ANAPC11, and CDKN1A were found in cluster 10, along with other genes with proliferative roles, e.g., TGFB1, ILK, BRK1, CIB1, AREG, and HES1.

We conducted an extensive interrogation of genes for transcription factors (TF) [49], oncogenes [50], EMT [51,52], or cancer stem cells (CSC) [53,54,55,56], detected in individual cells of cluster 10 (see heat maps on individual sheets in File S3); individual cells of cluster 10 from each patient are shown on the top of the chart, each gene is indicated along the left side, and the level of expression of each gene is scaled for that gene). A high-level perusal of the data showed that cells from Lin+ cell populations of each patient were more similar in their expression patterns than the Lin− cells. For example, ETS1 was only found in Lin+ cells while ARID4B and IKZF1 were extensively expressed in the Lin+ fraction of patients 1 and 3; and ELF1, KLF13, ARRB2, REL, and ZEB2 were most striking in patient 1 with patchy expression in Lin+ fractions of the other patients. Second, the expression of transcription modulators was more patient specific in Lin− fractions of cluster 10. Patient 3 expressed none of the widely expressed TF with the exception of TSC22D1, while patient 1 expressed many TF extensively, however only few TFs were detected in all cells of the Lin− fraction. Expression of TSC22D1, MAX, and LYL1, coincident with a lower level of expression of the more ubiquitous TFs may mark a subset of CTCs in patient 1. TF expression in patient 2 was even more patchwork, and at a lower level for some of the ubiquitous genes such as JUN, JUNB, KLF2, and ELF (File S3, sheet 1). These differing TF patterns could set-up differential responses and cellular behaviors, although it is not clear which TF are critical to modulate cell survival, organotropism, proliferation or other responses to the microenvironment.

A number of TF are known oncogenes, e.g., ELF3, ERBB2, ERBB3, GATA3, SOX2, SOX9, FOXA1, CCND1, while others act as tumor suppressors, e.g., ATM, ARID1A, CBL, CDKN2A, TBX3, and EGR1 [50]. The level of expression of oncogenes and tumor suppressors in the individual cells of cluster 10 are shown in File S3 sheet 2. Similar to TF expression, there were several oncogenes expressed at high levels in nearly all of cluster 10 cells. There was also a greater similarity of expression patterns of these genes between the Lin+ fractions while a greater heterogeneity was observed in Lin− fractions: patient 1 was marked by extensive expression of EPCAM, ELF3, ID3, GATA3, TPM3, DDX5, PLK2, YWHAE, EZR, LMNA, CCND1, KIF5B, and TPM4, patient 2 by a decreased level of expression overall with fewer genes having extensive expression (ELF3, ID3, PLK2, and EPCAM), and patient 3 was characterized by expression of TPM3, TPM4, CCND1, and NCOA4. Some of the genes in Lin+ fractions with the most striking differential expression included PTPRC, LTB, CXCR4, SAMHD1, CD74, RNF213, and LCK, while additional genes were extensively expressed only in Lin+ cells from patient 1 (File S3, sheet 2).

The comparison of genes expressed in cluster 10 cells with an extensive listing of genes involved in EMT [51,52,54,56] showed intriguing patterns of expression (File S3 sheet 3). The Lin+ fractions from each patient had enhanced gene expression at the mesenchymal end of the spectrum. Conversely, a variable level of gene expression at the epithelial end of the spectrum was detected, from almost none (patient 3) to higher levels in patient 2 as compared to patient 1. In Lin− cell populations, cluster 10 cells from patient 3 expressed primarily mesenchymal genes, patient 2 had low but extensive expression of epithelial genes, and patient 1 possessed high levels of expression of some epithelial genes with low levels of expression for only a few mesenchymal genes. Epithelial genes included KRT7, 8, 18, and 19 as well as CLDN 4 and 7, EPCAM, TACSTD2, ERBB3, ERBB2, JUP, CD24, while CDH1 was not detected. The mesenchymal genes included PTPRC, VIM, EMP3, SRGN, TUBA1A, CXCR4, GIMAP4, while GNG11, SPARC, and MYL9 marked a subset of cells from the Lin+ fractions of patients 1 and 3, suggesting a more mesenchymal attribution. 

Cancer stem cells (CSCs) are often proposed to be quiescent but capable of responding to changes in the cell’s microenvironment. This quiescence property allows these cells to often avoid the effects of chemotherapeutic agents, and then later be capable to initiate proliferation and the establishment of a metastatic tumor. The debate about which genes are indicative of CSCs is still vigorous, therefore we aggregated gene lists from several sources [53,55]. As with the other gene panels, cells from Lin+ cell populations possessed a greater similarity than ones from Lin− fractions (File S3, sheet 4). One of the most striking differences in Lin+ cells was the lack of expression of MGP, the gene with the largest log2FC in cluster 10 (File S2), in any of the 10x cluster cells from patient 3 (File S3, sheet 4). There were also genes (SNRPB2, ATRX, STK17A, TAX1BP1, VCAN, OXLD1, BRD7, HMGB2, and APLP2) with roles in chromatin remodeling or signaling which were extensively expressed in Lin+ cells (patient 1), but not in the other two patients. In Lin− fractions, there was a lower level of expression of genes in patient 3, while patient 2 expressed more extensively many of these genes, however still at lower levels. The Lin− cells from patient 1 fell into two subpopulations: a population expressing high levels of these CSC genes in both Lin+/Lin− fractions, and a population with lower levels of expression and fewer genes expressed, appearing similar to the Lin− cells from patient 2. Further examination showed that these cells are also ones with lower levels of expression of many TF, except for TSC22D1, MAX, and LYL1. They are also ones with low epithelial gene expression, except for F13A1, SRGN, SPARC, and GNG11.

Collectively, cells of cluster 10 expressed a variety of genes promoting essential biological processes: these cells can thus have potential adhesive, proliferative, and migratory properties, which are fundamental for metastatic competence.

## 4. Discussion

Breast cancer is the most common non-cutaneous cancer in women and the second leading cause of cancer-related death in the U.S. As such, improved understandings of progression from early stage to metastatic disease is critical. While the genotype and phenotype of breast cancer have been well studied, the extensive characterization of CTC subsets and their properties as a transitional circulatory state between primary and metastatic tumors is less well understood. Here, we report for the first time the comprehensive interrogation of Lin− and Lin+ cell populations isolated directly from the blood of mBC patients [13]; this experimental strategy results in the transcriptomic analysis and molecular classification of Lin− cell populations at single-cell level.

In this study, we employed a strategy using cutting-edge technologies to discriminate CTC subsets and differentiate them from normal PBMC cells. Patients enrolled in this study were diagnosed with distinct breast cancer subtypes, and at various stages of relapse and treatment (Table S1); however, CTCs were consistently detected from these patient samples using the RareCyte system. The number of classically annotated CTCs usually varied between 3 and 136 cells in approximately 1 mL of blood, although one patient did have one blood sample with greater than 15,000 CTC/ mL. This is in line with previous reports using CellSearch or other CTC platforms, where the variability in CTC number over time is known to be induced by a number of factors and biological conditions [17,21,37,57]. Baseline blood collections examined by Cytefinder II identified patients 1–3 as candidates for scRNA-Seq, as they had high numbers of CTCs (Table S1) as well as differing hormone receptor status and metastatic sites. At the latest collection (month 6), patient 1 had an unexpectedly high CTC count (>10E4/mL), which was confirmed after staining a second RareCyte slide. This high CTC count was in agreement with clinical parameters since this patient experienced significant systemic disease progression between collection 1 and 2 (baseline and month 3, respectfully), including the development of brain metastasis, which could explain the rapid rise of CTCs [13].

Considering established notions of the prognostic relevance of classically defined CTCs in mBC [10,11], we sought to interrogate gene expression profiles of circulatory neoplastic cells, including but also extending this definition. We employed FACS to separate hematopoietic Lin+ from Lin− cells [13]. RNA-Seq was performed to elucidate gene expression differences between patient-derived Lin+/Li− cell populations. The comparison of Lin+ cells to normal PBMCs from healthy donors indicated an altered gene expression profile in Lin+ cells from mBC patients. This gene expression pattern did not significantly differ among patient samples, and possibly indicates the activation of Lin+ cells, either as a direct response to mBC onset, or as result of chemo/radiotherapy regimens applied to these patients. Conversely, comparisons between patient Lin− and Lin+ cell populations resulted in a number of genes indicative of human mammary tissue, as well as genes linked to cell adhesion, cell junctions, and response to various stimuli pathways. Even grouping the patients by hormone receptor status shows variations in gene expression in the Lin− populations. These data show that we have been successful isolating mBC CTCs within the Lin− fraction. However, the percentage of CTCs in the Lin− population is still not high enough to definitively examine the transcriptional patterns in the CTCs.

Although RNA-Seq is a useful tool for assessing changes in gene expression at the population level, this technology does not fully explore individual differences between cells, which may contribute to cell survival, adhesion, migration, and proliferation, leading to metastasis. Accordingly, we performed single-cell RNA sequencing using feature barcoding technology (scRNA-Seq; 10x Genomics Chromium) to further classify all cells within the Lin− and Lin+ cell populations on a cell-by-cell basis. Our scRNA-Seq identified 16 distinct clusters from the Lin− and Lin+ cell populations from the three patients analyzed. Of critical relevance to the objective of these investigations, we identified cluster 10 as CTCs based on epithelial/mammary tissue gene expression and in synergy with the classification of the 15 other clusters identified as T cells, NK cells, B cells, neutrophils, and monocytes (Figure 5 andFigure 6). Many cell types, such as platelets, were detected in multiple clusters. This heterogeneity in platelet transcriptomes has been reported in other cancers and could be indicative of the role of platelets in the dissemination of CTCs [58]. It should be noted that we detected a number of genes which are known to be related to immune cell activation and inflammatory response (CCL5, IL32, GZMK), signifying a snapshot of active immune response at the time of these analyses. Notably, many genes implicated in cell adhesion (EPCAM, CEACAM6), survival (VEGFB, IGFBP2, TGF, CCND1), and cell-cell communication (TACSTD2, CRABP2), were significantly present in cluster 10 vs. the other clusters. These findings expand the classical definition of CTCs, and provide valuable insights on how some CTCs survive while in the circulation: these genes not only allow for the activation of survival signals, but also may improve CTC communications with immune cells, allowing CTC immune evasion until implantation at secondary target sites. Unexpectedly, we discovered Lin+ cells from patients 1 and 2, which expressed EpCAM and/or TACSTD2, as well as several KRTs in addition to CD45 expression. CD45+ cells expressing EpCAM and some KRTs have been detailed in work from other labs [20]. The finding of such cells in the scRNA-Seq data led us to reexamine the RareCyte slides from these patients and we verified that such cells were also detected by this antibody-based technology. Further examination of the scRNA-Seq data for the cells in Cluster 10 details the transcription factors and oncogenes whose expression is enhanced in these cells. TFs such as ETS1, ELF1, ARRB2, REL, and ZEB2 mark CD45+ cells while LYL1, MAX, and TSC22D may mark a subset of CTCs. The oncogenes, such as CCND3, PNRC1, LTB, JAK1, RAC2, and CXCR4 mark the CD45+ cells and KIF5BTPM4, and NCOA4 may mark subsets of CD45− CTCs. These changes in gene expression lead to further changes in gene expression in those genes involved in EMT processes and in modulating behaviors of CSCs. Further work could address the differences between patients, and whether the subpopulation seen with the Pt1 Lin− population is indicative of changes in the cell’s ability to proliferate, or adhere to specific anatomical sites. These findings substantiate concepts of CTC biomarker heterogeneity and are either evidence of or the result of cancer cell/immune-cell interactions, and/or gene regulation crosstalk, leading to a similar gene expression between the two cell types. A significant number of CTCs are also possibly quiescent [13], thus evading chemo/radiotherapies. CTC quiescence and its reversibility can be a larger contributing factor to metastasis than previously thought, and an important area to evaluate for therapeutic targeting. 

This represents the first report, which examined single-cell transcriptomes of all individual circulating neoplastic cells isolated from patients with mBC. Although of significance as the basis for future advances in this area, there are some limitations to this study. A small cohort of patients was analyzed, which is not representative of the entire mBC spectrum and certainly not representative of CTCs shed from primary BC tumors over its lifetime. Second, there was significant heterogeneity with regard to ER/PR/HER2 status and line of therapy. Either factor alone could contribute to gene expression differences among Lin− or Lin+ cells. Further, gene expression differences in CTCs may not translate to differences in protein levels and protein functionalities. It is also not clear whether adherence to other cells in a cluster in the blood modulates gene and/ protein activity. Third, the sort efficiency of the Lin− cell population varied between 30–70%, with some contamination of Lin+ cells as a technical artifact caused by the scarcity of Lin− cells, compared to the high number of total cells, which were sorted as the Lin+ cell population. This drastic difference may have skewed the Lin−/Lin+ ratio of cells being sequenced, especially in the RNA-Seq experiments, which can mask other significant differences in gene expression. However, this technical issue would not affect the scRNA-Seq, as in this technique, the assignment of a cell to a specific cluster is based on its entire transcriptomic profile and not its source.

## 5. Conclusions

These studies provide first-time evidence of a comprehensive analysis for distinct circulatory cell populations in blood from patients with mBC at the single-cell level, identifying distinct immune cell clusters, and describing their gene characteristics, and identifying cluster 10 as containing cells of epithelial origin, that is, CTCs. These cell populations possessed significant differences in gene expression at the single-cell level. This is especially evident in cluster 10 where analysis shows subpopulations that express a variety of TFs, oncogenes and genes critical to EMT and stem cell populations. Of relevance, by describing these differences, conclusions of this study revise the classical notion of what a CTC is, suggesting that the neoplastic cell capacity is distributed across heterogeneous cell profiles in circulation. It will be highly instrumental to replace rigid definitions (presence/absence of EpCAM, presence/absence of EMT or CSC markers, etc.) with more flexible, solid, if experimentally challenging, notions on how a CTC functions. We foresee that the extension of these findings, and/or their application in pre-clinical models to interrogate the precise functionality and metastatic potency of circulatory neoplastic states will significantly aid towards the formulation of more efficacious therapies in breast cancer.

## Data Availability

The authors declare that all 10x Genomics Chromium data supporting manuscript findings are available from the Gene Expression Omnibus as GSE174463: https://urldefense.com/v3/__https://www.ncbi.nlm.nih.gov/geo/query/acc.cgi?acc=GSE174463__;!!KXH1hvEXyw!N30RRwlTdFQHzW1Vhk8cAjM-ec9SKN-fSgFPTEeuDVyW-5uzQNLEoYQm0wonGl0Ol1mpNA (accessed on 21 August 2021).

## Supplementary Materials

The following are available online at https://www.mdpi.com/article/10.3390/cancers13194885/s1, Table S1: Clinical Parameters of Patients used in this study. File S1. Differentially expressed genes between populations by RNA-Seq. The first sheet lists the DEG between Lin+ and normal PBMCs and sheet 2 shows the most significant pathways as done by GSEA analysis. Sheet 3 shows differentially expressed genes from the Lin− and Lin+ fractions. The fourth sheet shows the results of GSEA analysis for the DEG from sheet 3 using the cell markers, KEGG pathways, and GO Ontologies databases. File S2. Pathway analysis of all scRNA-Seq clusters. Sheets showing analysis of significant Gene ontology MF, GO BP, oncogenic, immunologic, Regulatory Targets and reactome databases to show the differences between the clusters. File S3. Cluster 10 Analysis. A listing of the most significant genes of cluster 10 cells, which is, these genes have an adjusted p value ≤ 0.01. Genes were sorted by pct.1, which is the percent of cells in cluster 10 expressing the gene (first sheet). The expression level of that gene in all other clusters combined is listed under pct.2 and the dDR in the difference between these two values. The second sheet shows the sorting of genes by average logFC, e.g., a positive number indicates expression upregulation of that gene in cluster 10. The mean in cluster column shows the average level of expression of the specific gene across all cluster 10 cells, while the mean out of cluster is the average level of expression across all cells outside of that cluster. The DiffMean column indicates the differences in those averages. The analysis sheets show Gene Ontology Biological Processes (BP) and KEGG Pathways found to be significantly enhanced using these genes on the DAVID analysis website (https://david.ncifcrf.gov/home.jsp) (accessed on 26 May 2021) [59]. Figure S1. Profiles of selected gene expression in each cell of cluster 10. (a) Expression of transcription factors from http://humantfs.ccbr.utoronto.ca/ (accessed 13 April 2021) in each cell from cluster 10 [36]. Patient number and Lin−/ Lin+ fractions are listed at top; gene name is listed on left. Genes are sorted by the number of cells in cluster 10 in which the expression of the specific gene is detected and genes expressed in less than 5 cells are not listed. (b) Expression of oncogenes from https://www.oncokb.org/cancerGenes [50] (accessed 13 April 2021) in each cell of cluster 10. (c) Gene expression from a more extensive list of tumor-specific genes involved in EMT [51,52]. Epithelial-associated genes are shown in teal while mesenchymal-related genes are indicated in pink. Genes detected in less than 20 cells were removed from the list. PTGIS and WWTR1 were the only mesenchymal genes solely detected in cluster 10. Most of epithelial genes were only detected in cluster 10; however, SPINT2 and RAB11FIP1 genes were expressed extensively, and several other genes were expressed in multiple clusters. (d) Expression of cluster 10 genes implicated in cancer cell stemness [53,54,55,56]. Genes were sorted by the number of cells in cluster 10 expressing that gene.
