# Supplementary material for: Heterogeneity of Circulating Tumor Cell Neoplastic Subpopulations Outlined by Single-Cell Transcriptomics
Source: Cancers (Basel). 2021 Sep 29;13(19):4885. doi: 10.3390/cancers13194885 (PMC8508335; doi:10.3390/cancers13194885)
Supplement: Supplementary file 1 [file cancers-13-04885-s001.zip › cancers-1367663-supplementary.pdf]

Lin Pos

Gene ID	log 2Fold Change (edgeR)	pValue (edgeR)	pAdj (edgeR)
U2AF1	7.86	0.0002	0.0401
CXCL2	3.23	0.0000	0.0051
CCL3	3.20	0.0000	0.0016
LOC102724	3.11	0.0000	0.0072
CCR2	2.98	0.0001	0.0265
CXCL1	2.91	0.0000	0.0011
CCL3L3	2.50	0.0001	0.0340
HSPA6	2.11	0.0000	0.0011
LRRC37A4P	1.96	0.0002	0.0407
MFSD14B	1.95	0.0001	0.0306
ABHD16B	1.85	0.0001	0.0336
TLR8	1.77	0.0000	0.0123
COPB2	1.70	0.0000	0.0011
CX3CR1	1.68	0.0001	0.0310
PRF1	1.68	0.0002	0.0423
MAT2A	1.52	0.0000	0.0106
CYBC1	1.51	0.0000	0.0144
DCAF7	1.51	0.0000	0.0123
GIMAP6	1.47	0.0002	0.0401
IL6R	1.42	0.0000	0.0034
SIGLEC9	1.40	0.0001	0.0308
WSB1	1.40	0.0000	0.0009
BRD8	1.39	0.0001	0.0341
LTB4R	1.34	0.0000	0.0055
FAM217B	1.30	0.0001	0.0324
FAM78A	1.28	0.0001	0.0341
ARHGAP27	1.27	0.0001	0.0205
PISD	1.22	0.0000	0.0102
LARP4B	1.20	0.0001	0.0293
CTDSP1	1.17	0.0002	0.0401
DGCR2	1.14	0.0001	0.0330
ZSWIM6	1.14	0.0000	0.0159

PBMC

Gene ID	log2FoldChange(edgeR)	pValue(edgeR)	pAdj(edgeR)
RPS4Y1	-10.61	0.0000	0.0000
CTAG1B	-9.29	0.0000	0.0000
DDX3Y	-9.29	0.0000	0.0000
TXLNGY	-8.96	0.0000	0.0000
EIF1AY	-7.97	0.0000	0.0000
UTY	-7.80	0.0000	0.0000
HBA2	-7.76	0.0000	0.0000
TTTY15	-7.41	0.0000	0.0001
HBB	-7.28	0.0000	0.0000
ZFY	-7.25	0.0000	0.0000
PRKY	-6.98	0.0000	0.0000
LINC00278	-6.82	0.0001	0.0164
HBA1	-6.81	0.0000	0.0000
USP9Y	-6.76	0.0000	0.0000
LOC105377225	-6.56	0.0000	0.0011
KDM5D	-6.30	0.0000	0.0000
LINC01784	-6.16	0.0000	0.0159
BCORP1	-5.74	0.0001	0.0256
CELP	-5.66	0.0001	0.0301
LOC105373978	-5.16	0.0001	0.0330
ACTRT2	-5.14	0.0002	0.0397
CYS1	-5.13	0.0000	0.0054
LOC105372219	-5.01	0.0001	0.0282
MYL7	-5.00	0.0001	0.0324
NKX2-1-AS1	-4.92	0.0000	0.0123
LOC105379303	-4.81	0.0002	0.0458
LOC107985976	-4.64	0.0000	0.0000
LHX1-DT	-4.60	0.0000	0.0138
KCNG4	-4.55	0.0000	0.0106
PRSS22	-4.45	0.0002	0.0456
LOC105378226	-4.10	0.0002	0.0379
FAM183A	-3.96	0.0002	0.0373
PNPLA5	-3.95	0.0002	0.0364
MMP2	-3.93	0.0002	0.0365
SMIM1	-3.90	0.0003	0.0499
PTGES	-3.88	0.0000	0.0142
LOC105376995	-3.79	0.0000	0.0001
CPT1C	-3.78	0.0001	0.0205
SLC4A1	-3.71	0.0000	0.0004
LOC105372711	-3.70	0.0003	0.0501
SLCO5A1	-3.41	0.0000	0.0001
GRM6	-3.34	0.0002	0.0382
LRAT	-3.25	0.0002	0.0454
LOC102723604	-3.22	0.0000	0.0000

MOK	-3.14	0.0000	0.0000
LOC107987026	-2.96	0.0002	0.0390
C15orf48	-2.82	0.0001	0.0324
CTTN	-2.76	0.0000	0.0004
SNCA	-2.69	0.0000	0.0017
LOC101927051	-2.55	0.0000	0.0001
TUBB1	-2.46	0.0000	0.0052
MIR3175	-2.45	0.0003	0.0501
ADAM11	-2.44	0.0000	0.0101
IRGQ	-2.43	0.0000	0.0000
COL6A1	-2.35	0.0001	0.0348
H1-4	-2.30	0.0000	0.0000
RPS16P5	-2.27	0.0000	0.0000
CSF1	-2.27	0.0000	0.0000
ADORA2A	-2.21	0.0000	0.0009
LOC105374775	-2.19	0.0001	0.0162
CFAP45	-2.18	0.0000	0.0001
ITGB3	-2.17	0.0001	0.0265
IGSF9	-2.15	0.0000	0.0009
TLCD2	-2.13	0.0001	0.0265
WHAMM	-2.10	0.0000	0.0000
SESN2	-2.10	0.0000	0.0004
H1-2	-2.09	0.0000	0.0000
NXT1	-2.05	0.0001	0.0265
CDC42BPG	-2.04	0.0000	0.0001
H2AC20	-2.01	0.0000	0.0138
H1-3	-1.99	0.0000	0.0000
NANOS3	-1.98	0.0000	0.0101
UBL5	-1.96	0.0000	0.0004
GRPEL1	-1.94	0.0000	0.0007
PIGA	-1.94	0.0000	0.0000
IL1RN	-1.93	0.0001	0.0256
ADORA2A-AS1	-1.90	0.0001	0.0193
GBAP1	-1.89	0.0002	0.0429
RAB5IF	-1.89	0.0002	0.0382
KLF10	-1.86	0.0000	0.0001
PIM2	-1.83	0.0000	0.0000
LOC105370877	-1.83	0.0000	0.0004
H2AC8	-1.83	0.0001	0.0306
SLC7A5	-1.81	0.0000	0.0142
SH3D21	-1.77	0.0000	0.0073
GLB1L	-1.76	0.0001	0.0301
HIF1A	-1.76	0.0000	0.0011
SERTAD2	-1.75	0.0000	0.0000
ZC3H12A	-1.74	0.0000	0.0020
H1-10	-1.73	0.0000	0.0008
ICAM1	-1.71	0.0002	0.0361

ZNF324	-1.70	0.0000	0.0000
RPL37A	-1.69	0.0000	0.0159
IER5	-1.69	0.0000	0.0000
LINC01578	-1.67	0.0000	0.0000
PELI1	-1.66	0.0000	0.0055
CFAP20	-1.59	0.0000	0.0138
SPATA21	-1.58	0.0002	0.0485
SLC35A2	-1.58	0.0000	0.0003
MRPL55	-1.56	0.0000	0.0125
LOC101927441	-1.56	0.0000	0.0138
PSMC1	-1.54	0.0001	0.0264
MIR22HG	-1.53	0.0000	0.0087
PLK3	-1.52	0.0000	0.0037
MAP1A	-1.50	0.0000	0.0138
MYADM	-1.50	0.0000	0.0004
CHD4	-1.49	0.0000	0.0000
IRAK2	-1.49	0.0002	0.0486
FUZ	-1.47	0.0002	0.0482
CLP1	-1.47	0.0002	0.0361
SPATA2	-1.47	0.0000	0.0006
SNHG7	-1.46	0.0002	0.0442
SF3B5	-1.46	0.0000	0.0073
CIR1	-1.46	0.0000	0.0090
ZBTB10	-1.44	0.0000	0.0025
GADD45B	-1.44	0.0003	0.0496
SBDS	-1.41	0.0000	0.0103
RILPL2	-1.40	0.0001	0.0205
FBXO33	-1.39	0.0000	0.0051
ELL	-1.39	0.0000	0.0087
NFKBIE	-1.38	0.0002	0.0482
GTF2B	-1.38	0.0000	0.0040
PHF13	-1.37	0.0000	0.0087
CCDC85C	-1.36	0.0001	0.0213
MTX1	-1.36	0.0000	0.0142
IKZF5	-1.36	0.0000	0.0027
ZFAND2A	-1.34	0.0000	0.0011
IRF2BP2	-1.32	0.0000	0.0001
PPP1R15B	-1.32	0.0000	0.0060
CREB3	-1.30	0.0000	0.0012
ZFAS1	-1.29	0.0000	0.0101
PI4K2A	-1.29	0.0000	0.0057
YOD1	-1.28	0.0000	0.0023
SUCO	-1.28	0.0000	0.0052
RLF	-1.27	0.0000	0.0006
HEXIM1	-1.25	0.0000	0.0087
ZNF326	-1.25	0.0000	0.0130
YPEL5	-1.25	0.0001	0.0174

C1orf52	-1.22	0.0000	0.0055
WDR48	-1.21	0.0000	0.0014
SYF2	-1.20	0.0001	0.0265
WBP11	-1.19	0.0000	0.0130
C9orf78	-1.18	0.0001	0.0306
CDKN2D	-1.18	0.0002	0.0485
KLHL15	-1.17	0.0002	0.0401
DLST	-1.14	0.0000	0.0143
TMEM167B	-1.14	0.0001	0.0162
CHMP4B	-1.14	0.0000	0.0148
YTHDC1	-1.12	0.0001	0.0213
NECAP1	-1.12	0.0002	0.0460
VAMP2	-1.10	0.0001	0.0219
CDK11B	-1.07	0.0001	0.0293
BIN3	-1.05	0.0002	0.0380
TRA2A	-1.01	0.0001	0.0341
TNIP1	-1.01	0.0001	0.0243
SMU1	-0.97	0.0002	0.0401

pathway	SYMBOL	pval
GOBP_CELL_ACTIVATION	A1BG|ADA|SIGLEC	1.00E-10
GOBP_CELL_ACTIVATION_INVOLVED_IN_IMMUNE_RESPONSE	A1BG|ADA|SIGLEC	1.00E-10
GOBP_CYTOKINE_PRODUCTION	MIR675|CDH3|MIR	1.00E-10
GOBP_DEFENSE_RESPONSE	ADA|MIR675|SIGLI	1.00E-10
GOBP_IMMUNE_EFFECTOR_PROCESS	A1BG|ADA|SIGLEC	1.00E-10
GOBP_INFLAMMATORY_RESPONSE	ADA|MIR675|MIR9	1.00E-10
GOBP_LEUKOCYTE_MEDIATED_IMMUNITY	A1BG|SIGLEC14|C1	1.00E-10
GOBP_MYELOID_LEUKOCYTE_ACTIVATION	A1BG|SIGLEC14|TC	1.00E-10
GOBP_REGULATION_OF_IMMUNE_RESPONSE	ADA|ABI1|BTNL10	1.58E-10
GOBP_NEGATIVE_REGULATION_OF_IMMUNE_SYSTEM_PROCE	ADA|PARP3|MICA|	1.81E-10
GOBP_REGULATION_OF_IMMUNE_SYSTEM_PROCESS	ADA|ABI1|BTNL10	1.88E-10
GOBP_CELLULAR_LIPID_METABOLIC_PROCESS	ACOT8|PLA2G4B|P	2.28E-10
GOBP_RESPONSE_TO_BIOTIC_STIMULUS	MIR675|SIGLEC14|	7.10E-10
GOBP_SMALL_MOLECULE_METABOLIC_PROCESS	ADA|NAALAD2|MI	3.09E-09
GOBP_CYTOKINE_MEDIATED_SIGNALING_PATHWAY	CD24|SH2B3|NR1H	1.69E-08
GOBP_REGULATION_OF_RESPONSE_TO_EXTERNAL_STIMULUS	ADA|MIR892B|MIR	7.80E-08
GOBP_LEUKOCYTE_MIGRATION	ADA|CD300H|KLRC	7.91E-08
GOBP_LIPID_BIOSYNTHETIC_PROCESS	ACOT8|PLA2G4B|P	1.19E-07
GOBP_POSITIVE_REGULATION_OF_MULTICELLULAR_ORGANISI	ADA|AKT3|MIR675	1.28E-07
GOBP_NEGATIVE_REGULATION_OF_RESPONSE_TO_EXTERNAL_	ADA|MIR892B|MIR	1.58E-07
GOBP_NEGATIVE_REGULATION_OF_IMMUNE_RESPONSE	PARP3|MICA|NR1H	5.17E-07
GOBP_ORGANOPHOSPHATE_METABOLIC_PROCESS	ADA|MIR675|ACOT	5.37E-07
GOBP_LEUKOCYTE_DIFFERENTIATION	ADA|HDAC5|C17or	5.68E-07
GOBP_MONONUCLEAR_CELL_MIGRATION	KLRC4-KLRK1|ADAI	5.67E-07
GOBP_MYELOID_LEUKOCYTE_MIGRATION	CD300H|DNM1L|A	6.70E-07
KEGG_CYTOKINE_CYTOKINE_RECEPTOR_INTERACTION	CCL26|TNFSF13|HC	1.32E-07
KEGG_JAK_STAT_SIGNALING_PATHWAY	STAT3|STAT4|STAT	7.72E-06
KEGG_CHEMOKINE_SIGNALING_PATHWAY	CCL26|STAT3|STAT	8.22E-06
REACTOME_SIGNALING_BY_INTERLEUKINS	CRLF1|RALA|NOS2	1.68E-08
REACTOME_SIGNALING_BY_GPCR	WNT16|CAMKK1|C	1.42E-07
REACTOME_CLASS_A_1_RHODOPSIN_LIKE_RECEPTORS	TAC1|CX3CL1|TBXA	1.94E-07
REACTOME_INTERLEUKIN_10_SIGNALING	TNFRSF1B|TNFRSF1	5.04E-06
REACTOME_IMMUNOREGULATORY_INTERACTIONS_BETWEEN_	CD99|ITGAL|TYROI	6.71E-05
GSE10325_BCELL_VS_MYELOID_DN	HTRA1|ACTG1|ICA	1.00E-10
GSE10325_CD4_TCELL_VS_MYELOID_DN	LY86|NCF1C|BASP1	1.00E-10
GSE10325_LUPUS_BCELL_VS_LUPUS_MYELOID_DN	MAPKAPK3|FCGRT	1.00E-10
GSE10325_LUPUS_CD4_TCELL_VS_LUPUS_MYELOID_DN	COQ2|PISD|GCA|P	1.00E-10
GSE11057_CD4_CENT_MEM_VS_PBMC_DN	JUP|CD9|ZDHHC7|	1.00E-10
GSE11057_PBMC_VS_MEM_CD4_TCELL_UP	BMP2K|SLC7A7|TB	1.00E-10
GSE22886_DAY0_VS_DAY1_MONOCYTE_IN_CULTURE_UP	RPS4X|KLF2|RERE|	1.00E-10
GSE22886_NAIVE_CD4_TCELL_VS_MONOCYTE_DN	CSTA|LAT2|IFI30|H	1.00E-10

GSE22886_NAIVE_CD8_TCELL_VS_MONOCYTE_DN	FCER1G|CHST15|IF	1.00E-10
GSE22886_NAIVE_TCELL_VS_MONOCYTE_DN	KCNMB1|PTGS1|LY	1.00E-10
GSE24634_TEFF_VS_TCONV_DAY3_IN_CULTURE_DN	CCR5|LY96|HLA-DF	1.00E-10
GSE26495_NAIVE_VS_PD1HIGH_CD8_TCELL_DN	RAP1GAP2|EOMES	1.00E-10
GSE26495_NAIVE_VS_PD1LOW_CD8_TCELL_DN	EOMES|S100A4|RA	1.00E-10
GSE29615_CTRL_VS_DAY3_LAIV_IFLU_VACCINE_PBMC_DN	LCN12|CASP6|NR5	1.00E-10
GSE29618_MONOCYTE_VS_MDC_DAY7_FLU_VACCINE_UP	CD14|SCPEP1|C5A	1.00E-10
GSE29618_MONOCYTE_VS_PDC_DAY7_FLU_VACCINE_UP	ALOX5|WDFY3|CSF	1.00E-10
GSE34156_TLR1_TLR2_LIGAND_VS_NOD2_AND_TLR1_TLR2_LI	RAB7A|RRBP1|CHS	1.00E-10
GSE34156_UNTREATED_VS_24H_NOD2_LIGAND_TREATED_MC	ARHGEF2|WDFY3|	1.00E-10
GSE34156_UNTREATED_VS_6H_TLR1_TLR2_LIGAND_TREATED_	SULF2|VCAN|NCF4	1.00E-10
GSE37416_0H_VS_6H_F_TULARENSIS_LVS_NEUTROPHIL_UP	GAA|USP6|FCGR3E	1.00E-10
GSE45365_NK_CELL_VS_CD8_TCELL_MCMV_INFECTION_UP	ECHS1|CAPRIN2|LC	1.00E-10
GSE9988_ANTI_TREM1_VS_CTRL_TREATED_MONOCYTES_DN	FAM89B|PTTG1IP|	1.00E-10
HARALAMBIEVA_PBMC_M_M_R_II_AGE_11_22YO_VACCINATE	VSIG4|GGTA1|TRE	1.00E-10
HAY_BONE_MARROW_NEUTROPHIL	S100A9|S100A8|S1	1.00E-10
HAY_BONE_MARROW_NK_CELLS	GNLY|NKG7|KLRF1	1.00E-10
HOEK_NK_CELL_2011_2012_TIV_3D_VS_0DY_ADULT_3D_DN	SAMD11|RBP7|PA	1.00E-10
HOWARD_PBMC_INACT_MONOV_INFLUENZA_A_INDONESIA_(	FBXO6|MNDA|ADA	1.00E-10
HU_FETAL_RETINA_MICROGLIA	AIF1|TYROBP|LAPT	1.00E-10
GSE34156_NOD2_LIGAND_VS_TLR1_TLR2_LIGAND_6H_TREAT	SAT1|LINC01016|Z	2.14E-10
GSE24634_TREG_VS_TCONV_POST_DAY10_IL4_CONVERSION_	FGL2|LGR4|APBA2	2.70E-10
GSE22886_NAIVE_CD4_TCELL_VS_DC_DN	VAMP8|TM9SF1|N	2.87E-10
GSE24634_TEFF_VS_TCONV_DAY10_IN_CULTURE_DN	CLIP4|HSPA6|ADA	3.13E-10
GSE9988_ANTI_TREM1_AND_LPS_VS_CTRL_TREATED_MONOC	FAM89B|ARL6IP5|	3.54E-10
GSE9988_LOW_LPS_VS_CTRL_TREATED_MONOCYTE_DN	PHF23|CTDSP2|FB	5.32E-10
GSE29618_BCELL_VS_MONOCYTE_DN	GMFG|SDCBP|RXR	7.20E-10
GSE21670_STAT3_KO_VS_WT_CD4_TCELL_TGFB_IL6_TREATED	PTPRJ|RAP2B|TSC2	7.28E-10
GSE22886_NAIVE_BCELL_VS_MONOCYTE_DN	CTBP2|MYO1F|PKN	9.97E-10
KAZMIN_PBMC_P_FALCIPARUM_RTSS_AS01_AGE_UNKNOWN_	ARL4C|PRF1|XCL2|	1.12E-09
GSE9988_ANTI_TREM1_AND_LPS_VS_VEHICLE_TREATED_MON	RGS19|NT5DC2|GA	1.23E-09
GSE29617_CTRL_VS_DAY3_TIV_FLU_VACCINE_PBMC_2008_DN	MRPL51|MTCH2|N	2.04E-09
GSE9988_LOW_LPS_VS_VEHICLE_TREATED_MONOCYTE_DN	RGS19|CXCR4|GRN	2.15E-09
GSE9988_ANTI_TREM1_VS_VEHICLE_TREATED_MONOCYTES_D	RGS19|ARRB1|HCK	3.44E-09
GSE3565_CTRL_VS_LPS_INJECTED_DUSP1_KO_SPLENOCYTES_U	SMPDL3B|CXCR6|E	3.88E-09
GSE22886_NAIVE_TCELL_VS_DC_DN	TST|MAN2B1|GCL	7.33E-09
GSE29618_BCELL_VS_MDC_DN	TUBA1B|NDRG2|R	7.60E-09
GSE4984_LPS_VS_VEHICLE_CTRL_TREATED_DC_DN	TOX|SH3TC1|MYO	8.73E-09
GSE15767_MED_VS_SCS_MAC_LN_UP	RAB38|NFIC|CDS1	1.17E-08
GSE2935_UV_INACTIVATED_VS_LIVE_SENDAI_VIRUS_INF_MAC	RGS1|ZDHHC2|SM	1.32E-08
GSE9988_LPS_VS_VEHICLE_TREATED_MONOCYTE_DN	RGS19|CXCR4|CLIC	1.57E-08
GSE11057_NAIVE_CD4_VS_PBMC_CD4_TCELL_DN	RHOU|PLSCR1|QSC	1.63E-08
GSE29618_PDC_VS_MDC_DN	LY86|ASAP1|PILRA	1.73E-08
GSE22886_NAIVE_BCELL_VS_NEUTROPHIL_DN	MIR22HG|CD300A	2.30E-08
GSE26343_WT_VS_NFAT5_KO_MACROPHAGE_LPS_STIM_UP	SDHAF2|HSD17B6|	2.80E-08
GSE22886_NAIVE_CD8_TCELL_VS_DC_DN	M6PR|NPTN|FABP	2.90E-08
GSE29618_MONOCYTE_VS_MDC_UP	ASAH1|SLC7A7|ITG	3.40E-08

GSE3982_EFF_MEMORY_CD4_TCELL_VS_NKCELL_DN	LAT2|AFDN-DT|SEC	3.49E-08
GSE9006_HEALTHY_VS_TYPE_1_DIABETES_PBMC_AT_DX_DN	EGR3|CCR1|IL1B|P	3.99E-08
JAATINEN_HEMATOPOIETIC_STEM_CELL_DN	SECTM1|ITGB2-AS1	1.08E-07
GSE36888_STAT5_AB_KNOCKIN_VS_WT_TCELL_IL2_TREATED_	GCHFR|ING3|AC02	1.11E-07
GSE29618_PDC_VS_MDC_DAY7_FLU_VACCINE_DN	ATP1B1|CLIC2|SEC	1.23E-07
GSE22935_WT_VS_MYD88_KO_MACROPHAGE_UP	MOB1A|ZFP36L2|I	1.47E-07
GSE29618_MONOCYTE_VS_PDC_UP	PELI1|TLE4|RIN2|T	2.41E-07
GSE45382_UNTREATED_VS_TGFB_TREATED_MACROPHAGES_U	DUSP23|FHL3|ITPR	2.59E-07
HAY_BONE_MARROW_MONOCYTE	LST1|FCGR3A|AIF1	2.77E-07
GSE15330_HSC_VS_LYMPHOID_PRIMED_MULTIPOTENT_PROG	LMNB1|LAMC1|CH	6.62E-07
GSE22886_NAIVE_TCELL_VS_NEUTROPHIL_DN	IL1B|RUBCNL|H4C8	7.78E-07

padj	NES	size	Enrichment	PMBC_LINP_genes
2.69E-08	1.864	1002	Up-regulated	GNLY, NKG7, KLRF1, CTSW, PRF1, KLRI
2.69E-08	2.023	581	Up-regulated	GCK, SBDS, CYB5R3, GATA1, HBB, HBA
2.69E-08	1.859	503	Up-regulated	TRPV2, PSAP, RNASE2, FFAR2, LPL, ZFF
2.69E-08	1.747	967	Up-regulated	LSM6, KIR2DL3, CARS2, CNPY3, HLA-F,
2.69E-08	1.932	898	Up-regulated	TIGAR, TWF1, H2BC12, FCER2, FGL2, T
2.69E-08	1.995	404	Up-regulated	ARL4C, PRF1, XCL2, RORA, CST7, GZMI
2.69E-08	2.113	600	Up-regulated	SDHD, ACAT1, HMGCL, IFT43, KIF23, M
2.69E-08	2.110	525	Up-regulated	HBB, STEAP3, GLRX5, BCS1L, CP, PIGA
4.02E-08	1.789	625	Up-regulated	SYNE1, FBXL7, LMO4, GPC3, TSPAN7, I
4.57E-08	2.094	242	Up-regulated	AIF1, TYROBP, LAPTM5, CD74, CX3CR1
4.71E-08	1.668	973	Up-regulated	HBZ, AHSP, HBM, ALAS2, GYPB, GYPA,
5.55E-08	1.805	577	Up-regulated	AASDHPPT, AATK, ABLIM3, ACTL6B, AC
1.52E-07	1.667	902	Up-regulated	CCL26, TNFSF13, HGF, TNFSF12, TNFRS
6.00E-07	1.607	1040	Up-regulated	HBG2, HBA2, RPL15, HBG1, RPS4Y1, RI
2.74E-06	1.758	508	Up-regulated	ZNF710, H2BC17, CYBC1, H2AC21, H2E
1.09E-05	1.651	609	Up-regulated	C3AR1, CFB, C5AR1, CFD, CFI, CR1, TYF
1.09E-05	1.892	250	Up-regulated	SRGN, GADD45G, CLEC2B, CPQ, ADIPC
1.56E-05	1.734	402	Up-regulated	CCL26, STAT3, STAT1, STAT2, CCL2, GN
1.67E-05	1.609	744	Up-regulated	SLC16A2, ALDH1A1, GATM, C1QB, IGF
1.98E-05	1.950	197	Up-regulated	IL1R2, CSF1R, CD1B, CD1C, CD1A, TLR8
5.48E-05	2.127	96	Up-regulated	CCL3, CXCL12, CCL2, CCL21, CXCR4, PL
5.65E-05	1.613	623	Up-regulated	ANXA2, CD69, MVP, NKG7, LRRC8C, LC
5.90E-05	1.753	365	Up-regulated	CLIP2, NDUFA4, NODAL, PDHX, UNC80
5.90E-05	2.100	100	Up-regulated	RNASE4, RAB31, GEM, RAB13, DDX3Y,
6.77E-05	2.057	115	Up-regulated	RGS5, ACKR1, CCL3, EDNRA, AKAP12, I
1.71E-05	2.159	98	Up-regulated	CRLF1, RALA, NOS2, PSMB1, IL32, CD4
5.68E-04	2.034	74	Up-regulated	CCL2, ADAMDEC1, SPINK4, CDKN1A, C
6.00E-04	1.928	120	Up-regulated	PIK3CG, GIMAP2, C4A, C1orf216, RHO
2.74E-06	1.846	320	Up-regulated	BCAR1, GPC6, TEAD2, TEAD4, THBS2, T
1.82E-05	1.865	265	Up-regulated	OXTR, TRHR, OR2S2, P2RY4, P2RY6, P2
2.38E-05	2.165	83	Up-regulated	RPS6KB2, PTPN11, RPS6KB1, SYK, STAT
3.96E-04	2.167	31	Up-regulated	TGFBR1, CXCL16, PLEK, SFT2D2, ITGAN
3.44E-03	1.895	83	Up-regulated	UAP1, YAP1, LTBP1, SYPL1, RABGAP1L
2.69E-08	2.601	186	Up-regulated	STAR, DNAJB9, HSPA5, SELENOK, XBP1
2.69E-08	2.389	182	Up-regulated	IFITM2, IFITM1, NKG7, KLRF1, CST7, G
2.69E-08	2.494	196	Up-regulated	GSTA1, RBP1, TNNI3, MAGED2, STMN
2.69E-08	2.767	194	Up-regulated	TCF21, TNFRSF12A, DCN, GPRC5A, IQC
2.69E-08	2.275	172	Up-regulated	ADAMDEC1, CCR1, CCR2, CCR4, CCR5,
2.69E-08	2.340	166	Up-regulated	MELK, FCGR1A, SCNN1A, FER1L4, VCA
2.69E-08	2.253	191	Up-regulated	ADA, PARP3, MICA, KLRC4-KLRK1, NR1
2.69E-08	2.590	191	Up-regulated	ADA, ABI1, BTNL10, CD300LD, CD24, C

2.69E-08	2.504	191	Up-regulated	TANK, MIR892B, KHDC1L, PDCD6, BCL
2.69E-08	2.613	194	Up-regulated	ABI1, IGLL5, NR1H3, ARPC5, ARPC4, A
2.69E-08	2.432	169	Up-regulated	PPARGC1A, RPL35, LARP4, RPL39L, RP
2.69E-08	2.291	177	Up-regulated	HDAC6, PLA2G4B, PDCD6, WASHC1, T
2.69E-08	2.398	182	Up-regulated	ADA, BTNL10, CD24, CDH5, IGLL5, MI
2.69E-08	2.365	105	Up-regulated	SIGLEC14, KLRF2, KLRC4-KLRK1, CLEC3
2.69E-08	2.347	192	Up-regulated	LINC00623, GOLGA7, P2RX4, WWP1,
2.69E-08	2.209	199	Up-regulated	HTRA1, ACTG1, ICAM1, ADM, SLC43A3
2.69E-08	2.376	164	Up-regulated	POLR2E, SMAD7, SH3PXD2A, TWF2, D
2.69E-08	2.206	172	Up-regulated	CCR1, GPR160, FCHSD1, KCNK4, GLUL,
2.69E-08	2.391	164	Up-regulated	PASD1, SSX5, CCDC157, EOMES, PABP
2.69E-08	2.286	181	Up-regulated	XAF1, SIGLEC1, OAS1, IFI44, IFI44L, M
2.69E-08	2.185	172	Up-regulated	TRIM14, SLC16A6, PPP1R3B, ATP6V0D
2.69E-08	2.434	193	Up-regulated	PKNOX1, MMACHC, NEMP1, PUS10, C
2.69E-08	2.131	441	Up-regulated	CLIC1, CLDND2, GZMA, H2AC15, S1PR
2.69E-08	2.316	418	Up-regulated	TULP3, ZFP36L2, PAPSS1, SEPTIN9, FC
2.69E-08	2.179	308	Up-regulated	ECHS1, CAPRIN2, LGALS8, NDUFB2, D
2.69E-08	2.706	204	Up-regulated	ATP13A3, TGIF2, MAPKAPK2, STX4, R
2.69E-08	2.366	299	Up-regulated	CTNNB1, ARHGEF1, CAPN11, ABL1, PT
2.69E-08	2.156	349	Up-regulated	CD8A, CD8B, KLRK1, CRTAM, GTF3C1,
5.32E-08	2.236	154	Up-regulated	TBC1D22A, FRMD4A, TWF2, FAM111A
6.45E-08	2.227	154	Up-regulated	CNGA3, NFASC, BIN1, ANK3, KCNQ2, K
6.81E-08	2.172	182	Up-regulated	ADA, ABI1, BTNL10, CD300LD, CD24, C
7.32E-08	2.196	166	Up-regulated	SMC4, CENPA, SMC2, H2BE1, H4-16, H
8.16E-08	2.139	192	Up-regulated	SCYL2, PTP4A1, ALG13, ELF2, ZFP36, T
1.17E-07	2.115	194	Up-regulated	PKNOX1, ST8SIA4, LYAR, BRIX1, NUBP
1.53E-07	2.134	198	Up-regulated	HBA1, HBA2, HBM, HBB, HBD, HBE1, H
1.54E-07	2.165	175	Up-regulated	INPP1, PLCG2, C17orf75, HIPK2, ZDHH
2.10E-07	2.112	192	Up-regulated	PIBF1, IL24, CNTF, IL31RA, CSF1R, CSF
2.34E-07	2.501	33	Up-regulated	SULF2, VCAN, NCF4, NID1, ANXA5, SIR
2.56E-07	2.106	192	Up-regulated	ANKS1A, GOT2, OSBPL3, PAICS, FEN1,
4.16E-07	2.146	155	Up-regulated	EDIL3, ENAM, PRG4, PRG3, SPON1, FB
4.35E-07	2.085	195	Up-regulated	POLD2, PRELID1, EIF2B2, SRM, PDCD2
6.62E-07	2.083	189	Up-regulated	RGS2, PPP3CC, SOCS3, SOCS1, TP53AI
7.38E-07	2.152	145	Up-regulated	ATG4A, FAM98B, PAAF1, DCAF4, FAM
1.31E-06	2.069	172	Up-regulated	ADA, ACOT8, POM121C, PLA2G4B, PIG
1.35E-06	2.064	184	Up-regulated	EBI3, LILRB2, CXCR6, CCR9, LILRB1, GP
1.53E-06	2.096	168	Up-regulated	DCAF8, ITGA6, HECTD3, PGGT1B, P2R
1.99E-06	2.136	141	Up-regulated	A1BG, ADA, SIGLEC14, CD24, HDAC5,
2.21E-06	2.168	138	Up-regulated	KCNJ2, DUSP16, OTUD1, SOCS2, MXI1,
2.58E-06	2.033	192	Up-regulated	FAR2, MLKL, ATOX1, CDC25A, ZNF232,
2.67E-06	2.075	181	Up-regulated	FGR, ZMYND10, ST7, ETV1, MPND, FA
2.79E-06	2.022	188	Up-regulated	NOD1, PGLYRP2, PGLYRP3, DMBT1, F
3.56E-06	2.023	190	Up-regulated	NOD1, PRG3, LAMTOR5, PARK7, TIRA
4.31E-06	2.096	143	Up-regulated	LYN, HOPX, SH2D1B, FOXD1, NOTCH1,
4.44E-06	2.042	179	Up-regulated	SH2B3, IFNL4, CDK5, PIBF1, STAMBP,
5.14E-06	2.010	190	Up-regulated	MASP2, VSIG4, CR1, CRP, CD93, APCS,

5.24E-06	2.095	137	Up-regulated	METTL27, SEPTIN9, GALNT7, AFP, CDK
5.96E-06	2.071	154	Up-regulated	MAN1A2, FBXO22, PPT2, CAT, NDUFA
1.44E-05	1.976	190	Up-regulated	TFEC, MPEG1, PTP4A2, CTSZ, LY86, FK
1.48E-05	2.007	176	Up-regulated	RGS1, ZDHHC2, SMPDL3B, SLC52A3, C
1.62E-05	1.974	185	Up-regulated	LY86, NCF1C, BASP1, CD1C, MEF2C, H
1.87E-05	1.989	159	Up-regulated	MOB1A, ZFP36L2, IGSF6, TMEM123, I
2.91E-05	1.932	197	Up-regulated	NR2E3, CD300H, CD300LD, C17orf99,
3.10E-05	1.939	170	Up-regulated	CDH1, VIT, GEM, GRN, EOMES, HPSE,
3.27E-05	1.925	197	Up-regulated	CXCR4, DUSP1, ICAM3, RPL21, CSF3R,
6.70E-05	1.989	133	Up-regulated	STK38, EHD3, SLAMF6, ZYG11B, TSR1,
7.75E-05	2.011	127	Up-regulated	IFNL1, TBX21, ANXA1, HLX, IFNA2, IFN

pathway	SYMBOL
GOBP_CHROMATIN_ORGANIZATION_INVOLVED_IN_REGULATI	CDKN2B-AS1|HOTA
GOBP_MRNA_METABOLIC_PROCESS	SNRPGP15|RNU4AT
GOBP_NEGATIVE_REGULATION_OF_GENE_EXPRESSION_EPIGE	CDKN2B-AS1|HOTA
REACTOME_CELLULAR_RESPONSES_TO_EXTERNAL_STIMULI	FKBP4|CDC27|CRE
REACTOME_EUKARYOTIC_TRANSLATION_ELONGATION	RPS20|RPL26L1|RP
REACTOME_INFLUENZA_INFECTION	POLR2J|RPS20|NU
REACTOME_METABOLISM_OF_RNA	LAS1L|RBM5|UPF1
REACTOME_REGULATION_OF_EXPRESSION_OF_SLITS_AND_RO	PSMB1|RPS20|PSM
REACTOME_DEVELOPMENTAL_BIOLOGY	CYP51A1|PLXND1|
NAKAYA_PBMC_FLUARIX_FLUVIRIN_AGE_18_50YO_3DY_DN	SENP1|CHD2|DDX5
PICCALUGA_ANGIOIMMUNOBLASTIC_LYMPHOMA_DN	MYLIP|CBX4|NR4A
HAY_BONE_MARROW_NAIVE_T_CELL	RPS27|RPL30|RPS2
PECE_MAMMARY_STEM_CELL_UP	RPS26|KRT17|MAR
TRAVAGLINI_LUNG_MESOTHELIAL_CELL	CALB2|KRT8|CFB|(
TRAVAGLINI_LUNG_PLATELET_MEGAKARYOCYTE_CELL	GNG11|RGS18|PPE
POOLA_INVASIVE_BREAST_CANCER_DN	INPP4B|MSX2|WN

pval	padj	NES	size	Enrichment
6.72E-05	0.00343633	-2.0311233	92	Down-regulated
1.00E-10	2.69E-08	-1.8659227	721	Down-regulated
1.62E-05	0.00106979	-2.2018141	72	Down-regulated
1.00E-10	2.69E-08	-1.9980866	532	Down-regulated
1.00E-10	2.69E-08	-3.1270261	85	Down-regulated
1.00E-10	2.69E-08	-2.5686348	143	Down-regulated
1.00E-10	2.69E-08	-1.8905345	584	Down-regulated
1.00E-10	2.69E-08	-2.9042671	146	Down-regulated
2.61E-07	3.10E-05	-1.6698627	581	Down-regulated
1.00E-10	2.69E-08	-2.8366985	405	Down-regulated
1.00E-10	2.69E-08	-2.6909338	126	Down-regulated
6.50E-08	9.24E-06	-1.9017142	346	Down-regulated
2.60E-07	3.10E-05	-2.3206286	101	Down-regulated
3.24E-07	3.74E-05	-1.7495754	401	Down-regulated
8.02E-07	7.97E-05	-1.7694544	392	Down-regulated
9.06E-05	0.00429789	-2.1497025	61	Down-regulated

PMBC_LINP_genes
ACTB, ACTG1, ATF4, ATP5F1B, ATP5F1E, ATP5PB, ATP5ME, ATP5MG, ATP5PO, BRK1, BZW1, ATP5MPL, FM
TNFRSF10D, H4-16, H4C8, H4C3, HSPA4L, H4C5, H1-3, H1-4, IL3, H2AC17, IFNB1, EPHB3, H2AC8, HSPA6, H
CTSS, TLE3, NR4A3, ATF7IP, BSDC1, SC5D, RNF114, PPARGC1A, SDF2, NAP1L1, CCN1, GTPBP1, UNC50, STX
SCYL3, STPG1, RAD52, BAD, LASP1, ARF5, AK2, KDM1A, RBM6, HSPB6, ZMYND10, UPF1, WDR54, RHBDD2,
CAST, MTSS1, SLC6A1, FGF17, RANGAP1, MXI1, SLC35A2, H2BC17, AGTR2, H2BC15, H2BC12, H2BC13, LIN
PF4, SECTM1, CCL23, SOD2, PLAUR, HK3, LILRB3, CCR1, CCL4, MARCKS, NCF1, CASP1, HOXB2, CCL3, DMXL
ABI3, ADM, AKR1A1, ANXA4, ASCL2, ASGR2, ATF3, ATF5, AXL, CCDC180, BLVRA, SMCO4, C1QB, C1QC, C2,
SMARCA1, WHRN, ABLIM1, APOBEC3A, TUBB2B, TMEM255A, CXCL12, DOCK4, CD109, SYNDIG1, SFRP2, TI
APOE, LIPA, TREM2, GPNMB, CHI3L1, CCL18, CHIT1, LILRB4, ACP5, CTSB, CTSZ, PRDX1, CSTB, APOC1, CAPG
GAA, USP6, FCGR3B, HK3, CSAD, TSPO, PSMF1, CX3CR1, SMIM20, RAP1GAP2, ITM2B, GLUD1, JAK2, MMP2
POLR2J, RPS20, NUP160, RPL26L1, POLR2B, TPR, EIF2AK2, NDC1, RPL18, IPO5, NUP133, RPL31, NUP37, HS
DCUN1D4, RCBTB2, F13A1, ZNF641, ADORA3, RGS18, ZNF844, TET3, CDCA7L, PWWP2B, DENND4C, ANKH,
PSMB1, PSMC4, MNAT1, PSMA4, SPI1, CBFB, PSME4, TCF3, ITCH, TP73, PSMC5, PSME1, PSMD5, ABL1, PSI
RNF181, CD22, MGAT4B, TRIM52, ERCC5, B3GNT2, DOK2, RPUSD2, GLB1, CCDC88A, PTGFRN, ANAPC15, N
PHF23, CTDSP2, FBXO7, C16orf70, CXCR4, ARRDC2, SETD1B, FRAT1, PHC2, ZDHHC7, RUNDC1, WIPI2, BMF
P2RY1, PAPSS2, HES1, SLC1A4, ACTR6, SEC61G, EDNRB, ENPP2, MEAK7, ZNF423, MYBL1, EMX2, SGCB, MP

IC1-LUC7L2, CFL1, CHCHD2, COX4I1, COX6B1, COX6C, DDX5, DYNLL1, EEF1A1, EEF1G, EEF2, EIF4A2, FAU, GABAR/
2BC8, RNF152, CH25H, H2AC20, WFIKKN1, LVRN, H2BC15, IFNG
12, MARCKS, IFT57, DDX27, OASL, NAMPT, ETV1, AKAP8L, TIGAR, ARID4B, TUT7, BRD2, NKX2-1, YY1AP1, RUNX1,

, GDE1, AP2B1, ETV1, TTC22, DBF4, PAF1, CDKL3, DNAH9, CEACAM7, LUC7L, DLEC1, IL32, POMT2, EEF1AKNMT, D
28A, H2BC11, RGN, SPRED1, FBXO21, H3-4, NSMCE3, CNTLN, PDS5A, MYH2, EID1, TRAF3IP2, TGIF1, PLAAT1, KLH
2, LGALS3BP, ARHGAP22, HOXA9, LTBP1, SLC2A6, CD163, LILRB2, SNX10, SMC4, VCAN, IGF2R, THBS1, WARS1, FI
TSPEAR-AS2, C3AR1, C5AR1, CALML4, CAMK1, CASP5, CBR1, CCL8, CCRL2, CD300C, CD300E, CDKN1A, CES1, CES1
MPRSS15, AGMAT, TYMP, FAM174B, NUAK1, ABHD12, GREB1, MFAP2, TLR4, TMEM37, RASSF4, ARPC1B, LCP2, S
i, NUPR1, PLD3, CD68, GRN, CTSD, HLA-DMB, LGMN, ANXA2, BRI3, C15orf48, CYP27A1, FBP1, TXN, PLA2G7, HLA-

25, MFSD14B, CSNK2A1, STX3, ICAM3, TBCB, HIPK3, NELFA, RESF1, NBR2, GCNA, NSFL1C, LRP10, MANSC1, PTPN1
SP90AA1, XPO1, RPS5, SEH1L, RPL6, RPLP0, NUP50, AAAS, NUP188, RPS18, POLR2E, POLR2F, RPL3, PABPN1, RAE1
, TLR4, NRGN, CDK4, POGK, ZNF571, RNF130, HRH1, ARHGAP15, PDP1, EIF4E3, TSPAN32, TACC1, HACD4, KCTD7,
MD8, PSMC6, PSMA3, PSMC1, PSMB5, PSMA6, PSME2, PSMA7, PSMD10, GATA1, PSMD7, H2AZ2, PSMA2, GATA3
MTMR10, RHOB, PMS1, DENND4C, BAIAP2-DT, PLA2G15, HEXA, CDK19, CLN6, GATM, PEMT, ZNF302, LAMP2, CO(
, DCAF12, ARL6IP5, SASH3, CXCL16, ELOVL1, USP22, MAP7D1, WDR82, VPS35, HINFP, PLEKHO1, HECA, KIAA2013
HOSPH9, NCOA1, SAC3D1, DLK1, MEG3, ALDOA, WT1-AS, SPIDR, ATP9A

AP, H3-3B, HNRNPA2B1, HNRNPDL, HNRNPK, HNRNPU, HSPA8, MARCKS, NACA2, NDUFA13, BEX3, PARK7, PCBP1,

NFU1, PLPP3, PLEKHM1, KDM6B, ARMCX6, STBD1, CD84, AKTIP, CDC14A, KLF2, SQSTM1, THEMIS2, HGSNAT, DU

DYRK4, LRRC23, MKS1, AKAP8L, RNF216, BRCA1, CALCOCO1, PSMC4, ANGEL1, RNF14, DDX11, ZC3H3, CAPN1, MD
L41, TDRD5, CELA3A, CYP51A1, ONECUT2, BMPR2, MEIS1, OTP, RASAL2, NDRG3, H4C1, LHX2, ARAP2, PPP2R2B,
PR1, GCH1, COL4A5, RHAG, NAMPT, CXCL3, C1QA, CYP1B1, ADGRE1, ABHD2, JAG1, H3C10, VNN1, TMEM176A, N
LP1, CFB, CFD, CLEC12A, CLEC6A, CMKLR1, CNIH4, CXCL10, CXCL11, CYGB, DDAH2, DHRS7B, EPHB2, FAM25A, TM
PP1, GPM6B, COTL1, ITGB7, ARID5A, ARHGAP6, RNASE6, CEBPD, CX3CR1, NMU, MCTP1, MGAT5, CCDC86, SHMT
-DQA1, FABP3, MRC1, GPX3, CTSH, C1QC, C1QB, SLAMF8, ALDH2, FN1, FPR3, FTL, KCNMA1, TMEM176A, MMP9,

18, CREB5, COP1, TPM3, INTS3, INO80E, ARGLU1, TRIM24, NDUFB1, PHKA2, PYGL, CHAC2, GCA, ARSA, RCBTB1, A
1, DNAJC3, KPNA3, NUP93, POLR2C, RPS16, POLR2I, TGFB1, RPS19, RPL18A, RPL28, RPL19, KPNB1, NUP88, RPL34
ANKRD50, CXCR2, C11orf21, TRIQK, SLC25A48, CHPT1, SLC2A8, RHBDF1, DPEP2, AP1S2, ADCY7, DOK2, EMB, PA
3, PSMD3, PSMD11, PSMD9, PSMD14, KMT2A, MYB, H2BC11, PSMF1, PSMB2, SEM1, PSMA1, PSME3, H3-3B, CDK
Q4, GRN, EPAS1, ANKRD6, ITM2B, PHYH, CLN3, PCM1, ZNF496, ABHD14A, ANP32A, ZNF252P, TMEM223, MSH2,
, DDX17, NCOA6, CMTM3, USP4, GLE1, SYNRG, ATG9A, PRELID1, MNT, UBAC1, RAB11FIP4, PAFAH1B2, DNAJC5,

, PFDN5, PSMB4, RBM39, RBM8A, RPL13, RPL14, RPL15, RPL28, RPL3, RPL31, RPL34, RPL38, RPL41, RPL6, RPL7, F

JSP6, CXCR4, KDM7A, CCNL1, TRIM38, MBTPS1, ARG2, INS, OSER1, H2BC7, BRF2, ZNF215, GABARAPL1, COMP, M

DH1, NUDCD3, GLT8D1, RTF2, RNH1, SNAPC1, CCDC28A, HSD17B6, TOMM34, VIM, MIPEP, IFNGR1, SH2D2A, TBP
TCF4, BLNK, FGD4, H3C12, LIMA1, SLC6A13, KMT5A, FOXP1, DNAJB8, HOXB4, ETS1, H3C1, HOXB6, CPNE1, H3C2,
AP1L1, FGR, TYMP, EPB41L3, TNFAIP3, HOXB3, DEFB1, GGT1, H3C4, PRDM2, RUNX1, IER3, LAPTM4B, PLEK, CXCL
EM255A, FCAR, GPBAR1, GPR141, GPR35, GPR84, HAVCR2, HBEGF, HK3, ID1, IFI27, KCTD14, KIAA1958, KLHDC8E
T1, DPYSL2, CDH1, SLC25A20, TRIM47, PEBP1, DEFB4A, PYGB, ATP13A2, NTRK2
SERPINF1, TMEM176B, C1QA, HLA-DMA, PSAP, MARCO, GSN, IL4I1, UBD, ACP2, FUOM, PTMS, IFI30, A2M, PLXD

RHGAP9, FAM8A1, RPGRIP1, ZNF787, NASP, BICD2, CSNK1G3, DPEP3, LMF2, CIAO2A, CZIB, RMI1, FYB1, VCL, TRE
I, RPS13, NUP98, NUP107, RPS12, NUP155, KPNA1, RPL24, RPS15, RPL22, RPS25, NUP43, RPL21, RPL5, CLTA, RPS
QR8, DHX57, FAM189A2, RTN3, DCBLD1, TIAF1, RHOBTB1, PAK1, LST1, GNPDA1, CCSAP, HLA-DMB, DIAPH1, OTU
K7, CCNH, LMO2, PSMB7, YAP1, TCF12, PSMB6, PSMA5, RPS27A, H2BC1, UBC, PSMA8, H2BC5, H4C8, RUNX1, PSM
ANKRD34C, AVPI1, QSER1, ATP6V1D, APMAP, ATM, PLCB2, MMD, NLRC4, GDE1, TUFM, HACD3, GANC, RNF113A
CHAMP1, CTCF, GIT2, KLF13, BRPF1, WBP1L, FAM89B, NRBP1, SP1, ZNF398, WASF2, TACC1, ZNF45, ORAI3, SDHA

RPL7A, RPL8, RPL9, RPS27A, RPS4X, RPS8, RSRC2, SAP18, SEC61B, SEC61G, SERBP1, SF3B1, SKP1, SLC25A3, SON, S

IKRN1, SOCS1, TACC2, ARF4, ISG20, FRAT1, IMPA1, ARIH1, UFSP2, SCYL3, NAT9, SNORD52, CASP9, NECAP1, PTHL

L1, SLC39A9, ARID4A, PNPLA6, ZCCHC8, ASTE1, GABARAPL2, USP2, CLEC16A, PARP3, TDP1, AIFM2, SPATA7, CAP(
H3C3, H3C4, GRK5, H3C7, H3C10, E2F3, NRP1, ZNF710, NAP1L5, NLRC3, KCNK12, SDHAF3, RUNX1T1, KCNIP4, H2
10, PELI1, PIEZO2, PILRA, SCPEP1, C2, PTAFR, CD300C, HNMT, STS, C5AR1, HIP1, HOXB5, SAT1, QPRT, IL6, CD86,
, KMO, KRTAP5-10, LGALS1, LILRB1, LTF, MAFB, MARCO, MERTK, METRNL, SLC49A3, MIR1250, MIR185, MS4A4A

C2, NR1H3, ACE, SERPING1, C2, DAB2, LGALS3, CYBB, CD81, MMP14, HEXB, MSR1, MAFB, ENG, TYMP, CTSL, SLC(

RF1, CD33, EXOC6, ZMPSTE24, MCL1, EIF4E3, WDFY3, DPY19L3, EPS15, C1RL, RPS6KA3, IMPA2, RASA1, NRDC, H
10, NUP153, NUP85, GTF2F1, RPL23, NUP214, CANX, RPS4Y1, RPL36, RPL27, NUP210, RAN, GRSF1, RPS15A, NUP
LINL, SEPTIN9, PAQR5, NCAPH, TCFL5, LPAR6, SYNE3, SLC46A3, DUSP13, CPM, PITHD1, ZKSCAN4, ZCCHC24, STAR
ID4, PSMB4, PSMC2, TAL1, H3-3A, PSMD6, H2AZ1, PSMC3, LMO1, UBB, PSMD1, PSMD2, GATA2, H2AC6, H2BC4,
, PEPD, CCPG1, C7orf50, CHPT1, HTT, TXNDC12, HACD2, TMEM37, FN3KRP, GHDC, MTMR12, PAK1, PLBD1, AMD
P1, TNFAIP8L1, BRD8, TLR1, NLRC4, CHCHD4, PTP4A2, SRSF9, PLEKHO2, HARS2, RNF220, HHEX, DPP8, MKRN1, A

RP14, SRSF5, SUB1, SUMO2, ELOB, TSC22D1, TUBA1B, UBB, UBL5, UQCRB, UQCRQ

.H, BCL3, MAP3K13, EIF2AK3, FEM1C, JMJD6, MIS12, ZNF394, HLA-DRB6, DMTF1, BACH1, TSC22D3, POLR2A, TNF

G, TRIT1, CUL7, GEMIN8, FAM214A, EPN3, RFC2, JKAMP, FAM160A2, SIKE1, RRP12, ATG5, ATP2B4, CCAR1, SPHK
AC21, SERPINA7, TCERG1L, H2AC20, H2AW, H2BU1, H1-4, H1-3, H1-2, H1-5, INPPL1, H1-1, CACNG2, PRKCQ, SMC
TMEM176B, LILRA4, CPVL, ACSL1, FCGR3B, HOMER3, HMOX1, TNFAIP2, PTX3, C1QB, LMNA, TBXAS1, ITGB3, SMP
, MTHFD2, LINC00092, FAM225A, NFKBIA, NGFR, NLRP3, NOTCH2NLA, NR4A1, NSUN5P2, OR3A3, P2RY12, P2RY

2B1, CPM, HLA-DRA, RARRES1, MS4A7, HLA-DQB1, GPX4, FABP5, LYZ, RAB13, HLA-DRB1, HLA-DRB5, S100A11, H

SD17B11, CAPN1, ABHD18, C2CD2L, NUDT5, ACTN4, DSN1, PARVG, METTL7A, HSPA8, XRN2, LPCAT2, TMEM272,
42, RPL35, RPS6, RPLP1, RPS24, NUP54, NUP58, RPL3L, RPS2, CLTC, RPS11, RPL13A, RPL11, RPS8, PARP1, RPS27
D9, GAS7, C1orf112, TRIM36, MAP4K1, SNX24, ING2, FAM214A, PER3, MCOLN1, CHEK2, CTDSPL, DUS2, KIF13A,
H3C13, H2AC20, H2BC21, H2BC13, PSMD13, H2AX, H2AC7, H2BU1, H4C3, H3C12, PSMD12, H4C11, H3C4, H4-16
HD1, COQ8A, APBA1, RNF125, MXI1, OSGEP, ATRN, MAP1S, ZC3H6, NDUFC2, TIMMDC1, COMMD3, ALDH5A1, M
RPC1B, SH2D3C, KANSL1, FRAT2, GABARAP, NUP214, SF3B2, CRTC3, MARF1, MAP3K14, RALA, CALHM2, NUP50,

SF10, GLIPR1, RRNAD1, BHLHE41, PDE8A, CTSB, TBC1D3F, DXO, PPP1R10, PNLIPRP1, SLC2A3, NRBF2, TESC, ACKI

2, RPL18, MED29, CASP8, INTS13, ANKS1A, ZNF76, GNAI3, PDE4A, LRRC40, TRMT11, TP53BP1, OTUD5, TTC7A, IP
), H1-6, DHRS3, H2BC21, CA7, ATXN7L2, CLDN2, AOC2, H2AC4, H2AC6, H2AC1, H2AC11, H2AC7, H2AC8, CLDN10,
DL3A, PDGFD, HOXA10, CAST, HOXA5, CTSL, KCNK5, MAFB, AKR1C1, S100A6, CD36, ADCY2, MMP2, SNCAIP, HO
2, PER1, PILRA, PTPRO, RGL1, RRAS, RTP4, SAMD4A, SDC3, SDHB, SEMA4A, SERTAD1, SLC11A1, SLC27A3, SLC6A1

SD11B1, RAB31, LAMP2, NCEH1, HLA-DOA, CTSK, LGALS3BP, MS4A4A, NPL, ADAMDEC1, AGPAT2, ITGAM, PILRA

MEGF6, CCPG1, RFNG, DERA, CMTM7, MAP3K1, MPPE1, NDUFB3, RNF169, ZNF92, TLE4, SLF1, C14orf93, H2BC4
, POLR2D, RPL32, RPS3A, RPL37, RPL10, RPL7, POLR2K, RPL7A, RPS3, FAU, RANBP2, NUP205, RPL30, SEC13, CPS
IMPA2, CSK, ATP2B1, CPNE8, PARVG, CELF6, BBS9, ATM, GRAMD4, HSPBAP1, FEZ2, CNOT7, SLC35F6, PRKACB, M
, H2BC12, LDB1, H2AC18, PSMB8, PSMB10, UBA52, PSMB11, H2BC15, PSMB9, H2AJ, H4C14, H2AC19, H4C12, H2
AP4K3, PLBD2, FCGRT, PXMP4, COMTD1, RNASET2, PTPN18, SMARCC1, C16orf54, SPATA12, EXOC4, EHMT2, TN
DSTYK, TOR1A, TCF20, STX6, PLXNB2, THEMIS2, TSC22D3, PSMG2, DOK2, TMEM250, PIGM, RNF44, RETREG3, M

R3, RNF11, RECQL5, GADD45B, NARF, GTF2B, CBX8, HBEGF, ZCCHC8, FAM53C, SPOP, RAPGEF2, MTMR6, NEMF, I

6K2, NUCKS1, LRP6, NUCB2, ASNS, CFAP20, PTPN21, SNX13, PRKACA, ACTN1, AFF4, AP1M1, XRCC1, NLE1, CLNS1
, GPM6B, STARD13, DGKG, PPP3CA, CNN1, USP34, CHD4, DPT, NFATC1, H2BC1, H2BC3, H2BC4, H2BC5, TBXAS1, S
XA7, CLU, H1-2, BCL6, H2BC4, PRKCD, PLA2G4A, GNS, PPBP, TREM1, HOXA4, HOXB6, ABCC4, MCTP1, CAT, CXCL2,
2, SNORA5B, SNORA80A, SPRR2F, TCN2, TLR7, TMEM150B, TNFSF10, TP53I3, TRIB1, VSTM1, ZBTB21

, HNMT, LHFPL2, AXL, IGSF6, C5AR1, SLC1A3, AVPI1, TMEM51, CD9, CD74, PHLDA3, VSIG4, SNX10, VAT1, HSPB1,

, CARHSP1, AIF1, NRBF2, FCHO2, EVI5, CAPRIN2, ATP13A1, KIZ, GRAMD1C, SLC35A1, DOP1B, DEF8, MSL3, CCDC1
F4, RPL8, RPL26, RPL29, NUP35, RPL22L1, RPL9, POLR2H, RPL39L, RPS14, RPL10L, RPL36AL, RPL27A, RPL13, POLR
IPPE1, NCAPG2, LRRC8C, CD33, ATPAF1, MTCL1, SENCR, PVT1, TPM1, ASB1, POMGNT2, ATP6V0E2, VCPIP1, ADO
BC14, H2BC8, H3C8, H2AB1, H2BC6, H4C6, H2BC17, H3C6, H4C13, H3C11, H2BC9, H3C1, PSMB3, H4C9, H2AC14,
RC6B, CD302, SLC2A9, NBPF10, SWAP70, SEC14L1, NDUFB10, PCYOX1, PAQR8, SLC35A1, RNF141, GPAM, CORO2
BD2, ZNF227, TRAPPC12, NELFB, CASP2, ENC1, DUSP7, GDE1, SNX17, CAB39, GRN, PGP, ARHGAP30, IFFO1, ASB7

RS2, ARID3B, PFDN2, DDX50, PPP1R15A, ALOX5AP, CHTOP, TRMT1L, BEX4, PNRC1, DUSP11, EIF1, NFE2L1, CCPG1

A, EED, TSG101, SLC24A1, ENO1, TUBE1, ACTR6, EIF4G3, MARK3, SLC25A3, FRYL, DLG1, ARHGEF1, UBE2T, EXOSC
SLC12A2, H2BC6, OLR1, H2BC7, H2BC8, H2BC9, SLC10A2, RNF44, CDH16, NRAP, DCDC1, USP47, H2AC13, USP46,
, MEIS1, AQP9, CX3CR1, G0S2, BCL2A1, ETS2, HCK, FBP1, LILRA1, AIM2, IL1B, SERPINA1, VNN2, CCL20, SERPINB2,

PTAFR, ABCC3, ATP6V1F, HLA-DPA1, CXCL9, SLC7A7, CLEC4E, CD63, GM2A, C1orf54, CSF1R, DNASE2, SLC15A3, C

25, CXXC1, TUBA1C, ATP5F1E, TBC1D1, ATM, SULT1B1, TCP11L2, STK38, ANKRD44, SPINT1, ZNF516, MSRB2, SLC
R2G, RPSA, RPS9, RPS21, RPS7, RPL38, RPL4, RPL15, RPLP2, POLR2L, RPS27, CALR, POLR2A, KPNA2, RPS17, RPL35A
RA2B, TMEM86A, HLA-DMA, AP3S1, CENPN, MEF2C, AATK, SOCS7, NFATC3, WWOX, ARL4C, ZNF441, SAMD13, IF
H2BC3, H4C5, H2AC8, H4C4, H2BC7, H3C7, H2AC4, H2BC10, H4C1, H4C2, H3C10, H3C2, H3C3
2A, KIAA2013, ZNF197, PLEKHA8, TP53INP1, DUSP28, KCNE3, SLC39A10, VPS41, C11orf21, RTL10, WDR61, KPNA3
, OSBPL11, MRM2, RGS19, CBL, CSK, PXN, PRR13, KDM3B, DCAF7, YY1, DNAJC13, BAG4, GIMAP8, ZNF747, POLDI

, BASP1, CDKN2AIP, PIP5K1B, NAP1L2, SEC23B, MOB4, THAP9-AS1, LAX1, PFKFB3, DUSP2, FOXD1, ZNF222, VDR,

5, POLD3, JADE1, SMC1B, TULP3, ZCWPW1, VDAC3, PCM1, THOC1, CIC, KEAP1, SRCAP, MOK, HSP90AA1, PCNP,
AMOT, H2AC12, H2AC15, DSC1, ID4, H2AC17, H2AC16
C3AR1, FGL2, CAMK1, TNF, LGALS2, FCGR3A, HCAR3, H2AC18, PRKAR2B, NFKBIA, GDF15, SMCO4, TLR4, TRIB1,

AVIN3, AKR1A1, FTH1, SCPEP1, SGK1, CORO1C, HLA-DPB1, CD151, HLA-DQB2, FCGR2A, CREG1, LTA4H, ATOX1,

8A1, CASP2, ALG6, PTOV1, SLC8B1, CLEC4C, SGPP1, NCOR1, CD46, SMC1A, ZYG11B, RABAC1, GNL3L, SORT1, DBI
, RPS27L, KPNA7, KPNA4, RPS23, ISG15, GTF2F2, RPL14, POM121, KPNA5, RPS26, RPL37A, RPL12, RPS4X, RPL2
T172, MLEC, MAN2A2, MYO1D, ASRGL1, PINK1, APBA1, TGFBR2, ZBTB4, ZFP36L2, CCDC14, TCEA1, SNX18, ASPH

, NUFIP2, MRTFB, AP1B1, DYRK2, STS, GCLC, FOXRED2, C8orf33, IARS2, TPCN1, SCCPDH, ETFRF1, ZBTB41, ZFHX3,
P3, TAF5, ORAI2, RTF1, TNFAIP8L2, FHOD1, ZNF226, USP3, CRBN, FOXK1, STAU1, RAP2B, TMEM248, CSF1R, ACV

GALNT3, RABGGTB, RIOK3,

MEF2C, ITGB5, DOP1A, LYRM2, G

ORT1, TNFSF10,

NCF2, GAA, UBE2E2, RBM4

, APBB1IP, TRAPPC1, FBXL5,

D2, LRRC37B, NUDT1, NDRG3, COG5, PAG

, GPRIN3, NMNAT1, EVI5, IQGAP2, MYCBP2, TM7SF3, RB
R1B, TRIM38, TFEB, SMC3, NLRP

Lin Pos vs Lin Neg

Lin Neg

Lin Pos

Gene ID	log 2Fold Change (edgeR)	pValue (edgeR)	pAdj (edgeR)
LOC107985565	-9.6	1.99E-04	5.71E-03
LINC02224	-9.5	4.10E-04	9.72E-03
LOC112268230	-9.3	1.04E-04	3.50E-03
LOC105377384	-8.9	5.66E-06	3.22E-04
CAVIN2	-8.9	2.30E-33	1.69E-29
LOC105371459	-8.8	2.02E-03	2.91E-02
ITGB3	-8.7	4.37E-42	1.60E-37
GP9	-8.7	1.18E-17	7.32E-15
LY6G6F	-8.6	3.62E-11	6.98E-09
DDX11L10	-8.5	4.31E-09	5.58E-07
TUBB1	-8.5	1.12E-41	2.06E-37
CTTN	-8.4	5.62E-31	2.29E-27
LY6G6E	-8.3	2.27E-23	3.46E-20
LOC107987071	-8.3	2.13E-04	6.04E-03
LOC105374242	-8.2	1.34E-03	2.20E-02
HBA2	-8.2	1.78E-05	8.54E-04
CMTM5	-8.1	3.89E-14	1.25E-11
LOC105377428	-8.1	1.99E-03	2.86E-02
LOC105372169	-8.0	1.77E-03	2.64E-02
MIR6843	-7.9	1.49E-15	5.86E-13
ROPN1	-7.9	2.07E-03	2.95E-02
LOC102724772	-7.8	3.30E-03	3.94E-02
LOC102724193	-7.8	5.51E-04	1.19E-02
LOC105377702	-7.8	2.23E-04	6.24E-03
LINC02258	-7.8	1.95E-03	2.83E-02
MT1M	-7.7	4.91E-03	5.07E-02
ITGA2B	-7.7	2.08E-29	6.34E-26
PPBP	-7.7	9.52E-27	2.05E-23
SH3BGRL2	-7.7	2.20E-23	3.46E-20
LTBP1	-7.6	8.32E-32	4.35E-28
INS-IGF2	-7.5	5.57E-03	5.44E-02
TRIM58	-7.4	1.17E-38	1.43E-34
PDGFA	-7.4	6.99E-26	1.28E-22
SPARC	-7.4	4.15E-32	2.53E-28
MYL9	-7.4	5.05E-25	8.80E-22
PF4	-7.4	2.90E-21	3.12E-18
LOC101930100	-7.3	6.36E-04	1.31E-02
PRKAR2B	-7.3	5.07E-26	9.77E-23
SLC4A1	-7.3	5.34E-10	8.54E-08
CALD1	-7.3	2.42E-21	2.77E-18
ANKRD30BP2	-7.3	4.14E-04	9.77E-03
STON2	-7.3	8.30E-30	2.76E-26
OR2L3	-7.3	3.15E-03	3.83E-02
LOC105374058	-7.3	2.77E-03	3.53E-02

MMRN1	-7.2	9.79E-23	1.43E-19
LOC107986763	-7.2	1.33E-05	6.63E-04
CLDN5	-7.2	2.11E-12	5.29E-10
ITGB5	-7.2	2.25E-28	6.34E-25
LOC105369391	-7.2	3.80E-04	9.20E-03
SEC14L5	-7.2	3.52E-19	2.74E-16
CSAG3	-7.2	7.83E-04	1.53E-02
HBB	-7.1	2.72E-12	6.55E-10
LOC107986956	-7.1	8.07E-05	2.88E-03
TINAGL1	-7.1	1.82E-07	1.59E-05
SMOX	-7.1	2.67E-21	2.96E-18
CXCL5	-7.1	1.06E-14	3.77E-12
PEAR1	-7.1	3.22E-27	7.36E-24
MIR548A3	-7.1	1.10E-04	3.66E-03
DUSP5P1	-7.0	9.94E-05	3.40E-03
MIR1268B	-7.0	3.97E-03	4.43E-02
LOC100131315	-7.0	4.96E-03	5.09E-02
HBG2	-7.0	4.48E-06	2.63E-04
MFAP3L	-7.0	1.90E-19	1.55E-16
LINC01645	-7.0	1.55E-03	2.42E-02
LOC102723730	-7.0	3.17E-04	8.09E-03
TREML1	-7.0	1.36E-17	8.29E-15
C2orf88	-6.9	1.33E-22	1.87E-19
LINC00989	-6.9	1.57E-09	2.21E-07
TAL1	-6.9	9.36E-20	7.97E-17
LOC107984360	-6.8	1.05E-10	1.89E-08
SOX2	-6.8	5.08E-04	1.13E-02
CST1	-6.8	1.62E-04	4.93E-03
MPL	-6.8	2.59E-14	8.87E-12
LOC101927854	-6.8	1.47E-08	1.65E-06
CCN1	-6.8	1.64E-06	1.11E-04
LOC107985376	-6.8	5.88E-08	5.83E-06
PDZK1IP1	-6.8	3.07E-10	5.08E-08
LOC105373466	-6.8	2.05E-03	2.93E-02
KRT8	-6.8	2.14E-06	1.40E-04
ADRA2A	-6.7	3.35E-12	7.96E-10
GUCY1B1	-6.7	1.89E-17	1.10E-14
LOC107984272	-6.7	4.66E-03	4.90E-02
TSPAN9	-6.7	2.85E-16	1.29E-13
SPOCD1	-6.7	8.15E-15	2.92E-12
LINC01994	-6.7	8.23E-04	1.58E-02
AVPR1A	-6.7	2.76E-10	4.64E-08
ABLIM3	-6.7	7.15E-19	5.23E-16
PROS1	-6.7	5.63E-14	1.75E-11
LIPH	-6.6	2.84E-14	9.63E-12
CLEC2L	-6.6	1.66E-08	1.84E-06
HBA1	-6.6	4.26E-07	3.45E-05

MEIS1	-6.6	2.28E-17	1.31E-14
SLFN14	-6.6	1.18E-15	4.70E-13
BEND2	-6.6	1.85E-16	8.89E-14
MYLK	-6.6	2.99E-31	1.37E-27
TFPI	-6.6	1.03E-15	4.23E-13
LOC101928797	-6.5	2.86E-03	3.61E-02
RAB27B	-6.5	1.02E-17	6.43E-15
LOC105379148	-6.5	5.42E-03	5.38E-02
CLU	-6.5	4.23E-28	1.11E-24
PCYT1B	-6.5	7.81E-21	8.17E-18
LOC102724207	-6.5	3.26E-03	3.92E-02
KRT81	-6.5	1.01E-04	3.42E-03
OSBP2	-6.5	3.05E-18	2.11E-15
TMEM40	-6.5	1.84E-17	1.08E-14
HNF4A-AS1	-6.5	1.34E-03	2.19E-02
LOC105370416	-6.5	4.81E-03	5.01E-02
LOC112268187	-6.5	1.90E-06	1.26E-04
ZNF25-DT	-6.5	2.99E-04	7.76E-03
NRGN	-6.4	2.48E-26	5.05E-23
LOC105371705	-6.4	1.14E-06	8.21E-05
LOC105372995	-6.4	1.03E-04	3.48E-03
ELOVL7	-6.4	2.61E-15	9.86E-13
LOC105370827	-6.4	2.18E-03	3.05E-02
LOC105372734	-6.4	2.26E-03	3.11E-02
PDE5A	-6.4	7.73E-24	1.29E-20
SYTL4	-6.4	8.77E-16	3.65E-13
LOC105370787	-6.4	4.18E-05	1.70E-03
FSTL1	-6.4	1.47E-13	4.25E-11
HEMGN	-6.4	1.26E-09	1.83E-07
DKK1	-6.4	3.80E-06	2.29E-04
OR2T8	-6.4	1.03E-03	1.85E-02
LOC105372462	-6.4	5.26E-04	1.15E-02
ENKUR	-6.3	1.67E-13	4.77E-11
LINC01583	-6.3	2.65E-03	3.42E-02
EPCAM	-6.3	1.06E-04	3.56E-03
PCSK6	-6.3	3.50E-17	1.88E-14
LOC105376025	-6.3	2.58E-04	6.92E-03
LOC105371078	-6.3	3.04E-03	3.75E-02
LOC105372478	-6.3	5.07E-04	1.13E-02
LOC105371836	-6.3	3.94E-03	4.41E-02
SPINK1	-6.3	3.55E-03	4.14E-02
ARHGAP6	-6.3	1.01E-16	5.00E-14
EGF	-6.2	1.58E-17	9.45E-15
RHOBTB1	-6.2	5.95E-22	7.77E-19
VEGFC	-6.2	4.05E-08	4.12E-06
LOC107984641	-6.2	5.00E-03	5.11E-02
LOC105370952	-6.2	3.01E-06	1.89E-04

LOC107985819	-6.2	1.52E-06	1.04E-04
LOC105373379	-6.2	1.08E-03	1.92E-02
PLAAT1	-6.2	1.63E-07	1.47E-05
LOC107986262	-6.2	9.45E-04	1.74E-02
AQP10	-6.2	6.34E-08	6.26E-06
BMP6	-6.1	8.59E-22	1.01E-18
KRT19	-6.1	4.61E-04	1.06E-02
SELP	-6.1	5.10E-17	2.67E-14
SERPINE1	-6.1	9.50E-13	2.50E-10
LOC283028	-6.1	1.45E-08	1.63E-06
F2RL3	-6.1	4.05E-11	7.72E-09
LOC105372548	-6.1	3.28E-04	8.27E-03
SPTB	-6.1	2.31E-33	1.69E-29
LRIG2-DT	-6.1	2.60E-04	6.96E-03
LINC02365	-6.1	3.30E-04	8.31E-03
DDX3Y	-6.1	5.90E-05	2.24E-03
LINC00595	-6.1	3.01E-03	3.73E-02
PRSS22	-6.1	1.79E-04	5.29E-03
TUBA8	-6.1	9.89E-17	4.96E-14
MMD	-6.0	3.08E-14	1.02E-11
TMPRSS11BNL	-6.0	7.59E-04	1.50E-02
LOC107986370	-6.0	2.11E-03	2.99E-02
SLC35D3	-6.0	8.01E-07	6.07E-05
RNASE3	-6.0	1.29E-05	6.52E-04
LAMC2	-6.0	2.87E-06	1.81E-04
LOC105376034	-6.0	2.11E-03	2.99E-02
LOC107985734	-6.0	1.22E-03	2.07E-02
PF4V1	-6.0	7.03E-07	5.43E-05
LOC107986852	-6.0	8.57E-04	1.63E-02
GFI1B	-6.0	3.05E-15	1.14E-12
LOC105378937	-6.0	4.09E-03	4.51E-02
HBQ1	-6.0	3.20E-04	8.13E-03
GAS1RR	-6.0	5.06E-04	1.13E-02
FN1	-5.9	7.60E-09	9.18E-07
BAMBI	-5.9	6.76E-08	6.62E-06
OR5AL1	-5.9	2.37E-03	3.19E-02
SPDYC	-5.9	1.22E-05	6.20E-04
PTCRA	-5.9	1.61E-11	3.38E-09
DUSP15	-5.9	3.22E-06	2.00E-04
CAVIN1	-5.9	6.12E-07	4.81E-05
NANOG	-5.9	1.20E-03	2.05E-02
LOC107985253	-5.9	1.10E-04	3.66E-03
KRT18	-5.9	1.27E-06	8.98E-05
C1orf198	-5.9	6.24E-20	5.57E-17
MYL1	-5.8	1.30E-03	2.16E-02
F13A1	-5.8	7.65E-22	9.34E-19
MYL4	-5.8	4.18E-05	1.70E-03

LOC101930421	-5.8	5.07E-03	5.15E-02
DMTN	-5.8	8.66E-20	7.55E-17
OVOL3	-5.8	3.22E-04	8.16E-03
LINC01815	-5.8	1.65E-03	2.51E-02
LOC105378500	-5.8	4.10E-08	4.16E-06
NAT8B	-5.8	8.51E-07	6.41E-05
TDRP	-5.8	2.92E-10	4.88E-08
DDX11L1	-5.8	1.01E-03	1.82E-02
TSPAN33	-5.7	3.70E-14	1.20E-11
MAP3K7CL	-5.7	3.53E-16	1.58E-13
PLOD2	-5.7	2.04E-11	4.20E-09
PDE3A	-5.7	2.32E-12	5.73E-10
HSD3B1	-5.7	5.97E-05	2.26E-03
OR2W3	-5.7	1.83E-08	2.01E-06
TRAPPC3L	-5.7	9.53E-08	9.08E-06
CRACD	-5.7	7.75E-14	2.34E-11
CYP2G1P	-5.7	3.18E-04	8.12E-03
PROSER2	-5.7	4.42E-14	1.41E-11
PRNT	-5.7	6.63E-04	1.35E-02
PKHD1L1	-5.7	3.36E-15	1.24E-12
H3C12	-5.7	3.32E-05	1.41E-03
GP5	-5.7	6.21E-12	1.38E-09
FAM131C	-5.7	4.41E-03	4.75E-02
VIL1	-5.7	2.65E-17	1.49E-14
TFPI2	-5.7	1.02E-04	3.47E-03
DNM3	-5.7	5.46E-18	3.63E-15
MGP	-5.7	4.52E-04	1.04E-02
LOC105371661	-5.7	9.69E-07	7.13E-05
GABRD	-5.7	7.54E-06	4.13E-04
LOC105376213	-5.7	9.74E-04	1.78E-02
LOC107984572	-5.7	3.92E-03	4.40E-02
GNAZ	-5.7	1.90E-20	1.88E-17
EGFL7	-5.7	1.00E-20	1.02E-17
ZNF837	-5.6	3.78E-06	2.29E-04
DAB2	-5.6	3.54E-17	1.88E-14
PBX1	-5.6	3.11E-17	1.72E-14
ABCC6P2	-5.6	3.13E-03	3.81E-02
LOC283214	-5.6	1.27E-03	2.12E-02
LOC107986194	-5.6	3.80E-03	4.32E-02
LOC107985029	-5.6	6.84E-04	1.38E-02
CAV2	-5.6	6.96E-07	5.38E-05
LOC105374465	-5.6	1.20E-03	2.05E-02
LOC105373914	-5.6	2.50E-03	3.31E-02
TM4SF1	-5.6	1.07E-09	1.56E-07
ESAM	-5.6	1.63E-15	6.35E-13
LOC105379064	-5.6	3.26E-03	3.92E-02
EHD3	-5.6	4.46E-16	1.97E-13

FAM183A	-5.6	5.01E-05	1.97E-03
LINC01579	-5.6	2.09E-03	2.98E-02
LOC105377276	-5.6	5.47E-04	1.18E-02
LOC112268191	-5.6	3.66E-03	4.22E-02
TCP11	-5.6	1.05E-03	1.88E-02
PSORS1C1	-5.6	4.50E-05	1.80E-03
SAMD14	-5.6	1.05E-09	1.53E-07
LOC105375542	-5.6	7.03E-04	1.41E-02
MGC34796	-5.6	3.77E-03	4.30E-02
LOC105372711	-5.5	1.16E-05	5.98E-04
COL8A1	-5.5	5.43E-06	3.10E-04
F3	-5.5	3.84E-06	2.31E-04
ASAP2	-5.5	2.68E-20	2.51E-17
C15orf54	-5.5	2.28E-09	3.11E-07
SH3TC2	-5.5	1.01E-17	6.43E-15
LOC105369969	-5.5	3.66E-03	4.22E-02
AGAP11	-5.5	5.87E-04	1.25E-02
LCE2A	-5.5	5.10E-03	5.17E-02
LOC105372479	-5.5	1.16E-03	2.01E-02
MYZAP	-5.5	7.47E-05	2.71E-03
LOC105372719	-5.5	2.54E-04	6.86E-03
GMPR	-5.5	8.16E-14	2.42E-11
P2RY1	-5.5	4.25E-12	9.79E-10
LTF	-5.5	5.56E-13	1.52E-10
MMP8	-5.4	2.36E-10	4.01E-08
ADAM21P1	-5.4	3.86E-03	4.36E-02
LOC101929538	-5.4	2.36E-05	1.07E-03
TMCC2	-5.4	5.33E-17	2.75E-14
C9orf170	-5.4	6.22E-04	1.29E-02
PKD2L1	-5.4	1.80E-05	8.60E-04
DENND2C	-5.4	7.59E-10	1.15E-07
MAP1A	-5.4	6.20E-22	7.82E-19
LOC728975	-5.4	2.50E-11	5.01E-09
LOC105370767	-5.4	4.24E-03	4.62E-02
OR56B1	-5.4	2.37E-03	3.20E-02
GAS2L1	-5.4	7.88E-18	5.15E-15
AZU1	-5.4	1.99E-07	1.73E-05
LOC283194	-5.4	3.84E-08	3.95E-06
LOC643387	-5.4	9.74E-04	1.78E-02
CABP5	-5.4	7.39E-07	5.66E-05
LGALSL	-5.4	1.74E-19	1.45E-16
SLC7A13	-5.4	1.23E-04	3.96E-03
PARD3	-5.4	4.77E-12	1.09E-09
LOC151760	-5.4	1.81E-04	5.30E-03
LINC02246	-5.4	6.59E-06	3.69E-04
PTGS1	-5.4	1.41E-22	1.92E-19
LINC01087	-5.4	3.15E-03	3.83E-02

LOC105369194	-5.4	4.73E-09	6.08E-07
SNAI2	-5.4	1.21E-03	2.07E-02
LOC105374384	-5.4	2.64E-03	3.42E-02
ANKRD1	-5.4	4.44E-04	1.03E-02
MAOB	-5.3	2.69E-11	5.32E-09
LOC101929622	-5.3	5.60E-03	5.46E-02
OLFM4	-5.3	8.02E-09	9.62E-07
JAM3	-5.3	1.97E-13	5.55E-11
HGD	-5.3	1.10E-10	1.95E-08
LOC112268215	-5.3	1.70E-03	2.57E-02
LOC105375051	-5.3	3.65E-04	8.91E-03
HRAT92	-5.3	2.95E-08	3.10E-06
GRB14	-5.3	4.09E-06	2.43E-04
LOC105377967	-5.3	7.75E-04	1.52E-02
LINC00184	-5.3	3.36E-03	3.99E-02
MGLL	-5.3	6.94E-19	5.19E-16
TTC41P	-5.3	4.59E-04	1.05E-02
LOC105377275	-5.3	5.07E-09	6.36E-07
CFAP161	-5.3	1.80E-07	1.59E-05
LOC100420587	-5.3	2.42E-05	1.10E-03
ANK1	-5.3	1.95E-20	1.88E-17
LOC105371028	-5.3	1.76E-09	2.47E-07
CDC14B	-5.3	1.15E-12	3.00E-10
LOC100507388	-5.3	1.24E-04	3.99E-03
C4orf36	-5.3	5.26E-04	1.15E-02
IZUMO2	-5.3	3.88E-03	4.37E-02
LINC01255	-5.3	3.40E-03	4.02E-02
GP1BA	-5.2	4.07E-12	9.45E-10
LOC107987126	-5.2	6.52E-04	1.33E-02
ISLR	-5.2	3.01E-04	7.79E-03
LINC01714	-5.2	4.24E-03	4.62E-02
LOC728024	-5.2	1.23E-03	2.09E-02
GNG11	-5.2	1.97E-10	3.44E-08
H2AC13	-5.2	1.12E-04	3.72E-03
P2RY12	-5.2	3.92E-08	4.00E-06
ZDHHC8P1	-5.2	1.47E-04	4.58E-03
SNCA	-5.2	1.92E-16	9.13E-14
GUCY1A1	-5.2	6.30E-16	2.71E-13
CTSG	-5.2	4.72E-05	1.86E-03
RASSF10	-5.2	2.17E-04	6.12E-03
LOC112267944	-5.2	3.19E-03	3.86E-02
LOC105375623	-5.2	3.12E-03	3.81E-02
LOC105378114	-5.2	3.03E-03	3.74E-02
LOC105372569	-5.2	5.22E-03	5.25E-02
TRHDE-AS1	-5.2	3.12E-06	1.94E-04
LANCL3	-5.2	5.19E-13	1.43E-10
DEPDC1-AS1	-5.2	1.21E-03	2.06E-02

BPI	-5.2	3.27E-10	5.37E-08
S100A16	-5.2	1.17E-03	2.02E-02
HJURP	-5.2	1.49E-09	2.12E-07
LOC105378012	-5.2	2.90E-03	3.65E-02
PITX2	-5.2	3.35E-05	1.42E-03
MAP1B	-5.2	8.20E-14	2.42E-11
KRT7	-5.2	1.35E-04	4.25E-03
LOC105377684	-5.1	8.42E-04	1.61E-02
CEACAM6	-5.1	6.07E-08	6.00E-06
LOC105372328	-5.1	5.05E-03	5.13E-02
RAB6B	-5.1	1.99E-09	2.75E-07
SLC8A3	-5.1	2.06E-11	4.22E-09
LOC107984321	-5.1	1.19E-03	2.03E-02
NDUFA4L2	-5.1	3.67E-03	4.23E-02
CACNA1G-AS1	-5.1	1.16E-03	2.01E-02
ZFPM2-AS1	-5.1	3.30E-03	3.94E-02
SCN7A	-5.1	1.33E-04	4.20E-03
CLMAT3	-5.1	1.46E-03	2.32E-02
OXTR	-5.1	2.34E-12	5.76E-10
CPN2	-5.1	2.51E-03	3.32E-02
FAXDC2	-5.1	5.69E-19	4.34E-16
PRTFDC1	-5.1	1.18E-08	1.36E-06
LOC440896	-5.1	3.72E-03	4.26E-02
GNG12	-5.1	3.60E-04	8.86E-03
LOC107986789	-5.1	2.30E-03	3.15E-02
IGFBP4	-5.1	5.07E-06	2.93E-04
SLCO5A1	-5.1	5.82E-10	9.21E-08
VWF	-5.1	4.76E-14	1.50E-11
LOC101928940	-5.1	1.53E-03	2.40E-02
CA2	-5.1	5.33E-09	6.65E-07
LOC105371606	-5.1	4.51E-04	1.04E-02
KLHL7-DT	-5.1	5.40E-04	1.17E-02
XK	-5.1	8.61E-12	1.87E-09
CEACAM8	-5.0	2.22E-11	4.52E-09
CLEC4O	-5.0	4.06E-03	4.49E-02
CATSPER4	-5.0	7.80E-04	1.53E-02
MPIG6B	-5.0	7.71E-19	5.53E-16
LOC107985770	-5.0	1.56E-08	1.73E-06
LINC00941	-5.0	1.50E-03	2.36E-02
LOC401324	-5.0	2.48E-03	3.29E-02
LOC101927098	-5.0	1.06E-03	1.89E-02
OR10AC1	-5.0	3.22E-03	3.88E-02
H3C10	-5.0	1.15E-08	1.34E-06
ENDOD1	-5.0	2.69E-16	1.23E-13
MMP1	-5.0	5.28E-05	2.05E-03
HOXC10	-5.0	3.08E-03	3.78E-02
LOC100506532	-5.0	3.48E-04	8.66E-03

WASF3	-5.0	9.37E-09	1.10E-06
ITGA9-AS1	-5.0	2.44E-05	1.10E-03
LOC105369439	-5.0	5.00E-03	5.11E-02
LOC105374995	-5.0	2.83E-04	7.42E-03
INTS4P1	-5.0	1.76E-03	2.63E-02
LOC105374329	-5.0	2.53E-03	3.33E-02
DEFA4	-5.0	8.93E-05	3.12E-03
PNCK	-5.0	2.53E-04	6.85E-03
POLR2J3	-5.0	8.43E-04	1.61E-02
LOC105376201	-5.0	1.26E-03	2.11E-02
THBS1	-5.0	1.96E-19	1.56E-16
LCN2	-5.0	6.51E-08	6.40E-06
LOC105372696	-5.0	5.02E-03	5.12E-02
SLC13A2	-5.0	1.12E-03	1.96E-02
TEAD1	-5.0	1.27E-05	6.45E-04
NT5M	-5.0	2.53E-12	6.12E-10
LOC105376567	-5.0	4.76E-04	1.08E-02
ABCC3	-5.0	1.38E-13	4.00E-11
LHX5	-5.0	5.21E-04	1.15E-02
LOC105372353	-5.0	1.96E-03	2.84E-02
LOC107986675	-4.9	3.15E-05	1.36E-03
VEPH1	-4.9	1.24E-08	1.42E-06
CCDC158	-4.9	1.40E-04	4.37E-03
LOC105370974	-4.9	1.44E-03	2.30E-02
TAC3	-4.9	1.57E-03	2.44E-02
SDC4	-4.9	2.80E-11	5.45E-09
LRRC26	-4.9	4.74E-03	4.95E-02
LOC105371520	-4.9	3.67E-03	4.22E-02
PDLIM1	-4.9	1.87E-12	4.75E-10
CTAGE1	-4.9	1.06E-03	1.89E-02
LOC105378608	-4.9	1.76E-03	2.64E-02
COL1A1	-4.9	7.30E-07	5.60E-05
LOC102724355	-4.9	4.93E-03	5.08E-02
HS6ST2	-4.9	5.23E-05	2.03E-03
AFAP1-AS1	-4.9	4.69E-05	1.86E-03
CRISP3	-4.9	5.72E-07	4.54E-05
LOC102723395	-4.9	2.44E-04	6.65E-03
EGLN3	-4.9	5.15E-08	5.14E-06
MUC7	-4.9	3.46E-03	4.07E-02
LOC730338	-4.9	3.07E-03	3.77E-02
VN1R4	-4.9	3.18E-03	3.85E-02
PTMAP11	-4.9	1.42E-03	2.27E-02
LOC100130698	-4.9	4.10E-04	9.72E-03
LOC286083	-4.9	5.21E-03	5.24E-02
ACCSL	-4.9	6.19E-04	1.29E-02
LOC105378781	-4.9	3.53E-05	1.48E-03
LOC100128398	-4.9	1.58E-04	4.85E-03

NEXN	-4.9	9.80E-12	2.11E-09
VSIG2	-4.8	1.84E-09	2.55E-07
LOC107984179	-4.8	1.25E-03	2.10E-02
PLXNB3	-4.8	1.04E-15	4.23E-13
INKA2-AS1	-4.8	6.16E-05	2.32E-03
LOC105376158	-4.8	2.19E-03	3.05E-02
PTPRF	-4.8	4.08E-12	9.45E-10
CAV1	-4.8	7.84E-05	2.81E-03
GPRC5A	-4.8	5.11E-06	2.94E-04
PGRMC1	-4.8	6.10E-14	1.86E-11
TTC7B	-4.8	8.32E-16	3.50E-13
SSX2IP	-4.8	1.10E-11	2.36E-09
IGFBP2	-4.8	1.38E-06	9.62E-05
PVALB	-4.8	1.33E-03	2.19E-02
SLC24A3	-4.8	2.51E-12	6.12E-10
GATA1	-4.8	3.17E-09	4.18E-07
CEACAM5	-4.8	9.64E-04	1.76E-02
IL11	-4.8	1.78E-03	2.66E-02
FAM83D	-4.8	1.26E-08	1.43E-06
H3C3	-4.8	1.07E-04	3.58E-03
LOC105370481	-4.8	3.74E-03	4.28E-02
IGF2BP3	-4.8	2.61E-09	3.50E-07
FGF17	-4.8	1.53E-05	7.49E-04
CXCR2P1	-4.8	1.12E-11	2.39E-09
SLC16A9	-4.8	3.80E-03	4.31E-02
PCCA-DT	-4.8	4.91E-03	5.06E-02
LOC729451	-4.8	2.54E-05	1.13E-03
FGF5	-4.8	1.27E-03	2.12E-02
FAM9A	-4.8	2.34E-03	3.17E-02
H2BC11	-4.8	3.10E-08	3.25E-06
LINC02793	-4.8	9.43E-04	1.74E-02
FGF2	-4.8	2.10E-06	1.38E-04
SAA1	-4.8	5.38E-03	5.34E-02
LOC107986181	-4.8	2.05E-03	2.93E-02
RAB13	-4.8	8.84E-08	8.52E-06
LINC01280	-4.8	4.89E-03	5.05E-02
MTURN	-4.7	2.22E-16	1.04E-13
NPY2R	-4.7	1.57E-03	2.44E-02
LOC101929507	-4.7	4.03E-04	9.62E-03
EHD2	-4.7	2.43E-07	2.07E-05
SLC2A2	-4.7	2.48E-03	3.29E-02
LOC101060588	-4.7	4.27E-03	4.64E-02
LINC01896	-4.7	8.75E-04	1.64E-02
LINC02284	-4.7	9.62E-09	1.13E-06
LOC105378909	-4.7	4.78E-08	4.81E-06
CTNNAL1	-4.7	2.17E-09	2.98E-07
ANKRD33B	-4.7	1.42E-18	1.00E-15

LHFPL6	-4.7	5.95E-09	7.33E-07
RNF208	-4.7	3.06E-05	1.33E-03
SLC8A2	-4.7	1.35E-05	6.73E-04
THCAT158	-4.7	5.68E-05	2.18E-03
ACRBP	-4.7	3.11E-14	1.02E-11
LOC105376810	-4.7	2.77E-03	3.53E-02
LINC02609	-4.7	1.72E-03	2.60E-02
LINC00316	-4.7	5.87E-04	1.25E-02
DDAH1	-4.7	1.16E-05	5.98E-04
DZIP1	-4.7	3.50E-04	8.67E-03
MYADML2	-4.7	2.18E-03	3.05E-02
LOC105373080	-4.7	4.02E-03	4.46E-02
PIWIL3	-4.7	2.53E-03	3.33E-02
LOC339685	-4.7	2.45E-04	6.67E-03
LOC102724977	-4.7	3.90E-03	4.38E-02
CYP4Z1	-4.7	3.50E-03	4.11E-02
LINC02558	-4.7	4.88E-03	5.05E-02
LOC105378040	-4.6	5.33E-03	5.31E-02
ELANE	-4.6	3.04E-05	1.32E-03
LOC107984271	-4.6	2.66E-03	3.44E-02
THCAT155	-4.6	4.09E-03	4.51E-02
ALAS2	-4.6	1.30E-04	4.13E-03
TNS1	-4.6	6.89E-17	3.50E-14
LOC107985206	-4.6	1.31E-03	2.17E-02
LOC107985270	-4.6	4.40E-03	4.75E-02
VCL	-4.6	5.32E-20	4.87E-17
LINC00645	-4.6	2.51E-03	3.32E-02
COL4A2	-4.6	1.20E-05	6.13E-04
STON1	-4.6	1.21E-06	8.63E-05
CAMP	-4.6	3.49E-08	3.62E-06
ACER2	-4.6	3.86E-08	3.96E-06
LOC107985524	-4.6	5.06E-04	1.13E-02
TBXA2R	-4.6	9.96E-11	1.80E-08
LOC107986788	-4.6	7.26E-04	1.45E-02
HSPA4L	-4.6	1.22E-05	6.22E-04
RAB38	-4.6	1.65E-03	2.51E-02
ARHGAP18	-4.6	2.98E-14	1.00E-11
IRX3	-4.6	9.58E-06	5.08E-04
FAM155A-IT1	-4.6	5.28E-03	5.28E-02
LOC107987228	-4.6	3.24E-05	1.39E-03
STC1	-4.6	1.76E-04	5.23E-03
CCN2	-4.6	1.27E-03	2.12E-02
SENP3	-4.6	1.38E-03	2.24E-02
NR2F2	-4.6	5.56E-06	3.17E-04
LOC105370740	-4.6	2.36E-03	3.18E-02
MEIS1-AS2	-4.6	2.02E-03	2.90E-02
EXOC3L2	-4.6	4.08E-07	3.33E-05

ARHGEF12	-4.6	1.44E-27	3.51E-24
NUP62CL	-4.5	4.26E-03	4.64E-02
LOC105376088	-4.5	4.39E-03	4.74E-02
COL3A1	-4.5	3.63E-05	1.51E-03
TUBB2B	-4.5	1.40E-03	2.25E-02
SPX	-4.5	1.83E-04	5.35E-03
MARCHF4	-4.5	9.39E-05	3.24E-03
LOC102723740	-4.5	6.38E-04	1.31E-02
FOXI2	-4.5	3.60E-03	4.18E-02
LRRC3C	-4.5	7.94E-08	7.72E-06
LOC105376214	-4.5	2.27E-08	2.46E-06
LOC101928338	-4.5	2.14E-03	3.02E-02
ATAD3C	-4.5	7.71E-04	1.52E-02
SLC6A4	-4.5	1.07E-07	1.01E-05
CPXM1	-4.5	2.67E-04	7.10E-03
ARHGAP21	-4.5	1.98E-30	7.26E-27
BEX3	-4.5	3.73E-10	6.07E-08
KRT15	-4.5	1.47E-03	2.33E-02
LOC107984628	-4.5	3.53E-03	4.13E-02
CD24	-4.5	1.11E-09	1.61E-07
LRP12	-4.5	5.36E-12	1.20E-09
MROH2A	-4.5	3.05E-05	1.32E-03
LOC112268038	-4.5	1.62E-04	4.93E-03
ANLN	-4.5	5.11E-06	2.94E-04
MPO	-4.5	2.54E-08	2.71E-06
TACSTD2	-4.5	3.91E-06	2.34E-04
AADACL3	-4.5	6.81E-04	1.38E-02
LOC107984301	-4.5	2.25E-03	3.11E-02
PSD3	-4.5	8.10E-11	1.50E-08
LOC105370841	-4.5	5.28E-03	5.28E-02
ADCY6	-4.5	7.19E-11	1.34E-08
LOC107984254	-4.5	2.53E-04	6.85E-03
CRMP1	-4.5	4.91E-06	2.84E-04
LOC105370844	-4.5	2.70E-04	7.16E-03
ZNF385D	-4.5	2.89E-07	2.41E-05
LHX1-DT	-4.5	9.97E-04	1.81E-02
KIFC3	-4.5	1.68E-12	4.32E-10
KCNJ4	-4.4	1.70E-04	5.10E-03
RND3	-4.4	9.94E-04	1.80E-02
H2BC3	-4.4	1.06E-03	1.89E-02
MPPED2-AS1	-4.4	2.34E-03	3.17E-02
LOC105374985	-4.4	8.86E-06	4.76E-04
REN	-4.4	8.44E-04	1.61E-02
CDH11	-4.4	2.46E-05	1.11E-03
TTLL7	-4.4	2.31E-09	3.14E-07
CDH6	-4.4	1.69E-06	1.14E-04
FGF7P3	-4.4	3.22E-03	3.88E-02

MYOSLID	-4.4	1.75E-04	5.21E-03
IGF2BP2	-4.4	2.24E-11	4.54E-09
ARSH	-4.4	2.29E-03	3.14E-02
LOC105376475	-4.4	2.91E-03	3.65E-02
LOC105372245	-4.4	1.82E-04	5.33E-03
LOC105375120	-4.4	4.18E-05	1.70E-03
SMTN	-4.4	2.67E-10	4.52E-08
LOC105378161	-4.4	1.42E-03	2.28E-02
DUSP27	-4.4	8.36E-05	2.97E-03
LOC101927995	-4.4	1.40E-03	2.25E-02
IRS1	-4.4	1.33E-09	1.91E-07
ITGA2	-4.4	1.05E-10	1.89E-08
LOC105378901	-4.4	1.87E-04	5.43E-03
C19orf57	-4.4	1.48E-04	4.58E-03
CYP1B1-AS1	-4.4	3.71E-03	4.26E-02
LOC105374445	-4.4	1.87E-04	5.43E-03
OR2Z1	-4.4	1.64E-03	2.50E-02
LOC102724765	-4.4	3.48E-07	2.88E-05
PRR16	-4.4	6.31E-05	2.36E-03
LRP2BP	-4.4	1.44E-04	4.50E-03
TMPRSS15	-4.4	2.55E-03	3.34E-02
SUCNR1	-4.4	8.31E-07	6.27E-05
LOC107986567	-4.4	5.58E-03	5.45E-02
PLCH1	-4.4	4.19E-07	3.40E-05
LOC105375035	-4.4	3.42E-03	4.03E-02
LOC107984851	-4.4	2.59E-04	6.95E-03
PRKG1	-4.4	3.18E-08	3.33E-06
TFAP2A	-4.4	2.82E-04	7.40E-03
SLC5A12	-4.4	1.20E-03	2.05E-02
BDNF	-4.4	1.67E-06	1.13E-04
LINC02533	-4.4	1.09E-03	1.92E-02
CHRNA3	-4.3	5.49E-03	5.41E-02
FHL1	-4.3	3.87E-11	7.41E-09
LOC105376454	-4.3	3.26E-03	3.91E-02
LOC105375207	-4.3	5.46E-03	5.39E-02
LOC105373980	-4.3	1.13E-03	1.96E-02
LURAP1L	-4.3	5.46E-04	1.18E-02
LOC105372699	-4.3	2.31E-03	3.15E-02
LOC101929268	-4.3	5.11E-03	5.17E-02
FAT1	-4.3	3.76E-06	2.28E-04
EVPL	-4.3	2.68E-05	1.19E-03
TMEM51-AS1	-4.3	3.78E-03	4.30E-02
GIPC3	-4.3	7.67E-07	5.86E-05
RAB3C	-4.3	4.37E-04	1.02E-02
MYCT1	-4.3	9.22E-07	6.87E-05
ALDH1L1-AS2	-4.3	6.83E-04	1.38E-02
LOC105372683	-4.3	3.45E-03	4.07E-02

LOC105374343	-4.3	1.64E-03	2.50E-02
COL6A3	-4.3	2.60E-09	3.50E-07
OTOS	-4.3	3.48E-03	4.09E-02
TSKU	-4.3	1.10E-03	1.94E-02
SLPI	-4.3	1.81E-04	5.30E-03
DEFA1B	-4.3	1.93E-03	2.80E-02
RUFY1	-4.3	2.57E-15	9.82E-13
PROX1-AS1	-4.3	3.08E-03	3.78E-02
DEFA3	-4.3	4.67E-05	1.85E-03
KCNG4	-4.3	1.22E-04	3.94E-03
GJA1	-4.3	2.38E-03	3.20E-02
GPR45	-4.3	1.94E-03	2.81E-02
LOC105370167	-4.2	1.86E-03	2.74E-02
ENAH	-4.2	1.02E-06	7.48E-05
SIX2	-4.2	7.02E-05	2.58E-03
ETV5	-4.2	3.62E-04	8.87E-03
C1orf116	-4.2	2.67E-09	3.56E-07
LOC105371896	-4.2	4.65E-03	4.89E-02
HOXD1	-4.2	3.62E-03	4.19E-02
SLC5A8	-4.2	1.04E-03	1.87E-02
POU4F2	-4.2	8.35E-04	1.60E-02
SFTA3	-4.2	1.18E-03	2.03E-02
BFSP2	-4.2	2.64E-03	3.42E-02
PRTN3	-4.2	1.05E-03	1.88E-02
COL6A1	-4.2	2.01E-05	9.41E-04
GP6	-4.2	1.12E-13	3.28E-11
RDH14	-4.2	2.04E-03	2.92E-02
CCND1	-4.2	2.95E-05	1.29E-03
IGFBP5	-4.2	2.84E-04	7.45E-03
INHBA	-4.2	1.85E-06	1.24E-04
MSRB3	-4.2	5.84E-14	1.80E-11
RGS18	-4.2	2.10E-15	8.10E-13
PSAPL1	-4.2	2.64E-06	1.69E-04
TMEM158	-4.2	3.49E-05	1.47E-03
CLGN	-4.2	2.87E-04	7.50E-03
FLNC	-4.2	1.65E-05	7.94E-04
LOC107984031	-4.2	4.74E-03	4.95E-02
PTGIR	-4.2	2.04E-08	2.23E-06
LOC107985467	-4.2	5.47E-03	5.39E-02
CDC14C	-4.2	2.83E-03	3.58E-02
PDGFRA	-4.2	1.02E-09	1.50E-07
FNBP1L	-4.2	2.48E-09	3.36E-07
TPM1	-4.2	6.93E-15	2.51E-12
MS4A3	-4.2	1.30E-05	6.56E-04
TBX2	-4.2	2.21E-04	6.19E-03
LOC105370998	-4.2	6.14E-04	1.28E-02
EFEMP1	-4.2	1.36E-03	2.22E-02

WWTR1	-4.2	1.57E-04	4.83E-03
LARGE-AS1	-4.2	2.16E-03	3.03E-02
TAGLN3	-4.2	1.85E-03	2.73E-02
SHROOM4	-4.1	3.26E-10	5.37E-08
ITGA3	-4.1	3.70E-05	1.54E-03
TWSG1	-4.1	1.93E-08	2.12E-06
LOC105373693	-4.1	1.75E-03	2.62E-02
ABCC4	-4.1	3.61E-11	6.98E-09
LOC107984634	-4.1	5.15E-04	1.14E-02
COL1A2	-4.1	1.20E-04	3.90E-03
LOC105376995	-4.1	1.23E-08	1.40E-06
INAFM2	-4.1	1.50E-14	5.20E-12
ZFPM2	-4.1	7.57E-04	1.50E-02
ADGRF5	-4.1	5.19E-04	1.14E-02
LINC01923	-4.1	2.56E-03	3.35E-02
LOC105378570	-4.1	3.48E-04	8.66E-03
AMELX	-4.1	2.57E-03	3.35E-02
RSPO3	-4.1	8.93E-05	3.12E-03
TUBB2A	-4.1	4.14E-05	1.69E-03
FJX1	-4.1	1.01E-03	1.82E-02
LOC102724960	-4.1	4.46E-03	4.78E-02
BMP4	-4.1	3.93E-04	9.47E-03
LOC101927145	-4.1	2.32E-03	3.16E-02
CTDSPL	-4.1	1.75E-13	4.98E-11
TMEFF2	-4.1	2.14E-03	3.02E-02
APLNR	-4.1	1.74E-03	2.62E-02
APBB2	-4.1	4.43E-05	1.78E-03
SPSB1	-4.1	2.14E-06	1.40E-04
CDC42BPA	-4.1	3.05E-10	5.07E-08
GRAP2	-4.1	3.45E-15	1.26E-12
ALOX12	-4.1	2.35E-13	6.57E-11
LOC107984564	-4.1	3.06E-03	3.77E-02
LOC107986717	-4.1	1.44E-03	2.30E-02
VWA1	-4.1	8.17E-04	1.58E-02
LOC105372203	-4.1	2.47E-03	3.28E-02
LOC105372411	-4.0	1.35E-03	2.20E-02
LAPTM4B	-4.0	1.05E-07	9.95E-06
SSPN	-4.0	5.29E-04	1.16E-02
PLA2G12A	-4.0	9.24E-10	1.38E-07
PIGR	-4.0	1.34E-03	2.20E-02
LOC107986438	-4.0	5.10E-03	5.17E-02
LOC105374577	-4.0	2.48E-03	3.29E-02
SPINK8	-4.0	1.83E-03	2.71E-02
FBN1	-4.0	1.50E-05	7.34E-04
VWCE	-4.0	2.40E-04	6.58E-03
HSPB9	-4.0	3.58E-03	4.16E-02
EN1	-4.0	9.16E-04	1.70E-02

DNAJC6	-4.0	4.30E-08	4.35E-06
PANX1	-4.0	1.26E-14	4.38E-12
PXDC1	-4.0	2.69E-07	2.27E-05
GLIS1	-4.0	3.22E-04	8.16E-03
PTK2	-4.0	8.46E-11	1.56E-08
ABCA13	-4.0	3.50E-12	8.27E-10
TRIM31	-4.0	3.29E-03	3.93E-02
LOC102724805	-4.0	4.43E-05	1.78E-03
CALHM5	-4.0	1.45E-06	1.00E-04
AMOTL2	-4.0	1.27E-03	2.12E-02
TRIM10	-4.0	2.81E-06	1.78E-04
CENPE	-4.0	4.93E-05	1.94E-03
PDGFB	-4.0	2.33E-08	2.51E-06
SELENBP1	-4.0	9.40E-04	1.74E-02
GRHL1	-4.0	9.25E-08	8.84E-06
CREB3L1	-4.0	7.28E-04	1.45E-02
ANO6	-4.0	2.60E-16	1.20E-13
MITF	-4.0	4.92E-12	1.12E-09
MAGI2-AS3	-4.0	2.76E-06	1.76E-04
FAM228A	-4.0	2.16E-03	3.03E-02
LOC112268079	-4.0	1.02E-03	1.84E-02
LOC105374257	-4.0	1.08E-03	1.92E-02
KCNK3	-4.0	1.61E-03	2.48E-02
LRRTM1	-4.0	7.66E-04	1.51E-02
MET	-4.0	2.19E-04	6.16E-03
LAMP5	-4.0	1.46E-04	4.55E-03
LOC105374986	-3.9	9.29E-07	6.91E-05
ZC3HAV1L	-3.9	1.86E-07	1.63E-05
HOXC9	-3.9	5.95E-04	1.26E-02
OVCH1-AS1	-3.9	1.77E-03	2.64E-02
H1-6	-3.9	3.59E-03	4.17E-02
PYCR1	-3.9	9.82E-04	1.79E-02
NYX	-3.9	2.06E-03	2.94E-02
LOC105376541	-3.9	2.43E-03	3.24E-02
GPM6A	-3.9	4.63E-03	4.88E-02
LOC107984449	-3.9	2.97E-03	3.70E-02
RGS6	-3.9	7.59E-10	1.15E-07
FRMD3	-3.9	3.76E-12	8.82E-10
LINC01181	-3.9	2.31E-03	3.15E-02
KCNE5	-3.9	2.86E-03	3.61E-02
PCAT6	-3.9	4.03E-03	4.47E-02
UTY	-3.9	1.65E-04	5.00E-03
GRIA4	-3.9	8.08E-05	2.88E-03
LOC102724081	-3.9	1.16E-03	2.01E-02
LGALS12	-3.9	4.74E-08	4.78E-06
CYP4F2	-3.9	2.72E-03	3.49E-02
LOC105374200	-3.9	3.93E-03	4.40E-02

CLEC1B	-3.9	1.49E-06	1.03E-04
CDKN3	-3.9	2.94E-03	3.68E-02
HBG1	-3.9	4.62E-03	4.88E-02
ROBO2	-3.9	3.10E-04	7.97E-03
LOC105375749	-3.9	2.72E-03	3.49E-02
PLPPR5	-3.9	1.85E-03	2.73E-02
KCND3	-3.9	1.75E-07	1.56E-05
FGF12	-3.9	1.61E-03	2.48E-02
LOC101927392	-3.9	5.32E-03	5.30E-02
LOC105375184	-3.9	4.41E-03	4.75E-02
TOP2A	-3.9	3.46E-05	1.46E-03
PCDHGA11	-3.9	3.71E-03	4.26E-02
LOC107985174	-3.9	1.60E-04	4.89E-03
LOC107984270	-3.9	2.50E-03	3.31E-02
BCAR1	-3.9	4.50E-05	1.80E-03
OVOL1	-3.9	1.30E-03	2.15E-02
SHROOM3	-3.9	7.43E-04	1.47E-02
LINC01030	-3.9	3.12E-03	3.81E-02
PLS3	-3.9	3.75E-04	9.11E-03
H3C2	-3.9	2.97E-05	1.30E-03
LOC101927978	-3.9	6.03E-04	1.27E-02
SYNM	-3.9	9.95E-11	1.80E-08
HNF1B	-3.9	1.28E-03	2.13E-02
NPHS1	-3.9	9.90E-04	1.80E-02
GPR1	-3.9	2.93E-03	3.68E-02
CNN3	-3.9	1.30E-04	4.12E-03
PRR35	-3.8	2.15E-04	6.10E-03
VWA2	-3.8	1.94E-03	2.81E-02
DCN	-3.8	1.05E-03	1.88E-02
PRKAR1B	-3.8	7.45E-10	1.14E-07
GBX2	-3.8	7.54E-06	4.13E-04
EMILIN1	-3.8	1.15E-07	1.07E-05
PCDHGA3	-3.8	1.80E-03	2.67E-02
LOC105377714	-3.8	3.14E-03	3.82E-02
LOC107985342	-3.8	3.72E-03	4.27E-02
LOC105375610	-3.8	3.36E-03	3.99E-02
AFAP1L2	-3.8	3.51E-08	3.64E-06
NCKAP1	-3.8	1.82E-09	2.53E-07
MTCL1	-3.8	1.08E-07	1.01E-05
ZNF213-AS1	-3.8	1.48E-04	4.59E-03
CNN1	-3.8	1.95E-04	5.63E-03
LIMS1	-3.8	2.14E-12	5.32E-10
TCN1	-3.8	5.20E-05	2.02E-03
PPP1R14A	-3.8	9.72E-06	5.15E-04
PARVB	-3.8	9.29E-10	1.38E-07
MYOM1	-3.8	6.41E-13	1.71E-10
MLPH	-3.8	1.76E-04	5.24E-03

LOC105374880	-3.8	4.58E-03	4.85E-02
SLC23A3	-3.8	5.51E-04	1.19E-02
CNST	-3.8	1.92E-11	3.97E-09
PTGR1	-3.8	2.76E-03	3.52E-02
SAV1	-3.8	1.87E-10	3.27E-08
COL4A5	-3.8	1.11E-03	1.95E-02
KIRREL1	-3.8	6.25E-04	1.30E-02
A4GALT	-3.8	2.32E-03	3.16E-02
SYNPO2L	-3.8	7.70E-04	1.51E-02
INKA1	-3.8	6.59E-07	5.13E-05
LOC105374557	-3.8	4.84E-03	5.02E-02
E2F1	-3.8	5.42E-09	6.73E-07
AQP1	-3.8	2.07E-04	5.91E-03
MYEOV	-3.8	1.39E-06	9.65E-05
LRRC32	-3.8	5.32E-05	2.06E-03
EPB42	-3.8	1.30E-03	2.16E-02
PERCC1	-3.8	1.45E-03	2.32E-02
YAP1	-3.8	4.87E-04	1.10E-02
FHL2	-3.8	9.15E-10	1.37E-07
PXDN	-3.8	1.09E-04	3.65E-03
GJA4	-3.8	3.29E-04	8.30E-03
KLHL4	-3.8	2.42E-03	3.24E-02
RNF133	-3.8	5.56E-03	5.44E-02
SLC7A14	-3.7	1.13E-03	1.97E-02
ZNF781	-3.7	2.33E-03	3.16E-02
CDR2L	-3.7	1.15E-04	3.78E-03
ZWINT	-3.7	1.09E-03	1.93E-02
CLUL1	-3.7	2.94E-03	3.68E-02
AJUBA	-3.7	9.16E-05	3.18E-03
ELAVL3	-3.7	1.34E-04	4.21E-03
WNT11	-3.7	1.22E-03	2.07E-02
AR	-3.7	1.48E-07	1.35E-05
ARTN	-3.7	4.12E-03	4.54E-02
TPTEP1	-3.7	7.95E-06	4.32E-04
S100A2	-3.7	2.39E-03	3.21E-02
NME2	-3.7	4.02E-04	9.61E-03
SLC18A2	-3.7	6.23E-05	2.34E-03
IL20RB	-3.7	9.69E-04	1.77E-02
CDK1	-3.7	4.37E-04	1.02E-02
CIB2	-3.7	1.93E-03	2.80E-02
FN3K	-3.7	1.45E-06	1.00E-04
CREB3L3	-3.7	3.54E-03	4.13E-02
STAC2	-3.7	2.37E-03	3.19E-02
LOC107984429	-3.7	5.13E-03	5.18E-02
PRR15	-3.7	4.89E-03	5.06E-02
ZNF367	-3.7	7.18E-09	8.73E-07
ESPNL	-3.7	1.24E-03	2.09E-02

LOC107985720	-3.7	5.15E-03	5.20E-02
MFSD2B	-3.7	1.78E-06	1.19E-04
ADAMTS9	-3.7	9.95E-05	3.40E-03
GATA6	-3.7	3.28E-03	3.93E-02
RBPMS2	-3.7	4.21E-05	1.71E-03
LOC112268448	-3.7	2.07E-03	2.95E-02
DNM1	-3.7	1.35E-08	1.52E-06
WNK2	-3.7	4.72E-05	1.86E-03
RHD	-3.7	1.39E-03	2.24E-02
SPATA4	-3.7	2.21E-04	6.19E-03
BOK-AS1	-3.7	5.12E-03	5.17E-02
CPE	-3.7	9.61E-04	1.76E-02
PITPNM2	-3.7	5.51E-10	8.78E-08
AP1M2	-3.7	5.36E-05	2.07E-03
MARCHF2	-3.7	8.16E-14	2.42E-11
ADAMTS12	-3.7	1.79E-04	5.30E-03
XIRP2	-3.7	4.94E-06	2.86E-04
GATA2-AS1	-3.7	5.46E-04	1.18E-02
LOC105371887	-3.7	8.21E-04	1.58E-02
UGT2B11	-3.7	3.94E-03	4.41E-02
CCDC3	-3.7	9.99E-05	3.40E-03
FOXC1	-3.7	9.59E-05	3.30E-03
AXL	-3.7	4.95E-04	1.11E-02
LOC107984123	-3.7	4.15E-03	4.56E-02
EIF4E1B	-3.7	3.76E-03	4.29E-02
LOC105371970	-3.7	4.56E-03	4.85E-02
CAND2	-3.7	5.20E-04	1.14E-02
KLHDC8B	-3.6	1.07E-04	3.58E-03
LOC107984120	-3.6	4.28E-03	4.65E-02
SPTA1	-3.6	2.12E-04	6.02E-03
CEP55	-3.6	2.35E-03	3.18E-02
SH2D4A	-3.6	1.18E-03	2.03E-02
MAB21L1	-3.6	2.24E-03	3.10E-02
GSDMC	-3.6	4.57E-03	4.85E-02
ATL1	-3.6	2.68E-05	1.19E-03
OPHN1	-3.6	1.17E-08	1.36E-06
LINC00534	-3.6	5.26E-03	5.27E-02
PNMA1	-3.6	3.60E-08	3.72E-06
GGTA1P	-3.6	3.32E-05	1.41E-03
HNF4A	-3.6	2.10E-03	2.98E-02
FRRS1L	-3.6	1.15E-03	1.99E-02
NRG2	-3.6	2.38E-04	6.53E-03
YWHAH	-3.6	1.12E-15	4.52E-13
CYP11A1	-3.6	2.97E-03	3.70E-02
EFCAB6	-3.6	4.66E-04	1.06E-02
LOC105370356	-3.6	3.93E-03	4.40E-02
TOM1L1	-3.6	6.14E-04	1.28E-02

KLK11	-3.6	6.63E-04	1.35E-02
CD109	-3.6	4.39E-05	1.77E-03
FMOD	-3.6	5.17E-03	5.22E-02
SSUH2	-3.6	2.67E-04	7.10E-03
LOC107985686	-3.6	6.60E-04	1.35E-02
LIMCH1	-3.6	4.41E-05	1.78E-03
LOC105377700	-3.6	4.41E-03	4.75E-02
GAS1	-3.6	6.34E-04	1.31E-02
UROC1	-3.6	1.14E-03	1.98E-02
SHANK3	-3.6	4.62E-05	1.84E-03
TLCD4	-3.6	6.42E-04	1.32E-02
INF2	-3.6	2.39E-13	6.63E-11
COL5A1	-3.6	2.75E-05	1.22E-03
EDNRB	-3.6	1.16E-03	2.01E-02
COL12A1	-3.6	4.80E-04	1.08E-02
CD276	-3.6	1.11E-03	1.94E-02
ZNF542P	-3.6	1.01E-05	5.31E-04
PLEKHA8P1	-3.6	9.86E-06	5.20E-04
CPNE5	-3.6	2.45E-08	2.63E-06
CTTNBP2	-3.6	1.25E-03	2.10E-02
TCEAL9	-3.6	1.12E-03	1.96E-02
NKX3-2	-3.6	4.20E-03	4.59E-02
CFAP74	-3.6	5.81E-04	1.24E-02
LOC105370691	-3.6	5.27E-03	5.28E-02
LINC02280	-3.6	7.07E-04	1.42E-02
HES3	-3.6	1.25E-03	2.10E-02
ATP1B1	-3.6	1.80E-05	8.58E-04
ATP13A5	-3.6	2.91E-03	3.66E-02
ZNF502	-3.6	3.76E-03	4.29E-02
PCDH18	-3.6	2.64E-03	3.41E-02
FBXO27	-3.6	3.45E-04	8.61E-03
TMEM145	-3.5	1.21E-03	2.06E-02
SLC2A5	-3.5	7.74E-07	5.90E-05
FAM238B	-3.5	4.57E-03	4.85E-02
LOC105378150	-3.5	3.92E-03	4.40E-02
LOC105374768	-3.5	1.04E-06	7.57E-05
TPX2	-3.5	1.56E-04	4.80E-03
CNRIP1	-3.5	2.64E-04	7.04E-03
TARM1	-3.5	2.12E-03	3.00E-02
LOC105376030	-3.5	1.50E-03	2.36E-02
RIPOR3	-3.5	2.76E-10	4.64E-08
PTK7	-3.5	1.68E-08	1.85E-06
LOC102723430	-3.5	5.64E-03	5.49E-02
CLIP2	-3.5	7.31E-10	1.12E-07
FAM107A	-3.5	3.44E-03	4.06E-02
CHIT1	-3.5	6.30E-05	2.36E-03
WNT9B	-3.5	3.17E-03	3.84E-02

LYPLAL1-DT	-3.5	8.59E-04	1.63E-02
HTRA3	-3.5	8.40E-05	2.98E-03
ITIH5	-3.5	1.24E-03	2.10E-02
H4C1	-3.5	3.94E-03	4.41E-02
EFHC2	-3.5	9.75E-08	9.27E-06
RPL23AP7	-3.5	4.40E-05	1.78E-03
CCDC168	-3.5	2.59E-04	6.95E-03
LOC102724808	-3.5	6.76E-06	3.76E-04
LINC00599	-3.5	3.95E-03	4.42E-02
KIF2A	-3.5	6.19E-10	9.72E-08
FERMT2	-3.5	4.47E-04	1.03E-02
RORB	-3.5	4.49E-03	4.80E-02
LOC105378798	-3.5	5.63E-03	5.49E-02
LOC105374510	-3.5	3.16E-03	3.84E-02
PROSER2-AS1	-3.5	3.19E-04	8.13E-03
LOC107986294	-3.5	6.48E-05	2.42E-03
ILDR2	-3.5	1.87E-05	8.82E-04
HSPA2	-3.5	6.95E-06	3.86E-04
CHST8	-3.5	5.10E-06	2.94E-04
HMGA2	-3.5	8.24E-04	1.58E-02
CST6	-3.5	3.90E-03	4.38E-02
MED12L	-3.5	4.28E-07	3.45E-05
ZNF185	-3.5	6.03E-13	1.62E-10
SMIM5	-3.5	4.30E-07	3.47E-05
MSANTD3	-3.5	1.92E-05	9.05E-04
DOC2A	-3.5	3.41E-04	8.52E-03
TRIM40	-3.5	3.37E-03	3.99E-02
DCLK2	-3.5	5.14E-04	1.14E-02
LGR4	-3.5	1.02E-04	3.47E-03
GXYLT2	-3.5	1.66E-03	2.52E-02
HP	-3.5	1.57E-05	7.66E-04
TNFSF4	-3.5	7.43E-12	1.62E-09
LINGO1	-3.5	8.16E-04	1.58E-02
FREM1	-3.5	2.21E-04	6.19E-03
H2BC13	-3.5	5.54E-04	1.19E-02
SLC9A3R2	-3.5	1.82E-04	5.33E-03
PKD1L2	-3.5	8.74E-04	1.64E-02
PRDM16	-3.5	1.92E-05	9.05E-04
ACKR2	-3.5	1.13E-03	1.96E-02
CRAT	-3.5	8.75E-09	1.04E-06
CD9	-3.5	9.85E-10	1.46E-07
GCOM1	-3.5	1.87E-03	2.74E-02
LOC107986820	-3.4	5.24E-03	5.26E-02
SIX1	-3.4	5.51E-03	5.41E-02
RN7SL3	-3.4	1.23E-04	3.97E-03
LOC101926892	-3.4	3.38E-03	4.00E-02
H1-0	-3.4	1.15E-16	5.61E-14

THNSL2	-3.4	2.94E-03	3.68E-02
NRK	-3.4	2.22E-03	3.08E-02
MCPH1-AS1	-3.4	4.42E-04	1.03E-02
AQP5	-3.4	4.68E-03	4.92E-02
KCNN2	-3.4	6.61E-04	1.35E-02
PRRT4	-3.4	1.43E-04	4.46E-03
ABCB5	-3.4	2.29E-03	3.14E-02
EGFR	-3.4	6.66E-04	1.36E-02
TRHDE	-3.4	1.18E-03	2.03E-02
SORBS2	-3.4	5.27E-04	1.15E-02
PAWR	-3.4	2.33E-07	1.99E-05
MOB1B	-3.4	2.78E-11	5.45E-09
CXCL3	-3.4	1.47E-04	4.58E-03
SRC	-3.4	2.25E-10	3.84E-08
LOC730101	-3.4	1.79E-03	2.67E-02
ARMC3	-3.4	7.70E-05	2.78E-03
MIR100HG	-3.4	2.65E-03	3.42E-02
EPHA2	-3.4	1.30E-04	4.13E-03
HS3ST6	-3.4	1.46E-03	2.32E-02
EMID1	-3.4	5.95E-04	1.26E-02
RNF11	-3.4	6.52E-12	1.44E-09
SIAE	-3.4	4.96E-09	6.26E-07
CCDC80	-3.4	3.53E-04	8.75E-03
CEBPE	-3.4	2.30E-04	6.37E-03
ARHGAP23	-3.4	1.91E-04	5.53E-03
LAMA3	-3.4	7.93E-04	1.54E-02
ARHGAP29	-3.4	9.02E-04	1.68E-02
TIMP3	-3.4	2.06E-04	5.89E-03
DEPP1	-3.4	5.04E-05	1.97E-03
LINC01750	-3.4	3.17E-03	3.84E-02
TUBB3	-3.4	3.33E-04	8.36E-03
LOC105372347	-3.4	4.43E-03	4.76E-02
LOC105372352	-3.4	3.99E-04	9.56E-03
BIRC5	-3.4	8.63E-04	1.63E-02
SMARCA1	-3.3	8.15E-04	1.58E-02
ZNF385C	-3.3	4.69E-03	4.92E-02
E2F7	-3.3	2.59E-03	3.37E-02
PFN4	-3.3	4.54E-03	4.84E-02
MIR9-3HG	-3.3	4.15E-03	4.56E-02
HMGB4	-3.3	3.23E-03	3.89E-02
STRIP2	-3.3	1.30E-05	6.56E-04
EFNB1	-3.3	6.34E-07	4.96E-05
TGFB2	-3.3	6.02E-04	1.27E-02
PTH2R	-3.3	5.34E-03	5.32E-02
LOC102724479	-3.3	3.34E-04	8.38E-03
RNASEH2A	-3.3	4.45E-04	1.03E-02
PADI3	-3.3	1.91E-03	2.78E-02

POU2F3	-3.3	1.83E-04	5.35E-03
CCNA2	-3.3	1.65E-03	2.51E-02
SLC35G1	-3.3	2.54E-04	6.85E-03
LOC101927666	-3.3	2.35E-03	3.18E-02
ATP9A	-3.3	8.62E-10	1.30E-07
LARP6	-3.3	3.96E-03	4.43E-02
CTXN1	-3.3	2.78E-03	3.54E-02
BCL2L1	-3.3	1.33E-09	1.91E-07
PLA2G4F	-3.3	2.17E-03	3.04E-02
BCAM	-3.3	5.93E-04	1.26E-02
STOM	-3.3	8.67E-10	1.31E-07
BUB1	-3.3	2.08E-04	5.93E-03
PTPRR	-3.3	1.45E-03	2.31E-02
IQGAP3	-3.3	1.25E-04	4.00E-03
DKFZp451B082	-3.3	2.60E-03	3.38E-02
FRMD6	-3.3	5.65E-04	1.21E-02
PRSS12	-3.3	2.01E-03	2.89E-02
ADCY5	-3.3	2.24E-04	6.25E-03
ARHGAP32	-3.3	7.14E-09	8.71E-07
DDX11L2	-3.3	1.88E-03	2.75E-02
TMEM17	-3.3	1.32E-03	2.18E-02
TEX101	-3.3	2.43E-03	3.24E-02
TSC22D1	-3.3	3.38E-10	5.52E-08
MIS18A	-3.2	5.65E-04	1.21E-02
LMNA	-3.2	7.08E-13	1.88E-10
DRC7	-3.2	2.24E-05	1.02E-03
OR2L13	-3.2	3.73E-03	4.27E-02
MELK	-3.2	2.78E-04	7.32E-03
CHRDL2	-3.2	4.39E-03	4.74E-02
H2AC12	-3.2	4.76E-03	4.97E-02
LOC102725121	-3.2	1.23E-03	2.09E-02
LOC101929216	-3.2	1.32E-03	2.18E-02
ERG	-3.2	7.38E-05	2.69E-03
TBX18	-3.2	8.58E-04	1.63E-02
GLYATL2	-3.2	3.79E-04	9.18E-03
SYBU	-3.2	1.56E-03	2.42E-02
C5orf30	-3.2	2.29E-07	1.96E-05
MICALL2	-3.2	8.50E-05	3.01E-03
EIF2AK1	-3.2	5.44E-18	3.63E-15
DMGDH	-3.2	2.28E-03	3.14E-02
UBL4A	-3.2	2.82E-09	3.74E-07
LOC101929595	-3.2	5.02E-03	5.12E-02
CIT	-3.2	1.10E-05	5.70E-04
PHF21B	-3.2	1.25E-03	2.10E-02
LOC105378979	-3.2	3.17E-03	3.84E-02
LOC102723324	-3.2	9.51E-04	1.75E-02
ATP5F1E	-3.2	6.91E-07	5.36E-05

HRC	-3.2	3.79E-03	4.31E-02
CT70	-3.2	5.64E-03	5.49E-02
RHBDL3	-3.2	2.11E-03	2.99E-02
FOXM1	-3.2	6.08E-04	1.27E-02
RSU1	-3.2	6.55E-12	1.44E-09
CCDC9B	-3.2	3.19E-09	4.19E-07
MAST4	-3.2	1.67E-11	3.49E-09
SH2D4B	-3.2	9.02E-04	1.68E-02
ECM1	-3.2	1.28E-04	4.08E-03
LOC107984690	-3.2	2.36E-03	3.18E-02
TPM4	-3.2	1.22E-14	4.28E-12
SMIM3	-3.2	1.39E-06	9.65E-05
LOX	-3.2	1.22E-04	3.94E-03
RDH11	-3.2	1.06E-08	1.24E-06
PDZD2	-3.2	6.64E-07	5.16E-05
LOC105371055	-3.2	4.96E-03	5.09E-02
AIF1L	-3.2	9.08E-04	1.69E-02
PODNL1	-3.2	2.32E-03	3.16E-02
EXO1	-3.2	1.74E-03	2.61E-02
RPE	-3.2	5.03E-04	1.12E-02
MIR12136	-3.2	1.28E-03	2.13E-02
IRX2	-3.2	4.55E-04	1.05E-02
ZBTB32	-3.2	1.05E-03	1.88E-02
DIPK2B	-3.2	2.52E-03	3.32E-02
LAMC1	-3.2	9.04E-09	1.07E-06
KEL	-3.2	4.30E-04	1.00E-02
SLC6A9	-3.2	1.17E-03	2.02E-02
UNC13C	-3.2	1.35E-03	2.21E-02
TDRD10	-3.2	4.98E-03	5.10E-02
BBOF1	-3.2	7.05E-04	1.42E-02
FBXO17	-3.2	1.70E-03	2.57E-02
LOC105371026	-3.2	7.82E-06	4.27E-04
GATA2	-3.2	2.38E-07	2.03E-05
MYO1C	-3.2	1.25E-10	2.21E-08
TSPAN18	-3.2	2.68E-07	2.27E-05
ODC1	-3.2	2.71E-09	3.61E-07
MYH15	-3.2	1.92E-03	2.79E-02
LINC02009	-3.2	7.16E-04	1.43E-02
LOC105372759	-3.1	4.94E-03	5.08E-02
WNT7B	-3.1	1.57E-03	2.44E-02
SEMA3D	-3.1	2.79E-03	3.55E-02
GAD1	-3.1	5.59E-03	5.45E-02
ST8SIA5	-3.1	6.39E-04	1.31E-02
CD226	-3.1	4.87E-08	4.89E-06
RNVU1-1	-3.1	3.29E-03	3.93E-02
CDC20	-3.1	2.54E-03	3.34E-02
LOC112268292	-3.1	7.15E-04	1.43E-02

LIPC	-3.1	2.53E-04	6.85E-03
LOC283856	-3.1	4.82E-03	5.01E-02
OLFML2B	-3.1	4.90E-04	1.10E-02
HUNK	-3.1	5.01E-03	5.11E-02
CLEC1A	-3.1	3.24E-03	3.90E-02
LOC105375242	-3.1	5.26E-03	5.27E-02
ASS1	-3.1	4.30E-03	4.66E-02
RAMP1	-3.1	2.59E-03	3.38E-02
HOMER2	-3.1	1.06E-06	7.70E-05
KCND2	-3.1	2.93E-03	3.68E-02
GINS3	-3.1	3.14E-03	3.82E-02
TMEM64	-3.1	6.52E-07	5.09E-05
TUBA1C	-3.1	9.51E-09	1.12E-06
TROAP	-3.1	2.96E-03	3.70E-02
LOC112267877	-3.1	6.37E-04	1.31E-02
LOC105376328	-3.1	3.54E-03	4.13E-02
GREM1	-3.1	4.21E-03	4.59E-02
LOC105375282	-3.1	3.07E-03	3.77E-02
LOC105375840	-3.1	1.90E-03	2.77E-02
CFAP61	-3.1	2.82E-03	3.57E-02
LOC107985747	-3.1	5.02E-03	5.12E-02
LAMB2	-3.1	1.18E-06	8.49E-05
H2AC4	-3.1	3.73E-03	4.27E-02
AHRR	-3.1	6.03E-04	1.27E-02
DLL3	-3.1	2.89E-03	3.64E-02
FSTL4	-3.1	1.18E-04	3.84E-03
CACNA1B	-3.1	2.67E-03	3.44E-02
LRRC72	-3.1	5.31E-03	5.30E-02
DCBLD2	-3.1	2.15E-04	6.10E-03
SERPINE2	-3.1	1.15E-04	3.77E-03
PIN4P1	-3.1	4.73E-03	4.94E-02
B4GALNT1	-3.1	3.78E-03	4.31E-02
ACSBG1	-3.1	3.01E-04	7.79E-03
LOC112268068	-3.1	4.31E-03	4.67E-02
LHX1	-3.1	3.52E-03	4.12E-02
SERPINB10	-3.1	6.16E-04	1.29E-02
HEYL	-3.1	5.57E-03	5.44E-02
DAB2IP	-3.1	5.30E-04	1.16E-02
ANKRD29	-3.1	3.32E-03	3.96E-02
RGS10	-3.1	8.20E-07	6.20E-05
AMOTL1	-3.1	1.66E-06	1.13E-04
HES7	-3.1	2.29E-03	3.14E-02
KIF3C	-3.1	4.60E-10	7.42E-08
MAPK10	-3.1	5.13E-04	1.13E-02
FAH	-3.1	1.84E-05	8.72E-04
OR2M7	-3.1	3.81E-03	4.32E-02
KALRN	-3.1	9.54E-07	7.04E-05

MUC2	-3.1	2.06E-04	5.89E-03
CDH3	-3.1	1.47E-03	2.33E-02
CA13	-3.0	2.48E-03	3.29E-02
EFS	-3.0	3.20E-03	3.86E-02
LOC112694756	-3.0	3.28E-04	8.28E-03
DYNC1I1	-3.0	6.20E-04	1.29E-02
LOC105372439	-3.0	1.34E-03	2.20E-02
TENT5C	-3.0	5.64E-13	1.53E-10
ANKRD9	-3.0	4.98E-09	6.26E-07
CLVS2	-3.0	4.52E-03	4.83E-02
PTPRG	-3.0	2.30E-03	3.15E-02
EFR3B	-3.0	3.02E-03	3.73E-02
VWC2	-3.0	2.03E-03	2.91E-02
B3GALNT1	-3.0	1.52E-03	2.39E-02
LOC105369965	-3.0	3.71E-03	4.26E-02
TMEM108	-3.0	1.08E-03	1.92E-02
LINC01168	-3.0	4.52E-03	4.83E-02
STXBP1	-3.0	1.51E-04	4.66E-03
LOC107987222	-3.0	2.56E-03	3.35E-02
SLC44A4	-3.0	2.71E-03	3.48E-02
GNA15	-3.0	5.32E-06	3.05E-04
LOC105372861	-3.0	4.95E-04	1.11E-02
BMNCR	-3.0	5.45E-03	5.38E-02
TJP2	-3.0	1.26E-11	2.66E-09
KDM4A-AS1	-3.0	5.65E-03	5.49E-02
ADGRV1	-3.0	5.43E-04	1.18E-02
COL11A1	-3.0	2.18E-03	3.05E-02
BCL2L15	-3.0	2.52E-03	3.32E-02
FZD7	-3.0	4.67E-03	4.91E-02
TNC	-3.0	8.15E-04	1.58E-02
MGAT5B	-3.0	2.32E-04	6.42E-03
TGM2	-3.0	1.09E-03	1.92E-02
SCUBE2	-3.0	4.45E-03	4.78E-02
AFAP1	-3.0	3.14E-07	2.61E-05
SHISA7	-3.0	3.89E-03	4.38E-02
LOC100287036	-3.0	1.89E-04	5.48E-03
TMPRSS2	-3.0	9.82E-04	1.79E-02
NEIL2	-3.0	5.12E-03	5.18E-02
MMP2	-3.0	4.42E-03	4.76E-02
NCK2	-3.0	5.08E-12	1.14E-09
PLAT	-3.0	5.56E-03	5.44E-02
P3H2	-3.0	1.03E-03	1.85E-02
CDCA2	-3.0	2.98E-03	3.70E-02
CTIF	-3.0	3.78E-07	3.10E-05
NEURL1B	-3.0	2.50E-04	6.79E-03
PHACTR3	-3.0	2.31E-03	3.15E-02
SYT7	-3.0	1.36E-03	2.22E-02

MSC-AS1	-3.0	2.58E-03	3.37E-02
LOC105374811	-3.0	2.54E-03	3.34E-02
STEAP1B	-3.0	5.21E-03	5.24E-02
EFNB2	-3.0	8.26E-05	2.94E-03
RUNDC3A	-3.0	4.89E-03	5.06E-02
PHLDB1	-3.0	6.23E-04	1.29E-02
ZNF273	-3.0	1.91E-03	2.78E-02
TGFB1I1	-3.0	4.81E-04	1.08E-02
NIPSNAP3B	-3.0	4.25E-03	4.63E-02
MRC2	-3.0	1.39E-03	2.24E-02
NES	-3.0	3.31E-03	3.95E-02
NFATC4	-3.0	1.18E-03	2.03E-02
AGPAT1	-3.0	1.02E-09	1.50E-07
DAAM1	-3.0	1.23E-07	1.13E-05
LOC105379003	-3.0	4.59E-03	4.86E-02
LOC105375026	-3.0	5.37E-03	5.34E-02
PRUNE1	-3.0	5.34E-08	5.31E-06
LOC101927484	-3.0	4.65E-03	4.89E-02
COL25A1	-3.0	7.96E-04	1.55E-02
AIG1	-3.0	4.72E-05	1.86E-03
SOX5	-3.0	7.27E-04	1.45E-02
CLCN3	-3.0	5.88E-07	4.65E-05
MYO1B	-3.0	2.91E-03	3.66E-02
JSRP1	-2.9	1.82E-03	2.70E-02
KITLG	-2.9	1.54E-03	2.40E-02
TEX15	-2.9	4.70E-03	4.92E-02
SPATA25	-2.9	3.50E-03	4.11E-02
ADGRB3	-2.9	4.92E-03	5.07E-02
ZNRF2P1	-2.9	5.36E-03	5.33E-02
LEPR	-2.9	5.04E-05	1.97E-03
KCNA1	-2.9	1.93E-03	2.80E-02
MISP	-2.9	2.92E-03	3.66E-02
LOC105372321	-2.9	2.60E-03	3.38E-02
ANGPT1	-2.9	2.42E-04	6.61E-03
FOXC2	-2.9	4.20E-03	4.59E-02
WASF1	-2.9	3.46E-04	8.62E-03
CDC45	-2.9	1.87E-03	2.74E-02
IL17RE	-2.9	4.56E-03	4.85E-02
IL15RA	-2.9	2.31E-03	3.15E-02
ADAMTS19	-2.9	2.10E-03	2.98E-02
CCDC92	-2.9	3.49E-05	1.47E-03
TLN1	-2.9	3.22E-17	1.76E-14
TRPC6	-2.9	3.17E-04	8.09E-03
MPP1	-2.9	3.57E-14	1.17E-11
TRDN	-2.9	5.55E-03	5.44E-02
CLEC3B	-2.9	5.64E-03	5.49E-02
MCRIP1	-2.9	2.94E-03	3.68E-02

LOC105378726	-2.9	1.19E-03	2.04E-02
C21orf58	-2.9	1.55E-06	1.05E-04
KRT84	-2.9	3.82E-03	4.33E-02
KBTBD12	-2.9	1.47E-03	2.33E-02
SPNS2	-2.9	1.33E-03	2.19E-02
LRRC8B	-2.9	3.28E-07	2.72E-05
KIF11	-2.9	1.74E-03	2.61E-02
CNNM1	-2.9	5.12E-03	5.17E-02
NNMT	-2.9	2.29E-03	3.14E-02
TUBA4A	-2.9	9.93E-11	1.80E-08
RTCA-AS1	-2.9	3.09E-03	3.78E-02
SNN	-2.9	3.55E-09	4.62E-07
DPF3	-2.9	1.51E-04	4.66E-03
LOC100129098	-2.9	9.04E-05	3.15E-03
PTPRU	-2.9	4.16E-04	9.79E-03
PRKAA2	-2.9	5.55E-03	5.44E-02
FLG	-2.9	5.06E-03	5.14E-02
SDK1	-2.9	8.97E-04	1.67E-02
CTNND2	-2.9	3.01E-04	7.79E-03
ICAM5	-2.9	5.12E-05	2.00E-03
MAGI2	-2.9	2.11E-04	6.00E-03
WHAMMP3	-2.9	3.23E-05	1.38E-03
COL4A1	-2.9	4.77E-04	1.08E-02
COL13A1	-2.9	3.29E-03	3.93E-02
ATP8B5P	-2.8	2.46E-03	3.27E-02
PRELID2	-2.8	1.06E-03	1.89E-02
LOC105374031	-2.8	4.09E-04	9.72E-03
TJP1	-2.8	1.32E-05	6.63E-04
P4HA2	-2.8	2.72E-03	3.49E-02
RSPH9	-2.8	5.01E-03	5.11E-02
USP6	-2.8	1.83E-03	2.70E-02
TRO	-2.8	1.50E-03	2.36E-02
LOC107984830	-2.8	2.44E-03	3.25E-02
COL16A1	-2.8	2.95E-03	3.69E-02
TRIM55	-2.8	3.43E-03	4.04E-02
PTPRZ1	-2.8	5.57E-03	5.44E-02
SLA2	-2.8	3.75E-06	2.28E-04
DUOX2	-2.8	1.47E-03	2.33E-02
ISCA2	-2.8	5.23E-03	5.25E-02
LOC105372249	-2.8	4.95E-03	5.09E-02
F2R	-2.8	5.74E-06	3.25E-04
APP	-2.8	5.19E-16	2.26E-13
KIF4A	-2.8	2.28E-03	3.14E-02
KIFC1	-2.8	1.60E-03	2.47E-02
ADAMTS15	-2.8	1.57E-03	2.43E-02
CDC25C	-2.8	2.20E-03	3.06E-02
MKI67	-2.8	5.81E-05	2.22E-03

THRB	-2.8	1.57E-04	4.82E-03
SLC39A3	-2.8	3.57E-06	2.18E-04
KCTD20	-2.8	6.49E-16	2.76E-13
ATP13A4	-2.8	1.33E-04	4.20E-03
DGKI	-2.8	3.28E-03	3.93E-02
SORBS1	-2.8	2.19E-04	6.16E-03
CDCA5	-2.8	2.55E-03	3.35E-02
GPX1	-2.8	1.69E-11	3.52E-09
ATP8	-2.8	7.76E-04	1.52E-02
LOC105371362	-2.8	2.14E-03	3.01E-02
GRK5	-2.8	2.85E-08	3.01E-06
INSM1	-2.8	1.53E-03	2.39E-02
MCUR1	-2.8	1.63E-05	7.91E-04
SOX12	-2.8	1.35E-06	9.49E-05
MYO18B	-2.8	7.66E-04	1.51E-02
HSPB1	-2.8	4.11E-07	3.34E-05
C3orf80	-2.8	2.74E-03	3.50E-02
ASXL3	-2.7	1.93E-04	5.59E-03
SGCZ	-2.7	3.81E-03	4.32E-02
SNORA104	-2.7	1.96E-04	5.66E-03
SIX4	-2.7	1.98E-03	2.85E-02
MCM6	-2.7	2.15E-05	9.92E-04
BICC1	-2.7	2.59E-03	3.37E-02
LOC101928462	-2.7	2.26E-03	3.11E-02
GABRE	-2.7	1.97E-03	2.85E-02
CLDN4	-2.7	4.56E-03	4.85E-02
STBD1	-2.7	4.21E-03	4.59E-02
SEPTIN11	-2.7	2.59E-08	2.76E-06
NKAIN3	-2.7	4.47E-03	4.79E-02
LOC112268224	-2.7	4.70E-03	4.93E-02
PLK1	-2.7	2.16E-03	3.03E-02
LOXL2	-2.7	1.95E-03	2.83E-02
MAD2L1BP	-2.7	1.93E-06	1.28E-04
JAKMIP3	-2.7	1.21E-03	2.06E-02
TXNDC16	-2.7	1.78E-03	2.66E-02
CCDC24	-2.7	3.54E-03	4.13E-02
VKORC1L1	-2.7	9.91E-06	5.22E-04
ANKRD28	-2.7	6.76E-08	6.62E-06
H2BC17	-2.7	1.88E-03	2.76E-02
DNAJC18	-2.7	3.90E-03	4.38E-02
ARG1	-2.7	4.50E-04	1.04E-02
RAP1B	-2.7	9.45E-07	6.99E-05
CCDC8	-2.7	2.19E-03	3.05E-02
FBLN1	-2.7	4.98E-03	5.10E-02
HDC	-2.7	2.91E-04	7.59E-03
PRTG	-2.7	3.56E-03	4.15E-02
ADAMTS16	-2.7	4.41E-03	4.75E-02

MOB3C	-2.7	8.16E-06	4.42E-04
MS4A2	-2.7	3.82E-03	4.33E-02
WNT5B	-2.7	6.54E-04	1.34E-02
NEURL3	-2.7	4.10E-03	4.52E-02
CENPF	-2.7	2.27E-04	6.31E-03
COL17A1	-2.7	1.14E-03	1.98E-02
LOC105372698	-2.7	5.28E-03	5.28E-02
CCNB1	-2.7	4.54E-04	1.05E-02
SLC4A11	-2.7	4.27E-04	1.00E-02
CLCN4	-2.7	1.79E-06	1.19E-04
NPAS2	-2.7	2.63E-03	3.41E-02
TLK1	-2.7	1.21E-08	1.39E-06
BANK1	-2.7	6.51E-05	2.43E-03
CD151	-2.7	4.81E-09	6.12E-07
DGKG	-2.7	1.38E-05	6.83E-04
PLA2G4A	-2.7	6.95E-05	2.56E-03
H2AC11	-2.7	3.58E-04	8.84E-03
ELOVL6	-2.7	6.01E-04	1.27E-02
CORO1C	-2.7	6.37E-10	9.97E-08
KANK2	-2.6	4.00E-04	9.59E-03
ARFGEF3	-2.6	2.32E-03	3.16E-02
LOC107984836	-2.6	5.09E-03	5.16E-02
ANO2	-2.6	2.81E-03	3.56E-02
H2AC6	-2.6	4.89E-07	3.91E-05
H2BC15	-2.6	1.36E-03	2.22E-02
CEL	-2.6	6.25E-04	1.30E-02
RAB37	-2.6	3.04E-06	1.91E-04
MAP2	-2.6	8.28E-04	1.59E-02
GNAT1	-2.6	5.65E-03	5.50E-02
NT5C3A	-2.6	3.10E-05	1.34E-03
NFASC	-2.6	1.18E-03	2.03E-02
RIMS3	-2.6	1.16E-03	2.01E-02
PDGFRB	-2.6	2.45E-03	3.26E-02
STARD8	-2.6	1.11E-06	8.02E-05
MTSS2	-2.6	1.04E-05	5.43E-04
SEPTIN4	-2.6	6.31E-04	1.30E-02
VIT	-2.6	4.39E-03	4.74E-02
GPD2	-2.6	7.22E-07	5.55E-05
ARSB	-2.6	1.09E-03	1.92E-02
CDH4	-2.6	4.61E-03	4.87E-02
CYB5R1	-2.6	7.95E-08	7.72E-06
OBSL1	-2.6	8.57E-04	1.63E-02
CMBL	-2.6	1.40E-03	2.25E-02
SH3BP4	-2.6	2.78E-03	3.55E-02
PDGFC	-2.6	2.99E-05	1.31E-03
CAPN11	-2.6	4.56E-03	4.85E-02
CDK2AP1	-2.6	6.60E-06	3.69E-04

DUSP4	-2.6	5.05E-03	5.14E-02
MCM10	-2.6	2.81E-03	3.56E-02
PALM2AKAP2	-2.6	5.86E-09	7.25E-07
LOC102724687	-2.6	4.19E-03	4.58E-02
LOC107986454	-2.6	5.31E-03	5.30E-02
PRG2	-2.6	2.42E-03	3.24E-02
IPP	-2.6	3.14E-03	3.82E-02
ADRB3	-2.6	5.02E-03	5.12E-02
DSG2	-2.6	2.79E-03	3.55E-02
ACTR3B	-2.6	4.00E-06	2.38E-04
STMP1	-2.6	3.18E-05	1.36E-03
NORAD	-2.6	2.00E-08	2.18E-06
B3GNT6	-2.6	5.51E-03	5.41E-02
BTBD11	-2.6	7.25E-05	2.65E-03
H2BC9	-2.6	7.92E-04	1.54E-02
XPNPEP1	-2.6	6.58E-06	3.69E-04
KCNQ2	-2.6	2.37E-03	3.19E-02
THSD7A	-2.6	2.45E-03	3.26E-02
BUB1B	-2.6	4.00E-03	4.45E-02
MEG3	-2.6	4.13E-04	9.75E-03
TXNL4B	-2.6	3.69E-06	2.25E-04
SLC37A1	-2.6	2.48E-06	1.60E-04
NLGN1	-2.6	3.15E-03	3.83E-02
NRXN2	-2.6	7.07E-04	1.42E-02
NIPA1	-2.6	2.06E-05	9.60E-04
FAM110A	-2.6	5.93E-06	3.35E-04
PTPRD	-2.6	4.62E-03	4.88E-02
LOC107984751	-2.6	3.42E-03	4.03E-02
NUSAP1	-2.6	5.03E-04	1.12E-02
LVRN	-2.6	3.50E-03	4.11E-02
RN7SK	-2.6	1.08E-04	3.60E-03
CLIC4	-2.6	6.98E-06	3.87E-04
DOC2B	-2.5	4.90E-04	1.10E-02
RELN	-2.5	4.61E-03	4.87E-02
PRR29	-2.5	3.72E-04	9.06E-03
LOC101929295	-2.5	1.01E-03	1.83E-02
MNX1	-2.5	1.14E-03	1.98E-02
KIAA1324L	-2.5	3.98E-03	4.43E-02
ARNT2	-2.5	1.67E-03	2.53E-02
RIOK3	-2.5	6.02E-09	7.39E-07
PIP5K1B	-2.5	2.25E-04	6.26E-03
WWC1	-2.5	5.32E-04	1.16E-02
L1CAM	-2.5	2.84E-03	3.59E-02
GTPBP2	-2.5	1.42E-09	2.03E-07
ZNF385B	-2.5	2.44E-03	3.25E-02
CTSA	-2.5	2.04E-12	5.16E-10
SCD	-2.5	7.05E-06	3.91E-04

BIVM	-2.5	1.79E-03	2.66E-02
DPY19L1	-2.5	5.29E-05	2.05E-03
OPRD1	-2.5	4.43E-03	4.76E-02
H4C2	-2.5	4.62E-03	4.88E-02
USP31	-2.5	3.94E-06	2.35E-04
DIMT1	-2.5	3.86E-06	2.31E-04
KLHDC7A	-2.5	4.74E-03	4.95E-02
ACTG1P20	-2.5	4.00E-03	4.45E-02
LCA5	-2.5	2.10E-03	2.98E-02
PCDH17	-2.5	2.28E-03	3.13E-02
SERPINF1	-2.5	4.62E-03	4.88E-02
SVIP	-2.5	3.30E-05	1.41E-03
DNAH5	-2.5	5.66E-03	5.50E-02
CLSPN	-2.5	9.43E-04	1.74E-02
SLC40A1	-2.5	1.28E-07	1.17E-05
LOC100288203	-2.5	6.01E-05	2.27E-03
LOC100287290	-2.5	3.93E-03	4.40E-02
CABLES1	-2.5	4.66E-03	4.90E-02
LRIG3	-2.5	3.19E-03	3.86E-02
NXN	-2.5	8.31E-04	1.59E-02
KIFAP3	-2.5	3.45E-05	1.46E-03
SPACA4	-2.5	3.20E-03	3.87E-02
DOCK6	-2.5	8.44E-04	1.61E-02
MGAT4B	-2.5	1.77E-07	1.57E-05
WNT5A	-2.5	4.96E-03	5.09E-02
DSE	-2.5	9.53E-06	5.06E-04
BCL2L2	-2.5	5.68E-06	3.23E-04
LAMA2	-2.5	4.17E-03	4.57E-02
LINC02531	-2.4	1.45E-03	2.31E-02
CELF4	-2.4	2.62E-03	3.40E-02
SHANK2	-2.4	2.15E-03	3.02E-02
PTPRS	-2.4	2.35E-05	1.07E-03
ACSM1	-2.4	3.01E-03	3.73E-02
BBC3	-2.4	4.77E-05	1.88E-03
ACTA1	-2.4	4.62E-03	4.88E-02
COMMD7	-2.4	2.38E-04	6.53E-03
TRIP10	-2.4	6.46E-04	1.33E-02
ASAP3	-2.4	2.97E-03	3.70E-02
ADIPOR2	-2.4	9.21E-08	8.83E-06
IGFN1	-2.4	3.22E-03	3.88E-02
GLRX5	-2.4	5.28E-03	5.28E-02
AOC4P	-2.4	3.11E-03	3.81E-02
ATP1B2	-2.4	2.21E-03	3.07E-02
CHSY3	-2.4	5.20E-03	5.24E-02
TSPAN15	-2.4	6.26E-04	1.30E-02
PHGDH	-2.4	5.13E-03	5.18E-02
GCSAML	-2.4	6.01E-04	1.27E-02

DIPK1B	-2.4	9.46E-04	1.74E-02
PCDH7	-2.4	2.81E-03	3.56E-02
CALB2	-2.4	4.43E-03	4.76E-02
CDIP1	-2.4	6.34E-07	4.96E-05
NREP	-2.4	1.80E-03	2.67E-02
FRMPD4	-2.4	3.50E-03	4.10E-02
FERMT3	-2.4	5.23E-11	9.87E-09
OSMR	-2.4	5.11E-03	5.17E-02
FRAS1	-2.4	3.52E-03	4.12E-02
DNAH10	-2.4	1.75E-04	5.22E-03
PTCD2	-2.4	2.96E-03	3.69E-02
LOC399975	-2.4	2.63E-03	3.41E-02
UACA	-2.4	1.06E-03	1.89E-02
GNAO1	-2.4	7.29E-05	2.66E-03
FSCN1	-2.4	4.32E-03	4.68E-02
MEIS2	-2.4	2.02E-03	2.90E-02
ITGA9	-2.4	3.62E-04	8.88E-03
H2AJ	-2.4	1.63E-03	2.50E-02
ZNF175	-2.4	1.31E-04	4.15E-03
DZIP1L	-2.4	1.90E-03	2.77E-02
FARP1	-2.4	4.17E-03	4.57E-02
PYGB	-2.4	3.42E-08	3.57E-06
SLC22A23	-2.4	8.64E-05	3.05E-03
SLX4	-2.3	2.37E-07	2.02E-05
CCSER1	-2.3	2.43E-03	3.24E-02
FUT8	-2.3	2.45E-06	1.58E-04
F8	-2.3	1.14E-03	1.98E-02
PLA2G4C	-2.3	1.17E-03	2.01E-02
MIR4435-2HG	-2.3	3.69E-05	1.53E-03
CDH20	-2.3	4.06E-03	4.49E-02
MCU	-2.3	1.19E-07	1.10E-05
MYBL2	-2.3	2.19E-04	6.16E-03
PTMS	-2.3	3.36E-04	8.42E-03
RAB6D	-2.3	5.29E-03	5.28E-02
CCL5	-2.3	2.76E-07	2.33E-05
LHFPL2	-2.3	2.62E-06	1.68E-04
DPYSL3	-2.3	2.28E-03	3.14E-02
LINC01814	-2.3	4.13E-03	4.54E-02
ATP2B2	-2.3	2.62E-03	3.40E-02
CCDC40	-2.3	3.68E-03	4.23E-02
RASAL2	-2.3	2.12E-03	3.00E-02
MAX	-2.3	1.01E-05	5.29E-04
EPDR1	-2.3	4.63E-03	4.88E-02
CEMIP	-2.3	2.16E-04	6.10E-03
NLK	-2.3	3.54E-05	1.48E-03
SHE	-2.3	3.24E-03	3.90E-02
BRIP1	-2.3	3.58E-03	4.16E-02

NET1	-2.3	4.22E-05	1.71E-03
MINDY1	-2.3	7.34E-08	7.16E-06
ABCA3	-2.3	4.14E-04	9.77E-03
H2AC15	-2.3	4.22E-03	4.60E-02
LDLRAP1	-2.3	4.73E-06	2.76E-04
ACVR1	-2.3	9.81E-05	3.36E-03
PTPN14	-2.3	2.76E-03	3.52E-02
TUBB6	-2.3	2.03E-03	2.91E-02
STARD13	-2.3	3.19E-03	3.86E-02
KIAA1217	-2.3	2.84E-03	3.59E-02
UBE2O	-2.3	1.72E-07	1.54E-05
GPR55	-2.3	2.31E-03	3.15E-02
SCN8A	-2.3	4.10E-03	4.51E-02
KDM7A-DT	-2.3	1.50E-03	2.36E-02
ST3GAL3	-2.3	6.66E-05	2.47E-03
PLEKHG4B	-2.3	5.08E-03	5.15E-02
SHB	-2.3	4.89E-03	5.05E-02
NFIB	-2.3	1.34E-03	2.19E-02
ARHGEF10	-2.3	5.19E-03	5.23E-02
KLF5	-2.3	1.58E-03	2.45E-02
RET	-2.3	1.30E-03	2.16E-02
SATB2	-2.3	4.30E-03	4.66E-02
ROBO1	-2.3	4.01E-03	4.45E-02
PTGER3	-2.3	4.36E-03	4.72E-02
STX1A	-2.2	2.69E-03	3.46E-02
PKIG	-2.2	1.06E-03	1.89E-02
CYB5R3	-2.2	1.45E-06	1.00E-04
TMEM185A	-2.2	3.07E-05	1.33E-03
PHLDA1	-2.2	3.32E-03	3.96E-02
NRP1	-2.2	5.59E-03	5.46E-02
SLC45A3	-2.2	1.74E-03	2.61E-02
PCDH9	-2.2	3.60E-04	8.85E-03
ARMH1	-2.2	3.12E-03	3.81E-02
LGMN	-2.2	1.34E-03	2.19E-02
PLCB4	-2.2	1.97E-03	2.85E-02
FBXO40	-2.2	1.40E-03	2.25E-02
PGLYRP1	-2.2	3.85E-03	4.36E-02
AMIGO2	-2.2	7.67E-04	1.51E-02
PTPN18	-2.2	2.61E-09	3.50E-07
FLNA	-2.2	1.79E-12	4.59E-10
SPTBN2	-2.2	9.10E-04	1.69E-02
PRDX6	-2.2	1.07E-03	1.90E-02
LINC01011	-2.2	2.79E-03	3.55E-02
CNKSR3	-2.2	1.79E-03	2.67E-02
NAP1L1	-2.2	1.07E-08	1.25E-06
TMEM132E	-2.2	4.34E-03	4.70E-02
EEF1A2	-2.2	4.85E-03	5.03E-02

ETFA	-2.2	1.03E-06	7.52E-05
LPAR5	-2.2	5.04E-04	1.12E-02
DSEL	-2.2	5.56E-03	5.44E-02
MYB	-2.2	1.63E-03	2.50E-02
PPM1L	-2.2	9.50E-06	5.06E-04
CLTCL1	-2.2	1.12E-03	1.96E-02
CMIP	-2.2	2.80E-11	5.45E-09
PTPRJ	-2.2	3.50E-07	2.88E-05
SWI5	-2.2	1.54E-03	2.40E-02
WHAMMP2	-2.2	8.58E-04	1.63E-02
USP12	-2.2	5.93E-04	1.26E-02
PITX1	-2.2	4.98E-03	5.10E-02
LRBA	-2.2	1.07E-07	1.01E-05
TMEM60	-2.2	2.18E-03	3.05E-02
HERC2P3	-2.2	4.11E-03	4.52E-02
HMCN2	-2.2	3.38E-03	4.00E-02
NID1	-2.2	5.90E-05	2.24E-03
KCTD10	-2.1	2.93E-06	1.84E-04
SNX9	-2.1	1.94E-06	1.28E-04
SLC7A5	-2.1	8.76E-05	3.07E-03
LAMA5	-2.1	2.54E-03	3.33E-02
AKAP12	-2.1	6.57E-04	1.34E-02
ZNRF3	-2.1	5.58E-03	5.45E-02
COL24A1	-2.1	1.63E-03	2.50E-02
CACNB3	-2.1	5.12E-03	5.17E-02
RAB30	-2.1	8.65E-05	3.05E-03
H2BC12	-2.1	3.89E-04	9.37E-03
ESPL1	-2.1	2.53E-03	3.33E-02
MEST	-2.1	2.36E-03	3.18E-02
RAP2B	-2.1	1.52E-08	1.69E-06
MYO10	-2.1	2.15E-03	3.02E-02
KNL1	-2.1	3.47E-03	4.08E-02
KCNK6	-2.1	1.51E-07	1.37E-05
PSMB7	-2.1	2.56E-03	3.35E-02
LOC105371709	-2.1	4.69E-03	4.92E-02
ST7	-2.1	7.15E-04	1.43E-02
NDST1	-2.1	1.80E-07	1.59E-05
JCAD	-2.1	3.56E-03	4.15E-02
APCDD1	-2.1	1.80E-03	2.68E-02
RAB4A	-2.1	3.92E-04	9.45E-03
VWA5A	-2.1	5.18E-04	1.14E-02
KIAA1586	-2.1	5.95E-04	1.26E-02
TPST2	-2.1	3.42E-07	2.83E-05
CALM3	-2.1	1.21E-06	8.63E-05
FOSL1	-2.1	1.77E-03	2.64E-02
WRNIP1	-2.1	1.17E-05	6.00E-04
TGFB1	-2.1	1.09E-07	1.02E-05

CAPN1	-2.1	1.17E-07	1.08E-05
GADD45A	-2.1	1.78E-04	5.27E-03
TMEM70	-2.1	2.99E-03	3.71E-02
FOPNL	-2.1	9.23E-05	3.20E-03
CCDC85B	-2.1	1.62E-04	4.93E-03
LINC01137	-2.1	3.11E-03	3.81E-02
IRAK2	-2.1	4.04E-04	9.64E-03
MGAT3	-2.1	3.45E-03	4.06E-02
NAV2	-2.1	2.24E-03	3.10E-02
SPRY2	-2.1	5.36E-03	5.33E-02
MAFG	-2.0	4.62E-06	2.71E-04
ST8SIA6	-2.0	1.62E-03	2.49E-02
ILK	-2.0	4.28E-04	1.00E-02
TTC28	-2.0	2.38E-04	6.54E-03
H2AC17	-2.0	1.55E-03	2.42E-02
DBN1	-2.0	1.32E-06	9.27E-05
ABHD4	-2.0	6.91E-04	1.39E-02
HMGA1	-2.0	3.59E-03	4.17E-02
CCR4	-2.0	1.46E-03	2.32E-02
XPO7	-2.0	5.37E-09	6.69E-07
ATP8B4	-2.0	5.58E-05	2.14E-03
BTBD3	-2.0	4.77E-03	4.98E-02
ITGB4	-2.0	2.14E-03	3.02E-02
VKORC1	-2.0	4.47E-03	4.79E-02
PYCR2	-2.0	8.05E-05	2.88E-03
BLM	-2.0	4.52E-03	4.82E-02
ZNF792	-2.0	8.86E-04	1.66E-02
DAPP1	-2.0	2.10E-05	9.74E-04
ND6	-2.0	1.15E-03	1.99E-02
KLHL6	-2.0	9.35E-06	5.00E-04
MEA1	-2.0	3.49E-05	1.47E-03
FRMD4B	-2.0	4.30E-04	1.00E-02
PPM1A	-2.0	1.95E-07	1.70E-05
GNB5	-2.0	2.73E-04	7.21E-03
ZNF778	-2.0	5.76E-04	1.23E-02
LOC105376220	-2.0	3.56E-03	4.15E-02
PDE4D	-2.0	1.07E-05	5.56E-04
TTC33	-2.0	1.47E-03	2.33E-02
CDC42BPB	-2.0	1.29E-04	4.11E-03
DST	-2.0	2.92E-03	3.66E-02
ACTN1	-2.0	2.41E-11	4.84E-09
PHKB	-1.9	1.43E-06	9.91E-05
PNKD	-1.9	3.49E-04	8.66E-03
ATP2C1	-1.9	7.29E-04	1.45E-02
RAB11A	-1.9	4.12E-05	1.68E-03
ZC3H12C	-1.9	8.15E-04	1.58E-02
ADCY3	-1.9	8.06E-05	2.88E-03

CHI3L1	-1.9	3.04E-03	3.75E-02
PNP	-1.9	2.49E-05	1.12E-03
HIPK2	-1.9	4.84E-10	7.78E-08
ITGB1	-1.9	3.04E-07	2.54E-05
ZNF271P	-1.9	4.19E-04	9.85E-03
TM2D2	-1.9	9.40E-04	1.74E-02
TMEM104	-1.9	4.12E-04	9.75E-03
CDCA7L	-1.9	1.53E-03	2.40E-02
ROCK2	-1.9	1.21E-04	3.92E-03
NAV1	-1.9	4.47E-03	4.78E-02
PKM	-1.9	4.13E-06	2.45E-04
HSPG2	-1.9	4.49E-03	4.80E-02
LRP6	-1.9	4.15E-03	4.56E-02
SPIRE1	-1.9	4.96E-03	5.09E-02
CDKN1A	-1.9	6.07E-05	2.29E-03
ABLIM1	-1.9	1.64E-05	7.94E-04
LZTS2	-1.9	2.81E-03	3.56E-02
ARL15	-1.9	3.01E-03	3.73E-02
MGAT5	-1.9	1.88E-06	1.25E-04
KLHL5	-1.9	1.29E-04	4.10E-03
ABCB6	-1.9	2.20E-03	3.07E-02
ATP2A3	-1.9	7.30E-10	1.12E-07
VDAC3	-1.9	1.37E-03	2.23E-02
PCP2	-1.8	4.32E-03	4.68E-02
EHBP1	-1.8	4.14E-04	9.77E-03
VANGL1	-1.8	1.75E-03	2.62E-02
GRB10	-1.8	2.53E-04	6.85E-03
HS2ST1	-1.8	3.97E-03	4.43E-02
TSPOAP1-AS1	-1.8	3.64E-03	4.21E-02
TLR5	-1.8	2.30E-03	3.15E-02
ATP7B	-1.8	4.64E-03	4.88E-02
PHTF2	-1.8	4.49E-04	1.04E-02
MAGED2	-1.8	1.29E-04	4.11E-03
LIPA	-1.8	5.52E-03	5.42E-02
NCKIPSD	-1.8	4.65E-04	1.06E-02
GSTO1	-1.8	2.55E-03	3.34E-02
MBTD1	-1.8	1.37E-03	2.23E-02
MDM1	-1.8	3.58E-03	4.16E-02
GCLM	-1.8	1.13E-03	1.97E-02
LOXL3	-1.8	4.04E-03	4.47E-02
GSN	-1.8	3.85E-06	2.31E-04
ZNF664	-1.8	3.13E-04	8.03E-03
MFSD1	-1.8	1.21E-04	3.93E-03
LAMTOR1	-1.8	4.62E-04	1.06E-02
WDR44	-1.8	2.20E-03	3.06E-02
UBASH3B	-1.8	5.37E-04	1.17E-02
SIAH2	-1.8	8.38E-04	1.60E-02

CTNS	-1.8	2.76E-03	3.52E-02
NUTF2	-1.8	8.46E-05	3.00E-03
PIK3CB	-1.8	5.34E-04	1.16E-02
SOCS2	-1.8	4.57E-03	4.85E-02
MADD	-1.8	1.12E-04	3.71E-03
CSF1	-1.8	1.38E-03	2.24E-02
PLEKHO1	-1.8	2.97E-04	7.73E-03
LEPROT	-1.8	4.12E-03	4.53E-02
AMFR	-1.8	1.17E-03	2.01E-02
RDX	-1.7	3.31E-03	3.95E-02
GNAS	-1.7	4.98E-09	6.26E-07
MAP4K5	-1.7	1.31E-03	2.17E-02
UBE2H	-1.7	1.71E-06	1.15E-04
PIP4K2A	-1.7	2.42E-05	1.10E-03
SPINT2	-1.7	1.49E-03	2.35E-02
SLC27A4	-1.7	1.66E-03	2.51E-02
H2BC5	-1.7	5.24E-03	5.26E-02
EPB41L3	-1.7	8.53E-04	1.62E-02
CERS2	-1.7	2.29E-05	1.04E-03
TBC1D13	-1.7	4.22E-04	9.91E-03
ABHD16A	-1.7	3.83E-06	2.31E-04
KIAA0513	-1.7	1.47E-05	7.22E-04
UHRF1BP1	-1.7	1.76E-04	5.24E-03
UGCG	-1.7	8.33E-04	1.59E-02
CCNG1	-1.7	1.53E-03	2.39E-02
CEACAM1	-1.7	5.27E-04	1.15E-02
LYL1	-1.7	1.40E-03	2.25E-02
KLF9	-1.7	1.08E-03	1.92E-02
SLC10A3	-1.7	2.34E-03	3.17E-02
VIM-AS1	-1.7	3.82E-03	4.33E-02
WDR1	-1.6	2.50E-08	2.68E-06
TMBIM1	-1.6	1.31E-05	6.60E-04
SH3PXD2A	-1.6	9.05E-04	1.68E-02
PTTG1IP	-1.6	7.36E-04	1.46E-02
UHRF1	-1.6	4.29E-03	4.66E-02
ANXA3	-1.6	1.86E-03	2.74E-02
SUSD1	-1.6	5.16E-04	1.14E-02
ADAM9	-1.6	3.72E-04	9.06E-03
NFE2	-1.6	4.06E-04	9.67E-03
BRD3	-1.6	1.61E-04	4.91E-03
EIF4G3	-1.6	3.45E-05	1.46E-03
TIMP1	-1.6	2.96E-03	3.69E-02
HDGF	-1.6	2.05E-05	9.57E-04
DAP	-1.6	2.76E-03	3.52E-02
ND5	-1.6	3.12E-03	3.81E-02
RYR3	-1.6	5.20E-03	5.24E-02
MYH9	-1.6	6.12E-11	1.15E-08

TUBB4B	-1.6	9.14E-05	3.18E-03
PKP4	-1.6	1.45E-03	2.31E-02
BTK	-1.6	2.23E-03	3.10E-02
XYLT2	-1.6	1.48E-03	2.34E-02
LOXHD1	-1.6	4.40E-03	4.75E-02
PTPN12	-1.6	2.44E-03	3.25E-02
YES1	-1.5	2.47E-03	3.28E-02
LAT	-1.5	2.10E-03	2.99E-02
CRKL	-1.5	1.80E-04	5.30E-03
VAPB	-1.5	1.34E-03	2.20E-02
MCTP1	-1.5	8.38E-04	1.60E-02
MAPRE2	-1.5	4.26E-04	1.00E-02
ELK3	-1.5	2.24E-03	3.10E-02
P2RX1	-1.5	4.99E-04	1.11E-02
TNIK	-1.5	5.64E-04	1.21E-02
DIAPH1	-1.5	9.96E-05	3.40E-03
ATE1	-1.5	4.36E-04	1.02E-02
MTMR12	-1.5	4.99E-03	5.10E-02
GNAQ	-1.5	5.92E-04	1.26E-02
BICD2	-1.5	4.98E-04	1.11E-02
SLC44A1	-1.5	3.25E-03	3.91E-02
RHBDD1	-1.5	2.98E-03	3.70E-02
CCND3	-1.5	8.60E-04	1.63E-02
LYPLAL1	-1.5	5.11E-03	5.17E-02
DGKD	-1.4	2.81E-04	7.37E-03
PCGF5	-1.4	4.76E-03	4.97E-02
UXS1	-1.4	1.80E-03	2.68E-02
PSTPIP2	-1.4	3.71E-03	4.26E-02
CUX1	-1.4	9.92E-07	7.29E-05
ZCCHC17	-1.4	4.56E-03	4.85E-02
VDR	-1.4	2.75E-03	3.52E-02
ND4	-1.4	1.52E-03	2.39E-02
FADS2	-1.4	5.00E-03	5.11E-02
KANSL3	-1.4	1.04E-04	3.50E-03
FER	-1.4	2.76E-03	3.52E-02
YBX3	-1.4	1.85E-03	2.73E-02
CMPK1	-1.4	2.29E-03	3.14E-02
SNAP23	-1.4	1.56E-03	2.42E-02
MFN2	-1.4	2.13E-03	3.01E-02
F11R	-1.4	8.31E-04	1.59E-02
MINK1	-1.4	1.34E-05	6.67E-04
ARRB1	-1.4	4.09E-04	9.72E-03
IL6ST	-1.4	1.95E-04	5.63E-03
PRKCA	-1.4	2.70E-03	3.47E-02
TTYH3	-1.4	5.43E-03	5.38E-02
NPTN	-1.4	1.81E-03	2.69E-02
HK1	-1.3	5.14E-05	2.01E-03

ATG9A	-1.3	2.26E-03	3.12E-02
OAZ1	-1.3	5.29E-06	3.03E-04
DNAJB6	-1.3	1.34E-03	2.19E-02
CENPT	-1.3	3.79E-03	4.31E-02
MBNL1	-1.3	5.83E-05	2.22E-03
PADI4	-1.3	1.53E-03	2.39E-02
ADIPOR1	-1.3	5.68E-04	1.22E-02
RAB1B	-1.3	8.55E-04	1.62E-02
VPS37B	-1.3	4.13E-04	9.75E-03
ASAP1	-1.3	6.32E-05	2.36E-03
TUBA1B	-1.3	1.66E-03	2.52E-02
GPI	-1.3	4.78E-04	1.08E-02
WBP2	-1.3	1.82E-05	8.67E-04
CDKN2D	-1.2	2.68E-03	3.46E-02
LPP	-1.2	1.77E-03	2.64E-02
YWHAZ	-1.2	9.11E-07	6.82E-05
TAGLN2	-1.2	8.56E-05	3.02E-03
RAB31	-1.2	5.64E-03	5.49E-02
YOD1	-1.2	2.25E-03	3.10E-02
BCR	-1.2	5.00E-03	5.11E-02
RAB8A	-1.2	3.05E-03	3.76E-02
BNIP3L	-1.1	3.19E-03	3.86E-02
BSDC1	-1.1	2.03E-03	2.91E-02
AP2M1	-1.1	8.91E-04	1.66E-02
MLXIP	-1.1	3.05E-03	3.75E-02
MAP3K5	-1.1	3.99E-03	4.44E-02
USP22	-1.1	5.50E-03	5.41E-02
GOLGA2	-1.1	7.44E-04	1.47E-02
BIN2	-1.1	2.36E-03	3.18E-02
TMOD3	-1.1	4.03E-03	4.47E-02
ITPR2	-1.0	4.03E-04	9.63E-03
MAVS	-1.0	2.26E-03	3.12E-02
PLXDC2	-1.0	8.27E-04	1.59E-02
RABGAP1L	-1.0	2.06E-03	2.94E-02
MAPK6	-1.0	3.53E-03	4.13E-02
ARF3	-1.0	5.63E-03	5.49E-02
FURIN	-1.0	4.41E-04	1.03E-02

Gene ID	log 2Fold Change (edgeR)	pValue (edgeR)	pAdj (edgeR)
LOC112267867	6.8	5.10E-14	1.60E-11
LOC105369595	6.4	4.79E-09	6.11E-07
LOC102723809	6.1	2.55E-11	5.06E-09
SNORD14E	5.9	5.17E-11	9.80E-09
PCCA-AS1	5.9	2.74E-08	2.90E-06
C10orf91	5.9	1.20E-12	3.11E-10
SNORA27	5.8	2.97E-12	7.11E-10
SNORA16B	5.7	1.27E-08	1.43E-06
SNORA70G	5.6	1.24E-10	2.19E-08
MIR1250	5.6	1.13E-06	8.14E-05
LOC101928576	5.6	5.99E-10	9.45E-08
LGALS16	5.5	1.14E-04	3.76E-03
SNORA55	5.5	1.74E-09	2.44E-07
LOC100505585	5.5	3.49E-09	4.56E-07
LOC101927884	5.4	1.41E-05	7.00E-04
UBE2D3-AS1	5.4	2.20E-09	3.02E-07
LSP1P4	5.3	1.10E-04	3.67E-03
LOC105379331	5.3	1.64E-05	7.92E-04
LOC100129083	5.3	4.78E-09	6.11E-07
PLCB1-IT1	5.3	5.06E-07	4.03E-05
MIR640	5.3	4.22E-06	2.49E-04
SNORD175	5.2	3.14E-09	4.14E-07
SNORD45B	5.2	1.70E-06	1.14E-04
LOC107987304	5.2	1.06E-10	1.89E-08
**HEY1**	5.2	5.05E-12	1.14E-09
LOC112268097	5.2	1.61E-07	1.44E-05
**IFNG**	5.2	8.12E-09	9.72E-07
LOC105376899	5.2	2.43E-04	6.62E-03
MIR326	5.2	1.08E-07	1.01E-05
LOC101929154	5.2	5.66E-07	4.50E-05
GUSBP10	5.2	1.64E-04	4.98E-03
MIR6077	5.2	1.35E-07	1.24E-05
LOC105369747	5.2	8.49E-09	1.01E-06
TREML5P	5.1	3.61E-08	3.72E-06
LOC107984650	5.1	1.08E-07	1.01E-05
LOC105378078	5.1	3.93E-08	4.00E-06
LOC101059954	5.1	1.56E-09	2.21E-07
LOC101927164	5.1	2.14E-05	9.88E-04
LOC107985707	5.1	2.60E-03	3.38E-02
LOC107984667	5.1	7.13E-10	1.11E-07
LOC105375220	5.1	4.88E-04	1.10E-02
MIR378E	5.1	9.12E-08	8.76E-06
LOC112268014	5.0	4.33E-09	5.58E-07
**IL12A**	5.0	5.56E-06	3.17E-04

LOC112268247	5.0	1.97E-05	9.24E-04
**CXCL10**	5.0	4.38E-06	2.59E-04
LOC105377621	5.0	2.10E-10	3.63E-08
LOC105369225	5.0	2.62E-08	2.78E-06
SNORD14B	5.0	1.80E-09	2.51E-07
MIR3613	4.9	1.14E-07	1.07E-05
**ZNF540**	4.9	4.22E-10	6.83E-08
CNNM3-DT	4.9	3.27E-06	2.02E-04
LOC107985200	4.9	4.48E-03	4.79E-02
LOC105373433	4.8	5.66E-04	1.21E-02
SNORD103A	4.8	3.11E-06	1.94E-04
KCNQ1-AS1	4.8	2.30E-08	2.48E-06
SNORD53	4.8	1.32E-07	1.21E-05
FLNC-AS1	4.8	1.16E-05	5.96E-04
SPANXD	4.8	2.08E-03	2.96E-02
MIR7850	4.8	3.20E-04	8.13E-03
C2-AS1	4.8	7.47E-06	4.10E-04
LOC107985522	4.8	3.62E-09	4.70E-07
MIR221	4.8	6.77E-06	3.76E-04
LOC112268251	4.8	1.59E-07	1.44E-05
SNORD98	4.8	2.74E-07	2.31E-05
LOC107985118	4.8	5.82E-04	1.24E-02
MIR372	4.8	1.47E-05	7.23E-04
KRT16P1	4.8	5.04E-03	5.12E-02
NPHP3	4.8	6.60E-10	1.03E-07
SEPSECS-AS1	4.7	3.70E-06	2.25E-04
MIR590	4.7	2.04E-07	1.77E-05
SNORD116-6	4.7	1.20E-04	3.90E-03
LOC105371762	4.7	1.81E-07	1.59E-05
LRRK2-DT	4.7	1.79E-04	5.29E-03
TMEM191B	4.7	3.99E-07	3.26E-05
LOC105373124	4.7	8.02E-04	1.56E-02
LOC112268043	4.7	2.09E-06	1.38E-04
LOC101929290	4.7	6.55E-06	3.68E-04
ANKRD20A11P	4.7	3.86E-05	1.60E-03
LINC02551	4.6	1.10E-05	5.70E-04
LOC107986576	4.6	1.97E-06	1.30E-04
LINC02508	4.6	2.34E-03	3.17E-02
KRTAP12-1	4.6	1.69E-03	2.55E-02
LINC00593	4.6	2.80E-06	1.77E-04
MIR647	4.6	1.48E-05	7.25E-04
C1QTNF3-AMACR	4.6	2.58E-03	3.36E-02
LOC101927972	4.6	7.42E-06	4.08E-04
SNORD103B	4.6	3.20E-06	1.99E-04
LOC107984986	4.5	2.73E-05	1.21E-03
MIR6835	4.5	1.17E-03	2.01E-02
MIR4648	4.5	8.86E-06	4.76E-04

LOC101928077	4.5	3.37E-06	2.07E-04
MIR3939	4.5	2.50E-06	1.61E-04
MIR619	4.5	1.93E-04	5.58E-03
GTF3C2-AS1	4.5	2.12E-06	1.39E-04
CREB3L2-AS1	4.5	2.55E-05	1.14E-03
GEMIN7-AS1	4.5	1.27E-04	4.05E-03
LOC101927620	4.5	2.06E-05	9.59E-04
MIR181A2HG	4.5	3.49E-05	1.47E-03
DTX1	4.5	2.23E-10	3.83E-08
SNORD134	4.5	3.79E-06	2.29E-04
MIR6884	4.5	9.74E-05	3.35E-03
LOC105372934	4.4	1.79E-04	5.29E-03
LOC102723692	4.4	1.60E-06	1.09E-04
MMP23B	4.4	7.66E-09	9.22E-07
LOC105369593	4.4	1.01E-04	3.42E-03
LOC105379273	4.4	7.35E-05	2.68E-03
FARSA-AS1	4.4	4.82E-04	1.09E-02
LOC541473	4.4	4.58E-06	2.69E-04
OR2A14	4.4	1.12E-03	1.96E-02
UBXN10-AS1	4.4	2.14E-06	1.40E-04
MIR3183	4.4	3.16E-05	1.36E-03
LOC102724723	4.4	3.37E-05	1.43E-03
LOC101928716	4.4	7.78E-04	1.53E-02
LOC102723838	4.4	2.80E-03	3.55E-02
LOC107985997	4.4	6.87E-05	2.54E-03
LOC105378450	4.4	1.80E-04	5.30E-03
ATRIP	4.4	8.94E-09	1.06E-06
LOC105369728	4.4	3.91E-05	1.61E-03
GUCA2A	4.4	8.03E-05	2.87E-03
SNORD63	4.4	1.90E-06	1.26E-04
LOC107984734	4.4	7.35E-04	1.46E-02
MIR7848	4.4	4.17E-06	2.47E-04
ZHX1-C8orf76	4.4	2.74E-06	1.75E-04
LOC105370485	4.4	3.64E-07	2.99E-05
LOC107984336	4.4	2.48E-05	1.11E-03
LOC105375215	4.4	4.13E-04	9.77E-03
LOC107986948	4.4	2.36E-06	1.53E-04
LOC107984750	4.3	2.98E-04	7.74E-03
KCNRG	4.3	5.10E-08	5.10E-06
LOC105379227	4.3	3.21E-04	8.15E-03
TMEM269-DT	4.3	9.14E-06	4.89E-04
CHRFAM7A	4.3	1.54E-06	1.05E-04
LOC105372829	4.3	1.15E-04	3.77E-03
TNFRSF6B	4.3	1.34E-06	9.41E-05
LOC105378698	4.3	1.79E-07	1.59E-05
AS3MT	4.3	2.70E-03	3.47E-02
SNORA87	4.3	2.77E-05	1.22E-03

LOC105376861	4.3	6.48E-04	1.33E-02
LOC105372486	4.3	3.16E-05	1.36E-03
PWARSN	4.3	1.38E-06	9.62E-05
ZBTB20-AS3	4.3	3.50E-05	1.47E-03
LOC101928893	4.3	1.67E-06	1.13E-04
LOC105377331	4.3	1.32E-03	2.18E-02
LOC105373255	4.3	1.03E-06	7.52E-05
RNASE4	4.3	8.53E-08	8.24E-06
OR52N1	4.3	4.56E-03	4.85E-02
MIR5087	4.3	2.23E-05	1.02E-03
LOC105373393	4.3	1.49E-03	2.35E-02
LINC02311	4.3	9.56E-04	1.75E-02
LOC102724015	4.3	8.03E-11	1.49E-08
LOC105372960	4.3	3.03E-05	1.32E-03
FOXD4L4	4.2	1.26E-03	2.11E-02
RNU6-2	4.2	1.20E-05	6.13E-04
MIR193A	4.2	1.79E-04	5.29E-03
RASSF1-AS1	4.2	1.48E-08	1.65E-06
MIR6731	4.2	1.43E-03	2.29E-02
LOC105376578	4.2	8.66E-07	6.50E-05
USP12-AS1	4.2	4.80E-06	2.79E-04
ZFX-AS1	4.2	1.09E-05	5.66E-04
WSPAR	4.2	1.64E-04	4.97E-03
LOC105374777	4.2	4.36E-07	3.51E-05
OSBPL10-AS1	4.2	1.28E-03	2.14E-02
MIR6842	4.2	7.69E-06	4.20E-04
LOC105373652	4.2	1.44E-06	9.99E-05
LOC105378672	4.2	2.81E-07	2.36E-05
LOC105372441	4.2	1.35E-03	2.20E-02
LOC105371316	4.2	2.09E-04	5.95E-03
LOC107985943	4.2	6.31E-04	1.31E-02
LOC105370452	4.2	5.83E-04	1.25E-02
LOC105378355	4.2	1.72E-04	5.14E-03
THRA1/BTR	4.1	3.62E-05	1.51E-03
MIR765	4.1	2.20E-05	1.01E-03
LOC100129203	4.1	1.16E-06	8.32E-05
LOC102723575	4.1	5.04E-04	1.12E-02
BMS1P4	4.1	1.30E-05	6.56E-04
LOC105374332	4.1	7.12E-05	2.61E-03
PATE4	4.1	4.55E-03	4.85E-02
MIR3177	4.1	1.22E-04	3.95E-03
LOC105379343	4.1	2.51E-05	1.13E-03
LOC101927573	4.1	1.24E-05	6.30E-04
SNORD83A	4.1	2.70E-05	1.20E-03
LOC105371910	4.1	3.09E-06	1.94E-04
RNU5D-1	4.1	3.35E-06	2.07E-04
PNMA6A	4.1	4.72E-04	1.07E-02

KLRK1	4.1	1.01E-05	5.31E-04
LINC02236	4.1	3.38E-03	4.00E-02
KIAA1614-AS1	4.1	5.57E-04	1.20E-02
LINC02478	4.1	2.00E-04	5.74E-03
LOC107985683	4.1	1.09E-03	1.93E-02
CHAC2	4.1	1.28E-05	6.49E-04
LOC105379241	4.1	7.73E-05	2.78E-03
LOC100507387	4.1	4.27E-05	1.73E-03
LOC101927243	4.0	1.19E-06	8.52E-05
MIR12114	4.0	6.03E-04	1.27E-02
SNORD54	4.0	1.02E-05	5.33E-04
LOC105373689	4.0	1.25E-06	8.86E-05
LOC107985873	4.0	4.39E-07	3.53E-05
LOC105374721	4.0	4.06E-05	1.67E-03
NDUFAF2	4.0	2.17E-07	1.87E-05
LOC107986577	4.0	5.43E-03	5.38E-02
OR4S2	4.0	4.04E-03	4.48E-02
LOC107985262	4.0	9.53E-06	5.06E-04
SNORA54	4.0	2.73E-06	1.74E-04
LOC112268099	4.0	1.02E-05	5.35E-04
MIR6075	4.0	2.41E-06	1.55E-04
MIR4742	4.0	3.90E-05	1.61E-03
LOC107986490	4.0	1.87E-04	5.43E-03
EML2-AS1	4.0	8.63E-07	6.48E-05
UCN	4.0	7.59E-05	2.75E-03
RNVU1-4	4.0	1.98E-05	9.31E-04
LOC107985020	4.0	1.12E-05	5.80E-04
LOC105373488	4.0	3.88E-04	9.37E-03
TFF1	4.0	4.19E-03	4.58E-02
LOC101928140	4.0	1.98E-04	5.70E-03
RPS10P7	4.0	2.17E-07	1.87E-05
MDS2	4.0	4.80E-06	2.79E-04
SNORD136	4.0	1.73E-05	8.32E-04
TMLHE-AS1	4.0	1.71E-04	5.12E-03
FSCB	4.0	2.42E-03	3.24E-02
LOC105376326	4.0	1.24E-03	2.09E-02
LOC105376281	4.0	1.06E-04	3.57E-03
LOC105376876	4.0	6.62E-06	3.69E-04
LOC107984676	4.0	2.26E-03	3.12E-02
SNORD11	3.9	4.93E-05	1.94E-03
LOC102723305	3.9	7.90E-04	1.54E-02
LOC728613	3.9	1.73E-07	1.55E-05
LINC01395	3.9	6.56E-05	2.44E-03
PRAMEF6	3.9	3.01E-03	3.73E-02
LIMS1-AS1	3.9	2.18E-05	1.01E-03
LOC107986700	3.9	3.26E-03	3.92E-02
LOC105372664	3.9	3.78E-03	4.31E-02

MIR6885	3.9	4.02E-03	4.46E-02
CD27	3.9	2.02E-10	3.51E-08
LOC105370943	3.9	1.22E-06	8.72E-05
LOC105372432	3.9	3.35E-05	1.42E-03
LOC112268418	3.9	1.07E-03	1.90E-02
LINC02241	3.9	1.64E-03	2.50E-02
TAF1A-AS1	3.9	8.55E-05	3.02E-03
LOC100996385	3.9	1.19E-05	6.09E-04
LINC01659	3.9	8.87E-04	1.66E-02
MIRLET7G	3.9	3.54E-03	4.13E-02
MIR548AR	3.9	8.51E-04	1.62E-02
MIR4691	3.9	1.44E-04	4.49E-03
LOC101927278	3.9	2.31E-07	1.98E-05
IFITM5	3.9	6.12E-07	4.81E-05
MIR4470	3.9	1.32E-04	4.15E-03
SNORA41	3.9	7.57E-05	2.74E-03
MIR151B	3.9	4.48E-06	2.63E-04
LOC105374968	3.9	1.39E-04	4.36E-03
LOC107985452	3.9	1.77E-03	2.64E-02
DDIT4L	3.9	9.20E-04	1.70E-02
MIR1207	3.9	1.31E-04	4.15E-03
LOC105372114	3.9	2.87E-04	7.50E-03
ANKRD19P	3.9	7.39E-09	8.95E-07
WASH5P	3.9	2.62E-04	7.01E-03
LINC02207	3.9	1.21E-04	3.93E-03
LOC107985688	3.8	7.27E-06	4.01E-04
EOMES	3.8	9.35E-07	6.93E-05
SNORA38	3.8	3.49E-04	8.67E-03
MEAT6	3.8	1.97E-03	2.85E-02
BEX2	3.8	2.05E-07	1.77E-05
LOC392196	3.8	9.87E-04	1.79E-02
TAS2R14	3.8	1.11E-04	3.67E-03
LOC101927354	3.8	9.84E-05	3.37E-03
MIR1285-1	3.8	4.58E-03	4.85E-02
LOC107984946	3.8	7.73E-05	2.78E-03
LOC105375705	3.8	4.88E-03	5.05E-02
LOC101928556	3.8	1.18E-03	2.02E-02
LOC107986903	3.8	3.62E-03	4.18E-02
LOC105373924	3.8	5.42E-03	5.37E-02
MIR199A1	3.8	2.94E-04	7.66E-03
LOC105377889	3.8	5.03E-03	5.12E-02
LOC107984204	3.8	7.79E-07	5.93E-05
LOC105377995	3.8	5.65E-03	5.50E-02
LOC105378061	3.8	4.50E-05	1.80E-03
LOC112268218	3.8	2.24E-05	1.02E-03
GNRH1	3.8	9.20E-07	6.87E-05
MIR4730	3.8	4.69E-03	4.92E-02

MIR4518	3.8	1.73E-04	5.16E-03
LOC105375767	3.8	2.68E-04	7.12E-03
MIR7854	3.8	2.00E-03	2.87E-02
LOC105372952	3.8	8.04E-06	4.36E-04
MIR7152	3.8	5.18E-05	2.02E-03
LOC105372653	3.8	8.69E-06	4.68E-04
LOC101928323	3.8	9.99E-05	3.40E-03
LOC107986938	3.8	2.07E-03	2.95E-02
LOC107986876	3.8	1.50E-06	1.03E-04
LOC105375781	3.8	3.65E-07	3.00E-05
LINC02424	3.8	4.73E-03	4.94E-02
KRT17P2	3.8	4.61E-05	1.83E-03
LOC105374945	3.8	6.17E-05	2.32E-03
SNORA118	3.8	3.36E-05	1.42E-03
SEC24B-AS1	3.8	1.28E-04	4.09E-03
LOC101928879	3.8	7.40E-05	2.69E-03
LPGAT1-AS1	3.8	7.62E-04	1.50E-02
LOC105371050	3.7	1.83E-04	5.36E-03
MIR4715	3.7	3.96E-04	9.52E-03
SRGAP2-AS1	3.7	4.51E-05	1.81E-03
LOC105374616	3.7	2.63E-03	3.41E-02
BTBD8	3.7	4.23E-05	1.72E-03
RPP38-DT	3.7	9.86E-06	5.20E-04
MIR6859-4	3.7	1.08E-04	3.63E-03
LOC107986935	3.7	4.08E-03	4.50E-02
LOC105372232	3.7	4.02E-05	1.65E-03
AGAP1-IT1	3.7	2.27E-03	3.12E-02
RASA4	3.7	4.74E-07	3.80E-05
KRTAP5-AS1	3.7	1.06E-06	7.70E-05
HOXB4	3.7	1.53E-07	1.39E-05
LINC00868	3.7	4.46E-03	4.78E-02
GRASP-AS1	3.7	9.34E-07	6.93E-05
ACSL3-AS1	3.7	3.98E-05	1.64E-03
LOC107984543	3.7	6.74E-05	2.50E-03
IFNL2	3.7	2.35E-03	3.17E-02
LINC01732	3.7	4.83E-03	5.02E-02
MIR3198-1	3.7	1.26E-04	4.04E-03
RASA4DP	3.7	8.48E-08	8.21E-06
LINC02595	3.7	6.96E-04	1.40E-02
GBP1P1	3.7	6.67E-05	2.47E-03
LOC100653133	3.7	2.94E-05	1.29E-03
LOC729609	3.7	1.91E-03	2.79E-02
LOC105371168	3.7	1.31E-05	6.59E-04
LOC107984813	3.7	1.63E-05	7.90E-04
LOC112268467	3.7	1.69E-05	8.13E-04
FAM153B	3.7	4.78E-06	2.78E-04
FLJ46906	3.7	2.25E-04	6.26E-03

LOC107984736	3.7	5.90E-05	2.24E-03
MIR6773	3.7	5.88E-05	2.24E-03
MIR3140	3.7	3.05E-04	7.87E-03
LOC107985782	3.7	2.36E-03	3.19E-02
LRFN3	3.7	2.20E-08	2.39E-06
LOC107985894	3.7	5.10E-03	5.17E-02
LOC107984997	3.7	1.81E-03	2.69E-02
SNORD46	3.7	3.02E-04	7.82E-03
LOC105377994	3.7	1.55E-05	7.56E-04
LOC107984139	3.7	2.64E-04	7.04E-03
ALG1L9P	3.7	1.83E-05	8.69E-04
LMF1-AS1	3.7	1.47E-04	4.58E-03
ARHGAP26-IT1	3.7	3.24E-06	2.00E-04
LOC107985568	3.7	1.83E-04	5.35E-03
**WNT7A**	3.7	1.55E-07	1.40E-05
LOC102723594	3.7	8.78E-04	1.65E-02
FLVCR2	3.7	2.79E-06	1.77E-04
LOC105371425	3.7	5.43E-03	5.38E-02
LOC105373785	3.7	1.28E-06	9.08E-05
MIR32	3.7	2.33E-03	3.16E-02
SNORA70B	3.7	5.05E-03	5.14E-02
MIR4285	3.6	1.15E-04	3.77E-03
LOC112268296	3.6	3.41E-06	2.10E-04
LOC100505555	3.6	1.57E-04	4.83E-03
LOC102724301	3.6	2.60E-03	3.38E-02
MCRIP2	3.6	2.60E-06	1.67E-04
LOC105378925	3.6	1.04E-04	3.51E-03
LOC105376712	3.6	2.07E-05	9.62E-04
LOC105376755	3.6	7.79E-04	1.53E-02
MIR548O	3.6	2.81E-04	7.39E-03
IFNA14	3.6	4.83E-03	5.02E-02
H4C6	3.6	6.26E-06	3.53E-04
LOC107987192	3.6	5.52E-06	3.15E-04
LOC105375947	3.6	2.05E-03	2.93E-02
TMEM256-PLSCR3	3.6	8.79E-04	1.65E-02
LOC105371792	3.6	5.31E-04	1.16E-02
SNORA5C	3.6	1.57E-05	7.66E-04
LOC102723582	3.6	1.44E-05	7.12E-04
MIR4446	3.6	4.92E-04	1.10E-02
KU-MEL-3	3.6	2.64E-03	3.42E-02
LOC107986662	3.6	2.28E-03	3.14E-02
SNORD139	3.6	4.87E-06	2.82E-04
RALY-AS1	3.6	1.83E-05	8.69E-04
PRR15L	3.6	4.91E-04	1.10E-02
MIR4633	3.6	8.62E-04	1.63E-02
SNORD58A	3.6	3.06E-04	7.89E-03
SNORD13F	3.6	1.20E-04	3.91E-03

LINC01629	3.6	1.11E-03	1.94E-02
SNORD42A	3.6	1.64E-03	2.50E-02
LINC02574	3.6	3.07E-05	1.33E-03
LOC107984844	3.6	3.64E-04	8.91E-03
GCHFR	3.6	4.02E-05	1.65E-03
LOC101929767	3.6	8.55E-06	4.62E-04
RNU5E-1	3.6	7.18E-05	2.62E-03
LOC101929579	3.6	2.29E-03	3.14E-02
MIR4434	3.6	4.41E-04	1.03E-02
MIR3161	3.6	5.92E-04	1.26E-02
SNORD5	3.5	2.87E-05	1.26E-03
MIR617	3.5	5.11E-03	5.17E-02
LINC01887	3.5	2.68E-03	3.45E-02
OR4L1	3.5	2.58E-03	3.37E-02
LOC112267973	3.5	4.57E-05	1.82E-03
LOC101928100	3.5	8.70E-06	4.68E-04
PGAM4	3.5	4.00E-06	2.38E-04
OSER1-DT	3.5	5.96E-07	4.70E-05
SNORD77	3.5	5.52E-05	2.13E-03
SPDYE10P	3.5	1.40E-04	4.38E-03
ANTXRLP1	3.5	7.52E-05	2.73E-03
MIR544B	3.5	1.92E-03	2.79E-02
MIR7150	3.5	3.92E-05	1.62E-03
LINC01500	3.5	2.11E-03	2.99E-02
LOC107986186	3.5	6.15E-05	2.31E-03
LINC00221	3.5	2.60E-04	6.96E-03
FAM240A	3.5	1.11E-03	1.95E-02
LOC105370165	3.5	1.39E-03	2.25E-02
MIR3921	3.5	4.51E-04	1.04E-02
LOC105376409	3.5	1.11E-03	1.95E-02
LOC105378939	3.5	1.60E-04	4.89E-03
**CNTF**	3.5	1.97E-03	2.85E-02
GGNBP1	3.5	8.46E-04	1.61E-02
DINOL	3.5	3.90E-03	4.38E-02
LINC02843	3.5	2.55E-03	3.34E-02
LOC105374492	3.5	6.65E-04	1.35E-02
LOC107984699	3.5	8.84E-04	1.65E-02
CDC37L1-DT	3.5	2.41E-04	6.60E-03
LOC102724080	3.5	6.56E-04	1.34E-02
LOC105369755	3.5	6.93E-05	2.55E-03
CHAER1	3.5	2.72E-03	3.49E-02
H2BE1	3.5	6.89E-05	2.54E-03
MIR505	3.5	2.96E-03	3.69E-02
CERNA2	3.5	3.57E-04	8.82E-03
LOC107985556	3.5	7.90E-06	4.30E-04
LOC105378706	3.5	1.21E-03	2.06E-02
LOC107985282	3.5	2.16E-04	6.11E-03

LINC01646	3.5	9.44E-04	1.74E-02
LINC01032	3.5	1.66E-04	5.01E-03
LOC102723996	3.5	2.32E-04	6.40E-03
LOC107985542	3.5	3.29E-04	8.30E-03
LOC105369811	3.5	2.41E-03	3.23E-02
SLC25A19	3.5	4.40E-06	2.59E-04
LOC101927476	3.5	2.99E-03	3.71E-02
LOC107985499	3.5	2.99E-03	3.71E-02
IL4I1	3.5	1.52E-06	1.04E-04
MORN2	3.5	3.39E-04	8.48E-03
NKX3-1	3.5	1.29E-04	4.10E-03
CHI3L2	3.5	2.46E-05	1.11E-03
TMSB15B-AS1	3.5	1.07E-05	5.57E-04
LOC105374101	3.5	2.56E-04	6.89E-03
SNORD19B	3.5	6.30E-04	1.30E-02
ARL2-SNX15	3.5	4.71E-03	4.93E-02
MIR6728	3.5	7.01E-04	1.41E-02
LOC105370255	3.4	1.38E-03	2.24E-02
MIR4266	3.4	5.41E-03	5.37E-02
LINC01863	3.4	1.20E-03	2.05E-02
**CD3D**	3.4	3.85E-06	2.31E-04
LOC105377191	3.4	7.66E-04	1.51E-02
ZNF451-AS1	3.4	2.87E-07	2.41E-05
ENHO	3.4	3.59E-04	8.84E-03
SREBF2-AS1	3.4	7.22E-07	5.55E-05
LOC112267909	3.4	4.78E-03	4.99E-02
LOC105372821	3.4	5.16E-03	5.20E-02
MIR4655	3.4	1.02E-03	1.84E-02
LOC105376568	3.4	5.58E-05	2.14E-03
LOC105370148	3.4	1.97E-04	5.67E-03
LOC105374209	3.4	1.98E-04	5.70E-03
LOC105379327	3.4	2.17E-03	3.04E-02
C11orf98	3.4	5.01E-03	5.11E-02
GLYCAM1	3.4	2.68E-03	3.46E-02
LOC107986418	3.4	4.17E-03	4.57E-02
LOC105370459	3.4	1.73E-04	5.16E-03
SNORA11F	3.4	2.66E-04	7.09E-03
LOC105374470	3.4	3.33E-03	3.97E-02
CCER2	3.4	5.04E-05	1.97E-03
LMNTD2-AS1	3.4	4.38E-04	1.02E-02
HDAC4-AS1	3.4	5.08E-05	1.99E-03
LOC107987281	3.4	8.71E-05	3.06E-03
OR8G5	3.4	1.06E-03	1.89E-02
SNORD82	3.4	9.46E-04	1.74E-02
MIR4740	3.4	2.67E-03	3.44E-02
MIR9902-2	3.4	3.53E-04	8.74E-03
PLSCR3	3.4	4.68E-06	2.74E-04

ALOX15P1	3.4	4.25E-05	1.72E-03
MRGPRD	3.4	2.15E-04	6.10E-03
LOC105371592	3.4	2.73E-04	7.22E-03
CLEC10A	3.4	4.63E-05	1.84E-03
LINC01072	3.4	2.81E-03	3.56E-02
VASH1-AS1	3.4	2.35E-04	6.49E-03
LOC105378481	3.4	1.42E-03	2.28E-02
FALEC	3.4	1.97E-03	2.85E-02
MIR191	3.4	1.25E-03	2.10E-02
LRRC70	3.4	2.76E-04	7.28E-03
LOC105371677	3.4	2.49E-03	3.30E-02
LOC105371372	3.4	1.19E-03	2.04E-02
LOC105369441	3.4	1.35E-03	2.21E-02
APOA1-AS	3.4	6.01E-04	1.27E-02
LOC107986116	3.4	2.81E-03	3.56E-02
CMC4	3.4	9.25E-05	3.21E-03
LOC105373331	3.4	3.65E-04	8.93E-03
DLGAP1-AS4	3.4	4.72E-03	4.94E-02
MIR646	3.4	2.28E-03	3.13E-02
GIMAP2	3.3	2.46E-05	1.11E-03
LOC105373977	3.3	7.07E-05	2.59E-03
KIF1C-AS1	3.3	8.70E-05	3.06E-03
MIR5587	3.3	4.88E-03	5.05E-02
MEF2B	3.3	9.34E-04	1.73E-02
LOC613038	3.3	4.27E-04	1.00E-02
LINC02768	3.3	9.07E-04	1.68E-02
SMIM27	3.3	3.07E-05	1.33E-03
LOC112267855	3.3	6.34E-06	3.56E-04
SNORA63	3.3	8.74E-04	1.64E-02
CATSPER2P1	3.3	1.44E-05	7.13E-04
LOC105372396	3.3	1.50E-03	2.36E-02
LOC105376572	3.3	3.50E-03	4.10E-02
TNNC1	3.3	3.83E-04	9.26E-03
LOC105369892	3.3	3.26E-03	3.91E-02
LOC107986845	3.3	1.85E-04	5.40E-03
LOC101928708	3.3	4.18E-03	4.58E-02
LOC105378414	3.3	2.02E-03	2.90E-02
LOC107985031	3.3	2.46E-03	3.27E-02
LOC105377166	3.3	5.38E-03	5.34E-02
LOC102723963	3.3	3.13E-03	3.82E-02
LOC112268469	3.3	1.67E-03	2.53E-02
ARHGEF35-AS1	3.3	1.13E-03	1.97E-02
LOC107986433	3.3	1.13E-04	3.74E-03
LOC105370202	3.3	3.31E-03	3.95E-02
LOC105375763	3.3	2.66E-03	3.44E-02
LOC105371532	3.3	9.45E-06	5.04E-04
LOC107987292	3.3	2.03E-03	2.91E-02

HMSD	3.3	7.00E-04	1.41E-02
FFAR1	3.3	1.89E-03	2.77E-02
SNORD24	3.3	5.86E-04	1.25E-02
LOC105369816	3.3	1.68E-04	5.06E-03
SNORD143	3.3	1.47E-03	2.33E-02
SNORD36C	3.3	1.90E-03	2.77E-02
ZNF703	3.3	2.08E-05	9.67E-04
VCAN-AS1	3.3	2.04E-04	5.84E-03
SNORD16	3.3	5.87E-04	1.25E-02
PRSS1	3.3	5.98E-05	2.26E-03
MSH5	3.3	1.62E-04	4.93E-03
LOC107985848	3.3	4.43E-03	4.76E-02
LOC107985573	3.3	1.16E-04	3.80E-03
LOC105372553	3.3	9.77E-05	3.35E-03
LOC102724378	3.3	1.01E-05	5.31E-04
MIR4522	3.3	1.24E-03	2.09E-02
SCARNA4	3.3	4.71E-04	1.07E-02
LOC107985125	3.3	3.11E-03	3.81E-02
FAM153CP	3.2	1.53E-04	4.72E-03
PTRH1	3.2	1.11E-03	1.95E-02
LOC105370688	3.2	2.03E-03	2.91E-02
APOOP5	3.2	7.08E-04	1.42E-02
SNORA119	3.2	5.43E-03	5.38E-02
SAC3D1	3.2	1.34E-05	6.69E-04
LOC107986668	3.2	2.29E-05	1.04E-03
RNU5F-1	3.2	9.82E-06	5.19E-04
SNORA16A	3.2	3.38E-04	8.46E-03
LOC107987244	3.2	2.44E-05	1.10E-03
S100B	3.2	1.72E-04	5.14E-03
LOC107985863	3.2	3.12E-03	3.81E-02
CBX3P2	3.2	2.86E-05	1.26E-03
IL12RB1	3.2	3.19E-06	1.98E-04
LOC105374363	3.2	2.76E-04	7.28E-03
LOC155060	3.2	2.03E-05	9.48E-04
AGPAT4-IT1	3.2	7.73E-05	2.78E-03
SNORA37	3.2	9.09E-04	1.69E-02
LOC105371912	3.2	1.47E-05	7.22E-04
**TBX21**	3.2	1.37E-05	6.80E-04
LOC105373741	3.2	4.08E-03	4.50E-02
TEX21P	3.2	1.13E-03	1.97E-02
LOC105372300	3.2	2.12E-05	9.79E-04
LOC105373719	3.2	3.48E-04	8.66E-03
SNORD124	3.2	5.22E-03	5.25E-02
FMC1-LUC7L2	3.2	7.03E-05	2.58E-03
LOC101927822	3.2	3.99E-03	4.44E-02
LOC105373527	3.2	8.32E-04	1.59E-02
LOC105372098	3.2	1.88E-04	5.46E-03

VHLL	3.2	4.80E-03	5.00E-02
LOC105371814	3.2	3.81E-03	4.32E-02
LOC105374369	3.2	1.44E-04	4.50E-03
KLRC3	3.2	7.92E-05	2.83E-03
SNORD101	3.2	5.23E-04	1.15E-02
LOC105375378	3.2	1.39E-03	2.25E-02
SNORD151	3.2	3.64E-03	4.20E-02
MIR573	3.2	1.61E-03	2.48E-02
BRD7P3	3.2	1.16E-04	3.80E-03
CCDC65	3.2	1.61E-05	7.84E-04
LOC105377469	3.2	1.47E-03	2.33E-02
LOC102724389	3.2	3.95E-03	4.41E-02
LOC105374958	3.1	1.55E-03	2.42E-02
MIR186	3.1	8.86E-05	3.10E-03
RNVU1-19	3.1	1.12E-03	1.96E-02
LOC102723752	3.1	7.68E-05	2.77E-03
MIR6880	3.1	5.23E-04	1.15E-02
LINC01355	3.1	3.05E-03	3.75E-02
MCEE	3.1	1.33E-05	6.63E-04
NRIR	3.1	2.29E-03	3.14E-02
C2CD4D-AS1	3.1	5.40E-05	2.09E-03
LOC105372697	3.1	3.08E-03	3.78E-02
LOC105377095	3.1	2.86E-03	3.61E-02
LOC101926948	3.1	4.15E-04	9.78E-03
BRWD1-AS1	3.1	3.86E-03	4.36E-02
MIR1204	3.1	5.45E-03	5.38E-02
LOC107987150	3.1	2.89E-05	1.27E-03
CD52	3.1	1.01E-04	3.42E-03
LOC105374419	3.1	2.32E-03	3.16E-02
SETDB2-PHF11	3.1	3.91E-03	4.39E-02
MIR663AHG	3.1	1.90E-03	2.77E-02
SNORD11B	3.1	2.01E-04	5.76E-03
LOC101929291	3.1	2.05E-03	2.93E-02
LOC105374875	3.1	1.55E-03	2.41E-02
LINC02861	3.1	3.11E-04	7.98E-03
LOC105372804	3.1	3.64E-03	4.21E-02
CA5BP1-CA5B	3.1	1.84E-05	8.72E-04
GZMA	3.1	9.89E-05	3.38E-03
MIR3975	3.1	2.49E-03	3.30E-02
MIR4656	3.1	3.58E-03	4.16E-02
MIR548N	3.1	1.68E-03	2.54E-02
LOC105372233	3.1	3.98E-04	9.56E-03
CD8A	3.1	1.75E-04	5.22E-03
LOC107986583	3.1	1.17E-03	2.01E-02
LOC100630923	3.1	2.56E-04	6.89E-03
SCAT8	3.1	1.20E-05	6.13E-04
PACRGL	3.1	3.62E-06	2.21E-04

FOXP4-AS1	3.1	2.11E-03	2.99E-02
MIR6739	3.1	2.22E-04	6.20E-03
LOC101929691	3.1	2.87E-04	7.50E-03
MIR31HG	3.1	3.11E-03	3.81E-02
SCARNA26B	3.1	9.50E-04	1.75E-02
LOC102724323	3.1	4.72E-04	1.07E-02
SFRP5	3.1	1.07E-03	1.90E-02
LOC107984710	3.1	3.97E-03	4.43E-02
LOC101927057	3.1	1.18E-03	2.03E-02
ATRIP-TREX1	3.1	1.10E-03	1.94E-02
MIR4258	3.1	3.13E-03	3.81E-02
SIRPG	3.1	1.57E-04	4.83E-03
LOC105378457	3.1	3.10E-03	3.80E-02
LINC02257	3.1	1.57E-03	2.43E-02
NRN1L	3.0	4.07E-05	1.67E-03
LOC107985004	3.0	4.49E-03	4.80E-02
LOC107985717	3.0	4.59E-03	4.86E-02
MIR3663HG	3.0	1.59E-03	2.45E-02
LOC107986517	3.0	2.51E-04	6.81E-03
LOC101929633	3.0	8.10E-04	1.57E-02
MIR6832	3.0	1.29E-03	2.15E-02
AFM	3.0	4.96E-03	5.09E-02
MED9	3.0	3.12E-05	1.34E-03
LOC101928126	3.0	1.23E-04	3.97E-03
GIMAP7	3.0	5.47E-05	2.11E-03
HUS1B	3.0	4.27E-04	1.00E-02
HSD11B1L	3.0	1.67E-05	8.03E-04
LOC102724618	3.0	4.12E-04	9.75E-03
LAIR2	3.0	1.63E-04	4.95E-03
SPRY3	3.0	1.33E-03	2.19E-02
LOC107987238	3.0	1.09E-03	1.92E-02
NANOS3	3.0	4.13E-05	1.69E-03
LINC01315	3.0	1.75E-03	2.62E-02
LOC107984839	3.0	2.24E-03	3.10E-02
SNORA5A	3.0	4.68E-04	1.07E-02
ENDOG	3.0	4.58E-05	1.82E-03
SNORD110	3.0	1.18E-03	2.02E-02
CENPM	3.0	8.22E-05	2.93E-03
LOC107985414	3.0	3.70E-04	9.03E-03
LOC105373690	3.0	2.34E-03	3.17E-02
LOC105369772	3.0	6.23E-04	1.29E-02
PROC	3.0	2.29E-05	1.04E-03
TRIM69	3.0	7.86E-04	1.54E-02
FGF7P6	3.0	3.13E-03	3.82E-02
SNORA21B	3.0	2.11E-03	2.99E-02
SERPINA2	3.0	6.91E-04	1.39E-02
LINC01900	3.0	4.98E-03	5.10E-02

SH2D1A	3.0	6.80E-05	2.51E-03
LOC100505501	3.0	5.89E-05	2.24E-03
LINC02363	3.0	1.68E-04	5.07E-03
EDRF1-AS1	3.0	1.00E-03	1.82E-02
NCR3	3.0	1.48E-04	4.59E-03
ZNF32	3.0	1.88E-03	2.76E-02
FGFBP2	3.0	6.29E-04	1.30E-02
LOC105371074	3.0	2.37E-05	1.07E-03
LOC105370790	3.0	1.69E-04	5.08E-03
LOC101927709	3.0	5.01E-03	5.11E-02
DDN-AS1	3.0	1.62E-05	7.88E-04
USP30-AS1	3.0	1.71E-04	5.12E-03
SNORA11	3.0	3.37E-03	3.99E-02
LOC284950	3.0	5.47E-03	5.39E-02
PRR34-AS1	3.0	9.35E-05	3.23E-03
LALBA	3.0	4.97E-03	5.09E-02
SNORD9	3.0	2.30E-03	3.15E-02
LOC105369705	2.9	4.31E-03	4.67E-02
UBE2F-SCLY	2.9	4.29E-03	4.66E-02
LOC105376525	2.9	5.08E-03	5.15E-02
MID1IP1-AS1	2.9	1.55E-03	2.41E-02
C14orf178	2.9	5.76E-04	1.23E-02
LOC107984459	2.9	2.72E-03	3.49E-02
FOXN3-AS2	2.9	1.72E-04	5.14E-03
LOC105375045	2.9	5.62E-03	5.48E-02
CD1C	2.9	5.81E-04	1.24E-02
DTD2	2.9	5.70E-05	2.18E-03
SCARNA9L	2.9	1.28E-03	2.14E-02
LOC107985130	2.9	3.84E-03	4.34E-02
MIR7-1	2.9	3.30E-03	3.94E-02
CELA3B	2.9	4.82E-03	5.01E-02
MIR8064	2.9	2.22E-03	3.08E-02
LOC105372484	2.9	5.11E-03	5.17E-02
SNORD13	2.9	4.02E-04	9.61E-03
TNF	2.9	3.51E-05	1.47E-03
ZBTB42	2.9	7.24E-06	4.00E-04
LOC107985521	2.9	4.81E-03	5.01E-02
HSD17B7	2.9	4.55E-04	1.05E-02
C11orf65	2.9	2.09E-05	9.71E-04
LOC102606465	2.9	1.45E-03	2.31E-02
LOC101928816	2.9	1.80E-04	5.30E-03
SNORA4	2.9	3.37E-04	8.45E-03
LOC105371516	2.9	2.20E-03	3.07E-02
LINC02397	2.9	1.99E-03	2.87E-02
SNORD38C	2.9	4.34E-04	1.01E-02
LINC00266-1	2.9	1.68E-03	2.54E-02
LOC102724660	2.9	3.61E-03	4.18E-02

LOC101927950	2.9	1.26E-03	2.12E-02
ITPR1-DT	2.9	2.21E-04	6.19E-03
OLFML3	2.9	8.57E-05	3.02E-03
PHOSPHO2	2.9	2.18E-04	6.14E-03
CCDC58	2.9	2.19E-03	3.05E-02
LOC105379034	2.9	8.87E-04	1.66E-02
LOC105374717	2.9	5.11E-03	5.17E-02
LOC105374340	2.9	1.60E-03	2.46E-02
CD6	2.9	2.90E-06	1.83E-04
MS4A4A	2.9	7.13E-04	1.42E-02
LOC107986075	2.9	2.70E-04	7.16E-03
LPEQ6126	2.9	1.49E-04	4.59E-03
EEF1DP3	2.9	7.73E-04	1.52E-02
ZNF56	2.9	4.90E-04	1.10E-02
LOC101928037	2.9	2.42E-03	3.24E-02
BIRC7	2.9	2.73E-03	3.50E-02
LOC644656	2.9	3.00E-04	7.76E-03
LINC00894	2.9	7.64E-05	2.76E-03
ZNF624	2.9	1.39E-05	6.91E-04
SNORD36B	2.9	1.66E-03	2.52E-02
LPAR6	2.9	1.68E-04	5.07E-03
LOC107984874	2.9	2.39E-03	3.22E-02
LOC105374245	2.9	2.81E-03	3.56E-02
PLEKHB1	2.9	9.03E-05	3.15E-03
LOC101927543	2.9	6.02E-04	1.27E-02
NPIPB6	2.8	6.87E-04	1.39E-02
MIR4804	2.8	6.70E-04	1.36E-02
ISM1	2.8	1.03E-04	3.49E-03
LOC105369761	2.8	5.46E-04	1.18E-02
PRF1	2.8	5.07E-04	1.13E-02
AGAP4	2.8	8.67E-04	1.63E-02
RNASEH2B-AS1	2.8	8.64E-04	1.63E-02
SMIM40	2.8	3.73E-03	4.27E-02
LOC105373467	2.8	1.59E-04	4.88E-03
LINC02580	2.8	2.63E-03	3.41E-02
ACTG1P4	2.8	5.56E-05	2.14E-03
MIR7113	2.8	2.43E-03	3.24E-02
LOC105379322	2.8	1.33E-03	2.19E-02
SCAT2	2.8	3.95E-05	1.63E-03
SPATA5L1	2.8	2.52E-05	1.13E-03
PRH2	2.8	3.40E-03	4.02E-02
TEPP	2.8	3.98E-04	9.56E-03
LOC105373673	2.8	3.71E-03	4.26E-02
HLA-DPB2	2.8	3.78E-03	4.31E-02
ASPDH	2.8	4.58E-04	1.05E-02
SNORD74	2.8	3.74E-04	9.09E-03
LOC112267951	2.8	3.54E-04	8.75E-03

LOC107987285	2.8	6.16E-04	1.29E-02
NAGPA	2.8	5.17E-05	2.02E-03
PFN1P2	2.8	1.49E-03	2.35E-02
SNORD117	2.8	2.71E-04	7.17E-03
IGSF8	2.8	1.75E-05	8.39E-04
LINC02361	2.8	3.70E-04	9.02E-03
C1QTNF12	2.8	4.50E-04	1.04E-02
MSRB2	2.8	4.50E-05	1.80E-03
FOXP3	2.8	1.12E-04	3.72E-03
HLA-DRB5	2.8	1.13E-04	3.74E-03
LOC107984875	2.8	2.56E-03	3.35E-02
TRUB1	2.8	8.59E-04	1.63E-02
FKSG29	2.8	1.34E-03	2.20E-02
LINC00662	2.8	2.42E-04	6.61E-03
LOC107985306	2.8	3.25E-03	3.90E-02
LOC105369949	2.8	1.33E-03	2.19E-02
RPS14P3	2.8	4.28E-05	1.73E-03
LOC105376467	2.8	2.98E-03	3.70E-02
MIR3138	2.8	4.39E-03	4.74E-02
CSNK1G2-AS1	2.8	2.68E-04	7.12E-03
ANKRD7	2.8	4.57E-03	4.85E-02
LOC101928525	2.8	4.60E-03	4.87E-02
PWP2	2.8	3.35E-03	3.98E-02
LOC107984043	2.8	5.24E-04	1.15E-02
MAN1C1	2.8	2.76E-05	1.22E-03
SIT1	2.8	5.15E-04	1.14E-02
LOC107986487	2.8	4.05E-03	4.49E-02
LOC107984741	2.8	8.71E-05	3.06E-03
MIR4489	2.8	2.00E-03	2.88E-02
LINC01353	2.8	1.32E-03	2.18E-02
LOC100505728	2.8	1.36E-03	2.22E-02
LOC105373925	2.8	2.18E-03	3.05E-02
HCG20	2.8	3.89E-03	4.38E-02
ALG5	2.8	3.33E-04	8.36E-03
MIR3945	2.8	6.00E-04	1.27E-02
IFNG-AS1	2.8	2.92E-04	7.60E-03
LOC112268048	2.8	9.43E-04	1.74E-02
FCRL6	2.8	1.74E-03	2.61E-02
LOC101927226	2.8	5.54E-03	5.44E-02
LINC01948	2.8	1.41E-03	2.26E-02
LOC105375082	2.8	3.57E-03	4.16E-02
PPP4R1-AS1	2.8	3.38E-03	4.00E-02
LINC01002	2.8	2.20E-03	3.06E-02
GRASP	2.8	1.47E-04	4.57E-03
NELL2	2.8	4.62E-04	1.06E-02
TTC9C	2.8	1.23E-04	3.96E-03
CCDC194	2.8	6.48E-04	1.33E-02

TMEM262	2.8	4.66E-03	4.90E-02
LOC112267985	2.8	2.94E-03	3.68E-02
LOC101928370	2.8	4.00E-04	9.59E-03
ZNF596	2.7	2.04E-04	5.84E-03
LOC105376717	2.7	1.41E-03	2.26E-02
ZNF295-AS1	2.7	2.03E-04	5.84E-03
GTF2IRD2	2.7	8.83E-04	1.65E-02
SNORD63B	2.7	1.05E-03	1.88E-02
LOC400464	2.7	3.47E-03	4.07E-02
GNLY	2.7	1.47E-05	7.22E-04
SNORA33	2.7	8.84E-04	1.65E-02
LOC107986604	2.7	2.54E-03	3.33E-02
LOC105370259	2.7	1.62E-03	2.49E-02
SNORD23	2.7	4.93E-03	5.07E-02
LOC114841035	2.7	9.46E-05	3.26E-03
LOC105372095	2.7	1.06E-03	1.89E-02
LOC100130548	2.7	1.49E-03	2.35E-02
LINC02084	2.7	2.56E-04	6.89E-03
LOC101930496	2.7	3.17E-04	8.10E-03
PCBP2-OT1	2.7	7.70E-05	2.78E-03
KLRF1	2.7	4.02E-04	9.62E-03
TPSG1	2.7	2.98E-03	3.70E-02
MIR29B1	2.7	3.61E-03	4.18E-02
RORA-AS1	2.7	1.10E-03	1.94E-02
LOC100289230	2.7	2.39E-03	3.21E-02
CCL4L2	2.7	5.92E-04	1.26E-02
DPRXP4	2.7	3.85E-04	9.31E-03
RNU105B	2.7	5.49E-03	5.41E-02
LOC105369820	2.7	1.07E-03	1.90E-02
C9orf72	2.7	8.96E-05	3.13E-03
MIR6071	2.7	5.56E-03	5.44E-02
MIR7845	2.7	1.63E-03	2.50E-02
LOC105373021	2.7	3.73E-03	4.27E-02
PRKRIP1	2.7	1.47E-04	4.57E-03
C10orf95	2.7	4.49E-03	4.80E-02
LOC112268274	2.7	9.31E-05	3.22E-03
LOC105376023	2.7	4.87E-03	5.05E-02
ABHD14A	2.7	1.32E-03	2.18E-02
TAS2R4	2.7	1.51E-03	2.37E-02
LOC105377021	2.7	2.67E-04	7.10E-03
SETDB2	2.7	4.43E-04	1.03E-02
KLRD1	2.7	1.87E-05	8.84E-04
LOC101929650	2.7	5.36E-03	5.33E-02
GIMAP4	2.7	1.12E-06	8.07E-05
ARF4-AS1	2.7	1.90E-03	2.77E-02
LINC01635	2.7	1.95E-03	2.83E-02
LOC107984224	2.7	5.26E-03	5.27E-02

LINC02723	2.7	5.08E-04	1.13E-02
GON7	2.7	5.29E-04	1.16E-02
PAXIP1-AS2	2.7	2.27E-04	6.31E-03
SNORD50B	2.7	9.71E-04	1.77E-02
DAPK1-IT1	2.7	1.59E-03	2.45E-02
CCL3	2.7	7.82E-04	1.53E-02
CCDC112	2.7	2.25E-04	6.27E-03
PMS2P3	2.7	3.90E-05	1.61E-03
PNOC	2.7	4.09E-04	9.72E-03
LOC101926886	2.7	2.91E-04	7.58E-03
C2orf74	2.7	6.89E-04	1.39E-02
THEMIS	2.7	1.17E-03	2.01E-02
LOC340357	2.7	3.22E-03	3.88E-02
CCR5	2.7	4.08E-04	9.70E-03
LINC00304	2.7	8.59E-04	1.63E-02
LSM10	2.7	2.08E-04	5.94E-03
SNHG25	2.7	1.87E-03	2.75E-02
KLRC4	2.7	3.13E-03	3.81E-02
ARMC1	2.7	7.26E-04	1.45E-02
TNFRSF18	2.7	3.05E-04	7.86E-03
MS4A7	2.7	3.59E-04	8.84E-03
PCOLCE-AS1	2.7	5.22E-04	1.15E-02
MIR12122	2.7	2.56E-03	3.35E-02
NOP14-AS1	2.6	2.47E-05	1.11E-03
EEF1E1	2.6	1.28E-03	2.13E-02
TIFA	2.6	1.25E-04	4.00E-03
LOC107986463	2.6	1.73E-03	2.60E-02
DIAPH1-AS1	2.6	4.71E-04	1.07E-02
C17orf77	2.6	2.43E-03	3.24E-02
CARD11	2.6	2.87E-05	1.26E-03
RNA45SN3	2.6	5.32E-04	1.16E-02
PI3	2.6	3.40E-03	4.02E-02
LOC105372497	2.6	4.64E-03	4.88E-02
ZSWIM3	2.6	2.25E-04	6.27E-03
UFSP2	2.6	1.70E-04	5.11E-03
TLDC2	2.6	2.94E-04	7.66E-03
LOC105373241	2.6	1.31E-03	2.17E-02
CLECL1	2.6	2.58E-03	3.36E-02
LOC102723795	2.6	1.66E-03	2.52E-02
REREP3	2.6	2.32E-03	3.15E-02
RIOK2	2.6	1.49E-03	2.35E-02
TGDS	2.6	7.79E-04	1.53E-02
IL6R-AS1	2.6	8.15E-04	1.58E-02
SIGIRR	2.6	7.83E-05	2.81E-03
LOC105376713	2.6	2.41E-03	3.23E-02
SNORD116-18	2.6	3.11E-03	3.81E-02
EZR-AS1	2.6	2.07E-03	2.95E-02

LOC107986673	2.6	2.86E-03	3.61E-02
VCAN	2.6	6.30E-09	7.71E-07
LOC200772	2.6	6.83E-04	1.38E-02
CHCHD4	2.6	6.39E-04	1.31E-02
KBTBD8	2.6	1.68E-04	5.06E-03
LINC00412	2.6	4.14E-03	4.56E-02
MRPL54	2.6	3.75E-04	9.11E-03
TMOD4	2.6	2.08E-03	2.96E-02
SNORD2	2.6	6.18E-04	1.29E-02
LOC105372235	2.6	2.39E-03	3.22E-02
MBOAT4	2.6	1.14E-03	1.97E-02
ARHGAP26-AS1	2.6	1.33E-03	2.19E-02
ARHGAP27P2	2.6	9.42E-04	1.74E-02
LOC105378671	2.6	2.62E-03	3.40E-02
C19orf73	2.6	5.52E-03	5.42E-02
MIR155HG	2.6	3.13E-04	8.01E-03
LINC002481	2.6	6.02E-06	3.40E-04
LOC100505622	2.6	2.44E-04	6.66E-03
MS4A6A	2.6	1.65E-04	4.98E-03
ADAT3	2.6	1.08E-04	3.63E-03
LGALS2	2.6	3.38E-03	4.00E-02
SNORD142	2.6	4.17E-03	4.57E-02
ANGPTL6	2.6	2.04E-04	5.84E-03
PDK4	2.6	6.94E-04	1.40E-02
FH	2.6	1.84E-04	5.37E-03
SLC16A11	2.6	4.52E-05	1.81E-03
RAB3A	2.6	1.82E-03	2.70E-02
LOC105375060	2.6	1.97E-03	2.85E-02
MRS2P2	2.6	7.10E-04	1.42E-02
LOC105369326	2.6	1.92E-03	2.79E-02
CD247	2.6	9.64E-05	3.31E-03
LOC101929704	2.6	4.09E-03	4.51E-02
EREG	2.6	4.74E-04	1.07E-02
CCDC78	2.6	7.24E-04	1.44E-02
PRRT1	2.6	6.21E-04	1.29E-02
TMEM218	2.6	1.00E-04	3.41E-03
PEX13	2.6	9.56E-05	3.30E-03
GPR52	2.6	3.75E-03	4.28E-02
MIR624	2.6	3.98E-03	4.44E-02
DOC2GP	2.6	5.51E-03	5.41E-02
TBC1D22A-AS1	2.6	1.24E-03	2.10E-02
SNORD20	2.6	3.35E-03	3.98E-02
LOC105379185	2.6	3.96E-03	4.42E-02
WFIKKN1	2.5	2.46E-03	3.27E-02
CD3G	2.5	1.67E-04	5.04E-03
HOOK1	2.5	4.58E-04	1.05E-02
LOC102724608	2.5	1.24E-03	2.09E-02

IL11RA	2.5	1.16E-04	3.80E-03
EPHA1-AS1	2.5	4.61E-04	1.05E-02
THOC7-AS1	2.5	5.50E-03	5.41E-02
KLRB1	2.5	4.86E-03	5.04E-02
SNRK-AS1	2.5	4.56E-04	1.05E-02
LOC107987063	2.5	2.15E-03	3.02E-02
CFAP58-DT	2.5	2.43E-03	3.24E-02
NPIPA7	2.5	1.51E-03	2.37E-02
LOC107986859	2.5	2.81E-03	3.56E-02
TRIM59	2.5	3.04E-03	3.75E-02
MIR9902-1	2.5	5.34E-03	5.31E-02
STX16-NPEPL1	2.5	1.13E-04	3.74E-03
GLRX2	2.5	1.65E-03	2.51E-02
NOS3	2.5	8.18E-04	1.58E-02
MIR3619	2.5	1.85E-03	2.73E-02
CX3CR1	2.5	3.17E-04	8.09E-03
LOC107984952	2.5	1.42E-03	2.28E-02
EDAR	2.5	2.30E-04	6.37E-03
GPA33	2.5	1.90E-03	2.77E-02
LFNG	2.5	3.45E-06	2.12E-04
ERI3-IT1	2.5	4.82E-03	5.01E-02
ASGR2	2.5	8.54E-04	1.62E-02
FAM209A	2.5	1.82E-03	2.70E-02
NDUFAF8	2.5	4.33E-04	1.01E-02
CEPT1	2.5	2.11E-04	6.00E-03
MAPRE3-AS1	2.5	2.74E-03	3.51E-02
BCL2L1-AS1	2.5	3.36E-03	3.99E-02
GIMAP6	2.5	6.45E-05	2.41E-03
FOXN3-AS1	2.5	5.56E-04	1.20E-02
LOC105372401	2.5	1.91E-04	5.54E-03
ANO9	2.5	8.31E-05	2.95E-03
IBA57-DT	2.5	2.77E-03	3.53E-02
CA6	2.5	9.98E-04	1.81E-02
GPR162	2.5	9.55E-04	1.75E-02
LOC101928855	2.5	9.58E-04	1.76E-02
NAPSB	2.5	1.06E-03	1.89E-02
**GATA3**	2.5	4.53E-05	1.81E-03
LOC105374808	2.5	4.53E-03	4.83E-02
LIMD1-AS1	2.5	1.42E-03	2.28E-02
LOC105375505	2.5	1.58E-03	2.44E-02
NCBP2AS2	2.5	4.73E-04	1.07E-02
CNTD1	2.5	1.13E-03	1.97E-02
TNFRSF25	2.5	3.64E-04	8.91E-03
SNORD56B	2.5	4.22E-03	4.60E-02
LOC105371686	2.5	2.15E-03	3.02E-02
**ITGA4**	2.5	2.36E-06	1.53E-04
**CD14**	2.5	6.00E-05	2.27E-03

ERCC5	2.5	8.91E-04	1.66E-02
LOC105371088	2.5	1.36E-03	2.22E-02
LAG3	2.5	4.07E-03	4.50E-02
PTGDS	2.5	1.41E-03	2.27E-02
ZNF157	2.5	4.85E-03	5.03E-02
FAM117B	2.5	1.64E-04	4.97E-03
OSGEPL1	2.5	3.12E-04	8.00E-03
TLR7	2.5	2.38E-03	3.21E-02
SLAMF1	2.5	9.74E-04	1.78E-02
LOC645967	2.5	2.92E-03	3.67E-02
LOC105376478	2.5	2.69E-03	3.46E-02
LOC107986655	2.5	3.24E-04	8.21E-03
MAP3K5-AS1	2.5	3.75E-03	4.28E-02
APBA3	2.5	1.12E-03	1.96E-02
FGD2	2.5	1.33E-04	4.19E-03
MYH3	2.5	5.16E-04	1.14E-02
LMNTD2	2.5	4.57E-04	1.05E-02
FBXO6	2.5	9.91E-04	1.80E-02
CMKLR1	2.5	4.95E-04	1.11E-02
**GZMH**	2.5	2.51E-03	3.31E-02
PAIP2B	2.5	1.06E-03	1.89E-02
DRAM2	2.5	2.71E-04	7.17E-03
LOC101927572	2.5	5.32E-03	5.31E-02
CCL3L3	2.5	1.06E-03	1.89E-02
SNORD141B	2.5	5.08E-04	1.13E-02
AMIGO1	2.4	6.14E-04	1.28E-02
CHKB	2.4	1.91E-03	2.78E-02
DUSP2	2.4	9.95E-04	1.80E-02
LOC107986939	2.4	7.10E-04	1.42E-02
C5orf34-AS1	2.4	2.34E-03	3.17E-02
IRAK1BP1	2.4	1.63E-03	2.50E-02
RAC3	2.4	1.31E-03	2.17E-02
MAIP1	2.4	1.37E-03	2.23E-02
C3orf18	2.4	3.77E-05	1.57E-03
ZACN	2.4	2.15E-03	3.02E-02
MARCHF9	2.4	2.98E-04	7.75E-03
LOC105378629	2.4	3.62E-03	4.19E-02
SLC18B1	2.4	1.58E-03	2.45E-02
LOC102724159	2.4	6.32E-04	1.31E-02
PPP1R2C	2.4	2.88E-03	3.63E-02
FCGR3A	2.4	5.73E-05	2.19E-03
LOC105371224	2.4	2.18E-04	6.14E-03
LINC01366	2.4	5.48E-03	5.40E-02
OLFM1	2.4	1.14E-04	3.76E-03
NAAA	2.4	1.14E-04	3.76E-03
MAFB	2.4	1.21E-04	3.93E-03
MAP3K2-DT	2.4	3.28E-04	8.28E-03

GZMB	2.4	2.16E-03	3.03E-02
RNASE6	2.4	1.18E-04	3.84E-03
ALDOC	2.4	2.62E-03	3.40E-02
MPHOSPH6	2.4	2.86E-05	1.26E-03
SCML4	2.4	2.75E-05	1.22E-03
SSBP4	2.4	7.17E-05	2.62E-03
ZFP37	2.4	4.40E-03	4.75E-02
DTX2P1-UPK3BP1	2.4	1.27E-03	2.12E-02
LINC01238	2.4	4.27E-03	4.64E-02
SLC25A26	2.4	4.63E-04	1.06E-02
MIR3648-2	2.4	1.27E-03	2.12E-02
OPA1-AS1	2.4	9.43E-04	1.74E-02
EDEM2	2.4	4.49E-03	4.80E-02
OAF	2.4	8.17E-04	1.58E-02
LOC102724814	2.4	5.57E-04	1.20E-02
LOC107986714	2.4	1.86E-03	2.73E-02
LOC100287896	2.4	9.73E-04	1.78E-02
LSM3	2.4	8.38E-04	1.60E-02
TMEM9B-AS1	2.4	3.36E-03	3.99E-02
BCL11B	2.4	3.26E-05	1.39E-03
LOC105371963	2.4	4.93E-03	5.07E-02
ASTL	2.4	1.50E-03	2.36E-02
HOPX	2.4	1.62E-03	2.49E-02
SCARNA21	2.4	1.21E-03	2.06E-02
BTBD19	2.4	5.07E-04	1.13E-02
PLAAT4	2.4	6.02E-04	1.27E-02
PRKAG2-AS1	2.4	4.70E-03	4.93E-02
ENTPD2	2.4	1.65E-03	2.51E-02
RNU6-9	2.4	2.94E-03	3.68E-02
LOC101928237	2.4	3.60E-03	4.18E-02
MARS2	2.4	4.38E-04	1.02E-02
LOC105373976	2.4	3.13E-03	3.82E-02
ALOX12-AS1	2.4	5.23E-04	1.15E-02
TMEM19	2.4	1.23E-03	2.09E-02
LINC01972	2.4	5.32E-03	5.30E-02
MLLT6	2.4	1.26E-08	1.43E-06
CTLA4	2.4	1.86E-03	2.74E-02
PVRIG	2.4	1.00E-03	1.82E-02
LOC105370792	2.4	3.74E-03	4.28E-02
MIR6752	2.4	2.07E-03	2.95E-02
NFYC-AS1	2.4	1.09E-03	1.92E-02
IFI30	2.4	1.28E-05	6.50E-04
FCMR	2.4	6.12E-04	1.28E-02
CPT1B	2.4	8.28E-04	1.59E-02
SLC39A6	2.4	3.88E-04	9.37E-03
NTN3	2.4	1.47E-03	2.33E-02
CD3E	2.4	2.24E-03	3.10E-02

SRRD	2.4	6.33E-04	1.31E-02
SLFN5	2.4	3.18E-05	1.36E-03
CD300E	2.4	4.00E-04	9.59E-03
LINC00910	2.4	3.25E-03	3.90E-02
CD248	2.4	4.55E-04	1.05E-02
LOC100291105	2.4	3.82E-04	9.25E-03
SMIM25	2.4	2.37E-04	6.51E-03
LOC112267961	2.4	2.84E-03	3.59E-02
LOC107984247	2.4	8.19E-04	1.58E-02
CLEC2D	2.4	4.72E-04	1.07E-02
TNFRSF4	2.4	7.29E-04	1.45E-02
MPV17	2.4	6.58E-05	2.45E-03
LINC02611	2.3	4.55E-03	4.84E-02
LEF1	2.3	1.84E-04	5.38E-03
P2RX7	2.3	2.17E-04	6.12E-03
LOC112268121	2.3	2.98E-03	3.70E-02
IKZF3	2.3	1.74E-05	8.35E-04
RASGEF1B	2.3	8.85E-05	3.10E-03
BATF3	2.3	4.16E-03	4.56E-02
HACL1	2.3	7.31E-04	1.45E-02
C5	2.3	7.81E-04	1.53E-02
FBXO2	2.3	2.44E-03	3.25E-02
TM7SF2	2.3	1.61E-03	2.48E-02
IL10RB-DT	2.3	4.60E-03	4.87E-02
MIR6766	2.3	3.76E-03	4.29E-02
CAGE1	2.3	5.25E-03	5.26E-02
LOC101927069	2.3	3.71E-03	4.26E-02
ERVK3-1	2.3	2.68E-03	3.46E-02
CD163	2.3	9.45E-04	1.74E-02
LEPROTL1	2.3	2.57E-04	6.91E-03
RASA4B	2.3	1.64E-03	2.50E-02
LINC01136	2.3	2.51E-03	3.31E-02
FAM83H	2.3	2.63E-04	7.03E-03
LOC105373582	2.3	3.78E-03	4.30E-02
LOC107985325	2.3	3.36E-03	3.99E-02
LOC105378539	2.3	3.11E-04	7.98E-03
GPR89B	2.3	2.84E-03	3.59E-02
BCAP29	2.3	4.98E-03	5.10E-02
LOC107985785	2.3	1.86E-03	2.73E-02
EBAG9	2.3	1.76E-03	2.64E-02
SCNM1	2.3	2.33E-03	3.16E-02
SEPTIN1	2.3	4.70E-04	1.07E-02
ARL14EP	2.3	1.70E-03	2.57E-02
LILRA1	2.3	1.28E-04	4.08E-03
CRTAM	2.3	1.11E-03	1.95E-02
DBP	2.3	2.94E-03	3.68E-02
POLR3H	2.3	5.68E-04	1.22E-02

SRFBP1	2.3	2.23E-03	3.09E-02
AGPAT4	2.3	5.77E-05	2.20E-03
ATP2B1-AS1	2.3	2.10E-05	9.74E-04
CD72	2.3	3.01E-03	3.73E-02
TAF12	2.3	9.57E-04	1.75E-02
CCRL2	2.3	1.88E-03	2.76E-02
RPF1	2.3	3.31E-04	8.31E-03
RPL23AP64	2.3	2.48E-03	3.29E-02
CD4	2.3	8.73E-04	1.64E-02
LOC107986630	2.3	4.81E-03	5.01E-02
LINC00926	2.3	2.49E-03	3.30E-02
CD5	2.3	8.32E-04	1.59E-02
WASHC3	2.3	3.04E-04	7.85E-03
LOC105377203	2.3	4.89E-03	5.06E-02
RECK	2.3	2.47E-04	6.73E-03
LOC105371933	2.3	1.56E-03	2.42E-02
JAKMIP1	2.3	3.18E-03	3.85E-02
PLPP6	2.3	4.81E-03	5.01E-02
PDZD4	2.3	2.16E-04	6.10E-03
GNMT	2.3	3.89E-03	4.38E-02
ADGRG1	2.3	5.14E-04	1.14E-02
TNK1	2.3	1.96E-03	2.84E-02
LINC00260	2.3	3.96E-03	4.42E-02
CSF1R	2.3	3.18E-04	8.12E-03
CYP27A1	2.3	4.69E-03	4.92E-02
STIM2	2.3	2.29E-04	6.37E-03
CCR8	2.3	4.04E-03	4.48E-02
IL7R	2.3	1.26E-06	8.96E-05
LOC100505549	2.3	4.43E-03	4.76E-02
MOCS2	2.3	4.57E-03	4.85E-02
PDE1B	2.3	1.21E-03	2.07E-02
ARAP1-AS2	2.3	1.89E-03	2.77E-02
C20orf204	2.3	1.61E-03	2.48E-02
SLC7A6	2.3	2.55E-04	6.87E-03
TGFBI	2.3	3.45E-04	8.61E-03
TSEN54	2.3	6.11E-04	1.28E-02
LOC101928045	2.3	3.68E-03	4.23E-02
TSPAN17	2.3	4.64E-03	4.89E-02
NACA4P	2.3	3.91E-03	4.39E-02
CCDC25	2.3	2.93E-03	3.68E-02
HIF1A-AS3	2.3	3.57E-03	4.16E-02
RPUSD4	2.2	1.21E-03	2.06E-02
PP2D1	2.2	3.86E-03	4.36E-02
MFAP4	2.2	3.22E-03	3.88E-02
SNORD56	2.2	2.30E-03	3.15E-02
TTYH2	2.2	2.78E-04	7.32E-03
PRR22	2.2	1.10E-03	1.94E-02

CYSLTR2	2.2	3.49E-03	4.10E-02
SLFN11	2.2	4.57E-04	1.05E-02
HMGN3	2.2	9.96E-04	1.81E-02
CHIC1	2.2	8.13E-04	1.58E-02
C6orf136	2.2	3.78E-04	9.16E-03
ASAP1-IT2	2.2	8.70E-04	1.64E-02
GDF7	2.2	4.44E-04	1.03E-02
NEK8	2.2	4.85E-04	1.09E-02
MYBL1	2.2	1.40E-03	2.25E-02
GBP5	2.2	3.17E-04	8.09E-03
RASGRP1	2.2	2.33E-03	3.16E-02
DKFZP586I1420	2.2	2.69E-04	7.13E-03
TK2	2.2	3.61E-04	8.87E-03
TAF13	2.2	8.77E-04	1.65E-02
LINC02210	2.2	2.98E-03	3.70E-02
LOC105372295	2.2	3.61E-03	4.18E-02
RPL9	2.2	1.58E-04	4.84E-03
CYB561	2.2	1.57E-03	2.43E-02
CDKN1C	2.2	5.18E-03	5.22E-02
FAAP24	2.2	3.46E-03	4.07E-02
COQ10A	2.2	3.27E-03	3.92E-02
NDUFS7	2.2	9.78E-04	1.78E-02
CTSH	2.2	6.83E-04	1.38E-02
SMAP1	2.2	1.40E-04	4.39E-03
CXXC5	2.2	5.49E-04	1.19E-02
C9orf85	2.2	4.08E-03	4.50E-02
CD300LB	2.2	2.50E-03	3.31E-02
RINL	2.2	3.54E-03	4.14E-02
LTB	2.2	6.63E-05	2.46E-03
LOC105378949	2.2	5.44E-03	5.38E-02
MIF-AS1	2.2	8.83E-04	1.65E-02
TGFBR3	2.2	4.71E-03	4.93E-02
MYRF	2.2	1.80E-04	5.30E-03
APOBEC3D	2.2	3.99E-03	4.44E-02
HPDL	2.2	1.59E-03	2.45E-02
FAM114A2	2.2	2.41E-04	6.60E-03
NSMCE4A	2.2	4.19E-04	9.85E-03
NAIF1	2.2	1.23E-03	2.09E-02
NRIP2	2.2	1.61E-03	2.48E-02
ERAP2	2.2	6.70E-04	1.36E-02
FLYWCH2	2.2	2.57E-03	3.35E-02
LOC105373826	2.2	5.47E-03	5.39E-02
CCZ1	2.2	1.79E-03	2.66E-02
SLAMF6	2.2	1.36E-03	2.22E-02
STX12	2.2	6.61E-04	1.35E-02
SAT2	2.2	2.21E-03	3.07E-02
LOC101927420	2.2	3.64E-03	4.20E-02

LOC107984341	2.2	5.47E-03	5.39E-02
DDB2	2.2	1.31E-03	2.17E-02
EDDM13	2.2	4.16E-03	4.57E-02
SNX4	2.2	4.81E-03	5.01E-02
RPA3	2.2	3.03E-03	3.74E-02
LOC105371763	2.2	3.61E-03	4.18E-02
TMEM102	2.2	1.95E-03	2.83E-02
NDUFA3	2.2	2.53E-03	3.33E-02
CD2	2.2	3.50E-03	4.11E-02
LOC102724646	2.2	5.28E-03	5.28E-02
HLA-DRB1	2.2	1.29E-04	4.11E-03
TSHZ1	2.2	2.46E-04	6.69E-03
CROCC2	2.2	3.47E-04	8.65E-03
HLA-DMA	2.2	5.10E-04	1.13E-02
ZNF235	2.2	4.44E-03	4.76E-02
CTRL	2.2	6.71E-04	1.36E-02
MED6	2.1	1.82E-03	2.70E-02
CXCL2	2.1	3.46E-03	4.07E-02
SLC22A18	2.1	3.17E-03	3.85E-02
FARP2	2.1	1.09E-04	3.65E-03
FAM49A	2.1	6.40E-04	1.31E-02
LOC107984315	2.1	3.89E-03	4.38E-02
RBM44	2.1	3.86E-03	4.36E-02
UFSP1	2.1	1.61E-03	2.47E-02
FBLN7	2.1	6.57E-04	1.34E-02
RPL22	2.1	1.47E-03	2.33E-02
SNIP1	2.1	1.24E-03	2.09E-02
FBXO33	2.1	3.50E-04	8.67E-03
NKRF	2.1	1.34E-03	2.19E-02
LOC107986556	2.1	5.33E-03	5.31E-02
DPP4	2.1	2.85E-03	3.60E-02
RPL31	2.1	1.30E-06	9.17E-05
RORA	2.1	1.24E-04	3.98E-03
PRPF39	2.1	6.01E-04	1.27E-02
HAUS3	2.1	8.81E-04	1.65E-02
ABI3	2.1	4.99E-03	5.10E-02
ARL4C	2.1	2.36E-04	6.51E-03
HLA-DQB1	2.1	1.51E-03	2.37E-02
PHYH	2.1	4.86E-03	5.04E-02
MIER3	2.1	5.88E-04	1.25E-02
EIF1AX-AS1	2.1	5.16E-03	5.21E-02
MMRN2	2.1	2.56E-03	3.35E-02
CHST13	2.1	4.16E-03	4.56E-02
ZNF506	2.1	7.40E-04	1.47E-02
SAMHD1	2.1	8.70E-06	4.68E-04
RUNX3	2.1	3.62E-04	8.88E-03
CAMK4	2.1	7.41E-04	1.47E-02

MAP4K1	2.1	2.73E-03	3.50E-02
CHORDC1	2.1	8.08E-04	1.57E-02
LOC105370525	2.1	3.46E-03	4.07E-02
LRRC14B	2.1	4.33E-03	4.69E-02
ZNF497	2.1	5.34E-04	1.16E-02
KMT5C	2.1	5.22E-04	1.15E-02
UNC50	2.1	1.64E-03	2.50E-02
SMYD4	2.1	3.45E-03	4.07E-02
PRKX	2.1	5.36E-04	1.17E-02
SLC7A7	2.1	1.21E-03	2.06E-02
MIR6822	2.1	5.40E-03	5.36E-02
USP18	2.1	4.16E-03	4.56E-02
PRKD3	2.1	5.69E-05	2.18E-03
GIN1	2.1	4.63E-03	4.88E-02
RNF125	2.1	1.10E-03	1.94E-02
LOC107984847	2.1	1.37E-03	2.22E-02
LOC107985207	2.1	3.20E-04	8.14E-03
LINC00869	2.1	1.49E-03	2.35E-02
ANKAR	2.1	4.84E-03	5.02E-02
SMIM18	2.1	4.33E-03	4.69E-02
LTBP3	2.1	3.54E-04	8.75E-03
ZNF92	2.1	6.36E-04	1.31E-02
USE1	2.1	2.50E-03	3.31E-02
ZDHHC8	2.1	3.68E-04	8.99E-03
SLFN12L	2.1	1.80E-03	2.67E-02
TRAP1	2.1	2.74E-03	3.51E-02
TCF7	2.1	7.99E-07	6.07E-05
PRR5	2.1	4.28E-03	4.65E-02
CCR7	2.1	1.39E-03	2.24E-02
CTDSPL2	2.1	7.10E-04	1.42E-02
DNLZ	2.1	2.84E-03	3.59E-02
NOC4L	2.1	8.22E-04	1.58E-02
LOC100127955	2.1	1.06E-03	1.89E-02
SMDT1	2.1	1.14E-03	1.97E-02
IFT52	2.1	2.92E-03	3.67E-02
FGL2	2.1	1.29E-04	4.10E-03
KMO	2.1	5.14E-03	5.19E-02
ARMT1	2.1	4.40E-03	4.75E-02
PBDC1	2.1	5.01E-03	5.11E-02
LOC646976	2.1	4.67E-04	1.06E-02
DIS3	2.1	8.15E-04	1.58E-02
SUCLG1	2.1	3.72E-03	4.27E-02
SNORA53	2.1	4.77E-03	4.98E-02
SLC16A6	2.1	3.66E-03	4.22E-02
ADAP2	2.1	3.81E-03	4.32E-02
H1-3	2.1	2.98E-03	3.70E-02
PEX1	2.1	2.55E-03	3.34E-02

AATBC	2.1	6.32E-04	1.31E-02
SYS1	2.1	2.02E-03	2.91E-02
TYSND1	2.1	3.55E-03	4.14E-02
CALHM2	2.1	7.27E-04	1.45E-02
S1PR5	2.1	3.15E-03	3.83E-02
TNFSF8	2.1	5.45E-03	5.38E-02
ATRAID	2.1	9.83E-04	1.79E-02
BCL2	2.1	5.99E-04	1.27E-02
BCL7A	2.1	2.29E-03	3.14E-02
LRP1	2.1	9.23E-05	3.20E-03
MPEG1	2.1	6.20E-05	2.33E-03
NEFL	2.1	1.68E-03	2.54E-02
GIMAP8	2.0	9.98E-04	1.81E-02
TRAF1	2.0	9.57E-04	1.75E-02
ECHDC1	2.0	4.55E-03	4.85E-02
LOC107986924	2.0	5.45E-03	5.38E-02
PCED1B-AS1	2.0	4.42E-03	4.76E-02
BATF	2.0	3.80E-03	4.32E-02
RPL21	2.0	7.86E-05	2.82E-03
ADAMTS10	2.0	1.41E-03	2.26E-02
GPR183	2.0	1.68E-03	2.54E-02
POLR2F	2.0	7.69E-04	1.51E-02
CEBPG	2.0	1.63E-03	2.49E-02
IL32	2.0	4.39E-03	4.74E-02
LOC105375945	2.0	3.27E-03	3.92E-02
KCTD17	2.0	3.02E-03	3.73E-02
LOC105376548	2.0	5.24E-03	5.26E-02
LOC105374341	2.0	1.47E-03	2.33E-02
KAT14	2.0	3.34E-03	3.98E-02
TIGD7	2.0	4.85E-03	5.03E-02
MS4A1	2.0	5.23E-03	5.25E-02
SNHG11	2.0	1.64E-03	2.50E-02
S100A4	2.0	2.85E-04	7.46E-03
AGAP2-AS1	2.0	2.52E-03	3.32E-02
SH3YL1	2.0	1.24E-03	2.10E-02
ZFYVE28	2.0	1.02E-03	1.84E-02
LOC105375363	2.0	4.45E-03	4.77E-02
FAAH2	2.0	3.87E-03	4.37E-02
APOL1	2.0	4.96E-04	1.11E-02
ZNF284	2.0	4.38E-03	4.73E-02
MFSD4B	2.0	3.56E-03	4.15E-02
TTF1	2.0	7.72E-04	1.52E-02
RAB20	2.0	2.99E-03	3.71E-02
METTL17	2.0	4.02E-03	4.46E-02
OLIG2	2.0	5.06E-03	5.14E-02
ANAPC1	2.0	2.41E-04	6.60E-03
ARMH2	2.0	4.71E-03	4.93E-02

FLT3LG	2.0	3.95E-03	4.41E-02
CLUHP3	2.0	3.60E-03	4.18E-02
DDX51	2.0	3.03E-04	7.83E-03
PCBD2	2.0	3.59E-03	4.17E-02
LAX1	2.0	2.97E-03	3.70E-02
IFT43	2.0	2.90E-03	3.65E-02
LOC105376875	2.0	5.24E-03	5.26E-02
ATG3	2.0	1.45E-03	2.32E-02
IL10RA	2.0	1.10E-04	3.67E-03
COG2	2.0	2.40E-03	3.22E-02
ST6GALNAC6	2.0	4.54E-03	4.84E-02
ZKSCAN3	2.0	2.90E-03	3.65E-02
DTX2	2.0	2.24E-03	3.10E-02
HLA-DRA	2.0	9.58E-05	3.30E-03
SYTL2	2.0	5.54E-03	5.43E-02
B3GAT1	2.0	2.23E-03	3.09E-02
PSMD6-AS2	2.0	3.52E-03	4.12E-02
TPMT	2.0	1.89E-03	2.76E-02
ZNF763	2.0	3.74E-03	4.28E-02
CD33	2.0	4.84E-03	5.02E-02
SIGLEC10	2.0	3.36E-03	3.99E-02
ISG20	2.0	2.51E-04	6.81E-03
SMPD2	2.0	3.38E-03	4.00E-02
EAPP	2.0	2.35E-03	3.18E-02
TRIM11	2.0	1.73E-04	5.16E-03
LOC105371441	2.0	3.79E-03	4.31E-02
PPP1R16B	2.0	1.31E-03	2.17E-02
DNAJC30	2.0	5.57E-03	5.44E-02
EPHA1	2.0	2.24E-03	3.10E-02
TERF1	2.0	5.47E-03	5.39E-02
LYNX1	2.0	4.56E-03	4.85E-02
TOR3A	2.0	8.48E-04	1.61E-02
SEC31B	2.0	1.13E-03	1.97E-02
ZNF804A	2.0	4.28E-03	4.65E-02
GBP3	2.0	4.38E-03	4.73E-02
CLDND2	2.0	4.13E-03	4.54E-02
PLXNB2	2.0	7.39E-06	4.07E-04
CCDC159	2.0	7.02E-04	1.41E-02
NBPF9	2.0	1.56E-05	7.62E-04
EVL	2.0	1.34E-04	4.21E-03
DSC2	2.0	6.80E-04	1.38E-02
ITGAL	2.0	1.15E-07	1.07E-05
MIEF2	1.9	3.27E-03	3.92E-02
AAK1	1.9	2.39E-06	1.55E-04
UNC93B1	1.9	5.49E-04	1.19E-02
PICK1	1.9	1.45E-03	2.31E-02
RABEP2	1.9	4.00E-03	4.45E-02

FAM53B	1.9	3.26E-04	8.25E-03
TEX264	1.9	5.30E-04	1.16E-02
HMGN3-AS1	1.9	5.53E-03	5.42E-02
TMEM243	1.9	3.41E-03	4.03E-02
ZBTB25	1.9	1.37E-03	2.23E-02
CRIP1	1.9	2.41E-03	3.23E-02
VPS9D1-AS1	1.9	5.37E-03	5.33E-02
NMUR1	1.9	5.54E-04	1.19E-02
PIGL	1.9	2.15E-03	3.02E-02
ISG20L2	1.9	1.22E-03	2.08E-02
GOLGA8B	1.9	4.93E-03	5.08E-02
GVINP1	1.9	2.18E-03	3.05E-02
TRPV2	1.9	1.48E-03	2.34E-02
MRPL9	1.9	3.34E-03	3.97E-02
CLUAP1	1.9	3.34E-03	3.98E-02
BIRC3	1.9	6.18E-04	1.29E-02
CD300A	1.9	2.31E-04	6.39E-03
OAS2	1.9	2.27E-03	3.12E-02
CCDC69	1.9	3.60E-04	8.85E-03
ITK	1.9	4.94E-03	5.08E-02
UAP1L1	1.9	2.67E-03	3.45E-02
CEP85L	1.9	1.30E-03	2.16E-02
ZNF101	1.9	3.64E-03	4.21E-02
ATP7A	1.9	3.88E-03	4.38E-02
NSA2	1.9	3.13E-03	3.82E-02
MARCHF1	1.9	3.85E-03	4.36E-02
GCH1	1.9	3.90E-03	4.38E-02
ADA2	1.9	1.14E-03	1.98E-02
MDFIC	1.9	2.30E-03	3.14E-02
TRMT5	1.9	1.28E-03	2.14E-02
APOBEC3G	1.9	1.24E-03	2.09E-02
SEPTIN7P2	1.9	3.19E-03	3.86E-02
LRRC14	1.9	8.82E-04	1.65E-02
SEMA4C	1.9	1.98E-03	2.86E-02
CD83	1.9	4.08E-03	4.50E-02
LILRB1	1.9	1.89E-03	2.77E-02
GPR3	1.9	2.15E-03	3.02E-02
ABHD3	1.9	4.42E-03	4.76E-02
ANKRD13C	1.9	1.70E-03	2.57E-02
NAXD	1.9	2.74E-03	3.51E-02
SH3TC1	1.9	3.98E-04	9.56E-03
RPL13	1.9	1.17E-05	6.00E-04
MTO1	1.9	3.24E-03	3.90E-02
RPS21	1.9	2.14E-03	3.02E-02
CD69	1.9	1.71E-03	2.58E-02
TMEM184C	1.9	2.31E-03	3.15E-02
APOL6	1.9	6.78E-05	2.51E-03

JUN	1.9	1.93E-07	1.68E-05
ZNF277	1.9	1.67E-03	2.53E-02
PSMB10	1.9	1.38E-03	2.24E-02
SIGLEC1	1.9	5.32E-03	5.30E-02
IRF5	1.9	4.92E-03	5.07E-02
RFTN1	1.9	1.25E-03	2.10E-02
SOCS4	1.9	1.36E-03	2.21E-02
TTC38	1.9	4.10E-03	4.51E-02
ARHGEF10L	1.9	5.60E-03	5.46E-02
PKD1P1	1.9	3.59E-03	4.17E-02
LRRC25	1.9	1.22E-03	2.07E-02
TAF4	1.9	1.30E-03	2.16E-02
ORMDL1	1.9	5.96E-04	1.26E-02
CIITA	1.9	1.20E-04	3.91E-03
TFCP2	1.9	2.12E-03	3.00E-02
PSME1	1.9	5.86E-06	3.32E-04
CPVL	1.9	3.86E-03	4.36E-02
SPAG7	1.9	3.89E-03	4.38E-02
SGK1	1.8	4.02E-03	4.46E-02
LOC100289511	1.8	4.97E-03	5.09E-02
QRSL1	1.8	2.42E-03	3.24E-02
UFL1	1.8	1.25E-03	2.10E-02
TUT1	1.8	5.56E-03	5.44E-02
SLC66A3	1.8	3.76E-03	4.29E-02
GTF3A	1.8	1.63E-03	2.49E-02
ZAP70	1.8	1.26E-03	2.11E-02
RPL18	1.8	6.21E-04	1.29E-02
DNAJB13	1.8	4.60E-03	4.87E-02
ASB6	1.8	4.04E-03	4.48E-02
BICRA	1.8	2.53E-03	3.33E-02
PDP1	1.8	1.28E-03	2.13E-02
EIF3K	1.8	4.00E-03	4.45E-02
LINC00877	1.8	3.82E-03	4.33E-02
NAB2	1.8	4.58E-03	4.86E-02
SLFN12	1.8	5.60E-03	5.46E-02
THAP9-AS1	1.8	2.74E-03	3.50E-02
TGIF2	1.8	2.53E-03	3.33E-02
NOD2	1.8	3.17E-03	3.85E-02
IGSF6	1.8	4.19E-04	9.85E-03
LY9	1.8	4.56E-03	4.85E-02
NYAP1	1.8	3.01E-03	3.73E-02
GBP4	1.8	9.02E-04	1.68E-02
TMEM205	1.8	3.72E-03	4.27E-02
TTC22	1.8	5.02E-03	5.12E-02
PDE7A	1.8	3.73E-04	9.06E-03
EVI2B	1.8	1.07E-03	1.90E-02
TMEM234	1.8	2.23E-03	3.10E-02

BICDL1	1.8	3.90E-03	4.38E-02
WDR97	1.8	1.93E-03	2.80E-02
PREP	1.8	2.09E-03	2.97E-02
MAP3K14	1.8	5.91E-04	1.26E-02
DECR1	1.8	1.80E-03	2.68E-02
VMP1	1.8	1.02E-03	1.83E-02
UXT	1.8	1.50E-03	2.36E-02
EED	1.8	3.17E-03	3.85E-02
EMILIN2	1.8	6.98E-04	1.41E-02
PRDM1	1.8	1.65E-03	2.51E-02
WDR25	1.8	3.71E-03	4.26E-02
SLC12A7	1.8	5.40E-03	5.36E-02
GOLGA2P5	1.8	4.00E-03	4.45E-02
SEC24B	1.8	7.45E-04	1.47E-02
UCK1	1.8	4.10E-03	4.52E-02
CYHR1	1.8	2.95E-03	3.68E-02
CTSS	1.8	6.88E-05	2.54E-03
PCED1A	1.8	3.56E-03	4.15E-02
PPIF	1.8	9.01E-04	1.68E-02
TRAF5	1.8	2.05E-03	2.93E-02
BCL2L11	1.8	1.63E-03	2.50E-02
EXOSC2	1.8	2.69E-03	3.46E-02
SECTM1	1.8	3.41E-03	4.03E-02
UQCRC2	1.8	1.37E-03	2.22E-02
JAZF1	1.8	3.77E-03	4.30E-02
PCNX2	1.8	3.68E-03	4.23E-02
LOC105369536	1.8	4.03E-03	4.47E-02
RGS12	1.8	1.10E-03	1.94E-02
RPL5	1.8	3.84E-05	1.60E-03
IL2RG	1.7	1.40E-03	2.25E-02
ZNF107	1.7	5.33E-03	5.31E-02
CHMP4A	1.7	4.65E-03	4.89E-02
IRF8	1.7	1.81E-03	2.69E-02
OTUD1	1.7	1.38E-03	2.23E-02
SLC4A7	1.7	5.59E-03	5.45E-02
ZNF330	1.7	4.55E-03	4.85E-02
MALT1	1.7	1.83E-03	2.71E-02
GORASP2	1.7	5.44E-03	5.38E-02
LOC112268267	1.7	4.44E-03	4.76E-02
PILRA	1.7	3.33E-03	3.97E-02
GSDMB	1.7	3.36E-03	3.99E-02
UBE2D4	1.7	5.03E-03	5.12E-02
EGR1	1.7	6.13E-05	2.31E-03
INPP4A	1.7	1.02E-03	1.84E-02
SLC27A1	1.7	3.93E-03	4.40E-02
VAMP2	1.7	7.86E-04	1.54E-02
IPMK	1.7	1.31E-03	2.17E-02

CD53	1.7	1.56E-03	2.42E-02
DDX28	1.7	5.52E-03	5.42E-02
ANKS3	1.7	3.21E-03	3.88E-02
ARHGEF40	1.7	2.06E-03	2.94E-02
DUSP10	1.7	3.26E-04	8.25E-03
TRIB2	1.7	9.40E-04	1.74E-02
TXNDC12	1.7	4.90E-03	5.06E-02
CST7	1.7	2.20E-03	3.07E-02
CASP8	1.7	4.06E-04	9.66E-03
NSMCE1	1.7	1.11E-03	1.95E-02
SPOCK2	1.7	8.46E-04	1.61E-02
ICAM1	1.7	1.98E-03	2.85E-02
ABHD17A	1.7	2.80E-04	7.35E-03
UFC1	1.7	2.27E-03	3.12E-02
MFHAS1	1.7	4.81E-03	5.01E-02
DGKA	1.7	1.17E-04	3.83E-03
CD96	1.7	4.46E-03	4.78E-02
CHST15	1.7	5.41E-04	1.18E-02
DOCK10	1.7	1.28E-03	2.13E-02
SNX27	1.7	1.99E-03	2.87E-02
CYCS	1.7	3.29E-03	3.93E-02
CLEC7A	1.7	1.83E-03	2.71E-02
LMTK3	1.7	3.80E-03	4.32E-02
NOP9	1.7	9.60E-04	1.76E-02
PRKCH	1.7	4.11E-03	4.52E-02
MRPL16	1.7	4.62E-03	4.88E-02
AIP	1.7	2.40E-03	3.23E-02
CXCR2	1.7	3.20E-04	8.13E-03
RPS6	1.7	5.42E-05	2.09E-03
VOPP1	1.7	9.41E-04	1.74E-02
TECPR1	1.7	1.17E-03	2.01E-02
MED10	1.7	2.05E-03	2.94E-02
DCAF15	1.7	3.32E-03	3.96E-02
TNFSF13B	1.7	4.58E-03	4.85E-02
NOP53	1.7	1.53E-03	2.39E-02
WARS1	1.7	2.76E-05	1.22E-03
ETS1	1.7	3.22E-06	2.00E-04
WDR81	1.7	4.51E-04	1.04E-02
PSMB9	1.7	9.80E-04	1.78E-02
KIAA1143	1.7	4.87E-03	5.04E-02
FAM49B	1.7	2.79E-03	3.55E-02
SLC2A6	1.7	3.54E-03	4.14E-02
NLRC3	1.7	4.82E-04	1.09E-02
FLYWCH1	1.7	3.94E-03	4.41E-02
CSTF2T	1.7	1.86E-03	2.73E-02
RPS15A	1.7	2.75E-03	3.52E-02
LPIN1	1.7	3.83E-03	4.34E-02

FOS	1.7	2.23E-06	1.45E-04
GNG2	1.7	2.54E-04	6.85E-03
RPS19	1.7	1.62E-04	4.93E-03
HMGCR	1.7	1.35E-03	2.20E-02
DENND2D	1.7	2.23E-03	3.09E-02
MCMBP	1.7	5.30E-03	5.29E-02
PIAS2	1.7	5.14E-03	5.19E-02
NAA16	1.7	5.43E-03	5.38E-02
RPL32	1.7	8.90E-04	1.66E-02
TRIM26	1.7	1.68E-03	2.54E-02
JADE2	1.7	1.61E-03	2.48E-02
ZNF609	1.7	1.13E-04	3.74E-03
PPT1	1.7	1.31E-03	2.17E-02
SELPLG	1.7	1.39E-03	2.25E-02
ECHDC2	1.7	1.34E-03	2.19E-02
RASA2	1.7	1.08E-03	1.92E-02
ZNF777	1.7	2.13E-03	3.00E-02
RPS17	1.6	4.18E-03	4.57E-02
JAML	1.6	2.45E-04	6.68E-03
MAP3K12	1.6	4.44E-03	4.76E-02
LGALS9	1.6	3.56E-04	8.79E-03
TRMT44	1.6	5.25E-03	5.26E-02
RNMT	1.6	2.58E-03	3.36E-02
LPXN	1.6	1.55E-03	2.41E-02
CD74	1.6	2.26E-05	1.04E-03
CTSC	1.6	3.16E-03	3.83E-02
SYNRG	1.6	1.44E-03	2.31E-02
IFNAR2	1.6	5.54E-03	5.43E-02
TRMT2B	1.6	5.51E-03	5.41E-02
ABCA2	1.6	1.07E-03	1.90E-02
SNX20	1.6	1.64E-03	2.50E-02
CEMIP2	1.6	4.25E-03	4.63E-02
ATM	1.6	3.08E-04	7.92E-03
DPM1	1.6	4.95E-03	5.09E-02
RPS6KA4	1.6	4.90E-03	5.06E-02
MAP2K7	1.6	7.57E-04	1.50E-02
NFATC3	1.6	2.39E-03	3.21E-02
RPS23	1.6	1.48E-03	2.33E-02
PITPNA	1.6	1.34E-03	2.20E-02
SIDT2	1.6	2.18E-03	3.05E-02
CNBP	1.6	1.51E-03	2.37E-02
GABPB1-IT1	1.6	3.23E-03	3.89E-02
MEFV	1.6	1.97E-03	2.85E-02
IFNGR2	1.6	1.60E-03	2.47E-02
PTPRC	1.6	1.97E-06	1.30E-04
LMBR1L	1.6	4.45E-03	4.77E-02
SLC43A2	1.6	4.91E-03	5.07E-02

FAM126B	1.6	1.63E-03	2.49E-02
TYROBP	1.6	3.84E-04	9.28E-03
EFR3A	1.6	2.28E-03	3.14E-02
GMEB1	1.6	1.85E-03	2.73E-02
MYO1G	1.6	2.73E-05	1.21E-03
MIAT	1.6	5.03E-03	5.12E-02
PRKAG2	1.6	5.49E-03	5.41E-02
TES	1.6	4.26E-03	4.63E-02
RPL7	1.6	6.66E-04	1.36E-02
STAT2	1.6	1.80E-03	2.68E-02
RPL3	1.6	8.97E-06	4.81E-04
MIA3	1.6	3.07E-03	3.78E-02
RPS20	1.6	3.72E-04	9.06E-03
DDX56	1.6	4.16E-03	4.56E-02
ANKRD44	1.6	2.30E-04	6.37E-03
RPS12	1.6	3.06E-03	3.77E-02
TRANK1	1.5	8.41E-06	4.55E-04
RAPGEF1	1.5	1.23E-03	2.09E-02
RPS27A	1.5	8.60E-04	1.63E-02
SARAF	1.5	4.75E-05	1.87E-03
CUL4A	1.5	3.21E-03	3.88E-02
CAMK2D	1.5	5.27E-03	5.28E-02
RPS3A	1.5	1.60E-04	4.90E-03
CAMLG	1.5	3.00E-03	3.72E-02
SPG7	1.5	2.32E-03	3.16E-02
PPTC7	1.5	5.19E-03	5.23E-02
PEX11B	1.5	3.50E-03	4.10E-02
FCHO1	1.5	3.88E-03	4.37E-02
PARP8	1.5	2.33E-03	3.16E-02
DUSP1	1.5	7.27E-05	2.65E-03
BTN3A3	1.5	4.06E-03	4.49E-02
TTC7A	1.5	8.40E-04	1.60E-02
APOL2	1.5	2.13E-03	3.01E-02
CASP1	1.5	4.66E-03	4.90E-02
SETD1A	1.5	2.54E-03	3.34E-02
HIVEP2	1.5	7.91E-04	1.54E-02
XRN2	1.5	1.59E-03	2.45E-02
CD44	1.5	4.76E-04	1.08E-02
OXNAD1	1.5	5.03E-03	5.12E-02
CMTR1	1.5	4.69E-03	4.92E-02
BRAT1	1.5	5.45E-03	5.38E-02
SNX18	1.5	5.37E-03	5.33E-02
BANP	1.5	4.79E-03	4.99E-02
EPHA4	1.5	5.35E-03	5.32E-02
SNORD141A	1.5	2.39E-03	3.21E-02
SERPINA1	1.5	2.43E-03	3.25E-02
TMEM154	1.5	3.29E-03	3.93E-02

LINC-PINT	1.5	1.25E-03	2.10E-02
RBM3	1.5	1.52E-03	2.39E-02
SMCHD1	1.5	3.14E-05	1.35E-03
HNRNPDL	1.5	1.47E-03	2.33E-02
CPPED1	1.5	1.48E-03	2.34E-02
AKNA	1.5	1.07E-04	3.58E-03
TAF1	1.5	1.76E-03	2.64E-02
ZHX2	1.5	2.57E-03	3.36E-02
PIM2	1.5	1.43E-03	2.29E-02
TOB2	1.5	2.81E-03	3.56E-02
FFAR2	1.5	4.21E-03	4.60E-02
ERP29	1.5	3.97E-03	4.43E-02
RNF130	1.5	3.98E-04	9.56E-03
PGGHG	1.5	7.08E-04	1.42E-02
MAP3K1	1.5	6.06E-04	1.27E-02
FNBP1	1.5	1.85E-05	8.75E-04
DENND4B	1.5	2.11E-03	2.99E-02
TNFAIP3	1.5	3.65E-03	4.22E-02
LITAF	1.5	2.52E-03	3.32E-02
SULF2	1.5	1.13E-03	1.97E-02
SERINC5	1.5	2.05E-03	2.93E-02
FBXL5	1.4	2.14E-03	3.02E-02
SELL	1.4	1.05E-03	1.88E-02
SRP54	1.4	5.29E-03	5.29E-02
RPS4X	1.4	7.33E-04	1.46E-02
TMC8	1.4	5.47E-03	5.39E-02
MDM4	1.4	5.92E-04	1.26E-02
GAA	1.4	4.49E-03	4.80E-02
RAB11FIP4	1.4	4.65E-03	4.89E-02
HCK	1.4	1.65E-03	2.51E-02
SLC38A1	1.4	8.20E-04	1.58E-02
ZFP36	1.4	9.71E-04	1.77E-02
DMXL2	1.4	4.49E-03	4.80E-02
MX2	1.4	3.06E-03	3.76E-02
FAM120AOS	1.4	1.57E-03	2.43E-02
ATP2B1	1.4	2.51E-03	3.32E-02
AHNAK	1.4	1.40E-06	9.72E-05
CYTH1	1.4	2.11E-04	6.01E-03
RNF167	1.4	1.35E-03	2.20E-02
RPS8	1.4	3.81E-03	4.32E-02
RNASET2	1.4	2.55E-04	6.87E-03
MBTPS1	1.4	4.21E-03	4.60E-02
SPATA13	1.4	2.93E-03	3.68E-02
GNS	1.4	2.86E-04	7.48E-03
DDX60L	1.4	3.77E-03	4.30E-02
PIK3AP1	1.4	1.42E-03	2.27E-02
RARA	1.4	8.09E-04	1.57E-02

GGNBP2	1.4	5.07E-03	5.14E-02
TRIM44	1.4	3.57E-03	4.16E-02
IER2	1.4	2.08E-04	5.93E-03
NBPF26	1.4	1.64E-03	2.50E-02
RPL19	1.4	3.28E-03	3.93E-02
TNFRSF1B	1.4	1.03E-03	1.85E-02
STAT5A	1.4	4.61E-03	4.87E-02
STK17B	1.4	1.80E-04	5.30E-03
IFITM2	1.4	2.98E-03	3.70E-02
ARHGEF11	1.4	2.17E-03	3.04E-02
ATP2B4	1.4	4.26E-04	1.00E-02
G0S2	1.4	7.07E-04	1.42E-02
RNF149	1.4	1.39E-03	2.25E-02
ARHGEF1	1.4	5.59E-04	1.20E-02
NACA	1.4	3.46E-03	4.07E-02
RPL6	1.4	3.50E-03	4.11E-02
RXRA	1.4	3.03E-03	3.74E-02
XPC	1.4	4.17E-03	4.57E-02
ANXA6	1.4	1.68E-03	2.54E-02
FCGR3B	1.4	5.57E-03	5.44E-02
ODF3B	1.4	5.63E-03	5.49E-02
STK10	1.4	8.61E-04	1.63E-02
TRIM14	1.3	2.50E-03	3.31E-02
MOB3A	1.3	2.37E-04	6.51E-03
IQGAP1	1.3	3.30E-05	1.41E-03
SYNE2	1.3	1.40E-04	4.37E-03
ARIH2	1.3	4.16E-03	4.56E-02
LIMK2	1.3	3.92E-03	4.40E-02
SIPA1L1	1.3	3.22E-03	3.88E-02
EIF2S3	1.3	5.18E-03	5.22E-02
NECAP2	1.3	4.19E-03	4.58E-02
RER1	1.3	4.28E-03	4.65E-02
VPS52	1.3	4.07E-03	4.50E-02
RPL13A	1.3	1.13E-04	3.73E-03
EEF1A1	1.3	2.01E-05	9.41E-04
MIR5047	1.3	4.41E-03	4.75E-02
DPYSL2	1.3	1.26E-03	2.12E-02
ARAP2	1.3	4.91E-03	5.07E-02
IKZF1	1.3	2.81E-03	3.56E-02
PSMB8	1.3	2.94E-03	3.68E-02
OGFRL1	1.3	3.13E-03	3.82E-02
GDI2	1.3	5.25E-03	5.26E-02
SP110	1.3	4.95E-03	5.09E-02
ZNF652	1.3	5.42E-04	1.18E-02
STAT1	1.3	1.76E-04	5.24E-03
SART1	1.3	3.75E-03	4.29E-02
AGPAT3	1.3	3.03E-03	3.74E-02

ZNF217	1.3	2.16E-03	3.03E-02
CDC42SE1	1.3	1.78E-03	2.66E-02
VPS13C	1.3	2.17E-04	6.12E-03
RNF19B	1.3	1.74E-03	2.62E-02
SLC25A28	1.3	2.44E-03	3.25E-02
RBMXL1	1.3	1.89E-03	2.76E-02
XIST	1.3	6.44E-04	1.32E-02
ITGB2	1.3	3.25E-04	8.23E-03
ETNK1	1.3	3.65E-03	4.22E-02
CCNL1	1.3	4.77E-03	4.98E-02
NCKAP1L	1.3	2.08E-03	2.96E-02
LCP1	1.3	2.28E-04	6.32E-03
SDCBP	1.3	5.07E-03	5.14E-02
TNFAIP2	1.3	2.21E-03	3.07E-02
SOCS3	1.3	5.55E-03	5.44E-02
RNF213	1.3	1.83E-05	8.69E-04
PIEZO1	1.3	4.75E-04	1.08E-02
SHKBP1	1.3	8.70E-04	1.64E-02
TLE5	1.3	2.69E-03	3.46E-02
SF3B1	1.3	4.09E-04	9.72E-03
RASSF3	1.2	1.85E-03	2.73E-02
RPLP2	1.2	2.75E-03	3.51E-02
HLA-F	1.2	1.83E-03	2.71E-02
SRSF5	1.2	2.60E-04	6.97E-03
NAMPT	1.2	2.57E-03	3.35E-02
PNISR	1.2	3.70E-03	4.26E-02
PSAP	1.2	5.84E-07	4.63E-05
INPP5D	1.2	1.45E-03	2.32E-02
SRRM1	1.2	3.42E-04	8.55E-03
LSP1	1.2	1.84E-03	2.72E-02
RESF1	1.2	3.67E-03	4.23E-02
SMG1	1.2	8.73E-04	1.64E-02
OGT	1.2	1.73E-03	2.61E-02
LAPTM5	1.2	7.11E-06	3.93E-04
PLCB2	1.2	1.27E-03	2.12E-02
CNOT6L	1.2	2.62E-03	3.40E-02
IKBKB	1.2	4.53E-03	4.83E-02
CYTH4	1.2	5.43E-03	5.38E-02
ARHGAP30	1.2	8.22E-04	1.58E-02
ATRX	1.2	1.20E-03	2.05E-02
EEF1G	1.2	3.01E-03	3.72E-02
FAM193A	1.2	2.90E-03	3.65E-02
ZDHHC18	1.2	2.67E-03	3.45E-02
EFHD2	1.2	3.59E-04	8.84E-03
MCL1	1.2	2.64E-04	7.04E-03
TCF25	1.2	9.90E-04	1.80E-02
NIBAN1	1.2	5.47E-03	5.39E-02

ARGLU1	1.2	3.92E-03	4.40E-02
DNAJB1	1.2	5.32E-03	5.30E-02
RBM33	1.2	4.89E-04	1.10E-02
GLG1	1.2	7.89E-04	1.54E-02
CDV3	1.2	3.99E-03	4.44E-02
PHF12	1.2	5.24E-03	5.26E-02
TPT1	1.1	5.91E-04	1.26E-02
EIF3A	1.1	3.06E-03	3.76E-02
NOTCH2	1.1	1.15E-03	1.99E-02
CRLF3	1.1	4.90E-03	5.06E-02
AOAH	1.1	3.21E-03	3.88E-02
RIN3	1.1	5.23E-03	5.25E-02
NFKBIA	1.1	3.86E-03	4.36E-02
XPO6	1.1	3.99E-03	4.44E-02
EEF1D	1.1	3.48E-04	8.65E-03
SAT1	1.1	1.14E-03	1.98E-02
PBXIP1	1.1	8.11E-04	1.57E-02
RBL2	1.1	5.22E-03	5.25E-02
YLPM1	1.1	6.71E-04	1.36E-02
LYN	1.1	1.84E-03	2.72E-02
RPL4	1.1	2.30E-04	6.37E-03
TAPBP	1.1	6.96E-04	1.40E-02
KLF6	1.1	1.70E-03	2.56E-02
DTX3L	1.1	5.46E-03	5.39E-02
SP3	1.1	4.70E-03	4.93E-02
NSD3	1.1	2.44E-03	3.25E-02
FCN1	1.1	2.88E-03	3.63E-02
EHBP1L1	1.1	9.29E-05	3.22E-03
KAT7	1.1	5.50E-03	5.41E-02
KDM2A	1.1	3.60E-03	4.18E-02
RIPOR2	1.1	5.90E-04	1.26E-02
DDX5	1.1	3.30E-04	8.31E-03
LRRK2	1.1	4.24E-03	4.62E-02
IRF1	1.1	7.91E-04	1.54E-02
RPL15	1.0	4.92E-03	5.07E-02
TUBA1A	1.0	3.54E-03	4.14E-02
SEPTIN9	1.0	2.43E-03	3.24E-02
RAC2	1.0	4.60E-03	4.87E-02
TXNIP	1.0	2.12E-03	3.00E-02
ADAR	1.0	4.87E-03	5.05E-02
JMJD1C	1.0	5.00E-03	5.11E-02
CSF3R	1.0	2.67E-03	3.44E-02
IQSEC1	1.0	5.38E-03	5.34E-02
DOCK2	1.0	4.30E-03	4.66E-02
EEF2	1.0	5.90E-04	1.26E-02
DDX3X	1.0	5.59E-03	5.46E-02
HNRNPC	1.0	2.96E-03	3.70E-02

EIF4A2	1.0	2.94E-03	3.68E-02
SORL1	1.0	3.08E-03	3.78E-02
DDX17	1.0	3.08E-03	3.78E-02
VSIR	1.0	2.18E-03	3.04E-02

pathway	SYMBOL	pval	padj	NES
HALLMARK_EPITHELIAL_MESENCHYMAL_TRANSITION	COL3A1|C	1.00E-10	9.07E-09	-2.308
HALLMARK_APICAL_JUNCTION	ACTN1|CL	1.53E-10	1.35E-08	-1.875
HALLMARK_COAGULATION	F2|PROC|	3.18E-09	2.06E-07	-1.956
HALLMARK_MYOGENESIS	ACTA1|TN	8.27E-09	4.79E-07	-1.821
HALLMARK_G2M_CHECKPOINT	AURKA|CC	1.49E-06	4.67E-05	-1.702
HALLMARK_MITOTIC_SPINDLE	ARHGEF2|	2.16E-06	6.40E-05	-1.670
HALLMARK_ESTROGEN_RESPONSE_EARLY	GREB1|CA	4.77E-05	8.62E-04	-1.571
HALLMARK_ANGIOGENESIS	VCAN|POS	6.60E-05	1.13E-03	-1.890
HALLMARK_ESTROGEN_RESPONSE_LATE	TFF1|SLC9	1.12E-04	1.74E-03	-1.572

KEGG_ECM_RECEPTOR_INTERACTION	GP1BA|CO	1.00E-10	9.07E-09	-2.242
KEGG_FOCAL_ADHESION	JUN|ELK1|	1.00E-10	9.07E-09	-2.092
KEGG_REGULATION_OF_ACTIN_CYTOSKELETON	BRK1|FN1	1.00E-10	9.07E-09	-1.946
KEGG_GAP_JUNCTION	PLCB2|GR	3.03E-10	2.49E-08	-2.106
KEGG_ENDOCYTOSIS	F2R|EPN3	7.63E-06	1.86E-04	-1.654
KEGG_BLADDER_CANCER	HRAS|E2F	2.89E-05	5.68E-04	-1.916

REACTOME_DEVELOPMENTAL_BIOLOGY	CYP51A1|	1.00E-10	9.07E-09	-1.497
REACTOME_ECM_PROTEOGLYCANS	ITGA2B|D	1.00E-10	9.07E-09	-2.270
REACTOME_EXTRACELLULAR_MATRIX_ORGANIZATION	ITGAL|ITG	1.00E-10	9.07E-09	-2.115
REACTOME_L1CAM_INTERACTIONS	ITGA2B|A	1.00E-10	9.07E-09	-2.075
REACTOME_NON_INTEGRIN_MEMBRANE_ECM_INTERACTIONS	TNC|LAM	1.00E-10	9.07E-09	-2.386
REACTOME_PLATELET_ACTIVATION_SIGNALING_AND_AGGRE	LAMP2|IT	1.00E-10	9.07E-09	-2.271
REACTOME_PLATELET_AGGREGATION_PLUG_FORMATION	ITGA2B|B	1.00E-10	9.07E-09	-2.418
REACTOME_RHO_GTPASE_EFFECTORS	CFTR|MAD	1.00E-10	9.07E-09	-1.802
REACTOME_SIGNALING_BY_RECEPTOR_TYROSINE_KINASES	POLR2J|IT	1.00E-10	9.07E-09	-1.679
REACTOME_SIGNALING_BY_RHO_GTPASES_MIRO_GTPASES_A	CFTR|MAD	1.00E-10	9.07E-09	-1.598

GOBP_ACTIN_FILAMENT_BASED_PROCESS	ABI1|KCN	1.00E-10	9.07E-09	-1.723
GOBP_ACTOMYOSIN_STRUCTURE_ORGANIZATION	PDCD6IP|	1.00E-10	9.07E-09	-1.979
GOBP_AMEBOIDAL_TYPE_CELL_MIGRATION	CDH2|AKT	1.00E-10	9.07E-09	-1.764
GOBP_ANATOMICAL_STRUCTURE_FORMATION_INVOLVED_IN	ADA|AKT3	1.00E-10	9.07E-09	-1.596
GOBP_ANIMAL_ORGAN_MORPHOGENESIS	CDH2|AKT	1.00E-10	9.07E-09	-1.618
GOBP_BIOLOGICAL_ADHESION	ADA|CDH2	1.00E-10	9.07E-09	-1.633
GOBP_CELL_CELL_SIGNALING	CDH2|ZBT	1.00E-10	9.07E-09	-1.448
GOBP_CELL_JUNCTION_ORGANIZATION	CDH2|CDH	1.00E-10	9.07E-09	-1.784
GOBP_CELL_MIGRATION	ADA|CDH2	1.00E-10	9.07E-09	-1.650
GOBP_CELL_MORPHOGENESIS	CDH2|GJE	1.00E-10	9.07E-09	-1.648
GOBP_CELL_POPULATION_PROLIFERATION	ADA|CDH2	1.00E-10	9.07E-09	-1.365
GOBP_CELL_SUBSTRATE_ADHESION	MIR939|B	1.00E-10	9.07E-09	-1.905
GOBP_CELL_SUBSTRATE_JUNCTION_ORGANIZATION	CORO2B|D	1.00E-10	9.07E-09	-2.248
GOBP_CIRCULATORY_SYSTEM_DEVELOPMENT	CDH2|AKT	1.00E-10	9.07E-09	-1.545
GOBP_CIRCULATORY_SYSTEM_PROCESS	ADA|KCN	1.00E-10	9.07E-09	-1.694
GOBP_CYTOSKELETON_ORGANIZATION	ABI1|HDA	1.00E-10	9.07E-09	-1.579
GOBP_ENZYME_LINKED_RECEPTOR_PROTEIN_SIGNALING_PAT	ABI1|CDH	1.00E-10	9.07E-09	-1.668
GOBP_EPITHELIUM_DEVELOPMENT	CDH2|CDH	1.00E-10	9.07E-09	-1.532

GOBP_HOMOTYPIC_CELL_CELL_ADHESION	SH2B3|PTF	1.00E-10	9.07E-09	-2.287
GOBP_LOCOMOTION	ADA|CDH2	1.00E-10	9.07E-09	-1.581
GOBP_PLATELET_ACTIVATION	SH2B3|TSF	1.00E-10	9.07E-09	-2.349
GOBP_PLATELET_DEGRANULATION	A1BG|LHFI	1.00E-10	9.07E-09	-2.428
GOBP_REGULATION_OF_ANATOMICAL_STRUCTURE_MORPHO	ADA|CDH2	1.00E-10	9.07E-09	-1.558
GOBP_REGULATION_OF_TRANSPORT	ADA|CDH2	1.00E-10	9.07E-09	-1.424
GOBP_RESPONSE_TO_ENDOGENOUS_STIMULUS	NR2E3|SRA	1.00E-10	9.07E-09	-1.550
GOBP_RESPONSE_TO_GROWTH_FACTOR	HDAC6|PD	1.00E-10	9.07E-09	-1.721
GOBP_RESPONSE_TO_OXYGEN_CONTAINING_COMPOUND	ADA|MIR6	1.00E-10	9.07E-09	-1.419
GOBP_SECRETION	A1BG|ADA	1.00E-10	9.07E-09	-1.529
GOBP_TISSUE_MORPHOGENESIS	APELA|GP	1.00E-10	9.07E-09	-1.595
GOBP_WOUND_HEALING	CDH3|MIR	1.00E-10	9.07E-09	-2.132
GOBP_LAMELLIPODIUM_ORGANIZATION	OCLN|ARP	1.26E-10	1.12E-08	-2.125
GOBP_MULTICELLULAR_ORGANISMAL_HOMEOSTASIS	AKT3|CDH	2.82E-10	2.34E-08	-1.610
GOBP_NEGATIVE_REGULATION_OF_DEVELOPMENTAL_PROCES	AKT3|MIR	3.49E-10	2.81E-08	-1.487
GOBP_POSITIVE_REGULATION_OF_DEVELOPMENTAL_PROCESS	ADA|CDH2	6.61E-10	4.95E-08	-1.400
GOBP_INTEGRIN_MEDIATED_SIGNALING_PATHWAY	TSPAN32|	5.93E-09	3.56E-07	-2.014
GOBP_REGULATION_OF_CELL_DIFFERENTIATION	ADA|CDH2	7.04E-09	4.12E-07	-1.342
GOBP_REGULATION_OF_PROTEIN_PHOSPHORYLATION	ABI1|HDA	1.83E-08	9.72E-07	-1.374
GOBP_SMALL_GTPASE_MEDIATED_SIGNAL_TRANSDUCTION	RASA4B|SH	2.95E-08	1.48E-06	-1.523
GOBP_HOMOPHILIC_CELL_ADHESION_VIA_PLASMA_MEMBRA	CDH2|CDH	3.46E-08	1.71E-06	-1.860
GOBP_NEGATIVE_REGULATION_OF_SIGNALING	ADA|CDH2	3.47E-08	1.71E-06	-1.358
GOBP_EPITHELIAL_CELL_PROLIFERATION	AKT3|CDH	4.88E-08	2.34E-06	-1.624

BERTUCCI_MEDULLARY_VS_DUCTAL_BREAST_CANCER_DN	ZBTB43|LS	1.00E-10	9.07E-09	-2.177
CHARAFE_BREAST_CANCER_LUMINAL_VS_BASAL_DN	CAV2|RND	1.00E-10	9.07E-09	-1.791
CHARAFE_BREAST_CANCER_LUMINAL_VS_MESENCHYMAL_DN	LYN|HACD	1.00E-10	9.07E-09	-2.005
DESERT_STEM_CELL_HEPATOCELLULAR_CARCINOMA_SUBCLA	ADAM9|A	1.00E-10	9.07E-09	-1.817
DUTERTRE_ESTRADIOL_RESPONSE_24HR_DN	MME|ENT	1.00E-10	9.07E-09	-1.699
GRAHAM_CML_DIVIDING_VS_NORMAL_QUIESCENT_UP	MINPP1|C	1.00E-10	9.07E-09	-1.976
GRUETZMANN_PANCREATIC_CANCER_UP	APLP2|DYF	1.00E-10	9.07E-09	-1.715
HAY_BONE_MARROW_CD34_POS_MKP	ITGA2B|AL	1.00E-10	9.07E-09	-2.380
HAY_BONE_MARROW_PLATELET	PPBP|CAV	1.00E-10	9.07E-09	-3.037
HAY_BONE_MARROW_STROMAL	CXCL12|AF	1.00E-10	9.07E-09	-2.066
LIM_MAMMARY_STEM_CELL_UP	COL17A1|T	1.00E-10	9.07E-09	-1.789
MURARO_PANCREAS_DUCTAL_CELL	SPP1|CFTR	1.00E-10	9.07E-09	-1.595
MURARO_PANCREAS_ENDOTHELIAL_CELL	FLT1|KDR|	1.00E-10	9.07E-09	-1.793
MURARO_PANCREAS_MESENCHYMAL_STROMAL_CELL	COL1A1|C	1.00E-10	9.07E-09	-1.941
NABA_CORE_MATRISOME	ABI3BP|AD	1.00E-10	9.07E-09	-2.007
NABA_MATRISOME	ABI3BP|AD	1.00E-10	9.07E-09	-1.664
P53_DN.V1_UP	F3|CDKN2A	1.00E-10	9.07E-09	-2.125
PILON_KLF1_TARGETS_UP	COL4A1|PT	1.00E-10	9.07E-09	-1.843
SMID_BREAST_CANCER_BASAL_UP	KRT23|H2B	1.00E-10	9.07E-09	-1.761
SWEET_LUNG_CANCER_KRAS_DN	COX7A1|T	1.00E-10	9.07E-09	-1.840
WANG_SMARCE1_TARGETS_UP	MAN2A1|	1.00E-10	9.07E-09	-1.880
WONG_ADULT_TISSUE_STEM_MODULE	CCN2|MAF	1.00E-10	9.07E-09	-1.667
WP_PI3KAKT_SIGNALING_PATHWAY	HGF|TCL1B	1.00E-10	9.07E-09	-1.856

WU_CELL_MIGRATION	RAB25|TH	1.00E-10	9.07E-09	-1.983
PID_AVB3_INTEGRIN_PATHWAY	ANGPTL3|	1.03E-10	9.35E-09	-2.189
GRAHAM_CML_QUIESCENT_VS_NORMAL_QUIESCENT_UP	MPST|TPS	3.56E-10	2.86E-08	-2.114
PEDERSEN_TARGETS_OF_611CTF_ISOFORM_OF_ERBB2	PLAUR|SLC	4.78E-10	3.74E-08	-2.139
SARRIO_EPITHELIAL_MESENCHYMAL_TRANSITION_UP	PLK1|H4C3	6.36E-10	4.77E-08	-1.897
PEDERSEN_METASTASIS_BY_ERBB2_ISOFORM_7	H2AZ2|ECI	2.83E-09	1.86E-07	-1.605
SUNG_METASTASIS_STROMA_UP	SDC4|DKK	3.94E-09	2.48E-07	-1.990
TURASHVILI_BREAST_DUCTAL_CARCINOMA_VS_DUCTAL_NORI	RARRES1|(	4.83E-09	2.96E-07	-1.844
DUTERTRE_ESTRADIOL_RESPONSE_24HR_UP	GREB1|MY	5.31E-09	3.22E-07	-1.672
DELYS_THYROID_CANCER_UP	KRT19|CRL	8.72E-09	5.03E-07	-1.559
FISCHER_G2_M_CELL_CYCLE	CDCA3|TTI	9.91E-09	5.65E-07	-1.733
SOTIRIOU_BREAST_CANCER_GRADE_1_VS_3_UP	NCAPG|MA	1.67E-08	9.04E-07	-1.875
DELYS_THYROID_CANCER_DN	ARL4A|PLA	1.68E-08	9.08E-07	-1.756
CORRE_MULTIPLE_MYELOMA_UP	MYO1D|TF	1.98E-08	1.03E-06	-2.017
CHIANG_LIVER_CANCER_SUBCLASS_PROLIFERATION_UP	AURKA|CC	2.24E-08	1.15E-06	-1.836
SCHUETZ_BREAST_CANCER_DUCTAL_INVASIVE_UP	LPXN|NUA	7.19E-08	3.31E-06	-1.597
GOTZMANN_EPITHELIAL_TO_MESENCHYMAL_TRANSITION_UP	MAP4|CSF	1.44E-07	6.11E-06	-2.005
SWEET_KRAS_TARGETS_UP	TIMP3|GLI	2.26E-07	8.93E-06	-1.959
PEDERSEN_METASTASIS_BY_ERBB2_ISOFORM_4	IGFBP5|SP	2.51E-07	9.79E-06	-1.878
IWANAGA_CARCINOGENESIS_BY_KRAS_PTEN_DN	FGF14|GRI	4.56E-07	1.65E-05	-1.560
IGLESIAS_E2F_TARGETS_UP	CYFIP1|CLI	5.06E-07	1.80E-05	-1.814
SMID_BREAST_CANCER_BASAL_DN	PAX2|LRR(	1.67E-06	5.14E-05	-1.396
HUPER_BREAST_BASAL_VS_LUMINAL_DN	FUT3|BACI	2.73E-06	7.81E-05	-1.990
CHARAFE_BREAST_CANCER_BASAL_VS_MESENCHYMAL_DN	ZCCHC24|I	5.56E-06	1.42E-04	-1.964
TURASHVILI_BREAST_LOBULAR_CARCINOMA_VS_DUCTAL_NOI	KRT17|IL2(	1.15E-05	2.64E-04	-1.844
LIEN_BREAST_CARCINOMA_METAPLASTIC	THBS1|STA	2.47E-05	5.03E-04	-1.971
LIM_MAMMARY_LUMINAL_MATURE_DN	LAMA1|AE	5.95E-05	1.04E-03	-1.750
LIAO_METASTASIS	PPP1R9A|S	7.05E-05	1.19E-03	-1.369
FARMER_BREAST_CANCER_BASAL_VS_LULMINAL	VGLL1|PRN	1.42E-04	2.08E-03	-1.445
FISCHER_G1_S_CELL_CYCLE	POLD3|MC	1.45E-04	2.12E-03	-1.531

MIR6867_5P	PIAS2|SLIT	1.00E-10	9.07E-09	-1.522
MIR3662	VSIG1|NUI	3.16E-08	1.57E-06	-1.410
MIR3681_3P	MBOAT2|S	7.71E-08	3.52E-06	-1.580
MIR96_5P	SOX6|FARI	1.09E-07	4.78E-06	-1.530
MIR335_3P	NUDT15|A	1.93E-07	7.84E-06	-1.383
MIR182_5P	NUDT15|P	4.13E-07	1.52E-05	-1.479
MIR651_3P	ESRRG|IPC	5.83E-07	2.05E-05	-1.392
MIR9985	UNKL|TME	6.26E-07	2.18E-05	-1.442
MIR130A_5P	PCDHA2|C	1.02E-06	3.36E-05	-1.560
MIR1277_5P	CXXC5|ZCF	2.36E-06	6.91E-05	-1.353
MIR8068	ITGA4|MM	2.67E-06	7.65E-05	-1.507
MIR493_5P	AP4S1|NU	4.18E-06	1.12E-04	-1.449
MIR6868_3P	PTPRM|TN	4.26E-06	1.14E-04	-1.588
MIR9_3P	AGFG1|ME	5.71E-06	1.45E-04	-1.520
MIR548P	PXK|AGFG	7.16E-06	1.76E-04	-1.401
MIR664B_3P	PIAS2|AGF	7.43E-06	1.82E-04	-1.431

MIR9_5P	CREB5|SIX	7.43E-06	1.82E-04	-1.440
MIR124_3P	MBOAT2|I	7.57E-06	1.85E-04	-1.352
MIR374A_5P	AHSA2P|P	8.13E-06	1.97E-04	-1.463
MIR5197_5P	DIP2B|UN	8.20E-06	1.98E-04	-1.670
MIR144_3P	PRDM16|N	8.96E-06	2.14E-04	-1.427
MIR98_3P	NUDT4|PR	9.80E-06	2.31E-04	-1.367
MIR548AH_5P	AGFG1|AD	9.94E-06	2.34E-04	-1.508
MIR6124	MBOAT2|E	1.08E-05	2.51E-04	-1.426
MIR6809_3P	CD84|ESR	1.49E-05	3.30E-04	-1.404
MIR588	TMEM159	1.67E-05	3.63E-04	-1.734
MIR340_5P	EVI2B|BTB	1.75E-05	3.77E-04	-1.340
MIR548AT_5P	AGFG1|SE	1.91E-05	4.07E-04	-1.489
MIR4755_5P	ESRRG|KX	2.39E-05	4.90E-04	-1.467
MIR29A_3P	ARHGEF10	2.58E-05	5.20E-04	-1.434
MIR129_5P	MDM4|CR	2.67E-05	5.33E-04	-1.464
MIR20B_5P	PXK|MINK	2.69E-05	5.37E-04	-1.363
MIR4753_3P	BTBD1|PR	2.69E-05	5.37E-04	-1.389
MIR145_5P	MEST|DD	2.84E-05	5.59E-04	-1.495
MIR7110_3P	ESRRG|AP	2.95E-05	5.78E-04	-1.439
MIR6798_5P	PRDM16|N	3.56E-05	6.78E-04	-1.913
MIR6768_3P	SIX4|ARPC	3.57E-05	6.80E-04	-1.665
MIR206	TMCC1|DD	3.62E-05	6.86E-04	-1.450

size
198.00
192.00
129.00
179.00
198.00
199.00
192.00
33.00
186.00

82.00
192.00
200.00
88.00
181.00
40.00

975.00
74.00
289.00
120.00
58.00
249.00
38.00
307.00
486.00
693.00

739.00
179.00
391.00
980.00
931.00
1370.00
1516.00
665.00
1333.00
964.00
1683.00
339.00
103.00
975.00
551.00
1305.00
980.00
1069.00

83.00
1674.00
155.00
120.00
891.00
1566.00
1461.00
655.00
1486.00
1340.00
586.00
491.00
85.00
476.00
786.00
1144.00
103.00
1460.00
1097.00
480.00
152.00
1267.00
339.00

171.00
442.00
456.00
239.00
497.00
180.00
353.00
60.00
251.00
685.00
455.00
1244.00
355.00
671.00
256.00
863.00
185.00
468.00
589.00
393.00
276.00
683.00
303.00

171.00
73.00
90.00
72.00
163.00
380.00
107.00
181.00
314.00
429.00
234.00
152.00
209.00
74.00
173.00
340.00
68.00
81.00
104.00
336.00
143.00
650.00
56.00
50.00
82.00
30.00
90.00
524.00
314.00
192.00

842.00
927.00
383.00
443.00
847.00
552.00
784.00
602.00
313.00
864.00
386.00
449.00
257.00
335.00
583.00
479.00

467.00
722.00
419.00
178.00
479.00
705.00
341.00
436.00
526.00
120.00
765.00
345.00
353.00
422.00
361.00
605.00
526.00
299.00
382.00
45.00
135.00
365.00

LINPN_genes
FILIP1L, CNN3, COL1A1, MYL9, DPYSL3, COL6A3, SPON1, PCOLCE, COL3A1, COL5A2, CALD1, ACTN1, AQP1, PTGIS, I
KRT19, MYH11, LAMB1, TNC, SNAP25, DYNC1I1, KIF5C, STMN2, NEFL, GAS7, LAMA5, INA, STMN1, KRT7, LMNB1, K
SLC16A4, CD55, FBXL7, CFD, COL1A1, MYL9, GPC3, MARF1, CD14, TSPAN7, RNASE4, TGFBR2, ALDH1A1, FCGRT, CC
SLC16A3, WFS1, CNN3, RRAD, TNFAIP3, CFD, COL1A1, MYL9, GPC3, MARF1, CD14, EMP2, FCGRT, COL6A3, PCOLCE
CALD1, GJA1, FXYD1, CXCL12, CRYAB, FKBP1B, FLII, CHRNB1, GSN, GNAO1, TMSB4Y, VIPR1, RYR1, OXTR, NOS1, CA
CFD, MYL9, TSPAN7, PCOLCE, CALD1, CHGA, ENPP2, SELENOP, PDGFRA, VWF, IL1R1, ITGA6, SLPI, GJA1, LRP1, BCL6
MYL9, SPON1, KRT19, GSTM1, IGFBP2, PLA2G2A, AKR1C1, PRSS23, ECM2, SLPI, GPX3, CDKN1A, FAT1, ADIRF, DLK1
SLC16A3, FILIP1L, CNN3, GPC3, PLSCR1, EMP2, MDK, TGFBR2, ALDH1A1, DPYSL3, FCGRT, IFI16, PCOLCE, DEPP1, KF
KRT19, TM4SF1, AKR1C1, ITGA6, CAV2, DUSP6, CAV1, SERPINE1, PTPRK, DST, ALDH1A3, S100A8, MAOA, ITGB4, S1

GDA, SLC2A4RG, RORB, CUL3, TAF5L, MAP3K5, EPC2, MAP3K9, FOXF2, MAP3K8, PHTF2, FSTL5, OGT, BHLHE41, RC
UBFD1, SEPTIN4, THRB, XPO4, RPS6KB1, ITSN2, UBQLN2, TBC1D19, ZIC1, UXS1, CD2AP, ATP2B2, ANK1, CLK3, ANK
SLC9A8, JAKMIP2, SCN3A, VKORC1L1, ITSN1, AMOTL2, ADGRL3, CUL2, EPC2, MAP3K5, FAP, PHTF2, SERPINE1, PPT
SLC9A9, KCNE5, ESRP1, MEF2A, KIF24, LTBP2, PPP2R5B, HNRNPF, SAMM50, SYNCRIP, H1-10, RORA, AMOTL1, AM
MEF2C, CTHRC1, OSMR, LRRC8E, HNRNPD, MED25, PLEC, ATG13, AGBL5, ISG20, BTK, ADGRL2, MAP3K6, DCAKD, D
ABCC3, ABCE1, ABLIM3, SDHAF3, ACRBP, ACSBG1, ACVR1, ADD3, ADK, AGFG1, AHCTF1, AKAP11, AKAP7, ALG5, AL

PRKCG, CREB1, GRIA3, GNAI1, GRIA2, GRIA1, ADCY8, PRKCA, ACTG1, PRKCB, CAMK4, ACTG2, DRD4, CAMK2A, GJB
BAMBI, TFDP2, PROS1, CPM, SERPINE2, VPS13A, ZMYND8, ELOVL5, TUSC3, LY86, STAC, FAM20B, PIR, GGH, COA1,
BCAR1, GPC6, TEAD2, TEAD4, THBS2, TEAD1, EDNRA, MIR509-3, TEAD3, PBX3, TWIST1, COL3A1, SNAI2, FN1, COL5
WEE1, CSNK1E, BACE2, FZD5, SOX9, SEMA4G, PIK3C3, ASAP1, MAP2K2, ELF3, PRKCD, FGFR3, DUSP1, GYS2, UBD, F
MPRIP, KCNK2, GDAP1, NBEA, GPX4, RHOQ, PLAAT3, PUS7, SLC38A1, KDM5B, GDF3, EFNB2, TGIF2, PCLAF, SETD2,
MIAT, FBN3, CPNE7, PCDH11X, LINC00342, CAVIN4, SSTR2, GOLGA8B, CHD5, PRKAR2B, KIAA0319, NCOR2, SRRM3
SDK2, GPC1, MARCKSL1, FAM229B, SDK1, EPM2AIP1, CELF2-AS2, DBNDD1, FOXP1, TLE5, STK25, C12orf73, RIT2, S
NCK1, LMX1B, CD2AP, PODXL, UTRN, TGFB1, FAT1, LAMB2, CR1, SCARB2, CTNNB1, PLCG1, MYO1E, ITGA3, SYNPO,
VEGFA, POU3F4, ABCB9, ICAM1, HIPK3, NPAS2, BMP10, G0S2, KXD1, GTSF1L, NYX, DPYSL3, SORBS2, RPS6KA5, PIK
RCAN3, AGO4, AKIRIN1, RPS8, PLK3, L1TD1, GADD45A, MIGA1, CCN1, LRRC8C, FNBP1L, LRIG2, PIP5K1A, TUFT1, IL(

RASSF5, DRAIC, DAG1, KDELR2, CBR1, NFIX, NCEH1, CYB561, ANK3, MYLK, MARK1, SLC22A15, TP53INP2, ATP2A3,
MDK, PLK2, TUBB2B, SPOCK1, SESN1, CCN1, GAP43, GHR, GPC3, KIT, ACTR3B, NEBL, CRABP1, TIAM1, MAGI2, PLCL
EMCN, LDB2, FLT1, PTPRB, HECW2, RAPGEF4, PLAT, TEK, MEIS2, PLPP1, CHRM3, ITGA8, PREX2, GNA14, SLCO2A1,
IFT22, MTTP, SUOX, GOLGA6L9, NRG1, MBNL3, OXTR, SNAP23, ASF1A, KLHL41, UBE2D3, NR3C1, QKI, VPS13C, TNI
AASDHPPT, AATK, ABLIM3, ACTL6B, ACVR1B, ACVR2A, ADARB2, ADAT2, ADD2, AFF2, AJAP1, AKAP1, AKR1C1, AME
SSBP2, GNA13, KRT36, ANK3, CLU, FASTKD2, UBQLN4, FUBP1, TMCC3, RBBP6, H2BC14, PIP, SLC35A2, RIOK2, RPS8
STAU1, FAM106A, MASP1, CCSER2, ADCY6, SYT7, HNRNPL, AQP2, VSTM2A, ART4, GRIN2B, SEMA7A, LCAT, PLA2G
CYP1A1, CEACAM5, AKR1B10, OTULINL, CEMIP, COL5A2, SGPP1, ALDH1A3, CYP1B1, UPK3B, S100A6, UPK2, MEF2C
ABHD17A, ACBD7, ACSL6, ACSS1, ADAMTS3, ADAMTS6, ADCYAP1R1, AFF1, ALDH9A1, ANXA5, APBA1, AQP4, ARAF
SLC8A1, LINC01099, CALB1, TEX41, SNTG1, PIK3C2G, PDE4D, SCN2A, CADPS2, PRKG1, DACH1, ARL15, KCNIP4, ME(
ACOT7, ACSL4, ACSL5, ACTL6B, ACVR1B, ADARB2, ADCY2, ADCY8, AJAP1, AKAP1, AKAP12, AKR1C1, AKR1C2, AMPH
ADAM12, ADAMTS5, ADAMTS8, ADCY3, ADORA2B, AGPAT4, AIDA, AK5, ALG10, ANLN, ANXA3, APCDD1L, ARHGAP
IPO5, MBTPS2, SSX2IP, TMEFF1, NID1, CBL, ZADH2, MCFD2, PHC3, ROBO1, PDK3, SLC44A1, MCTP1, RPN2, ROBO2
VSIG1, MBTPS2, CREB5, AMZ2, TMEM170A, SLC25A21, SLC4A10, MDM2, TAF1C, ZNF148, AFF2, KCNJ1, ARMC1, PL
KDELR3, LCN2, HOXC6, THBS1, CPE, BCL2A1, CTSS, KIFAP3, POU2AF1, LGMN, PLAT, IGFBP1, ABHD2, S100A9, ANKF
CSF1R, CD9, CD1B, CD1C, CD1A, TLR8, TBXAS1, ASGR2, TNFSF13B, ASGR1, BCL6, PCTP, HLA-DPB1, HLA-DPA1, HLA-
THBS1, STAG2, TIMP3, PKD2, HTRA3, SPARC, RUNX1T1, TBX2, PDGFA, IQCA1, TPM4, PDGFRA, KCNE4, LUM, MSX1,
TAF7L, LGALSL, ZNF423, HOMER1, RECK, CERT1, VLDLR, CCNA2, B3GALNT1, ANKRD13A, FBXW7, DCAF13, ZNF277,

SP8, UNC5D, RAB11FIP4, KCNQ2, DNAH11, GJB2, ATP2B3, DMRT2, NFATC2, PTH2, HRH3, MAFA, LHX8, TLX1, SPTB
HCRTR1, CELSR1, SCN4B, HOXB3, KCNQ1, KCNK3, CYP26A1, SFRP5, NKX2-1, ONECUT3, KCNQ2, SLC22A3, TLX3, NK
CLDN10, LRRK1, MAFB, KRT18, LRATD1, DNM1, ABCC3, SLC2A5, CEACAM1, CITED1, ETS2, ANO1, SNORC, TJP2, CD
CCNB1, TUBB4B, PALM2AKAP2, CENPA, NUF2, KIF15, RCAN1, UBE2T, HMGB3, KIF14, NCAPG, LETM1, MZB1, RUFY
BCAP31, G6PD, MYO5B, MAP2, NEUROD4, WARS1, BMP1, GABRP, NEDD4, NABP1, PSAP, RPL3L, GPD1, CERS4, QS
NUDT15, AGFG1, ITPRID2, CASTOR2, SIX4, AFF4, SLCO1A2, ELN, DIP2B, ZNF23, AFF2, NSFL1C, C5orf38, NTRK2, ARI
ACVR2B, ADAMTS5, AFAP1, AJAP1, AMER2, ANK2, AP3B2, APOL4, ASIC3, ASXL3, ATCAY, ATL1, ATP1A3, AUTS2, BC
PNOC, DDC, CREB5, TCF12, SYT17, OLFM3, BPGM, ZNF148, ZKSCAN8, ADAL, AUTS2, SPATA33, IKZF1, PIWIL2, FN1,
PXK, MYB, SSX2IP, PAK3, ATP5MC3, ZNF471, CLEC2D, MEF2C, PPP6R3, CDC27, SSR3, CAPS2, FPGT, LPAR1, HDAC9,
PXK, MINK1, AGFG1, CREB5, PCDHA2, SIKE1, HLF, ZNF148, CAPRIN2, FCHO2, AGTPBP1, GAB1, IL6ST, PRRG1, NEDD
MTTP, TRMT1L, ADAM22, YY1AP1, UBN2, PALM2AKAP2, CUL2, SETX, PSMA8, DNAJB4, MID1, DYNLT1, CTBP2, ETS
MDM4, CREB5, AFF2, ZEB1, FMO1, MORF4L2, ARHGEF7, NEK1, GOLT1B, BTBD11, CBL, SHISA9, TRAM1, SPATA13,
ATM, ADAM22, SLC30A6, MCF2, SLC25A21, RCHY1, ARMC1, PDPK1, PDE6D, CA2, MBNL3, ZNF32, CTNNA1, URI1, N
DOK1, DDX5, WRNIP1, CDKL5, CBL, MAP3K7CL, EGLN1, PPP3CB, SLC30A7, PDZD2, HNRNPH3, CRISP1, ATP9A, ROR
EVI2B, BTBD1, GUCY1B1, PRDM16, SLC30A6, RANGRF, ATF1, CTCFL, SELENOI, CDKL5, DMD, STOX1, BVES, RBM24,
KDM5D, AGFG1, MBOAT2, GUCY1B1, PCDHA2, TMEM159, DIP2B, HMCES, MDM2, ZNF280D, PFDN4, CUL2, SHMT
COL17A1, TP63, ECRG4, CCND2, DLK2, KRT5, ACTG2, MYH11, ISM1, MYLK, IGFBP2, SOBP, PDPN, LAMA1, DKK3, KC
ESR1, SOX6, SMARCA2, MDM4, PCDHA2, HNRNPUL1, CCM2, EHBP1, CASTOR2, SIX4, LGALSL, ZNF217, CBX6, C11o
MYH11, NTRK3, PDZRN4, MCAM, TAGLN, KCNAB1, CACNA1C, ACTA2, HPSE2, SPARCL1, DGKG, SLIT3, PDE3A, GRID
GSTM4, EBP, SH2D2A, MNS1, ORC1, KIF14, SLC12A2, ILF3, NSD2, AKAP12, CTSA, PSMA1, GEM, CENPU, DAB2, SEC
CCND2, STAT1, SAA1, MFAP5, LAMC2, H19, COX7A1, KRT7, KRT17, IFI27, MYH3, ASAH1, TNFSF4, PAPPA, C3, KRT1
MREG, TIAM1, RBM24, BCL2, NBPF1, SLC39A6, NSUN5P1, RAB11FIP3, AMFR, TUBB6, MAGED4B, RERG, ISOC1, FZD
MBTPS2, PAK3, GOLT1B, TMCO1, MCFD2, LPAR1, RBPJ, C21orf91, PDK3, RPS6KA5, ASAP2, PURB, MED21, PRPF40

SLC16A4, FILIP1L, FBXL7, RNASE4, CFH, COL4A1, FSTL3, MYH11, THBS2, FN1, SFRP1, CCN5, PDLIM3, COX7A1, CFH
GNA13, N6AMT1, NOG, CHERP, HMGN2, POSTN, ZIC1, CBX5, HOXC6, BDNF, GRIN2B, SND1, HOXC4, MACROH2A1,
EEF1D, ABCB1, APP, ABHD10, ZNF274, STXBP6, RIPOR1, GSDMB, BIN1, TTC3, RUNX1T1, BAIAP3, HSPB2, BCR, PITP
AMOTL2, ANKRD1, AXL, BICC1, BIRC5, CDC20, CDKN2C, CENPF, COL4A3, CRIM1, CCN2, CCN1, DAB2, DDAH1, ASAP
RBM5, RUNX1, PTN, SLC10A3, PLP1, NR2F2, REL, MYH11, ZNF646, ZNF148, NOS2, PRSS53, VAPB, CNN1, NR4A1, P
KMT2E, ITGA2B, KMT2C, PRKCQ, CBFB, TNRC6C, TNRC6A, AGO1, SETD1A, TNRC6B, EP300, MYL9, GATA1, H2AZ2,
HNRNPK, MEF2C, DLC1, AJUBA, MLIP, RFT1, LTBP2, HTN1, PLEC, PRKG1, CITED2, SRSF6, CDH22, BDNF, DDX17, FAI
STAT1, IRF1, HAPLN3, GBP1, GBP2, GBP4, APOL6, UBE2L6, TRIM69, GBP5, PARP9, FBXO6, INPP1, EPSTI1
MEF2C, LTBP1, CDX2, C5orf64, SLC9A5, EDN2, STOML2, SYNCRIP, PTPN22, WT1-AS, BBOX1, CTNNB1, BDNF, FLI1,
H3C2, NUTF2, AIDA, RGS1, MSMO1, ADM, RGS16, SPP1, DUSP7, SLC29A2, FCGR2B, ALOX12, E2F8, PDK1, CSRP3, C
ITGAL, TYROBP, CD22, SLAMF7, CDH1, COL17A1, CLEC2D, FCGR2B, PVR, ICAM3, PILRA, SIGLEC1, ICAM1, CD200, T
IL12RB2, SPIRE1, CACNA2D2, GZMH, RNF165, PRSS23, PDGFD, RASGEF1A, COLQ, IL2RB, KLRB1, GNLY, CCL5, PRF1,
PFKFB4, CCNG2, CREB3L1, STON1, ANG, ZDHHC13, DEPP1, NFKBIZ, SYT17, NNMT, CNTNAP3, CXCR4, WNK3, TMTC
SORT1, RNF43, SDR42E1, APOC1, THRSP, LTF, TMEM184A, KLC3, NIPAL2, C1orf56, KIF21A, DENND2D, RAB25, C1o
MEF2C, MEF2B, CDIPT, PPP2R5B, HNRNPD, OGN, EPC1, CDH20, EPC2, DHX38, RPLP0, STAG3, PPP1R2B, PDHA1, B
MEF2C, CTHRC1, SCN3A, PPP2R5C, HNRNPR, RORA, ITSN2, PLEC, AGBL5, ADGRL2, MYOT, VNN1, STAG2, PIK3CG, I
DNAAF3, ZYX, MANSC1, PRTFDC1, FCN1, EPHX2, ROCK2, ZNF185, ARHGAP21, ACTN1, MS4A3, STX11, TTK, BANK1,
LTF, SNCAIP, LYZ, TSPAN15, GGA1, CST3, GGA2, CALB1, TSPAN14, ODAM, APOA4, CALCA, APH1A, APOA1, TTR, TG
MGST3, CA12, CD59, CXCR2, GDF10, LGALS3, TRAPPC12, CD44, SSH1, HOXA11, ACSM3, YLPM1, GAS6, LAMA4, AN
JAKMIP2, STOML2, RORB, RORA, AQP3, HOXD10, WDR73, LIN28A, BAIAP2L2, GTPBP1, PHOX2B, TYRO3, ATRNL1, T
CD3E, KRAS, FOS, CD247, IFNAR2, IFNA16, EGR4, MAPK1, TNFRSF18, PPP3R1, CD3D, PRKCQ, B2M, NFATC1, TNFRS
CRISP3, DEFA4, BPI, ABCA13, CEACAM8, DEFA1B, LTF, MMP8, OLR1, RNASE3, CAMP, DNM1P46, TCN1, MS4A3, SL
ARHGAP12, SETD2, USP9X, CEMIP, ADGRG6, ATP13A3, HIVEP2, GIGYF2, NDC80, CBLB, EIF4G3, STAG1, DHX15, HE

PIK3R1, PYGL, TBC1D9, PSD3, UBA6, RPS6KA2, AZGP1, TMEM33, NAT1, NMI, AKAP11, ABAT, PDE10A, SOX9, NAM
BEX2, EIF4E3, CDC14A, MAGI3, FYN, IGF2BP3, SOX4, TSPY26P, AQP11, LST1, OOSP2, PTGR1, DNMT3A, PRKD3, RM
LAS1L, ELAC2, TSR3, RPS20, UTP18, TFB1M, RPL26L1, MTREX, NOP58, RIOK2, RPL18, WDR3, WDR18, THUMPD1, R
AP2B1, FYN, AP2S1, EPHA3, NGEF, RHOA, ROCK1, CLTCL1, CDC42, EPHA8, NCK2, ACTB, PAK3, EPHA6, PSEN1, MM
MALAT1, DNAJB14, SEH1L, ANO1, NT5DC1, USO1, ZBTB10, SPAG9, SSBP2, UBE2W, CAPZA2, SLC12A2, HAND2-AS1
ITGA2B, DCN, IBSP, MUSK, VCAN, TNC, COL9A2, LAMA3, ITGA8, COL5A3, COL4A4, ITGB5, LAMB1, COL9A3, TGFB2,
SERPINH1, NUAK1, WFDC2, SMPD2, CDC42EP1, STAB1, CALU, PIGR, DKK3, ANXA2, IFI27L2, LTF, USP31, TPSAB1, C
RBPMS2, MEF2C, VKORC1L1, WTAP, HOXD10, CUL3, ADGRL2, MAP3K5, CLK3, HNRNPU, TLK1, ZNF395, CAB39, FAS
EPSTI1, TNFAIP6, C3orf80, LGALS3BP, HERC5, SLC9A3, AMMECR1, LTBP3, CDH1, PLD4, DEFB1, PRSS23, TPSAB1, F2
PAK1, BIRC3, HLA-DQB1, NELL2, KLRB1, CD6, TRAT1, PRKCH, XCL2, SH2D1A, LEF1, SAMD3, GNLY, CD96, CD3G, IL3
CP, TM4SF18, TM4SF1, TM4SF1-AS1, TM4SF4, WWTR1, WWTR1-AS1, COMMD2, ANKUB1, RNF13, PFN2, TMEM18
ITGA2B, PSMB1, RANBP9, FYN, PPP5C, PSMC4, HGF, LRRC7, VCL, CUL3, PSMA4, KITLG, PIK3CB, RASGRF1, CAMK2B
CD58, C1QC, LY96, C1QA, CD86, C1QB, TYROBP, SIRPA, HLA-DQA1, NFKBIA, TNFAIP3, RIPK2, NLRP3, NOD2, TLR4,
SPATA31A7, TRPV6, KRT81, HOXB4, GALNT14, ARHGAP24, LINC01940, FGFBP1, MSTO1, NLRP3, DLX1, SYTL3, FAT2
RXFP1, SHISA2, PLCH1, JPH1, TNFSF10, SERPINB10, OGDHL, BTLA, SLCO4C1, SERPINB2, CYP4F3, NLRC3, ID3, TPD5
UBE2E3, FABP7, TUBA1B, L1CAM, COL4A1, TNC, CDH2, CCT7, NRP1, SQLE, FDPS, CHL1, CCT4, TARS1, ST8SIA2, TUB
TMEM45A, ST3GAL5, CITED1, MGST3, ALDH3B1, NDST4, PTGES, ARMCX3, PRODH, SPRY4, BAG1, FGFR4, CELSR2,
APOD, LUM, DCN, ADH1B, MYOC, C3, CFD, ABCA8, PTGDS, GSN, SMOC2, DPT, PDGFRL, CXCL14, COL6A3, C1S, FBL
S100A4, ABLIM1, DEFA1, ATM, CTSG, ANXA2, NFKB2, MYLK, S100A9, S100A8, ARL4C, OLFM4, DEFA4, AZU1, BAX,
LEPR, PDGFRB, TXN, SPRY2, ITGA2B, SRC, INS, DOK1, STAT3, RHOA, CAV1, CRK, CSN2, JAK2, CAPN1, STAT5B, FER,
MEF2C, STOML2, ITSN1, SOHLH2, WWP2, ABI3BP, MAP2K7, STAG2, PITX2, PHOX2B, VANGL1, ANKS1B, ROCK1, GP
PTP4A1, EREG, CDH3, DDX21, EMP1, ROCK1, RDX, DUSP7, ID1, TOP2B, SEMA3C, PRKAR1A, ITGA6, THBD, CCND1,
POSTN, DENND2B, CETN2, SERPINH1, FCMR, FLT3LG, LPP, IQSEC3, MOCS3, HAS2, RAP2A, PDGFRL, FLNA, HBB, GA
SLC9A6, OSMR, SYT9, AQP4, CASK, TNFSF12, SSR1, RGL2, HOXC8, EPC2, PLPP6, CLK2, GATA6, SFSWAP, LIN28A, GC
TGOLN2, CTHRC1, JAKMIP2, CCSER2, PPP2R5D, FSTL1, DSE, FLI1, BRPF3, PLPP6, PHTF2, LUC7L2, ZNF395, TLK1, PTE
NPTX2, NRCAM, CYP4X1, RSPO3, FABP6, BCL2, AFAP1-AS1, RBP7, PARM1, CLDN10, NLRP7, TSPAN2, STXBP6, SHIS
GATA2, CRADD, MMRN1, FOXO1, LYL1, ISG15, PLOD2, MYC, ICAM2, GJA1, SERPINB1, MAP1B, OAS2, THBD, RIN1,
TMPRSS4, BIK, SERPINB5, KRT19, TRIM31, BORA, CDH3, CAPG, CEACAM6, IGFL2, HK2, STYK1, CEACAM5, QPCT, LA
SPI1, CBFB, CEBPE, EP300, E2F1, KLF5, H2AZ2, TFDP2, KMT2A, CREB1, MYB, CSF3R, CDK2, H2BC11, CDKN1A, DEK,
UHRF1, NRP1, TSPAN5, LSAMP, ANXA3, KCNN2, HELLS, PCLAF, ALDH1L2, GINS1, OXTR, C1QTNF2, RALGPS2, GMN

ARL4A, PLA2G7, TAL1, CLCNKB, RGS16, PLA2R1, SGCD, ITIH5, ZFPM2, GAS1, ZFP36L2, ITM2A, HLF, IQGAP2, EPB41
ITGB3BP, VSIG2, SYNCRIP, ITSN2, OPALIN, MXI1, SLC35A2, BPIFB2, RAB1A, BTK, DDX17, CRY2, GRIN2B, SFSWAP, B
SPOCD1, LCK, KCNA3, CD1A, CD1B, CD1E, CD247, LINC01222, LINC01221, LINC02789, MDM4, ITPKB-IT1, LINC009
COL3A1, DCN, COL1A1, PDGFRA, FBLN1, MFAP4, MYH11, CXCL14, COL6A3, EMILIN1, CD248, PTCH1, FENDRR, ACT
CGA, CSH1, CYP19A1, TFPI2, ADAM12, ANXA1, TGM2, SVEP1, PAPPA, SERPINE1, GRIP1, FAM184A, TACC2, CSH2, P
HRH1, MS4A7, SIGLEC1, C1QA, HMOX1, TIMD4, LILRB5, CD5L, KCNAB1, ADAP2, C1QC, C1QB, CSF1R, CD163, NDST
GAP43, MARCHF11, PRPH, NPY, HMX1, HS3ST5, NTRK1, PTCHD1, TMEFF2, IL7, EYA4, STMN4, TMEM132C, PTCHD
PPBP, TUBB1, PF4, GP9, ITGA2B, ITGB3, THBS1, LTBP1, TLN1, DNM3, SLC2A3, PLEK, VCL, PDE3A, P2RX1, TGFB1, NE
COL1A2, COL1A1, COL3A1, IGF2, POSTN, SPARC, H19, DCN, FBN1, COL5A1, COL5A2, FSTL1, VCAN, SEMA3C, ISLR, C
KRT19, CRLF1, H2AX, C3AR1, VAMP8, GALE, COL1A1, ACAA2, FAS, PLXNB1, BHLHE40, ABCC3, SFN, NINJ1, GPRC5B,
HBM, HBZ, SLC4A1, HBG1, TRIM10, HBA1, HBG2, RHAG, HBA2, AHSP, HBB, GYPB, GYPA, SPTA1, HEMGN, ALAS2, A
SPP1, CD74, HLA-DRB1, HLA-DRA, HLA-DPA1, LYZ, C1QC, RBPJ, C1QA, PLTP, CXCL9, HLA-DPB1, LGMN, HLA-DQA1,
GADD45B, UBE2M, ZFP36, CCL3, LTA, HMGCS1, BCL2A1, IRF7, TNFAIP3, SIAH2, CCL4, TNF, EEF1A1, DUSP2, FTH1,
C1QA, C1QC, C1QB, THEMIS2, C1orf162, CTSS, TNFAIP8L2, S100A12, RGL1, IL10, CR1, NLRP3, CD207, MARCO, STA
CSF1R, HLA-DPA1, CYBB, SPP1, HLA-DRB1, HLA-DRA, CD74, LYZ, RBM47, RBPJ, C1QC, HLA-DQA1, MS4A7, MPEG1,
TMEM40, PF4, ITGB3, RAB27B, RYR3, MFAP3L, MED12L, SLC37A1, ITGA2B, ABCC3, MTURN, TUBB1, PPBP, SELP, C

LGMN, SLC8A1, CD163L1, DSCAM, MS4A7, CHN2, KCNMA1, PID1, CD163, WWP1, NCKAP5, PTPRE, HCK, PPARG, M
HBM, HBA1, HBA2, HBB, HBG2, HBG1, SLC25A37, SPTB, TSPAN5, SLC2A1, SOX6, ANK1, RGS6, MARCHF3, SNCA, PR
RALGAPA2, NRG3, TFPI, PGM5, STON2, MMRN1, MPP7, NFATC1, TLL1, NTS, LINC02147, STAB2, PARD6G, PTGS2, T
MYH11, TAGLN, MYL9, C11orf96, ACTA2, TPM2, RGS5, RGS16, CALD1, TPM1, MYLK, FILIP1L, NDUFA4L2, DSTN, AD
PSCA, UPK1A, ELF5, AQP2, EHF, DHRS2, FXYD3, S100P, KRT7, SNX31, SCNN1G, MUC6, ADIRF, ADH1C, HMGCS2, TA
SULT1E1, HHIP, MGP, SFRP2, DCN, MSC-AS1, ITGA11, OGN, DKK2, RSPO3, COL3A1, LUM, CCDC102B, SCARA5, PRR
ADAM9, AGPAT5, ALDOA, AMD1, ANXA5, ARHGEF2, ARPC2, ASAP1, ASRGL1, ATIC, ATP11B, ATP1B3, BAMBI, BICC
MIR133A1HG, MYH3, TTN, CASQ1, SYNPO2L, MYBPC1, ARHGAP36, LDB3, MYH8, CACNG1, MYLPF, ACTA1, ATP2A1
HBG2, HBA2, HBM, HBG1, HBA1, ANK1, SLC25A37, RGS6, EPB41, SPECC1, TSPAN5, CPEB4, OSBP2, HBB, C17orf99,
EDN1, STOML2, LUZP1, RORB, WT1-AS, RAB1A, HOXD10, DDX17, BDNF, FAM122A, MAP2K5, TWIST1, PHOX2B, TY
GP9, PF4, PPBP, GP1BA, ITGA2B, ITGB3, P2RX1, THBS1, PLEK, FERMT3, ZYX, TUBB1, MMRN1, LTBP1, ARHGAP6, AC
BACH2, SKAP1, LEF1, BCL11B, IL7R, CD247, TCF7, THEMIS, CD52, LTB, TMC8, CD3E, CD6, CD69, NIBAN3, ITK, CD3D
SNTG1, MYRIP, MECOM, PTPRB, FGF12, RAPGEF5, MAGI1, SIPA1L1, MAGI1-IT1, ADAMTSL1, CHRM3, BBOX1-AS1,
DDI2, CROCCP2, E2F2, RHD, RHCE, MACO1, ERMAP, CDC20, SLC6A9, UROD, STIL, ORC1, DNAJC6, TLCD4, CD58, SE
CD74, CST3, RGL1, SPP1, RBPJ, HLA-DRA, HLA-DRB1, CSF1R, RBM47, CYBB, HRH1, CHN2, WWP1, ATP8B4, CD163L
CD247, THEMIS, SKAP1, LTB, LEF1, IL7R, RHOH, TXK, SAMD3, NIBAN3, TCF7, CD3E, PAX5, ITK, SCML4, MS4A1, IL2R
ALX1, NKAIN2, TNC, APCDD1, RERG, LAMC3, ATRNL1, TCF21, PALLD, GRID2, CCBE1, TMEFF2, CCN4, LAMA2, AGTR
AKAP12, GK, CRYAB, PRSS1, REN, CD69, SPARC, TNFRSF9, SMAD7, PHLDA2, GSN, CCRL2, CD44, ATP1B1, STC1, TFP
CFTR, PPARGC1A, C6, SLC4A4, UGT2A3, CALB1, LINC02532, VTCN1, DCDC2, LEFTY1, LYPD6B, PPP1R1B, FGFR2, LAN
S100A4, S100A6, FGL2, HLA-DRA, LYZ, CD74, HLA-DPA1, HLA-DPB1, HLA-DRB1, TYMP, CSTA, IFI30, CD52, S100A9,
NTRK2, AQP4, GJA1, CLU, PLPP3, CPE, MGST1, EDNRB, S1PR1, ATP1A2, TTYH1, GASK1B, TIMP3, GABBR2, APOE, GI
AJAP1, DISP3, EPHA8, MYT1L-AS1, SLC30A3, GAD1, CHL1, KIT, UNC5C, NEUROG2, LINC02144, HCRTR2, MSRA, XKR

PECAM1, DAB2, JUN, CTSK, MYLK, COL15A1, SGCE, AEBP1, SELENOP, PDGFRA, LUM, VWF, COL6A2, COL4A1, AKR
KIF11, ZWINT, CDC20, KRT4, CORO1A, SFTPD, ARHGAP4, KRT14, KRT5, DSP, FAM107A, KRT6A, CENPF, OCLN, DTN
OL6A3, AMT, PCOLCE, KRT19, COL3A1, GATM, COL5A2, ACTN1, GSTM1, ZYX, C1QB, IGFBP2, VEGFA, TM4SF1, AQP
E, DEPP1, KRT19, COL3A1, COL5A2, ZYX, GPNMB, C1QB, IGFBP2, TM4SF1, TGM2, PEG10, PECAM1, DAB2, ENPP2,
CNG1, GJA5, ACTC1, KCNA1, HRC, SSPN, CKMT2, ACTA1, PLN, MYL3, DSTN, CLCN1, TRDN
5, FAT1, MFAP2, C7, IFIT1, STAB1, CLEC2B, COL6A1, TPM2, COX7A1, CFHR1, ADIRF, HSPG2, LGMN, IGFBP6, CXCL1
, FMOD, SDC1, CYB5A, CD24, NGFR, ANK3, TSPAN8, H2BC21, STMN1, KRT7, EPHA2, KRT18, MYO10, CAVIN1, BM
RT19, COL5A2, CALD1, ACTN1, ZYX, GPNMB, IGFBP2, VEGFA, TM4SF1, TGM2, PEG10, PECAM1, DAB2, SGCE, ENG,
100A9, CXCL1, AKR1C3, KRT15, KRT14, KRT5, SERPINB5, TP63, S100A2, TRIM29, PI3, CSTA, GPX2, DSP, KRT6A, BA

OCK2, KIF5A, ZHX2, MECP2, BTBD7, C14orf28, PTPRT, ZBTB46, RSRP1, ZNF236, ZBTB18, NAA30, MAPK6, PPP6R2,
3, CLK2, UNC5A, SAR1B, RAI14, SRSF1, MIB1, SLITRK4, DOC2A, LRCH2, ATP2C1, SCYL2, PPP2R5E, STMN1, SEPTIN7
C7, STAG2, ADAM9, TMEFF1, MAGI2, ZNF644, ZNF507, PIK3CD, MECP2, BTBD7, LIFR, UBE2J1, BSN, SCYL3, BCL2L
OTL2, CITED2, ACTG2, OGN, MYOT, AQP11, FAP, PPP1R2B, PSMA8, SMOX, TIGD4, PDHA1, PPTC7, PLS3, VNN2, LF
DIRAS1, APP, RAB27B, TMEM14B, CDH23, LRRC2, AIG1, BARHL2, MECP2, KDM5C, ZNF503, MARK1, MARK2, SSTR
LG6, ALOX12, AMD1, ANAPC10, ANKRD12, ANKRD13A, ANKRD46, ANTXR2, AQP10, ARFIP1, ARL6IP5, ARL8B, ARP

1, GRIN2A, DRD2, GRIN1, DRD1, MAPK1, MAPK3, RAF1, PPP1CA, GRM1, GNAS, RAP1A, RAP1B, ARAF, GRIA4, ADC
ZNF91, TNFRSF1B, ICAM2, ITGAX, ALDH1A1, AHNAK, ARID5B, CD180, CSF2RB, C2orf27A, ITSN2, MED21, TJP2, IT
5A1, COL1A1, SPARC, YAP1
ROBO2, MAPK13, LINGO1, GADD45A, CLDN4, IGF1, HNF4A, ARPC5L, ARHGEF15, GRB7, DKK1, MAP3K5, BAK1, CLD
SERPINA5, CTNND2, BAMBI, NRCAM, ADIPOR2, RAMP1, NR2F2, PCCB, ESPN, LXN, SNORD62A, XIST, CUX1, DNAJ
3, PODXL2, TMEM63C, SYCP2, MEG3, SLC4A7, AP1G2, SH3PXD2A, CHST2, CNTNAP1, ULK2, CELF6, PRKCB, SNAPC4
ULT4A1, WWC1, MDGA1, HMGCR, NRXN1, SAMD4B, JPH3, C1orf216, C11orf95, PIK3R2, RAB3A, ZBTB18, NCOA1,
, YWHAQ, LAMA5, TLR4, ITGB3, PAX2, ITGB4, PCNA, CLDN1, WT1, TLN1, CDKN1C, CDKN1B, CDKN1A, SMARCAL1,
FYVE, KLHL28, NOVA1, SYCE1, OR7C1, PLK1, BRICD5, CRISP3, BTG2, RTKN2, TBC1D9, NHS, PLCXD3, IL23A, EIF4E,
6R, IER5, LAMC2, NEK7, OPTC, RASSF5, IL20, TGFB2, LINC01341, LINC00115, CCNL2, KLHL21, IFFO2, CRYBG2, RNF

SLC9A6, MICB, SMAP2, RPS6KC1, FFAR2, ATG2B, OLFML2A, LINC01588, TMEM131L, BAIAP2, SYNJ1, FLVCR2, ACS
1, PDZD2, AHNAK, LGR4, FGFR1, LGR5, SLIT2, MPPED2, ADGRG2, SYT1, NRXN1, ANK3, OMD, COL5A3, PCDH9, ITG
IGFBP5, ZEB1, SHANK3, RBMS3, NRG3, EPAS1, ADGRF5, CDH13, TIMP3, ELMO1, SYNE1, NOSTRIN, GRB10, TCF4,
K, FAM13A, HNRNPLL, STX7, SAMD5, PTBP3, MTDH, ZNF367, GYS1, MSL2, PPP1R9A, ZBTB20, NAB1, PHTF2, DNE
ER2, AMER3, AMPH, ANK2, ANK3, ANKIB1, ANKRD13B, ANKRD44, ANKS1B, APBA2, APC, APC2, APLP1, APOL6, AR
3, GJB2, SUPT6H, SF3A2, PTPRF, CXXC5, GRK4, ADGRG1, WRAP73, SCYL1, CAMKK2, PRSS12, GNMT, IGFBP4, MTF2
1B, GRID2, RAB24, GUCA2A, PITX2, LRRC4, PHOX2B, ANKS1B, LIFR, MYH4, PKN1, PPARGC1A, UNC13D, CD34, HTF
C, EPAS1, CEACAM6, ALDH3A1, FGFR3, S100P, CLMN, ASPH, PTGER4, DUSP4, SLC2A10, CXADR, TNFRSF21, PPARG
P2, ARC, ARHGAP42, ARHGEF4, ARHGEF6, ATP13A4, ATP1B1, ATP1B2, ATP2B2, ADGRB1, ADGRB3, BCAN, BIRC5,
COM, CCSER1, ESRRG, COBLL1, WNK1, ERBB4, XIST, CCDC178, PCDH7, BICC1, NAALADL2, MPPED2, TOX3, FGF13,
H, ANK1, ANK2, ANK3, ANKRD12, ANKRD13B, ANKRD36B, ANKRD55, AP1S2, APLP1, APOL6, APP, ARG2, ARHGAP2
P11A, ARHGAP19, ARHGAP31, ARHGEF2, ARID3A, ARNT2, ARSI, ASF1B, ASPM, ATAD2, ATF3, ATRIP, AURKA, AURK
, KLF12, PTPN22, PIAS1, ZNF182, CCDC25, GLS, VASH2, ZNF367, JAG1, MSL2, COL4A4, CALD1, FGD5, CSNK1G3, TE
LS1, GSN, SLC38A2, RIPPLY3, CAMK2D, MEF2C, NEK1, CUL2, ELOVL7, HMBOX1, FBXO22, TCEA1, MAP3K7, MARCH
RD1, NOTCH3, NKTR, MMP12, LAMA5, CD24, TFF3, NDRG1, SPP1, ANXA5, TLR1, PPBP, MCM6, FABP4, APCS, TM4
-DRA, HLA-DQA1, HLA-DRB1, HLA-F, TLR7, HLA-A, HLA-B, HLA-E, HLA-G, CD83, SLAMF8, RIN2, IL4I1, PRKCD, IL1RN
, POSTN, EDIL3, PPP1R12A, THBS2, IGF2, SFRP2, RARRES2, MMP16, ADAMTS5, PRRX1, P4HB, HOXA7, COL18A1,
BASP1, CROT, ADAMTS9, LRIT1, ORC4, SORCS1, PUM2, ELF2, NR2C2, SDR16C5, ATP6V1A, MBNL1, B3GALT2, GX

N2, CDH11, VIPR1, TMEM150C, DMBX1, SNTG1, VAT1L, WNT1, NKX2-4, GABRG3, FOXQ1, DSCAM, MROH5, SYND
X2-3, BMP6, MIXL1, RAB11FIP4, IL11, SIM2, LAD1, SP8, KCNH1, PTH1R, GABRA5, EVX1, ALDH1A2, EVX2, LRFN5, L
CP1, CXCL14, COPS2, TGIF1, EGR2, ALDOC, CYB5R3, KCNB1, ATP8A1, TFAP2C, SIAH2, PGAP2, P2RX5, GAS1, CCN1,
3, CXXC5, ASPM, KIF20A, BUB1, TOP2A, NDC80, KNL1, CDK1, SYK, TMSB15A, ADA, NUSAP1, CD1E, CKS2, TFDP2, C
OX1, CCT2, AVPR1A, CHD4, HR, PDS5A, CASQ1, FBP2, WDR12, SLC25A13, SIRPA, APP, CAPRIN1, RTP4, KRT16, GSI
MC1, GOLGA6L9, SLC38A2, HOXA13, CEP68, C2CD6, EYA4, NEK1, PALM2AKAP2, KALRN, ZNF32, OXTR, MEI4, GAB
L11A, BHLHE22, BRSK2, BSN, BZW2, C11orf95, C1orf35, C3orf14, CADM1, CADPS, CALB2, CASD1, CBFA2T2, CCBE
NEDD4L, PLAGL1, LYSMD1, COL4A5, WASF3, CCDC120, HTR2A, PAX5, AMER1, ABLIM1, THRB, KLF12, LRCH1, FBX
LPGAT1, HTR2A, LPIN1, TBR1, KLF12, GALK2, UBE4B, EIF3M, DCDC2, ARMC4, BMI1, PRPS2, PEX5L, DOCK5, KIF1C
4L, MYLIP, OXR1, KLF11, WNK3, CXCL6, ZBTB7A, PFKFB3, ANKRD13C, ADARB1, URI1, PHC3, RPS6KA5, SCAMP5, P
1, ANKRD28, RELB, A1CF, RUNX1T1, CCDC198, PLA2G12B, ZDHHC3, PPFIA2, IFI27, NUDT9, SLFN13, GRIP1, ZNF53
MIER3, DGKH, SLC44A1, ASAP2, DCUN1D4, MSANTD4, NSL1, PRPF40A, API5, ATP2C1, ZNF532, PPFIA2, ZNF302, G
MAPK8, ANK3, RPS6KA5, B4GALT6, APAF1, AP1S2, PCMTD1, ROBO2, CCDC198, SEC24B, UBA6, ETNK1, TRABD2B,
A, PDLIM4, ANKRD44, ASAP2, COBLL1, ZSWIM5, PTPN22, CDC42BPA, PAFAH1B2, SLC35F1, REPS2, CTNND1, DDH
TP63, ITPR2, MBNL3, RPAP3, OGT, MYLIP, SSR3, TNFAIP3, STXBP4, CEP290, SLC30A7, DPP10, NDC1, BCL10, ELOC
1, TCEA1, TCF3, OTOGL, CBL, PLAGL1, MYLIP, SSR3, TRAPPC13, SLC22A2, ZADH2, WNK3, TFEC, DNAJB4, ZBTB7A, Z
NMB1, IL17B, CLMP, ADAMTS1, IRX4, DLL1, MYL9, PROS1, CXCL14, COL7A1, IGFBP3, EPHB1, MSRB3, SNAI2, TPM
rf96, PHTF2, CAPRIN2, TGIF1, RAI2, TNPO2, DMXL2, IMPDH1, NRBP1, TENT2, TNFRSF11A, FBXO32, AMMECR1L, E
2, SYNPO2, EBF1, FLNA, ADGRL3, PIP5K1B, FOSB, PRKG1, CALD1, RCAN2, NR4A1, MEIS2, LHFPL6, DMD, MSRB3, N
24A, MOB2, NCKIPSD, ADAM15, MGP, CDCA8, CLN3, VPS37C, RFC3, LOXL2, TOP2A, AMOTL2, PRUNE1, SNX5, PHF
9, ALDH1A3, MMP3, KRT8, PF4V1, OLR1, H2BC6, AQP1, TIMP3, H2AC6, SCG5, ANXA3, ANXA8, SRGN, LGMN, MAC
1, DSCAM, SGK3, CBX5, DUBR, SFXN2, MAPT, AFAP1L2, PPM1K, STC1, MYB, HPCAL1, APTX, CA12, PRSS23, HNM
A, KLF12, CTNNA2, TSPAN9, SNX3, SLC35F1, THUMPD2, GALNT3, XIAP, PRDM5, LAMA3, CR1, MME, TENM1, PPP

1, EPHX1, SNCG, FHL2, PDGFRL, TNC, IGF1, LOXL1, NCAM1, SV2B, ADH1B, IGF2, ABLIM3, PCK1, NEFL, LEPR, LAM
SLC4A2, RARB, WNT6, MAP2K7, NQO2, GTPBP2, PHOX2B, RREB1, MDGA1, FUT11, AIG1, STMN4, BARHL2, EOME
NB, SOX11, MRC1, CHI3L1, SLC13A3, MRPS22, SIDT1, STAT4, MRPS2, ATP2C1, HLF, BTN3A3, ASAP3, HSD17B12, E
1, DLC1, DUSP1, DUT, ECT2, EMP2, ETV5, FGF2, FLNA, FSCN1, FSTL1, GADD45B, GAS6, GGH, GLS, HEXB, HMMR,
GK1, CPN1, RORB, PCDH11X, RAB30, PRKG2, P2RX1, ACTG2, MOS, ANG, ZNF212, ACADSB, DLG3, NCAPH, GP1BA,
KAT2B, HDAC1, RBBP5, KMT2A, NR4A3, NFE2, H2BC11, AGO3, PRMT1, SIN3B, ASH2L, H3-3B, AGO4, THBS1, SETD
, RPLP0, TLK1, FOXO3, STAG2, PHOX2B, ANKS1B, CNTLN, PTPRG, RCOR2, BARHL2, EMX2, LIFR, GLDN, SLF2, KLHL

ATP2B4, APOE, FAP, H2AX, STAG2, FAM122A, PITX2, COL10A1, RPP25, PHOX2B, EFNB3, ZHX1, MYH2, EOMES, PIN
ST7, SLC2A3, GNA13, CYP51A1, BCAT1, DCTN4, HMGCS1, ZNF395, MEIS1, SSR3, AGO2, EGR1, ZIC1, TBC1D30, ALI
REM2, NCR2, MADCAM1, CD40, CD40LG, LILRB1, LILRA1, SIGLEC8, ICAM4, ICAM5, CD33, SIGLEC6, SIGLEC5, LILRB
TGFBR3, PHLDB2, IGFBP3, FCRL6, KLRF1, AGAP1, CD247, SLC4A4, YPEL1, SYNE1, FGFBP2, HOPX, NCR1, SH2D1B,
1, OBSL1, NOG, DNAH5, EPHB3, ITPR1, MBTD1, TCN1, C2CD4A, ACSL3, NAA25, PLA2G7, NPTN-IT1, KDR, CCDC15
rf210, FXYD3, GRHL2, STARD10, GRB7, WWC1, TNK1, IL17RE, STAP2, EHF, PRSS8, SPINT2, RHPN2, CLDN3, CEACA
RCS6, CDH23, CRTAM, NSMCE3, NODAL, MYH2, SIPA1L2, LIG3, KDM5C, PROX1, HEXD, SERPINF1, TAGLN, PCYT2
NA, SCN2B, SPACA7, BARHL2, MYH2, LRRC1, LIFR, MYH4, SMIM29, PKHD1L1, PROX1, MYH8, SSTR3, BACE1, HSD1
CORO1A, CLCN2, SLC9A1, LY6G6D, LILRA2, CCND1, ITGB1BP2, SMIM8, ACRBP, MEIS1, MPL, MRPS14, C3orf52, SI
FBI, IAPP, SEMG1, USP9X, H2BC11, GGA3, APOE, H3-3B, APCS, ITM2B, SORL1, ADAM10, APH1B, MFGE8, FURIN,
GPT1, IGFBP5, TP53I3, MTMR11, RIPOR2, GRIA3, KIAA0930, LINC00461, MGMT, CRIM1, CDH13, TPM2, VWA5A,
TMIE, ZHX1, MYH2, TMEM119, TBR1, LUC7L3, SARS2, PRDM8, PCF11, UHRF1, ZBTB18, BACE2, ZNF384, SMPX, MA
F9, RAF1, MAP2K1, JUN, PRF1, HRAS, IFNA14, CD8B, CD8A, EGR1, FOSL1, NFATC3, FASLG, EOMES, TNF, ELK1, PRI
PI, CPA3, TKTL1, ARG1, FOLR3
G1, PKP4, ZFYVE9, AKAP9, RRAS2, VPS13A, PHF21A, PTPN12, KIF20B, PICALM, ZC3H4, E2F2, CENPA, CEP135, DCU

PT, ADAM10, LGALS3BP, PLAAT4, CUL3, SCN1A, GULP1, PMS1, SMARCA5, SERINC5, EFR3A, CPD, CTSO, LAPTM4B
DN2, PHPT1, RGS18, CTDSPL, PAK1, IQGAP2, CCNYL1, MAOA, KHDRBS3, LTBP1, ATXN10, TNS3, NEDD4L, BCR, LR
RPL31, BUD23, HSD17B10, EXOSC7, EXOSC5, DIS3, RPS5, DIMT1, NOP14, XRN2, RPL6, RPLP0, RPS18, PES1, SNU13
P2, EFNB1, MYL6, EFNA2, MYH9, MMP9, MYL9, MYL12A, ARHGEF7, MYH14, EPHB6, WASL, LIMK1, DNM1, GIT1, E
, MMP10, LIFR, SFXN1, SLC25A32, PARM1, BOD1L1, MIR99AHG, CTTNBP2, WASHC4, NAA15, SYBU, SQLE, GATM,
LAMA1, TGFB1, PTPRS, COMP, SERPINE1, ASPN, COL1A1, VTN, COL9A1, LAMA4, SPARC, ITGB6, FN1, TNR, TGFB3
RELD2, PAPPA2, COL14A1, COL1A2, SOAT1, ACER3, C4A, RPN2, SSR4, SLC46A3, TNC, CLDN3, MRAS, ISLR, COL3A1
S, CCAR1, LRRC4, MYH1, MAGI1, MAP3K21, TTC7B, MYH2, BTBD7, MYH4, TRIL, HNF4G, ADAMTS6, ZBTB18, TADA
2R, REN, SMIM24, CHST2, TYRP1, THBS1, IRX3, SPOCK1, IFIH1, NR4A2, RBM11, SOX4, CCDC71L, ASB2, KLF1, PTGS
2, GATA3, BCL11B, GIMAP7, IL7R, LAT, MAL, GZMK, GZMB, GZMA, GZMM, GZMH, CD27, SIRPG, CD3E, CD3D, ITM
3B, LINC01998, LINC01213, LINC01214, TSC22D2, SERP1, EIF2A, SELENOT, ERICH6, ERICH6-AS1, SIAH2, CLRN1-AS
, ERBB3, PRKCQ, PPP2R5A, FGFR2, FGFR3, PSME4, PPP2R5B, MAPK6, PTPN3, SPTB, FGF10, FGF22, CAMK2A, CDC
RXRA, NLRC4
2, ADAD1, NME8, TFDP3, ARMC5, LRPAP1, GTPBP1, FCGR1A, MTX2, CPN1, ANP32D, MAS1, MPST, NOX1, ADAMT
2L1, GPRASP2, CACNA2D3, LINC00926, TMEM45A, RGS16, BACE2, BCL2, CARS2, GAS2, BEND5, CAMK4, NFE2, UC
3B, ACTG1, TUBA1A, ACTG2, TUBB2B, FN1, CCT5, CCT8, USP22, LPL, MYEF2, NCAM1, MMP14, FABP5, FASN, TBCA
RET, RGS3, TPM1, ADCY1, OGFRL1, SLC27A2, PMP22, GPC4, HGD, GATA4, ARMCX2, CTSA, LAMA4, SLC9A3R1, KRT
N2, SERPINF1, MGP, FBLN1, CFH, COL1A2, PCOLCE, CILP, SCN7A, ABCA10, IGF1, LTBP4, COL6A2, FBLN5, IGFBP6, L
LYZ, MPO, IL32, FLNA, SLC1A4, TYROBP
PTPN1, AKT1, LEP, TYK2, LYN, LAT, YBX1, CSF1R, STAT5A, PDGFB, ITGB3, TRPV6, PIK3R1, SOCS3, BCAR1, CDH2, YE
R135, LUC7L3, ZFHX3, ZBTB18, MNT, STC1, MRPL48, RAB10, HAUS2, AKAP12, ASB11, MYT1, ARHGAP45, CHCHD
IAK1, LAMA3, TACC1, CALD1, ITGA2, MYC, IGF1R, NAP1L1, RRM2, NBN, SF3A2, FN1, AHNAK, THBS1, EGFR, PLEC
LE, MAZ, ESM1, CDC42EP2, NR1H3, GFAP, TDRD3, ATXN3, IFT20, CCND1, TULP3, NTRK2, TRPC2, THBS3, ROM1, E
NT4, SCRT2, SMCR8, IGDCC4, LRP1B, MECP2, C14orf28, SOCS4, ODF3B, TRERF1, NME4, MAP4K3, UHRF2, DOC2A
BP2, MAP2K7, TBPL1, ADGRL1, MYH1, SCN2B, PTBP1, LRRC1, HMG20A, LHFPL6, SCYL3, MYH9, JUP, ADAMTS6, PF
A2, HPGD, PPFIA1, DLX2, HOXD13, PRAME, FDCSP, PABPC4L, MYCN, PCOLCE2, ERP27, LIFR, VASH2, SOSTDC1, SYT
KLRC1, PFN2, ZBTB48, MC1R, TNFSF10, AKR1B1, GAL, KLRK1, IFI6, GP9, TBXAS1, FXR2, H1-10, A2M, IFI27, ITGB5
MB3, TSPAN1, SLC6A14, RHBDL2, MXRA5, LPAR5, CTSE, ADAM8, DTX2, ASPHD2, PLBD1, CYP2S1, FERMT1, ANKR
RARA, H3-3B, CDK4, MYC, LEF1, PML, H2BC1, FLI1, H2BC5, H4C8, RUNX1, IL6R, TAL1, GFI1, H3-3A, H2AZ1, STAT3,
N, CCDC80, BNC2, FAP, TMEM106C, ELOVL2, CAV1, NUPR1, MCM4, MSH2, KNL1, HPRT1, SCARA3, PDGFD, SMC4,

L3, FBLN1, SLC14A1, ROR2, MYOC, CTH, DPP6, SH3BGR, SETBP1, MATN2, HBB, SYNE1, CHRDL1, ALDH7A1, FGL2,
TBD3, PLA2G1B, MACROH2A1, DHX30, ROS1, PRMT3, BHLHE41, TYRO3, ARHGEF38, DAB2IP, MATN4, RARG, ANK
54, SLC8A1-AS1, MTA3, CD8A, CD8B, ANKRD36BP2, LCT-AS1, LINC01934, BOLL, IKZF2, PDCD1, SATB1, SATB1-AS1,
G2, HSPB6, NELL1, ACTA2, SFRP2, NRK, EBF2, PCDH18, FOXF1, C7, SCARA5, SRPX, TAGLN, TMEM119, BMP5, CAP
PKIB, KISS1, LINC00882, ANGPT2, PAPPA2, PSG2, KANK4, RASA1, PSG9, INHBA, EFEMP1, PSG5, PRKCZ, MMP11, PC
T3, TPRG1, CD163L1, CMKLR1, AXL, KCNMA1, CST3, MSR1, CYBB, CTSB, DMXL2, LIPA, MERTK, RAB31, CTSS, RBM4
1-AS, RPH3A, TUBB2B, TUBB2A, ALK, KCNB2, PLXNA4, FAT3, RBFOX1, GREM1, MLLT11, ANKFN1, CNTFR, ELAVL2,
EXN, FLNA, PRKAR2B, RAP1B, CD226, CD84, RAB27B, RGS18, CNST, GP1BA, ZYX, LY6G6F, DGKD, SELP, NRGN, FERI
COL6A1, LUM, COL12A1, CKAP4, DLK1, MXRA5, FBN2, LTBP4, ELN, THBS2, ASPN, FBLN1, OGN, MEST, C1R, PCOLC
, TPSB2, ENTPD1, WARS1, SLC16A4, EPS8, LGALS1, TNFSF15, CD1A, COL3A1, KDELR3, LGALS3, NPC2, RPS6KA2, CD
NK1, KLF1, C17orf99, MYL4, EPB42, FECH, RHCE, SLC25A37, HMBS, SLC25A21, CPOX, ABCB10, UROD, SPTB, TLCD
TYROBP, C1QB, CYBB, CD14, CTSZ, CPVL, TFEC, ADAP2, STAB1, HLA-DMA, MS4A7, FGL2, MARCHF1, CD163, SIGLE
DUSP5, ACTB, EGR1, EIF1, KDM6B, EGR3, NFKBIE, MAN2B1, JUP, PIK3CG, TRAF1, NPPB, EGR2, EEF2, JUNB, NFKBIZ
B1, FRMD4B, CD86, MIR3945HG, CD180, HBEGF, CD14, TIMD4, HK3, AIF1, HLA-DQB1-AS1, AOAH-IT1, NAMPT, W
TYROBP, CD14, MPO, SPI1, PLD4, C1QA, LGMN, MS4A6A, CD163L1, SLCO2B1, CIITA, FGD2, IRF8, HCK, HLA-DRB6,
OL24A1, RUFY1, MMRN1, CXCL3, RBPMS2, DGKG, PPIF, LGALSL, PTGS1, EYS, CNST, MYOM1, PADI4, EFCAB13, GP

IERTK, ADAP2, DMXL2, MS4A6A, ZNF710, HRH1, TIMD4, SAMSN1, SIGLEC1, MKNK1, NABP1, KCNAB1, MCOLN1,
DX2, BLVRB, AHSP, HEMGN, SPTA1, HBZ, ALAS2, TESC, PIP5K1B, XPO7, BSG, SLC4A1, CA8, GYPA, OSBP2, RELN,
TRMT9B, FAM189A2, CTHRC1, GPM6A, RASSF9, TNFRSF11A, ANO9, F8, CEMIP, IRX3, TNFAIP8L3, FZD10, C20orf20
AMTS1, FHL1, THBS1, MFGE8, FRZB, GUCY1A1, CAV1, RERGL, GEM, TIMP3, TINAGL1, MCAM, PDLIM3, RHOB, PP
CSTD2, PIK3C2G, UPK2, GGT6, SCNN1B, SCIN, ELF3, TMEM45B, KRT19, FOXQ1, MACC1, L1CAM, LINC00645, CLD
X1, ABCC9, PCOLCE, LAMC3, LRRC17, PDGFRA, ZNF385D, ADAMTSL3, LOX, CLDN11, ABCA6, COL6A3, EDNRA, PA
1, BLM, BLMH, BUB1, BUB1B, BZW2, BORA, INAVA, SUCO, NREP, HILPDA, TCIM, CALU, CAMSAP2, CAPG, CBX3, C
, MYOZ2, ATP1B4, HJV, SLN, EEF1A2, TNNC2, MYBPH, TNNI1, CKM, IL17B, CAVIN4, FGFRL1, TNNI2, SMYD1, TTN-
RIPOR3, SNCA, SGMS1, BLVRB, AHSP, HECTD4, SLC4A1, SPTA1, PRDX2, ALAS2, SLC2A1, NPL, XPO7, BCL2L1, SPTB
RO3, RARG, CRYAB, RCOR1, EMX2, MYH2, MYH4, SRSF1, ZFHX3, LUC7L3, PPARGC1A, CTNNA3, ADAMTS6, SMPX,
TN1, LIMS1, TLN1, RAB27B, MPIG6B, PKHD1L1, COL24A1, SELP, RGS18, RSU1, CPA3, CMTM6, HPSE, NRGN, CNS
, SCML4, CD7, CD3G, LCK, SAMD3, CD28, STAT4, TRAF3IP3, TXK, CD2, CCR7, SH2D1A, ICOS, CD5, SELL, PTPN7, CD
ERG, HECW2, STAB2, CYYR1, CALCRL, ADGRL4, THSD7A, AFAP1L1, BTNL9, GABBR2, GALNT18, LRIG3, KIAA1671,
LENBP1, PKLR, LINC02772, SPTA1, PIGC, CENPL, TMCC2, DYRK3, CR1L, IBA57, BTNL10, ABCB10, EXO1, TRIM58, N
, CD14, FAM20A, ADAP2, MS4A7, HLA-DPB1, LYZ, HLA-DPA1, FGD2, FMN1, CTSS, C1QC, RTN1, FOLR2, HCK, HPG
G, LCK, SATB1-AS1, RASGRP1, NKG7, LINC01934, CD7, CD52, CCR7, STK17A, STAT4, CD3G, CD79A, IKZF3, PYHIN1
2, KLHL13, ENPP1, LRRIQ1, METTL24, SYNPO2, COL25A1, AGTR1, NKD1, CPA6, PHACTR3, COL8A1, REN, LINC0092
I2, THBS1, HSD17B1, GNG11, ARHGAP25, COL1A1, IL13RA2, PRSS3, ITGB3, SERPINE1, GRIK1, DUSP5, COL6A3, AC
MA1, FGFR3, SFRP5, NRTN, CCDC198, KCNJ16, ANXA4, VEPH1, CFAP221, SHANK2, SCTR, PKHD1, EHF, PLD1, NUAK
PLAC8, FCN1, IFITM3, LSP1, S100A10, ANXA2, HLA-B, EMP3, FGR, NEAT1, IFITM2, COTL1, LILRB2, TIMP1, CD44,
LUD1, AHCYL1, SLC7A11, ID4, SPON1, MT3, ALDOC, MT1E, PMP2, ATP1B2, NDRG2, SLC1A2, PBXIP1, VCAM1, SLC
4, BHLHE22, SH3GL2, PBX3, ADARB2-AS1, GAD2, SLC6A5, ROBO3, MGAT4C, ANO4, LHX5, SLC35F4, SKOR1, GLCE,

1C1, RARRES2, LTBP2, MAF, CNN1, THBS2, FN1, GJA1, RGS5, ITGB5, LRP1, COL1A2, CDKN1A, NOTCH3, IGFBP4, T[illegible]
[illegible]B, MAPT, SNPH, STX1A, MAP2, AMPH, TACC2, PALM, LZTS3, KRT2, KIF2C, SEPTIN4, TUBA4A, KRT8, KRT13, EVPL,
[illegible]1, SLC2A3, TGM2, CHGA, PEG10, ENPP2, MYLK, SGCE, AEBP1, ENG, SELENOP, PDGFRA, PER1, SELE, CFH, COL6A2
MYLK, COL15A1, ENG, FOSB, PDGFRA, PER1, VWF, COL6A2, PLA2G2A, COL4A1, CDKN1C, TBC1D2B, ANXA1, FLN[illegible]

[illegible]2, SOD3, AKAP12, LTBP1, EMP1, IRS1, TRIB2, CACNB3, KCNMB1, PKD1, CDC42EP1, KLF4, FOSL2, MLLT11, PTN, G[illegible]
P4, ZKSCAN3, FBP1, EPCAM, PPL, WFDC2, GPRC5A, MUC1, FOLR1, DEFA3, IL1RN, HPGD, AGR2, CLDN7, ALPL, SC[illegible]
TYRO3, PDGFRA, PER1, VWF, MYC, COL4A1, EPS8, ALDH7A1, ANXA1, FLNA, CITED2, PRSS23, GNG12, ITGA6, CA[illegible]
RX2, AREG, CA2, FBN2, SPRR2C, GJB5, EFNB1, SERPINB2, COL7A1, CALML3, ADRB2, GABRE, KRT13, SPRR1B, LMT[illegible]

PPP6R3, RYR3, MAPK4, VGLL3, DCBLD2, C18orf25, ERBB4, PCDH20, CACNB1, AHCTF1, BCL2L2, NAGK, TMEM50B,
[illegible], DCUN1D5, DIAPH1, HDGF, CACNB2, SORCS1, CHEK1, RPGRIP1L, DAZAP2, EPHB1, KCNMB2, LINGO1, TRIM67, Z[illegible]
11, KIAA1522, ADAMTS6, ADAMTS9, GRB10, DOC2A, ASCC3, ARL8B, ADAMTS3, RAD23B, PLCXD3, RALGPS1, GNA
RC8A, RAB27A, H1-0, WNT10A, PHOX2B, TENT5A, C4A, MYH1, RCOR2, C4B, KLHL32, CRYAB, MYH3, LRRC2, CCN[illegible]
2, PCYT2, CAVIN1, PHLDB3, SIPA1L1, F3, MAPK3, PMP22, ZBTB41, CHCHD4, TOR1AIP1, TMEM43, ARX, PFN1, ARI[illegible]
[illegible]5, ASAP2, ASB8, ATG5, ATP6V1G1, ATP9A, BAX, BAZ2B, BCL2, BCL2L1, BCLAF1, BMI1, BMP6, BMPR2, STAP1, AN[illegible]

[illegible]Y1, PRKACA, PPP1CB, CALM1, PRKACB, PPP1CC, PPP1R1A, PRKACG, ACTB, GRM5, CALM2, MAP2K1, MAP2K2
GB3, ITGA6, PCNA, SERPINH1, DRAP1, EVI2A, CFH, HOXA9, PBX1, NLRP3, CD58, GATA2, CDC42BPA, PKIG, HDGFL[illegible]

[illegible]N1, YWHAZ, CLDN2, CDKN2A, PIK3C2G, TCF7L2, RARG, ESR1, MMP11, SEMA3C, DDR1, MERTK, MYLK, SMAD5, [illegible]
C1, PLK2, TIMP3, ZNF217, TNFRSF4, DBN1, GPR180, IGFBP4, RBPMS, RSL1D1, PXDN, SCGB2A1, ULK1, SLC22A23,
[illegible], FGF9, C2CD4C, LIMS2, OSBP2, VSTM2B, ARFGEF3, ZNF316, PDE1C, SLCO3A1, COL18A1, DPYD, GNAL, CNTN4, S[illegible]
, BAALC, FABP3, KIF21B, BOK, NPDC1, SHC2, SPTAN1, NEO1, TNKS2, OPCML, AK1, MAPRE3, FABP7, PTPRK, FAM1[illegible]
ITGAV, VTN, NOTCH1, AKT1, AGRN, ITGB1, PTK2, MME, TRPC6, CDH2, COL4A4, COL4A3, CTSL, ILK, KIRREL3, PAR[illegible]
MAPK11, GEM, GAL3ST4, F2RL2, ITGB8, AGBL5, VWF, ABCC11, FUBP3, MARCKS, EMCN, DRP2, MAGEA5, CHRDL2
19B, STK40, CITED4, EDN2, JUN, BCL10, GBP3, GBP1, DIPK1A, BCAR3, CNN3, DENND2C, S100A16, ARHGEF2, ME[illegible]

[illegible]L1, PLEKHA7, XPNPEP1, SCYL3, HTR7P1, SLC25A16, PACSIN1, DIAPH2, DHRS3, MTUS1, TRAF5, C3orf70, UGGT2, [illegible]
[illegible]B1BP1, DSPP, DMP1, GLCE, POLI, H3-3B, PER3, NAP1L3, EGR1, PER2, DPP6, CPE, EIF1AX, AOC2, ST8SIA1, NUDT4
SNED1, PTPRM, ST6GALNAC3, DOCK4, EBF1, TGFBR2, PLPP3, HSPA1A, RASAL2, CACNA1C, TMTC1, DNAJB1, PBX1
R, RBM12, DDX5, SINHCAF, NR2C2, GPATCH2, TIMP3, TIMM23B, RHCE, CEP170, CNOT6, TAF2, NANOS1, ZNF236,
PP21, ASIC1, ASIC4, ASXL3, ATCAY, ATP1A3, ATP8A1, AUTS2, B3GALT2, ADGRB3, BASP1, BCL11A, BCL11B, BEND4
[illegible], CPT1A, PINLYP, CRTC3, PLCB1, KMT5A, ATP5IF1, SLC1A5, EGLN3, GIP, WWC1, KRT71, PLOD2, HMGA1, SMARC[illegible]
[illegible]7, NOB1, CYB5D1, AMFR, PRDM1, BIN1, MCTS1, MYO18A, PPP2R3A, RBP3, PLEKHG6, PPT2, LYL1, NECTIN2, HOX
[illegible], LHFPL6, LOXL1, BACE2, GALNT12, KRT81, PKIA, TGFBI, EGLN3, PLPP1, EPHA2, TBC1D8, OPN3, ATXN1, ADGRL2,
[illegible]1orf21, C21orf58, C21orf62, C22orf15, C2orf72, THUMPD2, C6orf118, VXN, C9orf24, CA12, CCDC39, CCDC8, CD[illegible]
DPY19L2, NR3C2, KITLG, GLIS3, LRP1B, ADAMTS9-AS2, BMPR1B, PKHD1, FRAS1, FHOD3, IMMP2L, DCDC2, MID1
[illegible]3, ARHGEF3, ARL8A, ASNS, ATCAY, ATP1A3, ATP2B2, ATP8A1, BASP1, BCL11A, BEX2, BEX5, BIN1, ARMH4, C1orf[illegible]
B, ADGRB2, BARD1, BATF3, BCAS4, BCL11B, BCL2L12, BDKRB1, BIRC5, BLM, BNC2, BRCA1, BRCA2, BUB1, BUB1B,
[illegible]NM1, CLUAP1, KDM6A, CRISPLD1, BCL11B, ENKUR, ADAT2, ZBTB10, UBE2H, NR2C2, PGRMC1, ZBBX, PUM2, PRP
[illegible]F6, NR2F1, STAM, MYLIP, PFKFB3, TNRC6C, MID1, UBE2D3, DDX3X, MAPK8, VIT, SPATA13, HDAC9, MIA2, MLEC,
SF4, SOX9, RGS2, VCAM1, PEA15, PLEK, NUP205, CSN3, GPC3, DYNLT1, B4GALT6, FBLN2, VNN3, PTPRE, LY6E, AN
[illegible], CXCL2, CD86, RGL1, PLXDC2, CD1D, HLA-DMA, METTL7A, FAR2, CYP1B1, HMOX1, RTN1, PTGS2, PLAUR, TNF, N[illegible]
[illegible]OL16A1, MXRA8, MCAM, TMEM217, ACAN
[illegible]LT1, DNAJC22, LYPLA1, ABCD3, RGS17, GTF3C3, CRLF3, ARID5B, OSBPL2, ROR1, NPAS3, RBM27, PPP2R2C, GFRA

)IG1L, GDNF, RBFOX3, PRMT8, PRPH, WNT9B, ARID3C, STBD1, SRCIN1, PHLDA2, MAL, PTF1A, CHRNA4, BIK, LHX6
.HX5, PAX1, SLC6A20, TBX21, RTN4R, SPINT1, LRRC26, GJD2, KCNA7, S1PR5, EIF4E3, CDH1, NPAS2, FAM163B, SLC
, FOXA1, EFEMP1, ID4, HSPA1B, LGALS3, HAL, STC2, FN1, TRPS1, ELOVL6, CDH5, EDNRA, BEX3, GYS2, CD55, SFRP
GINS1, CEP128, PALLD, NEK2, KIF18B, CDCA3, DLGAP5, TTK, GGH, MCM2, RRM2, MELK, NEIL3, CCNB2, UHRF1, CD
K3B, UCP2, BAD, ALAS1, CTDSP2, FGL2, IL2RG, LPIN1, ACTN3, TWSG1, CLDN5, MGAT2, CLCF1, RAB2B, RAB40C, CL
RB3, NEDD4L, BRSK2, TBCK, HOMER1, NCOA5, UTY, ZBTB7A, ANKRD13C, NHSL1, RUFY2, NCOA7, TMEM178B, AN
1, CCSAP, CD2, CD24, CD27-AS1, CDC42EP3, ADA2, TMEM121B, CELF3, CELSR3, CHML, CHN2, CLVS1, CLVS2, CNR
:W7, PDE4A, SAR1A, ST3GAL3, ZDHHC15, MCM8, ARRB1, KCNN3, CD53, SLC11A2, CYSLTR2, ZFAND5, THOC5, PSD
;, DYNC1I2, DRAM2, UBAP1, STEAP2, CYP7A1, KCTD15, PCOLCE2, PAN3, ENKUR, STK26, C3orf35, FUT9, PNN, TAC
'URB, USP28, CHRM2, TSPAN9, VANGL1, EGR2, TGFBR2, DCUN1D1, VASH2, FLT1, LAMA3, ZNF367, LZIC, KAT2B, L
4, ERICH1, EPHA3, PSD3, ZNF326, ZBTB20, SH3D19, UBASH3B, LHX6, SSH2, DSC3, ABR, DIO2, ELAVL4, CRCP, CRA
GDNF, MYB, LDLRAD3, SSH2, REST, PARP14, F13A1, ITM2B, FNIP2, ADAT2, SDCBP, DNAJB14, ARPP19, TBL1XR1, FU
ZNF326, GNGT1, SEPSECS, IPO7, DSP, RUBCNL, ARHGAP12, APPBP2, UHRF1, ARMC8, HELQ, AQP4, DNAJB14, PLC
D2, HTR3D, PLPPR1, TEX11, MIEF2, IGF1R, USP27X, KCTD5, INTS7, C1QTNF9, SYCP1, ERP27, FAM126B, UNG, SGP
:, GALNT18, IRF2BP1, DGKH, PTPRE, C21orf91, PALLD, RPS6KA5, QKI, ASAP2, HSD11B1, ANKRD28, COBLL1, MYL1
ZNF438, SLC30A7, NCAPH2, TMEM178B, RPS6KB1, PIP5K1A, HDAC9, ABI2, DGKH, PHC3, PGBD2, ETS1, CREB3L2,
12, CPNE8, SBSPON, WIF1, TAGLN, LAMA3, MME, FHOD3, PAMR1, SULF1, KRT14, AEBP1, ARC, COL4A2, SLC1A3,
:2F8, MIER1, SATB1, ITGA6, OTUD1, SOGA1, HIPK3, ARAP2, RNF11, SLC4A7, HOMER1, TNFAIP3, TNRC6C, SIPA1L1
MEF2C, CDH13, DLC1, DGKB, ADIRF, CTNNA3, RBMS3, A2M, FOS, RGS6, ATF3, ATP10A, AFF3, GPC6, TMTC1, JUNB
=19, HEYL, PELI1, CCNB1, INSIG1, TTC39A, MTHFD2, CXCL1, DLGAP4, SLC17A5, DEDD, MGAT1, ST3GAL2, TCF25, T
GEA4, H2BC21, CHI3L1
T, STARD13, TMPRSS3, RBM33, LETM2, SYTL4, TMCO6, FRK, SCARB1, KANSL1-AS1, TOP1MT, CDV3, ZNRD2, LINC
4R3A, CDC37L1, KIF23, RBM12, FNIP2, MIER3, AQP4, TMEFF2, PTGDR, WNT2B, CCNG1, PSG5, PDE1C, ATG4A, GG

A4, MARCO, AQP4, HP, CYP27A1, LAMP3, CYP2B6, KLF5, SERPINB3, COL9A3, PLPP2, CHKA, ARSB, ADORA1, NRP1
:S, DLL1, ARNTL, TRERF1, BTBD9, ELL2, ZNF770, ARMH4, CCDC3, HNRNPA1, CBLN2, PRDM9, MSX1, SPAG5, HOXD
:PS15, ELMO1, ANP32C, NOVA2, RBPMS, PTPN14, NUDT11, GTDC1, MYO5C, STAT5A, TAL1, CIR1, HSF4, PGF, NPR
AGFG2, ITGB2, ITGB5, LHFPL6, MARCKS, MDFIC, NDRG1, PDLIM2, PHGDH, PMP22, SCHIP1, CAVIN2, SERPINE1, SG
INPP5E, ROR1, STAC, MAOB, HLA-DPB1, CPM, CHRNA5, ASPA, MYLK, MT3, F10, MAP1B, RANBP3, JMJD1C, KCNA
1B, H2BC1, MOV10, H2BC5, H4C8, RUNX1, DPY30, H3-3A, PF4, H2AZ1, KMT2D, SIN3A, ZFPM1, H2AC6, H2BC4, H
28, DCTN3, FRA10AC1, FAM53B, JUP, AP5M1, CX3CR1, PDGFRA, TMEM178A, SMPX, PRDM1, PIAS1, NEK7, CASZ

M1, MYH4, FRA10AC1, MYH8, LUC7L3, PPARGC1A, SPAG9, MYO18B, ZBTB18, CGN, CORO6, CXCR5, TMEM31, STC
)OC, RB1, UBE2E3, IGFBP2, SMAGP, CDC6, EEF1AKMT3, NPTX2, TBXA2R, NFATC3, GPM6B, DHCR7, TES, FCGR2A,
5, NPDC1, ICAM2, COL1A1, CRTAM, CD81, CLEC2B, KLRB1, ULBP1, TREML2, ITGA4, CD160, PILRB, TREM1, KIR2D
ATP2B4, CST7, PRR5L, MCTP2, KIR3DL1, FRMPD3, SIGLEC17P, NMUR1, GZMB, NKG7, ADCY9, ELOVL6
), IGFBP5, PGAP1, BAMBI, ARMH4, PAK6, MMP13, ANKRD36B, TET1, MCAM, PDK1, MSANTD2, NDRG1, ANK3, M
M1, KCNK1, PPFIBP2, DSG2, DDR1, TFAP2C, ATP6V1C2, SLC5A9, ELF5, EPB41L4B, ACOT1, ILDR1, TMC4, CXADR, N
, SERBP1, PMP22, EIF2AK3, PPP2R3A, DUSP10, CHCHD7, ARIH1, PFN2, DUSP14, PPP2R2B, MED12, C19orf25, TEC
1B1, MCTS1, RALGPS2, KIF4A, OSTF1, QRFP, ZBTB41, DUSP10, CHCHD7, RAB7A, TBC1D8B, ARX, ALCAM, TBXT, PI
H3PXD2A, SERPINF1, PLCG2, BAMBI, CTSG, CD14, SELP, PROS1, ARHGEF3, SAMD14, ILK, RBPMS2, ADRA2A, WASF
APP, HSPG2, RPS27A, NAT8, SNCA, H2BC1, GSN, UBC, UBE2L6, H2BC5, H4C8, TSPAN33, NCSTN, H3-3A, H2AZ1, B2
AP3S2, CTSO, TIMP3, CAV1, COL5A2, POLR3G, BTN3A3, CDKN1A, CEMIP, TMEM187, GPR176, PTEN, GJA4, SEPTI
APK9, FCHSD1, PIAS1, VGLL4, ADAMTS5, TDRD5, QRFP, PDLIM5, KRT20, OTP, ADCYAP1, HESX1, NPHP4, CD68, MY
KCB, MAPK3, STAT4, JUNB, NRAS, IFNAR1, IFNA8, IFNA2, MAP2K2, TNFRSF4, IFNA6, IFNG, IFNA1, MAPK9, IL2RA,

N1D4, CNOT2, CDS2, MED13L, WDR43, IL7R, TGFB2, TRIM33, STAU2, IPO7, C2CD3, EPHA4, MAPK6, MARCHF6, P

, IRF9, TMEM123, STC1, PDS5A, TACC1, EIF2AK2, ARFGEF1, CEBPD, CARD10, BASP1, IFITM3, RABGAP1L, UPF3B,
CH1, ZNF496, RUNX3, PDXP, SDC1, EGFL7, ZNF704, SATB1, RALB, PDIA5, TRAF3, TGM2, GSTM1, PTPN14, NCKAP1
, RPL3, PRORP, NOP56, RIOK3, ERI1, RPS16, FBL, RPS19, RPL18A, DDX49, RBM28, EXOSC3, RPL28, RPL19, FTSJ3,
EFNB3, ARPC3, ACTR3, EPHA4, APH1A, MYL12B, CLTA, EFNB2, ARPC1B, MYH10, EPHB2, MYH11, VAV3, ROCK2, EF
ADA, TGS1, FLRT2, KYNU, GREM1, MCTP1, NASP, PRKD3, SLC44A5, ENPP2, ZNF678, CAPN8, WDFY1, CADPS, MB
, TNN, FMOD, MATN4, NCAN, COL5A1, LAMA5, MATN3, BCAN, LRP4, COL4A2, ITGA7, LAMC1, ITGAV, COL2A1, LU
, NNMT, PLOD3, ITPR3, C1R, DEGS1, TNFRSF11B, LUM, SNORD12C, RBP1, BHLHE41, NFIX, RPL27, MYDGF, MAP4,
1, VGLL3, PPP2R5E, ST18, RAD23B, PLCXD3, ARF6, AMBRA1, SCAF4, GLYR1, SH3PXD2A, DMBX1, ZEB1, TGFBR2,
2, RSAD2, TAL1, GBP1, GAD1, TSC22D1, TGM5, SLC27A6, XAF1, CRISP3, ISYNA1, GPR18, RBPMS, IDO1, FGF18, M
2A, TIGIT, CD2, NKG7, GIMAP6, CCL5, PRKCQ, CD28, CD247, GPR171, LCK, RASGRP1, SIT1, ICOS, RORA, UBASH3A
1, MINDY4B, CLRN1, MED12L, GPR171, P2RY14, GPR87, P2RY13, P2RY12, IGSF10, MIR5186, LINC02066, AADACL
42, PRKACA, FGF4, RASAL2, MARK3, ACTB, DLG1, PAK3, APBB1IP, ACTN2, FGFR1, ARAF, PPP2R5C, FGF20, TNRC6

S13, PLSCR2, IL17A, ZBTB8A, FAM27E5, PSG4, SPRY1, METTL17, SLCO6A1, TRIM42, GKAP1, ARMC3, SSX5, SWI5,
A1, STAR, CDC42EP3, SLC18B1, SLC6A15, PPP1R3C, GABRA5, SERPINB8, HSD17B8, KCNN2, MTURN, ABLIM1, PIP4

T13, CACNA1I, PTP4A1, WNT4, ARHGAP26, H1-2, PFN2, HMGCS2, DGKZ, RAPGEFL1, H4C12, CHST8, H2AC8, ESRR
LAMA2, COL6A1, PDGFRA, ANGPTL1, SFRP4, PLAC9, C1R, LRP1, COL15A1, MMP2, CHRDL1, PODN, HSD11B1, FN1,

S1, INSR, BLK, CSF1, EGF, HCK, NOX4, PRL, PRLR, SHC1, CSK, GRB2, FCGR2A, IRS1, FGR, FYN, LCK, PIK3CA, EGFR
7, ACKR1, ADCYAP1, PRUNE1, DMD, KLHL40, TCF4, NECAP1, NKX2-2, CDC42EP5, PLAG1, ASXL2, KLF7, SMAD6, AS

N2, OLFML2A, CALR, CEP170B, PPP6R2, IFITM2, DLGAP1, NORAD, OSMR, EEA1, TMCO6, PNOC, TJP1, HYAL1, PPP
, RCC2, RIOK3, PPP6R2, VSNL1, COL1A2, PDGFRA, SDCBP, CPD, COL1A1, COL24A1, MIEF1, CDV3, CNIH1, EXOC3L
P6R3, BACE1, VGLL4, PMP22, ADAMTS5, C18orf25, ZNF131, AMBRA1, TAFA4, KCNJ2, SCRIB, TAFA5, ALCAM, TRA
T13, CYP24A1, MAP7D2, SFRP1, SVIP, ADAM23, LTO1, HSPD1, TSPYL5, COL9A2, SLC35F3, ADD2, IL17RD, LGR5, M

D22, MISP, COL11A1, PSCA, ADGRF1, ZBED2, KRT17, CD55, ANLN, KCNK1, CORO2A, EPHX3, ITGB6, KLK7, PPP1R1
, CEBPB, GATA2, H2AC6, H2BC4, H3C13, H2AC20, H2BC21, H2BC13, RXRA, H2AX, H2AC7, H2BU1, H4C3, H3C12, H
RBBP8, PCK2, SLIT2, CDC25B, FBLN1, DCN, AKR1C1, RAD51AP1, GPC6, COL3A1, TTC3, FGF1, PCOLCE, PTX3, IGFB

SH3BP5, CDC42EP2, MT1L, TLE1, GDF10, ALDH4A1, ALDH6A1, WWOX, KIT, DPT, TNXB, GRB14, GNA14, MEIS2, C
S1B, PTPRG, RXRB, BARHL1, TSR1, PPARGC1A, TGIF1, ADAMTS9, UNC13D, DHX29, KRT17, ATP2C1, EFNA5, EPS8L
LZTFL1, CCR9, TRAT1, CD96, IL20RB-AS1, PXYLP1, TFDP2, LETM1, CD38, TBC1D19, RHOH, CYP2U1, LEF1, LEF1-AS
N6, PLAC9, VSTM2A, SHISA3, FOXF2, DPT, ANGPTL6, OSR2, TWIST1, GLIS1, TWIST2, GLYATL2, ITGA11, CNN1, MD
CDH11X, PSG7, RIMKLB, CGB3, PSG1, LINC02055, GDF15, CAP2, CAPN6, ACKR2, HOPX, SPTLC3, PGF, PSG3, SLC6A
7, LGMN, SDC3, SAMHD1, FGD2, MARCHF1, CETP, CPVL, VCAM1, MARCO, IL10RA, CD68, ME1, GFRA2, PDK4, MS
REEP1, CCND1, SLC44A5, MAB21L1, GAL, EYA1, SLC6A2, RYR2, MAP1B, ISL1, BASP1, ST8SIA2, HECW1, BRINP2, K
MT3, F2R, GP6, TAL1, DGKG, RIPOR3, NLK, MPIG6B, RUFY1, TSPAN32, LYL1, F2RL3, MAX, LINC00534, NBEAL2, NT
E, CD248, MFAP5, GPC3, ADAM12, MMP2, CCDC80, PLAT, SFRP2, COL14A1, IGFBP5, MMP14, NTRK2, PLAC9, MG
H3, VCAN, ENDOD1, ALDH3B1, S100B, CXCL8, SDC1, SH2D1A, MRC1, ICAM4, MGRN1, ROR1, ANXA8, NCDN, CY
4, XPO7, RGS6, BLVRB, RIPOR3, GCLC, SLC2A1, TRAK2, PRDX2, SLC25A39, CAT, OSBP2, TFRC, CDC27, TFR2, SNCA,
C1, MMP9, CXCL10, HLA-DQB1, CD163L1, FCGR3A, ICAM1, MAFB, RASSF4, TPRG1, CCL2, MPEG1, VSIG4, IL4I1, S
, RPS9, TRAF4, IER2, JUN, LCP1, ACKR3, PTK2B, MCL1, JUND, MYC, RPS10, GPR183, SERPINF2, HSP90AB1, ICAM1
WP1, CCDC26, MANCR, SRGN, RRP12, VENTX, SPI1, MS4A6A, MS4A4E, MS4A4A, MS4A7, CABP4, JAML, CD163L1
C1QB, ITGAX, CD163, JAML, PRTN3, FLT3, CLEC7A, LRRK1, NAPSB, CD86, MS4A4E, CLEC10A, ADAM28, CCR1, HL
9, EHD3, ST8SIA6, PIP5K1C, INAFM2, PCYT1B, H2BC4, XRCC2, NRGN, GABRE, KIAA0513, PKHD1L1, SEPTIN5, PTCR

CMKLR1, ENPP2, RAB20, LILRB1, IGSF21, EPB41L3, ADGRE2, KCNK13, FMN1, GPR137B, LRRK1, MS4A4E, FLVCR2,
CEH1, CAT, TLCD4, EPB42, SELENBP1, TRAK2, GCLC, TMCC2, MICAL2, FECH, DPF3, RHCE, TENT5C, SLC14A1, GYPB
04, FOXC2, TNFRSF9, BMPER, INO80C, LINC01252, CXCL5, BMP6, TBX1, ELMOD1, LRRC69, ABCA4, ADD3-AS1, GN
P1R14A, ADIRF, HIGD1B, NR2F2, FLNA, EGR1, KLF2, SOD3, PLN, NOTCH3, CSRP1, ACTG2, CRISPLD2, ECRG4, DEPP
N4, MYO3B, KCTD8, SNTG1, UPK3A, PRDM16, ESRP1, ACER2, KRT8, SAMD12, BCAS1, SNCG, SPINK1, TFAP2A, AD
MR1, C7, BICC1, COL1A1, MXRA5, GLI2, COL12A1, GAS2, POSTN, CD248, PCDH18, IGFBP3, FREM1, CCDC80, ACTA
L20, CCT2, CDC25A, CDC25B, CDK1, CDK4, CENPA, CENPE, CENPM, CEP55, CHD7, CKAP4, CKS2, CLIC1, CNOT6, C
AS1, DES, KLHL41, CA3, MYL1, ENO3, TPM2, XIRP1, TMEM182, LMOD3, ACTN2, HSPB6, CMYA5, SYPL2, MYPN, NE
, TMOD1, DMTN, HEMGN, TMCC2, MOB1B, SLC25A21, TRAK2, EPB42, SELENBP1, NCEH1, CAT, TLCD4, RBM38, G
STC1, HAS2, PRDM1, EXT1, MCTS1, PTCH1, RTKN2, CHCHD7, ACKR1, OTP, ANKRD46, APOOL, IL25, DMD, NECAP1
, LY6G6F, PDLIM1, PTGS1, HBD, SLC37A1, GP6, LAT, CLEC1B, TREML1, MYOM1, ELOVL7, RUFY1, FCER1A, CBFA2T
27, LINC01934, GPSM3, SEPTIN1, TBC1D10C, PRKCQ, IL21R, MFNG, RASGRP2, RASAL3, GATA3, FAM78A, GVINP1
GKE, SNCAIP, GREB1L, NOSTRIN, KLHL4, PTCHD4, FAP, CHRNA7, HOXD3, ZNF503-AS1, RGS7BP, CALCR, SLC5A4,
RIR, CMPK2, RSAD2, MBOAT2, RRM2, CAMKMT, HTRA2, FAM178B, MRPS9, STEAP3, GYPC, UGGT1, TRAK2, ABCB
DS, C1QA, CD86, ZNF710, BLNK, MKNK1, LINC00278, SMIM35, SPI1, CD163, MERTK, VSIG4, CYTH4, ITGAM, CR1,
, CD3D, IFNG-AS1, IGLL1, ZAP70, CD79B, CD8A, SH2D1A, SEPTIN1, FCMR, CD5, LINC00861, IL2RB, CD2, DGKA, CD
4, INSYN2A, RARRES1, MYOCD, SLC7A14, PITPNM3, SEZ6L2, NKD2, SHC3, RMDN2-AS1, LINC02720, BDNF, FOXS1
VR1, ITGA2B
2, ELF3, CLDN10, ATP1A1, ADAMTS16, WNK2, TTYH1, IFFO2, GMNN, ACSM3, ATP1B1, RHOV, OLFM4, PAQR5, U
S4A6A, CFP, LGALS1, CRIP1, TKT, S100A11, VIM, ADGRE5, C1orf162, LY6E, UTRN, LILRB3, TNFSF13B, PLAUR, NA
1A4, FGFR3, METTL7A, GRAMD2B, SDC2, F3, TRIL, BMPR1B, NR2F2, RAMP1, MYBPC1, OAT, WLS, SLC3A2, ABCA1,
, CPLX4, CDH22

GFBI, SRGN, TMEM47, PDGFRB, TPM1, FGFR1, EMP3, RAB31, MXRA5, DDR2, FBLN1, MFAP2, GNG11, SEMA3C, CC
MYH7, MID1, LAMA3, KIF23, CENPE, BIRC5, SEPTIN5, KRT17, KRT6B, SH3GL2, ACTR1A, CCNT1, ACTR1B, KIF5A, LA
, PLA2G2A, COL4A1, PPP4R2, TBC1D2B, ALDH7A1, CITED2, AKR1C1, EFS, RARRES2, IL1R1, GNG12, AR, ITGA6, CAV
, CITED2, EFS, RARRES2, IL1R1, LTBP2, FSTL3, CRIM1, MYH11, SRPX, FN1, DHRS3, LAMB2, ITGB5, GPX3, LRP1, CO

DF15, LIPA, CCL21, DNM1, PHLDA2, FKBP1B, FABP4, ADM, CD24, MX1, ADH1A, MAOA, FBLN2, CLDN5, THRA, POI
IN1A, S100P, CEACAM6, TACSTD2, NEBL, CLDN4, CDH1, CNKSR1, LTF, ELF3, KRT14, SLC9A3R1, KRT5, AZGP1, GPX
/2, CRIM1, SRPX, THBS2, SVIL, FN1, UBXN1, GJA1, RGS5, ITGB5, DUSP6, SFRP1, S100A11, CDKN1A, IGFBP4, TGFBI,
K3, PTGS2, MMP14, ANXA8L1, LAMA3, SAA1, TENM2, DMRT2, CLDN8, FXYD3, HAS3, KRT17, KRT6B, KRT1, LAMB3

KBTBD2, PFN2, SH3PXD2A, ZDHHC9, ASXL2, LAPTM4A, KLF12, KLF11, TAOK3, TGFBR2, CELF2, SFMBT1, CAMK2N
EB2, RNF165, DMD, GGT7, NKX2-2, SLC30A7, SGIP1, IQSEC2, DIXDC1, RAB8B, NIN, MIR1915HG, DOCK9, NR4A3, F
I2, PCDH20, CACNB2, AFAP1L2, MYBL2, KCNJ3, SORCS3, SH3RF1, PFN2, SH3PXD2A, SYPL1, GIGYF2, MTCP1, DNM
/, MYH2, LIFR, OLFML2A, MYH4, COLEC12, C3orf36, PROX1, MYH8, CTNNA3, MARK2, SPAG9, SSTR3, AGAP2, ROR
H1, PEA15, SH3RF1, DUSP13, SLURP1, DUSP14, SPATS2, KLHL40, GIGYF2, ARNTL2, PPP2R2B, PPP2R2C, ASXL1, ESI
APC16, C12orf76, JKAMP, RTRAF, IRF2BPL, BBOF1, ADPRM, C1GALT1, C1GALT1C1, C1orf198, ZFAS1, YBEY, MAP3

3, GP1BA, FANCI, MYO16, MANBA, FADS1, PDCD10, RYR3, RUNX1T1, NCAM1, NTRK1, ATXN1, ELL2, PCCB, DLEU1

PLCB1, CCNB2, CYCS, PDGFRB, ITGB5
IL36RN, RBM7, SLK, PRLR, SDHA, NFYC, NLN, PTPRK, SULF1, B4GALT1, NETO2, PTPN3, MCPH1, PIK3CA, HSPA1B, (
PHKAP, GCN1, ANKRD44, ACAP3, TCAF1, ABCC5, STXBP6, BIN1, SLC7A5P2, ADAP1, MAPK11, RIMBP2, FAM89B, A
10B, CTXN1, C12orf57, NBEA, PPP5C, ZSCAN29, KRT31, DCLK2, MAP6, NAV1, MVD, MOAP1, ATP6V0E2, AMER2,
VA, VCL, CD151, KIRREL2, CAMK2B, DNM1, KRT8, NPHS1, MYH9, DKK1, LRP6, MIR6852, LRP5, VIM, INF2, MKI67,
, KIF1B, GABRD, IL1A, ELOVL7, SEMA6C, SLC1A2, TRAF1, HAS2, CATSPERG, PNLIP, KLF5, DOK6, P3H2, TCTN1, RAB
F2D, CD244, XCL2, SLC19A2, SERPINC1, ABL2, ZNF281, DDX59, CSRP1, ARL8A, LAMB3, DUSP10, SDE2, TAF5L, SIPA

DIPK1A, NUPR1, GLRX, HOXB2, PSEN1, SMIM13, FAM149A, KRT81, HACD2, CCPG1, PDE8A, NUCB2, DIDO1, SLC44
, CA2, TRPC1, ATP9A, ATP1A2, PDZD8, OSR2, CRYZ, COL8A2, XIST, PEG10, CALB1, TMEM255A, SPON1, GPM6A, A
, PIP4K2A, ADGRL2, PTPRG, HIVEP3, ASAP1, PITPNC1, PEAK1, FOS, AKAP12, FBXL7, SEC14L1, ANKS1A, AKT3, ARH
LUC7L3, PRELID2, C8orf44-SGK3, MARCHF1, FAM126B, AGO1, TMEM168, FIP1L1, ESR1, IAPP, MAP4K3, MBNL1,
, BHLHE22, BICD1, BSN, TSPOAP1, BZW2, C1orf35, C22orf42, CACNA1G, CACNB3, CACNG2, CADPS, CASKIN1, CAS
02, BMERB1, ATP11A, UBC, EDC4, CLK1, GOLGA4, ENC1, PRELP, CLEC10A, UNC5B, GGT1, MAN2C1, ITPR3, ST14, L
A10, LHX6, HNF1A, MAML3, NTF4, MACO1, EN1, KCNK3, ABCG8, DLX3, SFRP5, ABCG5, KRT72, ECHDC2, MFF, ZNF
GABBR2, PDK4, CAPN2, ENO2, STIP1, PTPRE, SH3GLB2, LRP8, IGF2BP2, FADS2, KRT7, PTMS, CXCR4, ABCC4, CADF
44, CD82, CDCA5, CDH13, CENPF, CENPK, CENPN, CFAP44, CHKA, CHST3, CHST6, COL27A1, COL2A1, COL9A3, CPN
, RAD51B, GRIP1, CDK6, GPC5, AGBL4, DEFB1, MLLT3, DANT2, SPIRE1, ZNF704, TMTC2, FHIT, ATXN1, RHOBTB3, K
5, C1QL1, C22orf42, C2CD4C, COL8A1, CA1, CACNA1E, CACNG2, CADM1, CADPS, CALB2, CAMK2B, CAMK2N1, CA
LCOR, C10orf90, C11orf80, DDIAS, PARPBP, TEX30, TEDC1, KNSTRN, TICRR, USB1, TEDC2, HROB, FAAP24, C1orf1
F39, CCDC88A, SMTNL2, DHFR, CADM2, CIPC, MLLT10, NABP1, DHFR2, CREB1, NANOS1, GRPR, LUC7L3, GGPS1, (
DGKH, B4GALT4, UBE2E2, TERB2, QKI, VPS13C, ABRAXAS1, COBLL1, ARHGEF28, MECOM, PATJ, TCF4, HNRNPR, J
XA1, ANXA2, MFGE8, GATM, LEPR, LPL, RPL36, KIF11, COL4A5, COL4A2, ID1, RAD51B, CD63, COL4A1, TAGLN2, LY
FKBID, FUT7, MAP3K8, SAMSN1, RELB, EBI3, ICAM1, CCL20, PDE4B, GCH1, IFNG, CCL4, TNFAIP6, SERPINB9, IL1B

L, SETD7, BTRC, ARMC8, NAV1, PLEKHG7, KCTD10, GSTM3, BICRAL, ETFA, ASH1L, RNF165, STRBP, KNSTRN, CLASI

, RAP1GAP2, CLSTN2, BNC2, DOCK8, SALL4, PGBD5, AMER3, CITED4, PRDM16, ISL1, STAC2, SLC30A10, SOX18, SL
C30A3, ALX1, LBX1, SULT4A1, LIN28A, NKX6-1, TMEM130, BHLHE23, WNT4, OTOP1, SIX6, EPHA8, MAST1, FOXE3,
1, PDK1, TEAD2, SC5D, BASP1, LIPG, PERP, CUX1, FXYD3, MYH11, COL8A1, TMEM268, C1orf210, KIAA1522, RAB2
D1A, OIP5, GNA15, DNTT, GIHCG, TYMS, SLC1A4, SGO2, MPP1, MKI67, LRR1, MYB, RAD51AP1, HMMR, SCRN1, BI
LDN11, YIPF4, STOM, TSLP, C1QB, EXTL3, ABCE1, KRT79, DDX3X, IKZF1, ERC1, RAMP2, ELOVL6, ERRFI1, LYZ, CES4
IK3, RPS6KB1, HDAC9, PCM1, TSHZ2, ETS1, ROBO1, RAPGEF1, NAA35, PARP11, ETF1, MARCHF7, GCNT1, ZNF345,
R1, CNTN2, CRB1, CRMP1, CSMD3, CSRNP3, CTNNA2, CXADR, DCC, DCLK1, DCX, DLL3, DMRTA2, DNAJB5, DPYSL3,
)3, ADAMTS9, XYLB, REST, METTL8, ARHGAP12, CEACAM1, PRKAB1, SLC35A1, TXNRD1, NRSN1, ARPP19, CPEB2, F
3, CT45A10, TFAM, MYLK, C14orf132, LRIG3, RIMKLB, CACHD1, MARK1, KLHDC1, GRIA4, TRIM65, PLP1, MCU, IRI
MO3, CMTR2, PKD2, MTF1, FBXL3, ZBTB20, CALD1, TMBIM6, FGD5, SSH2, P2RX4, CROT, REST, MKRN1, AKAP11,
CR2A, CAVIN1, IL7R, SEC14L4, EBF1, CADM2, RBM41, ZNF426, HMGXB4, GPR161, GRIA1, NRXN3, NRG3, SPATA31
UT9, SCAPER, DUSP16, ZIC3, SUZ12, CD274, INPP5F, AP1S3, CARMIL1, NABP1, EIF4G2, TAF2, LPIN1, KCNJ13, RPP1
B4, NR2C2, TNKS2, SLC16A7, RBM41, ACOT2, GPM6B, KBTBD3, PKIB, RAP2B, GPR1, HACE1, TVP23A, INPP4A, SH3
L1, DHFR, KLF7, ZBTB33, GPM6B, SPTLC2, NF1, NFATC2IP, CPPED1, BRWD1, GPD1, NPAS3, KATNBL1, TMED10, FA
2A, PCMTD1, ENAH, SLC13A1, LRRC18, FHIT, ZSWIM5, SNX3, KIAA0586, BLCAP, MCMBP, DCUN1D1, TMEM74, CC
HNRNPD, PAX5, AMER1, QKI, DCUN1D4, SMAD3, PURB, FAR2, GLCE, WDR41, PCMTD1, RUNX1T1, ARL13B, HNRN
FST, SEMA5A, TGFB1I1, APOE, ARHGAP25, COL14A1, NTF3, ANGPTL2, ELOVL4, CNN1, FJX1, OXTR, TSHZ2, GYPC, C
., DDX3X, PGR, MAPK8, RALGPS1, RAP1B, RHEBL1, PCDHA10, KPNA3, DICER1, CACNA1C, AKAP1, ZNF518A, RORA,
, HSPA1A, UTRN, INPP4B, CACNB2, MYLK, NR2F2-AS1, HSPA1B, TBX2, AKAP6, EGR1, FBXL7, TIMP3, SLC8A1, SOX5
UBA1A, COL5A2, CDC6, CEBPA, ACTN1, CENPE, GNL3L, APOBEC3B, KIF21B, TMPO, TEAD2, PDXDC1, VASN, ABI3B

)0992, ZBTB10, PGR, SOX4, TPBG, ATP2B4, RMST, IL17RB, LONRF2, SRI, ATP11A, PMAIP1, LARGE1, FYB2, ZNF703,
PS1, MXD1, AMFR, PIEZO2, PCNP, KL, ZFX, MPP1, STXBP5, EGR3, VTI1A, ECT2, SLC16A12, CAAP1, SYT14, GRM5, S

, GPC5, EPN2, CKMT1B, EPHA4, FGFR2, MEIS2, APBB1, GPRC5B, WASF3, FZD7, TRIM9, SLC1A3, LZTS3, NAP1L3, C
)3, PMP22, ASB5, MCTS1, SPTB, ADAMTSL1, DRD3, PKHD1, HOXA11, TEX26, SOX4, KCNJ2, ARX, LHX6, SKIL, IL1RAI
3, KATNB1, RUNX1, RXYLT1, HPGD, YES1, TFPI, SPRY1, BCAT1, DCUN1D4, DAPK1, MAST4, PRKCQ, TRIM24, OPN1
GK1, SH2D4A, SHCBP1, SLIT2, STMN1, TGFB2, TGM2, THBS1, TK1, TNNT2, TNS1, TOP2A, TSPAN3
B1, GREM1, MAPRE3, FCAR, TAL1, PDE6A, SPA17, CA12, MYL2, AGRN, ABLIM1, FXN, HNRNPF
3C13, H2AC20, H2BC21, H2BC13, GP1BA, H2AX, WDR5, H2AC7, H2BU1, H4C3, H3C12, H4C11, H3C4, H4-16, H2BC
L, ACP6, NFKBIA, BCL2L2, CHCHD7, RIMS2, ASB18, DMD, HNF1A, AMD1, PPP2R2B, TCF4, IL1RAPL1, UBE2K, BMP4

C1, NEK7, EMCN, GRK2, CACNB3, ITGBL1, EPHB2, ADCYAP1, A1CF, DMD, NUP54, HNF1A, AMD1, IL1RAPL1, BMP4,
SLC2A5, ATF5, MYO1B, MPI, TBL1XR1, TEX264, CPNE3, GINS4, SP1, C1orf112, ECH1, WBP2, HELLS, GABRA4, MA
L1, C3, HCST, SIGLEC9, NECTIN2, ULBP3, LILRB2, SFTPD, KLRD1, KLRC1, HLA-F, KLRG1, PIANP, COL2A1, ITGB7, SIGI

AGEA3, DENND1B, VWA5A, PHEX, FN1, TENT5C, COL1A1, TMEM30B, PAK3, TMEM45A, RBP4, MLLT3, PDK3, CXC
IRTN, GRHL1, SPTBN2, TMEM79, CCDC120, POF1B, CKMT1A, NECTIN4, CHDH, MYH14, SHROOM3, PTPRF, CELSR2
TA, ESRRG, CLUH, PRPSAP1, NKAIN2, GNGT1, KCNQ1DN, TSGA10, TSGA13, MAB21L1, RERE, OPRM1, FGF5, FGF9
N1, KBTBD2, KBTBD4, INTS9, ZC3H18, GIGYF2, PPP2R2B, SREK1, LAPTM4B, UNC119, GUCY2F, NTF3, ESRRG, CLU
3, FAR2, IRS2, GUCY1B1, USP20, TTC27, EMILIN1, ALOX12, IFI16, TRIM8, ITGB5, CAPN11, COTL1, PSTPIP2, ANGP
M, TSPAN5, UBB, FGA, PRL, SAA1, NPPA, H2AC6, H2BC4, SIAH2, H3C13, H2AC20, H2BC21, H2BC13, PRKN, BACE1
11, DNMBP, ALDH1A1, RIN2, HOXA9, EXT1, RECK, ANXA4, PRKCA, PKLR, KIT, NIPAL3, SEMA3C, GRIK2, SSPN, CFL
YO15A, DMD, ST3GAL5, CDC42EP3, SUGP2, BMP4, CYP46A1, ANKMY2, ESRRG, EN1, EN2, SMAD1, GH1, DLX1, TRI
HLA-A, IFNA4, NFATC2, BRAF, MAPK8, IL2RG, PTPN7, IL2, IFNA21, IFNA10, IFNA7, PPP3CA, PRKCE, IFNA5, CD3G, C

RR3, TAB2, MARK3, TSPAN5, BPTF, DENND5A, UBR2, SAMD4A, MTX2, DNMBP, MBD2, BUB1B, SRSF3, CAMSAP2,

COL3A1, IL6ST, PRNP, IGFBP5, RBPJ, FRYL, CLGN, CXCR4, ELOVL2, HMGCS1, NFU1, AP1AR, RBMS1, DSP, CD302, SI
L, PTPN3, ARHGAP15, PLCB4, HOOK1, TSPAN6, CMTM7, ITPR1, MOB3A, CAVIN2, GRB10, RGCC, PDGFD, DACH2,
UTP6, RPL34, GAR1, RPS13, KRR1, NOP2, RPS12, BYSL, RPL24, RRP9, NCL, RPS15, WDR75, PNO1, RPL22, EBNA1BP
PHA7, RAC1, SDCBP, ADAM10, ACTR2, APH1B, CLTC, EPHA2, EFNA3, PSEN2, EPHA5, RASA1, EPHA1, PAK1, EPHB1,
NL2, RPE, FYB2, CDKAL1, MEGF10, PTAR1, FOXP2, ABCA17P, IL15, NDUFS1, C2CD4A, TTC3, PPIG, SFRP2, CALB1, T
JM, ITGAX, COL6A1, COL6A2, APP, HSPG2, ITGA9, HAPLN1, NCAM1, ITGB1, DSPP, DMP1, ACAN, MATN1, COL6A3
, C1QB, TXNDC5, PDIA4, PGM2L1, ANXA2P3, TBCB, RNF213, KCND3, FEZ2, C3, VCAM1, TRIM47, CFB, TIMP2, ALPK
CELF2, ATP11B, KCNK3, PCDH19, NKAIN2, FANCI, ZBTB44, PKP4, TGFBR3, NACC1, MARCKS, TNK2, NACC2, MAB21
K1, RHOBTB1, OAS1, IFIT2, IGLL1, HOXA9, IL22RA2, DHRS3, MYCT1, HOXB-AS3, DLC1, SDSL, OSBPL6, USP18, MEIS
A, ZAP70, ETS1, EOMES, ITK, NLRC3
L2, AADACL2-AS1, AADACP1, AADAC, SUCNR1, MBNL1, MBNL1-AS1, TMEM14EP, P2RY1, RAP2B, LINC02006, ARH
C, CDC14A, CDC14B, DLG3, XPO1, PSMC5, MMP2, MAPKAPK5, PEBP1, BRAP, FLT3LG, TNRC6A, IL5RA, PSME1, AG

RSU1, FTMT, GRAMD1C, LCN1, MKRN3, ASPN, TRMT12, HDAC11, EGFLAM, PPARD, NUP35, SPAG11B, TRIM49, ZI
P2, SYDE2, SH3D19, CCDC26, ACAP1, BFSP2-AS1, C1orf115, C10orf95, ASNS, TEX15, BPI, CCL5, MS4A3, VKORC1L

G, ETFB, ASB13, SLCO2A1, COLEC12, TNIK, SEC61A1, AMBP, SERPINA1, GP2, MSN, FGR, C4A, STARD8, SRPX, WDR
ISLR, MFAP5, CCDC80, SERPING1, PGF, ABCA9, MME, NOVA1, COL3A1, CST3, PMP22, CCN5, TNXB, TIMP1, MXRA

XL1, WAPL, GARS1, EN1, ZNF521, PLXNA2, ITIH6, RGS3, NTRK2, SUPT16H, MARCKS, TCF12, SOBP, JPH4, BACH2, R

2R3A, ABCB6, ACY1, TGFBI, BTN3A2, PTGDR2, CDH6, CAPNS1, MAP1B, STK17A, TNFSF11, MBOAT7, MAP2K6, PO
1, PAQR3, LIN28B, NR2C2, HOXA1, PPP1R16B, HOXA5, DENND6A, HOXA7, DCAF15, PCYT1B, DNAAF3, SLC31A1, M
A2B, HNF1B, ARHGAP39, RANBP17, KLF12, ASXL1, ACACA, ATP11A, PCNP, KCNK4, FNBP1, BTBD10, SMURF2, MAP
YB, FRZB, ADGRE1, LEMD1, CLDN3, PTX3, PLCB4, SYNPO2, COLCA2, CRABP1, PENK, PEG10, PCDHB2, CENPV, FGF

B, NRP2, PLEK2, C15orf48, GJB5, CEMIP, FA2H, VDR, AP1S3, PTPRR, SLC4A11, DAPP1, MYEOV, USP54, SLC16A3, G
H4C11, H3C4, H4-16, H2BC12, TFDP1, H2AC18, H2BC15, CEBPA, H2AJ, H4C15, H4C14, H2AC19, H4C12, H2BC14, H
P3, CCBE1, VAT1L, NREP, ADGRG6, IQGAP3, PCDH7, NFXL1, SULF1, EZR, TIMELESS, CHAC2, CA12, CCNB1, PSIP1, N

OL9A3, EMCN, MT1H, RPS6KA5, FXYD6, AKR1C1, KCNAB1, GSTM3, GHR, FLRT2, LPAR1, RYR2, HGD, DNASE1L3, EP
L2, SLC2A12, ZC3H10, PTCH1, ITGA10, CDK2AP2, SLC29A1, TCF21, ZBTB37, ANKRD30BP2, DMD, STX17, CLEC4D, N
S1, TNIP3, LINC02273, NEIL3, LINC02059, CHD1, CAMK4, CCNI2, TCF7, MZB1, LINC02520, CCND3, PTCRA, ELOVL4,
FI, ANGPTL1, INHBA, PTGER1, TRPA1, VSTM2A-OT1, PI16
4, KRT8, STS, ERRFI1, EXPH5, ANXA3, CHODL, PAGE4, KMO, SDC1, PSG4, HSD11B2, L1TD1, MET, GYG2P1, PSG6, S
S4A4E, TYROBP, CFP, FCGR3A, FMN1, MANCR, LILRB1, EPB41L3, AGBL4, KCNK13, SCIMP, MCOLN1, IGSF21, NABP
CNK10, KCNG1, TRIM67, ONECUT2, CADM3, SV2C, HAND1, DUSP8, INSM2, TTC9B, PIRT, LINC01201, TMEM35A, F
T5C3A, LAT, MPP1, LGALSL, EGF, ENDOD1, TREML1, BMP6, CTSA, CLEC1B, KIAA0513, TMEM91, SRC, LINC02284, T
P, BGN, LOX, SERPING1, MRC2, OLFML3, PRRX1, TENT5A, SRPX, PI16, SCARA5, LRP1, COL6A6, ADAMTSL3, TIMP1
IP2, MSMO1, ACOT7, SNX1, CYP1B1, ALOX15B, TGM2, CAPG, CLDN9, NT5E, CDKN1A, ITGB7, PRDM1, PLD3, ST14,
NFE2, TRIM58, TMCC2, TAL1, RHD, KEL, TSPAN32, E2F2, EIF2AK1, GATA1, PIP5K1B, ERMAP, MAP2K3, RNF224, S
LC7A7, HCK, FOLR2, FPR3, HPGDS, CIITA, MS4A6A, GPR141, ACP5, CD86, CXCL16, CD83, THEMIS2, CLEC7A, IGSF6,
, HERPUD1, NFKB2, CD69, TNFSF9, KLF6, RPS16, NFKBIA, PPP1R15A, ZC3H12A, BIRC3, CD83, INSIG1, BTG2, SEC24
L, CD163, CLEC4A, CLEC7A, CMKLR1, SH2B3, SDS, RILPL2, GPR183, RNASE6, SNX6, LGMN, DMXL2, PYCARD, SCIMP
A-DMB, VSIG4, SMIM35, CCDC26, MNDA, SIGLEC1, MS4A4A, C3AR1, SIRPB2, IGSF6, NCF2, AZU1, MMP9, LINC002
A, EXOSC5, CENPS, SUCNR1, TNNI3, XYLT2, EFHC2, CMTM5, EGF, HPSE, KLF9, ZNF32, LINC02284, INTS5, ADRA2A,

LILRB5, CD86, IL10RA, CLNK, RAB39A, S100Z, ITGAX, MEGF11, CD200R1, SMIM35, FAM20A, RGL3, RASSF4, LYZ, L
, MAP2K3, MARCHF8, TANGO2, HMBS, C17orf99, ABCB10, ERMAP, MYL4, SLC25A39, DCAF12, TRIM58, FHDC1, T
A14-AS1, DHODH, SLC30A3, PDF, TRIM54, KCNJ2-AS1, LINC00636, GPR182, GABRP, MYCBPAP, CCL20, DDX59-AS1
1, ARID5B, SPARC, ATF3, LHFPL6, MAP1B, ID4, ISYNA1, CAVIN3, IGFBP7, SEPTIN7, STEAP4, SOCS3, LMOD1, MT2A,
GRF1, PDE7B, ALDH1A1, RDH10, C4orf19, MAL2, GRHL2, HPGD, SHROOM1, SPINT1, LYPD6B, LINC00511, MAP3K2
A2, CDH11, ISLR, PRICKLE1, FNDC1, PRELP, C1R, PTH1R, PDE1A, ABI3BP, THBS2, ZFPM2-AS1, TAGLN, TWIST2, TBX1
OCH, COL1A1, COL1A2, COL4A1, COL5A2, CORO1C, CSE1L, CSRNP2, CTSC, DAB2, DDX39A, DKC1, DKK1, DLG5, DL
EB, MYOM3, CAV3, TXLNB, SRL, UNC45B, ACTC1, PTGFR, PRKAA2, TNNT3, AK1, FHL1, NNAT, BIN1, AGL, CFL2, TNN
YPA, FAM117A, PIM1, HMBS, TANGO2, HBZ, RHCE, RHAG, SLC25A39, SLC1A5, ART4, UBE2O, TFR2, FECH, ERMAP
L, IL1RAPL1, SREK1, KLF5, PLAG1, KLF7, CYP46A1, NIM1K, ACACA, LINC00314, RALYL, SEMA6A, NOTCH2, CYP17A1
T3, ADCY6, F2RL3, THCAT158, LINC00534, EGF, PROSER2, TMEM91, LINC00504, SERPINB1, LGALSL, SLC8A3, ALOX
., TRAT1, ZAP70, KLRB1, MATK, NKG7, TMIGD2, AGAP2, EPHA1-AS1, LAT, PAX5, PCED1B-AS1, SIRPG, CD79B, MS4
UNC80, GCOM1, TMEM233, PROX1-AS1, LINC01594, LINC02160, LINC02356, RFX8, MIR34AHG, ODF3L2, LINC015
6, HJURP, SEC22C, RNF123, CPOX, RSRC1, SEC62, NCEH1, DCUN1D1, TFRC, NCAPG, LINC02506, THEGL, MOB1B, S
IL10RA, SIGLEC1, HLA-DMA, PLD4, KYNU, MS4A6A, CABLES1, LY86, CD200R1, LILRB5, GLIPR1, MS4A4E, IRF8, TLR1
27, KLRB1, TRAT1, TBC1D10C, JCHAIN, BLK, GZMA, LY9, KLRC4-KLRK1, PCED1B-AS1, SIRPG, SLAMF6, FCRL5, GPR1
, TYRP1, IGLON5, TBX22, SLC6A1, LINC01914, PGM5P4, CGB7, PITX3, FGF19, ELOVL3, NDP, LINC02319, GNG8, GC

PK1B, ATP12A, LAD1, PLPP2, TMEM72, CLDN9, HOGA1, LHFPL3-AS2, OGDHL, NCMAP, ADORA1, HMGA2-AS1, ANG
PSB, SERPINA1, CARD16, SPI1, PLXNB2, KLF2, POU2F2, KLF4, CEBPB, TNFAIP2, SECTM1, DOK2, HLA-A, IRF1, APOB
, SLC15A2, ADIRF, SERPINE2, PTTG1IP, AIFM3, ASPH, NR2F1, HACD3, ADGRG1, CAMK2G, PLTP, DST, EZR, SPOCK2,

)L6A1, CFHR1, IL6, SERPINE1, CLEC3B, WWTR1, SMTN, G0S2, IGFBP3, GAS1, EDNRA, FBN1, LAMB1, CXCL12, CRYA
MC2, TUBB4A, CYTH1, VAMP2, TRIM13, NUMA1, KLC1, PKP4, HIP1
/2, CRIM1, ECM2, SLPI, MYH11, SRPX, FN1, DHRS3, UBXN1, LAMB2, ITGB5, GPX3, ACKR1, DUSP6, SFRP1, LRP1, SF
L1A2, S100A11, CDKN1A, NOTCH3, IGFBP4, TGFBI, GADD45G, PDGFRB, TPM1, FGFR1, EMP3, RAB31, FBLN1, MFA

N2, ARHGDIG, ASAH1, TLE1, MMD, KRT7, MEST, EPHA2, UCK2, PLOD2, THBS1, ANPEP, MMP1, KRT18, DTYMK, RR
2, PRSS8, DSP, B3GNT3, KRT6A, REG1B, CD4, TAGLN3, CDKN2A, OCLN, LAD1, APOA1, HOOK1, C4BPB, C4B, CLU, C
SRGN, TPM1, FGFR1, SOX4, EMP3, FAT1, MFAP2, IER3, GNG11, SEMA3C, TNFRSF10B, CAV1, IFIT1, TRAM2, CD15
3, FGFBP1, FASN, F2RL1, CDH3, KLK10, TFPI2, LAMC2, COL17A1, EIF2B3, ANGPTL4, PGAP4, PTHLH, LIPG, PPP1R14

2, MIER3, ZNF217, BTBD10, MAP4, MAP7, MAB21L1, ZNFX1, SOBP, TSG101, FGF9, NBEA, RTN1, RTN2, KDM2A, C
URIN, NKAIN2, PCNX2, SALL1, TRPS1, RFX1, MFF, CREBRF, SCN8A, MAB21L1, CHRD, PHF6, NRP2, TSHZ3, ARID4B,
IT3A, AQP4-AS1, TAOK1, KLF10, CELF2, PCDH10, ESRRG, PCDH17, CAMK2N2, KIAA2026, SYNGR3, PCDH19, MIER3
1, PPP2R5E, EMCN, ERBB2, CACNB2, NDUFA13, CACNB3, KY, ADCYAP1, ATP6V0C, ARIH1, NPHP4, MUSK, NECTIN
RRG, KRT78, DUSP22, ATP11C, LRRC32, COL16A1, ARHGAP32, GGN, PRRG4, GNGT2, KCNQ1DN, RGS2, ARF1, ASS1
K7CL, MMADHC, DIPK2A, GASK1B, HPF1, FAM172A, FAXDC2, CREBRF, C5orf51, C6orf62, MTURN, MSANTD3, CAE

, MPPE1, SC5D, TMSB15A, NUP50, MYH10, ORAI2, DDHD2, UBXN8, AP1S2, DDB1, GOLGA8A, ITGA2B, RAB27B, M

GSTM3, CD44, ELOVL2, CA2, SEMA3C, MAGED2, EFNA5, JAK1, NPHS2, CHD6, RAB31, NUMA1, UGDH, GP9, COQ4,
RPP21, SLC25A29, CLVS2, PDIA2, SYT13, SRCIN1, EPB41L1, CACNA1E, NEXMIF, PPFIA2, CARMIL3, SEMA3C, STX1A
SUZ12P1, DAAM1, NGRN, CHRNA4, ATXN2, PCBP4, SDC3, AKT2, LSM14B, IMPDH1, LRRN3, GCSH, PFN2, ARL4C, S
JAG1, WNT1, FYN, PLCE1, CD80, COL4A5, IRF6, ACTN4, PTPRO, PLAUR, LIMS1, DAG1, NPHS2
39A, PTX3, EMP2, PDGFB, HAGLROS, NEDD4L, STMN4, NKAIN3, MIIP, AFF4, NBN, PNLIPRP1, GAD2, ARL4D, B4GA
1L2, IRF2BP2, LYST, AHCTF1, KIAA1217, ARID5B, REEP3, DDIT4, PLAU, VCL, STAMBPL1, IFIT5, ENTPD7, SLK, ABRA

A2, TMEM39A, IFT57, SH3BP4, CEACAM6, DRAM1, BZW1, CRYM, DOCK5, MPZL2, H3-3B, SHISA9, RASGEF1A, IL1
DIRF, KIF5C, OLFML1, GPR37, CDH12, CYFIP2, ZNF423, LINC00667, ABLIM1, SPOCK3, PGAP1, NBPF10, TRIM2, PT
GAP26, ST6GAL1, KALRN, LIMCH1, PPFIBP1, SPTBN1, GNAQ, LIFR, TACC1, ARGLU1, PRKCA, APBB2, ITGA1, PRKG1,
CBFB, USP25, CNDP2, TNRC6B, AGPS, ARFGEF3, NFIB, FSD1L, BCL11A, GORAB, SYNM, INTS6, LEPR, DCLRE1B, AC
Z1, CBLN4, CCDC112, CCDC85A, CCDC85B, CD2, CD24, CDH13, CDH4, CDK5R1, TMEM121B, CELF3, CELF4, CELF5,
GR5, NCDN, TJP2, FHDC1, POLR2L, ATP5PD, SPINT1, TFDP1, DMD, SOX2, RPL23, RRAD, CUX1, WRN, CD248, SPES
F219, BMP6, NRP2, KCNJ15, SCHIP1, ELF1, ACVRL1, AP1B1, SUSD1, USH1G, CPEB4, SYNJ1, MITF, GPLD1, PMS2P5,
S2, AQP3, AKR1C3, DBNDD2, SLC6A6, GNE, ARHGDIB, LIMCH1, MALL, CCNJL, WNT11, TTC9, INAVA, MYO1D, SLC
NE5, CREB5, CRTAP, CSGALNACT1, CSMD1, CSMD2, CSMD3, CSPG5, CST3, CTNNA3, CTNND2, CYP26B1, DBI, DCLK
LF12, ATP1B1, NFE2L2, MAP4K3, SLIT2, CSGALNACT1, GAREM1, MITF, TRHDE, PPARGC1A, SETBP1, PCDH9, PTPN
SZ1, CBLN2, CCDC136, CCSER1, CD2, CD24, CD27-AS1, CDC42EP3, CDH13, CDH4, CDK5R1, ADA2, TMEM121B, CE
12, AUNIP, STPG1, CCSAP, C20orf27, C21orf58, TRABD2A, C4orf46, C6orf99, ARHGEF39, AOPEP, C9orf40, CACYB
CDH13, ZFX, FBXO34, TRAPPC11, MYT1, SMG7, TMEM47, CUL4B, TMEM168, FIP1L1, SS18L1, FBXO32, IL17B, USP
AK2, ATF2, C1orf210, CCDC90B, SNX3, SPOCK3, ZNF532, C6orf62, UBA6, DCUN1D1, PTBP2, AKAP12, LAMP2, PHA
/6D

'1, TCF21, TLK1, LCLAT1, ZBTB18, SCIMP, WEE1, GPD1L, PNISR, B4GALT5, MOB4, RBM24, GATM, KCNA4, FOXO1,

JLT4A1, OCLN, SORCS3, ALDH1A2, CACNA1I, SLC30A3, RET, NKX2-1, RAB11FIP1, ASCL2, KCNC4, SFRP1, COLEC12,
CLDN7, LRAT, OPRD1, HOXB2, SLC12A5, PYY, SOX18, PITX1, HOXB7, PTPRN, GRIK3, FAM43B, UCN, PDX1, CA10, I
.7B, UNC119, C20orf27, BMP1, GATA3, LCN2, TSPAN8, TLCD4, RNASE2, DLAT, BTN1A1, WWC1, PTPN14, TMPRSS
RC5, DIAPH3, CEP55, GFI1, HHIP, UBE2C, PON1, NSD2, CDCA2, BUB1B, ATAD2, FANCI, DTL, CD1B, E2F2, AEBP1, C
A, RAD21, ACSL1, WWC1, ADD3, CEP85, SMAD5, IMMT, BABAM2, ACOX1, CALD1, IDE, HCFC1, PCOLCE, HIPK3, FC
THRB, SGCZ, ANKRD50, PRPF40A, COBLL1, B3GALNT1, PCMTD1, RUNX1T1, CHD9, KLF12, TCF4, ELMO1, SLC13A1
DPYSL4, DPYSL5, DSCAML1, DUSP26, EBF1, EBF2, EBF3, EDIL3, EFS, ELAVL2, ELAVL3, ELAVL4, EN2, EPB41, FAM2
PUM2, SLC16A7, AKTIP, IKZF2, SEPTIN9, CADPS2, EDA2R, ESF1, PDLIM5, PDE1C, ACTN1, OPRM1, EYA2, USP33, TN
F2BPL, TOGARAM1, EML1, MINAR1, SLC24A4, AGPS, ADRA2B, SH2D1A, BAZ1A, ABCC2, SLITRK5, RBM41, PEG10,
CDC37L1, U2SURP, DENND10, ATG16L1, HBP1, FRS2, ARHGAP12, BCL11B, SMAD5, PTPRD, TMX3, TAFA1, FYCO1,
LC1, OPRM1, ANKRD17, UGT2A1, NDRG2, ZKSCAN1, MRE11, TRAF3, ZNF254, SLC22A5, CER1, RAP1GAP2, PLOD2,
L4, TES, RNF170, TFCP2, HNRNPK, ITGB6, STXBP5, AGO1, RAP2C, PDE4D, CP, ROR1, DET1, TAF9B, STXBP5L, NRXN
3GL3, CNKSR2, C2orf83, CLNS1A, TBX3, MAGEE2, CDK1, RABGGTB, ZNF567, PDE4D, SGIP1, PRR16, RPRD1A, PAK5
AM133B, BBX, GASK1A, KIF3A, WASHC4, EGLN3, CCNT2, CDYL2, MSRB3, PHACTR2, FAM133A, ARRDC3, WNK1, R
CDC47, ETNK1, SLC11A2, TBC1D15, RHOA, KCNH5, FBXL3, SFXN1, EPHA3, BCOR, SORBS2, PRPSAP2, PLPPR1, EDN
IPF, STAT1, SC5D, FUCA2, COX20, MOB1B, TRPS1, C1orf198, ZDHHC15, GRIA1, DSCC1, DCUN1D1, PCDH11Y, VASH
OL18A1, NUDT11, PPP1R14A, TRO, COL4A1, POSTN, DPYSL3, HS3ST3A1, LHFPL6, TNS4, CALD1, NRG1, SYNM, SC
, C2CD5, MAP3K12, ACBD5, ZDHHC18, KCNJ2, CNTFR, ELOVL5, RPS6KA5, QKI, ASAP2, ZDHHC23, FAM234A, WDR
5, FRY, PPP1R12B, PLCL1, ZFP36, PPP1R12A, EPS8, SDK1, LPP, ZFHX3, EPAS1, MYO1D, DNAJB1, KALRN, NRXN3, TE
P, ARRB2, HCLS1, SAE1, COL15A1, TNFAIP1, HSD3B7, MCM6, OR4F16, EPHX1, KLHL6, MINK1, EFNA1, TEX30, PMS

, RAB31, IGSF1, SH3BGR, TMEM164, GFRA1, DUXAP8, BLVRA, DUSP2, SEMA4C, NEIL2, HNRNPA0, PPP1R26-AS1,
STXBP5L, RB1CC1, HS3ST2, GUCY1A2, FAM217B, CAMSAP2, DCTN5, C1orf21, CCNT2, TLCD3A, TTLL2, C2orf69, RC

OL11A1, UGT8, NRG1, FGF6, COMP, GULP1, PAPSS2, GH2, FETUB, SLC22A1, SLC10A1, SLC22A18, KCND1, BBOX1,
PL1, GAD1, NKX2-2, AFF3, ESRRA, KMT2A, FSCN2, MAML2, SLC6A14, PCDH12, EN1, SMAD1, NXF1, THUMPD2, KC
SW, CFAP410, NAA16, LAMC3, EGFL7, KMT2A, ATP2A3, KIR3DL2, AGRN, ZMYND8, ST18, TPSAB1, FCER1A, NIT2,

C12, PRMT6, H2AC18, H2BC15, H2AJ, H4C15, H4C14, H2AC19, KMT2B, H4C12, H2BC14, H2BC8, H3C8, H2AB1, H2
, PLAG1, KLF12, PAIP2, TP53BP1, C2orf73, PLXNA2, EN1, SELENON, UNC45A, DLX2, DLX1, TEX264, FNBP4, TMOD

, KLF5, TECTA, GPR157, RNF43, NDFIP1, GPR150, NXF1, DDX5, SHOX2, ITIH6, KRT73, DLX1, SYNE2, KCNJ8, ATP2A
CROD1, CDT1, ORC1, SAR1A, NPY, ALDH1A3, RFWD3, TOMM34, SLC38A1, PLK4, RNF144A, CDC45, MVD, GPATCH
LEC10, KLRF1, ITGB1, FCGR1A, CD226, CD96, CD8A, CXADR, COLEC12, CD1D, CD1A, CD1C, CD1B, ITGB2, JAML, CD

L16, C5, SPARC, SRGAP3, PPP1R3B, LINC00907, TP53INP1, IREB2, TRABD2B, PCSK1N, CLIP1, ARID2, TSPAN31, TB
, MAP7, AP1M2, KDF1, LSR, PATJ, TMEM30B, LRBA, ELMO3, EPS8L2, FAM83H, FZD6, CRB3, ZNF185, MPZL2, RIP
, ELF4, DPP10, NBEA, MECOM, MAP1LC3A, SNPH, FOXB1, AMY2A, MRPL2, RAP2A, KDELR2, HYAL2, CERCAM, PIK
H, LINC01089, ZNF521, XRCC6, ZC3H11A, NXF1, AGFG2, GAS7, TONSL, KCNQ1DN, ASS1, RGS3, EMC10, WBP2NL,
T1, CGRRF1, PPIF, NOTCH1, WFDC1, LOXL3, IL7, DOK3, PF4V1, EFNB2, CD84, ARMCX5, ASAP2, CD93, C6orf226, Z
, H2AX, SIAH1, H2AC7, H2BU1, H4C3, H3C12, H4C11, H3C4, H4-16, H2BC12, H2AC18, PSENEN, UBA52, H2BC15,
AR, DDAH1, PDLIM7, COL8A1, MYH8, CCN3, B9D1, ABCA8, BNIP3L, IFITM1, COL6A3, OAZ1, SPAG5, DDX17, ATP5
PS1, PKP4, MAP2, HMCES, GRK6, TMCC2, ZNFX1, RTN4, CSH1, CSH2, NRP1, SEPHS1, MYBPC1, FGF9, LINC00052,
GZMB, IFNA17, IL2RB, PRKCA, PPP3CB

PDE8A, SLC25A13, PIK3C3, AUH, RAB6A, UTP18, KIF2A, FGF5, ZNF638, RAD23B, KAT6B, PPM1B, ASAP2, TRIO, TC

LC30A9, VPS13B, PAPOLA, ZFP36L1, PLCB1, SYBU, DNAJC15, DLG1, ZNF217, UGT1A10, CYP1B1, RAI14, SLBP, CREI
CTBP2, ARRDC4, PHGDH, HPCAL1, CHM, CNN3, F5, REPIN1, SLC4A7, CTPS2, DNMT3B, MACROH2A1, NKG7, FCHSI
P2, NSUN4, UTP25, RPS25, HEATR1, FCF1, RCL1, EXOSC8, UTP20, RPL21, MTERF4, RPL5, MRM2, EXOSC9, MPHOSI
TIAM1, KALRN, VAV2, AP2M1, ARPC5, NCSTN, ARPC2, EFNA1, PTK2, SDC2, CFL1, HRAS, CLTB, YES1, GRIN1, PAK2
TFPI, TXLNGY, F5, NADK2, LACTB2, DSC2, TMTC1, DNAJC21, SULF1, CRISP3, CSPP1, B3GALNT1, KRBOX1, TC2N, FAI
, ITGA2, COL1A2, TNXB, COL3A1, COL4A3, LAMB2, COL6A5, DAG1, BGN, COL4A1, COL4A5, AGRN, LAMA2, COL4A
X2, LINC02086, PCDHA9, ZEB2, ATL3, RGS1, RAB34, MGLL, COL27A1, SPARCL1, PXDN, TMED3, SPON1, CTHRC1, SI
L2, TSHZ3, ELF2, NUAK2, NUAK1, MARCKSL1, ELF5, SYNJ1, NAP1L1, EIF3A, PDIA6, NAP1L5, AUH, SLMAP, QKI, WD
1, MAP7, ABCB1, HBBP1, STAT1, MECOM, HIC1, MYH10, CMBL, FOXC1, GZMB, CAVIN2, LY6E, OAS2, MYCN, PPP1

GEF26-AS1, ARHGEF26, DHX36, GPR149, MME, LINC01487, STRIT1, PLCH1, PLCH1-AS2, C3orf33, SLC33A1, GMPS
O1, PSMD5, JAK2, PSMD8, MAPK1, PDGFB, TNRC6B, CSF2RB, IL2RB, RBX1, PSMC6, PSMA3, PSMC1, PSMB5, PSM

NF595, CES1, ABCA11P, TKTL2, ST18, FCN2, RGS18, KRT73, NUDT9, PLXNA4, SLCO3A1, BICD1, TAC1, KIF26B, CTBI
1, STC2, CHPT1, RIPOR2, CRYBG1, LPAR3, AFF3, KHDC1, ELOVL6, CA8, ADCY3, TMEFF1, DDIT4L, CAST, ACOX2, VC

45B, CTSD, GALNT6, ARMCX6, FASN, TGFBR3, KCNMA1, LRP4, IGFBP3, SLC16A2, SPINK4, LRRC15
A8, SDC2, COL1A1, S100A4, SPON2, MFAP4, SSPN, VIT, PI16, CYBRD1, SELENOP, ALDH1A1, CPQ, IGFBP7, CXCL12,

RBM14, ARHGAP30, RTN3, PHF21A, CHST11, SEMA3C, ZBTB20, HHIP, ANGPT2, RAP2C, CCNI, PIK3C2A, COL25A1, C

N1, OPRD1, CUBN, DOK1, PDIA5, ZHX3, GOLGA2, OPRK1, ITGB2, CFH, PAX2, SKAP1, KAT6B, IDUA, HNRNPU, DTN
MGAT4A, ABCB9, GAS7, DOCK3, PCDH19, EPS15, EPHA7, DCAF7, CDKN1B, COL14A1, HOXB7, RNF5, SMARCC1, HO
7, DHX40, CHST15, SLC9A1, LZTS2, GPR85, ELF1, SLC20A2, GNPNAT1, SYNJ1, NAP1L1, AUH, CCNE2, FBXL19, MTH
19, FAM71E1, CLDN11, FRAS1, RNF150, PON3, DLX1, SBSPON, COL22A1, GPR27, ARNT2, GPR158, AS3MT, PRRX1

GPR160, TJP3, MYH14, SCIN, S100P, CDCP1, FXYD3, BHLHE41, AUNIP, PPP1R14D, SFN, MSLN, ALDH3B1, HSH2D, C
2BC8, H3C8, H2AB1, H2BC6, H4C6, H2BC17, H3C6, H4C13, H3C11, H2BC9, H3C1, H4C9, H2AC14, H2BC3, H4C5, H
NR2F2, TOP2A, MSH6, C1orf21, SMC2, CCNA2, ZFHX4-AS1, STMN3, IDH2, BUB1, TMPO, MCM7, MGARP, PTN, KIF

PHA3, EGR2, PRKX, SYNDIG1, TGFBR3, SLC38A1, TNFAIP8, IRS1, DLG2, UCHL5, GCAT, ALDH2, JUN, CLDN5, HSD17B
MN1, IL1RAPL1, PLAG1, MGAT4B, KLF7, MACO1, PLXNA2, MYOZ2, MCAM, PCDH17, DCHS1, FAM13C, ITIH6, SEMA
, CEP85L, THEMIS, WAKMAR2, CALN1, MCPH1, PDE7A, IDNK, PRKCQ-AS1, FAM238B, PDSS1, ACSM6, ARHGAP19-

S100P, MORC4, GH2, ZFAT, GRHL1, SPIRE2, MFSD2A, SLC22A11, HSD3B1, TMC1, LRP2, OLR1, GSTA3, LVRN, LMO7
1, FGL2, MAFB, GPR137B, PLA2G7, CD86, RGL3, FOLR2, VSIG4, MPEG1, CXCR2P1, OSBPL11, IGSF6, IFNGR1, SIGLE
PLPP4, LRRTM2, JPH3, TRIM36, AMER2, IGLON5, INSRR, PEX5L, SCUBE1, FAM131B, KRTAP5-AS1, FRMPD3, ARSF,
TBXA2R, ACRBP, NFE2, SSX2IP, GUCY1B1, FAM110A, ATP2C1, THCAT158, PTGIR, MARCHF2, PDLIM7, P2RY1, C11o
, ADAMTS5, FBLN2, COL5A3, CHPF, GXYLT2, HTRA3, ANOS1, EBF2, GPX8, FMOD, SSPN, PDPN, C1S, MFGE8, CCDC
, ITGA3, PLXND1, PXDN, PLIN2, ADM, CXCL2, EMILIN2, ELF4, CORO1C, SEL1L3, MRC2, APOE, SLPI, GJB3, MXRA8, I
LC38A5, ALAD, RRM2, UBAC1, FAM117A, GYPE, TANGO2, SLC14A1, ART4, PHOSPHO1, UBE2O, UROS, GMPR, ACS
, C5AR1, FGD2, SLC38A6, HLA-DRB6, LAIR1, CHI3L1, SLAMF8, FAM20C, RUBCNL, SLC11A1, LILRB2, LILRB4, MNDA,
4C, SLAMF1, GNAT2
P, CLEC10A, ABI3, CD300LB, CD300C, LINC01478, MCOLN1, RETN, CD209, MAN2B1, IFI30, LRRC25, TYROBP, PLAU
278, SLC38A6, SCIMP, GPR34, S100A8, TMEM106A, LILRB1, LRRC25, P2RY13, FCN1, PILRA, LILRB5, SLC24A4, TREM
, SLC8A3, MPIG6B, FMO5, SLC39A3, LY6G6F, LIPC-AS1, TCEAL1, SRPRB, ASB2, PEX5, GP1BA, GP6, EXOC3L4, P2RY

Y96, THEMIS2, LINC00996, TGFA, MS4A4A, LILRB2, RGS1, CLEC7A, CCDC170, LILRB4, IL1B, ADGRE4P, IFI44L, SPIC,
FR2, EPOR, ARRDC2, KLF1, TRIM10, ESPN, SLC6A9, HBE1, CPOX, PPME1, UBAC1, BPGM, SEC14L4, ALAD, UBR2, Q
1, HOXD10, RPS15AP10, KRT18P55, FLNC-AS1, CACNA2D1-AS1, P3H2-AS1, IL5RA
, RCAN2, MT1M, SYNPO2, IGFBP5, LPP, PPP1R12A, PLAC9, MAP3K20, FOS, CARMN, CNN1, SPARCL1, CAVIN1, ZFP
21, PPFIBP2, MYH14, SDK1, KRT18, NIPAL1, COBLL1, IL1RAPL2, RAB11FIP2, RET, TMPRSS2, PPL, ANXA3, SDC1, AC
18, PTCH2, CBLN4, TRPC4, RXFP1, COLEC11, IGFBP6, PI15, FMOD, SULF1, CARMN, PI16, MYH11, TMEM119, ASPN
GAP5, DNMT1, DRAM1, DSG2, AGO2, EIF3D, EIF3E, EIF3H, ELF3, ENO1, ENPP2, EPCAM, EPRS1, EPS8L3, ETV5, EXT
NT2, MYOZ1, OBSCN, TPM1, SMPX, JPH1, MYL4, ASB5, MYL5, HSPB8, TMEM38A, HSPB2, TRIM55, SRPK3, MGC27
, FAM210B, UBR2, H1-2, MYO18B, CPOX, EIF2AK1, ARRDC2, TRIM58, TRIM10, TAL1, TSPAN32, E2F2, GYPB, MYL4
L, H3-3B, RERE, SOBP, LZTS2, SEPHS2, GLRA2, PCDHA5, KCNJ13, HHATL, NR2F6, SERTAD4, NR2F2, LINC00525, NR
12, LINC02284, ACRBP, ANKRD33B, EFHC2, RBPMS2, SEPTIN5, PTCRA, XYLT2, WFDC1, CXCL3, INAFM2, LY6G6F-L
A1, CD79A, SASH3, SHISAL2A, LTA, CTSW, RAC2, GZMM, ICAM3, S1PR4, PYHIN1, MYO1G, CHI3L2, OAS2, IL2RB, Z
45, TACR1, TRPM6, PRSS48
NCA, TSPAN5, CISD2, CENPE, GYPE, GYPB, GYPA, RAPGEF2, FCHO2, RFESD, MARCHF3, ACSL6, LARP1, FAXDC2, HN
1, MS4A4A, HLA-DQA1, CD83, AIF1, CMKLR1, CLEC7A, C3AR1, TLR2, NCF2, FLT3, SLC7A8, FCN1, CIITA, JAML, MND
71, TMIGD2, ICOS, SIT1, LEF1-AS1, VPREB3, FCRL1, NCR1, KLRC1, SLAMF1, SHISAL2A, STAP1, PTGDR, GZMM, LTA
DT1L1, LINC02820

O3, ROCR, SDC1, LINC02343, S100A14, PRSS22, HKDC1, CAPS, PTPRU, KCNJ15, CA4, NECTIN4, ANXA9, ANXA13, T
EC3A, HCST, ISG15, CTSS, HLA-DQB1, NBPF10, FXYD5, NFKBIA, JAML, HCK, TGFBI, PSMB9, CLEC2B, CPPED1, GPBA
, SEMA6D, LIFR, HSD17B6, MT1X, LIX1, MT2A, TFPI, MFGE8, IQCA1, GABBR1, FIBIN, TMEM47, GDPD2, SLC39A12,

AB, CDH11, PDGFRL, P4HA1, LTBP1, TNC, LOXL1, C1S, COL16A1, SERPINH1, THY1, CALU, ACTG2, PLXND1, CD34, P

RRM2, COL1A2, S100A11, IGFBP4, TGFBI, SRGN, GADD45G, EGR1, FGFR1, EMP3, NFIL3, FAT1, RAB31, CCN5, MFA
AP2, IER3, GNG11, CAV1, TRAM2, CD151, F13A1, STAB1, NECTIN2, COL6A1, TPM2, HSPG2, QSOX1, RNASE1, LGMI

M2, GJB1, ERF, ELF4, PPP5C, PTX3, SMAD3, ITGA5, KRT4, EPAS1, UBE2H, PRB4, SECTM1, MMP9, TNFAIP2, ACP5,
A11, PRSS2, GPRC5B, MAP7, ST3GAL5, XDH, HYAL1, LGALS4, IGFBP1, CALML3, GRB7, ANXA9, TSKU, GDA, GPRC5
51, STAB1, TPM2, HSPG2, QSOX1, RNASE1, SERPINE1, WWTR1, SMTN, GAS1, APOE, RBP1, IFI27, DLK1, FHL2, CCN
C, KLK5, MRAP2, ROPN1L, KLHL13, DSC3, B3GNT5, CSF2

QKI, MASTL, MYB, USP15, SERTAD2, STX6, ZDHHC1, RAP2C, MMP14, ARHGEF11, BRWD1, EREG, SRGAP3, RASD1,
SLC6A10P, KDM5C, KCNIP4, PLCB4, TOGARAM1, PHC1, AKT3, CCN1, ZFAND6, PCDH9, MMP16, PCDH8, MBNL1, I
, NKAIN2, RGS2, TRPS1, ARF4, BTBD10, DHX40, MAP6, TLL2, CEP41, RANBP10, SOBP, HLF, AP3S1, SPTSSB, FAM2
1, CTDNEP1, HNF1A, TCF4, CDC42EP3, PPP2R2C, C9orf24, SPOP, PHYHIPL, GPD1, KLF12, ESRRG, HGF, NXF1, LINC
L, RGS3, PKP3, RGS8, DHX40, RERE, BTBD11, BACH1, GFAP, SHROOM1, PDZD9, ZNF385B, FGF9, BNIP3, LRRC15, M
BP5, CALD1, CAMLG, CAST, CBFB, CFAP36, CCDC34, CCDC6, CCDC65, CCDC90B, CCDC91, CCNB1IP1, CCNG1, CD22

IYL4, CXADR, HMGCS1, ANXA2, TIMP2, KIF2A, IL2RG, PLOD2, TRPC1, HERC2P2, PTPN4, DHCR24, PIM2, CAPN2, CD

, TNNT3, IPO7, PPIP5K2, TMSB4X, PODXL, SLC39A10, CACNG4, MCF2L, PWAR1, ARMT1, MUC3A, HPSE, CDC42BP
, SLIT1, PRDM2, RNF165, NEFM, SCG2, OBSCN, CDKL2, NFIX, PLXNA3, SCN3A, SRRM4, C11orf95, DLX1, NGEF, DN
TARD10, NTRK2, ANK2, PENK, MAP1A, TSC2, DLX2, TUBA1A, WASF1, CYP27C1, PHACTR3, GDA, ENHO, CAMKV, P

ALT1, SLC38A3, LEKR1, MAP3K19, TLR2, GAGE1, FUT4, DDX6, EHD1, CTNNA2, PIM3, CCN5, LINC00221, RIPK2, CLI
XAS2, PTPRE, KLF6, USP6NL, BEND7, ARHGAP21, ITGB1, NRP1, SGMS1, EGR2, SLC25A16, NOC3L, AVPI1, PITX3, C

BRA1, RPS18, NUDT4, AAK1, IDH1, TRAM2, CD55, DLX1, CALML5, WDR45B, ZHX1, FAM160B1, SLC4A11, IER3, TRI
PRK, DIO3, LTBP1, ITM2A, PHIP, SORBS2, PHLPP1, ISYNA1
, PIK3C2A, MTSS1, ZBTB16, RBMS1, NTN4, HLA-E, ARL15, TTC28, HERC1, HSPH1, SMYD3, PLXDC2, MAML3, IL6ST,
TN4, FOXP1, SERTAD2, SP3, OSBPL8, BCL7B, ZDHHC9, DTWD2, AP3M2, RBMS3, CSMD3, C16orf72, CCDC186, RNF
, CELSR3, CHGA, CHN2, CHST1, CLCN4, CLUL1, CMIP, CNGB1, CNR1, CNTNAP2, COMTD1, CORO2A, CPEB2, CPEB3,
P1, MSX2, PADI3, KRTAP19-1, SCAF11, DCT, CDK2AP1, DNAJC17, KRT72, RETREG3, RCC2, CELSR2, LEP, ZNF148, E
WBP4, CCNE1, ANGPT1, AGPAT4, FBXL18, HYAL3, GSE1, ICAM2, LINC00670, CRLS1, DOK2, ILRUN, C22orf31, ADI
9A6, SPTBN2, RAI2, TNS3, LRP5, TMPRSS4, SGCE, CSGALNACT1, F2RL1, GRAMD2B, CLDN9, MAP3K9, SLC12A2, SD
1, DCLK2, DHFR, DHX32, DNAAF3, DNAH6, DNAH7, DNER, DOCK1, DPF3, DPP6, DPP7, DTNA, EDNRB, EEPD1, EFH
N13, MED13L, LIMCH1, GPHN, WSB1, MYO1B, RBPMS, ADGRL2, HSD11B2, SH3RF1, FNDC3B, BCAS3, FBXL17, MA
LF3, CELF4, CELF5, CELSR3, CGN, CHD5, CHGA, CHL1, CHRNA4, CHRNA7, CHST1, CLCN4, CLSTN2, CLUL1, CLVS1, C
P, CALHM3, KNL1, CASQ1, CATSPER1, MCUB, CCDC134, CCDC15, MTCL1, CCDC169, CCDC34, CCDC74A, CCDC77,
46, SHFL, NDUFB6, SLC6A5, CREM, ARHGEF4, PCDH15, CDON, LASP1, PCGF5, NETO1, HSP90AA1, A1CF, C2orf69,
ACTR3, PRPS2, BZW1, ZBTB20, NAB1, NOTCH3, SSH2, PHTF2, CSNK1G3, PGAP1, ATE1, ZC3H12B, PTPN20, ZNRF2,

CFAP44, ADAM9, MSTN, ELF5, TRHDE, ELOVL7, TCHHL1, DMXL1, ZC3H6, KIAA0825, ATP6V1G3, SDK1, NUMB, KI

GBX1, AMIGO2, RASGEF1C, KCNK1, WNT6, IHH, CDH8, P4HA2, BAIAP2L1, DLX5, VSX1, LAD1, ANKRD63, ZNF536,
LSR, BMP4, SOWAHB, FSTL4, FOXN4, NEUROG3, VGLL2, TFAP2C, PROK2, DMRT3, C1QTNF4, CRTAC1, VIPR1, HOX
2, PHLDA1, RASSF3, MTCH2, LY6D, PRPF19, RUNX1, KRT8, TACSTD2, SOX9, GAB1, TNFRSF12A, RNF149, ADGRD1,
DKN3, GSE1, TNFRSF21, CPVL, RAG1, FAM72C, CDC25A, DSCC1, PBK, E2F8, NLGN4X, CRNDE, LDLRAD4, MIR646H
XP1, NHSL1, INPPL1, COL4A5, CA13, RACGAP1, DGCR2, ATF2, SPIN1, TMEM106B, HSPB7, SMARCA4, SLC25A10,
, FMN2, MAGT1, TMTC4, VPS29, TCAIM, RTL9, LYPLAL1, CACNB4, SAMD5, PHF24, ANK1, FRMD4B, DCUN1D1, KL
14A, TLCD3B, DIS3L2, FGF9, FMN2, FNDC5, FOXA1, FOXA2, FOXP2, GABARAPL1, GAP43, GAS2, GDAP1L1, GDPD1,
FRSF21, SLC29A1, TRAF3, SET, CD276, CER1, ISG20, STAT2, AMD1, TBL1X, BACE1, COLEC11, CDYL, SERF2, SLC35A
DACH1, NPR3, CHST9, LHFPL2, TNNI2, PMP2, ARHGEF38, CLCA4, PRDX3, HES5, SOX4, NLRP11, PLXNB1, RDX, SLC
ITPRIPL2, ARHGEF10, DDX5, MKNK2, SALL3, PSG3, BICC1, NR2C2, GPATCH2, SEPTIN2, TNKS2, ATAD2, FAM13C,
NTNG1, RAP2C, ZNF559, VSTM4, EEF1A2, ACSL1, FZD1, MBNL1, GUCY1A2, ATG13, NAA40, SGMS2, CXXC4, ZSCA
1, YTHDF3, UBE2K, WASHC4, EPHA5, PJA1, CD24, RAMAC, COQ9, KRT1, FSD1L, C18orf63, MGAT4B, CYP51A1, RC
, ERCC4, ASTN2, CXXC4, RBFOX1, SNTB1, CCN3, IL17A, LRRC8B, NMBR, SNX2, SREK1, NETO1, GSKIP, COL11A1, A
APGEF2, SLC1A1, CASK, DCUN1D3, ANTXR1, DESI2, FBXO33, CCDC25, MED12L, CNTNAP2, NOS1, MED28, DPH3,
RB, UBL3, SECISBP2L, ANKRD40, GYPA, PHTF2, LUZP2, REST, TENM1, IPO7, ZC3H12B, RGS4, ICE1, TNS1, CTBS, FRS
H2, XIAP, C1orf43, TBC1D15, HSPA4L, FBXL3, BCOR, MSL2, LSM12, SCP2, GPATCH2L, ZBTB20, PTGER3, SUV39H2,
HIP1, LRRC8C, ITM2A, MEG3, RASIP1, VCAN, MAMDC2, TIMP3, CDH3, BMP7, RELN, PSD2, OSR1, TENM3, FMOD,
45B, PMEPA1, PITPNM2, GRIN2A, CCNA2, F3, VTI1A, ADCY9, MECOM, ROBO2, TBR1, BAG5, DNAJC16, PRUNE2, C
3C1D1, ITGA1, SLCO3A1, ZEB1, IGFBP5, TPM1, NEAT1, ARHGEF7, JUN, DSTN, FCHSD2, GPX3, ROCK1, MAML3, DU
2P4, MOBP, PDE1A, RCC1L, RFC4, ZNF703, CASP6, BLVRB, LGALS2, EBF1, SLC43A3, MOCS1, GNB3, LCOR, TOMM

RXRA, ESR1, CELSR2, ADAMTS19, NAB2, S100A7, ST8SIA4, MEGF9, SNTG2-AS1, TIPARP, POLR1D, TMPRSS4, SLC1
BTB1, ARID4B, CAV2, COL4A2-AS2, MAGI2, INTS6, IGSF10, AEBP2, PAK6, PI4K2B, CEP135, PEX5L, KCNB1, GFRA1,

BMPR1B, GHRHR, GPR39, KIR3DL1, CHRNA2, PRPH2, PTPRS, CCR5, CCR9, KIR2DL4, PRSS12, PTAFR, RARB, EFNA4
TD5, VAV1, FOXP1, FOXP2, SHOX2, FOXG1, HOXB4, DLX1, EPHA7, HOXB7, EBF2, DLX5, ZIC4, SALL1, RAB2A, GRK6
MYRF, HS2ST1, RHOB, BRD8, SRP72, GGT5, MYH10, TPD52, LRP6, DNAH2, METAP1, CKB, BARX2, MPL, TM4SF1, S

BC6, H4C6, H2BC17, H3C6, H4C13, H3C11, H2BC9, H3C1, H4C9, H2AC14, H2BC3, H4C5, H2AC8, H4C4, H2BC7, H3
3, RIT1, ARSB, NRP2, JPH3, NRP1, BACH2, ZNF385B, MITF, PPARG, LINC00052, NAP1L2, MCTP1, GFI1B, AP1S2, Z

3, NTRK2, MAP2, RWDD3, MTFR1L, ZNF219, BMP5, TSHZ3, NRP1, MYBPC2, CPEB4, DPP10, MITF, TMEM81, ACTR
2L, RAB33A, GINS3, TRMT2B, FANCG, CRIP1, PRIM2, ALDH5A1, MFAP4, EPS8, NPR3, EXOC1, ALKBH1, HOXA5, FA
3G, SIGLEC11, CD300LG, TREML1, VCAM1, SLAMF6, CD200R1, RAET1E, COL1A2, B2M, CD3D, LAIR1, LAIR2, KIR3

1D8B, APOBEC3G, MMP10, ZBTB47, ALK, VIM, PPFIA4, FOXA1, PPL, C4orf47, CDH12, ID2, RBPMS, PRKRA, PDLIM
4, PKP3, JUP, DSP, EPS8L1, HIP1R, ESRP2, IRF6, CDH1, SPINT1, CDCP1, MAL2, TMPRSS13, EPCAM, ESRP1, ERBB3,
3C2B, MGAT4C, MMP17, TAOK1, CACNG3, MMP15, OGFOD1, ELMO2, KLHDC3, INHBA, NAALAD2, CLIC3, EIF4A2,
AGXT2, MTFR1L, MAB21L1, RERE, CDC42BPB, TSHZ3, FOXA2, TSG101, ELF5, NBEA, MECOM, TMEM147, MMP21,
PM1, TSPAN32, CD47, PECAM1, GP9, SLC9A9, SPX, MARCHF5, RUFY1, MYEOV, FCER1G, TLN1, CTDSPL, EAF2, SM
NS, H4C15, H4C14, H2AC19, H4C12, H2BC14, H2BC8, H3C8, H2AB1, H2BC6, H4C6, H2BC17, H3C6, H4C13, H3C11,
PD, COL13A1, B2M, RPS6KA2, SUCLG2, RRAS2, TKT, DRD3, DST, MEST, MXD4, TGFBR2, FIG4, IGFBP3, BST1, BLK,
GDPD4, GPR88, DPY19L3, RTN3, STAC2, VCPKMT, FMNL1, TLE3, CACNG2, OTUD7B, INHBA, SLC26A8, C12orf42, M

PBP1, ID1, RASA1, FYN, DPYD, FOXK2, AGAP1, NPEPPS, DUSP1, ZNF292, NEK7, MYO10, GINS1, FAM169A, FAM1

3ZF, NNT, KIAA0232, APOD, OAS1, IFI6, MAP3K7, AOPEP, FAT1, CRYBG1, MXI1, INPP4B, CISH, DGLUCY, CTNND2, /
D2, BEX4, ELL2, CDC42EP3, NUDT12, CYFIP2, CCDC69, CELF2, DENND11, MYCT1, KYAT3, SKIL, CCND1, DCUN1D4,
PH10, RRP36, RPS10, RIOK1, RPP40, RPL23, EMG1, RPS4Y1, RPL36, EXOSC2, DKC1, NOL11, RPL27, UTP3, NIP7, RP
, LIMK2, EPHB3, AP2A2, EPHA10, ACTG1, EFNA5, EPHB4, AP2A1, SRC, PSENEN, ITSN1, ARHGEF28, ARPC4, ARPC1/
R2, TRNT1, NKTR, UGT8, FABP4, ZNF567, LPL, SLC7A11, DESI2, PTPN13, REL, TRIM2, IGSF10, GALNT7, CYCS, POST
A6, COL5A2, COL6A6, ITGB3
PTLC3, CTTNBP2NL, FN1, TMEM243, SERPINE2, FCAMR, GPX8, RPS25, LIX1, MFGE8, NFKBIZ, MYOF, RPS10, ERICH
R33, FOSB, IL6R, PPP4R4, RADX, CCND1, BRWD1, SRGAP3, TMCO2, TBC1D9, ADGRG2, AZIN1, ZNF655, CIPC, ZNF(
R14A, IFIT5, RAB27B, HOXA5, CLEC2B, GDPD1, IRF7, MIR155HG, MTSS1, DDX58, HOXA3, IFI44, DNAJC6, ATP7B, (

, KCNAB1, KCNAB1-AS2, KCNAB1-AS1, SSR3, TIPARP-AS1, TIPARP, LINC00886, PA2G4P4, LEKR1, LINC00880, LINC
A6, PSME2, PSMA7, PSMD10, FGF9, MAPK3, PSMD7, CSK, PPP2CB, RASAL3, TYK2, GRIN2D, PPP2R1A, JAK3, PIK3F

P2, SPAG11A, SLC26A4, SSX3, MYH7, SSR3, KCNAB2, PIWIL1, KLK1, KLK15, NAGS, GK2, MAPRE2, MUC4, LARS1, PT
L, PIK3CB, TENT5C, ARHGEF26, MUSTN1, APBB2, ANPEP, GPR84, ORM1, MAPK14, DHRS11, FAM117B, KLRG2, AL

MGST1, TFPI, S100A6, HTRA3, LSP1, NUPR1, VCAN, SGCE, AKAP12, GPNMB, VIM, ITM2B, ANXA1, PLPP3, LAPTM4

CACNG2, ARRDC3, CLC, ELMO3, KLF9, MAP3K11, LIMS1, NNAT, IKZF2, IKZF1, SF3B5, GET4, LRRC57, BCL11A, PDE1

A, PEMT, PCF11, SERPINB7, TSPAN31, MAGEA12, RENBP, JUND, BRSK2, ATG5, ATP2C2, ZYX, SLC22A18, SRI, HSPA
XB6, PARP6, CHRD, RANBP10, RBM26, BACH1, LTN1, SCHIP1, ZNF385B, OPCML, ELF4, DICER1, NAP1L1, CCM2L, L
FD2, TMEM109, AP1S2, P4HA2, SPART, QKI, BRD4, ARHGEF2, RAP2C, ZDHHC5, PGAP1, GMEB2, ARHGEF5, GZF1,
, LAMA1, LSAMP, OLFM1, ANKRD6, NLGN4X, CECR2, ZNF704, ODAM, GPC4, CD200, SPIB, DAPL1, TCIM, EDAR, TN

CLIC3, BCO1, CDH17, REG4, LIPH, MYO1A, MTMR11, DHDH, SLC6A20, FAM83D, GPA33, NOXO1, NMU, ST6GALNA
I2AC8, H4C4, H2BC7, H3C7, H2AC4, H2BC10, H4C1, H4C2, H3C10, H3C2, H3C3
20A, USP1, MATN2, CLU, SSRP1, LUM, IMPA2, DEPDC1, OLFML3, FBXO11, BAALC, LIN7A, TNFSF4, ANP32E, PRICK

33, ID4, LRP1B, FHL1, PLN, SLC26A4, MYCN, LIPG, EDN3, TRAPPC6A, TNFRSF11B, FCGBP, PRKCQ, SPTBN1, ADH1A,
A6A, EPHA7, ARHGAP44, SMURF1, FCGBP, RERE, KLF3, PDZD2, TSHZ3, JPH4, ZNF385B, ELF4, ATP5F1B, GLRA2, MI
SLIT1, PGGHG, RAG2, DOC2GP, CD3G, HDAC7, TESPA1, DGKA, PITPNM2, RASL11A, LINC00426, DLEU7, RNASEH2I

7-AS1, C5orf17, TMC5, PERP, KRT18, ATF3, GNGT1, LCMT1-AS2, ERVW-1, EFHD1, GPX3, GRHL2, USP43, ITGB4, ST
C11, SLC49A4, LINC00970, EYA2, NFAM1, CCR1, FPR3, LILRB2, PLA2G15, RASSF4, RAB39A, RNF135, OTULINL, ITG
TMEM196, DIRAS1, ARHGDIG, PKIA-AS1, SPHKAP, AMER3, DCLK3, SERTAD4, HECW1-IT1, FOXN4, C9orf24, C9orf1
orf21, TRIM58, SYTL4, ANK1, MYOM1, LRP12, ILK, CMTM5, RAB37, PTCRA, ADCY6, GAS2L1, GATA1, ZFPM1, OR2W
8, LGALS3BP, HSPB6, PDGFRA, PMP22, SFRP1, SERPINE2, S100A6, FLNC, ADAMTSL4, FKBP10, P3H4, NOVA1, FKB
ETV1, COL11A1, PLAAT3, ADAM12, NCKAP1L, NR1D1, NMU, NFE2L3, G0S2, C2, PMAIP1, IL1RN, GRB7, ACTN4, IER
L6, TFDP1, GLRX5, EPOR, BPGM, SEC14L4, DCAF12, ESPN, RFESD, GFI1B, HMMR, SNX22, OR2W3, XK, ANKLE1, Al
ADGRE2, KCNJ5, S100A8, P2RX7, LTA4H, SDS, ARHGAP22, MILR1, ADAMDEC1, C1orf162, SCIMP, FCGR1A, TLR6,

R, C5AR2, SIGLEC16, SIGLEC9, SIGLEC7, CD33, SIGLEC12, FPR2, FPR3, LILRB3, LILRA6, LILRB5, LILRB2, LILRA5, LILR
12, LINC00996, TLR2, TIMD4, HLA-DQA2, PLB1, PLA2G7, C5AR1, CLEC9A, NFAM1, LILRB4, IDO2, SPNS3, MS4A14,
1, PIGP, MLYCD, GRM3, INPP5E, F5, ADAM32, GTF2E1, FAM186B, TUBA8, EREG, RPS6KL1, RAB3C, CFAP58, ERICH

, VSIG4, IGSF6, ITGAD, NCF2, EBI3, CABP4, CDS1, PRAM1, FCN1, IL15, C3AR1, LINC01357, SIRPB2, NEXMIF, MNDA
SOX2, PIGQ, RHD, UBE2O, RNF224, FKBP8, GYPE, NFE2, NARF, CR1L, SMIM5, DHRS13, HAGH, TCP11L2, FBXO9, A

P36, HSPA1A, CRYAB, KANK2, GGT5, EBF1, PPP1R12B, PDGFRB, PALLD, EFHD1, JUN, COX4I2, COL14A1, MYL6, PTP
OXL, PERP, RASEF, CLIC6, SPTLC3, MUC1, FER1L4, AOC1, CLDN7, FBP1, UPK1B, MIR31HG, LINC02343, KSR2, WNT
I, CYP1B1, PLA2G5, EBF3, CCN4, MEOX2, ADAMTS15, OLFML1, OSR1, FAP, ITGBL1, ANO1, FGF7, AGTR2, SGCG, PD
T1, FBL, G6PD, GNAZ, GNPDA1, GPD1L, GPX7, MACROH2A2, HDAC2, HEATR1, HELLS, HGF, HIC2, HIF1A, H4C3, HJU
382, CAMK2A, SGCG, KLHL31, KLHL40, PRR16, MRLN, TCEAL7, NMRK2, ADCY2, SPHKAP, ADPRHL1, CACNA1S, CAC
-, UBAC1, SMIM1, SLC6A9, SNX22, ATG14, ZNF451, RHD, LINC02506, CR1L, GMPR, PPME1, XK, DYRK3, DCAF12, A
2F1, PEPD, POGZ, ANP32D, MGAT4C, ARRDC3, GRM1, CHRDL1, ENTR1, RAB11FIP1, RAPGEFL1, CER1, CD180, SCM
Y6G6D, LINC00989, MPL, LINC02770, GCSAML, MDM1, CXCL2, TUBA8, SEC14L5, LINC00211, PADI4, GP5, HYAL3,
NF831, CORO1A, LINC02328, GZMA, TAP2

MMR, TMEM14B, GMPR, H4C3, H1-5, TRIM10, TBC1D22B, TSPO2, RHAG, FBXO9, GCLC, KIAA1586, ZNF451, SLC22
DA, HLA-DQB1, ARHGAP22, HMOX1, CD1C, LY75, TRPM2, NCF4, SIRPB2, RASGRP4, AGR2, C9, CFP, S100A9, NAPSE
, PRF1, VPREB1, IL23R, UBASH3A, IGLL5, CXCR3, S1PR4, MATK, GNLY, MZB1, LINC01215, LINC01891, CD19, FCRL

RPV6, CDX2, GADL1, SLC3A1, TMEM139, F2RL1, GS1-24F4.2, ATP13A4, DEFB1, MMP7, SLC34A2, PART1, DEGS2, I
R1, PLEKHO1, GRK2, NFKBIZ, AP1S2, PNPLA6, TXNIP, PTP4A2, IER5, APOBR, CLEC12A, ITGAX, CD48, C5AR1, MYO
SESN3, GLUL, GJB6, SLC4A4, PDLIM5, NRCAM, CACHD1, SH3GL2, DTNA, CD44, CDO1, LUZP2, LGR4, GARNL3, SO

DPN, MLLT11, NNMT, PTN, SCG2, SV2B, SLIT2, CLIP3, CDH5, ZEB1, TIMP3, ALDH1A3, CXCR4, CYP1B1, FBLN2, MM

P2, IER3, TNFRSF10B, CAV1, C7, IFIT1, FXYD1, STAB1, NECTIN2, FOS, COL6A1, TPM2, NCALD, COX7A1, CFHR1, AD
N, SERPINE1, TGFBR3, CLEC3B, IGFBP3, GAS1, APOE, RBP1, IFI27, ATF3, DLK1, CCN1, MAFF, RHOB, CRYAB, SOD3,

MUC1, CYBB, CCL5, FCN1, MSLN, LST1, CORO1A, HCK, RAC2, CAPG, NKX2-1, SELL, KLHDC3, SCNN1A, PRR4, TCIR
D, HLA-DRB5, LGALS3BP, MST1R, PTPRF, TNFRSF10A, LILRB4, TAGLN, KIAA0040, KRT8, ZFP36L2, CRABP2, MMP7,
1, MAFF, LDLR, LAMB1, CCL2, MAOB, JUNB, TIMP2, AKAP12, ORM2, TNC, EMP1, DBN1, PLAT, SDC1, GET1, PLEC,

GRAMD1A, CCDC88A, CMPK1, CEP97, TBC1D9, ZNF367, ABHD3, ABHD2, EPB41L4B, SNRK, NPAT, PKD2, SLC4A7,
ENG8, ATL1, KIF1B, CNTLN, SAV1, PRND, MIP, CHD9, WT1-AS, SORBS2, SYN3, C5orf24, AFF2, GRIA3, DGKH, ATP8
14A, STOX2, SLC38A7, CCNE2, ACAP2, P4HA2, QKI, MICAL1, CLDND1, FBXL17, RAP2C, FBXL20, ARHGEF6, GMEB2,
00314, TNNI1, DCSTAMP, KCNK3, EMILIN1, OSR1, SHOX2, CYP17A1, TRPS1, CMYA5, MFF, SYTL2, MAB21L1, MAB
ECOM, LNPEP, MMP20, SNPH, USP12, FGF1, AMY2B, SLC22A2, SPINK6, USP13, AVP, PIK3C2G, SEPTIN4, MMP19
6, CD3G, CD8B, CDC23, CDKN2AIP, CDV3, CENPK, CGRRF1, CHD9, FOXN3, CHM, CHSY1, CLDN5, CLEC1B, CLEC2D,

164, CAPG, VCL, GJA4, RRAS2, TXNRD1, TBPL1, AIF1, CRISP3, UNC119, MAN1A1, PSMG1, TNFAIP2, STAT4, USP8,

A, RPL37, LAMA5, CMTR2, UNC5C, ZNF138, HSPH1, EXOC3, KRT80, REL, ARID5B, RETREG1, RPS6KB1, MAP3K1, N
M3, TPTE2P1, RNF112, MYO16, CCBE1, ZFP90, TMEM151B, RGS11, SYBU, ZNF37A, THRB, KIAA0895L, CXADR, JA
KD1, LRRC4C, IVNS1ABP, GPR12, MT3, LMO4, DTX1, BHLHE22, RAB9B, MYT1L, CADM1, GUCY1A1, WDR59, RBM

T1, AKAP1, TP53AIP1, NEB, DYNC1I1, STXBP6, BCL2L1, ANGPTL4, REL, FPR2, NOX4, TPD52, ZHX2, RDX, LINC006
10orf120, CHST15, TEX36, ARNTL, LDHAL6A, SLC6A5, HIPK3, FRMD8, BIRC2, ADAMTS15, RNH1, TOLLIP, PHLDA2,

QK, DDR1, FGD6, TNS3, COPZ2, CDS1, VSIG10, CRISP3, CDK6, LNX1, RNF144B, NCOA1, ANXA3, MFAP3L, DENND4

FCHSD2, PIAS1, BMPR2, GPX3, DNAJC1, UTRN, NKTR, CACNA2D1, MEF2A, LDLRAD4, COL4A2, DPYD, LRCH1, STA
2, GOLGA6L4, EIF4EBP2, LPP, CCDC92, JKAMP, SGK3, RIMKLB, PPP2R5E, RBM18, PTP4A2, ARL4A, FXN, BBIP1, RC
CPEB4, CRMP1, CSRNP3, CXADR, DACT1, DACT3, DCC, DCX, DGKE, DICER1, DLG3, DMTN, DNAJC16, DNAJC6, DN
NB1, CAMK2B, WFDC21P, XIST, FGFBP1, EIF3E, PLEC, MYO10, CTNNBIP1, RPS21, KRTAP8-1, KRT32, CES4A, RPL3
, GADD45G, SLC26A9, UROD, TBX19, TPH2, CSF2, FOXO4, RAB3C, ZBTB7B, TFR2, TPI1P2, TRIM15, TRIM10, CCL2
CBP, ABCG1, SCCPDH, SORL1, SLC2A6, GPX3, CBLB, TNFRSF11B, BCAM, FERMT2, ABCC3, FOSL1, MAOA, ALDH4A
D2, EGFR, EGLN3, EGR1, ELN, EPHA3, ERBB3, ERF, ETV1, ETV4, ETV5, F3, FABP5, FABP7, FAM181B, INKA2, FAM8
K8, ADK, LRMDA, BABAM2, MAML2, RALYL, PTGER3, CA12, PCCA, BRAF, PTH2R, GBE1, EXOC6B, RORA, SMYD3,
LVS2, CNGB1, CNTN1, COBL, COLQ, CPEB4, CPNE4, CRHBP, CRMP1, CSMD1, CSRNP3, CTNNA2, CTTNBP2, CTXN2,
CCDC80, SPDL1, CCNA2, CCNB1, CCNE2, CCNF, CCNG2, CCP110, CCR7, CDC20, CDC25A, CDC25B, CDC25C, CDC42
CGRRF1, GSK3B, CDH1, RC3H1, INTS6, NUP50, PRDM12, FBXO45, SPRED1, SH3BGRL2, YRDC, TRIM71, COG6, HM
CLPX, F13A1, RANBP3L, ENC1, PAX6, SCRN3, CRIPT, ZNF761, TAFA1, CENPBD1, ZBTB10, SOX30, FAM20B, ARPP19

A1549, LIN7C, CLOCK, MEIS1, RYBP, PAK1, RASSF3

FOXF1, EMX1, RERG, SLC22A3, FGF8, FAM155B, STX3, NOTUM, JPH3, KCNK12, CADM3, SLC26A4, CADPS2, RALYL
D11, COLEC12, PAX7, FGF4, SCRT1, RASGEF1C, DACT2, GATA5, PTF1A, HS3ST6, MPPED1, KCTD8, ABCG1, PHF24, (
RASSF2, SLC38A10, RAD51AP1, ESRP1, GABRP, ST14, EHF, MFGE8, FGF13, CEMIP2, SLC15A2, HDAC11, TMEM79,
G, PRC1, AURKA, PCLAF, TSHR, CENPW, PTTG1, RAPGEF5, MCM4, KIF11, TUBB, KIF2C, GALNT7, CEP70, MCM10, (
CLDN10, PHB2, RAB31, RPTN, IL6ST, RBMS1, HMGB1, PMEPA1, UBC, ITGA9, ITIH5, SMAD1, NAV2, RTN4, SHMT1,
HL13, TBC1D3D, TMEM74, KAT2B, FLRT3, SFMBT2, WASHC3, TNIP3, F2R, SFXN1, BCOR, ACSL6, SEPTIN14, UBL3,
GDPD3, GFRA1, GNG2, GNG3, GPC2, GPM6A, ADGRG1, GREM2, GRIK2, GRIN3A, GRM8, GSE1, HEPACAM2, H2A\
3, CUL4B, ZNF587, RPRD1A, DBT, MAN1A2, MCPH1, SLC1A2, MED17, SERPINB13, ARHGEF4, EGLN3, DIPK2A, DEF
39A8, KRR1, VPS13A, TRNT1, SPTSSB, MAP4, MAGEB6, CD164, JUN, ZFX, GRAMD2B, MFSD14B, ZFYVE9, FRMD6, (
AKTIP, CEP97, CD274, EMSY, TET3, GPR63, NABP1, CNOT6, TGM2, ETV1, NANOS1, ZNF236, RBL2, GXYLT1, PBX3, I
N31, ATP10B, SLC43A1, FHL1, NRXN2, TNRC6B, CLDN18, LDLRAD1, ARFGEF3, SPATA31C2, ATP2A2, LMAN1, CAM
BTB1, TTC3, CITED2, ST8SIA3, NKAIN2, PTPN11, NUP50, RAPGEF2, ITGA6, GPR12, VCL, COPS8, CELF1, QDPR, ACA
RID4B, C1QL3, RPS6KA6, ALDH18A1, ARHGEF38, IGF2BP3, FAM133B, SPRED1, ACTR3, CHMP2B, DCAF10, ACTN2,
PAX7, RAB21, ABRAXAS2, GPD2, PTGFR, ACTN2, SV2C, CFAP65, PTPRG, NYAP1, YY1, SERINC3, CUX1, TNRC6B, HIF
2, PAIP1, ELAVL4, TMX3, FAM91A1, TAFA1, DLL4, DLC1, ZNF292, DDX5, HELQ, TBL1XR1, DYNC1I2, NR2C2, JRK, R
PRKD3, RWDD2A, IPO7, R3HDM1, ICE1, DLG1, TSLP, WDFY1, RANBP3L, PAXBP1, NUDT13, SMAD5, B3GALNT1, IN
THY1, STAC2, ARHGAP24, LAMB1, ENC1, TPST1, NGFR, ID4, NDN, BTBD11, GJA1, ITGA1, LMOD1, SCARF2, SEMA3
CSER2, ACSL4, LONRF3, KLHL20, ITGA2, SGK1, ATF2, SP100, CDC42BPA, MOB1B, MPPED2, ELMOD2, SLC35F1, MA
SP1, KIF13A, COL18A1, NBEAL1, SSBP2, MBNL1, PALLD, PLPP1, ROCK2, ESYT2, ACTN4, PTPRG, CBLB, VCL, UBC, RA
34, PALM2AKAP2, MAL, NDRG2, SRGAP2, ATAD2, FLVCR2, KIF4A, MAP3K14, RCBTB1, MAP1A, TMEM125, KDELR2

4, PDCD4, MPPED2, CISH, COL5A1, IRX3, PLIN5, HPGD, LINC00408, AP1S2, LAMP2, PRAG1, HR, RHOBTB3, ADCK2
CYBRD1, ZFP42, PHEX, NRG1, JAM3, VPS37A, ZNF34, OSBPL1A, TTC1, KRBOX4, TENT5D, CD55, TM4SF20, YIPF4, (

, GABRG3, FCER2, MS4A2, PPARA, CNTFR, CRLF1, FZD9, LPAR1, EPHA7, LIFR, TMPRSS15, OPRM1, ERBB4, PLA2R1
, WBP2NL, CACNA1H, SYTL2, DNAJB4, CEP41, MAB21L1, MAB21L2, PDZD2, HABP2, CCKAR, NPEPL1, CDC14A, KCI
LC18A2, STAT5B, DLC1, CDH16, PYCR1, HACD1, MROH7, JAK1, MDK, ZSWIM8, PPT2, ISYNA1, CTDSPL, KRT18, MY

C7, H2AC4, H2BC10, H4C1, H4C2, H3C10, H3C2, H3C3
IF654, KMT5C, SEMA3A, WDR47, JPH1, ARHGEF7, TNMD, TENT4B, CACNG2, OTUD7B, NEO1, ZFP36L1, NR1D1, PF

3, GPX1, HHATL, STAC2, AP1S2, PBXIP1, ZNF654, ARFGAP2, CAP1, FGF3, TUBB4A, NR2F1, ARHGEF2, POGZ, ARHG
DS1, SPON2, SCAMP4, TMCO6, CEP55, DLX5, APOBEC3B, PER2, ERO1A, SERINC5, GRB10, COCH, SOX18, HHEX, SL
DL1, CD300C, CD300A, COL3A1, SIGLEC7, OSCAR, CD8B, CD34, CD19, CD300LB, CLEC4G, MICA, SH2D1A, IFITM1, (

14, CEMIP, CADPS2, SRP54, GXYLT2, WHAMMP3, CCDC141, LINC00960, CRIP1, TLCD4, MGAT5, ETFA, NBEAP1, AF
ST14, CLDN7, ARHGEF16, CLDN4, CBLC, LLGL2, ELOVL7, EPB41L5, MARVELD2, OCLN, TMPRSS2, TJP3, BSPRY, PLE
MINDY2, PLXDC2, EPB41L5, OGA, WNT9B, ALOX12, CSF3, MIA2, SCML1, ZNF367, ABI2, MKNK1, ZNF366, ZNF365
STAC2, SCRG1, WFIKKN2, MRPL1, AR, SEPTIN4, SLC22A8, ENGASE, OVOL2, ELMO2, CARD10, ELMO1, GAPDH, INI
IAD6, GP1BA, H4C2, TREML1, LYZ, MSRB3, PLEK, CRACR2B, PBX1, ETS1, CD9, OGFRL1, STX1A, ARAP3, GFOD1, HE
, H2BC9, H3C1, H4C9, H2AC14, H2BC3, H4C5, H2AC8, H4C4, H2BC7, H3C7, H2AC4, H2BC10, H4C1, H4C2, H3C10,
S100A4, OLFML1, N4BP3, BACH1, ACOX1, BIRC5, MXRA5, ERCC2, PDGFRB, TEAD1, FBN2, UROS, NR1H3, SLC14A1,
IAP3K13, TPH2, FOXO4, IKZF2, STK40, MRPL17, GBX2, PRKAA1, THBS2, PCSK5, FOXD3, FLRT3, FZD8, IL3, IL5, ARM

71A1, WASF3, TIPARP, CNOT4, USP24, PDS5A, PAWR, CTBP2, BARD1, NUP98, CENPF, ORC2, ABI1, UBE2D2, OGT,

AKR1C1, APOL6, SLTM, UXS1, MAPKAPK2, COMMD8, RAI2, RAB38, EMP1, NRCAM, SEMA3C, HS2ST1, PTPN12, M
MNDA, SLAIN1, RNF144A, TDRKH, LRRC56, SLC25A4, SVIL, GK5, ZFP2, RAPGEF2, LMO4, PDLIM1, EMB, OXCT1, CD
S15A, WDR36, NAT10, LTV1, MPHOSPH6, IMP4, TEX10, RPL35, RPS6, RPLP1, RPS24, WDR12, RPL3L, RPS2, UTP4,
A, EFNA4, LYN, GRIN2B
N, TNC, VWDE, CFTR, TNFRSF11A, CHROMR, NCEH1, DHX9, PTPRR, TOP1, ENPP4, PTX3, ADAMTS1, MTHFD2, LU

3, RPN1, CDO1, DPP3, CYFIP1, TMEM200A, CXCL14, CLDN1, SERPING1, ITM2C, GPNMB, ADCY10P1, PDGFC, GFR
552, NUFIP2, PTK2B, DHX15, CFAP47, CAPN6, MAGOHB, DNM3, MAT2A, ANKHD1, ZFP2, CEMIP2, FNIP1, DUSP5,
GJA1, ISG15, IGFBP4, DDX60, ENPP2, OAS3, RXFP1, GCH1, SPTA1, MEF2C, PALLD, CNKSR3, NEGR1, SLC6A13, LXN,

02029, LINC00881, CCNL1, VEPH1, PTX3, SLC66A1L, SHOX2, RSRC1, MLF1, GFM1, LXN, RARRES1, MFSD1, IQCJ, M
R2, RASA4, MET, HSPB1, PSMA2, ABHD17B, FGF8, SHOC2, PSMD3, PSMD11, LAMTOR3, AREG, IL2, RAPGEF2, VW

P4A3, MATN4, SSU72, VTI1A, MDM2, HOXA5, ADD1, ZFY, RHOA, TNF, DUOXA1, NGDN, ACSS3, PPIAL4A, TNFRSF
OX5, FLT3, NPR3, CD3D, DHRS9, DEFA4, TBL1Y, SH3GL3, ZNF829, PADI2, PROCR, CDA, SLC35F1, INAVA, POGLUT2

4A, EMP1, EFEMP1, LAMC1, S100A13, RND3, ACKR3, SPRY1, CKS1B, GPX3, MEG3, FGL2, FSTL1, FXYD1, LGALS3BP,

A, ETV1, C8orf82, FOXN3, BCL6, PHLDA3, SIM1, WNT8B, JARID2, NLK, TSSK4, PPP1R10, PDZRN4, GRIA3, ITPR3, S

4, RCAN2, FADS1, H4C9, DHRS1, LAMC2, NCK1, PLCD1, LMO7, SIGLEC6, MYB, IFNG, SYCP1, IGFBP5, MYF5, C8G, C
RRC17, ACER2, CBFA2T3, TSPAN12, CCDC93, WDR37, RSPO2, YOD1, RANBP2, IGF2BP1, USP15, IGF2BP3, NKAPD
ARHGEF17, MMP16, KLHL42, OTUD7B, ZNF362, SRGAP3, C1QL1, SORT1, KIAA1217, OSBPL11, DOLPP1, ARL4C, C
MEM108, ZIC2, IL13RA2, GABRP, RASGEF1A, ZNF483, LGR6, COL9A3, UCHL1, OCA2, SELE, TSPAN8, TNFRSF11B, D

C1, APOBEC1, ITGA2, GPR87, CEP55, HSPA1B, BCL2L15, ST14, GJB3, SIX4, OLR1, BUB1, FGFBP1, VCAN, COL1A2, C

LE1, POSTN, SCUBE3, CACYBP, C1R, STXBP6, CCNE2, GMPS, RPA3, DOCK10, DPYSL3, ADAMTS6, HAUS1, HERC4, C

ID3, FEZ1, HSPA12A, RGS17, CPQ, HBG2, CA4, FOS, TFPI, SLC4A4, FGF13, GPC3, LRP2, GNG11, ZNF302, EYA2, MT
TF, GPLD1, CD151, NAP1L5, SORBS2, SPIB, SERPINA1, MAGEH1, SRPK2, ATP11AUN, ZNF282, ATP4A, PCDH11Y, P
B, CEP128, TSHR, LINC02312, LINC01550, BCL11B, LINC02352, TCF12, ATF7IP2, NFATC3, MEAK7, ACSF3, P2RX5, S

EAP4, TNFRSF12A, LINC01194, BZW2, PHLDA2, HSD17B1, DACT2, C4orf36, ERVH48-1, HSPB8, SP6, PSG11, PPP1R
AX, CLEC7A, HK3, ADGRE4P, C9orf72, P2RY13, CFD, TYMP, RGS1, SPIC, MS4A4A, PILRA, THEMIS2, CREG1, CCDC1
29, CAMK2A, TLX3, CAVIN4, SHOX2, HTR3A, CREG2, SLC22A9, ENTPD3, PRKAG2-AS1, LINC02605, PHEX-AS1, SER
V3, MFAP3L, LINC00504, MFSD2B, C2orf88, GFI1B, INHBA-AS1, ANKRD55, HPSE, MLC1, RHD, SMOX, RHAG, LINC0
P9, NCAM2, GJC1, CERCAM, METRNL, TMEM39A, ADAMTS15, C3, ZNF503, PCDH7, TCEAL9, CCN3, KARS1, FKBP1
3, MS4A6A, MAP2, TUSC3, KRT13, INHBB, NPTX2, PAPSS1, MDK, MAP4K4, MVP, ME3, MED13, PCSK2, DHCR7, E
NKRD9, ABCC13, SLC22A4, HBQ1, MFSD2B, STAM, SMIM1, HBE1, ICAM4, STEAP3, TSPO2, FAM104A, CTSE, LINC0
SIGLEC10, HK3, CXCL11, LRRC25, NFAM1, CALHM6, SIGLEC9, ST18, MARCO, C3AR1, LY86, LST1, GNA15, HLA-DR

B1, LILRA1, FCAR, SIGLEC1, SAMHD1, IFNAR2, LINC01678, HMOX1, NCF4, TLR8
SLC11A1, OSCAR, CYP2S1, CCL22, IL1RN, SIGLEC14, EBI3, CCR2, LINC01094, LINC02712, TLR7, XCR1, FPR1, P2RY6
6, ANKRD20A11P, WFDC10B, NT5M, CDK9, DGKI, GCSAML, LINC00534, INHBA-AS1, TTC4, ZNF852, DNAH10, ZN

, OSCAR, PDCD1LG2, TRPV4, HK3, NAIP, SIGLEC16, CD300LF, CLEC9A, FABP5, SIGLEC7, RASGEF1C, NLRP3, DSCAM
CSL6, ABCC13, TMOD1, GLRX5, CROCCP2, TBCEL, TBC1D22B, YOD1, AMMECR1, ART4, TMEM63B, FAM83D, SNX2

4A3, JUNB, TNS1, TGFB1I1, CCL2, GADD45B, HSPA1B, TCIM
7B, SLC38A11, CFI, C6orf132, VTCN1, LAD1, ST14, CNGA1, ARHGEF38, RNF43, SLC9A2, GRHL3, BICDL2, PROM2, R
GFRL, ADH1B, CYP4X1, NGF, METTL24, TCF21, C1QTNF7, LINC02643, BMP4, NPAS1, SRPX2, PODN, GATA4, KDEL
RP, HK2, HMGA2, JPT1, HOMER1, IARS1, IFNGR2, IGF2BP2, IGSF3, CXCL8, ILF2, INTS8, ITGA5, ITGAE, JARID2, KDE
NB1, COL22A1, SMTNL2, PFKM, LRTM1, TRIM54, PPP1R3C, TECRL, MB, LANCL1-AS1, MYH2, ITGB1BP2, PRR32, C
MMECR1, ANKRD9, SLC14A1, RNF224, DCK, CHST2, ACSL6, SLC22A4, ABCC13, GBGT1, TCP11L2, MFSD2B, GYPE, I
L1, IKZF2, EHF, HPSE2, LRRTM3, BCL11B, LRRTM1, BCL11A, HTR3B, BUB3, GDI1, DLG2, PDZRN4, GRIA3, FZD2, PT
AQP10, LEFTY1, EXOC3L4, CCDC175, BEND2, H4C8, LINC02470, HGD, XIRP2, C15orf54, KCNMB1, CRISP3, ITGB1B

2A16, EIF2AK1, ABCB5, DBF4, TFR2, NAPEPLD, CBLL1, KEL, XPO7, DMTN, SLC25A37, BNIP3L, WRN, ANK1, CA1, SM
3, CCDC170, P2RY13, GNA15, HLA-DRB6, SIGLEC11, EAF2, CD209, TEC, RENBP, IGSF6, SEMA4A, IRF5, P2RY6, TLR6
3, LINC00426, IL2RA, CRTAM, TCL1A, P2RX5, IL18RAP, CST7, CD8B, FCRL2, KLRC2, CHRM3-AS2, CORO1A, CD160,

NEURL1-AS1, SLC22A8, SLC5A9, NEURL3, CTD-2297D10.2, SCNN1A, SALL4, KRT4, CALHM3, TMPRSS3, MUC20, FO
1F, HLA-DMA, STXBP2, ITGAL, CSK, PTGS2, LILRA5, ADA2, CLEC7A, CHD1, S100A8, CD300E, SOCS3, MX2, LRRC25,
X2, SPATS2L, NTSR2, EFEMP1, PRODH, ETNPPL, MARCKS, GPC5, ACKR3, MT1F, LAMA4, ITPR2, NFIB, RORA, FUT9,

ID, THBS1, LOX, LOXL2, ADAM9, PLAUR, CAVIN1, F2R, FLII, LRRC32, LDB2, TEK, POSTN, VCAN, BMP1, PLPP2, APOA

IRF, HSPG2, RNASE1, CCND2, LGMN, SERPINE1, EPHX1, CLEC3B, G0S2, IGFBP3, GAS1, NR4A1, APOE, RBP1, IFI27,
JUNB, CCND1, TIMP2, LTBP1, IGF1, SLC23A2, SDC1, RHOBTB3, PLEC, MFAP4, SERPINH1, CRIP1, TNXB, LPCAT1, DE

G1, FGFR4, DEFB4A, TPSAB1, AKR1C3, TFF3, ELF3, KRT5, MAT1A, CRYM, ALOX15, CD6, AZGP1, TFF1, GPX2, PRSS8,
LMTK3, GALNT3, ZFP41, GSTM4, GATA3, TSC22D3, SLC5A4, RHOBTB1, CYP26A1, NCF1C, FGG, CXCL14, NBL1, H1
SERPINH1, BIRC2, SLC39A6, ADIPOR2, CALU, CHPF, SEC11A, DDX21, GYG1, UGDH, EFNA2, SCP2, TSPAN3, GBE1, N

PPP3CA, ACSL4, FBXW11, MTMR4, USP32, BRMS1L, CSRNP3, ATRN, SPTY2D1, IGF1, PPP1R10, RICTOR, LMX1A, PK
B5P, XPNPEP1, SH3BGRL, DBT, TMEM47, WDR26, SP3, BNC2, METRNL, SP8
GZF1, GLCCI1, PPP4R4, ZFAND5, GTF2H1, BRWD1, TENT2, XPR1, SRGAP3, ZMYND8, WDR44, KSR1, DOLPP1, RAS
21L2, SOBP, ZNFX1, GFAP, BACH2, FOXA2, ELF4, MAST4, GPLD1, ISG15, NAP1L3, YBX2, MECOM, PBXIP1, MAPT, Z
, CLIC1, CIDEC, MMP13, MMP12, KLF9, DOK1, GAPDH, EPB41L1, TAF15, SERPINB5, SERPINB7, SERPINB2, CSF3, EI
CLIC4, CLK4, CNOT7, COPB1, COPG2, COPS8, CORO1C, COX7A2L, CPD, CTDSPL, CYP4V2, DAB2, DEK, DENR, DHX2

, DEAF1, CSTA, S100A10, TIMP3, LAIR1, TOM1L1, PKIA, GIPC1, AKR1C1, ANXA5, LARS2, NR4A3, NET1, ILF2, GABRE

AALADL2, TMEM164, HOOK1, THBS1, SULF2, TPM1, NUP50, NIBAN2, ST3GAL2, SBK1, COL12A1, PTBP3, DDX39A,
G2, CDC42BPB, GAB2, ZNF780B, SETX, UNC79, FBXO41, MGRN1, PCLO, FAM189B, VASH2, RPS6KC1, PTPRU, ZNF4
4B, ARL8A, OPA1, DLG3, ATCAY, CLVS2, PTPN5, TCF4, NCAM1, NETO2, PTPRO, DPYSL2, ZNF324, LPAR2, RNF40, AD

36, LINC00112, OTUD7B, MAP2K3, HSPA1B, RNF144B, ADAM3A, OR51B5, TGFBR3, TMEM67, PURB, DNAH14, PA
TRAF6, KBTBD4, EHD1, FOSL1, LTO1, PGM2L1, CREBZF, MIR100HG, OR10G7, PTMS, ENO2, GPRC5A, RASSF8, TME

A, ULBP2, COLEC12, PYM1, ATP8A1, UBE2N, GALNT10, C2CD4D-AS1, SH2D4A, SFMBT2, HOXC13, RSAD2, RND3, T

RD13, ALDOB, CADPS2, FKBP5, MED13L, MYO1E, RFX3, HSP90AA1, CALD1, APP, ARHGEF3, KIAA1217, CEP112, UA
3H1, SEC63, RMND5A, PTAR1, CTAGE15, PRKAR2B, CPNE3, SLC30A4, MRTFB, OAZ1, MYRIP, HTATIP2, CHST11, PP
ER, DNM3, DOK6, DOT1L, CTSC, DPYSL3, DSCAM, DUSP26, DUSP4, EFCAB7, ELAVL2, ELAVL3, ELAVL4, EMX2, EMX
L, BRD3, ZDHHC14, MAP4, ALDH1A3, RHOU, KRTAP1-3, XDH, ABCE1, C22orf39, WDR75, RPS17, MBP, ERC1, PIGQ
27, STARD13, RAB43, HPSE2, BCL6, ABCA12, MOGAT2, PI4K2A, PNLIPRP1, NAA80, TBX5, ELAVL4, FZD4, CAPN1, AS
1, COTL1, B3GNT3, MPZL2, ETV5, TIMP2, RAI14, CAPN5, AKR1C2, NPC2, TRIM29, NT5DC2, PCDH1, EPHX1, BCL3, I
1A, LRATD2, FAM89A, FANCB, FANCI, FBLN2, FGFBP3, FGFR3, FOXL2, FOXM1, FRMD6-AS1, FUT9, GABBR2, GATM
BTBD9, FAF1, TCF12, ARHGAP6, SPIDR, DYNC2H1, OSBPL3, MAPK10, PRKCA, EFNA5, EPB41L3, LRBA, SBF2, LAMB
CXADR, DAAM1, DACT1, DCLK1, DCX, DDC, DGKE, DIRAS3, DLG2, DLG4, DLK1, DMTN, DNASE1L1, DNER, DNM3, I
EP3, CDC45, CDC6, CDC7, CDCA3, CDCA4, CDCA5, CDCA7, CDCA8, CDCP1, CDK1, CDK2, CDKL1, CDKN3, CENPA, CE
GB1, RFX3, GPM6A, UBL4B, AFG1L, TMPO, SYT4, TMEM33, NHS, NAV3, GORAB, MON2, CNKSR2, SLC16A1, YWHA
, TBL1XR1, CPEB2, FUT9, DUSP16, RADX, UTRN, PRPF39, ZDHHC23, KCNN3, CCDC88A, SLC16A7, ACVR1C, CADM2

, TMEM163, RASGRF1, SEMA7A, KCNS2, AHR, TP73, DMRT3, BNC1, CABP7, PPP2R2C, MAP3K21, CADPS, PDGFB,
C1QL1, TLX1, MAL2, B3GNT7, HOXB5, GHSR, EMX1, DLGAP2, TNFRSF1B, SGPP2, TP73, TMEM150C, SKAP1, BCL11
SGTB, CARHSP1, PRKCE, TNC, IGFBP2, WFDC2, COL16A1, HSPB8, CSPG4, SDC1, ELF5, C2CD2L, MRC2, SLC25A35,
CENPU, E2F7, PRR11, ARPP21, FAIM
TSHR, SNCG, PC, LCP1, PURB, CP, CLNS1A, HACD2, RASSF3, CER1, BCL2L13, HCAR3, BTC, CCND2, CD36, LTC4S, TE
ZBTB20, SEPSECS, LMOD2, SH3D19, DISC1, XYLB, HSPA1B, TENT5C, REST, MTO1, PGAP1, PPIL1, RIC3, ZNF480, CL
W, JPT1, HS3ST1, IGFBPL1, INA, INHBA-AS1, INSM1, KCNH8, KCNQ3, CRACD, KIF21B, KIF5C, KLC1, KLHL35, LCOR,
PTOR, MSANTD3, CELF2, SPATA6L, SVIP, AK6, MLX, SCN3B, KCTD12, TTN, INTS6, SF3A3, DDX6, GSPT1, FBXO21, A
CNP, NFIL3, TEKT3, RCN1, UBE3A, SMC1A, ALX1, SH2B3, RIPK2, SNRPF, AMER3, SNX13, CYLD, ASAP3, DENND6A,
DDHD1, CYBRD1, LYPD6, DPYSL5, ANKRD17, CFL2, TNFRSF21, TSG101, ZXDA, MASTL, PCDHA12, SRCIN1, FAM126
K2G, DCLK1, MLX, CREBBP, SENP8, KATNA1, RNF182, ZNF607, GSPT1, RBFOX2, HTR1F, PPP1R15B, PRTG, DPYSL2,
DSB, C17orf58, RBMS3, SMNDC1, PHEX, MLLT3, TEKT3, GRAMD2B, KRAS, ACER3, ZNHIT6, TM9SF2, ZNF33B, C2or
FAM135A, ZBTB1, RASGRF1, POLR3G, PHF21B, RCAN3, HMGB1, SERTAD2, ZNF772, CEP135, GAGE1, GPR34, GPR
LA, RBPJ, RIOK3, FOXJ3, WWC2, DSC2, RAB23, UNC13C, SLC2A5, MAP2, KLF3, TNKS, KCNS2, ARID1A, SLC45A3, EF
ADX, TNKS2, PUM2, UTRN, ANKRD62, CYLD, GALNT13, URB1, CADM2, RBM41, RAD50, ARFRP1, CYP26B1, SUZ12,
SC, SOX30, RAD23B, RAD54B, LRP5L, SMIM14, ZCCHC10, TBL1XR1, POU5F1, HAUS6, FUT9, TRERF1, DUSP16, GPR
C, TMEM178A, TSHZ3, PPP1R3C, CDH13, TGFBR3, GNG11, ENPP2, AXL, HTRA1, ACTA2, JAM2, SOX11, CD70, RASL
APK1, PTPRD, ZNF367, CHIC1, RBBP8, RIMKLA, DNAI1, SLC6A8, ARC, LY75, SAMD4A, LSM12, UBL3, MSMO1, TMB
ASAL2, PDLIM5, RERG, MAP3K5, CLMN, TTC28, APBB2, TNS1, NHS, THSD4, ARID5B, LRCH1, DST, UBR3, ARHGAP6,
2, SGO2, CISH, NEK2, ERBB3, ABCF2, HIP1, NRP2, ZWINT, KIF11, DQX1, PRRC1, CD80, RASSF5, CDCP2, SUSD6, EM

2, TEAD1, NRIP1, MGP, CUX1, CADM1, ADRA2A, MOCS2, DDAH1, GREB1, PDLIM1, C1orf226, TCN1, ADD3, NRCA
CTNNA3, RBM26, EPB41L1, SH3BGRL, SARAF, VPS35L, UBE2N, PRKX, PACC1, RPGRIP1L, ATXN7, AFTPH, RAB23, KL

, GLRA3, PRKCA, CHRNA3, CXCR2, ERBB2, TNFRSF8, IL12RB1, SLC26A3, MSI1, ETS1, ID4, MAPK12, HNF1A, RIN1,
NAB1, ZNF532, NR6A1, MITF, CBFA2T2, RUNX1T1, CRX, SPRY2, POU4F2, SIK3, UTP4, CAP1, NKX2-1, IGF2BP3, NR2
H11, NBL1, IFIT1, WFDC3, PTGIR, NPM1, SMARCA1, FSCN1, FUT2, BCKDHB, NID2, LHFPL2, STAP1, TBXA2R, CD1E,

P2CA, FOXN3, BUB3, WNT8B, TBX6, FLRT1, DLGAP4, PHF12, MID1, TMPRSS3, EXOC5, RBFOX1, POLDIP3, ATP6V

EF7, PGAP1, MMP14, MMP12, ELMO2, C8A, CCND1, YPEL4, SLC26A7, ATL3, UHRF1BP1, NSD1, UBASH3B, PART1,
C25A1, ELOVL6, TMEM106C, COL9A3, NME4, FCGR2C, MICB, RHOBTB3, SHMT1, CIDEB, NCAPD2, MEX3D, PSIP1,
CD300LF, CD300E, LILRB4, LILRA5, TREML4, NCR3LG1, SELL, KIR2DL4, NCR1, SH2D1B, CD247, CD3E, FCGR3A, CD3

RF3, GPR160, AKR1C3, ZNF395, MMP1, EXOC6, ITGB8, KLC1, TSPAN8, CSGALNACT1, ANGPTL4, ENO2, DHRS2, TRI
KHH1, BCL11A, MYO5B, CD24, CLDN1, GJB2, TMEM54, PPIF, IDE, DEDD2, CALML3, DSC2, CLIC3, EVPL, CYSRT1, O
, CIPC, ARL6IP5, SORBS1, LRRTM4, STK40, FMO3, CALCOCO1, SERPINB10, ARMCX2, USP36, SYNRG, PHLDA2, USP
HBA, EIF4A2, C12orf42, CLIC6, URI1, WNT9A, C9, SCML2, OFCC1, PART1, ABHD2, SPO11, PID1, CFB, SORBS1, LRR
XIM2, GAPT, XYLT2, UBASH3B, PADI4, GP6, PTGIR, TMEM163, CLDN5, KLHDC1, CARD19, NINJ2, ACOT11, SUSD3,
H3C2, H3C3
PRKAB2, THBS1, PRKG1, CALD1, KCNMA1, RAB4A, S100A10, RHOA, ITGA2, CPS1, TM7SF2, CDON, PARVA, FADS1
C6, DLGAP4, LMNA, PDZRN4, KRT28, ELAVL4, PDE2A, BNC2, CLRN1, DPYD, DUSP9, APBB1, NHLH1, BCAR3, LMO3

MDN1, WASHC4, SOS1, LARP4, ATP2B1, PUM2, ATR, FOXJ3, WDR7, FLNB, DOP1B, CLOCK, NFIB, CRY1, KDM5A, N

FAP3L, PHTF2, CLUAP1, PEG10, NUDT4, ESR1, EEF1A2, CDH3, NIPSNAP2, THAP9-AS1, RAB31, EIF1AX, ATP8A2, ZB
DIP1, H1-0, FGFBP3, EPB41L2, RERE, DOCK2, RGS19, PLXNA2, MARCKSL1, LCP1, PDE5A, NUBPL, JCAD, ZNF518B, T
NOB1, PELP1, CSNK1D, RPS11, RPL13A, RPL11, RPS8, ISG20L2, RPS27A, RPL32, RPS3A, RPL37, NHP2, RPL10, RPL

C7L3, C5orf24, HSPD1, CLK4, DHRS2, SAMD9L, HSP90AB1, PTPRG-AS1, CNR1, GJB6, DYNC1I1, THRAP3, CEP295, PI

A1, ANTXR1, ANTXR2, ZCCHC24, RAD54L, MTHFD1L, CDH11, STS, NR2F1, ABI3BP, RPL35, VEGFC, PPP1R14A, TPM
PKNOX1, PHF19, NAA15, MCFD2, BNIP3L, USP46, MPPED2, ZBTB2, HTR2C, WDR20, LRP5, MTSS1, PRPF4B, ATMIN
NMU, ADCYAP1, FLT3, IFITM1, CTSG, ANGPT1, IFIT1, HOXA7, BDNF, EIF2AK2, HBB, APOC1, RPS6KA5, HERC6, JCH

IIR3919, IQCJ-SCHIP1-AS1, SCHIP1, IL12A-AS1, IL12A, LINC01100, C3orf80, IFT80, SMC4, MIR15B, MIR16-2, TRIM
F, PSMD9, FGF6, DUSP16, RASAL1, CCND3, PPP2R5D, HBEGF, RASGRF2, IL5, PPP2CA, FGF1, PDGFRB, PSMD14, SP

4, CCDC80, CALN1, RGS6, SDR16C5, IGF1R, MTMR4, SERBP1, ZNF804A, CDH13, GGA1, NHLRC3, GSG1, HOXB6, C1
2, WDR36, CHML, SLC43A1, FBLIM1, OXCT2, CRIP2, FAM241A, GABRA4, LINC02239, ABHD14B, GRB14, PAWR, PD

, TIMP2, ADD3, SELENOM, CD63, HSPG2, EPHX1, ANXA2, LIMA1, PRDX4, MAP1B, AKR1B1, FHL1, DAB2, ARL6IP5,

GTB, FGGY, DUSP4, NRAS, UBTF, MEOX2, CFAP161, C12orf50, GSK3B, NHLH2, ZNF462, NLN, CDH10, LIX1L, JDP2,

OPN1SW, PPARG, MMP16, RRAS2, FAP, REEP5, IFI35, VDR, ACTR1B, KLHL9, RNH1, COL6A2, FRMPD4, TBCD, SEC16
1, ZCCHC3, CCNJ, SOX11, OTX1, SOX12, ARHGAP28, CCDC47, GAN, HMGA2, HMGA1, LCORL, LCOR, ACVR2A, MAN
LOCK, RASD2, TBC1D8, ZNF655, ZNF365, ZNF654, ITM2B, ITM2C, PCSK2, SORBS3, DGKB, SNRK, LRRTM4, ARMCX2
MD, CRACD, CXCL5, ZNF711, TMSB15A, MOXD1, STAR

C1GALT1, CENPE, PTK6, DLGAP5, GPRC5A, KLK6, ELL3, MMP12, TPX2, SMPDL3B, CBLC, SPOCD1, CKMT1B, KLF5, S

DC7, KIF4A, NSD2, CDCA8, ASNS, THSD4, SGO2, ADAMTS2, ANKRD13A, FOXM1, BGN, TBL1XR1, MCM6, CEP57, K

TMR4, PLCH1, MEOX2, SEMA3D, CSRP2, PRKY, RAP1GAP, GPER1, TOB1, NCAM1, APOD, EML1, TSPAN7, RERGL, F
CDH11X, TNMD, TLE4, SERINC2, ZNF334, ADTRP, RPL41, GIPR, ORMDL3, GANAB, KCNIP3, USP5, SCML1, CPN1, M
SH2, ARL5C, MAP3K14-AS1, TNRC6C, LINC01882, LDLRAD4, PRKCG, TOX2, MIR646HG, LINC01749, LINC00649, U

13L, DEPP1, LINC00470, ZBED9, XAGE3, CBR3-AS1, CSRP2, NFE2L3, TPRXL, ARHGEF5, MAP11, MAFF, SERINC2, LI
70, NEXMIF, MX2, TNFAIP2, PDCD1LG2, FUCA1, SMIM35, SLC15A3, C3AR1, SPSB4, GAA, LRRC25, IFIT2, PHKA1, H
TAD4-AS1, NUDT10, LINC02074, ADCY8, LINC02269, RORB-AS1, LINC01539, FAR2P1, NRG1-IT3, LINC02525, LINC
0989, SLC39A3, PLEKHF2, LEFTY1, WFDC1, CXCL3, XK, GMPR, CTNS, RTN2, AQP10, UBL4A, PLXNB3, KEL, PTGER3,
4, RCN3, CRABP2, HCG11, FGF18, FAM227A, EMILIN1, CDK2AP1, LINGO2, PI15, ADAM33, S1PR3, CREB3L1, CLEC1
LF3, CHIT1, CCL17, TMSB10, ANXA2P1, TGFA, MALL, PLXNC1, KCNQ3, MAP3K6, PCDH7, RREB1, SLC6A1, RHOBTB
2506, SLC22A16, YPEL4, YOD1, KRT1, PLEK2, MYO18B, TF, C9orf40, GALNT5, FLACC1, PAQR9, CDC25A, SLFN14, T
35, TNFAIP8L2, PADI2, LACC1, RYR1, LAMP3, PRAM1, RAB39A, ANKRD22, KCNE1, BCL2A1, HLA-DOA, PPM1M, TLR

, CD209, SLAMF8, CD1D, CLEC4A, CD300LF, CLEC5A, PADI2, C19orf38, FGL1, LINC01605, NOD2, SIGLEC11, CD1E,
425, LEFTY1, CLEC1B, UPK1A-AS1, LINC01786, TXNL4B, MLIP, ALOX12, MYH7B, XIRP2, YTHDF3-AS1, PDE6A, ZNF5

/I-IT1, LINC01645, IDO1, CARD14, BATF3, LILRB3, TMEM63C, IL1RN, ANKRD24, CD274, TRIM36, CXCL10, AGR2, M
22, ATG14, PHOSPHO1, ICAM4, ANKRD9, SLC43A1, UROS, SLC36A1, SBF2-AS1, FLACC1, SMIM1, RFESD, GFI1B, RN

ASSF6, NECTIN4, EVPL, VSIG2, TMC4, GRHL1, ITGB4, TSPAN1, CALB1, ATP10B, CPXM2, MYEOV, CTXND1, GPX2, IT
LR3, ADAMTS9-AS1, MYOCD, LINC01031, ADRA1D, BPI, ADAMTS14, FHL5, WT1, TDO2, WT1-AS, FOXS1, EMX2OS,
LR3, KHDRBS1, KIF11, KIF15, KIF20A, KIF23, KIF2C, LAMB1, LAMC1, LAPTM4B, LBR, LGALS3, LHFPL2, LMNB2, LPC
CXCL13, RRAGD, SHISA2, ACTN3, CYP2J2, ANKRD1, PACSIN3, MYBPC2, CARNS1, LRRC38, DPYSL5, TMEM52, SMTN
BPGM, TBCEL, STRADB, NFE2, PIGQ, UROD, KLF1, UROS, SEC14L4, PHOSPHO1, DHRS13, EPOR, PPOX, ALAD, HBE1
GFR, ELAVL4, MID1, GJB1, P2RY12, NRAS, LRP1, CSNK1E, NEUROD2, NHLH2, PTCH2, NEUROD6, TOB1, DNAJC5B,
P2, PCP2, CFAP45, DNAAF3, TUBAL3, GRM3-AS1, CMTM2, CHRNA2, CAPN11, MCEMP1, C1orf116, LINC02701

IILR, RECQL4, RCL1, ANKRD18B, DCAF12, C9orf40, ZNF367, HEMGN, ANP32B, ALAD, GAPVD1, MIGA2, UBAC1, QS
, MCOLN2, NOXA1, C2, CASP1, PLB1, NFAM1, DNASE1L3, PIPOX, CYTIP, LACC1, GPBAR1, ST18, ZEB2-AS1, PLA2G7
GPA33, LAX1, GZMK, FCRLA, LINC02325, CR2, LINC01222, XCL1, FOXP3, RAG2, SH2D1B, KLRK1, KLRF1, MEOX1, TI

LR1, FUT6, HABP2, SYT8, AGR3, A4GNT, FAM245A, CLCNKB, SLC17A4, SPEF1, LINC01836, TMEM229A, KLHDC7A,
NAMPT, LRRFIP1, EFHD2, TMEM176B, LST1, TRIM25, TMEM131L, SIN3B, CFD, CXCR4, WARS1, NLRP1, KLF13, HSI
PCDH9, MSMO1, BBOX1, SLC25A18, ALCAM, C9orf24, ANOS1, DDIT4, MT1M, B4GAT1, LAPTM4A, ST8SIA1, NFIA,

A1, ADNP, SERPING1, C1R, SCG5, SPOCK1, FZD7, RGS16, PFKFB3, PLAU, MMP11, BASP1, MOXD1, HOXA5, COL18A

PNRC1, DLK1, ZBTB20, SNCG, CCN1, DBP, LDLR, RHOB, CXCL12, CRYAB, NUPR1, SOD3, CCL2, MAOB, JUNB, CCND
ENND2B, HES1, CDC42EP1, CALU, TFAP2B, CHPF, ACTG2, PPP1R15A, PLXND1, CD34, PDPN, ROR2, RBPMS, FOSL2,

, ACR, MYH2, MYL2, CPD, ST3GAL4, TCL1A, ITIH1, PDCD1, ARL4A, DCAF7, HOXB7, CSRP2, GCH1, LAD1, FXYD2, CH
9, CTSE, PSG5, EGLN3, VAV3, FAM83D, GINS1, E2F8, CDCA5, GGT1, PXMP4, WWC1, FXYD3, GAL, KRT17, KRT6B, C
MSX1, PSMC2, CAP2, TFRC, SCHIP1, MLLT11, NNMT, SOX10, DDIT3, CYB5A, GDF15, S100A4, RBP4, DDIT4, ALCAM

KNOX1, BNC2, ZBTB4, SELENOI, DUSP8, WDR20, DNM2, STK38, STYX, TSPAN9, TNFSF12, CNOT7, CNOT6, MOB3B,

D1, EIF5A2, NUFIP2, NAGPA, FAM131B, RGCC, PER2, RAB11A, USP37, TMEM121, FOXD1, RASA1, BRD1, CAPN5, N
ZNF654, ANP32A, SMTNL2, WDR47, KCNT2, GHR, FMNL1, ZDHHC1, GIP, ACO1, ARMC8, ANP32D, PGAP1, ARHGEF
PHX4, ABHD4, ABI3, ABHD2, IL7R, SH3D21, COL7A1, LRRTM3, STK40, ACSL4, ACSL3, SNAP25, ETFB, PHLDA2, WNT
29, DIAPH2, LINC01011, DLEU1, DNAJB9, DNAJC15, DYNLL1, DNM3, DOCK11, DPY30, EBAG9, GPR183, ECHDC1, EI

E, NAE1, FGFR3, CFP, SFXN3, TRIB2, CLCN4, TNFRSF4, AMD1, SQLE, MAX, SCRN1, CASP1, GAD1, LGALS1, NAP1L3,

ARMH3, NDUFB8, DYRK1B, PREX1, CIT, POMT1, RELN, UBL3, ADCY1, DSCAM, AGR2, PMEPA1, TSPYL5, ISOC1, OLI
145, PDP1, CACNA1A, EXD3, AGAP2, CLDN5, CAMKV, STK11, RIMKLA, PSD, TMEM108-AS1, TRIM46, SVOP, HMGA
DCY1, BEX1, CDH10, NSG2, FADS3, MAP4, KCNA6, SGSM2, FCHO1, TUBB2B, FBXW7, TARS3, RAMP3, AMPH, SYP,

CRG, PALM2AKAP2, SVEP1, PLAUR, GFER, KRT6A, FGFR3, SEPTIN7, GLYATL1, SBSPON, IGSF3, HBA1, MPP3, FAM7
EM117, ATF1, ATG101, SRGAP1, SOCS2, ANKRD13A, DDX55, GDF3, SMCO3, CPNE8, SLC38A2, CCNT1, CSRNP2, TA

TAPT1, CINP, ELL2, USP54, NUAK1, SMIM5, OTUD7B, JAG1, TENT4B, TMEM41B, RAD18, CSNK1E, DENND3, ATP2C

ACA, QKI, MACROD2, EXOC6B, JMJD1C, GPC5, MACF1, SBF2, PRKCE, NFIA, TJP1, ZSWIM6, FMNL2, DYRK1A, PAN3
P1R3A, BTG2, TMEM67, CKS2, CEP350, AGL, MAPK8, SMAD2, TEAD1, FGFR1OP2, PTPRC, MBD6, GNB4, NDFIP2,
2OS, ENC1, ENO2, EPB41, EPHA3, ERC2, ESRRG, EYA1, F11R, FABP3, TCAF1, FAM117B, FAM155A, TAFA2, FAM21
, DYNLT1, RNF149, SDHAF4, CALD1, RAB12, PIGB, RASL11B, HLA-B, PFN2, ETHE1, RHOG, CEBPG, CYP17A1, ESRP1
SIC2, SAMD7, NAA38, FYN, PTP4A1, BNC2, RBFOX1, ATP6V0A1, GAS2L1, BAMBI, NR5A2, CALM2, TOB1, GFRA3, SL
TGB4, BMP7, EDN1, TUBB2A, PCDH9, GOLM1, SIX2, PACS1, ACSL1, RAB5C, PDLIM7, GDF15, ST6GALNAC2, PTPRU
, GDPD2, GINS1, GLTP, GMPR, GNG12, GNG7, GPM6A, GPM6B, ADGRG1, GRAMD1C, GRIA1, GRID2, GRIP2, GRM
1, SHROOM3, CD2AP, ITGA2, ITPR1, KIAA1958, KMT2C, STARD13, SLC16A7, TRPM3, FNDC3A, FAM172A, NEBL, SC
DOCK4, DOK4, DOK6, DPP6, DPYSL3, DSCAM, EBF1, EBF2, EBF3, EDIL3, EEF1A2, EEPD1, EHBP1, EID2B, ELAVL2, EL
ENPE, CENPH, CENPI, CENPL, CENPM, CENPN, CENPO, CENPP, CENPQ, CENPW, CEP128, CEP135, CEP55, CEP57L1,
AQ, PCSK6, ZZZ3, ARL5A, RBM15, TSFM, CD226, HSDL2, LPP, ZNF235, BACH2, PAPPA2, RNF217, SBNO1, TBX22, KC
2, MARF1, TSHZ1, SUZ12, CADPS, CDC42EP3, ZNF512, AP1S3, CDK1, MTMR7, GOPC, ZNF507, ASPH, DERL1, GPR63

SOWAHB, GSX1, TBX1, SLCO4C1, AGPAT2, TDRP, HRK, STXBP2, IKZF3, ONECUT3, FOXD4, SPIRE2, FOXE1, GABBR2
A, DMRT2, NEFH, ATP12A, CALCR, MESP1, TMEM132E, WNT3A, GSX2, RPRML, GABRG3, FGF3, NEUROD2, PENK,
FSCN1, UHRF1BP1, COMT, MAPK8IP1, LRP5, CABLES1, NFE2L3, CYP2D6, MYB, CLU, PI16, LALBA, PROM2, JCHAIN,

NM4, KAT7, GZMB, ATM, CAMK4, COL1A1, TFDP1, CACNA2D1, CENPB, TNFRSF19, MT1X, PFKFB3, CAV3, APOA4,
PX, CRISPLD1, CYP7A1, INTS14, ATAT1, CTBS, FRS2, PAX6, RBM12, SMAD5, CRIPT, TMED4, CNOT2, CNTN5, TXNRD
LMOD3, LPAR2, LRRC55, LRRTM2, LRRTM4, LZTS1, MAB21L1, MAML3, MAP1B, MAP2, MAP3K9, MAP6, MAPK6,
SPH, TFRC, PAPOLA, LYST, METTL4, HPS1, CLEC12B, XRN1, OPCML, C15orf40, CDH7, RECQL5, C2orf73, CAMK4, G
DNAJC6, SLC5A3, ATL2, ZNF519, INO80D, ANKRD34B, NEXMIF, CSE1L, SLC35D3, H2AX, ENGASE, BBS7, SRSF2, JMY
B, STXBP5, ARID4A, TMEM168, PRR15, ANKH, ZFAND4, RAP2C, RORA, PAPOLA, TAGAP, GUCY1A1, E2F5, PRR16, S
ASPH, ARHGAP9, LYST, IGF2BP2, ERBB3, CHIC1, GABARAP, EHF, TTL, GTF2I, SETD6, ZFP30, RBMS3, BHLHE22, LCF
rf68, SLF2, POMP, NEDD4, TEX2, CALM1, LEPR, STARD13, RAB2A, USP37, HMMR, MICOS10, HIPK3, TJP1, CHRNA9
85, IQGAP2, MON2, VPS4B, DDX59, KIF21A, ZFHX3, KDM7A, MTRF1, MDGA2, WDR33, RRM2, CACNB2, KLHL7, W
CAB14, RHOXF2, FAM171A1, PTF1A, EML5, TLCD4-RWDD3, RAB6B, NOB1, MTM1, CDK12, DUSP22, TMEM86A, G
SLK, XPOT, SHCBP1, CARMIL1, ZNF507, ZNF417, SV2B, HNRNPH3, UGDH, IRAK4, SUMO2, FAM160B1, CREB1, SLC
R65, MASP1, ZDHHC23, ATAD2, FAM13C, PLCG2, ZIC3, RAB2B, SLK, WDR47, CEP97, FMR1, CAST, BRAF, PDE7A, CE
L12, MAOB, JAM3, SNCA, NECTIN3, ANTXR1, GNAI1, FABP5, ITGA9, VSNL1, UCN2, PGF, SORBS1, ARHGAP20, KLHL
IM6, SH3D19, SECISBP2L, NAP1L2, PARPBP, RBMS1, CCND2, PLXNA4, REST, WDR20, MKRN1, ABR, IVNS1ABP, ZBT
HIP1, ANKRD36, PBX1, PDE1A, FTX, CAMK2D, PTEN, COL4A2, FER, AKT3, SIK3, RAPGEF2, MYH9, ALDOB, COL4A1,
P2, JPT1, KIFC1, CDCA4, PEPD, CCSAP, PRRX1, GTSE1, ZNF286A, ATG9B, RRP1, FAM50A, PLK1, SLC9A3R1, ST8SIA3

M, ELP2, RHBDF2, ZNRF2, ANXA6, IRX5, PNPLA3, CXCL12, SYBU, SLC26A2, CCNA1, SUSD4, IGF1R, RNF167, MYBL1,
F3, SON, RGS13, C4orf3, RHBDD1, CDK15, SGCD, AKT3, BICRAL, NNT, GABRB2, PPP1R3B, PSMD11, SLC9A4, UNC5

AKAP6, CCDC106, STC1, XCL1, NR2E1, GJC2, CTSZ, SOX21, MMP14, PRDM1, SLC7A8, CGA, ASL, MAD1L1, CCL8, FG
F1, ADAM11, NOL4, RAP2C, ACTA1, TP53, CDK5, FLNA, ELMO1, INHBA, FAM13B, BSPRY, EYA1, KIF1B, SYT9, SHKE
SLC24A3, CD200, IFI44, GABRE, ITM2A, RPS21, TEAD4, OLA1, CAV1, POU4F1, CYTL1, CSAD, IFITM1, PPP1R16B, R

A1, BAMBI, FGF19, LMO1, C17orf102, AP1G1, LMO3, LMO4, IL19, RPS6KB1, FGF13, JAG1, GDAP1L1, DAB1, CDKN

SFN, NEO1, CLDN14, ZFP36L1, ZFP36L2, CYLD, FMO5, LRRTM3, LRRTM1, PER1, FOXN3, NUP210L, ETV6, TUT1, M
SLC7A2, ARRB1, TIPIN, HACD1, HMGN5, MCM10, BHLHE40, SV2A, SLC29A1, SREBF1, FZD6, HILPDA, BFAR, BRCA2
00LD, NCR3, MICB, HLA-C, LILRB3, HLA-E, HLA-G, HLA-B, HLA-A, KLRK1, LILRA4, LILRA2, KIR3DL2, KIR2DL3, LILRA6

32, TPM4, MCOLN3, TTN, NSUN6, RAP2A, KMT5B, FAM131B, SLC22A23, SPRY4, TM4SF1, SLC14A1
VOL1, ENDOU, BLMH, CDH3, PLEKHG6, EPHA1, EFNA3, KRT15, GSDMA
31, FOXD3, FAM27E5, ABCA10, MB21D2, CS, IL9, AXL, LMNA, NTNG2, FAM169B, CYP4V2, PHF12, LMX1A, ETF1,
TM3, LRRTM1, SERPINC1, USP34, SNAP25, ACSL3, USP32, WNT8B, ACSL5, USP31, MYF6, MB21D2, CSRNP3, MYF5
NFIB, BATF, SH3BGRL2, PDLIM1, PLEKHO2, IRF8, SBDSP1, PDIA5, LHFPL2, TMC8, GJA4, LGALS12, PCYOX1L, C5, BI

, FGF8, RDH5, TAF1B, FDPS, ACTA2, PRIM2, HIPK2, AP4S1, SGCB, FBN1, PDE1C, PRSS23, MMP1, LAMC1, PTPN13,
, RIPK4, FGF12, JAG1, ZIC1, ZIC2, HOXC6, FHDC1, GATA6, ANK3, ZMIZ1, ZNF579, TRMT112, SLC25A26, TFAP2D, Z

COA3, ZDHHC17, ATRX, PARD3, CASK, SPEN, SRSF7, THBS2, MYC, SRPK2, KRIT1, RBMS1, FARS2, BDNF, RFTN1, M

;TB18, STEAP3, CLK1, SP3, ATP2C2, ALCAM, SLC35A3, INSIG2, WAC, TCEAL9, GJA1, ATXN1, LGALS8, SCCPDH, PTPN
JP2, SPRED1, SLC39A8, UBE2E2, ZC2HC1A, SEPTIN4, PNPO, PDE4B, PRSS50, ATP8B4, CNTLN, PTPN18, TTC8, GPR1
7, RPL7A, RPP30, PDCD11, RPS3, FAU, DHX37, RPP38, RPL30, UTP14A, RRP1, RPL8, SENP3, RPL26, RPL29, NOL9, F

HLDA2, LPAR3, LMO7, POLR3G, IGFBP5, HNMT, TMEM158, PDC, XAF1, SPIB, RASSF6, CDC25A, SPIC, NEAT1, CXCL

1, MIR34AHG, LOXL1, OCLNP1, RPS27L, HGF, HIKESHI, ANXA1, LACC1, COL6A3, CHRNA3, TPBG, CCL2, CDH6, MIO
N, STK35, ZIC1, OTUB2, CXCL12, IL11, CSNK2A2, MDFIC, PDE4B, DNAJC6, ZNF579, PUM2, LPAR1, ELOVL6, CIB2, M
IAIN, TSLP, IFIT3, HOXB3, AUTS2, TTC28, LAMP5, HBD, CCR7, METTL7B, OLFM3, PRKAR2B, CALB1, DENND6B, IFI2

59, KPNA4, SCARNA7, ARL14, SNORA72, PPM1L
TBN1, FN1, SOS1, ICMT, RAP1A, LAMTOR2, ARTN, FOXO3, FGF23, DUSP1, TEK, KBTBD7, DUSP4, LYPLA1, PIK3CA,

QB, IK, CCDC178, VNN3, UCN3, SBF2, CLDN1, SLC20A2, GRB14, GDF10, SRGN, GLI2, DCAF4L2, PARGP1, FMOD, N
CD2L, CRIP3, CXCL16, MCTP2, PROK2, OBSL1, PCDH8, PODXL, LRATD2, TEKT2, RAPGEF5, PHACTR3, TRIT1, DZIP1,

EID1, MARCKS, APP, SLC66A3, ATRAID, LMNA, EMP3, TCEAL9, BEX3, FCGRT, MALAT1, CHD9, CD81, KDELR2, TME

ARL6IP6, LMO3, SKIDA1, FGF14, LMO4, MORF4L2, TGFB3, CNOT2, HLX, MINDY1, WNT2, PAPPA, RBMS3, WDPCP,

5A, APRT, UBE3A, JCAD, VRK2, CLCA1, NGF, LZTS3, MYH11, SSPN, LOX, PAX4, TRAF3IP3, PCSK7, NFATC4, CAMP, D
NEAL, LARP4, NRK, PYY2, PPP1R15B, ING5, ZMYND11, BLOC1S6, CPM, NRARP, PPP1R12B, COL3A1, SNX16, RDX, Z
2, PCSK6, FLRT3, ZNF263, PI4K2A, PDK4, COL15A1, AXL, NTNG1, LMNA, CCAR2, ELAVL1, NID2, MARCHF6, FNIP1, A

APCD2, LGALS3, OLFM4, NFE2L3, LGALS4, MUC17, KLK10, UNC93B1, TFCP2L1, KRT15, SERPINB2, TMC7, KRT6B, U

CNK2, CBX5, PLPP3, KIF14, SCD, PSAT1, RBP1, RNF138, TOX, KCNMA1, GINS2, GTSE1, DEK, HAS2, TPR, NRXN3, CE

ABP5, CPNE3, GLDC, PTN, OGN, CDH16, KCNK2, FBLN5, PHYH, FMOD, P2RY14, SLC25A15, LIFR, PROM1, IPCEF1, N
YCL, GALK2, LIN54, NR1D1, GPM6A, KIRREL1, FAM214B, GBX2, FOXN3, FOXD3, HAPLN1, TBXAS1, JARID2, NLK, DL
3ASH3A, GRAP2, LINC01281, SEPTIN6, SH2D1A

NC02112, FOXO6, KRT23, ATP6V1C2, SCIN, ASS1, ITGB6, SLC47A2, EPS8L1, CNKSR1, MIR4307HG, PROSER2-AS1, F
LA-DQA1, SIGLEC9, P2RY6, SUCNR1, NAPSB, NCF2, TNFRSF11A, LILRB4, CABP4, C1orf162, LINC01357, SEMA4A, IF
02600, LINC02066, LINC02607, IRX6, C1orf229, MGC15885
CXCR2P1, MYO18B, NT5M, HTR2A, H2BC15, EFCAB13, E2F1, STRIP2, XIRP2, PCP2, GP5, ZNF792, EXOC3L4, TRAP
1A, FNDC1, IL11RA, PSENEN, MFAP4, DHRS1, MAGED2, TNS2, P4HA2, ANGPTL2, NCAM1, SSC5D, LSP1P4, ITGB8,
3, ICAM1, CTSB, COL5A2, GJA4, KLHDC8A, PNP, RASAL2, PDZK1IP1, SCEL, S100A1, MFAP2, HPN, FYN, ATP1B3, OP
DH, STYK1, IFIT1B, CCNE1, ABCB6, BTNL10, PKLR, SCARNA22, SNORD3A, TLCD4-RWDD3, NRIR, LINC01399, HBBP
R2, PLA2G2D, NLRP3, MRPL2, TLR7, EREG, OSCAR, HS3ST2, SUCNR1, RNASE6, LINC02798, CHIT1, SLC37A2, CYP2S

HLA-DRB5, MARCO, CSTA, LILRA1, UPK3A, MYCL, EGR2, SIRPB1, DEFA3, TLR8, CLEC4GP1, PDCD1LG2, SOWAHD, C
543, PLAC9P1, MAST1, ZG16B, ANKRD62, ZNF234, LINC01123, CLDN2, ZFP3, FKBP1B, LINC02267, NXT2, RBM15-A

RO, EYA2, ADGRE1, FCAR, IL10, ALMS1P1, RUFY4, SIRPB1, FCGR1A, LILRA5, GHRL, NRIR, OSM, IL1R2, NOD2
F123, ANKLE1, ARL4A, AURKA, ACHE, SLC5A4-AS1, SLC6A8, NRIR, DYRK3, TSPO2, HBQ1, PPOX, SLC22A4, RSAD2,

TGB6, WNT9B, FAM3B, SLC38A4, MAOA, SMIM22, OVOL1, RNF128, TTC6, FOXA1, CLDN8, FA2H, IRF6, PRDM16-D
RSPO2, REM1, NPY5R, DCDC2C, C6, GLP2R, CHRDL1, MSC, AARD, IGFN1, DIO3, FMO1, LINC02126, DNAJC22, LIN
AT1, LRRC1, LRRC8B, MAPK13, MAPRE1, MARCKS, MARCKSL1, MAT2A, MCM2, MCM3, MCM7, ME2, MEP1A, MI
L1, GADL1, KLF5, VWA5A, C1orf105, HSPB3, TRIM63, FBP2, FGF6, CKMT2, UFL1-AS1, UGT3A1, HCN1, MEG9, LIN
, OR2W3, PDCD10, FLACC1, FAM83D, GPR146, SLC2A1-AS1, YOD1, CMAS, GLRX5, GATA1, SLC22A16, RSAD2, RIL
SLITRK2, LMO3, LMO4, GRIK3, EIF5, CASK, FGF13, RPS6KB1, FGF12, ZIC1, PRMT9, TGFB2, HOXC6, ATOH1, CDKN2

OX2, RNF224, STAM, MARCHF8, HK1, MINPP1, IFIT1B, UROS, HBB, HBBP1, BGLT3, HBG1, HBG2, HBE1, CAT, SLC4
, LINC02649, LINC01503, UNC93B1, LINC01725, MIR155HG, MFSD13A, FGL1, LINC00671, KCNE1, GLT1D1, LHCG
GIT, SLC14A1, APOBEC3D, CTLA4, CHI3L2, LINC01259, KLRC3, NCR3, FLT3LG, CDHR1, S1PR5, PDCD1, CXCR6, DNT

UGT2B15, POU5F1, FAM86FP, LINC00514, BAAT, VGLL1, LINC01152, SELENOV, GFAP, FUT3, UPK1A, LINC00379,
PA7, MAFB, ANXA1, PANK2, DNAJA4, RETN, IFI44L, RIPOR2, WIPI2, VCAN, IQGAP1, OSCAR, AIP, ATF3, ARHGEF2,
ITM2C, VSIR, HIF3A, CSGALNACT1, APC, MACF1, PLSCR4, DDAH1, DNER, PLXDC2, CTNNA2, RIDA, SLITRK2, KCNN

A1, HSD17B6, ECM1, TNFRSF11B, FPR1, FRZB, TIE1, TAGLN, SPARC, ACTA2, TNFAIP6, MARCKS, HNMT, DES, MMP2

1, P4HA1, ORM2, IGF1, PLAT, C1S, SDC1, ABLIM1, PLEC, TNXB, KCNMB1, SLC43A1, CDC42EP1, DHRS2, TFAP2B, GI
ACD, HEG1, HSPB2, PIM1, SFXN3, FUCA1, MSX1, TRIP6, SLC6A2, ADAM19, VAMP5, MAN2C1, RAMP2, SOX10, CR

KA, CCL20, TNNT1, C3, CR2, MAPK10, MAPK8IP2, PLXNB1, RIMS3, KLK6, EPHA4, TMSB15A, ELMO1, KMT2A, SPOC
CAMKV, LYZ, PLBD1, CYP3A5, TP53I3, F2RL1, ARHGEF5, DNMT3B, CDH10, VWA1, PELI1, GSTM2, CDH3, SELENBP1
, FADS2, PHLDA2, ITPR3, GOT1, DDX3Y, PTP4A3, FABP4, PDLIM4, INSIG1, ANGPT1, TIMP3, CD24, IGF2, PLIN2, CX(

CNOT4, USP32P2, KCND2, PI4KB, YTHDF2, ZNF711, PKIA, BICD2, WEE1, MIB1, HIF1A, GABBR2, TESK2, VAMP4, PT

MAT2A, NADK, ELAVL3, ANKHD1, MARCHF6, MARCHF4, LARGE1, CNTD1, JADE3, BNC2, PTP4A1, BNC1, JADE2, BN
6, MGAT4C, TSSK6, ARHGEF12, SYT9, SERPINB5, CLIC5, MINDY2, INHBC, ZMYND8, SRGAP2, FMOD, BPTF, EVA1C,
T8B, IL6, BRD2, LRSAM1, TRIM29, IL9, LMNA, TRIM25, LEXM, VEZF1, NRAS, WNT7B, LRP1, CNTF, PTP4A1, DPYD, P
EF1B2, EIF1AX, EIF4G2, CELA1, ENSA, DMTN, EPC1, ERGIC2, ERH, ESAM, ETF1, F13A1, RETREG1, CCSER2, SINHCAF

POU4F1, PRSS3, PAFAH1B3, CYB5B, KEL, CTR9, IL10RB, MAST4, BAG2, CD200, TYMP, SAMHD1, TPM1, PNN, ULK2

FM1, EIF5, NAT1, TJP1, RBFOX1, FAM102A
1, KCNT2, RESF1, SLC38A1, PDCD7, ZNF324, MDN1, TMEM44, RFLNB, MEF2C, SLC4A3, TSPAN17, GRK3, PLXNA4,
CELSR2, B4GAT1, EPHA5, KIF1B, GNAL, NUDCD3, DPYSL4, MATK, SLIT1, CDC42EP3, GNG4, PDIA2, RAP1GDS1, PRK

1B, LITAF, USF3, HPS4, LIMS1, AXDND1, KREMEN1, ZC3H12C, HNF4G, KRT40, BRINP1, CSF3, HECTD2, CD44, IER3,
FA2, BBS10, RASSF9, CEP290, BTG1, CDK17, NT5DC3, KCTD10, CDK2AP1, CCNA1, LRCH1, RB1, TPTE2P2, USP12, T

C2, SLC11A2, LRRC8E, HS6ST2, POGLUT3, KLF12, TIMP2, STX12, PTGFRN, MANSC1, BCL6, CARD14, SRARP, DSCR8,

, ARHGEF12, PDE8A, COL4A1, EPB41L4A, SVIL, RABGAP1L, BCAS3, NCOA1, SORBS2, MBNL1, HIPK2, SASH1, HIBCH
KDM1B, DCBLD2, GOLGA4, IQCH, LRP6, MIB1, PCSK5, VMA21, HMGN1, TMED7, PAX9, MACO1, RBPMS2, ADAM7
4A, TLCD3B, RIPOR2, FAM81A, LRATD1, FBXL12, FGF14, FGF9, FICD, FMN2, FNDC9, FOXA2, FRMD5, FRY, FSCN1, I
., TSPO, KRT25, RPS24, TIMP3, HMGCS1, GTF2B, FZD6, UBL5, BCR, MST1R, DSTYK, KRTAP4-3, ATM, SULT2B1, DOF
LC8A3, CAST, LMO2, XPO6, SKIDA1, FGF16, CNOT3, RNF112, GATA1, DAB2, GATA6, SH3TC2, RFLNB, SLC4A1, S1PF
J, CRLF1, FGB, RAB8B, EPHA1, LAMC1, CORO1A, NT5C2, DBNDD1, GPX2, C1orf115, GCNT1, CHKA, CAMK2N1, PAC
3, GRM5, HAPLN3, HAS2, HBA1, HBA2, HBG1, HBG2, HEPACAM, HES1, HES5, HRH1, HTRA1, IER3IP1, IFI44L, IGFBF
CAPER, TMEM117, SSH2, ANK3, KAZN, NLK, PPFIBP1, RABGAP1L, MACROD2, COL4A3, GULP1, VAV3, AUH, ZFAND
AVL3, ELAVL4, ELOVL3, ELOVL4, EN1, EN2, ENC1, ENO2, EPB41L1, EPHA5, ERBB4, ERC2, TMEM185B, RETREG1, F.
, CEP76, CEP78, CEP97, CHAF1A, CHAF1B, CHEK1, CHEK2, CHORDC1, CHRNA5, CIT, CKAP2L, CKLF, CKS1B, CKS2, C(
CNK6, RBMXL2, OTUD6B, SNX5, RAB30, FBXO11, ASAH1, TAB2, LYRM1, EDN1, KLHL9, LYSMD3, POMP, PDS5A, PU
3, FAM160B1, CREB1, IREB2, GXYLT1, KCNT2, MXD1, CDH13, EYA2, GAB3, KL, ZNF765, PSD3, LIN54, MARK1, ZFX,

:, SCN5A, TBX3, GRIK3, KCNG3, PTGER3, WSCD2, ADRA2C, GALNT14, GJA3, DLGAP2, GJD3, KCNH8, WNT9A, SHISA
CPLX1, VDR, RXRG, SCN5A, PRDM16, NXPH2, PITPNM3, TFAP2B, C6orf132, MAL, HCN2, SLCO4C1, DOCK8, HOXD
, GM2A, AQP5, ATP6V0A1, ACLY, GSTA3, FBXO32, LTF, DNAJC12, PADI2, FGG, TMEM109, SRPX2, DSP, ATP6V1B1,

RNF41, FUT2, HBP1, CAPZB, TREX2, TCF12, CAP1, CRIM1, PNPLA2, ATP2A1, CAV2, SLC2A4, GPSM1, GSTZ1, EIF4G
)1, ZBTB10, RAD23B, IL1RAP, FAM20B, SMIM14, HELQ, DNAJB14, ZCCHC10, TBL1XR1, C1orf53, AP4E1, RFESD, IFT
MAPK8, MCF2L, MDGA1, MEGF6, MIAT, MIB1, MICAL1, MIR124-2HG, MIR7-3, MLLT11, MTUS2, MYT1, NAPB, NA
TDC1, TFPI, LSAMP, EIF4E, USP6NL, TRMT9B, SESN1, KDM7A, ING4, CCDC186, RBM23, FSTL4, ZNF385D, ZFP82, R
/, PTPN4, FOXC1, MSI2, RAB27B, KCNC2, KIAA0825, BLID, PINX1, MMP13, C5orf15, NR4A3, JADE3, SRBD1, SLC44
SEMA7A, RB1CC1, APP, PFKP, USP46, MEX3D, OLFM3, RGMA, RNH1, SALL1, ZBTB4, TRPV6, CD69, CEP120, KLHL1
P1, MON2, GRAP2, RBM15, TMEM207, CREG1, SPIC, VPS37A, FAM43A, COMMD3, EIF4EBP2, IDNK, DIS3, ZNF420,
, FAT1, SLC10A7, CLDN10, UBE2D2, RHOBTB1, KIF5B, SLC17A6, BICRAL, CDC5L, PPP1CB, SSBP2, TMEM64, ULK2,
EE1, FNDC3B, ARL1, PKN2, LCORL, RAB11A, MID2, ZNHIT6, PIK3CA, JRKL, KHDRBS1, TLR4, STMN2, CCSER2, EMC8
NAI1, SERTAD4, KLC1, RHOXF2B, TLN2, OSTF1, RCOR1, CCR2, DLG2, SRPK1, ANAPC7, TMTC2
C4A7, TUBE1, KCNJ13, PRELID2, TTC8, SH3GL3, LIN9, PRDM1, ARHGAP6, LIN54, MRE11, PGR, MTX3, MINDY3, TRF
ENPK, RO60, HCFC2, NUTF2, SLC4A7, RAP2B, GPR1, PIK3CG, GXYLT1, PRMT9, CBLL1, PDE1C, RHOQ, CTH, TRA2A,
29, CAV1, GPC3, AQP9, PARD6G, LGALS1, HAS2, CAVIN2, IGFBP4, TBX2, MEF2C, LIMS2, BNC1, MATN2, MFNG, DS
B46, ATG16L1, KLHL42, HBP1, ARHGAP12, PPP1R12A, SMAD5, PCDH7, G3BP2, RAB14, MAP4K3, AFF1, RAP1A, S
, DGKH, AUTS2, AKAP12, PAN3, DYRK1A, TACC1, RBPMS, AKAP13, CRIM1, HSPH1, ARID1B, WSB1, CDC42BPA, DL
, MYBL2, IDO1, ICAM3, CHAF1B, PYCR1, APLNR, DLGAP5, SH3KBP1, PHF2, FERMT3, UBE2C, CHDH, PATL1, SEC13,

GLA, CPE, MTFP1, GTF3C2-AS1, COL4A5, TST, FADS3, IL27RA, ELOVL2, H19, PIKFYVE, COPS9, SIAH2, KDM1B, PDZ
D, NEGR1, SLC2A1, PHF8, WDR26, TRIM2, ST18, TENT4B, CDKN1B, C6orf47, ADGRL3, ANO4, GPR137C, AKNAD1,

F7, CD101, IL11, IL9, FASLG, CEBPE, PSTPIP1, AIRE, C8G, AGRP, CCL1, PYY, GBX2, TNFSF14, AFP, HTN3, EML1, KC
P1, WRAP53, C12orf42, UROD, TMEM59L, GPR12, MORC4, LIMS1, ING3, RTN4RL1, SPOCK2, HSD3B7, NDP, PCDH
ASL10A, CLIP3, HOXB2, C1orf174, UBE2E1, ITGAV, STOM, PRF1, WASF1, CAVIN1, MCF2L, SIPA1L1, CXCL2, RPP40,

N2C, AAK1, HOXC4, ATOH7, RNF148, TFAP2D, WDPCP, MS4A7, IL21, TMTC2, HOXD4, FBXO11, DRD3, TCEAL7, SO

YF5, CS, CSNK1A1L, DLG2, PDZRN4, KRT25, KRT28, GRIA1, RBFOX1, NSD3, BAMBI, TOB1, DUSP6, FGF19, LMO1, T
, STAM, MT1G, CBS, GADD45GIP1, ITGB5, NELFCD, MEN1, XK, FADS2, FANCL, PFAS, HOXA9, ATAD2, HSPA6, MC
, SIGLEC12

MEF2D, LYG2, NRAP, JADE3, BNC2, JADE2, NSD3, TUBAL3, FOXE1, AMOT, DUSP7, LRP5, IL2, GRIK3, LMO4, TGFB3,
, CS, MID1, CDH13, PLSCR1, CNTF, LYG2, FAR2, SDHC, LRP8, DPYD, LRP2, OGG1, AP4B1, UBE2U, JDP2, SLC13A5,
N1, DCLRE1A, DLK1, TCEAL1, NDUFAF3, SPNS3, SLC37A1, ITPKB, MYLK, PIP4P2, GNG11, CLCN4, GPX1, RASGRP3,

SLC16A4, SLC16A2, CCNG1, DIAPH2, DBP, ARHGEF9, CROCC, PTPRM, SEMA5A, FGF1, ECI1, UBB, LPAR1, BTG1, A
NF593, STMN2, IL21, PTHLH, SLITRK1, SLITRK4, ROGDI, TMTC2, CADM1, TIAL1, SLC37A2, SIX1, SPRYD3, SPTB, SYT

ICAL3, NR2F2, USP6, CHD1, EPS15, PTPRK, MAP4K5, CEP170, PPP2R2A, SLC4A7, SMCHD1, PRKCI, RCOR1, FOXO3,

[illegible]11, TM4SF1, CASK, CNN3, RANBP2, SCGB2A2, PDLIM5, LRBA, VAV3, ENC1, PPAT, ACOX2, MMP16, SLC1A1, SCN[illegible]
[illegible]71, SH3TC2, IFT81, SETMAR, GMFG, RPS6KA6, NCF2, LATS2, SCML2, MEI4, MAN2A2, SGPL1, PPP1R9A, GALNT7, [illegible]
[illegible]RPL22L1, RPL9, RPP14, WDR43, RPL39L, GNL3, UTP15, RPS14, DCAF13, NOL6, RPL10L, RPL36AL, DDX21, BMS1,

[illegible]8, SERPINI1, ELSPBP1, RAP2A, BLVRA, SLITRK6, FLJ40194, EIF5A2, IGF2BP3, HSPA4L, PSG5, WDR3, NETO2, ANXA3[illegible]

[illegible]S, P4HB, C1QA, HEXB, ADGRG1, FIBIN, DLGAP1, ASNS, GGT5, SYTL2, B3GAT3, C7, RBMS3, RPL22L1, MICALL2, C1S[illegible]
[illegible]TUS1, MCM3AP, FMR1, PKIA, FAM234B, CCDC6, VSNL1, RFX6, FBXO11, HSP90B1, CAMTA1, HMGB2, LRIG1, CCN[illegible]
[illegible]7, CELA2B, DDIT4, FHL2, SLC40A1, IFI6, MX2, HOPX, ALDH1A1, RHAG, ADRB1, FAT1, HS3ST3B1, KRT18, GDF15,

[illegible]FLT3, SEPTIN7, WDR83, AGO2, NCOA3, EREG, PSPN, PSMF1, GFRA4, PSMB2, AGO3, MAP2K2, RAP1B, SEM1, PSM[illegible]

[illegible]COA4, TMED3, HBG1, FAM178B, DYNLT1, GMCL2, LINC01657, NMUR2, SSX2, MAGED4B, JAKMIP1, PASD1, CD86,
[illegible]SH3BGRL2, ZEB1-AS1, PRDM11, TMEM273, WDR18, GPX7, GPM6B, CCDC125, ANKLE1, RPL3L, TBC1D30, TONSL,

[illegible]M59, PSAP, DDAH2, REEP5, S100A11, RAB2A, CYB5A, AP3S1, RBPJ, CYB5R3, NR3C1, TSPAN4, HSP90B1, SRI, NDF[illegible]

[illegible] MTUS1, DSCAM, SOCS1, SIX4, HOXD9, ARHGAP36, SLITRK1, ROGDI, MRPS18B, TIMELESS, HNRNPA0, ZMYM2, S[illegible]

[illegible]HFR, CCL27, LAMB1, SIX1, FN1, MMP3, NDUFA6, IGFBP4, DPP4, PSG3, PBX1, RFX1, VASH1-AS1, RAG2, SPPL2B, M[illegible]
[illegible]NF655, GPCPD1, ZDHHC21, CDYL, HAND1, DDX19A, DDX19B, ACSL6, ERG, SLC8A2, KATNBL1, BIRC6, RICTOR, TSP[illegible]
[illegible]ASIC1, RPS6KA4, C2CD2, PLSCR3, NR5A2, PHLDB2, CMTM6, SHTN1, AP4E1, CAPZA1, EIF5, RBM5, RBFOX2, RNF14[illegible]

[illegible]UBE2C, VSTM2L, VILL, MYB, SPIRE2, PKP3, SOX21, CDC20, CDX2, PMAIP1, ARHGAP27, PLAC8, ANXA3, CDCA3, GP[illegible]

[illegible]NPF, JAM2, AXL, RARRES1, EIF4EBP1, MEIS2, CDC42BPA, CDH6, MYLK, SMARCC1, SLC14A1, PRSS12, TMSB15A, C[illegible]

[illegible]IRN1, HSPB11, FOSB, FMO2, NDRG4, ADD3, NEB, FXYD1, ALDH1A1, CXCL12, LMO2, SNTA1, PRKACB, IER2, PRPSA[illegible]
[illegible]G2, MID1, ITPR3, C11orf53, RBFOX1, SVIL, ORAI3, UBA1, NEUROD2, DENND2D, CCDC30, GFRA1, C1orf122, LUC7[illegible]

[illegible]RASSF6, MAGI1-AS1, LINC00578, NLRP2, TCL6, LINC02583, MIR4713HG, IMPA1P1, DUSP9, TRIM29, MCOLN3, SPI[illegible]
[illegible]IT3, LINC01504, SIRPB2, SLC37A2, IFI27, RASGEF1C, CARD14, HORMAD1, TBC1D30, SIGLEC7, SEZ6L, SCN1B, MS4[illegible]

[illegible]PC3L, ING5, RBPMS2, SUCNR1, LINC02267, GCSAML, TNFSF4, LY6G6F-LY6G6D, LGALS12, TNNI3, RILP, ANKRD9, C[illegible]
[illegible]ZDBF2, GAS1, PTPMT1, SLC39A13, TSPAN17, FCF1, OBSL1, ARMCX2, DNAJC25, CCBE1, TSSC4
[illegible]CML, CPD, CLDN7, TP53I3, PLPP2, COL8A1, ODC1, TSPO, MYO1D, CITED1, TNFRSF21, C1QB, C4A, MUC1, IL13RA1,
[illegible]1, GKN2
[illegible]1, TLDC2, SIRPB2, CD5L, LGALS2, ZEB2-AS1, LRRC39, LINC00970, CCL13, TMEM273, TOR4A, ARL11, LINC01094, C[illegible]

[illegible]CLEC4F, NLRC4, SLC37A2, LILRB3, WFDC21P, NRIR, ANO7L1, HK3, IL1R2
[illegible]S1, ACTR5, C9orf152, DPY19L2P1, PLXNB3, LINC01331, EEF2KMT, IL5RA, CHRFAM7A, LINC01141, KCNH6, MARC[illegible]

RUNDC3A-AS1, KRT1, MYT1, SLC2A1-AS1, YPEL4, CCNE1, GALNT5, TERC, SLC30A10, TF, FAM229A, AEN, RUNDC3[illegible]

T, FRK, CBLC, TMEM184A, VWA5B1, KRT15, FAM83B, EPB41L4B, CGN, B3GNT3, MIR205HG, GATA3-AS1, DEGS2,
C02507, VIPR2, POM121L9P, RXFP2, PCOLCE-AS1, ACTG2, CYP4Z1, DMKN, NGF-AS1, WNT2, LINC02082, TRPA1, G[illegible]
D1IP1, MMD, MMP11, MMP12, MMP9, MMS19, STK26, MTHFD2, MTMR2, MYO6, NAP1L1, NCAPD2, NCAPG, NC[illegible]
C01497, DHRS7C, RPL3L, CA3-AS1, GPD1, PPP1R27, IL20RA, NRAP, GPR37, C10orf71, LINC00595, ASB18, SLC8A3,
P, RFESD, FAM214B, TUBB1, ST6GALNAC4, RNF123, CCDC144CP, GPLD1, ISCA1, ATG4D, CYP3A4, FOXO4, C9orf15[illegible]
C, SAMD11, HOXC4, ZFAND6, PDGFC, LOX, SLC4A4, MTUS1, MIR137HG, NMNAT3, FUT11, RELA, MYO3B, PDE4D,

3A3, YPEL4, C2CD3, PPME1, HMBS, TBCEL, WNK1, LPCAT3, ART4, TROAP, SLC11A2, ESPL1, USP15, LINC02444, P[illegible]
R
T, XCL2, TNFRSF9, TBX21, TNFRSF13B, GZMB, CXCR5, LINC02273, FASLG, CA6, GZMH, VAV3-AS1, AGMAT,

FOXA1, RHPN1-AS1, SOWAHB, LINC01659, LINC01186
CALHM6, ARF6, TNFRSF14, PLCB2, MT2A, TENT5A, PRAM1, TSPAN32, SCIMP, BCL2A1, CHCHD10
3, GABRG1, FBLN1, KMT2C, ARHGEF26, FAM189A2, SSPN, LAMA1, COL6A1, RIC3, ACSL6, ALDH1L1, AK1, C1orf19[illegible]

2, DENND4B, ENP

FPT2,
ABP1, GDF15, S10

K1, AP
, CDH17, LAMC2,
CR4,

TGFRN, SLC40A1, NETO2, HMGB

IIP3L, USP47, DGKZ, USP48,
ABHD5, NNAT, PDRG1, CIPC,
RSS22, EIF3J, IL16,
, FBXO33, FEZ2, FHL1,

2, NR4A2, CD

MMP17, EVL, MMD, NBEA, HRAS
AR1B, FXR2, MAST1, KNDC1, ACA

CYP2C9, AD
SC2

TBC1D8B, HDLBP, SNAP29,

H
, ANKRD33B, XPO4, NDST3, SLC1
FSD2, FTO, FUT9, GABRA1
1B, POLG,
2, NMNAT2, SH2B3,
SIN3, KLF4, VASP, ARL4
2, ILDR2, IQCA1, IRX1, IT
3, LCOR, RALGAPA1, COMMD10, S
AM13C, FAM155A, TAFA1,
ORO1C, COTL1, CPA4, CR
RG, ACSM2A, S1PR3, PECR, NE
MTX3, AXIN2, SEM1, MAP

6, BMP8A, SCRT1, LRRC26, X
10, PRKCZ, HR, RAB11F
GADD45A, GABA

1, CCNL2, NTN
81, BICC1, PLCB4, F
V1, NCAM1, NCAN, NEDD4L,
NF217, TAF7,
5, THUMPD1
5, MYT1L, UNC80, PTHL
ZNF704, SYPL2, NRBF2,
WWP1, GPCP
, CTNNA3, ZNF396, PUM2, ZBTB6

AP, GNAL, SLC3
LL1, FAM237A, PIEZO2, PPM1A,
T, THSD1, POPDC2, TME
TK26, DIPK1A, ARSD, RAF1, PCDHA
2, DOCK4, CACN
HSPD1, SUN2, TAC

K1, CTPS2, NREP, C
ZMIZ1, ASF1B, X

GC3, KIF9, DPY30, LINC00311
TRIM16, HP

5, ELK3, RCAN1, AN

HRA, TNNC1, LMO3, RBM4
3AP-AS1, H1-10, NRGN, LMA

, OTUB2, TGFB2, PACSIN3, PACSI
MO1, IL19, GNA12, E
MCEMP1,

BCC1, PAMR1
16, TNFRSF12A,

IBTK, SCHIP1, CDC27,

N1A, SNRK, PLEKH
RASSF5, TM6SF1, CAP

3, SON, HELLS,

, VASH2, UBE2Z,
G1, MEIS1, CCL7, MEIS2, RE

A1, SHC2, ABHD17A, DUSP9,

, AK5, TRPV3, DEFB132, ASZ1,
PRKAR2B, SPRY1, P

P1, FMO2, S100

MARCA1, EMP1, FBXO1

ILH1, PMS2, TEP
AN18, FNIP1, NRAS, ADCY9, S
4A, ERC2, PACSIN1, A

R35, PLA2G10, GAL

DCA7, IFI16, LRRC17

P1, PDE10A, D
L, TOB1, DUSP6, SLITR

NT1, ISM2,
A14, NLRC4

R1L, BEND2

, KCNN4, CD151, B

D28, IGS

HF4, CMTM2

A, LINC02506, CTSE, A

S100A2, TRIM29, SLC9
GJB2, GDNF-AS1, IL6, CN
CL, NRCAM, NSMAF, NT5DC2,
LINC00578, KCNQ4, DDN, LT
53, YPEL4, KCNN4, ANKLE1
RELCH, NCAM2, HNRNPA0,

ARPBP, TCP11L2, HECT

4, FAT3, BBS2, HAP

Cluster	ID	Description	GeneRatio	BgRatio
0	GO:0051015	actin filament binding	30/462	206/18352
0	GO:0003779	actin binding	43/462	437/18352
0	GO:0045296	cadherin binding	33/462	332/18352
0	GO:0005543	phospholipid binding	37/462	454/18352
0	GO:0044769	ATPase activity, coupled to transmembrane movem	9/462	20/18352
0	GO:0046961	proton-transporting ATPase activity, rotational mech	9/462	20/18352
0	GO:0035091	phosphatidylinositol binding	27/462	262/18352
0	GO:0003924	GTPase activity	29/462	326/18352
0	GO:0019003	GDP binding	13/462	74/18352
1	GO:0015078	proton transmembrane transporter activity	23/652	127/18352
1	GO:0045296	cadherin binding	35/652	332/18352
1	GO:0015252	proton channel activity	9/652	26/18352
1	GO:0004860	protein kinase inhibitor activity	11/652	65/18352
2	GO:0003954	NADH dehydrogenase activity	13/564	46/18352
2	GO:0045296	cadherin binding	30/564	332/18352
2	GO:0070003	threonine-type peptidase activity	9/564	31/18352
2	GO:0045182	translation regulator activity	18/564	139/18352
3	GO:0140375	immune receptor activity	25/524	135/18352
3	GO:0005178	integrin binding	25/524	144/18352
3	GO:0001618	virus receptor activity	17/524	74/18352
3	GO:0019955	cytokine binding	22/524	135/18352
3	GO:0005518	collagen binding	16/524	70/18352
3	GO:0019865	immunoglobulin binding	8/524	24/18352
3	GO:0019864	IgG binding	6/524	11/18352
3	GO:0003823	antigen binding	19/524	168/18352
3	GO:0042287	MHC protein binding	9/524	42/18352
3	GO:0019956	chemokine binding	7/524	33/18352
3	GO:0004896	cytokine receptor activity	11/524	97/18352
4	GO:0045296	cadherin binding	19/309	332/18352
4	GO:0015631	tubulin binding	18/309	365/18352
4	GO:0004860	protein kinase inhibitor activity	7/309	65/18352
4	GO:0003777	microtubule motor activity	7/309	77/18352
4	GO:0005200	structural constituent of cytoskeleton	8/309	104/18352
4	GO:0008200	ion channel inhibitor activity	5/309	38/18352
4	GO:0016248	channel inhibitor activity	5/309	39/18352
5	GO:0048306	calcium-dependent protein binding	9/144	85/18352
5	GO:0009055	electron transfer activity	9/144	111/18352
5	GO:0044548	S100 protein binding	3/144	14/18352
6	GO:0003712	transcription coregulator activity	69/1059	498/18352
6	GO:0016887	ATPase activity	57/1059	423/18352
6	GO:0042393	histone binding	37/1059	238/18352
6	GO:0003714	transcription corepressor activity	28/1059	194/18352
6	GO:0030332	cyclin binding	9/1059	30/18352
7	GO:0005085	guanyl-nucleotide exchange factor activity	40/800	215/18352
7	GO:0030695	GTPase regulator activity	44/800	307/18352

7	GO:0017016	Ras GTPase binding	50/800	415/18352
7	GO:0017137	Rab GTPase binding	18/800	172/18352
7	GO:0048365	Rac GTPase binding	10/800	73/18352
7	GO:0005217	intracellular ligand-gated ion channel activity	6/800	29/18352
8	GO:0003712	transcription coregulator activity	81/1249	498/18352
8	GO:0045296	cadherin binding	51/1249	332/18352
8	GO:0070491	repressing transcription factor binding	14/1249	74/18352
8	GO:0016538	cyclin-dependent protein serine/threonine kinase re[illegible]	11/1249	50/18352
8	GO:0016922	nuclear receptor binding	17/1249	101/18352
8	GO:1990841	promoter-specific chromatin binding	11/1249	58/18352
8	GO:0019887	protein kinase regulator activity	24/1249	185/18352
9	GO:0019210	kinase inhibitor activity	13/921	69/18352
9	GO:0019887	protein kinase regulator activity	23/921	185/18352
9	GO:0140297	DNA-binding transcription factor binding	35/921	347/18352
9	GO:0019207	kinase regulator activity	25/921	216/18352
9	GO:0050840	extracellular matrix binding	10/921	57/18352
9	GO:0051427	hormone receptor binding	20/921	177/18352
9	GO:0035257	nuclear hormone receptor binding	17/921	144/18352
10	GO:0045296	cadherin binding	12/170	332/18352
10	GO:0003779	actin binding	13/170	437/18352
10	GO:0044325	ion channel binding	7/170	132/18352
10	GO:0005001	transmembrane receptor protein tyrosine phosphata[illegible]	3/170	17/18352

pvalue	p.adjust	qvalue	NCBI Entrez Gene Number	Count
7.60E-15	5.15E-12	4.18E-12	822/3936/10095/10109/10094/10092/1	30
2.07E-14	7.03E-12	5.70E-12	822/6275/3936/10095/4046/9168/1048	43
1.91E-11	4.32E-09	3.50E-09	6282/822/302/5052/10890/1192/301/7	33
3.61E-10	5.58E-08	4.52E-08	822/302/6386/5341/311/301/307/341/2	37
4.94E-10	5.58E-08	4.52E-08	526/529/51382/523/528/51606/9114/8	9
4.94E-10	5.58E-08	4.52E-08	526/529/51382/523/528/51606/9114/8	9
6.08E-10	5.89E-08	4.77E-08	822/302/6386/5341/29887/11344/5504	27
4.33E-09	3.67E-07	2.97E-07	5908/10890/397/375/5880/998/55207/5	29
3.57E-08	2.69E-06	2.18E-06	5908/10890/375/55207/5878/5898/787	13
1.10E-10	8.90E-09	7.40E-09	1346/514/513/4697/1340/9551/1350/5	23
9.79E-09	4.21E-07	3.50E-07	1266/6147/6231/3939/10399/5901/202	35
1.55E-07	6.25E-06	5.20E-06	514/513/9551/516/521/506/10632/539/	9
1.63E-05	4.79E-04	3.99E-04	10399/1029/11142/3315/55450/1030/7	11
7.64E-10	7.86E-08	6.60E-08	4697/4718/4696/4708/4716/4706/5107	13
1.44E-07	8.11E-06	6.81E-06	6147/6128/6187/11315/6159/6624/590	30
2.53E-07	1.28E-05	1.07E-05	5683/5689/5694/5693/5690/5682/5691	9
2.69E-07	1.28E-05	1.07E-05	7298/6208/6134/1978/1938/1936/6203	18
8.24E-14	5.48E-11	4.15E-11	3123/972/3119/920/2213/3122/3113/1	25
3.81E-13	1.27E-10	9.59E-11	10457/975/3689/928/7077/8754/3684/	25
2.20E-11	2.12E-09	1.60E-09	920/975/6510/949/4179/3916/942/436	17
3.66E-11	3.04E-09	2.30E-09	972/920/2/3597/7133/10630/2022/704	22
9.30E-11	6.87E-09	5.20E-09	1520/1508/1514/8754/7045/51144/367	16
2.06E-07	6.82E-06	5.16E-06	920/2213/2217/2212/11027/2209/2207	8
2.15E-07	6.82E-06	5.16E-06	2213/2217/2212/2209/2207/2214	6
3.48E-07	1.05E-05	7.96E-06	3123/3106/3119/3122/3113/3117/5660	19
2.27E-06	6.28E-05	4.75E-05	972/920/29992/975/3133/6892/10288/	9
3.33E-05	0.00062	4.69E-04	2/10630/1230/3688/5355/9034/3685	7
0.00010	0.00148	1.12E-03	972/920/3597/1230/3587/3459/960/90	11
3.86E-06	0.00053	4.64E-04	29091/6711/6130/3799/10399/6138/59	19
5.06E-05	0.00463	4.05E-03	1410/4131/9638/9448/3800/3799/547/	18
0.00011	0.00660	5.78E-03	55450/1028/10399/4869/65125/9467/9	7
0.00031	0.01439	0.01261	3800/3799/547/1780/83658/1783/1781	7
0.00037	0.01583	0.01387	10382/23336/4747/71/6711/55914/60/	8
0.00042	0.01635	0.01433	857/6324/10399/65125/805	5
0.00047	0.01694	0.01484	857/6324/10399/65125/805	5
2.18E-08	7.92E-06	6.16E-06	6280/301/6275/307/808/6281/6282/30	9
2.25E-07	4.09E-05	3.18E-05	4688/2745/1528/2230/10327/217/6392	9
0.00016	9.05E-03	7.04E-03	6282/302/6277	3
1.04E-11	8.48E-09	7.68E-09	2521/10521/10443/84441/58508/3192/	69
1.95E-09	5.31E-07	4.81E-07	80205/55636/10521/55605/7516/7919/	57
3.45E-08	4.29E-06	3.89E-06	9678/55904/4678/58508/4297/2186/59	37
6.86E-06	3.99E-04	3.62E-04	10443/3192/7091/64324/2146/9139/91	28
3.26E-05	1.33E-03	1.20E-03	8621/1021/80279/55294/51755/5936/9	9
5.24E-15	4.42E-12	3.99E-12	55704/8567/27128/23077/25780/5578	40
3.24E-12	1.37E-09	1.23E-09	55704/4650/6002/25780/8829/116985/	44

7.29E-11	1.09E-08	9.85E-09	23312/4650/8567/64780/79083/752/11	50
0.00053	0.021190	0.019103	23312/8567/64780/79083/9905/22898/	18
0.00121	0.031867	0.028729	752/393/2316/9732/10160/23380/5752	10
0.00135	0.032575	0.029368	55283/3709/7226/57192/53373/25523	6
1.55E-13	1.44E-10	1.27E-10	5396/6664/7091/283248/6659/11168/5	81
3.67E-08	4.85E-06	4.30E-06	1627/1004/1501/28969/1266/10528/66	51
0.00041	0.012833	0.011364	7091/6929/3066/1108/23468/2958/659	14
0.00044	0.012833	0.011364	1031/595/1163/1019/4331/894/1027/91	11
0.00044	0.012833	0.011364	8805/25879/811/6605/142/23028/8289	17
0.00163	0.031376	0.027785	3066/55636/1660/23028/10155/6881/2	11
0.00175	0.031423	0.027826	28951/1031/5569/10926/10253/595/57	24
3.33E-05	0.007145	0.006513	11142/94032/8692/5570/10253/28951/	13
5.55E-05	0.007145	0.006513	100133941/11142/51704/94032/8692/5	23
7.08E-05	0.007145	0.006513	55859/5396/6604/7088/6662/5629/715	35
8.80E-05	0.007488	0.006826	100133941/11142/51704/94032/8692/5	25
0.00049	0.025833	0.023550	9806/3673/2817/6469/9289/1605/7422	10
0.00059	0.029472	0.026867	6604/5629/2534/1616/7205/55791/81/2	20
0.00090	0.038455	0.035056	6604/5629/1616/7205/55791/81/22938	17
6.42E-05	0.024252	0.021949	23499/55082/28513/1739/114793/5797	12
0.00022	0.029089	0.026327	23499/130271/667/88/11155/114793/1	13
0.00023	0.029089	0.026327	88/1739/9472/288/9001/5142/23241	7
0.00048	0.036489	0.033024	5789/5797/5796	3

Cluster	ID	GeneRatio	BgRatio	pvalue
0	GO_RESPONSE_TO_INTERLEUKIN_12	16/457	50/17901	5.57E-14
0	GO_REGULATION_OF_CELLULAR_COMPONEN	39/457	383/17901	1.83E-13
0	GO_MYELOID_CELL_DIFFERENTIATION	40/457	431/17901	1.78E-12
0	GO_FC_RECEPTOR_MEDIATED_STIMULATORY	23/457	144/17901	2.04E-12
0	GO_PLATELET_AGGREGATION	14/457	62/17901	3.89E-10
0	GO_HOMOTYPIC_CELL_CELL_ADHESION	16/457	86/17901	4.70E-10
0	GO_PLATELET_ACTIVATION	20/457	158/17901	3.92E-09
0	GO_FC_RECEPTOR_SIGNALING_PATHWAY	24/457	240/17901	1.30E-08
1	GO_OXIDATIVE_PHOSPHORYLATION	55/633	149/17901	1.88E-41
1	GO_ATP_METABOLIC_PROCESS	71/633	311/17901	1.63E-37
1	GO_ATP_SYNTHESIS_COUPLED_ELECTRON_T	43/633	98/17901	1.90E-36
1	GO_RESPIRATORY_ELECTRON_TRANSPORT_	45/633	116/17901	3.34E-35
1	GO_CELLULAR_RESPIRATION	52/633	187/17901	3.40E-32
2	GO_COTRANSLATIONAL_PROTEIN_TARGETIN	72/547	106/17901	1.88E-84
2	GO_NUCLEAR_TRANSCRIBED_MRNA_CATABO	73/547	120/17901	3.24E-80
2	GO_ESTABLISHMENT_OF_PROTEIN_LOCALIZA	73/547	121/17901	7.96E-80
2	GO_RNA_CATABOLIC_PROCESS	112/547	415/17901	4.60E-75
2	GO_NUCLEAR_TRANSCRIBED_MRNA_CATABO	84/547	210/17901	4.11E-72
3	GO_ANTIGEN_PROCESSING_AND_PRESENTAT	42/512	232/17901	5.85E-22
3	GO_T_CELL_ACTIVATION	53/512	483/17901	3.33E-17
3	GO_LEUKOCYTE_CELL_CELL_ADHESION	42/512	364/17901	1.33E-14
3	GO_MYELOID_LEUKOCYTE_DIFFERENTIATION	30/512	210/17901	3.57E-13
3	GO_POSITIVE_REGULATION_OF_PHAGOCYTO	18/512	68/17901	4.14E-13
3	GO_REGULATION_OF_CELL_CELL_ADHESION	44/512	439/17901	4.62E-13
3	GO_REGULATION_OF_LEUKOCYTE_DIFFEREN	35/512	290/17901	6.15E-13
3	GO_POSITIVE_REGULATION_OF_CELL_ADHES	43/512	428/17901	7.96E-13
3	GO_LEUKOCYTE_PROLIFERATION	36/512	313/17901	1.21E-12
3	GO_REGULATION_OF_LEUKOCYTE_MIGRATIO	29/512	212/17901	2.70E-12
4	GO_COTRANSLATIONAL_PROTEIN_TARGETIN	43/296	106/17901	4.16E-49
4	GO_ESTABLISHMENT_OF_PROTEIN_LOCALIZA	43/296	121/17901	4.17E-46
4	GO_NUCLEAR_TRANSCRIBED_MRNA_CATABO	42/296	120/17901	1.04E-44
4	GO_TRANSLATIONAL_INITIATION	48/296	192/17901	4.09E-43
4	GO_PROTEIN_LOCALIZATION_TO_ENDOPLASM	44/296	149/17901	4.56E-43
4	GO_ESTABLISHMENT_OF_PROTEIN_LOCALIZA	55/296	339/17901	1.28E-38
4	GO_VIRAL_GENE_EXPRESSION	42/296	195/17901	7.91E-35
4	GO_REGULATION_OF_CELLULAR_RESPONSE_	8/296	129/17901	0.00137803
4	GO_APICAL_PROTEIN_LOCALIZATION	3/296	14/17901	0.00142305
4	GO_ANTIGEN_PROCESSING_AND_PRESENTAT	11/296	232/17901	0.00172218
4	GO_RAS_PROTEIN_SIGNAL_TRANSDUCTION	14/296	346/17901	0.00194533
5	GO_ELECTRON_TRANSPORT_CHAIN	13/144	184/17901	3.04E-09
5	GO_REGULATION_OF_LEUKOCYTE_PROLIFER	13/144	240/17901	7.28E-08
5	GO_CELL_CHEMOTAXIS	14/144	310/17901	2.13E-07
5	GO_T_CELL_PROLIFERATION	11/144	195/17901	5.12E-07
5	GO_LEUKOCYTE_CELL_CELL_ADHESION	14/144	364/17901	1.45E-06
5	GO_LEUKOCYTE_PROLIFERATION	13/144	313/17901	1.51E-06

5	GO_POSITIVE_REGULATION_OF_T_CELL_PRO	8/144	101/17901	1.55E-06
5	GO_LEUKOCYTE_CHEMOTAXIS	11/144	231/17901	2.70E-06
5	GO_ANTIGEN_PROCESSING_AND_PRESENTAT	11/144	232/17901	2.82E-06
5	GO_REGULATION_OF_T_CELL_ACTIVATION	13/144	332/17901	2.89E-06
6	GO_RNA_SPLICING	85/975	484/17901	3.27E-22
6	GO_REGULATION_OF_RNA_SPLICING	42/975	155/17901	1.44E-18
6	GO_REGULATION_OF_MRNA_PROCESSING	39/975	152/17901	1.89E-16
6	GO_RNA_SPLICING_VIA_TRANSESTERIFICATIO	65/975	391/17901	5.36E-16
6	GO_REGULATION_OF_MRNA_SPLICING_VIA_S	32/975	113/17901	4.59E-15
6	GO_POSITIVE_REGULATION_OF_CELL_CYCLE	63/975	396/17901	1.29E-14
6	GO_POSITIVE_REGULATION_OF_CELL_CYCLE_	52/975	302/17901	1.15E-13
6	GO_POSITIVE_REGULATION_OF_CHROMOSOM	37/975	182/17901	2.64E-12
6	GO_DNA_REPLICATION	45/975	273/17901	2.46E-11
6	GO_REGULATION_OF_CHROMOSOME_ORGAN	52/975	356/17901	7.40E-11
6	GO_REGULATION_OF_CHROMATIN_ORGANIZA	34/975	195/17901	1.51E-09
6	GO_SISTER_CHROMATID_SEGREGATION	33/975	196/17901	6.53E-09
6	GO_CELL_CYCLE_CHECKPOINT	35/975	219/17901	9.28E-09
6	GO_REGULATION_OF_CELL_CYCLE_PHASE_T	56/975	486/17901	9.20E-08
6	GO_CHROMATIN_REMODELING	33/975	218/17901	9.34E-08

6	GO_POSITIVE_REGULATION_OF_DNA_METABOLIC	31/975	200/17901	1.32E-07
6	GO_CHROMOSOME_SEGREGATION	43/975	334/17901	1.40E-07
6	GO_REGULATION_OF_SMALL_GTPASE_MEDIATED	42/975	323/17901	1.48E-07
6	GO_DENDRITE_DEVELOPMENT	35/975	247/17901	2.03E-07
6	GO_RNA_3_END_PROCESSING	26/975	153/17901	2.14E-07
6	GO_TELENCEPHALON_DEVELOPMENT	36/975	259/17901	2.21E-07
6	GO_POSITIVE_REGULATION_OF_CHROMATIN_ORG	21/975	106/17901	2.22E-07
6	GO_POSITIVE_REGULATION_OF_STEM_CELL_PROL	13/975	43/17901	2.72E-07
6	GO_NEGATIVE_REGULATION_OF_NERVOUS_SYSTE	41/975	319/17901	2.85E-07
6	GO_DENDRITE_MORPHOGENESIS	25/975	146/17901	3.14E-07
6	GO_REGULATION_OF_HISTONE_METHYLATION	16/975	67/17901	4.32E-07
6	GO_REGULATION_OF_HISTONE_MODIFICATION	25/975	149/17901	4.68E-07
6	GO_NEGATIVE_REGULATION_OF_RNA_SPLICING	10/975	26/17901	5.24E-07
6	GO_DNA_INTEGRITY_CHECKPOINT	26/975	161/17901	5.98E-07
6	GO_REGULATION_OF_DNA_METABOLIC_PROCESS	44/975	365/17901	6.47E-07
6	GO_NUCLEOBASE_CONTAINING_COMPOUND_TRA	35/975	260/17901	7.05E-07
6	GO_POSTSYNAPSE_ORGANIZATION	27/975	173/17901	7.33E-07
6	GO_ORGANELLE_FISSION	52/975	467/17901	7.68E-07
6	GO_COMMISSURAL_NEURON_AXON_GUIDANCE	7/975	12/17901	8.66E-07
6	GO_MICROTUBULE_CYTOSKELETON_ORGANIZATIO	24/975	144/17901	8.96E-07
6	GO_REGULATION_OF_SYNAPSE_STRUCTURE_OR_A	33/975	240/17901	9.00E-07
6	GO_METHYLATION	44/975	371/17901	1.01E-06
6	GO_SISTER_CHROMATID_COHESION	15/975	63/17901	1.03E-06
6	GO_NEGATIVE_REGULATION_OF_MRNA_SPLICING	9/975	22/17901	1.06E-06
6	GO_REGULATION_OF_DNA_TEMPLATED_TRANSCR	13/975	48/17901	1.11E-06
6	GO_DNA_DAMAGE_RESPONSE_SIGNAL_TRANSDU	20/975	107/17901	1.14E-06

6	GO_REGULATION_OF_POSTSYNAPSE_ORGANIZATI	20/975	107/17901	1.14E-06
6	GO_POSITIVE_REGULATION_OF_HISTONE_METHY	12/975	41/17901	1.16E-06
6	GO_MITOTIC_DNA_INTEGRITY_CHECKPOINT	20/975	108/17901	1.33E-06
6	GO_POSITIVE_REGULATION_OF_CELL_CYCLE_ARR	17/975	81/17901	1.34E-06
6	GO_POSITIVE_REGULATION_OF_MITOTIC_CELL_C	26/975	168/17901	1.38E-06
6	GO_POSITIVE_REGULATION_OF_NEUROBLAST_PR	9/975	23/17901	1.66E-06
6	GO_NEGATIVE_REGULATION_OF_CELL_DEVELOPM	41/975	342/17901	1.79E-06
6	GO_DNA_RECOMBINATION	37/975	294/17901	1.79E-06
6	GO_SIGNAL_TRANSDUCTION_INVOLVED_IN_CELL_	16/975	74/17901	1.80E-06
6	GO_CALCIUM_DEPENDENT_CELL_CELL_ADHESION	12/975	43/17901	2.03E-06
6	GO_DNA_DEPENDENT_DNA_REPLICATION	24/975	151/17901	2.15E-06
6	GO_SPINDLE_ORGANIZATION	27/975	183/17901	2.24E-06
6	GO_MRNA_EXPORT_FROM_NUCLEUS	20/975	113/17901	2.76E-06
6	GO_RNA_EXPORT_FROM_NUCLEUS	23/975	144/17901	3.20E-06
6	GO_REGULATION_OF_GENE_EXPRESSION_EPIGEN	29/975	209/17901	3.39E-06
6	GO_POSITIVE_REGULATION_OF_RESPONSE_TO_D	19/975	105/17901	3.47E-06
6	GO_SIGNAL_TRANSDUCTION_BY_P53_CLASS_MED	34/975	267/17901	3.59E-06
6	GO_REGULATION_OF_CELL_CYCLE_ARREST	19/975	106/17901	4.02E-06
6	GO_POSITIVE_REGULATION_OF_SISTER_CHROMAT	6/975	10/17901	4.47E-06
6	GO_REGULATION_OF_CHROMOSOME_SEGREGATI	19/975	107/17901	4.64E-06
6	GO_RESPONSE_TO_RADIATION	48/975	446/17901	5.24E-06
6	GO_MITOTIC_SISTER_CHROMATID_COHESION	9/975	26/17901	5.48E-06
6	GO_MITOTIC_SPINDLE_ORGANIZATION	20/975	118/17901	5.50E-06
6	GO_POSITIVE_REGULATION_OF_CHROMOSOME_S	10/975	33/17901	6.44E-06
6	GO_MITOTIC_G1_S_TRANSITION_CHECKPOINT	14/975	64/17901	6.85E-06
6	GO_PALLIUM_DEVELOPMENT	25/975	173/17901	7.67E-06
6	GO_CELLULAR_RESPONSE_TO_CHOLESTEROL	8/975	21/17901	8.11E-06
6	GO_NEURON_PROJECTION_ORGANIZATION	17/975	92/17901	8.38E-06
6	GO_REGULATION_OF_NEUROBLAST_PROLIFERATI	10/975	34/17901	8.68E-06
6	GO_SPLICEOSOMAL_COMPLEX_ASSEMBLY	16/975	83/17901	8.75E-06
6	GO_HISTONE_H3_K4_METHYLATION	13/975	57/17901	8.95E-06
6	GO_REGULATION_OF_DNA_REPAIR	21/975	132/17901	9.01E-06
6	GO_SIGNAL_TRANSDUCTION_IN_RESPONSE_TO_D	21/975	133/17901	1.02E-05
6	GO_REGULATION_OF_SISTER_CHROMATID_SEGRE	16/975	84/17901	1.03E-05
6	GO_REGULATION_OF_CELL_MORPHOGENESIS	51/975	499/17901	1.15E-05
6	GO_REPLICATION_FORK_PROCESSING	10/975	35/17901	1.16E-05
6	GO_REGULATION_OF_DOUBLE_STRAND_BREAK_R	16/975	85/17901	1.20E-05
6	GO_POSITIVE_REGULATION_OF_MITOTIC_SISTER_	8/975	22/17901	1.21E-05
6	GO_CELL_JUNCTION_ASSEMBLY	46/975	434/17901	1.22E-05
6	GO_REGULATION_OF_STEM_CELL_PROLIFERATION	15/975	76/17901	1.23E-05
6	GO_NUCLEAR_TRANSPORT	40/975	357/17901	1.24E-05
6	GO_SPINDLE_ASSEMBLY	19/975	115/17901	1.37E-05
6	GO_MICROTUBULE_ORGANIZING_CENTER_ORGAN	21/975	136/17901	1.45E-05
6	GO_NEGATIVE_REGULATION_OF_NEURON_DIFFEF	29/975	225/17901	1.45E-05
6	GO_POSITIVE_REGULATION_OF_DOUBLE_STRAND	10/975	36/17901	1.52E-05
6	GO_REGULATION_OF_CELL_MORPHOGENESIS_INV	36/975	310/17901	1.53E-05
6	GO_DNA_DEPENDENT_DNA_REPLICATION_MAINT	11/975	44/17901	1.73E-05

6	GO_REGULATION_OF_DNA_RECOMBINATION	18/975	107/17901	1.78E-05
6	GO_MAINTENANCE_OF_SISTER_CHROMATID_COH	6/975	12/17901	1.79E-05
6	GO_STEM_CELL_PROLIFERATION	20/975	128/17901	1.92E-05
6	GO_MRNA_SPLICE_SITE_SELECTION	12/975	53/17901	2.14E-05
6	GO_POSITIVE_REGULATION_OF_DNA_RECOMBINA	11/975	45/17901	2.17E-05
6	GO_REGULATION_OF_NEURON_MIGRATION	11/975	45/17901	2.17E-05
6	GO_SYNAPSE_ASSEMBLY	25/975	184/17901	2.27E-05
6	GO_NEGATIVE_REGULATION_OF_MITOTIC_CELL_C	38/975	341/17901	2.29E-05
6	GO_NEUROBLAST_PROLIFERATION	13/975	62/17901	2.36E-05
6	GO_POSITIVE_REGULATION_OF_DNA_REPAIR	14/975	71/17901	2.42E-05
6	GO_POSITIVE_REGULATION_OF_CELL_PROJECTION	42/975	394/17901	2.53E-05
6	GO_NEGATIVE_REGULATION_OF_CELL_CYCLE_G1_	20/975	132/17901	3.04E-05
6	GO_CELL_CYCLE_G1_S_PHASE_TRANSITION	35/975	310/17901	3.63E-05
6	GO_NEURON_PROJECTION_GUIDANCE	33/975	285/17901	3.63E-05
6	GO_CELL_CYCLE_G2_M_PHASE_TRANSITION	32/975	273/17901	3.72E-05
6	GO_REGULATION_OF_MESENCHYMAL_CELL_PROL	9/975	33/17901	4.79E-05
6	GO_NUCLEAR_EXPORT	26/975	204/17901	4.81E-05
6	GO_CELLULAR_RESPONSE_TO_STEROL	8/975	26/17901	4.88E-05
6	GO_IONOTROPIC_GLUTAMATE_RECEPTOR_SIGNA	8/975	26/17901	4.88E-05
6	GO_DNA_PACKAGING	29/975	240/17901	4.89E-05
6	GO_PROTEIN_LOCALIZATION_TO_CHROMOSOME	15/975	85/17901	4.93E-05
6	GO_CILIARY_BASAL_BODY_PLASMA_MEMBRANE_	16/975	95/17901	5.06E-05
6	GO_REGULATION_OF_ORGANELLE_ASSEMBLY	25/975	193/17901	5.10E-05
6	GO_DNA_GEOMETRIC_CHANGE	18/975	116/17901	5.43E-05
6	GO_DNA_TEMPLATED_TRANSCRIPTION_ELONGAT	18/975	116/17901	5.43E-05
6	GO_PROTEIN_CONTAINING_COMPLEX_LOCALIZAT	33/975	291/17901	5.49E-05
6	GO_REGULATION_OF_CELL_CYCLE_G1_S_PHASE_T	26/975	206/17901	5.68E-05
6	GO_RHYTHMIC_PROCESS	34/975	305/17901	6.02E-05
6	GO_CILIUM_ORGANIZATION	41/975	396/17901	6.11E-05
6	GO_PEPTIDYL_LYSINE_ACETYLATION	23/975	172/17901	6.12E-05
6	GO_MULTICELLULAR_ORGANISM_GROWTH	21/975	150/17901	6.44E-05
6	GO_CENTROSOME_DUPLICATION	13/975	68/17901	6.58E-05
6	GO_POSITIVE_REGULATION_OF_DNA_TEMPLATED	8/975	27/17901	6.60E-05
6	GO_CEREBRAL_CORTEX_DEVELOPMENT	18/975	118/17901	6.83E-05
6	GO_RESPONSE_TO_UV	20/975	140/17901	7.16E-05
6	GO_REGULATION_OF_NOTCH_SIGNALING_PATHW	17/975	108/17901	7.22E-05
6	GO_CELL_CYCLE_ARREST	28/975	234/17901	7.78E-05
6	GO_NEGATIVE_REGULATION_OF_STEM_CELL_DIFF	7/975	21/17901	8.25E-05
6	GO_NEGATIVE_REGULATION_OF_CELL_PROJECTIO	24/975	187/17901	8.29E-05
6	GO_MAINTENANCE_OF_CELL_NUMBER	22/975	164/17901	8.36E-05
6	GO_NUCLEUS_LOCALIZATION	8/975	28/17901	8.80E-05
6	GO_NEGATIVE_REGULATION_OF_NEURON_PROJE	21/975	154/17901	9.48E-05
6	GO_PEPTIDYL_LYSINE_TRIMETHYLATION	11/975	53/17901	0.0001094
6	GO_REGULATION_OF_SISTER_CHROMATID_COHES	7/975	22/17901	0.00011527
6	GO_REGULATION_OF_HISTONE_H3_K4_METHYLAT	8/975	29/17901	0.0001158
6	GO_PROTEIN_ACETYLATION	25/975	203/17901	0.00011663
6	GO_HOMOPHILIC_CELL_ADHESION_VIA_PLASMA_	22/975	168/17901	0.00011989

6	GO_MITOTIC_CHROMOSOME_CONDENSATION	6/975	16/17901	0.00012838
6	GO_REGULATION_OF_RNA_EXPORT_FROM_NUCL	6/975	16/17901	0.00012838
6	GO_CYTOPLASMIC_MICROTUBULE_ORGANIZATIO	12/975	63/17901	0.00012929
6	GO_PRODUCTION_OF_SMALL_RNA_INVOLVED_IN	11/975	54/17901	0.00013071
6	GO_REGULATION_OF_CELL_CYCLE_G2_M_PHASE_	26/975	217/17901	0.00013534
6	GO_NEURONAL_STEM_CELL_POPULATION_MAINT	7/975	23/17901	0.00015795
6	GO_NUCLEAR_MIGRATION	7/975	23/17901	0.00015795
6	GO_STRESS_GRANULE_ASSEMBLY	7/975	23/17901	0.00015795
6	GO_REGULATION_OF_DOUBLE_STRAND_BREAK_R	10/975	47/17901	0.00017979
6	GO_PROTEIN_ACYLATION	28/975	248/17901	0.00021117
6	GO_REGULATION_OF_DENDRITIC_SPINE_MORPHO	10/975	48/17901	0.00021615
6	GO_RNA_POLYADENYLATION	10/975	48/17901	0.00021615
6	GO_REGULATION_OF_NUCLEAR_DIVISION	23/975	188/17901	0.00023729
6	GO_CENTRIOLE_ASSEMBLY	9/975	40/17901	0.00024066
6	GO_KIDNEY_EPITHELIUM_DEVELOPMENT	19/975	142/17901	0.00025728
6	GO_HISTONE_H3_K9_MODIFICATION	10/975	49/17901	0.00025848
6	GO_CELL_CELL_ADHESION_VIA_PLASMA_MEMBRA	30/975	277/17901	0.00026534
6	GO_NEURAL_PRECURSOR_CELL_PROLIFERATION	20/975	154/17901	0.00026848
6	GO_NEGATIVE_REGULATION_OF_CELL_DIVISION	6/975	18/17901	0.0002709
6	GO_POSITIVE_REGULATION_OF_HISTONE_H3_K4_	6/975	18/17901	0.0002709
6	GO_REGULATION_OF_DENDRITE_DEVELOPMENT	20/975	155/17901	0.00029282
6	GO_RESPONSE_TO_LIGHT_STIMULUS	33/975	318/17901	0.0002964
6	GO_POSITIVE_REGULATION_OF_GENE_EXPRESSIO	11/975	59/17901	0.00029796
6	GO_PROTEIN_LOCALIZATION_TO_MICROTUBULE_	8/975	33/17901	0.00030852
6	GO_NEGATIVE_REGULATION_OF_CELL_CYCLE_PRO	36/975	359/17901	0.00031326
6	GO_HINDBRAIN_DEVELOPMENT	20/975	156/17901	0.00031908
6	GO_CIRCADIAN_RHYTHM	25/975	218/17901	0.000356
6	GO_GLUTAMATE_RECEPTOR_SIGNALING_PATHWA	15/975	101/17901	0.00036228
6	GO_HISTONE_H4_ACETYLATION	12/975	70/17901	0.00036311
6	GO_REGULATION_OF_ANDROGEN_RECEPTOR_SIG	7/975	26/17901	0.00036717
6	GO_DOSAGE_COMPENSATION	6/975	19/17901	0.00037779
6	GO_REGULATION_OF_NUCLEOBASE_CONTAINING	6/975	19/17901	0.00037779
6	GO_NEGATIVE_REGULATION_OF_CELL_CYCLE_PHA	29/975	270/17901	0.00038282
6	GO_DENDRITIC_SPINE_DEVELOPMENT	15/975	102/17901	0.00040377
6	GO_NOTCH_SIGNALING_PATHWAY	23/975	195/17901	0.000404
6	GO_CHROMATIN_ASSEMBLY_OR_DISASSEMBLY	25/975	220/17901	0.00040885
6	GO_POSITIVE_REGULATION_OF_NUCLEAR_DIVISIO	12/975	71/17901	0.00041566
6	GO_PRIMARY_MIRNA_PROCESSING	5/975	13/17901	0.00042284
6	GO_ANDROGEN_RECEPTOR_SIGNALING_PATHWA	9/975	43/17901	0.00042854
6	GO_RESPONSE_TO_NERVE_GROWTH_FACTOR	10/975	52/17901	0.00042911
6	GO_METENCEPHALON_DEVELOPMENT	16/975	114/17901	0.00044918
6	GO_POSITIVE_REGULATION_OF_NEURON_DIFFERE	37/975	380/17901	0.00045988
6	GO_REGULATION_OF_MEGAKARYOCYTE_DIFFERE	13/975	82/17901	0.00046235
6	GO_DENDRITIC_SPINE_MORPHOGENESIS	11/975	62/17901	0.00046619
6	GO_REGULATION_OF_TRANSCRIPTION_ELONGATI	7/975	27/17901	0.00047257
6	GO_RESPONSE_TO_STEROL	8/975	35/17901	0.00047475
6	GO_NON_RECOMBINATIONAL_REPAIR	15/975	104/17901	0.00049909

6	GO_MESENCHYMAL_CELL_PROLIFERATION	9/975	44/17901	0.00051304
6	GO_DNA_METHYLATION_DEPENDENT_HETEROCH	6/975	20/17901	0.00051498
6	GO_POSITIVE_REGULATION_OF_NEURON_PROJEC	30/975	290/17901	0.00057645
6	GO_STEM_CELL_DIFFERENTIATION	28/975	264/17901	0.00058555
6	GO_POSITIVE_REGULATION_OF_NEURAL_PRECUR	10/975	54/17901	0.00058818
6	GO_PATTERN_SPECIFICATION_PROCESS	42/975	455/17901	0.00059733
6	GO_PROTEIN_LOCALIZATION_TO_CHROMATIN	7/975	28/17901	0.00060076
6	GO_REGULATION_OF_DENDRITE_MORPHOGENES	14/975	95/17901	0.00060937
6	GO_POSITIVE_REGULATION_OF_CHROMATIN_ASS	5/975	14/17901	0.00062836
6	GO_ESTABLISHMENT_OF_CELL_POLARITY	18/975	141/17901	0.00065594
6	GO_POSITIVE_REGULATION_OF_ANIMAL_ORGAN_	13/975	85/17901	0.00065871
6	GO_REGULATION_OF_STEM_CELL_DIFFERENTIATI	16/975	118/17901	0.00066211
6	GO_MICROTUBULE_BASED_TRANSPORT	22/975	190/17901	0.00068798
6	GO_REGULATION_OF_MICROTUBULE_CYTOSKELET	22/975	190/17901	0.00068798
6	GO_P_BODY_ASSEMBLY	6/975	21/17901	0.00068799
6	GO_REGULATION_OF_CHROMATIN_ASSEMBLY	6/975	21/17901	0.00068799
6	GO_MITOTIC_SPINDLE_ASSEMBLY	11/975	65/17901	0.00070743
6	GO_G0_TO_G1_TRANSITION	9/975	46/17901	0.00072311
6	GO_POSITIVE_REGULATION_OF_GTPASE_ACTIVITY	38/975	404/17901	0.00075179
6	GO_RESPIRATORY_SYSTEM_DEVELOPMENT	23/975	204/17901	0.00076309
6	GO_CHROMATIN_ORGANIZATION_INVOLVED_IN_	19/975	156/17901	0.00084413
6	GO_HISTONE_H3_K9_METHYLATION	8/975	38/17901	0.00085337
6	GO_WNT_SIGNALING_PATHWAY_CALCIUM_MODU	8/975	38/17901	0.00085337
6	GO_NEGATIVE_REGULATION_OF_DNA_TEMPLATE	5/975	15/17901	0.00090049
6	GO_REGULATION_OF_GTPASE_ACTIVITY	43/975	479/17901	0.00091065
6	GO_POSITIVE_REGULATION_OF_MITOTIC_NUCLEA	10/975	57/17901	0.00091569
6	GO_POSITIVE_REGULATION_OF_NOTCH_SIGNALIN	10/975	57/17901	0.00091569
6	GO_ADHERENS_JUNCTION_ORGANIZATION	11/975	67/17901	0.00091984
6	GO_POSITIVE_REGULATION_OF_INTRACELLULAR_	24/975	221/17901	0.00100851
6	GO_MEGAKARYOCYTE_DIFFERENTIATION	14/975	100/17901	0.00102427
6	GO_REGULATION_OF_CARBOHYDRATE_CATABOLI	13/975	90/17901	0.00114063
6	GO_POSITIVE_REGULATION_OF_NEUROTRANSMIT	6/975	23/17901	0.00116563
6	GO_REGULATION_OF_CENTRIOLE_REPLICATION	6/975	23/17901	0.00116563
6	GO_REGULATION_OF_INTRACELLULAR_PROTEIN_	27/975	263/17901	0.00118851
6	GO_TRANSPORT_ALONG_MICROTUBULE	19/975	161/17901	0.00123569
6	GO_REGULATION_OF_ATP_METABOLIC_PROCESS	16/975	125/17901	0.00124284
6	GO_HETEROCHROMATIN_ORGANIZATION	12/975	80/17901	0.00124875
6	GO_REGULATION_OF_POSTSYNAPTIC_DENSITY_O	5/975	16/17901	0.00125145
6	GO_REGULATION_OF_NEURAL_PRECURSOR_CELL_	13/975	91/17901	0.0012658
6	GO_CARDIAC_SEPTUM_DEVELOPMENT	15/975	114/17901	0.00131566
6	GO_CYTOSKELETON_DEPENDENT_INTRACELLULAR	22/975	200/17901	0.00135857
6	GO_AORTA_DEVELOPMENT	10/975	60/17901	0.00137963
6	GO_EPITHELIAL_CELL_PROLIFERATION_INVOLVED_	4/975	10/17901	0.00140969
6	GO_HISTONE_H4_K20_METHYLATION	4/975	10/17901	0.00140969
6	GO_REGULATION_OF_CYTOPLASMIC_MRNA_PROC	4/975	10/17901	0.00140969
6	GO_POSITIVE_REGULATION_OF_MRNA_METABOL	12/975	82/17901	0.00155463
6	GO_POSITIVE_REGULATION_OF_TELOMERE_MAIN	9/975	51/17901	0.00156515

6	GO_REGULATION_OF_PEPTIDYL_LYSINE_ACETYLAT	10/975	61/17901	0.00157113
6	GO_CEREBRAL_CORTEX_CELL_MIGRATION	8/975	42/17901	0.00169868
6	GO_NEGATIVE_REGULATION_OF_RESPONSE_TO_D	12/975	83/17901	0.00172926
6	GO_REGULATION_OF_DEVELOPMENTAL_GROWTH	33/975	353/17901	0.00179411
6	GO_NEGATIVE_REGULATION_OF_SYNAPSE_ORGA	6/975	25/17901	0.00186286
6	GO_POSITIVE_REGULATION_OF_MESENCHYMAL_	6/975	25/17901	0.00186286
6	GO_RENAL_TUBULE_DEVELOPMENT	13/975	95/17901	0.00188651
6	GO_REGULATION_OF_SIGNAL_TRANSDUCTION_BY	20/975	180/17901	0.00194871
6	GO_REGULATION_OF_SYNAPSE_ASSEMBLY	14/975	107/17901	0.00198376
6	GO_REGULATION_OF_TRANS_SYNAPTIC_SIGNALIN	40/975	455/17901	0.0019849
6	GO_POSTSYNAPTIC_SPECIALIZATION_ORGANIZATI	7/975	34/17901	0.00205223
6	GO_POSITIVE_REGULATION_OF_DNA_METHYLATI	4/975	11/17901	0.00212016
6	GO_REGULATION_OF_INTRACELLULAR_TRANSPOR	33/975	357/17901	0.00215214
6	GO_RESPONSE_TO_IONIZING_RADIATION	17/975	144/17901	0.00217072
6	GO_NEGATIVE_REGULATION_OF_GLUCONEOGENE	5/975	18/17901	0.00224137
6	GO_NEURON_PROJECTION_ARBORIZATION	6/975	26/17901	0.00231156
6	GO_REGULATION_OF_CHROMATIN_ASSEMBLY_O	6/975	26/17901	0.00231156
6	GO_REGULATION_OF_CENTROSOME_DUPLICATIO	8/975	44/17901	0.00231699
6	GO_REGULATION_OF_PROTEIN_ACETYLATION	11/975	75/17901	0.00236115
6	GO_NEGATIVE_REGULATION_OF_EPITHELIAL_CELL	9/975	54/17901	0.00236483
6	GO_SPINAL_CORD_DEVELOPMENT	14/975	109/17901	0.00236565
6	GO_DEVELOPMENTAL_GROWTH_INVOLVED_IN_M	24/975	236/17901	0.0024545
6	GO_REGULATION_OF_CELL_DIVISION	19/975	171/17901	0.00249447
6	GO_NEGATIVE_REGULATION_OF_CELL_MORPHOG	13/975	98/17901	0.00250115
6	GO_NEPHRON_EPITHELIUM_DEVELOPMENT	14/975	110/17901	0.00257819
6	GO_NEGATIVE_REGULATION_OF_CELLULAR_CATA	26/975	264/17901	0.00259719
6	GO_CELLULAR_RESPONSE_TO_ACID_CHEMICAL	11/975	76/17901	0.00262793
6	GO_RIBONUCLEOPROTEIN_COMPLEX_SUBUNIT_O	24/975	238/17901	0.00274073
6	GO_GLIOGENESIS	29/975	307/17901	0.00283201
6	GO_REGULATION_OF_AXONOGENESIS	20/975	186/17901	0.00286768
6	GO_SOMATIC_DIVERSIFICATION_OF_IMMUNOGL	10/975	66/17901	0.00287737
6	GO_NEGATIVE_REGULATION_OF_GENE_SILENCIN	7/975	36/17901	0.00289678
6	GO_NUCLEAR_TRANSCRIBED_MRNA_POLY_A_TAI	7/975	36/17901	0.00289678
6	GO_NEGATIVE_REGULATION_OF_DOUBLE_STRAN	5/975	19/17901	0.00290693
6	GO_PERIPHERAL_NERVOUS_SYSTEM_DEVELOPME	11/975	77/17901	0.00291851
6	GO_SOMATIC_DIVERSIFICATION_OF_IMMUNE_RE	11/975	77/17901	0.00291851
6	GO_NEGATIVE_REGULATION_OF_CATABOLIC_PRO	30/975	322/17901	0.00297319
6	GO_TELOMERE_ORGANIZATION	19/975	174/17901	0.00303578
6	GO_DISTAL_TUBULE_DEVELOPMENT	4/975	12/17901	0.00304405
6	GO_MAINTENANCE_OF_ORGANELLE_LOCATION	4/975	12/17901	0.00304405
6	GO_NEGATIVE_REGULATION_OF_NEURON_MIGR	4/975	12/17901	0.00304405
6	GO_POSITIVE_REGULATION_OF_DNA_DEPENDENT	4/975	12/17901	0.00304405
6	GO_POSITIVE_REGULATION_OF_SYNAPTIC_VESICL	4/975	12/17901	0.00304405
6	GO_REGULATION_OF_DNA_REPLICATION	14/975	112/17901	0.00305051
6	GO_REGULATION_OF_NUCLEOCYTOPLASMIC_TRA	14/975	112/17901	0.00305051
6	GO_RECEPTOR_CLUSTERING	9/975	56/17901	0.00305755
6	GO_REGULATION_OF_DNA_TEMPLATED_TRANSC	8/975	46/17901	0.00309914

6	GO_POSITIVE_REGULATION_OF_CELLULAR_PROTE	31/975	337/17901	0.00310263
6	GO_MUSCLE_TISSUE_DEVELOPMENT	36/975	409/17901	0.00318004
6	GO_POSITIVE_REGULATION_OF_SYNAPSE_ASSEMI	10/975	67/17901	0.0032208
6	GO_REGULATION_OF_DENDRITIC_SPINE_DEVELOF	11/975	78/17901	0.0032344
6	GO_APPENDAGE_MORPHOGENESIS	17/975	150/17901	0.00334089
6	GO_RETINA_DEVELOPMENT_IN_CAMERA_TYPE_E	17/975	150/17901	0.00334089
6	GO_POSITIVE_REGULATION_OF_RNA_SPLICING	7/975	37/17901	0.00340772
6	GO_REGULATION_OF_ISOTYPE_SWITCHING	7/975	37/17901	0.00340772
6	GO_REGULATION_OF_TRANSCRIPTION_INITIATION	7/975	37/17901	0.00340772
6	GO_POSITIVE_REGULATION_OF_ISOTYPE_SWITCH	6/975	28/17901	0.00344687
6	GO_POSITIVE_REGULATION_OF_NEUROTRANSMIT	6/975	28/17901	0.00344687
6	GO_REGULATION_OF_MRNA_3_END_PROCESSING	6/975	28/17901	0.00344687
6	GO_SKIN_EPIDERMIS_DEVELOPMENT	12/975	90/17901	0.00345572
6	GO_PROTEIN_LOCALIZATION_TO_CYTOSKELETON	9/975	57/17901	0.00345915
6	GO_MICROTUBULE_BASED_MOVEMENT	33/975	368/17901	0.00347001
6	GO_CHROMOSOME_CONDENSATION	8/975	47/17901	0.00356013
6	GO_ARTERY_DEVELOPMENT	13/975	102/17901	0.00356664
6	GO_REGULATION_OF_GENE_SILENCING	16/975	139/17901	0.00372117
6	GO_CELLULAR_RESPONSE_TO_ALCOHOL	12/975	91/17901	0.00378836
6	GO_MESONEPHROS_DEVELOPMENT	13/975	103/17901	0.00388366
6	GO_REGULATION_OF_TELOMERE_MAINTENANCE	11/975	80/17901	0.00394833
6	GO_REGULATION_OF_SPINDLE_ORGANIZATION	7/975	38/17901	0.00398451
7	GO_REGULATION_OF_GTPASE_ACTIVITY	54/770	479/17901	9.36E-11
7	GO_POSITIVE_REGULATION_OF_GTPASE_ACTIVITY	45/770	404/17901	5.22E-09
7	GO_REGULATION_OF_SMALL_GTPASE_MEDIATED	38/770	323/17901	1.91E-08
7	GO_RNA_SPLICING	49/770	484/17901	2.47E-08
7	GO_REGULATION_OF_RNA_SPLICING	24/770	155/17901	4.94E-08
7	GO_VACUOLAR_TRANSPORT	23/770	150/17901	1.12E-07
7	GO_LYSOSOMAL_TRANSPORT	19/770	110/17901	2.12E-07
7	GO_SEMAPHORIN_PLEXIN_SIGNALING_PATHWAY_	7/770	13/17901	3.63E-07
7	GO_VACUOLAR_ACIDIFICATION	8/770	23/17901	3.12E-06
7	GO_POSITIVE_REGULATION_OF_CELL_ADHESION	40/770	428/17901	3.63E-06
7	GO_LYTIC_VACUOLE_ORGANIZATION	13/770	67/17901	4.61E-06
7	GO_COVALENT_CHROMATIN_MODIFICATION	43/770	481/17901	4.94E-06
7	GO_RNA_SPLICING_VIA_TRANSESTERIFICATION_R	37/770	391/17901	6.31E-06
7	GO_REGULATION_OF_MRNA_SPLICING_VIA_SPLIC	17/770	113/17901	6.52E-06
7	GO_REGULATION_OF_AUTOPHAGY	34/770	347/17901	7.10E-06
7	GO_CELLULAR_MONOVALENT_INORGANIC_CATIO	17/770	114/17901	7.37E-06
7	GO_REGULATION_OF_MRNA_PROCESSING	20/770	152/17901	8.40E-06
7	GO_REGULATION_OF_PHAGOCYTOSIS	15/770	97/17901	1.61E-05
7	GO_POSITIVE_REGULATION_OF_AXONOGENESIS	14/770	86/17901	1.68E-05
7	GO_CYTOSOLIC_TRANSPORT	20/770	165/17901	2.86E-05
7	GO_POSITIVE_REGULATION_OF_PHAGOCYTOSIS	12/770	68/17901	2.92E-05
7	GO_REGULATION_OF_MYELOID_LEUKOCYTE_MED	11/770	58/17901	3.11E-05
7	GO_DEVELOPMENTAL_CELL_GROWTH	25/770	237/17901	3.39E-05
7	GO_REGULATION_OF_CELLULAR_PH	14/770	94/17901	4.71E-05
7	GO_MONOVALENT_INORGANIC_CATION_HOMEO	19/770	159/17901	5.47E-05

7	GO_MAINTENANCE_OF_LOCATION_IN_CELL	23/770	216/17901	5.96E-05
7	GO_ESTABLISHMENT_OF_RNA_LOCALIZATION	22/770	202/17901	6.11E-05
7	GO_VACUOLE_ORGANIZATION	20/770	174/17901	6.13E-05
7	GO_REGULATION_OF_CELL_SUBSTRATE_ADHESIO	23/770	217/17901	6.40E-05
7	GO_MAINTENANCE_OF_LOCATION	30/770	324/17901	6.97E-05
7	GO_AXON_EXTENSION	16/770	123/17901	7.55E-05
7	GO_CELL_SUBSTRATE_ADHESION	32/770	359/17901	8.37E-05
7	GO_GOLGI_ORGANIZATION	18/770	151/17901	8.80E-05
7	GO_NEGATIVE_REGULATION_OF_CELL_ACTIVATIO	22/770	207/17901	8.81E-05
7	GO_REGULATION_OF_CELL_MATRIX_ADHESION	16/770	125/17901	9.18E-05
7	GO_POSITIVE_REGULATION_OF_CELL_MATRIX_AD	10/770	55/17901	0.00010318
7	GO_CLATHRIN_DEPENDENT_ENDOCYTOSIS	9/770	45/17901	0.00010579
7	GO_REGULATION_OF_PH	14/770	103/17901	0.00013013
7	GO_AMINO_SUGAR_CATABOLIC_PROCESS	5/770	13/17901	0.00014018
7	GO_NEGATIVE_REGULATION_OF_PLATELET_DERIV	5/770	13/17901	0.00014018
7	GO_PROTEIN_LOCALIZATION_TO_GOLGI_APPARAT	7/770	28/17901	0.0001421
7	GO_POST_GOLGI_VESICLE_MEDIATED_TRANSPOR	14/770	104/17901	0.00014453
7	GO_POSITIVE_REGULATION_OF_CELL_MORPHOGE	18/770	157/17901	0.00014548
7	GO_REGULATION_OF_LEUKOCYTE_DEGRANULATIO	9/770	47/17901	0.00015063
7	GO_PROTEIN_CONTAINING_COMPLEX_LOCALIZAT	27/770	291/17901	0.00015277
7	GO_CELL_MATRIX_ADHESION	23/770	230/17901	0.00015486
7	GO_PH_REDUCTION	10/770	58/17901	0.00016386
7	GO_POSITIVE_REGULATION_OF_LEUKOCYTE_DIFF	18/770	159/17901	0.00017089
7	GO_PHAGOSOME_MATURATION	9/770	48/17901	0.0001784
7	GO_POSITIVE_REGULATION_OF_HEMOPOIESIS	21/770	204/17901	0.00019779
7	GO_RNA_LOCALIZATION	23/770	235/17901	0.00021277
7	GO_POSITIVE_REGULATION_OF_CELL_SUBSTRATE_	15/770	122/17901	0.00023675
7	GO_PROTEIN_TARGETING_TO_VACUOLE	8/770	40/17901	0.00025552
7	GO_SEMAPHORIN_PLEXIN_SIGNALING_PATHWAY	8/770	40/17901	0.00025552
7	GO_POSITIVE_REGULATION_OF_IMMUNE_EFFECT	22/770	223/17901	0.00025883
7	GO_NEUTRAL_LIPID_BIOSYNTHETIC_PROCESS	9/770	51/17901	0.00028842
7	GO_REGULATION_OF_ENDOCYTOSIS	21/770	210/17901	0.0002947
7	GO_PIGMENTATION	13/770	99/17901	0.00031708
7	GO_CELLULAR_PIGMENTATION	9/770	52/17901	0.00033566
7	GO_ALTERNATIVE_MRNA_SPLICING_VIA_SPLICEOS	12/770	87/17901	0.00033721
7	GO_LYSOSOME_LOCALIZATION	11/770	75/17901	0.00034426
7	GO_MRNA_EXPORT_FROM_NUCLEUS	14/770	113/17901	0.00034918
7	GO_TYPE_I_INTERFERON_PRODUCTION	15/770	127/17901	0.00036795
7	GO_CELL_SUBSTRATE_JUNCTION_ORGANIZATION	13/770	102/17901	0.00042604
7	GO_REGULATION_OF_PLATELET_DERIVED_GROWT	6/770	24/17901	0.00042965
7	GO_TRIGLYCERIDE_BIOSYNTHETIC_PROCESS	8/770	43/17901	0.00042996
7	GO_REGULATION_OF_AXONOGENESIS	19/770	186/17901	0.0004312
7	GO_POSITIVE_REGULATION_OF_CELL_PROJECTION	32/770	394/17901	0.00044973
7	GO_REGULATION_OF_MRNA_METABOLIC_PROCES	29/770	344/17901	0.00044987
7	GO_NEURON_PROJECTION_EXTENSION	18/770	172/17901	0.00045209
7	GO_RNA_EXPORT_FROM_NUCLEUS	16/770	144/17901	0.00047565
7	GO_REGULATION_OF_CELL_SUBSTRATE_JUNCTION	10/770	66/17901	0.0004862

7	GO_DEVELOPMENTAL_GROWTH_INVOLVED_IN_M	22/770	236/17901	0.00056678
7	GO_DICHOTOMOUS_SUBDIVISION_OF_AN_EPITHE	4/770	10/17901	0.0005794
7	GO_POSITIVE_REGULATION_OF_SISTER_CHROMAT	4/770	10/17901	0.0005794
7	GO_T_CELL_ACTIVATION_VIA_T_CELL_RECEPTOR_	4/770	10/17901	0.0005794
7	GO_REGULATION_OF_MACROAUTOPHAGY	18/770	176/17901	0.00059562
7	GO_PROTEIN_AUTOPHOSPHORYLATION	22/770	237/17901	0.00060013
7	GO_REGULATION_OF_ANDROGEN_RECEPTOR_SIG	6/770	26/17901	0.00068288
7	GO_REGULATION_OF_RAS_PROTEIN_SIGNAL_TRA	19/770	194/17901	0.00072564
7	GO_ACTIVATION_OF_GTPASE_ACTIVITY	13/770	108/17901	0.00074105
7	GO_INTEGRIN_MEDIATED_SIGNALING_PATHWAY	13/770	108/17901	0.00074105
7	GO_REGULATION_OF_CLATHRIN_DEPENDENT_EN	5/770	18/17901	0.00077992
7	GO_REGULATION_OF_LEUKOCYTE_MEDIATED_IM	20/770	211/17901	0.0008051
7	GO_REGULATION_OF_CELL_SIZE	18/770	181/17901	0.00082881
7	GO_FOCAL_ADHESION_ASSEMBLY	11/770	83/17901	0.0008313
7	GO_SPLICEOSOMAL_COMPLEX_ASSEMBLY	11/770	83/17901	0.0008313
7	GO_PLATELET_DERIVED_GROWTH_FACTOR_RECE	9/770	59/17901	0.00087698
7	GO_POSITIVE_REGULATION_OF_MONOCYTE_DIFF	4/770	11/17901	0.00087958
7	GO_AMYLOID_BETA_CLEARANCE	7/770	37/17901	0.00088229
7	GO_NUCLEOBASE_CONTAINING_COMPOUND_TRA	23/770	260/17901	0.00088678
7	GO_SEQUESTERING_OF_CALCIUM_ION	14/770	124/17901	0.00089743
7	GO_MACROAUTOPHAGY	26/770	310/17901	0.00093649
7	GO_NEGATIVE_REGULATION_OF_LYMPHOCYTE_A	16/770	154/17901	0.00099239
7	GO_PROTEIN_CATABOLIC_PROCESS_IN_THE_VACU	5/770	19/17901	0.00102127
7	GO_CALCIUM_ION_TRANSMEMBRANE_IMPORT_I	15/770	140/17901	0.00103074
7	GO_RAS_PROTEIN_SIGNAL_TRANSDUCTION	28/770	346/17901	0.0010533
7	GO_ENDOMEMBRANE_SYSTEM_ORGANIZATION	35/770	468/17901	0.00110277
7	GO_POSITIVE_REGULATION_OF_CATABOLIC_PROC	34/770	453/17901	0.00121466
7	GO_INTERFERON_BETA_PRODUCTION	8/770	50/17901	0.00122151
7	GO_POSITIVE_REGULATION_OF_CELL_SUBSTRATE_	6/770	29/17901	0.00126229
7	GO_NEGATIVE_REGULATION_OF_INNATE_IMMUN	9/770	62/17901	0.00126262
7	GO_RESPONSE_TO_MACROPHAGE_COLONY_STIM	4/770	12/17901	0.00127466
7	GO_CELL_GROWTH	36/770	490/17901	0.00128821
7	GO_LYMPHOCYTE_DIFFERENTIATION	29/770	368/17901	0.00130367
7	GO_MACROPHAGE_ACTIVATION	12/770	101/17901	0.00131162
7	GO_NUCLEAR_EXPORT	19/770	204/17901	0.00132408
8	GO_RNA_SPLICING_VIA_TRANSESTERIFICATION_R	88/1204	391/17901	1.89E-24
8	GO_RNA_SPLICING	98/1204	484/17901	1.94E-23
8	GO_REGULATION_OF_MRNA_METABOLIC_PROCES	70/1204	344/17901	3.69E-17
8	GO_DNA_REPLICATION	60/1204	273/17901	1.64E-16
8	GO_VIRAL_GENE_EXPRESSION	49/1204	195/17901	3.61E-16
8	GO_PROTEIN_LOCALIZATION_TO_ENDOPLASMIC_	42/1204	149/17901	5.80E-16
8	GO_RNA_CATABOLIC_PROCESS	76/1204	415/17901	7.55E-16
8	GO_PROTEIN_DNA_COMPLEX_SUBUNIT_ORGANIZ	60/1204	282/17901	8.08E-16
8	GO_REGULATION_OF_CHROMOSOME_ORGANIZAT	69/1204	356/17901	8.67E-16
8	GO_DNA_CONFORMATION_CHANGE	67/1204	355/17901	8.96E-15
8	GO_COTRANSLATIONAL_PROTEIN_TARGETING_TO	33/1204	106/17901	3.34E-14
8	GO_ESTABLISHMENT_OF_PROTEIN_LOCALIZATION	35/1204	121/17901	6.67E-14

8	GO_CHROMOSOME_SEGREGATION	62/1204	334/17901	1.98E-13
8	GO_REGULATION_OF_CELL_CYCLE_PHASE_TRANSI	78/1204	486/17901	4.81E-13
8	GO_CHROMATIN_REMODELING	47/1204	218/17901	6.73E-13
8	GO_SISTER_CHROMATID_SEGREGATION	44/1204	196/17901	8.41E-13
8	GO_REGULATION_OF_RNA_SPLICING	38/1204	155/17901	1.73E-12
8	GO_CELL_CYCLE_G1_S_PHASE_TRANSITION	57/1204	310/17901	2.83E-12
8	GO_REGULATION_OF_MRNA_PROCESSING	37/1204	152/17901	4.27E-12
8	GO_POSITIVE_REGULATION_OF_CHROMOSOME_(	41/1204	182/17901	4.49E-12
8	GO_DNA_RECOMBINATION	54/1204	294/17901	1.11E-11
8	GO_POSITIVE_REGULATION_OF_CELL_CYCLE	65/1204	396/17901	1.64E-11
8	GO_NUCLEAR_CHROMOSOME_SEGREGATION	51/1204	272/17901	1.90E-11
8	GO_MITOTIC_SISTER_CHROMATID_SEGREGATION	37/1204	161/17901	2.66E-11
8	GO_MITOTIC_NUCLEAR_DIVISION	51/1204	277/17901	3.81E-11
8	GO_POSITIVE_REGULATION_OF_CELL_CYCLE_PRO	53/1204	302/17901	1.02E-10
8	GO_REGULATION_OF_MRNA_SPLICING_VIA_SPLIC	29/1204	113/17901	2.27E-10
8	GO_NEGATIVE_REGULATION_OF_CELL_CYCLE_PR(	58/1204	359/17901	3.86E-10
8	GO_NUCLEAR_TRANSCRIBED_MRNA_CATABOLIC_	41/1204	210/17901	5.07E-10
8	GO_CHROMATIN_ASSEMBLY_OR_DISASSEMBLY	42/1204	220/17901	6.43E-10
8	GO_NUCLEOSOME_ORGANIZATION	37/1204	183/17901	1.28E-09
8	GO_REGULATION_OF_CHROMOSOME_SEGREGATI	27/1204	107/17901	1.42E-09
8	GO_MICROTUBULE_CYTOSKELETON_ORGANIZATI(	32/1204	144/17901	1.43E-09
8	GO_TRANSLATIONAL_INITIATION	38/1204	192/17901	1.45E-09
8	GO_ALTERNATIVE_MRNA_SPLICING_VIA_SPLICEOS	24/1204	87/17901	1.66E-09
8	GO_NEGATIVE_REGULATION_OF_MRNA_METABO	24/1204	88/17901	2.13E-09
8	GO_PROTEIN_FOLDING	42/1204	230/17901	2.62E-09
8	GO_DNA_PACKAGING	43/1204	240/17901	3.10E-09
8	GO_REGULATION_OF_SISTER_CHROMATID_SEGRE	23/1204	84/17901	4.23E-09
8	GO_NUCLEAR_TRANSCRIBED_MRNA_CATABOLIC_	28/1204	120/17901	4.76E-09
8	GO_RNA_LOCALIZATION	42/1204	235/17901	5.09E-09
8	GO_REGULATION_OF_DNA_METABOLIC_PROCESS	56/1204	365/17901	5.38E-09
8	GO_PEPTIDYL_LYSINE_MODIFICATION	60/1204	405/17901	5.93E-09
8	GO_CELL_CYCLE_G2_M_PHASE_TRANSITION	46/1204	273/17901	6.65E-09
8	GO_NEGATIVE_REGULATION_OF_MITOTIC_CELL_(	53/1204	341/17901	8.80E-09
8	GO_RIBONUCLEOPROTEIN_COMPLEX_BIOGENESIS	67/1204	480/17901	9.08E-09
8	GO_SIGNAL_TRANSDUCTION_BY_P53_CLASS_MED	45/1204	267/17901	9.63E-09
8	GO_RECOMBINATIONAL_REPAIR	30/1204	140/17901	1.14E-08
8	GO_REGULATION_OF_CELL_CYCLE_G2_M_PHASE_	39/1204	217/17901	1.52E-08
8	GO_ATP_DEPENDENT_CHROMATIN_REMODELING	23/1204	90/17901	1.79E-08
8	GO_RESPONSE_TO_ENDOPLASMIC_RETICULUM_S	47/1204	293/17901	2.25E-08
8	GO_RIBONUCLEOPROTEIN_COMPLEX_SUBUNIT_O	41/1204	238/17901	2.30E-08
8	GO_CELL_CYCLE_DNA_REPLICATION	19/1204	65/17901	2.92E-08
8	GO_NEGATIVE_REGULATION_OF_RNA_SPLICING	12/1204	26/17901	3.24E-08
8	GO_NEGATIVE_REGULATION_OF_CELL_CYCLE_PHA	44/1204	270/17901	3.92E-08
8	GO_DNA_GEOMETRIC_CHANGE	26/1204	116/17901	4.06E-08
8	GO_PROTEIN_TARGETING_TO_MEMBRANE	37/1204	207/17901	4.12E-08
8	GO_NEGATIVE_REGULATION_OF_CHROMOSOME_	30/1204	148/17901	4.35E-08
8	GO_REGULATION_OF_MRNA_CATABOLIC_PROCES	37/1204	208/17901	4.69E-08

8	GO_ESTABLISHMENT_OF_PROTEIN_LOCALIZATION	51/1204	339/17901	4.97E-08
8	GO_POSITIVE_REGULATION_OF_DNA_METABOLIC	36/1204	200/17901	5.20E-08
8	GO_FOREBRAIN_DEVELOPMENT	56/1204	391/17901	6.27E-08
8	GO_DNA_DEPENDENT_DNA_REPLICATION	30/1204	151/17901	6.98E-08
8	GO_REGULATION_OF_CHROMATIN_ORGANIZATIO	35/1204	195/17901	8.55E-08
8	GO_RESPONSE_TO_TOPOLOGICALLY_INCORRECT_	36/1204	204/17901	8.75E-08
8	GO_ORGANELLE_FISSION	63/1204	467/17901	9.09E-08
8	GO_NUCLEAR_TRANSPORT	52/1204	357/17901	1.07E-07
8	GO_NEURON_MIGRATION	31/1204	164/17901	1.39E-07
8	GO_CHROMOSOME_SEPARATION	22/1204	94/17901	1.97E-07
8	GO_MITOTIC_SPINDLE_ORGANIZATION	25/1204	118/17901	2.35E-07
8	GO_COVALENT_CHROMATIN_MODIFICATION	63/1204	481/17901	2.66E-07
8	GO_SIGNAL_TRANSDUCTION_INVOLVED_IN_CELL_	19/1204	74/17901	2.84E-07
8	GO_NEGATIVE_REGULATION_OF_MRNA_PROCESS	12/1204	31/17901	3.45E-07
8	GO_PEPTIDYL_LYSINE_ACETYLATION	31/1204	172/17901	4.20E-07
8	GO_SPINDLE_ORGANIZATION	32/1204	183/17901	5.56E-07
8	GO_NEGATIVE_REGULATION_OF_MRNA_SPLICING	10/1204	22/17901	5.59E-07
8	GO_ESTABLISHMENT_OF_RNA_LOCALIZATION	34/1204	202/17901	6.26E-07
8	GO_REGULATION_OF_HISTONE_MODIFICATION	28/1204	149/17901	6.27E-07
8	GO_TELENCEPHALON_DEVELOPMENT	40/1204	259/17901	6.85E-07
8	GO_PROTEIN_CONTAINING_COMPLEX_LOCALIZAT	43/1204	291/17901	9.04E-07
8	GO_DOUBLE_STRAND_BREAK_REPAIR	40/1204	262/17901	9.27E-07
8	GO_REGULATION_OF_CHROMOSOME_SEPARATIO	17/1204	66/17901	1.13E-06
8	GO_CELL_CYCLE_CHECKPOINT	35/1204	219/17901	1.50E-06
8	GO_DNA_REPLICATION_INDEPENDENT_NUCLEOS(	15/1204	54/17901	1.71E-06
8	GO_REGULATION_OF_CELL_CYCLE_ARREST	22/1204	106/17901	1.77E-06
8	GO_MITOTIC_CELL_CYCLE_CHECKPOINT	29/1204	166/17901	1.90E-06
8	GO_CHROMATIN_DISASSEMBLY	9/1204	20/17901	2.33E-06
8	GO_PROTEIN_DNA_COMPLEX_DISASSEMBLY	9/1204	20/17901	2.33E-06
8	GO_REGULATION_OF_CELL_CYCLE_G1_S_PHASE_T	33/1204	206/17901	2.82E-06
8	GO_CENTROMERE_COMPLEX_ASSEMBLY	15/1204	56/17901	2.83E-06
8	GO_REGULATION_OF_NUCLEAR_DIVISION	31/1204	188/17901	3.02E-06
8	GO_PROTEIN_TARGETING	56/1204	441/17901	3.23E-06
8	GO_REGULATION_OF_STEM_CELL_DIFFERENTIATI(	23/1204	118/17901	3.23E-06
8	GO_POSITIVE_REGULATION_OF_DNA_REPAIR	17/1204	71/17901	3.39E-06
8	GO_RNA_EXPORT_FROM_NUCLEUS	26/1204	144/17901	3.40E-06
8	GO_MITOTIC_G1_S_TRANSITION_CHECKPOINT	16/1204	64/17901	3.58E-06
8	GO_DOUBLE_STRAND_BREAK_REPAIR_VIA_BREAK	7/1204	12/17901	3.59E-06
8	GO_REGULATION_OF_ESTABLISHMENT_OF_PROTE	7/1204	12/17901	3.59E-06
8	GO_CYTOPLASMIC_TRANSLATION	21/1204	103/17901	4.07E-06
8	GO_NEGATIVE_REGULATION_OF_RESPONSE_TO_E	14/1204	51/17901	4.41E-06
8	GO_MAINTENANCE_OF_CELL_NUMBER	28/1204	164/17901	4.51E-06
8	GO_NEGATIVE_REGULATION_OF_CHROMOSOME_	13/1204	45/17901	5.19E-06
8	GO_KINETOCHORE_ORGANIZATION	9/1204	22/17901	6.10E-06
8	GO_POSITIVE_REGULATION_OF_CHROMATIN_OR(	21/1204	106/17901	6.55E-06
8	GO_NEGATIVE_REGULATION_OF_CELL_CYCLE_G1_	24/1204	132/17901	7.06E-06
8	GO_METAPHASE_ANAPHASE_TRANSITION_OF_CE	15/1204	60/17901	7.18E-06

8	GO_REGULATION_OF_PEPTIDYL_LYSINE_ACETYLAT	15/1204	61/17901	8.93E-06
8	GO_REGULATION_OF_SIGNAL_TRANSDUCTION_BY	29/1204	180/17901	9.90E-06
8	GO_NEURAL_PRECURSOR_CELL_PROLIFERATION	26/1204	154/17901	1.20E-05
8	GO_NUCLEOSOME_ASSEMBLY	25/1204	145/17901	1.21E-05
8	GO_SISTER_CHROMATID_COHESION	15/1204	63/17901	1.36E-05
8	GO_REGULATION_OF_PROTEIN_LOCALIZATION_TO	7/1204	14/17901	1.38E-05
8	GO_PROTEIN_ACETYLATION	31/1204	203/17901	1.50E-05
8	GO_POSITIVE_REGULATION_OF_ESTABLISHMENT_	6/1204	10/17901	1.52E-05
8	GO_PROTEIN_FOLDING_IN_ENDOPLASMIC_RETICU	6/1204	10/17901	1.52E-05
8	GO_POSITIVE_REGULATION_OF_CELL_CYCLE_G2_	10/1204	30/17901	1.58E-05
8	GO_NUCLEAR_EXPORT	31/1204	204/17901	1.66E-05
8	GO_PROTEOGLYCAN_BIOSYNTHETIC_PROCESS	15/1204	64/17901	1.67E-05
8	GO_CELL_CYCLE_ARREST	34/1204	234/17901	1.75E-05
8	GO_POSITIVE_REGULATION_OF_NEURON_DIFFERE	48/1204	380/17901	1.84E-05
8	GO_GLYCOPROTEIN_METABOLIC_PROCESS	51/1204	413/17901	1.90E-05
8	GO_RESPONSE_TO_UV	24/1204	140/17901	1.98E-05
8	GO_NEGATIVE_REGULATION_OF_METAPHASE_AN	11/1204	37/17901	2.04E-05
8	GO_POSITIVE_REGULATION_OF_MITOTIC_CELL_CY	27/1204	168/17901	2.07E-05
8	GO_CHONDROITIN_SULFATE_PROTEOGLYCAN_BIO	10/1204	31/17901	2.20E-05
8	GO_PEPTIDYL_ASPARAGINE_MODIFICATION	10/1204	31/17901	2.20E-05
8	GO_POSITIVE_REGULATION_OF_CELL_CYCLE_ARRE	17/1204	81/17901	2.22E-05
8	GO_REGULATION_OF_DNA_REPAIR	23/1204	132/17901	2.23E-05
8	GO_GLYCOPROTEIN_BIOSYNTHETIC_PROCESS	44/1204	341/17901	2.41E-05
8	GO_SIGNAL_TRANSDUCTION_IN_RESPONSE_TO_D	23/1204	133/17901	2.53E-05
8	GO_ENDOPLASMIC_RETICULUM_UNFOLDED_PROT	22/1204	125/17901	2.85E-05
8	GO_NUCLEOBASE_CONTAINING_COMPOUND_TRA	36/1204	260/17901	2.92E-05
8	GO_REGULATION_OF_HISTONE_METHYLATION	15/1204	67/17901	2.99E-05
8	GO_REGULATION_OF_RESPONSE_TO_ENDOPLASM	18/1204	91/17901	3.00E-05
8	GO_OSTEOBLAST_DIFFERENTIATION	33/1204	230/17901	3.01E-05
8	GO_NEGATIVE_REGULATION_OF_NUCLEAR_DIVISI	14/1204	60/17901	3.35E-05
8	GO_PALLIUM_DEVELOPMENT	27/1204	173/17901	3.54E-05
8	GO_MRNA_3_END_PROCESSING	19/1204	101/17901	3.80E-05
8	GO_POSITIVE_REGULATION_OF_CELL_CYCLE_PHA	20/1204	110/17901	4.01E-05
8	GO_POSITIVE_REGULATION_OF_CHROMOSOME_S	10/1204	33/17901	4.05E-05
8	GO_DERMATAN_SULFATE_PROTEOGLYCAN_META	7/1204	16/17901	4.08E-05
8	GO_CELLULAR_RESPONSE_TO_TOPOLOGICALLY_IN	26/1204	166/17901	4.61E-05
8	GO_CHROMATIN_REMODELING_AT_CENTROMERE	12/1204	47/17901	4.68E-05
8	GO_REGULATION_OF_RESPONSE_TO_DNA_DAMA	32/1204	226/17901	5.13E-05
8	GO_REGULATION_OF_NUCLEASE_ACTIVITY	8/1204	22/17901	5.58E-05
8	GO_REGULATION_OF_SISTER_CHROMATID_COHES	8/1204	22/17901	5.58E-05
8	GO_RESPONSE_TO_DRUG	48/1204	397/17901	5.67E-05
8	GO_REGULATION_OF_DNA_TEMPLATED_TRANSCR	12/1204	48/17901	5.86E-05
8	GO_POSITIVE_REGULATION_OF_VIRAL_TRANSCRIP	11/1204	41/17901	5.89E-05
8	GO_PROTEIN_LOCALIZATION_TO_NUCLEAR_BODY	6/1204	12/17901	5.94E-05
8	GO_DNA_TEMPLATED_TRANSCRIPTION_INITIATIO	34/1204	249/17901	6.39E-05
8	GO_SYNAPSE_ORGANIZATION	51/1204	433/17901	6.67E-05
8	GO_POSITIVE_REGULATION_OF_PROTEIN_LOCALIZ	16/1204	80/17901	7.12E-05

8	GO_DNA_INTEGRITY_CHECKPOINT	25/1204	161/17901	7.39E-05
8	GO_REGULATION_OF_DNA_BINDING	21/1204	124/17901	7.68E-05
8	GO_DNA_BIOSYNTHETIC_PROCESS	28/1204	191/17901	8.01E-05
8	GO_NEURONAL_STEM_CELL_POPULATION_MAINT	8/1204	23/17901	8.06E-05
8	GO_HISTONE_METHYLATION	23/1204	143/17901	8.17E-05
8	GO_CELLULAR_RESPONSE_TO_UV	16/1204	81/17901	8.33E-05
8	GO_RHYTHMIC_PROCESS	39/1204	305/17901	8.35E-05
8	GO_REGULATION_OF_VIRAL_TRANSCRIPTION	14/1204	65/17901	8.58E-05
8	GO_DNA_DAMAGE_RESPONSE_SIGNAL_TRANSDU	19/1204	107/17901	8.64E-05
8	GO_POSITIVE_REGULATION_OF_VIRAL_PROCESS	19/1204	107/17901	8.64E-05
8	GO_REGULATION_OF_DNA_RECOMBINATION	19/1204	107/17901	8.64E-05
8	GO_NEGATIVE_REGULATION_OF_ORGANELLE_OR	49/1204	415/17901	8.66E-05
8	GO_RNA_3_END_PROCESSING	24/1204	153/17901	8.68E-05
8	GO_DNA_TEMPLATED_TRANSCRIPTION_ELONGAT	20/1204	116/17901	8.73E-05
8	GO_NEURON_PROJECTION_GUIDANCE	37/1204	285/17901	9.16E-05
8	GO_MITOTIC_DNA_INTEGRITY_CHECKPOINT	19/1204	108/17901	9.83E-05
8	GO_MICROTUBULE_POLYMERIZATION_OR_DEPOL	20/1204	117/17901	9.87E-05
8	GO_ESTABLISHMENT_OF_PROTEIN_LOCALIZATION	7/1204	18/17901	0.00010074
8	GO_POSITIVE_REGULATION_OF_HISTONE_H3_K4_	7/1204	18/17901	0.00010074
8	GO_TELOMERE_ORGANIZATION	26/1204	174/17901	0.00010311
8	GO_DERMATAN_SULFATE_METABOLIC_PROCESS	6/1204	13/17901	0.00010399
8	GO_NEGATIVE_REGULATION_OF_NERVOUS_SYSTE	40/1204	319/17901	0.00010481
8	GO_PROTEIN_LOCALIZATION_TO_CHROMOSOME_	9/1204	30/17901	0.00010752
8	GO_CEREBRAL_CORTEX_DEVELOPMENT	20/1204	118/17901	0.00011148
8	GO_REGULATION_OF_DNA_BIOSYNTHETIC_PROCE	19/1204	109/17901	0.00011169
8	GO_NEGATIVE_REGULATION_OF_NEURON_DIFFEF	31/1204	225/17901	0.00011175
8	GO_ANAPHASE_PROMOTING_COMPLEX_DEPENDI	16/1204	83/17901	0.00011285
8	GO_CHONDROITIN_SULFATE_PROTEOGLYCAN_ME	11/1204	44/17901	0.00011861
8	GO_REGULATION_OF_PROTEIN_ACETYLATION	15/1204	75/17901	0.00011876
8	GO_HISTONE_EXCHANGE	13/1204	59/17901	0.00011923
8	GO_DNA_STRAND_ELONGATION_INVOLVED_IN_D	7/1204	19/17901	0.00015028
8	GO_PROTEIN_LOCALIZATION_TO_CHROMOSOME	16/1204	85/17901	0.00015126
8	GO_POSITIVE_REGULATION_OF_DNA_BIOSYNTHET	14/1204	69/17901	0.00016905
8	GO_REGULATION_OF_TELOMERE_MAINTENANCE_	13/1204	61/17901	0.00017062
8	GO_MAINTENANCE_OF_PROTEIN_LOCALIZATION_	6/1204	14/17901	0.00017163
8	GO_PROTEIN_LOCALIZATION_TO_NUCLEOPLASM	6/1204	14/17901	0.00017163
8	GO_MRNA_TRANSPORT	23/1204	151/17901	0.00018939
8	GO_NEGATIVE_REGULATION_OF_PROTEIN_CATAB	21/1204	132/17901	0.00019161
8	GO_PEPTIDYL_LYSINE_METHYLATION	21/1204	132/17901	0.00019161
8	GO_SPLICEOSOMAL_SNRNP_ASSEMBLY	12/1204	54/17901	0.00019841
8	GO_POSITIVE_REGULATION_OF_RESPONSE_TO_D	18/1204	105/17901	0.00020828
8	GO_CHONDROITIN_SULFATE_BIOSYNTHETIC_PROC	8/1204	26/17901	0.00021417
8	GO_DNA_STRAND_ELONGATION	8/1204	26/17901	0.00021417
8	GO_MITOTIC_SISTER_CHROMATID_COHESION	8/1204	26/17901	0.00021417
8	GO_SPINDLE_ASSEMBLY	19/1204	115/17901	0.000231
8	GO_RNA_DEPENDENT_DNA_BIOSYNTHETIC_PROC	14/1204	71/17901	0.00023208
8	GO_OSSIFICATION	47/1204	410/17901	0.00023947

8	GO_REGULATION_OF_MESENCHYMAL_CELL_PROL	9/1204	33/17901	0.00024132
8	GO_RIBOSOME_BIOGENESIS	38/1204	310/17901	0.00024564
8	GO_REGULATION_OF_TELOMERE_MAINTENANCE	15/1204	80/17901	0.00025199
8	GO_PROTEIN_METHYLATION	26/1204	184/17901	0.00025838
8	GO_POSITIVE_REGULATION_OF_SISTER_CHROMAT	5/1204	10/17901	0.00025849
8	GO_NEGATIVE_REGULATION_OF_CATABOLIC_PRO	39/1204	322/17901	0.0002624
8	GO_POSITIVE_REGULATION_OF_TELOMERASE_RN	6/1204	15/17901	0.00026981
8	GO_ESTABLISHMENT_OF_PROTEIN_LOCALIZATION	8/1204	27/17901	0.00028653
8	GO_NUCLEOBASE_CONTAINING_SMALL_MOLECUL	8/1204	27/17901	0.00028653
8	GO_TELOMERE_MAINTENANCE_VIA_TELOMERE_L	15/1204	81/17901	0.00029034
8	GO_PROTEIN_ACYLATION	32/1204	248/17901	0.0002967
8	GO_POSITIVE_REGULATION_OF_HISTONE_METHY	10/1204	41/17901	0.00029946
8	GO_PROTEOGLYCAN_METABOLIC_PROCESS	16/1204	90/17901	0.0003006
8	GO_DENDRITE_MORPHOGENESIS	22/1204	146/17901	0.00030229
8	GO_ATTACHMENT_OF_SPINDLE_MICROTUBULES_	9/1204	34/17901	0.00030879
8	GO_NEGATIVE_REGULATION_OF_DNA_METABOLI	20/1204	127/17901	0.00030963
8	GO_PROTEIN_N_LINKED_GLYCOSYLATION	14/1204	73/17901	0.00031433
8	GO_MITOTIC_SPINDLE_ASSEMBLY	13/1204	65/17901	0.00033213
8	GO_CENTRAL_NERVOUS_SYSTEM_NEURON_DEVE	15/1204	82/17901	0.0003336
8	GO_RIBOSOMAL_LARGE_SUBUNIT_BIOGENESIS	14/1204	74/17901	0.00036406
8	GO_MAINTENANCE_OF_PROTEIN_LOCALIZATION_	10/1204	42/17901	0.00036961
8	GO_SPLICEOSOMAL_COMPLEX_ASSEMBLY	15/1204	83/17901	0.0003823
8	GO_REGULATION_OF_PROTEIN_STABILITY	36/1204	295/17901	0.0003849
8	GO_POSITIVE_REGULATION_OF_CYCLIN_DEPENDE	9/1204	35/17901	0.00039111
8	GO_REGULATION_OF_TRANSCRIPTION_INVOLVED	9/1204	35/17901	0.00039111
8	GO_RNA_STABILIZATION	12/1204	58/17901	0.00040208
8	GO_MITOTIC_DNA_REPLICATION	6/1204	16/17901	0.00040721
8	GO_GLIOGENESIS	37/1204	307/17901	0.0004102
8	GO_REGULATION_OF_MICROTUBULE_CYTOSKELET	26/1204	190/17901	0.00042954
8	GO_AGING	38/1204	319/17901	0.00043443
8	GO_REGULATION_OF_SYMBIOTIC_PROCESS	30/1204	232/17901	0.00043539
8	GO_VIRAL_LATENCY	5/1204	11/17901	0.00044767
8	GO_RIBOSOME_ASSEMBLY	13/1204	67/17901	0.00045282
8	GO_CALCIUM_DEPENDENT_CELL_CELL_ADHESION	10/1204	43/17901	0.00045293
8	GO_REGULATION_OF_ANIMAL_ORGAN_MORPHO	32/1204	254/17901	0.0004547
8	GO_REGULATION_OF_DNA_REPLICATION	18/1204	112/17901	0.00047205
8	GO_CELL_REDOX_HOMEOSTASIS	12/1204	59/17901	0.0004743
8	GO_POSITIVE_REGULATION_OF_TELOMERE_MAIN	11/1204	51/17901	0.00047742
8	GO_REGULATION_OF_HISTONE_H3_K4_METHYLAT	8/1204	29/17901	0.00049108
8	GO_PROTEIN_LOCALIZATION_TO_NUCLEUS	34/1204	277/17901	0.00049628
8	GO_REGULATION_OF_MICROTUBULE_POLYMERIZ	15/1204	85/17901	0.00049819
8	GO_NEGATIVE_REGULATION_OF_RNA_CATABOLIC	13/1204	68/17901	0.00052593
8	GO_MRNA_EXPORT_FROM_NUCLEUS	18/1204	113/17901	0.00052689
8	GO_HEMATOPOIETIC_PROGENITOR_CELL_DIFFERE	24/1204	172/17901	0.0005279
8	GO_MICROTUBULE_POLYMERIZATION	14/1204	77/17901	0.00055547
8	GO_LUNG_MORPHOGENESIS	11/1204	52/17901	0.00056922
8	GO_POSTREPLICATION_REPAIR	11/1204	52/17901	0.00056922

8	GO_KINETOCHORE_ASSEMBLY	6/1204	17/17901	0.00059368
8	GO_POSITIVE_REGULATION_OF_TELOMERE_MAIN	9/1204	37/17901	0.00061016
8	GO_TRANSCRIPTION_PREINITIATION_COMPLEX_A	9/1204	37/17901	0.00061016
8	GO_POSITIVE_REGULATION_OF_FILOPODIUM_ASS	8/1204	30/17901	0.00063056
8	GO_HEMATOPOIETIC_STEM_CELL_DIFFERENTIATIO	15/1204	87/17901	0.00064284
8	GO_REGULATION_OF_CELL_MORPHOGENESIS	53/1204	499/17901	0.00064455
8	GO_POSITIVE_REGULATION_OF_CELL_PROJECTION	44/1204	394/17901	0.00064969
8	GO_POSITIVE_REGULATION_OF_CELLULAR_PROTE	39/1204	337/17901	0.00065096
8	GO_HISTONE_H4_ACETYLATION	13/1204	70/17901	0.00070236
8	GO_COMMISSURAL_NEURON_AXON_GUIDANCE	5/1204	12/17901	0.00072502
8	GO_ER_OVERLOAD_RESPONSE	5/1204	12/17901	0.00072502
8	GO_REGULATION_OF_HELICASE_ACTIVITY	5/1204	12/17901	0.00072502
8	GO_REGULATION_OF_ATPASE_ACTIVITY	14/1204	79/17901	0.00072578
8	GO_DNA_DAMAGE_RESPONSE_DETECTION_OF_D	9/1204	38/17901	0.00075238
8	GO_ENDOPLASMIC_RETICULUM_CALCIUM_ION_H	7/1204	24/17901	0.00076685
8	GO_PROTEIN_MANNOSYLATION	7/1204	24/17901	0.00076685
8	GO_REGULATION_OF_UBIQUITIN_PROTEIN_LIGAS	7/1204	24/17901	0.00076685
8	GO_REGULATION_OF_SYNAPSE_STRUCTURE_OR_A	30/1204	240/17901	0.00076767
8	GO_MICROTUBULE_ORGANIZING_CENTER_LOCALI	8/1204	31/17901	0.00080032
8	GO_G0_TO_G1_TRANSITION	10/1204	46/17901	0.00080116
8	GO_REGULATION_OF_PROTEIN_LOCALIZATION_TO	19/1204	127/17901	0.00082861
8	GO_ANATOMICAL_STRUCTURE_HOMEOSTASIS	50/1204	469/17901	0.00083329
8	GO_NEGATIVE_REGULATION_OF_CELL_DEVELOPM	39/1204	342/17901	0.00086422
8	GO_NEGATIVE_REGULATION_OF_CELLULAR_CATA	32/1204	264/17901	0.0008864
8	GO_STEM_CELL_DIFFERENTIATION	32/1204	264/17901	0.0008864
8	GO_TRANSCRIPTION_INITIATION_FROM_RNA_POL	25/1204	189/17901	0.00090718
8	GO_POSITIVE_REGULATION_OF_ATPASE_ACTIVITY	11/1204	55/17901	0.00093699
8	GO_REGULATION_OF_DOUBLE_STRAND_BREAK_R	10/1204	47/17901	0.00095724
8	GO_REGULATION_OF_CELL_MORPHOGENESIS_INV	36/1204	310/17901	0.00096747
8	GO_HEPARAN_SULFATE_PROTEOGLYCAN_METABO	8/1204	32/17901	0.00100491
8	GO_VENTRICULAR_SYSTEM_DEVELOPMENT	8/1204	32/17901	0.00100491
8	GO_REGULATION_OF_NEURAL_PRECURSOR_CELL_	15/1204	91/17901	0.0010412
8	GO_REGULATION_OF_HEMATOPOIETIC_STEM_CE	13/1204	73/17901	0.0010587
8	GO_NEGATIVE_REGULATION_OF_ENDOPLASMIC_	5/1204	13/17901	0.00111317
8	GO_REGULATION_OF_ATTACHMENT_OF_SPINDLE	5/1204	13/17901	0.00111317
8	GO_POSITIVE_REGULATION_OF_NEURON_PROJEC	34/1204	290/17901	0.00111963
8	GO_IMPORT_INTO_NUCLEUS	23/1204	171/17901	0.0011371
8	GO_TELOMERASE_RNA_LOCALIZATION	6/1204	19/17901	0.00115854
8	GO_SENSORY_SYSTEM_DEVELOPMENT	43/1204	394/17901	0.00118055
8	GO_IN_UTERO_EMBRYONIC_DEVELOPMENT	42/1204	383/17901	0.00121956
8	GO_DENDRITE_DEVELOPMENT	30/1204	247/17901	0.00122281
8	GO_REGULATION_OF_PROTEIN_CATABOLIC_PROC	43/1204	395/17901	0.0012411
8	GO_HISTONE_H3_K4_METHYLATION	11/1204	57/17901	0.00127735
8	GO_INTRACELLULAR_ESTROGEN_RECEPTOR_SIGNA	11/1204	57/17901	0.00127735
8	GO_HEPARAN_SULFATE_PROTEOGLYCAN_BIOSYNT	7/1204	26/17901	0.00129474
8	GO_HISTONE_H3_K9_MODIFICATION	10/1204	49/17901	0.00134427
8	GO_POSITIVE_REGULATION_OF_NUCLEOCYTOPLA	12/1204	66/17901	0.00135072

8	GO_RIBOSOMAL_SMALL_SUBUNIT_BIOGENESIS	13/1204	75/17901	0.00137163
8	GO_NOTCH_SIGNALING_PATHWAY	25/1204	195/17901	0.00142131
8	GO_POSITIVE_REGULATION_OF_ANIMAL_ORGAN_	14/1204	85/17901	0.00152227
8	GO_VIRAL_LIFE_CYCLE	38/1204	341/17901	0.00152517
8	GO_IRE1_MEDIATED_UNFOLDED_PROTEIN_RESPO	12/1204	67/17901	0.00154663
8	GO_NEURON_RECOGNITION	10/1204	50/17901	0.00158076
8	GO_REGULATION_OF_CHOLESTEROL_BIOSYNTHET	10/1204	50/17901	0.00158076
8	GO_SPINDLE_LOCALIZATION	10/1204	50/17901	0.00158076
8	GO_CILIARY_BASAL_BODY_PLASMA_MEMBRANE_	15/1204	95/17901	0.00163022
8	GO_REGULATION_OF_TRANSCRIPTION_ELONGATI	7/1204	27/17901	0.00164761
8	GO_NEGATIVE_REGULATION_OF_CELLULAR_PROT	14/1204	86/17901	0.0017081
8	GO_REGULATION_OF_HEMATOPOIETIC_PROGENIT	14/1204	86/17901	0.0017081
8	GO_PROTEIN_STABILIZATION	24/1204	187/17901	0.00172581
8	GO_AMINOGLYCAN_BIOSYNTHETIC_PROCESS	17/1204	115/17901	0.00173778
8	GO_CAMERA_TYPE_EYE_DEVELOPMENT	37/1204	332/17901	0.0017435
8	GO_HINDBRAIN_DEVELOPMENT	21/1204	156/17901	0.00180667
8	GO_DEOXYRIBONUCLEOTIDE_METABOLIC_PROCES	8/1204	35/17901	0.00187863
8	GO_ESTABLISHMENT_OF_MITOTIC_SPINDLE_LOCA	8/1204	35/17901	0.00187863
8	GO_REGULATION_OF_CYCLIN_DEPENDENT_PROTE	16/1204	106/17901	0.00188031
8	GO_MEIOTIC_CELL_CYCLE	30/1204	254/17901	0.00189626
8	GO_RETROGRADE_VESICLE_MEDIATED_TRANSPOR	14/1204	87/17901	0.00191239
8	GO_TRANSCRIPTION_ELONGATION_FROM_RNA_P	14/1204	87/17901	0.00191239
8	GO_PROTEIN_PEPTIDYL_PROLYL_ISOMERIZATION	9/1204	43/17901	0.00192236
8	GO_RESPONSE_TO_LEUKEMIA_INHIBITORY_FACTO	15/1204	97/17901	0.00201608
8	GO_RIBOSOMAL_SMALL_SUBUNIT_ASSEMBLY	6/1204	21/17901	0.00206324
8	GO_REGULATION_OF_ENDOPLASMIC_RETICULUM	7/1204	28/17901	0.00207092
8	GO_REGULATION_OF_POSTSYNAPSE_ORGANIZATI	16/1204	107/17901	0.00207608
8	GO_REGULATION_OF_DNA_DEPENDENT_DNA_RE	10/1204	52/17901	0.00215477
8	GO_RESPONSE_TO_NERVE_GROWTH_FACTOR	10/1204	52/17901	0.00215477
8	GO_CHROMOSOME_LOCALIZATION	13/1204	79/17901	0.00222968
8	GO_FILOPODIUM_ASSEMBLY	11/1204	61/17901	0.00226329
8	GO_HIPPO_SIGNALING	9/1204	44/17901	0.00227493
8	GO_MESENCHYMAL_CELL_PROLIFERATION	9/1204	44/17901	0.00227493
8	GO_CENTRAL_NERVOUS_SYSTEM_NEURON_AXON	8/1204	36/17901	0.00227519
8	GO_ESTROUS_CYCLE	5/1204	15/17901	0.00231945
8	GO_NEGATIVE_REGULATION_OF_CENTROSOME_C	5/1204	15/17901	0.00231945
8	GO_NEGATIVE_REGULATION_OF_DNA_BINDING	10/1204	53/17901	0.00249884
8	GO_MEIOTIC_CELL_CYCLE_PROCESS	24/1204	193/17901	0.00263605
8	GO_POSITIVE_REGULATION_OF_DNA_RECOMBINA	9/1204	45/17901	0.00267701
8	GO_VENTRICULAR_SEPTUM_MORPHOGENESIS	9/1204	45/17901	0.00267701
8	GO_NEGATIVE_REGULATION_OF_RHO_PROTEIN_S	6/1204	22/17901	0.0026774
8	GO_MANNOSYLATION	8/1204	37/17901	0.00273451
8	GO_POSITIVE_REGULATION_OF_VIRAL_GENOME_	8/1204	37/17901	0.00273451
8	GO_NEGATIVE_REGULATION_OF_EPITHELIAL_CELL	10/1204	54/17901	0.00288556
8	GO_REGULATION_OF_UBIQUITIN_PROTEIN_TRANS	10/1204	54/17901	0.00288556
8	GO_REGULATION_OF_APOPTOTIC_SIGNALING_PA	43/1204	413/17901	0.00290419
8	GO_GLUTAMATE_RECEPTOR_SIGNALING_PATHWA	15/1204	101/17901	0.0030172

8	GO_ATF6_MEDIATED_UNFOLDED_PROTEIN_RESP(	4/1204	10/17901	0.00307894
8	GO_EPITHELIAL_CELL_PROLIFERATION_INVOLVED_	4/1204	10/17901	0.00307894
8	GO_HINDBRAIN_RADIAL_GLIA_GUIDED_CELL_MIG	4/1204	10/17901	0.00307894
8	GO_MEMBRANOUS_SEPTUM_MORPHOGENESIS	4/1204	10/17901	0.00307894
8	GO_REGULATION_OF_DEOXYRIBONUCLEASE_ACTI	4/1204	10/17901	0.00307894
8	GO_REGULATION_OF_FILOPODIUM_ASSEMBLY	9/1204	46/17901	0.0031333
8	GO_REGULATION_OF_MICROTUBULE_BASED_PRO	28/1204	240/17901	0.0031442
8	GO_DEOXYRIBONUCLEOTIDE_BIOSYNTHETIC_PRO(	5/1204	16/17901	0.00318866
8	GO_DNA_UNWINDING_INVOLVED_IN_DNA_REPLI	5/1204	16/17901	0.00318866
8	GO_HISTONE_H3_K36_METHYLATION	5/1204	16/17901	0.00318866
8	GO_MITOTIC_CHROMOSOME_CONDENSATION	5/1204	16/17901	0.00318866
8	GO_NEGATIVE_REGULATION_OF_UBIQUITIN_PRO	5/1204	16/17901	0.00318866
8	GO_REGULATION_OF_RNA_EXPORT_FROM_NUCL	5/1204	16/17901	0.00318866
8	GO_CARTILAGE_DEVELOPMENT	24/1204	196/17901	0.00322617
8	GO_HISTONE_H3_K9_METHYLATION	8/1204	38/17901	0.00326307
8	GO_REGULATION_OF_SPINDLE_ORGANIZATION	8/1204	38/17901	0.00326307
8	GO_REGULATION_OF_PROTEIN_MODIFICATION_B	28/1204	241/17901	0.00333586
8	GO_MAINTENANCE_OF_PROTEIN_LOCATION_IN_(	11/1204	64/17901	0.00334871
8	GO_NEGATIVE_REGULATION_OF_ESTABLISHMENT	22/1204	175/17901	0.00339555
8	GO_NEGATIVE_REGULATION_OF_CELLULAR_SENE	6/1204	23/17901	0.00341894
8	GO_NEGATIVE_REGULATION_OF_PROTEIN_ACETY	6/1204	23/17901	0.00341894
8	GO_POSITIVE_REGULATION_OF_OLIGODENDROCY	6/1204	23/17901	0.00341894
8	GO_REGULATION_OF_HISTONE_H3_K9_METHYLAT	6/1204	23/17901	0.00341894
8	GO_MAINTENANCE_OF_PROTEIN_LOCATION	14/1204	93/17901	0.0036073
8	GO_MEIOTIC_CHROMOSOME_SEGREGATION	14/1204	93/17901	0.0036073
8	GO_CHROMOSOME_CONDENSATION	9/1204	47/17901	0.00364868
8	GO_MITOTIC_METAPHASE_PLATE_CONGRESSION	9/1204	47/17901	0.00364868
8	GO_INTRINSIC_APOPTOTIC_SIGNALING_PATHWAY	15/1204	103/17901	0.00365351
8	GO_MRNA_CIS_SPLICING_VIA_SPLICEOSOME	10/1204	56/17901	0.00380167
8	GO_SOMATIC_RECOMBINATION_OF_IMMUNOGL(	10/1204	56/17901	0.00380167
8	GO_REGULATION_OF_INTRINSIC_APOPTOTIC_SIGI	21/1204	166/17901	0.00385057
8	GO_RAC_PROTEIN_SIGNAL_TRANSDUCTION	8/1204	39/17901	0.00386761
8	GO_INTRINSIC_APOPTOTIC_SIGNALING_PATHWAY	32/1204	290/17901	0.0039967
8	GO_REGULATION_OF_TELOMERASE_ACTIVITY	9/1204	48/17901	0.00422821
8	GO_POSITIVE_REGULATION_BY_HOST_OF_VIRAL_	5/1204	17/17901	0.0042699
8	GO_REGULATION_OF_NUCLEAR_CELL_CYCLE_DNA	5/1204	17/17901	0.0042699
8	GO_REGULATION_OF_RAC_PROTEIN_SIGNAL_TRA	5/1204	17/17901	0.0042699
8	GO_REGULATION_OF_DOUBLE_STRAND_BREAK_R	13/1204	85/17901	0.00430203
8	GO_REGULATION_OF_DNA_TEMPLATED_TRANSCF	16/1204	115/17901	0.00433993
9	GO_CELL_GROWTH	52/880	490/17901	1.48E-07
9	GO_GLIAL_CELL_DIFFERENTIATION	31/880	230/17901	3.37E-07
9	GO_PROTEIN_FOLDING	31/880	230/17901	3.37E-07
9	GO_CELLULAR_PROTEIN_COMPLEX_DISASSEMBLY	30/880	224/17901	5.96E-07
9	GO_DEVELOPMENTAL_GROWTH_INVOLVED_IN_M	31/880	236/17901	5.97E-07
9	GO_REGULATION_OF_CELL_MORPHOGENESIS	51/880	499/17901	6.18E-07
9	GO_FOREBRAIN_DEVELOPMENT	43/880	391/17901	6.85E-07
9	GO_POSITIVE_REGULATION_OF_CELL_PROJECTION	43/880	394/17901	8.42E-07

9	GO_RESPONSE_TO_TOPOLOGICALLY_INCORRECT_	28/880	204/17901	8.54E-07
9	GO_ATP_SYNTHESIS_COUPLED_ELECTRON_TRANS	18/880	98/17901	1.18E-06
9	GO_MITOCHONDRIAL_TRANSLATIONAL_TERMINA	17/880	89/17901	1.32E-06
9	GO_CELL_CYCLE_ARREST	30/880	234/17901	1.51E-06
9	GO_REGULATION_OF_CELL_MORPHOGENESIS_INV	36/880	310/17901	1.56E-06
9	GO_MITOCHONDRIAL_TRANSLATION	21/880	135/17901	2.66E-06
9	GO_RESPONSE_TO_ENDOPLASMIC_RETICULUM_S	34/880	293/17901	3.07E-06
9	GO_GLIOGENESIS	35/880	307/17901	3.30E-06
9	GO_TRANSLATIONAL_TERMINATION	18/880	105/17901	3.33E-06
9	GO_ATP_METABOLIC_PROCESS	35/880	311/17901	4.42E-06
9	GO_POSITIVE_REGULATION_OF_NEURON_DIFFERE	40/880	380/17901	4.94E-06
9	GO_POSITIVE_REGULATION_OF_NEURON_PROJEC	33/880	290/17901	6.51E-06
9	GO_PROTEIN_CONTAINING_COMPLEX_DISASSEME	36/880	330/17901	6.59E-06
9	GO_NEGATIVE_REGULATION_OF_NERVOUS_SYSTE	35/880	319/17901	7.77E-06
9	GO_REGULATION_OF_CELL_SIZE	24/880	181/17901	9.30E-06
9	GO_OXIDATIVE_PHOSPHORYLATION	21/880	149/17901	1.30E-05
9	GO_RESPIRATORY_ELECTRON_TRANSPORT_CHAIN	18/880	116/17901	1.40E-05
9	GO_NEGATIVE_REGULATION_OF_NEURON_DIFFER	27/880	225/17901	1.71E-05
9	GO_REGULATION_OF_WNT_SIGNALING_PATHWA	38/880	375/17901	2.02E-05
9	GO_KIDNEY_EPITHELIUM_DEVELOPMENT	20/880	142/17901	2.09E-05
9	GO_NEURAL_PRECURSOR_CELL_PROLIFERATION	21/880	154/17901	2.16E-05
9	GO_POSITIVE_REGULATION_OF_VIRAL_TRANSCRI	10/880	41/17901	2.18E-05
9	GO_CELLULAR_RESPONSE_TO_TOPOLOGICALLY_IN	22/880	166/17901	2.18E-05
9	GO_REGULATION_OF_EXTENT_OF_CELL_GROWTH	17/880	111/17901	2.87E-05
9	GO_AXON_EXTENSION	18/880	123/17901	3.17E-05
9	GO_TRANSLATIONAL_ELONGATION	19/880	135/17901	3.37E-05
9	GO_RESPONSE_TO_COPPER_ION	10/880	43/17901	3.42E-05
9	GO_NEGATIVE_REGULATION_OF_CELL_DEVELOPM	35/880	342/17901	3.45E-05
9	GO_REGULATION_OF_NEURAL_PRECURSOR_CELL_	15/880	91/17901	3.48E-05
9	GO_NEURON_PROJECTION_EXTENSION	22/880	172/17901	3.81E-05
9	GO_ENDOPLASMIC_RETICULUM_UNFOLDED_PRO	18/880	125/17901	3.95E-05
9	GO_CELL_SUBSTRATE_ADHESION	36/880	359/17901	4.12E-05
9	GO_DEVELOPMENTAL_CELL_GROWTH	27/880	237/17901	4.31E-05
9	GO_REGULATION_OF_AXONOGENESIS	23/880	186/17901	4.39E-05
9	GO_CELL_JUNCTION_ASSEMBLY	41/880	434/17901	4.93E-05
9	GO_PROTEIN_MATURATION	31/880	293/17901	5.10E-05
9	GO_POSITIVE_REGULATION_OF_ARP2_3_COMPLE	5/880	10/17901	5.82E-05
9	GO_PROTEIN_FOLDING_IN_ENDOPLASMIC_RETICU	5/880	10/17901	5.82E-05
9	GO_RENAL_TUBULE_DEVELOPMENT	15/880	95/17901	5.83E-05
9	GO_MITOCHONDRIAL_GENE_EXPRESSION	21/880	165/17901	6.10E-05
9	GO_MITOCHONDRIAL_ELECTRON_TRANSPORT_NA	11/880	55/17901	6.24E-05
9	GO_REGULATION_OF_POSTSYNAPSE_ORGANIZATI	16/880	107/17901	6.59E-05
9	GO_CELL_MATRIX_ADHESION	26/880	230/17901	6.77E-05
9	GO_NEURON_APOPTOTIC_PROCESS	27/880	244/17901	7.15E-05
9	GO_MYOBLAST_DIFFERENTIATION	14/880	86/17901	7.25E-05
9	GO_NEURON_PROJECTION_GUIDANCE	30/880	285/17901	7.36E-05
9	GO_POSITIVE_REGULATION_OF_OLIGODENDROCY	7/880	23/17901	8.30E-05

9	GO_POSITIVE_REGULATION_OF_CELL_MORPHOGE	20/880	157/17901	8.94E-05
9	GO_NEPHRON_EPITHELIUM_DEVELOPMENT	16/880	110/17901	9.25E-05
9	GO_GLIAL_CELL_DEVELOPMENT	17/880	122/17901	9.76E-05
9	GO_ELECTRON_TRANSPORT_CHAIN	22/880	184/17901	0.00010573
9	GO_ENDOPLASMIC_RETICULUM_CALCIUM_ION_H	7/880	24/17901	0.00011227
9	GO_CELL_REDOX_HOMEOSTASIS	11/880	59/17901	0.00012197
9	GO_MUSCLE_TISSUE_DEVELOPMENT	38/880	409/17901	0.00013025
9	GO_MITOCHONDRIAL_RESPIRATORY_CHAIN_COM	15/880	102/17901	0.00013389
9	GO_REGULATION_OF_SYNAPSE_STRUCTURE_OR_	26/880	240/17901	0.00013683
9	GO_CORTICAL_CYTOSKELETON_ORGANIZATION	11/880	60/17901	0.0001428
9	GO_PROTEIN_TRANSMEMBRANE_TRANSPORT	11/880	60/17901	0.0001428
9	GO_REGULATION_OF_DEVELOPMENTAL_GROWTH	34/880	353/17901	0.00014673
9	GO_OLIGODENDROCYTE_DIFFERENTIATION	15/880	103/17901	0.00014973
9	GO_CANONICAL_WNT_SIGNALING_PATHWAY	33/880	339/17901	0.00014977
9	GO_NEURON_PROJECTION_ORGANIZATION	14/880	92/17901	0.00015324
9	GO_REGULATION_OF_CELLULAR_COMPONENT_SI	36/880	383/17901	0.00015439
9	GO_COMMISSURAL_NEURON_AXON_GUIDANCE	5/880	12/17901	0.00016829
9	GO_ER_OVERLOAD_RESPONSE	5/880	12/17901	0.00016829
9	GO_REGULATION_OF_CELL_SUBSTRATE_ADHESIO	24/880	217/17901	0.00017805
9	GO_POSITIVE_REGULATION_OF_AXON_EXTENSION	9/880	43/17901	0.00020053
9	GO_UROGENITAL_SYSTEM_DEVELOPMENT	32/880	330/17901	0.00020118
9	GO_SYNAPSE_ORGANIZATION	39/880	433/17901	0.00020365
9	GO_APPENDAGE_DEVELOPMENT	21/880	181/17901	0.00022942
9	GO_NEPHRON_DEVELOPMENT	18/880	143/17901	0.00023002
9	GO_HIPPO_SIGNALING	9/880	44/17901	0.00024123
9	GO_MESENCHYMAL_CELL_PROLIFERATION	9/880	44/17901	0.00024123
9	GO_DIENCEPHALON_DEVELOPMENT	12/880	74/17901	0.00024312
9	GO_POSTTRANSLATIONAL_PROTEIN_TARGETING_	5/880	13/17901	0.00026242
9	GO_POSITIVE_REGULATION_OF_AXONOGENESIS	13/880	86/17901	0.00027927
9	GO_REGULATION_OF_TRANS_SYNAPTIC_SIGNALIN	40/880	455/17901	0.00028025
9	GO_OSSIFICATION	37/880	410/17901	0.00028276
9	GO_NADH_DEHYDROGENASE_COMPLEX_ASSEMBL	11/880	65/17901	0.00029818
9	GO_REGULATION_OF_VIRAL_TRANSCRIPTION	11/880	65/17901	0.00029818
9	GO_CELLULAR_RESPONSE_TO_COPPER_ION	7/880	28/17901	0.00032334
9	GO_POSTSYNAPSE_ORGANIZATION	20/880	173/17901	0.00033583
9	GO_REGULATION_OF_CELL_MATRIX_ADHESION	16/880	125/17901	0.00041578
9	GO_APPENDAGE_MORPHOGENESIS	18/880	150/17901	0.00041618
9	GO_TRANSITION_METAL_ION_HOMEOSTASIS	17/880	138/17901	0.00043849
9	GO_REGULATION_OF_ACTIN_FILAMENT_BASED_P	36/880	405/17901	0.00045184
9	GO_TELENCEPHALON_DEVELOPMENT	26/880	259/17901	0.00045562
9	GO_MUSCLE_ORGAN_DEVELOPMENT	36/880	407/17901	0.00049524
9	GO_RNA_SPLICING	41/880	484/17901	0.00050551
9	GO_AMYLOID_BETA_FORMATION	8/880	39/17901	0.00052191
9	GO_RAC_PROTEIN_SIGNAL_TRANSDUCTION	8/880	39/17901	0.00052191
9	GO_REGULATION_OF_SKELETAL_MUSCLE_TISSUE_	9/880	49/17901	0.00056104
9	GO_MITOCHONDRIAL_ELECTRON_TRANSPORT_UE	5/880	15/17901	0.00056392
9	GO_POSITIVE_REGULATION_OF_OSSIFICATION	13/880	93/17901	0.00060716

9	GO_SKELETAL_MUSCLE_ORGAN_DEVELOPMENT	19/880	168/17901	0.00061558
9	GO_BRANCHING_MORPHOGENESIS_OF_AN_EPITH	18/880	155/17901	0.00061867
9	GO_POSITIVE_REGULATION_OF_CELLULAR_PROTE	31/880	337/17901	0.00062172
9	GO_REGULATION_OF_OLIGODENDROCYTE_DIFFER	8/880	40/17901	0.00062464
9	GO_RELEASE_OF_CYTOCHROME_C_FROM_MITOC	10/880	60/17901	0.00063055
9	GO_AMYLOID_PRECURSOR_PROTEIN_METABOLIC_	11/880	71/17901	0.00065254
9	GO_AMYLOID_PRECURSOR_PROTEIN_CATABOLIC_	9/880	50/17901	0.0006548
9	GO_SPINDLE_LOCALIZATION	9/880	50/17901	0.0006548
9	GO_HEART_VALVE_DEVELOPMENT	10/880	61/17901	0.00072152
9	GO_REGULATION_OF_CYSTEINE_TYPE_ENDOPEPT	24/880	239/17901	0.00073831
9	GO_RESPONSE_TO_PEPTIDE_HORMONE	38/880	447/17901	0.0007477
9	GO_DNA_DAMAGE_RESPONSE_SIGNAL_TRANSDU	14/880	107/17901	0.00074908
9	GO_POSITIVE_REGULATION_OF_VIRAL_PROCESS	14/880	107/17901	0.00074908
9	GO_AMYLOID_BETA_METABOLIC_PROCESS	9/880	51/17901	0.00076097
9	GO_ER_NUCLEUS_SIGNALING_PATHWAY	9/880	51/17901	0.00076097
9	GO_NEGATIVE_REGULATION_OF_KINASE_ACTIVITY	26/880	268/17901	0.00076242
9	GO_LEARNING	17/880	145/17901	0.00077735
9	GO_POSITIVE_REGULATION_OF_ACTIN_NUCLEATI	5/880	16/17901	0.00078723
9	GO_NEUROBLAST_PROLIFERATION	10/880	62/17901	0.00082303
9	GO_LUNG_MORPHOGENESIS	9/880	52/17901	0.00088075
9	GO_PALLIUM_DEVELOPMENT	19/880	173/17901	0.00088167
9	GO_LIMBIC_SYSTEM_DEVELOPMENT	14/880	109/17901	0.00090204
9	GO_POSITIVE_REGULATION_OF_NEURON_DEATH	13/880	97/17901	0.00091045
9	GO_CELLULAR_RESPIRATION	20/880	187/17901	0.00091291
9	GO_REGULATION_OF_MESENCHYMAL_CELL_PROL	7/880	33/17901	0.00094148
9	GO_RNA_SPLICING_VIA_TRANSESTERIFICATION_R	34/880	391/17901	0.00094344
9	GO_ATF6_MEDIATED_UNFOLDED_PROTEIN_RESP	4/880	10/17901	0.0009598
9	GO_HINDBRAIN_RADIAL_GLIA_GUIDED_CELL_MIG	4/880	10/17901	0.0009598
9	GO_RESPONSE_TO_STEROID_HORMONE	31/880	346/17901	0.00096059
9	GO_POSITIVE_REGULATION_OF_STEM_CELL_PROL	8/880	43/17901	0.00103399
9	GO_NEGATIVE_REGULATION_OF_NEURON_APOPT	17/880	149/17901	0.00105679
9	GO_NEGATIVE_REGULATION_OF_SYNAPSE_ORGA	6/880	25/17901	0.00109864
9	GO_NEGATIVE_REGULATION_OF_CYCLIN_DEPEND	7/880	34/17901	0.00113592
9	GO_POSITIVE_REGULATION_OF_NEURAL_PRECUR	9/880	54/17901	0.00116624
9	GO_REGULATION_OF_PROTEIN_DEPOLYMERIZATI	12/880	88/17901	0.00121389
9	GO_CAMERA_TYPE_EYE_MORPHOGENESIS	15/880	125/17901	0.00122182
9	GO_CELLULAR_RESPONSE_TO_HEAT	15/880	125/17901	0.00122182
9	GO_RESPONSE_TO_DRUG	34/880	397/17901	0.00122386
9	GO_CENTRAL_NERVOUS_SYSTEM_NEURON_DIFFE	20/880	192/17901	0.00126503
9	GO_ENERGY_DERIVATION_BY_OXIDATION_OF_OR	26/880	278/17901	0.00130142
9	GO_DNA_TEMPLATED_TRANSCRIPTION_INITIATIO	24/880	249/17901	0.00130579
9	GO_ENSHEATHMENT_OF_NEURONS	16/880	139/17901	0.00133363
9	GO_MESONEPHRIC_TUBULE_MORPHOGENESIS	10/880	66/17901	0.00135332
9	GO_ESTABLISHMENT_OF_MITOTIC_SPINDLE_LOCA	7/880	35/17901	0.00136049
9	GO_ESTABLISHMENT_OF_PROTEIN_LOCALIZATION	30/880	339/17901	0.00139693
9	GO_LIMB_BUD_FORMATION	4/880	11/17901	0.00144979
9	GO_CELL_SUBSTRATE_JUNCTION_ORGANIZATION	13/880	102/17901	0.00145893

9	GO_IRE1_MEDIATED_UNFOLDED_PROTEIN_RESPO	10/880	67/17901	0.00152206
9	GO_MESONEPHROS_DEVELOPMENT	13/880	103/17901	0.00159629
9	GO_TISSUE_REGENERATION	11/880	79/17901	0.00161303
9	GO_ESTABLISHMENT_OF_SPINDLE_ORIENTATION	7/880	36/17901	0.00161828
9	GO_REGULATION_OF_OSSIFICATION	21/880	210/17901	0.0016209
9	GO_REGULATION_OF_RESPONSE_TO_ENDOPLASM	12/880	91/17901	0.00163068
9	GO_NUCLEOSIDE_DIPHOSPHATE_METABOLIC_PRO	17/880	155/17901	0.00163349
9	GO_CELLULAR_TRANSITION_METAL_ION_HOMEO	14/880	116/17901	0.00166009
9	GO_PLATELET_DEGRANULATION	15/880	129/17901	0.00168158
9	GO_CELL_DIFFERENTIATION_INVOLVED_IN_KIDNE	9/880	57/17901	0.00173023
9	GO_NEURON_DEATH	31/880	359/17901	0.00173082
9	GO_REGULATION_OF_PHOSPHOPROTEIN_PHOSPH	14/880	117/17901	0.00180255
10	GO_ENSHEATHMENT_OF_NEURONS	14/167	139/17901	4.67E-11
10	GO_CELL_JUNCTION_ASSEMBLY	20/167	434/17901	4.31E-09
10	GO_PROTEIN_LOCALIZATION_TO_AXON	5/167	10/17901	1.61E-08
10	GO_PERIPHERAL_NERVOUS_SYSTEM_DEVELOPME	7/167	77/17901	7.53E-06
10	GO_POSTSYNAPSE_ORGANIZATION	9/167	173/17901	3.62E-05
10	GO_MYELIN_ASSEMBLY	4/167	21/17901	3.86E-05
10	GO_CELL_SUBSTRATE_JUNCTION_ORGANIZATION	7/167	102/17901	4.76E-05
10	GO_SYNAPSE_ORGANIZATION	14/167	433/17901	5.66E-05
10	GO_PROTEIN_LOCALIZATION_TO_PLASMA_MEMB	11/167	281/17901	6.80E-05
10	GO_PROTEIN_LOCALIZATION_TO_CELL_PERIPHERY	12/167	340/17901	8.57E-05
10	GO_CELLULAR_COMPONENT_ASSEMBLY_INVOLVE	7/167	112/17901	8.64E-05
10	GO_PRESYNAPTIC_MEMBRANE_ORGANIZATION	3/167	11/17901	0.00012454
10	GO_FOCAL_ADHESION_ASSEMBLY	6/167	83/17901	0.00012564
10	GO_GLIAL_CELL_DEVELOPMENT	7/167	122/17901	0.00014777
10	GO_REGULATION_OF_CELL_MORPHOGENESIS_INV	11/167	310/17901	0.00016187
10	GO_ALTERNATIVE_MRNA_SPLICING_VIA_SPLICEOS	6/167	87/17901	0.00016301
10	GO_SCHWANN_CELL_DEVELOPMENT	4/167	31/17901	0.00018887
10	GO_REGULATION_OF_CELL_MORPHOGENESIS	14/167	499/17901	0.0002498
10	GO_REGULATION_OF_ACTIN_FILAMENT_ORGANIZ	10/167	278/17901	0.00028878
10	GO_POSITIVE_REGULATION_OF_CELL_PROJECTION	12/167	394/17901	0.00033497
10	GO_SCHWANN_CELL_DIFFERENTIATION	4/167	39/17901	0.00046588

p.adjust	qvalue	NCBI Entrez Gene Number	Count
2.82E-11	2.16E-11	6648/302/6888/5908/3936/9446/4478/3	16
7.18E-11	5.51E-11	822/5341/10095/9168/10109/10094/10	39
5.25E-10	4.03E-10	302/3958/7305/6688/25946/29887/220	40
5.55E-10	4.25E-10	10095/10109/10094/10092/2207/10096	23
6.88E-08	5.27E-08	5341/1192/10627/83706/7094/3315/59	14
7.92E-08	6.07E-08	3956/5341/1192/10627/83706/7094/37	16
4.78E-07	3.66E-07	5341/1192/10627/2207/83706/7094/33	20
1.44E-06	1.10E-06	10095/10109/10094/10092/2207/10096	24
6.15E-38	5.54E-38	1346/514/513/10975/4697/1340/54205	55
2.67E-34	2.41E-34	1346/514/513/10975/4697/2597/1340/5	71
2.08E-33	1.87E-33	10975/4697/1340/54205/4717/27089/4	43
2.74E-32	2.46E-32	10975/4697/1340/54205/4717/27089/4	45
2.23E-29	2.01E-29	513/10975/4697/1340/54205/4717/270	52
5.57E-81	4.94E-81	6193/6209/6223/6142/6210/6189/6147	72
4.80E-77	4.26E-77	6193/6209/6223/6142/6210/6189/6147	73
7.85E-77	6.97E-77	6193/6209/6223/6142/6210/6189/6147	73
3.41E-72	3.02E-72	6193/6209/6223/6142/6210/6189/6147	112
2.44E-69	2.16E-69	6193/6209/6223/6142/6210/6189/6147	84
1.98E-18	1.44E-18	1509/3123/1520/972/567/3106/3119/15	42
3.76E-14	2.73E-14	10457/3123/972/567/1514/920/2213/31	53
1.13E-11	8.20E-12	10457/3123/972/920/2213/3113/975/36	42
1.31E-10	9.50E-11	3123/972/714/920/975/126014/54209/2	30
1.31E-10	9.50E-11	2213/718/54209/1535/5788/140885/40	18
1.31E-10	9.50E-11	10457/3123/972/920/2213/3113/975/36	44
1.60E-10	1.17E-10	3123/972/3106/714/920/28959/2213/54	35
1.93E-10	1.40E-10	972/920/3113/975/3689/5724/5788/140	43
2.57E-10	1.87E-10	10457/3123/972/920/2213/3113/975/54	36
5.10E-10	3.70E-10	972/5641/975/54209/928/5724/728/719	29
1.20E-45	1.05E-45	6136/6194/6141/6130/6229/6138/6202	43
6.01E-43	5.24E-43	6136/6194/6141/6130/6229/6138/6202	43
9.97E-42	8.70E-42	6136/6194/6141/6130/6229/6138/6202	42
2.63E-40	2.29E-40	10209/6136/6194/6141/6130/6229/613	48
2.63E-40	2.29E-40	6136/6194/6141/6130/6229/6138/6202	44
5.27E-36	4.60E-36	4118/10971/6136/6711/6194/6141/613	55
2.85E-32	2.49E-32	6136/6194/6141/6130/6229/6138/6202	42
0.03482548	0.03038537	858/5654/1028/23089/857/28996/3326	8
0.03521805	0.03072789	4118/5962/60	3
0.04100491	0.03577695	1780/9363/1783/1211/375/114876/571	11
0.04483586	0.03911947	9448/5962/4804/1902/11135/55914/38	14
6.44E-06	5.18E-06	4688/6622/2745/1528/4715/2230/1032	13
7.70E-05	6.19E-05	3113/301/3606/199/29108/5880/7940/3	13
0.00014996	0.00012057	6280/301/409/7941/199/5880/7423/391	14
0.0002712	0.00021805	3113/301/3606/199/29108/5880/3115/8	11
0.00041065	0.00033017	6280/3113/301/3606/199/29108/5880/3	14
0.00041065	0.00033017	3113/301/3606/199/29108/5880/7940/3	13

0.00041065	0.00033017	3113/301/3606/199/29108/3115/10673	8
0.00050962	0.00040974	6280/301/7941/199/5880/7423/6347/22	11
0.00050962	0.00040974	3113/4688/8673/3117/29108/3108/109	11
0.00050962	0.00040974	3113/301/3606/199/29108/5880/3115/6	13
1.32E-18	1.14E-18	4857/2521/6651/10147/51747/3181/64	85
2.92E-15	2.52E-15	4857/2521/6651/3181/9584/10521/643	42
2.54E-13	2.19E-13	4857/6651/3181/9584/8106/10521/643	39
5.41E-13	4.67E-13	4857/2521/6651/51747/3181/6421/958	65
3.71E-12	3.20E-12	4857/6651/3181/9584/10521/6431/298	32
8.66E-12	7.47E-12	4194/6421/55904/4850/5310/7919/319	63
5.17E-11	4.46E-11	4194/6421/55904/4850/3192/351/5536	52
1.07E-09	9.19E-10	26053/3181/94104/6421/55904/3192/4	37
8.28E-09	7.14E-09	5980/4678/4176/4774/79915/672/2537	45
2.30E-08	1.98E-08	26053/3181/94104/6421/55904/55209/	52
3.40E-07	2.93E-07	26053/94104/55904/55209/3192/64324	34
1.26E-06	1.08E-06	6421/3192/1663/26993/11044/546/231	33
1.70E-06	1.47E-06	4194/4850/7919/55367/80279/65123/6	35
9.44E-06	8.14E-06	4194/55904/1021/4850/5310/8481/351	56
9.44E-06	8.14E-06	55636/4678/23314/29994/3070/57492/	33

1.30E-05	1.12E-05	2521/3181/7919/2969/79915/672/53	31
1.35E-05	1.16E-05	6421/23353/3192/672/1663/26993/1	43
1.39E-05	1.20E-05	26053/10252/7204/89941/81848/648	42
1.86E-05	1.61E-05	4133/351/2146/57492/5764/577/163	35
1.91E-05	1.65E-05	8106/6431/7919/23318/351/9295/80	26
1.91E-05	1.65E-05	55636/1021/23353/166614/23236/21	36
1.91E-05	1.65E-05	26053/94104/55904/4297/672/54904	21
2.29E-05	1.98E-05	10443/3192/7976/6664/6604/6608/2	13
2.35E-05	2.03E-05	4133/351/5764/9139/1630/6092/666	41
2.54E-05	2.19E-05	4133/5764/577/10152/23543/7804/1	25
3.42E-05	2.95E-05	26053/94104/55904/64324/4297/672	16
3.64E-05	3.14E-05	26053/94104/55904/55209/64324/42	25
3.99E-05	3.44E-05	3181/6431/10772/27316/57060/5815	10
4.47E-05	3.86E-05	4194/4850/7919/55367/80279/672/5	26
4.75E-05	4.10E-05	2521/3181/7644/7919/3192/2969/79	44
5.09E-05	4.39E-05	3181/6421/8106/6431/65110/7514/7	35
5.20E-05	4.48E-05	2895/5764/9863/85461/10152/9378/	27
5.35E-05	4.61E-05	8621/23353/3192/23236/4603/7552/	52
5.93E-05	5.12E-05	7976/4684/6608/4781/7422/6259/57	7
5.96E-05	5.14E-05	5310/3192/10252/29899/114327/290	24
5.96E-05	5.14E-05	55209/351/2895/5764/577/2823/609	33
6.60E-05	5.69E-05	26053/94104/57604/55904/55209/58	44
6.60E-05	5.69E-05	6421/1663/11044/546/23126/8243/5	15
6.71E-05	5.78E-05	3181/6431/10772/27316/57060/6432	9
6.86E-05	5.91E-05	8621/55209/7919/3192/2146/905/28	13
6.86E-05	5.91E-05	4194/4850/55367/672/57060/1655/1	20

6.86E-05	5.91E-05	2895/5764/10152/9378/9456/7804/1	20
6.88E-05	5.93E-05	26053/94104/55904/4297/672/54904	12
7.76E-05	6.69E-05	4194/4850/55367/80279/57060/1869	20
7.76E-05	6.69E-05	4194/4850/55367/672/57060/1869/4	17
7.85E-05	6.77E-05	55904/3192/351/23236/5586/4212/6	26
9.32E-05	8.04E-05	7976/6604/6608/259266/7422/63925	9
9.72E-05	8.38E-05	4133/351/5764/9139/1630/6664/575	41
9.72E-05	8.38E-05	2521/6421/5980/7516/4176/79915/6	37
9.72E-05	8.38E-05	4194/4850/55367/672/6664/57060/1	16
0.00010811	9.32E-05	56127/1004/56122/222256/64403/10	12
0.00011277	9.72E-05	5980/4176/79915/253714/55345/166	24
0.00011626	0.00010024	5310/3192/29899/114327/55835/546	27
0.00014135	0.00012187	3181/8106/6431/7919/9295/26993/6	20
0.00016143	0.00013918	3181/8106/6431/7514/7919/64062/9	23
0.00016906	0.00014577	7503/10984/3192/58487/2146/57492	29
0.00017102	0.00014746	2521/2969/672/5396/1655/1663/847	19
0.00017472	0.00015065	4194/4850/55367/80279/79915/672/	34
0.00019318	0.00016657	4194/4850/55367/672/57060/1869/4	19
0.00021246	0.00018319	6421/1663/23137/55719/84250/2583	6
0.0002179	0.00018787	6421/3192/1663/4288/546/348654/7	19
0.00024347	0.00020992	7516/1153/351/65123/5764/9112/67	48
0.00024963	0.00021524	546/23126/8243/23137/23383/55719	9
0.00024963	0.00021524	5310/3192/29899/114327/152185/28	20
0.00028907	0.00024924	6421/3192/1663/23137/9212/55719/	10
0.00030435	0.00026242	4194/4850/55367/57060/1869/4848/	14
0.00033694	0.00029052	1021/23353/166614/23236/2146/232	25
0.00035222	0.00030369	4040/7804/6608/23209/3157/5727/5	8
0.00036035	0.0003107	351/85461/10152/9456/7804/170506	17
0.00036826	0.00031752	7976/5764/6604/6608/259266/7422/	10
0.00036826	0.00031752	51747/55692/6431/7919/10181/1077	16
0.00037168	0.00032047	26053/55904/58508/55870/4297/672	13
0.00037168	0.00032047	2521/2969/672/5396/1663/29072/84	21
0.00041489	0.00035772	4194/4850/55367/79915/672/57060/	21
0.00041493	0.00035776	6421/3192/1663/546/348654/7756/2	16
0.00045794	0.00039484	3679/4133/23499/4289/5764/577/16	51
0.00045794	0.00039484	253714/55345/1663/259282/675/546	10
0.00046684	0.00040251	2521/1663/29072/84787/7158/7468/	16
0.00046684	0.00040251	3192/23137/9212/55719/84250/2583	8
0.00046684	0.00040251	56127/667/55209/1004/23499/56122	46
0.00046684	0.00040251	10443/3192/7976/5764/6664/6604/6	15
0.00046684	0.00040251	3181/8106/6431/65110/7514/5310/7	40
0.00051241	0.00044181	3192/29899/259266/152185/440270/	19
0.00053382	0.00046027	7514/7516/672/675/55835/152185/3	21
0.00053382	0.00046027	4133/351/9139/1630/57556/7025/66	29
0.00055182	0.00047579	2521/1663/84787/23347/11284/5571	10
0.00055182	0.00047579	4133/23499/577/1630/55604/6092/5	36
0.00061713	0.0005321	253714/55345/1663/259282/675/542	11

0.00062859	0.00054198	2521/79915/7552/29072/84787/2297	18
0.00062859	0.00054198	546/23137/23383/55719/84250/2583	6
0.00066954	0.00057728	10443/3192/7976/5764/6664/374887	20
0.00073775	0.0006361	51747/55692/6431/10772/27316/228	12
0.00073775	0.0006361	2521/79915/7552/84787/22976/7158	11
0.00073775	0.0006361	57556/9289/22854/9181/26012/2853	11
0.00076348	0.00065829	56127/55209/56122/351/2895/9863/	25
0.00076417	0.00065888	4194/1021/4850/55367/2146/80279/	38
0.00078122	0.00067358	7976/5764/6604/6608/259266/85440	13
0.00079363	0.00068428	2521/2969/672/5396/1663/84787/23	14
0.00082288	0.0007095	26053/1268/23499/2146/5764/9139/	42
0.00098345	0.00084794	4194/1021/4850/55367/2146/79027/	20
0.00115405	0.00099504	4194/55904/4678/1021/4850/5310/4	35
0.00115405	0.00099504	2115/2817/351/7204/7976/1630/609	33
0.00117558	0.0010136	8481/351/23236/80279/79915/672/8	32
0.00148461	0.00128006	9678/5764/5396/6608/4781/93986/2	9
0.00148461	0.00128006	3181/8106/6431/7514/5310/7919/64	26
0.00148461	0.00128006	4040/7804/6608/23209/3157/5727/5	8
0.00148461	0.00128006	2891/2892/2893/2897/351/2895/289	8
0.00148461	0.00128006	4678/3070/22985/257218/79829/592	29
0.00148776	0.00128277	2146/84515/675/29072/546/23383/9	15
0.00151373	0.00130516	8481/84131/55835/10142/54806/347	16
0.00151428	0.00130564	3192/2895/672/23224/29899/9378/4	25
0.00158935	0.00137036	80205/3181/55636/4176/1663/84515	18
0.00158935	0.00137036	8621/55209/7919/3192/2146/79101/	18
0.00159749	0.00137738	3181/6421/8106/6431/7514/7919/31	33
0.00163882	0.00141302	4194/55904/1021/4850/5310/55367/	26
0.0017267	0.00148879	6421/3192/2146/5764/4297/9112/26	34
0.00172872	0.00149053	8481/54558/5991/23224/84131/5992	41
0.00172872	0.00149053	26053/9678/55209/284058/4297/672	23
0.00180746	0.00155842	55636/7516/351/5454/6608/546/557	21
0.00182707	0.00157533	7514/672/675/55835/152185/348654	13
0.00182707	0.00157533	8621/7919/905/10915/51755/9984/2	8
0.00187788	0.00161914	23353/23236/23224/4040/5454/7804	18
0.00195452	0.00168522	1153/5764/90313/675/170506/1643/	20
0.00195718	0.00168751	84441/9139/6092/22905/374887/395	17
0.00209685	0.00180793	4194/55904/1021/4850/5310/55367/	28
0.00220293	0.0018994	8621/10443/3192/51755/182/4851/6	7
0.00220293	0.0018994	4133/9139/1630/57556/7025/22902/	24
0.0022083	0.00190403	23318/51593/2146/5764/5396/6657/	22
0.00230992	0.00199165	23353/23224/80336/85440/5108/574	8
0.00247122	0.00213072	4133/9139/1630/57556/7025/22902/	21
0.00283396	0.00244348	55904/55209/4297/54904/8473/2907	11
0.00296166	0.00255359	6421/1663/546/23137/55719/84250/	7
0.00296166	0.00255359	26053/55904/4297/672/9757/22976/	8
0.00296407	0.00255566	26053/9678/55209/284058/4297/672	25
0.00302789	0.00261069	120114/56127/5101/1004/5310/5612	22

0.00320239	0.00276115	26993/11044/23310/64151/10051/25	6
0.00320239	0.00276115	64062/26993/29072/11052/7175/571	6
0.00320532	0.00276367	667/29911/55835/5108/85378/63979	12
0.0032207	0.00277694	3181/23318/51593/1655/54487/1926	11
0.00331457	0.00285787	8481/351/80279/79915/672/84131/6	26
0.00379925	0.00327577	51593/5396/6657/259266/5108/182/	7
0.00379925	0.00327577	23353/23224/85440/5108/5747/1778	7
0.00379925	0.00327577	636/1153/5562/11273/1778/6311/23	7
0.00429898	0.00370664	2521/29072/7158/23347/10721/6397	10
0.00501972	0.00432807	26053/9678/55209/284058/4297/672	28
0.00507823	0.00437852	10152/7804/170506/115703/10059/6	10
0.00507823	0.00437852	23318/351/80336/11044/11052/5158	10
0.00554274	0.00477904	8621/3192/23236/7552/4288/546/34	23
0.00558907	0.00481898	672/55835/152185/284403/2648/985	9
0.00593474	0.00511702	5310/10252/9863/6092/55366/3911/	19
0.00593474	0.00511702	55209/55870/4297/672/2122/546/26	10
0.00605783	0.00522315	120114/56127/5101/1004/5310/5612	30
0.00608181	0.00524383	5803/7976/5764/5454/6604/58155/6	20
0.00608181	0.00524383	23594/259266/27152/144455/9212/5	6
0.00608181	0.00524383	26053/55904/4297/672/22976/1786	6
0.00653754	0.00563676	2146/5764/577/1630/10152/7804/17	20
0.00657951	0.00567295	1153/351/5764/9112/90313/85461/4	33
0.00657951	0.00567295	57492/79101/80336/23451/29028/54	11
0.00677579	0.00584219	64770/636/2804/5108/9851/4926/23	8
0.00684261	0.0058998	4194/1021/4850/55367/2146/80279/	36
0.00693221	0.00597705	2146/2895/5764/4040/23543/58155/	20
0.00769309	0.0066331	6421/3192/2146/4297/9112/8473/55	25
0.00776361	0.0066939	2891/2892/2893/59283/2897/351/23	15
0.00776361	0.0066939	26053/284058/4297/672/8473/79829	12
0.00780911	0.00673313	9063/1655/6597/3275/9612/2033/51	7
0.00795121	0.00685565	7503/3192/672/23347/64783/56339	6
0.00795121	0.00685565	64062/26993/29072/11052/7175/571	6
0.00801542	0.00691101	4194/1021/4850/55367/2146/80279/	29
0.00837208	0.00721853	57492/10152/7804/170506/115703/1	15
0.00837208	0.00721853	1021/1004/84441/351/9139/6092/22	23
0.00842944	0.00726799	4678/3070/257218/79829/5928/5572	25
0.00852635	0.00735154	3192/23236/7552/55023/23137/2597	12
0.00862978	0.00744073	3181/51593/54487/56339/6774	5
0.00867027	0.00747563	10521/9063/1655/6597/3275/9612/2	9
0.00867027	0.00747563	351/5764/9863/1869/57498/23095/2	10
0.00903054	0.00778627	2146/2895/5764/4040/58155/65250/	16
0.00919992	0.00793231	1268/6925/23499/2146/5764/9139/9	37
0.00920367	0.00793554	55904/58508/4850/4297/27327/2311	13
0.0092346	0.00796221	10152/7804/170506/115703/10059/6	11
0.00931289	0.00802971	8621/3192/2146/51497/10915/51755	7
0.00931289	0.00802971	4040/7804/6608/23209/3157/5727/5	8
0.00974301	0.00840057	672/580/84787/286257/22976/7158/	15

0.00995706	0.00858512	9678/5764/5396/6608/4781/93986/2	9
0.00995706	0.00858512	3070/55729/1786/9869/1788/51535	6
0.01109254	0.00956416	1268/23499/2146/5764/9139/9863/6	30
0.01121156	0.00966678	55553/8621/10443/1021/3192/12454	28
0.01121156	0.00966678	7976/6604/6608/259266/9289/7422/	10
0.01133246	0.00977102	80055/7516/23314/8481/10252/5991	42
0.01134418	0.00978112	2146/84515/29072/23383/25836/157	7
0.01145325	0.00987517	577/10152/7804/170506/5789/11570	14
0.01175561	0.01013587	55729/1786/9869/7175/51535	5
0.0122103	0.0105279	5310/4133/10252/889/29899/85440/	18
0.0122103	0.0105279	6431/10252/5764/6092/55366/6608/	13
0.01221727	0.01053392	55553/8621/10443/1021/3192/5764/	16
0.01246708	0.0107493	6421/667/636/4133/23353/3192/848	22
0.01246708	0.0107493	7514/636/5310/4133/3192/672/2989	22
0.01246708	0.0107493	4848/8994/255967/11273/1778/6311	6
0.01246708	0.0107493	79829/55729/1786/9869/7175/51535	6
0.01276214	0.01100371	3192/152185/8243/7756/90990/9212	11
0.01298711	0.01119768	2146/672/5928/1869/23269/11335/1	9
0.01344235	0.01159019	116983/23236/2146/577/4301/9743/	38
0.01358439	0.01171267	9678/55636/6431/5310/10252/5764/	23
0.01496118	0.01289975	10984/2146/3070/57492/80336/7982	19
0.01499343	0.01292756	55209/55870/672/2122/546/1786/55	8
0.01499343	0.01292756	23236/7976/27327/23112/192669/26	8
0.01575274	0.01358224	3192/2146/51497/10915/6829	5
0.01581327	0.01363444	116983/23236/10252/2146/577/4301	43
0.01581327	0.01363444	3192/55023/23137/25970/9212/5571	10
0.01581327	0.01363444	84441/6092/22905/3955/182/4851/2	10
0.01581736	0.01363796	1004/222256/4301/253559/64403/10	11
0.01726867	0.0148893	4133/288/64062/7545/1869/6608/23	24
0.01746447	0.01505813	55904/58508/4850/4297/27327/2311	14
0.01936671	0.01669827	26137/351/8671/8473/5562/9883/10	13
0.0196263	0.01692209	1268/112476/23025/6804/10059/228	6
0.0196263	0.01692209	672/55835/152185/2648/85459/7840	6
0.01992854	0.01718269	7514/7327/288/7545/26993/1869/29	27
0.02063405	0.01779098	6421/667/636/4133/23353/3192/351	19
0.02064125	0.0177972	26137/351/8671/8473/5562/9883/20	16
0.02064125	0.0177972	3070/79829/55729/2122/50809/2334	12
0.02064125	0.0177972	2895/9378/5789/23613/4916	5
0.02079307	0.0179281	5803/7976/5764/6604/58155/6608/2	13
0.02152457	0.01855881	4194/55636/6092/6664/65250/6608/	15
0.02213697	0.01908683	6421/667/636/4133/23353/3192/351	22
0.0223899	0.01930491	55636/6092/56999/5311/11174/182/	10
0.02260532	0.01949065	6431/4781/93986/6662	4
0.02260532	0.01949065	64324/672/84787/7468	4
0.02260532	0.01949065	4848/255967/11273/6311	4
0.02483101	0.02140967	29896/1153/27316/27327/6625/2311	12
0.02489773	0.0214672	3181/170506/546/5514/11284/9212/	9

0.02489773	0.0214672	26053/55209/4297/672/5562/26155/	10
0.026814	0.02311943	23353/23224/5454/114327/9289/233	8
0.02719043	0.023444	7919/79915/1191/5514/7158/1859/2	12
0.02810083	0.02422895	55636/1848/7919/4133/23499/351/2	33
0.02895309	0.02496379	5764/6092/5747/2534/22871/7337	6
0.02895309	0.02496379	5396/6608/93986/2260/7422/6662	6
0.02920838	0.0251839	5310/55366/3911/6608/1282/54806/	13
0.03005629	0.02591498	4194/80279/79915/672/1655/5928/5	20
0.03038248	0.02619623	55209/351/2895/577/6092/576/9378	14
0.03038248	0.02619623	1268/112476/59283/2897/351/8525/	40
0.03129461	0.02698268	2895/9378/5789/22871/8831/23613/	7
0.03220894	0.02777103	55729/1786/9869/51535	4
0.03257226	0.02808429	7514/4133/7327/288/64062/7545/26	33
0.03273095	0.02822111	7516/65123/9112/672/221927/675/5	17
0.03367048	0.02903119	1196/283871/140710/4485/2033	5
0.03442269	0.02967976	23499/5764/22854/22871/1139/934	6
0.03442269	0.02967976	79829/55729/1786/9869/7175/5153	6
0.03442269	0.02967976	7514/672/55835/152185/348654/264	8
0.0347621	0.0299724	26053/55209/4297/672/5562/26155/	11
0.0347621	0.0299724	6431/10252/2146/6608/10253/7422/	9
0.0347621	0.0299724	55553/5310/5764/1630/6092/6664/7	14
0.03593711	0.03098551	26053/4133/23499/351/10252/9863/	24
0.03635671	0.0313473	5586/5764/675/114327/23594/2907	19
0.03635671	0.0313473	4133/1630/57556/22902/5747/8003	13
0.03734218	0.03219698	5310/9863/55366/3911/6608/54806/	14
0.03748296	0.03231837	2521/1268/3192/1153/7327/80279/8	26
0.03779163	0.03258451	1282/1290/5311/53335/1786/26012/	11
0.03927413	0.03386274	51747/55692/6431/7919/10181/107	24
0.04043875	0.03486689	5803/55553/1021/23353/2817/351/2	29
0.04066788	0.03506445	4133/23499/1630/6092/57556/5454/	20
0.04066788	0.03506445	79915/84787/22976/7158/7468/107	10
0.04066788	0.03506445	283131/373863/29028/54454/9612/6	7
0.04066788	0.03506445	4850/23318/27327/23112/11044/48	7
0.04066788	0.03506445	7158/23347/10721/55183/2187	5
0.04066788	0.03506445	2115/2817/576/5454/23114/6334/20	11
0.04066788	0.03506445	79915/84787/22976/7158/7468/107	11
0.04118522	0.03551051	4194/2521/1268/3192/1153/7327/80	30
0.04118522	0.03551051	3181/3192/675/5426/84444/170506/	19
0.04118522	0.03551051	5310/5311/182/4851	4
0.04118522	0.03551051	29899/259266/10142/57584	4
0.04118522	0.03551051	9289/23380/6774/54328	4
0.04118522	0.03551051	546/144455/2260/58525	4
0.04118522	0.03551051	1268/112476/10059/22871	4
0.04118522	0.03551051	79915/675/546/144455/2260/5930/6	14
0.04118522	0.03551051	7514/64062/7545/26993/29072/580/	14
0.04118522	0.03551051	5764/9863/2898/9378/375790/2901/	9
0.04151561	0.03579538	55729/170506/22976/9282/9969/260	8

0.04151561	0.03579538	636/288/80279/5764/29899/7545/18	31
0.04241101	0.03656741	55553/55636/55692/10521/7919/319	36
0.04281329	0.03691426	2895/577/576/9378/57633/23284/37	10
0.0428531	0.03694858	10152/7804/170506/115703/10059/6	11
0.04397563	0.03791645	55636/5396/6664/4040/65250/546/3	17
0.04397563	0.03791645	55636/490/5764/2823/4040/6604/12	17
0.04437435	0.03826023	29896/1153/27316/6625/6434/1859/	7
0.04437435	0.03826023	79915/84787/22976/7158/7468/5518	7
0.04437435	0.03826023	55729/170506/22976/9282/9969/260	7
0.04437435	0.03826023	79915/84787/22976/7158/7468/5518	6
0.04437435	0.03826023	1268/112476/23025/6804/10059/228	6
0.04437435	0.03826023	170506/580/11052/79882/79869/682	6
0.04437435	0.03826023	7976/55504/55366/114803/3911/660	12
0.04437435	0.03826023	64770/636/3192/2804/5108/9851/49	9
0.04437435	0.03826023	6421/667/55605/636/4133/23353/31	33
0.04532326	0.03907839	22985/26993/11044/23310/64151/10	8
0.04532326	0.03907839	55636/5396/6092/56999/5311/11174	13
0.0471387	0.04064369	283131/1655/27327/373863/29028/5	16
0.04783994	0.04124831	490/672/4040/7804/6608/23209/556	12
0.04889062	0.04215422	5310/10252/6092/55366/3911/6608/	13
0.04955037	0.04272307	3181/3192/170506/546/5514/23049/	11
0.04984956	0.04298103	5310/3192/29899/84722/4926/1778/	7
3.63E-07	3.28E-07	4650/23129/6002/25780/8829/11698	54
1.01E-05	9.14E-06	4650/6002/25780/8829/116985/9905	45
2.40E-05	2.17E-05	10161/4650/8567/27128/8829/11698	38
2.40E-05	2.17E-05	10180/6651/55599/23524/3187/5851	49
3.83E-05	3.46E-05	6651/3187/58517/2521/1195/8227/1	24
7.28E-05	6.57E-05	9765/1130/4035/54832/55275/3482/	23
0.0001175	0.00010606	9765/1130/4035/55275/3482/5660/9	19
0.00017598	0.00015886	23129/8829/8828/55558/5361/10154	7
0.00134418	0.00121337	23312/10312/1657/535/6556/2896/1	8
0.00140899	0.00127187	8764/472/5788/8829/6239/342184/3	40
0.00159703	0.00144161	5027/1130/2548/2896/1201/79158/8	13
0.00159703	0.00144161	472/54737/3299/55870/58508/4297/	43
0.00178713	0.00161321	10180/6651/55599/23524/3187/5851	37
0.00178713	0.00161321	6651/58517/10181/1655/6432/10658	17
0.00178713	0.00161321	472/54832/535/23049/5660/3587/16	34
0.00178713	0.00161321	23312/154881/10312/1657/9497/535	17
0.0019173	0.00173071	6651/58517/10181/1655/6432/10658	20
0.0034426	0.00310757	949/4035/5788/718/6556/2268/6458	15
0.0034426	0.00310757	23129/23499/4035/2803/8829/27185	14
0.00539702	0.00487179	26088/54832/9043/1601/9905/55275	20
0.00539702	0.00487179	4035/5788/718/6556/64581/2889/10	12
0.00548407	0.00495037	8832/718/3566/84106/2268/6810/57	11
0.00571403	0.00515795	23499/4035/2803/8829/27185/393/8	25
0.00761655	0.00687532	23312/10312/1657/9497/535/9498/4	14
0.008494	0.00766738	23312/154881/10312/1657/9497/535	19

0.00849994	0.00767275	55704/55283/5027/3709/10142/722(	23
0.00849994	0.00767275	472/9295/6432/6430/23049/7919/67	22
0.00849994	0.00767275	89849/10312/5027/1130/81671/2548	20
0.00857015	0.00773612	23499/4035/5328/8829/6239/342184	23
0.009019	0.00814129	55704/55283/10062/949/5027/3709/	30
0.00945885	0.00853833	23499/4035/2803/8829/27185/393/8	16
0.01005331	0.00907494	23499/4035/5328/8829/6239/342184	32
0.01005331	0.00907494	23015/2804/440270/10142/81671/9(	18
0.01005331	0.00907494	8764/10062/472/8832/5788/3566/22	22
0.01018485	0.00919368	23499/4035/5328/8829/342184/2718	16
0.01109912	0.01001897	8829/342184/27185/7402/1435/2185	10
0.01109912	0.01001897	1601/6614/22848/55589/50618/2580	9
0.01306865	0.01179683	23312/10312/1657/9497/535/9498/4	14
0.01306865	0.01179683	1118/55577/51005/80896/1116	5
0.01306865	0.01179683	4035/5739/9146/5782/5771	5
0.01306865	0.01179683	54832/9648/23230/2580/4926/7956	7
0.01306865	0.01179683	26088/23499/2803/54832/8906/681(	14
0.01306865	0.01179683	23129/23499/4035/2803/8829/6239/	18
0.01306865	0.01179683	8832/3566/84106/2268/6810/57102/	9
0.01306865	0.01179683	7127/472/9295/6432/6430/23049/84	27
0.01306865	0.01179683	23499/4035/5328/8829/342184/640	23
0.01353406	0.01221695	23312/10312/1657/535/6556/2896/1	10
0.01382055	0.01247556	81501/5788/4323/3566/22985/861/8	18
0.01413365	0.01275818	10312/5027/535/57192/200576/120	9
0.01535675	0.01386226	10443/81501/5788/85477/4323/356	21
0.01619567	0.01461953	472/9295/6432/6430/23049/7919/67	23
0.01767446	0.01595442	8829/6239/342184/3675/27185/231	15
0.01826847	0.01649061	9765/54832/9648/23230/79158/492	8
0.01826847	0.01649061	23129/8829/8828/55558/2316/5361/	8
0.01826847	0.01649061	8764/5027/8832/5788/718/3566/841	22
0.01999355	0.01804781	10062/949/718/84879/64900/23659/	9
0.02007072	0.01811747	9765/10062/4035/1601/718/22848/9	21
0.02122241	0.01915708	1130/79083/9839/23334/114803/464	13
0.02181732	0.0196941	1130/79083/9839/4644/200576/464	9
0.02181732	0.0196941	58517/10181/1655/6432/10658/643(	12
0.02186288	0.01973522	8832/9043/3566/9842/2268/23207/5	11
0.02186288	0.01973522	9295/6432/6430/23049/7919/57187/	14
0.02267256	0.02046611	3663/9208/81844/5966/84868/3654/	15
0.02498404	0.02255263	5339/23499/4035/8829/342184/667/	13
0.02498404	0.02255263	4035/868/5739/9146/5782/5771	6
0.02498404	0.02255263	10062/949/718/84879/64900/2180/2	8
0.02498404	0.02255263	23129/23499/4035/2803/23077/882	19
0.02507183	0.02263188	8837/55704/5027/23129/23499/403	32
0.02507183	0.02263188	6651/58517/2521/10181/1655/6432/	29
0.02507183	0.02263188	23499/4035/2803/8829/27185/393/8	18
0.0260069	0.02347595	9295/6432/6430/23049/7919/57187/	16
0.02621442	0.02366328	23499/4035/8829/342184/4323/798	10

0.02959508	0.02671494	23499/4035/2803/8829/342184/2718	22
0.02959508	0.02671494	23129/8829/1512/5361	4
0.02959508	0.02671494	55719/6421/25836/23137	4
0.02959508	0.02671494	84868/3683/3109/10859	4
0.02986795	0.02696125	54832/535/23049/4864/200576/1201	18
0.02986795	0.02696125	472/8569/1195/5788/22848/23049/1	22
0.03355645	0.03029079	1655/1601/10013/3275/9063/9667	6
0.03508247	0.0316683	10161/4650/8567/27128/8829/9267/	19
0.03508247	0.0316683	9905/58504/23102/2889/55357/2352	13
0.03508247	0.0316683	3687/8829/3675/667/84106/83706/2	13
0.03647754	0.03292761	1601/22848/55589/10188/1785	5
0.03709304	0.03348321	5027/8832/5788/718/3566/84106/84	20
0.03709304	0.03348321	5027/23499/4035/2803/8829/27185/	18
0.03709304	0.03348321	23499/4035/8829/342184/4323/7983	11
0.03709304	0.03348321	10181/23451/10658/6430/7536/5174	11
0.03782963	0.03414812	4035/8829/868/5739/2889/9146/578	9
0.03782963	0.03414812	81501/22985/677/1435	4
0.03782963	0.03414812	4035/718/3459/4481/3684/10347/60	7
0.03782963	0.03414812	123096/5027/9295/6432/6430/23049	23
0.03786745	0.03418226	55283/5027/3709/7226/80024/5788/	14
0.03909109	0.03528681	89849/10312/54832/535/81671/2304	26
0.04098375	0.03699528	8764/472/3566/2268/10457/861/848	16
0.04168071	0.03762442	10312/4035/5660/23230/1201	5
0.04168071	0.03762442	55283/5027/3709/7226/80024/5788/	15
0.04215355	0.03805124	10161/4650/8567/27128/25780/8829	28
0.04368306	0.0394319	23015/5027/1130/2804/440270/1014	35
0.04741892	0.04280419	26088/91304/5027/472/4035/54832/	34
0.04741892	0.04280419	3663/81844/5966/140885/7187/1085	8
0.04803482	0.04336015	8829/342184/7248/6093/4088/9647	6
0.04803482	0.04336015	10062/2/2896/84868/54625/10859/3	9
0.04803482	0.04336015	81501/1435/7097/5771	4
0.04803482	0.04336015	23499/81501/4035/2803/84162/8829	36
0.04803482	0.04336015	10312/472/1316/5788/4323/5660/35	29
0.04803482	0.04336015	10062/472/5788/3459/3566/6556/28	12
0.04803819	0.0433632	9295/6432/6430/23049/7919/57187/	19
8.27E-21	7.16E-21	4857/57060/11168/4841/5411/51319	88
4.23E-20	3.66E-20	4857/57060/11168/4841/5411/51319	98
5.38E-14	4.65E-14	59284/4857/57060/29896/580/6431/	70
1.79E-13	1.55E-13	4781/4176/4774/4784/4678/580/976	60
3.15E-13	2.73E-13	51497/60559/7175/100101267/6598/	49
4.21E-13	3.64E-13	60559/11014/9789/10945/55973/330	42
4.21E-13	3.64E-13	59284/57060/4848/4850/10535/1064	76
4.21E-13	3.64E-13	4176/6662/4678/6925/6879/8467/46	60
4.21E-13	3.64E-13	26053/6879/6421/55904/7175/6749/	69
3.91E-12	3.39E-12	4176/6662/4678/3149/55636/80205/	67
1.33E-11	1.15E-11	6746/29927/23204/11231/6745/6747	33
2.43E-11	2.10E-11	60559/9789/3309/29927/11231/2897	35

6.67E-11	5.77E-11	23353/7155/6421/84722/91272/7175	62
1.50E-10	1.30E-10	5087/1031/57060/23236/6659/351/4	78
1.96E-10	1.70E-10	6662/4678/3149/3066/55636/9031/8	47
2.30E-10	1.99E-10	7155/6421/84722/91272/7175/8243/	44
4.45E-10	3.85E-10	4857/57060/29896/6431/23543/1065	38
6.87E-10	5.94E-10	1031/4176/57060/23236/6659/4678/	57
9.80E-10	8.48E-10	4857/57060/29896/580/6431/23543/	37
9.80E-10	8.48E-10	26053/6421/55904/7175/6598/10574	41
2.32E-09	2.00E-09	4176/6929/4841/3149/64110/7155/5	54
3.25E-09	2.81E-09	29899/5087/3642/57060/23236/6659	65
3.61E-09	3.12E-09	23353/7155/6421/84722/91272/7175	51
4.84E-09	4.19E-09	84722/91272/7175/8243/7756/25836	37
6.66E-09	5.77E-09	84722/91272/7175/8621/8243/7756/	51
1.71E-08	1.48E-08	29899/5087/3642/57060/23236/6659	53
3.68E-08	3.18E-08	4857/57060/29896/6431/23543/1065	29
6.03E-08	5.22E-08	1031/57060/6659/4194/3276/1021/4	58
7.63E-08	6.60E-08	4848/4850/23318/22803/23112/5101	41
9.36E-08	8.10E-08	6662/4678/9031/8467/4676/7994/71	42
1.81E-07	1.56E-07	6662/4678/8467/4676/7994/6598/66	37
1.87E-07	1.61E-07	6421/7175/7756/1457/25836/324/22	27
1.87E-07	1.61E-07	29899/2770/10252/84722/10253/717	32
1.87E-07	1.61E-07	84919/7175/27102/8668/1973/8666/	38
2.07E-07	1.79E-07	4857/51319/6431/23543/10656/6421	24
2.59E-07	2.24E-07	57060/580/6431/10772/1660/79171/	24
3.09E-07	2.67E-07	2770/10130/9601/2923/23193/1191/	42
3.56E-07	3.08E-07	6662/4678/8467/4676/7994/7175/51	43
4.74E-07	4.10E-07	6421/7175/7756/25836/324/2237/31	23
5.20E-07	4.50E-07	55110/6218/6129/4736/6175/3921/6	28
5.42E-07	4.70E-07	6431/8106/6421/10212/7175/100101	42
5.60E-07	4.84E-07	5396/10715/64110/5591/7644/10574	56
6.02E-07	5.21E-07	26053/6659/5352/3066/9678/6879/1	60
6.61E-07	5.72E-07	5087/23236/351/10763/5569/22822/	46
8.54E-07	7.39E-07	1031/57060/6659/7976/4194/2619/3	53
8.62E-07	7.46E-07	51575/11168/10528/55636/6431/115	67
8.95E-07	7.74E-07	57060/6659/4194/3066/580/3276/88	45
1.04E-06	8.97E-07	4176/64110/6421/142/23028/5531/4	30
1.36E-06	1.18E-06	5087/351/5569/23476/595/5885/572	39
1.56E-06	1.35E-06	4678/3066/8467/6598/6605/51773/6	23
1.93E-06	1.67E-06	54499/10130/9601/2923/55829/8491	47
1.93E-06	1.67E-06	11168/6431/5591/10772/1660/8668/	41
2.40E-06	2.08E-06	4176/10926/5425/5111/4171/7013/6	19
2.62E-06	2.27E-06	57060/6431/10772/79171/27316/700	12
3.12E-06	2.70E-06	1031/57060/6659/4194/3276/1021/4	44
3.15E-06	2.73E-06	4176/3149/55636/80205/142/1660/1	26
3.15E-06	2.73E-06	2534/9747/6746/3309/29927/23204/	37
3.27E-06	2.83E-06	6879/7175/6598/142/23028/7756/41	30
3.47E-06	3.00E-06	59284/57060/10643/1660/1869/1023	37

3.62E-06	3.13E-06	2534/9747/6746/3309/1869/2181/70	51
3.72E-06	3.22E-06	5396/5591/10574/142/1660/10155/5	36
4.42E-06	3.82E-06	23224/6091/4781/4760/80055/6657/	56
4.84E-06	4.19E-06	4176/84268/10926/11177/5425/511	30
5.83E-06	5.05E-06	26053/6879/55904/7175/6749/6598/	35
5.88E-06	5.09E-06	10130/55829/84919/1191/821/2137/	36
6.01E-06	5.21E-06	23236/4603/23353/7155/84722/912	63
6.98E-06	6.04E-06	4760/11168/5569/51319/580/6431/8	52
8.94E-06	7.74E-06	2823/26053/9289/2534/7976/6656/1	31
1.25E-05	1.08E-05	7155/7175/7756/1457/324/80010/58	22
1.47E-05	1.27E-05	29899/2770/84722/7175/8243/6494	25
1.64E-05	1.42E-05	26053/283248/3066/9678/3276/903	63
1.72E-05	1.49E-05	6664/57060/6659/4194/3276/4848/5	19
2.07E-05	1.79E-05	57060/580/6431/10772/79171/2731	12
2.48E-05	2.15E-05	26053/6659/3066/9678/6879/11177/	31
3.22E-05	2.78E-05	29899/2770/7430/84722/7175/8243/	32
3.22E-05	2.78E-05	57060/6431/10772/79171/27316/64	10
3.51E-05	3.04E-05	6431/8106/6421/10212/7175/10010	34
3.51E-05	3.04E-05	26053/6879/55904/6598/4297/2302	28
3.79E-05	3.28E-05	23224/6091/4781/4760/9289/23236/	40
4.94E-05	4.27E-05	1948/1627/59284/254263/6431/743	43
5.00E-05	4.33E-05	4176/580/64110/6421/5591/142/28	40
6.04E-05	5.23E-05	7175/7756/1457/324/5885/11065/10	17
7.92E-05	6.85E-05	6664/57060/6659/4194/3276/7155/4	35
8.89E-05	7.70E-05	4678/8467/51773/64946/11198/592	15
9.08E-05	7.86E-05	4760/3642/57060/6659/4194/3276/4	22
9.65E-05	8.36E-05	57060/6659/4194/3276/7155/4848/5	29
0.00011571	0.00010017	6598/6605/6599/8289/6597/6602/11	9
0.00011571	0.00010017	6598/6605/6599/8289/6597/6602/11	9
0.00013725	0.0001188	1031/57060/23236/6659/4194/3276/	33
0.00013725	0.0001188	4678/8467/51773/64946/5928/1063/	15
0.0001449	0.00012543	23236/7175/811/8621/7756/25836/3	31
0.00015189	0.00013148	2534/9747/6746/1191/60559/9789/3	56
0.00015189	0.00013148	6938/6662/182/5764/6929/55553/10	23
0.00015637	0.00013536	5396/5591/142/1660/10155/5111/29	17
0.00015637	0.00013536	6431/8106/10212/7175/100101267/1	26
0.00016004	0.00013854	57060/6659/4194/3276/4848/5591/4	16
0.00016004	0.00013854	4176/4171/51659/4175/4172/4174/4	7
0.00016004	0.00013854	10574/7013/10694/10576/7203/908/	7
0.00017954	0.00015542	200916/1660/8668/1973/51121/866	21
0.00019259	0.00016671	55829/84919/1191/3309/51060/109	14
0.00019498	0.00016878	5087/8324/5396/6662/182/6657/57	28
0.00022232	0.00019245	7175/7756/324/5885/1063/4085/26	13
0.00025889	0.0002241	64946/1063/201254/10051/9787/10	9
0.00027507	0.00023811	26053/55904/7175/6598/4297/2302	21
0.00029385	0.00025437	1031/57060/6659/4194/3276/1021/4	24
0.00029567	0.00025594	7175/7756/324/5885/11065/1063/84	15

0.0003645	0.00031552	26053/6659/3066/6879/6598/4297/2	15
0.00040032	0.00034653	4194/3066/580/8805/6879/7994/674	29
0.00047963	0.00041518	1627/3642/9289/5764/7976/5454/58	26
0.00047963	0.00041518	6662/4678/8467/4676/7994/51773/5	25
0.00053573	0.00046374	6421/91272/8243/25836/2237/5885/	15
0.00053822	0.00046589	10574/7013/10694/10576/7203/908/	7
0.00057648	0.00049901	26053/6659/3066/9678/6879/11177/	31
0.00057648	0.00049901	10574/10694/10576/7203/908/1736	6
0.00057648	0.00049901	2923/821/3309/811/7184/54431	6
0.0005957	0.00051565	5087/351/23476/595/1019/79915/26	10
0.0006177	0.0005347	580/6431/8106/10212/7175/1001012	31
0.0006177	0.0005347	2137/92126/1464/1462/79586/26229	15
0.0006438	0.00055729	1031/4760/3642/6657/57060/6659/4	34
0.00067053	0.00058042	1627/6091/6938/4760/6664/59284/2	48
0.00068738	0.00059501	5806/23236/2817/23193/6185/81618	51
0.00070806	0.00061291	4239/5764/10715/9768/56160/90313	24
0.00072578	0.00062825	7175/7756/324/5885/1063/4085/262	11
0.00072988	0.00063181	5087/23236/351/55904/7175/5586/2	27
0.00076099	0.00065873	1464/1462/79586/26229/63827/6413	10
0.00076099	0.00065873	6185/1650/3998/54344/79053/29880	10
0.00076201	0.00065961	3642/57060/6659/4194/3276/4848/4	17
0.00076201	0.00065961	5396/64110/5591/142/1660/23028/1	23
0.00081655	0.00070683	23236/6185/81618/1650/200185/213	44
0.00085041	0.00073613	57060/6659/4194/3276/4848/5591/4	23
0.00094891	0.0008214	10130/55829/84919/821/2137/3309/	22
0.00096668	0.00083679	6431/8106/6421/10212/7175/100101	36
0.00097321	0.00084244	26053/55904/6598/4297/23028/2347	15
0.00097321	0.00084244	55829/84919/1191/7873/3309/51060	18
0.00097321	0.00084244	55366/6664/6662/182/6657/4090/10	33
0.00107483	0.0009304	7175/7756/324/5885/1063/4085/111	14
0.00112776	0.00097622	23224/6091/4760/9289/23236/5454/	27
0.00120408	0.00104228	351/580/6431/8106/10212/10250/23	19
0.00125924	0.00109003	5087/23236/351/55904/23476/595/1	20
0.00126305	0.00109333	6421/25836/2237/3192/5885/23137/	10
0.00126427	0.00109438	92126/1464/1462/26229/63827/1067	7
0.00141668	0.00122632	10130/55829/84919/821/2137/3309/	26
0.0014296	0.00123749	4678/8467/51773/64946/5928/20125	12
0.00155545	0.00134644	5396/1191/64110/5591/142/1660/23	32
0.00167041	0.00144595	5111/7013/7009/3148/6917/3146/48	8
0.00167041	0.00144595	6421/25836/2237/5885/23137/10664	8
0.00168434	0.00145801	3485/4176/4760/2534/5764/7976/18	48
0.00172597	0.00149404	51497/6919/8621/23476/3192/11198	12
0.00172597	0.00149404	51497/6598/1660/51773/5438/6597/	11
0.00172905	0.00149671	10574/10694/10576/7203/908/1736	6
0.00184907	0.0016006	6662/57106/7025/6879/8467/6598/5	34
0.00191758	0.00165991	8404/2823/1948/1627/2149/1501/25	51
0.00203241	0.0017593	2534/7175/10574/142/10155/8692/1	16

0.00209644	0.00181474	57060/6659/4194/3276/7155/4848/5	25
0.0021643	0.00187348	4760/6664/3066/142/51773/1869/33	21
0.00224189	0.00194064	10715/7298/9768/10574/5425/5111/	28
0.00224189	0.00194064	5396/182/6657/51593/1000/5108/32	8
0.00225786	0.00195447	26053/3276/55904/6598/4297/23028	23
0.00227977	0.00197342	4239/5764/10715/56160/142/23028/	16
0.00227977	0.00197342	55366/5806/5764/9500/4841/4192/3	39
0.00228237	0.00197568	51497/6598/1660/51773/5438/6597/	14
0.00228237	0.00197568	57060/6659/4194/3276/4848/4850/1	19
0.00228237	0.00197568	51497/7155/6598/5586/1660/51773/	19
0.00228237	0.00197568	64110/142/23028/80762/5531/2956/	19
0.00228237	0.00197568	114088/4133/10391/7082/1191/1003	49
0.00228237	0.00197568	351/580/6431/8106/10212/10250/30	24
0.00228237	0.00197568	51497/6919/8621/6749/23476/5438/	20
0.00238187	0.00206181	1948/55816/6091/4781/2115/2534/7	37
0.00253637	0.00219555	57060/6659/4194/3276/7155/4848/5	19
0.00253637	0.00219555	50861/4133/1003/10297/84722/3482	20
0.00255844	0.00221465	10574/7013/10694/10576/7203/908/	7
0.00255844	0.00221465	26053/55904/4297/1786/23168/7936	7
0.00260331	0.00225349	5591/10574/142/5425/5111/3184/70	26
0.00261054	0.00225975	92126/1464/1462/63827/10675/5550	6
0.002616	0.00226448	1948/5087/6664/6662/182/6657/928	40
0.00266839	0.00230983	10574/7013/10694/10576/7203/908/	9
0.00272699	0.00236056	23224/6091/9289/23236/5454/5455/	20
0.00272699	0.00236056	10715/10574/5111/3184/7013/3192/	19
0.00272699	0.00236056	1948/5087/6662/182/6657/4133/351	31
0.00273856	0.00237057	5721/5690/11065/27338/8452/5720/	16
0.00284585	0.00246344	92126/1464/1462/79586/26229/6382	11
0.00284585	0.00246344	26053/6659/3066/6879/6598/4297/2	15
0.00284585	0.00246344	4678/8467/51773/81611/64946/5928	13
0.00356745	0.00308808	4176/5425/5111/51659/5984/4172/4	7
0.00357143	0.00309152	91272/10574/7013/25836/5885/1069	16
0.00396666	0.00343365	10574/5111/3184/10694/10576/7203	14
0.00396666	0.00343365	10574/142/7013/3192/10694/10576/	13
0.00396666	0.00343365	11014/10945/3309/8733/10956/1107	6
0.00396666	0.00343365	10574/10694/10576/7203/908/1736	6
0.00435402	0.00376895	6431/8106/10212/7175/100101267/1	23
0.00435919	0.00377343	2534/55353/4194/6599/51374/7076/	21
0.00435919	0.00377343	26053/55904/6598/4297/23028/2347	21
0.00449041	0.00388702	10772/6636/24148/11171/6628/9733	12
0.00468955	0.00405939	5396/5591/142/1660/10155/5111/97	18
0.0047488	0.00411068	1464/1462/79586/63827/64131/1067	8
0.0047488	0.00411068	4176/5425/5111/64710/51659/5984/	8
0.0047488	0.00411068	91272/8243/25836/5885/23137/2304	8
0.00509416	0.00440964	29899/7175/8243/7756/3192/10735/	19
0.00509416	0.00440964	10574/7013/3192/10694/10576/7203	14
0.00523	0.00452722	1290/55366/5087/6664/6662/182/66	47

0.00524423	0.00453954	4781/5396/6662/5764/6935/9678/64	9
0.0053116	0.00459785	51575/10528/55636/115416/114049/	38
0.00542206	0.00469347	10574/142/3184/7013/3192/10694/1	15
0.00550782	0.00476771	26053/3276/55904/6598/4297/23028	26
0.00550782	0.00476771	6421/25836/2237/5885/23137	5
0.00556385	0.00481621	2534/55353/1268/4194/154791/1660	39
0.00569338	0.00492833	10574/10694/10576/7203/908/1736	6
0.00598843	0.00518374	10574/7013/25836/10694/10576/720	8
0.00598843	0.00518374	7298/205/4832/1841/6240/4833/185	8
0.00603897	0.00522749	10574/142/7013/3192/10694/10576/	15
0.00614202	0.00531669	26053/6659/3066/9678/6879/11177/	32
0.00616434	0.00533601	26053/55904/4297/23476/54904/178	10
0.00616434	0.00533601	2817/2137/92126/1464/1462/79586/	16
0.00617006	0.00534096	1627/1501/2534/5764/54664/4133/1	22
0.00626133	0.00541997	91272/7756/324/3192/55920/891/83	9
0.00626133	0.00541997	10715/64110/7644/142/4297/80762/	20
0.00632722	0.005477	6185/1650/200185/3998/54344/7905	14
0.00665379	0.0057597	7175/8243/7756/3192/10735/3796/4	13
0.00665379	0.0057597	6091/4781/4133/6656/166614/11324	15
0.00722826	0.00625697	115416/8886/65003/51121/6129/261	14
0.00730531	0.00632366	580/23353/11014/10945/3309/8733/	10
0.00752199	0.00651122	11168/6431/10772/23451/6732/2414	15
0.00753917	0.0065261	6659/482/4194/1191/1650/8805/543	36
0.00759278	0.0065725	84722/595/1163/4331/894/1027/116	9
0.00759278	0.0065725	7298/1719/1869/3398/7029/23476/5	9
0.00777118	0.00672693	1660/1869/1994/3184/2521/3192/10	12
0.00783564	0.00678273	4171/4175/604/4172/4173/2260	6
0.00785858	0.00680259	23224/4781/1031/6664/6662/6657/9	37
0.00819322	0.00709226	29899/2770/50861/4133/1003/10297	26
0.00823278	0.00712651	3485/6558/351/1910/490/1268/1284	38
0.00823278	0.00712651	388419/5806/51497/7155/3609/6598	30
0.00842854	0.00729596	11168/64710/7520/8815/5478	5
0.0084517	0.00731601	5591/65003/7520/6175/3921/6193/6	13
0.0084517	0.00731601	1004/56127/1003/5098/56132/22225	10
0.0084517	0.00731601	55366/6091/4781/8324/6662/182/57	32
0.00873688	0.00756287	10926/1660/5111/7013/8914/64710/	18
0.00874149	0.00756686	10130/9601/2923/55829/10549/2307	12
0.00876207	0.00758467	10574/3184/10694/10576/7203/908/	11
0.00897513	0.0077691	26053/55904/4297/23028/1786/2316	8
0.00902939	0.00781607	6662/2534/9500/166614/5569/580/2	34
0.00902939	0.00781607	50861/4133/1003/10297/84722/3482	15
0.00945024	0.00818037	1660/1869/1994/3184/2521/3192/10	13
0.00945024	0.00818037	6431/8106/10212/7175/100101267/1	18
0.00945024	0.00818037	6938/83700/6659/6929/5803/1021/5	24
0.00990323	0.00857249	4133/1003/84722/7756/3925/440145	14
0.01006627	0.00871362	4781/6664/6662/10252/6431/10253/	11
0.01006627	0.00871362	9768/5425/5111/6119/7334/4436/59	11

0.01045635	0.00905129	64946/1063/201254/9787/64105/106	6
0.01066065	0.00922813	10574/10694/10576/7203/908/7520/	9
0.01066065	0.00922813	6879/6598/55729/6597/2958/55832/	9
0.0109733	0.00949877	2823/6624/54874/57451/163404/897	8
0.01112809	0.00963276	6938/6929/1021/5591/4297/5721/56	15
0.01112809	0.00963276	1627/6091/3679/59284/22836/2534/	53
0.01115048	0.00965214	2823/1627/6091/26053/59284/2534/	44
0.01115048	0.00965214	29899/2770/2534/5764/9747/7430/7	39
0.01198409	0.01037373	26053/6879/7994/6598/4297/284058	13
0.01219311	0.01055466	4781/7976/1605/6259/4684	5
0.01219311	0.01055466	54499/55829/84919/3309/57003	5
0.01219311	0.01055466	4171/2956/4436/4331/6742	5
0.01219311	0.01055466	1266/482/1660/29128/51726/4171/2	14
0.01259159	0.0108996	6659/142/5425/64949/5111/6119/59	9
0.01265359	0.01095328	54499/351/57003/58515/7009/488/5	7
0.01265359	0.01095328	54344/23753/11041/6388/84899/147	7
0.01265359	0.01095328	11065/27338/4085/26271/991/6125/	7
0.01265359	0.01095328	2823/1627/2534/5764/351/23394/23	30
0.01310655	0.01134537	23224/29899/23353/7430/4085/1014	8
0.01310655	0.01134537	1869/7029/23468/5928/5932/1111/2	10
0.01350515	0.0116904	2534/9500/166614/5569/7175/10574	19
0.01353091	0.0117127	2149/4760/83700/6558/6662/482/49	50
0.01398116	0.01210245	1948/5087/6664/6662/182/6657/928	39
0.01423459	0.01232182	55353/1268/1660/57533/6599/1869/	32
0.01423459	0.01232182	2823/6938/6664/6662/182/5764/692	32
0.01451487	0.01256444	6662/7025/6879/367/55729/10155/6	25
0.01493714	0.01292997	482/1660/29128/51726/2956/131118	11
0.01520448	0.01316139	64110/142/23028/5531/2521/8914/1	10
0.01531128	0.01325384	1627/6091/4133/5454/4192/23394/8	36
0.01578932	0.01366764	2817/2137/26229/64131/57216/1267	8
0.01578932	0.01366764	482/1952/113246/80031/4628/1021/	8
0.01630091	0.01411049	3642/9289/5764/7976/5803/10763/4	15
0.0165158	0.0142965	6938/6929/1021/5591/4297/5721/56	13
0.01724224	0.01492532	84919/3309/65992/1388/23376	5
0.01724224	0.01492532	324/3192/55920/891/1894	5
0.01728104	0.01495891	1627/6091/59284/2534/5764/482/54	34
0.01748889	0.01513883	5569/84268/7175/100101267/6636/1	23
0.01775615	0.01537018	10574/10694/10576/7203/908/1736	6
0.01803021	0.01560741	2823/1290/5087/341640/4760/6664/	43
0.01854591	0.01605381	1627/50861/55553/4678/6746/55636	42
0.01854591	0.01605381	1627/1501/2534/5764/54664/4133/3	30
0.0187582	0.01623758	28951/1917/6662/2534/55353/4194/	43
0.01917335	0.01659694	26053/55904/4297/23028/58508/178	11
0.01917335	0.01659694	23543/6879/4848/142/367/8289/659	11
0.01936788	0.01676533	2137/26229/64131/57216/126792/93	7
0.02004018	0.01734729	6598/4297/23028/1786/2122/1111/5	10
0.02006791	0.01737129	7175/1660/10155/8692/11052/10657	12

0.02030944	0.01758037	25879/5591/6218/7520/3921/6217/6	13
0.02097391	0.01815555	6091/5087/10098/1004/6662/182/35	25
0.02235553	0.01935152	55366/6091/6662/182/5764/9500/10	14
0.02235553	0.01935152	1948/388419/5806/4774/7155/3609/	38
0.02259426	0.01955817	10130/2137/3309/56005/51726/1089	12
0.02286337	0.01979112	6091/351/1268/682/2290/6092/4897	10
0.02286337	0.01979112	6713/2194/2222/3156/2224/5447/71	10
0.02286337	0.01979112	29899/10252/10253/3688/8976/4085	10
0.02350094	0.02034302	115106/7283/10142/8636/9793/1125	15
0.02367356	0.02049244	51497/8621/23476/3192/11198/2146	7
0.02438225	0.0211059	55353/6599/51374/1457/10956/6599	14
0.02438225	0.0211059	6938/6929/1021/5591/4297/5721/56	14
0.0245548	0.02125527	6659/482/4194/1191/811/10574/547	24
0.02464495	0.0213333	2817/92126/1464/1462/79586/11041	17
0.02464594	0.02133416	2823/4760/6664/6662/182/5764/665	37
0.02545652	0.02203582	4781/4760/5764/482/4192/23543/46	21
0.02624021	0.0227142	7298/4913/4521/1841/30833/6240/1	8
0.02624021	0.0227142	29899/10252/10253/3688/4085/6993	8
0.02624021	0.0227142	1031/84722/7026/595/1163/324/101	16
0.02637855	0.02283395	23236/4603/23353/7155/811/8243/2	30
0.02643459	0.02288246	54732/11014/10945/10959/10960/51	14
0.02643459	0.02288246	51497/8621/6749/23476/5438/3192/	14
0.02648856	0.02292918	5479/2286/4820/5480/55033/60681/	9
0.0276926	0.02397143	7091/6656/8467/10253/10765/9774/	15
0.02825018	0.02445408	5591/7520/3921/6193/6223/6209	6
0.02825018	0.02445408	84919/3309/65992/1388/23376/581/	7
0.02825018	0.02445408	1627/2534/5764/9456/10152/4897/7	16
0.02913949	0.02522389	10926/5111/7013/8914/26271/546/6	10
0.02913949	0.02522389	5764/351/3309/1869/23095/3397/57	10
0.03005934	0.02602013	23353/84722/91272/7013/3192/1063	13
0.03029884	0.02622745	2823/6624/4651/7430/54874/57451/	11
0.03029884	0.02622745	6664/7082/7003/8463/100505385/75	9
0.03029884	0.02622745	4781/5396/6662/5764/6935/9678/64	9
0.03029884	0.02622745	4781/113246/5747/1630/2043/9334/	8
0.03070115	0.0265757	5764/4192/23394/5111/8648	5
0.03070115	0.0265757	9738/27243/55755/672/4869	5
0.03297565	0.02854456	6664/3066/51773/1869/3398/23028/	10
0.03468153	0.03002122	23236/4603/23353/7155/811/2956/7	24
0.03491011	0.03021908	142/2521/8914/79915/51010/7468/4	9
0.03491011	0.03021908	6091/6664/6659/3398/6092/8321/57	9
0.03491011	0.03021908	143872/3688/3925/8452/253980/604	6
0.0354431	0.03068046	54344/79053/23753/11041/6388/848	8
0.0354431	0.03068046	7155/5586/5479/6732/64710/7153/5	8
0.03718031	0.03218422	6662/182/6935/10252/6431/10253/3	10
0.03718031	0.03218422	7334/11065/27338/4085/26271/1131	10
0.03731025	0.0322967	7857/2534/9500/2923/4841/55829/8	43
0.03864846	0.0334551	2893/59284/81831/2534/23236/2897	15

0.03886945	0.03364639	3309/811/7184/1388	4
0.03886945	0.03364639	4781/6662/6431/93986	4
0.03886945	0.03364639	482/23543/473/3688	4
0.03886945	0.03364639	3398/8321/57216/7468	4
0.03886945	0.03364639	5111/3146/4869/6188	4
0.03934479	0.03405786	2823/6624/4651/54874/57451/16340	9
0.03934479	0.03405786	29899/2770/50861/4133/254263/100	28
0.03934479	0.03405786	7298/1841/6240/1854/6241	5
0.03934479	0.03405786	4176/4171/6742/4175/4173	5
0.03934479	0.03405786	23476/54904/64324/7468/55209	5
0.03934479	0.03405786	25836/10051/51203/10592/64151	5
0.03934479	0.03405786	4085/26271/11315/6125/6201	5
0.03934479	0.03405786	7175/1660/11052/10657/64062	5
0.03969553	0.03436147	4781/3696/5396/6662/55553/6935/4	24
0.03992466	0.03455981	6598/23028/1786/2122/55209/546/5	8
0.03992466	0.03455981	29899/2770/84722/7175/3192/22974	8
0.04070119	0.03523199	2534/6659/56160/80758/10362/3309	28
0.04074418	0.03526921	1627/580/23353/11014/10945/3309/	11
0.04102729	0.03551427	55366/2149/10391/5569/580/90007/	22
0.04102729	0.03551427	1021/5591/26271/5933/604/4904	6
0.04102729	0.03551427	3066/6879/26155/6418/11315/672	6
0.04102729	0.03551427	5764/5803/4192/3066/1605/7852	6
0.04102729	0.03551427	6598/23028/1786/546/55183/672	6
0.0430511	0.03726614	1627/580/23353/11014/10945/3309/	14
0.0430511	0.03726614	23353/7155/7013/10609/80010/5885	14
0.04324804	0.03743661	25836/7153/10051/8815/51203/1059	9
0.04324804	0.03743661	84722/91272/3192/8452/27243/891/	9
0.04324804	0.03743661	1191/5591/1869/23028/2956/55031/	15
0.04475929	0.03874479	11168/6431/10772/23020/6427/6426	10
0.04475929	0.03874479	6929/5591/80762/2956/79915/51010	10
0.04521317	0.03913768	4841/55829/1191/6421/142/23028/9	21
0.04529151	0.03920549	50861/26053/117178/10152/199731/	8
0.04667806	0.04040572	4841/55829/1191/6421/5591/142/18	32
0.04925017	0.04263221	10715/3184/7013/10576/7520/3181/	9
0.04934106	0.04271089	6598/6597/64710/1105/3725	5
0.04934106	0.04271089	10926/7013/546/604/2260	5
0.04934106	0.04271089	50861/26053/117178/199731/6993	5
0.04958113	0.0429187	64110/5591/142/23028/5531/2521/8	13
0.04988633	0.04318289	3309/9774/5721/5690/7009/5720/56	16
0.00039715	0.00034226	4487/131566/2137/374946/5607/517	52
0.00039715	0.00034226	5454/6664/10215/9580/1000/2596/4	31
0.00039715	0.00034226	8425/9601/3309/10549/27000/11328	31
0.00039715	0.00034226	7111/10763/4130/65005/63931/5147	30
0.00039715	0.00034226	374946/51760/6662/23394/6585/986	31
0.00039715	0.00034226	5454/3679/7070/816/11178/22871/3	51
0.00039715	0.00034226	5307/7026/5454/4487/54820/10215/	43
0.00039715	0.00034226	3673/65009/2823/816/22871/51760/	43

0.00039715	0.00034226	2137/6464/57414/84919/51528/1616	28
0.0004945	0.00042615	57017/790955/1537/4698/4695/4729	18
0.00050104	0.00043178	65005/63931/64949/51264/64983/61	17
0.00050104	0.00043178	220296/9863/6659/6657/7832/324/2	30
0.00050104	0.00043178	5454/7070/816/11178/22871/374946	36
0.00079525	0.00068532	65005/63931/51335/64949/115416/5	21
0.00081935	0.00070609	2137/8406/6464/81542/9601/57414/	34
0.00081935	0.00070609	5454/6664/10215/9580/1000/2596/4	35
0.00081935	0.00070609	65005/63931/23708/64949/51264/64	18
0.00102809	0.00088598	221937/8671/669/2026/283209/1001	35
0.00108895	0.00093842	65009/6664/816/51704/22871/51760	40
0.00131274	0.00113128	65009/816/22871/51760/23394/9863	33
0.00131274	0.00113128	7111/6604/10763/84557/4130/3480/	36
0.0014793	0.00127481	1400/6664/7070/182/10215/22871/3	35
0.00169272	0.00145873	374946/23394/56262/22902/1182/48	24
0.00225918	0.00194689	57017/1346/790955/1537/4698/4695	21
0.00235037	0.00202548	57017/790955/1537/4698/4695/4729	18
0.00274736	0.00236759	1400/7070/182/10215/22871/374946	27
0.00294879	0.00254118	219287/5218/4435/25937/51704/374	38
0.00294879	0.00254118	8642/100133941/6382/4435/255743/	20
0.00294879	0.00254118	5454/54820/1000/6604/10763/5629/	21
0.00294879	0.00254118	6829/1105/51773/22938/7936/5430/	10
0.00294879	0.00254118	2137/6464/57414/84919/1616/3309/	22
0.00375941	0.00323975	374946/23394/22902/4897/91584/10	17
0.00393689	0.00339269	374946/23394/6585/22902/4897/915	18
0.00393689	0.00339269	1917/65005/63931/64949/51264/649	19
0.00393689	0.00339269	84557/5066/1616/351/5621/6284/45	10
0.00393689	0.00339269	1400/6664/7070/182/10215/22871/3	35
0.00393689	0.00339269	1000/6604/10763/5629/6469/7976/7	15
0.00419188	0.00361243	374946/51760/23394/6585/22902/78	22
0.00424071	0.00365452	2137/6464/57414/84919/3309/10130	18
0.00431585	0.00371927	56999/9806/151887/3673/3679/2557	36
0.00437535	0.00377054	374946/51760/6662/23394/6585/229	27
0.00437535	0.00377054	5454/7070/374946/1000/23394/2603	23
0.00480257	0.00413871	8642/9806/3673/7070/2823/83700/2	41
0.00485312	0.00418228	10098/64110/5054/25825/27344/646	31
0.00519529	0.00447714	10787/10152/10810/8976/55845	5
0.00519529	0.00447714	3309/5611/7184/821/811	5
0.00519529	0.00447714	8642/100133941/4435/1282/255743/	15
0.00531828	0.00458313	65005/63931/51335/64949/115416/5	21
0.00533011	0.00459333	57017/4698/4695/4729/4701/4716/4	11
0.00551886	0.00475599	816/22871/1000/58489/2596/2534/2	16
0.00555591	0.00478792	56999/3673/3679/255743/7070/8370	26
0.00570731	0.00491838	2020/4435/1271/374946/10763/2339	27
0.00570731	0.00491838	5307/6382/9547/182/6662/6469/361	14
0.00570731	0.00491838	1400/3800/374946/6464/2596/4781/	30
0.00631946	0.00544592	10215/6469/5803/5764/4192/1605/6	7

0.00668286	0.00575909	816/23394/8874/22902/170506/9158	20
0.00679308	0.00585407	8642/100133941/4435/255743/2593	16
0.00704372	0.00607006	5454/6664/25825/116448/6469/665	17
0.00750154	0.0064646	57017/112812/1346/51218/790955/	22
0.00783293	0.00675018	817/488/54499/351/58515/581/5102	7
0.00836969	0.00721275	81542/9601/56255/889/10549/1013	11
0.00879369	0.00757814	56999/59269/1525/5307/7026/1466/	38
0.0088965	0.00766673	116228/55744/51287/790955/4698/	15
0.00894922	0.00771217	2823/816/22871/1000/576/58489/25	26
0.00905709	0.00780512	22871/10787/388/22836/11215/947	11
0.00905709	0.00780512	3309/1191/11231/23471/131118/29	11
0.00908607	0.0078301	1525/5454/374946/51760/23394/56	34
0.00908607	0.0078301	6664/10215/9580/6662/116448/646	15
0.00908607	0.0078301	219287/6382/5218/25937/51704/37	33
0.00910262	0.00784436	816/22871/2534/26037/4130/10152/	14
0.00910262	0.00784436	7111/374946/5217/10787/23394/56	36
0.00964996	0.00831604	4781/7976/4684/1605/7422	5
0.00964996	0.00831604	84919/3309/54499/55829/1649	5
0.01007197	0.00867972	9806/151887/255743/7070/182/505	24
0.0110712	0.00954082	23394/22902/9037/3611/51330/742	9
0.0110712	0.00954082	8642/100133941/6382/4435/1282/2	32
0.0110712	0.00954082	9806/1282/2823/816/22871/1000/5	39
0.01218817	0.0105034	5307/7026/4487/6664/5396/5087/6	21
0.01218817	0.0105034	8642/100133941/4435/255743/2593	18
0.01241089	0.01069533	8642/6664/25937/7003/9686/836/7	9
0.01241089	0.01069533	8642/4487/5396/4781/6662/6469/96	9
0.01241089	0.01069533	5307/5454/4487/10215/6469/9001/	12
0.01323465	0.01140522	3309/11231/29927/7095/23480	5
0.01376325	0.01186075	23394/22902/91584/9037/8153/361	13
0.01376325	0.01186075	9590/7425/816/11178/22871/1000/	40
0.01376325	0.01186075	8642/4435/255743/6664/25937/182/	37
0.0141841	0.01222343	55744/4698/4695/4729/4701/4716/	11
0.0141841	0.01222343	6829/1105/51773/22938/7936/5430/	11
0.01520807	0.01310586	84557/1616/351/5621/4501/4504/45	7
0.01561997	0.01346082	816/22871/1000/58489/2596/9863/	20
0.01893611	0.01631857	7070/182/5054/780/22998/395/857	16
0.01893611	0.01631857	5307/4487/6664/5396/5087/6662/32	18
0.01973655	0.01700836	25800/8692/57181/7922/51312/351/	17
0.02007614	0.01730101	83700/7111/5217/10787/5629/817/	36
0.02007614	0.01730101	5454/54820/1000/4781/5629/6469/	26
0.02159441	0.01860941	59269/1525/5307/7026/4487/3679/	36
0.02181506	0.01879956	83707/6100/10656/8996/10189/516	41
0.02206785	0.01901741	9001/9475/351/55851/1191/836/51	8
0.02206785	0.01901741	9855/199731/10787/10152/3845/51	8
0.02337183	0.02014113	2817/6469/406/26263/3156/10211/	9
0.02337183	0.02014113	790955/1537/7386/54205/7388	5
0.02466735	0.02125758	255743/6664/182/4086/490/51430/	13

0.02466735	0.02125758	59269/5307/7026/6664/1271/2817/6	19
0.02466735	0.02125758	8642/4435/1282/255743/5087/6662/	18
0.02466735	0.02125758	9747/58489/23208/245812/8692/253	31
0.02466735	0.02125758	10215/6469/5803/5764/4192/3400/1	8
0.02466798	0.02125812	8996/1191/51499/25994/572/79017/	10
0.02491815	0.02147371	25825/9001/81618/9475/351/55851/	11
0.02491815	0.02147371	9001/9475/351/55851/1191/836/511	9
0.02491815	0.02147371	54820/10253/4926/8976/10252/3688	9
0.02704643	0.0233078	8642/56999/182/6662/6659/23462/5	10
0.02704643	0.0233078	80758/5607/2149/2534/8996/6657/2	24
0.02704643	0.0233078	4435/7425/25948/6464/1036/2887/2	38
0.02704643	0.0233078	4487/56950/6659/7832/754/10397/5	14
0.02704643	0.0233078	6829/1105/51773/6733/22938/51495	14
0.02704643	0.0233078	25825/9001/9475/351/55851/1191/8	9
0.02704643	0.0233078	84919/488/3309/54499/55829/7184/	9
0.02704643	0.0233078	7026/11142/7070/25937/81848/9403	26
0.02734442	0.0235646	65009/23208/2534/4130/4212/3845/	17
0.02746112	0.02366516	10787/10152/10810/8976/55845	5
0.02847293	0.02453711	54820/6604/6469/7976/5764/3400/7	10
0.03000531	0.02585767	6664/4781/6662/6469/10253/3845/8	9
0.03000531	0.02585767	5454/54820/1000/5629/5455/7832/2	19
0.03032902	0.02613663	5454/5629/9001/7832/836/22803/41	14
0.03032902	0.02613663	2534/1616/997/6733/1191/22943/83	13
0.03032902	0.02613663	57017/790955/1537/3091/4698/4695	20
0.03069495	0.02645198	5396/4781/6662/6469/9678/5764/74	7
0.03069495	0.02645198	10656/8996/10189/51631/51634/673	34
0.03069495	0.02645198	3309/7184/811/1649	4
0.03069495	0.02645198	482/51286/3688/51176	4
0.03069495	0.02645198	6382/1036/10266/5066/1616/406/38	31
0.03279011	0.02825753	4435/6664/6604/6469/7976/7422/80	8
0.03326118	0.02866348	2020/4435/1271/374946/10763/2339	17
0.03432035	0.02957624	22871/6585/2534/22943/5764/5621	6
0.03522193	0.03035319	7026/55450/324/836/1030/1029/102	7
0.03589632	0.03093437	6604/10763/5629/6469/7976/9289/4	9
0.0365933	0.031535	7111/10763/4130/51474/9037/324/1	12
0.0365933	0.031535	6664/7070/182/6096/6662/5629/241	15
0.0365933	0.031535	816/817/488/100101267/1616/56892	15
0.0365933	0.031535	3673/3757/2534/7976/5066/3309/90	34
0.03755624	0.03236483	10215/374946/4781/1454/5629/3226	20
0.03822393	0.03294023	283209/57017/55829/5834/790955/1	26
0.03822393	0.03294023	57106/25937/7003/6096/6662/7025/	24
0.03876783	0.03340895	5454/83700/10215/2817/23114/1081	16
0.03900692	0.03361499	8642/4435/255743/5087/6662/6469/	10
0.03900692	0.03361499	54820/10253/4926/10252/3688/5048	7
0.03977925	0.03428056	100133941/9747/2534/83871/3309/2	30
0.04098718	0.03532151	6664/6662/6469/6659	4
0.04098718	0.03532151	3673/7070/3915/8874/7205/22998/3	13

0.0424755	0.0366041	2137/6464/3309/10130/10897/1200/	10
0.04383193	0.03777304	8642/6382/4435/255743/5087/6662/	13
0.04383193	0.03777304	2596/6657/5764/4192/1605/3475/79	11
0.04383193	0.03777304	54820/10253/4926/10252/3688/5048	7
0.04383193	0.03777304	4435/255743/6664/182/5087/6096/6	21
0.04383193	0.03777304	84919/3309/10955/1191/5611/55829	12
0.04383193	0.03777304	221937/8671/669/2026/283209/1001	17
0.044262	0.03814365	25800/57181/7922/351/51310/15117	14
0.04455128	0.03839295	5054/29106/10916/351/81/54495/11	15
0.04528266	0.03902323	100133941/4435/25937/182/9863/64	9
0.04528266	0.03902323	2020/4435/1271/374946/10763/2339	31
0.04686619	0.04038786	22853/9863/84919/81706/57181/262	14
9.24E-08	8.64E-08	79628/1739/5010/745/20/4155/7368	14
4.27E-06	3.99E-06	23499/667/11141/88/28513/1739/50	20
1.07E-05	9.97E-06	7368/6900/23114/288/1314	5
0.00372917	0.0034873	79628/60484/7368/9639/23114/1631	7
0.01274941	0.01192249	11141/1739/26052/9863/5789/2895/	9
0.01274941	0.01192249	20/7368/23114/23405	4
0.01346476	0.01259145	23499/667/88/1307/10395/2017/579	7
0.01401694	0.01310782	11141/83992/1739/26052/9863/5789	14
0.01495649	0.01398642	9053/23499/88/1739/23114/4301/28	11
0.01555006	0.0145415	9053/23499/88/1739/20/23114/4301	12
0.01555006	0.0145415	88/23187/20/7368/11155/23114/234	7
0.01913534	0.01789424	11141/5789/6900	3
0.01913534	0.01789424	23499/88/1307/10395/2017/5796	6
0.0201726	0.01886422	79628/745/20/6900/9639/163175/23	7
0.0201726	0.01886422	23499/11141/80005/26052/5789/690	11
0.0201726	0.01886422	6421/10521/10181/6430/58517/9444	6
0.02199776	0.020571	79628/9639/163175/23405	4
0.02747852	0.02569629	23499/11141/80005/1739/26052/578	14
0.03009419	0.0281423	130271/88/1739/961/9639/23671/51	10
0.03316195	0.0310111	23499/11141/10690/26052/9863/578	12
0.04392606	0.04107706	79628/9639/163175/23405	4

Cluster	ID	GeneRatio	BgRatio	pvalue	p.adjust	qvalue
0	P53_DN.V1_DN	23/345	194/10913	4.56E-08	8.20E-06	6.96E-06
0	RPS14_DN.V1_UP	21/345	191/10913	6.37E-07	5.73E-05	4.86E-05
0	STK33_SKM_UP	24/345	277/10913	7.36E-06	0.0004418	0.0003746
0	HOXA9_DN.V1_UP	18/345	192/10913	3.68E-05	0.0014206	0.0012046
0	RAF_UP.V1_UP	18/345	193/10913	3.95E-05	0.0014206	0.0012046
0	NFE2L2.V2	30/345	469/10913	0.00019	0.00519	0.0043993
0	CAMP_UP.V1_UP	17/345	200/10913	0.00020	0.00519	0.0043993
0	ERBB2_UP.V1_UP	14/345	190/10913	0.00283	0.04124	0.0349714
0	EGFR_UP.V1_UP	14/345	192/10913	0.00312	0.04124	0.0349714
0	LEF1_UP.V1_UP	14/345	194/10913	0.00342	0.04124	0.0349714
0	YAP1_UP	6/345	47/10913	0.00344	0.04124	0.0349714
1	EIF4E_UP	14/338	100/10913	2.27E-06	0.00033	0.0003158
1	CAMP_UP.V1_UP	20/338	200/10913	3.80E-06	0.00033	0.0003158
1	CSR_LATE_UP.V1_UP	17/338	166/10913	1.49E-05	0.00086	0.0008261
1	SIRNA_EIF4GI_UP	11/338	95/10913	0.0001632	0.00710	0.0067876
2	RB_DN.V1_UP	11/250	117/10913	7.46E-05	0.00750	0.0069377
2	CSR_LATE_UP.V1_UP	13/250	166/10913	0.00011	0.00750	0.0069377
2	RB_P130_DN.V1_UP	11/250	129/10913	0.00018	0.00750	0.0069377
2	RB_P107_DN.V1_UP	11/250	130/10913	0.00019	0.00750	0.0069377
3	RPS14_DN.V1_UP	39/407	191/10913	1.32E-18	2.41E-16	2.04E-16
3	HOXA9_DN.V1_UP	35/407	192/10913	3.37E-15	3.09E-13	2.61E-13
3	MEL18_DN.V1_UP	20/407	141/10913	2.57E-07	1.57E-05	1.33E-05
3	SNF5_DN.V1_UP	22/407	173/10913	4.55E-07	2.08E-05	1.76E-05
3	STK33_UP	29/407	285/10913	8.65E-07	3.17E-05	2.68E-05
3	STK33_NOMO_UP	28/407	290/10913	3.77E-06	0.0001151	9.73E-05
3	P53_DN.V1_DN	21/407	194/10913	1.12E-05	0.0002552	0.0002158
4	KRAS.KIDNEY_UP.V1_UP	15/186	141/10913	1.59E-08	2.68E-06	2.49E-06
4	CAHOY_OLIGODENDROCUTIC	8/186	100/10913	0.00030	0.02530	0.0234818
4	P53_DN.V1_DN	11/186	194/10913	0.00048	0.02719	0.0252302
5	RPS14_DN.V1_UP	13/101	191/10913	2.04E-08	2.84E-06	2.58E-06
5	ESC_V6.5_UP_EARLY.V1_DN	7/101	161/10913	0.00072	0.04997	0.0454105
6	JAK2_DN.V1_DN	31/687	145/10913	1.24E-09	2.29E-07	2.15E-07
6	TBK1.DF_DN	43/687	285/10913	6.78E-08	6.27E-06	5.89E-06
6	PIGF_UP.V1_UP	30/687	190/10913	2.58E-06	0.00016	0.0001495
6	GCNP_SHH_UP_LATE.V1_UP	25/687	181/10913	0.00017	0.00611	0.0057393
6	MTOR_UP.N4.V1_DN	25/687	183/10913	0.00020	0.00611	0.0057393
6	VEGF_A_UP.V1_DN	24/687	193/10913	0.00105	0.02427	0.0227893
6	KRAS.KIDNEY_UP.V1_UP	19/687	141/10913	0.00132	0.02711	0.025449
7	STK33_NOMO_UP	41/555	290/10913	2.16E-09	4.05E-07	3.79E-07
7	STK33_UP	34/555	285/10913	3.00E-06	0.00024	0.0002223
7	JAK2_DN.V1_DN	22/555	145/10913	3.79E-06	0.00024	0.0002223
7	RPS14_DN.V1_UP	25/555	191/10913	1.26E-05	0.00053	0.0004917
7	BCAT_BILD_ET_AL_DN	11/555	46/10913	1.40E-05	0.00053	0.0004917
8	RB_P107_DN.V1_UP	30/814	130/10913	1.75E-08	3.25E-06	2.81E-06
8	VEGF_A_UP.V1_DN	37/814	193/10913	7.66E-08	7.12E-06	6.17E-06
8	CSR_LATE_UP.V1_UP	33/814	166/10913	1.60E-07	9.90E-06	8.57E-06
8	TBK1.DF_DN	46/814	285/10913	4.51E-07	2.10E-05	1.82E-05
8	E2F1_UP.V1_UP	34/814	188/10913	1.07E-06	3.97E-05	3.43E-05
9	TBK1.DF_DN	35/610	285/10913	9.07E-06	0.00089	0.000817
9	P53_DN.V1_UP	27/610	194/10913	1.01E-05	0.00089	0.000817
9	TBK1.DN.48HRS_UP	12/610	50/10913	1.43E-05	0.00089	0.000817

9	LEF1_UP.V1_UP	26/610	194/10913	2.87E-05	0.00134	0.0012331
9	KRAS.KIDNEY_UP.V1_UP	20/610	141/10913	0.00011	0.00401	0.0036829

geneID	Count
2171/597/123/11343/341/10409/231/31	23
822/597/7305/4688/199/7941/1545/408	21
3956/28984/597/8878/7305/301/4688/3	24
7305/6280/4688/29887/2207/1890/261	18
6386/2745/6280/80896/2760/831/1013	18
26471/2512/8878/586/2495/6888/4688	30
2745/3987/25946/3069/375/9659/1890	17
6386/1601/6277/831/5878/2771/1545/5	14
6386/2745/6280/11343/58472/3726/23	14
3956/28984/6275/51177/4478/2014/59	14
28984/4478/23433/7534/23325/64092	6
9551/4191/63875/29058/51160/404672	14
4502/22861/6142/4830/4191/1351/506	20
4502/6611/4828/2280/6723/112495/19	17
23741/3329/4728/27335/292/3315/108	11
3925/5094/4833/5111/10572/1786/290	11
3146/11065/4150/3608/91057/51155/1	13
6628/25804/11065/280636/3094/5438/	11
3151/3925/5757/6119/6993/5111/5152	11
10457/1520/2517/3119/29992/1512/92	39
2517/1514/3122/2217/51816/3108/513	35
1520/3117/6510/5329/9308/719/10184	20
348/714/968/963/3689/928/51338/2364	22
928/728/5328/84868/23643/9976/3587	29
10457/714/54/2/728/5328/719/84868/2	28
10457/1522/1514/5552/54/2040/10154	21
1410/5375/5354/760/10382/2628/6414	15
8537/22933/51090/7368/128853/745/2	8
1410/6285/760/4118/6271/4747/3306/6	11
597/4688/7941/199/29108/3108/7940/2	13
951/5176/301/6275/26471/302/6277	7
283131/25957/9988/80055/51380/2897	31
25957/51747/1021/10643/490/2186/91	43
7503/9988/80055/5980/5189/29994/51	30
51747/7514/3192/1462/27332/10772/1	25
283131/29896/51351/26137/10252/632	25
80055/51351/4289/91304/9497/26278/	24
2891/2115/1848/5101/28951/5178/288	19
283131/81501/25943/1316/25780/5328	41
10161/145389/51351/25943/1316/2578	34
283131/25957/23015/57018/440270/47	22
7127/3687/23312/4542/25780/5328/15	25
1195/79101/374868/55870/9462/79048	11
4176/10656/1719/10549/1869/5111/64	30
7525/80055/7025/5411/7112/4001/102	37
81831/91057/29128/3608/51726/7076/	33
1290/10098/7525/5050/51575/7586/97	46
1948/7091/4678/10528/768211/3149/7	34
1525/3673/1282/10098/9747/7003/102	35
4232/1525/5307/100133941/7026/3673	27
7158/780/54499/6657/54681/8731/485	12

9590/2020/1114/7111/5054/56967/227	26
1114/2823/6252/7111/51704/3800/114	20

Cluster	ID	GeneRatio	BgRatio	pvalue
0	GSE22886_NAIVE_BCELL_VS_MONOCYTE_DN	53/476	200/20394	8.73E-41
0	GSE29618_PDC_VS_MDC_DN	50/476	200/20394	3.75E-37
0	GSE22886_NAIVE_TCELL_VS_MONOCYTE_DN	49/476	200/20394	5.76E-36
0	GSE29618_BCELL_VS_MONOCYTE_DN	47/476	200/20394	1.25E-33
0	GSE22886_NAIVE_CD8_TCELL_VS_MONOCYTE_	46/476	200/20394	1.75E-32
0	GSE22886_NAIVE_CD4_TCELL_VS_MONOCYTE_	45/476	200/20394	2.39E-31
0	GSE29618_MONOCYTE_VS_PDC_UP	44/476	199/20394	2.52E-30
0	GSE22886_DAY0_VS_DAY1_MONOCYTE_IN_CUL	42/476	200/20394	5.04E-28
0	GSE3982_MAC_VS_NKCELL_UP	32/476	199/20394	5.92E-18
0	GSE3982_DC_VS_NKCELL_UP	30/476	200/20394	4.73E-16
1	GSE45837_WT_VS_GFI1_KO_PDC_DN	58/692	200/20394	8.18E-38
1	GSE2405_0H_VS_9H_A_PHAGOCYTOPHILUM_ST	56/692	200/20394	1.25E-35
1	GSE41978_ID2_KO_VS_ID2_KO_AND_BIM_KO_KL	47/692	200/20394	2.23E-26
1	GSE21927_SPLEEN_C57BL6_VS_EL4_TUMOR_BA	42/692	200/20394	1.13E-21
1	GSE42724_NAIVE_VS_B1_BCELL_DN	41/692	199/20394	7.40E-21
1	GSE26030_TH1_VS_TH17_RESTIMULATED_DAY5	41/692	200/20394	9.01E-21
1	GSE3982_EOSINOPHIL_VS_TH1_DN	40/692	200/20394	6.96E-20
1	GSE26030_TH1_VS_TH17_RESTIMULATED_DAY1	37/692	199/20394	2.21E-17
1	GSE22886_NAIVE_CD4_TCELL_VS_48H_ACT_TH	34/692	199/20394	6.26E-15
1	GSE26156_DOUBLE_POSITIVE_VS_CD4_SINGLE_	34/692	199/20394	6.26E-15
2	GSE3720_UNSTIM_VS_LPS_STIM_VD2_GAMMAD	35/578	167/20394	9.93E-21
2	GSE12845_NAIVE_VS_PRE_GC_TONSIL_BCELL_	37/578	200/20394	6.90E-20
2	GSE15930_NAIVE_VS_48H_IN_VITRO_STIM_CD8_	37/578	200/20394	6.90E-20
2	GSE18893_TCONV_VS_TREG_24H_TNF_STIM_U	37/578	200/20394	6.90E-20
2	GSE9006_HEALTHY_VS_TYPE_2_DIABETES_PBM	37/578	200/20394	6.90E-20
2	GSE22886_UNSTIM_VS_IL15_STIM_NKCELL_DN	36/578	200/20394	5.67E-19
2	GSE9006_TYPE_1_VS_TYPE_2_DIABETES_PBMC	36/578	200/20394	5.67E-19
2	GSE26030_TH1_VS_TH17_RESTIMULATED_DAY1	34/578	199/20394	2.94E-17
2	GSE21670_UNTREATED_VS_TGFB_IL6_TREATED	33/578	198/20394	1.87E-16
2	GSE14699_DELETIONAL_TOLERANCE_VS_ACTIV	32/578	186/20394	2.08E-16
3	GSE24634_TREG_VS_TCONV_POST_DAY10_IL4_	82/541	200/20394	2.47E-76
3	GSE29618_MONOCYTE_VS_MDC_DAY7_FLU_VA	59/541	200/20394	4.33E-45
3	GSE29618_MONOCYTE_VS_MDC_UP	54/541	200/20394	4.70E-39
3	GSE22886_NAIVE_CD4_TCELL_VS_MONOCYTE_	50/541	200/20394	1.94E-34
3	GSE22935_WT_VS_MYD88_KO_MACROPHAGE_	45/541	200/20394	6.02E-29
3	GSE29618_BCELL_VS_PDC_DN	44/541	198/20394	4.40E-28
3	GSE3982_MAC_VS_CENT_MEMORY_CD4_TCELL	38/541	200/20394	8.08E-22
3	GSE15767_MED_VS_SCS_MAC_LN_UP	37/541	200/20394	7.39E-21
3	GSE3982_MAC_VS_NKCELL_UP	30/541	199/20394	1.26E-14
3	GSE3982_MAC_VS_TH2_UP	30/541	199/20394	1.26E-14
4	GSE2405_0H_VS_9H_A_PHAGOCYTOPHILUM_ST	45/324	200/20394	7.99E-39
4	GSE41978_ID2_KO_VS_ID2_KO_AND_BIM_KO_KL	35/324	200/20394	2.35E-26
4	GSE22886_NAIVE_TCELL_VS_DC_UP	27/324	199/20394	1.32E-17
4	GSE3720_UNSTIM_VS_LPS_STIM_VD2_GAMMAD	24/324	167/20394	2.31E-16
4	GSE3720_UNSTIM_VS_PMA_STIM_VD2_GAMMAD	23/324	175/20394	7.24E-15
4	GSE21927_SPLEEN_C57BL6_VS_4T1_TUMOR_BA	22/324	200/20394	1.14E-12
4	GSE34205_HEALTHY_VS_FLU_INF_INFANT_PBM	19/324	198/20394	4.36E-10
4	GSE26156_DOUBLE_POSITIVE_VS_CD4_SINGLE_	19/324	199/20394	4.75E-10
4	GSE22886_NAIVE_TCELL_VS_NKCELL_UP	17/324	198/20394	1.97E-08
4	GSE3982_CENT_MEMORY_CD4_TCELL_VS_NKC	17/324	198/20394	1.97E-08
5	GSE29618_MONOCYTE_VS_PDC_UP	27/149	199/20394	1.08E-26

5	GSE29618_BCELL_VS_MDC_DN	26/149	200/20394	3.14E-25
5	GSE29618_PDC_VS_MDC_DN	26/149	200/20394	3.14E-25
5	GSE10325_LUPUS_BCELL_VS_LUPUS_MYELOID_	24/149	200/20394	1.73E-22
5	GSE22886_NAIVE_BCELL_VS_MONOCYTE_DN	24/149	200/20394	1.73E-22
5	GSE22886_NAIVE_CD4_TCELL_VS_MONOCYTE_	21/149	200/20394	1.48E-18
5	GSE10325_LUPUS_CD4_TCELL_VS_LUPUS_MYE	20/149	200/20394	2.70E-17
5	GSE22886_NAIVE_CD8_TCELL_VS_MONOCYTE_	20/149	200/20394	2.70E-17
5	GSE22886_NAIVE_TCELL_VS_MONOCYTE_DN	19/149	200/20394	4.62E-16
6	GSE27241_WT_VS_RORGT_KO_TH17_POLARIZE	49/1112	180/20394	1.77E-21
6	GSE7509_FCGRIIB_VS_TNFA_IL1B_IL6_PGE_STI	39/1112	154/20394	3.37E-16
6	GSE7509_UNSTIM_VS_FCGRIIB_STIM_MONOCY	39/1112	166/20394	5.06E-15
6	GSE29617_DAY3_VS_DAY7_TIV_FLU_VACCINE_	41/1112	191/20394	2.76E-14
6	GSE18791_UNSTIM_VS_NEWCATSLE_VIRUS_DC	39/1112	181/20394	1.03E-13
6	GSE25146_UNSTIM_VS_HELIOBACTER_PYLORI_	38/1112	180/20394	4.22E-13
6	GSE22886_NAIVE_CD4_TCELL_VS_12H_ACT_TH	38/1112	199/20394	1.08E-11
6	GSE23502_BM_VS_COLON_TUMOR_MYELOID_D	37/1112	200/20394	5.29E-11
6	GSE21670_UNTREATED_VS_TGFB_IL6_TREATED	36/1112	194/20394	8.80E-11
7	GSE25146_UNSTIM_VS_HELIOBACTER_PYLORI_	42/850	165/20394	9.86E-22
7	GSE10325_LUPUS_CD4_TCELL_VS_LUPUS_MYE	44/850	200/20394	5.27E-20
7	GSE24634_TREG_VS_TCONV_POST_DAY7_IL4_C	43/850	198/20394	2.38E-19
7	GSE10325_LUPUS_BCELL_VS_LUPUS_MYELOID_	43/850	200/20394	3.57E-19
7	GSE2770_TGFB_AND_IL4_VS_IL12_TREATED_AC	37/850	200/20394	1.78E-14
7	GSE7509_UNSTIM_VS_FCGRIIB_STIM_MONOCY	33/850	166/20394	5.53E-14
8	GSE2405_0H_VS_9H_A_PHAGOCYTOPHILUM_ST	61/1301	200/20394	9.44E-26
8	GSE1460_INTRATHYMIC_T_PROGENITOR_VS_N	58/1301	199/20394	1.98E-23
8	GSE22886_UNSTIM_VS_IL15_STIM_NKCELL_DN	57/1301	200/20394	1.62E-22
8	GSE36476_CTRL_VS_TSST_ACT_72H_MEMORY_	55/1301	200/20394	5.81E-21
8	GSE14415_NATURAL_TREG_VS_TCONV_DN	51/1301	180/20394	3.70E-20
9	GSE21927_SPLEEN_C57BL6_VS_EL4_TUMOR_B	34/971	200/20394	9.43E-11
9	GSE27786_NKCELL_VS_MONO_MAC_UP	33/971	200/20394	3.99E-10
9	GSE29618_MONOCYTE_VS_PDC_DAY7_FLU_VA	31/971	199/20394	5.64E-09
9	GSE22886_NEUTROPHIL_VS_DC_DN	31/971	200/20394	6.38E-09
9	GSE3982_EOSINOPHIL_VS_MAST_CELL_DN	30/971	198/20394	1.91E-08
9	GSE32986_UNSTIM_VS_GMCSF_STIM_DC_DN	30/971	199/20394	2.14E-08
10	GSE21927_C26GM_VS_4T1_TUMOR_MONOCYTE	14/186	199/20394	4.25E-09
10	GSE7509_UNSTIM_VS_FCGRIIB_STIM_MONOCY	11/186	166/20394	3.73E-07
10	GSE40274_FOXP3_VS_FOXP3_AND_GATA1_TRA	11/186	200/20394	2.35E-06
10	GSE7460_WT_VS_FOXP3_HET_ACT_WITH_TGFB	10/186	200/20394	1.57E-05

p.adjust	qvalue	geneID	Count
2.10E-37	1.06E-37	6282/822/3956/302/6281/6275/3958/2	53
6.02E-34	3.03E-34	1476/3956/302/6281/6275/3958/597/3	50
6.93E-33	3.49E-33	6282/822/3958/2512/6386/6888/526/2	49
9.99E-31	5.03E-31	6282/6648/2512/6386/5341/526/2745/	47
1.20E-29	6.06E-30	6282/822/302/3958/2512/6386/7305/6	46
1.44E-28	7.23E-29	6282/822/302/3958/2512/6386/6888/2	45
1.35E-27	6.78E-28	6282/6648/6281/6275/3958/597/2512/	44
1.73E-25	8.71E-26	1476/6648/586/2495/6888/123/7295/1	42
6.94E-16	3.50E-16	2171/28984/6386/2495/2745/8566/308	32
3.79E-14	1.91E-14	1476/28984/3958/586/2495/80896/103	30
3.95E-34	2.91E-34	514/1622/10975/1340/9551/27089/297	58
2.01E-32	1.48E-32	1340/51142/6147/6231/6142/6155/510	56
2.70E-23	1.98E-23	10975/475/9551/51142/6147/6231/503	47
1.09E-18	8.04E-19	6635/3281/90480/27089/5425/7388/14	42
5.96E-18	4.38E-18	79017/5425/7388/10399/54976/894/13	41
6.22E-18	4.57E-18	10975/54543/4708/4701/23480/26521/	41
4.20E-17	3.09E-17	9040/79006/6611/51065/4830/7388/10	40
7.63E-15	5.61E-15	6717/475/6147/3281/29796/4701/2348	37
1.04E-12	7.63E-13	6635/475/7167/79017/51142/7388/140	34
1.04E-12	7.63E-13	1622/475/3281/8655/4701/4830/3939/	34
2.49E-18	1.83E-18	6223/6147/6635/6202/6187/6175/6204	35
1.43E-17	1.05E-17	51142/6613/4697/23406/11065/6637/3	37
1.43E-17	1.05E-17	3925/65108/27338/1622/8815/3148/70	37
1.43E-17	1.05E-17	10376/51142/203068/1345/25804/116	37
1.43E-17	1.05E-17	65108/27338/23406/23480/1622/2079/	37
1.08E-16	7.95E-17	10376/27338/6628/6637/1163/6119/59	36
1.08E-16	7.95E-17	65108/27338/5757/2079/6181/8815/86	36
5.38E-15	3.96E-15	3146/27338/6147/23480/6637/6161/10	34
3.30E-14	2.42E-14	6210/6189/1345/6128/6635/23480/615	33
3.47E-14	2.55E-14	3151/27338/801/1622/2079/4521/5902	32
1.19E-72	6.39E-73	1508/2896/2517/1522/1514/3988/713/	82
3.49E-42	1.87E-42	1509/1520/1508/3106/2350/2517/1514	59
1.89E-36	1.02E-36	1509/1520/1508/1675/3106/1514/9445	54
4.27E-32	2.29E-32	1520/1508/2896/4069/29992/1512/880	50
8.81E-27	4.73E-27	29992/427/84034/3109/51296/3916/71	45
6.25E-26	3.36E-26	1508/5641/1075/9375/1200/11006/348	44
6.97E-20	3.74E-20	1520/2517/2213/1512/334/1075/929/5	38
5.85E-19	3.14E-19	3123/968/8804/3117/54/427/79887/44	37
6.19E-13	3.32E-13	2213/1512/718/10154/5329/3916/3115	30
6.19E-13	3.32E-13	3123/972/2517/1512/5547/718/8832/2	30
3.79E-35	3.25E-35	6415/1267/6414/71/6136/6194/6141/6	45
3.72E-23	3.18E-23	71/6136/6194/6141/10399/6202/6122/	35
1.25E-14	1.07E-14	6130/6122/6125/6188/1933/6228/6154	27
1.83E-13	1.57E-13	51660/6136/5305/6141/6202/6122/612	24
4.38E-12	3.75E-12	1915/6136/6141/9448/6122/6188/1933	23
4.50E-10	3.86E-10	71/6141/6130/6324/6138/6202/6122/6	22
1.25E-07	1.07E-07	6141/6130/10399/6138/6125/6188/193	19
1.25E-07	1.07E-07	10983/4118/6202/6228/6134/6206/615	19
3.59E-06	3.07E-06	4118/58473/9452/10541/6194/64077/2	17
3.59E-06	3.07E-06	4747/6136/6130/3800/23446/6125/736	17
4.55E-23	3.56E-23	397/597/6688/6280/4688/301/9535/79	27

4.43E-22	3.46E-22	1601/3606/6001/2745/4046/9535/2910	26
4.43E-22	3.46E-22	397/597/6688/6280/4688/301/6001/953	26
1.46E-19	1.14E-19	4688/409/199/2512/7940/6275/50865/8	24
1.46E-19	1.14E-19	6280/4688/301/2745/409/2512/6275/80	24
1.04E-15	8.17E-16	6688/6280/4688/2745/2512/23643/109	21
1.27E-14	9.92E-15	6280/4688/10409/199/3108/2512/7940	20
1.27E-14	9.92E-15	6280/4688/6622/2512/80896/23643/10	20
1.78E-13	1.39E-13	6280/4688/2745/2512/80896/23643/10	19
8.63E-18	7.26E-18	55636/58508/284058/23318/23499/218	49
8.21E-13	6.91E-13	378938/7503/81669/51747/55692/667/	39
6.16E-12	5.19E-12	7503/80205/81669/6651/9988/10443/2	39
2.69E-11	2.26E-11	55082/81669/55553/100129387/11326	41
8.40E-11	7.07E-11	378938/283131/10180/3187/29896/585	39
2.94E-10	2.47E-10	6431/3187/3192/3070/79915/22985/79	38
6.57E-09	5.53E-09	25957/4820/9988/28951/7644/23353/1	38
2.87E-08	2.41E-08	9584/10521/1848/8899/57035/64770/2	37
4.29E-08	3.61E-08	10147/51747/5101/55608/55367/4297/	36
4.79E-18	2.95E-18	25957/4820/6574/57018/3187/196441/	42
1.28E-16	7.90E-17	7127/3687/83937/23312/4671/55577/2	44
3.47E-16	2.14E-16	10180/6303/9765/55704/10161/145389	43
3.47E-16	2.14E-16	7127/83937/23312/6303/10161/23129/	43
5.41E-12	3.33E-12	10161/57674/8567/57018/54103/23360	37
1.58E-11	9.75E-12	9765/7503/10312/10443/6651/23499/1	33
4.60E-22	2.91E-22	4176/9618/9768/8106/90007/200916/8	61
4.82E-20	3.05E-20	6938/6659/5955/3066/3276/9805/1171	58
2.64E-19	1.67E-19	4176/4678/6713/3066/79174/7873/273	57
7.07E-18	4.48E-18	4176/3149/7298/9768/8836/1719/1404	55
3.60E-17	2.28E-17	4176/4603/813/254263/60559/4001/10	51
4.59E-07	3.32E-07	9601/54969/25843/79174/29923/8727/	34
9.70E-07	7.02E-07	816/9855/57153/4784/245812/10152/1	33
4.44E-06	3.21E-06	2273/9601/4130/9618/58516/9500/659	31
4.44E-06	3.21E-06	23014/22883/6856/8065/55153/9255/1	31
1.16E-05	8.40E-06	6659/55326/2766/4212/10329/324/925	30
1.16E-05	8.40E-06	221981/58489/10787/55798/488/388/7	30
1.79E-05	1.68E-05	23499/26118/1739/285527/57035/4820	14
0.00078	0.00074	23499/7503/23187/9765/84251/10443/	11
0.00329	0.00310	88/158471/4147/114823/131096/9781/	11
0.01101	0.01036	1739/9639/22993/85461/9205/26137/5	10

Cluster	ID	GeneRatio	BgRatio	pvalue	p.adjust	qvalue
0	ELF1_Q6	22/477	249/26190	1.31E-09	3.23E-06	2.66E-06
0	MIR3714	29/477	434/26190	2.30E-09	3.23E-06	2.66E-06
0	TCANNTGAY_SREBP1_01	29/477	479/26190	2.07E-08	1.71E-05	1.40E-05
0	AP1_Q6_01	21/477	267/26190	2.42E-08	1.71E-05	1.40E-05
0	PSMB5_TARGET_GENES	22/477	304/26190	5.00E-08	2.82E-05	2.32E-05
0	MIR3910	24/477	403/26190	4.66E-07	0.000219	0.0001804
1	FOXR2_TARGET_GENES	30/695	231/26190	7.95E-13	1.70E-09	1.63E-09
1	PSMB5_TARGET_GENES	34/695	304/26190	1.81E-12	1.70E-09	1.63E-09
1	NR1I2_TARGET_GENES	32/695	340/26190	7.18E-10	5.05E-07	4.82E-07
1	TTCYRGAA_UNKNOWN	29/695	340/26190	4.04E-08	1.89E-05	1.81E-05
1	RFX7_TARGET_GENES	36/695	492/26190	5.10E-08	2.05E-05	1.96E-05
1	ATF5_TARGET_GENES	34/695	456/26190	7.66E-08	2.51E-05	2.40E-05
1	NPM1_TARGET_GENES	29/695	352/26190	8.56E-08	2.51E-05	2.40E-05
1	NRF2_01	25/695	273/26190	8.92E-08	2.51E-05	2.40E-05
1	ELK1_02	24/695	256/26190	1.04E-07	2.65E-05	2.54E-05
1	ZNF830_TARGET_GENES	25/695	279/26190	1.36E-07	3.18E-05	3.04E-05
1	DMRT1_TARGET_GENES	31/695	406/26190	1.70E-07	3.68E-05	3.52E-05
1	ALKBH3_TARGET_GENES	32/695	433/26190	2.26E-07	4.53E-05	4.33E-05
2	PSMB5_TARGET_GENES	48/581	304/26190	7.79E-27	2.17E-23	1.95E-23
2	FOXR2_TARGET_GENES	31/581	231/26190	1.08E-15	1.22E-12	1.09E-12
2	E2F_03	32/581	249/26190	1.31E-15	1.22E-12	1.09E-12
2	E2F_Q4_01	31/581	238/26190	2.51E-15	1.75E-12	1.57E-12
2	NPM1_TARGET_GENES	34/581	352/26190	7.97E-13	2.78E-10	2.50E-10
2	GABP_B	28/581	265/26190	1.02E-11	3.16E-09	2.84E-09
2	E2F1_Q6	26/581	235/26190	2.00E-11	5.27E-09	4.74E-09
2	NRF2_01	28/581	273/26190	2.08E-11	5.27E-09	4.74E-09
3	ZNF354B_TARGET_GENES	21/541	201/26190	1.39E-09	3.77E-06	3.54E-06
3	MAML1_TARGET_GENES	20/541	309/26190	7.82E-06	0.0080248	0.0075456
3	IRF_Q6	17/541	244/26190	1.45E-05	0.0085636	0.0080522
3	MIR6778_3P	16/541	222/26190	1.69E-05	0.0085636	0.0080522
3	MIR205_5P	16/541	224/26190	1.89E-05	0.0085636	0.0080522
3	MIR4782_5P	13/541	163/26190	3.63E-05	0.0113749	0.0106955
3	MIR5706	13/541	163/26190	3.63E-05	0.0113749	0.0106955
4	PSMB5_TARGET_GENES	24/324	304/26190	7.68E-13	1.98E-09	1.67E-09
4	GTF2E2_TARGET_GENES	21/324	409/26190	4.91E-08	6.31E-05	5.34E-05
4	MAX_01	14/324	266/26190	6.53E-06	0.0056	0.0047
4	CATTGTYY_SOX9_B1	16/324	368/26190	1.61E-05	0.0090	0.0076
4	TGACCTTG_SF1_Q6	13/324	253/26190	1.80E-05	0.0090	0.0076
4	AAGCCAT_MIR135A_MIR135B	15/324	335/26190	2.10E-05	0.0090	0.0076
4	FOXR2_TARGET_GENES	12/324	231/26190	3.39E-05	0.0116	0.0098
5	PSMB5_TARGET_GENES	11/152	304/26190	1.75E-06	0.00239	0.00239
5	GTF2E2_TARGET_GENES	11/152	409/26190	2.85E-05	0.01952	0.01952
5	NKX2_8_TARGET_GENES	4/152	39/26190	7.66E-05	0.03497	0.03497
6	TGCACTT_MIR519C_MIR519B_MIR5	74/1126	449/26190	1.54E-23	4.75E-20	1.57E-20
6	TGAATGT_MIR181A_MIR181B_MIR1	75/1126	488/26190	5.94E-22	9.15E-19	3.03E-19

6	E2F_Q6	50/1126	235/26190	3.34E-21	3.30E-18	1.09E-18
6	MIR300	70/1126	449/26190	6.42E-21	3.30E-18	1.09E-18
6	MIR381_3P	70/1126	449/26190	6.42E-21	3.30E-18	1.09E-18
6	MIR1283	72/1126	477/26190	1.17E-20	5.14E-18	1.70E-18
6	MIR3065_5P	69/1126	466/26190	2.22E-19	8.54E-17	2.82E-17
6	MIR101_3P	68/1126	459/26190	3.95E-19	1.23E-16	4.08E-17
6	MIR4495	70/1126	483/26190	4.00E-19	1.23E-16	4.08E-17
7	TGAATGT_MIR181A_MIR181B_MIR1	54/867	488/26190	9.45E-15	2.15E-11	1.32E-11
7	MIR32_5P	53/867	477/26190	1.42E-14	2.15E-11	1.32E-11
7	MIR92B_3P	52/867	474/26190	4.24E-14	4.13E-11	2.53E-11
7	MIR92A_3P	52/867	477/26190	5.45E-14	4.13E-11	2.53E-11
7	MIR367_3P	47/867	426/26190	5.90E-13	3.58E-10	2.19E-10
7	MIR363_3P	46/867	425/26190	2.06E-12	9.66E-10	5.92E-10
7	MIR548AS_3P	35/867	261/26190	2.23E-12	9.66E-10	5.92E-10
7	MIR25_3P	44/867	420/26190	1.86E-11	7.06E-09	4.32E-09
8	E2F_Q6	63/1307	235/26190	7.28E-29	2.25E-25	8.65E-26
8	E2F_Q4	62/1307	237/26190	8.81E-28	1.36E-24	5.23E-25
8	E2F1_Q6	60/1307	235/26190	2.60E-26	2.67E-23	1.03E-23
8	E2F_02	60/1307	237/26190	4.23E-26	3.27E-23	1.26E-23
8	E2F1DP1_01	59/1307	237/26190	2.84E-25	1.25E-22	4.81E-23
8	E2F1DP2_01	59/1307	237/26190	2.84E-25	1.25E-22	4.81E-23
8	E2F4DP2_01	59/1307	237/26190	2.84E-25	1.25E-22	4.81E-23
8	E2F4DP1_01	58/1307	241/26190	4.64E-24	1.79E-21	6.89E-22
8	E2F1_Q6_01	58/1307	243/26190	7.28E-24	2.50E-21	9.61E-22
8	E2F_Q6_01	57/1307	242/26190	3.62E-23	1.12E-20	4.30E-21
9	ACATTCC_MIR1_MIR206	41/979	301/26190	8.87E-13	2.71E-09	1.38E-09
9	CTTTGTA_MIR524	50/979	434/26190	1.90E-12	2.89E-09	1.47E-09
9	ATF3_Q6	34/979	253/26190	1.14E-10	1.16E-07	5.89E-08
9	MIR3065_5P	48/979	466/26190	2.66E-10	2.03E-07	1.03E-07
9	PXR_Q2	33/979	260/26190	9.57E-10	5.84E-07	2.97E-07
9	NR1I2_TARGET_GENES	38/979	340/26190	2.06E-09	9.91E-07	5.05E-07
9	MIR561_3P	48/979	497/26190	2.27E-09	9.91E-07	5.05E-07
9	ACACTAC_MIR1423P	22/979	130/26190	2.95E-09	1.13E-06	5.73E-07
9	SPZ1_01	30/979	235/26190	4.80E-09	1.47E-06	7.48E-07
9	MIR613	36/979	321/26190	4.99E-09	1.47E-06	7.48E-07
9	CREBP1_01	26/979	183/26190	5.33E-09	1.47E-06	7.48E-07
9	TST1_01	32/979	265/26190	5.92E-09	1.47E-06	7.48E-07
10	MIR92B_3P	17/187	474/26190	5.96E-08	3.37E-05	2.13E-05
10	MIR32_5P	17/187	477/26190	6.53E-08	3.37E-05	2.13E-05
10	MIR92A_3P	17/187	477/26190	6.53E-08	3.37E-05	2.13E-05
10	MIR25_3P	16/187	420/26190	6.54E-08	3.37E-05	2.13E-05
10	MIR363_3P	16/187	425/26190	7.69E-08	3.37E-05	2.13E-05
10	MIR367_3P	16/187	426/26190	7.94E-08	3.37E-05	2.13E-05
10	MIR3666	15/187	398/26190	1.96E-07	7.14E-05	4.52E-05
10	MIR130B_3P	15/187	404/26190	2.38E-07	7.57E-05	4.79E-05
10	SOX10_TARGET_GENES	12/187	301/26190	1.90E-06	0.00017	0.00011

10	HNF1_C	10/187	249/26190	1.31E-05	0.00061	0.00038
10	SMAD3_Q6	9/187	242/26190	6.45E-05	0.00177	0.00112
10	OCT1_04	9/187	243/26190	6.66E-05	0.00180	0.00114

geneID	Count
7305/397/1192/3987/10109/2207/2698	22
9516/3936/10493/311/10092/8566/308	29
526/3936/10092/51382/523/528/84418	29
8878/6386/10493/29887/523/7763/837	21
3956/2512/5052/2495/6277/9168/2698	22
3936/311/308/51123/8661/5898/8992/1	24
2280/6155/28974/6181/6217/6222/103	30
26472/2597/54205/6234/7167/5441/80	34
9040/54543/51065/4830/5478/23741/8	32
3336/8655/6222/3329/521/9588/27166	29
389541/6222/6171/51024/4738/1029/6	36
6717/3336/7388/26010/3329/6742/550	34
6635/4713/5478/51024/10273/6223/13	29
6155/7388/6222/10467/11007/26521/1	25
6611/7388/140465/6222/26521/1351/1	24
6635/2597/4708/4713/3939/7431/5102	25
6717/4697/5478/10399/154791/6171/6	31
51142/801/4830/6222/6742/51398/659	32
10376/3925/7846/27338/801/5757/618	48
6193/6128/6227/6222/2079/6217/6181	31
3151/3925/65108/3178/5757/9768/259	32
3151/3925/3178/6223/5757/9768/2597	31
6223/5478/6189/6635/7979/6202/4736	34
23658/6161/8815/5094/5902/60/9158/5	28
3925/9768/2597/11335/5902/4150/663	26
6193/6128/6222/1163/6204/6159/6155	28
1509/2896/5476/6948/3916/5538/1200	21
1522/963/1535/3109/951/57121/22000	20
567/2213/10154/51296/3107/684/6892	17
1508/5724/3916/3140/728/1777/481/64	16
10313/9375/4853/4035/10184/2180/80	16
6646/5027/56889/8763/55824/56851/6	13
6646/5027/56889/8763/55824/56851/6	13
1915/5757/6271/10209/71/6136/6122/4	24
1915/5757/65108/71/6136/6194/10399	21
5757/1995/4869/1933/90701/666/5202	14
4155/4638/5757/6271/4131/10971/944	16
760/4118/10382/4131/1995/10231/734	13
2824/6695/222166/23446/57509/292/9	15
1915/6194/10399/127687/6232/6230/7	12
2512/84790/2495/3956/6277/7114/505	11
301/2512/84790/10327/10627/2495/49	11
9270/4707/808/302	4
80205/57035/4850/10643/4176/23236/	74
80205/4857/2891/26118/55553/55636/	75

51747/10521/8899/4678/4176/2893/14	50
26053/25957/2521/4820/94104/1268/4	70
26053/25957/2521/4820/94104/1268/4	70
5803/120114/6651/55692/6925/6431/1	72
26118/55553/6651/6925/5101/9534/92	69
4857/120114/5980/8899/113263/9223/	68
9678/51678/80055/7514/58508/3696/2	70
26118/57035/23015/440270/1316/1062	54
25957/23015/1130/1657/25943/2803/1	53
25957/23015/1130/1657/25943/2803/1	52
25957/23015/1130/1657/25943/2803/1	52
25957/23015/1130/1657/25943/2803/1	47
25957/1130/1657/25943/2803/196441/	46
55704/25957/5027/9043/4703/94101/5	35
25957/1130/1657/25943/2803/196441/	44
4176/2893/1501/3642/4678/9768/7112	63
4176/2893/1501/3642/4678/9768/7112	62
4176/2893/1501/4678/9768/7112/4628	60
4176/2893/5178/4678/9768/7112/4628	60
4176/2893/5178/4678/9768/7112/4628	59
4176/2893/5178/4678/9768/7112/4628	59
4176/2893/5178/4678/9768/7112/4628	59
4176/2893/5178/4678/9768/7112/4628	58
4176/1501/3642/4678/6935/7112/4628	58
4176/2893/1501/283248/5178/1848/46	57
9590/5218/9586/56262/51157/4130/11	41
7026/1400/6664/2823/81848/5396/220	50
1114/10215/79586/2669/9586/54704/8	34
219287/1282/2823/10098/23635/7088/	48
23632/2118/5396/254263/5607/6464/2	33
4232/196410/1466/399694/4784/1717/	38
6382/7026/5218/131566/22871/5087/8	48
6664/25843/488/406/54874/55729/514	22
56999/7026/1400/1271/23635/81848/5	30
59269/5218/5087/9586/6662/56262/11	36
59269/2118/2823/81848/23208/26511/	26
1525/7026/5218/2823/10215/6252/560	32
80005/83992/5010/344148/253559/593	17
80005/83992/5010/344148/253559/593	17
80005/83992/5010/344148/253559/593	17
83992/5010/344148/253559/59338/578	16
83992/5010/344148/253559/59338/578	16
83992/5010/344148/253559/59338/578	16
57584/4036/158471/10690/11113/3441	15
57584/4036/158471/10690/11113/3441	15
57475/28513/83992/9781/81671/288/1	12

56899/51747/23187/344148/4684/1756	10
56899/4036/130271/88/60484/51747/8	9
51747/160335/253559/4684/5789/1756	9

Cluster	ID	Description	GeneRatio	BgRatio
0	R-HSA-6798695	Neutrophil degranulation	64/349	480/10704
0	R-HSA-9664422	FCGR3A-mediated phagocytosis	19/349	59/10704
0	R-HSA-5663213	RHO GTPases Activate WASPs and W	14/349	36/10704
0	R-HSA-2029480	Fcgamma receptor (FCGR) dependent	20/349	86/10704
0	R-HSA-8950505	Gene and protein expression by JAK-S	14/349	38/10704
0	R-HSA-9020591	Interleukin-12 signaling	15/349	47/10704
0	R-HSA-194315	Signaling by Rho GTPases	43/349	455/10704
0	R-HSA-76002	Platelet activation, signaling and aggre	31/349	263/10704
0	R-HSA-354192	Integrin signaling	5/349	27/10704
0	R-HSA-6802957	Oncogenic MAPK signaling	9/349	84/10704
1	R-HSA-72766	Translation	81/488	291/10704
1	R-HSA-163200	Respiratory electron transport, ATP syn	56/488	125/10704
1	R-HSA-1428517	The citric acid (TCA) cycle and respirat	62/488	176/10704
1	R-HSA-611105	Respiratory electron transport	47/488	101/10704
1	R-HSA-5628897	TP53 Regulates Metabolic Genes	24/488	87/10704
2	R-HSA-376176	Signaling by ROBO receptors	90/434	218/10704
2	R-HSA-69242	S Phase	31/434	162/10704
2	R-HSA-187577	SCF(Skp2)-mediated degradation of p2	19/434	60/10704
2	R-HSA-453279	Mitotic G1 phase and G1/S transition	29/434	149/10704
2	R-HSA-9604323	Negative regulation of NOTCH4 signali	18/434	54/10704
2	R-HSA-69202	Cyclin E associated events during G1/S	21/434	83/10704
2	R-HSA-69206	G1/S Transition	26/434	131/10704
2	R-HSA-9020702	Interleukin-1 signaling	20/434	103/10704
3	R-HSA-6798695	Neutrophil degranulation	105/404	480/10704
3	R-HSA-198933	Immunoregulatory interactions betweer	30/404	132/10704
3	R-HSA-202733	Cell surface interactions at the vascula	28/404	137/10704
3	R-HSA-1660662	Glycosphingolipid metabolism	17/404	46/10704
3	R-HSA-1474244	Extracellular matrix organization	38/404	301/10704
3	R-HSA-8957275	Post-translational protein phosphorylati	21/404	108/10704
3	R-HSA-1236975	Antigen processing-Cross presentation	19/404	98/10704
3	R-HSA-2132295	MHC class II antigen presentation	21/404	123/10704
3	R-HSA-202427	Phosphorylation of CD3 and TCR zeta	9/404	22/10704
4	R-HSA-9010553	Regulation of expression of SLITs and	45/204	171/10704
4	R-HSA-3371511	HSF1 activation	4/204	12/10704
4	R-HSA-3371497	HSP90 chaperone cycle for steroid hor	7/204	55/10704
4	R-HSA-195258	RHO GTPase Effectors	17/204	327/10704
4	R-HSA-2132295	MHC class II antigen presentation	9/204	123/10704
5	R-HSA-6798695	Neutrophil degranulation	20/108	480/10704
5	R-HSA-9660826	Purinergic signaling in leishmaniasis in	4/108	24/10704
5	R-HSA-9664424	Cell recruitment (pro-inflammatory resp	4/108	24/10704
5	R-HSA-844456	The NLRP3 inflammasome	3/108	15/10704
6	R-HSA-3247509	Chromatin modifying enzymes	38/641	274/10704
6	R-HSA-4839726	Chromatin organization	38/641	274/10704
6	R-HSA-68877	Mitotic Prometaphase	30/641	204/10704
7	R-HSA-194840	Rho GTPase cycle	20/492	138/10704

7	R-HSA-6798695	Neutrophil degranulation	41/492	480/10704
7	R-HSA-983712	Ion channel transport	21/492	186/10704
8	R-HSA-72172	mRNA Splicing	57/832	191/10704
8	R-HSA-72706	GTP hydrolysis and joining of the 60S r	34/832	112/10704
8	R-HSA-453279	Mitotic G1 phase and G1/S transition	39/832	149/10704
8	R-HSA-69206	G1/S Transition	35/832	131/10704
8	R-HSA-2555396	Mitotic Metaphase and Anaphase	50/832	237/10704
8	R-HSA-69620	Cell Cycle Checkpoints	56/832	294/10704
9	R-HSA-5368287	Mitochondrial translation	18/579	93/10704
9	R-HSA-373753	Nephrin family interactions	7/579	23/10704
9	R-HSA-8957275	Post-translational protein phosphorylati	16/579	108/10704
9	R-HSA-3700989	Transcriptional Regulation by TP53	36/579	365/10704
9	R-HSA-170834	Signaling by TGF-beta Receptor Comp	12/579	73/10704
10	R-HSA-1500931	Cell-Cell communication	7/93	129/10704

pvalue	p.adjust	qvalue	geneID	Count
8.39E-23	9.04E-20	7.28E-20	6282/1476/302/2171/3958/2512/6386/7	64
1.48E-14	3.99E-12	3.21E-12	10095/10109/10094/10092/10096/998/	19
2.35E-12	4.22E-10	3.39E-10	10095/10109/10094/10092/10096/998/	14
2.76E-12	4.25E-10	3.42E-10	10095/10109/10094/10092/10096/998/	20
5.64E-12	7.59E-10	6.11E-10	6648/302/6888/5908/3936/9446/4478/3	14
1.08E-11	1.29E-09	1.04E-09	6648/302/6888/5908/3936/9446/4478/3	15
2.45E-10	1.88E-08	1.52E-08	397/10095/6280/10627/808/10109/100	43
4.65E-10	3.34E-08	2.69E-08	5341/5908/11343/808/10487/2207/308	31
0.00160	0.01986	0.01599	5908/2885/7094/5906/6850	5
0.00160	0.01986	0.01599	5908/808/7094/4000/5906/8826/5594/9	9
2.23E-42	1.72E-39	1.24E-39	6234/6147/90480/6231/6142/6155/510	81
3.17E-42	1.72E-39	1.24E-39	514/513/10975/4697/1340/54205/9551	56
3.65E-39	1.32E-36	9.51E-37	514/513/10975/4697/1340/54205/9551	62
1.75E-36	4.75E-34	3.42E-34	10975/4697/1340/54205/4717/27089/4	47
4.39E-13	1.32E-11	9.52E-12	4697/1340/54205/7001/389541/1350/1	24
4.55E-69	2.34E-67	1.51E-67	6193/6209/6223/6142/6210/6189/6147	90
3.22E-13	9.19E-12	5.92E-12	27338/7979/11065/1163/6119/7311/56	31
1.06E-12	2.94E-11	1.90E-11	7979/1163/7311/5683/5689/1019/5694	19
1.22E-12	3.22E-11	2.08E-11	7298/7979/1163/6119/894/1021/7311/5	29
1.56E-12	4.00E-11	2.58E-11	7979/7311/5683/5689/5694/5714/9978	18
8.69E-12	2.07E-10	1.34E-10	7979/1163/7311/5683/5928/5689/1019	21
1.16E-11	2.58E-10	1.66E-10	7298/7979/1163/6119/7311/5683/5928	26
4.29E-09	4.31E-08	2.78E-08	3146/7979/7311/5683/5689/5694/5714	20
6.20E-53	3.81E-50	3.15E-50	1509/1520/1508/567/1675/3106/2896/2	105
8.98E-16	2.76E-13	2.28E-13	567/3106/29992/2213/975/126014/718	30
1.46E-13	3.00E-11	2.48E-11	972/3689/5175/140885/8832/3684/912	28
2.94E-13	4.51E-11	3.74E-11	3074/5660/427/5476/23344/3073/2760	17
4.55E-11	5.59E-09	4.63E-09	1509/1520/1508/1514/3689/5175/2/840	38
4.64E-10	4.74E-08	3.93E-08	348/1471/334/718/10618/4924/2519/11	21
3.34E-09	2.56E-07	2.12E-07	1520/567/3106/1514/929/1535/3105/31	19
5.53E-09	3.39E-07	2.81E-07	1509/3123/1520/972/1508/3119/1514/5	21
4.57E-08	2.01E-06	1.66E-06	3123/3119/920/3122/3113/3117/5788/3	9
1.57E-39	6.21E-38	5.40E-38	6136/6194/6141/6130/6229/6138/6202	45
5.62E-05	0.00136	0.00119	1915/7531/3326/3320	4
7.70E-05	0.00175	0.00153	10382/3306/1780/1783/3326/1781/332	7
0.00017	0.00375	0.00326	94274/4638/10382/10971/71/3799/178	17
0.00056	0.01173	0.01020	10382/3799/1780/1783/1211/375/1148	9
5.45E-08	2.56E-05	2.33E-05	6280/8673/9535/29108/2512/391/2657	20
8.91E-05	0.01397	0.01270	3606/29108/834/7295	4
8.91E-05	0.01397	0.01270	3606/29108/834/7295	4
0.00042	0.03924	0.03568	29108/834/7295	3
9.81E-07	0.00017	0.00016	55904/58508/284058/55870/64324/214	38
9.81E-07	0.00017	0.00016	55904/58508/284058/55870/64324/214	38
4.14E-06	0.00063	0.00058	7514/8481/84131/55835/10142/9735/5	30
4.68E-06	0.00447	0.00435	4650/116985/393/23092/2/221472/720	20

8.91E-05	0.03135	0.03055	3687/10312/1118/7226/1778/535/5788	41
0.00013	0.03135	0.03055	10312/55283/7226/374868/535/57192/	21
9.90E-20	6.12E-17	4.76E-17	6431/51593/8106/23350/10772/1660/6	57
1.66E-12	2.58E-10	2.01E-10	200916/8668/1973/51121/8666/7458/8	34
6.88E-12	7.79E-10	6.07E-10	1031/4176/7298/1021/10926/1719/186	39
4.47E-11	2.85E-09	2.22E-09	4176/7298/10926/1719/1869/7029/511	35
4.61E-11	2.85E-09	2.22E-09	7112/4001/347733/348235/8243/6494	50
2.08E-10	1.03E-08	7.99E-09	4176/57060/4194/580/10926/348235/6	56
1.96E-06	0.00096	0.00088	65005/63931/64949/51264/64983/618	18
0.00015	0.02195	0.02008	9863/2534/8573/81/8976/8826/6711	7
0.00021	0.02559	0.02340	51280/1114/7425/1000/3915/29106/78	16
0.00034	0.03604	0.03295	56950/6829/1616/143686/7832/23112/	36
0.00050	0.03721	0.03403	25937/5054/22938/56937/1030/25805/	12
0.00013	0.03723	0.03614	667/88/5010/9863/253559/961/4301	7

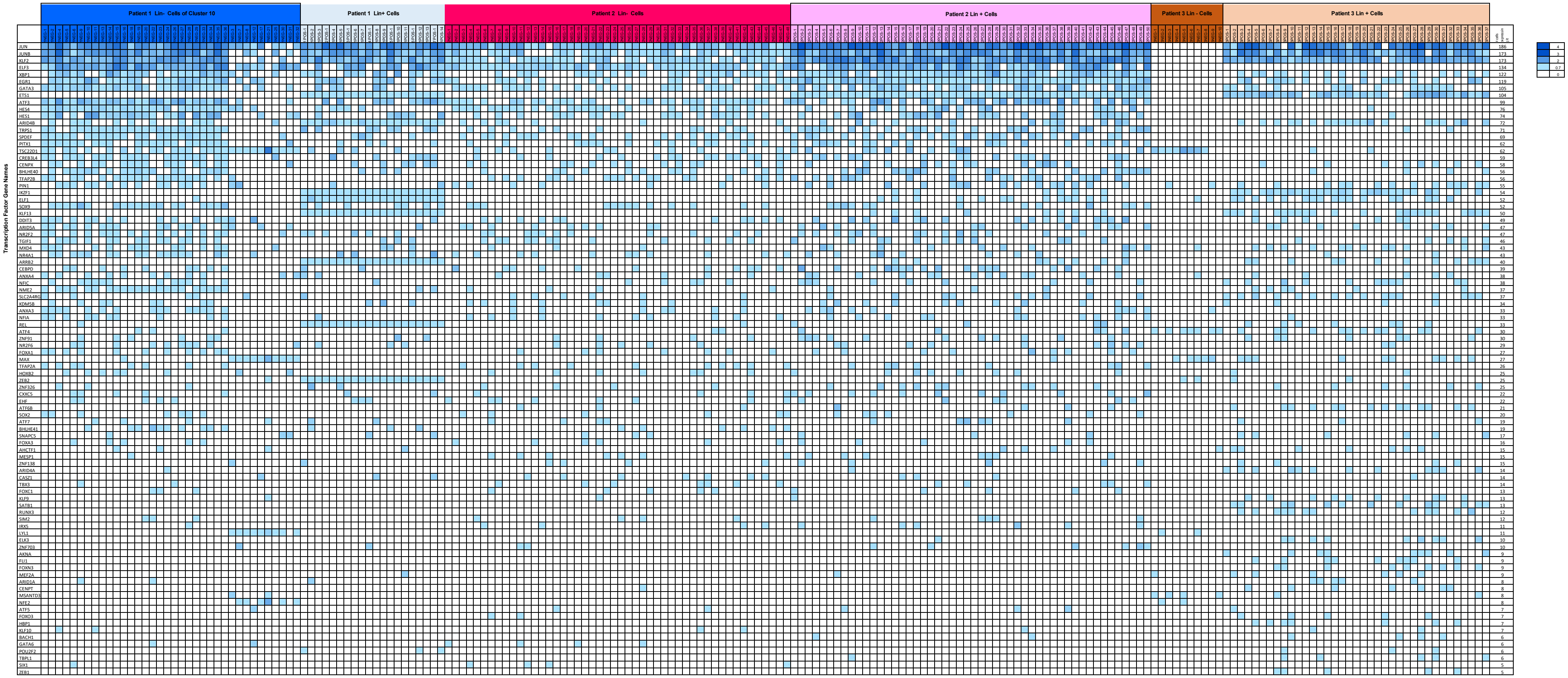
Patient 1 Lin- Cells of Cluster 10
Patient 1 Lin+ Cells
Patient 2 Lin- Cells
Patient 2 Lin + Cells
Patient 3 Lin - Cells
Patient 3 Lin + Cells
Transcription Factor Gene Names
cells expressing it
JUN 186
JUNB 173
KLF2 173
ELF3 134
XBP1 122
EGR1 119
GATA3 105
ETS1 104
ATF3 99
HES4 76
HES1 74
ARID4B 72
TRPS1 71
SPDEF 69
PITX1 62
TSC22D1 62
CREB3L4 59
CENPX 58
BHLHE40 56
TFAP2B 56
PIN1 55
IKZF1 54
ELF1 52
SOX9 52
KLF13 50
DDIT3 49
ARID5A 47
NR2F2 47
TGIF1 46
MXD4 43
NR4A1 43
ARRB2 40
CEBPD 39
ANXA4 38
NFIC 38
NME2 37
SLC2A4RG 37
KDM5B 34
ANXA3 33
NFIA 33
REL 33
ATF4 30
ZNF91 30
NR2F6 29
FOXA1 27
MAX 27
TFAP2A 26
HOXB2 25
ZEB2 25
ZNF326 25
CXXC5 22
EHF 22
ATF6B 21
SOX2 20
ATF7 19
BHLHE41 19
SNAPC5 17
FOXA3 16
AHCTF1 15
MESP1 15
ZNF138 15
ARID4A 14
CASZ1 14
TBX3 14
FOXC1 13
KLF9 13
SATB1 13
RUNX3 12
SIM2 12
IRX5 11
LYL1 11
ELK3 10
ZNF703 10
AKNA 9
FLI1 9
FOXN3 9
MEF2A 9
ARID1A 8
CENPT 8
MSANTD3 8
NFE2 8
ATF5 7
FOXO3 7
HBP1 7
KLF10 7
BACH1 6
GATA6 6
POU2F2 6
TBPL1 6
SIX1 5
ZEB1 5
4
3
2
0.7
0

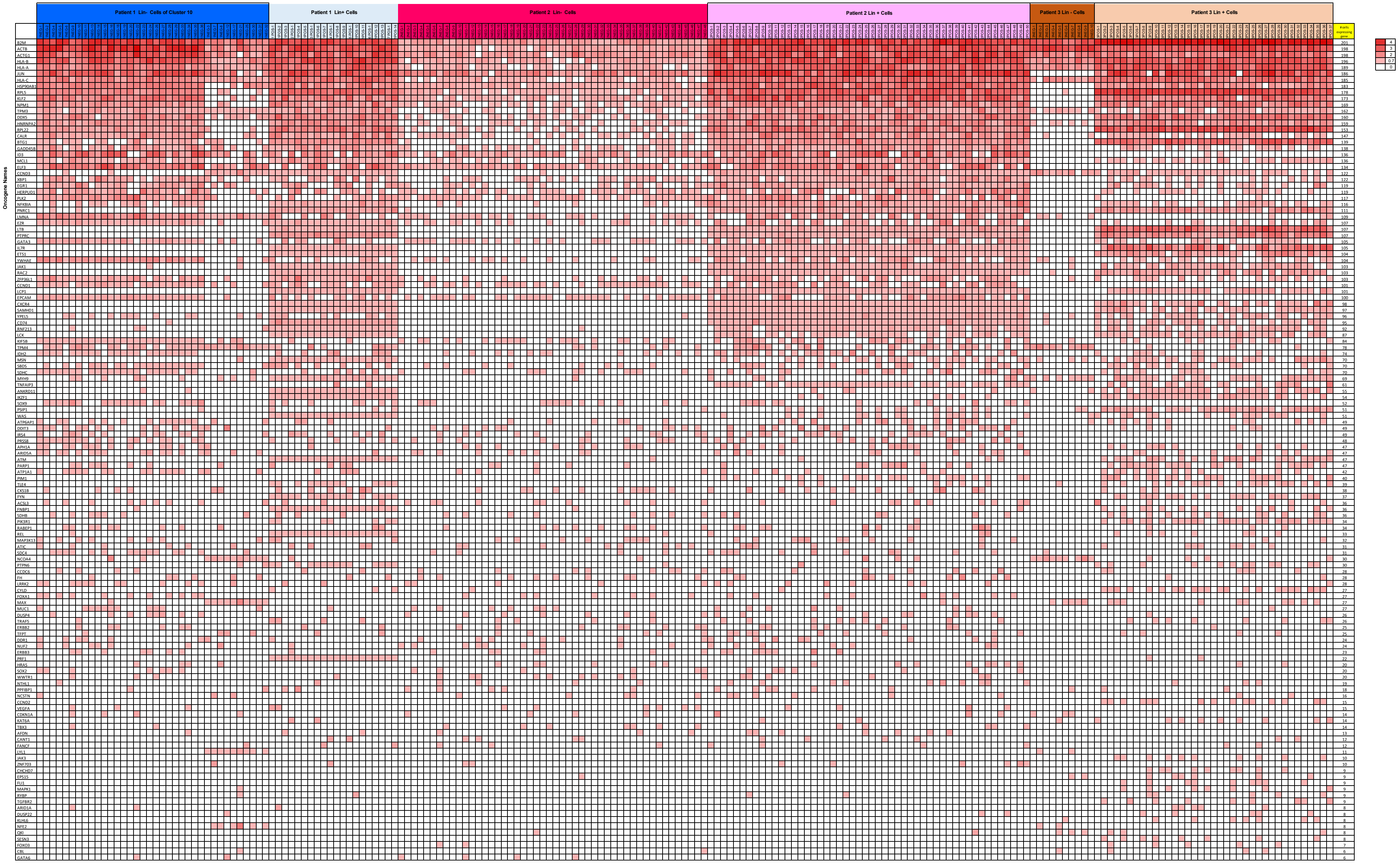

Patient 1 Lin- Cells of Cluster 10
Patient 1 Lin+ Cells
Patient 2 Lin- Cells
Patient 2 Lin + Cells
Patient 3 Lin - Cells
Patient 3 Lin + Cells
Oncogene Names
cells expressing gene
B2M 201
ACTB 198
ACTG1 198
HLA-B 196
HLA-A 189
JUN 186
HLA-C 185
HSP90AB1 183
RPL5 178
KLF2 173
NPM1 169
TPM3 162
DDX5 160
HNRNPA2 159
RPL22 153
CALR 147
BTG1 139
GADD45B 138
ID3 136
MCL1 136
ELF3 134
CCND3 122
XBP1 122
EGR1 119
HERPUD1 119
PLK2 117
NFKBIA 116
PNRC1 111
LMNA 109
EZR 107
LTB 107
PTPRC 107
GATA3 105
IL7R 105
ETS1 104
YWHAE 104
JAK1 103
RAC2 103
ZFP36L1 103
CCND1 101
LCP1 101
EPCAM 100
CXCR4 98
SAMHD1 97
YPEL5 96
CD74 95
RNF213 92
LCK 87
KIF5B 84
TPM4 78
IDH2 74
MSN 70
SBDS 70
SDHC 70
MYH9 69
TNFAIP3 61
ANKRD11 55
IKZF1 54
SOX9 52
PSIP1 51
WAS 51
ATP6AP1 49
DDIT3 49
IRS4 49
PRSS8 48
APH1A 47
ARID5A 47
ATM 47
PARP1 47
ATP1A1 42
PIM1 40
TLE4 39
CKS1B 38
FYN 37
ACSL3 36
FNBP1 36
SDHB 36
PIK3R1 34
RABEP1 34
REL 33
MAP3K13 32
ATIC 31
SDC4 31
NCOA4 30
PTPN6 30
CCDC6 28
FH 28
LRRK2 28
CYLD 27
FOXA1 27
MAX 27
MUC1 27
DUSP4 26
TRAF5 26
ERBB2 25
TFPT 25
DDR1 24
NUF2 24
ERBB3 23
PRF1 22
HRAS 20
SOX2 20
WWTR1 20
NTHL1 19
PPFIBP1 18
NCSTN 16
CCND2 15
VEGFA 15
CDKN1A 14
KAT6A 14
TBX3 14
AFDN 13
CANT1 12
FANCF 12
LYL1 11
JAK3 10
ZNF703 10
CHCHD7 9
EPS15 9
FLI1 9
MAPK1 9
RYBP 9
TGFBR2 9
ARID1A 8
DUSP22 8
KLHL6 8
NFE2 8
QKI 8
SESN3 8
FOXO3 7
CBL 6
GATA6 6
4
3
2
0.7
0

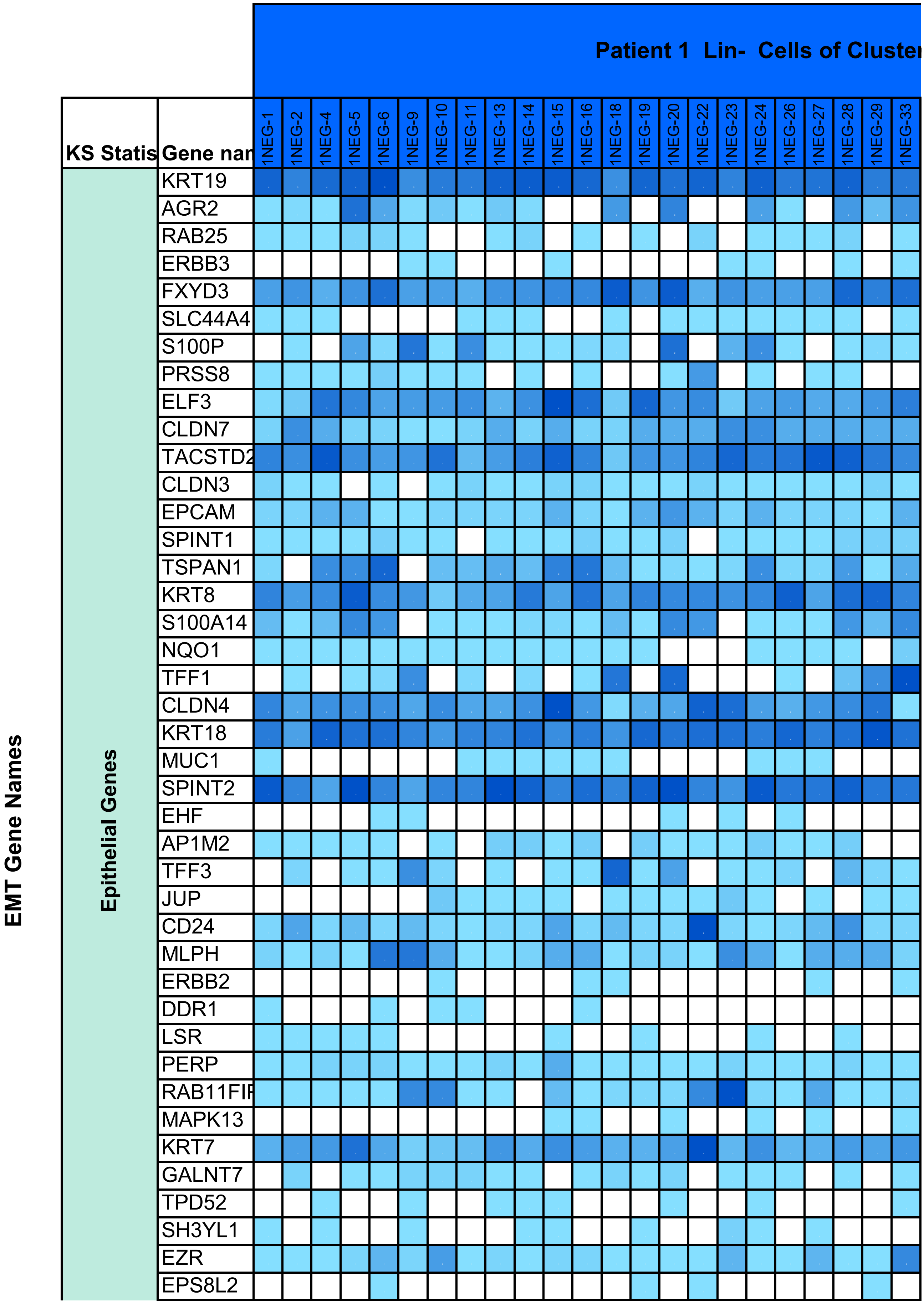

Patient 1 Lin- Cells of Cluster
KS Statis
Gene nar
1NEG-1
1NEG-2
1NEG-4
1NEG-5
1NEG-6
1NEG-9
1NEG-10
1NEG-11
1NEG-13
1NEG-14
1NEG-15
1NEG-16
1NEG-18
1NEG-19
1NEG-20
1NEG-22
1NEG-23
1NEG-24
1NEG-26
1NEG-27
1NEG-28
1NEG-29
1NEG-33
EMT Gene Names
Epithelial Genes
KRT19
AGR2
RAB25
ERBB3
FXYD3
SLC44A4
S100P
PRSS8
ELF3
CLDN7
TACSTD2
CLDN3
EPCAM
SPINT1
TSPAN1
KRT8
S100A14
NQO1
TFF1
CLDN4
KRT18
MUC1
SPINT2
EHF
AP1M2
TFF3
JUP
CD24
MLPH
ERBB2
DDR1
LSR
PERP
RAB11FIF
MAPK13
KRT7
GALNT7
TPD52
SH3YL1
EZR
EPS8L2

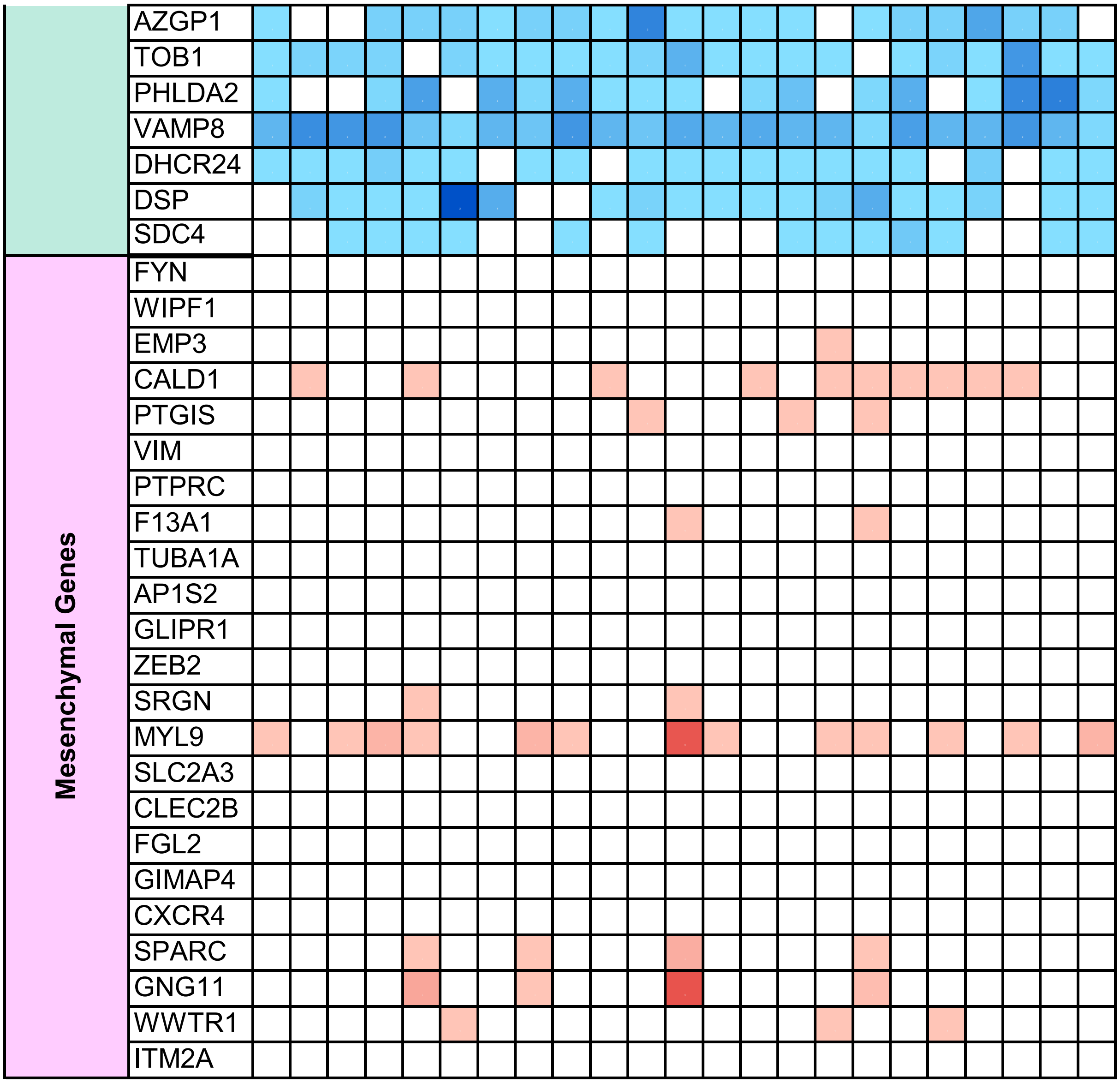
AZGP1
TOB1
PHLDA2
VAMP8
DHCR24
DSP
SDC4
Mesenchymal Genes
FYN
WIPF1
EMP3
CALD1
PTGIS
VIM
PTPRC
F13A1
TUBA1A
AP1S2
GLIPR1
ZEB2
SRGN
MYL9
SLC2A3
CLEC2B
FGL2
GIMAP4
CXCR4
SPARC
GNG11
WWTR1
ITM2A

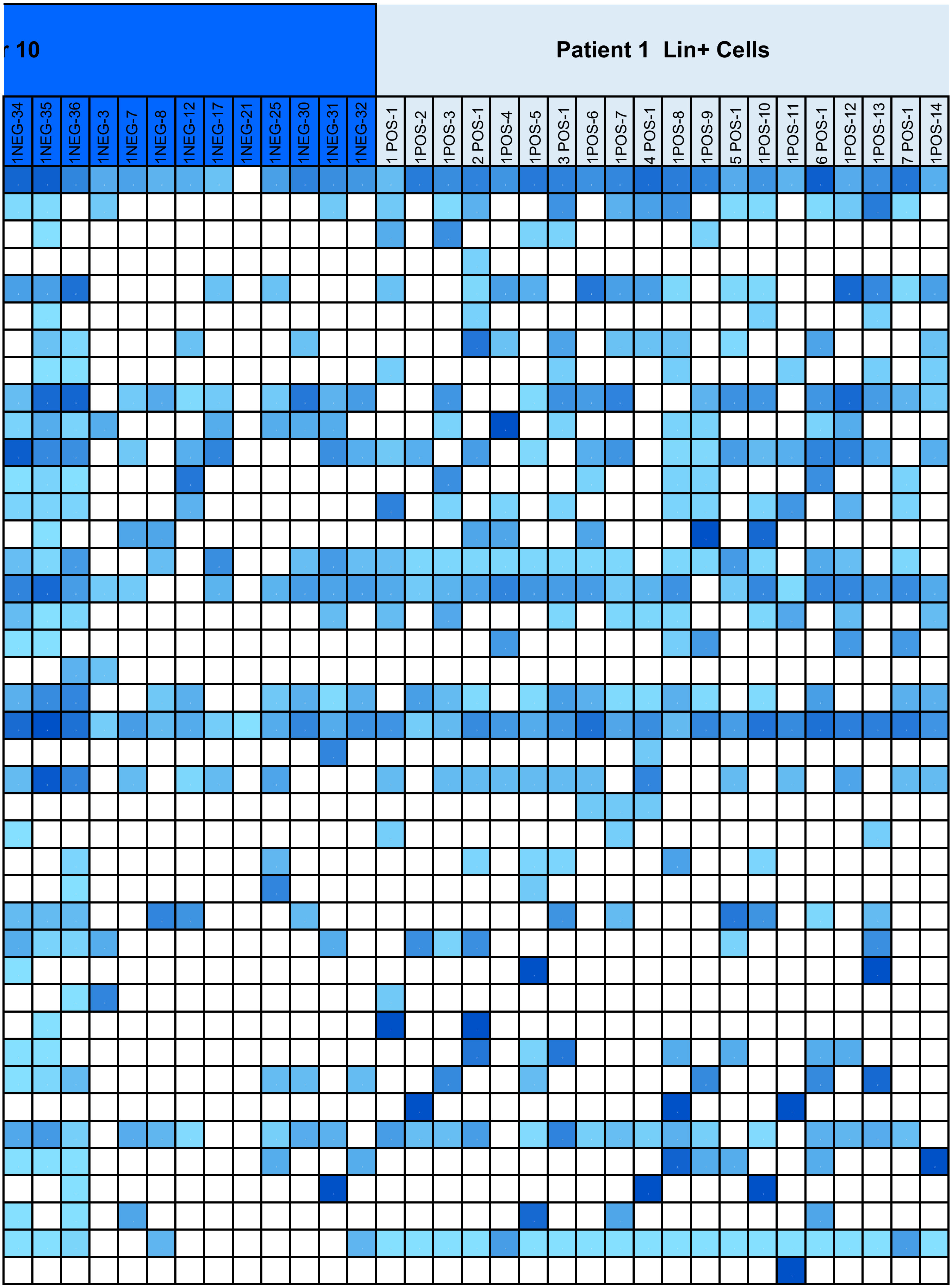
r 10
Patient 1 Lin+ Cells
1NEG-34
1NEG-35
1NEG-36
1NEG-3
1NEG-7
1NEG-8
1NEG-12
1NEG-17
1NEG-21
1NEG-25
1NEG-30
1NEG-31
1NEG-32
1 POS-1
1POS-2
1POS-3
2 POS-1
1POS-4
1POS-5
3 POS-1
1POS-6
1POS-7
4 POS-1
1POS-8
1POS-9
5 POS-1
1POS-10
1POS-11
6 POS-1
1POS-12
1POS-13
7 POS-1
1POS-14

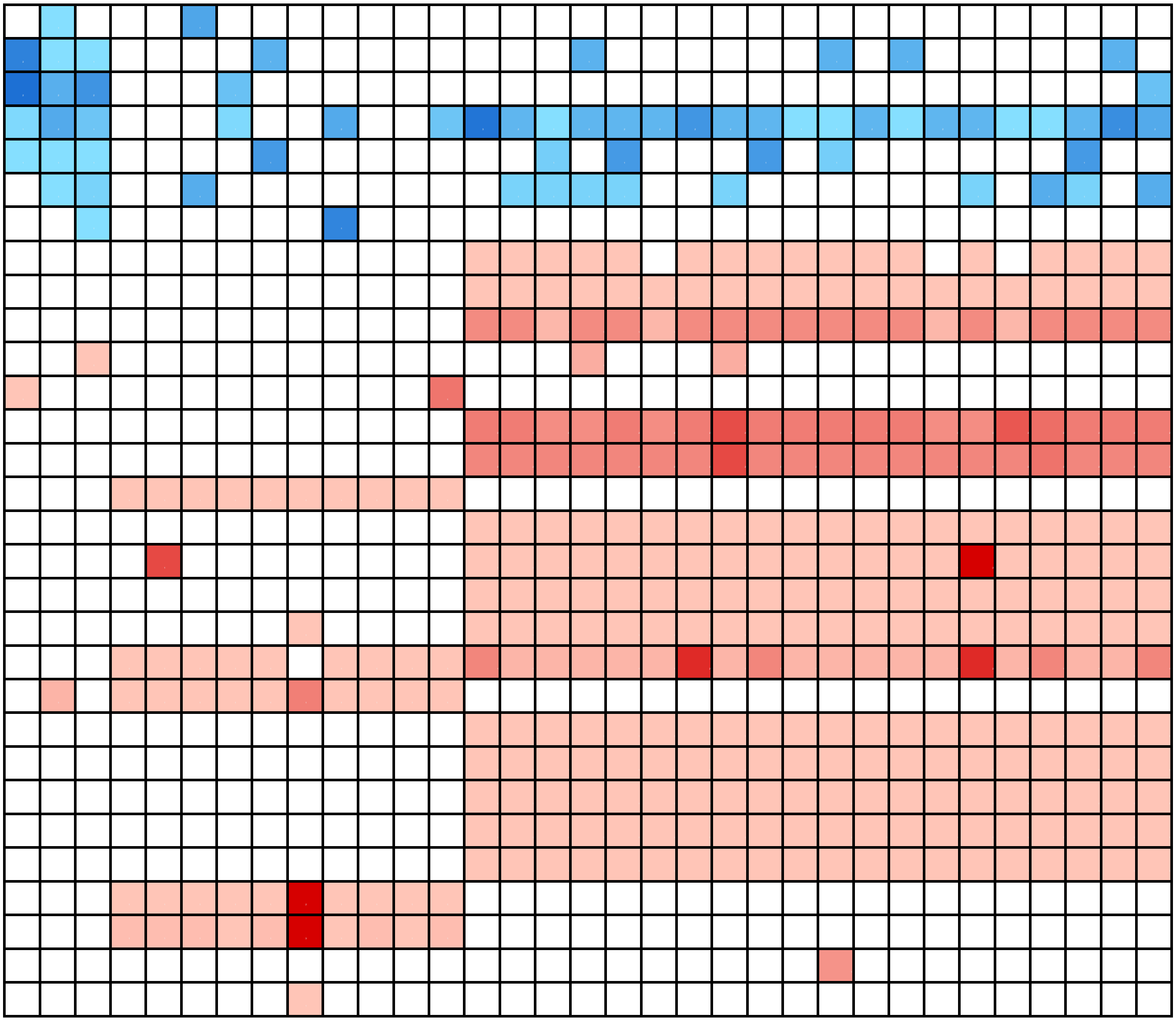

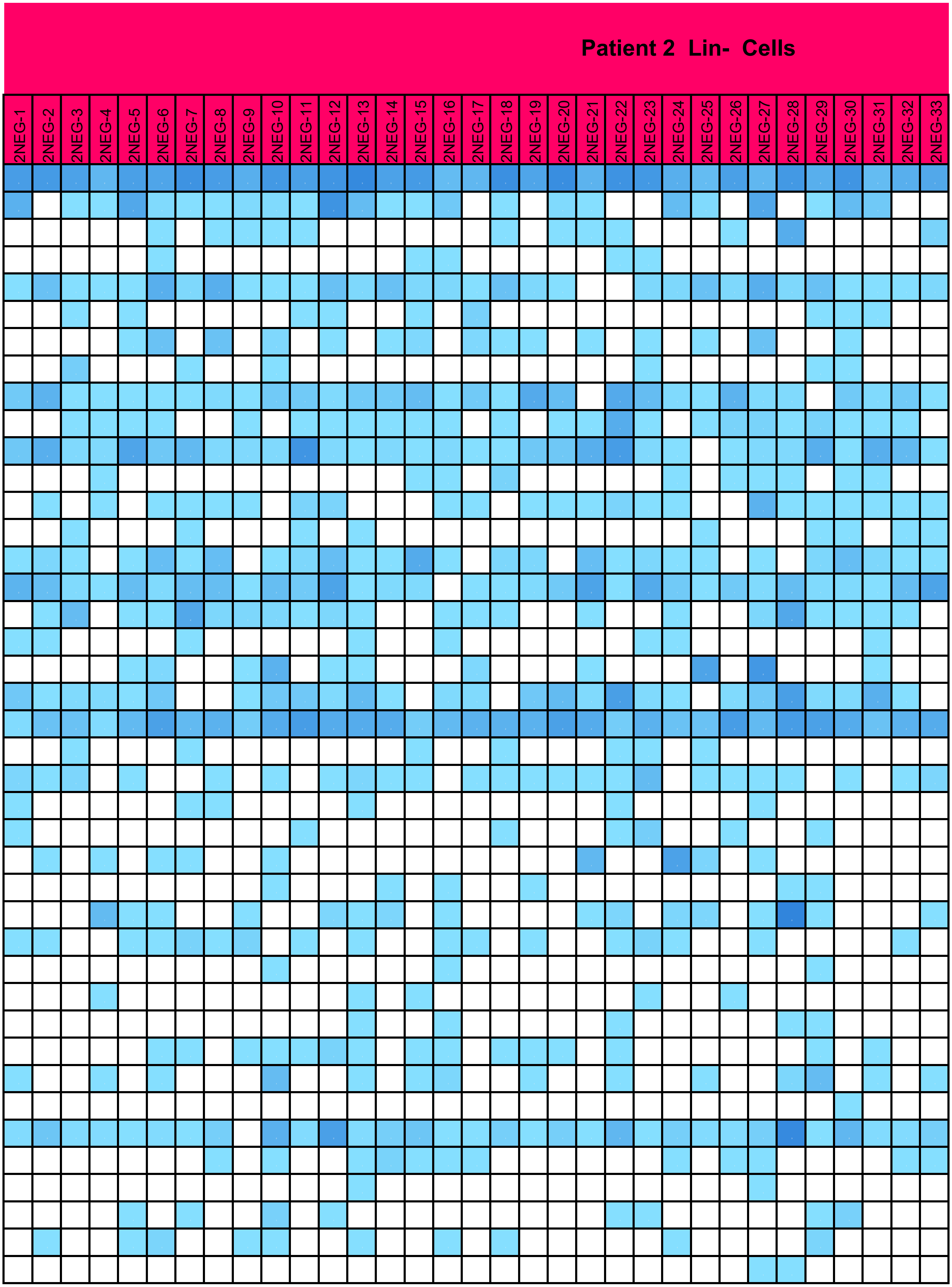
Patient 2 Lin- Cells
2NEG-1
2NEG-2
2NEG-3
2NEG-4
2NEG-5
2NEG-6
2NEG-7
2NEG-8
2NEG-9
2NEG-10
2NEG-11
2NEG-12
2NEG-13
2NEG-14
2NEG-15
2NEG-16
2NEG-17
2NEG-18
2NEG-19
2NEG-20
2NEG-21
2NEG-22
2NEG-23
2NEG-24
2NEG-25
2NEG-26
2NEG-27
2NEG-28
2NEG-29
2NEG-30
2NEG-31
2NEG-32
2NEG-33

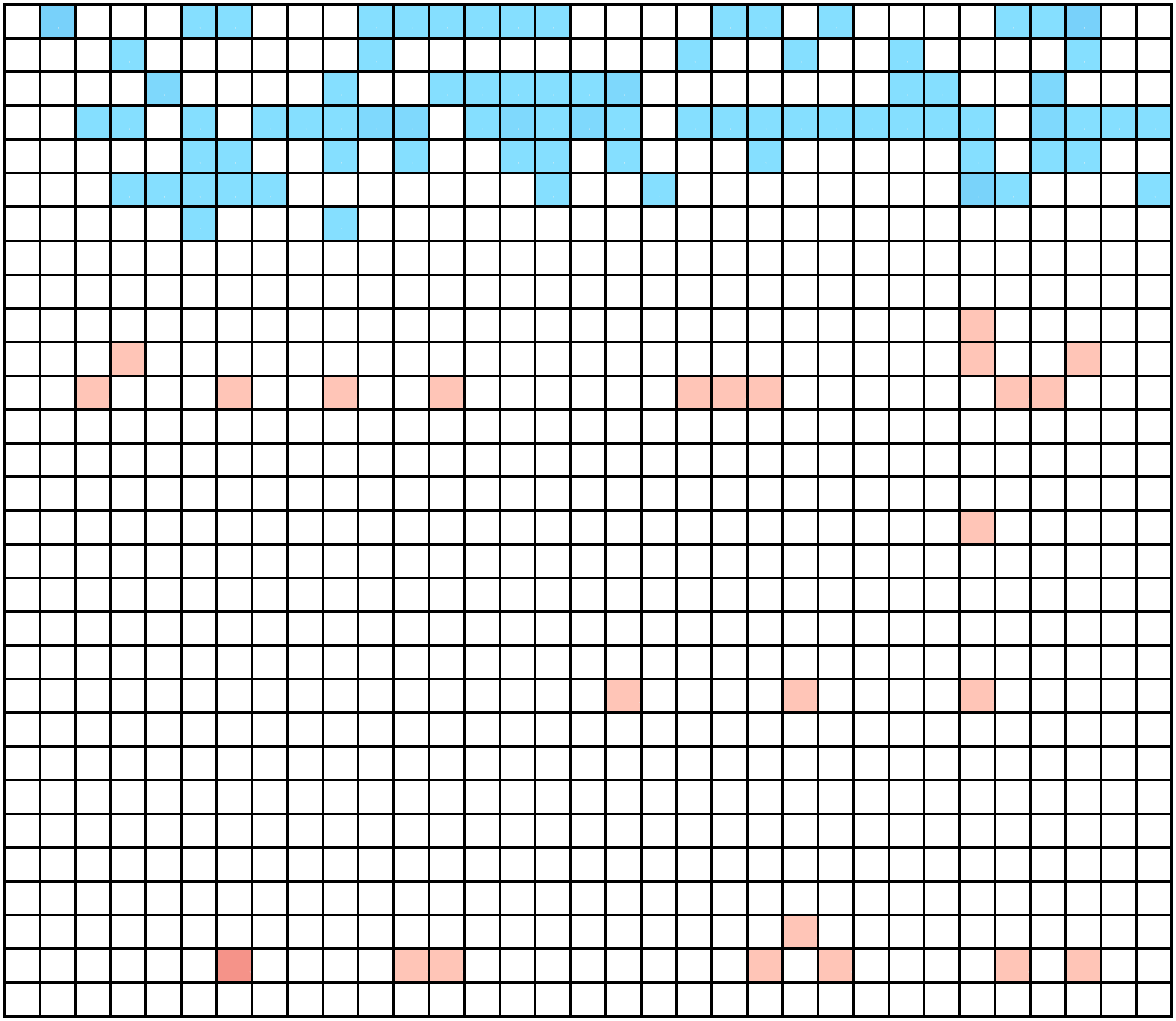

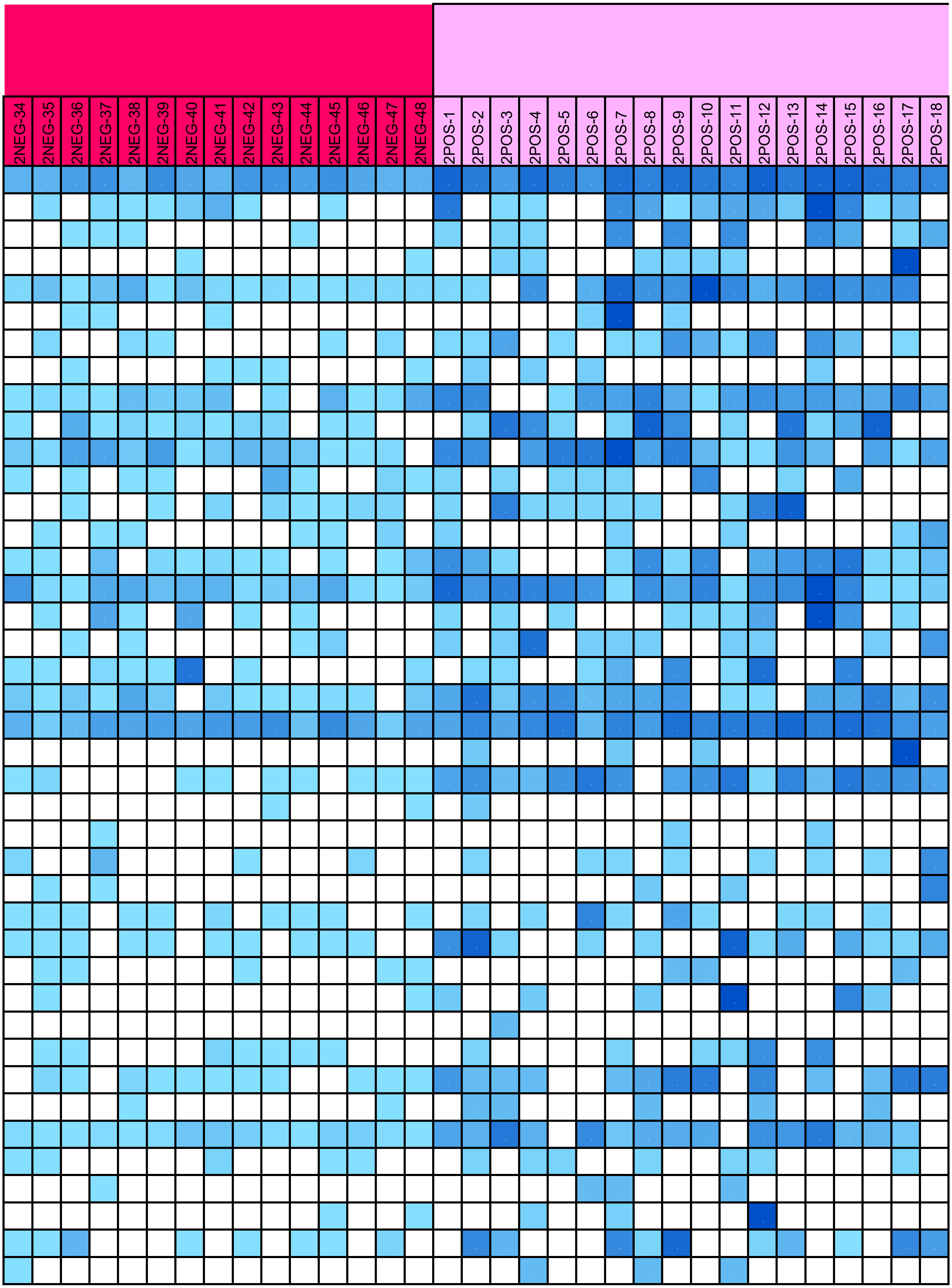
2NEG-34
2NEG-35
2NEG-36
2NEG-37
2NEG-38
2NEG-39
2NEG-40
2NEG-41
2NEG-42
2NEG-43
2NEG-44
2NEG-45
2NEG-46
2NEG-47
2NEG-48
2POS-1
2POS-2
2POS-3
2POS-4
2POS-5
2POS-6
2POS-7
2POS-8
2POS-9
2POS-10
2POS-11
2POS-12
2POS-13
2POS-14
2POS-15
2POS-16
2POS-17
2POS-18

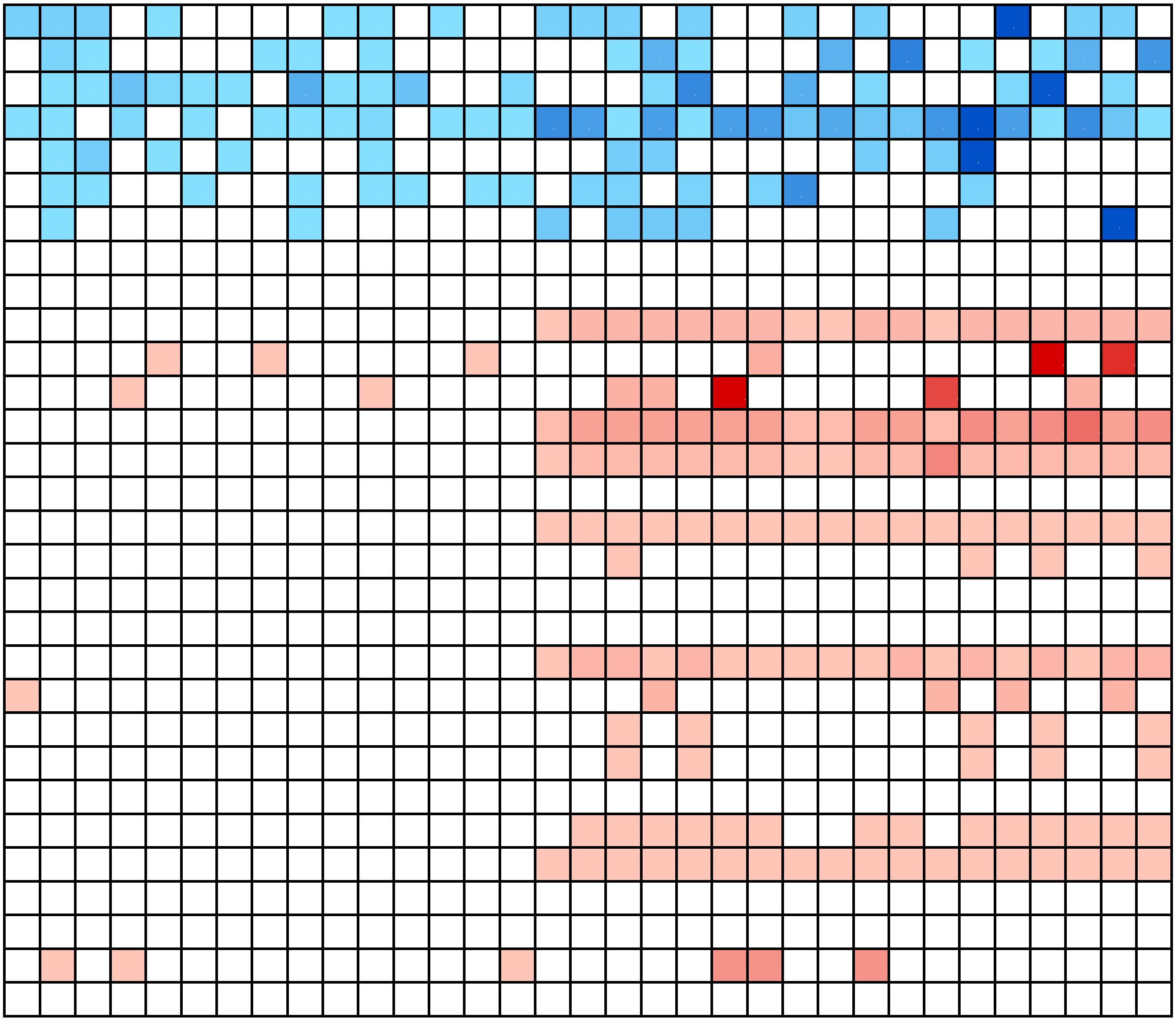

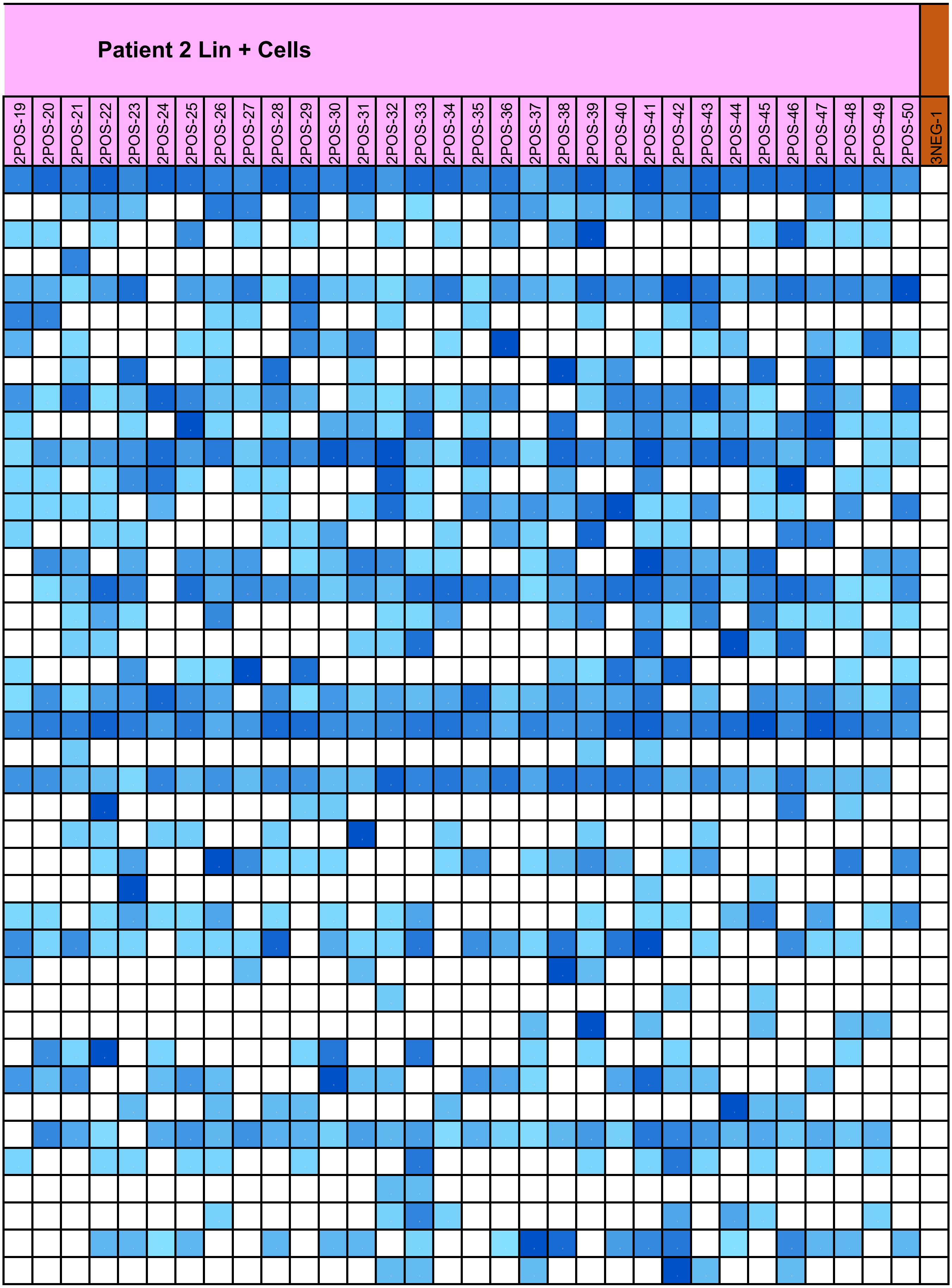
Patient 2 Lin + Cells
2POS-19
2POS-20
2POS-21
2POS-22
2POS-23
2POS-24
2POS-25
2POS-26
2POS-27
2POS-28
2POS-29
2POS-30
2POS-31
2POS-32
2POS-33
2POS-34
2POS-35
2POS-36
2POS-37
2POS-38
2POS-39
2POS-40
2POS-41
2POS-42
2POS-43
2POS-44
2POS-45
2POS-46
2POS-47
2POS-48
2POS-49
2POS-50
3NEG-1

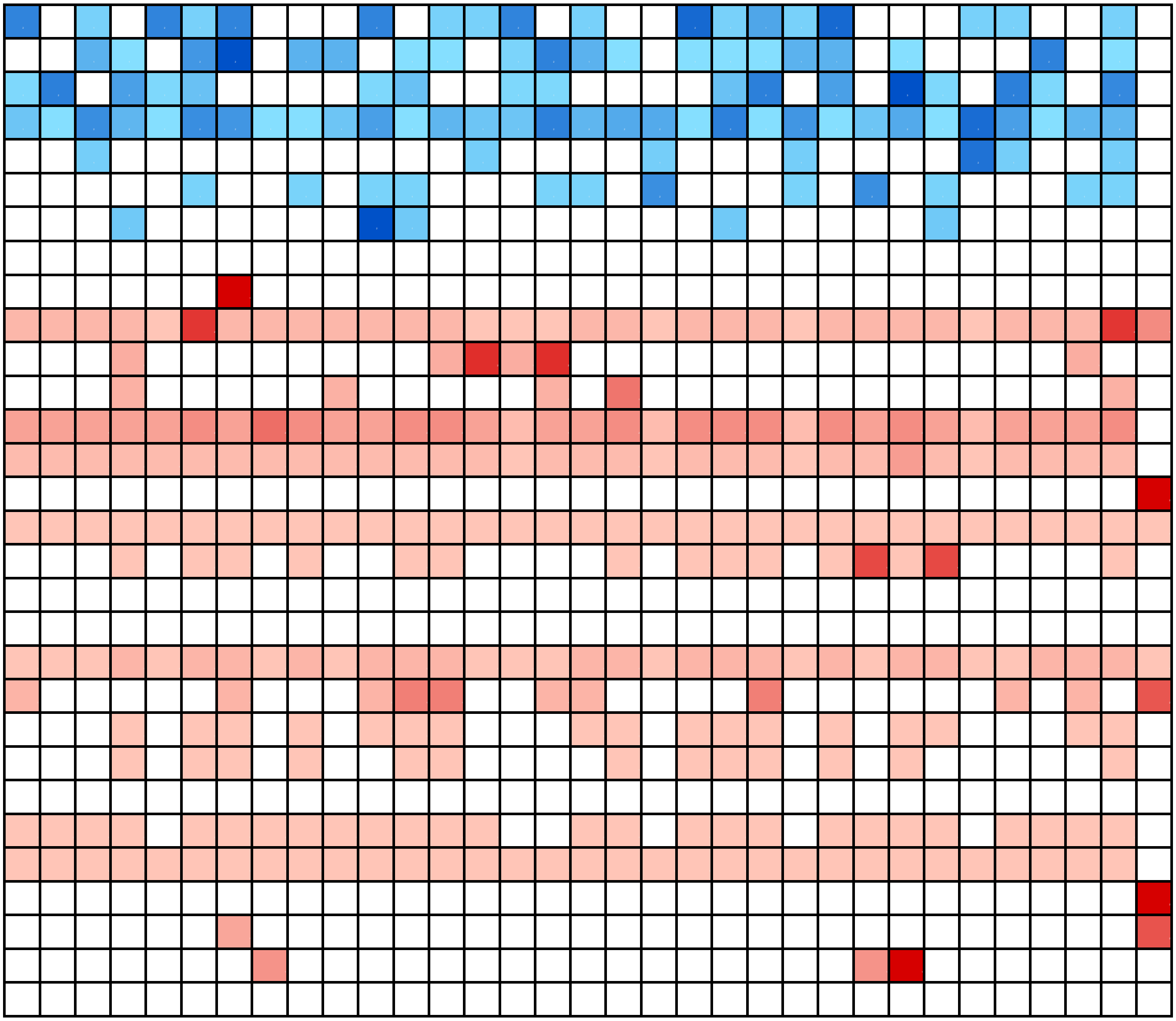

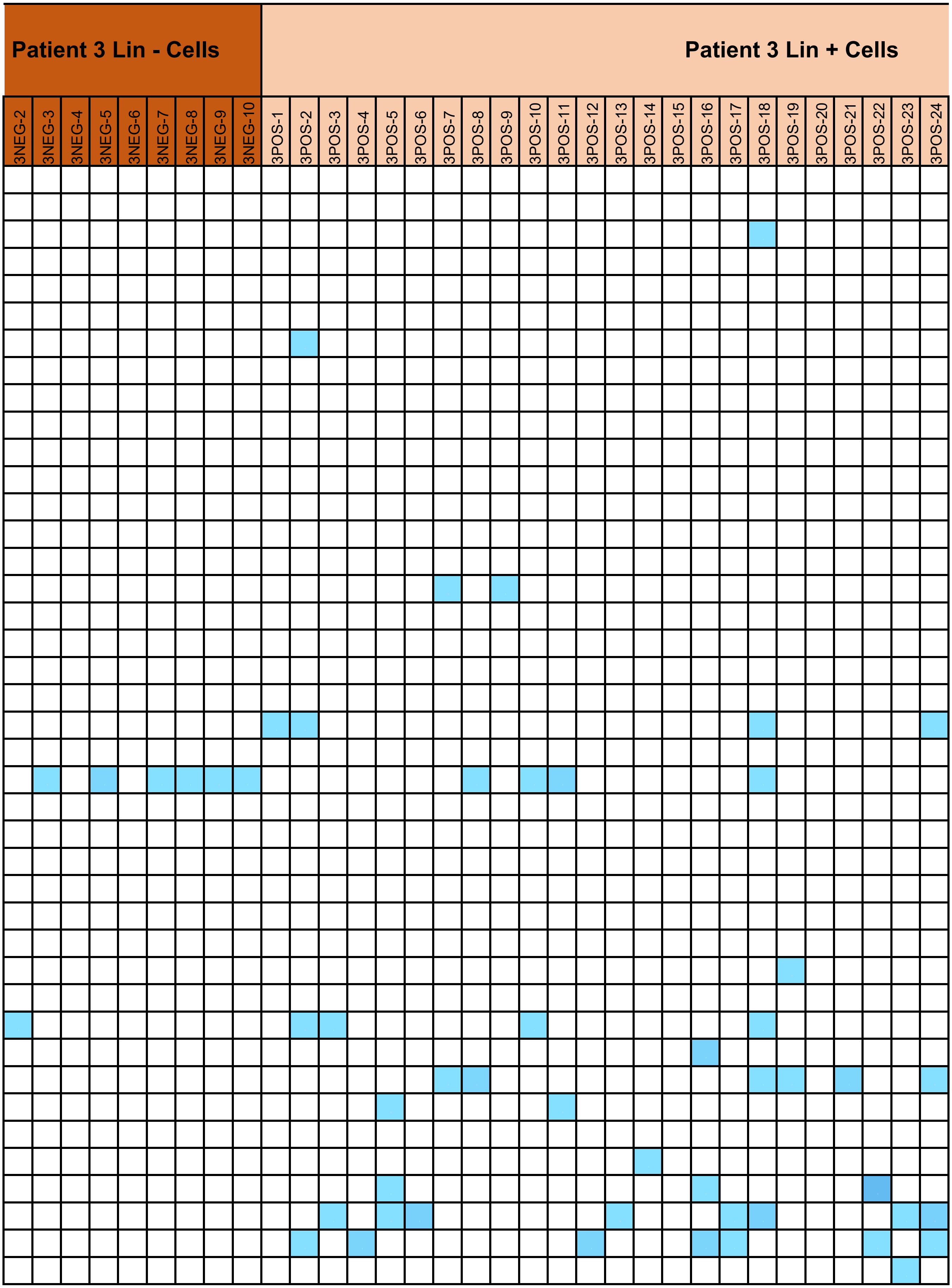
Patient 3 Lin - Cells
Patient 3 Lin + Cells
3NEG-2
3NEG-3
3NEG-4
3NEG-5
3NEG-6
3NEG-7
3NEG-8
3NEG-9
3NEG-10
3POS-1
3POS-2
3POS-3
3POS-4
3POS-5
3POS-6
3POS-7
3POS-8
3POS-9
3POS-10
3POS-11
3POS-12
3POS-13
3POS-14
3POS-15
3POS-16
3POS-17
3POS-18
3POS-19
3POS-20
3POS-21
3POS-22
3POS-23
3POS-24

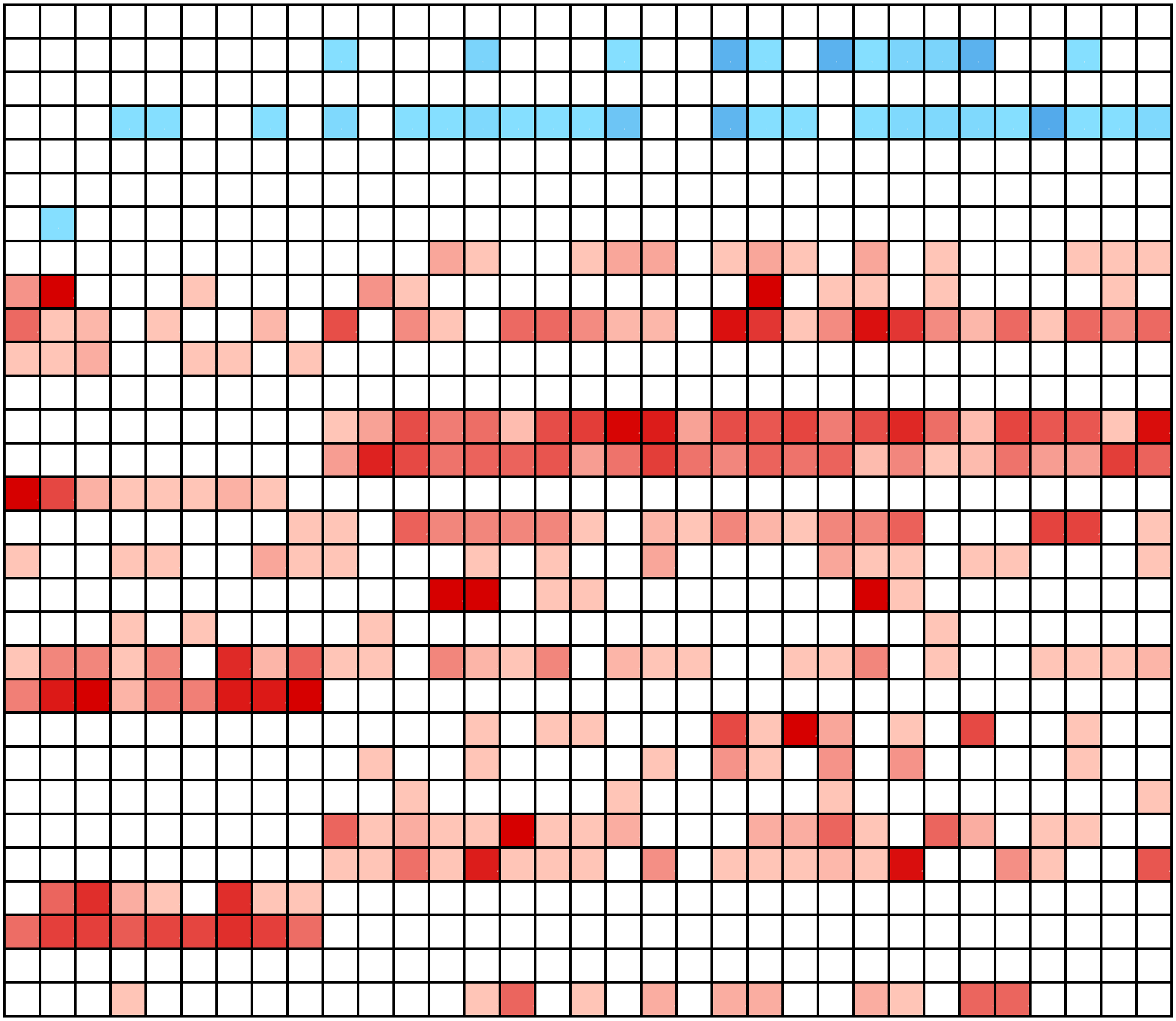

3POS-25	3POS-26	3POS-27	3POS-28	3POS-29	3POS-30	3POS-31	3POS-32	3POS-33	3POS-34	3POS-35	3POS-36	3POS-37	# cells expressing gene
													153
													98
													65
													23
													134
													44
													81
													48
													134
													108
													139
													74
													100
													63
													118
													149
													90
													58
													55
													131
													158
													27
													136
													22
													41
													62
													31
													89
													96
													25
													24
													31
													73
													94
													29
													139
													66
													28
													49
													107
													20

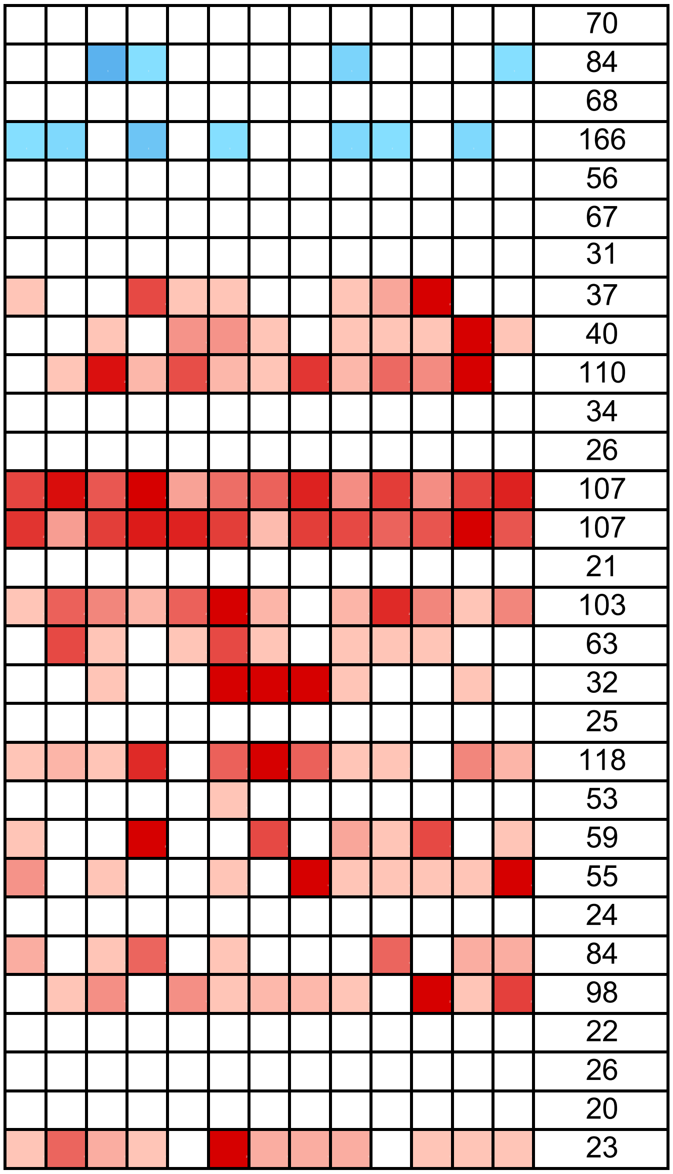
70
84
68
166
56
67
31
37
40
110
34
26
107
107
21
103
63
32
25
118
53
59
55
24
84
98
22
26
20
23

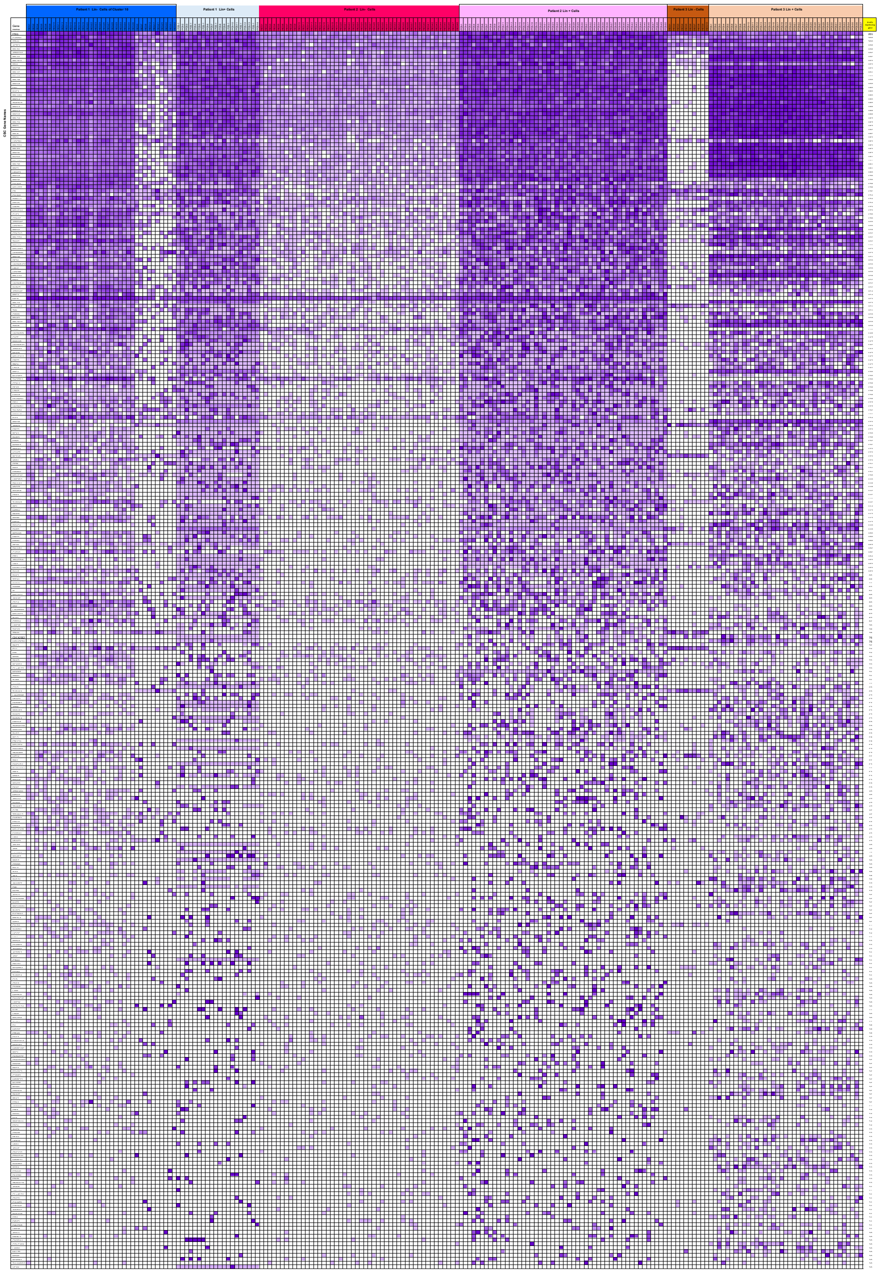

Patient 1 Lin- Cells of Cluster 10
Patient 1 Lin+ Cells
Patient 2 Lin- Cells
Patient 2 Lin + Cells
Patient 3 Lin - Cells
Patient 3 Lin + Cells
Gene name
CSC Gene Names

cluster	gene	description	p_val
10	B2M	beta-2-microglobulin [Source:HGNC Symbol;Acc:HGNC:914]	4.16E-158
10	MT-CO2	mitochondrially encoded cytochrome c oxidase II [Source:HGNC Sym	6.82E-19
10	RPLP1	ribosomal protein lateral stalk subunit P1 [Source:HGNC Symbol;Acc:	3.27E-69
10	GAPDH	glyceraldehyde-3-phosphate dehydrogenase [Source:HGNC Symbol;A	6.82E-32
10	UBC	ubiquitin C [Source:HGNC Symbol;Acc:HGNC:12468]	1.18E-32
10	SERF2	small EDRK-rich factor 2 [Source:HGNC Symbol;Acc:HGNC:10757]	1.73E-16
10	S100A6	S100 calcium binding protein A6 [Source:HGNC Symbol;Acc:HGNC:10	8.18E-107
10	ACTB	actin beta [Source:HGNC Symbol;Acc:HGNC:132]	2.55E-45
10	HLA-B	major histocompatibility complex, class I, B [Source:HGNC Symbol;Ac	4.43E-34
10	MT-ND1	mitochondrially encoded NADH:ubiquinone oxidoreductase core sub	3.28E-16
10	ACTG1	actin gamma 1 [Source:HGNC Symbol;Acc:HGNC:144]	6.76E-08
10	RPL13	ribosomal protein L13 [Source:HGNC Symbol;Acc:HGNC:10303]	3.14E-54
10	RPS14	ribosomal protein S14 [Source:HGNC Symbol;Acc:HGNC:10387]	1.04E-23
10	PPIA	peptidylprolyl isomerase A [Source:HGNC Symbol;Acc:HGNC:9253]	2.01E-46
10	TPT1	tumor protein, translationally-controlled 1 [Source:HGNC Symbol;Ac	1.72E-40
10	RPS12	ribosomal protein S12 [Source:HGNC Symbol;Acc:HGNC:10385]	4.90E-39
10	RPS3	ribosomal protein S3 [Source:HGNC Symbol;Acc:HGNC:10420]	6.94E-18
10	RPL35A	ribosomal protein L35a [Source:HGNC Symbol;Acc:HGNC:10345]	2.13E-08
10	TMSB4X	thymosin beta 4 X-linked [Source:HGNC Symbol;Acc:HGNC:11881]	1.02E-117
10	SAT1	spermidine/spermine N1-acetyltransferase 1 [Source:HGNC Symbol;	2.34E-43
10	RPL32	ribosomal protein L32 [Source:HGNC Symbol;Acc:HGNC:10336]	2.44E-30
10	HLA-A	major histocompatibility complex, class I, A [Source:HGNC Symbol;Ac	4.90E-29
10	JUN	Jun proto-oncogene, AP-1 transcription factor subunit [Source:HGNC	1.45E-25
10	S100A11	S100 calcium binding protein A11 [Source:HGNC Symbol;Acc:HGNC:1	1.06E-95
10	HLA-C	major histocompatibility complex, class I, C [Source:HGNC Symbol;Ac	1.09E-32
10	RPS3A	ribosomal protein S3A [Source:HGNC Symbol;Acc:HGNC:10421]	1.19E-24
10	RPL26	ribosomal protein L26 [Source:HGNC Symbol;Acc:HGNC:10327]	4.15E-21
10	RPS15A	ribosomal protein S15a [Source:HGNC Symbol;Acc:HGNC:10389]	4.25E-14
10	RPL29	ribosomal protein L29 [Source:HGNC Symbol;Acc:HGNC:10331]	7.71E-36
10	RPLP2	ribosomal protein lateral stalk subunit P2 [Source:HGNC Symbol;Acc:	4.29E-33
10	MT-ND2	mitochondrially encoded NADH:ubiquinone oxidoreductase core sub	1.87E-15
10	IER2	immediate early response 2 [Source:HGNC Symbol;Acc:HGNC:28871	1.27E-56
10	TMSB10	thymosin beta 10 [Source:HGNC Symbol;Acc:HGNC:11879]	1.81E-31
10	HSP90AB1	heat shock protein 90 alpha family class B member 1 [Source:HGNC S	3.65E-36
10	RPL5	ribosomal protein L5 [Source:HGNC Symbol;Acc:HGNC:10360]	4.70E-27
10	MYL12A	myosin light chain 12A [Source:HGNC Symbol;Acc:HGNC:16701]	1.53E-25
10	RPS7	ribosomal protein S7 [Source:HGNC Symbol;Acc:HGNC:10440]	4.67E-24
10	RPL39	ribosomal protein L39 [Source:HGNC Symbol;Acc:HGNC:10350]	4.48E-20
10	PFN1	profilin 1 [Source:HGNC Symbol;Acc:HGNC:8881]	1.34E-11
10	RPL11	ribosomal protein L11 [Source:HGNC Symbol;Acc:HGNC:10301]	3.08E-62
10	MYL12B	myosin light chain 12B [Source:HGNC Symbol;Acc:HGNC:29827]	1.00E-25

10	COX7C	cytochrome c oxidase subunit 7C [Source:HGNC Symbol;Acc:HGNC:2	3.01E-25
10	CALM2	calmodulin 2 [Source:HGNC Symbol;Acc:HGNC:1445]	1.95E-12
10	COX6A1	cytochrome c oxidase subunit 6A1 [Source:HGNC Symbol;Acc:HGNC:	2.79E-73
10	GSTP1	glutathione S-transferase pi 1 [Source:HGNC Symbol;Acc:HGNC:4638	8.34E-57
10	ELOB	elongin B [Source:HGNC Symbol;Acc:HGNC:11619]	2.83E-33
10	BTF3	basic transcription factor 3 [Source:HGNC Symbol;Acc:HGNC:1125]	5.39E-16
10	OST4	oligosaccharyltransferase complex subunit 4, non-catalytic [Source:H	7.79E-12
10	KLF2	Kruppel like factor 2 [Source:HGNC Symbol;Acc:HGNC:6347]	1.24E-11
10	RPL6	ribosomal protein L6 [Source:HGNC Symbol;Acc:HGNC:10362]	2.91E-44
10	OAZ1	ornithine decarboxylase antizyme 1 [Source:HGNC Symbol;Acc:HGN	8.08E-37
10	ATP5MC2	ATP synthase membrane subunit c locus 2 [Source:HGNC Symbol;Ac	2.31E-22
10	SRP14	signal recognition particle 14 [Source:HGNC Symbol;Acc:HGNC:1129	6.25E-07
10	FTL	ferritin light chain [Source:HGNC Symbol;Acc:HGNC:3999]	2.89E-64
10	CHCHD2	coiled-coil-helix-coiled-coil-helix domain containing 2 [Source:HGNC	9.59E-47
10	COX8A	cytochrome c oxidase subunit 8A [Source:HGNC Symbol;Acc:HGNC:2	1.68E-65
10	NPM1	nucleophosmin 1 [Source:HGNC Symbol;Acc:HGNC:7910]	3.78E-16
10	CD63	CD63 molecule [Source:HGNC Symbol;Acc:HGNC:1692]	7.50E-74
10	RPS21	ribosomal protein S21 [Source:HGNC Symbol;Acc:HGNC:10409]	2.89E-19
10	RPS16	ribosomal protein S16 [Source:HGNC Symbol;Acc:HGNC:10396]	1.31E-17
10	HINT1	histidine triad nucleotide binding protein 1 [Source:HGNC Symbol;Ac	1.88E-17
10	RPL36	ribosomal protein L36 [Source:HGNC Symbol;Acc:HGNC:13631]	2.20E-12
10	RPS25	ribosomal protein S25 [Source:HGNC Symbol;Acc:HGNC:10413]	1.01E-08
10	HSPA5	heat shock protein family A (Hsp70) member 5 [Source:HGNC Symbo	4.07E-133
10	DSTN	destrin, actin depolymerizing factor [Source:HGNC Symbol;Acc:HGN	3.08E-101
10	RPL37	ribosomal protein L37 [Source:HGNC Symbol;Acc:HGNC:10347]	4.16E-26
10	S100A10	S100 calcium binding protein A10 [Source:HGNC Symbol;Acc:HGNC:1	4.42E-11
10	KRT18	keratin 18 [Source:HGNC Symbol;Acc:HGNC:6430]	2.83E-224
10	COX5B	cytochrome c oxidase subunit 5B [Source:HGNC Symbol;Acc:HGNC:2	1.36E-52
10	RPL9	ribosomal protein L9 [Source:HGNC Symbol;Acc:HGNC:10369]	9.62E-12
10	SELENOW	selenoprotein W [Source:HGNC Symbol;Acc:HGNC:10752]	1.52E-56
10	ENO1	enolase 1 [Source:HGNC Symbol;Acc:HGNC:3350]	2.98E-49
10	GUK1	guanylate kinase 1 [Source:HGNC Symbol;Acc:HGNC:4693]	7.00E-35
10	VAMP8	vesicle associated membrane protein 8 [Source:HGNC Symbol;Acc:H	1.20E-35
10	NDUFS5	NADH:ubiquinone oxidoreductase subunit S5 [Source:HGNC Symbol;	1.72E-19
10	DYNLL1	dynein light chain LC8-type 1 [Source:HGNC Symbol;Acc:HGNC:1547	2.09E-13
10	MGP	matrix Gla protein [Source:HGNC Symbol;Acc:HGNC:7060]	1.65E-263
10	KRT19	keratin 19 [Source:HGNC Symbol;Acc:HGNC:6436]	6.02E-230
10	ANXA2	annexin A2 [Source:HGNC Symbol;Acc:HGNC:537]	2.33E-93
10	RPL34	ribosomal protein L34 [Source:HGNC Symbol;Acc:HGNC:10340]	8.55E-65
10	C12orf57	chromosome 12 open reading frame 57 [Source:HGNC Symbol;Acc:H	3.15E-21
10	HSPB1	heat shock protein family B (small) member 1 [Source:HGNC Symbol	7.96E-125
10	NDUFB2	NADH:ubiquinone oxidoreductase subunit B2 [Source:HGNC Symbol;	3.94E-39
10	ARHGDIB	Rho GDP dissociation inhibitor beta [Source:HGNC Symbol;Acc:HGN	7.38E-21
10	KRT8	keratin 8 [Source:HGNC Symbol;Acc:HGNC:6446]	1.75E-193
10	PEBP1	phosphatidylethanolamine binding protein 1 [Source:HGNC Symbol;	2.92E-62
10	COX6C	cytochrome c oxidase subunit 6C [Source:HGNC Symbol;Acc:HGNC:2	7.56E-33
10	CRABP2	cellular retinoic acid binding protein 2 [Source:HGNC Symbol;Acc:HG	1.26E-219

10	COX7A2	cytochrome c oxidase subunit 7A2 [Source:HGNC Symbol;Acc:HGNC:	4.09E-54
10	ITM2B	integral membrane protein 2B [Source:HGNC Symbol;Acc:HGNC:617	1.95E-42
10	POLR2L	RNA polymerase II, I and III subunit L [Source:HGNC Symbol;Acc:HGN	1.28E-37
10	RPL22	ribosomal protein L22 [Source:HGNC Symbol;Acc:HGNC:10315]	2.99E-33
10	RHOB	ras homolog family member B [Source:HGNC Symbol;Acc:HGNC:668]	4.59E-112
10	SPINT2	serine peptidase inhibitor, Kunitz type 2 [Source:HGNC Symbol;Acc:H	3.09E-78
10	PRDX1	peroxiredoxin 1 [Source:HGNC Symbol;Acc:HGNC:9352]	4.98E-72
10	HSP90B1	heat shock protein 90 beta family member 1 [Source:HGNC Symbol;A	2.35E-39
10	UBL5	ubiquitin like 5 [Source:HGNC Symbol;Acc:HGNC:13736]	5.95E-12
10	COX7B	cytochrome c oxidase subunit 7B [Source:HGNC Symbol;Acc:HGNC:2	5.18E-40
10	RPL13A	ribosomal protein L13a [Source:HGNC Symbol;Acc:HGNC:10304]	2.86E-30
10	TMBIM6	transmembrane BAX inhibitor motif containing 6 [Source:HGNC Sym	8.62E-15
10	RPL21	ribosomal protein L21 [Source:HGNC Symbol;Acc:HGNC:10313]	3.29E-24
10	SLC25A5	solute carrier family 25 member 5 [Source:HGNC Symbol;Acc:HGNC:	1.59E-32
10	SOD1	superoxide dismutase 1 [Source:HGNC Symbol;Acc:HGNC:11179]	2.17E-29
10	MGST3	microsomal glutathione S-transferase 3 [Source:HGNC Symbol;Acc:H	1.54E-98
10	UQCRQ	ubiquinol-cytochrome c reductase complex III subunit VII [Source:HG	3.41E-73
10	TMEM59	transmembrane protein 59 [Source:HGNC Symbol;Acc:HGNC:1239]	1.81E-61
10	NDUFB4	NADH:ubiquinone oxidoreductase subunit B4 [Source:HGNC Symbol;	5.08E-59
10	TSTD1	thiosulfate sulfurtransferase like domain containing 1 [Source:HGNC	9.25E-90
10	EDF1	endothelial differentiation related factor 1 [Source:HGNC Symbol;Ac	1.81E-26
10	SSR2	signal sequence receptor subunit 2 [Source:HGNC Symbol;Acc:HGNC	2.39E-13
10	TPI1	triosephosphate isomerase 1 [Source:HGNC Symbol;Acc:HGNC:1200	2.02E-11
10	UQCR11	ubiquinol-cytochrome c reductase, complex III subunit XI [Source:HG	1.57E-08
10	TACSTD2	tumor associated calcium signal transducer 2 [Source:HGNC Symbol;	2.39E-198
10	ID1	inhibitor of DNA binding 1, HLH protein [Source:HGNC Symbol;Acc:H	1.92E-176
10	ID3	inhibitor of DNA binding 3, HLH protein [Source:HGNC Symbol;Acc:H	4.31E-156
10	CALM1	calmodulin 1 [Source:HGNC Symbol;Acc:HGNC:1442]	8.52E-20
10	KRT7	keratin 7 [Source:HGNC Symbol;Acc:HGNC:6445]	6.61E-190
10	CD9	CD9 molecule [Source:HGNC Symbol;Acc:HGNC:1709]	1.81E-55
10	TXN	thioredoxin [Source:HGNC Symbol;Acc:HGNC:12435]	9.39E-44
10	ZFAS1	ZNFX1 antisense RNA 1 [Source:HGNC Symbol;Acc:HGNC:33101]	2.59E-22
10	HMGB1	high mobility group box 1 [Source:HGNC Symbol;Acc:HGNC:4983]	2.03E-21
10	GNG5	G protein subunit gamma 5 [Source:HGNC Symbol;Acc:HGNC:4408]	2.30E-20
10	NCL	nucleolin [Source:HGNC Symbol;Acc:HGNC:7667]	1.51E-08
10	JTB	jumping translocation breakpoint [Source:HGNC Symbol;Acc:HGNC:6	7.82E-63
10	RPS29	ribosomal protein S29 [Source:HGNC Symbol;Acc:HGNC:10419]	3.11E-15
10	RPL37A	ribosomal protein L37a [Source:HGNC Symbol;Acc:HGNC:10348]	8.50E-09
10	HNRNPA2B1	heterogeneous nuclear ribonucleoprotein A2/B1 [Source:HGNC Sym	6.31E-08
10	ATP6V1G1	ATPase H+ transporting V1 subunit G1 [Source:HGNC Symbol;Acc:HG	7.08E-10
10	PPIB	peptidylprolyl isomerase B [Source:HGNC Symbol;Acc:HGNC:9255]	7.69E-10
10	ELF3	E74 like ETS transcription factor 3 [Source:HGNC Symbol;Acc:HGNC:3	5.12E-185
10	BTG2	BTG anti-proliferation factor 2 [Source:HGNC Symbol;Acc:HGNC:113	1.76E-27
10	MZT2B	mitotic spindle organizing protein 2B [Source:HGNC Symbol;Acc:HGN	3.37E-09
10	CLDN4	claudin 4 [Source:HGNC Symbol;Acc:HGNC:2046]	7.31E-181
10	FXYD3	FXYD domain containing ion transport regulator 3 [Source:HGNC Sym	1.56E-151
10	DBI	diazepam binding inhibitor, acyl-CoA binding protein [Source:HGNC S	9.24E-34

10	NDUFA13	NADH:ubiquinone oxidoreductase subunit A13 [Source:HGNC Symbo	4.00E-19
10	ATP5F1D	ATP synthase F1 subunit delta [Source:HGNC Symbol;Acc:HGNC:837]	5.39E-19
10	SH3BGRL3	SH3 domain binding glutamate rich protein like 3 [Source:HGNC Sym	2.12E-84
10	NDUFA2	NADH:ubiquinone oxidoreductase subunit A2 [Source:HGNC Symbol;	3.47E-64
10	UFC1	ubiquitin-fold modifier conjugating enzyme 1 [Source:HGNC Symbol;	1.23E-33
10	UQCR10	ubiquinol-cytochrome c reductase, complex III subunit X [Source:HG	1.49E-13
10	PRDX5	peroxiredoxin 5 [Source:HGNC Symbol;Acc:HGNC:9355]	2.54E-11
10	NDUFB7	NADH:ubiquinone oxidoreductase subunit B7 [Source:HGNC Symbol;	2.66E-53
10	ATP5MC3	ATP synthase membrane subunit c locus 3 [Source:HGNC Symbol;Acc	2.73E-30
10	HSPE1	heat shock protein family E (Hsp10) member 1 [Source:HGNC Symbo	1.73E-26
10	SLC25A3	solute carrier family 25 member 3 [Source:HGNC Symbol;Acc:HGNC:	7.75E-16
10	ARPC2	actin related protein 2/3 complex subunit 2 [Source:HGNC Symbol;A	9.00E-13
10	CAST	calpastatin [Source:HGNC Symbol;Acc:HGNC:1515]	8.21E-11
10	PDIA3	protein disulfide isomerase family A member 3 [Source:HGNC Symbo	4.33E-24
10	NDUFA1	NADH:ubiquinone oxidoreductase subunit A1 [Source:HGNC Symbol;	6.24E-07
10	ATP5PF	ATP synthase peripheral stalk subunit F6 [Source:HGNC Symbol;Acc:	2.26E-25
10	RPL38	ribosomal protein L38 [Source:HGNC Symbol;Acc:HGNC:10349]	1.00E-15
10	MARCKSL1	MARCKS like 1 [Source:HGNC Symbol;Acc:HGNC:7142]	3.68E-51
10	PRDX2	peroxiredoxin 2 [Source:HGNC Symbol;Acc:HGNC:9353]	2.26E-29
10	RPL7	ribosomal protein L7 [Source:HGNC Symbol;Acc:HGNC:10363]	2.82E-19
10	TXNIP	thioredoxin interacting protein [Source:HGNC Symbol;Acc:HGNC:169	1.29E-08
10	ENSA	endosulfine alpha [Source:HGNC Symbol;Acc:HGNC:3360]	1.90E-36
10	CALR	calreticulin [Source:HGNC Symbol;Acc:HGNC:1455]	1.30E-23
10	VPS28	VPS28 subunit of ESCRT-I [Source:HGNC Symbol;Acc:HGNC:18178]	2.44E-16
10	BST2	bone marrow stromal cell antigen 2 [Source:HGNC Symbol;Acc:HGNC	1.28E-33
10	NDUFB9	NADH:ubiquinone oxidoreductase subunit B9 [Source:HGNC Symbol;	3.81E-25
10	ATP5ME	ATP synthase membrane subunit e [Source:HGNC Symbol;Acc:HGNC	3.33E-21
10	NDUFB11	NADH:ubiquinone oxidoreductase subunit B11 [Source:HGNC Symbo	3.57E-19
10	PTGES3	prostaglandin E synthase 3 [Source:HGNC Symbol;Acc:HGNC:16049]	4.89E-12
10	TUBB4B	tubulin beta 4B class IVb [Source:HGNC Symbol;Acc:HGNC:20771]	6.70E-40
10	ATP5F1B	ATP synthase F1 subunit beta [Source:HGNC Symbol;Acc:HGNC:830]	2.91E-25
10	TSPAN1	tetraspanin 1 [Source:HGNC Symbol;Acc:HGNC:20657]	1.71E-163
10	SCCPDH	saccharopine dehydrogenase (putative) [Source:HGNC Symbol;Acc:H	4.53E-145
10	STARD10	StAR related lipid transfer domain containing 10 [Source:HGNC Symb	1.24E-133
10	TPM3	tropomyosin 3 [Source:HGNC Symbol;Acc:HGNC:12012]	9.47E-17
10	RPS27L	ribosomal protein S27 like [Source:HGNC Symbol;Acc:HGNC:18476]	2.60E-12
10	SPCS1	signal peptidase complex subunit 1 [Source:HGNC Symbol;Acc:HGNC	3.13E-12
10	CAMK2N1	calcium/calmodulin dependent protein kinase II inhibitor 1 [Source:H	5.46E-116
10	PLK2	polo like kinase 2 [Source:HGNC Symbol;Acc:HGNC:19699]	1.13E-115
10	NUCKS1	nuclear casein kinase and cyclin dependent kinase substrate 1 [Sourc	1.27E-16
10	ATP5PO	ATP synthase peripheral stalk subunit OSCP [Source:HGNC Symbol;A	8.48E-10
10	MDH2	malate dehydrogenase 2 [Source:HGNC Symbol;Acc:HGNC:6971]	2.49E-28
10	ATP5MD	ATP synthase membrane subunit DAPIT [Source:HGNC Symbol;Acc:H	1.73E-13
10	ATP5PD	ATP synthase peripheral stalk subunit d [Source:HGNC Symbol;Acc:H	1.97E-23
10	SEC62	SEC62 homolog, preprotein translocation factor [Source:HGNC Symb	1.36E-17
10	CUTA	cutA divalent cation tolerance homolog [Source:HGNC Symbol;Acc:H	1.12E-14
10	HERPUD1	homocysteine inducible ER protein with ubiquitin like domain 1 [Sou	4.28E-48

10	PYCARD	PYD and CARD domain containing [Source:HGNC Symbol;Acc:HGNC:1	7.60E-22
10	ATP5MF	ATP synthase membrane subunit f [Source:HGNC Symbol;Acc:HGNC:	2.46E-12
10	LGALS3	galectin 3 [Source:HGNC Symbol;Acc:HGNC:6563]	1.85E-48
10	NENF	neudesin neurotrophic factor [Source:HGNC Symbol;Acc:HGNC:3038	4.39E-40
10	BRK1	BRICK1 subunit of SCAR/WAVE actin nucleating complex [Source:HG	4.47E-13
10	SLC3A1	solute carrier family 3 member 1 [Source:HGNC Symbol;Acc:HGNC:1	1.20E-133
10	NDUFS6	NADH:ubiquinone oxidoreductase subunit S6 [Source:HGNC Symbol;	5.07E-49
10	HLA-E	major histocompatibility complex, class I, E [Source:HGNC Symbol;Ac	2.20E-89
10	SRP9	signal recognition particle 9 [Source:HGNC Symbol;Acc:HGNC:11304	1.41E-46
10	TNFSF10	TNF superfamily member 10 [Source:HGNC Symbol;Acc:HGNC:11925	4.63E-34
10	RHOC	ras homolog family member C [Source:HGNC Symbol;Acc:HGNC:669]	7.33E-25
10	COX5A	cytochrome c oxidase subunit 5A [Source:HGNC Symbol;Acc:HGNC:2	1.06E-14
10	ATP5IF1	ATP synthase inhibitory factor subunit 1 [Source:HGNC Symbol;Acc:H	2.19E-14
10	C19orf53	chromosome 19 open reading frame 53 [Source:HGNC Symbol;Acc:H	9.32E-10
10	CLDN7	claudin 7 [Source:HGNC Symbol;Acc:HGNC:2049]	3.29E-127
10	CCND1	cyclin D1 [Source:HGNC Symbol;Acc:HGNC:1582]	6.48E-127
10	LMNA	lamin A/C [Source:HGNC Symbol;Acc:HGNC:6636]	5.46E-24
10	TOMM20	translocase of outer mitochondrial membrane 20 [Source:HGNC Sym	6.87E-18
10	GADD45B	growth arrest and DNA damage inducible beta [Source:HGNC Symbo	1.00E-10
10	SLC9A3R2	SLC9A3 regulator 2 [Source:HGNC Symbol;Acc:HGNC:11076]	3.09E-122
10	TM4SF1	transmembrane 4 L six family member 1 [Source:HGNC Symbol;Acc:H	6.30E-118
10	TMEM141	transmembrane protein 141 [Source:HGNC Symbol;Acc:HGNC:28211	9.52E-92
10	SEM1	SEM1 26S proteasome complex subunit [Source:HGNC Symbol;Acc:H	4.98E-32
10	PPP1R14B	protein phosphatase 1 regulatory inhibitor subunit 14B [Source:HGN	8.77E-52
10	HEBP2	heme binding protein 2 [Source:HGNC Symbol;Acc:HGNC:15716]	1.30E-40
10	CD151	CD151 molecule (Raph blood group) [Source:HGNC Symbol;Acc:HGN	2.36E-35
10	CSNK1A1	casein kinase 1 alpha 1 [Source:HGNC Symbol;Acc:HGNC:2451]	1.56E-17
10	BTG1	BTG anti-proliferation factor 1 [Source:HGNC Symbol;Acc:HGNC:113	2.75E-14
10	CTSD	cathepsin D [Source:HGNC Symbol;Acc:HGNC:2529]	1.70E-12
10	S100A13	S100 calcium binding protein A13 [Source:HGNC Symbol;Acc:HGNC:1	5.88E-128
10	GATA3	GATA binding protein 3 [Source:HGNC Symbol;Acc:HGNC:4172]	6.87E-71
10	SPCS2	signal peptidase complex subunit 2 [Source:HGNC Symbol;Acc:HGNC	1.34E-15
10	TRMT112	tRNA methyltransferase subunit 11-2 [Source:HGNC Symbol;Acc:HGN	3.32E-11
10	ARL6IP5	ADP ribosylation factor like GTPase 6 interacting protein 5 [Source:H	2.21E-08
10	NDUFA11	NADH:ubiquinone oxidoreductase subunit A11 [Source:HGNC Symbo	2.58E-08
10	SEC61B	SEC61 translocon subunit beta [Source:HGNC Symbol;Acc:HGNC:169	1.07E-07
10	S100A16	S100 calcium binding protein A16 [Source:HGNC Symbol;Acc:HGNC:2	1.87E-130
10	GCHFR	GTP cyclohydrolase I feedback regulator [Source:HGNC Symbol;Acc:H	2.83E-90
10	MRPL51	mitochondrial ribosomal protein L51 [Source:HGNC Symbol;Acc:HGN	7.00E-63
10	AURKAIP1	aurora kinase A interacting protein 1 [Source:HGNC Symbol;Acc:HGN	9.50E-35
10	NDUFC1	NADH:ubiquinone oxidoreductase subunit C1 [Source:HGNC Symbol;	4.20E-34
10	NDUFAB1	NADH:ubiquinone oxidoreductase subunit AB1 [Source:HGNC Symbo	1.61E-30
10	P4HB	prolyl 4-hydroxylase subunit beta [Source:HGNC Symbol;Acc:HGNC:8	1.95E-29
10	CST3	cystatin C [Source:HGNC Symbol;Acc:HGNC:2475]	5.09E-24
10	WDR83OS	WD repeat domain 83 opposite strand [Source:HGNC Symbol;Acc:HG	1.03E-07
10	TSC22D3	TSC22 domain family member 3 [Source:HGNC Symbol;Acc:HGNC:30	1.24E-07
10	ECH1	enoyl-CoA hydratase 1 [Source:HGNC Symbol;Acc:HGNC:3149]	2.92E-26

10	CHMP2A	charged multivesicular body protein 2A [Source:HGNC Symbol;Acc:H	5.49E-25
10	NDUFV2	NADH:ubiquinone oxidoreductase core subunit V2 [Source:HGNC Syr	6.22E-25
10	SRSF9	serine and arginine rich splicing factor 9 [Source:HGNC Symbol;Acc:H	2.82E-09
10	ATP6V0B	ATPase H+ transporting V0 subunit b [Source:HGNC Symbol;Acc:HGN	9.36E-18
10	VDAC2	voltage dependent anion channel 2 [Source:HGNC Symbol;Acc:HGNC	1.55E-15
10	NOP10	NOP10 ribonucleoprotein [Source:HGNC Symbol;Acc:HGNC:14378]	5.02E-15
10	FXYD5	FXYD domain containing ion transport regulator 5 [Source:HGNC Syn	2.65E-13
10	YWHAE	tyrosine 3-monooxygenase/tryptophan 5-monooxygenase activation	4.28E-09
10	CIB1	calcium and integrin binding 1 [Source:HGNC Symbol;Acc:HGNC:169	2.22E-07
10	AGR2	anterior gradient 2, protein disulphide isomerase family member [So	6.33E-133
10	REEP5	receptor accessory protein 5 [Source:HGNC Symbol;Acc:HGNC:3007	8.79E-51
10	COX7A2L	cytochrome c oxidase subunit 7A2 like [Source:HGNC Symbol;Acc:HG	1.41E-17
10	KRT10	keratin 10 [Source:HGNC Symbol;Acc:HGNC:6413]	8.28E-13
10	MCL1	MCL1 apoptosis regulator, BCL2 family member [Source:HGNC Symb	4.16E-10
10	RPL31	ribosomal protein L31 [Source:HGNC Symbol;Acc:HGNC:10334]	4.40E-08
10	EPCAM	epithelial cell adhesion molecule [Source:HGNC Symbol;Acc:HGNC:1	5.54E-133
10	MLPH	melanophilin [Source:HGNC Symbol;Acc:HGNC:29643]	5.34E-125
10	DEGS1	delta 4-desaturase, sphingolipid 1 [Source:HGNC Symbol;Acc:HGNC:	2.62E-77
10	DNAJC1	DnaJ heat shock protein family (Hsp40) member C1 [Source:HGNC Sy	1.41E-75
10	ARPC1B	actin related protein 2/3 complex subunit 1B [Source:HGNC Symbol;	1.53E-47
10	RTN4	reticulon 4 [Source:HGNC Symbol;Acc:HGNC:14085]	3.54E-13
10	RBM8A	RNA binding motif protein 8A [Source:HGNC Symbol;Acc:HGNC:9905	2.89E-11
10	ATF3	activating transcription factor 3 [Source:HGNC Symbol;Acc:HGNC:78	2.42E-70
10	CITED4	Cbp/p300 interacting transactivator with Glu/Asp rich carboxy-termi	2.37E-50
10	MRPL41	mitochondrial ribosomal protein L41 [Source:HGNC Symbol;Acc:HGN	4.82E-31
10	SUMO1	small ubiquitin like modifier 1 [Source:HGNC Symbol;Acc:HGNC:1250	3.78E-18
10	SELENOH	selenoprotein H [Source:HGNC Symbol;Acc:HGNC:18251]	5.54E-11
10	NUPR1	nuclear protein 1, transcriptional regulator [Source:HGNC Symbol;Ac	2.48E-128
10	LAMTOR2	late endosomal/lysosomal adaptor, MAPK and MTOR activator 2 [So	5.17E-36
10	NDUFS8	NADH:ubiquinone oxidoreductase core subunit S8 [Source:HGNC Syr	7.51E-34
10	DPP7	dipeptidyl peptidase 7 [Source:HGNC Symbol;Acc:HGNC:14892]	1.26E-27
10	COX17	cytochrome c oxidase copper chaperone COX17 [Source:HGNC Symb	8.04E-26
10	CYBA	cytochrome b-245 alpha chain [Source:HGNC Symbol;Acc:HGNC:257	1.60E-20
10	PPA1	inorganic pyrophosphatase 1 [Source:HGNC Symbol;Acc:HGNC:9226]	5.39E-18
10	RPS20	ribosomal protein S20 [Source:HGNC Symbol;Acc:HGNC:10405]	2.48E-16
10	CLTA	clathrin light chain A [Source:HGNC Symbol;Acc:HGNC:2090]	1.88E-09
10	HSPD1	heat shock protein family D (Hsp60) member 1 [Source:HGNC Symbo	7.40E-08
10	ID4	inhibitor of DNA binding 4, HLH protein [Source:HGNC Symbol;Acc:H	6.21E-126
10	RHOV	ras homolog family member V [Source:HGNC Symbol;Acc:HGNC:183	3.93E-123
10	CYB5A	cytochrome b5 type A [Source:HGNC Symbol;Acc:HGNC:2570]	2.90E-73
10	PAFAH1B3	platelet activating factor acetylhydrolase 1b catalytic subunit 3 [Sour	5.24E-71
10	YWHAZ	tyrosine 3-monooxygenase/tryptophan 5-monooxygenase activation	2.67E-28
10	ALKBH7	alkB homolog 7 [Source:HGNC Symbol;Acc:HGNC:21306]	2.21E-19
10	TUFM	Tu translation elongation factor, mitochondrial [Source:HGNC Symbo	7.56E-14
10	SNU13	small nuclear ribonucleoprotein 13 [Source:HGNC Symbol;Acc:HGNC	2.96E-11
10	NPW	neuropeptide W [Source:HGNC Symbol;Acc:HGNC:30509]	2.18E-118
10	NME1	NME/NM23 nucleoside diphosphate kinase 1 [Source:HGNC Symbol;	1.43E-60

10	IER3	immediate early response 3 [Source:HGNC Symbol;Acc:HGNC:5392]	1.17E-30
10	RAB11FIP1	RAB11 family interacting protein 1 [Source:HGNC Symbol;Acc:HGNC:	1.11E-29
10	MAGED2	MAGE family member D2 [Source:HGNC Symbol;Acc:HGNC:16353]	4.51E-21
10	GADD45GIP1	GADD45G interacting protein 1 [Source:HGNC Symbol;Acc:HGNC:29	1.73E-19
10	SNRPG	small nuclear ribonucleoprotein polypeptide G [Source:HGNC Symbo	6.23E-17
10	NDUFB10	NADH:ubiquinone oxidoreductase subunit B10 [Source:HGNC Symbo	8.97E-12
10	S100A14	S100 calcium binding protein A14 [Source:HGNC Symbol;Acc:HGNC:1	5.07E-118
10	NDUFB3	NADH:ubiquinone oxidoreductase subunit B3 [Source:HGNC Symbol;	1.57E-44
10	NAP1L1	nucleosome assembly protein 1 like 1 [Source:HGNC Symbol;Acc:HG	1.17E-38
10	NDUFS7	NADH:ubiquinone oxidoreductase core subunit S7 [Source:HGNC Syr	6.71E-20
10	FIS1	fission, mitochondrial 1 [Source:HGNC Symbol;Acc:HGNC:21689]	6.09E-10
10	SSBP1	single stranded DNA binding protein 1 [Source:HGNC Symbol;Acc:HG	1.46E-09
10	CD24	CD24 molecule [Source:HGNC Symbol;Acc:HGNC:1645]	2.94E-86
10	RAB13	RAB13, member RAS oncogene family [Source:HGNC Symbol;Acc:HG	4.23E-58
10	EGR1	early growth response 1 [Source:HGNC Symbol;Acc:HGNC:3238]	1.13E-25
10	PSMD8	proteasome 26S subunit, non-ATPase 8 [Source:HGNC Symbol;Acc:H	6.41E-15
10	PSME1	proteasome activator subunit 1 [Source:HGNC Symbol;Acc:HGNC:95	8.18E-09
10	EPHX1	epoxide hydrolase 1 [Source:HGNC Symbol;Acc:HGNC:3401]	3.69E-103
10	TMEM238	transmembrane protein 238 [Source:HGNC Symbol;Acc:HGNC:40042	3.64E-40
10	XBP1	X-box binding protein 1 [Source:HGNC Symbol;Acc:HGNC:12801]	8.45E-20
10	PHB	prohibitin [Source:HGNC Symbol;Acc:HGNC:8912]	1.03E-15
10	RPL36A	ribosomal protein L36a [Source:HGNC Symbol;Acc:HGNC:10359]	1.99E-15
10	IER5	immediate early response 5 [Source:HGNC Symbol;Acc:HGNC:5393]	6.19E-24
10	RNF7	ring finger protein 7 [Source:HGNC Symbol;Acc:HGNC:10070]	4.40E-15
10	CD164	CD164 molecule [Source:HGNC Symbol;Acc:HGNC:1632]	5.46E-12
10	SEC11A	SEC11 homolog A, signal peptidase complex subunit [Source:HGNC S	3.28E-09
10	LAGE3	L antigen family member 3 [Source:HGNC Symbol;Acc:HGNC:26058]	1.83E-65
10	ANAPC11	anaphase promoting complex subunit 11 [Source:HGNC Symbol;Acc:	8.87E-15
10	EZR	ezrin [Source:HGNC Symbol;Acc:HGNC:12691]	5.62E-13
10	ZFP36	ZFP36 ring finger protein [Source:HGNC Symbol;Acc:HGNC:12862]	2.98E-08
10	PSMC5	proteasome 26S subunit, ATPase 5 [Source:HGNC Symbol;Acc:HGNC	1.62E-07
10	LAPTM4A	lysosomal protein transmembrane 4 alpha [Source:HGNC Symbol;Ac	1.35E-29
10	RPN2	ribophorin II [Source:HGNC Symbol;Acc:HGNC:10382]	4.71E-27
10	KDELR1	KDEL endoplasmic reticulum protein retention receptor 1 [Source:H	1.50E-22
10	ROMO1	reactive oxygen species modulator 1 [Source:HGNC Symbol;Acc:HGN	2.01E-20
10	NDUFB5	NADH:ubiquinone oxidoreductase subunit B5 [Source:HGNC Symbol;	3.49E-13
10	PSMB6	proteasome 20S subunit beta 6 [Source:HGNC Symbol;Acc:HGNC:95	7.16E-10
10	SEC61G	SEC61 translocon subunit gamma [Source:HGNC Symbol;Acc:HGNC:1	4.63E-07
10	TMEM14C	transmembrane protein 14C [Source:HGNC Symbol;Acc:HGNC:20952	4.39E-34
10	NDUFC2	NADH:ubiquinone oxidoreductase subunit C2 [Source:HGNC Symbol;	2.15E-14
10	SNRPF	small nuclear ribonucleoprotein polypeptide F [Source:HGNC Symbo	2.32E-14
10	MRPL20	mitochondrial ribosomal protein L20 [Source:HGNC Symbol;Acc:HGN	2.09E-12
10	ZNHIT1	zinc finger HIT-type containing 1 [Source:HGNC Symbol;Acc:HGNC:21	2.17E-10
10	CAPZB	capping actin protein of muscle Z-line subunit beta [Source:HGNC Sy	2.28E-08
10	TMEM176B	transmembrane protein 176B [Source:HGNC Symbol;Acc:HGNC:2959	6.20E-50
10	PTMS	parathymosin [Source:HGNC Symbol;Acc:HGNC:9629]	6.07E-42
10	SNRPE	small nuclear ribonucleoprotein polypeptide E [Source:HGNC Symbo	2.42E-35

10	COX20	cytochrome c oxidase assembly factor COX20 [Source:HGNC Symbol;	1.83E-29
10	FAM162A	family with sequence similarity 162 member A [Source:HGNC Symbo	1.18E-28
10	SRI	sorcin [Source:HGNC Symbol;Acc:HGNC:11292]	2.24E-19
10	MYDGF	myeloid derived growth factor [Source:HGNC Symbol;Acc:HGNC:169	5.00E-18
10	GSTO1	glutathione S-transferase omega 1 [Source:HGNC Symbol;Acc:HGNC:	1.62E-15
10	COX14	cytochrome c oxidase assembly factor COX14 [Source:HGNC Symbol;	9.07E-14
10	LAMTOR1	late endosomal/lysosomal adaptor, MAPK and MTOR activator 1 [So	6.40E-11
10	ZFP36L1	ZFP36 ring finger protein like 1 [Source:HGNC Symbol;Acc:HGNC:110	1.43E-09
10	CD81	CD81 molecule [Source:HGNC Symbol;Acc:HGNC:1701]	2.90E-09
10	TALDO1	transaldolase 1 [Source:HGNC Symbol;Acc:HGNC:11559]	8.01E-09
10	LRRC26	leucine rich repeat containing 26 [Source:HGNC Symbol;Acc:HGNC:3	1.53E-82
10	SLC12A2	solute carrier family 12 member 2 [Source:HGNC Symbol;Acc:HGNC:	7.56E-78
10	TXNDC17	thioredoxin domain containing 17 [Source:HGNC Symbol;Acc:HGNC:2	1.49E-46
10	IDH2	isocitrate dehydrogenase (NADP(+)) 2 [Source:HGNC Symbol;Acc:HG	2.84E-39
10	NDUFA3	NADH:ubiquinone oxidoreductase subunit A3 [Source:HGNC Symbol;	5.34E-15
10	LMAN2	lectin, mannose binding 2 [Source:HGNC Symbol;Acc:HGNC:16986]	4.53E-12
10	NOL7	nucleolar protein 7 [Source:HGNC Symbol;Acc:HGNC:21040]	2.62E-07
10	S100P	S100 calcium binding protein P [Source:HGNC Symbol;Acc:HGNC:105	9.26E-79
10	FBP1	fructose-bisphosphatase 1 [Source:HGNC Symbol;Acc:HGNC:3606]	8.43E-49
10	RAP1B	RAP1B, member of RAS oncogene family [Source:HGNC Symbol;Acc:	1.05E-40
10	NME3	NME/NM23 nucleoside diphosphate kinase 3 [Source:HGNC Symbol;	1.10E-20
10	TMCO1	transmembrane and coiled-coil domains 1 [Source:HGNC Symbol;Acc	1.71E-17
10	TIMM10	translocase of inner mitochondrial membrane 10 [Source:HGNC Sym	1.42E-45
10	ATOX1	antioxidant 1 copper chaperone [Source:HGNC Symbol;Acc:HGNC:79	1.72E-38
10	TMED3	transmembrane p24 trafficking protein 3 [Source:HGNC Symbol;Acc:	8.80E-31
10	BLVRB	biliverdin reductase B [Source:HGNC Symbol;Acc:HGNC:1063]	1.16E-29
10	KIF5B	kinesin family member 5B [Source:HGNC Symbol;Acc:HGNC:6324]	2.12E-18
10	CCT3	chaperonin containing TCP1 subunit 3 [Source:HGNC Symbol;Acc:HG	8.56E-11
10	PPIG	peptidylprolyl isomerase G [Source:HGNC Symbol;Acc:HGNC:14650]	4.84E-08
10	SSU72	SSU72 homolog, RNA polymerase II CTD phosphatase [Source:HGNC	2.32E-07
10	AZGP1	alpha-2-glycoprotein 1, zinc-binding [Source:HGNC Symbol;Acc:HGN	1.83E-96
10	CRACR2B	calcium release activated channel regulator 2B [Source:HGNC Symbo	6.79E-87
10	TMEM205	transmembrane protein 205 [Source:HGNC Symbol;Acc:HGNC:29631	6.47E-47
10	TIMM13	translocase of inner mitochondrial membrane 13 [Source:HGNC Sym	5.36E-19
10	LAMTOR5	late endosomal/lysosomal adaptor, MAPK and MTOR activator 5 [So	3.09E-18
10	GSN	gelsolin [Source:HGNC Symbol;Acc:HGNC:4620]	5.29E-18
10	CALM3	calmodulin 3 [Source:HGNC Symbol;Acc:HGNC:1449]	4.05E-15
10	MRPS34	mitochondrial ribosomal protein S34 [Source:HGNC Symbol;Acc:HGN	2.08E-14
10	PDCD5	programmed cell death 5 [Source:HGNC Symbol;Acc:HGNC:8764]	2.15E-09
10	MRPS21	mitochondrial ribosomal protein S21 [Source:HGNC Symbol;Acc:HGN	1.99E-07
10	PSMA2	proteasome 20S subunit alpha 2 [Source:HGNC Symbol;Acc:HGNC:95	4.84E-07
10	TPM4	tropomyosin 4 [Source:HGNC Symbol;Acc:HGNC:12013]	1.10E-10
10	WFDC2	WAP four-disulfide core domain 2 [Source:HGNC Symbol;Acc:HGNC:1	6.14E-99
10	C1orf43	chromosome 1 open reading frame 43 [Source:HGNC Symbol;Acc:HG	6.41E-40
10	C19orf33	chromosome 19 open reading frame 33 [Source:HGNC Symbol;Acc:H	1.09E-28
10	MPC2	mitochondrial pyruvate carrier 2 [Source:HGNC Symbol;Acc:HGNC:24	3.65E-26
10	ATRAID	all-trans retinoic acid induced differentiation factor [Source:HGNC Sy	5.94E-22

10	ATP5MC1	ATP synthase membrane subunit c locus 1 [Source:HGNC Symbol;Acc	1.05E-13
10	ETFB	electron transfer flavoprotein subunit beta [Source:HGNC Symbol;Ac	2.14E-11
10	BUB3	BUB3 mitotic checkpoint protein [Source:HGNC Symbol;Acc:HGNC:1	3.91E-10
10	PDIA6	protein disulfide isomerase family A member 6 [Source:HGNC Symbo	3.95E-10
10	ATP1B1	ATPase Na+/K+ transporting subunit beta 1 [Source:HGNC Symbol;Ac	6.25E-42
10	C1orf122	chromosome 1 open reading frame 122 [Source:HGNC Symbol;Acc:H	3.17E-40
10	SDHC	succinate dehydrogenase complex subunit C [Source:HGNC Symbol;A	7.76E-36
10	MFSD10	major facilitator superfamily domain containing 10 [Source:HGNC Sy	6.81E-31
10	RNF181	ring finger protein 181 [Source:HGNC Symbol;Acc:HGNC:28037]	1.77E-28
10	MIEN1	migration and invasion enhancer 1 [Source:HGNC Symbol;Acc:HGNC:	3.61E-23
10	OCIAD2	OCIA domain containing 2 [Source:HGNC Symbol;Acc:HGNC:28685]	6.59E-09
10	UBXN4	UBX domain protein 4 [Source:HGNC Symbol;Acc:HGNC:14860]	3.38E-08
10	PERP	p53 apoptosis effector related to PMP22 [Source:HGNC Symbol;Acc:	7.23E-82
10	RAB25	RAB25, member RAS oncogene family [Source:HGNC Symbol;Acc:HG	2.95E-79
10	SPTSSB	serine palmitoyltransferase small subunit B [Source:HGNC Symbol;Ac	4.56E-76
10	ZG16B	zymogen granule protein 16B [Source:HGNC Symbol;Acc:HGNC:3045	7.52E-75
10	HES4	hes family bHLH transcription factor 4 [Source:HGNC Symbol;Acc:HG	4.22E-48
10	PNKD	PNKD metallo-beta-lactamase domain containing [Source:HGNC Sym	2.08E-24
10	FKBP2	FKBP prolyl isomerase 2 [Source:HGNC Symbol;Acc:HGNC:3718]	1.14E-17
10	LSM4	LSM4 homolog, U6 small nuclear RNA and mRNA degradation associ	9.68E-16
10	SNRPD1	small nuclear ribonucleoprotein D1 polypeptide [Source:HGNC Symb	7.04E-09
10	MIF	macrophage migration inhibitory factor [Source:HGNC Symbol;Acc:H	1.13E-07
10	CLDN3	claudin 3 [Source:HGNC Symbol;Acc:HGNC:2045]	9.83E-89
10	ALOX15B	arachidonate 15-lipoxygenase type B [Source:HGNC Symbol;Acc:HGN	2.68E-87
10	SMIM22	small integral membrane protein 22 [Source:HGNC Symbol;Acc:HGN	9.23E-86
10	RCAN1	regulator of calcineurin 1 [Source:HGNC Symbol;Acc:HGNC:3040]	1.73E-81
10	CAPG	capping actin protein, gelsolin like [Source:HGNC Symbol;Acc:HGNC:	5.00E-23
10	NDUFV1	NADH:ubiquinone oxidoreductase core subunit V1 [Source:HGNC Sy	4.31E-21
10	COMT	catechol-O-methyltransferase [Source:HGNC Symbol;Acc:HGNC:222	7.04E-19
10	CD46	CD46 molecule [Source:HGNC Symbol;Acc:HGNC:6953]	6.28E-17
10	MEAF6	MYST/Esa1 associated factor 6 [Source:HGNC Symbol;Acc:HGNC:256	1.08E-13
10	SMIM26	small integral membrane protein 26 [Source:HGNC Symbol;Acc:HGN	3.39E-13
10	UQCRFS1	ubiquinol-cytochrome c reductase, Rieske iron-sulfur polypeptide 1 [	8.85E-12
10	SBDS	SBDS ribosome maturation factor [Source:HGNC Symbol;Acc:HGNC:1	2.83E-11
10	TUBB	tubulin beta class I [Source:HGNC Symbol;Acc:HGNC:20778]	4.05E-10
10	TOB1	transducer of ERBB2, 1 [Source:HGNC Symbol;Acc:HGNC:11979]	1.64E-09
10	SPDEF	SAM pointed domain containing ETS transcription factor [Source:HG	1.84E-86
10	COA3	cytochrome c oxidase assembly factor 3 [Source:HGNC Symbol;Acc:H	8.66E-58
10	GALNT7	polypeptide N-acetylgalactosaminyltransferase 7 [Source:HGNC Sym	1.65E-55
10	NAXE	NAD(P)HX epimerase [Source:HGNC Symbol;Acc:HGNC:18453]	3.14E-39
10	ARPC5	actin related protein 2/3 complex subunit 5 [Source:HGNC Symbol;A	4.09E-38
10	ECHS1	enoyl-CoA hydratase, short chain 1 [Source:HGNC Symbol;Acc:HGNC	4.77E-25
10	TECR	trans-2,3-enoyl-CoA reductase [Source:HGNC Symbol;Acc:HGNC:455	5.67E-19
10	HSPA1A	heat shock protein family A (Hsp70) member 1A [Source:HGNC Symb	4.55E-13
10	COPS6	COP9 signalosome subunit 6 [Source:HGNC Symbol;Acc:HGNC:2174	1.17E-12
10	PDCD6	programmed cell death 6 [Source:HGNC Symbol;Acc:HGNC:8765]	1.18E-12
10	SRP72	signal recognition particle 72 [Source:HGNC Symbol;Acc:HGNC:1130	1.30E-07

10	DAPL1	death associated protein like 1 [Source:HGNC Symbol;Acc:HGNC:214	1.69E-73
10	HES1	hes family bHLH transcription factor 1 [Source:HGNC Symbol;Acc:HG	1.90E-37
10	DDAH2	dimethylarginine dimethylaminohydrolase 2 [Source:HGNC Symbol;A	1.09E-33
10	POLR2I	RNA polymerase II subunit I [Source:HGNC Symbol;Acc:HGNC:9196]	8.59E-33
10	TCEAL4	transcription elongation factor A like 4 [Source:HGNC Symbol;Acc:HG	5.14E-32
10	NDUFS2	NADH:ubiquinone oxidoreductase core subunit S2 [Source:HGNC Syr	1.90E-31
10	MRPL33	mitochondrial ribosomal protein L33 [Source:HGNC Symbol;Acc:HGN	9.13E-17
10	NDUFA5	NADH:ubiquinone oxidoreductase subunit A5 [Source:HGNC Symbol;	2.98E-13
10	DUT	deoxyuridine triphosphatase [Source:HGNC Symbol;Acc:HGNC:3078]	7.82E-13
10	NDUFA10	NADH:ubiquinone oxidoreductase subunit A10 [Source:HGNC Symbo	9.79E-13
10	TMEM50A	transmembrane protein 50A [Source:HGNC Symbol;Acc:HGNC:3059(	9.51E-11
10	CCDC190	coiled-coil domain containing 190 [Source:HGNC Symbol;Acc:HGNC:2	1.41E-85
10	TRPS1	transcriptional repressor GATA binding 1 [Source:HGNC Symbol;Acc:	9.27E-60
10	PMVK	phosphomevalonate kinase [Source:HGNC Symbol;Acc:HGNC:9141]	2.28E-39
10	TSC22D1	TSC22 domain family member 1 [Source:HGNC Symbol;Acc:HGNC:16	2.35E-11
10	SSNA1	SS nuclear autoantigen 1 [Source:HGNC Symbol;Acc:HGNC:11321]	9.73E-11
10	PFDN2	prefoldin subunit 2 [Source:HGNC Symbol;Acc:HGNC:8867]	1.10E-10
10	TTC3	tetratricopeptide repeat domain 3 [Source:HGNC Symbol;Acc:HGNC:	1.17E-09
10	PHPT1	phosphohistidine phosphatase 1 [Source:HGNC Symbol;Acc:HGNC:3(	2.29E-08
10	PITX1	paired like homeodomain 1 [Source:HGNC Symbol;Acc:HGNC:9004]	1.67E-82
10	MIA3	MIA SH3 domain ER export factor 3 [Source:HGNC Symbol;Acc:HGNC	4.53E-29
10	DCXR	dicarbonyl and L-xylulose reductase [Source:HGNC Symbol;Acc:HGN(	2.59E-22
10	HSD17B10	hydroxysteroid 17-beta dehydrogenase 10 [Source:HGNC Symbol;Ac	1.15E-19
10	GOLGB1	golgin B1 [Source:HGNC Symbol;Acc:HGNC:4429]	2.96E-14
10	NFKBIA	NFKB inhibitor alpha [Source:HGNC Symbol;Acc:HGNC:7797]	2.65E-13
10	PPP1R15A	protein phosphatase 1 regulatory subunit 15A [Source:HGNC Symbol	2.33E-12
10	VDAC1	voltage dependent anion channel 1 [Source:HGNC Symbol;Acc:HGNC	4.22E-11
10	OAT	ornithine aminotransferase [Source:HGNC Symbol;Acc:HGNC:8091]	1.03E-38
10	SMIM14	small integral membrane protein 14 [Source:HGNC Symbol;Acc:HGN(	1.02E-33
10	NDUFA8	NADH:ubiquinone oxidoreductase subunit A8 [Source:HGNC Symbol;	2.09E-20
10	TIMM8B	translocase of inner mitochondrial membrane 8 homolog B [Source:H	1.21E-19
10	RPL17	ribosomal protein L17 [Source:HGNC Symbol;Acc:HGNC:10307]	1.60E-11
10	GOLGA4	golgin A4 [Source:HGNC Symbol;Acc:HGNC:4427]	3.20E-11
10	UQCRC2	ubiquinol-cytochrome c reductase core protein 2 [Source:HGNC Sym	7.46E-11
10	MRPL54	mitochondrial ribosomal protein L54 [Source:HGNC Symbol;Acc:HGN	1.75E-08
10	CLU	clusterin [Source:HGNC Symbol;Acc:HGNC:2095]	6.22E-07
10	DSP	desmoplakin [Source:HGNC Symbol;Acc:HGNC:3052]	1.51E-70
10	NANS	N-acetylneuraminate synthase [Source:HGNC Symbol;Acc:HGNC:192	1.19E-28
10	SDF2L1	stromal cell derived factor 2 like 1 [Source:HGNC Symbol;Acc:HGNC:	4.92E-22
10	RNF19A	ring finger protein 19A, RBR E3 ubiquitin protein ligase [Source:HGN(	1.57E-17
10	LRPAP1	LDL receptor related protein associated protein 1 [Source:HGNC Sym	1.15E-14
10	DCTN3	dynactin subunit 3 [Source:HGNC Symbol;Acc:HGNC:2713]	4.56E-13
10	MTPN	myotrophin [Source:HGNC Symbol;Acc:HGNC:15667]	1.54E-12
10	PIN1	peptidylprolyl cis/trans isomerase, NIMA-interacting 1 [Source:HGN(	6.15E-12
10	OCIAD1	OCIA domain containing 1 [Source:HGNC Symbol;Acc:HGNC:16074]	5.38E-11
10	TMEM256	transmembrane protein 256 [Source:HGNC Symbol;Acc:HGNC:28618	5.30E-09
10	CCT2	chaperonin containing TCP1 subunit 2 [Source:HGNC Symbol;Acc:HG	1.16E-08

10	CDK2AP2	cyclin dependent kinase 2 associated protein 2 [Source:HGNC Symbo	2.86E-07
10	RGS10	regulator of G protein signaling 10 [Source:HGNC Symbol;Acc:HGNC:	4.39E-29
10	DYNLT1	dynein light chain Tctex-type 1 [Source:HGNC Symbol;Acc:HGNC:116	1.98E-23
10	NDUFS4	NADH:ubiquinone oxidoreductase subunit S4 [Source:HGNC Symbol;	1.86E-15
10	BUD31	BUD31 homolog [Source:HGNC Symbol;Acc:HGNC:29629]	6.75E-11
10	NHP2	NHP2 ribonucleoprotein [Source:HGNC Symbol;Acc:HGNC:14377]	6.45E-10
10	CCT5	chaperonin containing TCP1 subunit 5 [Source:HGNC Symbol;Acc:HG	3.02E-07
10	EMP2	epithelial membrane protein 2 [Source:HGNC Symbol;Acc:HGNC:333	1.30E-76
10	PHLDA2	pleckstrin homology like domain family A member 2 [Source:HGNC S	2.66E-56
10	BAMBI	BMP and activin membrane bound inhibitor [Source:HGNC Symbol;A	2.06E-38
10	NPDC1	neural proliferation, differentiation and control 1 [Source:HGNC Sym	9.11E-32
10	COMMD3	COMM domain containing 3 [Source:HGNC Symbol;Acc:HGNC:23332	2.11E-29
10	HINT2	histidine triad nucleotide binding protein 2 [Source:HGNC Symbol;Ac	1.01E-26
10	RER1	retention in endoplasmic reticulum sorting receptor 1 [Source:HGNC	1.67E-18
10	SLC50A1	solute carrier family 50 member 1 [Source:HGNC Symbol;Acc:HGNC:	2.12E-17
10	LSM2	LSM2 homolog, U6 small nuclear RNA and mRNA degradation associ	1.38E-12
10	REXO2	RNA exonuclease 2 [Source:HGNC Symbol;Acc:HGNC:17851]	8.99E-08
10	HACD3	3-hydroxyacyl-CoA dehydratase 3 [Source:HGNC Symbol;Acc:HGNC:2	7.24E-34
10	SELENOM	selenoprotein M [Source:HGNC Symbol;Acc:HGNC:30397]	6.79E-33
10	MRPL55	mitochondrial ribosomal protein L55 [Source:HGNC Symbol;Acc:HGN	1.06E-28
10	DPM3	dolichyl-phosphate mannosyltransferase subunit 3, regulatory [Sourc	3.87E-24
10	DDRGK1	DDRGK domain containing 1 [Source:HGNC Symbol;Acc:HGNC:16110	4.84E-23
10	CENPX	centromere protein X [Source:HGNC Symbol;Acc:HGNC:11422]	2.54E-21
10	SMS	spermine synthase [Source:HGNC Symbol;Acc:HGNC:11123]	2.49E-14
10	CNPY2	canopy FGF signaling regulator 2 [Source:HGNC Symbol;Acc:HGNC:13	2.36E-12
10	ACTR3	actin related protein 3 [Source:HGNC Symbol;Acc:HGNC:170]	3.38E-09
10	PRMT1	protein arginine methyltransferase 1 [Source:HGNC Symbol;Acc:HGN	5.33E-09
10	RAB2A	RAB2A, member RAS oncogene family [Source:HGNC Symbol;Acc:HG	1.84E-08
10	ARPC5L	actin related protein 2/3 complex subunit 5 like [Source:HGNC Symb	4.49E-07
10	SPINT1	serine peptidase inhibitor, Kunitz type 1 [Source:HGNC Symbol;Acc:H	5.32E-61
10	CAPS	calcyphosine [Source:HGNC Symbol;Acc:HGNC:1487]	1.52E-54
10	MGST1	microsomal glutathione S-transferase 1 [Source:HGNC Symbol;Acc:H	1.67E-46
10	TMEM176A	transmembrane protein 176A [Source:HGNC Symbol;Acc:HGNC:2493	6.59E-38
10	TMEM63A	transmembrane protein 63A [Source:HGNC Symbol;Acc:HGNC:29118	4.01E-31
10	MLF2	myeloid leukemia factor 2 [Source:HGNC Symbol;Acc:HGNC:7126]	9.55E-31
10	RNF187	ring finger protein 187 [Source:HGNC Symbol;Acc:HGNC:27146]	7.58E-22
10	TCEAL3	transcription elongation factor A like 3 [Source:HGNC Symbol;Acc:HG	1.32E-21
10	MPG	N-methylpurine DNA glycosylase [Source:HGNC Symbol;Acc:HGNC:7	9.21E-12
10	POLR2K	RNA polymerase II, I and III subunit K [Source:HGNC Symbol;Acc:HGN	1.17E-09
10	RBX1	ring-box 1 [Source:HGNC Symbol;Acc:HGNC:9928]	1.72E-09
10	TMED4	transmembrane p24 trafficking protein 4 [Source:HGNC Symbol;Acc:	1.58E-08
10	TMED9	transmembrane p24 trafficking protein 9 [Source:HGNC Symbol;Acc:	6.23E-08
10	MRPS26	mitochondrial ribosomal protein S26 [Source:HGNC Symbol;Acc:HGN	1.57E-16
10	MRPS15	mitochondrial ribosomal protein S15 [Source:HGNC Symbol;Acc:HGN	1.71E-15
10	UBE2D3	ubiquitin conjugating enzyme E2 D3 [Source:HGNC Symbol;Acc:HGN	1.21E-10
10	MGMT	O-6-methylguanine-DNA methyltransferase [Source:HGNC Symbol;A	1.56E-10
10	COPZ1	COPI coat complex subunit zeta 1 [Source:HGNC Symbol;Acc:HGNC:2	1.98E-10

10	SERPINB6	serpin family B member 6 [Source:HGNC Symbol;Acc:HGNC:8950]	5.68E-08
10	TFAP2B	transcription factor AP-2 beta [Source:HGNC Symbol;Acc:HGNC:1174	6.20E-70
10	ECI1	enoyl-CoA delta isomerase 1 [Source:HGNC Symbol;Acc:HGNC:2703]	2.39E-31
10	PPCS	phosphopantothenoylcysteine synthetase [Source:HGNC Symbol;Acc	1.16E-20
10	TMEM134	transmembrane protein 134 [Source:HGNC Symbol;Acc:HGNC:26142	3.39E-18
10	PTRHD1	peptidyl-tRNA hydrolase domain containing 1 [Source:HGNC Symbol;	1.01E-14
10	HLA-F	major histocompatibility complex, class I, F [Source:HGNC Symbol;Ac	1.50E-07
10	TFF3	trefoil factor 3 [Source:HGNC Symbol;Acc:HGNC:11757]	1.38E-77
10	GIPC1	GIPC PDZ domain containing family member 1 [Source:HGNC Symbo	1.64E-32
10	DUSP23	dual specificity phosphatase 23 [Source:HGNC Symbol;Acc:HGNC:21	3.24E-28
10	S100A4	S100 calcium binding protein A4 [Source:HGNC Symbol;Acc:HGNC:10	7.74E-22
10	DNAJC19	DnaJ heat shock protein family (Hsp40) member C19 [Source:HGNC S	6.24E-15
10	IMPDH2	inosine monophosphate dehydrogenase 2 [Source:HGNC Symbol;Acc	2.87E-12
10	MRPL14	mitochondrial ribosomal protein L14 [Source:HGNC Symbol;Acc:HGN	7.56E-10
10	PNISR	PNN interacting serine and arginine rich protein [Source:HGNC Symb	2.86E-07
10	TFF1	trefoil factor 1 [Source:HGNC Symbol;Acc:HGNC:11755]	5.49E-75
10	SOX9	SRY-box transcription factor 9 [Source:HGNC Symbol;Acc:HGNC:1120	1.39E-65
10	CREB3L4	cAMP responsive element binding protein 3 like 4 [Source:HGNC Sym	2.90E-57
10	PRDX4	peroxiredoxin 4 [Source:HGNC Symbol;Acc:HGNC:17169]	2.38E-51
10	CCL28	C-C motif chemokine ligand 28 [Source:HGNC Symbol;Acc:HGNC:177	4.94E-46
10	GNAI2	G protein subunit alpha i2 [Source:HGNC Symbol;Acc:HGNC:4385]	8.55E-20
10	RALA	RAS like proto-oncogene A [Source:HGNC Symbol;Acc:HGNC:9839]	2.48E-19
10	BHLHE40	basic helix-loop-helix family member e40 [Source:HGNC Symbol;Acc:	3.42E-13
10	MRPL21	mitochondrial ribosomal protein L21 [Source:HGNC Symbol;Acc:HGN	3.94E-09
10	NDUFAF8	NADH:ubiquinone oxidoreductase complex assembly factor 8 [Sourc	4.86E-09
10	CTSA	cathepsin A [Source:HGNC Symbol;Acc:HGNC:9251]	1.01E-07
10	SUCLG1	succinate-CoA ligase GDP/ADP-forming subunit alpha [Source:HGNC	1.07E-07
10	MRPL24	mitochondrial ribosomal protein L24 [Source:HGNC Symbol;Acc:HGN	2.81E-30
10	TMEM147	transmembrane protein 147 [Source:HGNC Symbol;Acc:HGNC:30414	6.88E-26
10	KIF2A	kinesin family member 2A [Source:HGNC Symbol;Acc:HGNC:6318]	5.81E-24
10	METTL5	methyltransferase like 5 [Source:HGNC Symbol;Acc:HGNC:25006]	1.35E-13
10	PSMB7	proteasome 20S subunit beta 7 [Source:HGNC Symbol;Acc:HGNC:95	2.37E-13
10	SELENOS	selenoprotein S [Source:HGNC Symbol;Acc:HGNC:30396]	1.61E-11
10	DDIT3	DNA damage inducible transcript 3 [Source:HGNC Symbol;Acc:HGNC	2.79E-08
10	IRS4	insulin receptor substrate 4 [Source:HGNC Symbol;Acc:HGNC:6128]	1.20E-72
10	KRT23	keratin 23 [Source:HGNC Symbol;Acc:HGNC:6438]	1.36E-63
10	NQO1	NAD(P)H quinone dehydrogenase 1 [Source:HGNC Symbol;Acc:HGNC	8.61E-58
10	SH3YL1	SH3 and SYLF domain containing 1 [Source:HGNC Symbol;Acc:HGNC:	7.55E-28
10	YIPF3	Yip1 domain family member 3 [Source:HGNC Symbol;Acc:HGNC:210	1.21E-26
10	GRHPR	glyoxylate and hydroxypyruvate reductase [Source:HGNC Symbol;Ac	5.60E-18
10	SLIRP	SRA stem-loop interacting RNA binding protein [Source:HGNC Symbo	5.23E-17
10	ILF2	interleukin enhancer binding factor 2 [Source:HGNC Symbol;Acc:HGN	7.74E-16
10	MRPS36	mitochondrial ribosomal protein S36 [Source:HGNC Symbol;Acc:HGN	7.35E-13
10	CAP1	cyclase associated actin cytoskeleton regulatory protein 1 [Source:H	5.05E-12
10	MRPL43	mitochondrial ribosomal protein L43 [Source:HGNC Symbol;Acc:HGN	5.43E-12
10	BAG1	BAG cochaperone 1 [Source:HGNC Symbol;Acc:HGNC:937]	1.56E-11
10	CMC1	C-X9-C motif containing 1 [Source:HGNC Symbol;Acc:HGNC:28783]	4.01E-09

10	SNF8	SNF8 subunit of ESCRT-II [Source:HGNC Symbol;Acc:HGNC:17028]	9.20E-09
10	NR2F2	nuclear receptor subfamily 2 group F member 2 [Source:HGNC Symb	6.46E-62
10	SPINT1-AS1	SPINT1 antisense RNA 1 [Source:HGNC Symbol;Acc:HGNC:53162]	1.35E-51
10	DHCR24	24-dehydrocholesterol reductase [Source:HGNC Symbol;Acc:HGNC:2	2.71E-45
10	ARPC1A	actin related protein 2/3 complex subunit 1A [Source:HGNC Symbol;	1.81E-29
10	EBNA1BP2	EBNA1 binding protein 2 [Source:HGNC Symbol;Acc:HGNC:15531]	1.76E-26
10	DCTPP1	dCTP pyrophosphatase 1 [Source:HGNC Symbol;Acc:HGNC:28777]	1.83E-25
10	ACADVL	acyl-CoA dehydrogenase very long chain [Source:HGNC Symbol;Acc:H	7.32E-24
10	ARPC4	actin related protein 2/3 complex subunit 4 [Source:HGNC Symbol;A	1.50E-12
10	VEGFB	vascular endothelial growth factor B [Source:HGNC Symbol;Acc:HGN	8.70E-11
10	COA4	cytochrome c oxidase assembly factor 4 homolog [Source:HGNC Sym	1.37E-10
10	VKORC1	vitamin K epoxide reductase complex subunit 1 [Source:HGNC Symbo	3.41E-10
10	NDUFS3	NADH:ubiquinone oxidoreductase core subunit S3 [Source:HGNC Syr	9.10E-09
10	CBX3	chromobox 3 [Source:HGNC Symbol;Acc:HGNC:1553]	1.78E-07
10	QSOX1	quiescin sulfhydryl oxidase 1 [Source:HGNC Symbol;Acc:HGNC:9756]	4.06E-37
10	MORF4L2	mortality factor 4 like 2 [Source:HGNC Symbol;Acc:HGNC:16849]	2.24E-28
10	TMEM165	transmembrane protein 165 [Source:HGNC Symbol;Acc:HGNC:30760	6.92E-27
10	METRN	meteorin, glial cell differentiation regulator [Source:HGNC Symbol;A	1.35E-25
10	ATP6AP1	ATPase H+ transporting accessory protein 1 [Source:HGNC Symbol;A	4.26E-24
10	IGSF8	immunoglobulin superfamily member 8 [Source:HGNC Symbol;Acc:H	6.63E-24
10	CBR1	carbonyl reductase 1 [Source:HGNC Symbol;Acc:HGNC:1548]	2.23E-18
10	CPNE3	copine 3 [Source:HGNC Symbol;Acc:HGNC:2316]	3.52E-16
10	LYPLA1	lysophospholipase 1 [Source:HGNC Symbol;Acc:HGNC:6737]	3.57E-13
10	BLVRA	biliverdin reductase A [Source:HGNC Symbol;Acc:HGNC:1062]	1.17E-12
10	APH1A	aph-1 homolog A, gamma-secretase subunit [Source:HGNC Symbol;A	1.40E-12
10	NCOA7	nuclear receptor coactivator 7 [Source:HGNC Symbol;Acc:HGNC:210	6.48E-12
10	ETHE1	ETHE1 persulfide dioxygenase [Source:HGNC Symbol;Acc:HGNC:232	3.64E-10
10	FAAP20	FA core complex associated protein 20 [Source:HGNC Symbol;Acc:H	1.35E-07
10	SAR1A	secretion associated Ras related GTPase 1A [Source:HGNC Symbol;A	3.74E-07
10	SERINC2	serine incorporator 2 [Source:HGNC Symbol;Acc:HGNC:23231]	1.67E-58
10	NT5DC2	5'-nucleotidase domain containing 2 [Source:HGNC Symbol;Acc:HGN	1.76E-49
10	TLN1	talin 1 [Source:HGNC Symbol;Acc:HGNC:11845]	7.61E-40
10	MBNL1	muscleblind like splicing regulator 1 [Source:HGNC Symbol;Acc:HGN	7.05E-30
10	TCEA3	transcription elongation factor A3 [Source:HGNC Symbol;Acc:HGNC:	1.66E-27
10	ARID5A	AT-rich interaction domain 5A [Source:HGNC Symbol;Acc:HGNC:173	1.04E-19
10	SYPL1	synaptophysin like 1 [Source:HGNC Symbol;Acc:HGNC:11507]	8.77E-18
10	MEA1	male-enhanced antigen 1 [Source:HGNC Symbol;Acc:HGNC:6986]	1.25E-15
10	SYAP1	synapse associated protein 1 [Source:HGNC Symbol;Acc:HGNC:1627	4.94E-15
10	ATP6V0E2	ATPase H+ transporting V0 subunit e2 [Source:HGNC Symbol;Acc:HG	7.83E-14
10	PIN4	peptidylprolyl cis/trans isomerase, NIMA-interacting 4 [Source:HGN	6.43E-13
10	YWHAH	tyrosine 3-monooxygenase/tryptophan 5-monooxygenase activation	7.01E-12
10	TMEM183A	transmembrane protein 183A [Source:HGNC Symbol;Acc:HGNC:2017	1.49E-10
10	NINJ1	ninjurin 1 [Source:HGNC Symbol;Acc:HGNC:7824]	2.11E-08
10	BCAS2	BCAS2 pre-mRNA processing factor [Source:HGNC Symbol;Acc:HGN	2.16E-07
10	SEC11C	SEC11 homolog C, signal peptidase complex subunit [Source:HGNC S	2.50E-07
10	WASL	WASP like actin nucleation promoting factor [Source:HGNC Symbol;A	2.78E-30
10	PEA15	proliferation and apoptosis adaptor protein 15 [Source:HGNC Symbo	5.44E-20

10	TMEM87A	transmembrane protein 87A [Source:HGNC Symbol;Acc:HGNC:24522	8.48E-17
10	EIF4EBP1	eukaryotic translation initiation factor 4E binding protein 1 [Source:H	1.98E-16
10	ERLEC1	endoplasmic reticulum lectin 1 [Source:HGNC Symbol;Acc:HGNC:252	2.08E-13
10	S100A9	S100 calcium binding protein A9 [Source:HGNC Symbol;Acc:HGNC:10	6.98E-12
10	TIMM17B	translocase of inner mitochondrial membrane 17B [Source:HGNC Sy	7.75E-12
10	YPEL5	yippee like 5 [Source:HGNC Symbol;Acc:HGNC:18329]	2.03E-10
10	PUF60	poly(U) binding splicing factor 60 [Source:HGNC Symbol;Acc:HGNC:1	7.41E-10
10	PSMB9	proteasome 20S subunit beta 9 [Source:HGNC Symbol;Acc:HGNC:954	1.71E-09
10	CYB5R1	cytochrome b5 reductase 1 [Source:HGNC Symbol;Acc:HGNC:13397]	1.75E-09
10	AUP1	AUP1 lipid droplet regulating VLDL assembly factor [Source:HGNC Sy	1.33E-08
10	TKT	transketolase [Source:HGNC Symbol;Acc:HGNC:11834]	1.89E-07
10	SIL1	SIL1 nucleotide exchange factor [Source:HGNC Symbol;Acc:HGNC:24	1.20E-32
10	CKS1B	CDC28 protein kinase regulatory subunit 1B [Source:HGNC Symbol;A	2.07E-27
10	CD99	CD99 molecule (Xg blood group) [Source:HGNC Symbol;Acc:HGNC:70	4.17E-24
10	TUBA4A	tubulin alpha 4a [Source:HGNC Symbol;Acc:HGNC:12407]	9.63E-19
10	CNIH4	cornichon family AMPA receptor auxiliary protein 4 [Source:HGNC Sy	7.25E-15
10	RRBP1	ribosome binding protein 1 [Source:HGNC Symbol;Acc:HGNC:10448]	4.01E-12
10	SLC2A4RG	SLC2A4 regulator [Source:HGNC Symbol;Acc:HGNC:15930]	7.48E-11
10	MOB1A	MOB kinase activator 1A [Source:HGNC Symbol;Acc:HGNC:16015]	1.39E-10
10	MRPS7	mitochondrial ribosomal protein S7 [Source:HGNC Symbol;Acc:HGNC	8.23E-10
10	NUDT16L1	nudix hydrolase 16 like 1 [Source:HGNC Symbol;Acc:HGNC:28154]	2.37E-09
10	BUD23	BUD23 rRNA methyltransferase and ribosome maturation factor [Sou	2.27E-08
10	MLEC	malectin [Source:HGNC Symbol;Acc:HGNC:28973]	2.43E-07
10	BCL7C	BAF chromatin remodeling complex subunit BCL7C [Source:HGNC Sy	5.39E-07
10	PRSS8	serine protease 8 [Source:HGNC Symbol;Acc:HGNC:9491]	4.11E-60
10	EFHD1	EF-hand domain family member D1 [Source:HGNC Symbol;Acc:HGNC	2.06E-59
10	PLPP2	phospholipid phosphatase 2 [Source:HGNC Symbol;Acc:HGNC:9230]	7.61E-56
10	SLC39A1	solute carrier family 39 member 1 [Source:HGNC Symbol;Acc:HGNC:	4.63E-30
10	C11orf1	chromosome 11 open reading frame 1 [Source:HGNC Symbol;Acc:HG	1.82E-23
10	AK2	adenylate kinase 2 [Source:HGNC Symbol;Acc:HGNC:362]	3.27E-17
10	SLC25A39	solute carrier family 25 member 39 [Source:HGNC Symbol;Acc:HGNC	1.69E-16
10	HDLBP	high density lipoprotein binding protein [Source:HGNC Symbol;Acc:H	1.82E-15
10	MRPL13	mitochondrial ribosomal protein L13 [Source:HGNC Symbol;Acc:HGN	1.17E-13
10	EMC7	ER membrane protein complex subunit 7 [Source:HGNC Symbol;Acc:	1.25E-13
10	NAA20	N-alpha-acetyltransferase 20, NatB catalytic subunit [Source:HGNC S	3.44E-13
10	CCDC90B	coiled-coil domain containing 90B [Source:HGNC Symbol;Acc:HGNC:2	2.25E-12
10	STAU1	staufen double-stranded RNA binding protein 1 [Source:HGNC Symb	6.52E-10
10	SERPINB1	serpin family B member 1 [Source:HGNC Symbol;Acc:HGNC:3311]	9.14E-08
10	MXD4	MAX dimerization protein 4 [Source:HGNC Symbol;Acc:HGNC:13906]	9.46E-08
10	CPE	carboxypeptidase E [Source:HGNC Symbol;Acc:HGNC:2303]	3.84E-53
10	ENAH	ENAH actin regulator [Source:HGNC Symbol;Acc:HGNC:18271]	1.39E-46
10	CCND3	cyclin D3 [Source:HGNC Symbol;Acc:HGNC:1585]	1.47E-33
10	EI24	EI24 autophagy associated transmembrane protein [Source:HGNC Sy	6.45E-33
10	CSRP1	cysteine and glycine rich protein 1 [Source:HGNC Symbol;Acc:HGNC:	2.31E-18
10	UROS	uroporphyrinogen III synthase [Source:HGNC Symbol;Acc:HGNC:125	7.16E-14
10	NME4	NME/NM23 nucleoside diphosphate kinase 4 [Source:HGNC Symbol;	3.06E-13
10	ORMDL2	ORMDL sphingolipid biosynthesis regulator 2 [Source:HGNC Symbol;	1.50E-12

10	HIGD1A	HIG1 hypoxia inducible domain family member 1A [Source:HGNC Syr	2.03E-11
10	CHCHD1	coiled-coil-helix-coiled-coil-helix domain containing 1 [Source:HGNC	4.58E-11
10	CHCHD5	coiled-coil-helix-coiled-coil-helix domain containing 5 [Source:HGNC	7.10E-10
10	TLK1	tousled like kinase 1 [Source:HGNC Symbol;Acc:HGNC:11841]	9.85E-10
10	UBE2V2	ubiquitin conjugating enzyme E2 V2 [Source:HGNC Symbol;Acc:HGN(	7.43E-08
10	TMEM54	transmembrane protein 54 [Source:HGNC Symbol;Acc:HGNC:24143]	7.24E-48
10	RUSC1	RUN and SH3 domain containing 1 [Source:HGNC Symbol;Acc:HGNC:	4.38E-35
10	ACSL3	acyl-CoA synthetase long chain family member 3 [Source:HGNC Sym	8.64E-28
10	PCBD1	pterin-4 alpha-carbinolamine dehydratase 1 [Source:HGNC Symbol;A	9.08E-24
10	GAMT	guanidinoacetate N-methyltransferase [Source:HGNC Symbol;Acc:H(	1.63E-22
10	FABP5	fatty acid binding protein 5 [Source:HGNC Symbol;Acc:HGNC:3560]	2.17E-20
10	JAK1	Janus kinase 1 [Source:HGNC Symbol;Acc:HGNC:6190]	3.93E-17
10	ANXA4	annexin A4 [Source:HGNC Symbol;Acc:HGNC:542]	8.48E-17
10	NDUFV3	NADH:ubiquinone oxidoreductase subunit V3 [Source:HGNC Symbol;	2.01E-15
10	NUDT22	nudix hydrolase 22 [Source:HGNC Symbol;Acc:HGNC:28189]	3.46E-15
10	MRPL12	mitochondrial ribosomal protein L12 [Source:HGNC Symbol;Acc:HGN	2.17E-13
10	MRPS16	mitochondrial ribosomal protein S16 [Source:HGNC Symbol;Acc:HGN	4.78E-11
10	DNPH1	2'-deoxynucleoside 5'-phosphate N-hydrolase 1 [Source:HGNC Symb	5.14E-10
10	MRPS12	mitochondrial ribosomal protein S12 [Source:HGNC Symbol;Acc:HGN	6.17E-10
10	BTF3L4	basic transcription factor 3 like 4 [Source:HGNC Symbol;Acc:HGNC:3(	7.73E-09
10	WASHC3	WASH complex subunit 3 [Source:HGNC Symbol;Acc:HGNC:24256]	1.86E-07
10	HMGCS2	3-hydroxy-3-methylglutaryl-CoA synthase 2 [Source:HGNC Symbol;A(	1.33E-53
10	NUPR2	nuclear protein 2, transcriptional regulator [Source:HGNC Symbol;Ac	9.21E-52
10	ZC3H12A	zinc finger CCCH-type containing 12A [Source:HGNC Symbol;Acc:HGN	2.18E-28
10	TGIF1	TGFB induced factor homeobox 1 [Source:HGNC Symbol;Acc:HGNC:1	4.34E-27
10	NIT2	nitrilase family member 2 [Source:HGNC Symbol;Acc:HGNC:29878]	1.04E-18
10	PDHB	pyruvate dehydrogenase E1 subunit beta [Source:HGNC Symbol;Acc:	4.71E-17
10	ALDH2	aldehyde dehydrogenase 2 family member [Source:HGNC Symbol;Ac	3.89E-15
10	PPA2	inorganic pyrophosphatase 2 [Source:HGNC Symbol;Acc:HGNC:2888	6.52E-13
10	CISD3	CDGSH iron sulfur domain 3 [Source:HGNC Symbol;Acc:HGNC:27578	3.63E-09
10	PRMT2	protein arginine methyltransferase 2 [Source:HGNC Symbol;Acc:HGN	1.35E-08
10	ABHD17A	abhydrolase domain containing 17A, depalmitoylase [Source:HGNC S	7.55E-08
10	BMP5	bone morphogenetic protein 5 [Source:HGNC Symbol;Acc:HGNC:107	1.87E-53
10	ARFIP2	ADP ribosylation factor interacting protein 2 [Source:HGNC Symbol;A	3.61E-40
10	SF3B4	splicing factor 3b subunit 4 [Source:HGNC Symbol;Acc:HGNC:10771]	2.40E-25
10	COMTD1	catechol-O-methyltransferase domain containing 1 [Source:HGNC Sy	7.36E-22
10	FKBP4	FKBP prolyl isomerase 4 [Source:HGNC Symbol;Acc:HGNC:3720]	1.25E-21
10	AP1M2	adaptor related protein complex 1 subunit mu 2 [Source:HGNC Symb	2.53E-21
10	SH3GLB2	SH3 domain containing GRB2 like, endophilin B2 [Source:HGNC Symb	7.59E-19
10	YIF1A	Yip1 interacting factor homolog A, membrane trafficking protein [So	1.50E-18
10	TRAPPC4	trafficking protein particle complex 4 [Source:HGNC Symbol;Acc:HGN	1.72E-13
10	SRA1	steroid receptor RNA activator 1 [Source:HGNC Symbol;Acc:HGNC:1	1.92E-12
10	PSMD14	proteasome 26S subunit, non-ATPase 14 [Source:HGNC Symbol;Acc:	1.41E-11
10	SHARPIN	SHANK associated RH domain interactor [Source:HGNC Symbol;Acc:H	3.32E-11
10	UQCC2	ubiquinol-cytochrome c reductase complex assembly factor 2 [Sourc	1.63E-10
10	CCDC47	coiled-coil domain containing 47 [Source:HGNC Symbol;Acc:HGNC:2	1.48E-09
10	CAPZA2	capping actin protein of muscle Z-line subunit alpha 2 [Source:HGNC	1.67E-08

10	CLPP	caseinolytic mitochondrial matrix peptidase proteolytic subunit [Sou	5.17E-07
10	PNRC1	proline rich nuclear receptor coactivator 1 [Source:HGNC Symbol;Ac	1.28E-58
10	S100A1	S100 calcium binding protein A1 [Source:HGNC Symbol;Acc:HGNC:10	5.54E-49
10	SELENBP1	selenium binding protein 1 [Source:HGNC Symbol;Acc:HGNC:10719]	5.04E-46
10	COL9A2	collagen type IX alpha 2 chain [Source:HGNC Symbol;Acc:HGNC:2218	2.66E-32
10	KDM5B	lysine demethylase 5B [Source:HGNC Symbol;Acc:HGNC:18039]	2.53E-31
10	MZB1	marginal zone B and B1 cell specific protein [Source:HGNC Symbol;A	7.82E-31
10	LGALS3BP	galectin 3 binding protein [Source:HGNC Symbol;Acc:HGNC:6564]	1.81E-28
10	IL18	interleukin 18 [Source:HGNC Symbol;Acc:HGNC:5986]	3.39E-27
10	CCDC85B	coiled-coil domain containing 85B [Source:HGNC Symbol;Acc:HGNC:2	1.90E-13
10	NFIC	nuclear factor I C [Source:HGNC Symbol;Acc:HGNC:7786]	5.36E-13
10	TSPAN13	tetraspanin 13 [Source:HGNC Symbol;Acc:HGNC:21643]	5.97E-12
10	MTCH2	mitochondrial carrier 2 [Source:HGNC Symbol;Acc:HGNC:17587]	6.07E-11
10	UGCG	UDP-glucose ceramide glucosyltransferase [Source:HGNC Symbol;Ac	2.10E-10
10	PSMB5	proteasome 20S subunit beta 5 [Source:HGNC Symbol;Acc:HGNC:954	3.14E-10
10	BOLA3	bolA family member 3 [Source:HGNC Symbol;Acc:HGNC:24415]	3.07E-09
10	EMC6	ER membrane protein complex subunit 6 [Source:HGNC Symbol;Acc:	2.51E-08
10	SDHB	succinate dehydrogenase complex iron sulfur subunit B [Source:HGN	4.31E-08
10	BORCS7	BLOC-1 related complex subunit 7 [Source:HGNC Symbol;Acc:HGNC:	1.10E-07
10	RND3	Rho family GTPase 3 [Source:HGNC Symbol;Acc:HGNC:671]	4.48E-47
10	STEAP1	STEAP family member 1 [Source:HGNC Symbol;Acc:HGNC:11378]	8.67E-43
10	TMEM132A	transmembrane protein 132A [Source:HGNC Symbol;Acc:HGNC:3109	1.09E-38
10	RABEP1	rabaptin, RAB GTPase binding effector protein 1 [Source:HGNC Symb	1.89E-28
10	GALNT6	polypeptide N-acetylgalactosaminyltransferase 6 [Source:HGNC Sym	1.68E-25
10	GLRX2	glutaredoxin 2 [Source:HGNC Symbol;Acc:HGNC:16065]	1.96E-24
10	EBAG9	estrogen receptor binding site associated antigen 9 [Source:HGNC Sy	1.60E-22
10	RSRP1	arginine and serine rich protein 1 [Source:HGNC Symbol;Acc:HGNC:2	1.48E-15
10	EEF1E1	eukaryotic translation elongation factor 1 epsilon 1 [Source:HGNC Sy	8.34E-15
10	OXLD1	oxidoreductase like domain containing 1 [Source:HGNC Symbol;Acc:	1.72E-12
10	HADHB	hydroxyacyl-CoA dehydrogenase trifunctional multienzyme complex	9.05E-11
10	NDUFAF2	NADH:ubiquinone oxidoreductase complex assembly factor 2 [Sourc	1.88E-10
10	CEBPD	CCAAT enhancer binding protein delta [Source:HGNC Symbol;Acc:HG	5.12E-08
10	PPIH	peptidylprolyl isomerase H [Source:HGNC Symbol;Acc:HGNC:14651]	1.90E-07
10	AC008771.1	novel transcript	1.82E-40
10	SDC1	syndecan 1 [Source:HGNC Symbol;Acc:HGNC:10658]	1.67E-39
10	LIMA1	LIM domain and actin binding 1 [Source:HGNC Symbol;Acc:HGNC:24	1.74E-27
10	MAP3K13	mitogen-activated protein kinase kinase kinase 13 [Source:HGNC Syr	5.26E-21
10	PTOV1	PTOV1 extended AT-hook containing adaptor protein [Source:HGNC	7.07E-21
10	DALRD3	DALR anticodon binding domain containing 3 [Source:HGNC Symbol;	1.74E-20
10	GTPBP6	GTP binding protein 6 (putative) [Source:HGNC Symbol;Acc:HGNC:30	4.43E-15
10	COMMD1	copper metabolism domain containing 1 [Source:HGNC Symbol;Acc:	2.34E-09
10	CCDC57	coiled-coil domain containing 57 [Source:HGNC Symbol;Acc:HGNC:27	3.19E-09
10	DNAJC2	DnaJ heat shock protein family (Hsp40) member C2 [Source:HGNC Sy	2.55E-08
10	SMIM19	small integral membrane protein 19 [Source:HGNC Symbol;Acc:HGN	4.62E-08
10	SLC44A4	solute carrier family 44 member 4 [Source:HGNC Symbol;Acc:HGNC:	1.64E-45
10	GPR160	G protein-coupled receptor 160 [Source:HGNC Symbol;Acc:HGNC:23	1.77E-30
10	LIMS1	LIM zinc finger domain containing 1 [Source:HGNC Symbol;Acc:HGN	1.04E-26

10	TMEM9	transmembrane protein 9 [Source:HGNC Symbol;Acc:HGNC:18823]	1.66E-25
10	MRPL32	mitochondrial ribosomal protein L32 [Source:HGNC Symbol;Acc:HGN	1.94E-24
10	ARL1	ADP ribosylation factor like GTPase 1 [Source:HGNC Symbol;Acc:HGN	1.54E-21
10	ANXA3	annexin A3 [Source:HGNC Symbol;Acc:HGNC:541]	3.24E-20
10	POLR2H	RNA polymerase II, I and III subunit H [Source:HGNC Symbol;Acc:HGN	4.13E-18
10	MCRIP2	MAPK regulated corepressor interacting protein 2 [Source:HGNC Syn	4.89E-18
10	UQCC3	ubiquinol-cytochrome c reductase complex assembly factor 3 [Sourc	4.12E-17
10	GPAA1	glycosylphosphatidylinositol anchor attachment 1 [Source:HGNC Syn	2.42E-16
10	NTPCR	nucleoside-triphosphatase, cancer-related [Source:HGNC Symbol;Ac	6.24E-16
10	SNRNP25	small nuclear ribonucleoprotein U11/U12 subunit 25 [Source:HGNC S	1.32E-15
10	CD59	CD59 molecule (CD59 blood group) [Source:HGNC Symbol;Acc:HGNC	2.62E-15
10	TPD52	tumor protein D52 [Source:HGNC Symbol;Acc:HGNC:12005]	8.89E-13
10	GPATCH4	G-patch domain containing 4 [Source:HGNC Symbol;Acc:HGNC:2598	4.26E-12
10	HSBP1L1	heat shock factor binding protein 1 like 1 [Source:HGNC Symbol;Acc:	3.35E-10
10	RPL26L1	ribosomal protein L26 like 1 [Source:HGNC Symbol;Acc:HGNC:17050	3.43E-09
10	IARS2	isoleucyl-tRNA synthetase 2, mitochondrial [Source:HGNC Symbol;Ac	1.51E-08
10	COMMD2	COMM domain containing 2 [Source:HGNC Symbol;Acc:HGNC:24993	3.29E-07
10	PPIC	peptidylprolyl isomerase C [Source:HGNC Symbol;Acc:HGNC:9256]	1.27E-37
10	PON2	paraoxonase 2 [Source:HGNC Symbol;Acc:HGNC:9205]	6.06E-32
10	CYP27A1	cytochrome P450 family 27 subfamily A member 1 [Source:HGNC Sy	3.67E-25
10	FH	fumarate hydratase [Source:HGNC Symbol;Acc:HGNC:3700]	7.28E-23
10	ARL16	ADP ribosylation factor like GTPase 16 [Source:HGNC Symbol;Acc:HG	5.36E-19
10	AP1S1	adaptor related protein complex 1 subunit sigma 1 [Source:HGNC Sy	8.16E-16
10	FLYWCH2	FLYWCH family member 2 [Source:HGNC Symbol;Acc:HGNC:25178]	1.76E-15
10	LEO1	LEO1 homolog, Paf1/RNA polymerase II complex component [Source	6.87E-15
10	POLR3K	RNA polymerase III subunit K [Source:HGNC Symbol;Acc:HGNC:1412	1.01E-14
10	POLD2	DNA polymerase delta 2, accessory subunit [Source:HGNC Symbol;Ac	1.56E-14
10	MRPL27	mitochondrial ribosomal protein L27 [Source:HGNC Symbol;Acc:HGN	2.48E-14
10	MTX1	metaxin 1 [Source:HGNC Symbol;Acc:HGNC:7504]	4.60E-13
10	ATP1A1	ATPase Na+/K+ transporting subunit alpha 1 [Source:HGNC Symbol;A	7.21E-12
10	ACOT13	acyl-CoA thioesterase 13 [Source:HGNC Symbol;Acc:HGNC:20999]	4.09E-11
10	GPI	glucose-6-phosphate isomerase [Source:HGNC Symbol;Acc:HGNC:44	6.65E-10
10	KDELR2	KDEL endoplasmic reticulum protein retention receptor 2 [Source:HG	9.39E-09
10	MMADHC	metabolism of cobalamin associated D [Source:HGNC Symbol;Acc:HG	2.35E-08
10	TMEM208	transmembrane protein 208 [Source:HGNC Symbol;Acc:HGNC:25015	1.20E-07
10	ILK	integrin linked kinase [Source:HGNC Symbol;Acc:HGNC:6040]	5.24E-07
10	DEFB1	defensin beta 1 [Source:HGNC Symbol;Acc:HGNC:2766]	1.05E-43
10	NUDT8	nudix hydrolase 8 [Source:HGNC Symbol;Acc:HGNC:8055]	4.08E-30
10	PHGDH	phosphoglycerate dehydrogenase [Source:HGNC Symbol;Acc:HGNC:8	5.63E-26
10	TSPAN15	tetraspanin 15 [Source:HGNC Symbol;Acc:HGNC:23298]	1.89E-25
10	ERI3	ERI1 exoribonuclease family member 3 [Source:HGNC Symbol;Acc:HG	1.44E-21
10	TUSC2	tumor suppressor 2, mitochondrial calcium regulator [Source:HGNC S	5.75E-20
10	MRPS14	mitochondrial ribosomal protein S14 [Source:HGNC Symbol;Acc:HGN	8.00E-16
10	PDZD11	PDZ domain containing 11 [Source:HGNC Symbol;Acc:HGNC:28034]	8.56E-14
10	AMD1	adenosylmethionine decarboxylase 1 [Source:HGNC Symbol;Acc:HGN	5.69E-13
10	MRPL37	mitochondrial ribosomal protein L37 [Source:HGNC Symbol;Acc:HGN	6.68E-12
10	TIMMDC1	translocase of inner mitochondrial membrane domain containing 1 [	5.23E-10

10	KNOP1	lysine rich nucleolar protein 1 [Source:HGNC Symbol;Acc:HGNC:3440	2.41E-09
10	ELOVL1	ELOVL fatty acid elongase 1 [Source:HGNC Symbol;Acc:HGNC:14418]	3.63E-09
10	PMF1	polyamine modulated factor 1 [Source:HGNC Symbol;Acc:HGNC:911	1.22E-08
10	GABARAP	GABA type A receptor-associated protein [Source:HGNC Symbol;Acc:	1.88E-08
10	RBBP6	RB binding protein 6, ubiquitin ligase [Source:HGNC Symbol;Acc:HGN	2.00E-08
10	CYP4Z1	cytochrome P450 family 4 subfamily Z member 1 [Source:HGNC Sym	2.65E-48
10	DEGS2	delta 4-desaturase, sphingolipid 2 [Source:HGNC Symbol;Acc:HGNC:2	1.51E-40
10	CA12	carbonic anhydrase 12 [Source:HGNC Symbol;Acc:HGNC:1371]	5.80E-38
10	TNFRSF12A	TNF receptor superfamily member 12A [Source:HGNC Symbol;Acc:H(	1.45E-35
10	PTGR1	prostaglandin reductase 1 [Source:HGNC Symbol;Acc:HGNC:18429]	1.28E-34
10	CNN3	calponin 3 [Source:HGNC Symbol;Acc:HGNC:2157]	6.16E-32
10	WEE1	WEE1 G2 checkpoint kinase [Source:HGNC Symbol;Acc:HGNC:12761]	7.47E-28
10	BCAS4	breast carcinoma amplified sequence 4 [Source:HGNC Symbol;Acc:H	5.41E-20
10	B3GAT3	beta-1,3-glucuronyltransferase 3 [Source:HGNC Symbol;Acc:HGNC:9	1.61E-19
10	MTX2	metaxin 2 [Source:HGNC Symbol;Acc:HGNC:7506]	2.86E-18
10	SMIM4	small integral membrane protein 4 [Source:HGNC Symbol;Acc:HGNC	3.97E-16
10	LSR	lipolysis stimulated lipoprotein receptor [Source:HGNC Symbol;Acc:H	7.34E-16
10	LINC01184	long intergenic non-protein coding RNA 1184 [Source:HGNC Symbol;	2.56E-14
10	MVB12A	multivesicular body subunit 12A [Source:HGNC Symbol;Acc:HGNC:25	1.13E-12
10	PSMG1	proteasome assembly chaperone 1 [Source:HGNC Symbol;Acc:HGNC	3.69E-12
10	MRPL19	mitochondrial ribosomal protein L19 [Source:HGNC Symbol;Acc:HGN	1.10E-11
10	NR4A1	nuclear receptor subfamily 4 group A member 1 [Source:HGNC Symb	1.22E-11
10	C15orf61	chromosome 15 open reading frame 61 [Source:HGNC Symbol;Acc:H	1.00E-08
10	STOML2	stomatin like 2 [Source:HGNC Symbol;Acc:HGNC:14559]	4.19E-08
10	PSIP1	PC4 and SFRS1 interacting protein 1 [Source:HGNC Symbol;Acc:HGN(	1.31E-07
10	PEX16	peroxisomal biogenesis factor 16 [Source:HGNC Symbol;Acc:HGNC:8	2.08E-07
10	CARD16	caspase recruitment domain family member 16 [Source:HGNC Symb	4.46E-07
10	CITED1	Cbp/p300 interacting transactivator with Glu/Asp rich carboxy-termi	3.60E-45
10	FOXA1	forkhead box A1 [Source:HGNC Symbol;Acc:HGNC:5021]	1.24E-39
10	SULT2B1	sulfotransferase family 2B member 1 [Source:HGNC Symbol;Acc:HGN	6.27E-37
10	IFITM2	interferon induced transmembrane protein 2 [Source:HGNC Symbol;	3.12E-33
10	NR2F6	nuclear receptor subfamily 2 group F member 6 [Source:HGNC Symb	1.92E-31
10	AAMDC	adipogenesis associated Mth938 domain containing [Source:HGNC S	3.02E-28
10	MAPK13	mitogen-activated protein kinase 13 [Source:HGNC Symbol;Acc:HGN	7.81E-23
10	DST	dystonin [Source:HGNC Symbol;Acc:HGNC:1090]	1.34E-21
10	NECAB3	N-terminal EF-hand calcium binding protein 3 [Source:HGNC Symbol;	1.86E-21
10	CHID1	chitinase domain containing 1 [Source:HGNC Symbol;Acc:HGNC:2847	8.51E-21
10	ZNF593	zinc finger protein 593 [Source:HGNC Symbol;Acc:HGNC:30943]	7.77E-18
10	EMC2	ER membrane protein complex subunit 2 [Source:HGNC Symbol;Acc:	1.30E-16
10	TSEN34	tRNA splicing endonuclease subunit 34 [Source:HGNC Symbol;Acc:HG	5.70E-16
10	SMIM1	small integral membrane protein 1 (Vel blood group) [Source:HGNC S	8.86E-15
10	ISOC2	isochorismatase domain containing 2 [Source:HGNC Symbol;Acc:HGI	7.20E-14
10	SDC4	syndecan 4 [Source:HGNC Symbol;Acc:HGNC:10661]	4.27E-13
10	FAM210B	family with sequence similarity 210 member B [Source:HGNC Symbo	1.27E-12
10	CXXC5	CXXC finger protein 5 [Source:HGNC Symbol;Acc:HGNC:26943]	3.27E-12
10	TWF1	twinfilin actin binding protein 1 [Source:HGNC Symbol;Acc:HGNC:962	3.46E-12
10	ZNF91	zinc finger protein 91 [Source:HGNC Symbol;Acc:HGNC:13166]	2.96E-11

10	AHCY	adenosylhomocysteinase [Source:HGNC Symbol;Acc:HGNC:343]	7.73E-11
10	ATIC	5-aminoimidazole-4-carboxamide ribonucleotide formyltransferase/	4.97E-08
10	CDV3	CDV3 homolog [Source:HGNC Symbol;Acc:HGNC:26928]	5.03E-08
10	FMC1	formation of mitochondrial complex V assembly factor 1 homolog [S	1.34E-07
10	PYDC1	pyrin domain containing 1 [Source:HGNC Symbol;Acc:HGNC:30261]	2.71E-43
10	HOOK2	hook microtubule tethering protein 2 [Source:HGNC Symbol;Acc:HGI	6.62E-30
10	CTSF	cathepsin F [Source:HGNC Symbol;Acc:HGNC:2531]	7.37E-27
10	IGFBP2	insulin like growth factor binding protein 2 [Source:HGNC Symbol;Ac	6.39E-24
10	GMNN	geminin DNA replication inhibitor [Source:HGNC Symbol;Acc:HGNC:1	2.48E-23
10	RCN1	reticulocalbin 1 [Source:HGNC Symbol;Acc:HGNC:9934]	5.68E-22
10	TMEM159	transmembrane protein 159 [Source:HGNC Symbol;Acc:HGNC:3013	3.49E-17
10	TUBB2A	tubulin beta 2A class IIa [Source:HGNC Symbol;Acc:HGNC:12412]	6.33E-17
10	PHAX	phosphorylated adaptor for RNA export [Source:HGNC Symbol;Acc:H	2.66E-11
10	ROGDI	rogdi atypical leucine zipper [Source:HGNC Symbol;Acc:HGNC:29478	8.77E-11
10	DNAJB11	DnaJ heat shock protein family (Hsp40) member B11 [Source:HGNC S	1.38E-10
10	RNF213	ring finger protein 213 [Source:HGNC Symbol;Acc:HGNC:14539]	5.11E-10
10	TIMM17A	translocase of inner mitochondrial membrane 17A [Source:HGNC Sy	1.34E-08
10	LZIC	leucine zipper and CTNNBIP1 domain containing [Source:HGNC Symb	1.80E-08
10	CCDC6	coiled-coil domain containing 6 [Source:HGNC Symbol;Acc:HGNC:18	2.30E-08
10	COMMD4	COMM domain containing 4 [Source:HGNC Symbol;Acc:HGNC:26027	3.31E-07
10	DNASE2	deoxyribonuclease 2, lysosomal [Source:HGNC Symbol;Acc:HGNC:29	6.25E-07
10	RERG	RAS like estrogen regulated growth inhibitor [Source:HGNC Symbol;A	1.83E-38
10	PDLIM4	PDZ and LIM domain 4 [Source:HGNC Symbol;Acc:HGNC:16501]	1.32E-37
10	STMND1	stathmin domain containing 1 [Source:HGNC Symbol;Acc:HGNC:446	4.44E-37
10	SRD5A3	steroid 5 alpha-reductase 3 [Source:HGNC Symbol;Acc:HGNC:25812]	8.71E-36
10	SELENOP	selenoprotein P [Source:HGNC Symbol;Acc:HGNC:10751]	1.14E-32
10	LYPD6B	LY6/PLAUR domain containing 6B [Source:HGNC Symbol;Acc:HGNC:2	1.88E-29
10	P4HTM	prolyl 4-hydroxylase, transmembrane [Source:HGNC Symbol;Acc:HG	4.53E-26
10	TRPT1	tRNA phosphotransferase 1 [Source:HGNC Symbol;Acc:HGNC:20316]	9.38E-26
10	C17orf58	chromosome 17 open reading frame 58 [Source:HGNC Symbol;Acc:H	5.38E-25
10	PXMP4	peroxisomal membrane protein 4 [Source:HGNC Symbol;Acc:HGNC:1	9.07E-20
10	ABHD11	abhydrolase domain containing 11 [Source:HGNC Symbol;Acc:HGNC:	3.86E-17
10	FAM241A	family with sequence similarity 241 member A [Source:HGNC Symbo	2.33E-16
10	RRAGA	Ras related GTP binding A [Source:HGNC Symbol;Acc:HGNC:16963]	2.77E-16
10	HOXB2	homeobox B2 [Source:HGNC Symbol;Acc:HGNC:5113]	3.00E-16
10	IER5L	immediate early response 5 like [Source:HGNC Symbol;Acc:HGNC:23	1.93E-15
10	ENDOG	endonuclease G [Source:HGNC Symbol;Acc:HGNC:3346]	6.27E-15
10	FAM120AOS	family with sequence similarity 120A opposite strand [Source:HGNC	1.16E-11
10	TERF2IP	TERF2 interacting protein [Source:HGNC Symbol;Acc:HGNC:19246]	6.83E-11
10	CALU	calumenin [Source:HGNC Symbol;Acc:HGNC:1458]	8.80E-11
10	SMIM30	small integral membrane protein 30 [Source:HGNC Symbol;Acc:HGN	1.18E-10
10	SMAP2	small ArfGAP2 [Source:HGNC Symbol;Acc:HGNC:25082]	3.85E-10
10	HRAS	HRas proto-oncogene, GTPase [Source:HGNC Symbol;Acc:HGNC:517	6.23E-10
10	RASSF7	Ras association domain family member 7 [Source:HGNC Symbol;Acc:	1.25E-09
10	NUDT2	nudix hydrolase 2 [Source:HGNC Symbol;Acc:HGNC:8049]	2.59E-09
10	MRPL36	mitochondrial ribosomal protein L36 [Source:HGNC Symbol;Acc:HGN	1.17E-08
10	CETN2	centrin 2 [Source:HGNC Symbol;Acc:HGNC:1867]	6.35E-08

10	TFAP2A	transcription factor AP-2 alpha [Source:HGNC Symbol;Acc:HGNC:117	1.16E-34
10	EFNA1	ephrin A1 [Source:HGNC Symbol;Acc:HGNC:3221]	3.87E-34
10	MORN2	MORN repeat containing 2 [Source:HGNC Symbol;Acc:HGNC:30166]	1.74E-28
10	CCL5	C-C motif chemokine ligand 5 [Source:HGNC Symbol;Acc:HGNC:1063	3.77E-26
10	NFIA	nuclear factor I A [Source:HGNC Symbol;Acc:HGNC:7784]	8.15E-22
10	TFPT	TCF3 fusion partner [Source:HGNC Symbol;Acc:HGNC:13630]	5.74E-17
10	ECHDC2	enoyl-CoA hydratase domain containing 2 [Source:HGNC Symbol;Acc	3.68E-16
10	HSD17B4	hydroxysteroid 17-beta dehydrogenase 4 [Source:HGNC Symbol;Acc:	1.29E-13
10	CBWD1	COBW domain containing 1 [Source:HGNC Symbol;Acc:HGNC:17134]	4.19E-08
10	WDR61	WD repeat domain 61 [Source:HGNC Symbol;Acc:HGNC:30300]	1.06E-07
10	ZNF326	zinc finger protein 326 [Source:HGNC Symbol;Acc:HGNC:14104]	5.22E-07
10	PACSIN3	protein kinase C and casein kinase substrate in neurons 3 [Source:HG	1.84E-35
10	SCUBE2	signal peptide, CUB domain and EGF like domain containing 2 [Source	4.34E-34
10	ARHGAP29	Rho GTPase activating protein 29 [Source:HGNC Symbol;Acc:HGNC:3	2.24E-28
10	ERBB2	erb-b2 receptor tyrosine kinase 2 [Source:HGNC Symbol;Acc:HGNC:3	9.54E-28
10	ANXA9	annexin A9 [Source:HGNC Symbol;Acc:HGNC:547]	2.39E-26
10	STK4	serine/threonine kinase 4 [Source:HGNC Symbol;Acc:HGNC:11408]	1.01E-23
10	DSC2	desmocollin 2 [Source:HGNC Symbol;Acc:HGNC:3036]	2.74E-22
10	NTHL1	nth like DNA glycosylase 1 [Source:HGNC Symbol;Acc:HGNC:8028]	4.72E-15
10	MRPL15	mitochondrial ribosomal protein L15 [Source:HGNC Symbol;Acc:HGN	3.15E-13
10	TSFM	Ts translation elongation factor, mitochondrial [Source:HGNC Symbo	9.16E-13
10	MYO1C	myosin IC [Source:HGNC Symbol;Acc:HGNC:7597]	1.27E-12
10	PAICS	phosphoribosylaminoimidazole carboxylase and phosphoribosylamin	7.94E-11
10	MRPL58	mitochondrial ribosomal protein L58 [Source:HGNC Symbol;Acc:HGN	2.34E-10
10	AK6	adenylate kinase 6 [Source:HGNC Symbol;Acc:HGNC:49151]	2.10E-09
10	SURF2	surfeit 2 [Source:HGNC Symbol;Acc:HGNC:11475]	1.92E-08
10	CHRAC1	chromatin accessibility complex subunit 1 [Source:HGNC Symbol;Acc	6.19E-08
10	ARRDC1	arrestin domain containing 1 [Source:HGNC Symbol;Acc:HGNC:2863	1.41E-07
10	TNRC6B	trinucleotide repeat containing adaptor 6B [Source:HGNC Symbol;Ac	5.30E-07
10	DIXDC1	DIX domain containing 1 [Source:HGNC Symbol;Acc:HGNC:23695]	1.16E-30
10	LIMCH1	LIM and calponin homology domains 1 [Source:HGNC Symbol;Acc:HG	5.92E-27
10	HILPDA	hypoxia inducible lipid droplet associated [Source:HGNC Symbol;Acc	2.43E-26
10	SMYD2	SET and MYND domain containing 2 [Source:HGNC Symbol;Acc:HGN	2.18E-18
10	PRSS23	serine protease 23 [Source:HGNC Symbol;Acc:HGNC:14370]	4.19E-18
10	OSBPL1A	oxysterol binding protein like 1A [Source:HGNC Symbol;Acc:HGNC:1	9.97E-17
10	FUNDC1	FUN14 domain containing 1 [Source:HGNC Symbol;Acc:HGNC:28746	8.22E-12
10	TRAF5	TNF receptor associated factor 5 [Source:HGNC Symbol;Acc:HGNC:1	1.12E-11
10	PDHA1	pyruvate dehydrogenase E1 subunit alpha 1 [Source:HGNC Symbol;A	2.42E-11
10	RSU1	Ras suppressor protein 1 [Source:HGNC Symbol;Acc:HGNC:10464]	1.98E-10
10	GOSR1	golgi SNAP receptor complex member 1 [Source:HGNC Symbol;Acc:H	5.62E-10
10	CISD1	CDGSH iron sulfur domain 1 [Source:HGNC Symbol;Acc:HGNC:30880	6.75E-10
10	RETREG1	reticulophagy regulator 1 [Source:HGNC Symbol;Acc:HGNC:25964]	1.80E-08
10	GGCT	gamma-glutamylcyclotransferase [Source:HGNC Symbol;Acc:HGNC:2	4.61E-07
10	SRGN	serglycin [Source:HGNC Symbol;Acc:HGNC:9361]	7.57E-64
10	AQP5	aquaporin 5 [Source:HGNC Symbol;Acc:HGNC:638]	1.50E-36
10	PTGIS	prostaglandin I2 synthase [Source:HGNC Symbol;Acc:HGNC:9603]	8.38E-34
10	DCDC2	doublecortin domain containing 2 [Source:HGNC Symbol;Acc:HGNC:1	1.71E-33

10	APOD	apolipoprotein D [Source:HGNC Symbol;Acc:HGNC:612]	2.09E-31
10	CRNDE	colorectal neoplasia differentially expressed [Source:HGNC Symbol;A	2.81E-25
10	ACADSB	acyl-CoA dehydrogenase short/branched chain [Source:HGNC Symbo	1.23E-24
10	KIF9	kinesin family member 9 [Source:HGNC Symbol;Acc:HGNC:16666]	2.05E-24
10	DUSP4	dual specificity phosphatase 4 [Source:HGNC Symbol;Acc:HGNC:307	1.23E-20
10	TRAF4	TNF receptor associated factor 4 [Source:HGNC Symbol;Acc:HGNC:1	1.24E-20
10	ADAM15	ADAM metallopeptidase domain 15 [Source:HGNC Symbol;Acc:HGN	7.65E-20
10	CDC42SE2	CDC42 small effector 2 [Source:HGNC Symbol;Acc:HGNC:18547]	2.11E-19
10	POMGNT1	protein O-linked mannose N-acetylglucosaminyltransferase 1 (beta 1	2.58E-18
10	JUP	junction plakoglobin [Source:HGNC Symbol;Acc:HGNC:6207]	3.59E-18
10	IP6K2	inositol hexakisphosphate kinase 2 [Source:HGNC Symbol;Acc:HGNC	1.69E-13
10	STXBP2	syntaxin binding protein 2 [Source:HGNC Symbol;Acc:HGNC:11445]	3.86E-13
10	COPRS	coordinator of PRMT5 and differentiation stimulator [Source:HGNC S	9.32E-13
10	OSTF1	osteoclast stimulating factor 1 [Source:HGNC Symbol;Acc:HGNC:851	1.12E-11
10	CLPTM1	CLPTM1 regulator of GABA type A receptor forward trafficking [Sour	1.34E-10
10	SAR1B	secretion associated Ras related GTPase 1B [Source:HGNC Symbol;A	4.73E-10
10	PSMG4	proteasome assembly chaperone 4 [Source:HGNC Symbol;Acc:HGNC	1.83E-09
10	LRRK2	leucine rich repeat kinase 2 [Source:HGNC Symbol;Acc:HGNC:18618]	3.34E-09
10	TMEM106C	transmembrane protein 106C [Source:HGNC Symbol;Acc:HGNC:287	7.74E-08
10	ZDHHC24	zinc finger DHHC-type containing 24 [Source:HGNC Symbol;Acc:HGN	1.54E-07
10	KRTCAP3	keratinocyte associated protein 3 [Source:HGNC Symbol;Acc:HGNC:2	1.94E-26
10	MUC1	mucin 1, cell surface associated [Source:HGNC Symbol;Acc:HGNC:75	7.86E-26
10	ERBB3	erb-b2 receptor tyrosine kinase 3 [Source:HGNC Symbol;Acc:HGNC:3	5.52E-24
10	WWTR1	WW domain containing transcription regulator 1 [Source:HGNC Sym	5.91E-24
10	MACROD1	mono-ADP ribosylhydrolase 1 [Source:HGNC Symbol;Acc:HGNC:295	8.23E-24
10	TRIM47	tripartite motif containing 47 [Source:HGNC Symbol;Acc:HGNC:1902	5.85E-22
10	SLC39A6	solute carrier family 39 member 6 [Source:HGNC Symbol;Acc:HGNC:	3.10E-21
10	CTNND1	catenin delta 1 [Source:HGNC Symbol;Acc:HGNC:2515]	2.24E-19
10	ICA1	islet cell autoantigen 1 [Source:HGNC Symbol;Acc:HGNC:5343]	1.97E-18
10	TCEAL1	transcription elongation factor A like 1 [Source:HGNC Symbol;Acc:H	2.15E-18
10	TMEM99	transmembrane protein 99 (putative) [Source:HGNC Symbol;Acc:HG	2.26E-18
10	SLC25A4	solute carrier family 25 member 4 [Source:HGNC Symbol;Acc:HGNC:	1.63E-17
10	ARV1	ARV1 homolog, fatty acid homeostasis modulator [Source:HGNC Sym	6.87E-14
10	LTBP3	latent transforming growth factor beta binding protein 3 [Source:HG	9.12E-14
10	CBX4	chromobox 4 [Source:HGNC Symbol;Acc:HGNC:1554]	1.44E-11
10	CSAD	cysteine sulfinic acid decarboxylase [Source:HGNC Symbol;Acc:HGN	3.48E-11
10	PIGX	phosphatidylinositol glycan anchor biosynthesis class X [Source:HGN	6.23E-11
10	B4GALT3	beta-1,4-galactosyltransferase 3 [Source:HGNC Symbol;Acc:HGNC:92	2.83E-10
10	POP7	POP7 homolog, ribonuclease P/MRP subunit [Source:HGNC Symbol;	3.37E-10
10	TAOK3	TAO kinase 3 [Source:HGNC Symbol;Acc:HGNC:18133]	8.69E-09
10	ALDH9A1	aldehyde dehydrogenase 9 family member A1 [Source:HGNC Symbo	1.18E-08
10	MPV17	mitochondrial inner membrane protein MPV17 [Source:HGNC Symb	6.00E-08
10	CCAR1	cell division cycle and apoptosis regulator 1 [Source:HGNC Symbol;A	9.54E-08
10	UNC50	unc-50 inner nuclear membrane RNA binding protein [Source:HGNC	5.29E-07
10	MAL2	mal, T cell differentiation protein 2 [Source:HGNC Symbol;Acc:HGNC	1.10E-35
10	SOX2	SRY-box transcription factor 2 [Source:HGNC Symbol;Acc:HGNC:111	1.95E-34
10	NRGN	neurogranin [Source:HGNC Symbol;Acc:HGNC:8000]	8.85E-30

10	SYDE2	synapse defective Rho GTPase homolog 2 [Source:HGNC Symbol;Acc	5.44E-26
10	RHOD	ras homolog family member D [Source:HGNC Symbol;Acc:HGNC:670	2.43E-22
10	FAM114A1	family with sequence similarity 114 member A1 [Source:HGNC Symb	1.18E-20
10	AUTS2	activator of transcription and developmental regulator AUTS2 [Sourc	2.46E-19
10	PTS	6-pyruvoyltetrahydropterin synthase [Source:HGNC Symbol;Acc:HGN	7.00E-17
10	ASL	argininosuccinate lyase [Source:HGNC Symbol;Acc:HGNC:746]	9.58E-16
10	HDHD3	haloacid dehalogenase like hydrolase domain containing 3 [Source:H	8.71E-15
10	GCSH	glycine cleavage system protein H [Source:HGNC Symbol;Acc:HGNC:4	7.27E-12
10	PLPP5	phospholipid phosphatase 5 [Source:HGNC Symbol;Acc:HGNC:25026	1.33E-11
10	MRPS18A	mitochondrial ribosomal protein S18A [Source:HGNC Symbol;Acc:HG	1.88E-11
10	DNPEP	aspartyl aminopeptidase [Source:HGNC Symbol;Acc:HGNC:2981]	2.06E-11
10	GSTM3	glutathione S-transferase mu 3 [Source:HGNC Symbol;Acc:HGNC:463	2.46E-11
10	MED19	mediator complex subunit 19 [Source:HGNC Symbol;Acc:HGNC:2960	1.10E-10
10	QTRT1	queuine tRNA-ribosyltransferase catalytic subunit 1 [Source:HGNC Sy	2.71E-09
10	TRAPPC12	trafficking protein particle complex 12 [Source:HGNC Symbol;Acc:HG	8.03E-09
10	RHOG	ras homolog family member G [Source:HGNC Symbol;Acc:HGNC:672	9.02E-08
10	ATG101	autophagy related 101 [Source:HGNC Symbol;Acc:HGNC:25679]	2.04E-07
10	G3BP2	G3BP stress granule assembly factor 2 [Source:HGNC Symbol;Acc:HG	2.19E-07
10	IFRD2	interferon related developmental regulator 2 [Source:HGNC Symbol;	4.54E-07
10	EMP3	epithelial membrane protein 3 [Source:HGNC Symbol;Acc:HGNC:333	2.28E-69
10	EHF	ETS homologous factor [Source:HGNC Symbol;Acc:HGNC:3246]	1.10E-28
10	GPRC5C	G protein-coupled receptor class C group 5 member C [Source:HGNC	2.09E-23
10	NCOA4	nuclear receptor coactivator 4 [Source:HGNC Symbol;Acc:HGNC:767	1.68E-21
10	MTA3	metastasis associated 1 family member 3 [Source:HGNC Symbol;Acc:	6.01E-20
10	MRPL17	mitochondrial ribosomal protein L17 [Source:HGNC Symbol;Acc:HGN	6.15E-19
10	RGL2	ral guanine nucleotide dissociation stimulator like 2 [Source:HGNC Sy	1.23E-17
10	IFT22	intraflagellar transport 22 [Source:HGNC Symbol;Acc:HGNC:21895]	1.68E-16
10	USP15	ubiquitin specific peptidase 15 [Source:HGNC Symbol;Acc:HGNC:126	1.95E-14
10	NDUFAF6	NADH:ubiquinone oxidoreductase complex assembly factor 6 [Sourc	1.15E-13
10	NEURL3	neuralized E3 ubiquitin protein ligase 3 [Source:HGNC Symbol;Acc:H	4.41E-13
10	LHFPL2	LHFPL tetraspan subfamily member 2 [Source:HGNC Symbol;Acc:HGI	7.36E-13
10	CASP4	caspase 4 [Source:HGNC Symbol;Acc:HGNC:1505]	1.57E-12
10	PIGT	phosphatidylinositol glycan anchor biosynthesis class T [Source:HGN	7.96E-12
10	WBP2	WW domain binding protein 2 [Source:HGNC Symbol;Acc:HGNC:127	1.12E-10
10	AAMP	angio associated migratory cell protein [Source:HGNC Symbol;Acc:H	1.12E-10
10	ATF7	activating transcription factor 7 [Source:HGNC Symbol;Acc:HGNC:79	1.27E-10
10	NECTIN2	nectin cell adhesion molecule 2 [Source:HGNC Symbol;Acc:HGNC:97	4.78E-10
10	JMJD8	jumonji domain containing 8 [Source:HGNC Symbol;Acc:HGNC:1414	6.38E-09
10	ACTN1	actinin alpha 1 [Source:HGNC Symbol;Acc:HGNC:163]	1.30E-07
10	SLC1A5	solute carrier family 1 member 5 [Source:HGNC Symbol;Acc:HGNC:1	4.95E-07
10	RIPOR2	RHO family interacting cell polarization regulator 2 [Source:HGNC Sy	5.09E-52
10	NOSTRIN	nitric oxide synthase trafficking [Source:HGNC Symbol;Acc:HGNC:202	2.81E-32
10	DNALI1	dynein axonemal light intermediate chain 1 [Source:HGNC Symbol;A	1.81E-31
10	RAMP2	receptor activity modifying protein 2 [Source:HGNC Symbol;Acc:HGN	1.38E-27
10	DDR1	discoidin domain receptor tyrosine kinase 1 [Source:HGNC Symbol;A	7.03E-24
10	TPRN	taperin [Source:HGNC Symbol;Acc:HGNC:26894]	6.39E-23
10	NEDD4L	NEDD4 like E3 ubiquitin protein ligase [Source:HGNC Symbol;Acc:HG	2.37E-21

10	TMEM97	transmembrane protein 97 [Source:HGNC Symbol;Acc:HGNC:28106]	6.17E-21
10	LAMB2	laminin subunit beta 2 [Source:HGNC Symbol;Acc:HGNC:6487]	1.32E-20
10	EPS8L1	EPS8 like 1 [Source:HGNC Symbol;Acc:HGNC:21295]	1.89E-20
10	FAM171B	family with sequence similarity 171 member B [Source:HGNC Symbo	5.21E-19
10	PHLDA3	pleckstrin homology like domain family A member 3 [Source:HGNC S	1.18E-16
10	SUCO	SUN domain containing ossification factor [Source:HGNC Symbol;Acc	8.34E-16
10	SNAP23	synaptosome associated protein 23 [Source:HGNC Symbol;Acc:HGNC	2.92E-12
10	PIGP	phosphatidylinositol glycan anchor biosynthesis class P [Source:HGN	3.34E-10
10	AREG	amphiregulin [Source:HGNC Symbol;Acc:HGNC:651]	3.80E-10
10	SERTAD3	SERTA domain containing 3 [Source:HGNC Symbol;Acc:HGNC:17931]	6.22E-10
10	IMPACT	impact RWD domain protein [Source:HGNC Symbol;Acc:HGNC:20387	7.17E-09
10	GGPS1	geranylgeranyl diphosphate synthase 1 [Source:HGNC Symbol;Acc:H	1.62E-08
10	SNAPC5	small nuclear RNA activating complex polypeptide 5 [Source:HGNC S	4.83E-08
10	NFKBIL1	NFKB inhibitor like 1 [Source:HGNC Symbol;Acc:HGNC:7800]	1.22E-07
10	PTPA	protein phosphatase 2 phosphatase activator [Source:HGNC Symbol;	3.02E-07
10	CCDC160	coiled-coil domain containing 160 [Source:HGNC Symbol;Acc:HGNC:3	2.20E-29
10	RUFY1	RUN and FYVE domain containing 1 [Source:HGNC Symbol;Acc:HGNC	1.64E-28
10	HLA-DRB1	major histocompatibility complex, class II, DR beta 1 [Source:HGNC S	8.44E-20
10	PODXL2	podocalyxin like 2 [Source:HGNC Symbol;Acc:HGNC:17936]	1.90E-19
10	GATA3-AS1	GATA3 antisense RNA 1 [Source:HGNC Symbol;Acc:HGNC:33786]	3.20E-19
10	CENPF	centromere protein F [Source:HGNC Symbol;Acc:HGNC:1857]	3.28E-17
10	AGPS	alkylglycerone phosphate synthase [Source:HGNC Symbol;Acc:HGNC	3.80E-16
10	DCAF10	DDB1 and CUL4 associated factor 10 [Source:HGNC Symbol;Acc:HGN	3.93E-14
10	AKAP13	A-kinase anchoring protein 13 [Source:HGNC Symbol;Acc:HGNC:371]	1.86E-13
10	PPBP	pro-platelet basic protein [Source:HGNC Symbol;Acc:HGNC:9240]	2.92E-12
10	CYB561	cytochrome b561 [Source:HGNC Symbol;Acc:HGNC:2571]	3.71E-12
10	NMRAL1	NmrA like redox sensor 1 [Source:HGNC Symbol;Acc:HGNC:24987]	2.33E-11
10	MBOAT2	membrane bound O-acyltransferase domain containing 2 [Source:HG	7.30E-11
10	ALCAM	activated leukocyte cell adhesion molecule [Source:HGNC Symbol;Ac	3.64E-10
10	PPP2R5C	protein phosphatase 2 regulatory subunit B'gamma [Source:HGNC Sy	7.28E-10
10	PABPN1	poly(A) binding protein nuclear 1 [Source:HGNC Symbol;Acc:HGNC:8	6.50E-09
10	GNPDA1	glucosamine-6-phosphate deaminase 1 [Source:HGNC Symbol;Acc:H	2.39E-08
10	RNPEP	arginyl aminopeptidase [Source:HGNC Symbol;Acc:HGNC:10078]	4.47E-08
10	GMFG	glia maturation factor gamma [Source:HGNC Symbol;Acc:HGNC:4374	3.61E-57
10	VIM	vimentin [Source:HGNC Symbol;Acc:HGNC:12692]	7.98E-56
10	FYB1	FYN binding protein 1 [Source:HGNC Symbol;Acc:HGNC:4036]	5.53E-51
10	GPSM3	G protein signaling modulator 3 [Source:HGNC Symbol;Acc:HGNC:13	6.72E-50
10	TGFB1	transforming growth factor beta 1 [Source:HGNC Symbol;Acc:HGNC:	2.44E-49
10	EDN1	endothelin 1 [Source:HGNC Symbol;Acc:HGNC:3176]	2.61E-32
10	SLC2A10	solute carrier family 2 member 10 [Source:HGNC Symbol;Acc:HGNC:	2.29E-25
10	ERICH5	glutamate rich 5 [Source:HGNC Symbol;Acc:HGNC:26823]	3.63E-25
10	EVL	Enah/Vasp-like [Source:HGNC Symbol;Acc:HGNC:20234]	6.38E-23
10	ALDH7A1	aldehyde dehydrogenase 7 family member A1 [Source:HGNC Symbol	2.40E-22
10	DLG3	discs large MAGUK scaffold protein 3 [Source:HGNC Symbol;Acc:HGN	1.23E-21
10	PPFIBP1	PPFIA binding protein 1 [Source:HGNC Symbol;Acc:HGNC:9249]	2.12E-20
10	PRSS22	serine protease 22 [Source:HGNC Symbol;Acc:HGNC:14368]	2.11E-17
10	RAP2B	RAP2B, member of RAS oncogene family [Source:HGNC Symbol;Acc:	7.87E-15

10	RHOBTB3	Rho related BTB domain containing 3 [Source:HGNC Symbol;Acc:HGI	1.94E-13
10	UAP1	UDP-N-acetylglucosamine pyrophosphorylase 1 [Source:HGNC Symb	1.12E-11
10	BNIP2	BCL2 interacting protein 2 [Source:HGNC Symbol;Acc:HGNC:1083]	1.03E-10
10	CMBL	carboxymethylenebutenolidase homolog [Source:HGNC Symbol;Acc:	2.15E-10
10	MT-ATP8	mitochondrially encoded ATP synthase membrane subunit 8 [Source	2.57E-10
10	MFSD1	major facilitator superfamily domain containing 1 [Source:HGNC Sym	3.00E-10
10	CRIP2	cysteine rich protein 2 [Source:HGNC Symbol;Acc:HGNC:2361]	2.19E-09
10	CYTIP	cytohesin 1 interacting protein [Source:HGNC Symbol;Acc:HGNC:950	1.05E-08
10	TRA2A	transformer 2 alpha homolog [Source:HGNC Symbol;Acc:HGNC:1664	5.50E-08
10	MAGEF1	MAGE family member F1 [Source:HGNC Symbol;Acc:HGNC:29639]	7.00E-08
10	TAX1BP3	Tax1 binding protein 3 [Source:HGNC Symbol;Acc:HGNC:30684]	1.81E-07
10	GPATCH2	G-patch domain containing 2 [Source:HGNC Symbol;Acc:HGNC:2549	1.93E-07
10	RDH11	retinol dehydrogenase 11 [Source:HGNC Symbol;Acc:HGNC:17964]	2.33E-07
10	HCST	hematopoietic cell signal transducer [Source:HGNC Symbol;Acc:HGN	8.16E-46
10	NME2	NME/NM23 nucleoside diphosphate kinase 2 [Source:HGNC Symbol;	2.45E-23
10	IGSF21	immunoglobin superfamily member 21 [Source:HGNC Symbol;Acc:H	4.67E-22
10	PCYOX1	prenylcysteine oxidase 1 [Source:HGNC Symbol;Acc:HGNC:20588]	6.41E-21
10	CXADR	CXADR Ig-like cell adhesion molecule [Source:HGNC Symbol;Acc:HGN	1.95E-19
10	APOO	apolipoprotein O [Source:HGNC Symbol;Acc:HGNC:28727]	1.99E-18
10	EXOSC3	exosome component 3 [Source:HGNC Symbol;Acc:HGNC:17944]	1.94E-17
10	LAD1	ladinin 1 [Source:HGNC Symbol;Acc:HGNC:6472]	3.80E-17
10	VASP	vasodilator stimulated phosphoprotein [Source:HGNC Symbol;Acc:H	6.24E-17
10	TNFAIP8	TNF alpha induced protein 8 [Source:HGNC Symbol;Acc:HGNC:17260	9.35E-16
10	NCK2	NCK adaptor protein 2 [Source:HGNC Symbol;Acc:HGNC:7665]	3.48E-15
10	POR	cytochrome p450 oxidoreductase [Source:HGNC Symbol;Acc:HGNC:	2.38E-12
10	PTPN18	protein tyrosine phosphatase non-receptor type 18 [Source:HGNC Sy	4.87E-12
10	TP53RK	TP53 regulating kinase [Source:HGNC Symbol;Acc:HGNC:16197]	6.76E-12
10	YIPF2	Yip1 domain family member 2 [Source:HGNC Symbol;Acc:HGNC:284	1.23E-11
10	EPS8L2	EPS8 like 2 [Source:HGNC Symbol;Acc:HGNC:21296]	4.83E-11
10	CARD19	caspase recruitment domain family member 19 [Source:HGNC Symb	4.87E-10
10	PHF20L1	PHD finger protein 20 like 1 [Source:HGNC Symbol;Acc:HGNC:24280	1.48E-09
10	CDKN2D	cyclin dependent kinase inhibitor 2D [Source:HGNC Symbol;Acc:HGN	1.00E-35
10	CD74	CD74 molecule [Source:HGNC Symbol;Acc:HGNC:1697]	1.23E-35
10	PYCR1	pyrroline-5-carboxylate reductase 1 [Source:HGNC Symbol;Acc:HGN	4.50E-23
10	MDK	midkine [Source:HGNC Symbol;Acc:HGNC:6972]	2.77E-20
10	TPD52L1	TPD52 like 1 [Source:HGNC Symbol;Acc:HGNC:12006]	5.12E-20
10	LTB	lymphotoxin beta [Source:HGNC Symbol;Acc:HGNC:6711]	9.69E-20
10	PLLP	plasmolipin [Source:HGNC Symbol;Acc:HGNC:18553]	1.19E-19
10	C15orf48	chromosome 15 open reading frame 48 [Source:HGNC Symbol;Acc:H	1.20E-16
10	R3HDM4	R3H domain containing 4 [Source:HGNC Symbol;Acc:HGNC:28270]	2.09E-13
10	TUBG1	tubulin gamma 1 [Source:HGNC Symbol;Acc:HGNC:12417]	2.94E-12
10	CNN2	calponin 2 [Source:HGNC Symbol;Acc:HGNC:2156]	1.02E-11
10	STK17A	serine/threonine kinase 17a [Source:HGNC Symbol;Acc:HGNC:11395	1.61E-11
10	NCSTN	nicastrin [Source:HGNC Symbol;Acc:HGNC:17091]	1.25E-09
10	RAB34	RAB34, member RAS oncogene family [Source:HGNC Symbol;Acc:HG	1.85E-09
10	HACD4	3-hydroxyacyl-CoA dehydratase 4 [Source:HGNC Symbol;Acc:HGNC:	7.38E-09
10	MED8	mediator complex subunit 8 [Source:HGNC Symbol;Acc:HGNC:19971	2.87E-07

10	PTPRC	protein tyrosine phosphatase receptor type C [Source:HGNC Symbol	1.44E-66
10	CD52	CD52 molecule [Source:HGNC Symbol;Acc:HGNC:1804]	1.30E-47
10	COTL1	coactosin like F-actin binding protein 1 [Source:HGNC Symbol;Acc:HG	3.96E-46
10	LAT	linker for activation of T cells [Source:HGNC Symbol;Acc:HGNC:1887	3.76E-27
10	STK17B	serine/threonine kinase 17b [Source:HGNC Symbol;Acc:HGNC:11396	2.95E-25
10	PRR15L	proline rich 15 like [Source:HGNC Symbol;Acc:HGNC:28149]	1.26E-19
10	PARVA	parvin alpha [Source:HGNC Symbol;Acc:HGNC:14652]	4.50E-19
10	BACE2	beta-secretase 2 [Source:HGNC Symbol;Acc:HGNC:934]	4.60E-17
10	CYP4X1	cytochrome P450 family 4 subfamily X member 1 [Source:HGNC Sym	2.23E-16
10	KIAA1522	KIAA1522 [Source:HGNC Symbol;Acc:HGNC:29301]	9.08E-16
10	RNF11	ring finger protein 11 [Source:HGNC Symbol;Acc:HGNC:10056]	8.91E-11
10	TOM1L1	target of myb1 like 1 membrane trafficking protein [Source:HGNC Sy	1.14E-09
10	C6orf62	chromosome 6 open reading frame 62 [Source:HGNC Symbol;Acc:HG	2.19E-08
10	HDGFL3	HDGF like 3 [Source:HGNC Symbol;Acc:HGNC:24937]	1.32E-07
10	MPZL1	myelin protein zero like 1 [Source:HGNC Symbol;Acc:HGNC:7226]	1.44E-07
10	FLNA	filamin A [Source:HGNC Symbol;Acc:HGNC:3754]	6.70E-33
10	CLCA2	chloride channel accessory 2 [Source:HGNC Symbol;Acc:HGNC:2016]	6.47E-24
10	NUF2	NUF2 component of NDC80 kinetochore complex [Source:HGNC Sym	1.03E-20
10	PF4	platelet factor 4 [Source:HGNC Symbol;Acc:HGNC:8861]	8.78E-17
10	VCL	vinculin [Source:HGNC Symbol;Acc:HGNC:12665]	3.50E-16
10	MYO6	myosin VI [Source:HGNC Symbol;Acc:HGNC:7605]	1.71E-15
10	TUSC1	tumor suppressor candidate 1 [Source:HGNC Symbol;Acc:HGNC:310	9.58E-13
10	IFT43	intraflagellar transport 43 [Source:HGNC Symbol;Acc:HGNC:29669]	1.69E-11
10	IL32	interleukin 32 [Source:HGNC Symbol;Acc:HGNC:16830]	2.00E-11
10	SHMT2	serine hydroxymethyltransferase 2 [Source:HGNC Symbol;Acc:HGNC	9.27E-11
10	EIF2B3	eukaryotic translation initiation factor 2B subunit gamma [Source:HG	1.55E-10
10	ST3GAL6	ST3 beta-galactoside alpha-2,3-sialyltransferase 6 [Source:HGNC Sym	7.40E-09
10	NME6	NME/NM23 nucleoside diphosphate kinase 6 [Source:HGNC Symbol;	9.84E-09
10	NR2C2AP	nuclear receptor 2C2 associated protein [Source:HGNC Symbol;Acc:H	1.07E-08
10	VPS13C	vacuolar protein sorting 13 homolog C [Source:HGNC Symbol;Acc:HG	1.38E-08
10	FGFR1OP2	FGFR1 oncogene partner 2 [Source:HGNC Symbol;Acc:HGNC:23098]	1.49E-08
10	SPATS2L	spermatogenesis associated serine rich 2 like [Source:HGNC Symbol;	3.06E-08
10	SH3KBP1	SH3 domain containing kinase binding protein 1 [Source:HGNC Symb	3.58E-08
10	BTG3	BTG anti-proliferation factor 3 [Source:HGNC Symbol;Acc:HGNC:113	5.39E-07
10	CORO1A	coronin 1A [Source:HGNC Symbol;Acc:HGNC:2252]	1.06E-63
10	CD37	CD37 molecule [Source:HGNC Symbol;Acc:HGNC:1666]	6.41E-53
10	ARL4D	ADP ribosylation factor like GTPase 4D [Source:HGNC Symbol;Acc:HG	2.90E-22
10	COBL	cordon-bleu WH2 repeat protein [Source:HGNC Symbol;Acc:HGNC:2	1.27E-20
10	CASZ1	castor zinc finger 1 [Source:HGNC Symbol;Acc:HGNC:26002]	3.12E-18
10	COX7A1	cytochrome c oxidase subunit 7A1 [Source:HGNC Symbol;Acc:HGNC:	4.13E-18
10	FOXA3	forkhead box A3 [Source:HGNC Symbol;Acc:HGNC:5023]	7.12E-18
10	PKP3	plakophilin 3 [Source:HGNC Symbol;Acc:HGNC:9025]	7.18E-18
10	PHYHD1	phytanoyl-CoA dioxygenase domain containing 1 [Source:HGNC Sym	1.07E-15
10	SLC49A3	solute carrier family 49 member 3 [Source:HGNC Symbol;Acc:HGNC:	2.15E-12
10	TRIB3	tribbles pseudokinase 3 [Source:HGNC Symbol;Acc:HGNC:16228]	4.94E-11
10	EFNA4	ephrin A4 [Source:HGNC Symbol;Acc:HGNC:3224]	2.73E-09
10	CD3E	CD3e molecule [Source:HGNC Symbol;Acc:HGNC:1674]	4.82E-09

10	ALDOA	aldolase, fructose-bisphosphate A [Source:HGNC Symbol;Acc:HGNC:4	6.85E-09
10	KPNA2	karyopherin subunit alpha 2 [Source:HGNC Symbol;Acc:HGNC:6395]	8.39E-08
10	HSD17B8	hydroxysteroid 17-beta dehydrogenase 8 [Source:HGNC Symbol;Acc:	4.01E-07
10	FERMT3	fermitin family member 3 [Source:HGNC Symbol;Acc:HGNC:23151]	1.05E-37
10	CD48	CD48 molecule [Source:HGNC Symbol;Acc:HGNC:1683]	1.35E-36
10	RASGRP2	RAS guanyl releasing protein 2 [Source:HGNC Symbol;Acc:HGNC:987	4.31E-24
10	MESP1	mesoderm posterior bHLH transcription factor 1 [Source:HGNC Symb	1.62E-21
10	BCAS1	brain enriched myelin associated protein 1 [Source:HGNC Symbol;Ac	1.55E-20
10	TMC4	transmembrane channel like 4 [Source:HGNC Symbol;Acc:HGNC:229	2.01E-19
10	ZYX	zyxin [Source:HGNC Symbol;Acc:HGNC:13200]	2.09E-19
10	NT5C3A	5'-nucleotidase, cytosolic IIIA [Source:HGNC Symbol;Acc:HGNC:1782	7.48E-19
10	STAP2	signal transducing adaptor family member 2 [Source:HGNC Symbol;A	2.42E-18
10	EPB41	erythrocyte membrane protein band 4.1 [Source:HGNC Symbol;Acc:	1.95E-17
10	PIGR	polymeric immunoglobulin receptor [Source:HGNC Symbol;Acc:HGN	2.62E-17
10	DAPP1	dual adaptor of phosphotyrosine and 3-phosphoinositides 1 [Source:	1.68E-16
10	TUBA1A	tubulin alpha 1a [Source:HGNC Symbol;Acc:HGNC:20766]	4.57E-16
10	NTN4	netrin 4 [Source:HGNC Symbol;Acc:HGNC:13658]	9.39E-16
10	EIF2AK1	eukaryotic translation initiation factor 2 alpha kinase 1 [Source:HGN	2.89E-15
10	CFB	complement factor B [Source:HGNC Symbol;Acc:HGNC:1037]	6.32E-14
10	PER2	period circadian regulator 2 [Source:HGNC Symbol;Acc:HGNC:8846]	1.88E-12
10	MIR4435-2HG	MIR4435-2 host gene [Source:HGNC Symbol;Acc:HGNC:35163]	3.73E-12
10	CDC42EP1	CDC42 effector protein 1 [Source:HGNC Symbol;Acc:HGNC:17014]	4.31E-12
10	HYAL2	hyaluronidase 2 [Source:HGNC Symbol;Acc:HGNC:5321]	6.20E-12
10	METTL18	methyltransferase like 18 [Source:HGNC Symbol;Acc:HGNC:28793]	6.66E-12
10	REL	REL proto-oncogene, NF-kB subunit [Source:HGNC Symbol;Acc:HGN	9.14E-12
10	CYB5R3	cytochrome b5 reductase 3 [Source:HGNC Symbol;Acc:HGNC:2873]	1.55E-11
10	HAGHL	hydroxyacylglutathione hydrolase like [Source:HGNC Symbol;Acc:HG	1.71E-11
10	PALLD	palladin, cytoskeletal associated protein [Source:HGNC Symbol;Acc:H	7.42E-11
10	PCGF5	polycomb group ring finger 5 [Source:HGNC Symbol;Acc:HGNC:2826	9.37E-11
10	PSMA3-AS1	PSMA3 antisense RNA 1 [Source:HGNC Symbol;Acc:HGNC:26445]	1.09E-08
10	KYAT3	kynurenine aminotransferase 3 [Source:HGNC Symbol;Acc:HGNC:33	1.38E-08
10	PXMP2	peroxisomal membrane protein 2 [Source:HGNC Symbol;Acc:HGNC:9	2.25E-08
10	IL7R	interleukin 7 receptor [Source:HGNC Symbol;Acc:HGNC:6024]	2.24E-07
10	LAPTM5	lysosomal protein transmembrane 5 [Source:HGNC Symbol;Acc:HGN	7.73E-50
10	ELF1	E74 like ETS transcription factor 1 [Source:HGNC Symbol;Acc:HGNC:3	4.30E-27
10	GNG11	G protein subunit gamma 11 [Source:HGNC Symbol;Acc:HGNC:4403]	5.60E-21
10	CNNM1	cyclin and CBS domain divalent metal cation transport mediator 1 [S	1.91E-20
10	TMEM41A	transmembrane protein 41A [Source:HGNC Symbol;Acc:HGNC:30544	1.24E-19
10	RASEF	RAS and EF-hand domain containing [Source:HGNC Symbol;Acc:HGN	1.41E-18
10	PCDH1	protocadherin 1 [Source:HGNC Symbol;Acc:HGNC:8655]	2.40E-18
10	TTC39A	tetratricopeptide repeat domain 39A [Source:HGNC Symbol;Acc:HGN	8.16E-17
10	ETS1	ETS proto-oncogene 1, transcription factor [Source:HGNC Symbol;Ac	7.55E-15
10	AC025154.2	novel transcript, antisense to AQP5	1.55E-14
10	BSPRY	B-box and SPRY domain containing [Source:HGNC Symbol;Acc:HGNC	3.19E-14
10	CANT1	calcium activated nucleotidase 1 [Source:HGNC Symbol;Acc:HGNC:1	1.37E-13
10	IFITM1	interferon induced transmembrane protein 1 [Source:HGNC Symbol;	1.80E-13
10	SPR	sepiapterin reductase [Source:HGNC Symbol;Acc:HGNC:11257]	1.06E-11

10	BMPR1B	bone morphogenetic protein receptor type 1B [Source:HGNC Symbo	1.10E-11
10	IDUA	alpha-L-iduronidase [Source:HGNC Symbol;Acc:HGNC:5391]	1.25E-11
10	UBAC2	UBA domain containing 2 [Source:HGNC Symbol;Acc:HGNC:20486]	5.21E-11
10	WTAP	WT1 associated protein [Source:HGNC Symbol;Acc:HGNC:16846]	2.63E-10
10	HSPBP1	HSPA (Hsp70) binding protein 1 [Source:HGNC Symbol;Acc:HGNC:24	3.12E-10
10	TRNT1	tRNA nucleotidyl transferase 1 [Source:HGNC Symbol;Acc:HGNC:173	2.57E-09
10	SPNS1	sphingolipid transporter 1 (putative) [Source:HGNC Symbol;Acc:HGN	2.88E-09
10	AKAP1	A-kinase anchoring protein 1 [Source:HGNC Symbol;Acc:HGNC:367]	1.17E-08
10	MAP1LC3A	microtubule associated protein 1 light chain 3 alpha [Source:HGNC S	3.30E-08
10	ERBIN	erbb2 interacting protein [Source:HGNC Symbol;Acc:HGNC:15842]	9.25E-08
10	TRAC	T cell receptor alpha constant [Source:HGNC Symbol;Acc:HGNC:1202	2.14E-07
10	DGKD	diacylglycerol kinase delta [Source:HGNC Symbol;Acc:HGNC:2851]	3.16E-07
10	CLCN3	chloride voltage-gated channel 3 [Source:HGNC Symbol;Acc:HGNC:2	5.74E-07
10	PIP4K2A	phosphatidylinositol-5-phosphate 4-kinase type 2 alpha [Source:HGN	1.17E-33
10	RNF39	ring finger protein 39 [Source:HGNC Symbol;Acc:HGNC:18064]	1.54E-20
10	AC068888.1	novel transcript, antisense to TENC1 & EIF4B	1.74E-17
10	TINAGL1	tubulointerstitial nephritis antigen like 1 [Source:HGNC Symbol;Acc:H	3.09E-17
10	TMEM177	transmembrane protein 177 [Source:HGNC Symbol;Acc:HGNC:28143	9.75E-17
10	AP1S2	adaptor related protein complex 1 subunit sigma 2 [Source:HGNC Sy	4.61E-16
10	TMEM125	transmembrane protein 125 [Source:HGNC Symbol;Acc:HGNC:28275	7.92E-16
10	FAM110C	family with sequence similarity 110 member C [Source:HGNC Symbo	3.03E-15
10	NECTIN4	nectin cell adhesion molecule 4 [Source:HGNC Symbol;Acc:HGNC:19	3.75E-15
10	SEZ6L2	seizure related 6 homolog like 2 [Source:HGNC Symbol;Acc:HGNC:30	3.87E-15
10	WDR55	WD repeat domain 55 [Source:HGNC Symbol;Acc:HGNC:25971]	6.79E-15
10	ITPKC	inositol-trisphosphate 3-kinase C [Source:HGNC Symbol;Acc:HGNC:1	7.81E-15
10	FAM174B	family with sequence similarity 174 member B [Source:HGNC Symbo	1.01E-14
10	BHLHE41	basic helix-loop-helix family member e41 [Source:HGNC Symbol;Acc:	2.09E-13
10	CD69	CD69 molecule [Source:HGNC Symbol;Acc:HGNC:1694]	2.18E-13
10	GIMAP7	GTPase, IMAP family member 7 [Source:HGNC Symbol;Acc:HGNC:22	1.44E-12
10	IL17RC	interleukin 17 receptor C [Source:HGNC Symbol;Acc:HGNC:18358]	7.05E-12
10	TMEM25	transmembrane protein 25 [Source:HGNC Symbol;Acc:HGNC:25890]	7.02E-12
10	IDS	iduronate 2-sulfatase [Source:HGNC Symbol;Acc:HGNC:5389]	8.03E-12
10	PSMB10	proteasome 20S subunit beta 10 [Source:HGNC Symbol;Acc:HGNC:9	8.23E-11
10	LYPD3	LY6/PLAUR domain containing 3 [Source:HGNC Symbol;Acc:HGNC:24	1.09E-10
10	PIK3R1	phosphoinositide-3-kinase regulatory subunit 1 [Source:HGNC Symb	1.79E-10
10	C20orf96	chromosome 20 open reading frame 96 [Source:HGNC Symbol;Acc:H	7.77E-09
10	RPP25L	ribonuclease P/MRP subunit p25 like [Source:HGNC Symbol;Acc:HGN	1.00E-08
10	FOXC1	forkhead box C1 [Source:HGNC Symbol;Acc:HGNC:3800]	1.04E-08
10	UBE2V1	ubiquitin conjugating enzyme E2 V1 [Source:HGNC Symbol;Acc:HGN	1.13E-08
10	IVD	isovaleryl-CoA dehydrogenase [Source:HGNC Symbol;Acc:HGNC:618	1.15E-08
10	HLA-DQB1	major histocompatibility complex, class II, DQ beta 1 [Source:HGNC S	3.10E-08
10	BDH1	3-hydroxybutyrate dehydrogenase 1 [Source:HGNC Symbol;Acc:HGN	1.26E-07
10	RILPL2	Rab interacting lysosomal protein like 2 [Source:HGNC Symbol;Acc:H	2.32E-07
10	LIMD2	LIM domain containing 2 [Source:HGNC Symbol;Acc:HGNC:28142]	4.53E-45
10	GP2	glycoprotein 2 [Source:HGNC Symbol;Acc:HGNC:4441]	1.61E-28
10	FAM107B	family with sequence similarity 107 member B [Source:HGNC Symbo	1.76E-24
10	SFN	stratifin [Source:HGNC Symbol;Acc:HGNC:10773]	3.45E-24

10	AGR3	anterior gradient 3, protein disulphide isomerase family member [So	5.48E-24
10	ADD3	adducin 3 [Source:HGNC Symbol;Acc:HGNC:245]	3.69E-23
10	ERP27	endoplasmic reticulum protein 27 [Source:HGNC Symbol;Acc:HGNC:2	2.62E-22
10	CD53	CD53 molecule [Source:HGNC Symbol;Acc:HGNC:1686]	4.49E-19
10	BICDL2	BICD family like cargo adaptor 2 [Source:HGNC Symbol;Acc:HGNC:33	1.99E-18
10	EDARADD	EDAR associated death domain [Source:HGNC Symbol;Acc:HGNC:143	5.54E-18
10	CADPS2	calcium dependent secretion activator 2 [Source:HGNC Symbol;Acc:H	1.42E-16
10	MANSC1	MANSC domain containing 1 [Source:HGNC Symbol;Acc:HGNC:25505	1.19E-15
10	DIAPH1	diaphanous related formin 1 [Source:HGNC Symbol;Acc:HGNC:2876]	1.38E-15
10	CBR3	carbonyl reductase 3 [Source:HGNC Symbol;Acc:HGNC:1549]	5.76E-15
10	ANKRD30A	ankyrin repeat domain 30A [Source:HGNC Symbol;Acc:HGNC:17234]	6.55E-14
10	TSPAN6	tetraspanin 6 [Source:HGNC Symbol;Acc:HGNC:11858]	7.00E-14
10	TSC22D4	TSC22 domain family member 4 [Source:HGNC Symbol;Acc:HGNC:21	3.15E-13
10	CKMT1B	creatine kinase, mitochondrial 1B [Source:HGNC Symbol;Acc:HGNC:1	3.54E-13
10	EPN2	epsin 2 [Source:HGNC Symbol;Acc:HGNC:18639]	6.75E-12
10	VEGFA	vascular endothelial growth factor A [Source:HGNC Symbol;Acc:HGN	1.08E-11
10	MAP7	microtubule associated protein 7 [Source:HGNC Symbol;Acc:HGNC:6	6.31E-11
10	KLF13	Kruppel like factor 13 [Source:HGNC Symbol;Acc:HGNC:13672]	8.22E-11
10	CREBRF	CREB3 regulatory factor [Source:HGNC Symbol;Acc:HGNC:24050]	2.54E-10
10	CYLD	CYLD lysine 63 deubiquitinase [Source:HGNC Symbol;Acc:HGNC:2584	4.18E-10
10	KAT6A	lysine acetyltransferase 6A [Source:HGNC Symbol;Acc:HGNC:13013]	4.25E-10
10	MAP2K3	mitogen-activated protein kinase kinase 3 [Source:HGNC Symbol;Acc	6.97E-09
10	UTRN	utrophin [Source:HGNC Symbol;Acc:HGNC:12635]	1.27E-08
10	ZNF138	zinc finger protein 138 [Source:HGNC Symbol;Acc:HGNC:12922]	4.31E-08
10	MAP3K20	mitogen-activated protein kinase kinase kinase 20 [Source:HGNC Syr	9.58E-08
10	AFDN	afadin, adherens junction formation factor [Source:HGNC Symbol;Ac	9.75E-08
10	RAC2	Rac family small GTPase 2 [Source:HGNC Symbol;Acc:HGNC:9802]	5.39E-51
10	TBX3	T-box transcription factor 3 [Source:HGNC Symbol;Acc:HGNC:11602]	1.70E-24
10	SOD2	superoxide dismutase 2 [Source:HGNC Symbol;Acc:HGNC:11180]	8.88E-24
10	PIP	prolactin induced protein [Source:HGNC Symbol;Acc:HGNC:8993]	8.17E-23
10	NOL3	nucleolar protein 3 [Source:HGNC Symbol;Acc:HGNC:7869]	8.32E-23
10	C5orf66-AS1	C5orf66 antisense RNA 1 [Source:HGNC Symbol;Acc:HGNC:49679]	6.73E-21
10	TRAF3IP3	TRAF3 interacting protein 3 [Source:HGNC Symbol;Acc:HGNC:30766]	2.08E-19
10	FAM3B	FAM3 metabolism regulating signaling molecule B [Source:HGNC Syr	5.97E-19
10	SELL	selectin L [Source:HGNC Symbol;Acc:HGNC:10720]	2.70E-16
10	RRNAD1	ribosomal RNA adenine dimethylase domain containing 1 [Source:HC	4.33E-16
10	RTKN	rhotekin [Source:HGNC Symbol;Acc:HGNC:10466]	7.67E-16
10	SIX1	SIX homeobox 1 [Source:HGNC Symbol;Acc:HGNC:10887]	5.06E-15
10	TACC2	transforming acidic coiled-coil containing protein 2 [Source:HGNC Sy	6.97E-14
10	TJP3	tight junction protein 3 [Source:HGNC Symbol;Acc:HGNC:11829]	8.98E-14
10	RAB40B	RAB40B, member RAS oncogene family [Source:HGNC Symbol;Acc:H	5.72E-13
10	FMOD	fibromodulin [Source:HGNC Symbol;Acc:HGNC:3774]	1.55E-12
10	ATM	ATM serine/threonine kinase [Source:HGNC Symbol;Acc:HGNC:795]	2.74E-11
10	PTPN6	protein tyrosine phosphatase non-receptor type 6 [Source:HGNC Syr	3.14E-11
10	PTPRF	protein tyrosine phosphatase receptor type F [Source:HGNC Symbol;	2.78E-09
10	GSTO2	glutathione S-transferase omega 2 [Source:HGNC Symbol;Acc:HGNC:	5.54E-09
10	DERA	deoxyribose-phosphate aldolase [Source:HGNC Symbol;Acc:HGNC:24	4.05E-08

10	BIN2	bridging integrator 2 [Source:HGNC Symbol;Acc:HGNC:1053]	5.81E-58
10	MSN	moesin [Source:HGNC Symbol;Acc:HGNC:7373]	2.96E-37
10	MBP	myelin basic protein [Source:HGNC Symbol;Acc:HGNC:6925]	1.87E-30
10	LCP1	lymphocyte cytosolic protein 1 [Source:HGNC Symbol;Acc:HGNC:652	2.56E-26
10	ICAM2	intercellular adhesion molecule 2 [Source:HGNC Symbol;Acc:HGNC:5	9.73E-23
10	MYCBP2	MYC binding protein 2 [Source:HGNC Symbol;Acc:HGNC:23386]	2.33E-19
10	TMEM40	transmembrane protein 40 [Source:HGNC Symbol;Acc:HGNC:25620]	1.61E-18
10	SRARP	steroid receptor associated and regulated protein [Source:HGNC Sym	5.34E-18
10	CSK	C-terminal Src kinase [Source:HGNC Symbol;Acc:HGNC:2444]	2.99E-17
10	RAB17	RAB17, member RAS oncogene family [Source:HGNC Symbol;Acc:HG	1.03E-16
10	SLPI	secretory leukocyte peptidase inhibitor [Source:HGNC Symbol;Acc:H	4.14E-16
10	IRX5	iroquois homeobox 5 [Source:HGNC Symbol;Acc:HGNC:14361]	3.64E-15
10	IL2RG	interleukin 2 receptor subunit gamma [Source:HGNC Symbol;Acc:HG	5.62E-15
10	CMYA5	cardiomyopathy associated 5 [Source:HGNC Symbol;Acc:HGNC:1430	6.65E-14
10	DNAAF4	dynein axonemal assembly factor 4 [Source:HGNC Symbol;Acc:HGNC	3.06E-13
10	GALE	UDP-galactose-4-epimerase [Source:HGNC Symbol;Acc:HGNC:4116]	1.04E-12
10	IGFBP4	insulin like growth factor binding protein 4 [Source:HGNC Symbol;Ac	2.33E-11
10	SPRYD4	SPRY domain containing 4 [Source:HGNC Symbol;Acc:HGNC:27468]	5.36E-11
10	CEACAM6	CEA cell adhesion molecule 6 [Source:HGNC Symbol;Acc:HGNC:1818	2.25E-10
10	NUTM2A-AS1	NUTM2A antisense RNA 1 [Source:HGNC Symbol;Acc:HGNC:45161]	1.71E-09
10	RHPN1	rhophilin Rho GTPase binding protein 1 [Source:HGNC Symbol;Acc:H	1.96E-09
10	PLEKHA5	pleckstrin homology domain containing A5 [Source:HGNC Symbol;Ac	1.21E-08
10	ACAP1	ArfGAP with coiled-coil, ankyrin repeat and PH domains 1 [Source:H	1.37E-08
10	PWWP2B	PWWP domain containing 2B [Source:HGNC Symbol;Acc:HGNC:2515	5.74E-08
10	LMTK3	lemur tyrosine kinase 3 [Source:HGNC Symbol;Acc:HGNC:19295]	3.74E-20
10	BTN3A2	butyrophilin subfamily 3 member A2 [Source:HGNC Symbol;Acc:HGN	4.97E-20
10	HCLS1	hematopoietic cell-specific Lyn substrate 1 [Source:HGNC Symbol;Ac	1.59E-17
10	TRBC2	T cell receptor beta constant 2 [Source:HGNC Symbol;Acc:HGNC:121	1.98E-16
10	ERICH1	glutamate rich 1 [Source:HGNC Symbol;Acc:HGNC:27234]	2.38E-15
10	SMPDL3B	sphingomyelin phosphodiesterase acid like 3B [Source:HGNC Symbol	3.41E-15
10	CYSRT1	cysteine rich tail 1 [Source:HGNC Symbol;Acc:HGNC:30529]	1.41E-14
10	AL049839.2	novel protein	1.74E-14
10	FAM181B	family with sequence similarity 181 member B [Source:HGNC Symbo	5.45E-14
10	TMBIM1	transmembrane BAX inhibitor motif containing 1 [Source:HGNC Sym	1.50E-13
10	RABGAP1L	RAB GTPase activating protein 1 like [Source:HGNC Symbol;Acc:HGN	2.93E-13
10	CYP2J2	cytochrome P450 family 2 subfamily J member 2 [Source:HGNC Sym	3.08E-13
10	CNST	consortin, connexin sorting protein [Source:HGNC Symbol;Acc:HGNC	7.08E-13
10	MPZL2	myelin protein zero like 2 [Source:HGNC Symbol;Acc:HGNC:3496]	1.08E-12
10	AL355338.1	novel transcript	3.69E-12
10	HID1	HID1 domain containing [Source:HGNC Symbol;Acc:HGNC:15736]	3.77E-12
10	MCM4	minichromosome maintenance complex component 4 [Source:HGNC	3.62E-11
10	CYTOR	cytoskeleton regulator RNA [Source:HGNC Symbol;Acc:HGNC:28717]	7.08E-11
10	CKLF	chemokine like factor [Source:HGNC Symbol;Acc:HGNC:13253]	3.13E-10
10	DYNLL2	dynein light chain LC8-type 2 [Source:HGNC Symbol;Acc:HGNC:2459	3.49E-09
10	VWA1	von Willebrand factor A domain containing 1 [Source:HGNC Symbol;	4.29E-09
10	LCK	LCK proto-oncogene, Src family tyrosine kinase [Source:HGNC Symbo	7.20E-08
10	ARHGEF35	Rho guanine nucleotide exchange factor 35 [Source:HGNC Symbol;A	1.43E-07

10	KCTD3	potassium channel tetramerization domain containing 3 [Source:HGN	6.00E-07
10	GP9	glycoprotein IX platelet [Source:HGNC Symbol;Acc:HGNC:4444]	6.96E-28
10	ALDH3B2	aldehyde dehydrogenase 3 family member B2 [Source:HGNC Symbol	3.22E-16
10	SHROOM1	shroom family member 1 [Source:HGNC Symbol;Acc:HGNC:24084]	6.46E-15
10	ANO6	anoctamin 6 [Source:HGNC Symbol;Acc:HGNC:25240]	4.61E-13
10	ASAP1	ArfGAP with SH3 domain, ankyrin repeat and PH domain 1 [Source:H	1.08E-12
10	ARPIN	actin related protein 2/3 complex inhibitor [Source:HGNC Symbol;Ac	2.59E-10
10	SDSL	serine dehydratase like [Source:HGNC Symbol;Acc:HGNC:30404]	5.42E-10
10	CD7	CD7 molecule [Source:HGNC Symbol;Acc:HGNC:1695]	1.12E-09
10	STXBP3	syntaxin binding protein 3 [Source:HGNC Symbol;Acc:HGNC:11446]	7.20E-09
10	BPHL	biphenyl hydrolase like [Source:HGNC Symbol;Acc:HGNC:1094]	3.99E-08
10	ADAM10	ADAM metallopeptidase domain 10 [Source:HGNC Symbol;Acc:HGN	1.14E-07
10	TUBB1	tubulin beta 1 class VI [Source:HGNC Symbol;Acc:HGNC:16257]	2.42E-31
10	DOK2	docking protein 2 [Source:HGNC Symbol;Acc:HGNC:2991]	9.01E-31
10	VSIR	V-set immunoregulatory receptor [Source:HGNC Symbol;Acc:HGNC:3	2.57E-26
10	PALMD	palmdelphin [Source:HGNC Symbol;Acc:HGNC:15846]	1.02E-19
10	ARHGAP18	Rho GTPase activating protein 18 [Source:HGNC Symbol;Acc:HGNC:2	7.56E-17
10	ARL4C	ADP ribosylation factor like GTPase 4C [Source:HGNC Symbol;Acc:HG	1.14E-14
10	IKZF1	IKAROS family zinc finger 1 [Source:HGNC Symbol;Acc:HGNC:13176]	2.20E-13
10	PRRG2	proline rich and Gla domain 2 [Source:HGNC Symbol;Acc:HGNC:9470	1.10E-12
10	TMEM186	transmembrane protein 186 [Source:HGNC Symbol;Acc:HGNC:24530	5.04E-12
10	RRP9	ribosomal RNA processing 9, U3 small nucleolar RNA binding protein	1.08E-11
10	GPRC5A	G protein-coupled receptor class C group 5 member A [Source:HGNC	1.19E-11
10	SAMHD1	SAM and HD domain containing deoxynucleoside triphosphate tripho	1.80E-11
10	SRSF8	serine and arginine rich splicing factor 8 [Source:HGNC Symbol;Acc:H	2.63E-11
10	HBP1	HMG-box transcription factor 1 [Source:HGNC Symbol;Acc:HGNC:23	4.65E-10
10	STK38	serine/threonine kinase 38 [Source:HGNC Symbol;Acc:HGNC:17847]	2.77E-09
10	EHD1	EH domain containing 1 [Source:HGNC Symbol;Acc:HGNC:3242]	4.47E-09
10	ENDOD1	endonuclease domain containing 1 [Source:HGNC Symbol;Acc:HGNC	5.23E-09
10	PMEPA1	prostate transmembrane protein, androgen induced 1 [Source:HGN	5.69E-08
10	CIB2	calcium and integrin binding family member 2 [Source:HGNC Symbol	8.26E-08
10	AHCTF1	AT-hook containing transcription factor 1 [Source:HGNC Symbol;Acc:	5.75E-07
10	RGS4	regulator of G protein signaling 4 [Source:HGNC Symbol;Acc:HGNC:1	8.73E-19
10	C9orf116	chromosome 9 open reading frame 116 [Source:HGNC Symbol;Acc:H	8.72E-17
10	SIM2	SIM bHLH transcription factor 2 [Source:HGNC Symbol;Acc:HGNC:10	4.51E-14
10	PTGFRN	prostaglandin F2 receptor inhibitor [Source:HGNC Symbol;Acc:HGNC	3.58E-13
10	CDKN1A	cyclin dependent kinase inhibitor 1A [Source:HGNC Symbol;Acc:HGN	5.89E-13
10	SSPN	sarcospan [Source:HGNC Symbol;Acc:HGNC:11322]	7.41E-10
10	TMEM8B	transmembrane protein 8B [Source:HGNC Symbol;Acc:HGNC:21427]	2.84E-09
10	P2RY2	purinergic receptor P2Y2 [Source:HGNC Symbol;Acc:HGNC:8541]	4.93E-08
10	DHFR	dihydrofolate reductase [Source:HGNC Symbol;Acc:HGNC:2861]	5.21E-08
10	SEMA4D	semaphorin 4D [Source:HGNC Symbol;Acc:HGNC:10732]	1.26E-07
10	LDLRAD3	low density lipoprotein receptor class A domain containing 3 [Source	1.58E-07
10	RGS19	regulator of G protein signaling 19 [Source:HGNC Symbol;Acc:HGNC:	3.04E-07
10	LSP1	lymphocyte specific protein 1 [Source:HGNC Symbol;Acc:HGNC:6707	1.33E-32
10	GRAP2	GRB2 related adaptor protein 2 [Source:HGNC Symbol;Acc:HGNC:45	1.82E-21
10	STK24	serine/threonine kinase 24 [Source:HGNC Symbol;Acc:HGNC:11403]	1.88E-19

10	CXCR4	C-X-C motif chemokine receptor 4 [Source:HGNC Symbol;Acc:HGNC:2	3.84E-17
10	ANO1	anoctamin 1 [Source:HGNC Symbol;Acc:HGNC:21625]	2.37E-16
10	MTUS1	microtubule associated scaffold protein 1 [Source:HGNC Symbol;Acc	1.27E-14
10	RAB8B	RAB8B, member RAS oncogene family [Source:HGNC Symbol;Acc:HG	2.11E-14
10	RASAL2	RAS protein activator like 2 [Source:HGNC Symbol;Acc:HGNC:9874]	6.52E-13
10	GYPC	glycophorin C (Gerbich blood group) [Source:HGNC Symbol;Acc:HGN	3.59E-12
10	MPST	mercaptopyruvate sulfurtransferase [Source:HGNC Symbol;Acc:HGN	7.11E-12
10	ARHGAP21	Rho GTPase activating protein 21 [Source:HGNC Symbol;Acc:HGNC:2	2.31E-11
10	XPNPEP1	X-prolyl aminopeptidase 1 [Source:HGNC Symbol;Acc:HGNC:12822]	9.71E-11
10	FBXO7	F-box protein 7 [Source:HGNC Symbol;Acc:HGNC:13586]	1.54E-10
10	RYBP	RING1 and YY1 binding protein [Source:HGNC Symbol;Acc:HGNC:104	8.42E-10
10	RYBP	RING1 and YY1 binding protein [Source:NCBI gene (formerly Entrezge	8.42E-10
10	EMD	emerin [Source:HGNC Symbol;Acc:HGNC:3331]	2.53E-09
10	CELF2	CUGBP Elav-like family member 2 [Source:HGNC Symbol;Acc:HGNC:2	9.86E-33
10	OAZ3	ornithine decarboxylase antizyme 3 [Source:HGNC Symbol;Acc:HGN(	9.58E-12
10	B9D1	B9 domain containing 1 [Source:HGNC Symbol;Acc:HGNC:24123]	1.00E-11
10	PRKACB	protein kinase cAMP-activated catalytic subunit beta [Source:HGNC S	1.57E-11
10	ARRB2	arrestin beta 2 [Source:HGNC Symbol;Acc:HGNC:712]	2.83E-10
10	SMIM3	small integral membrane protein 3 [Source:HGNC Symbol;Acc:HGNC	3.11E-10
10	ARID1A	AT-rich interaction domain 1A [Source:HGNC Symbol;Acc:HGNC:111	1.37E-09
10	MEF2A	myocyte enhancer factor 2A [Source:HGNC Symbol;Acc:HGNC:6993]	2.14E-08
10	DGKZ	diacylglycerol kinase zeta [Source:HGNC Symbol;Acc:HGNC:2857]	2.25E-08
10	PLEKHO1	pleckstrin homology domain containing O1 [Source:HGNC Symbol;Ac	2.80E-45
10	MAX	MYC associated factor X [Source:HGNC Symbol;Acc:HGNC:6913]	9.09E-45
10	ODC1	ornithine decarboxylase 1 [Source:HGNC Symbol;Acc:HGNC:8109]	9.03E-34
10	SEC14L1	SEC14 like lipid binding 1 [Source:HGNC Symbol;Acc:HGNC:10698]	2.70E-17
10	FNBP1	formin binding protein 1 [Source:HGNC Symbol;Acc:HGNC:17069]	2.98E-16
10	RGCC	regulator of cell cycle [Source:HGNC Symbol;Acc:HGNC:20369]	3.97E-15
10	PLAC8	placenta associated 8 [Source:HGNC Symbol;Acc:HGNC:19254]	7.08E-14
10	GIMAP4	GTPase, IMAP family member 4 [Source:HGNC Symbol;Acc:HGNC:21	1.02E-13
10	CHD9	chromodomain helicase DNA binding protein 9 [Source:HGNC Symbo	6.31E-13
10	INF2	inverted formin 2 [Source:HGNC Symbol;Acc:HGNC:23791]	2.06E-12
10	ASB8	ankyrin repeat and SOCS box containing 8 [Source:HGNC Symbol;Acc	2.41E-11
10	LDLRAP1	low density lipoprotein receptor adaptor protein 1 [Source:HGNC Sy	4.15E-11
10	B3GALT5-AS1	B3GALT5 antisense RNA 1 [Source:HGNC Symbol;Acc:HGNC:16424]	3.77E-10
10	CPEB4	cytoplasmic polyadenylation element binding protein 4 [Source:HGN	1.03E-09
10	SUSD3	sushi domain containing 3 [Source:HGNC Symbol;Acc:HGNC:28391]	9.13E-09
10	SSH2	slingshot protein phosphatase 2 [Source:HGNC Symbol;Acc:HGNC:30	2.03E-08
10	WIPF1	WAS/WASL interacting protein family member 1 [Source:HGNC Symb	2.89E-33
10	APBB1IP	amyloid beta precursor protein binding family B member 1 interactin	2.28E-21
10	IFI27L2	interferon alpha inducible protein 27 like 2 [Source:HGNC Symbol;Ac	7.07E-21
10	PNP	purine nucleoside phosphorylase [Source:HGNC Symbol;Acc:HGNC:7	4.65E-17
10	IKBKG	inhibitor of nuclear factor kappa B kinase regulatory subunit gamma	1.47E-14
10	PRKAR2B	protein kinase cAMP-dependent type II regulatory subunit beta [Sou	7.07E-14
10	C1GALT1	core 1 synthase, glycoprotein-N-acetylgalactosamine 3-beta-galactos	2.72E-11
10	VAMP5	vesicle associated membrane protein 5 [Source:HGNC Symbol;Acc:H(	2.72E-10
10	FANCF	FA complementation group F [Source:HGNC Symbol;Acc:HGNC:3587	3.16E-08

10	LBH	LBH regulator of WNT signaling pathway [Source:HGNC Symbol;Acc:I	8.45E-08
10	TRBC1	T cell receptor beta constant 1 [Source:HGNC Symbol;Acc:HGNC:121	2.82E-07
10	DCK	deoxycytidine kinase [Source:HGNC Symbol;Acc:HGNC:2704]	5.15E-07
10	RAB32	RAB32, member RAS oncogene family [Source:HGNC Symbol;Acc:HG	2.75E-35
10	FYN	FYN proto-oncogene, Src family tyrosine kinase [Source:HGNC Symbo	3.44E-29
10	CMIP	c-Maf inducing protein [Source:HGNC Symbol;Acc:HGNC:24319]	1.41E-26
10	F13A1	coagulation factor XIII A chain [Source:HGNC Symbol;Acc:HGNC:3531	2.38E-23
10	MTURN	maturin, neural progenitor differentiation regulator homolog [Source	5.86E-22
10	YIF1B	Yip1 interacting factor homolog B, membrane trafficking protein [So	8.36E-20
10	GRK6	G protein-coupled receptor kinase 6 [Source:HGNC Symbol;Acc:HGN	1.07E-15
10	MAPK14	mitogen-activated protein kinase 14 [Source:HGNC Symbol;Acc:HGN	6.99E-15
10	PTCRA	pre T cell antigen receptor alpha [Source:HGNC Symbol;Acc:HGNC:2	9.87E-15
10	CCDC92	coiled-coil domain containing 92 [Source:HGNC Symbol;Acc:HGNC:2	2.22E-14
10	MLH3	mutL homolog 3 [Source:HGNC Symbol;Acc:HGNC:7128]	2.87E-13
10	PIP4P2	phosphatidylinositol-4,5-bisphosphate 4-phosphatase 2 [Source:HGN	7.82E-11
10	SERPINA1	serpin family A member 1 [Source:HGNC Symbol;Acc:HGNC:8941]	4.10E-10
10	CCDC88C	coiled-coil domain containing 88C [Source:HGNC Symbol;Acc:HGNC:	1.73E-09
10	GZMM	granzyme M [Source:HGNC Symbol;Acc:HGNC:4712]	2.90E-07
10	RGS18	regulator of G protein signaling 18 [Source:HGNC Symbol;Acc:HGNC:	9.11E-42
10	SLC2A3	solute carrier family 2 member 3 [Source:HGNC Symbol;Acc:HGNC:1	4.40E-28
10	RHOF	ras homolog family member F, filopodia associated [Source:HGNC Sy	5.29E-27
10	TLE4	TLE family member 4, transcriptional corepressor [Source:HGNC Sym	6.69E-19
10	CHMP4A	charged multivesicular body protein 4A [Source:HGNC Symbol;Acc:H	1.30E-16
10	PPM1A	protein phosphatase, Mg2+/Mn2+ dependent 1A [Source:HGNC Sym	1.05E-15
10	DUSP22	dual specificity phosphatase 22 [Source:HGNC Symbol;Acc:HGNC:16	6.06E-12
10	CNOT6L	CCR4-NOT transcription complex subunit 6 like [Source:HGNC Symbo	1.38E-11
10	ANKRD9	ankyrin repeat domain 9 [Source:HGNC Symbol;Acc:HGNC:20096]	1.06E-10
10	ERV3-1	endogenous retrovirus group 3 member 1, envelope [Source:HGNC S	1.12E-10
10	SAV1	salvador family WW domain containing protein 1 [Source:HGNC Sym	2.10E-10
10	ORAI2	ORAI calcium release-activated calcium modulator 2 [Source:HGNC S	3.19E-10
10	CD247	CD247 molecule [Source:HGNC Symbol;Acc:HGNC:1677]	4.84E-10
10	GRK2	G protein-coupled receptor kinase 2 [Source:HGNC Symbol;Acc:HGN	9.71E-10
10	EPS15	epidermal growth factor receptor pathway substrate 15 [Source:HGN	2.96E-08
10	TMX4	thioredoxin related transmembrane protein 4 [Source:HGNC Symbol	5.61E-08
10	FOXO3	forkhead box O3 [Source:HGNC Symbol;Acc:HGNC:3821]	5.41E-07
10	PARVB	parvin beta [Source:HGNC Symbol;Acc:HGNC:14653]	3.12E-21
10	NPC2	NPC intracellular cholesterol transporter 2 [Source:HGNC Symbol;Ac	5.14E-18
10	CAVIN2	caveolae associated protein 2 [Source:HGNC Symbol;Acc:HGNC:106	2.76E-17
10	TSPAN33	tetraspanin 33 [Source:HGNC Symbol;Acc:HGNC:28743]	1.01E-16
10	PIM1	Pim-1 proto-oncogene, serine/threonine kinase [Source:HGNC Symb	2.57E-16
10	C12orf76	chromosome 12 open reading frame 76 [Source:HGNC Symbol;Acc:H	4.59E-16
10	MEPCE	methylphosphate capping enzyme [Source:HGNC Symbol;Acc:HGNC:	7.74E-16
10	EVI2B	ecotropic viral integration site 2B [Source:HGNC Symbol;Acc:HGNC:3	9.87E-16
10	MARK3	microtubule affinity regulating kinase 3 [Source:HGNC Symbol;Acc:H	7.71E-15
10	PSMB8-AS1	PSMB8 antisense RNA 1 (head to head) [Source:HGNC Symbol;Acc:H	1.06E-14
10	PSMB8-AS1	PSMB8 antisense RNA 1 (head to head) [Source:NCBI gene (formerly	1.06E-14
10	TMEM91	transmembrane protein 91 [Source:HGNC Symbol;Acc:HGNC:32393]	1.23E-14

10	MINDY1	MINDY lysine 48 deubiquitinase 1 [Source:HGNC Symbol;Acc:HGNC:2	1.65E-14
10	MTRNR2L12	MT-RNR2 like 12 [Source:HGNC Symbol;Acc:HGNC:37169]	5.63E-14
10	UBA7	ubiquitin like modifier activating enzyme 7 [Source:HGNC Symbol;Ac	1.24E-12
10	UBE2J1	ubiquitin conjugating enzyme E2 J1 [Source:HGNC Symbol;Acc:HGNC	2.84E-12
10	MTFR1L	mitochondrial fission regulator 1 like [Source:HGNC Symbol;Acc:HGN	2.43E-10
10	NUMB	NUMB endocytic adaptor protein [Source:HGNC Symbol;Acc:HGNC:8	8.72E-09
10	FGD3	FYVE, RhoGEF and PH domain containing 3 [Source:HGNC Symbol;Ac	3.50E-08
10	ITGB2	integrin subunit beta 2 [Source:HGNC Symbol;Acc:HGNC:6155]	4.12E-29
10	PRKCD	protein kinase C delta [Source:HGNC Symbol;Acc:HGNC:9399]	1.31E-23
10	STRN4	striatin 4 [Source:HGNC Symbol;Acc:HGNC:15721]	2.13E-16
10	MAPK1	mitogen-activated protein kinase 1 [Source:HGNC Symbol;Acc:HGNC	1.55E-15
10	SPPL3	signal peptide peptidase like 3 [Source:HGNC Symbol;Acc:HGNC:304	1.72E-15
10	MIS18BP1	MIS18 binding protein 1 [Source:HGNC Symbol;Acc:HGNC:20190]	3.21E-15
10	CLEC2B	C-type lectin domain family 2 member B [Source:HGNC Symbol;Acc:H	2.34E-13
10	IFI16	interferon gamma inducible protein 16 [Source:HGNC Symbol;Acc:H	5.74E-13
10	RFLNB	refilin B [Source:HGNC Symbol;Acc:HGNC:28705]	8.95E-12
10	SYMPK	symplekin [Source:HGNC Symbol;Acc:HGNC:22935]	1.13E-11
10	RNF166	ring finger protein 166 [Source:HGNC Symbol;Acc:HGNC:28856]	1.86E-11
10	CMTM5	CKLF like MARVEL transmembrane domain containing 5 [Source:HGN	2.12E-11
10	MSANTD3	Myb/SANT DNA binding domain containing 3 [Source:HGNC Symbol;	4.62E-11
10	ARHGAP30	Rho GTPase activating protein 30 [Source:HGNC Symbol;Acc:HGNC:2	1.22E-10
10	PNMA1	PNMA family member 1 [Source:HGNC Symbol;Acc:HGNC:9158]	1.35E-10
10	IL16	interleukin 16 [Source:HGNC Symbol;Acc:HGNC:5980]	1.07E-08
10	HIPK2	homeodomain interacting protein kinase 2 [Source:HGNC Symbol;Ac	1.24E-08
10	CD96	CD96 molecule [Source:HGNC Symbol;Acc:HGNC:16892]	8.54E-08
10	MPP1	membrane palmitoylated protein 1 [Source:HGNC Symbol;Acc:HGNC	1.56E-33
10	PRKCB	protein kinase C beta [Source:HGNC Symbol;Acc:HGNC:9395]	2.37E-32
10	AGPAT1	1-acylglycerol-3-phosphate O-acyltransferase 1 [Source:HGNC Symbo	1.88E-22
10	C12orf75	chromosome 12 open reading frame 75 [Source:HGNC Symbol;Acc:H	3.26E-20
10	HLA-DPB1	major histocompatibility complex, class II, DP beta 1 [Source:HGNC S	5.28E-19
10	TPP1	tripeptidyl peptidase 1 [Source:HGNC Symbol;Acc:HGNC:2073]	5.94E-18
10	PDLIM1	PDZ and LIM domain 1 [Source:HGNC Symbol;Acc:HGNC:2067]	7.91E-17
10	TACC1	transforming acidic coiled-coil containing protein 1 [Source:HGNC Sy	1.32E-15
10	PHKB	phosphorylase kinase regulatory subunit beta [Source:HGNC Symbol	1.34E-15
10	DMTN	dematin actin binding protein [Source:HGNC Symbol;Acc:HGNC:3382	5.02E-14
10	PACSIN2	protein kinase C and casein kinase substrate in neurons 2 [Source:HG	5.08E-14
10	ACRBP	acrosin binding protein [Source:HGNC Symbol;Acc:HGNC:17195]	5.41E-14
10	CDYL	chromodomain Y like [Source:HGNC Symbol;Acc:HGNC:1811]	1.02E-13
10	PPP6R1	protein phosphatase 6 regulatory subunit 1 [Source:HGNC Symbol;A	8.02E-13
10	TPP2	tripeptidyl peptidase 2 [Source:HGNC Symbol;Acc:HGNC:12016]	2.66E-12
10	MFNG	MFNG O-fucosylpeptide 3-beta-N-acetylglucosaminyltransferase [So	4.18E-12
10	CYREN	cell cycle regulator of NHEJ [Source:HGNC Symbol;Acc:HGNC:22432]	5.76E-12
10	KLF10	Kruppel like factor 10 [Source:HGNC Symbol;Acc:HGNC:11810]	3.90E-10
10	CHIC2	cysteine rich hydrophobic domain 2 [Source:HGNC Symbol;Acc:HGN	1.38E-09
10	C16orf54	chromosome 16 open reading frame 54 [Source:HGNC Symbol;Acc:H	2.58E-09
10	LPIN2	lipin 2 [Source:HGNC Symbol;Acc:HGNC:14450]	4.95E-09
10	HERC1	HECT and RLD domain containing E3 ubiquitin protein ligase family m	3.66E-08

10	AKIRIN2	akirin 2 [Source:HGNC Symbol;Acc:HGNC:21407]	4.87E-36
10	QKI	QKI, KH domain containing RNA binding [Source:HGNC Symbol;Acc:H	2.44E-26
10	SNN	stannin [Source:HGNC Symbol;Acc:HGNC:11149]	3.53E-26
10	SDCBP	syndecan binding protein [Source:HGNC Symbol;Acc:HGNC:10662]	1.65E-23
10	CTSC	cathepsin C [Source:HGNC Symbol;Acc:HGNC:2528]	1.63E-22
10	SNCA	synuclein alpha [Source:HGNC Symbol;Acc:HGNC:11138]	9.31E-18
10	KLHL5	kelch like family member 5 [Source:HGNC Symbol;Acc:HGNC:6356]	4.33E-14
10	OTUD5	OTU deubiquitinase 5 [Source:HGNC Symbol;Acc:HGNC:25402]	4.56E-14
10	LBR	lamin B receptor [Source:HGNC Symbol;Acc:HGNC:6518]	4.59E-14
10	SSBP3	single stranded DNA binding protein 3 [Source:HGNC Symbol;Acc:HG	9.20E-14
10	CENPT	centromere protein T [Source:HGNC Symbol;Acc:HGNC:25787]	2.98E-13
10	ITGA4	integrin subunit alpha 4 [Source:HGNC Symbol;Acc:HGNC:6140]	3.36E-13
10	ATP2A3	ATPase sarcoplasmic/endoplasmic reticulum Ca2+ transporting 3 [So	4.12E-13
10	RNF145	ring finger protein 145 [Source:HGNC Symbol;Acc:HGNC:20853]	1.58E-12
10	SNX9	sorting nexin 9 [Source:HGNC Symbol;Acc:HGNC:14973]	3.61E-12
10	AP003068.2	novel transcript, antisense to CAPN1	1.87E-11
10	CCND2	cyclin D2 [Source:HGNC Symbol;Acc:HGNC:1583]	2.62E-09
10	TREML1	triggering receptor expressed on myeloid cells like 1 [Source:HGNC S	4.01E-09
10	NT5M	5',3'-nucleotidase, mitochondrial [Source:HGNC Symbol;Acc:HGNC:1	6.34E-09
10	RBM38	RNA binding motif protein 38 [Source:HGNC Symbol;Acc:HGNC:1581	4.51E-29
10	PPP3R1	protein phosphatase 3 regulatory subunit B, alpha [Source:HGNC Syr	1.74E-20
10	WAS	WASP actin nucleation promoting factor [Source:HGNC Symbol;Acc:H	1.18E-19
10	TBPL1	TATA-box binding protein like 1 [Source:HGNC Symbol;Acc:HGNC:11	2.88E-17
10	TBC1D10C	TBC1 domain family member 10C [Source:HGNC Symbol;Acc:HGNC:2	3.26E-15
10	PRR7	proline rich 7, synaptic [Source:HGNC Symbol;Acc:HGNC:28130]	1.00E-14
10	CTSW	cathepsin W [Source:HGNC Symbol;Acc:HGNC:2546]	1.64E-14
10	HELZ	helicase with zinc finger [Source:HGNC Symbol;Acc:HGNC:16878]	2.02E-14
10	SF3A1	splicing factor 3a subunit 1 [Source:HGNC Symbol;Acc:HGNC:10765]	6.31E-14
10	STON2	stonin 2 [Source:HGNC Symbol;Acc:HGNC:30652]	6.95E-14
10	SPARC	secreted protein acidic and cysteine rich [Source:HGNC Symbol;Acc:H	1.06E-11
10	EMB	embigin [Source:HGNC Symbol;Acc:HGNC:30465]	3.68E-10
10	CFD	complement factor D [Source:HGNC Symbol;Acc:HGNC:2771]	3.63E-08
10	PAN3	poly(A) specific ribonuclease subunit PAN3 [Source:HGNC Symbol;Ac	9.98E-08
10	IVNS1ABP	influenza virus NS1A binding protein [Source:HGNC Symbol;Acc:HGN	2.34E-07
10	IFI30	IFI30 lysosomal thiol reductase [Source:HGNC Symbol;Acc:HGNC:539	2.52E-07
10	FCER1G	Fc fragment of IgE receptor Ig [Source:HGNC Symbol;Acc:HGNC:3611	6.42E-38
10	PSTPIP2	proline-serine-threonine phosphatase interacting protein 2 [Source:H	4.41E-28
10	KCTD20	potassium channel tetramerization domain containing 20 [Source:HG	4.45E-26
10	CTSS	cathepsin S [Source:HGNC Symbol;Acc:HGNC:2545]	5.28E-24
10	MAFG	MAF bZIP transcription factor G [Source:HGNC Symbol;Acc:HGNC:67	7.40E-24
10	LCP2	lymphocyte cytosolic protein 2 [Source:HGNC Symbol;Acc:HGNC:652	1.98E-20
10	ELK3	ETS transcription factor ELK3 [Source:HGNC Symbol;Acc:HGNC:3325]	4.03E-19
10	HLA-DPA1	major histocompatibility complex, class II, DP alpha 1 [Source:HGNC	2.24E-18
10	HIPK1	homeodomain interacting protein kinase 1 [Source:HGNC Symbol;Ac	2.46E-16
10	MAP4K4	mitogen-activated protein kinase kinase kinase kinase 4 [Source:HGN	6.79E-14
10	ADGRE5	adhesion G protein-coupled receptor E5 [Source:HGNC Symbol;Acc:H	6.09E-13
10	LGALSL	galectin like [Source:HGNC Symbol;Acc:HGNC:25012]	7.64E-13

10	TENT5C	terminal nucleotidyltransferase 5C [Source:HGNC Symbol;Acc:HGNC:	3.18E-12
10	C2orf88	chromosome 2 open reading frame 88 [Source:HGNC Symbol;Acc:HG	5.69E-12
10	RCSD1	RCSD domain containing 1 [Source:HGNC Symbol;Acc:HGNC:28310]	2.15E-11
10	ADIPOR2	adiponectin receptor 2 [Source:HGNC Symbol;Acc:HGNC:24041]	1.72E-10
10	FMNL1	formin like 1 [Source:HGNC Symbol;Acc:HGNC:1212]	3.68E-10
10	ARHGAP4	Rho GTPase activating protein 4 [Source:HGNC Symbol;Acc:HGNC:67	4.08E-09
10	SATB1	SATB homeobox 1 [Source:HGNC Symbol;Acc:HGNC:10541]	8.54E-08
10	CHCHD7	coiled-coil-helix-coiled-coil-helix domain containing 7 [Source:HGNC	1.53E-07
10	PLEK	pleckstrin [Source:HGNC Symbol;Acc:HGNC:9070]	1.06E-42
10	MOB1B	MOB kinase activator 1B [Source:HGNC Symbol;Acc:HGNC:29801]	3.54E-26
10	TACC3	transforming acidic coiled-coil containing protein 3 [Source:HGNC Sy	6.57E-26
10	UBXN11	UBX domain protein 11 [Source:HGNC Symbol;Acc:HGNC:30600]	7.61E-26
10	CARD8	caspase recruitment domain family member 8 [Source:HGNC Symbo	9.52E-21
10	AC020916.1	novel transcript, antisense to ZSWIM4	1.06E-18
10	AMFR	autocrine motility factor receptor [Source:HGNC Symbol;Acc:HGNC:4	1.53E-17
10	PLA2G12A	phospholipase A2 group XIIA [Source:HGNC Symbol;Acc:HGNC:1855	1.79E-17
10	AC000093.1	novel transcript	5.28E-17
10	SLCO3A1	solute carrier organic anion transporter family member 3A1 [Source:	1.85E-16
10	RPA1	replication protein A1 [Source:HGNC Symbol;Acc:HGNC:10289]	1.04E-15
10	DPYD	dihydropyrimidine dehydrogenase [Source:HGNC Symbol;Acc:HGNC:	2.83E-15
10	AC245297.3	novel transcript	1.70E-13
10	MPIG6B	megakaryocyte and platelet inhibitory receptor G6b [Source:HGNC S	6.68E-12
10	GMPR	guanosine monophosphate reductase [Source:HGNC Symbol;Acc:HG	1.95E-11
10	GLIPR1	GLI pathogenesis related 1 [Source:HGNC Symbol;Acc:HGNC:17001]	9.19E-11
10	AIF1	allograft inflammatory factor 1 [Source:HGNC Symbol;Acc:HGNC:352	1.08E-10
10	C16orf87	chromosome 16 open reading frame 87 [Source:HGNC Symbol;Acc:H	1.25E-10
10	AC147651.1	novel transcript	1.29E-10
10	AP001189.1	novel transcript	5.89E-10
10	CLEC1B	C-type lectin domain family 1 member B [Source:HGNC Symbol;Acc:H	6.64E-10
10	MSL3	MSL complex subunit 3 [Source:HGNC Symbol;Acc:HGNC:7370]	3.22E-08
10	ARRDC3	arrestin domain containing 3 [Source:HGNC Symbol;Acc:HGNC:2926	4.73E-08
10	PYGL	glycogen phosphorylase L [Source:HGNC Symbol;Acc:HGNC:9725]	3.31E-26
10	APOBEC3C	apolipoprotein B mRNA editing enzyme catalytic subunit 3C [Source:	2.68E-22
10	MXD1	MAX dimerization protein 1 [Source:HGNC Symbol;Acc:HGNC:6761]	1.64E-20
10	ABCC3	ATP binding cassette subfamily C member 3 [Source:HGNC Symbol;A	7.66E-19
10	YPEL2	yippee like 2 [Source:HGNC Symbol;Acc:HGNC:18326]	1.39E-17
10	RASA3	RAS p21 protein activator 3 [Source:HGNC Symbol;Acc:HGNC:20331]	6.22E-17
10	NFAT5	nuclear factor of activated T cells 5 [Source:HGNC Symbol;Acc:HGNC	4.55E-14
10	C1orf162	chromosome 1 open reading frame 162 [Source:HGNC Symbol;Acc:H	1.17E-13
10	ITGA2B	integrin subunit alpha 2b [Source:HGNC Symbol;Acc:HGNC:6138]	3.11E-13
10	MAP4K5	mitogen-activated protein kinase kinase kinase kinase 5 [Source:HGN	3.27E-13
10	LST1	leukocyte specific transcript 1 [Source:HGNC Symbol;Acc:HGNC:1418	1.61E-12
10	TET2	tet methylcytosine dioxygenase 2 [Source:HGNC Symbol;Acc:HGNC:2	1.04E-11
10	GPR183	G protein-coupled receptor 183 [Source:HGNC Symbol;Acc:HGNC:31	1.59E-08
10	CMTM3	CKLF like MARVEL transmembrane domain containing 3 [Source:HGN	7.78E-08
10	ST3GAL1	ST3 beta-galactoside alpha-2,3-sialyltransferase 1 [Source:HGNC Sym	1.34E-07
10	TAGAP	T cell activation RhoGTPase activating protein [Source:HGNC Symbol	1.45E-07

10	GABARAPL1	GABA type A receptor associated protein like 1 [Source:HGNC Symbo	1.63E-07
10	LINC00623	long intergenic non-protein coding RNA 623 [Source:HGNC Symbol;A	2.90E-07
10	TPST2	tyrosylprotein sulfotransferase 2 [Source:HGNC Symbol;Acc:HGNC:1	2.26E-38
10	CD226	CD226 molecule [Source:HGNC Symbol;Acc:HGNC:16961]	2.27E-28
10	GNA13	G protein subunit alpha 13 [Source:HGNC Symbol;Acc:HGNC:4381]	4.12E-20
10	MMD	monocyte to macrophage differentiation associated [Source:HGNC S	3.12E-19
10	CBL	Cbl proto-oncogene [Source:HGNC Symbol;Acc:HGNC:1541]	1.64E-17
10	MAP4K2	mitogen-activated protein kinase kinase kinase kinase 2 [Source:HGN	1.05E-16
10	TSPOAP1-AS1	TSPOAP1, SUPT4H1 and RNF43 antisense RNA 1 [Source:HGNC Symb	1.06E-16
10	SLA2	Src like adaptor 2 [Source:HGNC Symbol;Acc:HGNC:17329]	2.72E-16
10	FHL1	four and a half LIM domains 1 [Source:HGNC Symbol;Acc:HGNC:370	3.24E-13
10	SELP	selectin P [Source:HGNC Symbol;Acc:HGNC:10721]	5.19E-11
10	MAP3K7CL	MAP3K7 C-terminal like [Source:HGNC Symbol;Acc:HGNC:16457]	1.94E-10
10	ARHGEF3	Rho guanine nucleotide exchange factor 3 [Source:HGNC Symbol;Acc	1.47E-09
10	SLA	Src like adaptor [Source:HGNC Symbol;Acc:HGNC:10902]	3.07E-09
10	GZMA	granzyme A [Source:HGNC Symbol;Acc:HGNC:4708]	3.72E-08
10	RASSF5	Ras association domain family member 5 [Source:HGNC Symbol;Acc:	3.84E-08
10	PPP1R18	protein phosphatase 1 regulatory subunit 18 [Source:HGNC Symbol;A	1.84E-32
10	ARRB1	arrestin beta 1 [Source:HGNC Symbol;Acc:HGNC:711]	2.59E-27
10	GADD45A	growth arrest and DNA damage inducible alpha [Source:HGNC Symbo	1.17E-25
10	LYST	lysosomal trafficking regulator [Source:HGNC Symbol;Acc:HGNC:196	2.45E-24
10	BICD2	BICD cargo adaptor 2 [Source:HGNC Symbol;Acc:HGNC:17208]	3.18E-21
10	SSX2IP	SSX family member 2 interacting protein [Source:HGNC Symbol;Acc:H	1.69E-18
10	TGFBR2	transforming growth factor beta receptor 2 [Source:HGNC Symbol;Ac	9.26E-13
10	FOXN3	forkhead box N3 [Source:HGNC Symbol;Acc:HGNC:1928]	9.46E-13
10	JAK3	Janus kinase 3 [Source:HGNC Symbol;Acc:HGNC:6193]	6.59E-10
10	PLEKHA2	pleckstrin homology domain containing A2 [Source:HGNC Symbol;Ac	2.68E-08
10	STK10	serine/threonine kinase 10 [Source:HGNC Symbol;Acc:HGNC:11388]	3.15E-08
10	CD68	CD68 molecule [Source:HGNC Symbol;Acc:HGNC:1693]	3.96E-58
10	ATP6V1B2	ATPase H+ transporting V1 subunit B2 [Source:HGNC Symbol;Acc:HG	4.37E-30
10	FLI1	Fli-1 proto-oncogene, ETS transcription factor [Source:HGNC Symbol	1.18E-27
10	DAB2	DAB adaptor protein 2 [Source:HGNC Symbol;Acc:HGNC:2662]	7.56E-27
10	STX7	syntaxin 7 [Source:HGNC Symbol;Acc:HGNC:11442]	1.91E-26
10	VIM-AS1	VIM antisense RNA 1 [Source:HGNC Symbol;Acc:HGNC:44879]	2.68E-25
10	GTPBP2	GTP binding protein 2 [Source:HGNC Symbol;Acc:HGNC:4670]	3.94E-24
10	PRKAR1B	protein kinase cAMP-dependent type I regulatory subunit beta [Sour	3.24E-22
10	CCDC71L	coiled-coil domain containing 71 like [Source:HGNC Symbol;Acc:HGN	3.42E-20
10	BACH1	BTB domain and CNC homolog 1 [Source:HGNC Symbol;Acc:HGNC:93	4.44E-20
10	ZNF438	zinc finger protein 438 [Source:HGNC Symbol;Acc:HGNC:21029]	1.05E-18
10	DYRK1A	dual specificity tyrosine phosphorylation regulated kinase 1A [Source	4.61E-18
10	RPS6KA3	ribosomal protein S6 kinase A3 [Source:HGNC Symbol;Acc:HGNC:104	7.05E-18
10	LY6G6F	lymphocyte antigen 6 family member G6F [Source:HGNC Symbol;Acc	8.18E-18
10	SCN1B	sodium voltage-gated channel beta subunit 1 [Source:HGNC Symbol;	1.30E-16
10	MAP3K5	mitogen-activated protein kinase kinase kinase 5 [Source:HGNC Sym	2.84E-15
10	TNIK	TRAF2 and NCK interacting kinase [Source:HGNC Symbol;Acc:HGNC:3	1.57E-13
10	SERPINB9	serpin family B member 9 [Source:HGNC Symbol;Acc:HGNC:8955]	5.02E-13
10	CLDN5	claudin 5 [Source:HGNC Symbol;Acc:HGNC:2047]	6.16E-13

10	ESAM	endothelial cell adhesion molecule [Source:HGNC Symbol;Acc:HGNC	2.75E-12
10	AC090409.1	novel transcript	4.22E-12
10	HLA-DMA	major histocompatibility complex, class II, DM alpha [Source:HGNC S	4.47E-12
10	GP1BA	glycoprotein Ib platelet subunit alpha [Source:HGNC Symbol;Acc:HGI	7.10E-12
10	ATP2B1-AS1	ATP2B1 antisense RNA 1 [Source:HGNC Symbol;Acc:HGNC:27883]	9.12E-11
10	HSD17B11	hydroxysteroid 17-beta dehydrogenase 11 [Source:HGNC Symbol;Ac	1.15E-08
10	GNLY	granulysin [Source:HGNC Symbol;Acc:HGNC:4414]	1.98E-08
10	PRKX	protein kinase X-linked [Source:HGNC Symbol;Acc:HGNC:9441]	1.71E-07
10	LCN2	lipocalin 2 [Source:HGNC Symbol;Acc:HGNC:6526]	3.80E-07
10	NCF1	neutrophil cytosolic factor 1 [Source:HGNC Symbol;Acc:HGNC:7660]	5.73E-07
10	PTPRJ	protein tyrosine phosphatase receptor type J [Source:HGNC Symbol;	1.25E-33
10	PTGS1	prostaglandin-endoperoxide synthase 1 [Source:HGNC Symbol;Acc:H	6.62E-26
10	FURIN	furin, paired basic amino acid cleaving enzyme [Source:HGNC Symbo	1.77E-24
10	HHEX	hematopoietically expressed homeobox [Source:HGNC Symbol;Acc:H	1.11E-22
10	FNTB	farnesyltransferase, CAAX box, beta [Source:HGNC Symbol;Acc:HGN	1.56E-21
10	CYB5R4	cytochrome b5 reductase 4 [Source:HGNC Symbol;Acc:HGNC:20147]	1.16E-19
10	KLHL6	kelch like family member 6 [Source:HGNC Symbol;Acc:HGNC:18653]	7.16E-19
10	PDE4D	phosphodiesterase 4D [Source:HGNC Symbol;Acc:HGNC:8783]	1.43E-17
10	TRIM58	tripartite motif containing 58 [Source:HGNC Symbol;Acc:HGNC:2415	1.42E-16
10	SH3BGRL2	SH3 domain binding glutamate rich protein like 2 [Source:HGNC Sym	5.46E-16
10	MYO1G	myosin IG [Source:HGNC Symbol;Acc:HGNC:13880]	1.69E-11
10	GNB5	G protein subunit beta 5 [Source:HGNC Symbol;Acc:HGNC:4401]	5.40E-11
10	SESN3	sestrin 3 [Source:HGNC Symbol;Acc:HGNC:23060]	1.98E-08
10	SAMSN1	SAM domain, SH3 domain and nuclear localization signals 1 [Source:	3.82E-08
10	SNRK	SNF related kinase [Source:HGNC Symbol;Acc:HGNC:30598]	4.11E-08
10	FCGRT	Fc fragment of IgG receptor and transporter [Source:HGNC Symbol;A	1.60E-07
10	CORO1C	coronin 1C [Source:HGNC Symbol;Acc:HGNC:2254]	5.87E-53
10	CD36	CD36 molecule [Source:HGNC Symbol;Acc:HGNC:1663]	4.01E-48
10	PECAM1	platelet and endothelial cell adhesion molecule 1 [Source:HGNC Sym	4.95E-42
10	FRMD4B	FERM domain containing 4B [Source:HGNC Symbol;Acc:HGNC:24886	4.88E-35
10	INSIG1	insulin induced gene 1 [Source:HGNC Symbol;Acc:HGNC:6083]	9.80E-34
10	USP12	ubiquitin specific peptidase 12 [Source:HGNC Symbol;Acc:HGNC:204	1.85E-27
10	RAB37	RAB37, member RAS oncogene family [Source:HGNC Symbol;Acc:HG	4.77E-24
10	HLA-DRA	major histocompatibility complex, class II, DR alpha [Source:HGNC Sy	9.55E-19
10	ARHGAP6	Rho GTPase activating protein 6 [Source:HGNC Symbol;Acc:HGNC:67	1.34E-16
10	PDGFA	platelet derived growth factor subunit A [Source:HGNC Symbol;Acc:H	1.96E-13
10	PDE5A	phosphodiesterase 5A [Source:HGNC Symbol;Acc:HGNC:8784]	2.17E-13
10	ENKUR	enkurin, TRPC channel interacting protein [Source:HGNC Symbol;Acc	6.68E-13
10	TMEM158	transmembrane protein 158 [Source:HGNC Symbol;Acc:HGNC:30293	8.31E-12
10	NKG7	natural killer cell granule protein 7 [Source:HGNC Symbol;Acc:HGNC:	2.99E-11
10	OSBP2	oxysterol binding protein 2 [Source:HGNC Symbol;Acc:HGNC:8504]	3.97E-10
10	CST7	cystatin F [Source:HGNC Symbol;Acc:HGNC:2479]	6.18E-10
10	THEMIS2	thymocyte selection associated family member 2 [Source:HGNC Sym	8.72E-09
10	PLXDC2	plexin domain containing 2 [Source:HGNC Symbol;Acc:HGNC:21013]	1.13E-39
10	PTGIR	prostaglandin I2 receptor [Source:HGNC Symbol;Acc:HGNC:9602]	5.28E-32
10	INAFM2	InaF motif containing 2 [Source:HGNC Symbol;Acc:HGNC:35165]	6.28E-32
10	FCGR2A	Fc fragment of IgG receptor IIa [Source:HGNC Symbol;Acc:HGNC:361	5.89E-28

10	SUSD1	sushi domain containing 1 [Source:HGNC Symbol;Acc:HGNC:25413]	1.37E-24
10	E2F3	E2F transcription factor 3 [Source:HGNC Symbol;Acc:HGNC:3115]	5.65E-20
10	PF4V1	platelet factor 4 variant 1 [Source:HGNC Symbol;Acc:HGNC:8862]	1.40E-18
10	TMEM140	transmembrane protein 140 [Source:HGNC Symbol;Acc:HGNC:2187(	1.73E-18
10	MAN1A1	mannosidase alpha class 1A member 1 [Source:HGNC Symbol;Acc:H(	1.62E-16
10	DOCK2	dedicator of cytokinesis 2 [Source:HGNC Symbol;Acc:HGNC:2988]	8.03E-14
10	GLIPR2	GLI pathogenesis related 2 [Source:HGNC Symbol;Acc:HGNC:18007]	7.02E-12
10	F2R	coagulation factor II thrombin receptor [Source:HGNC Symbol;Acc:H	3.37E-10
10	CALHM6	calcium homeostasis modulator family member 6 [Source:HGNC Sym	1.50E-09
10	CCDC69	coiled-coil domain containing 69 [Source:HGNC Symbol;Acc:HGNC:2	9.18E-09
10	LYL1	LYL1 basic helix-loop-helix family member [Source:HGNC Symbol;Acc	6.29E-51
10	SKAP2	src kinase associated phosphoprotein 2 [Source:HGNC Symbol;Acc:H	1.84E-39
10	ZEB2	zinc finger E-box binding homeobox 2 [Source:HGNC Symbol;Acc:HG	2.15E-39
10	P2RX1	purinergic receptor P2X 1 [Source:HGNC Symbol;Acc:HGNC:8533]	2.65E-36
10	ANKRD28	ankyrin repeat domain 28 [Source:HGNC Symbol;Acc:HGNC:29024]	3.58E-28
10	IRS2	insulin receptor substrate 2 [Source:HGNC Symbol;Acc:HGNC:6126]	8.53E-23
10	CDIP1	cell death inducing p53 target 1 [Source:HGNC Symbol;Acc:HGNC:13	2.81E-21
10	TAL1	TAL bHLH transcription factor 1, erythroid differentiation factor [Sou	1.04E-19
10	ITGB3	integrin subunit beta 3 [Source:HGNC Symbol;Acc:HGNC:6156]	1.88E-19
10	TSPAN18	tetraspanin 18 [Source:HGNC Symbol;Acc:HGNC:20660]	1.89E-16
10	HEMGN	hemogen [Source:HGNC Symbol;Acc:HGNC:17509]	1.28E-15
10	TSPAN32	tetraspanin 32 [Source:HGNC Symbol;Acc:HGNC:13410]	1.96E-15
10	AGTPBP1	ATP/GTP binding protein 1 [Source:HGNC Symbol;Acc:HGNC:17258]	2.41E-11
10	PTPN7	protein tyrosine phosphatase non-receptor type 7 [Source:HGNC Syr	4.98E-08
10	STX11	syntaxin 11 [Source:HGNC Symbol;Acc:HGNC:11429]	9.16E-53
10	RNF24	ring finger protein 24 [Source:HGNC Symbol;Acc:HGNC:13779]	4.12E-35
10	NEXN	nexilin F-actin binding protein [Source:HGNC Symbol;Acc:HGNC:295	1.94E-29
10	SMOX	spermine oxidase [Source:HGNC Symbol;Acc:HGNC:15862]	9.15E-21
10	PADI4	peptidyl arginine deiminase 4 [Source:HGNC Symbol;Acc:HGNC:1836	9.85E-18
10	CPNE5	copine 5 [Source:HGNC Symbol;Acc:HGNC:2318]	1.69E-17
10	AL731557.1	novel transcript	1.16E-16
10	MYO1F	myosin IF [Source:HGNC Symbol;Acc:HGNC:7600]	2.13E-15
10	AQP10	aquaporin 10 [Source:HGNC Symbol;Acc:HGNC:16029]	6.85E-13
10	BEND2	BEN domain containing 2 [Source:HGNC Symbol;Acc:HGNC:28509]	2.67E-12
10	IPCEF1	interaction protein for cytohesin exchange factors 1 [Source:HGNC S	6.09E-10
10	FGL2	fibrinogen like 2 [Source:HGNC Symbol;Acc:HGNC:3696]	9.96E-10
10	AC004687.1	novel transcript, MIR142 host	2.87E-09
10	KCNAB2	potassium voltage-gated channel subfamily A regulatory beta subuni	7.41E-09
10	RPS6KA1	ribosomal protein S6 kinase A1 [Source:HGNC Symbol;Acc:HGNC:104	8.27E-09
10	HOPX	HOP homeobox [Source:HGNC Symbol;Acc:HGNC:24961]	2.75E-08
10	BANK1	B cell scaffold protein with ankyrin repeats 1 [Source:HGNC Symbol;/	7.42E-08
10	CEP85L	centrosomal protein 85 like [Source:HGNC Symbol;Acc:HGNC:21638]	5.22E-07
10	RAB31	RAB31, member RAS oncogene family [Source:HGNC Symbol;Acc:HG	1.71E-45
10	TNFSF13B	TNF superfamily member 13b [Source:HGNC Symbol;Acc:HGNC:1192	3.80E-42
10	KIAA0513	KIAA0513 [Source:HGNC Symbol;Acc:HGNC:29058]	4.35E-34
10	NFE2	nuclear factor, erythroid 2 [Source:HGNC Symbol;Acc:HGNC:7780]	1.71E-33
10	DSE	dermatan sulfate epimerase [Source:HGNC Symbol;Acc:HGNC:21144	1.93E-33

10	TBXA2R	thromboxane A2 receptor [Source:HGNC Symbol;Acc:HGNC:11608]	4.14E-25
10	TYROBP	transmembrane immune signaling adaptor TYROBP [Source:HGNC Sy	8.71E-22
10	GUCY1B1	guanylate cyclase 1 soluble subunit beta 1 [Source:HGNC Symbol;Acc	1.28E-21
10	MYLK	myosin light chain kinase [Source:HGNC Symbol;Acc:HGNC:7590]	4.39E-21
10	CXCL5	C-X-C motif chemokine ligand 5 [Source:HGNC Symbol;Acc:HGNC:10	5.05E-19
10	DOCK11	dedicator of cytokinesis 11 [Source:HGNC Symbol;Acc:HGNC:23483]	5.43E-15
10	POU2F2	POU class 2 homeobox 2 [Source:HGNC Symbol;Acc:HGNC:9213]	2.44E-13
10	SNX10	sorting nexin 10 [Source:HGNC Symbol;Acc:HGNC:14974]	8.18E-10
10	GZMB	granzyme B [Source:HGNC Symbol;Acc:HGNC:4709]	3.69E-07
10	CD300A	CD300a molecule [Source:HGNC Symbol;Acc:HGNC:19319]	5.45E-07
10	MEF2C	myocyte enhancer factor 2C [Source:HGNC Symbol;Acc:HGNC:6996]	2.44E-44
10	LYN	LYN proto-oncogene, Src family tyrosine kinase [Source:HGNC Symbo	2.09E-41
10	GFI1B	growth factor independent 1B transcriptional repressor [Source:HGN	2.34E-22
10	SLC6A6	solute carrier family 6 member 6 [Source:HGNC Symbol;Acc:HGNC:1	4.59E-22
10	FRMD3	FERM domain containing 3 [Source:HGNC Symbol;Acc:HGNC:24125]	7.33E-16
10	C11orf21	chromosome 11 open reading frame 21 [Source:HGNC Symbol;Acc:H	1.27E-14
10	LYZ	lysozyme [Source:HGNC Symbol;Acc:HGNC:6740]	1.09E-13
10	PRF1	perforin 1 [Source:HGNC Symbol;Acc:HGNC:9360]	1.45E-10
10	CCL4	C-C motif chemokine ligand 4 [Source:HGNC Symbol;Acc:HGNC:1063	2.17E-10
10	MNDA	myeloid cell nuclear differentiation antigen [Source:HGNC Symbol;Ac	2.56E-09
10	KLRD1	killer cell lectin like receptor D1 [Source:HGNC Symbol;Acc:HGNC:63	3.09E-09
10	PHACTR1	phosphatase and actin regulator 1 [Source:HGNC Symbol;Acc:HGNC:	2.46E-08
10	HLA-DMB	major histocompatibility complex, class II, DM beta [Source:HGNC Sy	2.74E-08
10	BTK	Bruton tyrosine kinase [Source:HGNC Symbol;Acc:HGNC:1133]	1.17E-63
10	TBXAS1	thromboxane A synthase 1 [Source:HGNC Symbol;Acc:HGNC:11609]	3.40E-50
10	TNFSF4	TNF superfamily member 4 [Source:HGNC Symbol;Acc:HGNC:11934]	7.28E-30
10	HOTAIRM1	HOXA transcript antisense RNA, myeloid-specific 1 [Source:HGNC Sy	1.74E-25
10	ITGAM	integrin subunit alpha M [Source:HGNC Symbol;Acc:HGNC:6149]	8.92E-24
10	ITGAX	integrin subunit alpha X [Source:HGNC Symbol;Acc:HGNC:6152]	7.80E-12
10	CFP	complement factor properdin [Source:HGNC Symbol;Acc:HGNC:8864	9.39E-11
10	FGFBP2	fibroblast growth factor binding protein 2 [Source:HGNC Symbol;Acc	2.11E-09
10	CCDC88A	coiled-coil domain containing 88A [Source:HGNC Symbol;Acc:HGNC:	5.65E-19
10	CYBB	cytochrome b-245 beta chain [Source:HGNC Symbol;Acc:HGNC:2578	8.28E-18
10	FCN1	ficolin 1 [Source:HGNC Symbol;Acc:HGNC:3623]	8.48E-16
10	SPI1	Spi-1 proto-oncogene [Source:HGNC Symbol;Acc:HGNC:11241]	2.31E-14
10	HLA-DQA1	major histocompatibility complex, class II, DQ alpha 1 [Source:HGNC	3.10E-13
10	FCGR3A	Fc fragment of IgG receptor IIIa [Source:HGNC Symbol;Acc:HGNC:36	2.63E-12
10	GZMH	granzyme H [Source:HGNC Symbol;Acc:HGNC:4710]	3.07E-12
10	FGR	FGR proto-oncogene, Src family tyrosine kinase [Source:HGNC Symb	4.23E-12
10	LAT2	linker for activation of T cells family member 2 [Source:HGNC Symbo	6.63E-12
10	SPON2	spondin 2 [Source:HGNC Symbol;Acc:HGNC:11253]	7.58E-10
10	AOAH	acyloxyacyl hydrolase [Source:HGNC Symbol;Acc:HGNC:548]	1.71E-09
10	LY86	lymphocyte antigen 86 [Source:HGNC Symbol;Acc:HGNC:16837]	6.41E-09
10	KLRF1	killer cell lectin like receptor F1 [Source:HGNC Symbol;Acc:HGNC:13	8.93E-09
10	C1orf21	chromosome 1 open reading frame 21 [Source:HGNC Symbol;Acc:HG	3.54E-08
10	CX3CR1	C-X3-C motif chemokine receptor 1 [Source:HGNC Symbol;Acc:HGNC	9.91E-08

avg_logFC	pct.1	pct.2	p_val_adj	mean in cluster	mean out of cluster	DiffMean	dDR<br>Numbers in blue are expressed in a higher percentage of cells in cluster 10 than in all other clusters combined
-0.8474	1.000	0.998	6.51E-154	3.797	4.797	1.000	0.002
0.2933	0.995	0.965	1.07E-14	3.799	3.426	0.373	0.030
0.5418	0.985	0.872	5.12E-65	4.507	3.463	1.044	0.113
0.7281	0.980	0.890	1.07E-27	3.168	2.195	0.973	0.090
0.7018	0.970	0.874	1.84E-28	2.919	2.096	0.823	0.096
0.4014	0.970	0.936	2.71E-12	3.174	2.573	0.601	0.034
1.6258	0.960	0.669	1.28E-102	3.646	1.611	2.035	0.291
-0.7817	0.960	0.973	4.00E-41	3.200	3.864	0.664	-0.013
-0.5083	0.955	0.977	6.93E-30	3.014	3.545	0.531	-0.022
0.2842	0.950	0.904	5.13E-12	2.807	2.407	0.400	0.046
0.3535	0.950	0.917	1.06E-03	3.053	2.530	0.523	0.033
-0.6156	0.935	0.766	4.92E-50	2.900	3.132	0.232	0.169
-0.3029	0.935	0.756	1.63E-19	3.176	2.955	0.221	0.179
0.7555	0.930	0.812	3.14E-42	2.553	1.720	0.833	0.118
-0.6711	0.925	0.899	2.69E-36	2.794	3.474	0.680	0.026
-0.5182	0.920	0.760	7.67E-35	3.063	3.215	0.153	0.160
-0.2552	0.915	0.756	1.09E-13	2.934	2.768	0.165	0.159
-0.3581	0.915	0.866	3.33E-04	2.724	2.964	0.239	0.049
-1.0572	0.910	0.990	1.60E-113	3.027	4.518	1.490	-0.080
0.8998	0.910	0.712	3.67E-39	3.191	1.711	1.479	0.198
-0.3236	0.910	0.731	3.81E-26	3.097	2.945	0.152	0.179
-0.5407	0.905	0.959	7.67E-25	2.454	3.087	0.633	-0.054
0.6863	0.905	0.660	2.27E-21	3.166	1.900	1.267	0.245
1.5990	0.900	0.483	1.66E-91	2.880	0.896	1.984	0.417
-0.5557	0.900	0.955	1.71E-28	2.240	2.842	0.601	-0.055
-0.4119	0.900	0.755	1.86E-20	2.878	2.886	0.008	0.145
-0.3079	0.900	0.751	6.49E-17	2.748	2.699	0.049	0.149
-0.5226	0.900	0.826	6.65E-10	2.752	3.057	0.305	0.074
-0.3265	0.896	0.740	1.21E-31	2.566	2.533	0.033	0.156
-0.4283	0.896	0.742	6.72E-29	2.593	2.697	0.104	0.154
-0.4213	0.896	0.944	2.92E-11	2.305	2.721	0.416	-0.048
1.3493	0.881	0.575	1.99E-52	2.696	1.122	1.574	0.306
-0.5004	0.876	0.773	2.83E-27	2.714	2.933	0.218	0.103
0.9296	0.871	0.650	5.72E-32	2.282	1.252	1.030	0.221
-0.3547	0.871	0.724	7.36E-23	2.468	2.472	0.004	0.147
-0.6254	0.861	0.938	2.39E-21	2.090	2.605	0.515	-0.077
-0.3515	0.861	0.753	7.32E-20	2.444	2.578	0.133	0.108
-0.4783	0.861	0.759	7.01E-16	2.634	2.919	0.285	0.102
-0.3383	0.861	0.916	2.10E-07	2.180	2.499	0.319	-0.055
-0.6718	0.856	0.766	4.82E-58	2.447	3.016	0.569	0.090
0.6022	0.856	0.780	1.57E-21	2.053	1.465	0.589	0.076

0.4674	0.851	0.709	4.71E-21	2.131	1.559	0.572	0.142
0.4596	0.846	0.750	3.06E-08	1.860	1.331	0.530	0.096
1.0632	0.841	0.642	4.36E-69	1.985	0.967	1.018	0.199
1.3444	0.836	0.474	1.31E-52	2.140	0.757	1.383	0.362
0.7109	0.836	0.680	4.44E-29	1.795	1.084	0.711	0.156
0.4268	0.836	0.706	8.44E-12	2.114	1.553	0.561	0.130
-0.6198	0.831	0.885	1.22E-07	1.874	2.106	0.231	-0.054
0.5489	0.831	0.676	1.95E-07	2.274	1.565	0.709	0.155
-0.5093	0.826	0.742	4.55E-40	2.303	2.642	0.338	0.084
-0.9029	0.826	0.926	1.27E-32	1.922	2.544	0.622	-0.100
0.6659	0.826	0.669	3.62E-18	1.970	1.265	0.705	0.157
0.2665	0.826	0.784	9.78E-03	1.868	1.596	0.271	0.042
-1.2032	0.821	0.961	4.52E-60	1.960	3.106	1.146	-0.140
0.8183	0.821	0.681	1.50E-42	1.872	1.089	0.783	0.140
1.0220	0.816	0.581	2.63E-61	1.828	0.833	0.994	0.235
0.3782	0.811	0.649	5.92E-12	1.897	1.365	0.531	0.162
1.1523	0.806	0.489	1.17E-69	2.059	0.832	1.227	0.317
-0.3577	0.806	0.697	4.52E-15	2.264	2.404	0.140	0.109
-0.2914	0.806	0.710	2.05E-13	2.083	2.197	0.114	0.096
0.5894	0.806	0.644	2.95E-13	1.823	1.192	0.632	0.162
-0.2762	0.806	0.708	3.44E-08	2.165	2.255	0.090	0.098
-0.4048	0.806	0.740	1.58E-04	2.320	2.524	0.204	0.066
2.2487	0.801	0.455	6.37E-129	2.376	0.643	1.734	0.346
1.1529	0.801	0.493	4.83E-97	1.929	0.718	1.211	0.308
-0.5900	0.801	0.735	6.52E-22	2.366	2.824	0.458	0.066
0.3928	0.801	0.612	6.91E-07	1.876	1.225	0.651	0.189
3.5015	0.786	0.032	4.43E-220	2.914	0.042	2.872	0.754
0.9185	0.786	0.609	2.13E-48	1.736	0.888	0.847	0.177
-0.3970	0.786	0.737	1.51E-07	2.092	2.375	0.283	0.049
0.9760	0.781	0.493	2.39E-52	1.547	0.643	0.904	0.288
0.9059	0.776	0.627	4.67E-45	1.785	0.950	0.836	0.149
0.7839	0.771	0.550	1.10E-30	1.507	0.759	0.748	0.221
0.8515	0.766	0.571	1.88E-31	1.589	0.802	0.787	0.195
0.4655	0.766	0.669	2.69E-15	1.580	1.041	0.539	0.097
0.2891	0.766	0.614	3.27E-09	1.823	1.182	0.642	0.152
5.9634	0.761	0.030	2.58E-259	5.243	0.054	5.190	0.731
4.2435	0.761	0.005	9.42E-226	3.458	0.012	3.446	0.756
1.7008	0.761	0.311	3.65E-89	1.966	0.455	1.511	0.450
-0.7485	0.756	0.740	1.34E-60	2.129	3.032	0.903	0.016
0.4469	0.756	0.624	4.93E-17	1.559	1.074	0.485	0.132
1.7186	0.751	0.305	1.25E-120	1.774	0.367	1.407	0.446
0.9543	0.751	0.507	6.17E-35	1.524	0.672	0.853	0.244
-0.4481	0.751	0.897	1.16E-16	1.599	2.163	0.564	-0.146
2.8861	0.746	0.019	2.75E-189	2.291	0.031	2.260	0.727
1.1687	0.746	0.447	4.57E-58	1.564	0.563	1.000	0.299
0.6642	0.746	0.657	1.18E-28	1.645	1.074	0.571	0.089
3.2811	0.741	0.004	1.98E-215	2.577	0.008	2.569	0.737

0.8571	0.736	0.607	6.41E-50	1.599	0.873	0.726	0.129
-0.7915	0.736	0.936	3.05E-38	1.623	2.504	0.881	-0.200
0.8697	0.736	0.511	2.00E-33	1.439	0.681	0.758	0.225
-0.5232	0.736	0.706	4.68E-29	1.765	2.213	0.449	0.030
2.1036	0.731	0.183	7.19E-108	1.986	0.246	1.739	0.548
1.0878	0.731	0.304	4.83E-74	1.651	0.462	1.188	0.427
1.3279	0.731	0.380	7.79E-68	1.558	0.449	1.109	0.351
1.1369	0.731	0.514	3.69E-35	1.606	0.741	0.864	0.217
0.3946	0.731	0.703	9.31E-08	1.437	1.114	0.322	0.028
0.8364	0.726	0.528	8.10E-36	1.410	0.693	0.717	0.198
-0.6515	0.726	0.724	4.48E-26	1.776	2.392	0.616	0.002
0.4920	0.726	0.667	1.35E-10	1.430	1.016	0.414	0.059
-0.6662	0.721	0.771	5.16E-20	1.899	2.717	0.818	-0.050
0.8725	0.716	0.518	2.49E-28	1.444	0.700	0.744	0.198
0.6046	0.716	0.599	3.40E-25	1.380	0.856	0.524	0.117
1.2457	0.711	0.354	2.42E-94	1.434	0.403	1.031	0.357
1.2151	0.711	0.434	5.34E-69	1.514	0.532	0.982	0.277
0.9267	0.711	0.510	2.84E-57	1.408	0.638	0.771	0.201
0.9245	0.711	0.538	7.95E-55	1.478	0.706	0.773	0.173
1.2433	0.706	0.396	1.45E-85	1.491	0.471	1.020	0.310
0.6243	0.706	0.605	2.83E-22	1.378	0.849	0.529	0.101
0.5196	0.706	0.586	3.74E-09	1.344	0.880	0.464	0.120
0.5339	0.706	0.563	3.17E-07	1.326	0.821	0.505	0.143
0.2639	0.706	0.752	2.45E-04	1.510	1.298	0.212	-0.046
2.8452	0.701	0.001	3.73E-194	2.111	0.003	2.107	0.700
2.8430	0.701	0.030	3.01E-172	2.147	0.035	2.113	0.671
2.7803	0.701	0.036	6.75E-152	2.044	0.038	2.006	0.665
-0.5744	0.701	0.887	1.33E-15	1.496	2.203	0.707	-0.186
2.8362	0.697	0.004	1.03E-185	2.084	0.007	2.077	0.693
1.0771	0.697	0.174	2.83E-51	1.732	0.409	1.323	0.523
0.9532	0.697	0.403	1.47E-39	1.392	0.521	0.871	0.294
0.5432	0.697	0.587	4.06E-18	1.417	0.899	0.518	0.110
-0.5601	0.697	0.885	3.18E-17	1.433	2.081	0.648	-0.188
0.6426	0.697	0.546	3.60E-16	1.338	0.763	0.575	0.151
0.4105	0.697	0.601	2.36E-04	1.356	0.980	0.377	0.096
0.9022	0.692	0.503	1.22E-58	1.370	0.618	0.752	0.189
-0.7361	0.692	0.748	4.88E-11	1.985	2.888	0.903	-0.056
-0.5277	0.692	0.717	1.33E-04	1.825	2.370	0.544	-0.025
-0.3322	0.692	0.810	9.88E-04	1.371	1.742	0.371	-0.118
0.3022	0.687	0.657	1.11E-05	1.238	0.978	0.260	0.030
0.4527	0.687	0.588	1.20E-05	1.326	0.903	0.423	0.099
2.7724	0.682	0.005	8.01E-181	1.998	0.006	1.992	0.677
1.0988	0.682	0.446	2.76E-23	1.549	0.643	0.906	0.236
0.4499	0.682	0.571	5.27E-05	1.183	0.819	0.364	0.111
2.5518	0.677	0.002	1.14E-176	1.873	0.005	1.869	0.675
2.3607	0.677	0.011	2.45E-147	1.810	0.019	1.791	0.666
0.9222	0.677	0.489	1.45E-29	1.411	0.655	0.756	0.188

0.5789	0.677	0.586	6.26E-15	1.262	0.810	0.452	0.091
0.6050	0.677	0.581	8.43E-15	1.287	0.817	0.470	0.096
-1.2400	0.672	0.945	3.32E-80	1.425	2.920	1.494	-0.273
1.0567	0.672	0.406	5.43E-60	1.308	0.476	0.832	0.266
0.8872	0.662	0.437	1.92E-29	1.218	0.530	0.687	0.225
0.4601	0.662	0.581	2.33E-09	1.215	0.809	0.406	0.081
0.3288	0.662	0.539	3.97E-07	1.188	0.782	0.405	0.123
0.9518	0.657	0.404	4.16E-49	1.199	0.476	0.723	0.253
0.8141	0.657	0.492	4.28E-26	1.235	0.612	0.623	0.165
0.9452	0.657	0.402	2.71E-22	1.225	0.490	0.735	0.255
0.5458	0.657	0.549	1.21E-11	1.176	0.733	0.443	0.108
-0.5012	0.657	0.802	1.41E-08	1.162	1.628	0.466	-0.145
0.3976	0.657	0.535	1.29E-06	1.246	0.852	0.394	0.122
0.6002	0.652	0.552	6.78E-20	1.258	0.769	0.490	0.100
0.2956	0.647	0.647	9.76E-03	1.173	0.962	0.211	0.000
0.5993	0.642	0.544	3.54E-21	1.209	0.713	0.496	0.098
-0.6165	0.642	0.708	1.57E-11	1.515	2.193	0.678	-0.066
1.2882	0.637	0.189	5.76E-47	1.256	0.211	1.046	0.448
0.8668	0.637	0.388	3.54E-25	1.153	0.464	0.689	0.249
-0.5327	0.637	0.708	4.42E-15	1.369	1.927	0.558	-0.071
-0.5992	0.637	0.754	2.02E-04	1.447	2.101	0.654	-0.117
0.7588	0.632	0.415	2.97E-32	1.110	0.492	0.618	0.217
0.7884	0.632	0.506	2.03E-19	1.314	0.720	0.594	0.126
0.4752	0.632	0.532	3.81E-12	1.152	0.732	0.420	0.100
1.1110	0.627	0.314	2.01E-29	1.235	0.378	0.857	0.313
0.5856	0.627	0.518	5.97E-21	1.143	0.684	0.459	0.109
0.5490	0.627	0.595	5.22E-17	1.243	0.874	0.369	0.032
0.6323	0.627	0.527	5.59E-15	1.160	0.694	0.466	0.100
0.4252	0.627	0.588	7.65E-08	1.092	0.803	0.288	0.039
0.7852	0.622	0.360	1.05E-35	1.305	0.551	0.753	0.262
0.7381	0.622	0.457	4.56E-21	1.102	0.542	0.560	0.165
2.0456	0.617	0.001	2.68E-159	1.441	0.002	1.439	0.616
1.9269	0.617	0.054	7.10E-141	1.449	0.053	1.396	0.563
1.7197	0.617	0.065	1.94E-129	1.304	0.065	1.239	0.552
-0.4638	0.617	0.805	1.48E-12	1.101	1.584	0.483	-0.188
0.4872	0.617	0.516	4.07E-08	1.121	0.693	0.428	0.101
0.4749	0.617	0.536	4.89E-08	1.041	0.689	0.352	0.081
1.8137	0.612	0.028	8.55E-112	1.316	0.029	1.287	0.584
1.8673	0.612	0.021	1.77E-111	1.381	0.025	1.357	0.591
0.4967	0.612	0.531	1.99E-12	1.061	0.733	0.329	0.081
0.4464	0.612	0.518	1.33E-05	1.027	0.690	0.337	0.094
0.8315	0.607	0.377	3.89E-24	1.061	0.418	0.643	0.230
0.4425	0.607	0.573	2.71E-09	1.105	0.795	0.311	0.034
0.6400	0.602	0.486	3.08E-19	1.113	0.625	0.488	0.116
0.4342	0.602	0.530	2.12E-13	1.059	0.715	0.344	0.072
0.5535	0.602	0.485	1.75E-10	1.014	0.607	0.407	0.117
1.2953	0.597	0.273	6.70E-44	1.208	0.311	0.897	0.324

0.9050	0.597	0.302	1.19E-17	1.132	0.410	0.723	0.295
0.4986	0.597	0.530	3.84E-08	1.049	0.698	0.351	0.067
1.1118	0.592	0.181	2.90E-44	1.209	0.289	0.920	0.411
0.7855	0.592	0.276	6.87E-36	1.088	0.370	0.718	0.316
0.3490	0.592	0.552	6.99E-09	1.082	0.765	0.318	0.040
2.2728	0.587	0.005	1.88E-129	1.488	0.005	1.482	0.582
0.9788	0.587	0.362	7.94E-45	1.085	0.402	0.683	0.225
-1.0631	0.582	0.946	3.45E-85	1.218	2.713	1.495	-0.364
0.7494	0.582	0.474	2.20E-42	1.086	0.566	0.520	0.108
1.1421	0.582	0.246	7.25E-30	1.129	0.296	0.832	0.336
0.8095	0.582	0.239	1.15E-20	1.132	0.371	0.761	0.343
0.7271	0.582	0.408	1.65E-10	1.002	0.477	0.525	0.174
0.5194	0.582	0.506	3.43E-10	0.983	0.638	0.345	0.076
0.3534	0.582	0.512	1.46E-05	0.939	0.661	0.278	0.070
1.7392	0.577	0.009	5.14E-123	1.226	0.009	1.217	0.568
1.5921	0.577	0.008	1.02E-122	1.128	0.010	1.118	0.569
0.5722	0.577	0.201	8.55E-20	1.229	0.458	0.771	0.376
0.5525	0.577	0.494	1.07E-13	0.985	0.618	0.367	0.083
0.8117	0.577	0.342	1.57E-06	1.112	0.462	0.650	0.235
1.7771	0.572	0.018	4.84E-118	1.250	0.016	1.234	0.554
1.8975	0.572	0.009	9.86E-114	1.274	0.015	1.259	0.563
1.3353	0.572	0.164	1.49E-87	1.110	0.162	0.948	0.408
0.6733	0.572	0.433	7.80E-28	0.974	0.504	0.470	0.139
1.2137	0.567	0.212	1.37E-47	1.104	0.225	0.879	0.355
0.9227	0.567	0.293	2.03E-36	0.994	0.323	0.671	0.274
0.6612	0.567	0.241	3.69E-31	1.112	0.403	0.708	0.326
0.4457	0.567	0.480	2.44E-13	0.935	0.597	0.338	0.087
-0.7254	0.567	0.738	4.31E-10	1.173	1.973	0.800	-0.171
0.5347	0.567	0.414	2.66E-08	1.018	0.579	0.440	0.153
1.7659	0.562	0.014	9.20E-124	1.222	0.013	1.209	0.548
1.2571	0.562	0.162	1.07E-66	1.062	0.171	0.891	0.400
0.4669	0.562	0.471	2.10E-11	0.914	0.587	0.327	0.091
0.5016	0.562	0.498	5.20E-07	0.948	0.614	0.334	0.064
-0.4482	0.562	0.771	3.45E-04	0.936	1.406	0.470	-0.209
0.3260	0.562	0.511	4.05E-04	0.903	0.660	0.243	0.051
0.4396	0.562	0.479	1.67E-03	0.958	0.619	0.339	0.083
1.7085	0.557	0.002	2.92E-126	1.155	0.004	1.151	0.555
1.5155	0.557	0.123	4.43E-86	1.107	0.121	0.986	0.434
1.0415	0.557	0.284	1.10E-58	1.008	0.293	0.715	0.273
0.7106	0.557	0.389	1.49E-30	0.958	0.442	0.516	0.168
0.6484	0.557	0.383	6.58E-30	0.930	0.449	0.482	0.174
0.8160	0.557	0.310	2.51E-26	0.871	0.318	0.553	0.247
0.7426	0.557	0.341	3.05E-25	0.932	0.390	0.542	0.216
-0.4268	0.557	0.322	7.97E-20	1.166	0.867	0.299	0.235
0.3143	0.557	0.518	1.61E-03	0.945	0.679	0.265	0.039
-0.5037	0.557	0.769	1.94E-03	1.001	1.563	0.563	-0.212
0.6774	0.552	0.345	4.57E-22	0.902	0.379	0.523	0.207

0.6637	0.552	0.362	8.59E-21	0.930	0.427	0.503	0.190
0.7024	0.552	0.372	9.74E-21	0.950	0.426	0.524	0.180
0.2865	0.552	0.515	4.41E-05	0.869	0.648	0.221	0.037
0.7075	0.547	0.340	1.47E-13	0.964	0.429	0.536	0.207
0.5050	0.547	0.427	2.42E-11	0.899	0.527	0.372	0.120
0.6101	0.547	0.412	7.86E-11	0.940	0.493	0.447	0.135
-0.4750	0.547	0.749	4.14E-09	0.904	1.409	0.505	-0.202
0.3308	0.547	0.421	6.70E-05	0.984	0.609	0.375	0.126
0.4157	0.547	0.465	3.48E-03	0.878	0.581	0.297	0.082
2.2400	0.542	0.001	9.91E-129	1.337	0.002	1.334	0.541
0.9424	0.542	0.318	1.38E-46	1.022	0.364	0.658	0.224
0.4469	0.542	0.444	2.21E-13	0.826	0.512	0.314	0.098
0.6026	0.542	0.429	1.30E-08	0.924	0.500	0.424	0.113
0.4629	0.542	0.421	6.51E-06	0.902	0.545	0.357	0.121
-0.4871	0.542	0.679	6.88E-04	1.184	1.788	0.604	-0.137
1.6414	0.537	0.001	8.68E-129	1.107	0.001	1.106	0.536
1.5820	0.537	0.001	8.37E-121	1.069	0.002	1.067	0.536
1.1277	0.537	0.181	4.10E-73	0.942	0.172	0.769	0.356
1.3492	0.537	0.186	2.21E-71	1.113	0.205	0.908	0.351
-0.9094	0.537	0.841	2.40E-43	0.941	1.932	0.991	-0.304
0.4239	0.537	0.459	5.54E-09	0.935	0.600	0.336	0.078
0.4971	0.537	0.433	4.53E-07	0.863	0.518	0.345	0.104
1.7372	0.532	0.056	3.79E-66	1.246	0.077	1.169	0.476
1.0708	0.532	0.138	3.70E-46	0.874	0.134	0.741	0.394
0.7997	0.532	0.291	7.55E-27	0.901	0.317	0.584	0.241
0.5091	0.532	0.397	5.91E-14	0.779	0.430	0.349	0.135
0.4251	0.532	0.514	8.67E-07	0.901	0.655	0.246	0.018
1.6072	0.527	0.001	3.89E-124	1.070	0.002	1.068	0.526
0.8821	0.527	0.261	8.10E-32	0.887	0.281	0.606	0.266
0.8127	0.527	0.302	1.18E-29	0.880	0.317	0.563	0.225
0.7459	0.527	0.332	1.97E-23	0.894	0.357	0.537	0.195
0.6154	0.527	0.359	1.26E-21	0.908	0.438	0.471	0.168
-0.8458	0.527	0.681	2.51E-16	0.847	1.476	0.629	-0.154
0.6882	0.527	0.327	8.44E-14	0.843	0.362	0.481	0.200
-0.7034	0.527	0.711	3.88E-12	1.096	1.927	0.831	-0.184
0.3978	0.527	0.413	2.95E-05	0.918	0.564	0.353	0.114
0.5538	0.527	0.376	1.16E-03	0.845	0.450	0.396	0.151
1.9009	0.522	0.001	9.72E-122	1.156	0.002	1.154	0.521
1.6164	0.522	0.001	6.15E-119	1.059	0.002	1.057	0.521
1.3283	0.522	0.142	4.53E-69	1.027	0.139	0.888	0.380
1.1650	0.522	0.098	8.21E-67	0.873	0.089	0.784	0.424
-0.7044	0.522	0.819	4.17E-24	0.849	1.604	0.755	-0.297
0.6635	0.522	0.341	3.46E-15	0.838	0.384	0.453	0.181
0.4958	0.522	0.405	1.18E-09	0.775	0.447	0.328	0.117
0.4421	0.522	0.476	4.64E-07	0.805	0.565	0.240	0.046
1.8490	0.517	0.002	3.42E-114	1.156	0.003	1.153	0.515
1.1363	0.517	0.133	2.24E-56	0.882	0.123	0.759	0.384

1.0115	0.517	0.128	1.83E-26	1.057	0.214	0.843	0.389
1.1203	0.517	0.210	1.74E-25	1.039	0.261	0.778	0.307
0.4883	0.517	0.260	7.06E-17	0.877	0.384	0.494	0.257
0.7012	0.517	0.324	2.71E-15	0.815	0.344	0.471	0.193
0.5575	0.517	0.421	9.75E-13	0.858	0.480	0.377	0.096
0.4863	0.517	0.437	1.40E-07	0.841	0.514	0.327	0.080
1.6593	0.512	0.001	7.93E-114	1.048	0.002	1.046	0.511
0.7905	0.512	0.351	2.46E-40	0.871	0.376	0.495	0.161
-1.0610	0.512	0.795	1.83E-34	0.843	1.735	0.891	-0.283
0.7259	0.512	0.319	1.05E-15	0.837	0.341	0.496	0.193
0.4273	0.512	0.433	9.53E-06	0.787	0.515	0.272	0.079
0.4268	0.512	0.442	2.29E-05	0.774	0.515	0.259	0.070
1.5710	0.507	0.024	4.60E-82	1.039	0.031	1.008	0.483
1.2067	0.507	0.076	6.62E-54	1.033	0.127	0.906	0.431
0.7588	0.507	0.160	1.77E-21	0.938	0.271	0.667	0.347
0.5193	0.507	0.403	1.00E-10	0.807	0.447	0.360	0.104
-0.3853	0.507	0.665	1.28E-04	0.857	1.260	0.403	-0.158
1.6658	0.502	0.016	5.78E-99	1.082	0.015	1.067	0.486
1.0071	0.502	0.198	5.69E-36	0.865	0.198	0.667	0.304
0.9170	0.502	0.331	1.32E-15	0.949	0.386	0.564	0.171
0.6412	0.502	0.291	1.60E-11	0.747	0.297	0.449	0.211
-0.7209	0.502	0.706	3.12E-11	1.117	1.941	0.825	-0.204
0.9432	0.498	0.207	9.69E-20	0.894	0.247	0.647	0.291
0.4684	0.498	0.358	6.90E-11	0.716	0.401	0.315	0.140
0.3993	0.498	0.411	8.55E-08	0.755	0.483	0.272	0.087
0.5356	0.498	0.388	5.13E-05	0.783	0.463	0.320	0.110
1.0598	0.493	0.181	2.86E-61	0.862	0.179	0.683	0.312
0.5471	0.493	0.364	1.39E-10	0.787	0.426	0.361	0.129
0.6280	0.493	0.377	8.81E-09	0.882	0.463	0.419	0.116
-0.3622	0.493	0.522	4.66E-04	0.836	0.947	0.111	-0.029
0.4757	0.493	0.380	2.53E-03	0.711	0.418	0.292	0.113
0.7198	0.488	0.265	2.11E-25	0.777	0.275	0.502	0.223
0.5818	0.488	0.349	7.38E-23	0.756	0.381	0.375	0.139
0.7014	0.488	0.300	2.35E-18	0.766	0.322	0.444	0.188
0.5824	0.488	0.381	3.14E-16	0.814	0.435	0.379	0.107
0.5841	0.488	0.284	5.46E-09	0.693	0.294	0.399	0.204
0.5695	0.488	0.349	1.12E-05	0.758	0.381	0.377	0.139
0.3017	0.488	0.487	7.24E-03	0.764	0.597	0.167	0.001
0.8873	0.483	0.217	6.88E-30	0.792	0.220	0.572	0.266
0.6213	0.483	0.339	3.36E-10	0.768	0.376	0.391	0.144
0.4540	0.483	0.396	3.62E-10	0.723	0.440	0.283	0.087
0.4854	0.483	0.396	3.27E-08	0.745	0.457	0.288	0.087
0.4477	0.483	0.391	3.39E-06	0.753	0.459	0.293	0.092
-0.4118	0.483	0.703	3.57E-04	0.745	1.153	0.408	-0.220
1.1533	0.478	0.041	9.71E-46	0.848	0.056	0.792	0.437
1.0561	0.478	0.118	9.50E-38	0.909	0.171	0.738	0.360
0.7419	0.478	0.326	3.78E-31	0.794	0.333	0.460	0.152

0.7740	0.478	0.273	2.87E-25	0.784	0.288	0.496	0.205
0.7869	0.478	0.221	1.84E-24	0.720	0.223	0.496	0.257
0.7068	0.478	0.313	3.50E-15	0.758	0.321	0.437	0.165
0.7117	0.478	0.263	7.83E-14	0.729	0.267	0.462	0.215
-0.7300	0.478	0.499	2.54E-11	0.716	0.995	0.278	-0.021
0.4318	0.478	0.364	1.42E-09	0.694	0.400	0.295	0.114
-0.6454	0.478	0.589	1.00E-06	0.799	1.110	0.312	-0.111
0.3544	0.478	0.402	2.24E-05	0.814	0.537	0.277	0.076
0.3078	0.478	0.425	4.54E-05	0.782	0.569	0.213	0.053
-0.5415	0.478	0.568	1.25E-04	0.774	1.059	0.285	-0.090
1.4708	0.473	0.006	2.40E-78	0.952	0.010	0.943	0.467
1.4455	0.473	0.047	1.18E-73	0.969	0.046	0.923	0.426
0.9843	0.473	0.190	2.33E-42	0.857	0.205	0.651	0.283
0.7606	0.473	0.221	4.44E-35	0.747	0.229	0.518	0.252
0.2976	0.473	0.538	8.36E-11	0.802	0.694	0.108	-0.065
0.4161	0.473	0.357	7.09E-08	0.681	0.396	0.285	0.116
0.4080	0.473	0.394	4.10E-03	0.691	0.440	0.251	0.079
1.5633	0.468	0.019	1.45E-74	0.973	0.030	0.943	0.449
1.1143	0.468	0.076	1.32E-44	0.800	0.077	0.723	0.392
-1.5044	0.468	0.802	1.64E-36	0.714	1.772	1.058	-0.334
0.7095	0.468	0.269	1.72E-16	0.742	0.278	0.464	0.199
0.5731	0.468	0.329	2.68E-13	0.725	0.361	0.364	0.139
0.8742	0.463	0.192	2.22E-41	0.734	0.186	0.548	0.271
0.9144	0.463	0.201	2.69E-34	0.846	0.234	0.612	0.262
0.7646	0.463	0.161	1.38E-26	0.735	0.180	0.555	0.302
0.9207	0.463	0.144	1.81E-25	0.833	0.187	0.647	0.319
0.5181	0.463	0.337	3.33E-14	0.748	0.385	0.363	0.126
0.5263	0.463	0.346	1.34E-06	0.696	0.368	0.328	0.117
0.4077	0.463	0.373	7.58E-04	0.670	0.438	0.232	0.090
0.2684	0.463	0.419	3.63E-03	0.648	0.486	0.162	0.044
1.3238	0.458	0.001	2.86E-92	0.829	0.002	0.827	0.457
1.3989	0.458	0.007	1.06E-82	0.868	0.008	0.860	0.451
1.0953	0.458	0.110	1.01E-42	0.801	0.105	0.696	0.348
0.6686	0.458	0.242	8.39E-15	0.662	0.242	0.420	0.216
0.5567	0.458	0.315	4.84E-14	0.684	0.330	0.353	0.143
0.6272	0.458	0.186	8.27E-14	0.897	0.340	0.557	0.272
-0.7604	0.458	0.637	6.34E-11	0.723	1.224	0.501	-0.179
0.4993	0.458	0.342	3.26E-10	0.667	0.377	0.290	0.116
0.5112	0.458	0.308	3.37E-05	0.654	0.328	0.326	0.150
0.4326	0.458	0.402	3.11E-03	0.684	0.459	0.225	0.056
0.2805	0.458	0.414	7.57E-03	0.643	0.467	0.176	0.044
-0.6135	0.453	0.474	1.72E-06	0.822	1.042	0.219	-0.021
1.4103	0.448	0.001	9.60E-95	0.832	0.001	0.831	0.447
0.6917	0.448	0.246	1.00E-35	0.723	0.260	0.463	0.202
0.7050	0.448	0.076	1.70E-24	0.885	0.183	0.702	0.372
0.5159	0.448	0.277	5.72E-22	0.671	0.304	0.367	0.171
0.6298	0.448	0.320	9.31E-18	0.709	0.336	0.373	0.128

0.5328	0.448	0.286	1.65E-09	0.628	0.290	0.337	0.162
0.5090	0.448	0.280	3.34E-07	0.639	0.297	0.342	0.168
0.3689	0.448	0.354	6.13E-06	0.610	0.390	0.220	0.094
0.4426	0.448	0.306	6.18E-06	0.632	0.336	0.296	0.142
1.0687	0.443	0.055	9.78E-38	0.840	0.087	0.753	0.388
0.8678	0.443	0.153	4.96E-36	0.701	0.150	0.551	0.290
0.7061	0.443	0.266	1.22E-31	0.685	0.258	0.427	0.177
0.7641	0.443	0.211	1.07E-26	0.683	0.206	0.476	0.232
0.7158	0.443	0.283	2.77E-24	0.712	0.301	0.412	0.160
0.6897	0.443	0.231	5.65E-19	0.660	0.236	0.424	0.212
0.3923	0.443	0.319	1.03E-04	0.605	0.357	0.248	0.124
0.2881	0.443	0.428	5.30E-04	0.669	0.522	0.147	0.015
1.1847	0.438	0.028	1.13E-77	0.756	0.026	0.730	0.410
1.2002	0.438	0.006	4.61E-75	0.740	0.006	0.734	0.432
1.4310	0.438	0.005	7.14E-72	0.854	0.008	0.846	0.433
1.3977	0.438	0.009	1.18E-70	0.820	0.009	0.811	0.429
1.3067	0.438	0.026	6.61E-44	0.855	0.034	0.821	0.412
0.4799	0.438	0.287	3.26E-20	0.668	0.330	0.338	0.151
0.6124	0.438	0.262	1.78E-13	0.658	0.284	0.375	0.176
0.5606	0.438	0.300	1.52E-11	0.658	0.314	0.344	0.138
0.4210	0.438	0.321	1.10E-04	0.582	0.332	0.250	0.117
0.2795	0.438	0.528	1.77E-03	0.787	0.700	0.086	-0.090
1.2822	0.433	0.001	1.54E-84	0.774	0.002	0.772	0.432
1.2723	0.433	0.002	4.20E-83	0.778	0.003	0.776	0.431
1.2865	0.433	0.001	1.44E-81	0.801	0.002	0.799	0.432
1.3026	0.433	0.031	2.70E-77	0.775	0.027	0.748	0.402
0.8823	0.433	0.152	7.82E-19	0.756	0.182	0.574	0.281
0.6135	0.433	0.200	6.74E-17	0.601	0.194	0.408	0.233
0.6695	0.433	0.244	1.10E-14	0.725	0.281	0.444	0.189
0.3963	0.433	0.363	9.82E-13	0.678	0.415	0.263	0.070
0.4219	0.433	0.340	1.70E-09	0.630	0.359	0.271	0.093
0.4372	0.433	0.323	5.31E-09	0.596	0.339	0.257	0.110
0.5140	0.433	0.328	1.38E-07	0.632	0.346	0.286	0.105
0.4151	0.433	0.308	4.42E-07	0.615	0.344	0.271	0.125
0.4182	0.433	0.343	6.35E-06	0.676	0.405	0.271	0.090
0.5772	0.433	0.290	2.56E-05	0.696	0.327	0.370	0.143
1.2392	0.428	0.001	2.88E-82	0.748	0.001	0.746	0.427
0.9436	0.428	0.163	1.36E-53	0.709	0.155	0.554	0.265
1.0705	0.428	0.069	2.58E-51	0.718	0.063	0.655	0.359
0.8400	0.428	0.150	4.92E-35	0.644	0.139	0.505	0.278
-0.8878	0.428	0.789	6.40E-34	0.629	1.511	0.882	-0.361
0.6991	0.428	0.196	7.47E-21	0.622	0.187	0.434	0.232
0.6227	0.428	0.260	8.88E-15	0.638	0.271	0.367	0.168
0.5750	0.428	0.154	7.12E-09	0.667	0.194	0.473	0.274
0.5364	0.428	0.296	1.82E-08	0.609	0.301	0.307	0.132
0.3551	0.428	0.345	1.85E-08	0.600	0.368	0.232	0.083
0.4107	0.428	0.325	2.03E-03	0.586	0.356	0.230	0.103

1.2371	0.423	0.016	2.64E-69	0.760	0.015	0.745	0.407
1.1404	0.423	0.045	2.98E-33	0.842	0.079	0.763	0.378
0.8386	0.423	0.102	1.70E-29	0.662	0.113	0.548	0.321
0.6638	0.423	0.245	1.34E-28	0.639	0.247	0.392	0.178
0.7858	0.423	0.121	8.05E-28	0.656	0.131	0.525	0.302
0.7012	0.423	0.236	2.98E-27	0.658	0.229	0.429	0.187
0.4718	0.423	0.343	1.43E-12	0.643	0.362	0.281	0.080
0.2566	0.423	0.390	4.66E-09	0.628	0.474	0.154	0.033
0.5311	0.423	0.315	1.22E-08	0.638	0.345	0.293	0.108
0.5157	0.423	0.259	1.53E-08	0.572	0.261	0.311	0.164
-0.6452	0.423	0.618	1.49E-06	0.548	0.989	0.442	-0.195
1.3111	0.418	0.001	2.21E-81	0.749	0.001	0.748	0.417
1.0758	0.418	0.092	1.45E-55	0.741	0.089	0.652	0.326
0.8000	0.418	0.128	3.57E-35	0.619	0.126	0.494	0.290
-1.0810	0.418	0.271	3.68E-07	0.722	0.868	0.147	0.147
0.3888	0.418	0.318	1.52E-06	0.561	0.332	0.230	0.100
0.4847	0.418	0.292	1.72E-06	0.604	0.303	0.300	0.126
0.3832	0.418	0.312	1.83E-05	0.567	0.347	0.220	0.106
0.5057	0.418	0.240	3.58E-04	0.576	0.270	0.306	0.178
1.1369	0.413	0.001	2.62E-78	0.685	0.001	0.683	0.412
0.6797	0.413	0.207	7.09E-25	0.609	0.203	0.405	0.206
0.7098	0.413	0.231	4.06E-18	0.640	0.236	0.404	0.182
0.6380	0.413	0.190	1.80E-15	0.578	0.178	0.399	0.223
0.4893	0.413	0.257	4.63E-10	0.616	0.291	0.325	0.156
-0.7179	0.413	0.501	4.15E-09	0.624	0.913	0.290	-0.088
-0.5506	0.413	0.594	3.65E-08	0.609	1.054	0.445	-0.181
0.4778	0.413	0.312	6.60E-07	0.594	0.336	0.259	0.101
0.8704	0.408	0.083	1.61E-34	0.622	0.077	0.545	0.325
0.8621	0.408	0.134	1.59E-29	0.670	0.143	0.527	0.274
0.6331	0.408	0.164	3.27E-16	0.558	0.161	0.397	0.244
0.5753	0.408	0.204	1.90E-15	0.588	0.207	0.381	0.204
-0.6097	0.408	0.671	2.51E-07	0.856	1.555	0.699	-0.263
0.4374	0.408	0.295	5.01E-07	0.597	0.339	0.259	0.113
0.4533	0.408	0.295	1.17E-06	0.539	0.293	0.246	0.113
0.4585	0.408	0.309	2.74E-04	0.566	0.324	0.242	0.099
-0.6711	0.408	0.253	9.74E-03	0.856	0.884	0.028	0.155
1.1816	0.403	0.006	2.36E-66	0.709	0.007	0.701	0.397
0.9369	0.403	0.102	1.87E-24	0.659	0.096	0.563	0.301
0.8062	0.403	0.199	7.70E-18	0.648	0.205	0.442	0.204
0.4986	0.403	0.259	2.46E-13	0.594	0.288	0.306	0.144
0.5600	0.403	0.287	1.81E-10	0.612	0.299	0.313	0.116
0.4621	0.403	0.304	7.13E-09	0.567	0.323	0.245	0.099
-0.6828	0.403	0.627	2.41E-08	0.575	1.101	0.525	-0.224
0.4766	0.403	0.284	9.63E-08	0.548	0.291	0.256	0.119
0.3037	0.403	0.401	8.42E-07	0.575	0.445	0.130	0.002
0.4075	0.403	0.290	8.30E-05	0.540	0.299	0.241	0.113
0.4054	0.403	0.270	1.82E-04	0.500	0.274	0.226	0.133

0.4111	0.403	0.276	4.48E-03	0.546	0.311	0.235	0.127
-1.0503	0.398	0.685	6.87E-25	0.694	1.587	0.893	-0.287
0.6661	0.398	0.191	3.10E-19	0.589	0.194	0.395	0.207
0.4479	0.398	0.240	2.92E-11	0.585	0.279	0.306	0.158
0.3861	0.398	0.302	1.06E-06	0.552	0.323	0.229	0.096
0.4866	0.398	0.292	1.01E-05	0.586	0.302	0.284	0.106
0.4311	0.398	0.307	4.73E-03	0.554	0.322	0.231	0.091
1.1212	0.393	0.001	2.04E-72	0.652	0.002	0.649	0.392
1.5165	0.393	0.022	4.17E-52	0.829	0.023	0.806	0.371
1.3520	0.393	0.029	3.22E-34	0.809	0.054	0.755	0.364
1.0159	0.393	0.063	1.43E-27	0.679	0.064	0.615	0.330
0.6888	0.393	0.170	3.30E-25	0.596	0.166	0.430	0.223
0.6746	0.393	0.184	1.59E-22	0.595	0.185	0.410	0.209
0.5053	0.393	0.287	2.61E-14	0.586	0.304	0.282	0.106
0.4447	0.393	0.185	3.31E-13	0.551	0.225	0.326	0.208
0.4617	0.393	0.222	2.17E-08	0.500	0.225	0.275	0.171
0.2852	0.393	0.238	1.41E-03	0.477	0.272	0.205	0.155
0.8513	0.388	0.083	1.13E-29	0.584	0.075	0.510	0.305
0.6962	0.388	0.158	1.06E-28	0.561	0.153	0.407	0.230
0.6493	0.388	0.161	1.67E-24	0.535	0.153	0.382	0.227
0.4561	0.388	0.276	6.06E-20	0.568	0.294	0.275	0.112
0.5900	0.388	0.211	7.58E-19	0.552	0.206	0.346	0.177
0.7024	0.388	0.176	3.97E-17	0.579	0.175	0.404	0.212
0.4653	0.388	0.218	3.90E-10	0.560	0.251	0.309	0.170
0.4621	0.388	0.283	3.69E-08	0.551	0.290	0.261	0.105
-0.4949	0.388	0.615	5.30E-05	0.479	0.895	0.416	-0.227
0.4223	0.388	0.270	8.34E-05	0.517	0.271	0.246	0.118
0.2577	0.388	0.364	2.88E-04	0.534	0.410	0.124	0.024
0.2867	0.388	0.317	7.03E-03	0.521	0.364	0.157	0.071
1.0832	0.383	0.024	8.33E-57	0.653	0.020	0.633	0.359
1.2787	0.383	0.041	2.37E-50	0.679	0.039	0.640	0.342
0.9879	0.383	0.047	2.61E-42	0.636	0.049	0.588	0.336
1.0728	0.383	0.024	1.03E-33	0.662	0.030	0.633	0.359
0.6079	0.383	0.154	6.28E-27	0.514	0.154	0.361	0.229
0.6764	0.383	0.209	1.50E-26	0.587	0.203	0.384	0.174
0.5631	0.383	0.175	1.19E-17	0.509	0.168	0.340	0.208
0.6004	0.383	0.108	2.07E-17	0.548	0.131	0.417	0.275
0.4385	0.383	0.211	1.44E-07	0.504	0.218	0.286	0.172
0.4651	0.383	0.265	1.83E-05	0.540	0.269	0.271	0.118
-0.7546	0.383	0.576	2.69E-05	0.506	0.919	0.413	-0.193
0.2558	0.383	0.336	2.47E-04	0.505	0.370	0.135	0.047
0.2901	0.383	0.333	9.75E-04	0.523	0.363	0.160	0.050
0.5915	0.378	0.190	2.46E-12	0.511	0.178	0.333	0.188
0.5841	0.378	0.218	2.67E-11	0.526	0.209	0.317	0.160
-0.5351	0.378	0.642	1.90E-06	0.470	0.952	0.482	-0.264
0.5207	0.378	0.206	2.44E-06	0.494	0.204	0.290	0.172
0.3432	0.378	0.285	3.09E-06	0.508	0.305	0.203	0.093

0.2840	0.378	0.261	8.89E-04	0.552	0.330	0.221	0.117
1.1022	0.373	0.001	9.70E-66	0.635	0.001	0.633	0.372
0.7349	0.373	0.117	3.74E-27	0.529	0.106	0.423	0.256
0.4582	0.373	0.282	1.82E-16	0.553	0.302	0.251	0.091
0.5427	0.373	0.182	5.31E-14	0.507	0.180	0.328	0.191
0.4993	0.373	0.198	1.58E-10	0.501	0.196	0.305	0.175
-0.4648	0.373	0.611	2.36E-03	0.478	0.901	0.423	-0.238
1.3017	0.368	0.001	2.16E-73	0.699	0.001	0.698	0.367
0.7380	0.368	0.115	2.56E-28	0.541	0.112	0.429	0.253
0.7143	0.368	0.119	5.07E-24	0.522	0.117	0.405	0.249
-1.2283	0.368	0.598	1.21E-17	0.693	1.683	0.990	-0.230
0.4090	0.368	0.261	9.76E-11	0.485	0.261	0.223	0.107
0.5281	0.368	0.192	4.49E-08	0.494	0.186	0.309	0.176
0.5026	0.368	0.204	1.18E-05	0.505	0.200	0.305	0.164
-0.4983	0.368	0.573	4.48E-03	0.444	0.857	0.413	-0.205
1.7410	0.363	0.001	8.59E-71	0.787	0.001	0.786	0.362
1.0167	0.363	0.001	2.18E-61	0.590	0.002	0.589	0.362
1.0825	0.363	0.017	4.54E-53	0.609	0.016	0.594	0.346
0.8395	0.363	0.079	3.73E-47	0.536	0.067	0.469	0.284
1.0870	0.363	0.065	7.73E-42	0.632	0.063	0.569	0.298
-0.7119	0.363	0.637	1.34E-15	0.435	1.009	0.575	-0.274
0.5142	0.363	0.179	3.88E-15	0.467	0.172	0.296	0.184
0.6190	0.363	0.181	5.36E-09	0.588	0.209	0.380	0.182
0.4743	0.363	0.211	6.17E-05	0.461	0.206	0.254	0.152
0.3813	0.363	0.241	7.62E-05	0.466	0.236	0.231	0.122
-0.6832	0.363	0.406	1.59E-03	0.587	0.904	0.317	-0.043
0.4004	0.363	0.219	1.68E-03	0.456	0.220	0.236	0.144
0.6563	0.358	0.118	4.39E-26	0.480	0.106	0.374	0.240
0.5630	0.358	0.191	1.08E-21	0.484	0.178	0.307	0.167
-1.2416	0.358	0.627	9.09E-20	0.469	1.234	0.765	-0.269
0.4941	0.358	0.211	2.11E-09	0.464	0.207	0.258	0.147
0.4130	0.358	0.238	3.70E-09	0.459	0.232	0.226	0.120
0.4965	0.358	0.239	2.51E-07	0.529	0.262	0.267	0.119
0.5547	0.358	0.146	4.37E-04	0.525	0.180	0.345	0.212
0.9904	0.353	0.001	1.88E-68	0.553	0.001	0.552	0.352
1.2126	0.353	0.002	2.13E-59	0.639	0.003	0.635	0.351
0.9551	0.353	0.012	1.35E-53	0.556	0.011	0.545	0.341
0.5926	0.353	0.158	1.18E-23	0.490	0.162	0.328	0.195
0.5306	0.353	0.202	1.90E-22	0.511	0.215	0.296	0.151
0.4382	0.353	0.219	8.77E-14	0.454	0.211	0.244	0.134
0.4402	0.353	0.288	8.19E-13	0.506	0.285	0.221	0.065
0.4501	0.353	0.270	1.21E-11	0.489	0.266	0.223	0.083
0.4575	0.353	0.221	1.15E-08	0.488	0.230	0.258	0.132
-0.5857	0.353	0.613	7.90E-08	0.450	0.925	0.475	-0.260
0.4692	0.353	0.241	8.49E-08	0.486	0.233	0.253	0.112
0.3423	0.353	0.270	2.45E-07	0.507	0.296	0.211	0.083
0.2608	0.353	0.189	6.28E-05	0.460	0.248	0.211	0.164

0.3972	0.353	0.240	1.44E-04	0.451	0.241	0.210	0.113
1.0101	0.348	0.001	1.01E-57	0.551	0.002	0.549	0.347
0.9215	0.348	0.006	2.11E-47	0.530	0.006	0.525	0.342
0.9549	0.348	0.012	4.25E-41	0.563	0.012	0.551	0.336
0.5662	0.348	0.129	2.84E-25	0.484	0.133	0.350	0.219
0.6329	0.348	0.105	2.76E-22	0.457	0.096	0.361	0.243
0.6270	0.348	0.115	2.86E-21	0.458	0.105	0.353	0.233
0.5339	0.348	0.175	1.15E-19	0.516	0.188	0.328	0.173
-0.5629	0.348	0.607	2.35E-08	0.446	0.918	0.473	-0.259
0.4460	0.348	0.180	1.36E-06	0.452	0.180	0.272	0.168
0.6093	0.348	0.176	2.14E-06	0.465	0.164	0.301	0.172
0.2758	0.348	0.219	5.33E-06	0.452	0.262	0.190	0.129
0.4142	0.348	0.203	1.42E-04	0.428	0.192	0.236	0.145
-0.5553	0.348	0.583	2.78E-03	0.403	0.824	0.420	-0.235
0.8572	0.343	0.060	6.36E-33	0.557	0.059	0.498	0.283
0.5296	0.343	0.139	3.51E-24	0.443	0.131	0.312	0.204
0.5118	0.343	0.215	1.08E-22	0.496	0.212	0.284	0.128
0.6469	0.343	0.094	2.12E-21	0.470	0.093	0.377	0.249
0.5591	0.343	0.179	6.67E-20	0.485	0.183	0.302	0.164
0.5462	0.343	0.093	1.04E-19	0.421	0.084	0.338	0.250
0.5704	0.343	0.145	3.50E-14	0.468	0.151	0.316	0.198
0.4511	0.343	0.213	5.51E-12	0.446	0.208	0.237	0.130
0.3801	0.343	0.229	5.58E-09	0.464	0.243	0.221	0.114
0.5673	0.343	0.124	1.83E-08	0.446	0.130	0.316	0.219
0.3397	0.343	0.259	2.19E-08	0.447	0.261	0.186	0.084
0.2773	0.343	0.276	1.01E-07	0.506	0.341	0.165	0.067
0.4325	0.343	0.189	5.69E-06	0.435	0.186	0.248	0.154
0.3444	0.343	0.287	2.11E-03	0.442	0.300	0.142	0.056
0.3013	0.343	0.230	5.86E-03	0.403	0.233	0.171	0.113
0.9127	0.338	0.005	2.61E-54	0.515	0.004	0.511	0.333
0.8458	0.338	0.007	2.75E-45	0.488	0.007	0.481	0.331
-1.1186	0.338	0.701	1.19E-35	0.538	1.535	0.997	-0.363
-0.8331	0.338	0.734	1.10E-25	0.469	1.277	0.807	-0.396
0.6198	0.338	0.064	2.60E-23	0.425	0.061	0.364	0.274
0.5518	0.338	0.171	1.63E-15	0.467	0.168	0.299	0.167
0.4741	0.338	0.196	1.37E-13	0.460	0.190	0.270	0.142
0.4272	0.338	0.216	1.95E-11	0.496	0.246	0.250	0.122
0.3787	0.338	0.252	7.74E-11	0.467	0.267	0.200	0.086
0.5019	0.338	0.163	1.23E-09	0.440	0.152	0.288	0.175
0.3472	0.338	0.228	1.01E-08	0.426	0.232	0.194	0.110
-0.8313	0.338	0.446	1.10E-07	0.503	0.958	0.455	-0.108
0.3424	0.338	0.178	2.34E-06	0.379	0.171	0.208	0.160
0.4770	0.338	0.162	3.30E-04	0.455	0.177	0.278	0.176
0.3180	0.338	0.209	3.38E-03	0.378	0.202	0.175	0.129
0.3160	0.338	0.250	3.92E-03	0.420	0.258	0.162	0.088
0.6034	0.333	0.075	4.36E-26	0.422	0.071	0.351	0.258
0.6514	0.333	0.072	8.51E-16	0.472	0.069	0.403	0.261

0.4180	0.333	0.177	1.33E-12	0.411	0.169	0.242	0.156
0.6483	0.333	0.112	3.10E-12	0.472	0.117	0.354	0.221
0.4621	0.333	0.208	3.26E-09	0.433	0.213	0.220	0.125
-1.5437	0.333	0.255	1.09E-07	0.533	0.587	0.054	0.078
0.3871	0.333	0.185	1.21E-07	0.410	0.189	0.221	0.148
-0.6795	0.333	0.555	3.17E-06	0.398	0.852	0.454	-0.222
0.2861	0.333	0.278	1.16E-05	0.425	0.275	0.150	0.055
-0.5038	0.333	0.583	2.68E-05	0.414	0.840	0.426	-0.250
0.2798	0.333	0.166	2.73E-05	0.454	0.229	0.225	0.167
0.3455	0.333	0.220	2.09E-04	0.415	0.223	0.192	0.113
-0.3075	0.333	0.384	2.96E-03	0.406	0.542	0.135	-0.051
0.6960	0.328	0.082	1.87E-28	0.452	0.073	0.379	0.246
0.7805	0.328	0.077	3.23E-23	0.464	0.076	0.389	0.251
-1.0420	0.328	0.655	6.53E-20	0.437	1.267	0.830	-0.327
-1.3513	0.328	0.508	1.51E-14	0.490	1.236	0.747	-0.180
0.5494	0.328	0.155	1.13E-10	0.458	0.148	0.310	0.173
0.6480	0.328	0.176	6.28E-08	0.493	0.187	0.307	0.152
0.3593	0.328	0.194	1.17E-06	0.378	0.191	0.187	0.134
-0.6169	0.328	0.556	2.18E-06	0.395	0.834	0.439	-0.228
0.4261	0.328	0.214	1.29E-05	0.403	0.206	0.197	0.114
0.3711	0.328	0.179	3.72E-05	0.376	0.169	0.207	0.149
0.3812	0.328	0.212	3.55E-04	0.393	0.203	0.191	0.116
0.3659	0.328	0.189	3.81E-03	0.431	0.215	0.217	0.139
0.3043	0.328	0.269	8.43E-03	0.411	0.280	0.131	0.059
0.9318	0.323	0.001	6.43E-56	0.508	0.001	0.507	0.322
0.9428	0.323	0.002	3.22E-55	0.507	0.002	0.505	0.321
0.9401	0.323	0.001	1.19E-51	0.513	0.001	0.513	0.322
0.5988	0.323	0.082	7.25E-26	0.423	0.077	0.345	0.241
0.5499	0.323	0.109	2.85E-19	0.391	0.100	0.291	0.214
0.5768	0.323	0.145	5.12E-13	0.442	0.137	0.304	0.178
0.5077	0.323	0.161	2.64E-12	0.423	0.150	0.273	0.162
0.5500	0.323	0.141	2.84E-11	0.427	0.136	0.292	0.182
0.4827	0.323	0.129	1.83E-09	0.389	0.126	0.263	0.194
0.3643	0.323	0.201	1.95E-09	0.395	0.195	0.200	0.122
0.4097	0.323	0.180	5.39E-09	0.399	0.166	0.232	0.143
0.4154	0.323	0.164	3.52E-08	0.397	0.160	0.237	0.159
0.2885	0.323	0.258	1.02E-05	0.423	0.281	0.142	0.065
-0.5581	0.323	0.511	1.43E-03	0.368	0.755	0.387	-0.188
0.3410	0.323	0.226	1.48E-03	0.420	0.238	0.182	0.097
0.8753	0.318	0.001	6.00E-49	0.473	0.001	0.473	0.317
0.8398	0.318	0.004	2.17E-42	0.481	0.005	0.476	0.314
-0.9091	0.318	0.708	2.30E-29	0.479	1.292	0.813	-0.390
0.6343	0.318	0.084	1.01E-28	0.421	0.074	0.347	0.234
0.5457	0.318	0.129	3.61E-14	0.440	0.130	0.310	0.189
0.4769	0.318	0.135	1.12E-09	0.375	0.127	0.249	0.183
0.4046	0.318	0.145	4.79E-09	0.448	0.178	0.271	0.173
0.4526	0.318	0.144	2.35E-08	0.406	0.136	0.270	0.174

0.3202	0.318	0.184	3.17E-07	0.433	0.210	0.223	0.134
0.3449	0.318	0.167	7.17E-07	0.378	0.162	0.215	0.151
0.4937	0.318	0.178	1.11E-05	0.433	0.177	0.257	0.140
-0.7449	0.318	0.506	1.54E-05	0.379	0.833	0.454	-0.188
0.3084	0.318	0.201	1.16E-03	0.357	0.198	0.159	0.117
0.8628	0.313	0.001	1.13E-43	0.455	0.001	0.453	0.312
0.6948	0.313	0.044	6.85E-31	0.434	0.040	0.394	0.269
0.6034	0.313	0.103	1.35E-23	0.413	0.095	0.318	0.210
0.6083	0.313	0.071	1.42E-19	0.413	0.062	0.351	0.242
0.6203	0.313	0.086	2.55E-18	0.423	0.087	0.336	0.227
0.8951	0.313	0.071	3.39E-16	0.539	0.077	0.462	0.242
-0.7341	0.313	0.652	6.16E-13	0.368	1.001	0.633	-0.339
0.5349	0.313	0.130	1.33E-12	0.416	0.132	0.284	0.183
0.4833	0.313	0.196	3.15E-11	0.423	0.193	0.229	0.117
0.4739	0.313	0.156	5.41E-11	0.395	0.155	0.240	0.157
0.5156	0.313	0.100	3.40E-09	0.366	0.090	0.277	0.213
0.3960	0.313	0.165	7.48E-07	0.375	0.157	0.218	0.148
0.3995	0.313	0.178	8.04E-06	0.360	0.167	0.193	0.135
0.4594	0.313	0.176	9.66E-06	0.396	0.165	0.231	0.137
0.3411	0.313	0.212	1.21E-04	0.383	0.201	0.181	0.101
0.3176	0.313	0.204	2.92E-03	0.374	0.210	0.165	0.109
0.9053	0.308	0.000	2.09E-49	0.497	0.000	0.497	0.308
0.9063	0.308	0.002	1.44E-47	0.459	0.002	0.456	0.306
0.7107	0.308	0.051	3.41E-24	0.448	0.050	0.398	0.257
0.6412	0.308	0.096	6.79E-23	0.447	0.091	0.357	0.212
0.5331	0.308	0.107	1.63E-14	0.402	0.098	0.304	0.201
0.4285	0.308	0.185	7.38E-13	0.393	0.179	0.214	0.123
0.6378	0.308	0.065	6.09E-11	0.447	0.074	0.373	0.243
0.4314	0.308	0.136	1.02E-08	0.346	0.122	0.223	0.172
0.4091	0.308	0.174	5.69E-05	0.383	0.175	0.208	0.134
-0.4215	0.308	0.538	2.12E-04	0.374	0.755	0.381	-0.230
-0.5528	0.308	0.522	1.18E-03	0.357	0.761	0.404	-0.214
0.7718	0.303	0.000	2.93E-49	0.431	0.001	0.431	0.303
0.6220	0.303	0.073	5.65E-36	0.397	0.062	0.335	0.230
0.4301	0.303	0.169	3.76E-21	0.379	0.159	0.220	0.134
0.5399	0.303	0.084	1.15E-17	0.392	0.082	0.310	0.219
0.5000	0.303	0.099	1.96E-17	0.363	0.088	0.275	0.204
0.6551	0.303	0.029	3.96E-17	0.432	0.044	0.387	0.274
0.5266	0.303	0.125	1.19E-14	0.401	0.118	0.282	0.178
0.5380	0.303	0.119	2.35E-14	0.395	0.108	0.286	0.184
0.3984	0.303	0.181	2.70E-09	0.375	0.183	0.191	0.122
0.4512	0.303	0.167	3.00E-08	0.394	0.166	0.228	0.136
0.4243	0.303	0.178	2.20E-07	0.379	0.175	0.204	0.125
0.2585	0.303	0.185	5.20E-07	0.336	0.190	0.147	0.118
0.4693	0.303	0.149	2.56E-06	0.388	0.138	0.250	0.154
0.4175	0.303	0.137	2.32E-05	0.362	0.130	0.232	0.166
-0.6755	0.303	0.459	2.62E-04	0.354	0.736	0.382	-0.156

0.2560	0.303	0.234	8.09E-03	0.338	0.227	0.111	0.069
-1.1616	0.299	0.730	2.00E-54	0.473	1.586	1.114	-0.431
0.7912	0.299	0.000	8.67E-45	0.444	0.001	0.443	0.299
0.8861	0.299	0.001	7.89E-42	0.471	0.002	0.469	0.298
0.7879	0.299	0.011	4.16E-28	0.431	0.012	0.419	0.288
0.5874	0.299	0.060	3.96E-27	0.367	0.056	0.311	0.239
0.8202	0.299	0.026	1.22E-26	0.464	0.035	0.429	0.273
0.6256	0.299	0.069	2.83E-24	0.403	0.063	0.340	0.230
0.7024	0.299	0.028	5.31E-23	0.398	0.026	0.372	0.271
-0.5389	0.299	0.564	2.98E-09	0.379	0.846	0.468	-0.265
0.3718	0.299	0.141	8.38E-09	0.368	0.136	0.232	0.158
0.5478	0.299	0.066	9.35E-08	0.459	0.108	0.352	0.233
0.4663	0.299	0.113	9.50E-07	0.370	0.105	0.265	0.186
0.4747	0.299	0.132	3.29E-06	0.389	0.131	0.259	0.167
0.4688	0.299	0.115	4.91E-06	0.355	0.107	0.248	0.184
0.3784	0.299	0.172	4.81E-05	0.350	0.168	0.182	0.127
0.2692	0.299	0.215	3.93E-04	0.367	0.221	0.146	0.084
0.3022	0.299	0.217	6.75E-04	0.345	0.205	0.140	0.082
0.3383	0.299	0.200	1.73E-03	0.348	0.196	0.152	0.099
0.9096	0.294	0.001	7.02E-43	0.465	0.001	0.464	0.293
0.6921	0.294	0.000	1.36E-38	0.384	0.000	0.383	0.294
0.6498	0.294	0.002	1.71E-34	0.382	0.002	0.380	0.292
0.5173	0.294	0.145	2.97E-24	0.388	0.139	0.250	0.149
0.6771	0.294	0.039	2.64E-21	0.401	0.035	0.366	0.255
0.5438	0.294	0.067	3.07E-20	0.362	0.059	0.303	0.227
0.4551	0.294	0.121	2.51E-18	0.341	0.113	0.227	0.173
-0.6209	0.294	0.562	2.32E-11	0.316	0.801	0.485	-0.268
0.3914	0.294	0.139	1.30E-10	0.326	0.129	0.197	0.155
0.3590	0.294	0.160	2.69E-08	0.328	0.154	0.174	0.134
0.3269	0.294	0.200	1.42E-06	0.361	0.193	0.168	0.094
0.3319	0.294	0.156	2.95E-06	0.340	0.166	0.173	0.138
0.4034	0.294	0.116	8.02E-04	0.453	0.195	0.258	0.178
0.3779	0.294	0.172	2.97E-03	0.340	0.162	0.178	0.122
0.7302	0.289	0.008	2.84E-36	0.392	0.007	0.384	0.281
0.7795	0.289	0.001	2.62E-35	0.419	0.001	0.419	0.288
0.6022	0.289	0.065	2.72E-23	0.388	0.063	0.325	0.224
0.5917	0.289	0.079	8.23E-17	0.386	0.074	0.311	0.210
0.4669	0.289	0.133	1.11E-16	0.348	0.127	0.221	0.156
0.4705	0.289	0.117	2.73E-16	0.348	0.105	0.242	0.172
0.4122	0.289	0.156	6.93E-11	0.335	0.146	0.189	0.133
0.3391	0.289	0.150	3.67E-05	0.326	0.146	0.180	0.139
0.3619	0.289	0.168	5.00E-05	0.322	0.165	0.157	0.121
0.3240	0.289	0.165	3.99E-04	0.315	0.163	0.152	0.124
0.3310	0.289	0.173	7.23E-04	0.340	0.172	0.168	0.116
0.8064	0.284	0.002	2.56E-41	0.433	0.002	0.431	0.282
0.6065	0.284	0.058	2.76E-26	0.383	0.052	0.331	0.226
-1.3387	0.284	0.468	1.62E-22	0.393	1.119	0.726	-0.184

0.5418	0.284	0.061	2.60E-21	0.358	0.061	0.298	0.223
0.5027	0.284	0.128	3.04E-20	0.370	0.115	0.255	0.156
0.5506	0.284	0.107	2.41E-17	0.389	0.095	0.294	0.177
0.5595	0.284	0.020	5.07E-16	0.389	0.039	0.351	0.264
0.4804	0.284	0.137	6.46E-14	0.361	0.132	0.229	0.147
0.5138	0.284	0.089	7.66E-14	0.342	0.076	0.266	0.195
0.4676	0.284	0.126	6.45E-13	0.351	0.117	0.233	0.158
0.4131	0.284	0.124	3.79E-12	0.345	0.121	0.224	0.160
0.5267	0.284	0.095	9.77E-12	0.341	0.085	0.256	0.189
0.6321	0.284	0.084	2.07E-11	0.377	0.080	0.297	0.200
0.4797	0.284	0.074	4.10E-11	0.335	0.070	0.264	0.210
0.3472	0.284	0.103	1.39E-08	0.309	0.102	0.207	0.181
0.3865	0.284	0.114	6.67E-08	0.301	0.112	0.189	0.170
0.3399	0.284	0.077	5.24E-06	0.322	0.081	0.241	0.207
0.3716	0.284	0.114	5.37E-05	0.322	0.108	0.214	0.170
0.3759	0.284	0.171	2.37E-04	0.339	0.158	0.181	0.113
0.3142	0.284	0.179	5.14E-03	0.316	0.170	0.146	0.105
0.6955	0.279	0.002	1.99E-33	0.388	0.003	0.385	0.277
0.6390	0.279	0.021	9.49E-28	0.376	0.019	0.357	0.258
0.6555	0.279	0.019	5.75E-21	0.378	0.020	0.358	0.260
0.6289	0.279	0.075	1.14E-18	0.367	0.064	0.303	0.204
0.3142	0.279	0.141	8.39E-15	0.285	0.132	0.153	0.138
0.4398	0.279	0.098	1.28E-11	0.337	0.100	0.237	0.181
0.5003	0.279	0.094	2.76E-11	0.335	0.083	0.252	0.185
0.4564	0.279	0.098	1.08E-10	0.342	0.095	0.247	0.181
0.4724	0.279	0.092	1.58E-10	0.345	0.084	0.260	0.187
0.3941	0.279	0.108	2.44E-10	0.304	0.095	0.208	0.171
0.4652	0.279	0.103	3.89E-10	0.345	0.095	0.250	0.176
0.4435	0.279	0.113	7.20E-09	0.323	0.101	0.222	0.166
0.2916	0.279	0.261	1.13E-07	0.368	0.258	0.110	0.018
0.4006	0.279	0.116	6.40E-07	0.340	0.117	0.224	0.163
0.3059	0.279	0.191	1.04E-05	0.332	0.184	0.148	0.088
0.3214	0.279	0.211	1.47E-04	0.318	0.202	0.116	0.068
0.3739	0.279	0.186	3.68E-04	0.333	0.177	0.156	0.093
0.3685	0.279	0.152	1.88E-03	0.314	0.144	0.170	0.127
-0.6702	0.279	0.341	8.21E-03	0.379	0.710	0.331	-0.062
0.8369	0.274	0.002	1.65E-39	0.415	0.002	0.413	0.272
0.7242	0.274	0.022	6.39E-26	0.390	0.021	0.370	0.252
0.5835	0.274	0.029	8.81E-22	0.350	0.035	0.315	0.245
0.6199	0.274	0.016	2.95E-21	0.345	0.017	0.328	0.258
0.4760	0.274	0.084	2.25E-17	0.302	0.073	0.229	0.190
0.4192	0.274	0.145	9.00E-16	0.336	0.144	0.192	0.129
0.3759	0.274	0.101	1.25E-11	0.295	0.094	0.201	0.173
0.4042	0.274	0.078	1.34E-09	0.310	0.073	0.237	0.196
-0.8088	0.274	0.521	8.91E-09	0.293	0.794	0.501	-0.247
0.3799	0.274	0.121	1.05E-07	0.335	0.120	0.215	0.153
0.3565	0.274	0.135	8.19E-06	0.311	0.127	0.184	0.139

0.3354	0.274	0.140	3.78E-05	0.315	0.142	0.173	0.134
0.3174	0.274	0.128	5.69E-05	0.297	0.121	0.176	0.146
0.2998	0.274	0.176	1.91E-04	0.319	0.166	0.153	0.098
-0.4312	0.274	0.484	2.94E-04	0.360	0.675	0.315	-0.210
-0.6861	0.274	0.423	3.14E-04	0.331	0.709	0.378	-0.149
0.7840	0.269	0.000	4.15E-44	0.407	0.000	0.407	0.269
0.6741	0.269	0.025	2.36E-36	0.366	0.023	0.342	0.244
0.6083	0.269	0.000	9.07E-34	0.329	0.000	0.329	0.269
0.9033	0.269	0.009	2.26E-31	0.428	0.007	0.421	0.260
0.6078	0.269	0.003	2.01E-30	0.323	0.003	0.320	0.266
0.7041	0.269	0.009	9.64E-28	0.383	0.008	0.375	0.260
0.6323	0.269	0.029	1.17E-23	0.372	0.032	0.340	0.240
0.5299	0.269	0.091	8.48E-16	0.343	0.087	0.256	0.178
0.4633	0.269	0.145	2.52E-15	0.329	0.132	0.197	0.124
0.5437	0.269	0.056	4.48E-14	0.312	0.049	0.263	0.213
0.4175	0.269	0.153	6.22E-12	0.353	0.141	0.212	0.116
0.4110	0.269	0.068	1.15E-11	0.320	0.074	0.246	0.201
0.4571	0.269	0.079	4.01E-10	0.303	0.070	0.232	0.190
0.4022	0.269	0.085	1.76E-08	0.297	0.077	0.220	0.184
0.4248	0.269	0.079	5.78E-08	0.297	0.069	0.227	0.190
0.3558	0.269	0.127	1.72E-07	0.302	0.114	0.188	0.142
0.7951	0.269	0.053	1.91E-07	0.447	0.056	0.391	0.216
0.3002	0.269	0.129	1.57E-04	0.284	0.121	0.164	0.140
0.3032	0.269	0.171	6.56E-04	0.310	0.160	0.150	0.098
-0.4163	0.269	0.482	2.06E-03	0.305	0.636	0.331	-0.213
0.2630	0.269	0.148	3.25E-03	0.275	0.135	0.140	0.121
-0.3973	0.269	0.415	6.99E-03	0.294	0.558	0.264	-0.146
0.8799	0.264	0.001	5.63E-41	0.430	0.001	0.429	0.263
0.6779	0.264	0.001	1.93E-35	0.349	0.001	0.348	0.263
0.6006	0.264	0.001	9.82E-33	0.325	0.001	0.324	0.263
-1.4594	0.264	0.644	4.89E-29	0.311	1.262	0.951	-0.380
0.5713	0.264	0.025	3.00E-27	0.324	0.021	0.303	0.239
0.5992	0.264	0.052	4.73E-24	0.358	0.047	0.310	0.212
0.4283	0.264	0.080	1.22E-18	0.313	0.079	0.234	0.184
0.6256	0.264	0.036	2.10E-17	0.346	0.034	0.312	0.228
0.4620	0.264	0.038	2.92E-17	0.314	0.047	0.268	0.226
0.4398	0.264	0.070	1.33E-16	0.292	0.062	0.230	0.194
0.4905	0.264	0.083	1.22E-13	0.318	0.075	0.243	0.181
0.4797	0.264	0.064	2.03E-12	0.293	0.057	0.236	0.200
0.4209	0.264	0.124	8.92E-12	0.313	0.117	0.196	0.140
0.4579	0.264	0.042	1.39E-10	0.331	0.065	0.266	0.222
0.5945	0.264	0.065	1.13E-09	0.333	0.060	0.273	0.199
0.5006	0.264	0.038	6.68E-09	0.344	0.056	0.289	0.226
0.4379	0.264	0.086	1.99E-08	0.311	0.083	0.227	0.178
0.4044	0.264	0.099	5.11E-08	0.330	0.113	0.218	0.165
0.3794	0.264	0.094	5.42E-08	0.274	0.087	0.188	0.170
0.3535	0.264	0.151	4.64E-07	0.307	0.144	0.163	0.113

0.4405	0.264	0.067	1.21E-06	0.292	0.056	0.235	0.197
0.3982	0.264	0.141	7.79E-04	0.318	0.129	0.189	0.123
-0.3850	0.264	0.476	7.87E-04	0.284	0.608	0.323	-0.212
0.2638	0.264	0.156	2.09E-03	0.281	0.147	0.135	0.108
1.0762	0.259	0.001	4.24E-39	0.492	0.002	0.490	0.258
0.4658	0.259	0.050	1.04E-25	0.291	0.044	0.247	0.209
0.5735	0.259	0.042	1.15E-22	0.323	0.037	0.285	0.217
0.3397	0.259	0.033	1.00E-19	0.383	0.085	0.298	0.226
0.7391	0.259	0.026	3.88E-19	0.379	0.026	0.353	0.233
0.5924	0.259	0.044	8.89E-18	0.324	0.037	0.287	0.215
0.4299	0.259	0.082	5.46E-13	0.320	0.085	0.235	0.177
0.5697	0.259	0.036	9.91E-13	0.347	0.041	0.306	0.223
0.3547	0.259	0.144	4.16E-07	0.303	0.137	0.166	0.115
0.4769	0.259	0.082	1.37E-06	0.328	0.086	0.242	0.177
0.3374	0.259	0.138	2.16E-06	0.304	0.132	0.172	0.121
-0.6799	0.259	0.537	8.00E-06	0.293	0.797	0.505	-0.278
0.3795	0.259	0.101	2.10E-04	0.290	0.091	0.199	0.158
0.2578	0.259	0.165	2.82E-04	0.300	0.167	0.132	0.094
0.3665	0.259	0.121	3.60E-04	0.310	0.118	0.192	0.138
0.3091	0.259	0.139	5.18E-03	0.295	0.134	0.161	0.120
0.3259	0.259	0.127	9.78E-03	0.289	0.119	0.170	0.132
0.6632	0.254	0.000	2.87E-34	0.327	0.001	0.327	0.254
0.6597	0.254	0.001	2.06E-33	0.345	0.002	0.344	0.253
0.6497	0.254	0.000	6.95E-33	0.338	0.000	0.337	0.254
0.6464	0.254	0.035	1.36E-31	0.349	0.030	0.319	0.219
0.5163	0.254	0.001	1.78E-28	0.297	0.001	0.295	0.253
0.5939	0.254	0.001	2.94E-25	0.325	0.002	0.323	0.253
0.4555	0.254	0.070	7.10E-22	0.293	0.063	0.229	0.184
0.4629	0.254	0.077	1.47E-21	0.294	0.071	0.223	0.177
0.4830	0.254	0.013	8.42E-21	0.281	0.012	0.268	0.241
0.4049	0.254	0.050	1.42E-15	0.282	0.051	0.230	0.204
0.4226	0.254	0.039	6.04E-13	0.286	0.038	0.248	0.215
0.5050	0.254	0.069	3.64E-12	0.297	0.060	0.237	0.185
0.3270	0.254	0.138	4.34E-12	0.274	0.128	0.146	0.116
0.4764	0.254	0.065	4.70E-12	0.305	0.061	0.244	0.189
0.5345	0.254	0.052	3.02E-11	0.330	0.053	0.277	0.202
0.4578	0.254	0.068	9.81E-11	0.299	0.062	0.236	0.186
0.3258	0.254	0.125	1.81E-07	0.277	0.116	0.160	0.129
-0.4764	0.254	0.497	1.07E-06	0.295	0.670	0.376	-0.243
0.3636	0.254	0.084	1.38E-06	0.285	0.081	0.204	0.170
0.3451	0.254	0.112	1.85E-06	0.292	0.110	0.182	0.142
-0.5361	0.254	0.483	6.03E-06	0.247	0.642	0.395	-0.229
0.3833	0.254	0.076	9.76E-06	0.275	0.072	0.203	0.178
0.3367	0.254	0.115	1.96E-05	0.278	0.107	0.171	0.139
0.2746	0.254	0.151	4.05E-05	0.256	0.138	0.118	0.103
0.3543	0.254	0.138	1.83E-04	0.301	0.132	0.169	0.116
0.2716	0.254	0.093	9.94E-04	0.282	0.108	0.174	0.161

0.5862	0.249	0.000	1.82E-30	0.306	0.000	0.306	0.249
0.6061	0.249	0.005	6.06E-30	0.311	0.005	0.306	0.244
0.4985	0.249	0.019	2.73E-24	0.274	0.016	0.258	0.230
-1.8330	0.249	0.487	5.89E-22	0.477	1.840	1.364	-0.238
0.5021	0.249	0.029	1.28E-17	0.306	0.027	0.279	0.220
0.5113	0.249	0.083	8.99E-13	0.313	0.079	0.234	0.166
0.3622	0.249	0.092	5.76E-12	0.274	0.082	0.191	0.157
0.3747	0.249	0.109	2.02E-09	0.292	0.103	0.189	0.140
0.2730	0.249	0.139	6.55E-04	0.282	0.124	0.158	0.110
0.2954	0.249	0.139	1.66E-03	0.275	0.127	0.148	0.110
0.2571	0.249	0.168	8.16E-03	0.278	0.169	0.109	0.081
0.6246	0.244	0.001	2.87E-31	0.318	0.001	0.316	0.243
0.7684	0.244	0.001	6.80E-30	0.338	0.001	0.337	0.243
0.5194	0.244	0.002	3.51E-24	0.274	0.002	0.272	0.242
0.5289	0.244	0.023	1.49E-23	0.301	0.025	0.276	0.221
0.5591	0.244	0.002	3.74E-22	0.285	0.003	0.283	0.242
-0.7026	0.244	0.630	1.59E-19	0.312	0.923	0.611	-0.386
0.5076	0.244	0.021	4.29E-18	0.292	0.023	0.269	0.223
0.4232	0.244	0.053	7.39E-11	0.266	0.047	0.219	0.191
0.4333	0.244	0.093	4.92E-09	0.288	0.083	0.205	0.151
0.4258	0.244	0.074	1.43E-08	0.277	0.065	0.212	0.170
0.4057	0.244	0.036	1.99E-08	0.279	0.047	0.232	0.208
0.3596	0.244	0.083	1.24E-06	0.257	0.074	0.183	0.161
0.3776	0.244	0.113	3.66E-06	0.273	0.101	0.172	0.131
0.3859	0.244	0.087	3.29E-05	0.262	0.079	0.183	0.157
0.3755	0.244	0.113	3.01E-04	0.266	0.102	0.164	0.131
0.2567	0.244	0.196	9.69E-04	0.280	0.193	0.087	0.048
0.2920	0.244	0.145	2.20E-03	0.275	0.139	0.136	0.099
-0.4835	0.244	0.480	8.29E-03	0.241	0.623	0.382	-0.236
0.6051	0.239	0.005	1.82E-26	0.297	0.004	0.293	0.234
0.4931	0.239	0.002	9.27E-23	0.266	0.002	0.263	0.237
0.5439	0.239	0.008	3.81E-22	0.280	0.007	0.273	0.231
0.3886	0.239	0.076	3.42E-14	0.278	0.074	0.204	0.163
0.3699	0.239	0.094	6.57E-14	0.304	0.140	0.164	0.145
0.4277	0.239	0.029	1.56E-12	0.267	0.029	0.238	0.210
0.3078	0.239	0.089	1.29E-07	0.253	0.089	0.164	0.150
0.3376	0.239	0.139	1.76E-07	0.280	0.137	0.143	0.100
0.3550	0.239	0.121	3.79E-07	0.273	0.112	0.161	0.118
-0.7641	0.239	0.380	3.10E-06	0.262	0.666	0.404	-0.141
0.2954	0.239	0.144	8.80E-06	0.244	0.133	0.111	0.095
0.4007	0.239	0.085	1.06E-05	0.271	0.080	0.192	0.154
0.2599	0.239	0.131	2.82E-04	0.256	0.124	0.132	0.108
0.3303	0.239	0.109	7.22E-03	0.257	0.102	0.155	0.130
-1.5311	0.234	0.756	1.19E-59	0.373	1.743	1.370	-0.522
0.5782	0.234	0.001	2.35E-32	0.296	0.001	0.295	0.233
0.6097	0.234	0.001	1.31E-29	0.298	0.001	0.298	0.233
0.6504	0.234	0.001	2.67E-29	0.313	0.001	0.312	0.233

0.6310	0.234	0.000	3.27E-27	0.337	0.000	0.337	0.234
0.5446	0.234	0.003	4.40E-21	0.287	0.004	0.283	0.231
0.5091	0.234	0.040	1.92E-20	0.271	0.037	0.235	0.194
0.5091	0.234	0.021	3.21E-20	0.261	0.020	0.241	0.213
0.5266	0.234	0.013	1.93E-16	0.289	0.014	0.275	0.221
0.5973	0.234	0.025	1.93E-16	0.321	0.023	0.298	0.209
0.5356	0.234	0.042	1.20E-15	0.294	0.036	0.257	0.192
-0.5910	0.234	0.591	3.31E-15	0.296	0.847	0.550	-0.357
0.3747	0.234	0.051	4.04E-14	0.239	0.046	0.193	0.183
0.5791	0.234	0.020	5.63E-14	0.319	0.017	0.302	0.214
0.2846	0.234	0.126	2.65E-09	0.256	0.119	0.137	0.108
-0.6906	0.234	0.361	6.05E-09	0.274	0.612	0.337	-0.127
0.4486	0.234	0.042	1.46E-08	0.261	0.037	0.224	0.192
-0.5899	0.234	0.477	1.75E-07	0.229	0.629	0.400	-0.243
0.2707	0.234	0.091	2.09E-06	0.233	0.082	0.151	0.143
0.2744	0.234	0.140	7.41E-06	0.245	0.131	0.114	0.094
0.3113	0.234	0.165	2.86E-05	0.278	0.153	0.125	0.069
0.2872	0.234	0.077	5.24E-05	0.305	0.101	0.204	0.157
0.2977	0.234	0.101	1.21E-03	0.277	0.116	0.161	0.133
0.4057	0.234	0.098	2.41E-03	0.273	0.091	0.182	0.136
0.4842	0.229	0.012	3.04E-22	0.259	0.011	0.248	0.217
0.5461	0.229	0.006	1.23E-21	0.288	0.005	0.283	0.223
0.5231	0.229	0.003	8.65E-20	0.273	0.003	0.270	0.226
0.5209	0.229	0.004	9.26E-20	0.279	0.005	0.274	0.225
0.4141	0.229	0.014	1.29E-19	0.232	0.012	0.220	0.215
0.4948	0.229	0.016	9.15E-18	0.245	0.013	0.233	0.213
0.4319	0.229	0.064	4.86E-17	0.255	0.056	0.200	0.165
0.5088	0.229	0.029	3.51E-15	0.276	0.025	0.251	0.200
0.3859	0.229	0.015	3.08E-14	0.227	0.015	0.212	0.214
0.3806	0.229	0.049	3.36E-14	0.243	0.047	0.196	0.180
0.4356	0.229	0.031	3.53E-14	0.252	0.028	0.224	0.198
0.4502	0.229	0.037	2.54E-13	0.253	0.034	0.219	0.192
0.4553	0.229	0.074	1.08E-09	0.255	0.068	0.186	0.155
0.3168	0.229	0.079	1.43E-09	0.240	0.073	0.167	0.150
0.3177	0.229	0.109	2.26E-07	0.257	0.103	0.154	0.120
0.3122	0.229	0.085	5.45E-07	0.252	0.078	0.174	0.144
0.3083	0.229	0.078	9.75E-07	0.240	0.074	0.166	0.151
0.3828	0.229	0.086	4.43E-06	0.241	0.076	0.165	0.143
0.3946	0.229	0.096	5.27E-06	0.253	0.087	0.166	0.133
-0.5502	0.229	0.447	1.36E-04	0.223	0.582	0.359	-0.218
0.2744	0.229	0.158	1.85E-04	0.251	0.144	0.107	0.071
0.2513	0.229	0.114	9.40E-04	0.231	0.107	0.123	0.115
0.2559	0.229	0.184	1.49E-03	0.261	0.181	0.080	0.045
0.2794	0.229	0.109	8.28E-03	0.237	0.101	0.136	0.120
0.5191	0.224	0.000	1.73E-31	0.250	0.001	0.250	0.224
0.5333	0.224	0.000	3.05E-30	0.271	0.000	0.271	0.224
-1.4051	0.224	0.320	1.39E-25	0.552	1.377	0.825	-0.096

0.4748	0.224	0.019	8.51E-22	0.256	0.016	0.240	0.205
0.4718	0.224	0.002	3.80E-18	0.240	0.001	0.238	0.222
0.4571	0.224	0.006	1.85E-16	0.241	0.006	0.234	0.218
0.4011	0.224	0.052	3.85E-15	0.231	0.050	0.182	0.172
0.3702	0.224	0.058	1.10E-12	0.243	0.054	0.189	0.166
0.4161	0.224	0.053	1.50E-11	0.258	0.048	0.210	0.171
0.3228	0.224	0.054	1.36E-10	0.219	0.048	0.171	0.170
0.3913	0.224	0.042	1.14E-07	0.228	0.036	0.191	0.182
0.2897	0.224	0.104	2.08E-07	0.268	0.123	0.145	0.120
0.3023	0.224	0.078	2.94E-07	0.226	0.072	0.154	0.146
0.3139	0.224	0.138	3.22E-07	0.251	0.124	0.128	0.086
0.3256	0.224	0.054	3.84E-07	0.254	0.068	0.186	0.170
0.2974	0.224	0.098	1.72E-06	0.236	0.092	0.144	0.126
0.2530	0.224	0.080	4.24E-05	0.217	0.070	0.147	0.144
0.3234	0.224	0.099	1.26E-04	0.243	0.093	0.150	0.125
-0.3683	0.224	0.378	1.41E-03	0.219	0.472	0.252	-0.154
0.4175	0.224	0.089	3.19E-03	0.278	0.081	0.197	0.135
-0.4368	0.224	0.390	3.42E-03	0.220	0.488	0.268	-0.166
0.3319	0.224	0.094	7.11E-03	0.231	0.080	0.150	0.130
-1.1252	0.219	0.788	3.56E-65	0.398	1.586	1.187	-0.569
0.5541	0.219	0.001	1.72E-24	0.273	0.001	0.272	0.218
0.4547	0.219	0.002	3.27E-19	0.246	0.001	0.245	0.217
-1.3702	0.219	0.408	2.63E-17	0.323	0.935	0.612	-0.189
0.3404	0.219	0.044	9.40E-16	0.217	0.038	0.179	0.175
0.3885	0.219	0.086	9.62E-15	0.249	0.075	0.174	0.133
0.3965	0.219	0.070	1.93E-13	0.261	0.064	0.197	0.149
0.3803	0.219	0.021	2.64E-12	0.209	0.019	0.190	0.198
-0.7134	0.219	0.487	3.05E-10	0.197	0.641	0.444	-0.268
0.3632	0.219	0.045	1.79E-09	0.239	0.042	0.197	0.174
0.4376	0.219	0.011	6.91E-09	0.247	0.016	0.232	0.208
0.4564	0.219	0.033	1.15E-08	0.278	0.044	0.234	0.186
-0.6053	0.219	0.453	2.45E-08	0.208	0.609	0.401	-0.234
0.3539	0.219	0.110	1.25E-07	0.241	0.099	0.142	0.109
-0.6851	0.219	0.359	1.75E-06	0.246	0.582	0.335	-0.140
0.3612	0.219	0.146	1.76E-06	0.255	0.133	0.122	0.073
0.3016	0.219	0.096	1.98E-06	0.234	0.092	0.142	0.123
0.3684	0.219	0.038	7.48E-06	0.251	0.051	0.200	0.181
0.3008	0.219	0.080	9.99E-05	0.224	0.076	0.148	0.139
-0.6967	0.219	0.324	2.04E-03	0.247	0.577	0.330	-0.105
0.3607	0.219	0.050	7.75E-03	0.246	0.045	0.201	0.169
-1.2579	0.214	0.734	7.97E-48	0.321	1.319	0.998	-0.520
0.5015	0.214	0.001	4.41E-28	0.250	0.000	0.250	0.213
0.4879	0.214	0.000	2.83E-27	0.240	0.000	0.240	0.214
0.4619	0.214	0.000	2.16E-23	0.235	0.001	0.235	0.214
0.5167	0.214	0.004	1.10E-19	0.256	0.004	0.252	0.210
0.4406	0.214	0.023	1.00E-18	0.242	0.021	0.221	0.191
0.4955	0.214	0.010	3.71E-17	0.266	0.010	0.256	0.204

0.4867	0.214	0.020	9.65E-17	0.239	0.017	0.222	0.194
0.5020	0.214	0.004	2.07E-16	0.240	0.004	0.235	0.210
0.5080	0.214	0.004	2.96E-16	0.241	0.004	0.237	0.210
0.3960	0.214	0.001	8.15E-15	0.222	0.001	0.221	0.213
0.5647	0.214	0.011	1.85E-12	0.276	0.014	0.263	0.203
0.3576	0.214	0.099	1.31E-11	0.243	0.091	0.153	0.115
-0.6065	0.214	0.369	4.58E-08	0.239	0.582	0.343	-0.155
0.2967	0.214	0.097	5.23E-06	0.226	0.086	0.139	0.117
0.6524	0.214	0.046	5.94E-06	0.323	0.059	0.265	0.168
0.3438	0.214	0.081	9.73E-06	0.249	0.081	0.168	0.133
0.4099	0.214	0.061	1.12E-04	0.236	0.053	0.183	0.153
0.2737	0.214	0.084	2.53E-04	0.223	0.077	0.145	0.130
0.3108	0.214	0.104	7.56E-04	0.226	0.097	0.129	0.110
0.2600	0.214	0.067	1.90E-03	0.200	0.058	0.142	0.147
0.2678	0.214	0.094	4.73E-03	0.234	0.097	0.137	0.120
0.5459	0.209	0.001	3.44E-25	0.250	0.001	0.249	0.208
-1.4423	0.209	0.334	2.57E-24	0.286	0.881	0.595	-0.125
-1.2485	0.209	0.306	1.32E-15	0.227	0.690	0.463	-0.097
0.3970	0.209	0.014	2.97E-15	0.209	0.012	0.197	0.195
0.4099	0.209	0.007	5.01E-15	0.219	0.005	0.214	0.202
0.8157	0.209	0.017	5.14E-13	0.292	0.018	0.274	0.192
0.3990	0.209	0.073	5.94E-12	0.242	0.064	0.178	0.136
0.3504	0.209	0.063	6.15E-10	0.217	0.055	0.162	0.146
-0.6198	0.209	0.479	2.92E-09	0.194	0.627	0.433	-0.270
-1.0346	0.209	0.272	4.57E-08	0.604	1.479	0.875	-0.063
0.3819	0.209	0.044	5.80E-08	0.223	0.040	0.183	0.165
0.3077	0.209	0.099	3.65E-07	0.216	0.093	0.123	0.110
0.3835	0.209	0.025	1.14E-06	0.241	0.033	0.208	0.184
0.4635	0.209	0.036	5.70E-06	0.253	0.034	0.219	0.173
-0.6821	0.209	0.485	1.14E-05	0.230	0.718	0.488	-0.276
-0.4821	0.209	0.402	1.02E-04	0.179	0.472	0.293	-0.193
0.2927	0.209	0.098	3.74E-04	0.216	0.087	0.129	0.111
0.3014	0.209	0.079	7.00E-04	0.219	0.069	0.151	0.130
-0.9259	0.204	0.722	5.65E-53	0.355	1.279	0.923	-0.518
-1.2809	0.204	0.679	1.25E-51	0.471	1.725	1.254	-0.475
-1.0185	0.204	0.716	8.66E-47	0.392	1.414	1.021	-0.512
-0.9651	0.204	0.692	1.05E-45	0.267	1.103	0.836	-0.488
-1.1921	0.204	0.645	3.81E-45	0.336	1.317	0.981	-0.441
1.1526	0.204	0.002	4.09E-28	0.409	0.003	0.406	0.202
0.5258	0.204	0.001	3.59E-21	0.241	0.001	0.241	0.203
0.4680	0.204	0.001	5.68E-21	0.227	0.001	0.226	0.203
-0.7749	0.204	0.579	9.99E-19	0.294	0.934	0.640	-0.375
0.3632	0.204	0.007	3.75E-18	0.199	0.006	0.193	0.197
0.3743	0.204	0.021	1.92E-17	0.209	0.020	0.190	0.183
0.4748	0.204	0.012	3.33E-16	0.232	0.013	0.220	0.192
0.4419	0.204	0.001	3.30E-13	0.232	0.001	0.231	0.203
-0.7087	0.204	0.405	1.23E-10	0.194	0.625	0.430	-0.201

0.3686	0.204	0.027	3.04E-09	0.215	0.032	0.184	0.177
0.3180	0.204	0.048	1.76E-07	0.216	0.045	0.171	0.156
-0.5773	0.204	0.397	1.62E-06	0.188	0.541	0.353	-0.193
0.3792	0.204	0.013	3.36E-06	0.221	0.020	0.201	0.191
-0.3341	0.204	0.339	4.03E-06	0.233	0.423	0.190	-0.135
-0.7097	0.204	0.290	4.69E-06	0.230	0.527	0.297	-0.086
0.2808	0.204	0.069	3.42E-05	0.208	0.067	0.141	0.135
-0.5401	0.204	0.454	1.64E-04	0.193	0.569	0.375	-0.250
-0.4154	0.204	0.414	8.62E-04	0.193	0.485	0.292	-0.210
0.2697	0.204	0.086	1.10E-03	0.214	0.077	0.137	0.118
-0.2604	0.204	0.187	2.83E-03	0.242	0.319	0.077	0.017
0.2602	0.204	0.092	3.01E-03	0.203	0.083	0.120	0.112
-0.6158	0.204	0.285	3.64E-03	0.202	0.474	0.272	-0.081
-1.1689	0.199	0.656	1.28E-41	0.320	1.284	0.964	-0.457
0.3376	0.199	0.098	3.84E-19	0.244	0.079	0.165	0.101
0.4356	0.199	0.000	7.31E-18	0.217	0.000	0.217	0.199
0.3819	0.199	0.056	1.00E-16	0.229	0.049	0.180	0.143
0.4052	0.199	0.002	3.05E-15	0.208	0.002	0.206	0.197
0.3482	0.199	0.029	3.11E-14	0.201	0.025	0.176	0.170
0.4469	0.199	0.056	3.04E-13	0.232	0.048	0.183	0.143
0.3919	0.199	0.001	5.95E-13	0.213	0.003	0.209	0.198
-0.7094	0.199	0.443	9.76E-13	0.188	0.643	0.455	-0.244
-0.4994	0.199	0.488	1.46E-11	0.255	0.648	0.392	-0.289
-0.9815	0.199	0.418	5.45E-11	0.200	0.710	0.510	-0.219
0.2604	0.199	0.107	3.73E-08	0.213	0.097	0.116	0.092
-0.8234	0.199	0.336	7.62E-08	0.229	0.600	0.371	-0.137
0.2993	0.199	0.047	1.06E-07	0.193	0.042	0.151	0.152
0.2636	0.199	0.059	1.93E-07	0.192	0.052	0.140	0.140
0.3136	0.199	0.052	7.56E-07	0.217	0.048	0.169	0.147
-0.6483	0.199	0.302	7.63E-06	0.264	0.567	0.303	-0.103
-0.2870	0.199	0.323	2.32E-05	0.247	0.458	0.211	-0.124
-1.1439	0.194	0.583	1.57E-31	0.303	1.091	0.789	-0.389
-2.1794	0.194	0.555	1.93E-31	0.210	1.229	1.018	-0.361
0.3861	0.194	0.004	7.05E-19	0.208	0.003	0.204	0.190
0.5188	0.194	0.008	4.34E-16	0.253	0.009	0.244	0.186
0.4193	0.194	0.003	8.02E-16	0.209	0.003	0.206	0.191
-1.0143	0.194	0.521	1.52E-15	0.534	1.429	0.895	-0.327
0.3613	0.194	0.012	1.86E-15	0.187	0.010	0.178	0.182
0.4936	0.194	0.013	1.88E-12	0.260	0.017	0.243	0.181
-0.6376	0.194	0.368	3.27E-09	0.205	0.556	0.351	-0.174
0.4083	0.194	0.022	4.60E-08	0.219	0.022	0.197	0.172
-0.4247	0.194	0.418	1.59E-07	0.199	0.512	0.313	-0.224
-0.5896	0.194	0.454	2.52E-07	0.196	0.610	0.413	-0.260
0.3124	0.194	0.078	1.96E-05	0.191	0.069	0.122	0.116
0.4603	0.194	0.049	2.90E-05	0.223	0.046	0.177	0.145
-0.4840	0.194	0.307	1.16E-04	0.216	0.470	0.254	-0.113
0.2722	0.194	0.093	4.49E-03	0.196	0.086	0.110	0.101

-1.2534	0.189	0.689	2.26E-62	0.405	1.651	1.245	-0.500
-1.0242	0.189	0.660	2.04E-43	0.509	1.712	1.203	-0.471
-1.0990	0.189	0.616	6.19E-42	0.317	1.229	0.912	-0.427
-0.9770	0.189	0.547	5.89E-23	0.235	0.900	0.666	-0.358
-0.8057	0.189	0.565	4.63E-21	0.248	0.869	0.621	-0.376
0.4107	0.189	0.000	1.97E-15	0.210	0.000	0.209	0.189
0.3372	0.189	0.001	7.04E-15	0.178	0.001	0.177	0.188
0.3621	0.189	0.006	7.20E-13	0.199	0.006	0.193	0.183
0.3120	0.189	0.000	3.49E-12	0.181	0.000	0.181	0.189
0.3523	0.189	0.003	1.42E-11	0.180	0.002	0.177	0.186
-0.6792	0.189	0.263	1.40E-06	0.176	0.453	0.277	-0.074
0.3072	0.189	0.007	1.79E-05	0.181	0.010	0.171	0.182
-0.3174	0.189	0.363	3.42E-04	0.195	0.421	0.226	-0.174
0.2680	0.189	0.056	2.07E-03	0.170	0.055	0.115	0.133
0.2752	0.189	0.039	2.25E-03	0.186	0.037	0.149	0.150
-0.9991	0.184	0.565	1.05E-28	0.271	1.031	0.760	-0.381
0.5175	0.184	0.000	1.01E-19	0.246	0.000	0.245	0.184
0.5464	0.184	0.007	1.61E-16	0.257	0.006	0.251	0.177
-1.3133	0.184	0.264	1.37E-12	0.447	1.274	0.828	-0.080
-0.9436	0.184	0.328	5.48E-12	0.230	0.696	0.466	-0.144
0.3401	0.184	0.018	2.68E-11	0.190	0.017	0.173	0.166
0.3313	0.184	0.005	1.50E-08	0.181	0.004	0.177	0.179
0.2632	0.184	0.052	2.64E-07	0.171	0.046	0.126	0.132
-0.9163	0.184	0.486	3.13E-07	0.435	1.200	0.765	-0.302
0.2561	0.184	0.094	1.45E-06	0.188	0.085	0.103	0.090
0.4326	0.184	0.068	2.43E-06	0.214	0.062	0.153	0.116
0.2924	0.184	0.032	1.16E-04	0.184	0.043	0.141	0.152
0.2846	0.184	0.074	1.54E-04	0.186	0.067	0.119	0.110
0.2837	0.184	0.068	1.67E-04	0.171	0.059	0.112	0.116
-0.3621	0.184	0.385	2.16E-04	0.179	0.442	0.263	-0.201
-0.5031	0.184	0.361	2.34E-04	0.157	0.438	0.281	-0.177
0.3257	0.184	0.036	4.79E-04	0.193	0.034	0.160	0.148
-0.4230	0.184	0.405	5.60E-04	0.172	0.483	0.312	-0.221
0.2797	0.184	0.102	8.44E-03	0.217	0.102	0.115	0.082
-1.1034	0.179	0.673	1.66E-59	0.296	1.295	0.999	-0.494
-1.0580	0.179	0.657	1.00E-48	0.299	1.184	0.884	-0.478
0.4055	0.179	0.011	4.54E-18	0.206	0.009	0.197	0.168
0.4558	0.179	0.001	1.98E-16	0.198	0.001	0.197	0.178
0.3823	0.179	0.018	4.88E-14	0.190	0.017	0.173	0.161
0.3805	0.179	0.000	6.46E-14	0.193	0.000	0.193	0.179
0.4346	0.179	0.001	1.11E-13	0.199	0.001	0.198	0.178
0.4280	0.179	0.001	1.12E-13	0.199	0.001	0.199	0.178
0.3660	0.179	0.004	1.67E-11	0.184	0.004	0.180	0.175
0.4131	0.179	0.011	3.37E-08	0.204	0.009	0.195	0.168
0.2819	0.179	0.023	7.73E-07	0.175	0.022	0.152	0.156
0.2603	0.179	0.021	4.28E-05	0.161	0.018	0.142	0.158
-0.7803	0.179	0.485	7.55E-05	0.338	0.964	0.626	-0.306

-0.3594	0.179	0.241	1.07E-04	0.162	0.302	0.140	-0.062
0.3008	0.179	0.059	1.31E-03	0.181	0.052	0.129	0.120
0.2674	0.179	0.070	6.27E-03	0.175	0.060	0.115	0.109
-1.1666	0.174	0.514	1.64E-33	0.258	1.016	0.758	-0.340
-0.7840	0.174	0.618	2.11E-32	0.333	1.100	0.767	-0.444
-0.8608	0.174	0.478	6.75E-20	0.256	0.749	0.493	-0.304
0.3745	0.174	0.004	2.54E-17	0.198	0.004	0.194	0.170
0.3808	0.174	0.002	2.43E-16	0.186	0.001	0.184	0.172
0.3276	0.174	0.003	3.15E-15	0.161	0.003	0.158	0.171
-0.7412	0.174	0.393	3.27E-15	0.196	0.632	0.436	-0.219
-1.2354	0.174	0.368	1.17E-14	0.193	0.725	0.532	-0.194
0.3682	0.174	0.004	3.79E-14	0.179	0.004	0.175	0.170
-0.6186	0.174	0.484	3.05E-13	0.191	0.649	0.458	-0.310
0.3869	0.174	0.001	4.10E-13	0.196	0.001	0.195	0.173
-0.8867	0.174	0.301	2.63E-12	0.202	0.607	0.405	-0.127
-0.6044	0.174	0.478	7.15E-12	0.271	0.706	0.435	-0.304
0.3468	0.174	0.002	1.47E-11	0.173	0.002	0.171	0.172
-0.6205	0.174	0.351	4.53E-11	0.206	0.575	0.369	-0.177
0.3654	0.174	0.000	9.89E-10	0.197	0.000	0.197	0.174
0.3748	0.174	0.034	2.94E-08	0.202	0.029	0.172	0.140
-0.9314	0.174	0.264	5.84E-08	0.161	0.524	0.362	-0.090
0.3489	0.174	0.008	6.75E-08	0.186	0.007	0.179	0.166
0.3081	0.174	0.014	9.70E-08	0.171	0.012	0.159	0.160
0.2975	0.174	0.042	1.04E-07	0.161	0.039	0.122	0.132
-0.3901	0.174	0.313	1.43E-07	0.158	0.391	0.233	-0.139
-0.4547	0.174	0.277	2.42E-07	0.190	0.437	0.248	-0.103
0.2899	0.174	0.053	2.68E-07	0.187	0.049	0.138	0.121
0.3395	0.174	0.032	1.16E-06	0.175	0.034	0.141	0.142
-0.3060	0.174	0.342	1.47E-06	0.189	0.428	0.239	-0.168
-0.3953	0.174	0.392	1.70E-04	0.162	0.447	0.285	-0.218
0.2851	0.174	0.085	2.16E-04	0.167	0.074	0.093	0.089
0.2646	0.174	0.034	3.52E-04	0.159	0.031	0.128	0.140
-0.6340	0.174	0.407	3.50E-03	0.432	1.001	0.570	-0.233
-0.9873	0.169	0.641	1.21E-45	0.240	1.059	0.819	-0.472
-0.9499	0.169	0.520	6.74E-23	0.163	0.734	0.571	-0.351
-1.3109	0.169	0.265	8.76E-17	0.375	1.152	0.777	-0.096
0.3748	0.169	0.001	3.00E-16	0.168	0.000	0.168	0.168
0.3126	0.169	0.058	1.95E-15	0.185	0.053	0.132	0.111
0.3748	0.169	0.001	2.21E-14	0.170	0.001	0.169	0.168
0.3386	0.169	0.001	3.76E-14	0.156	0.001	0.155	0.168
0.3805	0.169	0.001	1.28E-12	0.177	0.001	0.176	0.168
-0.8326	0.169	0.539	1.18E-10	0.292	0.996	0.704	-0.370
0.2933	0.169	0.000	2.42E-10	0.153	0.000	0.153	0.169
0.3295	0.169	0.004	5.00E-10	0.166	0.003	0.163	0.165
0.2584	0.169	0.039	2.14E-09	0.164	0.036	0.128	0.130
-0.8947	0.169	0.521	2.82E-09	0.277	0.966	0.689	-0.352
0.2607	0.169	0.004	1.65E-07	0.145	0.003	0.142	0.165

0.3107	0.169	0.001	1.72E-07	0.162	0.001	0.161	0.168
0.2943	0.169	0.021	1.96E-07	0.154	0.017	0.137	0.148
-0.5712	0.169	0.312	8.15E-07	0.166	0.453	0.287	-0.143
-0.3670	0.169	0.354	4.11E-06	0.139	0.393	0.255	-0.185
0.3266	0.169	0.050	4.88E-06	0.166	0.042	0.124	0.119
0.3009	0.169	0.107	4.03E-05	0.207	0.099	0.107	0.062
-0.6032	0.169	0.265	4.51E-05	0.198	0.465	0.267	-0.096
0.3229	0.169	0.043	1.84E-04	0.156	0.034	0.122	0.126
0.2573	0.169	0.038	5.16E-04	0.194	0.048	0.146	0.131
-0.3093	0.169	0.324	1.45E-03	0.172	0.372	0.200	-0.155
-0.6340	0.169	0.431	3.35E-03	0.328	0.816	0.489	-0.262
-0.2822	0.169	0.244	4.94E-03	0.159	0.305	0.145	-0.075
-0.3213	0.169	0.201	8.99E-03	0.186	0.314	0.128	-0.032
-0.7691	0.164	0.560	1.83E-29	0.237	0.887	0.650	-0.396
0.4802	0.164	0.000	2.41E-16	0.215	0.000	0.214	0.164
0.4187	0.164	0.003	2.72E-13	0.179	0.002	0.176	0.161
0.3478	0.164	0.000	4.84E-13	0.170	0.000	0.169	0.164
0.2884	0.164	0.017	1.53E-12	0.153	0.014	0.139	0.147
-0.5673	0.164	0.376	7.22E-12	0.218	0.575	0.356	-0.212
0.2692	0.164	0.000	1.24E-11	0.143	0.000	0.143	0.164
0.3576	0.164	0.002	4.75E-11	0.176	0.001	0.174	0.162
0.3600	0.164	0.001	5.86E-11	0.175	0.001	0.174	0.163
0.3708	0.164	0.001	6.05E-11	0.177	0.001	0.176	0.163
0.2523	0.164	0.083	1.06E-10	0.170	0.072	0.098	0.081
0.3285	0.164	0.010	1.22E-10	0.169	0.008	0.161	0.154
0.3182	0.164	0.007	1.58E-10	0.162	0.006	0.156	0.157
0.4210	0.164	0.002	3.27E-09	0.197	0.003	0.194	0.162
-1.0162	0.164	0.471	3.41E-09	0.280	0.869	0.589	-0.307
-0.6528	0.164	0.510	2.25E-08	0.276	0.843	0.567	-0.346
0.3341	0.164	0.012	1.10E-07	0.170	0.011	0.158	0.152
0.2530	0.164	0.027	1.10E-07	0.153	0.024	0.129	0.137
-0.4451	0.164	0.389	1.26E-07	0.165	0.480	0.315	-0.225
-0.4420	0.164	0.390	1.29E-06	0.131	0.439	0.308	-0.226
0.3155	0.164	0.014	1.71E-06	0.164	0.012	0.152	0.150
-0.4520	0.164	0.393	2.80E-06	0.189	0.489	0.300	-0.229
0.2641	0.164	0.012	1.22E-04	0.148	0.012	0.136	0.152
0.2575	0.164	0.088	1.57E-04	0.179	0.077	0.102	0.076
0.3367	0.164	0.009	1.63E-04	0.179	0.014	0.166	0.155
-0.2505	0.164	0.274	1.77E-04	0.125	0.287	0.161	-0.110
0.2658	0.164	0.086	1.80E-04	0.169	0.078	0.091	0.078
-0.3914	0.164	0.182	4.85E-04	0.157	0.285	0.128	-0.018
0.4156	0.164	0.036	1.97E-03	0.171	0.031	0.140	0.128
-0.3564	0.164	0.263	3.63E-03	0.148	0.325	0.178	-0.099
-0.9531	0.159	0.614	7.09E-41	0.234	1.032	0.798	-0.455
0.5312	0.159	0.000	2.52E-24	0.224	0.000	0.224	0.159
-0.6713	0.159	0.535	2.76E-20	0.221	0.759	0.538	-0.376
0.5236	0.159	0.001	5.41E-20	0.217	0.001	0.215	0.158

0.4776	0.159	0.000	8.58E-20	0.217	0.000	0.216	0.159
-0.5875	0.159	0.503	5.78E-19	0.208	0.670	0.462	-0.344
0.4265	0.159	0.018	4.09E-18	0.202	0.015	0.187	0.141
-0.6592	0.159	0.534	7.03E-15	0.211	0.742	0.531	-0.375
0.2802	0.159	0.000	3.11E-14	0.146	0.000	0.146	0.159
0.3453	0.159	0.007	8.67E-14	0.170	0.007	0.163	0.152
0.2744	0.159	0.000	2.22E-12	0.147	0.000	0.146	0.159
0.2936	0.159	0.003	1.86E-11	0.150	0.004	0.146	0.156
-0.5481	0.159	0.400	2.16E-11	0.182	0.540	0.358	-0.241
0.3310	0.159	0.034	9.02E-11	0.169	0.029	0.140	0.125
0.2892	0.159	0.001	1.03E-09	0.144	0.001	0.143	0.158
0.2746	0.159	0.003	1.10E-09	0.128	0.003	0.126	0.156
-0.4551	0.159	0.369	4.93E-09	0.149	0.445	0.296	-0.210
0.3358	0.159	0.000	5.54E-09	0.158	0.000	0.157	0.159
0.2628	0.159	0.028	1.06E-07	0.158	0.027	0.131	0.131
0.3111	0.159	0.030	1.70E-07	0.173	0.030	0.142	0.129
0.3227	0.159	0.008	9.88E-07	0.159	0.006	0.153	0.151
-0.4778	0.159	0.416	1.29E-06	0.149	0.483	0.334	-0.257
-0.5725	0.159	0.373	3.97E-06	0.137	0.450	0.313	-0.214
-0.5057	0.159	0.399	6.54E-06	0.161	0.494	0.334	-0.240
-0.4365	0.159	0.321	6.66E-06	0.148	0.407	0.258	-0.162
-0.3331	0.159	0.251	1.09E-04	0.155	0.340	0.186	-0.092
-0.4219	0.159	0.361	2.00E-04	0.152	0.430	0.278	-0.202
0.2579	0.159	0.087	6.75E-04	0.165	0.077	0.088	0.072
0.2877	0.159	0.031	1.50E-03	0.174	0.027	0.147	0.128
0.3285	0.159	0.026	1.53E-03	0.171	0.026	0.144	0.133
-0.9693	0.154	0.628	8.44E-47	0.255	1.068	0.813	-0.474
0.3845	0.154	0.000	2.67E-20	0.173	0.000	0.173	0.154
-0.9815	0.154	0.385	1.39E-19	0.167	0.665	0.498	-0.231
0.4088	0.154	0.000	1.28E-18	0.175	0.000	0.174	0.154
0.3679	0.154	0.005	1.30E-18	0.174	0.004	0.170	0.149
0.3300	0.154	0.000	1.05E-16	0.158	0.000	0.157	0.154
-0.6944	0.154	0.536	3.25E-15	0.215	0.781	0.566	-0.382
0.3178	0.154	0.001	9.35E-15	0.162	0.001	0.161	0.153
-0.8069	0.154	0.463	4.23E-12	0.173	0.731	0.559	-0.309
0.3428	0.154	0.027	6.78E-12	0.162	0.023	0.138	0.127
0.2581	0.154	0.001	1.20E-11	0.140	0.001	0.138	0.153
0.3049	0.154	0.001	7.92E-11	0.146	0.001	0.145	0.153
0.3160	0.154	0.000	1.09E-09	0.140	0.000	0.139	0.154
0.2679	0.154	0.009	1.41E-09	0.140	0.007	0.132	0.145
0.2740	0.154	0.016	8.96E-09	0.144	0.014	0.130	0.138
0.2651	0.154	0.000	2.42E-08	0.137	0.000	0.137	0.154
-0.6312	0.154	0.465	4.30E-07	0.172	0.632	0.461	-0.311
-0.3622	0.154	0.346	4.91E-07	0.148	0.409	0.261	-0.192
0.2905	0.154	0.008	4.36E-05	0.158	0.010	0.148	0.146
0.3379	0.154	0.011	8.67E-05	0.162	0.015	0.147	0.143
-0.3812	0.154	0.186	6.35E-04	0.160	0.306	0.146	-0.032

-1.2797	0.149	0.665	9.09E-54	0.192	1.127	0.935	-0.516
-0.9658	0.149	0.585	4.63E-33	0.184	0.908	0.723	-0.436
-0.8736	0.149	0.539	2.92E-26	0.159	0.780	0.622	-0.390
-1.0166	0.149	0.554	4.01E-22	0.195	0.914	0.719	-0.405
-0.8152	0.149	0.407	1.52E-18	0.165	0.608	0.443	-0.258
-0.5467	0.149	0.463	3.65E-15	0.163	0.591	0.427	-0.314
-1.2658	0.149	0.212	2.51E-14	0.236	0.680	0.444	-0.063
0.3548	0.149	0.001	8.36E-14	0.168	0.001	0.167	0.148
-0.4761	0.149	0.400	4.69E-13	0.144	0.471	0.327	-0.251
0.2792	0.149	0.001	1.62E-12	0.142	0.001	0.142	0.148
0.3496	0.149	0.002	6.48E-12	0.165	0.003	0.163	0.147
0.3006	0.149	0.001	5.70E-11	0.143	0.001	0.143	0.148
-0.7437	0.149	0.499	8.79E-11	0.203	0.737	0.534	-0.350
0.3842	0.149	0.002	1.04E-09	0.169	0.002	0.167	0.147
0.2708	0.149	0.006	4.79E-09	0.140	0.005	0.135	0.143
0.2990	0.149	0.028	1.62E-08	0.155	0.024	0.132	0.121
0.2973	0.149	0.012	3.65E-07	0.158	0.012	0.145	0.137
0.3217	0.149	0.024	8.38E-07	0.167	0.020	0.146	0.125
0.2793	0.149	0.001	3.52E-06	0.144	0.001	0.144	0.148
-0.2525	0.149	0.171	2.68E-05	0.111	0.222	0.111	-0.022
0.2922	0.149	0.028	3.07E-05	0.153	0.023	0.130	0.121
0.3066	0.149	0.019	1.89E-04	0.153	0.019	0.134	0.130
-0.5301	0.149	0.438	2.15E-04	0.172	0.548	0.376	-0.289
0.2814	0.149	0.032	8.99E-04	0.143	0.028	0.115	0.117
0.3024	0.144	0.007	5.85E-16	0.138	0.006	0.131	0.137
-0.5099	0.144	0.443	7.78E-16	0.170	0.567	0.397	-0.299
-0.6426	0.144	0.486	2.49E-13	0.157	0.616	0.460	-0.342
-0.9299	0.144	0.513	3.09E-12	0.289	1.033	0.744	-0.369
-0.5669	0.144	0.369	3.73E-11	0.119	0.458	0.339	-0.225
0.2845	0.144	0.001	5.34E-11	0.142	0.001	0.141	0.143
0.3125	0.144	0.001	2.21E-10	0.146	0.001	0.145	0.143
0.2810	0.144	0.000	2.72E-10	0.150	0.000	0.150	0.144
0.4411	0.144	0.001	8.52E-10	0.181	0.001	0.181	0.143
-0.3759	0.144	0.255	2.35E-09	0.142	0.350	0.207	-0.111
-0.4498	0.144	0.304	4.59E-09	0.146	0.410	0.263	-0.160
0.2652	0.144	0.002	4.81E-09	0.121	0.002	0.119	0.142
-0.7213	0.144	0.266	1.11E-08	0.152	0.431	0.279	-0.122
0.2607	0.144	0.002	1.69E-08	0.132	0.002	0.131	0.142
0.2766	0.144	0.006	5.78E-08	0.127	0.005	0.122	0.138
0.2501	0.144	0.002	5.90E-08	0.116	0.002	0.115	0.142
0.3098	0.144	0.018	5.67E-07	0.154	0.016	0.137	0.126
-0.6813	0.144	0.254	1.11E-06	0.126	0.416	0.290	-0.110
-0.4531	0.144	0.327	4.90E-06	0.123	0.395	0.272	-0.183
-0.3633	0.144	0.243	5.46E-05	0.115	0.287	0.171	-0.099
0.2533	0.144	0.000	6.71E-05	0.130	0.000	0.130	0.144
-0.5536	0.144	0.428	1.13E-03	0.188	0.587	0.400	-0.284
0.2802	0.144	0.015	2.23E-03	0.135	0.013	0.123	0.129

0.2603	0.144	0.007	9.39E-03	0.121	0.006	0.115	0.137
-1.2952	0.139	0.251	1.09E-23	0.299	0.964	0.665	-0.112
0.2886	0.139	0.000	5.03E-12	0.128	0.000	0.128	0.139
0.2528	0.139	0.005	1.01E-10	0.127	0.004	0.123	0.134
-0.5186	0.139	0.222	7.22E-09	0.165	0.387	0.222	-0.083
-0.5199	0.139	0.254	1.70E-08	0.118	0.365	0.248	-0.115
0.2758	0.139	0.004	4.06E-06	0.125	0.003	0.122	0.135
0.2638	0.139	0.007	8.49E-06	0.127	0.006	0.121	0.132
-0.7540	0.139	0.420	1.76E-05	0.194	0.656	0.462	-0.281
-0.3685	0.139	0.228	1.13E-04	0.085	0.260	0.175	-0.089
0.2529	0.139	0.022	6.25E-04	0.136	0.018	0.118	0.117
-0.3340	0.139	0.268	1.79E-03	0.102	0.296	0.194	-0.129
-1.6214	0.134	0.255	3.79E-27	0.353	1.095	0.742	-0.121
-0.7311	0.134	0.453	1.41E-26	0.187	0.699	0.512	-0.319
-0.6574	0.134	0.402	4.02E-22	0.140	0.565	0.425	-0.268
0.3623	0.134	0.000	1.60E-15	0.154	0.000	0.153	0.134
-0.6873	0.134	0.253	1.18E-12	0.205	0.555	0.350	-0.119
-1.0021	0.134	0.490	1.79E-10	0.196	0.857	0.661	-0.356
-0.6026	0.134	0.473	3.44E-09	0.155	0.602	0.447	-0.339
0.2764	0.134	0.013	1.72E-08	0.136	0.012	0.124	0.121
0.3022	0.134	0.026	7.90E-08	0.145	0.022	0.122	0.108
0.3269	0.134	0.040	1.69E-07	0.157	0.035	0.121	0.094
0.3008	0.134	0.000	1.86E-07	0.129	0.000	0.129	0.134
-0.5903	0.134	0.432	2.81E-07	0.166	0.578	0.412	-0.298
-0.4620	0.134	0.285	4.11E-07	0.104	0.354	0.249	-0.151
-0.4318	0.134	0.234	7.28E-06	0.083	0.283	0.200	-0.100
-0.3055	0.134	0.310	4.33E-05	0.140	0.354	0.214	-0.176
-0.4849	0.134	0.251	7.00E-05	0.101	0.330	0.229	-0.117
-0.4484	0.134	0.170	8.19E-05	0.156	0.336	0.180	-0.036
0.2712	0.134	0.024	8.90E-04	0.143	0.023	0.120	0.110
0.2641	0.134	0.011	1.29E-03	0.126	0.011	0.116	0.123
-0.3578	0.134	0.184	9.00E-03	0.143	0.269	0.126	-0.050
0.4722	0.129	0.000	1.37E-14	0.189	0.000	0.189	0.129
0.3135	0.129	0.001	1.37E-12	0.138	0.001	0.137	0.128
0.2512	0.129	0.000	7.07E-10	0.125	0.000	0.125	0.129
0.2841	0.129	0.002	5.61E-09	0.117	0.002	0.115	0.127
-0.3723	0.129	0.179	9.21E-09	0.171	0.312	0.141	-0.050
0.2661	0.129	0.002	1.16E-05	0.124	0.003	0.121	0.127
0.2971	0.129	0.017	4.45E-05	0.142	0.015	0.127	0.112
0.2594	0.129	0.005	7.72E-04	0.127	0.004	0.123	0.124
0.2786	0.129	0.040	8.15E-04	0.134	0.036	0.098	0.089
-0.3342	0.129	0.283	1.97E-03	0.112	0.313	0.201	-0.154
0.2566	0.129	0.009	2.47E-03	0.122	0.008	0.114	0.120
-0.3459	0.129	0.296	4.75E-03	0.124	0.337	0.213	-0.167
-0.9932	0.124	0.532	2.08E-28	0.127	0.800	0.673	-0.408
-1.0573	0.124	0.355	2.84E-17	0.224	0.796	0.572	-0.231
-0.6539	0.124	0.310	2.95E-15	0.091	0.414	0.323	-0.186

-0.8914	0.124	0.491	6.01E-13	0.176	0.787	0.612	-0.367
0.3450	0.124	0.000	3.71E-12	0.133	0.000	0.133	0.124
0.2736	0.124	0.009	1.99E-10	0.127	0.007	0.120	0.115
-0.3462	0.124	0.321	3.31E-10	0.132	0.385	0.253	-0.197
0.2678	0.124	0.005	1.02E-08	0.113	0.004	0.109	0.119
-0.6257	0.124	0.439	5.62E-08	0.152	0.587	0.434	-0.315
-0.2856	0.124	0.144	1.11E-07	0.095	0.201	0.106	-0.020
-0.3761	0.124	0.155	3.62E-07	0.129	0.261	0.132	-0.031
-0.3564	0.124	0.167	1.52E-06	0.146	0.295	0.149	-0.043
-0.2930	0.124	0.244	2.42E-06	0.097	0.266	0.169	-0.120
-0.5389	0.124	0.192	1.32E-05	0.104	0.293	0.189	-0.068
-0.5389	0.124	0.192	1.32E-05	0.104	0.293	0.189	-0.068
-0.2790	0.124	0.246	3.95E-05	0.105	0.274	0.169	-0.122
-0.7344	0.119	0.524	1.54E-28	0.159	0.703	0.545	-0.405
0.2585	0.119	0.004	1.50E-07	0.120	0.005	0.115	0.115
0.2565	0.119	0.002	1.57E-07	0.123	0.002	0.121	0.117
-0.4180	0.119	0.322	2.45E-07	0.120	0.376	0.257	-0.203
-0.4885	0.119	0.333	4.44E-06	0.097	0.393	0.295	-0.214
-0.7358	0.119	0.200	4.87E-06	0.166	0.449	0.283	-0.081
-0.3196	0.119	0.250	2.14E-05	0.074	0.263	0.189	-0.131
-0.2620	0.119	0.225	3.35E-04	0.096	0.251	0.154	-0.106
-0.3759	0.119	0.314	3.53E-04	0.102	0.341	0.240	-0.195
-1.2600	0.114	0.445	4.39E-41	0.183	0.903	0.720	-0.331
-1.3004	0.114	0.492	1.42E-40	0.155	0.888	0.733	-0.378
-1.3525	0.114	0.399	1.41E-29	0.181	0.895	0.714	-0.285
-0.6486	0.114	0.245	4.23E-13	0.116	0.379	0.263	-0.131
-0.5167	0.114	0.410	4.66E-12	0.112	0.478	0.366	-0.296
-0.5651	0.114	0.278	6.21E-11	0.150	0.408	0.259	-0.164
-0.7727	0.114	0.454	1.11E-09	0.145	0.647	0.502	-0.340
-0.6546	0.114	0.443	1.60E-09	0.141	0.599	0.458	-0.329
-0.3721	0.114	0.258	9.88E-09	0.128	0.328	0.201	-0.144
-0.3496	0.114	0.146	3.22E-08	0.102	0.244	0.141	-0.032
-0.3403	0.114	0.212	3.77E-07	0.087	0.258	0.170	-0.098
-0.5216	0.114	0.248	6.50E-07	0.079	0.319	0.240	-0.134
0.2891	0.114	0.000	5.90E-06	0.114	0.000	0.114	0.114
-0.4106	0.114	0.182	1.61E-05	0.079	0.236	0.157	-0.068
-0.3321	0.114	0.228	1.43E-04	0.110	0.274	0.164	-0.114
-0.3528	0.114	0.255	3.18E-04	0.086	0.279	0.193	-0.141
-0.6663	0.109	0.492	4.53E-29	0.139	0.658	0.520	-0.383
-0.6362	0.109	0.462	3.56E-17	0.120	0.565	0.445	-0.353
-0.4040	0.109	0.359	1.11E-16	0.166	0.494	0.328	-0.250
-0.4263	0.109	0.231	7.28E-13	0.131	0.336	0.205	-0.122
-0.3443	0.109	0.177	2.30E-10	0.106	0.254	0.147	-0.068
-1.3133	0.109	0.256	1.11E-09	0.233	0.809	0.576	-0.147
-0.2962	0.109	0.224	4.26E-07	0.102	0.263	0.162	-0.115
-0.3879	0.109	0.281	4.26E-06	0.099	0.324	0.225	-0.172
0.2537	0.109	0.049	4.94E-04	0.126	0.044	0.081	0.060

-0.4127	0.109	0.383	1.32E-03	0.159	0.485	0.326	-0.274
-0.7804	0.109	0.364	4.41E-03	0.226	0.750	0.524	-0.255
-0.2765	0.109	0.220	8.06E-03	0.077	0.228	0.151	-0.111
-1.1788	0.104	0.277	4.31E-31	0.149	0.667	0.518	-0.173
-0.7076	0.104	0.448	5.39E-25	0.106	0.568	0.461	-0.344
-0.7350	0.104	0.282	2.21E-22	0.105	0.459	0.354	-0.178
-0.8369	0.104	0.249	3.73E-19	0.193	0.635	0.442	-0.145
-1.3787	0.104	0.271	9.17E-18	0.141	0.709	0.568	-0.167
-0.4178	0.104	0.175	1.31E-15	0.084	0.251	0.167	-0.071
-0.3408	0.104	0.303	1.67E-11	0.104	0.333	0.229	-0.199
-0.3020	0.104	0.164	1.09E-10	0.082	0.207	0.125	-0.060
-1.2815	0.104	0.221	1.55E-10	0.204	0.741	0.536	-0.117
-0.5134	0.104	0.175	3.47E-10	0.072	0.259	0.188	-0.071
-0.6837	0.104	0.221	4.50E-09	0.134	0.414	0.280	-0.117
-0.5440	0.104	0.177	1.22E-06	0.105	0.297	0.191	-0.073
-0.4753	0.104	0.105	6.41E-06	0.089	0.218	0.128	-0.001
-0.3147	0.104	0.280	2.70E-05	0.100	0.305	0.205	-0.176
-0.6840	0.104	0.353	4.55E-03	0.110	0.495	0.385	-0.249
-1.7474	0.100	0.324	1.43E-37	0.253	1.169	0.916	-0.224
-0.8827	0.100	0.423	6.89E-24	0.114	0.607	0.493	-0.323
-0.6757	0.100	0.392	8.27E-23	0.091	0.490	0.399	-0.292
-0.5918	0.100	0.389	1.05E-14	0.107	0.476	0.369	-0.289
-0.4903	0.100	0.368	2.04E-12	0.093	0.416	0.323	-0.268
-0.5814	0.100	0.220	1.64E-11	0.090	0.334	0.245	-0.120
-0.3212	0.100	0.192	9.48E-08	0.087	0.241	0.154	-0.092
-0.3153	0.100	0.318	2.15E-07	0.107	0.344	0.237	-0.218
-0.4436	0.100	0.120	1.66E-06	0.102	0.240	0.139	-0.020
-0.5572	0.100	0.175	1.75E-06	0.075	0.295	0.220	-0.075
-0.4316	0.100	0.149	3.29E-06	0.101	0.265	0.163	-0.049
-0.3245	0.100	0.179	5.00E-06	0.098	0.248	0.151	-0.079
-0.8109	0.100	0.389	7.58E-06	0.122	0.581	0.458	-0.289
-0.3455	0.100	0.285	1.52E-05	0.069	0.302	0.233	-0.185
-0.2592	0.100	0.224	4.64E-04	0.072	0.232	0.160	-0.124
-0.3330	0.100	0.229	8.78E-04	0.069	0.251	0.181	-0.129
-0.2737	0.100	0.137	8.46E-03	0.069	0.168	0.099	-0.037
-0.4675	0.095	0.215	4.89E-17	0.190	0.470	0.280	-0.120
-0.6177	0.095	0.322	8.04E-14	0.101	0.433	0.332	-0.227
-1.6342	0.095	0.254	4.32E-13	0.250	1.015	0.765	-0.159
-1.1692	0.095	0.226	1.59E-12	0.124	0.554	0.430	-0.131
-0.5583	0.095	0.354	4.03E-12	0.111	0.452	0.341	-0.259
-0.6648	0.095	0.198	7.18E-12	0.057	0.296	0.239	-0.103
-0.3606	0.095	0.207	1.21E-11	0.100	0.286	0.186	-0.112
-0.8234	0.095	0.461	1.55E-11	0.104	0.643	0.540	-0.366
-0.3896	0.095	0.214	1.21E-10	0.049	0.248	0.199	-0.119
-0.4557	0.095	0.305	1.66E-10	0.077	0.343	0.266	-0.210
-0.4557	0.095	0.305	1.66E-10	0.077	0.343	0.266	-0.210
-0.6452	0.095	0.194	1.92E-10	0.118	0.372	0.253	-0.099

-0.4401	0.095	0.132	2.58E-10	0.066	0.224	0.158	-0.037
-0.3419	0.095	0.110	8.81E-10	0.056	0.166	0.110	-0.015
-0.3916	0.095	0.153	1.94E-08	0.080	0.230	0.150	-0.058
-0.3574	0.095	0.219	4.45E-08	0.073	0.257	0.184	-0.124
-0.2870	0.095	0.134	3.80E-06	0.073	0.182	0.110	-0.039
-0.2570	0.095	0.178	1.36E-04	0.072	0.199	0.127	-0.083
-0.3374	0.095	0.293	5.48E-04	0.087	0.307	0.220	-0.198
-1.0246	0.090	0.470	6.45E-25	0.097	0.739	0.641	-0.380
-0.4458	0.090	0.153	2.05E-19	0.051	0.215	0.164	-0.063
-0.2763	0.090	0.126	3.33E-12	0.067	0.177	0.111	-0.036
-0.4177	0.090	0.265	2.43E-11	0.074	0.302	0.228	-0.175
-0.2814	0.090	0.159	2.70E-11	0.073	0.204	0.131	-0.069
-0.4351	0.090	0.250	5.02E-11	0.061	0.293	0.232	-0.160
-0.6542	0.090	0.392	3.66E-09	0.092	0.496	0.404	-0.302
-0.6030	0.090	0.384	8.99E-09	0.086	0.471	0.385	-0.294
-0.4406	0.090	0.271	1.40E-07	0.078	0.314	0.236	-0.181
-0.2592	0.090	0.148	1.76E-07	0.088	0.190	0.102	-0.058
-0.3963	0.090	0.286	2.91E-07	0.070	0.306	0.235	-0.196
-1.0289	0.090	0.229	3.31E-07	0.213	0.736	0.523	-0.139
-0.3304	0.090	0.138	7.23E-07	0.101	0.233	0.132	-0.048
-0.4542	0.090	0.350	1.91E-06	0.084	0.387	0.303	-0.260
-0.3052	0.090	0.138	2.12E-06	0.097	0.219	0.123	-0.048
-0.3468	0.090	0.303	1.67E-04	0.091	0.320	0.228	-0.213
-0.2626	0.090	0.160	1.94E-04	0.076	0.192	0.117	-0.070
-0.3673	0.090	0.297	1.34E-03	0.094	0.335	0.240	-0.207
-1.2427	0.085	0.284	2.45E-29	0.170	0.718	0.548	-0.199
-0.7470	0.085	0.395	3.71E-28	0.099	0.543	0.444	-0.310
-0.4446	0.085	0.180	2.94E-18	0.100	0.293	0.193	-0.095
-0.9718	0.085	0.358	5.10E-16	0.115	0.622	0.507	-0.273
-1.3258	0.085	0.393	8.26E-15	0.085	0.699	0.614	-0.308
-0.2741	0.085	0.196	9.30E-14	0.066	0.234	0.169	-0.111
-1.1689	0.085	0.276	1.24E-12	0.140	0.700	0.561	-0.191
-0.3021	0.085	0.239	2.07E-11	0.085	0.273	0.188	-0.154
-0.3072	0.085	0.205	2.09E-11	0.065	0.234	0.169	-0.120
-0.6471	0.085	0.173	7.85E-10	0.106	0.358	0.252	-0.088
-0.3393	0.085	0.142	7.95E-10	0.062	0.190	0.129	-0.057
-1.4130	0.085	0.240	8.47E-10	0.205	0.851	0.646	-0.155
-0.2879	0.085	0.153	1.60E-09	0.056	0.184	0.129	-0.068
-0.2720	0.085	0.204	1.26E-08	0.084	0.231	0.147	-0.119
-0.3409	0.085	0.224	4.16E-08	0.055	0.243	0.188	-0.139
-0.3386	0.085	0.286	6.54E-08	0.089	0.300	0.211	-0.201
-0.3407	0.085	0.130	9.01E-08	0.057	0.182	0.125	-0.045
-0.2811	0.085	0.103	6.10E-06	0.080	0.128	0.048	-0.018
-0.2730	0.085	0.171	2.16E-05	0.044	0.179	0.134	-0.086
-0.3390	0.085	0.287	4.04E-05	0.091	0.310	0.219	-0.202
-0.2784	0.085	0.203	7.74E-05	0.067	0.213	0.146	-0.118
-0.2966	0.085	0.183	5.73E-04	0.064	0.201	0.137	-0.098

-0.9470	0.080	0.306	7.63E-32	0.097	0.523	0.426	-0.226
-0.4738	0.080	0.289	3.82E-22	0.083	0.359	0.276	-0.209
-0.6331	0.080	0.226	5.53E-22	0.143	0.455	0.311	-0.146
-0.5962	0.080	0.315	2.58E-19	0.078	0.400	0.322	-0.235
-0.7219	0.080	0.358	2.56E-18	0.054	0.471	0.417	-0.278
-0.6100	0.080	0.203	1.46E-13	0.166	0.500	0.333	-0.123
-0.2805	0.080	0.136	6.78E-10	0.063	0.174	0.112	-0.056
-0.3935	0.080	0.215	7.14E-10	0.057	0.249	0.192	-0.135
-0.4275	0.080	0.298	7.19E-10	0.070	0.332	0.262	-0.218
-0.2986	0.080	0.220	1.44E-09	0.064	0.253	0.189	-0.140
-0.4352	0.080	0.159	4.67E-09	0.060	0.228	0.169	-0.079
-0.5826	0.080	0.388	5.26E-09	0.108	0.503	0.395	-0.308
-0.4359	0.080	0.219	6.45E-09	0.075	0.297	0.221	-0.139
-0.3216	0.080	0.271	2.48E-08	0.078	0.277	0.199	-0.191
-0.2803	0.080	0.140	5.66E-08	0.074	0.185	0.112	-0.060
-0.6604	0.080	0.174	2.93E-07	0.148	0.425	0.277	-0.094
-0.4513	0.080	0.284	4.10E-05	0.073	0.327	0.253	-0.204
-1.0097	0.080	0.222	6.28E-05	0.191	0.680	0.489	-0.142
-0.4550	0.080	0.128	9.92E-05	0.103	0.267	0.164	-0.048
-0.9176	0.075	0.342	7.05E-25	0.080	0.517	0.437	-0.267
-0.3650	0.075	0.207	2.73E-16	0.069	0.263	0.195	-0.132
-0.5194	0.075	0.378	1.85E-15	0.086	0.442	0.356	-0.303
-0.7405	0.075	0.211	4.51E-13	0.052	0.329	0.277	-0.136
-0.5877	0.075	0.412	5.10E-11	0.083	0.496	0.412	-0.337
-0.3293	0.075	0.143	1.57E-10	0.060	0.195	0.134	-0.068
-1.2856	0.075	0.351	2.57E-10	0.080	0.687	0.607	-0.276
-0.3444	0.075	0.231	3.17E-10	0.039	0.238	0.199	-0.156
-0.2511	0.075	0.189	9.87E-10	0.061	0.199	0.138	-0.114
-0.6766	0.075	0.117	1.09E-09	0.046	0.258	0.212	-0.042
-0.8727	0.075	0.208	1.66E-07	0.162	0.580	0.419	-0.133
-0.5033	0.075	0.365	5.76E-06	0.082	0.426	0.343	-0.290
-0.4767	0.075	0.116	5.68E-04	0.039	0.192	0.153	-0.041
-0.2690	0.075	0.193	1.56E-03	0.040	0.191	0.152	-0.118
-0.3356	0.075	0.187	3.66E-03	0.065	0.204	0.139	-0.112
-0.5844	0.075	0.101	3.94E-03	0.043	0.208	0.165	-0.026
-1.0909	0.070	0.361	1.00E-33	0.151	0.860	0.709	-0.291
-0.5634	0.070	0.186	6.90E-24	0.068	0.305	0.237	-0.116
-0.4637	0.070	0.203	6.97E-22	0.073	0.287	0.214	-0.133
-1.5821	0.070	0.463	8.26E-20	0.089	0.821	0.732	-0.393
-0.4633	0.070	0.139	1.16E-19	0.030	0.203	0.173	-0.069
-0.3781	0.070	0.286	3.10E-16	0.077	0.307	0.230	-0.216
-0.3814	0.070	0.211	6.30E-15	0.056	0.244	0.188	-0.141
-1.3381	0.070	0.353	3.51E-14	0.076	0.657	0.580	-0.283
-0.3490	0.070	0.220	3.85E-12	0.041	0.235	0.193	-0.150
-0.4312	0.070	0.255	1.06E-09	0.054	0.280	0.226	-0.185
-0.5343	0.070	0.310	9.53E-09	0.064	0.368	0.304	-0.240
-0.9338	0.070	0.161	1.20E-08	0.080	0.421	0.341	-0.091

-0.3537	0.070	0.139	4.97E-08	0.040	0.187	0.148	-0.069
-1.1882	0.070	0.210	8.91E-08	0.138	0.614	0.476	-0.140
-0.4895	0.070	0.366	3.37E-07	0.077	0.411	0.334	-0.296
-0.2723	0.070	0.137	2.69E-06	0.046	0.164	0.118	-0.067
-0.5004	0.070	0.329	5.77E-06	0.073	0.382	0.308	-0.259
-0.2674	0.070	0.234	6.39E-05	0.062	0.233	0.171	-0.164
-0.3057	0.070	0.245	1.34E-03	0.076	0.265	0.189	-0.175
-0.2967	0.070	0.223	2.39E-03	0.048	0.223	0.174	-0.153
-1.3690	0.065	0.438	1.65E-38	0.100	0.920	0.820	-0.373
-0.4603	0.065	0.146	5.54E-22	0.061	0.235	0.173	-0.081
-0.6843	0.065	0.235	1.03E-21	0.065	0.353	0.287	-0.170
-0.6897	0.065	0.181	1.19E-21	0.051	0.297	0.246	-0.116
-0.4439	0.065	0.293	1.49E-16	0.063	0.323	0.259	-0.228
-0.4446	0.065	0.149	1.65E-14	0.069	0.232	0.163	-0.084
-0.2853	0.065	0.144	2.40E-13	0.062	0.194	0.132	-0.079
-0.5075	0.065	0.149	2.80E-13	0.071	0.264	0.194	-0.084
-0.7921	0.065	0.182	8.27E-13	0.118	0.462	0.344	-0.117
-0.3511	0.065	0.238	2.89E-12	0.075	0.271	0.196	-0.173
-0.4149	0.065	0.164	1.63E-11	0.051	0.219	0.167	-0.099
-0.3426	0.065	0.221	4.44E-11	0.055	0.242	0.187	-0.156
-0.3973	0.065	0.300	2.66E-09	0.071	0.332	0.261	-0.235
-0.5620	0.065	0.181	1.05E-07	0.152	0.442	0.291	-0.116
-0.7364	0.065	0.179	3.05E-07	0.123	0.443	0.320	-0.114
-0.5401	0.065	0.313	1.44E-06	0.067	0.390	0.323	-0.248
-1.2001	0.065	0.247	1.69E-06	0.083	0.491	0.408	-0.182
-0.2510	0.065	0.164	1.96E-06	0.066	0.182	0.117	-0.099
-0.8843	0.065	0.176	2.01E-06	0.107	0.463	0.356	-0.111
-0.6562	0.065	0.168	9.22E-06	0.130	0.415	0.285	-0.103
-0.7530	0.065	0.159	1.04E-05	0.124	0.389	0.265	-0.094
-0.2982	0.065	0.251	5.04E-04	0.057	0.258	0.200	-0.186
-0.2751	0.065	0.190	7.40E-04	0.051	0.200	0.149	-0.125
-0.4209	0.060	0.131	5.18E-22	0.053	0.206	0.154	-0.071
-0.4528	0.060	0.284	4.20E-18	0.073	0.345	0.271	-0.224
-0.5909	0.060	0.185	2.57E-16	0.041	0.271	0.230	-0.125
-0.6084	0.060	0.127	1.20E-14	0.063	0.271	0.208	-0.067
-0.2975	0.060	0.185	2.18E-13	0.057	0.205	0.149	-0.125
-0.4526	0.060	0.265	9.73E-13	0.057	0.308	0.251	-0.205
-0.3081	0.060	0.159	7.12E-10	0.033	0.178	0.144	-0.099
-0.6338	0.060	0.318	1.83E-09	0.069	0.427	0.358	-0.258
-0.9289	0.060	0.207	4.87E-09	0.148	0.578	0.430	-0.147
-0.2996	0.060	0.133	5.12E-09	0.044	0.171	0.127	-0.073
-0.9451	0.060	0.219	2.53E-08	0.073	0.387	0.314	-0.159
-0.2630	0.060	0.147	1.63E-07	0.043	0.160	0.117	-0.087
-0.3389	0.060	0.211	2.49E-04	0.070	0.255	0.184	-0.151
-0.3094	0.060	0.226	1.22E-03	0.055	0.239	0.185	-0.166
-0.3079	0.060	0.205	2.10E-03	0.036	0.209	0.173	-0.145
-0.3511	0.060	0.288	2.27E-03	0.071	0.307	0.236	-0.228

-0.3327	0.060	0.182	2.55E-03	0.048	0.212	0.164	-0.122
-0.3763	0.060	0.268	4.54E-03	0.052	0.282	0.230	-0.208
-1.0003	0.055	0.341	3.54E-34	0.086	0.611	0.525	-0.286
-0.8804	0.055	0.291	3.56E-24	0.061	0.481	0.420	-0.236
-0.2531	0.055	0.167	6.44E-16	0.046	0.194	0.148	-0.112
-1.1687	0.055	0.226	4.88E-15	0.103	0.556	0.454	-0.171
-0.2702	0.055	0.191	2.57E-13	0.050	0.201	0.151	-0.136
-0.3056	0.055	0.161	1.65E-12	0.052	0.195	0.143	-0.106
-0.4953	0.055	0.193	1.66E-12	0.091	0.332	0.241	-0.138
-0.4089	0.055	0.158	4.26E-12	0.081	0.256	0.175	-0.103
-0.4095	0.055	0.153	5.08E-09	0.094	0.284	0.190	-0.098
-0.3233	0.055	0.115	8.13E-07	0.097	0.233	0.136	-0.060
-0.8585	0.055	0.158	3.04E-06	0.109	0.394	0.285	-0.103
-0.2673	0.055	0.191	2.30E-05	0.045	0.199	0.154	-0.136
-0.2650	0.055	0.191	4.81E-05	0.055	0.201	0.146	-0.136
-1.2599	0.055	0.213	5.82E-04	0.079	0.533	0.454	-0.158
-0.3608	0.055	0.268	6.00E-04	0.047	0.273	0.227	-0.213
-0.7482	0.050	0.387	2.89E-28	0.040	0.482	0.443	-0.337
-0.2911	0.050	0.122	4.06E-23	0.051	0.175	0.123	-0.072
-0.9252	0.050	0.184	1.83E-21	0.059	0.353	0.294	-0.134
-0.5745	0.050	0.235	3.84E-20	0.025	0.308	0.283	-0.185
-0.2667	0.050	0.140	4.97E-17	0.050	0.171	0.121	-0.090
-0.6382	0.050	0.137	2.65E-14	0.048	0.265	0.217	-0.087
-0.3261	0.050	0.247	1.45E-08	0.054	0.258	0.204	-0.197
-0.3784	0.050	0.265	1.48E-08	0.038	0.272	0.234	-0.215
-0.3168	0.050	0.232	1.03E-05	0.047	0.235	0.187	-0.182
-0.2852	0.050	0.166	4.20E-04	0.022	0.173	0.150	-0.116
-0.3988	0.050	0.270	4.94E-04	0.041	0.284	0.242	-0.220
-0.7650	0.045	0.251	6.20E-54	0.100	0.533	0.433	-0.206
-0.2695	0.045	0.148	6.85E-26	0.041	0.177	0.135	-0.103
-0.4520	0.045	0.251	1.85E-23	0.034	0.277	0.243	-0.206
-1.1276	0.045	0.195	1.18E-22	0.083	0.496	0.413	-0.150
-0.4836	0.045	0.222	3.00E-22	0.044	0.290	0.246	-0.177
-0.9019	0.045	0.201	4.19E-21	0.052	0.390	0.339	-0.156
-0.5260	0.045	0.136	6.17E-20	0.039	0.237	0.198	-0.091
-0.5479	0.045	0.140	5.08E-18	0.041	0.244	0.203	-0.095
-0.3944	0.045	0.143	5.35E-16	0.026	0.187	0.161	-0.098
-0.2978	0.045	0.166	6.95E-16	0.049	0.194	0.144	-0.121
-0.3704	0.045	0.102	1.64E-14	0.053	0.181	0.127	-0.057
-0.2983	0.045	0.186	7.21E-14	0.035	0.196	0.162	-0.141
-0.3517	0.045	0.237	1.10E-13	0.043	0.246	0.203	-0.192
-0.3414	0.045	0.114	1.28E-13	0.085	0.233	0.148	-0.069
-0.6316	0.045	0.143	2.03E-12	0.065	0.328	0.263	-0.098
-0.2512	0.045	0.109	4.45E-11	0.035	0.137	0.103	-0.064
-0.4186	0.045	0.189	2.46E-09	0.027	0.225	0.198	-0.144
-0.3091	0.045	0.213	7.86E-09	0.054	0.231	0.177	-0.168
-0.7293	0.045	0.149	9.65E-09	0.081	0.386	0.305	-0.104

-0.7497	0.045	0.159	4.30E-08	0.096	0.405	0.309	-0.114
-0.7227	0.045	0.142	6.60E-08	0.065	0.322	0.257	-0.097
-0.4769	0.045	0.177	7.00E-08	0.038	0.253	0.215	-0.132
-0.6386	0.045	0.123	1.11E-07	0.068	0.269	0.201	-0.078
-0.5672	0.045	0.221	1.43E-06	0.051	0.312	0.261	-0.176
-0.3940	0.045	0.275	1.79E-04	0.036	0.283	0.248	-0.230
-2.4709	0.045	0.211	3.10E-04	0.061	0.675	0.614	-0.166
-0.2840	0.045	0.202	2.68E-03	0.036	0.201	0.166	-0.157
-0.7352	0.045	0.115	5.95E-03	0.055	0.271	0.216	-0.070
-0.6374	0.045	0.231	8.97E-03	0.056	0.337	0.281	-0.186
-0.5953	0.040	0.203	1.95E-29	0.043	0.302	0.259	-0.163
-0.7202	0.040	0.163	1.04E-21	0.068	0.360	0.292	-0.123
-0.3483	0.040	0.114	2.77E-20	0.013	0.150	0.136	-0.074
-0.3895	0.040	0.119	1.73E-18	0.033	0.170	0.137	-0.079
-0.4724	0.040	0.104	2.44E-17	0.044	0.206	0.162	-0.064
-0.3532	0.040	0.177	1.81E-15	0.017	0.194	0.176	-0.137
-0.2735	0.040	0.136	1.12E-14	0.038	0.161	0.123	-0.096
-0.4144	0.040	0.186	2.23E-13	0.047	0.248	0.201	-0.146
-0.5944	0.040	0.130	2.23E-12	0.069	0.290	0.221	-0.090
-0.7823	0.040	0.169	8.54E-12	0.088	0.399	0.311	-0.129
-0.4662	0.040	0.281	2.65E-07	0.036	0.315	0.280	-0.241
-0.2858	0.040	0.133	8.45E-07	0.038	0.165	0.127	-0.093
-0.2788	0.040	0.177	3.10E-04	0.036	0.187	0.151	-0.137
-0.2874	0.040	0.159	5.98E-04	0.034	0.164	0.130	-0.119
-0.3187	0.040	0.228	6.43E-04	0.035	0.225	0.190	-0.188
-0.3816	0.040	0.168	2.50E-03	0.032	0.210	0.178	-0.128
-0.4948	0.035	0.170	9.19E-49	0.049	0.280	0.232	-0.135
-0.7003	0.035	0.176	6.28E-44	0.039	0.325	0.287	-0.141
-0.5476	0.035	0.218	7.74E-38	0.043	0.312	0.269	-0.183
-0.7567	0.035	0.166	7.63E-31	0.031	0.290	0.260	-0.131
-0.5658	0.035	0.188	1.53E-29	0.019	0.259	0.239	-0.153
-0.4866	0.035	0.190	2.90E-23	0.029	0.247	0.219	-0.155
-0.6271	0.035	0.221	7.46E-20	0.046	0.344	0.298	-0.186
-1.8830	0.035	0.245	1.49E-14	0.045	0.644	0.599	-0.210
-0.7233	0.035	0.136	2.10E-12	0.061	0.311	0.250	-0.101
-0.7957	0.035	0.121	3.06E-09	0.034	0.284	0.250	-0.086
-0.5006	0.035	0.112	3.40E-09	0.059	0.231	0.172	-0.077
-0.6217	0.035	0.113	1.05E-08	0.047	0.245	0.198	-0.078
-0.7329	0.035	0.138	1.30E-07	0.056	0.311	0.255	-0.103
-2.3327	0.035	0.237	4.69E-07	0.044	0.734	0.690	-0.202
-0.3473	0.035	0.106	6.21E-06	0.081	0.219	0.138	-0.071
-1.2581	0.035	0.207	9.67E-06	0.040	0.493	0.453	-0.172
-0.2911	0.035	0.178	1.37E-04	0.030	0.188	0.159	-0.143
-0.2872	0.030	0.120	1.76E-35	0.041	0.175	0.135	-0.090
-0.6252	0.030	0.146	8.26E-28	0.042	0.264	0.222	-0.116
-0.3681	0.030	0.118	9.83E-28	0.053	0.215	0.162	-0.088
-0.4696	0.030	0.163	9.22E-24	0.075	0.289	0.214	-0.133

-0.2925	0.030	0.102	2.14E-20	0.020	0.138	0.119	-0.072
-0.2616	0.030	0.110	8.84E-16	0.030	0.140	0.109	-0.080
-0.8740	0.030	0.131	2.19E-14	0.034	0.316	0.282	-0.101
-0.8125	0.030	0.149	2.70E-14	0.059	0.333	0.274	-0.119
-0.2660	0.030	0.143	2.54E-12	0.042	0.183	0.141	-0.113
-0.4366	0.030	0.274	1.26E-09	0.023	0.293	0.269	-0.244
-0.4191	0.030	0.247	1.10E-07	0.042	0.288	0.246	-0.217
-0.4088	0.030	0.125	5.27E-06	0.040	0.205	0.165	-0.095
-0.3460	0.030	0.181	2.35E-05	0.031	0.212	0.181	-0.151
-0.3198	0.030	0.236	1.44E-04	0.038	0.244	0.206	-0.206
-1.1823	0.025	0.229	9.85E-47	0.040	0.469	0.429	-0.204
-0.6460	0.025	0.235	2.88E-35	0.031	0.349	0.318	-0.210
-0.8981	0.025	0.305	3.37E-35	0.038	0.516	0.478	-0.280
-0.3927	0.025	0.136	4.15E-32	0.062	0.244	0.182	-0.111
-0.5060	0.025	0.162	5.61E-24	0.023	0.227	0.204	-0.137
-0.2896	0.025	0.140	1.34E-18	0.030	0.164	0.134	-0.115
-0.4174	0.025	0.134	4.40E-17	0.018	0.185	0.167	-0.109
-0.6951	0.025	0.136	1.64E-15	0.045	0.300	0.255	-0.111
-0.6304	0.025	0.128	2.94E-15	0.041	0.278	0.237	-0.103
-0.3472	0.025	0.113	2.96E-12	0.026	0.161	0.135	-0.088
-0.7483	0.025	0.124	2.01E-11	0.034	0.262	0.227	-0.099
-0.3090	0.025	0.178	3.06E-11	0.026	0.194	0.168	-0.153
-0.3673	0.025	0.195	3.77E-07	0.016	0.209	0.193	-0.170
-0.3718	0.025	0.198	7.80E-04	0.022	0.222	0.200	-0.173
-0.5854	0.020	0.177	1.43E-48	0.035	0.293	0.258	-0.157
-0.5430	0.020	0.154	6.45E-31	0.020	0.236	0.216	-0.134
-0.9361	0.020	0.158	3.04E-25	0.035	0.346	0.310	-0.138
-0.8952	0.020	0.143	1.43E-16	0.035	0.341	0.307	-0.123
-0.4955	0.020	0.124	1.54E-13	0.022	0.204	0.181	-0.104
-0.2833	0.020	0.109	2.65E-13	0.036	0.164	0.128	-0.089
-0.6047	0.020	0.124	1.81E-12	0.041	0.268	0.227	-0.104
-0.6920	0.020	0.274	3.33E-11	0.016	0.374	0.358	-0.254
-0.6033	0.020	0.109	1.07E-08	0.044	0.238	0.194	-0.089
-0.5370	0.020	0.106	4.18E-08	0.032	0.219	0.187	-0.086
-0.2551	0.020	0.186	9.54E-06	0.025	0.190	0.165	-0.166
-0.6150	0.020	0.187	1.56E-05	0.014	0.283	0.269	-0.167
-0.3022	0.020	0.178	4.49E-05	0.019	0.184	0.166	-0.158
-0.2747	0.020	0.181	1.16E-04	0.013	0.175	0.163	-0.161
-0.2768	0.020	0.173	1.29E-04	0.015	0.170	0.155	-0.153
-0.6875	0.020	0.169	4.30E-04	0.015	0.292	0.277	-0.149
-0.4354	0.020	0.107	1.16E-03	0.020	0.186	0.165	-0.087
-0.2764	0.020	0.191	8.16E-03	0.014	0.183	0.169	-0.171
-0.5476	0.015	0.148	2.67E-41	0.027	0.241	0.214	-0.133
-0.3529	0.015	0.139	5.94E-38	0.025	0.188	0.163	-0.124
-0.2561	0.015	0.105	6.81E-30	0.036	0.156	0.120	-0.090
-0.8052	0.015	0.158	2.67E-29	0.042	0.348	0.307	-0.143
-0.3693	0.015	0.126	3.02E-29	0.016	0.175	0.159	-0.111

-0.5490	0.015	0.107	6.48E-21	0.004	0.200	0.196	-0.092
-1.8194	0.015	0.275	1.36E-17	0.029	0.773	0.744	-0.260
-0.6596	0.015	0.121	2.01E-17	0.022	0.254	0.232	-0.106
-0.7395	0.015	0.127	6.87E-17	0.032	0.282	0.250	-0.112
-0.7163	0.015	0.111	7.90E-15	0.016	0.245	0.229	-0.096
-0.3747	0.015	0.222	8.51E-11	0.011	0.229	0.219	-0.207
-0.5683	0.015	0.233	3.82E-09	0.010	0.304	0.294	-0.218
-0.3266	0.015	0.141	1.28E-05	0.006	0.163	0.157	-0.126
-0.9755	0.015	0.150	5.77E-03	0.033	0.352	0.319	-0.135
-0.2620	0.015	0.121	8.53E-03	0.012	0.141	0.129	-0.106
-0.6498	0.010	0.182	3.82E-40	0.019	0.306	0.287	-0.172
-0.7089	0.010	0.234	3.27E-37	0.012	0.354	0.342	-0.224
-0.7824	0.010	0.132	3.67E-18	0.013	0.287	0.274	-0.122
-0.2739	0.010	0.114	7.18E-18	0.011	0.131	0.120	-0.104
-0.7521	0.010	0.111	1.15E-11	0.024	0.252	0.228	-0.101
-0.2948	0.010	0.129	1.99E-10	0.008	0.145	0.137	-0.119
-2.5084	0.010	0.185	1.71E-09	0.017	0.529	0.512	-0.175
-1.0988	0.010	0.181	2.27E-06	0.007	0.393	0.386	-0.171
-0.8930	0.010	0.159	3.40E-06	0.013	0.302	0.289	-0.149
-1.0995	0.010	0.130	4.01E-05	0.008	0.313	0.306	-0.120
-0.8850	0.010	0.161	4.84E-05	0.016	0.353	0.337	-0.151
-0.4730	0.010	0.110	3.85E-04	0.014	0.166	0.152	-0.100
-0.3966	0.010	0.135	4.29E-04	0.006	0.187	0.181	-0.125
-0.6319	0.005	0.174	1.83E-59	0.002	0.269	0.267	-0.169
-0.3170	0.005	0.136	5.33E-46	0.015	0.179	0.165	-0.131
-0.6721	0.005	0.110	1.14E-25	0.001	0.226	0.225	-0.105
-0.2560	0.005	0.116	2.73E-21	0.004	0.130	0.126	-0.111
-0.2757	0.005	0.117	1.40E-19	0.002	0.137	0.136	-0.112
-0.3389	0.005	0.108	1.22E-07	0.001	0.142	0.141	-0.103
-0.3649	0.005	0.121	1.47E-06	0.004	0.161	0.158	-0.116
-1.0602	0.005	0.141	3.31E-05	0.003	0.342	0.339	-0.136
-0.2791	0.000	0.110	8.84E-15	0.000	0.133	0.133	-0.110
-0.6360	0.000	0.127	1.30E-13	0.000	0.236	0.236	-0.127
-1.0946	0.000	0.107	1.33E-11	0.000	0.290	0.290	-0.107
-0.5824	0.000	0.118	3.61E-10	0.000	0.215	0.215	-0.118
-0.6307	0.000	0.134	4.86E-09	0.000	0.229	0.229	-0.134
-0.9309	0.000	0.164	4.12E-08	0.000	0.341	0.341	-0.164
-1.2338	0.000	0.147	4.81E-08	0.000	0.386	0.386	-0.147
-0.4059	0.000	0.162	6.63E-08	0.000	0.207	0.207	-0.162
-0.2879	0.000	0.128	1.04E-07	0.000	0.147	0.147	-0.128
-0.6007	0.000	0.125	1.19E-05	0.000	0.217	0.217	-0.125
-0.2672	0.000	0.125	2.68E-05	0.000	0.139	0.139	-0.125
-0.2798	0.000	0.105	1.00E-04	0.000	0.133	0.133	-0.105
-0.4945	0.000	0.111	1.40E-04	0.000	0.195	0.195	-0.111
-0.3109	0.000	0.105	5.54E-04	0.000	0.143	0.143	-0.105
-0.3044	0.000	0.117	1.55E-03	0.000	0.147	0.147	-0.117

cluster	gene	description	p_val
10	MGP	matrix Gla protein [Source:HGNC Symbol;Acc:HGNC:7060]	1.65E-263
10	KRT19	keratin 19 [Source:HGNC Symbol;Acc:HGNC:6436]	6.02E-230
10	KRT18	keratin 18 [Source:HGNC Symbol;Acc:HGNC:6430]	2.83E-224
10	CRABP2	cellular retinoic acid binding protein 2 [Source:HGNC Symbol;Acc:HG	1.26E-219
10	KRT8	keratin 8 [Source:HGNC Symbol;Acc:HGNC:6446]	1.75E-193
10	TACSTD2	tumor associated calcium signal transducer 2 [Source:HGNC Symbol;	2.39E-198
10	ID1	inhibitor of DNA binding 1, HLH protein [Source:HGNC Symbol;Acc:H	1.92E-176
10	KRT7	keratin 7 [Source:HGNC Symbol;Acc:HGNC:6445]	6.61E-190
10	ID3	inhibitor of DNA binding 3, HLH protein [Source:HGNC Symbol;Acc:H	4.31E-156
10	ELF3	E74 like ETS transcription factor 3 [Source:HGNC Symbol;Acc:HGNC:3	5.12E-185
10	CLDN4	claudin 4 [Source:HGNC Symbol;Acc:HGNC:2046]	7.31E-181
10	FXYD3	FXYD domain containing ion transport regulator 3 [Source:HGNC Sym	1.56E-151
10	SLC3A1	solute carrier family 3 member 1 [Source:HGNC Symbol;Acc:HGNC:1	1.20E-133
10	HSPA5	heat shock protein family A (Hsp70) member 5 [Source:HGNC Symbo	4.07E-133
10	AGR2	anterior gradient 2, protein disulphide isomerase family member [So	6.33E-133
10	RHOB	ras homolog family member B [Source:HGNC Symbol;Acc:HGNC:668]	4.59E-112
10	TSPAN1	tetraspanin 1 [Source:HGNC Symbol;Acc:HGNC:20657]	1.71E-163
10	SCCPDH	saccharopine dehydrogenase (putative) [Source:HGNC Symbol;Acc:H	4.53E-145
10	ID4	inhibitor of DNA binding 4, HLH protein [Source:HGNC Symbol;Acc:H	6.21E-126
10	TM4SF1	transmembrane 4 L six family member 1 [Source:HGNC Symbol;Acc:	6.30E-118
10	PLK2	polo like kinase 2 [Source:HGNC Symbol;Acc:HGNC:19699]	1.13E-115
10	NPW	neuropeptide W [Source:HGNC Symbol;Acc:HGNC:30509]	2.18E-118
10	CAMK2N1	calcium/calmodulin dependent protein kinase II inhibitor 1 [Source:H	5.46E-116
10	SLC9A3R2	SLC9A3 regulator 2 [Source:HGNC Symbol;Acc:HGNC:11076]	3.09E-122
10	S100A13	S100 calcium binding protein A13 [Source:HGNC Symbol;Acc:HGNC:1	5.88E-128
10	TFF1	trefoil factor 1 [Source:HGNC Symbol;Acc:HGNC:11755]	5.49E-75
10	CLDN7	claudin 7 [Source:HGNC Symbol;Acc:HGNC:2049]	3.29E-127
10	ATF3	activating transcription factor 3 [Source:HGNC Symbol;Acc:HGNC:78	2.42E-70
10	STARD10	StAR related lipid transfer domain containing 10 [Source:HGNC Symb	1.24E-133
10	HSPB1	heat shock protein family B (small) member 1 [Source:HGNC Symbol	7.96E-125
10	S100A16	S100 calcium binding protein A16 [Source:HGNC Symbol;Acc:HGNC:2	1.87E-130
10	ANXA2	annexin A2 [Source:HGNC Symbol;Acc:HGNC:537]	2.33E-93
10	EPHX1	epoxide hydrolase 1 [Source:HGNC Symbol;Acc:HGNC:3401]	3.69E-103
10	S100A14	S100 calcium binding protein A14 [Source:HGNC Symbol;Acc:HGNC:1	5.07E-118
10	EPCAM	epithelial cell adhesion molecule [Source:HGNC Symbol;Acc:HGNC:1	5.54E-133
10	S100A6	S100 calcium binding protein A6 [Source:HGNC Symbol;Acc:HGNC:10	8.18E-107
10	RHOV	ras homolog family member V [Source:HGNC Symbol;Acc:HGNC:183	3.93E-123
10	NUPR1	nuclear protein 1, transcriptional regulator [Source:HGNC Symbol;Ac	2.48E-128
10	S100A11	S100 calcium binding protein A11 [Source:HGNC Symbol;Acc:HGNC:1	1.06E-95
10	CCND1	cyclin D1 [Source:HGNC Symbol;Acc:HGNC:1582]	6.48E-127
10	MLPH	melanophilin [Source:HGNC Symbol;Acc:HGNC:29643]	5.34E-125
10	CD24	CD24 molecule [Source:HGNC Symbol;Acc:HGNC:1645]	2.94E-86
10	S100P	S100 calcium binding protein P [Source:HGNC Symbol;Acc:HGNC:105	9.26E-79
10	PHLDA2	pleckstrin homology like domain family A member 2 [Source:HGNC S	2.66E-56
10	GCHFR	GTP cyclohydrolase I feedback regulator [Source:HGNC Symbol;Acc:	2.83E-90

10	LRRC26	leucine rich repeat containing 26 [Source:HGNC Symbol;Acc:HGNC:3	1.53E-82
10	SLC12A2	solute carrier family 12 member 2 [Source:HGNC Symbol;Acc:HGNC:	7.56E-78
10	SPTSSB	serine palmitoyltransferase small subunit B [Source:HGNC Symbol;Ac	4.56E-76
10	WFDC2	WAP four-disulfide core domain 2 [Source:HGNC Symbol;Acc:HGNC:1	6.14E-99
10	CRACR2B	calcium release activated channel regulator 2B [Source:HGNC Symbo	6.79E-87
10	ZG16B	zymogen granule protein 16B [Source:HGNC Symbol;Acc:HGNC:3045	7.52E-75
10	BAMBI	BMP and activin membrane bound inhibitor [Source:HGNC Symbol;A	2.06E-38
10	IER2	immediate early response 2 [Source:HGNC Symbol;Acc:HGNC:28871	1.27E-56
10	DNAJC1	DnaJ heat shock protein family (Hsp40) member C1 [Source:HGNC Sy	1.41E-75
10	GSTP1	glutathione S-transferase pi 1 [Source:HGNC Symbol;Acc:HGNC:4638	8.34E-57
10	TMEM141	transmembrane protein 141 [Source:HGNC Symbol;Acc:HGNC:28211	9.52E-92
10	CYB5A	cytochrome b5 type A [Source:HGNC Symbol;Acc:HGNC:2570]	2.90E-73
10	PRDX1	peroxiredoxin 1 [Source:HGNC Symbol;Acc:HGNC:9352]	4.98E-72
10	AZGP1	alpha-2-glycoprotein 1, zinc-binding [Source:HGNC Symbol;Acc:HGN	1.83E-96
10	CCDC190	coiled-coil domain containing 190 [Source:HGNC Symbol;Acc:HGNC:2	1.41E-85
10	HES4	hes family bHLH transcription factor 4 [Source:HGNC Symbol;Acc:HG	4.22E-48
10	RCAN1	regulator of calcineurin 1 [Source:HGNC Symbol;Acc:HGNC:3040]	1.73E-81
10	TFF3	trefoil factor 3 [Source:HGNC Symbol;Acc:HGNC:11757]	1.38E-77
10	HERPUD1	homocysteine inducible ER protein with ubiquitin like domain 1 [Sou	4.28E-48
10	MARCKSL1	MARCKS like 1 [Source:HGNC Symbol;Acc:HGNC:7142]	3.68E-51
10	SMIM22	small integral membrane protein 22 [Source:HGNC Symbol;Acc:HGN	9.23E-86
10	CLDN3	claudin 3 [Source:HGNC Symbol;Acc:HGNC:2045]	9.83E-89
10	CAPS	calcyphosine [Source:HGNC Symbol;Acc:HGNC:1487]	1.52E-54
10	ALOX15B	arachidonate 15-lipoxygenase type B [Source:HGNC Symbol;Acc:HGN	2.68E-87
10	GATA3	GATA binding protein 3 [Source:HGNC Symbol;Acc:HGNC:4172]	6.87E-71
10	MGST3	microsomal glutathione S-transferase 3 [Source:HGNC Symbol;Acc:H	1.54E-98
10	TSTD1	thiosulfate sulfurtransferase like domain containing 1 [Source:HGNC	9.25E-90
10	SPDEF	SAM pointed domain containing ETS transcription factor [Source:HG	1.84E-86
10	DAPL1	death associated protein like 1 [Source:HGNC Symbol;Acc:HGNC:214	1.69E-73
10	UQCRQ	ubiquinol-cytochrome c reductase complex III subunit VII [Source:HG	3.41E-73
10	PPP1R14B	protein phosphatase 1 regulatory inhibitor subunit 14B [Source:HGN	8.77E-52
10	KRT23	keratin 23 [Source:HGNC Symbol;Acc:HGNC:6438]	1.36E-63
10	RAB13	RAB13, member RAS oncogene family [Source:HGNC Symbol;Acc:HG	4.23E-58
10	RAB25	RAB25, member RAS oncogene family [Source:HGNC Symbol;Acc:HG	2.95E-79
10	PERP	p53 apoptosis effector related to PMP22 [Source:HGNC Symbol;Acc:	7.23E-82
10	DSP	desmoplakin [Source:HGNC Symbol;Acc:HGNC:3052]	1.51E-70
10	PEBP1	phosphatidylethanolamine binding protein 1 [Source:HGNC Symbol;A	2.92E-62
10	PAFAH1B3	platelet activating factor acetylhydrolase 1b catalytic subunit 3 [Sour	5.24E-71
10	TMEM176B	transmembrane protein 176B [Source:HGNC Symbol;Acc:HGNC:2959	6.20E-50
10	DSTN	destrin, actin depolymerizing factor [Source:HGNC Symbol;Acc:HGN	3.08E-101
10	EDN1	endothelin 1 [Source:HGNC Symbol;Acc:HGNC:3176]	2.61E-32
10	CD63	CD63 molecule [Source:HGNC Symbol;Acc:HGNC:1692]	7.50E-74
10	TNFSF10	TNF superfamily member 10 [Source:HGNC Symbol;Acc:HGNC:11925	4.63E-34
10	HES1	hes family bHLH transcription factor 1 [Source:HGNC Symbol;Acc:HG	1.90E-37
10	PITX1	paired like homeodomain 1 [Source:HGNC Symbol;Acc:HGNC:9004]	1.67E-82
10	HSP90B1	heat shock protein 90 beta family member 1 [Source:HGNC Symbol;A	2.35E-39
10	NME1	NME/NM23 nucleoside diphosphate kinase 1 [Source:HGNC Symbol;	1.43E-60

10	DEGS1	delta 4-desaturase, sphingolipid 1 [Source:HGNC Symbol;Acc:HGNC:1	2.62E-77
10	EMP2	epithelial membrane protein 2 [Source:HGNC Symbol;Acc:HGNC:333	1.30E-76
10	RAB11FIP1	RAB11 family interacting protein 1 [Source:HGNC Symbol;Acc:HGNC:	1.11E-29
10	FBP1	fructose-bisphosphatase 1 [Source:HGNC Symbol;Acc:HGNC:3606]	8.43E-49
10	LGALS3	galectin 3 [Source:HGNC Symbol;Acc:HGNC:6563]	1.85E-48
10	BST2	bone marrow stromal cell antigen 2 [Source:HGNC Symbol;Acc:HGN(	1.28E-33
10	TFAP2B	transcription factor AP-2 beta [Source:HGNC Symbol;Acc:HGNC:1174	6.20E-70
10	BTG2	BTG anti-proliferation factor 2 [Source:HGNC Symbol;Acc:HGNC:113	1.76E-27
10	TMEM205	transmembrane protein 205 [Source:HGNC Symbol;Acc:HGNC:29631	6.47E-47
10	SPINT2	serine peptidase inhibitor, Kunitz type 2 [Source:HGNC Symbol;Acc:H	3.09E-78
10	CCL28	C-C motif chemokine ligand 28 [Source:HGNC Symbol;Acc:HGNC:177	4.94E-46
10	SPINT1	serine peptidase inhibitor, Kunitz type 1 [Source:HGNC Symbol;Acc:H	5.32E-61
10	CREB3L4	cAMP responsive element binding protein 3 like 4 [Source:HGNC Sym	2.90E-57
10	CD9	CD9 molecule [Source:HGNC Symbol;Acc:HGNC:1709]	1.81E-55
10	PYDC1	pyrin domain containing 1 [Source:HGNC Symbol;Acc:HGNC:30261]	2.71E-43
10	TRPS1	transcriptional repressor GATA binding 1 [Source:HGNC Symbol;Acc:	9.27E-60
10	TMEM176A	transmembrane protein 176A [Source:HGNC Symbol;Acc:HGNC:2493	6.59E-38
10	CITED4	Cbp/p300 interacting transactivator with Glu/Asp rich carboxy-termi	2.37E-50
10	GALNT7	polypeptide N-acetylgalactosaminyltransferase 7 [Source:HGNC Sym	1.65E-55
10	ATP1B1	ATPase Na+/K+ transporting subunit beta 1 [Source:HGNC Symbol;A(	6.25E-42
10	COX6A1	cytochrome c oxidase subunit 6A1 [Source:HGNC Symbol;Acc:HGNC:	2.79E-73
10	LAGE3	L antigen family member 3 [Source:HGNC Symbol;Acc:HGNC:26058]	1.83E-65
10	NDUFA2	NADH:ubiquinone oxidoreductase subunit A2 [Source:HGNC Symbol;	3.47E-64
10	PTMS	parathymosin [Source:HGNC Symbol;Acc:HGNC:9629]	6.07E-42
10	MRPL51	mitochondrial ribosomal protein L51 [Source:HGNC Symbol;Acc:HGN	7.00E-63
10	COX8A	cytochrome c oxidase subunit 8A [Source:HGNC Symbol;Acc:HGNC:2	1.68E-65
10	SOX9	SRY-box transcription factor 9 [Source:HGNC Symbol;Acc:HGNC:112(	1.39E-65
10	NPDC1	neural proliferation, differentiation and control 1 [Source:HGNC Sym	9.11E-32
10	IER3	immediate early response 3 [Source:HGNC Symbol;Acc:HGNC:5392]	1.17E-30
10	NR2F2	nuclear receptor subfamily 2 group F member 2 [Source:HGNC Symb	6.46E-62
10	TMEM238	transmembrane protein 238 [Source:HGNC Symbol;Acc:HGNC:40042	3.64E-40
10	IRS4	insulin receptor substrate 4 [Source:HGNC Symbol;Acc:HGNC:6128]	1.20E-72
10	MGST1	microsomal glutathione S-transferase 1 [Source:HGNC Symbol;Acc:H	1.67E-46
10	TXNDC17	thioredoxin domain containing 17 [Source:HGNC Symbol;Acc:HGNC:2	1.49E-46
10	NDUFS6	NADH:ubiquinone oxidoreductase subunit S6 [Source:HGNC Symbol;	5.07E-49
10	SELENOW	selenoprotein W [Source:HGNC Symbol;Acc:HGNC:10752]	1.52E-56
10	NQO1	NAD(P)H quinone dehydrogenase 1 [Source:HGNC Symbol;Acc:HGN(	8.61E-58
10	DHCR24	24-dehydrocholesterol reductase [Source:HGNC Symbol;Acc:HGNC:2	2.71E-45
10	NDUFB2	NADH:ubiquinone oxidoreductase subunit B2 [Source:HGNC Symbol;	3.94E-39
10	TXN	thioredoxin [Source:HGNC Symbol;Acc:HGNC:12435]	9.39E-44
10	NDUFB7	NADH:ubiquinone oxidoreductase subunit B7 [Source:HGNC Symbol;	2.66E-53
10	HSPE1	heat shock protein family E (Hsp10) member 1 [Source:HGNC Symbo	1.73E-26
10	COA3	cytochrome c oxidase assembly factor 3 [Source:HGNC Symbol;Acc:H	8.66E-58
10	IER5	immediate early response 5 [Source:HGNC Symbol;Acc:HGNC:5393]	6.19E-24
10	EFHD1	EF-hand domain family member D1 [Source:HGNC Symbol;Acc:HGN(	2.06E-59
10	REEP5	receptor accessory protein 5 [Source:HGNC Symbol;Acc:HGNC:3007	8.79E-51
10	PLPP2	phospholipid phosphatase 2 [Source:HGNC Symbol;Acc:HGNC:9230]	7.61E-56

10	NANS	N-acetylneuraminate synthase [Source:HGNC Symbol;Acc:HGNC:192	1.19E-28
10	PRSS8	serine protease 8 [Source:HGNC Symbol;Acc:HGNC:9491]	4.11E-60
10	HSP90AB1	heat shock protein 90 alpha family class B member 1 [Source:HGNC S	3.65E-36
10	TMEM59	transmembrane protein 59 [Source:HGNC Symbol;Acc:HGNC:1239]	1.81E-61
10	NDUFB4	NADH:ubiquinone oxidoreductase subunit B4 [Source:HGNC Symbol;	5.08E-59
10	HEBP2	heme binding protein 2 [Source:HGNC Symbol;Acc:HGNC:15716]	1.30E-40
10	DBI	diazepam binding inhibitor, acyl-CoA binding protein [Source:HGNC S	9.24E-34
10	SPINT1-AS1	SPINT1 antisense RNA 1 [Source:HGNC Symbol;Acc:HGNC:53162]	1.35E-51
10	BLVRB	biliverdin reductase B [Source:HGNC Symbol;Acc:HGNC:1063]	1.16E-29
10	COX5B	cytochrome c oxidase subunit 5B [Source:HGNC Symbol;Acc:HGNC:2	1.36E-52
10	XBP1	X-box binding protein 1 [Source:HGNC Symbol;Acc:HGNC:12801]	8.45E-20
10	ATOX1	antioxidant 1 copper chaperone [Source:HGNC Symbol;Acc:HGNC:79	1.72E-38
10	SERINC2	serine incorporator 2 [Source:HGNC Symbol;Acc:HGNC:23231]	1.67E-58
10	RND3	Rho family GTPase 3 [Source:HGNC Symbol;Acc:HGNC:671]	4.48E-47
10	NUPR2	nuclear protein 2, transcriptional regulator [Source:HGNC Symbol;Ac	9.21E-52
10	ENO1	enolase 1 [Source:HGNC Symbol;Acc:HGNC:3350]	2.98E-49
10	HMGCS2	3-hydroxy-3-methylglutaryl-CoA synthase 2 [Source:HGNC Symbol;A	1.33E-53
10	PYCARD	PYD and CARD domain containing [Source:HGNC Symbol;Acc:HGNC:1	7.60E-22
10	TNFRSF12A	TNF receptor superfamily member 12A [Source:HGNC Symbol;Acc:H	1.45E-35
10	JTB	jumping translocation breakpoint [Source:HGNC Symbol;Acc:HGNC:6	7.82E-63
10	SAT1	spermidine/spermine N1-acetyltransferase 1 [Source:HGNC Symbol;	2.34E-43
10	FABP5	fatty acid binding protein 5 [Source:HGNC Symbol;Acc:HGNC:3560]	2.17E-20
10	TMEM14C	transmembrane protein 14C [Source:HGNC Symbol;Acc:HGNC:20952	4.39E-34
10	UFC1	ubiquitin-fold modifier conjugating enzyme 1 [Source:HGNC Symbol;	1.23E-33
10	SELENBP1	selenium binding protein 1 [Source:HGNC Symbol;Acc:HGNC:10719]	5.04E-46
10	CAPG	capping actin protein, gelsolin like [Source:HGNC Symbol;Acc:HGNC:	5.00E-23
10	LAMTOR2	late endosomal/lysosomal adaptor, MAPK and MTOR activator 2 [So	5.17E-36
10	CITED1	Cbp/p300 interacting transactivator with Glu/Asp rich carboxy-termi	3.60E-45
10	CPE	carboxypeptidase E [Source:HGNC Symbol;Acc:HGNC:2303]	3.84E-53
10	TIMM10	translocase of inner mitochondrial membrane 10 [Source:HGNC Sym	1.42E-45
10	SLC25A5	solute carrier family 25 member 5 [Source:HGNC Symbol;Acc:HGNC:	1.59E-32
10	OAT	ornithine aminotransferase [Source:HGNC Symbol;Acc:HGNC:8091]	1.03E-38
10	POLR2L	RNA polymerase II, I and III subunit L [Source:HGNC Symbol;Acc:HGN	1.28E-37
10	C1orf122	chromosome 1 open reading frame 122 [Source:HGNC Symbol;Acc:H	3.17E-40
10	PRDX2	peroxiredoxin 2 [Source:HGNC Symbol;Acc:HGNC:9353]	2.26E-29
10	TMEM54	transmembrane protein 54 [Source:HGNC Symbol;Acc:HGNC:24143]	7.24E-48
10	SMIM14	small integral membrane protein 14 [Source:HGNC Symbol;Acc:HGN	1.02E-33
10	QSOX1	quiescin sulfhydryl oxidase 1 [Source:HGNC Symbol;Acc:HGNC:9756]	4.06E-37
10	COX7A2	cytochrome c oxidase subunit 7A2 [Source:HGNC Symbol;Acc:HGNC:	4.09E-54
10	VAMP8	vesicle associated membrane protein 8 [Source:HGNC Symbol;Acc:H	1.20E-35
10	HACD3	3-hydroxyacyl-CoA dehydratase 3 [Source:HGNC Symbol;Acc:HGNC:2	7.24E-34
10	NT5DC2	5'-nucleotidase domain containing 2 [Source:HGNC Symbol;Acc:HGN	1.76E-49
10	NAXE	NAD(P)HX epimerase [Source:HGNC Symbol;Acc:HGNC:18453]	3.14E-39
10	ENAH	ENAH actin regulator [Source:HGNC Symbol;Acc:HGNC:18271]	1.39E-46
10	PRDX4	peroxiredoxin 4 [Source:HGNC Symbol;Acc:HGNC:17169]	2.38E-51
10	DDAH2	dimethylarginine dimethylaminohydrolase 2 [Source:HGNC Symbol;A	1.09E-33
10	DEFB1	defensin beta 1 [Source:HGNC Symbol;Acc:HGNC:2766]	1.05E-43

10	COX7B	cytochrome c oxidase subunit 7B [Source:HGNC Symbol;Acc:HGNC:2	5.18E-40
10	MDH2	malate dehydrogenase 2 [Source:HGNC Symbol;Acc:HGNC:6971]	2.49E-28
10	MZB1	marginal zone B and B1 cell specific protein [Source:HGNC Symbol;A	7.82E-31
10	CHCHD2	coiled-coil-helix-coiled-coil-helix domain containing 2 [Source:HGNC	9.59E-47
10	NDUFAB1	NADH:ubiquinone oxidoreductase subunit AB1 [Source:HGNC Symbo	1.61E-30
10	CENPF	centromere protein F [Source:HGNC Symbol;Acc:HGNC:1857]	3.28E-17
10	ATP5MC3	ATP synthase membrane subunit c locus 3 [Source:HGNC Symbol;Ac	2.73E-30
10	NDUFS8	NADH:ubiquinone oxidoreductase core subunit S8 [Source:HGNC Sy	7.51E-34
10	GADD45B	growth arrest and DNA damage inducible beta [Source:HGNC Symbo	1.00E-10
10	RHOC	ras homolog family member C [Source:HGNC Symbol;Acc:HGNC:669	7.33E-25
10	SLC44A4	solute carrier family 44 member 4 [Source:HGNC Symbol;Acc:HGNC:	1.64E-45
10	SDF2L1	stromal cell derived factor 2 like 1 [Source:HGNC Symbol;Acc:HGNC:	4.92E-22
10	PMVK	phosphomevalonate kinase [Source:HGNC Symbol;Acc:HGNC:9141]	2.28E-39
10	MRPL41	mitochondrial ribosomal protein L41 [Source:HGNC Symbol;Acc:HGN	4.82E-31
10	NR4A1	nuclear receptor subfamily 4 group A member 1 [Source:HGNC Symb	1.22E-11
10	S100A1	S100 calcium binding protein A1 [Source:HGNC Symbol;Acc:HGNC:1	5.54E-49
10	NDUFB3	NADH:ubiquinone oxidoreductase subunit B3 [Source:HGNC Symbol;	1.57E-44
10	CALR	calreticulin [Source:HGNC Symbol;Acc:HGNC:1455]	1.30E-23
10	COL9A2	collagen type IX alpha 2 chain [Source:HGNC Symbol;Acc:HGNC:2218	2.66E-32
10	FAM162A	family with sequence similarity 162 member A [Source:HGNC Symbo	1.18E-28
10	TCEAL4	transcription elongation factor A like 4 [Source:HGNC Symbol;Acc:H	5.14E-32
10	NENF	neudesin neurotrophic factor [Source:HGNC Symbol;Acc:HGNC:3038	4.39E-40
10	TUBB4B	tubulin beta 4B class IVb [Source:HGNC Symbol;Acc:HGNC:20771]	6.70E-40
10	CYP4Z1	cytochrome P450 family 4 subfamily Z member 1 [Source:HGNC Sym	2.65E-48
10	GUK1	guanylate kinase 1 [Source:HGNC Symbol;Acc:HGNC:4693]	7.00E-35
10	CKS1B	CDC28 protein kinase regulatory subunit 1B [Source:HGNC Symbol;A	2.07E-27
10	SDC1	syndecan 1 [Source:HGNC Symbol;Acc:HGNC:10658]	1.67E-39
10	COX20	cytochrome c oxidase assembly factor COX20 [Source:HGNC Symbol	1.83E-29
10	BMP5	bone morphogenetic protein 5 [Source:HGNC Symbol;Acc:HGNC:107	1.87E-53
10	SCUBE2	signal peptide, CUB domain and EGF like domain containing 2 [Sourc	4.34E-34
10	TMED3	transmembrane p24 trafficking protein 3 [Source:HGNC Symbol;Acc:	8.80E-31
10	MFSD10	major facilitator superfamily domain containing 10 [Source:HGNC Sy	6.81E-31
10	IDH2	isocitrate dehydrogenase (NADP(+)) 2 [Source:HGNC Symbol;Acc:HG	2.84E-39
10	EGR1	early growth response 1 [Source:HGNC Symbol;Acc:HGNC:3238]	1.13E-25
10	ENSA	endosulfine alpha [Source:HGNC Symbol;Acc:HGNC:3360]	1.90E-36
10	PPIA	peptidylprolyl isomerase A [Source:HGNC Symbol;Acc:HGNC:9253]	2.01E-46
10	SRP9	signal recognition particle 9 [Source:HGNC Symbol;Acc:HGNC:11304	1.41E-46
10	DPP7	dipeptidyl peptidase 7 [Source:HGNC Symbol;Acc:HGNC:14892]	1.26E-27
10	P4HB	prolyl 4-hydroxylase subunit beta [Source:HGNC Symbol;Acc:HGNC:8	1.95E-29
10	SNRPE	small nuclear ribonucleoprotein polypeptide E [Source:HGNC Symbo	2.42E-35
10	GMNN	geminin DNA replication inhibitor [Source:HGNC Symbol;Acc:HGNC:1	2.48E-23
10	ATP5F1B	ATP synthase F1 subunit beta [Source:HGNC Symbol;Acc:HGNC:830]	2.91E-25
10	GIPC1	GIPC PDZ domain containing family member 1 [Source:HGNC Symbo	1.64E-32
10	ECI1	enoyl-CoA delta isomerase 1 [Source:HGNC Symbol;Acc:HGNC:2703]	2.39E-31
10	AC008771.1	novel transcript	1.82E-40
10	GAPDH	glyceraldehyde-3-phosphate dehydrogenase [Source:HGNC Symbol;	6.82E-32
10	COX5A	cytochrome c oxidase subunit 5A [Source:HGNC Symbol;Acc:HGNC:2	1.06E-14

10	NDUFS7	NADH:ubiquinone oxidoreductase core subunit S7 [Source:HGNC Syr	6.71E-20
10	NUDT8	nudix hydrolase 8 [Source:HGNC Symbol;Acc:HGNC:8055]	4.08E-30
10	LAPTM4A	lysosomal protein transmembrane 4 alpha [Source:HGNC Symbol;Ac	1.35E-29
10	RNF181	ring finger protein 181 [Source:HGNC Symbol;Acc:HGNC:28037]	1.77E-28
10	DUSP23	dual specificity phosphatase 23 [Source:HGNC Symbol;Acc:HGNC:21	3.24E-28
10	MYDGF	myeloid derived growth factor [Source:HGNC Symbol;Acc:HGNC:169	5.00E-18
10	ELOB	elongin B [Source:HGNC Symbol;Acc:HGNC:11619]	2.83E-33
10	ZC3H12A	zinc finger CCCH-type containing 12A [Source:HGNC Symbol;Acc:HGN	2.18E-28
10	AURKAIP1	aurora kinase A interacting protein 1 [Source:HGNC Symbol;Acc:HGN	9.50E-35
10	DCXR	dicarbonyl and L-xylulose reductase [Source:HGNC Symbol;Acc:HGN	2.59E-22
10	NME3	NME/NM23 nucleoside diphosphate kinase 3 [Source:HGNC Symbol;	1.10E-20
10	ATP6V0B	ATPase H+ transporting V0 subunit b [Source:HGNC Symbol;Acc:HGN	9.36E-18
10	SRI	sorcin [Source:HGNC Symbol;Acc:HGNC:11292]	2.24E-19
10	SDHC	succinate dehydrogenase complex subunit C [Source:HGNC Symbol;	7.76E-36
10	C19orf33	chromosome 19 open reading frame 33 [Source:HGNC Symbol;Acc:H	1.09E-28
10	CNN3	calponin 3 [Source:HGNC Symbol;Acc:HGNC:2157]	6.16E-32
10	CENPX	centromere protein X [Source:HGNC Symbol;Acc:HGNC:11422]	2.54E-21
10	IL18	interleukin 18 [Source:HGNC Symbol;Acc:HGNC:5986]	3.39E-27
10	NDUFV2	NADH:ubiquinone oxidoreductase core subunit V2 [Source:HGNC Syr	6.22E-25
10	UBC	ubiquitin C [Source:HGNC Symbol;Acc:HGNC:12468]	1.18E-32
10	KDELR1	KDEL endoplasmic reticulum protein retention receptor 1 [Source:H	1.50E-22
10	NDUFS2	NADH:ubiquinone oxidoreductase core subunit S2 [Source:HGNC Syr	1.90E-31
10	GADD45GIP1	GADD45G interacting protein 1 [Source:HGNC Symbol;Acc:HGNC:29	1.73E-19
10	ECHS1	enoyl-CoA hydratase, short chain 1 [Source:HGNC Symbol;Acc:HGNC	4.77E-25
10	SELENOM	selenoprotein M [Source:HGNC Symbol;Acc:HGNC:30397]	6.79E-33
10	SIL1	SIL1 nucleotide exchange factor [Source:HGNC Symbol;Acc:HGNC:24	1.20E-32
10	PPIC	peptidylprolyl isomerase C [Source:HGNC Symbol;Acc:HGNC:9256]	1.27E-37
10	RUSC1	RUN and SH3 domain containing 1 [Source:HGNC Symbol;Acc:HGNC:	4.38E-35
10	STEAP1	STEAP family member 1 [Source:HGNC Symbol;Acc:HGNC:11378]	8.67E-43
10	C1orf43	chromosome 1 open reading frame 43 [Source:HGNC Symbol;Acc:H	6.41E-40
10	MIEN1	migration and invasion enhancer 1 [Source:HGNC Symbol;Acc:HGNC:	3.61E-23
10	COMMD3	COMM domain containing 3 [Source:HGNC Symbol;Acc:HGNC:23332	2.11E-29
10	PPA1	inorganic pyrophosphatase 1 [Source:HGNC Symbol;Acc:HGNC:9226]	5.39E-18
10	JUN	Jun proto-oncogene, AP-1 transcription factor subunit [Source:HGNC	1.45E-25
10	MIA3	MIA SH3 domain ER export factor 3 [Source:HGNC Symbol;Acc:HGNC	4.53E-29
10	FOXA1	forkhead box A1 [Source:HGNC Symbol;Acc:HGNC:5021]	1.24E-39
10	ECH1	enoyl-CoA hydratase 1 [Source:HGNC Symbol;Acc:HGNC:3149]	2.92E-26
10	GALNT6	polypeptide N-acetylgalactosaminyltransferase 6 [Source:HGNC Sym	1.68E-25
10	MLF2	myeloid leukemia factor 2 [Source:HGNC Symbol;Acc:HGNC:7126]	9.55E-31
10	HINT2	histidine triad nucleotide binding protein 2 [Source:HGNC Symbol;Ac	1.01E-26
10	DEGS2	delta 4-desaturase, sphingolipid 2 [Source:HGNC Symbol;Acc:HGNC:	1.51E-40
10	SEM1	SEM1 26S proteasome complex subunit [Source:HGNC Symbol;Acc:H	4.98E-32
10	COMT	catechol-O-methyltransferase [Source:HGNC Symbol;Acc:HGNC:222	7.04E-19
10	TIMM13	translocase of inner mitochondrial membrane 13 [Source:HGNC Sym	5.36E-19
10	DYNLT1	dynein light chain Tctex-type 1 [Source:HGNC Symbol;Acc:HGNC:116	1.98E-23
10	ATP5MC2	ATP synthase membrane subunit c locus 2 [Source:HGNC Symbol;Ac	2.31E-22
10	COX6C	cytochrome c oxidase subunit 6C [Source:HGNC Symbol;Acc:HGNC:2	7.56E-33

10	POLR2I	RNA polymerase II subunit I [Source:HGNC Symbol;Acc:HGNC:9196]	8.59E-33
10	CHMP2A	charged multivesicular body protein 2A [Source:HGNC Symbol;Acc:H	5.49E-25
10	ALKBH7	alkB homolog 7 [Source:HGNC Symbol;Acc:HGNC:21306]	2.21E-19
10	RERG	RAS like estrogen regulated growth inhibitor [Source:HGNC Symbol;A	1.83E-38
10	CD151	CD151 molecule (Raph blood group) [Source:HGNC Symbol;Acc:HGN	2.36E-35
10	PDLIM4	PDZ and LIM domain 4 [Source:HGNC Symbol;Acc:HGNC:16501]	1.32E-37
10	MRPL24	mitochondrial ribosomal protein L24 [Source:HGNC Symbol;Acc:HGN	2.81E-30
10	CYP27A1	cytochrome P450 family 27 subfamily A member 1 [Source:HGNC Sy	3.67E-25
10	AP1M2	adaptor related protein complex 1 subunit mu 2 [Source:HGNC Symb	2.53E-21
10	AREG	amphiregulin [Source:HGNC Symbol;Acc:HGNC:651]	3.80E-10
10	PEA15	proliferation and apoptosis adaptor protein 15 [Source:HGNC Symbo	5.44E-20
10	DCDC2	doublecortin domain containing 2 [Source:HGNC Symbol;Acc:HGNC:	1.71E-33
10	TMEM132A	transmembrane protein 132A [Source:HGNC Symbol;Acc:HGNC:310	1.09E-38
10	STMND1	stathmin domain containing 1 [Source:HGNC Symbol;Acc:HGNC:446	4.44E-37
10	MRPL55	mitochondrial ribosomal protein L55 [Source:HGNC Symbol;Acc:HGN	1.06E-28
10	NDUFC1	NADH:ubiquinone oxidoreductase subunit C1 [Source:HGNC Symbol;	4.20E-34
10	EIF4EBP1	eukaryotic translation initiation factor 4E binding protein 1 [Source:H	1.98E-16
10	RRBP1	ribosome binding protein 1 [Source:HGNC Symbol;Acc:HGNC:10448]	4.01E-12
10	METRN	meteorin, glial cell differentiation regulator [Source:HGNC Symbol;A	1.35E-25
10	SRD5A3	steroid 5 alpha-reductase 3 [Source:HGNC Symbol;Acc:HGNC:25812]	8.71E-36
10	GNG5	G protein subunit gamma 5 [Source:HGNC Symbol;Acc:HGNC:4408]	2.30E-20
10	TGIF1	TGFB induced factor homeobox 1 [Source:HGNC Symbol;Acc:HGNC:1	4.34E-27
10	PHB	prohibitin [Source:HGNC Symbol;Acc:HGNC:8912]	1.03E-15
10	ATP5PD	ATP synthase peripheral stalk subunit d [Source:HGNC Symbol;Acc:H	1.97E-23
10	PON2	paraoxonase 2 [Source:HGNC Symbol;Acc:HGNC:9205]	6.06E-32
10	HSD17B10	hydroxysteroid 17-beta dehydrogenase 10 [Source:HGNC Symbol;Ac	1.15E-19
10	ALDH2	aldehyde dehydrogenase 2 family member [Source:HGNC Symbol;Ac	3.89E-15
10	EI24	EI24 autophagy associated transmembrane protein [Source:HGNC Sy	6.45E-33
10	NDUFA8	NADH:ubiquinone oxidoreductase subunit A8 [Source:HGNC Symbol;	2.09E-20
10	EBNA1BP2	EBNA1 binding protein 2 [Source:HGNC Symbol;Acc:HGNC:15531]	1.76E-26
10	WEE1	WEE1 G2 checkpoint kinase [Source:HGNC Symbol;Acc:HGNC:12761]	7.47E-28
10	NDUFB11	NADH:ubiquinone oxidoreductase subunit B11 [Source:HGNC Symbo	3.57E-19
10	SNRNP25	small nuclear ribonucleoprotein U11/U12 subunit 25 [Source:HGNC	1.32E-15
10	APOD	apolipoprotein D [Source:HGNC Symbol;Acc:HGNC:612]	2.09E-31
10	ATRAID	all-trans retinoic acid induced differentiation factor [Source:HGNC Sy	5.94E-22
10	FH	fumarate hydratase [Source:HGNC Symbol;Acc:HGNC:3700]	7.28E-23
10	EZR	ezrin [Source:HGNC Symbol;Acc:HGNC:12691]	5.62E-13
10	GSN	gelsolin [Source:HGNC Symbol;Acc:HGNC:4620]	5.29E-18
10	DCTPP1	dCTP pyrophosphatase 1 [Source:HGNC Symbol;Acc:HGNC:28777]	1.83E-25
10	DST	dystonin [Source:HGNC Symbol;Acc:HGNC:1090]	1.34E-21
10	LGALS3BP	galectin 3 binding protein [Source:HGNC Symbol;Acc:HGNC:6564]	1.81E-28
10	PACSIN3	protein kinase C and casein kinase substrate in neurons 3 [Source:H	1.84E-35
10	EDF1	endothelial differentiation related factor 1 [Source:HGNC Symbol;Ac	1.81E-26
10	TECR	trans-2,3-enoyl-CoA reductase [Source:HGNC Symbol;Acc:HGNC:455	5.67E-19
10	ARFIP2	ADP ribosylation factor interacting protein 2 [Source:HGNC Symbol;A	3.61E-40
10	NDUFC2	NADH:ubiquinone oxidoreductase subunit C2 [Source:HGNC Symbol;	2.15E-14
10	GAMT	guanidinoacetate N-methyltransferase [Source:HGNC Symbol;Acc:H	1.63E-22

10	TSPAN15	tetraspanin 15 [Source:HGNC Symbol;Acc:HGNC:23298]	1.89E-25
10	TCEA3	transcription elongation factor A3 [Source:HGNC Symbol;Acc:HGNC:1	1.66E-27
10	BHLHE40	basic helix-loop-helix family member e40 [Source:HGNC Symbol;Acc:	3.42E-13
10	COX17	cytochrome c oxidase copper chaperone COX17 [Source:HGNC Symb	8.04E-26
10	NDUFV1	NADH:ubiquinone oxidoreductase core subunit V1 [Source:HGNC Syr	4.31E-21
10	FKBP2	FKBP prolyl isomerase 2 [Source:HGNC Symbol;Acc:HGNC:3718]	1.14E-17
10	NOP10	NOP10 ribonucleoprotein [Source:HGNC Symbol;Acc:HGNC:14378]	5.02E-15
10	PTGIS	prostaglandin I2 synthase [Source:HGNC Symbol;Acc:HGNC:9603]	8.38E-34
10	COA4	cytochrome c oxidase assembly factor 4 homolog [Source:HGNC Sym	1.37E-10
10	PCBD1	pterin-4 alpha-carbinolamine dehydratase 1 [Source:HGNC Symbol;A	9.08E-24
10	CA12	carbonic anhydrase 12 [Source:HGNC Symbol;Acc:HGNC:1371]	5.80E-38
10	TMEM63A	transmembrane protein 63A [Source:HGNC Symbol;Acc:HGNC:2911	4.01E-31
10	PTGR1	prostaglandin reductase 1 [Source:HGNC Symbol;Acc:HGNC:18429]	1.28E-34
10	GPR160	G protein-coupled receptor 160 [Source:HGNC Symbol;Acc:HGNC:23	1.77E-30
10	EFNA1	ephrin A1 [Source:HGNC Symbol;Acc:HGNC:3221]	3.87E-34
10	DIXDC1	DIX domain containing 1 [Source:HGNC Symbol;Acc:HGNC:23695]	1.16E-30
10	ATP5F1D	ATP synthase F1 subunit delta [Source:HGNC Symbol;Acc:HGNC:837]	5.39E-19
10	SOD1	superoxide dismutase 1 [Source:HGNC Symbol;Acc:HGNC:11179]	2.17E-29
10	WASL	WASP like actin nucleation promoting factor [Source:HGNC Symbol;A	2.78E-30
10	ACSL3	acyl-CoA synthetase long chain family member 3 [Source:HGNC Sym	8.64E-28
10	KRT10	keratin 10 [Source:HGNC Symbol;Acc:HGNC:6413]	8.28E-13
10	MYL12B	myosin light chain 12B [Source:HGNC Symbol;Acc:HGNC:29827]	1.00E-25
10	LIMA1	LIM domain and actin binding 1 [Source:HGNC Symbol;Acc:HGNC:24	1.74E-27
10	SULT2B1	sulfotransferase family 2B member 1 [Source:HGNC Symbol;Acc:HGN	6.27E-37
10	TCEAL3	transcription elongation factor A like 3 [Source:HGNC Symbol;Acc:HG	1.32E-21
10	PDIA3	protein disulfide isomerase family A member 3 [Source:HGNC Symbo	4.33E-24
10	ATP5PF	ATP synthase peripheral stalk subunit F6 [Source:HGNC Symbol;Acc:I	2.26E-25
10	AAMDC	adipogenesis associated Mth938 domain containing [Source:HGNC S	3.02E-28
10	SLC39A1	solute carrier family 39 member 1 [Source:HGNC Symbol;Acc:HGNC:	4.63E-30
10	TRAF4	TNF receptor associated factor 4 [Source:HGNC Symbol;Acc:HGNC:1	1.24E-20
10	ISOC2	isochorismatase domain containing 2 [Source:HGNC Symbol;Acc:HGN	7.20E-14
10	LYPD6B	LY6/PLAUR domain containing 6B [Source:HGNC Symbol;Acc:HGNC:2	1.88E-29
10	SH3YL1	SH3 and SYLF domain containing 1 [Source:HGNC Symbol;Acc:HGNC:	7.55E-28
10	RCN1	reticulocalbin 1 [Source:HGNC Symbol;Acc:HGNC:9934]	5.68E-22
10	MAP3K13	mitogen-activated protein kinase kinase kinase 13 [Source:HGNC Syr	5.26E-21
10	MRPS26	mitochondrial ribosomal protein S26 [Source:HGNC Symbol;Acc:HGN	1.57E-16
10	DDRGK1	DDRGK domain containing 1 [Source:HGNC Symbol;Acc:HGNC:16110	4.84E-23
10	HINT1	histidine triad nucleotide binding protein 1 [Source:HGNC Symbol;Ac	1.88E-17
10	KDM5B	lysine demethylase 5B [Source:HGNC Symbol;Acc:HGNC:18039]	2.53E-31
10	TFAP2A	transcription factor AP-2 alpha [Source:HGNC Symbol;Acc:HGNC:117	1.16E-34
10	NDUFB9	NADH:ubiquinone oxidoreductase subunit B9 [Source:HGNC Symbol;	3.81E-25
10	MRPS15	mitochondrial ribosomal protein S15 [Source:HGNC Symbol;Acc:HGN	1.71E-15
10	NDUFB5	NADH:ubiquinone oxidoreductase subunit B5 [Source:HGNC Symbol;	3.49E-13
10	PHGDH	phosphoglycerate dehydrogenase [Source:HGNC Symbol;Acc:HGNC:8	5.63E-26
10	ROMO1	reactive oxygen species modulator 1 [Source:HGNC Symbol;Acc:HGN	2.01E-20
10	RPN2	ribophorin II [Source:HGNC Symbol;Acc:HGNC:10382]	4.71E-27
10	JUP	junction plakoglobin [Source:HGNC Symbol;Acc:HGNC:6207]	3.59E-18

10	NDUFA13	NADH:ubiquinone oxidoreductase subunit A13 [Source:HGNC Symbo	4.00E-19
10	AQP5	aquaporin 5 [Source:HGNC Symbol;Acc:HGNC:638]	1.50E-36
10	TOB1	transducer of ERBB2, 1 [Source:HGNC Symbol;Acc:HGNC:11979]	1.64E-09
10	AK2	adenylate kinase 2 [Source:HGNC Symbol;Acc:HGNC:362]	3.27E-17
10	TIMM8B	translocase of inner mitochondrial membrane 8 homolog B [Source:I	1.21E-19
10	HSPA1A	heat shock protein family A (Hsp70) member 1A [Source:HGNC Symb	4.55E-13
10	CTSF	cathepsin F [Source:HGNC Symbol;Acc:HGNC:2531]	7.37E-27
10	TMCO1	transmembrane and coiled-coil domains 1 [Source:HGNC Symbol;Acc	1.71E-17
10	LMNA	lamin A/C [Source:HGNC Symbol;Acc:HGNC:6636]	5.46E-24
10	NR2F6	nuclear receptor subfamily 2 group F member 6 [Source:HGNC Symb	1.92E-31
10	CBR1	carbonyl reductase 1 [Source:HGNC Symbol;Acc:HGNC:1548]	2.23E-18
10	TUBB2A	tubulin beta 2A class IIa [Source:HGNC Symbol;Acc:HGNC:12412]	6.33E-17
10	PSMB6	proteasome 20S subunit beta 6 [Source:HGNC Symbol;Acc:HGNC:954	7.16E-10
10	BLVRA	biliverdin reductase A [Source:HGNC Symbol;Acc:HGNC:1062]	1.17E-12
10	ARPC1A	actin related protein 2/3 complex subunit 1A [Source:HGNC Symbol;	1.81E-29
10	PHLDA3	pleckstrin homology like domain family A member 3 [Source:HGNC S	1.18E-16
10	RNF187	ring finger protein 187 [Source:HGNC Symbol;Acc:HGNC:27146]	7.58E-22
10	TMEM147	transmembrane protein 147 [Source:HGNC Symbol;Acc:HGNC:30414	6.88E-26
10	LSM4	LSM4 homolog, U6 small nuclear RNA and mRNA degradation associ	9.68E-16
10	LRPAP1	LDL receptor related protein associated protein 1 [Source:HGNC Sym	1.15E-14
10	ANXA3	annexin A3 [Source:HGNC Symbol;Acc:HGNC:541]	3.24E-20
10	ANXA9	annexin A9 [Source:HGNC Symbol;Acc:HGNC:547]	2.39E-26
10	ATP6AP1	ATPase H+ transporting accessory protein 1 [Source:HGNC Symbol;A	4.26E-24
10	SNRPG	small nuclear ribonucleoprotein polypeptide G [Source:HGNC Symbo	6.23E-17
10	LAMTOR5	late endosomal/lysosomal adaptor, MAPK and MTOR activator 5 [So	3.09E-18
10	DDIT3	DNA damage inducible transcript 3 [Source:HGNC Symbol;Acc:HGNC	2.79E-08
10	EHF	ETS homologous factor [Source:HGNC Symbol;Acc:HGNC:3246]	1.10E-28
10	HSPD1	heat shock protein family D (Hsp60) member 1 [Source:HGNC Symbo	7.40E-08
10	CUTA	cutA divalent cation tolerance homolog [Source:HGNC Symbol;Acc:H	1.12E-14
10	TOMM20	translocase of outer mitochondrial membrane 20 [Source:HGNC Sym	6.87E-18
10	ARID5A	AT-rich interaction domain 5A [Source:HGNC Symbol;Acc:HGNC:1736	1.04E-19
10	ARL1	ADP ribosylation factor like GTPase 1 [Source:HGNC Symbol;Acc:HGN	1.54E-21
10	HDLBP	high density lipoprotein binding protein [Source:HGNC Symbol;Acc:H	1.82E-15
10	C11orf1	chromosome 11 open reading frame 1 [Source:HGNC Symbol;Acc:HG	1.82E-23
10	CNIH4	cornichon family AMPA receptor auxiliary protein 4 [Source:HGNC Sy	7.25E-15
10	ATP5ME	ATP synthase membrane subunit e [Source:HGNC Symbol;Acc:HGNC	3.33E-21
10	KLF2	Kruppel like factor 2 [Source:HGNC Symbol;Acc:HGNC:6347]	1.24E-11
10	TSPAN13	tetraspanin 13 [Source:HGNC Symbol;Acc:HGNC:21643]	5.97E-12
10	ANAPC11	anaphase promoting complex subunit 11 [Source:HGNC Symbol;Acc:	8.87E-15
10	NUF2	NUF2 component of NDC80 kinetochore complex [Source:HGNC Sym	1.03E-20
10	IGSF8	immunoglobulin superfamily member 8 [Source:HGNC Symbol;Acc:H	6.63E-24
10	MUC1	mucin 1, cell surface associated [Source:HGNC Symbol;Acc:HGNC:75	7.86E-26
10	CCDC160	coiled-coil domain containing 160 [Source:HGNC Symbol;Acc:HGNC:	2.20E-29
10	SLC25A3	solute carrier family 25 member 3 [Source:HGNC Symbol;Acc:HGNC:	7.75E-16
10	CSRP1	cysteine and glycine rich protein 1 [Source:HGNC Symbol;Acc:HGNC:	2.31E-18
10	CRNDE	colorectal neoplasia differentially expressed [Source:HGNC Symbol;A	2.81E-25
10	HILPDA	hypoxia inducible lipid droplet associated [Source:HGNC Symbol;Acc	2.43E-26

10	GLRX2	glutaredoxin 2 [Source:HGNC Symbol;Acc:HGNC:16065]	1.96E-24
10	MTX2	metaxin 2 [Source:HGNC Symbol;Acc:HGNC:7506]	2.86E-18
10	ZFAS1	ZNFX1 antisense RNA 1 [Source:HGNC Symbol;Acc:HGNC:33101]	2.59E-22
10	TMEM134	transmembrane protein 134 [Source:HGNC Symbol;Acc:HGNC:26142	3.39E-18
10	TMEM9	transmembrane protein 9 [Source:HGNC Symbol;Acc:HGNC:18823]	1.66E-25
10	RPLP1	ribosomal protein lateral stalk subunit P1 [Source:HGNC Symbol;Acc:	3.27E-69
10	COMTD1	catechol-O-methyltransferase domain containing 1 [Source:HGNC Sy	7.36E-22
10	YIF1A	Yip1 interacting factor homolog A, membrane trafficking protein [Sou	1.50E-18
10	COPS6	COP9 signalosome subunit 6 [Source:HGNC Symbol;Acc:HGNC:21749	1.17E-12
10	SEC11A	SEC11 homolog A, signal peptidase complex subunit [Source:HGNC S	3.28E-09
10	ADAM15	ADAM metallopeptidase domain 15 [Source:HGNC Symbol;Acc:HGNC	7.65E-20
10	ANXA4	annexin A4 [Source:HGNC Symbol;Acc:HGNC:542]	8.48E-17
10	CTSD	cathepsin D [Source:HGNC Symbol;Acc:HGNC:2529]	1.70E-12
10	IER5L	immediate early response 5 like [Source:HGNC Symbol;Acc:HGNC:23	1.93E-15
10	TPI1	triosephosphate isomerase 1 [Source:HGNC Symbol;Acc:HGNC:1200	2.02E-11
10	ACADVL	acyl-CoA dehydrogenase very long chain [Source:HGNC Symbol;Acc:H	7.32E-24
10	SOX2	SRY-box transcription factor 2 [Source:HGNC Symbol;Acc:HGNC:1119	1.95E-34
10	NIT2	nitrilase family member 2 [Source:HGNC Symbol;Acc:HGNC:29878]	1.04E-18
10	ATP5MC1	ATP synthase membrane subunit c locus 1 [Source:HGNC Symbol;Acc	1.05E-13
10	GP2	glycoprotein 2 [Source:HGNC Symbol;Acc:HGNC:4441]	1.61E-28
10	DUT	deoxyuridine triphosphatase [Source:HGNC Symbol;Acc:HGNC:3078]	7.82E-13
10	YIPF3	Yip1 domain family member 3 [Source:HGNC Symbol;Acc:HGNC:2102	1.21E-26
10	BCAS4	breast carcinoma amplified sequence 4 [Source:HGNC Symbol;Acc:H	5.41E-20
10	MORF4L2	mortality factor 4 like 2 [Source:HGNC Symbol;Acc:HGNC:16849]	2.24E-28
10	ERBB2	erb-b2 receptor tyrosine kinase 2 [Source:HGNC Symbol;Acc:HGNC:3	9.54E-28
10	IMPDH2	inosine monophosphate dehydrogenase 2 [Source:HGNC Symbol;Acc	2.87E-12
10	NTPCR	nucleoside-triphosphatase, cancer-related [Source:HGNC Symbol;Acc	6.24E-16
10	SH3GLB2	SH3 domain containing GRB2 like, endophilin B2 [Source:HGNC Symb	7.59E-19
10	DUSP4	dual specificity phosphatase 4 [Source:HGNC Symbol;Acc:HGNC:3070	1.23E-20
10	CCT3	chaperonin containing TCP1 subunit 3 [Source:HGNC Symbol;Acc:HG	8.56E-11
10	SLC2A10	solute carrier family 2 member 10 [Source:HGNC Symbol;Acc:HGNC:	2.29E-25
10	SFN	stratifin [Source:HGNC Symbol;Acc:HGNC:10773]	3.45E-24
10	ERBB3	erb-b2 receptor tyrosine kinase 3 [Source:HGNC Symbol;Acc:HGNC:3	5.52E-24
10	WWTR1	WW domain containing transcription regulator 1 [Source:HGNC Sym	5.91E-24
10	MGMT	O-6-methylguanine-DNA methyltransferase [Source:HGNC Symbol;A	1.56E-10
10	SSR2	signal sequence receptor subunit 2 [Source:HGNC Symbol;Acc:HGNC	2.39E-13
10	ARHGAP29	Rho GTPase activating protein 29 [Source:HGNC Symbol;Acc:HGNC:3	2.24E-28
10	ATP5IF1	ATP synthase inhibitory factor subunit 1 [Source:HGNC Symbol;Acc:H	2.19E-14
10	PSMD8	proteasome 26S subunit, non-ATPase 8 [Source:HGNC Symbol;Acc:H	6.41E-15
10	MAL2	mal, T cell differentiation protein 2 [Source:HGNC Symbol;Acc:HGNC	1.10E-35
10	MDK	midkine [Source:HGNC Symbol;Acc:HGNC:6972]	2.77E-20
10	KIF5B	kinesin family member 5B [Source:HGNC Symbol;Acc:HGNC:6324]	2.12E-18
10	CLCA2	chloride channel accessory 2 [Source:HGNC Symbol;Acc:HGNC:2016]	6.47E-24
10	RABEP1	rabaptin, RAB GTPase binding effector protein 1 [Source:HGNC Symb	1.89E-28
10	DDR1	discoidin domain receptor tyrosine kinase 1 [Source:HGNC Symbol;A	7.03E-24
10	SELENOP	selenoprotein P [Source:HGNC Symbol;Acc:HGNC:10751]	1.14E-32
10	MPC2	mitochondrial pyruvate carrier 2 [Source:HGNC Symbol;Acc:HGNC:24	3.65E-26

10	NDUFA10	NADH:ubiquinone oxidoreductase subunit A10 [Source:HGNC Symbo	9.79E-13
10	MRPL12	mitochondrial ribosomal protein L12 [Source:HGNC Symbol;Acc:HGN	2.17E-13
10	RALA	RAS like proto-oncogene A [Source:HGNC Symbol;Acc:HGNC:9839]	2.48E-19
10	UQCRFS1	ubiquinol-cytochrome c reductase, Rieske iron-sulfur polypeptide 1 [	8.85E-12
10	MCRIP2	MAPK regulated corepressor interacting protein 2 [Source:HGNC Syn	4.89E-18
10	TMEM165	transmembrane protein 165 [Source:HGNC Symbol;Acc:HGNC:30760	6.92E-27
10	TFPT	TCF3 fusion partner [Source:HGNC Symbol;Acc:HGNC:13630]	5.74E-17
10	PDCD5	programmed cell death 5 [Source:HGNC Symbol;Acc:HGNC:8764]	2.15E-09
10	KIF9	kinesin family member 9 [Source:HGNC Symbol;Acc:HGNC:16666]	2.05E-24
10	ACADSB	acyl-CoA dehydrogenase short/branched chain [Source:HGNC Symbo	1.23E-24
10	SUMO1	small ubiquitin like modifier 1 [Source:HGNC Symbol;Acc:HGNC:1250	3.78E-18
10	ETFB	electron transfer flavoprotein subunit beta [Source:HGNC Symbol;Ac	2.14E-11
10	CTNND1	catenin delta 1 [Source:HGNC Symbol;Acc:HGNC:2515]	2.24E-19
10	EPS8L1	EPS8 like 1 [Source:HGNC Symbol;Acc:HGNC:21295]	1.89E-20
10	SLC25A39	solute carrier family 25 member 39 [Source:HGNC Symbol;Acc:HGNC	1.69E-16
10	DSC2	desmocollin 2 [Source:HGNC Symbol;Acc:HGNC:3036]	2.74E-22
10	PHPT1	phosphohistidine phosphatase 1 [Source:HGNC Symbol;Acc:HGNC:3(	2.29E-08
10	RER1	retention in endoplasmic reticulum sorting receptor 1 [Source:HGNC	1.67E-18
10	VDAC2	voltage dependent anion channel 2 [Source:HGNC Symbol;Acc:HGNC	1.55E-15
10	FAM241A	family with sequence similarity 241 member A [Source:HGNC Symbo	2.33E-16
10	MRPL32	mitochondrial ribosomal protein L32 [Source:HGNC Symbol;Acc:HGN	1.94E-24
10	MRPL14	mitochondrial ribosomal protein L14 [Source:HGNC Symbol;Acc:HGN	7.56E-10
10	NFIA	nuclear factor I A [Source:HGNC Symbol;Acc:HGNC:7784]	8.15E-22
10	LAMB2	laminin subunit beta 2 [Source:HGNC Symbol;Acc:HGNC:6487]	1.32E-20
10	ATP6V0E2	ATPase H+ transporting V0 subunit e2 [Source:HGNC Symbol;Acc:HG	7.83E-14
10	TRMT112	tRNA methyltransferase subunit 11-2 [Source:HGNC Symbol;Acc:HGN	3.32E-11
10	NOSTRIN	nitric oxide synthase trafficking [Source:HGNC Symbol;Acc:HGNC:202	2.81E-32
10	SDC4	syndecan 4 [Source:HGNC Symbol;Acc:HGNC:10661]	4.27E-13
10	FLYWCH2	FLYWCH family member 2 [Source:HGNC Symbol;Acc:HGNC:25178]	1.76E-15
10	FKBP4	FKBP prolyl isomerase 4 [Source:HGNC Symbol;Acc:HGNC:3720]	1.25E-21
10	MRPS34	mitochondrial ribosomal protein S34 [Source:HGNC Symbol;Acc:HGN	2.08E-14
10	PTRHD1	peptidyl-tRNA hydrolase domain containing 1 [Source:HGNC Symbol;	1.01E-14
10	ATP5MF	ATP synthase membrane subunit f [Source:HGNC Symbol;Acc:HGNC:	2.46E-12
10	RNF19A	ring finger protein 19A, RBR E3 ubiquitin protein ligase [Source:HGN	1.57E-17
10	MORN2	MORN repeat containing 2 [Source:HGNC Symbol;Acc:HGNC:30166]	1.74E-28
10	RBM8A	RNA binding motif protein 8A [Source:HGNC Symbol;Acc:HGNC:9905	2.89E-11
10	NUCKS1	nuclear casein kinase and cyclin dependent kinase substrate 1 [Sourc	1.27E-16
10	SELENOS	selenoprotein S [Source:HGNC Symbol;Acc:HGNC:30396]	1.61E-11
10	TUFM	Tu translation elongation factor, mitochondrial [Source:HGNC Symbo	7.56E-14
10	NEDD4L	NEDD4 like E3 ubiquitin protein ligase [Source:HGNC Symbol;Acc:HG	2.37E-21
10	TRIM47	tripartite motif containing 47 [Source:HGNC Symbol;Acc:HGNC:1902	5.85E-22
10	METTL5	methyltransferase like 5 [Source:HGNC Symbol;Acc:HGNC:25006]	1.35E-13
10	CHCHD5	coiled-coil-helix-coiled-coil-helix domain containing 5 [Source:HGNC	7.10E-10
10	C15orf48	chromosome 15 open reading frame 48 [Source:HGNC Symbol;Acc:H	1.20E-16
10	LIMCH1	LIM and calponin homology domains 1 [Source:HGNC Symbol;Acc:HG	5.92E-27
10	TMBIM6	transmembrane BAX inhibitor motif containing 6 [Source:HGNC Sym	8.62E-15
10	ZNF593	zinc finger protein 593 [Source:HGNC Symbol;Acc:HGNC:30943]	7.77E-18

10	GOLGB1	golgin B1 [Source:HGNC Symbol;Acc:HGNC:4429]	2.96E-14
10	MAGED2	MAGE family member D2 [Source:HGNC Symbol;Acc:HGNC:16353]	4.51E-21
10	DNALI1	dynein axonemal light intermediate chain 1 [Source:HGNC Symbol;A	1.81E-31
10	RPS27L	ribosomal protein S27 like [Source:HGNC Symbol;Acc:HGNC:18476]	2.60E-12
10	TMEM97	transmembrane protein 97 [Source:HGNC Symbol;Acc:HGNC:28106]	6.17E-21
10	NHP2	NHP2 ribonucleoprotein [Source:HGNC Symbol;Acc:HGNC:14377]	6.45E-10
10	NDUFB10	NADH:ubiquinone oxidoreductase subunit B10 [Source:HGNC Symbo	8.97E-12
10	MRPL20	mitochondrial ribosomal protein L20 [Source:HGNC Symbol;Acc:HGN	2.09E-12
10	PFDN2	prefoldin subunit 2 [Source:HGNC Symbol;Acc:HGNC:8867]	1.10E-10
10	KRTCAP3	keratinocyte associated protein 3 [Source:HGNC Symbol;Acc:HGNC:2	1.94E-26
10	NDUFV3	NADH:ubiquinone oxidoreductase subunit V3 [Source:HGNC Symbol;	2.01E-15
10	C17orf58	chromosome 17 open reading frame 58 [Source:HGNC Symbol;Acc:H	5.38E-25
10	MRPL13	mitochondrial ribosomal protein L13 [Source:HGNC Symbol;Acc:HGN	1.17E-13
10	POLR2H	RNA polymerase II, I and III subunit H [Source:HGNC Symbol;Acc:HGN	4.13E-18
10	RNF39	ring finger protein 39 [Source:HGNC Symbol;Acc:HGNC:18064]	1.54E-20
10	PNKD	PNKD metallo-beta-lactamase domain containing [Source:HGNC Sym	2.08E-24
10	EMC2	ER membrane protein complex subunit 2 [Source:HGNC Symbol;Acc:	1.30E-16
10	CD59	CD59 molecule (CD59 blood group) [Source:HGNC Symbol;Acc:HGNC	2.62E-15
10	VDAC1	voltage dependent anion channel 1 [Source:HGNC Symbol;Acc:HGNC	4.22E-11
10	AGR3	anterior gradient 3, protein disulphide isomerase family member [So	5.48E-24
10	NINJ1	ninjurin 1 [Source:HGNC Symbol;Acc:HGNC:7824]	2.11E-08
10	UROS	uroporphyrinogen III synthase [Source:HGNC Symbol;Acc:HGNC:125	7.16E-14
10	ROGDI	rogdi atypical leucine zipper [Source:HGNC Symbol;Acc:HGNC:29478	8.77E-11
10	PIN1	peptidylprolyl cis/trans isomerase, NIMA-interacting 1 [Source:HGNC	6.15E-12
10	HOXB2	homeobox B2 [Source:HGNC Symbol;Acc:HGNC:5113]	3.00E-16
10	ERI3	ERI1 exoribonuclease family member 3 [Source:HGNC Symbol;Acc:H	1.44E-21
10	PSMC5	proteasome 26S subunit, ATPase 5 [Source:HGNC Symbol;Acc:HGNC	1.62E-07
10	VPS28	VPS28 subunit of ESCRT-I [Source:HGNC Symbol;Acc:HGNC:18178]	2.44E-16
10	SPCS1	signal peptidase complex subunit 1 [Source:HGNC Symbol;Acc:HGNC	3.13E-12
10	PPFIBP1	PPFIA binding protein 1 [Source:HGNC Symbol;Acc:HGNC:9249]	2.12E-20
10	SYDE2	synapse defective Rho GTPase homolog 2 [Source:HGNC Symbol;Acc	5.44E-26
10	UGCG	UDP-glucose ceramide glucosyltransferase [Source:HGNC Symbol;Ac	2.10E-10
10	MRPL21	mitochondrial ribosomal protein L21 [Source:HGNC Symbol;Acc:HGN	3.94E-09
10	SYPL1	synaptophysin like 1 [Source:HGNC Symbol;Acc:HGNC:11507]	8.77E-18
10	NUDT22	nudix hydrolase 22 [Source:HGNC Symbol;Acc:HGNC:28189]	3.46E-15
10	POLR3K	RNA polymerase III subunit K [Source:HGNC Symbol;Acc:HGNC:1412	1.01E-14
10	RGS4	regulator of G protein signaling 4 [Source:HGNC Symbol;Acc:HGNC:1	8.73E-19
10	RHOD	ras homolog family member D [Source:HGNC Symbol;Acc:HGNC:670	2.43E-22
10	MRPL33	mitochondrial ribosomal protein L33 [Source:HGNC Symbol;Acc:HGN	9.13E-17
10	DALRD3	DALR anticodon binding domain containing 3 [Source:HGNC Symbol;	1.74E-20
10	UQCC2	ubiquinol-cytochrome c reductase complex assembly factor 2 [Sourc	1.63E-10
10	MRPL43	mitochondrial ribosomal protein L43 [Source:HGNC Symbol;Acc:HGN	5.43E-12
10	PSMB5	proteasome 20S subunit beta 5 [Source:HGNC Symbol;Acc:HGNC:95	3.14E-10
10	RNF7	ring finger protein 7 [Source:HGNC Symbol;Acc:HGNC:10070]	4.40E-15
10	ERICH5	glutamate rich 5 [Source:HGNC Symbol;Acc:HGNC:26823]	3.63E-25
10	UQCC3	ubiquinol-cytochrome c reductase complex assembly factor 3 [Sourc	4.12E-17
10	COX7C	cytochrome c oxidase subunit 7C [Source:HGNC Symbol;Acc:HGNC:2	3.01E-25

10	PTOV1	PTOV1 extended AT-hook containing adaptor protein [Source:HGNC	7.07E-21
10	SPCS2	signal peptidase complex subunit 2 [Source:HGNC Symbol;Acc:HGNC	1.34E-15
10	MTCH2	mitochondrial carrier 2 [Source:HGNC Symbol;Acc:HGNC:17587]	6.07E-11
10	HOOK2	hook microtubule tethering protein 2 [Source:HGNC Symbol;Acc:HG	6.62E-30
10	NDUFS5	NADH:ubiquinone oxidoreductase subunit S5 [Source:HGNC Symbol;	1.72E-19
10	SMS	spermine synthase [Source:HGNC Symbol;Acc:HGNC:11123]	2.49E-14
10	MRPL27	mitochondrial ribosomal protein L27 [Source:HGNC Symbol;Acc:HGN	2.48E-14
10	POLR2K	RNA polymerase II, I and III subunit K [Source:HGNC Symbol;Acc:HGN	1.17E-09
10	ALCAM	activated leukocyte cell adhesion molecule [Source:HGNC Symbol;Ac	3.64E-10
10	B3GAT3	beta-1,3-glucuronyltransferase 3 [Source:HGNC Symbol;Acc:HGNC:9	1.61E-19
10	MCL1	MCL1 apoptosis regulator, BCL2 family member [Source:HGNC Symb	4.16E-10
10	TRPT1	tRNA phosphotransferase 1 [Source:HGNC Symbol;Acc:HGNC:20316]	9.38E-26
10	DCTN3	dynactin subunit 3 [Source:HGNC Symbol;Acc:HGNC:2713]	4.56E-13
10	ERLEC1	endoplasmic reticulum lectin 1 [Source:HGNC Symbol;Acc:HGNC:252	2.08E-13
10	CNPY2	canopy FGF signaling regulator 2 [Source:HGNC Symbol;Acc:HGNC:1	2.36E-12
10	NECAB3	N-terminal EF-hand calcium binding protein 3 [Source:HGNC Symbol;	1.86E-21
10	RAMP2	receptor activity modifying protein 2 [Source:HGNC Symbol;Acc:HGN	1.38E-27
10	LSM2	LSM2 homolog, U6 small nuclear RNA and mRNA degradation associ	1.38E-12
10	RAB34	RAB34, member RAS oncogene family [Source:HGNC Symbol;Acc:HG	1.85E-09
10	UQCR10	ubiquinol-cytochrome c reductase, complex III subunit X [Source:HG	1.49E-13
10	CALM2	calmodulin 2 [Source:HGNC Symbol;Acc:HGNC:1445]	1.95E-12
10	MRPS12	mitochondrial ribosomal protein S12 [Source:HGNC Symbol;Acc:HGN	6.17E-10
10	MRPL54	mitochondrial ribosomal protein L54 [Source:HGNC Symbol;Acc:HGN	1.75E-08
10	PPCS	phosphopantothenoylcysteine synthetase [Source:HGNC Symbol;Acc	1.16E-20
10	SMIM1	small integral membrane protein 1 (Vel blood group) [Source:HGNC	8.86E-15
10	ENDOG	endonuclease G [Source:HGNC Symbol;Acc:HGNC:3346]	6.27E-15
10	MRPS36	mitochondrial ribosomal protein S36 [Source:HGNC Symbol;Acc:HGN	7.35E-13
10	FAM114A1	family with sequence similarity 114 member A1 [Source:HGNC Symb	1.18E-20
10	LINC01184	long intergenic non-protein coding RNA 1184 [Source:HGNC Symbol;	2.56E-14
10	LEO1	LEO1 homolog, Paf1/RNA polymerase II complex component [Source	6.87E-15
10	LHFPL2	LHFPL tetraspan subfamily member 2 [Source:HGNC Symbol;Acc:HG	7.36E-13
10	DPM3	dolichyl-phosphate mannosyltransferase subunit 3, regulatory [Sour	3.87E-24
10	COBL	cordon-bleu WH2 repeat protein [Source:HGNC Symbol;Acc:HGNC:2	1.27E-20
10	P4HTM	prolyl 4-hydroxylase, transmembrane [Source:HGNC Symbol;Acc:HG	4.53E-26
10	ARV1	ARV1 homolog, fatty acid homeostasis modulator [Source:HGNC Sym	6.87E-14
10	EBAG9	estrogen receptor binding site associated antigen 9 [Source:HGNC Sy	1.60E-22
10	GPRC5C	G protein-coupled receptor class C group 5 member C [Source:HGNC	2.09E-23
10	SNRPF	small nuclear ribonucleoprotein polypeptide F [Source:HGNC Symbo	2.32E-14
10	UQCRC2	ubiquinol-cytochrome c reductase core protein 2 [Source:HGNC Sym	7.46E-11
10	PPIB	peptidylprolyl isomerase B [Source:HGNC Symbol;Acc:HGNC:9255]	7.69E-10
10	ORMDL2	ORMDL sphingolipid biosynthesis regulator 2 [Source:HGNC Symbol;	1.50E-12
10	SRA1	steroid receptor RNA activator 1 [Source:HGNC Symbol;Acc:HGNC:1	1.92E-12
10	CPNE3	copine 3 [Source:HGNC Symbol;Acc:HGNC:2316]	3.52E-16
10	SLC25A4	solute carrier family 25 member 4 [Source:HGNC Symbol;Acc:HGNC:	1.63E-17
10	ILF2	interleukin enhancer binding factor 2 [Source:HGNC Symbol;Acc:HGN	7.74E-16
10	MZT2B	mitotic spindle organizing protein 2B [Source:HGNC Symbol;Acc:HGN	3.37E-09
10	COPRS	coordinator of PRMT5 and differentiation stimulator [Source:HGNC S	9.32E-13

10	NDUFS4	NADH:ubiquinone oxidoreductase subunit S4 [Source:HGNC Symbol;	1.86E-15
10	ZNHIT1	zinc finger HIT-type containing 1 [Source:HGNC Symbol;Acc:HGNC:21	2.17E-10
10	EXOSC3	exosome component 3 [Source:HGNC Symbol;Acc:HGNC:17944]	1.94E-17
10	C12orf57	chromosome 12 open reading frame 57 [Source:HGNC Symbol;Acc:H	3.15E-21
10	COX7A2L	cytochrome c oxidase subunit 7A2 like [Source:HGNC Symbol;Acc:HG	1.41E-17
10	ATP5PO	ATP synthase peripheral stalk subunit OSCP [Source:HGNC Symbol;A	8.48E-10
10	VEGFB	vascular endothelial growth factor B [Source:HGNC Symbol;Acc:HGN	8.70E-11
10	CSNK1A1	casein kinase 1 alpha 1 [Source:HGNC Symbol;Acc:HGNC:2451]	1.56E-17
10	SLC50A1	solute carrier family 50 member 1 [Source:HGNC Symbol;Acc:HGNC:	2.12E-17
10	MTX1	metaxin 1 [Source:HGNC Symbol;Acc:HGNC:7504]	4.60E-13
10	PDIA6	protein disulfide isomerase family A member 6 [Source:HGNC Symbo	3.95E-10
10	ATP5MD	ATP synthase membrane subunit DAPIT [Source:HGNC Symbol;Acc:H	1.73E-13
10	SNU13	small nuclear ribonucleoprotein 13 [Source:HGNC Symbol;Acc:HGNC	2.96E-11
10	PRSS22	serine protease 22 [Source:HGNC Symbol;Acc:HGNC:14368]	2.11E-17
10	FAM181B	family with sequence similarity 181 member B [Source:HGNC Symbo	5.45E-14
10	TPRN	taperin [Source:HGNC Symbol;Acc:HGNC:26894]	6.39E-23
10	AHCY	adenosylhomocysteinase [Source:HGNC Symbol;Acc:HGNC:343]	7.73E-11
10	SLIRP	SRA stem-loop interacting RNA binding protein [Source:HGNC Symbo	5.23E-17
10	CHID1	chitinase domain containing 1 [Source:HGNC Symbol;Acc:HGNC:284	8.51E-21
10	AP1S1	adaptor related protein complex 1 subunit sigma 1 [Source:HGNC Sy	8.16E-16
10	SEC61B	SEC61 translocon subunit beta [Source:HGNC Symbol;Acc:HGNC:169	1.07E-07
10	MPG	N-methylpurine DNA glycosylase [Source:HGNC Symbol;Acc:HGNC:7	9.21E-12
10	GRHPR	glyoxylate and hydroxypyruvate reductase [Source:HGNC Symbol;Ac	5.60E-18
10	FAM210B	family with sequence similarity 210 member B [Source:HGNC Symbo	1.27E-12
10	NEURL3	neuralized E3 ubiquitin protein ligase 3 [Source:HGNC Symbol;Acc:H	4.41E-13
10	GOLGA4	golgin A4 [Source:HGNC Symbol;Acc:HGNC:4427]	3.20E-11
10	SMIM26	small integral membrane protein 26 [Source:HGNC Symbol;Acc:HGN	3.39E-13
10	IGSF21	immunoglobin superfamily member 21 [Source:HGNC Symbol;Acc:H	4.67E-22
10	TMEM99	transmembrane protein 99 (putative) [Source:HGNC Symbol;Acc:HG	2.26E-18
10	FOXA3	forkhead box A3 [Source:HGNC Symbol;Acc:HGNC:5023]	7.12E-18
10	SEC62	SEC62 homolog, preprotein translocation factor [Source:HGNC Symb	1.36E-17
10	MRPL15	mitochondrial ribosomal protein L15 [Source:HGNC Symbol;Acc:HGN	3.15E-13
10	EIF2B3	eukaryotic translation initiation factor 2B subunit gamma [Source:HG	1.55E-10
10	MRPS21	mitochondrial ribosomal protein S21 [Source:HGNC Symbol;Acc:HGN	1.99E-07
10	ETHE1	ETHE1 persulfide dioxygenase [Source:HGNC Symbol;Acc:HGNC:232	3.64E-10
10	SLC39A6	solute carrier family 39 member 6 [Source:HGNC Symbol;Acc:HGNC:	3.10E-21
10	COX14	cytochrome c oxidase assembly factor COX14 [Source:HGNC Symbol	9.07E-14
10	PPA2	inorganic pyrophosphatase 2 [Source:HGNC Symbol;Acc:HGNC:2888	6.52E-13
10	CCT5	chaperonin containing TCP1 subunit 5 [Source:HGNC Symbol;Acc:HG	3.02E-07
10	SF3B4	splicing factor 3b subunit 4 [Source:HGNC Symbol;Acc:HGNC:10771]	2.40E-25
10	TMEM159	transmembrane protein 159 [Source:HGNC Symbol;Acc:HGNC:3013	3.49E-17
10	PDHB	pyruvate dehydrogenase E1 subunit beta [Source:HGNC Symbol;Acc:	4.71E-17
10	MAPK13	mitogen-activated protein kinase 13 [Source:HGNC Symbol;Acc:HGN	7.81E-23
10	PKP3	plakophilin 3 [Source:HGNC Symbol;Acc:HGNC:9025]	7.18E-18
10	OSBPL1A	oxysterol binding protein like 1A [Source:HGNC Symbol;Acc:HGNC:1	9.97E-17
10	FIS1	fission, mitochondrial 1 [Source:HGNC Symbol;Acc:HGNC:21689]	6.09E-10
10	MEA1	male-enhanced antigen 1 [Source:HGNC Symbol;Acc:HGNC:6986]	1.25E-15

10	BTF3	basic transcription factor 3 [Source:HGNC Symbol;Acc:HGNC:1125]	5.39E-16
10	SSBP1	single stranded DNA binding protein 1 [Source:HGNC Symbol;Acc:HG	1.46E-09
10	ERP27	endoplasmic reticulum protein 27 [Source:HGNC Symbol;Acc:HGNC:2	2.62E-22
10	MRPS7	mitochondrial ribosomal protein S7 [Source:HGNC Symbol;Acc:HGNC	8.23E-10
10	TSFM	Ts translation elongation factor, mitochondrial [Source:HGNC Symbo	9.16E-13
10	PTGES3	prostaglandin E synthase 3 [Source:HGNC Symbol;Acc:HGNC:16049]	4.89E-12
10	SELENOH	selenoprotein H [Source:HGNC Symbol;Acc:HGNC:18251]	5.54E-11
10	PSMG1	proteasome assembly chaperone 1 [Source:HGNC Symbol;Acc:HGNC	3.69E-12
10	PSMD14	proteasome 26S subunit, non-ATPase 14 [Source:HGNC Symbol;Acc:	1.41E-11
10	RTN4	reticulon 4 [Source:HGNC Symbol;Acc:HGNC:14085]	3.54E-13
10	NTHL1	nth like DNA glycosylase 1 [Source:HGNC Symbol;Acc:HGNC:8028]	4.72E-15
10	ABHD11	abhydrolase domain containing 11 [Source:HGNC Symbol;Acc:HGNC:	3.86E-17
10	PRMT1	protein arginine methyltransferase 1 [Source:HGNC Symbol;Acc:HGN	5.33E-09
10	MEAF6	MYST/Esa1 associated factor 6 [Source:HGNC Symbol;Acc:HGNC:256	1.08E-13
10	BHLHE41	basic helix-loop-helix family member e41 [Source:HGNC Symbol;Acc:	2.09E-13
10	SNRPD1	small nuclear ribonucleoprotein D1 polypeptide [Source:HGNC Symb	7.04E-09
10	TSEN34	tRNA splicing endonuclease subunit 34 [Source:HGNC Symbol;Acc:HG	5.70E-16
10	TPD52L1	TPD52 like 1 [Source:HGNC Symbol;Acc:HGNC:12006]	5.12E-20
10	TUSC2	tumor suppressor 2, mitochondrial calcium regulator [Source:HGNC S	5.75E-20
10	AC068888.1	novel transcript, antisense to TENC1 & EIF4B	1.74E-17
10	TUBB	tubulin beta class I [Source:HGNC Symbol;Acc:HGNC:20778]	4.05E-10
10	TMEM87A	transmembrane protein 87A [Source:HGNC Symbol;Acc:HGNC:24522	8.48E-17
10	CCDC47	coiled-coil domain containing 47 [Source:HGNC Symbol;Acc:HGNC:24	1.48E-09
10	ATG101	autophagy related 101 [Source:HGNC Symbol;Acc:HGNC:25679]	2.04E-07
10	SMIM4	small integral membrane protein 4 [Source:HGNC Symbol;Acc:HGNC	3.97E-16
10	LMAN2	lectin, mannose binding 2 [Source:HGNC Symbol;Acc:HGNC:16986]	4.53E-12
10	ASL	argininosuccinate lyase [Source:HGNC Symbol;Acc:HGNC:746]	9.58E-16
10	CIB1	calcium and integrin binding 1 [Source:HGNC Symbol;Acc:HGNC:1692	2.22E-07
10	BDH1	3-hydroxybutyrate dehydrogenase 1 [Source:HGNC Symbol;Acc:HGN	1.26E-07
10	CCDC90B	coiled-coil domain containing 90B [Source:HGNC Symbol;Acc:HGNC:2	2.25E-12
10	SBDS	SBDS ribosome maturation factor [Source:HGNC Symbol;Acc:HGNC:1	2.83E-11
10	NDUFS3	NADH:ubiquinone oxidoreductase core subunit S3 [Source:HGNC Syn	9.10E-09
10	MACROD1	mono-ADP ribosylhydrolase 1 [Source:HGNC Symbol;Acc:HGNC:2959	8.23E-24
10	SLC49A3	solute carrier family 49 member 3 [Source:HGNC Symbol;Acc:HGNC:	2.15E-12
10	GPAA1	glycosylphosphatidylinositol anchor attachment 1 [Source:HGNC Sym	2.42E-16
10	PSMB7	proteasome 20S subunit beta 7 [Source:HGNC Symbol;Acc:HGNC:954	2.37E-13
10	GTPBP6	GTP binding protein 6 (putative) [Source:HGNC Symbol;Acc:HGNC:30	4.43E-15
10	CDK2AP2	cyclin dependent kinase 2 associated protein 2 [Source:HGNC Symbo	2.86E-07
10	LSR	lipolysis stimulated lipoprotein receptor [Source:HGNC Symbol;Acc:H	7.34E-16
10	SRP72	signal recognition particle 72 [Source:HGNC Symbol;Acc:HGNC:1130	1.30E-07
10	PRR15L	proline rich 15 like [Source:HGNC Symbol;Acc:HGNC:28149]	1.26E-19
10	NCL	nucleolin [Source:HGNC Symbol;Acc:HGNC:7667]	1.51E-08
10	IMPACT	impact RWD domain protein [Source:HGNC Symbol;Acc:HGNC:20387	7.17E-09
10	GATA3-AS1	GATA3 antisense RNA 1 [Source:HGNC Symbol;Acc:HGNC:33786]	3.20E-19
10	NAA20	N-alpha-acetyltransferase 20, NatB catalytic subunit [Source:HGNC S	3.44E-13
10	CISD3	CDGSH iron sulfur domain 3 [Source:HGNC Symbol;Acc:HGNC:27578	3.63E-09
10	DNAJC19	DnaJ heat shock protein family (Hsp40) member C19 [Source:HGNC S	6.24E-15

10	PIP	prolactin induced protein [Source:HGNC Symbol;Acc:HGNC:8993]	8.17E-23
10	TUBG1	tubulin gamma 1 [Source:HGNC Symbol;Acc:HGNC:12417]	2.94E-12
10	NOL7	nucleolar protein 7 [Source:HGNC Symbol;Acc:HGNC:21040]	2.62E-07
10	PPIG	peptidylprolyl isomerase G [Source:HGNC Symbol;Acc:HGNC:14650]	4.84E-08
10	TMEM256	transmembrane protein 256 [Source:HGNC Symbol;Acc:HGNC:28618	5.30E-09
10	MYO1C	myosin IC [Source:HGNC Symbol;Acc:HGNC:7597]	1.27E-12
10	ZDHHC24	zinc finger DHHC-type containing 24 [Source:HGNC Symbol;Acc:HGN	1.54E-07
10	ARL4D	ADP ribosylation factor like GTPase 4D [Source:HGNC Symbol;Acc:HG	2.90E-22
10	CCT2	chaperonin containing TCP1 subunit 2 [Source:HGNC Symbol;Acc:HG	1.16E-08
10	CXADR	CXADR Ig-like cell adhesion molecule [Source:HGNC Symbol;Acc:HGN	1.95E-19
10	PXMP4	peroxisomal membrane protein 4 [Source:HGNC Symbol;Acc:HGNC:1	9.07E-20
10	NME4	NME/NM23 nucleoside diphosphate kinase 4 [Source:HGNC Symbol;	3.06E-13
10	CXXC5	CXXC finger protein 5 [Source:HGNC Symbol;Acc:HGNC:26943]	3.27E-12
10	PDZD11	PDZ domain containing 11 [Source:HGNC Symbol;Acc:HGNC:28034]	8.56E-14
10	CEBPD	CCAAT enhancer binding protein delta [Source:HGNC Symbol;Acc:HG	5.12E-08
10	MVB12A	multivesicular body subunit 12A [Source:HGNC Symbol;Acc:HGNC:25	1.13E-12
10	SERF2	small EDRK-rich factor 2 [Source:HGNC Symbol;Acc:HGNC:10757]	1.73E-16
10	AUTS2	activator of transcription and developmental regulator AUTS2 [Sourc	2.46E-19
10	CISD1	CDGSH iron sulfur domain 1 [Source:HGNC Symbol;Acc:HGNC:30880	6.75E-10
10	ACOT13	acyl-CoA thioesterase 13 [Source:HGNC Symbol;Acc:HGNC:20999]	4.09E-11
10	SUCLG1	succinate-CoA ligase GDP/ADP-forming subunit alpha [Source:HGNC	1.07E-07
10	DNPH1	2'-deoxynucleoside 5'-phosphate N-hydrolase 1 [Source:HGNC Symb	5.14E-10
10	CD164	CD164 molecule [Source:HGNC Symbol;Acc:HGNC:1632]	5.46E-12
10	AGPS	alkylglycerone phosphate synthase [Source:HGNC Symbol;Acc:HGNC	3.80E-16
10	TRAPPC4	trafficking protein particle complex 4 [Source:HGNC Symbol;Acc:HGN	1.72E-13
10	ATIC	5-aminoimidazole-4-carboxamide ribonucleotide formyltransferase/	4.97E-08
10	CLTA	clathrin light chain A [Source:HGNC Symbol;Acc:HGNC:2090]	1.88E-09
10	CAST	calpastatin [Source:HGNC Symbol;Acc:HGNC:1515]	8.21E-11
10	SNF8	SNF8 subunit of ESCRT-II [Source:HGNC Symbol;Acc:HGNC:17028]	9.20E-09
10	PODXL2	podocalyxin like 2 [Source:HGNC Symbol;Acc:HGNC:17936]	1.90E-19
10	RGL2	ral guanine nucleotide dissociation stimulator like 2 [Source:HGNC Sy	1.23E-17
10	CD46	CD46 molecule [Source:HGNC Symbol;Acc:HGNC:6953]	6.28E-17
10	MRPS16	mitochondrial ribosomal protein S16 [Source:HGNC Symbol;Acc:HGN	4.78E-11
10	FAM171B	family with sequence similarity 171 member B [Source:HGNC Symbo	5.21E-19
10	POP7	POP7 homolog, ribonuclease P/MRP subunit [Source:HGNC Symbol;A	3.37E-10
10	UBL5	ubiquitin like 5 [Source:HGNC Symbol;Acc:HGNC:13736]	5.95E-12
10	POLD2	DNA polymerase delta 2, accessory subunit [Source:HGNC Symbol;Ac	1.56E-14
10	S100A10	S100 calcium binding protein A10 [Source:HGNC Symbol;Acc:HGNC:1	4.42E-11
10	OCIAD2	OCIA domain containing 2 [Source:HGNC Symbol;Acc:HGNC:28685]	6.59E-09
10	LAD1	ladinin 1 [Source:HGNC Symbol;Acc:HGNC:6472]	3.80E-17
10	EEF1E1	eukaryotic translation elongation factor 1 epsilon 1 [Source:HGNC Sy	8.34E-15
10	GCSH	glycine cleavage system protein H [Source:HGNC Symbol;Acc:HGNC:4	7.27E-12
10	SSNA1	SS nuclear autoantigen 1 [Source:HGNC Symbol;Acc:HGNC:11321]	9.73E-11
10	SMYD2	SET and MYND domain containing 2 [Source:HGNC Symbol;Acc:HGN	2.18E-18
10	MRPL17	mitochondrial ribosomal protein L17 [Source:HGNC Symbol;Acc:HGN	6.15E-19
10	TIMM17B	translocase of inner mitochondrial membrane 17B [Source:HGNC Sy	7.75E-12
10	PIGR	polymeric immunoglobulin receptor [Source:HGNC Symbol;Acc:HGN	2.62E-17

10	GPATCH4	G-patch domain containing 4 [Source:HGNC Symbol;Acc:HGNC:2598	4.26E-12
10	PYCR1	pyrroline-5-carboxylate reductase 1 [Source:HGNC Symbol;Acc:HGN	4.50E-23
10	BUD31	BUD31 homolog [Source:HGNC Symbol;Acc:HGNC:29629]	6.75E-11
10	ICA1	islet cell autoantigen 1 [Source:HGNC Symbol;Acc:HGNC:5343]	1.97E-18
10	AK6	adenylate kinase 6 [Source:HGNC Symbol;Acc:HGNC:49151]	2.10E-09
10	TBX3	T-box transcription factor 3 [Source:HGNC Symbol;Acc:HGNC:11602]	1.70E-24
10	CMYA5	cardiomyopathy associated 5 [Source:HGNC Symbol;Acc:HGNC:1430	6.65E-14
10	MBOAT2	membrane bound O-acyltransferase domain containing 2 [Source:H	7.30E-11
10	HRAS	HRas proto-oncogene, GTPase [Source:HGNC Symbol;Acc:HGNC:517	6.23E-10
10	TTC3	tetratricopeptide repeat domain 3 [Source:HGNC Symbol;Acc:HGNC:	1.17E-09
10	B4GALT3	beta-1,4-galactosyltransferase 3 [Source:HGNC Symbol;Acc:HGNC:92	2.83E-10
10	CASZ1	castor zinc finger 1 [Source:HGNC Symbol;Acc:HGNC:26002]	3.12E-18
10	CYB561	cytochrome b561 [Source:HGNC Symbol;Acc:HGNC:2571]	3.71E-12
10	PCYOX1	prenylcysteine oxidase 1 [Source:HGNC Symbol;Acc:HGNC:20588]	6.41E-21
10	NDUFAF8	NADH:ubiquinone oxidoreductase complex assembly factor 8 [Sourc	4.86E-09
10	BUD23	BUD23 rRNA methyltransferase and ribosome maturation factor [So	2.27E-08
10	BCAS1	brain enriched myelin associated protein 1 [Source:HGNC Symbol;Ac	1.55E-20
10	TCEAL1	transcription elongation factor A like 1 [Source:HGNC Symbol;Acc:H	2.15E-18
10	TTC39A	tetratricopeptide repeat domain 39A [Source:HGNC Symbol;Acc:HGN	8.16E-17
10	COX7A1	cytochrome c oxidase subunit 7A1 [Source:HGNC Symbol;Acc:HGNC:	4.13E-18
10	IFT22	intraflagellar transport 22 [Source:HGNC Symbol;Acc:HGNC:21895]	1.68E-16
10	LYPLA1	lysophospholipase 1 [Source:HGNC Symbol;Acc:HGNC:6737]	3.57E-13
10	MRPL37	mitochondrial ribosomal protein L37 [Source:HGNC Symbol;Acc:HGN	6.68E-12
10	TIMM17A	translocase of inner mitochondrial membrane 17A [Source:HGNC Sy	1.34E-08
10	TWF1	twinfilin actin binding protein 1 [Source:HGNC Symbol;Acc:HGNC:96	3.46E-12
10	CMBL	carboxymethylenebutenolidase homolog [Source:HGNC Symbol;Acc:	2.15E-10
10	SYAP1	synapse associated protein 1 [Source:HGNC Symbol;Acc:HGNC:1627	4.94E-15
10	BOLA3	bolA family member 3 [Source:HGNC Symbol;Acc:HGNC:24415]	3.07E-09
10	NPM1	nucleophosmin 1 [Source:HGNC Symbol;Acc:HGNC:7910]	3.78E-16
10	PPIH	peptidylprolyl isomerase H [Source:HGNC Symbol;Acc:HGNC:14651]	1.90E-07
10	MRPL58	mitochondrial ribosomal protein L58 [Source:HGNC Symbol;Acc:HGN	2.34E-10
10	IARS2	isoleucyl-tRNA synthetase 2, mitochondrial [Source:HGNC Symbol;A	1.51E-08
10	MRPS14	mitochondrial ribosomal protein S14 [Source:HGNC Symbol;Acc:HGN	8.00E-16
10	SURF2	surfeit 2 [Source:HGNC Symbol;Acc:HGNC:11475]	1.92E-08
10	RASEF	RAS and EF-hand domain containing [Source:HGNC Symbol;Acc:HGN	1.41E-18
10	CNNM1	cyclin and CBS domain divalent metal cation transport mediator 1 [S	1.91E-20
10	PER2	period circadian regulator 2 [Source:HGNC Symbol;Acc:HGNC:8846]	1.88E-12
10	POMGNT1	protein O-linked mannose N-acetylglucosaminyltransferase 1 (beta 1	2.58E-18
10	HSD17B4	hydroxysteroid 17-beta dehydrogenase 4 [Source:HGNC Symbol;Acc:	1.29E-13
10	MESP1	mesoderm posterior bHLH transcription factor 1 [Source:HGNC Symb	1.62E-21
10	DLG3	discs large MAGUK scaffold protein 3 [Source:HGNC Symbol;Acc:HGN	1.23E-21
10	MMADHC	metabolism of cobalamin associated D [Source:HGNC Symbol;Acc:H	2.35E-08
10	NFIC	nuclear factor I C [Source:HGNC Symbol;Acc:HGNC:7786]	5.36E-13
10	RPL26L1	ribosomal protein L26 like 1 [Source:HGNC Symbol;Acc:HGNC:17050	3.43E-09
10	NUDT16L1	nudix hydrolase 16 like 1 [Source:HGNC Symbol;Acc:HGNC:28154]	2.37E-09
10	SEZ6L2	seizure related 6 homolog like 2 [Source:HGNC Symbol;Acc:HGNC:30	3.87E-15
10	PTS	6-pyruvoyltetrahydropterin synthase [Source:HGNC Symbol;Acc:HGN	7.00E-17

10	PRSS23	serine protease 23 [Source:HGNC Symbol;Acc:HGNC:14370]	4.19E-18
10	BUB3	BUB3 mitotic checkpoint protein [Source:HGNC Symbol;Acc:HGNC:1	3.91E-10
10	RHOBTB3	Rho related BTB domain containing 3 [Source:HGNC Symbol;Acc:HGN	1.94E-13
10	TMEM208	transmembrane protein 208 [Source:HGNC Symbol;Acc:HGNC:25015	1.20E-07
10	NECTIN2	nectin cell adhesion molecule 2 [Source:HGNC Symbol;Acc:HGNC:97	4.78E-10
10	STAP2	signal transducing adaptor family member 2 [Source:HGNC Symbol;A	2.42E-18
10	NOL3	nucleolar protein 3 [Source:HGNC Symbol;Acc:HGNC:7869]	8.32E-23
10	CCDC6	coiled-coil domain containing 6 [Source:HGNC Symbol;Acc:HGNC:18	2.30E-08
10	PHYHD1	phytanoyl-CoA dioxygenase domain containing 1 [Source:HGNC Sym	1.07E-15
10	MLEC	malectin [Source:HGNC Symbol;Acc:HGNC:28973]	2.43E-07
10	CFB	complement factor B [Source:HGNC Symbol;Acc:HGNC:1037]	6.32E-14
10	EMC7	ER membrane protein complex subunit 7 [Source:HGNC Symbol;Acc:	1.25E-13
10	CALU	calumenin [Source:HGNC Symbol;Acc:HGNC:1458]	8.80E-11
10	ALDH7A1	aldehyde dehydrogenase 7 family member A1 [Source:HGNC Symbol	2.40E-22
10	NDUFAF6	NADH:ubiquinone oxidoreductase complex assembly factor 6 [Sourc	1.15E-13
10	PALMD	palmdelphin [Source:HGNC Symbol;Acc:HGNC:15846]	1.02E-19
10	ECHDC2	enoyl-CoA hydratase domain containing 2 [Source:HGNC Symbol;Acc	3.68E-16
10	BACE2	beta-secretase 2 [Source:HGNC Symbol;Acc:HGNC:934]	4.60E-17
10	CCDC57	coiled-coil domain containing 57 [Source:HGNC Symbol;Acc:HGNC:2	3.19E-09
10	PLLP	plasmolipin [Source:HGNC Symbol;Acc:HGNC:18553]	1.19E-19
10	AAMP	angio associated migratory cell protein [Source:HGNC Symbol;Acc:H	1.12E-10
10	SLC1A5	solute carrier family 1 member 5 [Source:HGNC Symbol;Acc:HGNC:1	4.95E-07
10	NECTIN4	nectin cell adhesion molecule 4 [Source:HGNC Symbol;Acc:HGNC:19	3.75E-15
10	PAICS	phosphoribosylaminoimidazole carboxylase and phosphoribosylamir	7.94E-11
10	SLC2A4RG	SLC2A4 regulator [Source:HGNC Symbol;Acc:HGNC:15930]	7.48E-11
10	OXLD1	oxidoreductase like domain containing 1 [Source:HGNC Symbol;Acc:	1.72E-12
10	FAM110C	family with sequence similarity 110 member C [Source:HGNC Symbo	3.03E-15
10	SUCO	SUN domain containing ossification factor [Source:HGNC Symbol;Acc	8.34E-16
10	TIMMDC1	translocase of inner mitochondrial membrane domain containing 1 [	5.23E-10
10	MRPL19	mitochondrial ribosomal protein L19 [Source:HGNC Symbol;Acc:HGN	1.10E-11
10	PDCD6	programmed cell death 6 [Source:HGNC Symbol;Acc:HGNC:8765]	1.18E-12
10	PDHA1	pyruvate dehydrogenase E1 subunit alpha 1 [Source:HGNC Symbol;A	2.42E-11
10	SRARP	steroid receptor associated and regulated protein [Source:HGNC Syn	5.34E-18
10	PHAX	phosphorylated adaptor for RNA export [Source:HGNC Symbol;Acc:H	2.66E-11
10	ZFP36L1	ZFP36 ring finger protein like 1 [Source:HGNC Symbol;Acc:HGNC:110	1.43E-09
10	MRPL36	mitochondrial ribosomal protein L36 [Source:HGNC Symbol;Acc:HGN	1.17E-08
10	PIGT	phosphatidylinositol glycan anchor biosynthesis class T [Source:HGN	7.96E-12
10	ZNF91	zinc finger protein 91 [Source:HGNC Symbol;Acc:HGNC:13166]	2.96E-11
10	ACTG1	actin gamma 1 [Source:HGNC Symbol;Acc:HGNC:144]	6.76E-08
10	C19orf53	chromosome 19 open reading frame 53 [Source:HGNC Symbol;Acc:H	9.32E-10
10	KIAA1522	KIAA1522 [Source:HGNC Symbol;Acc:HGNC:29301]	9.08E-16
10	DCAF10	DDB1 and CUL4 associated factor 10 [Source:HGNC Symbol;Acc:HGN	3.93E-14
10	SLPI	secretory leukocyte peptidase inhibitor [Source:HGNC Symbol;Acc:H	4.14E-16
10	BRK1	BRICK1 subunit of SCAR/WAVE actin nucleating complex [Source:HG	4.47E-13
10	CDC42EP1	CDC42 effector protein 1 [Source:HGNC Symbol;Acc:HGNC:17014]	4.31E-12
10	APOO	apolipoprotein O [Source:HGNC Symbol;Acc:HGNC:28727]	1.99E-18
10	TINAGL1	tubulointerstitial nephritis antigen like 1 [Source:HGNC Symbol;Acc:H	3.09E-17

10	PIN4	peptidylprolyl cis/trans isomerase, NIMA-interacting 4 [Source:HGN(	6.43E-13
10	TPD52	tumor protein D52 [Source:HGNC Symbol;Acc:HGNC:12005]	8.89E-13
10	NTN4	netrin 4 [Source:HGNC Symbol;Acc:HGNC:13658]	9.39E-16
10	AUP1	AUP1 lipid droplet regulating VLDL assembly factor [Source:HGNC Sy	1.33E-08
10	EDARADD	EDAR associated death domain [Source:HGNC Symbol;Acc:HGNC:143	5.54E-18
10	SMIM30	small integral membrane protein 30 [Source:HGNC Symbol;Acc:HGN(	1.18E-10
10	ANO1	anoctamin 1 [Source:HGNC Symbol;Acc:HGNC:21625]	2.37E-16
10	CHCHD1	coiled-coil-helix-coiled-coil-helix domain containing 1 [Source:HGNC	4.58E-11
10	FAAP20	FA core complex associated protein 20 [Source:HGNC Symbol;Acc:H(	1.35E-07
10	SERTAD3	SERTA domain containing 3 [Source:HGNC Symbol;Acc:HGNC:17931]	6.22E-10
10	COPZ1	COPI coat complex subunit zeta 1 [Source:HGNC Symbol;Acc:HGNC:2	1.98E-10
10	RRNAD1	ribosomal RNA adenine dimethylase domain containing 1 [Source:H(	4.33E-16
10	TMEM183A	transmembrane protein 183A [Source:HGNC Symbol;Acc:HGNC:2017	1.49E-10
10	BAG1	BAG cochaperone 1 [Source:HGNC Symbol;Acc:HGNC:937]	1.56E-11
10	BTF3L4	basic transcription factor 3 like 4 [Source:HGNC Symbol;Acc:HGNC:3(	7.73E-09
10	MXD4	MAX dimerization protein 4 [Source:HGNC Symbol;Acc:HGNC:13906]	9.46E-08
10	MTA3	metastasis associated 1 family member 3 [Source:HGNC Symbol;Acc:	6.01E-20
10	MYO6	myosin VI [Source:HGNC Symbol;Acc:HGNC:7605]	1.71E-15
10	HSBP1L1	heat shock factor binding protein 1 like 1 [Source:HGNC Symbol;Acc:	3.35E-10
10	APH1A	aph-1 homolog A, gamma-secretase subunit [Source:HGNC Symbol;A	1.40E-12
10	IGFBP2	insulin like growth factor binding protein 2 [Source:HGNC Symbol;Ac	6.39E-24
10	PALLD	palladin, cytoskeletal associated protein [Source:HGNC Symbol;Acc:H	7.42E-11
10	COMMD1	copper metabolism domain containing 1 [Source:HGNC Symbol;Acc:I	2.34E-09
10	PCDH1	protocadherin 1 [Source:HGNC Symbol;Acc:HGNC:8655]	2.40E-18
10	BORCS7	BLOC-1 related complex subunit 7 [Source:HGNC Symbol;Acc:HGNC:	1.10E-07
10	GSTO2	glutathione S-transferase omega 2 [Source:HGNC Symbol;Acc:HGNC:	5.54E-09
10	TRAF5	TNF receptor associated factor 5 [Source:HGNC Symbol;Acc:HGNC:1	1.12E-11
10	NME2	NME/NM23 nucleoside diphosphate kinase 2 [Source:HGNC Symbol;	2.45E-23
10	DNAJB11	DnaJ heat shock protein family (Hsp40) member B11 [Source:HGNC S	1.38E-10
10	PARVA	parvin alpha [Source:HGNC Symbol;Acc:HGNC:14652]	4.50E-19
10	FOXC1	forkhead box C1 [Source:HGNC Symbol;Acc:HGNC:3800]	1.04E-08
10	RASSF7	Ras association domain family member 7 [Source:HGNC Symbol;Acc:	1.25E-09
10	CKMT1B	creatine kinase, mitochondrial 1B [Source:HGNC Symbol;Acc:HGNC:1	3.54E-13
10	KNOP1	lysine rich nucleolar protein 1 [Source:HGNC Symbol;Acc:HGNC:344(	2.41E-09
10	IL17RC	interleukin 17 receptor C [Source:HGNC Symbol;Acc:HGNC:18358]	7.05E-12
10	NDUFAF2	NADH:ubiquinone oxidoreductase complex assembly factor 2 [Sourc	1.88E-10
10	IFRD2	interferon related developmental regulator 2 [Source:HGNC Symbol;	4.54E-07
10	TUSC1	tumor suppressor candidate 1 [Source:HGNC Symbol;Acc:HGNC:310	9.58E-13
10	SMIM19	small integral membrane protein 19 [Source:HGNC Symbol;Acc:HGN(	4.62E-08
10	CBR3	carbonyl reductase 3 [Source:HGNC Symbol;Acc:HGNC:1549]	5.76E-15
10	YWHAE	tyrosine 3-monooxygenase/tryptophan 5-monooxygenase activatior	4.28E-09
10	GGCT	gamma-glutamylcyclotransferase [Source:HGNC Symbol;Acc:HGNC:2	4.61E-07
10	C5orf66-AS1	C5orf66 antisense RNA 1 [Source:HGNC Symbol;Acc:HGNC:49679]	6.73E-21
10	BSPRY	B-box and SPRY domain containing [Source:HGNC Symbol;Acc:HGNC	3.19E-14
10	PRDX5	peroxiredoxin 5 [Source:HGNC Symbol;Acc:HGNC:9355]	2.54E-11
10	ITPKC	inositol-trisphosphate 3-kinase C [Source:HGNC Symbol;Acc:HGNC:1	7.81E-15
10	AFDN	afadin, adherens junction formation factor [Source:HGNC Symbol;Ac	9.75E-08

10	TMC4	transmembrane channel like 4 [Source:HGNC Symbol;Acc:HGNC:229	2.01E-19
10	RRAGA	Ras related GTP binding A [Source:HGNC Symbol;Acc:HGNC:16963]	2.77E-16
10	RRP9	ribosomal RNA processing 9, U3 small nucleolar RNA binding protein	1.08E-11
10	HADHB	hydroxyacyl-CoA dehydrogenase trifunctional multienzyme complex	9.05E-11
10	HSPBP1	HSPA (Hsp70) binding protein 1 [Source:HGNC Symbol;Acc:HGNC:24	3.12E-10
10	NDUFA11	NADH:ubiquinone oxidoreductase subunit A11 [Source:HGNC Symbo	2.58E-08
10	DNASE2	deoxyribonuclease 2, lysosomal [Source:HGNC Symbol;Acc:HGNC:29	6.25E-07
10	FAM120AOS	family with sequence similarity 120A opposite strand [Source:HGNC	1.16E-11
10	SPATS2L	spermatogenesis associated serine rich 2 like [Source:HGNC Symbol;	3.06E-08
10	GSTM3	glutathione S-transferase mu 3 [Source:HGNC Symbol;Acc:HGNC:463	2.46E-11
10	DNAJC2	DnaJ heat shock protein family (Hsp40) member C2 [Source:HGNC Sy	2.55E-08
10	TRAPPC12	trafficking protein particle complex 12 [Source:HGNC Symbol;Acc:HG	8.03E-09
10	AKAP1	A-kinase anchoring protein 1 [Source:HGNC Symbol;Acc:HGNC:367]	1.17E-08
10	HDHD3	haloacid dehalogenase like hydrolase domain containing 3 [Source:H	8.71E-15
10	MAP7	microtubule associated protein 7 [Source:HGNC Symbol;Acc:HGNC:6	6.31E-11
10	SPRYD4	SPRY domain containing 4 [Source:HGNC Symbol;Acc:HGNC:27468]	5.36E-11
10	KDELR2	KDEL endoplasmic reticulum protein retention receptor 2 [Source:H(	9.39E-09
10	HIGD1A	HIG1 hypoxia inducible domain family member 1A [Source:HGNC Syr	2.03E-11
10	FAM174B	family with sequence similarity 174 member B [Source:HGNC Symbo	1.01E-14
10	BCAS2	BCAS2 pre-mRNA processing factor [Source:HGNC Symbol;Acc:HGNC	2.16E-07
10	UAP1	UDP-N-acetylglucosamine pyrophosphorylase 1 [Source:HGNC Symb	1.12E-11
10	FAM3B	FAM3 metabolism regulating signaling molecule B [Source:HGNC Syr	5.97E-19
10	CBX4	chromobox 4 [Source:HGNC Symbol;Acc:HGNC:1554]	1.44E-11
10	WASHC3	WASH complex subunit 3 [Source:HGNC Symbol;Acc:HGNC:24256]	1.86E-07
10	ELOVL1	ELOVL fatty acid elongase 1 [Source:HGNC Symbol;Acc:HGNC:14418]	3.63E-09
10	LTBP3	latent transforming growth factor beta binding protein 3 [Source:HG	9.12E-14
10	SEC11C	SEC11 homolog C, signal peptidase complex subunit [Source:HGNC S	2.50E-07
10	TACC2	transforming acidic coiled-coil containing protein 2 [Source:HGNC Sy	6.97E-14
10	LYPD3	LY6/PLAUR domain containing 3 [Source:HGNC Symbol;Acc:HGNC:24	1.09E-10
10	WDR83OS	WD repeat domain 83 opposite strand [Source:HGNC Symbol;Acc:HG	1.03E-07
10	ARL16	ADP ribosylation factor like GTPase 16 [Source:HGNC Symbol;Acc:HG	5.36E-19
10	COMMD2	COMM domain containing 2 [Source:HGNC Symbol;Acc:HGNC:24993	3.29E-07
10	DNPEP	aspartyl aminopeptidase [Source:HGNC Symbol;Acc:HGNC:2981]	2.06E-11
10	EPS8L2	EPS8 like 2 [Source:HGNC Symbol;Acc:HGNC:21296]	4.83E-11
10	C9orf116	chromosome 9 open reading frame 116 [Source:HGNC Symbol;Acc:H	8.72E-17
10	TMEM41A	transmembrane protein 41A [Source:HGNC Symbol;Acc:HGNC:30544	1.24E-19
10	CYSRT1	cysteine rich tail 1 [Source:HGNC Symbol;Acc:HGNC:30529]	1.41E-14
10	NCSTN	nicastrin [Source:HGNC Symbol;Acc:HGNC:17091]	1.25E-09
10	CSAD	cysteine sulfinic acid decarboxylase [Source:HGNC Symbol;Acc:HGNC	3.48E-11
10	CYP4X1	cytochrome P450 family 4 subfamily X member 1 [Source:HGNC Sym	2.23E-16
10	PSMG4	proteasome assembly chaperone 4 [Source:HGNC Symbol;Acc:HGNC	1.83E-09
10	VEGFA	vascular endothelial growth factor A [Source:HGNC Symbol;Acc:HGN	1.08E-11
10	SNAPC5	small nuclear RNA activating complex polypeptide 5 [Source:HGNC S	4.83E-08
10	BMPR1B	bone morphogenetic protein receptor type 1B [Source:HGNC Symbo	1.10E-11
10	MCM4	minichromosome maintenance complex component 4 [Source:HGN(	3.62E-11
10	COMMD4	COMM domain containing 4 [Source:HGNC Symbol;Acc:HGNC:26027	3.31E-07
10	UBE2V2	ubiquitin conjugating enzyme E2 V2 [Source:HGNC Symbol;Acc:HGN(	7.43E-08

10	PIGX	phosphatidylinositol glycan anchor biosynthesis class X [Source:HGN	6.23E-11
10	HYAL2	hyaluronidase 2 [Source:HGNC Symbol;Acc:HGNC:5321]	6.20E-12
10	FUNDC1	FUN14 domain containing 1 [Source:HGNC Symbol;Acc:HGNC:28746	8.22E-12
10	CD81	CD81 molecule [Source:HGNC Symbol;Acc:HGNC:1701]	2.90E-09
10	NMRAL1	NmrA like redox sensor 1 [Source:HGNC Symbol;Acc:HGNC:24987]	2.33E-11
10	TOM1L1	target of myb1 like 1 membrane trafficking protein [Source:HGNC Sy	1.14E-09
10	PLEKHA5	pleckstrin homology domain containing A5 [Source:HGNC Symbol;Ac	1.21E-08
10	GPI	glucose-6-phosphate isomerase [Source:HGNC Symbol;Acc:HGNC:44	6.65E-10
10	SIX1	SIX homeobox 1 [Source:HGNC Symbol;Acc:HGNC:10887]	5.06E-15
10	BCL7C	BAF chromatin remodeling complex subunit BCL7C [Source:HGNC Sy	5.39E-07
10	OCIAD1	OCIA domain containing 1 [Source:HGNC Symbol;Acc:HGNC:16074]	5.38E-11
10	STOML2	stomatin like 2 [Source:HGNC Symbol;Acc:HGNC:14559]	4.19E-08
10	LMTK3	lemur tyrosine kinase 3 [Source:HGNC Symbol;Acc:HGNC:19295]	3.74E-20
10	MRPS18A	mitochondrial ribosomal protein S18A [Source:HGNC Symbol;Acc:HG	1.88E-11
10	SDHB	succinate dehydrogenase complex iron sulfur subunit B [Source:HGN	4.31E-08
10	ATP6V1G1	ATPase H+ transporting V1 subunit G1 [Source:HGNC Symbol;Acc:HG	7.08E-10
10	TMEM186	transmembrane protein 186 [Source:HGNC Symbol;Acc:HGNC:24530	5.04E-12
10	SEC61G	SEC61 translocon subunit gamma [Source:HGNC Symbol;Acc:HGNC:1	4.63E-07
10	ATF7	activating transcription factor 7 [Source:HGNC Symbol;Acc:HGNC:79	1.27E-10
10	RNPEP	arginyl aminopeptidase [Source:HGNC Symbol;Acc:HGNC:10078]	4.47E-08
10	SAR1A	secretion associated Ras related GTPase 1A [Source:HGNC Symbol;A	3.74E-07
10	TRNT1	tRNA nucleotidyl transferase 1 [Source:HGNC Symbol;Acc:HGNC:173	2.57E-09
10	KPNA2	karyopherin subunit alpha 2 [Source:HGNC Symbol;Acc:HGNC:6395]	8.39E-08
10	GPRC5A	G protein-coupled receptor class C group 5 member A [Source:HGNC	1.19E-11
10	JMJD8	jumonji domain containing 8 [Source:HGNC Symbol;Acc:HGNC:1414	6.38E-09
10	IRX5	iroquois homeobox 5 [Source:HGNC Symbol;Acc:HGNC:14361]	3.64E-15
10	C15orf61	chromosome 15 open reading frame 61 [Source:HGNC Symbol;Acc:H	1.00E-08
10	PMF1	polyamine modulated factor 1 [Source:HGNC Symbol;Acc:HGNC:911	1.22E-08
10	TP53RK	TP53 regulating kinase [Source:HGNC Symbol;Acc:HGNC:16197]	6.76E-12
10	GALE	UDP-galactose-4-epimerase [Source:HGNC Symbol;Acc:HGNC:4116]	1.04E-12
10	TMEM106C	transmembrane protein 106C [Source:HGNC Symbol;Acc:HGNC:2877	7.74E-08
10	NDUFA3	NADH:ubiquinone oxidoreductase subunit A3 [Source:HGNC Symbol;	5.34E-15
10	METTL18	methyltransferase like 18 [Source:HGNC Symbol;Acc:HGNC:28793]	6.66E-12
10	MED19	mediator complex subunit 19 [Source:HGNC Symbol;Acc:HGNC:2960	1.10E-10
10	IGFBP4	insulin like growth factor binding protein 4 [Source:HGNC Symbol;Ac	2.33E-11
10	TMEM8B	transmembrane protein 8B [Source:HGNC Symbol;Acc:HGNC:21427]	2.84E-09
10	PIGP	phosphatidylinositol glycan anchor biosynthesis class P [Source:HGN	3.34E-10
10	NDUFA1	NADH:ubiquinone oxidoreductase subunit A1 [Source:HGNC Symbol;	6.24E-07
10	GOSR1	golgi SNAP receptor complex member 1 [Source:HGNC Symbol;Acc:H	5.62E-10
10	WDR61	WD repeat domain 61 [Source:HGNC Symbol;Acc:HGNC:30300]	1.06E-07
10	IDUA	alpha-L-iduronidase [Source:HGNC Symbol;Acc:HGNC:5391]	1.25E-11
10	MANSC1	MANSC domain containing 1 [Source:HGNC Symbol;Acc:HGNC:25505	1.19E-15
10	MT-CO2	mitochondrially encoded cytochrome c oxidase II [Source:HGNC Sym	6.82E-19
10	AC025154.2	novel transcript, antisense to AQP5	1.55E-14
10	GNPDA1	glucosamine-6-phosphate deaminase 1 [Source:HGNC Symbol;Acc:H	2.39E-08
10	ST3GAL6	ST3 beta-galactoside alpha-2,3-sialyltransferase 6 [Source:HGNC Sym	7.40E-09
10	RHPN1	rhophilin Rho GTPase binding protein 1 [Source:HGNC Symbol;Acc:H	1.96E-09

10	ARRDC1	arrestin domain containing 1 [Source:HGNC Symbol;Acc:HGNC:2863	1.41E-07
10	ATP1A1	ATPase Na+/K+ transporting subunit alpha 1 [Source:HGNC Symbol;A	7.21E-12
10	PTPRF	protein tyrosine phosphatase receptor type F [Source:HGNC Symbol;	2.78E-09
10	TMED9	transmembrane p24 trafficking protein 9 [Source:HGNC Symbol;Acc:	6.23E-08
10	HAGHL	hydroxyacylglutathione hydrolase like [Source:HGNC Symbol;Acc:HG	1.71E-11
10	PLPP5	phospholipid phosphatase 5 [Source:HGNC Symbol;Acc:HGNC:25026	1.33E-11
10	ANKRD30A	ankyrin repeat domain 30A [Source:HGNC Symbol;Acc:HGNC:17234]	6.55E-14
10	DYNLL1	dynein light chain LC8-type 1 [Source:HGNC Symbol;Acc:HGNC:1547	2.09E-13
10	B3GALT5-AS1	B3GALT5 antisense RNA 1 [Source:HGNC Symbol;Acc:HGNC:16424]	3.77E-10
10	ALDH3B2	aldehyde dehydrogenase 3 family member B2 [Source:HGNC Symbol	3.22E-16
10	STAU1	staufen double-stranded RNA binding protein 1 [Source:HGNC Symb	6.52E-10
10	TMEM177	transmembrane protein 177 [Source:HGNC Symbol;Acc:HGNC:28143	9.75E-17
10	UBXN4	UBX domain protein 4 [Source:HGNC Symbol;Acc:HGNC:14860]	3.38E-08
10	MAP3K20	mitogen-activated protein kinase kinase kinase 20 [Source:HGNC Syr	9.58E-08
10	LRRK2	leucine rich repeat kinase 2 [Source:HGNC Symbol;Acc:HGNC:18618]	3.34E-09
10	ARPC5L	actin related protein 2/3 complex subunit 5 like [Source:HGNC Symb	4.49E-07
10	SRSF9	serine and arginine rich splicing factor 9 [Source:HGNC Symbol;Acc:H	2.82E-09
10	PUF60	poly(U) binding splicing factor 60 [Source:HGNC Symbol;Acc:HGNC:1	7.41E-10
10	REXO2	RNA exonuclease 2 [Source:HGNC Symbol;Acc:HGNC:17851]	8.99E-08
10	KYAT3	kynurenine aminotransferase 3 [Source:HGNC Symbol;Acc:HGNC:33	1.38E-08
10	NME6	NME/NM23 nucleoside diphosphate kinase 6 [Source:HGNC Symbol;	9.84E-09
10	IP6K2	inositol hexakisphosphate kinase 2 [Source:HGNC Symbol;Acc:HGNC	1.69E-13
10	SMPDL3B	sphingomyelin phosphodiesterase acid like 3B [Source:HGNC Symbol	3.41E-15
10	MT-ND1	mitochondrially encoded NADH:ubiquinone oxidoreductase core sub	3.28E-16
10	PTGFRN	prostaglandin F2 receptor inhibitor [Source:HGNC Symbol;Acc:HGNC	3.58E-13
10	SERPINB6	serpin family B member 6 [Source:HGNC Symbol;Acc:HGNC:8950]	5.68E-08
10	NR2C2AP	nuclear receptor 2C2 associated protein [Source:HGNC Symbol;Acc:H	1.07E-08
10	TRIB3	tribbles pseudokinase 3 [Source:HGNC Symbol;Acc:HGNC:16228]	4.94E-11
10	PWWP2B	PWWP domain containing 2B [Source:HGNC Symbol;Acc:HGNC:2515	5.74E-08
10	AL049839.2	novel protein	1.74E-14
10	CRIP2	cysteine rich protein 2 [Source:HGNC Symbol;Acc:HGNC:2361]	2.19E-09
10	PSMA2	proteasome 20S subunit alpha 2 [Source:HGNC Symbol;Acc:HGNC:95	4.84E-07
10	ARHGEF35	Rho guanine nucleotide exchange factor 35 [Source:HGNC Symbol;A	1.43E-07
10	BICDL2	BICD family like cargo adaptor 2 [Source:HGNC Symbol;Acc:HGNC:33	1.99E-18
10	CYB5R1	cytochrome b5 reductase 1 [Source:HGNC Symbol;Acc:HGNC:13397]	1.75E-09
10	BTG3	BTG anti-proliferation factor 3 [Source:HGNC Symbol;Acc:HGNC:113	5.39E-07
10	MIF	macrophage migration inhibitory factor [Source:HGNC Symbol;Acc:H	1.13E-07
10	UNC50	unc-50 inner nuclear membrane RNA binding protein [Source:HGNC	5.29E-07
10	CEACAM6	CEA cell adhesion molecule 6 [Source:HGNC Symbol;Acc:HGNC:1818	2.25E-10
10	RAB17	RAB17, member RAS oncogene family [Source:HGNC Symbol;Acc:HG	1.03E-16
10	DHFR	dihydrofolate reductase [Source:HGNC Symbol;Acc:HGNC:2861]	5.21E-08
10	NCOA7	nuclear receptor coactivator 7 [Source:HGNC Symbol;Acc:HGNC:210	6.48E-12
10	AL355338.1	novel transcript	3.69E-12
10	PRRG2	proline rich and Gla domain 2 [Source:HGNC Symbol;Acc:HGNC:9470	1.10E-12
10	ARPIN	actin related protein 2/3 complex inhibitor [Source:HGNC Symbol;Ac	2.59E-10
10	VKORC1	vitamin K epoxide reductase complex subunit 1 [Source:HGNC Symb	3.41E-10
10	MPZL1	myelin protein zero like 1 [Source:HGNC Symbol;Acc:HGNC:7226]	1.44E-07

10	TSPAN6	tetraspanin 6 [Source:HGNC Symbol;Acc:HGNC:11858]	7.00E-14
10	NUDT2	nudix hydrolase 2 [Source:HGNC Symbol;Acc:HGNC:8049]	2.59E-09
10	SAR1B	secretion associated Ras related GTPase 1B [Source:HGNC Symbol;A	4.73E-10
10	ALDH9A1	aldehyde dehydrogenase 9 family member A1 [Source:HGNC Symbol	1.18E-08
10	CADPS2	calcium dependent secretion activator 2 [Source:HGNC Symbol;Acc:H	1.42E-16
10	RAB40B	RAB40B, member RAS oncogene family [Source:HGNC Symbol;Acc:H	5.72E-13
10	GGPS1	geranylgeranyl diphosphate synthase 1 [Source:HGNC Symbol;Acc:H	1.62E-08
10	MTUS1	microtubule associated scaffold protein 1 [Source:HGNC Symbol;Acc	1.27E-14
10	CBWD1	COBW domain containing 1 [Source:HGNC Symbol;Acc:HGNC:17134]	4.19E-08
10	MED8	mediator complex subunit 8 [Source:HGNC Symbol;Acc:HGNC:19971	2.87E-07
10	CETN2	centrin 2 [Source:HGNC Symbol;Acc:HGNC:1867]	6.35E-08
10	PMEPA1	prostate transmembrane protein, androgen induced 1 [Source:HGNC	5.69E-08
10	DNAAF4	dynein axonemal assembly factor 4 [Source:HGNC Symbol;Acc:HGNC	3.06E-13
10	CLPTM1	CLPTM1 regulator of GABA type A receptor forward trafficking [Sour	1.34E-10
10	MAGEF1	MAGE family member F1 [Source:HGNC Symbol;Acc:HGNC:29639]	7.00E-08
10	TMEM125	transmembrane protein 125 [Source:HGNC Symbol;Acc:HGNC:28275	7.92E-16
10	EMC6	ER membrane protein complex subunit 6 [Source:HGNC Symbol;Acc:	2.51E-08
10	SSU72	SSU72 homolog, RNA polymerase II CTD phosphatase [Source:HGNC	2.32E-07
10	HDGFL3	HDGF like 3 [Source:HGNC Symbol;Acc:HGNC:24937]	1.32E-07
10	TJP3	tight junction protein 3 [Source:HGNC Symbol;Acc:HGNC:11829]	8.98E-14
10	RASAL2	RAS protein activator like 2 [Source:HGNC Symbol;Acc:HGNC:9874]	6.52E-13
10	PTPA	protein phosphatase 2 phosphatase activator [Source:HGNC Symbol;	3.02E-07
10	HSD17B8	hydroxysteroid 17-beta dehydrogenase 8 [Source:HGNC Symbol;Acc:	4.01E-07
10	SRP14	signal recognition particle 14 [Source:HGNC Symbol;Acc:HGNC:1129	6.25E-07
10	SSPN	sarcospan [Source:HGNC Symbol;Acc:HGNC:11322]	7.41E-10
10	IVD	isovaleryl-CoA dehydrogenase [Source:HGNC Symbol;Acc:HGNC:618	1.15E-08
10	CYP2J2	cytochrome P450 family 2 subfamily J member 2 [Source:HGNC Sym	3.08E-13
10	FMOD	fibromodulin [Source:HGNC Symbol;Acc:HGNC:3774]	1.55E-12
10	PXMP2	peroxisomal membrane protein 2 [Source:HGNC Symbol;Acc:HGNC:	2.25E-08
10	C20orf96	chromosome 20 open reading frame 96 [Source:HGNC Symbol;Acc:H	7.77E-09
10	CIB2	calcium and integrin binding family member 2 [Source:HGNC Symbol	8.26E-08
10	UQCR11	ubiquinol-cytochrome c reductase, complex III subunit XI [Source:HG	1.57E-08
10	FMC1	formation of mitochondrial complex V assembly factor 1 homolog [S	1.34E-07
10	SDSL	serine dehydratase like [Source:HGNC Symbol;Acc:HGNC:30404]	5.42E-10
10	YIPF2	Yip1 domain family member 2 [Source:HGNC Symbol;Acc:HGNC:284	1.23E-11
10	IFT43	intraflagellar transport 43 [Source:HGNC Symbol;Acc:HGNC:29669]	1.69E-11
10	PEX16	peroxisomal biogenesis factor 16 [Source:HGNC Symbol;Acc:HGNC:8	2.08E-07
10	EPN2	epsin 2 [Source:HGNC Symbol;Acc:HGNC:18639]	6.75E-12
10	CMC1	C-X9-C motif containing 1 [Source:HGNC Symbol;Acc:HGNC:28783]	4.01E-09
10	MPZL2	myelin protein zero like 2 [Source:HGNC Symbol;Acc:HGNC:3496]	1.08E-12
10	SPR	sepiapterin reductase [Source:HGNC Symbol;Acc:HGNC:11257]	1.06E-11
10	POR	cytochrome p450 oxidoreductase [Source:HGNC Symbol;Acc:HGNC:	2.38E-12
10	EFNA4	ephrin A4 [Source:HGNC Symbol;Acc:HGNC:3224]	2.73E-09
10	KCTD3	potassium channel tetramerization domain containing 3 [Source:HG	6.00E-07
10	GPATCH2	G-patch domain containing 2 [Source:HGNC Symbol;Acc:HGNC:2549	1.93E-07
10	NFKBIL1	NFKB inhibitor like 1 [Source:HGNC Symbol;Acc:HGNC:7800]	1.22E-07
10	RETREG1	reticulophagy regulator 1 [Source:HGNC Symbol;Acc:HGNC:25964]	1.80E-08

10	P2RY2	purinergic receptor P2Y2 [Source:HGNC Symbol;Acc:HGNC:8541]	4.93E-08
10	SHARPIN	SHANK associated RH domain interactor [Source:HGNC Symbol;Acc:H	3.32E-11
10	OAZ3	ornithine decarboxylase antizyme 3 [Source:HGNC Symbol;Acc:HGN	9.58E-12
10	CANT1	calcium activated nucleotidase 1 [Source:HGNC Symbol;Acc:HGNC:1	1.37E-13
10	RTKN	rhotekin [Source:HGNC Symbol;Acc:HGNC:10466]	7.67E-16
10	ZNF138	zinc finger protein 138 [Source:HGNC Symbol;Acc:HGNC:12922]	4.31E-08
10	LZIC	leucine zipper and CTNNBIP1 domain containing [Source:HGNC Symb	1.80E-08
10	RAB2A	RAB2A, member RAS oncogene family [Source:HGNC Symbol;Acc:HG	1.84E-08
10	RPP25L	ribonuclease P/MRP subunit p25 like [Source:HGNC Symbol;Acc:HGN	1.00E-08
10	MAP1LC3A	microtubule associated protein 1 light chain 3 alpha [Source:HGNC S	3.30E-08
10	ZNF326	zinc finger protein 326 [Source:HGNC Symbol;Acc:HGNC:14104]	5.22E-07
10	CHRAC1	chromatin accessibility complex subunit 1 [Source:HGNC Symbol;Acc	6.19E-08
10	LDLRAD3	low density lipoprotein receptor class A domain containing 3 [Source	1.58E-07
10	NDUFA5	NADH:ubiquinone oxidoreductase subunit A5 [Source:HGNC Symbol;	2.98E-13
10	B9D1	B9 domain containing 1 [Source:HGNC Symbol;Acc:HGNC:24123]	1.00E-11
10	SHMT2	serine hydroxymethyltransferase 2 [Source:HGNC Symbol;Acc:HGNC	9.27E-11
10	CLPP	caseinolytic mitochondrial matrix peptidase proteolytic subunit [Sou	5.17E-07
10	CCAR1	cell division cycle and apoptosis regulator 1 [Source:HGNC Symbol;A	9.54E-08
10	TMED4	transmembrane p24 trafficking protein 4 [Source:HGNC Symbol;Acc:	1.58E-08
10	FANCF	FA complementation group F [Source:HGNC Symbol;Acc:HGNC:3587	3.16E-08
10	VWA1	von Willebrand factor A domain containing 1 [Source:HGNC Symbol;	4.29E-09
10	QTRT1	queuine tRNA-ribosyltransferase catalytic subunit 1 [Source:HGNC Sy	2.71E-09
10	TMEM25	transmembrane protein 25 [Source:HGNC Symbol;Acc:HGNC:25890]	7.02E-12
10	BPHL	biphenyl hydrolase like [Source:HGNC Symbol;Acc:HGNC:1094]	3.99E-08
10	SHROOM1	shroom family member 1 [Source:HGNC Symbol;Acc:HGNC:24084]	6.46E-15
10	WDR55	WD repeat domain 55 [Source:HGNC Symbol;Acc:HGNC:25971]	6.79E-15
10	MPV17	mitochondrial inner membrane protein MPV17 [Source:HGNC Symbo	6.00E-08
10	SIM2	SIM bHLH transcription factor 2 [Source:HGNC Symbol;Acc:HGNC:10	4.51E-14
10	HID1	HID1 domain containing [Source:HGNC Symbol;Acc:HGNC:15736]	3.77E-12
10	UBE2V1	ubiquitin conjugating enzyme E2 V1 [Source:HGNC Symbol;Acc:HGN	1.13E-08
10	C16orf87	chromosome 16 open reading frame 87 [Source:HGNC Symbol;Acc:H	1.25E-10
10	SF3A1	splicing factor 3a subunit 1 [Source:HGNC Symbol;Acc:HGNC:10765]	6.31E-14
10	MAP3K5	mitogen-activated protein kinase kinase kinase 5 [Source:HGNC Sym	2.84E-15
10	NUTM2A-AS1	NUTM2A antisense RNA 1 [Source:HGNC Symbol;Acc:HGNC:45161]	1.71E-09
10	GNA13	G protein subunit alpha 13 [Source:HGNC Symbol;Acc:HGNC:4381]	4.12E-20
10	IPCEF1	interaction protein for cytohesin exchange factors 1 [Source:HGNC S	6.09E-10
10	RPS3	ribosomal protein S3 [Source:HGNC Symbol;Acc:HGNC:10420]	6.94E-18
10	HOTAIRM1	HOXA transcript antisense RNA, myeloid-specific 1 [Source:HGNC Sym	1.74E-25
10	KIAA0513	KIAA0513 [Source:HGNC Symbol;Acc:HGNC:29058]	4.35E-34
10	NUMB	NUMB endocytic adaptor protein [Source:HGNC Symbol;Acc:HGNC:8	8.72E-09
10	SYMPK	symplekin [Source:HGNC Symbol;Acc:HGNC:22935]	1.13E-11
10	EPS15	epidermal growth factor receptor pathway substrate 15 [Source:HGN	2.96E-08
10	TAX1BP3	Tax1 binding protein 3 [Source:HGNC Symbol;Acc:HGNC:30684]	1.81E-07
10	E2F3	E2F transcription factor 3 [Source:HGNC Symbol;Acc:HGNC:3115]	5.65E-20
10	CD300A	CD300a molecule [Source:HGNC Symbol;Acc:HGNC:19319]	5.45E-07
10	MEF2A	myocyte enhancer factor 2A [Source:HGNC Symbol;Acc:HGNC:6993]	2.14E-08
10	HIPK2	homeodomain interacting protein kinase 2 [Source:HGNC Symbol;Ac	1.24E-08

10	TET2	tet methylcytosine dioxygenase 2 [Source:HGNC Symbol;Acc:HGNC:2	1.04E-11
10	SLA	Src like adaptor [Source:HGNC Symbol;Acc:HGNC:10902]	3.07E-09
10	MAN1A1	mannosidase alpha class 1A member 1 [Source:HGNC Symbol;Acc:HG	1.62E-16
10	BICD2	BICD cargo adaptor 2 [Source:HGNC Symbol;Acc:HGNC:17208]	3.18E-21
10	AOAH	acyloxyacyl hydrolase [Source:HGNC Symbol;Acc:HGNC:548]	1.71E-09
10	ARHGEF3	Rho guanine nucleotide exchange factor 3 [Source:HGNC Symbol;Acc	1.47E-09
10	ARHGAP4	Rho GTPase activating protein 4 [Source:HGNC Symbol;Acc:HGNC:67	4.08E-09
10	PAN3	poly(A) specific ribonuclease subunit PAN3 [Source:HGNC Symbol;Ac	9.98E-08
10	ATP6V1B2	ATPase H+ transporting V1 subunit B2 [Source:HGNC Symbol;Acc:HG	4.37E-30
10	CBL	Cbl proto-oncogene [Source:HGNC Symbol;Acc:HGNC:1541]	1.64E-17
10	PPP6R1	protein phosphatase 6 regulatory subunit 1 [Source:HGNC Symbol;Ac	8.02E-13
10	ADIPOR2	adiponectin receptor 2 [Source:HGNC Symbol;Acc:HGNC:24041]	1.72E-10
10	CHIC2	cysteine rich hydrophobic domain 2 [Source:HGNC Symbol;Acc:HGNC	1.38E-09
10	KLHL6	kelch like family member 6 [Source:HGNC Symbol;Acc:HGNC:18653]	7.16E-19
10	FOXO3	forkhead box O3 [Source:HGNC Symbol;Acc:HGNC:3821]	5.41E-07
10	SLC6A6	solute carrier family 6 member 6 [Source:HGNC Symbol;Acc:HGNC:1	4.59E-22
10	TPP1	tripeptidyl peptidase 1 [Source:HGNC Symbol;Acc:HGNC:2073]	5.94E-18
10	KCNAB2	potassium voltage-gated channel subfamily A regulatory beta subuni	7.41E-09
10	ARRDC3	arrestin domain containing 3 [Source:HGNC Symbol;Acc:HGNC:2926	4.73E-08
10	ITGAM	integrin subunit alpha M [Source:HGNC Symbol;Acc:HGNC:6149]	8.92E-24
10	RPL36	ribosomal protein L36 [Source:HGNC Symbol;Acc:HGNC:13631]	2.20E-12
10	STRN4	striatin 4 [Source:HGNC Symbol;Acc:HGNC:15721]	2.13E-16
10	CEP85L	centrosomal protein 85 like [Source:HGNC Symbol;Acc:HGNC:21638]	5.22E-07
10	DCK	deoxycytidine kinase [Source:HGNC Symbol;Acc:HGNC:2704]	5.15E-07
10	RPS6KA1	ribosomal protein S6 kinase A1 [Source:HGNC Symbol;Acc:HGNC:104	8.27E-09
10	LPIN2	lipin 2 [Source:HGNC Symbol;Acc:HGNC:14450]	4.95E-09
10	SESN3	sestrin 3 [Source:HGNC Symbol;Acc:HGNC:23060]	1.98E-08
10	EMD	emerin [Source:HGNC Symbol;Acc:HGNC:3331]	2.53E-09
10	CCDC88A	coiled-coil domain containing 88A [Source:HGNC Symbol;Acc:HGNC:2	5.65E-19
10	LY86	lymphocyte antigen 86 [Source:HGNC Symbol;Acc:HGNC:16837]	6.41E-09
10	SNX9	sorting nexin 9 [Source:HGNC Symbol;Acc:HGNC:14973]	3.61E-12
10	KLHL5	kelch like family member 5 [Source:HGNC Symbol;Acc:HGNC:6356]	4.33E-14
10	KLF10	Kruppel like factor 10 [Source:HGNC Symbol;Acc:HGNC:11810]	3.90E-10
10	SPPL3	signal peptide peptidase like 3 [Source:HGNC Symbol;Acc:HGNC:304	1.72E-15
10	DGKD	diacylglycerol kinase delta [Source:HGNC Symbol;Acc:HGNC:2851]	3.16E-07
10	CPNE5	copine 5 [Source:HGNC Symbol;Acc:HGNC:2318]	1.69E-17
10	PRKX	protein kinase X-linked [Source:HGNC Symbol;Acc:HGNC:9441]	1.71E-07
10	PLEKHA2	pleckstrin homology domain containing A2 [Source:HGNC Symbol;Ac	2.68E-08
10	AMFR	autocrine motility factor receptor [Source:HGNC Symbol;Acc:HGNC:4	1.53E-17
10	MPST	mercaptopyruvate sulfurtransferase [Source:HGNC Symbol;Acc:HGN	7.11E-12
10	GNB5	G protein subunit beta 5 [Source:HGNC Symbol;Acc:HGNC:4401]	5.40E-11
10	PHF20L1	PHD finger protein 20 like 1 [Source:HGNC Symbol;Acc:HGNC:24280]	1.48E-09
10	MTFR1L	mitochondrial fission regulator 1 like [Source:HGNC Symbol;Acc:HGN	2.43E-10
10	PLXDC2	plexin domain containing 2 [Source:HGNC Symbol;Acc:HGNC:21013]	1.13E-39
10	SAMSN1	SAM domain, SH3 domain and nuclear localization signals 1 [Source:I	3.82E-08
10	LAT2	linker for activation of T cells family member 2 [Source:HGNC Symbo	6.63E-12
10	CDYL	chromodomain Y like [Source:HGNC Symbol;Acc:HGNC:1811]	1.02E-13

10	IRS2	insulin receptor substrate 2 [Source:HGNC Symbol;Acc:HGNC:6126]	8.53E-23
10	ARRB1	arrestin beta 1 [Source:HGNC Symbol;Acc:HGNC:711]	2.59E-27
10	THEMIS2	thymocyte selection associated family member 2 [Source:HGNC Sym	8.72E-09
10	RPS16	ribosomal protein S16 [Source:HGNC Symbol;Acc:HGNC:10396]	1.31E-17
10	SUSD1	sushi domain containing 1 [Source:HGNC Symbol;Acc:HGNC:25413]	1.37E-24
10	FBXO7	F-box protein 7 [Source:HGNC Symbol;Acc:HGNC:13586]	1.54E-10
10	C11orf21	chromosome 11 open reading frame 21 [Source:HGNC Symbol;Acc:H	1.27E-14
10	C1GALT1	core 1 synthase, glycoprotein-N-acetylgalactosamine 3-beta-galactos	2.72E-11
10	HERC1	HECT and RLD domain containing E3 ubiquitin protein ligase family m	3.66E-08
10	CHCHD7	coiled-coil-helix-coiled-coil-helix domain containing 7 [Source:HGNC	1.53E-07
10	YPEL2	yippee like 2 [Source:HGNC Symbol;Acc:HGNC:18326]	1.39E-17
10	BACH1	BTB domain and CNC homolog 1 [Source:HGNC Symbol;Acc:HGNC:93	4.44E-20
10	MSL3	MSL complex subunit 3 [Source:HGNC Symbol;Acc:HGNC:7370]	3.22E-08
10	DYRK1A	dual specificity tyrosine phosphorylation regulated kinase 1A [Source	4.61E-18
10	SSBP3	single stranded DNA binding protein 3 [Source:HGNC Symbol;Acc:HG	9.20E-14
10	MAP4K5	mitogen-activated protein kinase kinase kinase kinase 5 [Source:HGN	3.27E-13
10	MAPK14	mitogen-activated protein kinase 14 [Source:HGNC Symbol;Acc:HGN	6.99E-15
10	TACC1	transforming acidic coiled-coil containing protein 1 [Source:HGNC Sy	1.32E-15
10	AC004687.1	novel transcript, MIR142 host	2.87E-09
10	RPS14	ribosomal protein S14 [Source:HGNC Symbol;Acc:HGNC:10387]	1.04E-23
10	CX3CR1	C-X3-C motif chemokine receptor 1 [Source:HGNC Symbol;Acc:HGNC	9.91E-08
10	PNMA1	PNMA family member 1 [Source:HGNC Symbol;Acc:HGNC:9158]	1.35E-10
10	STK38	serine/threonine kinase 38 [Source:HGNC Symbol;Acc:HGNC:17847]	2.77E-09
10	MAP4K2	mitogen-activated protein kinase kinase kinase kinase 2 [Source:HGN	1.05E-16
10	SATB1	SATB homeobox 1 [Source:HGNC Symbol;Acc:HGNC:10541]	8.54E-08
10	PCGF5	polycomb group ring finger 5 [Source:HGNC Symbol;Acc:HGNC:2826	9.37E-11
10	PHKB	phosphorylase kinase regulatory subunit beta [Source:HGNC Symbol	1.34E-15
10	TKT	transketolase [Source:HGNC Symbol;Acc:HGNC:11834]	1.89E-07
10	RPL26	ribosomal protein L26 [Source:HGNC Symbol;Acc:HGNC:10327]	4.15E-21
10	ST3GAL1	ST3 beta-galactoside alpha-2,3-sialyltransferase 1 [Source:HGNC Sym	1.34E-07
10	NFAT5	nuclear factor of activated T cells 5 [Source:HGNC Symbol;Acc:HGNC	4.55E-14
10	TSPAN32	tetraspanin 32 [Source:HGNC Symbol;Acc:HGNC:13410]	1.96E-15
10	SERPINB9	serpin family B member 9 [Source:HGNC Symbol;Acc:HGNC:8955]	5.02E-13
10	ERBIN	erbb2 interacting protein [Source:HGNC Symbol;Acc:HGNC:15842]	9.25E-08
10	CMTM3	CKLF like MARVEL transmembrane domain containing 3 [Source:HGN	7.78E-08
10	C1orf21	chromosome 1 open reading frame 21 [Source:HGNC Symbol;Acc:HG	3.54E-08
10	CCDC88C	coiled-coil domain containing 88C [Source:HGNC Symbol;Acc:HGNC:1	1.73E-09
10	CNOT6L	CCR4-NOT transcription complex subunit 6 like [Source:HGNC Symbo	1.38E-11
10	JAK3	Janus kinase 3 [Source:HGNC Symbol;Acc:HGNC:6193]	6.59E-10
10	TBXAS1	thromboxane A synthase 1 [Source:HGNC Symbol;Acc:HGNC:11609]	3.40E-50
10	C6orf62	chromosome 6 open reading frame 62 [Source:HGNC Symbol;Acc:HG	2.19E-08
10	SNRK	SNF related kinase [Source:HGNC Symbol;Acc:HGNC:30598]	4.11E-08
10	ARID1A	AT-rich interaction domain 1A [Source:HGNC Symbol;Acc:HGNC:111	1.37E-09
10	CCDC69	coiled-coil domain containing 69 [Source:HGNC Symbol;Acc:HGNC:24	9.18E-09
10	DUSP22	dual specificity phosphatase 22 [Source:HGNC Symbol;Acc:HGNC:160	6.06E-12
10	CLCN3	chloride voltage-gated channel 3 [Source:HGNC Symbol;Acc:HGNC:2	5.74E-07
10	RNF145	ring finger protein 145 [Source:HGNC Symbol;Acc:HGNC:20853]	1.58E-12

10	SELP	selectin P [Source:HGNC Symbol;Acc:HGNC:10721]	5.19E-11
10	RPL32	ribosomal protein L32 [Source:HGNC Symbol;Acc:HGNC:10336]	2.44E-30
10	ORAI2	ORAI calcium release-activated calcium modulator 2 [Source:HGNC S	3.19E-10
10	TGFBR2	transforming growth factor beta receptor 2 [Source:HGNC Symbol;A	9.26E-13
10	RPL29	ribosomal protein L29 [Source:HGNC Symbol;Acc:HGNC:10331]	7.71E-36
10	SNX10	sorting nexin 10 [Source:HGNC Symbol;Acc:HGNC:14974]	8.18E-10
10	PRR7	proline rich 7, synaptic [Source:HGNC Symbol;Acc:HGNC:28130]	1.00E-14
10	MSANTD3	Myb/SANT DNA binding domain containing 3 [Source:HGNC Symbol;	4.62E-11
10	SUSD3	sushi domain containing 3 [Source:HGNC Symbol;Acc:HGNC:28391]	9.13E-09
10	HNRNPA2B1	heterogeneous nuclear ribonucleoprotein A2/B1 [Source:HGNC Sym	6.31E-08
10	GABARAPL1	GABA type A receptor associated protein like 1 [Source:HGNC Symbo	1.63E-07
10	TMX4	thioredoxin related transmembrane protein 4 [Source:HGNC Symbol	5.61E-08
10	MAP2K3	mitogen-activated protein kinase kinase 3 [Source:HGNC Symbol;Ac	6.97E-09
10	ADAM10	ADAM metallopeptidase domain 10 [Source:HGNC Symbol;Acc:HGN	1.14E-07
10	MT-ATP8	mitochondrially encoded ATP synthase membrane subunit 8 [Source	2.57E-10
10	SEMA4D	semaphorin 4D [Source:HGNC Symbol;Acc:HGNC:10732]	1.26E-07
10	IVNS1ABP	influenza virus NS1A binding protein [Source:HGNC Symbol;Acc:HGN	2.34E-07
10	FGD3	FYVE, RhoGEF and PH domain containing 3 [Source:HGNC Symbol;Ac	3.50E-08
10	PFN1	profilin 1 [Source:HGNC Symbol;Acc:HGNC:8881]	1.34E-11
10	MFNG	MFNG O-fucosylpeptide 3-beta-N-acetylglucosaminyltransferase [So	4.18E-12
10	ITGAX	integrin subunit alpha X [Source:HGNC Symbol;Acc:HGNC:6152]	7.80E-12
10	GPR183	G protein-coupled receptor 183 [Source:HGNC Symbol;Acc:HGNC:31	1.59E-08
10	C16orf54	chromosome 16 open reading frame 54 [Source:HGNC Symbol;Acc:H	2.58E-09
10	PACSIN2	protein kinase C and casein kinase substrate in neurons 2 [Source:HG	5.08E-14
10	ASB8	ankyrin repeat and SOCS box containing 8 [Source:HGNC Symbol;Acc	2.41E-11
10	CYREN	cell cycle regulator of NHEJ [Source:HGNC Symbol;Acc:HGNC:22432]	5.76E-12
10	GRK6	G protein-coupled receptor kinase 6 [Source:HGNC Symbol;Acc:HGN	1.07E-15
10	TPP2	tripeptidyl peptidase 2 [Source:HGNC Symbol;Acc:HGNC:12016]	2.66E-12
10	LY6G6F	lymphocyte antigen 6 family member G6F [Source:HGNC Symbol;Acc	8.18E-18
10	MTRNR2L12	MT-RNR2 like 12 [Source:HGNC Symbol;Acc:HGNC:37169]	5.63E-14
10	DPYD	dihydropyrimidine dehydrogenase [Source:HGNC Symbol;Acc:HGNC:	2.83E-15
10	IKBKG	inhibitor of nuclear factor kappa B kinase regulatory subunit gamma	1.47E-14
10	HELZ	helicase with zinc finger [Source:HGNC Symbol;Acc:HGNC:16878]	2.02E-14
10	GRK2	G protein-coupled receptor kinase 2 [Source:HGNC Symbol;Acc:HGN	9.71E-10
10	RGS19	regulator of G protein signaling 19 [Source:HGNC Symbol;Acc:HGNC:	3.04E-07
10	CALHM6	calcium homeostasis modulator family member 6 [Source:HGNC Sym	1.50E-09
10	RAB8B	RAB8B, member RAS oncogene family [Source:HGNC Symbol;Acc:HG	2.11E-14
10	IL16	interleukin 16 [Source:HGNC Symbol;Acc:HGNC:5980]	1.07E-08
10	TSPAN18	tetraspanin 18 [Source:HGNC Symbol;Acc:HGNC:20660]	1.89E-16
10	OSBP2	oxysterol binding protein 2 [Source:HGNC Symbol;Acc:HGNC:8504]	3.97E-10
10	FURIN	furin, paired basic amino acid cleaving enzyme [Source:HGNC Symbo	1.77E-24
10	HIPK1	homeodomain interacting protein kinase 1 [Source:HGNC Symbol;Ac	2.46E-16
10	INF2	inverted formin 2 [Source:HGNC Symbol;Acc:HGNC:23791]	2.06E-12
10	TAGAP	T cell activation RhoGTPase activating protein [Source:HGNC Symbol	1.45E-07
10	SLCO3A1	solute carrier organic anion transporter family member 3A1 [Source:	1.85E-16
10	RPS7	ribosomal protein S7 [Source:HGNC Symbol;Acc:HGNC:10440]	4.67E-24
10	RPS6KA3	ribosomal protein S6 kinase A3 [Source:HGNC Symbol;Acc:HGNC:104	7.05E-18

10	SSH2	slingshot protein phosphatase 2 [Source:HGNC Symbol;Acc:HGNC:30	2.03E-08
10	TNFSF13B	TNF superfamily member 13b [Source:HGNC Symbol;Acc:HGNC:1192	3.80E-42
10	CYB5R4	cytochrome b5 reductase 4 [Source:HGNC Symbol;Acc:HGNC:20147]	1.16E-19
10	TENT5C	terminal nucleotidyltransferase 5C [Source:HGNC Symbol;Acc:HGNC:	3.18E-12
10	RPL5	ribosomal protein L5 [Source:HGNC Symbol;Acc:HGNC:10360]	4.70E-27
10	XPNPEP1	X-prolyl aminopeptidase 1 [Source:HGNC Symbol;Acc:HGNC:12822]	9.71E-11
10	RILPL2	Rab interacting lysosomal protein like 2 [Source:HGNC Symbol;Acc:H	2.32E-07
10	UBE2J1	ubiquitin conjugating enzyme E2 J1 [Source:HGNC Symbol;Acc:HGNC	2.84E-12
10	RPS21	ribosomal protein S21 [Source:HGNC Symbol;Acc:HGNC:10409]	2.89E-19
10	AHCTF1	AT-hook containing transcription factor 1 [Source:HGNC Symbol;Acc:	5.75E-07
10	RPL35A	ribosomal protein L35a [Source:HGNC Symbol;Acc:HGNC:10345]	2.13E-08
10	ALDOA	aldolase, fructose-bisphosphate A [Source:HGNC Symbol;Acc:HGNC:4	6.85E-09
10	MEPCE	methylphosphate capping enzyme [Source:HGNC Symbol;Acc:HGNC:	7.74E-16
10	RASSF5	Ras association domain family member 5 [Source:HGNC Symbol;Acc:	3.84E-08
10	VPS13C	vacuolar protein sorting 13 homolog C [Source:HGNC Symbol;Acc:HG	1.38E-08
10	PTPN6	protein tyrosine phosphatase non-receptor type 6 [Source:HGNC Syr	3.14E-11
10	ZFP36	ZFP36 ring finger protein [Source:HGNC Symbol;Acc:HGNC:12862]	2.98E-08
10	DYNLL2	dynein light chain LC8-type 2 [Source:HGNC Symbol;Acc:HGNC:2459	3.49E-09
10	CFP	complement factor properdin [Source:HGNC Symbol;Acc:HGNC:8864	9.39E-11
10	PPP3R1	protein phosphatase 3 regulatory subunit B, alpha [Source:HGNC Syr	1.74E-20
10	WTAP	WT1 associated protein [Source:HGNC Symbol;Acc:HGNC:16846]	2.63E-10
10	CD96	CD96 molecule [Source:HGNC Symbol;Acc:HGNC:16892]	8.54E-08
10	AGTPBP1	ATP/GTP binding protein 1 [Source:HGNC Symbol;Acc:HGNC:17258]	2.41E-11
10	INAFM2	InaF motif containing 2 [Source:HGNC Symbol;Acc:HGNC:35165]	6.28E-32
10	RHOG	ras homolog family member G [Source:HGNC Symbol;Acc:HGNC:672	9.02E-08
10	STXBP3	syntaxin binding protein 3 [Source:HGNC Symbol;Acc:HGNC:11446]	7.20E-09
10	DSE	dermatan sulfate epimerase [Source:HGNC Symbol;Acc:HGNC:21144	1.93E-33
10	ZNF438	zinc finger protein 438 [Source:HGNC Symbol;Acc:HGNC:21029]	1.05E-18
10	PTPN7	protein tyrosine phosphatase non-receptor type 7 [Source:HGNC Syr	4.98E-08
10	CHD9	chromodomain helicase DNA binding protein 9 [Source:HGNC Symbo	6.31E-13
10	CDKN1A	cyclin dependent kinase inhibitor 1A [Source:HGNC Symbol;Acc:HGN	5.89E-13
10	DOCK11	dedicator of cytokinesis 11 [Source:HGNC Symbol;Acc:HGNC:23483]	5.43E-15
10	TMBIM1	transmembrane BAX inhibitor motif containing 1 [Source:HGNC Sym	1.50E-13
10	DGKZ	diacylglycerol kinase zeta [Source:HGNC Symbol;Acc:HGNC:2857]	2.25E-08
10	ARHGAP21	Rho GTPase activating protein 21 [Source:HGNC Symbol;Acc:HGNC:2	2.31E-11
10	LINC00623	long intergenic non-protein coding RNA 623 [Source:HGNC Symbol;A	2.90E-07
10	LCP2	lymphocyte cytosolic protein 2 [Source:HGNC Symbol;Acc:HGNC:652	1.98E-20
10	FOXN3	forkhead box N3 [Source:HGNC Symbol;Acc:HGNC:1928]	9.46E-13
10	DERA	deoxyribose-phosphate aldolase [Source:HGNC Symbol;Acc:HGNC:24	4.05E-08
10	ELK3	ETS transcription factor ELK3 [Source:HGNC Symbol;Acc:HGNC:3325]	4.03E-19
10	FCGRT	Fc fragment of IgG receptor and transporter [Source:HGNC Symbol;A	1.60E-07
10	CDV3	CDV3 homolog [Source:HGNC Symbol;Acc:HGNC:26928]	5.03E-08
10	PSME1	proteasome activator subunit 1 [Source:HGNC Symbol;Acc:HGNC:95	8.18E-09
10	VAMP5	vesicle associated membrane protein 5 [Source:HGNC Symbol;Acc:H	2.72E-10
10	HHEX	hematopoietically expressed homeobox [Source:HGNC Symbol;Acc:H	1.11E-22
10	MARK3	microtubule affinity regulating kinase 3 [Source:HGNC Symbol;Acc:H	7.71E-15
10	REL	REL proto-oncogene, NF-kB subunit [Source:HGNC Symbol;Acc:HGNC	9.14E-12

10	HLA-DQB1	major histocompatibility complex, class II, DQ beta 1 [Source:HGNC S	3.10E-08
10	UBA7	ubiquitin like modifier activating enzyme 7 [Source:HGNC Symbol;Ac	1.24E-12
10	P2RX1	purinergic receptor P2X 1 [Source:HGNC Symbol;Acc:HGNC:8533]	2.65E-36
10	OTUD5	OTU deubiquitinase 5 [Source:HGNC Symbol;Acc:HGNC:25402]	4.56E-14
10	HSD17B11	hydroxysteroid 17-beta dehydrogenase 11 [Source:HGNC Symbol;Ac	1.15E-08
10	CCDC71L	coiled-coil domain containing 71 like [Source:HGNC Symbol;Acc:HGN	3.42E-20
10	PSMA3-AS1	PSMA3 antisense RNA 1 [Source:HGNC Symbol;Acc:HGNC:26445]	1.09E-08
10	RNF166	ring finger protein 166 [Source:HGNC Symbol;Acc:HGNC:28856]	1.86E-11
10	HLA-DMB	major histocompatibility complex, class II, DM beta [Source:HGNC Sy	2.74E-08
10	RPL9	ribosomal protein L9 [Source:HGNC Symbol;Acc:HGNC:10369]	9.62E-12
10	AC245297.3	novel transcript	1.70E-13
10	CARD16	caspase recruitment domain family member 16 [Source:HGNC Symb	4.46E-07
10	STK10	serine/threonine kinase 10 [Source:HGNC Symbol;Acc:HGNC:11388]	3.15E-08
10	IFI27L2	interferon alpha inducible protein 27 like 2 [Source:HGNC Symbol;Ac	7.07E-21
10	RPS25	ribosomal protein S25 [Source:HGNC Symbol;Acc:HGNC:10413]	1.01E-08
10	FGR	FGR proto-oncogene, Src family tyrosine kinase [Source:HGNC Symb	4.23E-12
10	F2R	coagulation factor II thrombin receptor [Source:HGNC Symbol;Acc:H	3.37E-10
10	SLA2	Src like adaptor 2 [Source:HGNC Symbol;Acc:HGNC:17329]	2.72E-16
10	FHL1	four and a half LIM domains 1 [Source:HGNC Symbol;Acc:HGNC:3702	3.24E-13
10	CPEB4	cytoplasmic polyadenylation element binding protein 4 [Source:HGN	1.03E-09
10	CAPZB	capping actin protein of muscle Z-line subunit beta [Source:HGNC Sy	2.28E-08
10	RPS3A	ribosomal protein S3A [Source:HGNC Symbol;Acc:HGNC:10421]	1.19E-24
10	LBH	LBH regulator of WNT signaling pathway [Source:HGNC Symbol;Acc:H	8.45E-08
10	PDE4D	phosphodiesterase 4D [Source:HGNC Symbol;Acc:HGNC:8783]	1.43E-17
10	RPA1	replication protein A1 [Source:HGNC Symbol;Acc:HGNC:10289]	1.04E-15
10	TRA2A	transformer 2 alpha homolog [Source:HGNC Symbol;Acc:HGNC:1664	5.50E-08
10	PSIP1	PC4 and SFRS1 interacting protein 1 [Source:HGNC Symbol;Acc:HGN	1.31E-07
10	CDIP1	cell death inducing p53 target 1 [Source:HGNC Symbol;Acc:HGNC:13	2.81E-21
10	MAPK1	mitogen-activated protein kinase 1 [Source:HGNC Symbol;Acc:HGNC	1.55E-15
10	YIF1B	Yip1 interacting factor homolog B, membrane trafficking protein [Sou	8.36E-20
10	PRKACB	protein kinase cAMP-activated catalytic subunit beta [Source:HGNC S	1.57E-11
10	TNIK	TRAF2 and NCK interacting kinase [Source:HGNC Symbol;Acc:HGNC:3	1.57E-13
10	GLIPR2	GLI pathogenesis related 2 [Source:HGNC Symbol;Acc:HGNC:18007]	7.02E-12
10	PYGL	glycogen phosphorylase L [Source:HGNC Symbol;Acc:HGNC:9725]	3.31E-26
10	MT-ND2	mitochondrially encoded NADH:ubiquinone oxidoreductase core sub	1.87E-15
10	PRMT2	protein arginine methyltransferase 2 [Source:HGNC Symbol;Acc:HGN	1.35E-08
10	UTRN	utrophin [Source:HGNC Symbol;Acc:HGNC:12635]	1.27E-08
10	SH3KBP1	SH3 domain containing kinase binding protein 1 [Source:HGNC Symb	3.58E-08
10	CNN2	calponin 2 [Source:HGNC Symbol;Acc:HGNC:2156]	1.02E-11
10	PNP	purine nucleoside phosphorylase [Source:HGNC Symbol;Acc:HGNC:7	4.65E-17
10	CST3	cystatin C [Source:HGNC Symbol;Acc:HGNC:2475]	5.09E-24
10	LBR	lamin B receptor [Source:HGNC Symbol;Acc:HGNC:6518]	4.59E-14
10	RPLP2	ribosomal protein lateral stalk subunit P2 [Source:HGNC Symbol;Acc:	4.29E-33
10	MAP4K4	mitogen-activated protein kinase kinase kinase kinase 4 [Source:HGN	6.79E-14
10	GABARAP	GABA type A receptor-associated protein [Source:HGNC Symbol;Acc:	1.88E-08
10	SAV1	salvador family WW domain containing protein 1 [Source:HGNC Sym	2.10E-10
10	HBP1	HMG-box transcription factor 1 [Source:HGNC Symbol;Acc:HGNC:232	4.65E-10

10	MIS18BP1	MIS18 binding protein 1 [Source:HGNC Symbol;Acc:HGNC:20190]	3.21E-15
10	CENPT	centromere protein T [Source:HGNC Symbol;Acc:HGNC:25787]	2.98E-13
10	BANK1	B cell scaffold protein with ankyrin repeats 1 [Source:HGNC Symbol;A	7.42E-08
10	ATP2A3	ATPase sarcoplasmic/endoplasmic reticulum Ca2+ transporting 3 [So	4.12E-13
10	KAT6A	lysine acetyltransferase 6A [Source:HGNC Symbol;Acc:HGNC:13013]	4.25E-10
10	DOCK2	dedicator of cytokinesis 2 [Source:HGNC Symbol;Acc:HGNC:2988]	8.03E-14
10	G3BP2	G3BP stress granule assembly factor 2 [Source:HGNC Symbol;Acc:HG	2.19E-07
10	MINDY1	MINDY lysine 48 deubiquitinase 1 [Source:HGNC Symbol;Acc:HGNC:2	1.65E-14
10	RFLNB	refilin B [Source:HGNC Symbol;Acc:HGNC:28705]	8.95E-12
10	PSMB10	proteasome 20S subunit beta 10 [Source:HGNC Symbol;Acc:HGNC:9	8.23E-11
10	ANKRD9	ankyrin repeat domain 9 [Source:HGNC Symbol;Acc:HGNC:20096]	1.06E-10
10	CARD8	caspase recruitment domain family member 8 [Source:HGNC Symbo	9.52E-21
10	AGPAT1	1-acylglycerol-3-phosphate O-acyltransferase 1 [Source:HGNC Symb	1.88E-22
10	AC020916.1	novel transcript, antisense to ZSWIM4	1.06E-18
10	IDS	iduronate 2-sulfatase [Source:HGNC Symbol;Acc:HGNC:5389]	8.03E-12
10	PRKCD	protein kinase C delta [Source:HGNC Symbol;Acc:HGNC:9399]	1.31E-23
10	ARHGDIB	Rho GDP dissociation inhibitor beta [Source:HGNC Symbol;Acc:HGN	7.38E-21
10	ARL6IP5	ADP ribosylation factor like GTPase 6 interacting protein 5 [Source:H	2.21E-08
10	ENDOD1	endonuclease domain containing 1 [Source:HGNC Symbol;Acc:HGNC	5.23E-09
10	RABGAP1L	RAB GTPase activating protein 1 like [Source:HGNC Symbol;Acc:HGN	2.93E-13
10	CCND2	cyclin D2 [Source:HGNC Symbol;Acc:HGNC:1583]	2.62E-09
10	PIK3R1	phosphoinositide-3-kinase regulatory subunit 1 [Source:HGNC Symb	1.79E-10
10	FLI1	Fli-1 proto-oncogene, ETS transcription factor [Source:HGNC Symbol	1.18E-27
10	RASA3	RAS p21 protein activator 3 [Source:HGNC Symbol;Acc:HGNC:20331]	6.22E-17
10	APOBEC3C	apolipoprotein B mRNA editing enzyme catalytic subunit 3C [Source:	2.68E-22
10	CKLF	chemokine like factor [Source:HGNC Symbol;Acc:HGNC:13253]	3.13E-10
10	ARHGAP30	Rho GTPase activating protein 30 [Source:HGNC Symbol;Acc:HGNC:2	1.22E-10
10	CYB5R3	cytochrome b5 reductase 3 [Source:HGNC Symbol;Acc:HGNC:2873]	1.55E-11
10	NT5M	5',3'-nucleotidase, mitochondrial [Source:HGNC Symbol;Acc:HGNC:1	6.34E-09
10	TSC22D4	TSC22 domain family member 4 [Source:HGNC Symbol;Acc:HGNC:21	3.15E-13
10	PSMB8-AS1	PSMB8 antisense RNA 1 (head to head) [Source:HGNC Symbol;Acc:H	1.06E-14
10	PSMB8-AS1	PSMB8 antisense RNA 1 (head to head) [Source:NCBI gene (formerly	1.06E-14
10	MOB1B	MOB kinase activator 1B [Source:HGNC Symbol;Acc:HGNC:29801]	3.54E-26
10	SRSF8	serine and arginine rich splicing factor 8 [Source:HGNC Symbol;Acc:H	2.63E-11
10	MAFG	MAF bZIP transcription factor G [Source:HGNC Symbol;Acc:HGNC:67	7.40E-24
10	KCTD20	potassium channel tetramerization domain containing 20 [Source:HG	4.45E-26
10	TPM3	tropomyosin 3 [Source:HGNC Symbol;Acc:HGNC:12012]	9.47E-17
10	HLA-F	major histocompatibility complex, class I, F [Source:HGNC Symbol;Ac	1.50E-07
10	MYO1G	myosin IG [Source:HGNC Symbol;Acc:HGNC:13880]	1.69E-11
10	PARVB	parvin beta [Source:HGNC Symbol;Acc:HGNC:14653]	3.12E-21
10	FCGR2A	Fc fragment of IgG receptor IIa [Source:HGNC Symbol;Acc:HGNC:361	5.89E-28
10	FNTB	farnesyltransferase, CAAX box, beta [Source:HGNC Symbol;Acc:HGN	1.56E-21
10	PHACTR1	phosphatase and actin regulator 1 [Source:HGNC Symbol;Acc:HGNC:	2.46E-08
10	QKI	QKI, KH domain containing RNA binding [Source:HGNC Symbol;Acc:H	2.44E-26
10	FXYD5	FXYD domain containing ion transport regulator 5 [Source:HGNC Sym	2.65E-13
10	SERPINA1	serpin family A member 1 [Source:HGNC Symbol;Acc:HGNC:8941]	4.10E-10
10	CSK	C-terminal Src kinase [Source:HGNC Symbol;Acc:HGNC:2444]	2.99E-17

10	TERF2IP	TERF2 interacting protein [Source:HGNC Symbol;Acc:HGNC:19246]	6.83E-11
10	CFD	complement factor D [Source:HGNC Symbol;Acc:HGNC:2771]	3.63E-08
10	HLA-DMA	major histocompatibility complex, class II, DM alpha [Source:HGNC S	4.47E-12
10	KLF13	Kruppel like factor 13 [Source:HGNC Symbol;Acc:HGNC:13672]	8.22E-11
10	RPL39	ribosomal protein L39 [Source:HGNC Symbol;Acc:HGNC:10350]	4.48E-20
10	PABPN1	poly(A) binding protein nuclear 1 [Source:HGNC Symbol;Acc:HGNC:8	6.50E-09
10	TNRC6B	trinucleotide repeat containing adaptor 6B [Source:HGNC Symbol;Ac	5.30E-07
10	STX7	syntaxin 7 [Source:HGNC Symbol;Acc:HGNC:11442]	1.91E-26
10	HACD4	3-hydroxyacyl-CoA dehydratase 4 [Source:HGNC Symbol;Acc:HGNC:2	7.38E-09
10	EHD1	EH domain containing 1 [Source:HGNC Symbol;Acc:HGNC:3242]	4.47E-09
10	USP12	ubiquitin specific peptidase 12 [Source:HGNC Symbol;Acc:HGNC:204	1.85E-27
10	RPL31	ribosomal protein L31 [Source:HGNC Symbol;Acc:HGNC:10334]	4.40E-08
10	ARRB2	arrestin beta 2 [Source:HGNC Symbol;Acc:HGNC:712]	2.83E-10
10	RCSD1	RCSD domain containing 1 [Source:HGNC Symbol;Acc:HGNC:28310]	2.15E-11
10	CHMP4A	charged multivesicular body protein 4A [Source:HGNC Symbol;Acc:H	1.30E-16
10	KLRF1	killer cell lectin like receptor F1 [Source:HGNC Symbol;Acc:HGNC:133	8.93E-09
10	CORO1C	coronin 1C [Source:HGNC Symbol;Acc:HGNC:2254]	5.87E-53
10	ACTR3	actin related protein 3 [Source:HGNC Symbol;Acc:HGNC:170]	3.38E-09
10	TSPOAP1-AS1	TSPOAP1, SUPT4H1 and RNF43 antisense RNA 1 [Source:HGNC Symb	1.06E-16
10	PADI4	peptidyl arginine deiminase 4 [Source:HGNC Symbol;Acc:HGNC:1836	9.85E-18
10	PNISR	PNN interacting serine and arginine rich protein [Source:HGNC Symb	2.86E-07
10	TNFAIP8	TNF alpha induced protein 8 [Source:HGNC Symbol;Acc:HGNC:17260	9.35E-16
10	FMNL1	formin like 1 [Source:HGNC Symbol;Acc:HGNC:1212]	3.68E-10
10	TMSB10	thymosin beta 10 [Source:HGNC Symbol;Acc:HGNC:11879]	1.81E-31
10	PDE5A	phosphodiesterase 5A [Source:HGNC Symbol;Acc:HGNC:8784]	2.17E-13
10	ARPC2	actin related protein 2/3 complex subunit 2 [Source:HGNC Symbol;A	9.00E-13
10	FGFR1OP2	FGFR1 oncogene partner 2 [Source:HGNC Symbol;Acc:HGNC:23098]	1.49E-08
10	EMB	embigin [Source:HGNC Symbol;Acc:HGNC:30465]	3.68E-10
10	TSC22D3	TSC22 domain family member 3 [Source:HGNC Symbol;Acc:HGNC:30	1.24E-07
10	PSMB9	proteasome 20S subunit beta 9 [Source:HGNC Symbol;Acc:HGNC:954	1.71E-09
10	CYLD	CYLD lysine 63 deubiquitinase [Source:HGNC Symbol;Acc:HGNC:2584	4.18E-10
10	ANKRD28	ankyrin repeat domain 28 [Source:HGNC Symbol;Acc:HGNC:29024]	3.58E-28
10	PLA2G12A	phospholipase A2 group XIIA [Source:HGNC Symbol;Acc:HGNC:18554	1.79E-17
10	HLA-B	major histocompatibility complex, class I, B [Source:HGNC Symbol;Ac	4.43E-34
10	RPL6	ribosomal protein L6 [Source:HGNC Symbol;Acc:HGNC:10362]	2.91E-44
10	BTN3A2	butyrophilin subfamily 3 member A2 [Source:HGNC Symbol;Acc:HGN	4.97E-20
10	CCDC92	coiled-coil domain containing 92 [Source:HGNC Symbol;Acc:HGNC:29	2.22E-14
10	FNBP1	formin binding protein 1 [Source:HGNC Symbol;Acc:HGNC:17069]	2.98E-16
10	RPS12	ribosomal protein S12 [Source:HGNC Symbol;Acc:HGNC:10385]	4.90E-39
10	ANO6	anoctamin 6 [Source:HGNC Symbol;Acc:HGNC:25240]	4.61E-13
10	WAS	WASP actin nucleation promoting factor [Source:HGNC Symbol;Acc:H	1.18E-19
10	ASAP1	ArfGAP with SH3 domain, ankyrin repeat and PH domain 1 [Source:H	1.08E-12
10	LDLRAP1	low density lipoprotein receptor adaptor protein 1 [Source:HGNC Sy	4.15E-11
10	RPS15A	ribosomal protein S15a [Source:HGNC Symbol;Acc:HGNC:10389]	4.25E-14
10	RPL22	ribosomal protein L22 [Source:HGNC Symbol;Acc:HGNC:10315]	2.99E-33
10	GTPBP2	GTP binding protein 2 [Source:HGNC Symbol;Acc:HGNC:4670]	3.94E-24
10	RPL37A	ribosomal protein L37a [Source:HGNC Symbol;Acc:HGNC:10348]	8.50E-09

10	ACAP1	ArfGAP with coiled-coil, ankyrin repeat and PH domains 1 [Source:HG	1.37E-08
10	RPL7	ribosomal protein L7 [Source:HGNC Symbol;Acc:HGNC:10363]	2.82E-19
10	ADGRE5	adhesion G protein-coupled receptor E5 [Source:HGNC Symbol;Acc:H	6.09E-13
10	UBE2D3	ubiquitin conjugating enzyme E2 D3 [Source:HGNC Symbol;Acc:HGN	1.21E-10
10	SMAP2	small ArfGAP2 [Source:HGNC Symbol;Acc:HGNC:25082]	3.85E-10
10	BEND2	BEN domain containing 2 [Source:HGNC Symbol;Acc:HGNC:28509]	2.67E-12
10	RYBP	RING1 and YY1 binding protein [Source:HGNC Symbol;Acc:HGNC:104	8.42E-10
10	RYBP	RING1 and YY1 binding protein [Source:NCBI gene (formerly Entrezge	8.42E-10
10	CCDC85B	coiled-coil domain containing 85B [Source:HGNC Symbol;Acc:HGNC:2	1.90E-13
10	CYTIP	cytohesin 1 interacting protein [Source:HGNC Symbol;Acc:HGNC:950	1.05E-08
10	GLIPR1	GLI pathogenesis related 1 [Source:HGNC Symbol;Acc:HGNC:17001]	9.19E-11
10	HLA-A	major histocompatibility complex, class I, A [Source:HGNC Symbol;Ac	4.90E-29
10	TALDO1	transaldolase 1 [Source:HGNC Symbol;Acc:HGNC:11559]	8.01E-09
10	RNF24	ring finger protein 24 [Source:HGNC Symbol;Acc:HGNC:13779]	4.12E-35
10	PIP4P2	phosphatidylinositol-4,5-bisphosphate 4-phosphatase 2 [Source:HGN	7.82E-11
10	MYCBP2	MYC binding protein 2 [Source:HGNC Symbol;Acc:HGNC:23386]	2.33E-19
10	PECAM1	platelet and endothelial cell adhesion molecule 1 [Source:HGNC Sym	4.95E-42
10	RAB31	RAB31, member RAS oncogene family [Source:HGNC Symbol;Acc:HG	1.71E-45
10	PRKAR1B	protein kinase cAMP-dependent type I regulatory subunit beta [Sour	3.24E-22
10	DIAPH1	diaphanous related formin 1 [Source:HGNC Symbol;Acc:HGNC:2876]	1.38E-15
10	TBXA2R	thromboxane A2 receptor [Source:HGNC Symbol;Acc:HGNC:11608]	4.14E-25
10	TAOK3	TAO kinase 3 [Source:HGNC Symbol;Acc:HGNC:18133]	8.69E-09
10	PPP1R15A	protein phosphatase 1 regulatory subunit 15A [Source:HGNC Symbol	2.33E-12
10	ABHD17A	abhydrolase domain containing 17A, depalmitoylase [Source:HGNC S	7.55E-08
10	LCK	LCK proto-oncogene, Src family tyrosine kinase [Source:HGNC Symbo	7.20E-08
10	CBX3	chromobox 3 [Source:HGNC Symbol;Acc:HGNC:1553]	1.78E-07
10	HLA-C	major histocompatibility complex, class I, C [Source:HGNC Symbol;Ac	1.09E-32
10	ERV3-1	endogenous retrovirus group 3 member 1, envelope [Source:HGNC S	1.12E-10
10	SERPINB1	serpin family B member 1 [Source:HGNC Symbol;Acc:HGNC:3311]	9.14E-08
10	PIM1	Pim-1 proto-oncogene, serine/threonine kinase [Source:HGNC Symb	2.57E-16
10	HMGB1	high mobility group box 1 [Source:HGNC Symbol;Acc:HGNC:4983]	2.03E-21
10	MPIG6B	megakaryocyte and platelet inhibitory receptor G6b [Source:HGNC S	6.68E-12
10	ARPC4	actin related protein 2/3 complex subunit 4 [Source:HGNC Symbol;A	1.50E-12
10	PSTPIP2	proline-serine-threonine phosphatase interacting protein 2 [Source:H	4.41E-28
10	RGCC	regulator of cell cycle [Source:HGNC Symbol;Acc:HGNC:20369]	3.97E-15
10	INSIG1	insulin induced gene 1 [Source:HGNC Symbol;Acc:HGNC:6083]	9.80E-34
10	ERICH1	glutamate rich 1 [Source:HGNC Symbol;Acc:HGNC:27234]	2.38E-15
10	ATP2B1-AS1	ATP2B1 antisense RNA 1 [Source:HGNC Symbol;Acc:HGNC:27883]	9.12E-11
10	AP1S2	adaptor related protein complex 1 subunit sigma 2 [Source:HGNC Sy	4.61E-16
10	POU2F2	POU class 2 homeobox 2 [Source:HGNC Symbol;Acc:HGNC:9213]	2.44E-13
10	UBAC2	UBA domain containing 2 [Source:HGNC Symbol;Acc:HGNC:20486]	5.21E-11
10	CREBRF	CREB3 regulatory factor [Source:HGNC Symbol;Acc:HGNC:24050]	2.54E-10
10	CALM1	calmodulin 1 [Source:HGNC Symbol;Acc:HGNC:1442]	8.52E-20
10	LYST	lysosomal trafficking regulator [Source:HGNC Symbol;Acc:HGNC:196	2.45E-24
10	BNIP2	BCL2 interacting protein 2 [Source:HGNC Symbol;Acc:HGNC:1083]	1.03E-10
10	PPM1A	protein phosphatase, Mg2+/Mn2+ dependent 1A [Source:HGNC Sym	1.05E-15
10	SPI1	Spi-1 proto-oncogene [Source:HGNC Symbol;Acc:HGNC:11241]	2.31E-14

10	ITGA4	integrin subunit alpha 4 [Source:HGNC Symbol;Acc:HGNC:6140]	3.36E-13
10	IFI30	IFI30 lysosomal thiol reductase [Source:HGNC Symbol;Acc:HGNC:539	2.52E-07
10	STX11	syntaxin 11 [Source:HGNC Symbol;Acc:HGNC:11429]	9.16E-53
10	CAP1	cyclase associated actin cytoskeleton regulatory protein 1 [Source:H	5.05E-12
10	ADD3	adducin 3 [Source:HGNC Symbol;Acc:HGNC:245]	3.69E-23
10	TBC1D10C	TBC1 domain family member 10C [Source:HGNC Symbol;Acc:HGNC:2	3.26E-15
10	STK17A	serine/threonine kinase 17a [Source:HGNC Symbol;Acc:HGNC:11395	1.61E-11
10	OSTF1	osteoclast stimulating factor 1 [Source:HGNC Symbol;Acc:HGNC:851	1.12E-11
10	RPL37	ribosomal protein L37 [Source:HGNC Symbol;Acc:HGNC:10347]	4.16E-26
10	SAMHD1	SAM and HD domain containing deoxynucleoside triphosphate tripho	1.80E-11
10	MXD1	MAX dimerization protein 1 [Source:HGNC Symbol;Acc:HGNC:6761]	1.64E-20
10	CDC42SE2	CDC42 small effector 2 [Source:HGNC Symbol;Acc:HGNC:18547]	2.11E-19
10	TLE4	TLE family member 4, transcriptional corepressor [Source:HGNC Sym	6.69E-19
10	TRIM58	tripartite motif containing 58 [Source:HGNC Symbol;Acc:HGNC:2415	1.42E-16
10	PTPRJ	protein tyrosine phosphatase receptor type J [Source:HGNC Symbol;	1.25E-33
10	SDCBP	syndecan binding protein [Source:HGNC Symbol;Acc:HGNC:10662]	1.65E-23
10	TXNIP	thioredoxin interacting protein [Source:HGNC Symbol;Acc:HGNC:169	1.29E-08
10	SPON2	spondin 2 [Source:HGNC Symbol;Acc:HGNC:11253]	7.58E-10
10	IKZF1	IKAROS family zinc finger 1 [Source:HGNC Symbol;Acc:HGNC:13176]	2.20E-13
10	IFI16	interferon gamma inducible protein 16 [Source:HGNC Symbol;Acc:H	5.74E-13
10	SPNS1	sphingolipid transporter 1 (putative) [Source:HGNC Symbol;Acc:HGN	2.88E-09
10	AQP10	aquaporin 10 [Source:HGNC Symbol;Acc:HGNC:16029]	6.85E-13
10	TUBA1A	tubulin alpha 1a [Source:HGNC Symbol;Acc:HGNC:20766]	4.57E-16
10	AL731557.1	novel transcript	1.16E-16
10	CASP4	caspase 4 [Source:HGNC Symbol;Acc:HGNC:1505]	1.57E-12
10	SNAP23	synaptosome associated protein 23 [Source:HGNC Symbol;Acc:HGN	2.92E-12
10	ABCC3	ATP binding cassette subfamily C member 3 [Source:HGNC Symbol;A	7.66E-19
10	RPL17	ribosomal protein L17 [Source:HGNC Symbol;Acc:HGNC:10307]	1.60E-11
10	SNCA	synuclein alpha [Source:HGNC Symbol;Acc:HGNC:11138]	9.31E-18
10	TPM4	tropomyosin 4 [Source:HGNC Symbol;Acc:HGNC:12013]	1.10E-10
10	FGL2	fibrinogen like 2 [Source:HGNC Symbol;Acc:HGNC:3696]	9.96E-10
10	RPL13	ribosomal protein L13 [Source:HGNC Symbol;Acc:HGNC:10303]	3.14E-54
10	RDH11	retinol dehydrogenase 11 [Source:HGNC Symbol;Acc:HGNC:17964]	2.33E-07
10	RPL38	ribosomal protein L38 [Source:HGNC Symbol;Acc:HGNC:10349]	1.00E-15
10	MOB1A	MOB kinase activator 1A [Source:HGNC Symbol;Acc:HGNC:16015]	1.39E-10
10	NPC2	NPC intracellular cholesterol transporter 2 [Source:HGNC Symbol;Ac	5.14E-18
10	EPB41	erythrocyte membrane protein band 4.1 [Source:HGNC Symbol;Acc:	1.95E-17
10	AKAP13	A-kinase anchoring protein 13 [Source:HGNC Symbol;Acc:HGNC:371]	1.86E-13
10	OST4	oligosaccharyltransferase complex subunit 4, non-catalytic [Source:H	7.79E-12
10	EIF2AK1	eukaryotic translation initiation factor 2 alpha kinase 1 [Source:HGN	2.89E-15
10	RSRP1	arginine and serine rich protein 1 [Source:HGNC Symbol;Acc:HGNC:2	1.48E-15
10	ENKUR	enkurin, TRPC channel interacting protein [Source:HGNC Symbol;Acc	6.68E-13
10	PTGIR	prostaglandin I2 receptor [Source:HGNC Symbol;Acc:HGNC:9602]	5.28E-32
10	MYL12A	myosin light chain 12A [Source:HGNC Symbol;Acc:HGNC:16701]	1.53E-25
10	GYPC	glycophorin C (Gerbich blood group) [Source:HGNC Symbol;Acc:HGN	3.59E-12
10	RAB37	RAB37, member RAS oncogene family [Source:HGNC Symbol;Acc:HG	4.77E-24
10	ITGB3	integrin subunit beta 3 [Source:HGNC Symbol;Acc:HGNC:6156]	1.88E-19

10	HLA-DQA1	major histocompatibility complex, class II, DQ alpha 1 [Source:HGNC	3.10E-13
10	ATM	ATM serine/threonine kinase [Source:HGNC Symbol;Acc:HGNC:795]	2.74E-11
10	SCN1B	sodium voltage-gated channel beta subunit 1 [Source:HGNC Symbol;	1.30E-16
10	BTK	Bruton tyrosine kinase [Source:HGNC Symbol;Acc:HGNC:1133]	1.17E-63
10	SNN	stannin [Source:HGNC Symbol;Acc:HGNC:11149]	3.53E-26
10	C1orf162	chromosome 1 open reading frame 162 [Source:HGNC Symbol;Acc:H	1.17E-13
10	IL7R	interleukin 7 receptor [Source:HGNC Symbol;Acc:HGNC:6024]	2.24E-07
10	TRAC	T cell receptor alpha constant [Source:HGNC Symbol;Acc:HGNC:1202	2.14E-07
10	CYBB	cytochrome b-245 beta chain [Source:HGNC Symbol;Acc:HGNC:2578	8.28E-18
10	APBB1IP	amyloid beta precursor protein binding family B member 1 interactir	2.28E-21
10	NCF1	neutrophil cytosolic factor 1 [Source:HGNC Symbol;Acc:HGNC:7660]	5.73E-07
10	R3HDM4	R3H domain containing 4 [Source:HGNC Symbol;Acc:HGNC:28270]	2.09E-13
10	SSX2IP	SSX family member 2 interacting protein [Source:HGNC Symbol;Acc:I	1.69E-18
10	GP1BA	glycoprotein Ib platelet subunit alpha [Source:HGNC Symbol;Acc:HGI	7.10E-12
10	HCLS1	hematopoietic cell-specific Lyn substrate 1 [Source:HGNC Symbol;Ac	1.59E-17
10	TMEM91	transmembrane protein 91 [Source:HGNC Symbol;Acc:HGNC:32393]	1.23E-14
10	TMEM50A	transmembrane protein 50A [Source:HGNC Symbol;Acc:HGNC:3059(	9.51E-11
10	LAMTOR1	late endosomal/lysosomal adaptor, MAPK and MTOR activator 1 [So	6.40E-11
10	SKAP2	src kinase associated phosphoprotein 2 [Source:HGNC Symbol;Acc:H	1.84E-39
10	DMTN	dematin actin binding protein [Source:HGNC Symbol;Acc:HGNC:3382	5.02E-14
10	CARD19	caspase recruitment domain family member 19 [Source:HGNC Symb	4.87E-10
10	SEC14L1	SEC14 like lipid binding 1 [Source:HGNC Symbol;Acc:HGNC:10698]	2.70E-17
10	MEF2C	myocyte enhancer factor 2C [Source:HGNC Symbol;Acc:HGNC:6996]	2.44E-44
10	RPL13A	ribosomal protein L13a [Source:HGNC Symbol;Acc:HGNC:10304]	2.86E-30
10	GIMAP7	GTPase, IMAP family member 7 [Source:HGNC Symbol;Acc:HGNC:22	1.44E-12
10	STK24	serine/threonine kinase 24 [Source:HGNC Symbol;Acc:HGNC:11403]	1.88E-19
10	CLEC2B	C-type lectin domain family 2 member B [Source:HGNC Symbol;Acc:I	2.34E-13
10	GIMAP4	GTPase, IMAP family member 4 [Source:HGNC Symbol;Acc:HGNC:21	1.02E-13
10	AP001189.1	novel transcript	5.89E-10
10	VSIR	V-set immunoregulatory receptor [Source:HGNC Symbol;Acc:HGNC:3	2.57E-26
10	CD53	CD53 molecule [Source:HGNC Symbol;Acc:HGNC:1686]	4.49E-19
10	GUCY1B1	guanylate cyclase 1 soluble subunit beta 1 [Source:HGNC Symbol;Acc	1.28E-21
10	AP003068.2	novel transcript, antisense to CAPN1	1.87E-11
10	C12orf76	chromosome 12 open reading frame 76 [Source:HGNC Symbol;Acc:H	4.59E-16
10	RPL21	ribosomal protein L21 [Source:HGNC Symbol;Acc:HGNC:10313]	3.29E-24
10	WIPF1	WAS/WASL interacting protein family member 1 [Source:HGNC Symb	2.89E-33
10	ILK	integrin linked kinase [Source:HGNC Symbol;Acc:HGNC:6040]	5.24E-07
10	CLU	clusterin [Source:HGNC Symbol;Acc:HGNC:2095]	6.22E-07
10	TPT1	tumor protein, translationally-controlled 1 [Source:HGNC Symbol;Ac	1.72E-40
10	FAM107B	family with sequence similarity 107 member B [Source:HGNC Symbo	1.76E-24
10	RPL11	ribosomal protein L11 [Source:HGNC Symbol;Acc:HGNC:10301]	3.08E-62
10	TNFSF4	TNF superfamily member 4 [Source:HGNC Symbol;Acc:HGNC:11934]	7.28E-30
10	CAPZA2	capping actin protein of muscle Z-line subunit alpha 2 [Source:HGNC	1.67E-08
10	RHOF	ras homolog family member F, filopodia associated [Source:HGNC Sy	5.29E-27
10	STON2	stonin 2 [Source:HGNC Symbol;Acc:HGNC:30652]	6.95E-14
10	RNF11	ring finger protein 11 [Source:HGNC Symbol;Acc:HGNC:10056]	8.91E-11
10	YPEL5	yippee like 5 [Source:HGNC Symbol;Acc:HGNC:18329]	2.03E-10

10	RNF213	ring finger protein 213 [Source:HGNC Symbol;Acc:HGNC:14539]	5.11E-10
10	CYTOR	cytoskeleton regulator RNA [Source:HGNC Symbol;Acc:HGNC:28717]	7.08E-11
10	PPP2R5C	protein phosphatase 2 regulatory subunit B'gamma [Source:HGNC Sy	7.28E-10
10	MTPN	myotrophin [Source:HGNC Symbol;Acc:HGNC:15667]	1.54E-12
10	CTSA	cathepsin A [Source:HGNC Symbol;Acc:HGNC:9251]	1.01E-07
10	MLH3	mutL homolog 3 [Source:HGNC Symbol;Acc:HGNC:7128]	2.87E-13
10	GZMM	granzyme M [Source:HGNC Symbol;Acc:HGNC:4712]	2.90E-07
10	TACC3	transforming acidic coiled-coil containing protein 3 [Source:HGNC Sy	6.57E-26
10	WBP2	WW domain binding protein 2 [Source:HGNC Symbol;Acc:HGNC:127	1.12E-10
10	RBBP6	RB binding protein 6, ubiquitin ligase [Source:HGNC Symbol;Acc:HGN	2.00E-08
10	ARHGAP18	Rho GTPase activating protein 18 [Source:HGNC Symbol;Acc:HGNC:2	7.56E-17
10	HOPX	HOP homeobox [Source:HGNC Symbol;Acc:HGNC:24961]	2.75E-08
10	UBXN11	UBX domain protein 11 [Source:HGNC Symbol;Acc:HGNC:30600]	7.61E-26
10	STXBP2	syntaxin binding protein 2 [Source:HGNC Symbol;Acc:HGNC:11445]	3.86E-13
10	MYO1F	myosin IF [Source:HGNC Symbol;Acc:HGNC:7600]	2.13E-15
10	TRAF3IP3	TRAF3 interacting protein 3 [Source:HGNC Symbol;Acc:HGNC:30766]	2.08E-19
10	TAL1	TAL bHLH transcription factor 1, erythroid differentiation factor [Sou	1.04E-19
10	ACTN1	actinin alpha 1 [Source:HGNC Symbol;Acc:HGNC:163]	1.30E-07
10	CD36	CD36 molecule [Source:HGNC Symbol;Acc:HGNC:1663]	4.01E-48
10	STK4	serine/threonine kinase 4 [Source:HGNC Symbol;Acc:HGNC:11408]	1.01E-23
10	RPS20	ribosomal protein S20 [Source:HGNC Symbol;Acc:HGNC:10405]	2.48E-16
10	YWHAZ	tyrosine 3-monooxygenase/tryptophan 5-monooxygenase activation	2.67E-28
10	FYN	FYN proto-oncogene, Src family tyrosine kinase [Source:HGNC Symbo	3.44E-29
10	RAP2B	RAP2B, member of RAS oncogene family [Source:HGNC Symbol;Acc:	7.87E-15
10	LYN	LYN proto-oncogene, Src family tyrosine kinase [Source:HGNC Symbo	2.09E-41
10	VASP	vasodilator stimulated phosphoprotein [Source:HGNC Symbol;Acc:H	6.24E-17
10	MFSD1	major facilitator superfamily domain containing 1 [Source:HGNC Sym	3.00E-10
10	GNAI2	G protein subunit alpha i2 [Source:HGNC Symbol;Acc:HGNC:4385]	8.55E-20
10	USP15	ubiquitin specific peptidase 15 [Source:HGNC Symbol;Acc:HGNC:126	1.95E-14
10	CXCL5	C-X-C motif chemokine ligand 5 [Source:HGNC Symbol;Acc:HGNC:10	5.05E-19
10	NFKBIA	NFKB inhibitor alpha [Source:HGNC Symbol;Acc:HGNC:7797]	2.65E-13
10	PTGS1	prostaglandin-endoperoxide synthase 1 [Source:HGNC Symbol;Acc:H	6.62E-26
10	RPL36A	ribosomal protein L36a [Source:HGNC Symbol;Acc:HGNC:10359]	1.99E-15
10	CNST	consortin, connexin sorting protein [Source:HGNC Symbol;Acc:HGNC	7.08E-13
10	CTSC	cathepsin C [Source:HGNC Symbol;Acc:HGNC:2528]	1.63E-22
10	AC090409.1	novel transcript	4.22E-12
10	ARHGAP6	Rho GTPase activating protein 6 [Source:HGNC Symbol;Acc:HGNC:67	1.34E-16
10	BTG1	BTG anti-proliferation factor 1 [Source:HGNC Symbol;Acc:HGNC:113	2.75E-14
10	CLDN5	claudin 5 [Source:HGNC Symbol;Acc:HGNC:2047]	6.16E-13
10	GSTO1	glutathione S-transferase omega 1 [Source:HGNC Symbol;Acc:HGNC:	1.62E-15
10	DOK2	docking protein 2 [Source:HGNC Symbol;Acc:HGNC:2991]	9.01E-31
10	TMEM158	transmembrane protein 158 [Source:HGNC Symbol;Acc:HGNC:30293	8.31E-12
10	JAK1	Janus kinase 1 [Source:HGNC Symbol;Acc:HGNC:6190]	3.93E-17
10	CELF2	CUGBP Elav-like family member 2 [Source:HGNC Symbol;Acc:HGNC:2	9.86E-33
10	CMIP	c-Maf inducing protein [Source:HGNC Symbol;Acc:HGNC:24319]	1.41E-26
10	LCN2	lipocalin 2 [Source:HGNC Symbol;Acc:HGNC:6526]	3.80E-07
10	SMIM3	small integral membrane protein 3 [Source:HGNC Symbol;Acc:HGNC	3.11E-10

10	RPS29	ribosomal protein S29 [Source:HGNC Symbol;Acc:HGNC:10419]	3.11E-15
10	GMPR	guanosine monophosphate reductase [Source:HGNC Symbol;Acc:HG	1.95E-11
10	MYLK	myosin light chain kinase [Source:HGNC Symbol;Acc:HGNC:7590]	4.39E-21
10	TBPL1	TATA-box binding protein like 1 [Source:HGNC Symbol;Acc:HGNC:11	2.88E-17
10	ZYX	zyxin [Source:HGNC Symbol;Acc:HGNC:13200]	2.09E-19
10	IL2RG	interleukin 2 receptor subunit gamma [Source:HGNC Symbol;Acc:HG	5.62E-15
10	TLK1	tousled like kinase 1 [Source:HGNC Symbol;Acc:HGNC:11841]	9.85E-10
10	PRKCB	protein kinase C beta [Source:HGNC Symbol;Acc:HGNC:9395]	2.37E-32
10	PPP1R18	protein phosphatase 1 regulatory subunit 18 [Source:HGNC Symbol;	1.84E-32
10	HEMGN	hemogen [Source:HGNC Symbol;Acc:HGNC:17509]	1.28E-15
10	RPL34	ribosomal protein L34 [Source:HGNC Symbol;Acc:HGNC:10340]	8.55E-65
10	ESAM	endothelial cell adhesion molecule [Source:HGNC Symbol;Acc:HGNC	2.75E-12
10	FRMD3	FERM domain containing 3 [Source:HGNC Symbol;Acc:HGNC:24125]	7.33E-16
10	CLEC1B	C-type lectin domain family 1 member B [Source:HGNC Symbol;Acc:	6.64E-10
10	CD7	CD7 molecule [Source:HGNC Symbol;Acc:HGNC:1695]	1.12E-09
10	RBX1	ring-box 1 [Source:HGNC Symbol;Acc:HGNC:9928]	1.72E-09
10	FRMD4B	FERM domain containing 4B [Source:HGNC Symbol;Acc:HGNC:24886	4.88E-35
10	CALM3	calmodulin 3 [Source:HGNC Symbol;Acc:HGNC:1449]	4.05E-15
10	RSU1	Ras suppressor protein 1 [Source:HGNC Symbol;Acc:HGNC:10464]	1.98E-10
10	CD68	CD68 molecule [Source:HGNC Symbol;Acc:HGNC:1693]	3.96E-58
10	PIP4K2A	phosphatidylinositol-5-phosphate 4-kinase type 2 alpha [Source:HGN	1.17E-33
10	PLAC8	placenta associated 8 [Source:HGNC Symbol;Acc:HGNC:19254]	7.08E-14
10	EVL	Enah/Vasp-like [Source:HGNC Symbol;Acc:HGNC:20234]	6.38E-23
10	CD3E	CD3e molecule [Source:HGNC Symbol;Acc:HGNC:1674]	4.82E-09
10	TRBC1	T cell receptor beta constant 1 [Source:HGNC Symbol;Acc:HGNC:121	2.82E-07
10	ACTB	actin beta [Source:HGNC Symbol;Acc:HGNC:132]	2.55E-45
10	SH3BGRL2	SH3 domain binding glutamate rich protein like 2 [Source:HGNC Sym	5.46E-16
10	GFI1B	growth factor independent 1B transcriptional repressor [Source:HGN	2.34E-22
10	CD48	CD48 molecule [Source:HGNC Symbol;Acc:HGNC:1683]	1.35E-36
10	ITM2B	integral membrane protein 2B [Source:HGNC Symbol;Acc:HGNC:617	1.95E-42
10	AC000093.1	novel transcript	5.28E-17
10	PDGFA	platelet derived growth factor subunit A [Source:HGNC Symbol;Acc:	1.96E-13
10	NFE2	nuclear factor, erythroid 2 [Source:HGNC Symbol;Acc:HGNC:7780]	1.71E-33
10	STK17B	serine/threonine kinase 17b [Source:HGNC Symbol;Acc:HGNC:11396	2.95E-25
10	SELL	selectin L [Source:HGNC Symbol;Acc:HGNC:10720]	2.70E-16
10	AMD1	adenosylmethionine decarboxylase 1 [Source:HGNC Symbol;Acc:HG	5.69E-13
10	CD247	CD247 molecule [Source:HGNC Symbol;Acc:HGNC:1677]	4.84E-10
10	TMEM140	transmembrane protein 140 [Source:HGNC Symbol;Acc:HGNC:2187	1.73E-18
10	ICAM2	intercellular adhesion molecule 2 [Source:HGNC Symbol;Acc:HGNC:5	9.73E-23
10	PTPN18	protein tyrosine phosphatase non-receptor type 18 [Source:HGNC S	4.87E-12
10	EVI2B	ecotropic viral integration site 2B [Source:HGNC Symbol;Acc:HGNC:3	9.87E-16
10	YWHAH	tyrosine 3-monooxygenase/tryptophan 5-monooxygenase activatio	7.01E-12
10	ETS1	ETS proto-oncogene 1, transcription factor [Source:HGNC Symbol;Ac	7.55E-15
10	MBNL1	muscleblind like splicing regulator 1 [Source:HGNC Symbol;Acc:HGN	7.05E-30
10	F13A1	coagulation factor XIII A chain [Source:HGNC Symbol;Acc:HGNC:353	2.38E-23
10	CYBA	cytochrome b-245 alpha chain [Source:HGNC Symbol;Acc:HGNC:257	1.60E-20
10	B2M	beta-2-microglobulin [Source:HGNC Symbol;Acc:HGNC:914]	4.16E-158

10	MAP3K7CL	MAP3K7 C-terminal like [Source:HGNC Symbol;Acc:HGNC:16457]	1.94E-10
10	RASGRP2	RAS guanyl releasing protein 2 [Source:HGNC Symbol;Acc:HGNC:987	4.31E-24
10	SPARC	secreted protein acidic and cysteine rich [Source:HGNC Symbol;Acc:H	1.06E-11
10	MBP	myelin basic protein [Source:HGNC Symbol;Acc:HGNC:6925]	1.87E-30
10	PF4V1	platelet factor 4 variant 1 [Source:HGNC Symbol;Acc:HGNC:8862]	1.40E-18
10	CD226	CD226 molecule [Source:HGNC Symbol;Acc:HGNC:16961]	2.27E-28
10	SLC2A3	solute carrier family 2 member 3 [Source:HGNC Symbol;Acc:HGNC:1	4.40E-28
10	AC147651.1	novel transcript	1.29E-10
10	KLRD1	killer cell lectin like receptor D1 [Source:HGNC Symbol;Acc:HGNC:63	3.09E-09
10	DAPP1	dual adaptor of phosphotyrosine and 3-phosphoinositides 1 [Source:	1.68E-16
10	ARPC5	actin related protein 2/3 complex subunit 5 [Source:HGNC Symbol;A	4.09E-38
10	CXCR4	C-X-C motif chemokine receptor 4 [Source:HGNC Symbol;Acc:HGNC:	3.84E-17
10	CCL4	C-C motif chemokine ligand 4 [Source:HGNC Symbol;Acc:HGNC:1063	2.17E-10
10	IFITM1	interferon induced transmembrane protein 1 [Source:HGNC Symbol;	1.80E-13
10	SMOX	spermine oxidase [Source:HGNC Symbol;Acc:HGNC:15862]	9.15E-21
10	ZEB2	zinc finger E-box binding homeobox 2 [Source:HGNC Symbol;Acc:HG	2.15E-39
10	VIM-AS1	VIM antisense RNA 1 [Source:HGNC Symbol;Acc:HGNC:44879]	2.68E-25
10	OAZ1	ornithine decarboxylase antizyme 1 [Source:HGNC Symbol;Acc:HGN	8.08E-37
10	CCND3	cyclin D3 [Source:HGNC Symbol;Acc:HGNC:1585]	1.47E-33
10	ARPC1B	actin related protein 2/3 complex subunit 1B [Source:HGNC Symbol;	1.53E-47
10	IL32	interleukin 32 [Source:HGNC Symbol;Acc:HGNC:16830]	2.00E-11
10	RBM38	RNA binding motif protein 38 [Source:HGNC Symbol;Acc:HGNC:1581	4.51E-29
10	GADD45A	growth arrest and DNA damage inducible alpha [Source:HGNC Symb	1.17E-25
10	GMFG	glia maturation factor gamma [Source:HGNC Symbol;Acc:HGNC:437	3.61E-57
10	ITGA2B	integrin subunit alpha 2b [Source:HGNC Symbol;Acc:HGNC:6138]	3.11E-13
10	TRBC2	T cell receptor beta constant 2 [Source:HGNC Symbol;Acc:HGNC:121	1.98E-16
10	FCGR3A	Fc fragment of IgG receptor IIIa [Source:HGNC Symbol;Acc:HGNC:36	2.63E-12
10	MIR4435-2HG	MIR4435-2 host gene [Source:HGNC Symbol;Acc:HGNC:35163]	3.73E-12
10	LGALSL	galectin like [Source:HGNC Symbol;Acc:HGNC:25012]	7.64E-13
10	NEXN	nexilin F-actin binding protein [Source:HGNC Symbol;Acc:HGNC:295	1.94E-29
10	VCL	vinculin [Source:HGNC Symbol;Acc:HGNC:12665]	3.50E-16
10	LST1	leukocyte specific transcript 1 [Source:HGNC Symbol;Acc:HGNC:141	1.61E-12
10	AKIRIN2	akirin 2 [Source:HGNC Symbol;Acc:HGNC:21407]	4.87E-36
10	ELF1	E74 like ETS transcription factor 1 [Source:HGNC Symbol;Acc:HGNC:	4.30E-27
10	LIMD2	LIM domain containing 2 [Source:HGNC Symbol;Acc:HGNC:28142]	4.53E-45
10	GPSM3	G protein signaling modulator 3 [Source:HGNC Symbol;Acc:HGNC:13	6.72E-50
10	MSN	moesin [Source:HGNC Symbol;Acc:HGNC:7373]	2.96E-37
10	RAC2	Rac family small GTPase 2 [Source:HGNC Symbol;Acc:HGNC:9802]	5.39E-51
10	C12orf75	chromosome 12 open reading frame 75 [Source:HGNC Symbol;Acc:H	3.26E-20
10	GZMB	granzyme B [Source:HGNC Symbol;Acc:HGNC:4709]	3.69E-07
10	LAT	linker for activation of T cells [Source:HGNC Symbol;Acc:HGNC:1887	3.76E-27
10	NCK2	NCK adaptor protein 2 [Source:HGNC Symbol;Acc:HGNC:7665]	3.48E-15
10	SOD2	superoxide dismutase 2 [Source:HGNC Symbol;Acc:HGNC:11180]	8.88E-24
10	LAPTM5	lysosomal protein transmembrane 5 [Source:HGNC Symbol;Acc:HGN	7.73E-50
10	LSP1	lymphocyte specific protein 1 [Source:HGNC Symbol;Acc:HGNC:6707	1.33E-32
10	FLNA	filamin A [Source:HGNC Symbol;Acc:HGNC:3754]	6.70E-33
10	TPST2	tyrosylprotein sulfotransferase 2 [Source:HGNC Symbol;Acc:HGNC:1	2.26E-38

10	ARL4C	ADP ribosylation factor like GTPase 4C [Source:HGNC Symbol;Acc:HG	1.14E-14
10	TREML1	triggering receptor expressed on myeloid cells like 1 [Source:HGNC S	4.01E-09
10	LTB	lymphotoxin beta [Source:HGNC Symbol;Acc:HGNC:6711]	9.69E-20
10	CD69	CD69 molecule [Source:HGNC Symbol;Acc:HGNC:1694]	2.18E-13
10	LCP1	lymphocyte cytosolic protein 1 [Source:HGNC Symbol;Acc:HGNC:652	2.56E-26
10	FYB1	FYN binding protein 1 [Source:HGNC Symbol;Acc:HGNC:4036]	5.53E-51
10	CD52	CD52 molecule [Source:HGNC Symbol;Acc:HGNC:1804]	1.30E-47
10	ITGB2	integrin subunit beta 2 [Source:HGNC Symbol;Acc:HGNC:6155]	4.12E-29
10	CMTM5	CKLF like MARVEL transmembrane domain containing 5 [Source:HGN	2.12E-11
10	PPBP	pro-platelet basic protein [Source:HGNC Symbol;Acc:HGNC:9240]	2.92E-12
10	CD99	CD99 molecule (Xg blood group) [Source:HGNC Symbol;Acc:HGNC:70	4.17E-24
10	RGS10	regulator of G protein signaling 10 [Source:HGNC Symbol;Acc:HGNC:	4.39E-29
10	TMSB4X	thymosin beta 4 X-linked [Source:HGNC Symbol;Acc:HGNC:11881]	1.02E-117
10	GRAP2	GRB2 related adaptor protein 2 [Source:HGNC Symbol;Acc:HGNC:45	1.82E-21
10	CD37	CD37 molecule [Source:HGNC Symbol;Acc:HGNC:1666]	6.41E-53
10	FGFBP2	fibroblast growth factor binding protein 2 [Source:HGNC Symbol;Acc	2.11E-09
10	NAP1L1	nucleosome assembly protein 1 like 1 [Source:HGNC Symbol;Acc:HG	1.17E-38
10	HLA-E	major histocompatibility complex, class I, E [Source:HGNC Symbol;Ac	2.20E-89
10	TSC22D1	TSC22 domain family member 1 [Source:HGNC Symbol;Acc:HGNC:16	2.35E-11
10	FCER1G	Fc fragment of IgE receptor Ig [Source:HGNC Symbol;Acc:HGNC:3611	6.42E-38
10	FCN1	ficolin 1 [Source:HGNC Symbol;Acc:HGNC:3623]	8.48E-16
10	PRF1	perforin 1 [Source:HGNC Symbol;Acc:HGNC:9360]	1.45E-10
10	COTL1	coactosin like F-actin binding protein 1 [Source:HGNC Symbol;Acc:HG	3.96E-46
10	MNDA	myeloid cell nuclear differentiation antigen [Source:HGNC Symbol;Ac	2.56E-09
10	CORO1A	coronin 1A [Source:HGNC Symbol;Acc:HGNC:2252]	1.06E-63
10	TLN1	talin 1 [Source:HGNC Symbol;Acc:HGNC:11845]	7.61E-40
10	EMP3	epithelial membrane protein 3 [Source:HGNC Symbol;Acc:HGNC:333	2.28E-69
10	DAB2	DAB adaptor protein 2 [Source:HGNC Symbol;Acc:HGNC:2662]	7.56E-27
10	CDKN2D	cyclin dependent kinase inhibitor 2D [Source:HGNC Symbol;Acc:HGN	1.00E-35
10	PNRC1	proline rich nuclear receptor coactivator 1 [Source:HGNC Symbol;Ac	1.28E-58
10	FERMT3	fermitin family member 3 [Source:HGNC Symbol;Acc:HGNC:23151]	1.05E-37
10	MMD	monocyte to macrophage differentiation associated [Source:HGNC S	3.12E-19
10	HCST	hematopoietic cell signal transducer [Source:HGNC Symbol;Acc:HGN	8.16E-46
10	PDLIM1	PDZ and LIM domain 1 [Source:HGNC Symbol;Acc:HGNC:2067]	7.91E-17
10	TSPAN33	tetraspanin 33 [Source:HGNC Symbol;Acc:HGNC:28743]	1.01E-16
10	RAB32	RAB32, member RAS oncogene family [Source:HGNC Symbol;Acc:HG	2.75E-35
10	LYL1	LYL1 basic helix-loop-helix family member [Source:HGNC Symbol;Acc	6.29E-51
10	C2orf88	chromosome 2 open reading frame 88 [Source:HGNC Symbol;Acc:HG	5.69E-12
10	TGFB1	transforming growth factor beta 1 [Source:HGNC Symbol;Acc:HGNC:	2.44E-49
10	AIF1	allograft inflammatory factor 1 [Source:HGNC Symbol;Acc:HGNC:352	1.08E-10
10	FTL	ferritin light chain [Source:HGNC Symbol;Acc:HGNC:3999]	2.89E-64
10	S100A4	S100 calcium binding protein A4 [Source:HGNC Symbol;Acc:HGNC:10	7.74E-22
10	GZMH	granzyme H [Source:HGNC Symbol;Acc:HGNC:4710]	3.07E-12
10	NT5C3A	5'-nucleotidase, cytosolic IIIA [Source:HGNC Symbol;Acc:HGNC:1782	7.48E-19
10	SH3BGRL3	SH3 domain binding glutamate rich protein like 3 [Source:HGNC Sym	2.12E-84
10	KIF2A	kinesin family member 2A [Source:HGNC Symbol;Acc:HGNC:6318]	5.81E-24
10	MPP1	membrane palmitoylated protein 1 [Source:HGNC Symbol;Acc:HGNC	1.56E-33

10	HLA-DRB1	major histocompatibility complex, class II, DR beta 1 [Source:HGNC S	8.44E-20
10	PTPRC	protein tyrosine phosphatase receptor type C [Source:HGNC Symbol	1.44E-66
10	RIPOR2	RHO family interacting cell polarization regulator 2 [Source:HGNC Sy	5.09E-52
10	CST7	cystatin F [Source:HGNC Symbol;Acc:HGNC:2479]	6.18E-10
10	GZMA	granzyme A [Source:HGNC Symbol;Acc:HGNC:4708]	3.72E-08
10	PLEKHO1	pleckstrin homology domain containing O1 [Source:HGNC Symbol;Ac	2.80E-45
10	TMEM40	transmembrane protein 40 [Source:HGNC Symbol;Acc:HGNC:25620]	1.61E-18
10	BIN2	bridging integrator 2 [Source:HGNC Symbol;Acc:HGNC:1053]	5.81E-58
10	VIM	vimentin [Source:HGNC Symbol;Acc:HGNC:12692]	7.98E-56
10	PTCRA	pre T cell antigen receptor alpha [Source:HGNC Symbol;Acc:HGNC:2	9.87E-15
10	CTSW	cathepsin W [Source:HGNC Symbol;Acc:HGNC:2546]	1.64E-14
10	GP9	glycoprotein IX platelet [Source:HGNC Symbol;Acc:HGNC:4444]	6.96E-28
10	MAX	MYC associated factor X [Source:HGNC Symbol;Acc:HGNC:6913]	9.09E-45
10	GNG11	G protein subunit gamma 11 [Source:HGNC Symbol;Acc:HGNC:4403]	5.60E-21
10	PRKAR2B	protein kinase cAMP-dependent type II regulatory subunit beta [Sou	7.07E-14
10	PF4	platelet factor 4 [Source:HGNC Symbol;Acc:HGNC:8861]	8.78E-17
10	HLA-DPB1	major histocompatibility complex, class II, DP beta 1 [Source:HGNC S	5.28E-19
10	HLA-DPA1	major histocompatibility complex, class II, DP alpha 1 [Source:HGNC	2.24E-18
10	LIMS1	LIM zinc finger domain containing 1 [Source:HGNC Symbol;Acc:HGN	1.04E-26
10	TUBA4A	tubulin alpha 4a [Source:HGNC Symbol;Acc:HGNC:12407]	9.63E-19
10	ODC1	ornithine decarboxylase 1 [Source:HGNC Symbol;Acc:HGNC:8109]	9.03E-34
10	PLEK	pleckstrin [Source:HGNC Symbol;Acc:HGNC:9070]	1.06E-42
10	NCOA4	nuclear receptor coactivator 4 [Source:HGNC Symbol;Acc:HGNC:767	1.68E-21
10	MTURN	maturin, neural progenitor differentiation regulator homolog [Sourc	5.86E-22
10	NRGN	neurogranin [Source:HGNC Symbol;Acc:HGNC:8000]	8.85E-30
10	ACRBP	acrosin binding protein [Source:HGNC Symbol;Acc:HGNC:17195]	5.41E-14
10	RUFY1	RUN and FYVE domain containing 1 [Source:HGNC Symbol;Acc:HGN	1.64E-28
10	IFITM2	interferon induced transmembrane protein 2 [Source:HGNC Symbol;	3.12E-33
10	RAP1B	RAP1B, member of RAS oncogene family [Source:HGNC Symbol;Acc:	1.05E-40
10	SRGN	serglycin [Source:HGNC Symbol;Acc:HGNC:9361]	7.57E-64
10	S100A9	S100 calcium binding protein A9 [Source:HGNC Symbol;Acc:HGNC:1	6.98E-12
10	CTSS	cathepsin S [Source:HGNC Symbol;Acc:HGNC:2545]	5.28E-24
10	TUBB1	tubulin beta 1 class VI [Source:HGNC Symbol;Acc:HGNC:16257]	2.42E-31
10	CAVIN2	caveolae associated protein 2 [Source:HGNC Symbol;Acc:HGNC:106	2.76E-17
10	RGS18	regulator of G protein signaling 18 [Source:HGNC Symbol;Acc:HGNC:	9.11E-42
10	TYROBP	transmembrane immune signaling adaptor TYROBP [Source:HGNC Sy	8.71E-22
10	CCL5	C-C motif chemokine ligand 5 [Source:HGNC Symbol;Acc:HGNC:1063	3.77E-26
10	HLA-DRA	major histocompatibility complex, class II, DR alpha [Source:HGNC Sy	9.55E-19
10	CD74	CD74 molecule [Source:HGNC Symbol;Acc:HGNC:1697]	1.23E-35
10	NKG7	natural killer cell granule protein 7 [Source:HGNC Symbol;Acc:HGNC:	2.99E-11
10	GNLY	granulysin [Source:HGNC Symbol;Acc:HGNC:4414]	1.98E-08
10	LYZ	lysozyme [Source:HGNC Symbol;Acc:HGNC:6740]	1.09E-13

avg_logFC	pct.1	pct.2	p_val_adj	mean in cluster	mean out of cluster	DiffMean	dDR
5.9634	0.761	0.030	2.58E-259	5.243	0.054	5.190	0.731
4.2435	0.761	0.005	9.42E-226	3.458	0.012	3.446	0.756
3.5015	0.786	0.032	4.43E-220	2.914	0.042	2.872	0.754
3.2811	0.741	0.004	1.98E-215	2.577	0.008	2.569	0.737
2.8861	0.746	0.019	2.75E-189	2.291	0.031	2.260	0.727
2.8452	0.701	0.001	3.73E-194	2.111	0.003	2.107	0.700
2.8430	0.701	0.030	3.01E-172	2.147	0.035	2.113	0.671
2.8362	0.697	0.004	1.03E-185	2.084	0.007	2.077	0.693
2.7803	0.701	0.036	6.75E-152	2.044	0.038	2.006	0.665
2.7724	0.682	0.005	8.01E-181	1.998	0.006	1.992	0.677
2.5518	0.677	0.002	1.14E-176	1.873	0.005	1.869	0.675
2.3607	0.677	0.011	2.45E-147	1.810	0.019	1.791	0.666
2.2728	0.587	0.005	1.88E-129	1.488	0.005	1.482	0.582
2.2487	0.801	0.455	6.37E-129	2.376	0.643	1.734	0.346
2.2400	0.542	0.001	9.91E-129	1.337	0.002	1.334	0.541
2.1036	0.731	0.183	7.19E-108	1.986	0.246	1.739	0.548
2.0456	0.617	0.001	2.68E-159	1.441	0.002	1.439	0.616
1.9269	0.617	0.054	7.10E-141	1.449	0.053	1.396	0.563
1.9009	0.522	0.001	9.72E-122	1.156	0.002	1.154	0.521
1.8975	0.572	0.009	9.86E-114	1.274	0.015	1.259	0.563
1.8673	0.612	0.021	1.77E-111	1.381	0.025	1.357	0.591
1.8490	0.517	0.002	3.42E-114	1.156	0.003	1.153	0.515
1.8137	0.612	0.028	8.55E-112	1.316	0.029	1.287	0.584
1.7771	0.572	0.018	4.84E-118	1.250	0.016	1.234	0.554
1.7659	0.562	0.014	9.20E-124	1.222	0.013	1.209	0.548
1.7410	0.363	0.001	8.59E-71	0.787	0.001	0.786	0.362
1.7392	0.577	0.009	5.14E-123	1.226	0.009	1.217	0.568
1.7372	0.532	0.056	3.79E-66	1.246	0.077	1.169	0.476
1.7197	0.617	0.065	1.94E-129	1.304	0.065	1.239	0.552
1.7186	0.751	0.305	1.25E-120	1.774	0.367	1.407	0.446
1.7085	0.557	0.002	2.92E-126	1.155	0.004	1.151	0.555
1.7008	0.761	0.311	3.65E-89	1.966	0.455	1.511	0.450
1.6658	0.502	0.016	5.78E-99	1.082	0.015	1.067	0.486
1.6593	0.512	0.001	7.93E-114	1.048	0.002	1.046	0.511
1.6414	0.537	0.001	8.68E-129	1.107	0.001	1.106	0.536
1.6258	0.960	0.669	1.28E-102	3.646	1.611	2.035	0.291
1.6164	0.522	0.001	6.15E-119	1.059	0.002	1.057	0.521
1.6072	0.527	0.001	3.89E-124	1.070	0.002	1.068	0.526
1.5990	0.900	0.483	1.66E-91	2.880	0.896	1.984	0.417
1.5921	0.577	0.008	1.02E-122	1.128	0.010	1.118	0.569
1.5820	0.537	0.001	8.37E-121	1.069	0.002	1.067	0.536
1.5710	0.507	0.024	4.60E-82	1.039	0.031	1.008	0.483
1.5633	0.468	0.019	1.45E-74	0.973	0.030	0.943	0.449
1.5165	0.393	0.022	4.17E-52	0.829	0.023	0.806	0.371
1.5155	0.557	0.123	4.43E-86	1.107	0.121	0.986	0.434

1.4708	0.473	0.006	2.40E-78	0.952	0.010	0.943	0.467
1.4455	0.473	0.047	1.18E-73	0.969	0.046	0.923	0.426
1.4310	0.438	0.005	7.14E-72	0.854	0.008	0.846	0.433
1.4103	0.448	0.001	9.60E-95	0.832	0.001	0.831	0.447
1.3989	0.458	0.007	1.06E-82	0.868	0.008	0.860	0.451
1.3977	0.438	0.009	1.18E-70	0.820	0.009	0.811	0.429
1.3520	0.393	0.029	3.22E-34	0.809	0.054	0.755	0.364
1.3493	0.881	0.575	1.99E-52	2.696	1.122	1.574	0.306
1.3492	0.537	0.186	2.21E-71	1.113	0.205	0.908	0.351
1.3444	0.836	0.474	1.31E-52	2.140	0.757	1.383	0.362
1.3353	0.572	0.164	1.49E-87	1.110	0.162	0.948	0.408
1.3283	0.522	0.142	4.53E-69	1.027	0.139	0.888	0.380
1.3279	0.731	0.380	7.79E-68	1.558	0.449	1.109	0.351
1.3238	0.458	0.001	2.86E-92	0.829	0.002	0.827	0.457
1.3111	0.418	0.001	2.21E-81	0.749	0.001	0.748	0.417
1.3067	0.438	0.026	6.61E-44	0.855	0.034	0.821	0.412
1.3026	0.433	0.031	2.70E-77	0.775	0.027	0.748	0.402
1.3017	0.368	0.001	2.16E-73	0.699	0.001	0.698	0.367
1.2953	0.597	0.273	6.70E-44	1.208	0.311	0.897	0.324
1.2882	0.637	0.189	5.76E-47	1.256	0.211	1.046	0.448
1.2865	0.433	0.001	1.44E-81	0.801	0.002	0.799	0.432
1.2822	0.433	0.001	1.54E-84	0.774	0.002	0.772	0.432
1.2787	0.383	0.041	2.37E-50	0.679	0.039	0.640	0.342
1.2723	0.433	0.002	4.20E-83	0.778	0.003	0.776	0.431
1.2571	0.562	0.162	1.07E-66	1.062	0.171	0.891	0.400
1.2457	0.711	0.354	2.42E-94	1.434	0.403	1.031	0.357
1.2433	0.706	0.396	1.45E-85	1.491	0.471	1.020	0.310
1.2392	0.428	0.001	2.88E-82	0.748	0.001	0.746	0.427
1.2371	0.423	0.016	2.64E-69	0.760	0.015	0.745	0.407
1.2151	0.711	0.434	5.34E-69	1.514	0.532	0.982	0.277
1.2137	0.567	0.212	1.37E-47	1.104	0.225	0.879	0.355
1.2126	0.353	0.002	2.13E-59	0.639	0.003	0.635	0.351
1.2067	0.507	0.076	6.62E-54	1.033	0.127	0.906	0.431
1.2002	0.438	0.006	4.61E-75	0.740	0.006	0.734	0.432
1.1847	0.438	0.028	1.13E-77	0.756	0.026	0.730	0.410
1.1816	0.403	0.006	2.36E-66	0.709	0.007	0.701	0.397
1.1687	0.746	0.447	4.57E-58	1.564	0.563	1.000	0.299
1.1650	0.522	0.098	8.21E-67	0.873	0.089	0.784	0.424
1.1533	0.478	0.041	9.71E-46	0.848	0.056	0.792	0.437
1.1529	0.801	0.493	4.83E-97	1.929	0.718	1.211	0.308
1.1526	0.204	0.002	4.09E-28	0.409	0.003	0.406	0.202
1.1523	0.806	0.489	1.17E-69	2.059	0.832	1.227	0.317
1.1421	0.582	0.246	7.25E-30	1.129	0.296	0.832	0.336
1.1404	0.423	0.045	2.98E-33	0.842	0.079	0.763	0.378
1.1369	0.413	0.001	2.62E-78	0.685	0.001	0.683	0.412
1.1369	0.731	0.514	3.69E-35	1.606	0.741	0.864	0.217
1.1363	0.517	0.133	2.24E-56	0.882	0.123	0.759	0.384

1.1277	0.537	0.181	4.10E-73	0.942	0.172	0.769	0.356
1.1212	0.393	0.001	2.04E-72	0.652	0.002	0.649	0.392
1.1203	0.517	0.210	1.74E-25	1.039	0.261	0.778	0.307
1.1143	0.468	0.076	1.32E-44	0.800	0.077	0.723	0.392
1.1118	0.592	0.181	2.90E-44	1.209	0.289	0.920	0.411
1.1110	0.627	0.314	2.01E-29	1.235	0.378	0.857	0.313
1.1022	0.373	0.001	9.70E-66	0.635	0.001	0.633	0.372
1.0988	0.682	0.446	2.76E-23	1.549	0.643	0.906	0.236
1.0953	0.458	0.110	1.01E-42	0.801	0.105	0.696	0.348
1.0878	0.731	0.304	4.83E-74	1.651	0.462	1.188	0.427
1.0870	0.363	0.065	7.73E-42	0.632	0.063	0.569	0.298
1.0832	0.383	0.024	8.33E-57	0.653	0.020	0.633	0.359
1.0825	0.363	0.017	4.54E-53	0.609	0.016	0.594	0.346
1.0771	0.697	0.174	2.83E-51	1.732	0.409	1.323	0.523
1.0762	0.259	0.001	4.24E-39	0.492	0.002	0.490	0.258
1.0758	0.418	0.092	1.45E-55	0.741	0.089	0.652	0.326
1.0728	0.383	0.024	1.03E-33	0.662	0.030	0.633	0.359
1.0708	0.532	0.138	3.70E-46	0.874	0.134	0.741	0.394
1.0705	0.428	0.069	2.58E-51	0.718	0.063	0.655	0.359
1.0687	0.443	0.055	9.78E-38	0.840	0.087	0.753	0.388
1.0632	0.841	0.642	4.36E-69	1.985	0.967	1.018	0.199
1.0598	0.493	0.181	2.86E-61	0.862	0.179	0.683	0.312
1.0567	0.672	0.406	5.43E-60	1.308	0.476	0.832	0.266
1.0561	0.478	0.118	9.50E-38	0.909	0.171	0.738	0.360
1.0415	0.557	0.284	1.10E-58	1.008	0.293	0.715	0.273
1.0220	0.816	0.581	2.63E-61	1.828	0.833	0.994	0.235
1.0167	0.363	0.001	2.18E-61	0.590	0.002	0.589	0.362
1.0159	0.393	0.063	1.43E-27	0.679	0.064	0.615	0.330
1.0115	0.517	0.128	1.83E-26	1.057	0.214	0.843	0.389
1.0101	0.348	0.001	1.01E-57	0.551	0.002	0.549	0.347
1.0071	0.502	0.198	5.69E-36	0.865	0.198	0.667	0.304
0.9904	0.353	0.001	1.88E-68	0.553	0.001	0.552	0.352
0.9879	0.383	0.047	2.61E-42	0.636	0.049	0.588	0.336
0.9843	0.473	0.190	2.33E-42	0.857	0.205	0.651	0.283
0.9788	0.587	0.362	7.94E-45	1.085	0.402	0.683	0.225
0.9760	0.781	0.493	2.39E-52	1.547	0.643	0.904	0.288
0.9551	0.353	0.012	1.35E-53	0.556	0.011	0.545	0.341
0.9549	0.348	0.012	4.25E-41	0.563	0.012	0.551	0.336
0.9543	0.751	0.507	6.17E-35	1.524	0.672	0.853	0.244
0.9532	0.697	0.403	1.47E-39	1.392	0.521	0.871	0.294
0.9518	0.657	0.404	4.16E-49	1.199	0.476	0.723	0.253
0.9452	0.657	0.402	2.71E-22	1.225	0.490	0.735	0.255
0.9436	0.428	0.163	1.36E-53	0.709	0.155	0.554	0.265
0.9432	0.498	0.207	9.69E-20	0.894	0.247	0.647	0.291
0.9428	0.323	0.002	3.22E-55	0.507	0.002	0.505	0.321
0.9424	0.542	0.318	1.38E-46	1.022	0.364	0.658	0.224
0.9401	0.323	0.001	1.19E-51	0.513	0.001	0.513	0.322

0.9369	0.403	0.102	1.87E-24	0.659	0.096	0.563	0.301
0.9318	0.323	0.001	6.43E-56	0.508	0.001	0.507	0.322
0.9296	0.871	0.650	5.72E-32	2.282	1.252	1.030	0.221
0.9267	0.711	0.510	2.84E-57	1.408	0.638	0.771	0.201
0.9245	0.711	0.538	7.95E-55	1.478	0.706	0.773	0.173
0.9227	0.567	0.293	2.03E-36	0.994	0.323	0.671	0.274
0.9222	0.677	0.489	1.45E-29	1.411	0.655	0.756	0.188
0.9215	0.348	0.006	2.11E-47	0.530	0.006	0.525	0.342
0.9207	0.463	0.144	1.81E-25	0.833	0.187	0.647	0.319
0.9185	0.786	0.609	2.13E-48	1.736	0.888	0.847	0.177
0.9170	0.502	0.331	1.32E-15	0.949	0.386	0.564	0.171
0.9144	0.463	0.201	2.69E-34	0.846	0.234	0.612	0.262
0.9127	0.338	0.005	2.61E-54	0.515	0.004	0.511	0.333
0.9096	0.294	0.001	7.02E-43	0.465	0.001	0.464	0.293
0.9063	0.308	0.002	1.44E-47	0.459	0.002	0.456	0.306
0.9059	0.776	0.627	4.67E-45	1.785	0.950	0.836	0.149
0.9053	0.308	0.000	2.09E-49	0.497	0.000	0.497	0.308
0.9050	0.597	0.302	1.19E-17	1.132	0.410	0.723	0.295
0.9033	0.269	0.009	2.26E-31	0.428	0.007	0.421	0.260
0.9022	0.692	0.503	1.22E-58	1.370	0.618	0.752	0.189
0.8998	0.910	0.712	3.67E-39	3.191	1.711	1.479	0.198
0.8951	0.313	0.071	3.39E-16	0.539	0.077	0.462	0.242
0.8873	0.483	0.217	6.88E-30	0.792	0.220	0.572	0.266
0.8872	0.662	0.437	1.92E-29	1.218	0.530	0.687	0.225
0.8861	0.299	0.001	7.89E-42	0.471	0.002	0.469	0.298
0.8823	0.433	0.152	7.82E-19	0.756	0.182	0.574	0.281
0.8821	0.527	0.261	8.10E-32	0.887	0.281	0.606	0.266
0.8799	0.264	0.001	5.63E-41	0.430	0.001	0.429	0.263
0.8753	0.318	0.001	6.00E-49	0.473	0.001	0.473	0.317
0.8742	0.463	0.192	2.22E-41	0.734	0.186	0.548	0.271
0.8725	0.716	0.518	2.49E-28	1.444	0.700	0.744	0.198
0.8704	0.408	0.083	1.61E-34	0.622	0.077	0.545	0.325
0.8697	0.736	0.511	2.00E-33	1.439	0.681	0.758	0.225
0.8678	0.443	0.153	4.96E-36	0.701	0.150	0.551	0.290
0.8668	0.637	0.388	3.54E-25	1.153	0.464	0.689	0.249
0.8628	0.313	0.001	1.13E-43	0.455	0.001	0.453	0.312
0.8621	0.408	0.134	1.59E-29	0.670	0.143	0.527	0.274
0.8572	0.343	0.060	6.36E-33	0.557	0.059	0.498	0.283
0.8571	0.736	0.607	6.41E-50	1.599	0.873	0.726	0.129
0.8515	0.766	0.571	1.88E-31	1.589	0.802	0.787	0.195
0.8513	0.388	0.083	1.13E-29	0.584	0.075	0.510	0.305
0.8458	0.338	0.007	2.75E-45	0.488	0.007	0.481	0.331
0.8400	0.428	0.150	4.92E-35	0.644	0.139	0.505	0.278
0.8398	0.318	0.004	2.17E-42	0.481	0.005	0.476	0.314
0.8395	0.363	0.079	3.73E-47	0.536	0.067	0.469	0.284
0.8386	0.423	0.102	1.70E-29	0.662	0.113	0.548	0.321
0.8369	0.274	0.002	1.65E-39	0.415	0.002	0.413	0.272

0.8364	0.726	0.528	8.10E-36	1.410	0.693	0.717	0.198
0.8315	0.607	0.377	3.89E-24	1.061	0.418	0.643	0.230
0.8202	0.299	0.026	1.22E-26	0.464	0.035	0.429	0.273
0.8183	0.821	0.681	1.50E-42	1.872	1.089	0.783	0.140
0.8160	0.557	0.310	2.51E-26	0.871	0.318	0.553	0.247
0.8157	0.209	0.017	5.14E-13	0.292	0.018	0.274	0.192
0.8141	0.657	0.492	4.28E-26	1.235	0.612	0.623	0.165
0.8127	0.527	0.302	1.18E-29	0.880	0.317	0.563	0.225
0.8117	0.577	0.342	1.57E-06	1.112	0.462	0.650	0.235
0.8095	0.582	0.239	1.15E-20	1.132	0.371	0.761	0.343
0.8064	0.284	0.002	2.56E-41	0.433	0.002	0.431	0.282
0.8062	0.403	0.199	7.70E-18	0.648	0.205	0.442	0.204
0.8000	0.418	0.128	3.57E-35	0.619	0.126	0.494	0.290
0.7997	0.532	0.291	7.55E-27	0.901	0.317	0.584	0.241
0.7951	0.269	0.053	1.91E-07	0.447	0.056	0.391	0.216
0.7912	0.299	0.000	8.67E-45	0.444	0.001	0.443	0.299
0.7905	0.512	0.351	2.46E-40	0.871	0.376	0.495	0.161
0.7884	0.632	0.506	2.03E-19	1.314	0.720	0.594	0.126
0.7879	0.299	0.011	4.16E-28	0.431	0.012	0.419	0.288
0.7869	0.478	0.221	1.84E-24	0.720	0.223	0.496	0.257
0.7858	0.423	0.121	8.05E-28	0.656	0.131	0.525	0.302
0.7855	0.592	0.276	6.87E-36	1.088	0.370	0.718	0.316
0.7852	0.622	0.360	1.05E-35	1.305	0.551	0.753	0.262
0.7840	0.269	0.000	4.15E-44	0.407	0.000	0.407	0.269
0.7839	0.771	0.550	1.10E-30	1.507	0.759	0.748	0.221
0.7805	0.328	0.077	3.23E-23	0.464	0.076	0.389	0.251
0.7795	0.289	0.001	2.62E-35	0.419	0.001	0.419	0.288
0.7740	0.478	0.273	2.87E-25	0.784	0.288	0.496	0.205
0.7718	0.303	0.000	2.93E-49	0.431	0.001	0.431	0.303
0.7684	0.244	0.001	6.80E-30	0.338	0.001	0.337	0.243
0.7646	0.463	0.161	1.38E-26	0.735	0.180	0.555	0.302
0.7641	0.443	0.211	1.07E-26	0.683	0.206	0.476	0.232
0.7606	0.473	0.221	4.44E-35	0.747	0.229	0.518	0.252
0.7588	0.507	0.160	1.77E-21	0.938	0.271	0.667	0.347
0.7588	0.632	0.415	2.97E-32	1.110	0.492	0.618	0.217
0.7555	0.930	0.812	3.14E-42	2.553	1.720	0.833	0.118
0.7494	0.582	0.474	2.20E-42	1.086	0.566	0.520	0.108
0.7459	0.527	0.332	1.97E-23	0.894	0.357	0.537	0.195
0.7426	0.557	0.341	3.05E-25	0.932	0.390	0.542	0.216
0.7419	0.478	0.326	3.78E-31	0.794	0.333	0.460	0.152
0.7391	0.259	0.026	3.88E-19	0.379	0.026	0.353	0.233
0.7381	0.622	0.457	4.56E-21	1.102	0.542	0.560	0.165
0.7380	0.368	0.115	2.56E-28	0.541	0.112	0.429	0.253
0.7349	0.373	0.117	3.74E-27	0.529	0.106	0.423	0.256
0.7302	0.289	0.008	2.84E-36	0.392	0.007	0.384	0.281
0.7281	0.980	0.890	1.07E-27	3.168	2.195	0.973	0.090
0.7271	0.582	0.408	1.65E-10	1.002	0.477	0.525	0.174

0.7259	0.512	0.319	1.05E-15	0.837	0.341	0.496	0.193
0.7242	0.274	0.022	6.39E-26	0.390	0.021	0.370	0.252
0.7198	0.488	0.265	2.11E-25	0.777	0.275	0.502	0.223
0.7158	0.443	0.283	2.77E-24	0.712	0.301	0.412	0.160
0.7143	0.368	0.119	5.07E-24	0.522	0.117	0.405	0.249
0.7117	0.478	0.263	7.83E-14	0.729	0.267	0.462	0.215
0.7109	0.836	0.680	4.44E-29	1.795	1.084	0.711	0.156
0.7107	0.308	0.051	3.41E-24	0.448	0.050	0.398	0.257
0.7106	0.557	0.389	1.49E-30	0.958	0.442	0.516	0.168
0.7098	0.413	0.231	4.06E-18	0.640	0.236	0.404	0.182
0.7095	0.468	0.269	1.72E-16	0.742	0.278	0.464	0.199
0.7075	0.547	0.340	1.47E-13	0.964	0.429	0.536	0.207
0.7068	0.478	0.313	3.50E-15	0.758	0.321	0.437	0.165
0.7061	0.443	0.266	1.22E-31	0.685	0.258	0.427	0.177
0.7050	0.448	0.076	1.70E-24	0.885	0.183	0.702	0.372
0.7041	0.269	0.009	9.64E-28	0.383	0.008	0.375	0.260
0.7024	0.388	0.176	3.97E-17	0.579	0.175	0.404	0.212
0.7024	0.299	0.028	5.31E-23	0.398	0.026	0.372	0.271
0.7024	0.552	0.372	9.74E-21	0.950	0.426	0.524	0.180
0.7018	0.970	0.874	1.84E-28	2.919	2.096	0.823	0.096
0.7014	0.488	0.300	2.35E-18	0.766	0.322	0.444	0.188
0.7012	0.423	0.236	2.98E-27	0.658	0.229	0.429	0.187
0.7012	0.517	0.324	2.71E-15	0.815	0.344	0.471	0.193
0.6991	0.428	0.196	7.47E-21	0.622	0.187	0.434	0.232
0.6962	0.388	0.158	1.06E-28	0.561	0.153	0.407	0.230
0.6960	0.328	0.082	1.87E-28	0.452	0.073	0.379	0.246
0.6955	0.279	0.002	1.99E-33	0.388	0.003	0.385	0.277
0.6948	0.313	0.044	6.85E-31	0.434	0.040	0.394	0.269
0.6921	0.294	0.000	1.36E-38	0.384	0.000	0.383	0.294
0.6917	0.448	0.246	1.00E-35	0.723	0.260	0.463	0.202
0.6897	0.443	0.231	5.65E-19	0.660	0.236	0.424	0.212
0.6888	0.393	0.170	3.30E-25	0.596	0.166	0.430	0.223
0.6882	0.527	0.327	8.44E-14	0.843	0.362	0.481	0.200
0.6863	0.905	0.660	2.27E-21	3.166	1.900	1.267	0.245
0.6797	0.413	0.207	7.09E-25	0.609	0.203	0.405	0.206
0.6779	0.264	0.001	1.93E-35	0.349	0.001	0.348	0.263
0.6774	0.552	0.345	4.57E-22	0.902	0.379	0.523	0.207
0.6771	0.294	0.039	2.64E-21	0.401	0.035	0.366	0.255
0.6764	0.383	0.209	1.50E-26	0.587	0.203	0.384	0.174
0.6746	0.393	0.184	1.59E-22	0.595	0.185	0.410	0.209
0.6741	0.269	0.025	2.36E-36	0.366	0.023	0.342	0.244
0.6733	0.572	0.433	7.80E-28	0.974	0.504	0.470	0.139
0.6695	0.433	0.244	1.10E-14	0.725	0.281	0.444	0.189
0.6686	0.458	0.242	8.39E-15	0.662	0.242	0.420	0.216
0.6661	0.398	0.191	3.10E-19	0.589	0.194	0.395	0.207
0.6659	0.826	0.669	3.62E-18	1.970	1.265	0.705	0.157
0.6642	0.746	0.657	1.18E-28	1.645	1.074	0.571	0.089

0.6638	0.423	0.245	1.34E-28	0.639	0.247	0.392	0.178
0.6637	0.552	0.362	8.59E-21	0.930	0.427	0.503	0.190
0.6635	0.522	0.341	3.46E-15	0.838	0.384	0.453	0.181
0.6632	0.254	0.000	2.87E-34	0.327	0.001	0.327	0.254
0.6612	0.567	0.241	3.69E-31	1.112	0.403	0.708	0.326
0.6597	0.254	0.001	2.06E-33	0.345	0.002	0.344	0.253
0.6563	0.358	0.118	4.39E-26	0.480	0.106	0.374	0.240
0.6555	0.279	0.019	5.75E-21	0.378	0.020	0.358	0.260
0.6551	0.303	0.029	3.96E-17	0.432	0.044	0.387	0.274
0.6524	0.214	0.046	5.94E-06	0.323	0.059	0.265	0.168
0.6514	0.333	0.072	8.51E-16	0.472	0.069	0.403	0.261
0.6504	0.234	0.001	2.67E-29	0.313	0.001	0.312	0.233
0.6498	0.294	0.002	1.71E-34	0.382	0.002	0.380	0.292
0.6497	0.254	0.000	6.95E-33	0.338	0.000	0.337	0.254
0.6493	0.388	0.161	1.67E-24	0.535	0.153	0.382	0.227
0.6484	0.557	0.383	6.58E-30	0.930	0.449	0.482	0.174
0.6483	0.333	0.112	3.10E-12	0.472	0.117	0.354	0.221
0.6480	0.328	0.176	6.28E-08	0.493	0.187	0.307	0.152
0.6469	0.343	0.094	2.12E-21	0.470	0.093	0.377	0.249
0.6464	0.254	0.035	1.36E-31	0.349	0.030	0.319	0.219
0.6426	0.697	0.546	3.60E-16	1.338	0.763	0.575	0.151
0.6412	0.308	0.096	6.79E-23	0.447	0.091	0.357	0.212
0.6412	0.502	0.291	1.60E-11	0.747	0.297	0.449	0.211
0.6400	0.602	0.486	3.08E-19	1.113	0.625	0.488	0.116
0.6390	0.279	0.021	9.49E-28	0.376	0.019	0.357	0.258
0.6380	0.413	0.190	1.80E-15	0.578	0.178	0.399	0.223
0.6378	0.308	0.065	6.09E-11	0.447	0.074	0.373	0.243
0.6343	0.318	0.084	1.01E-28	0.421	0.074	0.347	0.234
0.6331	0.408	0.164	3.27E-16	0.558	0.161	0.397	0.244
0.6329	0.348	0.105	2.76E-22	0.457	0.096	0.361	0.243
0.6323	0.269	0.029	1.17E-23	0.372	0.032	0.340	0.240
0.6323	0.627	0.527	5.59E-15	1.160	0.694	0.466	0.100
0.6321	0.284	0.084	2.07E-11	0.377	0.080	0.297	0.200
0.6310	0.234	0.000	3.27E-27	0.337	0.000	0.337	0.234
0.6298	0.448	0.320	9.31E-18	0.709	0.336	0.373	0.128
0.6289	0.279	0.075	1.14E-18	0.367	0.064	0.303	0.204
0.6280	0.493	0.377	8.81E-09	0.882	0.463	0.419	0.116
0.6272	0.458	0.186	8.27E-14	0.897	0.340	0.557	0.272
0.6270	0.348	0.115	2.86E-21	0.458	0.105	0.353	0.233
0.6256	0.264	0.036	2.10E-17	0.346	0.034	0.312	0.228
0.6256	0.299	0.069	2.83E-24	0.403	0.063	0.340	0.230
0.6246	0.244	0.001	2.87E-31	0.318	0.001	0.316	0.243
0.6243	0.706	0.605	2.83E-22	1.378	0.849	0.529	0.101
0.6227	0.428	0.260	8.88E-15	0.638	0.271	0.367	0.168
0.6220	0.303	0.073	5.65E-36	0.397	0.062	0.335	0.230
0.6213	0.483	0.339	3.36E-10	0.768	0.376	0.391	0.144
0.6203	0.313	0.086	2.55E-18	0.423	0.087	0.336	0.227

0.6199	0.274	0.016	2.95E-21	0.345	0.017	0.328	0.258
0.6198	0.338	0.064	2.60E-23	0.425	0.061	0.364	0.274
0.6190	0.363	0.181	5.36E-09	0.588	0.209	0.380	0.182
0.6154	0.527	0.359	1.26E-21	0.908	0.438	0.471	0.168
0.6135	0.433	0.200	6.74E-17	0.601	0.194	0.408	0.233
0.6124	0.438	0.262	1.78E-13	0.658	0.284	0.375	0.176
0.6101	0.547	0.412	7.86E-11	0.940	0.493	0.447	0.135
0.6097	0.234	0.001	1.31E-29	0.298	0.001	0.298	0.233
0.6093	0.348	0.176	2.14E-06	0.465	0.164	0.301	0.172
0.6083	0.313	0.071	1.42E-19	0.413	0.062	0.351	0.242
0.6083	0.269	0.000	9.07E-34	0.329	0.000	0.329	0.269
0.6079	0.383	0.154	6.28E-27	0.514	0.154	0.361	0.229
0.6078	0.269	0.003	2.01E-30	0.323	0.003	0.320	0.266
0.6065	0.284	0.058	2.76E-26	0.383	0.052	0.331	0.226
0.6061	0.249	0.005	6.06E-30	0.311	0.005	0.306	0.244
0.6051	0.239	0.005	1.82E-26	0.297	0.004	0.293	0.234
0.6050	0.677	0.581	8.43E-15	1.287	0.817	0.470	0.096
0.6046	0.716	0.599	3.40E-25	1.380	0.856	0.524	0.117
0.6034	0.333	0.075	4.36E-26	0.422	0.071	0.351	0.258
0.6034	0.313	0.103	1.35E-23	0.413	0.095	0.318	0.210
0.6026	0.542	0.429	1.30E-08	0.924	0.500	0.424	0.113
0.6022	0.856	0.780	1.57E-21	2.053	1.465	0.589	0.076
0.6022	0.289	0.065	2.72E-23	0.388	0.063	0.325	0.224
0.6006	0.264	0.001	9.82E-33	0.325	0.001	0.324	0.263
0.6004	0.383	0.108	2.07E-17	0.548	0.131	0.417	0.275
0.6002	0.652	0.552	6.78E-20	1.258	0.769	0.490	0.100
0.5993	0.642	0.544	3.54E-21	1.209	0.713	0.496	0.098
0.5992	0.264	0.052	4.73E-24	0.358	0.047	0.310	0.212
0.5988	0.323	0.082	7.25E-26	0.423	0.077	0.345	0.241
0.5973	0.234	0.025	1.93E-16	0.321	0.023	0.298	0.209
0.5945	0.264	0.065	1.13E-09	0.333	0.060	0.273	0.199
0.5939	0.254	0.001	2.94E-25	0.325	0.002	0.323	0.253
0.5926	0.353	0.158	1.18E-23	0.490	0.162	0.328	0.195
0.5924	0.259	0.044	8.89E-18	0.324	0.037	0.287	0.215
0.5917	0.289	0.079	8.23E-17	0.386	0.074	0.311	0.210
0.5915	0.378	0.190	2.46E-12	0.511	0.178	0.333	0.188
0.5900	0.388	0.211	7.58E-19	0.552	0.206	0.346	0.177
0.5894	0.806	0.644	2.95E-13	1.823	1.192	0.632	0.162
0.5874	0.299	0.060	3.96E-27	0.367	0.056	0.311	0.239
0.5862	0.249	0.000	1.82E-30	0.306	0.000	0.306	0.249
0.5856	0.627	0.518	5.97E-21	1.143	0.684	0.459	0.109
0.5841	0.378	0.218	2.67E-11	0.526	0.209	0.317	0.160
0.5841	0.488	0.284	5.46E-09	0.693	0.294	0.399	0.204
0.5835	0.274	0.029	8.81E-22	0.350	0.035	0.315	0.245
0.5824	0.488	0.381	3.14E-16	0.814	0.435	0.379	0.107
0.5818	0.488	0.349	7.38E-23	0.756	0.381	0.375	0.139
0.5791	0.234	0.020	5.63E-14	0.319	0.017	0.302	0.214

0.5789	0.677	0.586	6.26E-15	1.262	0.810	0.452	0.091
0.5782	0.234	0.001	2.35E-32	0.296	0.001	0.295	0.233
0.5772	0.433	0.290	2.56E-05	0.696	0.327	0.370	0.143
0.5768	0.323	0.145	5.12E-13	0.442	0.137	0.304	0.178
0.5753	0.408	0.204	1.90E-15	0.588	0.207	0.381	0.204
0.5750	0.428	0.154	7.12E-09	0.667	0.194	0.473	0.274
0.5735	0.259	0.042	1.15E-22	0.323	0.037	0.285	0.217
0.5731	0.468	0.329	2.68E-13	0.725	0.361	0.364	0.139
0.5722	0.577	0.201	8.55E-20	1.229	0.458	0.771	0.376
0.5713	0.264	0.025	3.00E-27	0.324	0.021	0.303	0.239
0.5704	0.343	0.145	3.50E-14	0.468	0.151	0.316	0.198
0.5697	0.259	0.036	9.91E-13	0.347	0.041	0.306	0.223
0.5695	0.488	0.349	1.12E-05	0.758	0.381	0.377	0.139
0.5673	0.343	0.124	1.83E-08	0.446	0.130	0.316	0.219
0.5662	0.348	0.129	2.84E-25	0.484	0.133	0.350	0.219
0.5647	0.214	0.011	1.85E-12	0.276	0.014	0.263	0.203
0.5631	0.383	0.175	1.19E-17	0.509	0.168	0.340	0.208
0.5630	0.358	0.191	1.08E-21	0.484	0.178	0.307	0.167
0.5606	0.438	0.300	1.52E-11	0.658	0.314	0.344	0.138
0.5600	0.403	0.287	1.81E-10	0.612	0.299	0.313	0.116
0.5595	0.284	0.020	5.07E-16	0.389	0.039	0.351	0.264
0.5591	0.244	0.002	3.74E-22	0.285	0.003	0.283	0.242
0.5591	0.343	0.179	6.67E-20	0.485	0.183	0.302	0.164
0.5575	0.517	0.421	9.75E-13	0.858	0.480	0.377	0.096
0.5567	0.458	0.315	4.84E-14	0.684	0.330	0.353	0.143
0.5547	0.358	0.146	4.37E-04	0.525	0.180	0.345	0.212
0.5541	0.219	0.001	1.72E-24	0.273	0.001	0.272	0.218
0.5538	0.527	0.376	1.16E-03	0.845	0.450	0.396	0.151
0.5535	0.602	0.485	1.75E-10	1.014	0.607	0.407	0.117
0.5525	0.577	0.494	1.07E-13	0.985	0.618	0.367	0.083
0.5518	0.338	0.171	1.63E-15	0.467	0.168	0.299	0.167
0.5506	0.284	0.107	2.41E-17	0.389	0.095	0.294	0.177
0.5500	0.323	0.141	2.84E-11	0.427	0.136	0.292	0.182
0.5499	0.323	0.109	2.85E-19	0.391	0.100	0.291	0.214
0.5494	0.328	0.155	1.13E-10	0.458	0.148	0.310	0.173
0.5490	0.627	0.595	5.22E-17	1.243	0.874	0.369	0.032
0.5489	0.831	0.676	1.95E-07	2.274	1.565	0.709	0.155
0.5478	0.299	0.066	9.35E-08	0.459	0.108	0.352	0.233
0.5471	0.493	0.364	1.39E-10	0.787	0.426	0.361	0.129
0.5464	0.184	0.007	1.61E-16	0.257	0.006	0.251	0.177
0.5462	0.343	0.093	1.04E-19	0.421	0.084	0.338	0.250
0.5461	0.229	0.006	1.23E-21	0.288	0.005	0.283	0.223
0.5459	0.209	0.001	3.44E-25	0.250	0.001	0.249	0.208
0.5458	0.657	0.549	1.21E-11	1.176	0.733	0.443	0.108
0.5457	0.318	0.129	3.61E-14	0.440	0.130	0.310	0.189
0.5446	0.234	0.003	4.40E-21	0.287	0.004	0.283	0.231
0.5439	0.239	0.008	3.81E-22	0.280	0.007	0.273	0.231

0.5438	0.294	0.067	3.07E-20	0.362	0.059	0.303	0.227
0.5437	0.269	0.056	4.48E-14	0.312	0.049	0.263	0.213
0.5432	0.697	0.587	4.06E-18	1.417	0.899	0.518	0.110
0.5427	0.373	0.182	5.31E-14	0.507	0.180	0.328	0.191
0.5418	0.284	0.061	2.60E-21	0.358	0.061	0.298	0.223
0.5418	0.985	0.872	5.12E-65	4.507	3.463	1.044	0.113
0.5399	0.303	0.084	1.15E-17	0.392	0.082	0.310	0.219
0.5380	0.303	0.119	2.35E-14	0.395	0.108	0.286	0.184
0.5364	0.428	0.296	1.82E-08	0.609	0.301	0.307	0.132
0.5356	0.498	0.388	5.13E-05	0.783	0.463	0.320	0.110
0.5356	0.234	0.042	1.20E-15	0.294	0.036	0.257	0.192
0.5349	0.313	0.130	1.33E-12	0.416	0.132	0.284	0.183
0.5347	0.567	0.414	2.66E-08	1.018	0.579	0.440	0.153
0.5345	0.254	0.052	3.02E-11	0.330	0.053	0.277	0.202
0.5339	0.706	0.563	3.17E-07	1.326	0.821	0.505	0.143
0.5339	0.348	0.175	1.15E-19	0.516	0.188	0.328	0.173
0.5333	0.224	0.000	3.05E-30	0.271	0.000	0.271	0.224
0.5331	0.308	0.107	1.63E-14	0.402	0.098	0.304	0.201
0.5328	0.448	0.286	1.65E-09	0.628	0.290	0.337	0.162
0.5312	0.159	0.000	2.52E-24	0.224	0.000	0.224	0.159
0.5311	0.423	0.315	1.22E-08	0.638	0.345	0.293	0.108
0.5306	0.353	0.202	1.90E-22	0.511	0.215	0.296	0.151
0.5299	0.269	0.091	8.48E-16	0.343	0.087	0.256	0.178
0.5296	0.343	0.139	3.51E-24	0.443	0.131	0.312	0.204
0.5289	0.244	0.023	1.49E-23	0.301	0.025	0.276	0.221
0.5281	0.368	0.192	4.49E-08	0.494	0.186	0.309	0.176
0.5267	0.284	0.095	9.77E-12	0.341	0.085	0.256	0.189
0.5266	0.303	0.125	1.19E-14	0.401	0.118	0.282	0.178
0.5266	0.234	0.013	1.93E-16	0.289	0.014	0.275	0.221
0.5263	0.463	0.346	1.34E-06	0.696	0.368	0.328	0.117
0.5258	0.204	0.001	3.59E-21	0.241	0.001	0.241	0.203
0.5236	0.159	0.001	5.41E-20	0.217	0.001	0.215	0.158
0.5231	0.229	0.003	8.65E-20	0.273	0.003	0.270	0.226
0.5209	0.229	0.004	9.26E-20	0.279	0.005	0.274	0.225
0.5207	0.378	0.206	2.44E-06	0.494	0.204	0.290	0.172
0.5196	0.706	0.586	3.74E-09	1.344	0.880	0.464	0.120
0.5194	0.244	0.002	3.51E-24	0.274	0.002	0.272	0.242
0.5194	0.582	0.506	3.43E-10	0.983	0.638	0.345	0.076
0.5193	0.507	0.403	1.00E-10	0.807	0.447	0.360	0.104
0.5191	0.224	0.000	1.73E-31	0.250	0.001	0.250	0.224
0.5188	0.194	0.008	4.34E-16	0.253	0.009	0.244	0.186
0.5181	0.463	0.337	3.33E-14	0.748	0.385	0.363	0.126
0.5175	0.184	0.000	1.01E-19	0.246	0.000	0.245	0.184
0.5173	0.294	0.145	2.97E-24	0.388	0.139	0.250	0.149
0.5167	0.214	0.004	1.10E-19	0.256	0.004	0.252	0.210
0.5163	0.254	0.001	1.78E-28	0.297	0.001	0.295	0.253
0.5159	0.448	0.277	5.72E-22	0.671	0.304	0.367	0.171

0.5157	0.423	0.259	1.53E-08	0.572	0.261	0.311	0.164
0.5156	0.313	0.100	3.40E-09	0.366	0.090	0.277	0.213
0.5142	0.363	0.179	3.88E-15	0.467	0.172	0.296	0.184
0.5140	0.433	0.328	1.38E-07	0.632	0.346	0.286	0.105
0.5138	0.284	0.089	7.66E-14	0.342	0.076	0.266	0.195
0.5118	0.343	0.215	1.08E-22	0.496	0.212	0.284	0.128
0.5113	0.249	0.083	8.99E-13	0.313	0.079	0.234	0.166
0.5112	0.458	0.308	3.37E-05	0.654	0.328	0.326	0.150
0.5091	0.234	0.021	3.21E-20	0.261	0.020	0.241	0.213
0.5091	0.234	0.040	1.92E-20	0.271	0.037	0.235	0.194
0.5091	0.532	0.397	5.91E-14	0.779	0.430	0.349	0.135
0.5090	0.448	0.280	3.34E-07	0.639	0.297	0.342	0.168
0.5088	0.229	0.029	3.51E-15	0.276	0.025	0.251	0.200
0.5080	0.214	0.004	2.96E-16	0.241	0.004	0.237	0.210
0.5077	0.323	0.161	2.64E-12	0.423	0.150	0.273	0.162
0.5076	0.244	0.021	4.29E-18	0.292	0.023	0.269	0.223
0.5057	0.418	0.240	3.58E-04	0.576	0.270	0.306	0.178
0.5053	0.393	0.287	2.61E-14	0.586	0.304	0.282	0.106
0.5050	0.547	0.427	2.42E-11	0.899	0.527	0.372	0.120
0.5050	0.254	0.069	3.64E-12	0.297	0.060	0.237	0.185
0.5027	0.284	0.128	3.04E-20	0.370	0.115	0.255	0.156
0.5026	0.368	0.204	1.18E-05	0.505	0.200	0.305	0.164
0.5021	0.249	0.029	1.28E-17	0.306	0.027	0.279	0.220
0.5020	0.214	0.004	2.07E-16	0.240	0.004	0.235	0.210
0.5019	0.338	0.163	1.23E-09	0.440	0.152	0.288	0.175
0.5016	0.562	0.498	5.20E-07	0.948	0.614	0.334	0.064
0.5015	0.214	0.001	4.41E-28	0.250	0.000	0.250	0.213
0.5006	0.264	0.038	6.68E-09	0.344	0.056	0.289	0.226
0.5003	0.279	0.094	2.76E-11	0.335	0.083	0.252	0.185
0.5000	0.303	0.099	1.96E-17	0.363	0.088	0.275	0.204
0.4993	0.458	0.342	3.26E-10	0.667	0.377	0.290	0.116
0.4993	0.373	0.198	1.58E-10	0.501	0.196	0.305	0.175
0.4986	0.597	0.530	3.84E-08	1.049	0.698	0.351	0.067
0.4986	0.403	0.259	2.46E-13	0.594	0.288	0.306	0.144
0.4985	0.249	0.019	2.73E-24	0.274	0.016	0.258	0.230
0.4971	0.537	0.433	4.53E-07	0.863	0.518	0.345	0.104
0.4967	0.612	0.531	1.99E-12	1.061	0.733	0.329	0.081
0.4965	0.358	0.239	2.51E-07	0.529	0.262	0.267	0.119
0.4958	0.522	0.405	1.18E-09	0.775	0.447	0.328	0.117
0.4955	0.214	0.010	3.71E-17	0.266	0.010	0.256	0.204
0.4948	0.229	0.016	9.15E-18	0.245	0.013	0.233	0.213
0.4941	0.358	0.211	2.11E-09	0.464	0.207	0.258	0.147
0.4937	0.318	0.178	1.11E-05	0.433	0.177	0.257	0.140
0.4936	0.194	0.013	1.88E-12	0.260	0.017	0.243	0.181
0.4931	0.239	0.002	9.27E-23	0.266	0.002	0.263	0.237
0.4920	0.726	0.667	1.35E-10	1.430	1.016	0.414	0.059
0.4905	0.264	0.083	1.22E-13	0.318	0.075	0.243	0.181

0.4893	0.413	0.257	4.63E-10	0.616	0.291	0.325	0.156
0.4883	0.517	0.260	7.06E-17	0.877	0.384	0.494	0.257
0.4879	0.214	0.000	2.83E-27	0.240	0.000	0.240	0.214
0.4872	0.617	0.516	4.07E-08	1.121	0.693	0.428	0.101
0.4867	0.214	0.020	9.65E-17	0.239	0.017	0.222	0.194
0.4866	0.398	0.292	1.01E-05	0.586	0.302	0.284	0.106
0.4863	0.517	0.437	1.40E-07	0.841	0.514	0.327	0.080
0.4854	0.483	0.396	3.27E-08	0.745	0.457	0.288	0.087
0.4847	0.418	0.292	1.72E-06	0.604	0.303	0.300	0.126
0.4842	0.229	0.012	3.04E-22	0.259	0.011	0.248	0.217
0.4833	0.313	0.196	3.15E-11	0.423	0.193	0.229	0.117
0.4830	0.254	0.013	8.42E-21	0.281	0.012	0.268	0.241
0.4827	0.323	0.129	1.83E-09	0.389	0.126	0.263	0.194
0.4804	0.284	0.137	6.46E-14	0.361	0.132	0.229	0.147
0.4802	0.164	0.000	2.41E-16	0.215	0.000	0.214	0.164
0.4799	0.438	0.287	3.26E-20	0.668	0.330	0.338	0.151
0.4797	0.264	0.064	2.03E-12	0.293	0.057	0.236	0.200
0.4797	0.284	0.074	4.10E-11	0.335	0.070	0.264	0.210
0.4778	0.413	0.312	6.60E-07	0.594	0.336	0.259	0.101
0.4776	0.159	0.000	8.58E-20	0.217	0.000	0.216	0.159
0.4770	0.338	0.162	3.30E-04	0.455	0.177	0.278	0.176
0.4769	0.318	0.135	1.12E-09	0.375	0.127	0.249	0.183
0.4769	0.259	0.082	1.37E-06	0.328	0.086	0.242	0.177
0.4766	0.403	0.284	9.63E-08	0.548	0.291	0.256	0.119
0.4764	0.254	0.065	4.70E-12	0.305	0.061	0.244	0.189
0.4760	0.274	0.084	2.25E-17	0.302	0.073	0.229	0.190
0.4757	0.493	0.380	2.53E-03	0.711	0.418	0.292	0.113
0.4752	0.632	0.532	3.81E-12	1.152	0.732	0.420	0.100
0.4749	0.617	0.536	4.89E-08	1.041	0.689	0.352	0.081
0.4748	0.204	0.012	3.33E-16	0.232	0.013	0.220	0.192
0.4748	0.224	0.019	8.51E-22	0.256	0.016	0.240	0.205
0.4747	0.299	0.132	3.29E-06	0.389	0.131	0.259	0.167
0.4743	0.363	0.211	6.17E-05	0.461	0.206	0.254	0.152
0.4741	0.338	0.196	1.37E-13	0.460	0.190	0.270	0.142
0.4739	0.313	0.156	5.41E-11	0.395	0.155	0.240	0.157
0.4724	0.279	0.092	1.58E-10	0.345	0.084	0.260	0.187
0.4722	0.129	0.000	1.37E-14	0.189	0.000	0.189	0.129
0.4718	0.224	0.002	3.80E-18	0.240	0.001	0.238	0.222
0.4718	0.423	0.343	1.43E-12	0.643	0.362	0.281	0.080
0.4705	0.289	0.117	2.73E-16	0.348	0.105	0.242	0.172
0.4693	0.303	0.149	2.56E-06	0.388	0.138	0.250	0.154
0.4692	0.353	0.241	8.49E-08	0.486	0.233	0.253	0.112
0.4688	0.299	0.115	4.91E-06	0.355	0.107	0.248	0.184
0.4684	0.498	0.358	6.90E-11	0.716	0.401	0.315	0.140
0.4680	0.204	0.001	5.68E-21	0.227	0.001	0.226	0.203
0.4676	0.284	0.126	6.45E-13	0.351	0.117	0.233	0.158
0.4674	0.851	0.709	4.71E-21	2.131	1.559	0.572	0.142

0.4669	0.289	0.133	1.11E-16	0.348	0.127	0.221	0.156
0.4669	0.562	0.471	2.10E-11	0.914	0.587	0.327	0.091
0.4663	0.299	0.113	9.50E-07	0.370	0.105	0.265	0.186
0.4658	0.259	0.050	1.04E-25	0.291	0.044	0.247	0.209
0.4655	0.766	0.669	2.69E-15	1.580	1.041	0.539	0.097
0.4653	0.388	0.218	3.90E-10	0.560	0.251	0.309	0.170
0.4652	0.279	0.103	3.89E-10	0.345	0.095	0.250	0.176
0.4651	0.383	0.265	1.83E-05	0.540	0.269	0.271	0.118
0.4635	0.209	0.036	5.70E-06	0.253	0.034	0.219	0.173
0.4633	0.269	0.145	2.52E-15	0.329	0.132	0.197	0.124
0.4629	0.542	0.421	6.51E-06	0.902	0.545	0.357	0.121
0.4629	0.254	0.077	1.47E-21	0.294	0.071	0.223	0.177
0.4621	0.403	0.304	7.13E-09	0.567	0.323	0.245	0.099
0.4621	0.333	0.208	3.26E-09	0.433	0.213	0.220	0.125
0.4621	0.388	0.283	3.69E-08	0.551	0.290	0.261	0.105
0.4620	0.264	0.038	2.92E-17	0.314	0.047	0.268	0.226
0.4619	0.214	0.000	2.16E-23	0.235	0.001	0.235	0.214
0.4617	0.393	0.222	2.17E-08	0.500	0.225	0.275	0.171
0.4603	0.194	0.049	2.90E-05	0.223	0.046	0.177	0.145
0.4601	0.662	0.581	2.33E-09	1.215	0.809	0.406	0.081
0.4596	0.846	0.750	3.06E-08	1.860	1.331	0.530	0.096
0.4594	0.313	0.176	9.66E-06	0.396	0.165	0.231	0.137
0.4585	0.408	0.309	2.74E-04	0.566	0.324	0.242	0.099
0.4582	0.373	0.282	1.82E-16	0.553	0.302	0.251	0.091
0.4579	0.264	0.042	1.39E-10	0.331	0.065	0.266	0.222
0.4578	0.254	0.068	9.81E-11	0.299	0.062	0.236	0.186
0.4575	0.353	0.221	1.15E-08	0.488	0.230	0.258	0.132
0.4571	0.224	0.006	1.85E-16	0.241	0.006	0.234	0.218
0.4571	0.269	0.079	4.01E-10	0.303	0.070	0.232	0.190
0.4564	0.279	0.098	1.08E-10	0.342	0.095	0.247	0.181
0.4564	0.219	0.033	1.15E-08	0.278	0.044	0.234	0.186
0.4561	0.388	0.276	6.06E-20	0.568	0.294	0.275	0.112
0.4558	0.179	0.001	1.98E-16	0.198	0.001	0.197	0.178
0.4555	0.254	0.070	7.10E-22	0.293	0.063	0.229	0.184
0.4553	0.229	0.074	1.08E-09	0.255	0.068	0.186	0.155
0.4551	0.294	0.121	2.51E-18	0.341	0.113	0.227	0.173
0.4547	0.219	0.002	3.27E-19	0.246	0.001	0.245	0.217
0.4540	0.483	0.396	3.62E-10	0.723	0.440	0.283	0.087
0.4533	0.408	0.295	1.17E-06	0.539	0.293	0.246	0.113
0.4527	0.687	0.588	1.20E-05	1.326	0.903	0.423	0.099
0.4526	0.318	0.144	2.35E-08	0.406	0.136	0.270	0.174
0.4512	0.303	0.167	3.00E-08	0.394	0.166	0.228	0.136
0.4511	0.343	0.213	5.51E-12	0.446	0.208	0.237	0.130
0.4502	0.229	0.037	2.54E-13	0.253	0.034	0.219	0.192
0.4501	0.353	0.270	1.21E-11	0.489	0.266	0.223	0.083
0.4499	0.682	0.571	5.27E-05	1.183	0.819	0.364	0.111
0.4486	0.234	0.042	1.46E-08	0.261	0.037	0.224	0.192

0.4479	0.398	0.240	2.92E-11	0.585	0.279	0.306	0.158
0.4477	0.483	0.391	3.39E-06	0.753	0.459	0.293	0.092
0.4469	0.199	0.056	3.04E-13	0.232	0.048	0.183	0.143
0.4469	0.756	0.624	4.93E-17	1.559	1.074	0.485	0.132
0.4469	0.542	0.444	2.21E-13	0.826	0.512	0.314	0.098
0.4464	0.612	0.518	1.33E-05	1.027	0.690	0.337	0.094
0.4460	0.348	0.180	1.36E-06	0.452	0.180	0.272	0.168
0.4457	0.567	0.480	2.44E-13	0.935	0.597	0.338	0.087
0.4447	0.393	0.185	3.31E-13	0.551	0.225	0.326	0.208
0.4435	0.279	0.113	7.20E-09	0.323	0.101	0.222	0.166
0.4426	0.448	0.306	6.18E-06	0.632	0.336	0.296	0.142
0.4425	0.607	0.573	2.71E-09	1.105	0.795	0.311	0.034
0.4421	0.522	0.476	4.64E-07	0.805	0.565	0.240	0.046
0.4419	0.204	0.001	3.30E-13	0.232	0.001	0.231	0.203
0.4411	0.144	0.001	8.52E-10	0.181	0.001	0.181	0.143
0.4406	0.214	0.023	1.00E-18	0.242	0.021	0.221	0.191
0.4405	0.264	0.067	1.21E-06	0.292	0.056	0.235	0.197
0.4402	0.353	0.288	8.19E-13	0.506	0.285	0.221	0.065
0.4398	0.264	0.070	1.33E-16	0.292	0.062	0.230	0.194
0.4398	0.279	0.098	1.28E-11	0.337	0.100	0.237	0.181
0.4396	0.562	0.479	1.67E-03	0.958	0.619	0.339	0.083
0.4385	0.383	0.211	1.44E-07	0.504	0.218	0.286	0.172
0.4382	0.353	0.219	8.77E-14	0.454	0.211	0.244	0.134
0.4379	0.264	0.086	1.99E-08	0.311	0.083	0.227	0.178
0.4376	0.219	0.011	6.91E-09	0.247	0.016	0.232	0.208
0.4374	0.408	0.295	5.01E-07	0.597	0.339	0.259	0.113
0.4372	0.433	0.323	5.31E-09	0.596	0.339	0.257	0.110
0.4356	0.199	0.000	7.31E-18	0.217	0.000	0.217	0.199
0.4356	0.229	0.031	3.53E-14	0.252	0.028	0.224	0.198
0.4346	0.179	0.001	1.11E-13	0.199	0.001	0.198	0.178
0.4342	0.602	0.530	2.12E-13	1.059	0.715	0.344	0.072
0.4333	0.244	0.093	4.92E-09	0.288	0.083	0.205	0.151
0.4326	0.184	0.068	2.43E-06	0.214	0.062	0.153	0.116
0.4326	0.458	0.402	3.11E-03	0.684	0.459	0.225	0.056
0.4325	0.343	0.189	5.69E-06	0.435	0.186	0.248	0.154
0.4319	0.229	0.064	4.86E-17	0.255	0.056	0.200	0.165
0.4318	0.478	0.364	1.42E-09	0.694	0.400	0.295	0.114
0.4314	0.308	0.136	1.02E-08	0.346	0.122	0.223	0.172
0.4311	0.398	0.307	4.73E-03	0.554	0.322	0.231	0.091
0.4301	0.303	0.169	3.76E-21	0.379	0.159	0.220	0.134
0.4299	0.259	0.082	5.46E-13	0.320	0.085	0.235	0.177
0.4285	0.308	0.185	7.38E-13	0.393	0.179	0.214	0.123
0.4283	0.264	0.080	1.22E-18	0.313	0.079	0.234	0.184
0.4280	0.179	0.001	1.12E-13	0.199	0.001	0.199	0.178
0.4277	0.239	0.029	1.56E-12	0.267	0.029	0.238	0.210
0.4273	0.512	0.433	9.53E-06	0.787	0.515	0.272	0.079
0.4272	0.338	0.216	1.95E-11	0.496	0.246	0.250	0.122

0.4268	0.836	0.706	8.44E-12	2.114	1.553	0.561	0.130
0.4268	0.512	0.442	2.29E-05	0.774	0.515	0.259	0.070
0.4265	0.159	0.018	4.09E-18	0.202	0.015	0.187	0.141
0.4261	0.328	0.214	1.29E-05	0.403	0.206	0.197	0.114
0.4258	0.244	0.074	1.43E-08	0.277	0.065	0.212	0.170
0.4252	0.627	0.588	7.65E-08	1.092	0.803	0.288	0.039
0.4251	0.532	0.514	8.67E-07	0.901	0.655	0.246	0.018
0.4248	0.269	0.079	5.78E-08	0.297	0.069	0.227	0.190
0.4243	0.303	0.178	2.20E-07	0.379	0.175	0.204	0.125
0.4239	0.537	0.459	5.54E-09	0.935	0.600	0.336	0.078
0.4232	0.244	0.053	7.39E-11	0.266	0.047	0.219	0.191
0.4226	0.254	0.039	6.04E-13	0.286	0.038	0.248	0.215
0.4223	0.388	0.270	8.34E-05	0.517	0.271	0.246	0.118
0.4219	0.433	0.340	1.70E-09	0.630	0.359	0.271	0.093
0.4210	0.164	0.002	3.27E-09	0.197	0.003	0.194	0.162
0.4210	0.438	0.321	1.10E-04	0.582	0.332	0.250	0.117
0.4209	0.264	0.124	8.92E-12	0.313	0.117	0.196	0.140
0.4193	0.194	0.003	8.02E-16	0.209	0.003	0.206	0.191
0.4192	0.274	0.145	9.00E-16	0.336	0.144	0.192	0.129
0.4187	0.164	0.003	2.72E-13	0.179	0.002	0.176	0.161
0.4182	0.433	0.343	6.35E-06	0.676	0.405	0.271	0.090
0.4180	0.333	0.177	1.33E-12	0.411	0.169	0.242	0.156
0.4175	0.303	0.137	2.32E-05	0.362	0.130	0.232	0.166
0.4175	0.224	0.089	3.19E-03	0.278	0.081	0.197	0.135
0.4175	0.269	0.153	6.22E-12	0.353	0.141	0.212	0.116
0.4161	0.473	0.357	7.09E-08	0.681	0.396	0.285	0.116
0.4161	0.224	0.053	1.50E-11	0.258	0.048	0.210	0.171
0.4157	0.547	0.465	3.48E-03	0.878	0.581	0.297	0.082
0.4156	0.164	0.036	1.97E-03	0.171	0.031	0.140	0.128
0.4154	0.323	0.164	3.52E-08	0.397	0.160	0.237	0.159
0.4151	0.433	0.308	4.42E-07	0.615	0.344	0.271	0.125
0.4142	0.348	0.203	1.42E-04	0.428	0.192	0.236	0.145
0.4141	0.229	0.014	1.29E-19	0.232	0.012	0.220	0.215
0.4131	0.179	0.011	3.37E-08	0.204	0.009	0.195	0.168
0.4131	0.284	0.124	3.79E-12	0.345	0.121	0.224	0.160
0.4130	0.358	0.238	3.70E-09	0.459	0.232	0.226	0.120
0.4122	0.289	0.156	6.93E-11	0.335	0.146	0.189	0.133
0.4111	0.403	0.276	4.48E-03	0.546	0.311	0.235	0.127
0.4110	0.269	0.068	1.15E-11	0.320	0.074	0.246	0.201
0.4107	0.428	0.325	2.03E-03	0.586	0.356	0.230	0.103
0.4107	0.189	0.000	1.97E-15	0.210	0.000	0.209	0.189
0.4105	0.697	0.601	2.36E-04	1.356	0.980	0.377	0.096
0.4099	0.214	0.061	1.12E-04	0.236	0.053	0.183	0.153
0.4099	0.209	0.007	5.01E-15	0.219	0.005	0.214	0.202
0.4097	0.323	0.180	5.39E-09	0.399	0.166	0.232	0.143
0.4091	0.308	0.174	5.69E-05	0.383	0.175	0.208	0.134
0.4090	0.368	0.261	9.76E-11	0.485	0.261	0.223	0.107

0.4088	0.154	0.000	1.28E-18	0.175	0.000	0.174	0.154
0.4083	0.194	0.022	4.60E-08	0.219	0.022	0.197	0.172
0.4080	0.473	0.394	4.10E-03	0.691	0.440	0.251	0.079
0.4077	0.463	0.373	7.58E-04	0.670	0.438	0.232	0.090
0.4075	0.403	0.290	8.30E-05	0.540	0.299	0.241	0.113
0.4057	0.244	0.036	1.99E-08	0.279	0.047	0.232	0.208
0.4057	0.234	0.098	2.41E-03	0.273	0.091	0.182	0.136
0.4055	0.179	0.011	4.54E-18	0.206	0.009	0.197	0.168
0.4054	0.403	0.270	1.82E-04	0.500	0.274	0.226	0.133
0.4052	0.199	0.002	3.05E-15	0.208	0.002	0.206	0.197
0.4049	0.254	0.050	1.42E-15	0.282	0.051	0.230	0.204
0.4046	0.318	0.145	4.79E-09	0.448	0.178	0.271	0.173
0.4044	0.264	0.099	5.11E-08	0.330	0.113	0.218	0.165
0.4042	0.274	0.078	1.34E-09	0.310	0.073	0.237	0.196
0.4034	0.294	0.116	8.02E-04	0.453	0.195	0.258	0.178
0.4022	0.269	0.085	1.76E-08	0.297	0.077	0.220	0.184
0.4014	0.970	0.936	2.71E-12	3.174	2.573	0.601	0.034
0.4011	0.224	0.052	3.85E-15	0.231	0.050	0.182	0.172
0.4007	0.239	0.085	1.06E-05	0.271	0.080	0.192	0.154
0.4006	0.279	0.116	6.40E-07	0.340	0.117	0.224	0.163
0.4004	0.363	0.219	1.68E-03	0.456	0.220	0.236	0.144
0.3995	0.313	0.178	8.04E-06	0.360	0.167	0.193	0.135
0.3993	0.498	0.411	8.55E-08	0.755	0.483	0.272	0.087
0.3990	0.209	0.073	5.94E-12	0.242	0.064	0.178	0.136
0.3984	0.303	0.181	2.70E-09	0.375	0.183	0.191	0.122
0.3982	0.264	0.141	7.79E-04	0.318	0.129	0.189	0.123
0.3978	0.527	0.413	2.95E-05	0.918	0.564	0.353	0.114
0.3976	0.657	0.535	1.29E-06	1.246	0.852	0.394	0.122
0.3972	0.353	0.240	1.44E-04	0.451	0.241	0.210	0.113
0.3970	0.209	0.014	2.97E-15	0.209	0.012	0.197	0.195
0.3965	0.219	0.070	1.93E-13	0.261	0.064	0.197	0.149
0.3963	0.433	0.363	9.82E-13	0.678	0.415	0.263	0.070
0.3960	0.313	0.165	7.48E-07	0.375	0.157	0.218	0.148
0.3960	0.214	0.001	8.15E-15	0.222	0.001	0.221	0.213
0.3946	0.229	0.096	5.27E-06	0.253	0.087	0.166	0.133
0.3946	0.731	0.703	9.31E-08	1.437	1.114	0.322	0.028
0.3941	0.279	0.108	2.44E-10	0.304	0.095	0.208	0.171
0.3928	0.801	0.612	6.91E-07	1.876	1.225	0.651	0.189
0.3923	0.443	0.319	1.03E-04	0.605	0.357	0.248	0.124
0.3919	0.199	0.001	5.95E-13	0.213	0.003	0.209	0.198
0.3914	0.294	0.139	1.30E-10	0.326	0.129	0.197	0.155
0.3913	0.224	0.042	1.14E-07	0.228	0.036	0.191	0.182
0.3888	0.418	0.318	1.52E-06	0.561	0.332	0.230	0.100
0.3886	0.239	0.076	3.42E-14	0.278	0.074	0.204	0.163
0.3885	0.219	0.086	9.62E-15	0.249	0.075	0.174	0.133
0.3871	0.333	0.185	1.21E-07	0.410	0.189	0.221	0.148
0.3869	0.174	0.001	4.10E-13	0.196	0.001	0.195	0.173

0.3865	0.284	0.114	6.67E-08	0.301	0.112	0.189	0.170
0.3861	0.194	0.004	7.05E-19	0.208	0.003	0.204	0.190
0.3861	0.398	0.302	1.06E-06	0.552	0.323	0.229	0.096
0.3859	0.229	0.015	3.08E-14	0.227	0.015	0.212	0.214
0.3859	0.244	0.087	3.29E-05	0.262	0.079	0.183	0.157
0.3845	0.154	0.000	2.67E-20	0.173	0.000	0.173	0.154
0.3842	0.149	0.002	1.04E-09	0.169	0.002	0.167	0.147
0.3835	0.209	0.025	1.14E-06	0.241	0.033	0.208	0.184
0.3833	0.254	0.076	9.76E-06	0.275	0.072	0.203	0.178
0.3832	0.418	0.312	1.83E-05	0.567	0.347	0.220	0.106
0.3828	0.229	0.086	4.43E-06	0.241	0.076	0.165	0.143
0.3823	0.179	0.018	4.88E-14	0.190	0.017	0.173	0.161
0.3819	0.209	0.044	5.80E-08	0.223	0.040	0.183	0.165
0.3819	0.199	0.056	1.00E-16	0.229	0.049	0.180	0.143
0.3813	0.363	0.241	7.62E-05	0.466	0.236	0.231	0.122
0.3812	0.328	0.212	3.55E-04	0.393	0.203	0.191	0.116
0.3808	0.174	0.002	2.43E-16	0.186	0.001	0.184	0.172
0.3806	0.229	0.049	3.36E-14	0.243	0.047	0.196	0.180
0.3805	0.169	0.001	1.28E-12	0.177	0.001	0.176	0.168
0.3805	0.179	0.000	6.46E-14	0.193	0.000	0.193	0.179
0.3803	0.219	0.021	2.64E-12	0.209	0.019	0.190	0.198
0.3801	0.343	0.229	5.58E-09	0.464	0.243	0.221	0.114
0.3799	0.274	0.121	1.05E-07	0.335	0.120	0.215	0.153
0.3795	0.259	0.101	2.10E-04	0.290	0.091	0.199	0.158
0.3794	0.264	0.094	5.42E-08	0.274	0.087	0.188	0.170
0.3792	0.204	0.013	3.36E-06	0.221	0.020	0.201	0.191
0.3787	0.338	0.252	7.74E-11	0.467	0.267	0.200	0.086
0.3784	0.299	0.172	4.81E-05	0.350	0.168	0.182	0.127
0.3782	0.811	0.649	5.92E-12	1.897	1.365	0.531	0.162
0.3779	0.294	0.172	2.97E-03	0.340	0.162	0.178	0.122
0.3776	0.244	0.113	3.66E-06	0.273	0.101	0.172	0.131
0.3759	0.284	0.171	2.37E-04	0.339	0.158	0.181	0.113
0.3759	0.274	0.101	1.25E-11	0.295	0.094	0.201	0.173
0.3755	0.244	0.113	3.01E-04	0.266	0.102	0.164	0.131
0.3748	0.169	0.001	2.21E-14	0.170	0.001	0.169	0.168
0.3748	0.169	0.001	3.00E-16	0.168	0.000	0.168	0.168
0.3748	0.174	0.034	2.94E-08	0.202	0.029	0.172	0.140
0.3747	0.234	0.051	4.04E-14	0.239	0.046	0.193	0.183
0.3747	0.249	0.109	2.02E-09	0.292	0.103	0.189	0.140
0.3745	0.174	0.004	2.54E-17	0.198	0.004	0.194	0.170
0.3743	0.204	0.021	1.92E-17	0.209	0.020	0.190	0.183
0.3739	0.279	0.186	3.68E-04	0.333	0.177	0.156	0.093
0.3718	0.299	0.141	8.38E-09	0.368	0.136	0.232	0.158
0.3716	0.284	0.114	5.37E-05	0.322	0.108	0.214	0.170
0.3711	0.328	0.179	3.72E-05	0.376	0.169	0.207	0.149
0.3708	0.164	0.001	6.05E-11	0.177	0.001	0.176	0.163
0.3702	0.224	0.058	1.10E-12	0.243	0.054	0.189	0.166

0.3699	0.239	0.094	6.57E-14	0.304	0.140	0.164	0.145
0.3689	0.448	0.354	6.13E-06	0.610	0.390	0.220	0.094
0.3686	0.204	0.027	3.04E-09	0.215	0.032	0.184	0.177
0.3685	0.279	0.152	1.88E-03	0.314	0.144	0.170	0.127
0.3684	0.219	0.038	7.48E-06	0.251	0.051	0.200	0.181
0.3682	0.174	0.004	3.79E-14	0.179	0.004	0.175	0.170
0.3679	0.154	0.005	1.30E-18	0.174	0.004	0.170	0.149
0.3665	0.259	0.121	3.60E-04	0.310	0.118	0.192	0.138
0.3660	0.179	0.004	1.67E-11	0.184	0.004	0.180	0.175
0.3659	0.328	0.189	3.81E-03	0.431	0.215	0.217	0.139
0.3654	0.174	0.000	9.89E-10	0.197	0.000	0.197	0.174
0.3643	0.323	0.201	1.95E-09	0.395	0.195	0.200	0.122
0.3636	0.254	0.084	1.38E-06	0.285	0.081	0.204	0.170
0.3632	0.204	0.007	3.75E-18	0.199	0.006	0.193	0.197
0.3632	0.219	0.045	1.79E-09	0.239	0.042	0.197	0.174
0.3623	0.134	0.000	1.60E-15	0.154	0.000	0.153	0.134
0.3622	0.249	0.092	5.76E-12	0.274	0.082	0.191	0.157
0.3621	0.189	0.006	7.20E-13	0.199	0.006	0.193	0.183
0.3619	0.289	0.168	5.00E-05	0.322	0.165	0.157	0.121
0.3613	0.194	0.012	1.86E-15	0.187	0.010	0.178	0.182
0.3612	0.219	0.146	1.76E-06	0.255	0.133	0.122	0.073
0.3607	0.219	0.050	7.75E-03	0.246	0.045	0.201	0.169
0.3600	0.164	0.001	5.86E-11	0.175	0.001	0.174	0.163
0.3596	0.244	0.083	1.24E-06	0.257	0.074	0.183	0.161
0.3593	0.328	0.194	1.17E-06	0.378	0.191	0.187	0.134
0.3590	0.294	0.160	2.69E-08	0.328	0.154	0.174	0.134
0.3576	0.164	0.002	4.75E-11	0.176	0.001	0.174	0.162
0.3576	0.214	0.099	1.31E-11	0.243	0.091	0.153	0.115
0.3565	0.274	0.135	8.19E-06	0.311	0.127	0.184	0.139
0.3558	0.269	0.127	1.72E-07	0.302	0.114	0.188	0.142
0.3551	0.428	0.345	1.85E-08	0.600	0.368	0.232	0.083
0.3550	0.239	0.121	3.79E-07	0.273	0.112	0.161	0.118
0.3548	0.149	0.001	8.36E-14	0.168	0.001	0.167	0.148
0.3547	0.259	0.144	4.16E-07	0.303	0.137	0.166	0.115
0.3544	0.478	0.402	2.24E-05	0.814	0.537	0.277	0.076
0.3543	0.254	0.138	1.83E-04	0.301	0.132	0.169	0.116
0.3539	0.219	0.110	1.25E-07	0.241	0.099	0.142	0.109
0.3535	0.264	0.151	4.64E-07	0.307	0.144	0.163	0.113
0.3535	0.950	0.917	1.06E-03	3.053	2.530	0.523	0.033
0.3534	0.582	0.512	1.46E-05	0.939	0.661	0.278	0.070
0.3523	0.189	0.003	1.42E-11	0.180	0.002	0.177	0.186
0.3504	0.209	0.063	6.15E-10	0.217	0.055	0.162	0.146
0.3496	0.149	0.002	6.48E-12	0.165	0.003	0.163	0.147
0.3490	0.592	0.552	6.99E-09	1.082	0.765	0.318	0.040
0.3489	0.174	0.008	6.75E-08	0.186	0.007	0.179	0.166
0.3482	0.199	0.029	3.11E-14	0.201	0.025	0.176	0.170
0.3478	0.164	0.000	4.84E-13	0.170	0.000	0.169	0.164

0.3472	0.338	0.228	1.01E-08	0.426	0.232	0.194	0.110
0.3472	0.284	0.103	1.39E-08	0.309	0.102	0.207	0.181
0.3468	0.174	0.002	1.47E-11	0.173	0.002	0.171	0.172
0.3455	0.333	0.220	2.09E-04	0.415	0.223	0.192	0.113
0.3453	0.159	0.007	8.67E-14	0.170	0.007	0.163	0.152
0.3451	0.254	0.112	1.85E-06	0.292	0.110	0.182	0.142
0.3450	0.124	0.000	3.71E-12	0.133	0.000	0.133	0.124
0.3449	0.318	0.167	7.17E-07	0.378	0.162	0.215	0.151
0.3444	0.343	0.287	2.11E-03	0.442	0.300	0.142	0.056
0.3438	0.214	0.081	9.73E-06	0.249	0.081	0.168	0.133
0.3432	0.378	0.285	3.09E-06	0.508	0.305	0.203	0.093
0.3428	0.154	0.027	6.78E-12	0.162	0.023	0.138	0.127
0.3424	0.338	0.178	2.34E-06	0.379	0.171	0.208	0.160
0.3423	0.353	0.270	2.45E-07	0.507	0.296	0.211	0.083
0.3411	0.313	0.212	1.21E-04	0.383	0.201	0.181	0.101
0.3410	0.323	0.226	1.48E-03	0.420	0.238	0.182	0.097
0.3404	0.219	0.044	9.40E-16	0.217	0.038	0.179	0.175
0.3401	0.184	0.018	2.68E-11	0.190	0.017	0.173	0.166
0.3399	0.284	0.077	5.24E-06	0.322	0.081	0.241	0.207
0.3397	0.343	0.259	2.19E-08	0.447	0.261	0.186	0.084
0.3397	0.259	0.033	1.00E-19	0.383	0.085	0.298	0.226
0.3395	0.174	0.032	1.16E-06	0.175	0.034	0.141	0.142
0.3391	0.289	0.150	3.67E-05	0.326	0.146	0.180	0.139
0.3386	0.169	0.001	3.76E-14	0.156	0.001	0.155	0.168
0.3383	0.299	0.200	1.73E-03	0.348	0.196	0.152	0.099
0.3379	0.154	0.011	8.67E-05	0.162	0.015	0.147	0.143
0.3376	0.239	0.139	1.76E-07	0.280	0.137	0.143	0.100
0.3376	0.199	0.098	3.84E-19	0.244	0.079	0.165	0.101
0.3374	0.259	0.138	2.16E-06	0.304	0.132	0.172	0.121
0.3372	0.189	0.001	7.04E-15	0.178	0.001	0.177	0.188
0.3367	0.164	0.009	1.63E-04	0.179	0.014	0.166	0.155
0.3367	0.254	0.115	1.96E-05	0.278	0.107	0.171	0.139
0.3358	0.159	0.000	5.54E-09	0.158	0.000	0.157	0.159
0.3354	0.274	0.140	3.78E-05	0.315	0.142	0.173	0.134
0.3341	0.164	0.012	1.10E-07	0.170	0.011	0.158	0.152
0.3319	0.294	0.156	2.95E-06	0.340	0.166	0.173	0.138
0.3319	0.224	0.094	7.11E-03	0.231	0.080	0.150	0.130
0.3313	0.184	0.005	1.50E-08	0.181	0.004	0.177	0.179
0.3310	0.289	0.173	7.23E-04	0.340	0.172	0.168	0.116
0.3310	0.159	0.034	9.02E-11	0.169	0.029	0.140	0.125
0.3308	0.547	0.421	6.70E-05	0.984	0.609	0.375	0.126
0.3303	0.239	0.109	7.22E-03	0.257	0.102	0.155	0.130
0.3300	0.154	0.000	1.05E-16	0.158	0.000	0.157	0.154
0.3295	0.169	0.004	5.00E-10	0.166	0.003	0.163	0.165
0.3288	0.662	0.539	3.97E-07	1.188	0.782	0.405	0.123
0.3285	0.164	0.010	1.22E-10	0.169	0.008	0.161	0.154
0.3285	0.159	0.026	1.53E-03	0.171	0.026	0.144	0.133

0.3276	0.174	0.003	3.15E-15	0.161	0.003	0.158	0.171
0.3270	0.254	0.138	4.34E-12	0.274	0.128	0.146	0.116
0.3269	0.134	0.040	1.69E-07	0.157	0.035	0.121	0.094
0.3269	0.294	0.200	1.42E-06	0.361	0.193	0.168	0.094
0.3266	0.169	0.050	4.88E-06	0.166	0.042	0.124	0.119
0.3260	0.562	0.511	4.05E-04	0.903	0.660	0.243	0.051
0.3259	0.259	0.127	9.78E-03	0.289	0.119	0.170	0.132
0.3258	0.254	0.125	1.81E-07	0.277	0.116	0.160	0.129
0.3257	0.184	0.036	4.79E-04	0.193	0.034	0.160	0.148
0.3256	0.224	0.054	3.84E-07	0.254	0.068	0.186	0.170
0.3240	0.289	0.165	3.99E-04	0.315	0.163	0.152	0.124
0.3234	0.224	0.099	1.26E-04	0.243	0.093	0.150	0.125
0.3229	0.169	0.043	1.84E-04	0.156	0.034	0.122	0.126
0.3228	0.224	0.054	1.36E-10	0.219	0.048	0.171	0.170
0.3227	0.159	0.008	9.88E-07	0.159	0.006	0.153	0.151
0.3217	0.149	0.024	8.38E-07	0.167	0.020	0.146	0.125
0.3214	0.279	0.211	1.47E-04	0.318	0.202	0.116	0.068
0.3202	0.318	0.184	3.17E-07	0.433	0.210	0.223	0.134
0.3182	0.164	0.007	1.58E-10	0.162	0.006	0.156	0.157
0.3180	0.338	0.209	3.38E-03	0.378	0.202	0.175	0.129
0.3180	0.204	0.048	1.76E-07	0.216	0.045	0.171	0.156
0.3178	0.154	0.001	9.35E-15	0.162	0.001	0.161	0.153
0.3177	0.229	0.109	2.26E-07	0.257	0.103	0.154	0.120
0.3176	0.313	0.204	2.92E-03	0.374	0.210	0.165	0.109
0.3174	0.274	0.128	5.69E-05	0.297	0.121	0.176	0.146
0.3168	0.229	0.079	1.43E-09	0.240	0.073	0.167	0.150
0.3160	0.338	0.250	3.92E-03	0.420	0.258	0.162	0.088
0.3160	0.154	0.000	1.09E-09	0.140	0.000	0.139	0.154
0.3155	0.164	0.014	1.71E-06	0.164	0.012	0.152	0.150
0.3143	0.557	0.518	1.61E-03	0.945	0.679	0.265	0.039
0.3142	0.279	0.141	8.39E-15	0.285	0.132	0.153	0.138
0.3142	0.284	0.179	5.14E-03	0.316	0.170	0.146	0.105
0.3139	0.224	0.138	3.22E-07	0.251	0.124	0.128	0.086
0.3136	0.199	0.052	7.56E-07	0.217	0.048	0.169	0.147
0.3135	0.129	0.001	1.37E-12	0.138	0.001	0.137	0.128
0.3126	0.169	0.058	1.95E-15	0.185	0.053	0.132	0.111
0.3125	0.144	0.001	2.21E-10	0.146	0.001	0.145	0.143
0.3124	0.194	0.078	1.96E-05	0.191	0.069	0.122	0.116
0.3122	0.229	0.085	5.45E-07	0.252	0.078	0.174	0.144
0.3120	0.189	0.000	3.49E-12	0.181	0.000	0.181	0.189
0.3113	0.234	0.165	2.86E-05	0.278	0.153	0.125	0.069
0.3111	0.159	0.030	1.70E-07	0.173	0.030	0.142	0.129
0.3108	0.214	0.104	7.56E-04	0.226	0.097	0.129	0.110
0.3107	0.169	0.001	1.72E-07	0.162	0.001	0.161	0.168
0.3098	0.144	0.018	5.67E-07	0.154	0.016	0.137	0.126
0.3091	0.259	0.139	5.18E-03	0.295	0.134	0.161	0.120
0.3084	0.318	0.201	1.16E-03	0.357	0.198	0.159	0.117

0.3083	0.229	0.078	9.75E-07	0.240	0.074	0.166	0.151
0.3081	0.174	0.014	9.70E-08	0.171	0.012	0.159	0.160
0.3078	0.239	0.089	1.29E-07	0.253	0.089	0.164	0.150
0.3078	0.478	0.425	4.54E-05	0.782	0.569	0.213	0.053
0.3077	0.209	0.099	3.65E-07	0.216	0.093	0.123	0.110
0.3072	0.189	0.007	1.79E-05	0.181	0.010	0.171	0.182
0.3066	0.149	0.019	1.89E-04	0.153	0.019	0.134	0.130
0.3059	0.279	0.191	1.04E-05	0.332	0.184	0.148	0.088
0.3049	0.154	0.001	7.92E-11	0.146	0.001	0.145	0.153
0.3043	0.328	0.269	8.43E-03	0.411	0.280	0.131	0.059
0.3037	0.403	0.401	8.42E-07	0.575	0.445	0.130	0.002
0.3032	0.269	0.171	6.56E-04	0.310	0.160	0.150	0.098
0.3024	0.144	0.007	5.85E-16	0.138	0.006	0.131	0.137
0.3023	0.224	0.078	2.94E-07	0.226	0.072	0.154	0.146
0.3022	0.299	0.217	6.75E-04	0.345	0.205	0.140	0.082
0.3022	0.687	0.657	1.11E-05	1.238	0.978	0.260	0.030
0.3022	0.134	0.026	7.90E-08	0.145	0.022	0.122	0.108
0.3017	0.488	0.487	7.24E-03	0.764	0.597	0.167	0.001
0.3016	0.219	0.096	1.98E-06	0.234	0.092	0.142	0.123
0.3014	0.209	0.079	7.00E-04	0.219	0.069	0.151	0.130
0.3013	0.343	0.230	5.86E-03	0.403	0.233	0.171	0.113
0.3009	0.169	0.107	4.03E-05	0.207	0.099	0.107	0.062
0.3008	0.179	0.059	1.31E-03	0.181	0.052	0.129	0.120
0.3008	0.134	0.000	1.86E-07	0.129	0.000	0.129	0.134
0.3008	0.219	0.080	9.99E-05	0.224	0.076	0.148	0.139
0.3006	0.149	0.001	5.70E-11	0.143	0.001	0.143	0.148
0.3002	0.269	0.129	1.57E-04	0.284	0.121	0.164	0.140
0.2998	0.274	0.176	1.91E-04	0.319	0.166	0.153	0.098
0.2993	0.199	0.047	1.06E-07	0.193	0.042	0.151	0.152
0.2990	0.149	0.028	1.62E-08	0.155	0.024	0.132	0.121
0.2977	0.234	0.101	1.21E-03	0.277	0.116	0.161	0.133
0.2976	0.473	0.538	8.36E-11	0.802	0.694	0.108	-0.065
0.2975	0.174	0.042	1.04E-07	0.161	0.039	0.122	0.132
0.2974	0.224	0.098	1.72E-06	0.236	0.092	0.144	0.126
0.2973	0.149	0.012	3.65E-07	0.158	0.012	0.145	0.137
0.2971	0.129	0.017	4.45E-05	0.142	0.015	0.127	0.112
0.2967	0.214	0.097	5.23E-06	0.226	0.086	0.139	0.117
0.2956	0.647	0.647	9.76E-03	1.173	0.962	0.211	0.000
0.2954	0.239	0.144	8.80E-06	0.244	0.133	0.111	0.095
0.2954	0.249	0.139	1.66E-03	0.275	0.127	0.148	0.110
0.2943	0.169	0.021	1.96E-07	0.154	0.017	0.137	0.148
0.2936	0.159	0.003	1.86E-11	0.150	0.004	0.146	0.156
0.2933	0.995	0.965	1.07E-14	3.799	3.426	0.373	0.030
0.2933	0.169	0.000	2.42E-10	0.153	0.000	0.153	0.169
0.2927	0.209	0.098	3.74E-04	0.216	0.087	0.129	0.111
0.2924	0.184	0.032	1.16E-04	0.184	0.043	0.141	0.152
0.2922	0.149	0.028	3.07E-05	0.153	0.023	0.130	0.121

0.2920	0.244	0.145	2.20E-03	0.275	0.139	0.136	0.099
0.2916	0.279	0.261	1.13E-07	0.368	0.258	0.110	0.018
0.2905	0.154	0.008	4.36E-05	0.158	0.010	0.148	0.146
0.2901	0.383	0.333	9.75E-04	0.523	0.363	0.160	0.050
0.2899	0.174	0.053	2.68E-07	0.187	0.049	0.138	0.121
0.2897	0.224	0.104	2.08E-07	0.268	0.123	0.145	0.120
0.2892	0.159	0.001	1.03E-09	0.144	0.001	0.143	0.158
0.2891	0.766	0.614	3.27E-09	1.823	1.182	0.642	0.152
0.2891	0.114	0.000	5.90E-06	0.114	0.000	0.114	0.114
0.2886	0.139	0.000	5.03E-12	0.128	0.000	0.128	0.139
0.2885	0.323	0.258	1.02E-05	0.423	0.281	0.142	0.065
0.2884	0.164	0.017	1.53E-12	0.153	0.014	0.139	0.147
0.2881	0.443	0.428	5.30E-04	0.669	0.522	0.147	0.015
0.2877	0.159	0.031	1.50E-03	0.174	0.027	0.147	0.128
0.2872	0.234	0.077	5.24E-05	0.305	0.101	0.204	0.157
0.2867	0.388	0.317	7.03E-03	0.521	0.364	0.157	0.071
0.2865	0.552	0.515	4.41E-05	0.869	0.648	0.221	0.037
0.2861	0.333	0.278	1.16E-05	0.425	0.275	0.150	0.055
0.2852	0.393	0.238	1.41E-03	0.477	0.272	0.205	0.155
0.2851	0.174	0.085	2.16E-04	0.167	0.074	0.093	0.089
0.2846	0.184	0.074	1.54E-04	0.186	0.067	0.119	0.110
0.2846	0.234	0.126	2.65E-09	0.256	0.119	0.137	0.108
0.2845	0.144	0.001	5.34E-11	0.142	0.001	0.141	0.143
0.2842	0.950	0.904	5.13E-12	2.807	2.407	0.400	0.046
0.2841	0.129	0.002	5.61E-09	0.117	0.002	0.115	0.127
0.2840	0.378	0.261	8.89E-04	0.552	0.330	0.221	0.117
0.2837	0.184	0.068	1.67E-04	0.171	0.059	0.112	0.116
0.2819	0.179	0.023	7.73E-07	0.175	0.022	0.152	0.156
0.2814	0.149	0.032	8.99E-04	0.143	0.028	0.115	0.117
0.2810	0.144	0.000	2.72E-10	0.150	0.000	0.150	0.144
0.2808	0.204	0.069	3.42E-05	0.208	0.067	0.141	0.135
0.2805	0.458	0.414	7.57E-03	0.643	0.467	0.176	0.044
0.2802	0.144	0.015	2.23E-03	0.135	0.013	0.123	0.129
0.2802	0.159	0.000	3.11E-14	0.146	0.000	0.146	0.159
0.2798	0.333	0.166	2.73E-05	0.454	0.229	0.225	0.167
0.2797	0.184	0.102	8.44E-03	0.217	0.102	0.115	0.082
0.2795	0.438	0.528	1.77E-03	0.787	0.700	0.086	-0.090
0.2794	0.229	0.109	8.28E-03	0.237	0.101	0.136	0.120
0.2793	0.149	0.001	3.52E-06	0.144	0.001	0.144	0.148
0.2792	0.149	0.001	1.62E-12	0.142	0.001	0.142	0.148
0.2786	0.129	0.040	8.15E-04	0.134	0.036	0.098	0.089
0.2773	0.343	0.276	1.01E-07	0.506	0.341	0.165	0.067
0.2766	0.144	0.006	5.78E-08	0.127	0.005	0.122	0.138
0.2764	0.134	0.013	1.72E-08	0.136	0.012	0.124	0.121
0.2758	0.139	0.004	4.06E-06	0.125	0.003	0.122	0.135
0.2758	0.348	0.219	5.33E-06	0.452	0.262	0.190	0.129
0.2752	0.189	0.039	2.25E-03	0.186	0.037	0.149	0.150

0.2746	0.159	0.003	1.10E-09	0.128	0.003	0.126	0.156
0.2746	0.254	0.151	4.05E-05	0.256	0.138	0.118	0.103
0.2744	0.234	0.140	7.41E-06	0.245	0.131	0.114	0.094
0.2744	0.229	0.158	1.85E-04	0.251	0.144	0.107	0.071
0.2744	0.159	0.000	2.22E-12	0.147	0.000	0.146	0.159
0.2740	0.154	0.016	8.96E-09	0.144	0.014	0.130	0.138
0.2737	0.214	0.084	2.53E-04	0.223	0.077	0.145	0.130
0.2736	0.124	0.009	1.99E-10	0.127	0.007	0.120	0.115
0.2730	0.249	0.139	6.55E-04	0.282	0.124	0.158	0.110
0.2722	0.194	0.093	4.49E-03	0.196	0.086	0.110	0.101
0.2716	0.254	0.093	9.94E-04	0.282	0.108	0.174	0.161
0.2712	0.134	0.024	8.90E-04	0.143	0.023	0.120	0.110
0.2708	0.149	0.006	4.79E-09	0.140	0.005	0.135	0.143
0.2707	0.234	0.091	2.09E-06	0.233	0.082	0.151	0.143
0.2697	0.204	0.086	1.10E-03	0.214	0.077	0.137	0.118
0.2692	0.164	0.000	1.24E-11	0.143	0.000	0.143	0.164
0.2692	0.299	0.215	3.93E-04	0.367	0.221	0.146	0.084
0.2684	0.463	0.419	3.63E-03	0.648	0.486	0.162	0.044
0.2680	0.189	0.056	2.07E-03	0.170	0.055	0.115	0.133
0.2679	0.154	0.009	1.41E-09	0.140	0.007	0.132	0.145
0.2678	0.124	0.005	1.02E-08	0.113	0.004	0.109	0.119
0.2678	0.214	0.094	4.73E-03	0.234	0.097	0.137	0.120
0.2674	0.179	0.070	6.27E-03	0.175	0.060	0.115	0.109
0.2665	0.826	0.784	9.78E-03	1.868	1.596	0.271	0.042
0.2661	0.129	0.002	1.16E-05	0.124	0.003	0.121	0.127
0.2658	0.164	0.086	1.80E-04	0.169	0.078	0.091	0.078
0.2652	0.144	0.002	4.81E-09	0.121	0.002	0.119	0.142
0.2651	0.154	0.000	2.42E-08	0.137	0.000	0.137	0.154
0.2646	0.174	0.034	3.52E-04	0.159	0.031	0.128	0.140
0.2641	0.164	0.012	1.22E-04	0.148	0.012	0.136	0.152
0.2641	0.134	0.011	1.29E-03	0.126	0.011	0.116	0.123
0.2639	0.706	0.752	2.45E-04	1.510	1.298	0.212	-0.046
0.2638	0.264	0.156	2.09E-03	0.281	0.147	0.135	0.108
0.2638	0.139	0.007	8.49E-06	0.127	0.006	0.121	0.132
0.2636	0.199	0.059	1.93E-07	0.192	0.052	0.140	0.140
0.2632	0.184	0.052	2.64E-07	0.171	0.046	0.126	0.132
0.2630	0.269	0.148	3.25E-03	0.275	0.135	0.140	0.121
0.2628	0.159	0.028	1.06E-07	0.158	0.027	0.131	0.131
0.2608	0.353	0.189	6.28E-05	0.460	0.248	0.211	0.164
0.2607	0.144	0.002	1.69E-08	0.132	0.002	0.131	0.142
0.2607	0.169	0.004	1.65E-07	0.145	0.003	0.142	0.165
0.2604	0.199	0.107	3.73E-08	0.213	0.097	0.116	0.092
0.2603	0.179	0.021	4.28E-05	0.161	0.018	0.142	0.158
0.2603	0.144	0.007	9.39E-03	0.121	0.006	0.115	0.137
0.2602	0.204	0.092	3.01E-03	0.203	0.083	0.120	0.112
0.2600	0.214	0.067	1.90E-03	0.200	0.058	0.142	0.147
0.2599	0.239	0.131	2.82E-04	0.256	0.124	0.132	0.108

0.2594	0.129	0.005	7.72E-04	0.127	0.004	0.123	0.124
0.2585	0.303	0.185	5.20E-07	0.336	0.190	0.147	0.118
0.2585	0.119	0.004	1.50E-07	0.120	0.005	0.115	0.115
0.2584	0.169	0.039	2.14E-09	0.164	0.036	0.128	0.130
0.2581	0.154	0.001	1.20E-11	0.140	0.001	0.138	0.153
0.2579	0.159	0.087	6.75E-04	0.165	0.077	0.088	0.072
0.2578	0.259	0.165	2.82E-04	0.300	0.167	0.132	0.094
0.2577	0.388	0.364	2.88E-04	0.534	0.410	0.124	0.024
0.2575	0.164	0.088	1.57E-04	0.179	0.077	0.102	0.076
0.2573	0.169	0.038	5.16E-04	0.194	0.048	0.146	0.131
0.2571	0.249	0.168	8.16E-03	0.278	0.169	0.109	0.081
0.2567	0.244	0.196	9.69E-04	0.280	0.193	0.087	0.048
0.2566	0.129	0.009	2.47E-03	0.122	0.008	0.114	0.120
0.2566	0.423	0.390	4.66E-09	0.628	0.474	0.154	0.033
0.2565	0.119	0.002	1.57E-07	0.123	0.002	0.121	0.117
0.2561	0.184	0.094	1.45E-06	0.188	0.085	0.103	0.090
0.2560	0.303	0.234	8.09E-03	0.338	0.227	0.111	0.069
0.2559	0.229	0.184	1.49E-03	0.261	0.181	0.080	0.045
0.2558	0.383	0.336	2.47E-04	0.505	0.370	0.135	0.047
0.2537	0.109	0.049	4.94E-04	0.126	0.044	0.081	0.060
0.2533	0.144	0.000	6.71E-05	0.130	0.000	0.130	0.144
0.2530	0.224	0.080	4.24E-05	0.217	0.070	0.147	0.144
0.2530	0.164	0.027	1.10E-07	0.153	0.024	0.129	0.137
0.2529	0.139	0.022	6.25E-04	0.136	0.018	0.118	0.117
0.2528	0.139	0.005	1.01E-10	0.127	0.004	0.123	0.134
0.2523	0.164	0.083	1.06E-10	0.170	0.072	0.098	0.081
0.2513	0.229	0.114	9.40E-04	0.231	0.107	0.123	0.115
0.2512	0.129	0.000	7.07E-10	0.125	0.000	0.125	0.129
0.2501	0.144	0.002	5.90E-08	0.116	0.002	0.115	0.142
-0.2505	0.164	0.274	1.77E-04	0.125	0.287	0.161	-0.110
-0.2510	0.065	0.164	1.96E-06	0.066	0.182	0.117	-0.099
-0.2511	0.075	0.189	9.87E-10	0.061	0.199	0.138	-0.114
-0.2512	0.045	0.109	4.45E-11	0.035	0.137	0.103	-0.064
-0.2525	0.149	0.171	2.68E-05	0.111	0.222	0.111	-0.022
-0.2531	0.055	0.167	6.44E-16	0.046	0.194	0.148	-0.112
-0.2551	0.020	0.186	9.54E-06	0.025	0.190	0.165	-0.166
-0.2552	0.915	0.756	1.09E-13	2.934	2.768	0.165	0.159
-0.2560	0.005	0.116	2.73E-21	0.004	0.130	0.126	-0.111
-0.2561	0.015	0.105	6.81E-30	0.036	0.156	0.120	-0.090
-0.2570	0.095	0.178	1.36E-04	0.072	0.199	0.127	-0.083
-0.2592	0.090	0.148	1.76E-07	0.088	0.190	0.102	-0.058
-0.2592	0.100	0.224	4.64E-04	0.072	0.232	0.160	-0.124
-0.2604	0.204	0.187	2.83E-03	0.242	0.319	0.077	0.017
-0.2616	0.030	0.110	8.84E-16	0.030	0.140	0.109	-0.080
-0.2620	0.015	0.121	8.53E-03	0.012	0.141	0.129	-0.106
-0.2620	0.119	0.225	3.35E-04	0.096	0.251	0.154	-0.106
-0.2626	0.090	0.160	1.94E-04	0.076	0.192	0.117	-0.070

-0.2630	0.060	0.147	1.63E-07	0.043	0.160	0.117	-0.087
-0.2650	0.055	0.191	4.81E-05	0.055	0.201	0.146	-0.136
-0.2660	0.030	0.143	2.54E-12	0.042	0.183	0.141	-0.113
-0.2667	0.050	0.140	4.97E-17	0.050	0.171	0.121	-0.090
-0.2672	0.000	0.125	2.68E-05	0.000	0.139	0.139	-0.125
-0.2673	0.055	0.191	2.30E-05	0.045	0.199	0.154	-0.136
-0.2674	0.070	0.234	6.39E-05	0.062	0.233	0.171	-0.164
-0.2690	0.075	0.193	1.56E-03	0.040	0.191	0.152	-0.118
-0.2695	0.045	0.148	6.85E-26	0.041	0.177	0.135	-0.103
-0.2702	0.055	0.191	2.57E-13	0.050	0.201	0.151	-0.136
-0.2720	0.085	0.204	1.26E-08	0.084	0.231	0.147	-0.119
-0.2723	0.070	0.137	2.69E-06	0.046	0.164	0.118	-0.067
-0.2730	0.085	0.171	2.16E-05	0.044	0.179	0.134	-0.086
-0.2735	0.040	0.136	1.12E-14	0.038	0.161	0.123	-0.096
-0.2737	0.100	0.137	8.46E-03	0.069	0.168	0.099	-0.037
-0.2739	0.010	0.114	7.18E-18	0.011	0.131	0.120	-0.104
-0.2741	0.085	0.196	9.30E-14	0.066	0.234	0.169	-0.111
-0.2747	0.020	0.181	1.16E-04	0.013	0.175	0.163	-0.161
-0.2751	0.065	0.190	7.40E-04	0.051	0.200	0.149	-0.125
-0.2757	0.005	0.117	1.40E-19	0.002	0.137	0.136	-0.112
-0.2762	0.806	0.708	3.44E-08	2.165	2.255	0.090	0.098
-0.2763	0.090	0.126	3.33E-12	0.067	0.177	0.111	-0.036
-0.2764	0.020	0.191	8.16E-03	0.014	0.183	0.169	-0.171
-0.2765	0.109	0.220	8.06E-03	0.077	0.228	0.151	-0.111
-0.2768	0.020	0.173	1.29E-04	0.015	0.170	0.155	-0.153
-0.2784	0.085	0.203	7.74E-05	0.067	0.213	0.146	-0.118
-0.2788	0.040	0.177	3.10E-04	0.036	0.187	0.151	-0.137
-0.2790	0.124	0.246	3.95E-05	0.105	0.274	0.169	-0.122
-0.2791	0.000	0.110	8.84E-15	0.000	0.133	0.133	-0.110
-0.2798	0.000	0.105	1.00E-04	0.000	0.133	0.133	-0.105
-0.2803	0.080	0.140	5.66E-08	0.074	0.185	0.112	-0.060
-0.2805	0.080	0.136	6.78E-10	0.063	0.174	0.112	-0.056
-0.2811	0.085	0.103	6.10E-06	0.080	0.128	0.048	-0.018
-0.2814	0.090	0.159	2.70E-11	0.073	0.204	0.131	-0.069
-0.2822	0.169	0.244	4.94E-03	0.159	0.305	0.145	-0.075
-0.2833	0.020	0.109	2.65E-13	0.036	0.164	0.128	-0.089
-0.2840	0.045	0.202	2.68E-03	0.036	0.201	0.166	-0.157
-0.2852	0.050	0.166	4.20E-04	0.022	0.173	0.150	-0.116
-0.2853	0.065	0.144	2.40E-13	0.062	0.194	0.132	-0.079
-0.2856	0.124	0.144	1.11E-07	0.095	0.201	0.106	-0.020
-0.2858	0.040	0.133	8.45E-07	0.038	0.165	0.127	-0.093
-0.2870	0.199	0.323	2.32E-05	0.247	0.458	0.211	-0.124
-0.2870	0.095	0.134	3.80E-06	0.073	0.182	0.110	-0.039
-0.2872	0.030	0.120	1.76E-35	0.041	0.175	0.135	-0.090
-0.2874	0.040	0.159	5.98E-04	0.034	0.164	0.130	-0.119
-0.2879	0.000	0.128	1.04E-07	0.000	0.147	0.147	-0.128
-0.2879	0.085	0.153	1.60E-09	0.056	0.184	0.129	-0.068

-0.2896	0.025	0.140	1.34E-18	0.030	0.164	0.134	-0.115
-0.2911	0.050	0.122	4.06E-23	0.051	0.175	0.123	-0.072
-0.2911	0.035	0.178	1.37E-04	0.030	0.188	0.159	-0.143
-0.2914	0.806	0.710	2.05E-13	2.083	2.197	0.114	0.096
-0.2925	0.030	0.102	2.14E-20	0.020	0.138	0.119	-0.072
-0.2930	0.124	0.244	2.42E-06	0.097	0.266	0.169	-0.120
-0.2948	0.010	0.129	1.99E-10	0.008	0.145	0.137	-0.119
-0.2962	0.109	0.224	4.26E-07	0.102	0.263	0.162	-0.115
-0.2966	0.085	0.183	5.73E-04	0.064	0.201	0.137	-0.098
-0.2967	0.070	0.223	2.39E-03	0.048	0.223	0.174	-0.153
-0.2975	0.060	0.185	2.18E-13	0.057	0.205	0.149	-0.125
-0.2978	0.045	0.166	6.95E-16	0.049	0.194	0.144	-0.121
-0.2982	0.065	0.251	5.04E-04	0.057	0.258	0.200	-0.186
-0.2983	0.045	0.186	7.21E-14	0.035	0.196	0.162	-0.141
-0.2986	0.080	0.220	1.44E-09	0.064	0.253	0.189	-0.140
-0.2996	0.060	0.133	5.12E-09	0.044	0.171	0.127	-0.073
-0.3020	0.104	0.164	1.09E-10	0.082	0.207	0.125	-0.060
-0.3021	0.085	0.239	2.07E-11	0.085	0.273	0.188	-0.154
-0.3022	0.020	0.178	4.49E-05	0.019	0.184	0.166	-0.158
-0.3029	0.935	0.756	1.63E-19	3.176	2.955	0.221	0.179
-0.3044	0.000	0.117	1.55E-03	0.000	0.147	0.147	-0.117
-0.3052	0.090	0.138	2.12E-06	0.097	0.219	0.123	-0.048
-0.3055	0.134	0.310	4.33E-05	0.140	0.354	0.214	-0.176
-0.3056	0.055	0.161	1.65E-12	0.052	0.195	0.143	-0.106
-0.3057	0.070	0.245	1.34E-03	0.076	0.265	0.189	-0.175
-0.3060	0.174	0.342	1.47E-06	0.189	0.428	0.239	-0.168
-0.3072	0.085	0.205	2.09E-11	0.065	0.234	0.169	-0.120
-0.3075	0.333	0.384	2.96E-03	0.406	0.542	0.135	-0.051
-0.3079	0.900	0.751	6.49E-17	2.748	2.699	0.049	0.149
-0.3079	0.060	0.205	2.10E-03	0.036	0.209	0.173	-0.145
-0.3081	0.060	0.159	7.12E-10	0.033	0.178	0.144	-0.099
-0.3090	0.025	0.178	3.06E-11	0.026	0.194	0.168	-0.153
-0.3091	0.045	0.213	7.86E-09	0.054	0.231	0.177	-0.168
-0.3093	0.169	0.324	1.45E-03	0.172	0.372	0.200	-0.155
-0.3094	0.060	0.226	1.22E-03	0.055	0.239	0.185	-0.166
-0.3109	0.000	0.105	5.54E-04	0.000	0.143	0.143	-0.105
-0.3147	0.104	0.280	2.70E-05	0.100	0.305	0.205	-0.176
-0.3153	0.100	0.318	2.15E-07	0.107	0.344	0.237	-0.218
-0.3168	0.050	0.232	1.03E-05	0.047	0.235	0.187	-0.182
-0.3170	0.005	0.136	5.33E-46	0.015	0.179	0.165	-0.131
-0.3174	0.189	0.363	3.42E-04	0.195	0.421	0.226	-0.174
-0.3187	0.040	0.228	6.43E-04	0.035	0.225	0.190	-0.188
-0.3196	0.119	0.250	2.14E-05	0.074	0.263	0.189	-0.131
-0.3198	0.030	0.236	1.44E-04	0.038	0.244	0.206	-0.206
-0.3212	0.100	0.192	9.48E-08	0.087	0.241	0.154	-0.092
-0.3213	0.169	0.201	8.99E-03	0.186	0.314	0.128	-0.032
-0.3216	0.080	0.271	2.48E-08	0.078	0.277	0.199	-0.191

-0.3233	0.055	0.115	8.13E-07	0.097	0.233	0.136	-0.060
-0.3236	0.910	0.731	3.81E-26	3.097	2.945	0.152	0.179
-0.3245	0.100	0.179	5.00E-06	0.098	0.248	0.151	-0.079
-0.3261	0.050	0.247	1.45E-08	0.054	0.258	0.204	-0.197
-0.3265	0.896	0.740	1.21E-31	2.566	2.533	0.033	0.156
-0.3266	0.015	0.141	1.28E-05	0.006	0.163	0.157	-0.126
-0.3293	0.075	0.143	1.57E-10	0.060	0.195	0.134	-0.068
-0.3304	0.090	0.138	7.23E-07	0.101	0.233	0.132	-0.048
-0.3321	0.114	0.228	1.43E-04	0.110	0.274	0.164	-0.114
-0.3322	0.692	0.810	9.88E-04	1.371	1.742	0.371	-0.118
-0.3327	0.060	0.182	2.55E-03	0.048	0.212	0.164	-0.122
-0.3330	0.100	0.229	8.78E-04	0.069	0.251	0.181	-0.129
-0.3331	0.159	0.251	1.09E-04	0.155	0.340	0.186	-0.092
-0.3340	0.139	0.268	1.79E-03	0.102	0.296	0.194	-0.129
-0.3341	0.204	0.339	4.03E-06	0.233	0.423	0.190	-0.135
-0.3342	0.129	0.283	1.97E-03	0.112	0.313	0.201	-0.154
-0.3356	0.075	0.187	3.66E-03	0.065	0.204	0.139	-0.112
-0.3374	0.095	0.293	5.48E-04	0.087	0.307	0.220	-0.198
-0.3383	0.861	0.916	2.10E-07	2.180	2.499	0.319	-0.055
-0.3386	0.085	0.286	6.54E-08	0.089	0.300	0.211	-0.201
-0.3389	0.005	0.108	1.22E-07	0.001	0.142	0.141	-0.103
-0.3389	0.060	0.211	2.49E-04	0.070	0.255	0.184	-0.151
-0.3390	0.085	0.287	4.04E-05	0.091	0.310	0.219	-0.202
-0.3393	0.085	0.142	7.95E-10	0.062	0.190	0.129	-0.057
-0.3403	0.114	0.212	3.77E-07	0.087	0.258	0.170	-0.098
-0.3407	0.085	0.130	9.01E-08	0.057	0.182	0.125	-0.045
-0.3408	0.104	0.303	1.67E-11	0.104	0.333	0.229	-0.199
-0.3409	0.085	0.224	4.16E-08	0.055	0.243	0.188	-0.139
-0.3414	0.045	0.114	1.28E-13	0.085	0.233	0.148	-0.069
-0.3419	0.095	0.110	8.81E-10	0.056	0.166	0.110	-0.015
-0.3426	0.065	0.221	4.44E-11	0.055	0.242	0.187	-0.156
-0.3443	0.109	0.177	2.30E-10	0.106	0.254	0.147	-0.068
-0.3444	0.075	0.231	3.17E-10	0.039	0.238	0.199	-0.156
-0.3455	0.100	0.285	1.52E-05	0.069	0.302	0.233	-0.185
-0.3459	0.129	0.296	4.75E-03	0.124	0.337	0.213	-0.167
-0.3460	0.030	0.181	2.35E-05	0.031	0.212	0.181	-0.151
-0.3462	0.124	0.321	3.31E-10	0.132	0.385	0.253	-0.197
-0.3468	0.090	0.303	1.67E-04	0.091	0.320	0.228	-0.213
-0.3472	0.025	0.113	2.96E-12	0.026	0.161	0.135	-0.088
-0.3473	0.035	0.106	6.21E-06	0.081	0.219	0.138	-0.071
-0.3483	0.040	0.114	2.77E-20	0.013	0.150	0.136	-0.074
-0.3490	0.070	0.220	3.85E-12	0.041	0.235	0.193	-0.150
-0.3496	0.114	0.146	3.22E-08	0.102	0.244	0.141	-0.032
-0.3511	0.060	0.288	2.27E-03	0.071	0.307	0.236	-0.228
-0.3511	0.065	0.238	2.89E-12	0.075	0.271	0.196	-0.173
-0.3515	0.861	0.753	7.32E-20	2.444	2.578	0.133	0.108
-0.3517	0.045	0.237	1.10E-13	0.043	0.246	0.203	-0.192

-0.3528	0.114	0.255	3.18E-04	0.086	0.279	0.193	-0.141
-0.3529	0.015	0.139	5.94E-38	0.025	0.188	0.163	-0.124
-0.3532	0.040	0.177	1.81E-15	0.017	0.194	0.176	-0.137
-0.3537	0.070	0.139	4.97E-08	0.040	0.187	0.148	-0.069
-0.3547	0.871	0.724	7.36E-23	2.468	2.472	0.004	0.147
-0.3564	0.124	0.167	1.52E-06	0.146	0.295	0.149	-0.043
-0.3564	0.164	0.263	3.63E-03	0.148	0.325	0.178	-0.099
-0.3574	0.095	0.219	4.45E-08	0.073	0.257	0.184	-0.124
-0.3577	0.806	0.697	4.52E-15	2.264	2.404	0.140	0.109
-0.3578	0.134	0.184	9.00E-03	0.143	0.269	0.126	-0.050
-0.3581	0.915	0.866	3.33E-04	2.724	2.964	0.239	0.049
-0.3594	0.179	0.241	1.07E-04	0.162	0.302	0.140	-0.062
-0.3606	0.095	0.207	1.21E-11	0.100	0.286	0.186	-0.112
-0.3608	0.055	0.268	6.00E-04	0.047	0.273	0.227	-0.213
-0.3621	0.184	0.385	2.16E-04	0.179	0.442	0.263	-0.201
-0.3622	0.154	0.346	4.91E-07	0.148	0.409	0.261	-0.192
-0.3622	0.493	0.522	4.66E-04	0.836	0.947	0.111	-0.029
-0.3633	0.144	0.243	5.46E-05	0.115	0.287	0.171	-0.099
-0.3649	0.005	0.121	1.47E-06	0.004	0.161	0.158	-0.116
-0.3650	0.075	0.207	2.73E-16	0.069	0.263	0.195	-0.132
-0.3670	0.169	0.354	4.11E-06	0.139	0.393	0.255	-0.185
-0.3673	0.090	0.297	1.34E-03	0.094	0.335	0.240	-0.207
-0.3673	0.025	0.195	3.77E-07	0.016	0.209	0.193	-0.170
-0.3681	0.030	0.118	9.83E-28	0.053	0.215	0.162	-0.088
-0.3683	0.224	0.378	1.41E-03	0.219	0.472	0.252	-0.154
-0.3685	0.139	0.228	1.13E-04	0.085	0.260	0.175	-0.089
-0.3693	0.015	0.126	3.02E-29	0.016	0.175	0.159	-0.111
-0.3704	0.045	0.102	1.64E-14	0.053	0.181	0.127	-0.057
-0.3718	0.025	0.198	7.80E-04	0.022	0.222	0.200	-0.173
-0.3721	0.114	0.258	9.88E-09	0.128	0.328	0.201	-0.144
-0.3723	0.129	0.179	9.21E-09	0.171	0.312	0.141	-0.050
-0.3747	0.015	0.222	8.51E-11	0.011	0.229	0.219	-0.207
-0.3759	0.144	0.255	2.35E-09	0.142	0.350	0.207	-0.111
-0.3759	0.119	0.314	3.53E-04	0.102	0.341	0.240	-0.195
-0.3761	0.124	0.155	3.62E-07	0.129	0.261	0.132	-0.031
-0.3763	0.060	0.268	4.54E-03	0.052	0.282	0.230	-0.208
-0.3781	0.070	0.286	3.10E-16	0.077	0.307	0.230	-0.216
-0.3784	0.050	0.265	1.48E-08	0.038	0.272	0.234	-0.215
-0.3812	0.154	0.186	6.35E-04	0.160	0.306	0.146	-0.032
-0.3814	0.070	0.211	6.30E-15	0.056	0.244	0.188	-0.141
-0.3816	0.040	0.168	2.50E-03	0.032	0.210	0.178	-0.128
-0.3850	0.264	0.476	7.87E-04	0.284	0.608	0.323	-0.212
-0.3853	0.507	0.665	1.28E-04	0.857	1.260	0.403	-0.158
-0.3879	0.109	0.281	4.26E-06	0.099	0.324	0.225	-0.172
-0.3895	0.040	0.119	1.73E-18	0.033	0.170	0.137	-0.079
-0.3896	0.095	0.214	1.21E-10	0.049	0.248	0.199	-0.119
-0.3901	0.174	0.313	1.43E-07	0.158	0.391	0.233	-0.139

-0.3914	0.164	0.182	4.85E-04	0.157	0.285	0.128	-0.018
-0.3916	0.095	0.153	1.94E-08	0.080	0.230	0.150	-0.058
-0.3927	0.025	0.136	4.15E-32	0.062	0.244	0.182	-0.111
-0.3935	0.080	0.215	7.14E-10	0.057	0.249	0.192	-0.135
-0.3940	0.045	0.275	1.79E-04	0.036	0.283	0.248	-0.230
-0.3944	0.045	0.143	5.35E-16	0.026	0.187	0.161	-0.098
-0.3953	0.174	0.392	1.70E-04	0.162	0.447	0.285	-0.218
-0.3963	0.090	0.286	2.91E-07	0.070	0.306	0.235	-0.196
-0.3966	0.010	0.135	4.29E-04	0.006	0.187	0.181	-0.125
-0.3970	0.786	0.737	1.51E-07	2.092	2.375	0.283	0.049
-0.3973	0.065	0.300	2.66E-09	0.071	0.332	0.261	-0.235
-0.3973	0.269	0.415	6.99E-03	0.294	0.558	0.264	-0.146
-0.3988	0.050	0.270	4.94E-04	0.041	0.284	0.242	-0.220
-0.4040	0.109	0.359	1.11E-16	0.166	0.494	0.328	-0.250
-0.4048	0.806	0.740	1.58E-04	2.320	2.524	0.204	0.066
-0.4059	0.000	0.162	6.63E-08	0.000	0.207	0.207	-0.162
-0.4088	0.030	0.125	5.27E-06	0.040	0.205	0.165	-0.095
-0.4089	0.055	0.158	4.26E-12	0.081	0.256	0.175	-0.103
-0.4095	0.055	0.153	5.08E-09	0.094	0.284	0.190	-0.098
-0.4106	0.114	0.182	1.61E-05	0.079	0.236	0.157	-0.068
-0.4118	0.483	0.703	3.57E-04	0.745	1.153	0.408	-0.220
-0.4119	0.900	0.755	1.86E-20	2.878	2.886	0.008	0.145
-0.4127	0.109	0.383	1.32E-03	0.159	0.485	0.326	-0.274
-0.4144	0.040	0.186	2.23E-13	0.047	0.248	0.201	-0.146
-0.4149	0.065	0.164	1.63E-11	0.051	0.219	0.167	-0.099
-0.4154	0.204	0.414	8.62E-04	0.193	0.485	0.292	-0.210
-0.4163	0.269	0.482	2.06E-03	0.305	0.636	0.331	-0.213
-0.4174	0.025	0.134	4.40E-17	0.018	0.185	0.167	-0.109
-0.4177	0.090	0.265	2.43E-11	0.074	0.302	0.228	-0.175
-0.4178	0.104	0.175	1.31E-15	0.084	0.251	0.167	-0.071
-0.4180	0.119	0.322	2.45E-07	0.120	0.376	0.257	-0.203
-0.4186	0.045	0.189	2.46E-09	0.027	0.225	0.198	-0.144
-0.4191	0.030	0.247	1.10E-07	0.042	0.288	0.246	-0.217
-0.4209	0.060	0.131	5.18E-22	0.053	0.206	0.154	-0.071
-0.4213	0.896	0.944	2.92E-11	2.305	2.721	0.416	-0.048
-0.4215	0.308	0.538	2.12E-04	0.374	0.755	0.381	-0.230
-0.4219	0.159	0.361	2.00E-04	0.152	0.430	0.278	-0.202
-0.4230	0.184	0.405	5.60E-04	0.172	0.483	0.312	-0.221
-0.4247	0.194	0.418	1.59E-07	0.199	0.512	0.313	-0.224
-0.4263	0.109	0.231	7.28E-13	0.131	0.336	0.205	-0.122
-0.4268	0.557	0.322	7.97E-20	1.166	0.867	0.299	0.235
-0.4275	0.080	0.298	7.19E-10	0.070	0.332	0.262	-0.218
-0.4283	0.896	0.742	6.72E-29	2.593	2.697	0.104	0.154
-0.4312	0.070	0.255	1.06E-09	0.054	0.280	0.226	-0.185
-0.4312	0.274	0.484	2.94E-04	0.360	0.675	0.315	-0.210
-0.4316	0.100	0.149	3.29E-06	0.101	0.265	0.163	-0.049
-0.4318	0.134	0.234	7.28E-06	0.083	0.283	0.200	-0.100

-0.4351	0.090	0.250	5.02E-11	0.061	0.293	0.232	-0.160
-0.4352	0.080	0.159	4.67E-09	0.060	0.228	0.169	-0.079
-0.4354	0.020	0.107	1.16E-03	0.020	0.186	0.165	-0.087
-0.4359	0.080	0.219	6.45E-09	0.075	0.297	0.221	-0.139
-0.4365	0.159	0.321	6.66E-06	0.148	0.407	0.258	-0.162
-0.4366	0.030	0.274	1.26E-09	0.023	0.293	0.269	-0.244
-0.4368	0.224	0.390	3.42E-03	0.220	0.488	0.268	-0.166
-0.4401	0.095	0.132	2.58E-10	0.066	0.224	0.158	-0.037
-0.4406	0.090	0.271	1.40E-07	0.078	0.314	0.236	-0.181
-0.4420	0.164	0.390	1.29E-06	0.131	0.439	0.308	-0.226
-0.4436	0.100	0.120	1.66E-06	0.102	0.240	0.139	-0.020
-0.4439	0.065	0.293	1.49E-16	0.063	0.323	0.259	-0.228
-0.4446	0.085	0.180	2.94E-18	0.100	0.293	0.193	-0.095
-0.4446	0.065	0.149	1.65E-14	0.069	0.232	0.163	-0.084
-0.4451	0.164	0.389	1.26E-07	0.165	0.480	0.315	-0.225
-0.4458	0.090	0.153	2.05E-19	0.051	0.215	0.164	-0.063
-0.4481	0.751	0.897	1.16E-16	1.599	2.163	0.564	-0.146
-0.4482	0.562	0.771	3.45E-04	0.936	1.406	0.470	-0.209
-0.4484	0.134	0.170	8.19E-05	0.156	0.336	0.180	-0.036
-0.4498	0.144	0.304	4.59E-09	0.146	0.410	0.263	-0.160
-0.4513	0.080	0.284	4.10E-05	0.073	0.327	0.253	-0.204
-0.4520	0.164	0.393	2.80E-06	0.189	0.489	0.300	-0.229
-0.4520	0.045	0.251	1.85E-23	0.034	0.277	0.243	-0.206
-0.4526	0.060	0.265	9.73E-13	0.057	0.308	0.251	-0.205
-0.4528	0.060	0.284	4.20E-18	0.073	0.345	0.271	-0.224
-0.4531	0.144	0.327	4.90E-06	0.123	0.395	0.272	-0.183
-0.4542	0.090	0.350	1.91E-06	0.084	0.387	0.303	-0.260
-0.4547	0.174	0.277	2.42E-07	0.190	0.437	0.248	-0.103
-0.4550	0.080	0.128	9.92E-05	0.103	0.267	0.164	-0.048
-0.4551	0.159	0.369	4.93E-09	0.149	0.445	0.296	-0.210
-0.4557	0.095	0.305	1.66E-10	0.077	0.343	0.266	-0.210
-0.4557	0.095	0.305	1.66E-10	0.077	0.343	0.266	-0.210
-0.4603	0.065	0.146	5.54E-22	0.061	0.235	0.173	-0.081
-0.4620	0.134	0.285	4.11E-07	0.104	0.354	0.249	-0.151
-0.4633	0.070	0.139	1.16E-19	0.030	0.203	0.173	-0.069
-0.4637	0.070	0.203	6.97E-22	0.073	0.287	0.214	-0.133
-0.4638	0.617	0.805	1.48E-12	1.101	1.584	0.483	-0.188
-0.4648	0.373	0.611	2.36E-03	0.478	0.901	0.423	-0.238
-0.4662	0.040	0.281	2.65E-07	0.036	0.315	0.280	-0.241
-0.4675	0.095	0.215	4.89E-17	0.190	0.470	0.280	-0.120
-0.4696	0.030	0.163	9.22E-24	0.075	0.289	0.214	-0.133
-0.4724	0.040	0.104	2.44E-17	0.044	0.206	0.162	-0.064
-0.4730	0.010	0.110	3.85E-04	0.014	0.166	0.152	-0.100
-0.4738	0.080	0.289	3.82E-22	0.083	0.359	0.276	-0.209
-0.4750	0.547	0.749	4.14E-09	0.904	1.409	0.505	-0.202
-0.4753	0.104	0.105	6.41E-06	0.089	0.218	0.128	-0.001
-0.4761	0.149	0.400	4.69E-13	0.144	0.471	0.327	-0.251

-0.4764	0.254	0.497	1.07E-06	0.295	0.670	0.376	-0.243
-0.4767	0.075	0.116	5.68E-04	0.039	0.192	0.153	-0.041
-0.4769	0.045	0.177	7.00E-08	0.038	0.253	0.215	-0.132
-0.4778	0.159	0.416	1.29E-06	0.149	0.483	0.334	-0.257
-0.4783	0.861	0.759	7.01E-16	2.634	2.919	0.285	0.102
-0.4821	0.209	0.402	1.02E-04	0.179	0.472	0.293	-0.193
-0.4835	0.244	0.480	8.29E-03	0.241	0.623	0.382	-0.236
-0.4836	0.045	0.222	3.00E-22	0.044	0.290	0.246	-0.177
-0.4840	0.194	0.307	1.16E-04	0.216	0.470	0.254	-0.113
-0.4849	0.134	0.251	7.00E-05	0.101	0.330	0.229	-0.117
-0.4866	0.035	0.190	2.90E-23	0.029	0.247	0.219	-0.155
-0.4871	0.542	0.679	6.88E-04	1.184	1.788	0.604	-0.137
-0.4885	0.119	0.333	4.44E-06	0.097	0.393	0.295	-0.214
-0.4895	0.070	0.366	3.37E-07	0.077	0.411	0.334	-0.296
-0.4903	0.100	0.368	2.04E-12	0.093	0.416	0.323	-0.268
-0.4945	0.000	0.111	1.40E-04	0.000	0.195	0.195	-0.111
-0.4948	0.035	0.170	9.19E-49	0.049	0.280	0.232	-0.135
-0.4949	0.388	0.615	5.30E-05	0.479	0.895	0.416	-0.227
-0.4953	0.055	0.193	1.66E-12	0.091	0.332	0.241	-0.138
-0.4955	0.020	0.124	1.54E-13	0.022	0.204	0.181	-0.104
-0.4983	0.368	0.573	4.48E-03	0.444	0.857	0.413	-0.205
-0.4994	0.199	0.488	1.46E-11	0.255	0.648	0.392	-0.289
-0.5004	0.070	0.329	5.77E-06	0.073	0.382	0.308	-0.259
-0.5004	0.876	0.773	2.83E-27	2.714	2.933	0.218	0.103
-0.5006	0.035	0.112	3.40E-09	0.059	0.231	0.172	-0.077
-0.5012	0.657	0.802	1.41E-08	1.162	1.628	0.466	-0.145
-0.5031	0.184	0.361	2.34E-04	0.157	0.438	0.281	-0.177
-0.5033	0.075	0.365	5.76E-06	0.082	0.426	0.343	-0.290
-0.5037	0.557	0.769	1.94E-03	1.001	1.563	0.563	-0.212
-0.5038	0.333	0.583	2.68E-05	0.414	0.840	0.426	-0.250
-0.5057	0.159	0.399	6.54E-06	0.161	0.494	0.334	-0.240
-0.5060	0.025	0.162	5.61E-24	0.023	0.227	0.204	-0.137
-0.5075	0.065	0.149	2.80E-13	0.071	0.264	0.194	-0.084
-0.5083	0.955	0.977	6.93E-30	3.014	3.545	0.531	-0.022
-0.5093	0.826	0.742	4.55E-40	2.303	2.642	0.338	0.084
-0.5099	0.144	0.443	7.78E-16	0.170	0.567	0.397	-0.299
-0.5134	0.104	0.175	3.47E-10	0.072	0.259	0.188	-0.071
-0.5167	0.114	0.410	4.66E-12	0.112	0.478	0.366	-0.296
-0.5182	0.920	0.760	7.67E-35	3.063	3.215	0.153	0.160
-0.5186	0.139	0.222	7.22E-09	0.165	0.387	0.222	-0.083
-0.5194	0.075	0.378	1.85E-15	0.086	0.442	0.356	-0.303
-0.5199	0.139	0.254	1.70E-08	0.118	0.365	0.248	-0.115
-0.5216	0.114	0.248	6.50E-07	0.079	0.319	0.240	-0.134
-0.5226	0.900	0.826	6.65E-10	2.752	3.057	0.305	0.074
-0.5232	0.736	0.706	4.68E-29	1.765	2.213	0.449	0.030
-0.5260	0.045	0.136	6.17E-20	0.039	0.237	0.198	-0.091
-0.5277	0.692	0.717	1.33E-04	1.825	2.370	0.544	-0.025

-0.5301	0.149	0.438	2.15E-04	0.172	0.548	0.376	-0.289
-0.5327	0.637	0.708	4.42E-15	1.369	1.927	0.558	-0.071
-0.5343	0.070	0.310	9.53E-09	0.064	0.368	0.304	-0.240
-0.5351	0.378	0.642	1.90E-06	0.470	0.952	0.482	-0.264
-0.5361	0.254	0.483	6.03E-06	0.247	0.642	0.395	-0.229
-0.5370	0.020	0.106	4.18E-08	0.032	0.219	0.187	-0.086
-0.5389	0.124	0.192	1.32E-05	0.104	0.293	0.189	-0.068
-0.5389	0.124	0.192	1.32E-05	0.104	0.293	0.189	-0.068
-0.5389	0.299	0.564	2.98E-09	0.379	0.846	0.468	-0.265
-0.5401	0.204	0.454	1.64E-04	0.193	0.569	0.375	-0.250
-0.5401	0.065	0.313	1.44E-06	0.067	0.390	0.323	-0.248
-0.5407	0.905	0.959	7.67E-25	2.454	3.087	0.633	-0.054
-0.5415	0.478	0.568	1.25E-04	0.774	1.059	0.285	-0.090
-0.5430	0.020	0.154	6.45E-31	0.020	0.236	0.216	-0.134
-0.5440	0.104	0.177	1.22E-06	0.105	0.297	0.191	-0.073
-0.5467	0.149	0.463	3.65E-15	0.163	0.591	0.427	-0.314
-0.5476	0.035	0.218	7.74E-38	0.043	0.312	0.269	-0.183
-0.5476	0.015	0.148	2.67E-41	0.027	0.241	0.214	-0.133
-0.5479	0.045	0.140	5.08E-18	0.041	0.244	0.203	-0.095
-0.5481	0.159	0.400	2.16E-11	0.182	0.540	0.358	-0.241
-0.5490	0.015	0.107	6.48E-21	0.004	0.200	0.196	-0.092
-0.5502	0.229	0.447	1.36E-04	0.223	0.582	0.359	-0.218
-0.5506	0.413	0.594	3.65E-08	0.609	1.054	0.445	-0.181
-0.5528	0.308	0.522	1.18E-03	0.357	0.761	0.404	-0.214
-0.5536	0.144	0.428	1.13E-03	0.188	0.587	0.400	-0.284
-0.5553	0.348	0.583	2.78E-03	0.403	0.824	0.420	-0.235
-0.5557	0.900	0.955	1.71E-28	2.240	2.842	0.601	-0.055
-0.5572	0.100	0.175	1.75E-06	0.075	0.295	0.220	-0.075
-0.5581	0.323	0.511	1.43E-03	0.368	0.755	0.387	-0.188
-0.5583	0.095	0.354	4.03E-12	0.111	0.452	0.341	-0.259
-0.5601	0.697	0.885	3.18E-17	1.433	2.081	0.648	-0.188
-0.5620	0.065	0.181	1.05E-07	0.152	0.442	0.291	-0.116
-0.5629	0.348	0.607	2.35E-08	0.446	0.918	0.473	-0.259
-0.5634	0.070	0.186	6.90E-24	0.068	0.305	0.237	-0.116
-0.5651	0.114	0.278	6.21E-11	0.150	0.408	0.259	-0.164
-0.5658	0.035	0.188	1.53E-29	0.019	0.259	0.239	-0.153
-0.5669	0.144	0.369	3.73E-11	0.119	0.458	0.339	-0.225
-0.5672	0.045	0.221	1.43E-06	0.051	0.312	0.261	-0.176
-0.5673	0.164	0.376	7.22E-12	0.218	0.575	0.356	-0.212
-0.5683	0.015	0.233	3.82E-09	0.010	0.304	0.294	-0.218
-0.5712	0.169	0.312	8.15E-07	0.166	0.453	0.287	-0.143
-0.5725	0.159	0.373	3.97E-06	0.137	0.450	0.313	-0.214
-0.5744	0.701	0.887	1.33E-15	1.496	2.203	0.707	-0.186
-0.5745	0.050	0.235	3.84E-20	0.025	0.308	0.283	-0.185
-0.5773	0.204	0.397	1.62E-06	0.188	0.541	0.353	-0.193
-0.5814	0.100	0.220	1.64E-11	0.090	0.334	0.245	-0.120
-0.5824	0.000	0.118	3.61E-10	0.000	0.215	0.215	-0.118

-0.5826	0.080	0.388	5.26E-09	0.108	0.503	0.395	-0.308
-0.5844	0.075	0.101	3.94E-03	0.043	0.208	0.165	-0.026
-0.5854	0.020	0.177	1.43E-48	0.035	0.293	0.258	-0.157
-0.5857	0.353	0.613	7.90E-08	0.450	0.925	0.475	-0.260
-0.5875	0.159	0.503	5.78E-19	0.208	0.670	0.462	-0.344
-0.5877	0.075	0.412	5.10E-11	0.083	0.496	0.412	-0.337
-0.5896	0.194	0.454	2.52E-07	0.196	0.610	0.413	-0.260
-0.5899	0.234	0.477	1.75E-07	0.229	0.629	0.400	-0.243
-0.5900	0.801	0.735	6.52E-22	2.366	2.824	0.458	0.066
-0.5903	0.134	0.432	2.81E-07	0.166	0.578	0.412	-0.298
-0.5909	0.060	0.185	2.57E-16	0.041	0.271	0.230	-0.125
-0.5910	0.234	0.591	3.31E-15	0.296	0.847	0.550	-0.357
-0.5918	0.100	0.389	1.05E-14	0.107	0.476	0.369	-0.289
-0.5944	0.040	0.130	2.23E-12	0.069	0.290	0.221	-0.090
-0.5953	0.040	0.203	1.95E-29	0.043	0.302	0.259	-0.163
-0.5962	0.080	0.315	2.58E-19	0.078	0.400	0.322	-0.235
-0.5992	0.637	0.754	2.02E-04	1.447	2.101	0.654	-0.117
-0.6007	0.000	0.125	1.19E-05	0.000	0.217	0.217	-0.125
-0.6026	0.134	0.473	3.44E-09	0.155	0.602	0.447	-0.339
-0.6030	0.090	0.384	8.99E-09	0.086	0.471	0.385	-0.294
-0.6032	0.169	0.265	4.51E-05	0.198	0.465	0.267	-0.096
-0.6033	0.020	0.109	1.07E-08	0.044	0.238	0.194	-0.089
-0.6044	0.174	0.478	7.15E-12	0.271	0.706	0.435	-0.304
-0.6047	0.020	0.124	1.81E-12	0.041	0.268	0.227	-0.104
-0.6053	0.219	0.453	2.45E-08	0.208	0.609	0.401	-0.234
-0.6065	0.214	0.369	4.58E-08	0.239	0.582	0.343	-0.155
-0.6084	0.060	0.127	1.20E-14	0.063	0.271	0.208	-0.067
-0.6097	0.408	0.671	2.51E-07	0.856	1.555	0.699	-0.263
-0.6100	0.080	0.203	1.46E-13	0.166	0.500	0.333	-0.123
-0.6135	0.453	0.474	1.72E-06	0.822	1.042	0.219	-0.021
-0.6150	0.020	0.187	1.56E-05	0.014	0.283	0.269	-0.167
-0.6156	0.935	0.766	4.92E-50	2.900	3.132	0.232	0.169
-0.6158	0.204	0.285	3.64E-03	0.202	0.474	0.272	-0.081
-0.6165	0.642	0.708	1.57E-11	1.515	2.193	0.678	-0.066
-0.6169	0.328	0.556	2.18E-06	0.395	0.834	0.439	-0.228
-0.6177	0.095	0.322	8.04E-14	0.101	0.433	0.332	-0.227
-0.6186	0.174	0.484	3.05E-13	0.191	0.649	0.458	-0.310
-0.6198	0.209	0.479	2.92E-09	0.194	0.627	0.433	-0.270
-0.6198	0.831	0.885	1.22E-07	1.874	2.106	0.231	-0.054
-0.6205	0.174	0.351	4.53E-11	0.206	0.575	0.369	-0.177
-0.6209	0.294	0.562	2.32E-11	0.316	0.801	0.485	-0.268
-0.6217	0.035	0.113	1.05E-08	0.047	0.245	0.198	-0.078
-0.6252	0.030	0.146	8.26E-28	0.042	0.264	0.222	-0.116
-0.6254	0.861	0.938	2.39E-21	2.090	2.605	0.515	-0.077
-0.6257	0.124	0.439	5.62E-08	0.152	0.587	0.434	-0.315
-0.6271	0.035	0.221	7.46E-20	0.046	0.344	0.298	-0.186
-0.6304	0.025	0.128	2.94E-15	0.041	0.278	0.237	-0.103

-0.6307	0.000	0.134	4.86E-09	0.000	0.229	0.229	-0.134
-0.6312	0.154	0.465	4.30E-07	0.172	0.632	0.461	-0.311
-0.6316	0.045	0.143	2.03E-12	0.065	0.328	0.263	-0.098
-0.6319	0.005	0.174	1.83E-59	0.002	0.269	0.267	-0.169
-0.6331	0.080	0.226	5.53E-22	0.143	0.455	0.311	-0.146
-0.6338	0.060	0.318	1.83E-09	0.069	0.427	0.358	-0.258
-0.6340	0.174	0.407	3.50E-03	0.432	1.001	0.570	-0.233
-0.6340	0.169	0.431	3.35E-03	0.328	0.816	0.489	-0.262
-0.6360	0.000	0.127	1.30E-13	0.000	0.236	0.236	-0.127
-0.6362	0.109	0.462	3.56E-17	0.120	0.565	0.445	-0.353
-0.6374	0.045	0.231	8.97E-03	0.056	0.337	0.281	-0.186
-0.6376	0.194	0.368	3.27E-09	0.205	0.556	0.351	-0.174
-0.6382	0.050	0.137	2.65E-14	0.048	0.265	0.217	-0.087
-0.6386	0.045	0.123	1.11E-07	0.068	0.269	0.201	-0.078
-0.6426	0.144	0.486	2.49E-13	0.157	0.616	0.460	-0.342
-0.6452	0.095	0.194	1.92E-10	0.118	0.372	0.253	-0.099
-0.6452	0.423	0.618	1.49E-06	0.548	0.989	0.442	-0.195
-0.6454	0.478	0.589	1.00E-06	0.799	1.110	0.312	-0.111
-0.6460	0.025	0.235	2.88E-35	0.031	0.349	0.318	-0.210
-0.6471	0.085	0.173	7.85E-10	0.106	0.358	0.252	-0.088
-0.6483	0.199	0.302	7.63E-06	0.264	0.567	0.303	-0.103
-0.6486	0.114	0.245	4.23E-13	0.116	0.379	0.263	-0.131
-0.6498	0.010	0.182	3.82E-40	0.019	0.306	0.287	-0.172
-0.6515	0.726	0.724	4.48E-26	1.776	2.392	0.616	0.002
-0.6528	0.164	0.510	2.25E-08	0.276	0.843	0.567	-0.346
-0.6539	0.124	0.310	2.95E-15	0.091	0.414	0.323	-0.186
-0.6542	0.090	0.392	3.66E-09	0.092	0.496	0.404	-0.302
-0.6546	0.114	0.443	1.60E-09	0.141	0.599	0.458	-0.329
-0.6562	0.065	0.168	9.22E-06	0.130	0.415	0.285	-0.103
-0.6574	0.134	0.402	4.02E-22	0.140	0.565	0.425	-0.268
-0.6592	0.159	0.534	7.03E-15	0.211	0.742	0.531	-0.375
-0.6596	0.015	0.121	2.01E-17	0.022	0.254	0.232	-0.106
-0.6604	0.080	0.174	2.93E-07	0.148	0.425	0.277	-0.094
-0.6648	0.095	0.198	7.18E-12	0.057	0.296	0.239	-0.103
-0.6662	0.721	0.771	5.16E-20	1.899	2.717	0.818	-0.050
-0.6663	0.109	0.492	4.53E-29	0.139	0.658	0.520	-0.383
-0.6702	0.279	0.341	8.21E-03	0.379	0.710	0.331	-0.062
-0.6711	0.408	0.253	9.74E-03	0.856	0.884	0.028	0.155
-0.6711	0.925	0.899	2.69E-36	2.794	3.474	0.680	0.026
-0.6713	0.159	0.535	2.76E-20	0.221	0.759	0.538	-0.376
-0.6718	0.856	0.766	4.82E-58	2.447	3.016	0.569	0.090
-0.6721	0.005	0.110	1.14E-25	0.001	0.226	0.225	-0.105
-0.6755	0.303	0.459	2.62E-04	0.354	0.736	0.382	-0.156
-0.6757	0.100	0.392	8.27E-23	0.091	0.490	0.399	-0.292
-0.6766	0.075	0.117	1.09E-09	0.046	0.258	0.212	-0.042
-0.6792	0.189	0.263	1.40E-06	0.176	0.453	0.277	-0.074
-0.6795	0.333	0.555	3.17E-06	0.398	0.852	0.454	-0.222

-0.6799	0.259	0.537	8.00E-06	0.293	0.797	0.505	-0.278
-0.6813	0.144	0.254	1.11E-06	0.126	0.416	0.290	-0.110
-0.6821	0.209	0.485	1.14E-05	0.230	0.718	0.488	-0.276
-0.6828	0.403	0.627	2.41E-08	0.575	1.101	0.525	-0.224
-0.6832	0.363	0.406	1.59E-03	0.587	0.904	0.317	-0.043
-0.6837	0.104	0.221	4.50E-09	0.134	0.414	0.280	-0.117
-0.6840	0.104	0.353	4.55E-03	0.110	0.495	0.385	-0.249
-0.6843	0.065	0.235	1.03E-21	0.065	0.353	0.287	-0.170
-0.6851	0.219	0.359	1.75E-06	0.246	0.582	0.335	-0.140
-0.6861	0.274	0.423	3.14E-04	0.331	0.709	0.378	-0.149
-0.6873	0.134	0.253	1.18E-12	0.205	0.555	0.350	-0.119
-0.6875	0.020	0.169	4.30E-04	0.015	0.292	0.277	-0.149
-0.6897	0.065	0.181	1.19E-21	0.051	0.297	0.246	-0.116
-0.6906	0.234	0.361	6.05E-09	0.274	0.612	0.337	-0.127
-0.6920	0.020	0.274	3.33E-11	0.016	0.374	0.358	-0.254
-0.6944	0.154	0.536	3.25E-15	0.215	0.781	0.566	-0.382
-0.6951	0.025	0.136	1.64E-15	0.045	0.300	0.255	-0.111
-0.6967	0.219	0.324	2.04E-03	0.247	0.577	0.330	-0.105
-0.7003	0.035	0.176	6.28E-44	0.039	0.325	0.287	-0.141
-0.7026	0.244	0.630	1.59E-19	0.312	0.923	0.611	-0.386
-0.7034	0.527	0.711	3.88E-12	1.096	1.927	0.831	-0.184
-0.7044	0.522	0.819	4.17E-24	0.849	1.604	0.755	-0.297
-0.7076	0.104	0.448	5.39E-25	0.106	0.568	0.461	-0.344
-0.7087	0.204	0.405	1.23E-10	0.194	0.625	0.430	-0.201
-0.7089	0.010	0.234	3.27E-37	0.012	0.354	0.342	-0.224
-0.7094	0.199	0.443	9.76E-13	0.188	0.643	0.455	-0.244
-0.7097	0.204	0.290	4.69E-06	0.230	0.527	0.297	-0.086
-0.7119	0.363	0.637	1.34E-15	0.435	1.009	0.575	-0.274
-0.7134	0.219	0.487	3.05E-10	0.197	0.641	0.444	-0.268
-0.7163	0.015	0.111	7.90E-15	0.016	0.245	0.229	-0.096
-0.7179	0.413	0.501	4.15E-09	0.624	0.913	0.290	-0.088
-0.7202	0.040	0.163	1.04E-21	0.068	0.360	0.292	-0.123
-0.7209	0.502	0.706	3.12E-11	1.117	1.941	0.825	-0.204
-0.7213	0.144	0.266	1.11E-08	0.152	0.431	0.279	-0.122
-0.7219	0.080	0.358	2.56E-18	0.054	0.471	0.417	-0.278
-0.7227	0.045	0.142	6.60E-08	0.065	0.322	0.257	-0.097
-0.7233	0.035	0.136	2.10E-12	0.061	0.311	0.250	-0.101
-0.7254	0.567	0.738	4.31E-10	1.173	1.973	0.800	-0.171
-0.7293	0.045	0.149	9.65E-09	0.081	0.386	0.305	-0.104
-0.7300	0.478	0.499	2.54E-11	0.716	0.995	0.278	-0.021
-0.7311	0.134	0.453	1.41E-26	0.187	0.699	0.512	-0.319
-0.7329	0.035	0.138	1.30E-07	0.056	0.311	0.255	-0.103
-0.7341	0.313	0.652	6.16E-13	0.368	1.001	0.633	-0.339
-0.7344	0.119	0.524	1.54E-28	0.159	0.703	0.545	-0.405
-0.7350	0.104	0.282	2.21E-22	0.105	0.459	0.354	-0.178
-0.7352	0.045	0.115	5.95E-03	0.055	0.271	0.216	-0.070
-0.7358	0.119	0.200	4.87E-06	0.166	0.449	0.283	-0.081

-0.7361	0.692	0.748	4.88E-11	1.985	2.888	0.903	-0.056
-0.7364	0.065	0.179	3.05E-07	0.123	0.443	0.320	-0.114
-0.7395	0.015	0.127	6.87E-17	0.032	0.282	0.250	-0.112
-0.7405	0.075	0.211	4.51E-13	0.052	0.329	0.277	-0.136
-0.7412	0.174	0.393	3.27E-15	0.196	0.632	0.436	-0.219
-0.7437	0.149	0.499	8.79E-11	0.203	0.737	0.534	-0.350
-0.7449	0.318	0.506	1.54E-05	0.379	0.833	0.454	-0.188
-0.7470	0.085	0.395	3.71E-28	0.099	0.543	0.444	-0.310
-0.7482	0.050	0.387	2.89E-28	0.040	0.482	0.443	-0.337
-0.7483	0.025	0.124	2.01E-11	0.034	0.262	0.227	-0.099
-0.7485	0.756	0.740	1.34E-60	2.129	3.032	0.903	0.016
-0.7497	0.045	0.159	4.30E-08	0.096	0.405	0.309	-0.114
-0.7521	0.010	0.111	1.15E-11	0.024	0.252	0.228	-0.101
-0.7530	0.065	0.159	1.04E-05	0.124	0.389	0.265	-0.094
-0.7540	0.139	0.420	1.76E-05	0.194	0.656	0.462	-0.281
-0.7546	0.383	0.576	2.69E-05	0.506	0.919	0.413	-0.193
-0.7567	0.035	0.166	7.63E-31	0.031	0.290	0.260	-0.131
-0.7604	0.458	0.637	6.34E-11	0.723	1.224	0.501	-0.179
-0.7641	0.239	0.380	3.10E-06	0.262	0.666	0.404	-0.141
-0.7650	0.045	0.251	6.20E-54	0.100	0.533	0.433	-0.206
-0.7691	0.164	0.560	1.83E-29	0.237	0.887	0.650	-0.396
-0.7727	0.114	0.454	1.11E-09	0.145	0.647	0.502	-0.340
-0.7749	0.204	0.579	9.99E-19	0.294	0.934	0.640	-0.375
-0.7803	0.179	0.485	7.55E-05	0.338	0.964	0.626	-0.306
-0.7804	0.109	0.364	4.41E-03	0.226	0.750	0.524	-0.255
-0.7817	0.960	0.973	4.00E-41	3.200	3.864	0.664	-0.013
-0.7823	0.040	0.169	8.54E-12	0.088	0.399	0.311	-0.129
-0.7824	0.010	0.132	3.67E-18	0.013	0.287	0.274	-0.122
-0.7840	0.174	0.618	2.11E-32	0.333	1.100	0.767	-0.444
-0.7915	0.736	0.936	3.05E-38	1.623	2.504	0.881	-0.200
-0.7921	0.065	0.182	8.27E-13	0.118	0.462	0.344	-0.117
-0.7957	0.035	0.121	3.06E-09	0.034	0.284	0.250	-0.086
-0.8052	0.015	0.158	2.67E-29	0.042	0.348	0.307	-0.143
-0.8057	0.189	0.565	4.63E-21	0.248	0.869	0.621	-0.376
-0.8069	0.154	0.463	4.23E-12	0.173	0.731	0.559	-0.309
-0.8088	0.274	0.521	8.91E-09	0.293	0.794	0.501	-0.247
-0.8109	0.100	0.389	7.58E-06	0.122	0.581	0.458	-0.289
-0.8125	0.030	0.149	2.70E-14	0.059	0.333	0.274	-0.119
-0.8152	0.149	0.407	1.52E-18	0.165	0.608	0.443	-0.258
-0.8234	0.199	0.336	7.62E-08	0.229	0.600	0.371	-0.137
-0.8234	0.095	0.461	1.55E-11	0.104	0.643	0.540	-0.366
-0.8313	0.338	0.446	1.10E-07	0.503	0.958	0.455	-0.108
-0.8326	0.169	0.539	1.18E-10	0.292	0.996	0.704	-0.370
-0.8331	0.338	0.734	1.10E-25	0.469	1.277	0.807	-0.396
-0.8369	0.104	0.249	3.73E-19	0.193	0.635	0.442	-0.145
-0.8458	0.527	0.681	2.51E-16	0.847	1.476	0.629	-0.154
-0.8474	1.000	0.998	6.51E-154	3.797	4.797	1.000	0.002

-0.8585	0.055	0.158	3.04E-06	0.109	0.394	0.285	-0.103
-0.8608	0.174	0.478	6.75E-20	0.256	0.749	0.493	-0.304
-0.8727	0.075	0.208	1.66E-07	0.162	0.580	0.419	-0.133
-0.8736	0.149	0.539	2.92E-26	0.159	0.780	0.622	-0.390
-0.8740	0.030	0.131	2.19E-14	0.034	0.316	0.282	-0.101
-0.8804	0.055	0.291	3.56E-24	0.061	0.481	0.420	-0.236
-0.8827	0.100	0.423	6.89E-24	0.114	0.607	0.493	-0.323
-0.8843	0.065	0.176	2.01E-06	0.107	0.463	0.356	-0.111
-0.8850	0.010	0.161	4.84E-05	0.016	0.353	0.337	-0.151
-0.8867	0.174	0.301	2.63E-12	0.202	0.607	0.405	-0.127
-0.8878	0.428	0.789	6.40E-34	0.629	1.511	0.882	-0.361
-0.8914	0.124	0.491	6.01E-13	0.176	0.787	0.612	-0.367
-0.8930	0.010	0.159	3.40E-06	0.013	0.302	0.289	-0.149
-0.8947	0.169	0.521	2.82E-09	0.277	0.966	0.689	-0.352
-0.8952	0.020	0.143	1.43E-16	0.035	0.341	0.307	-0.123
-0.8981	0.025	0.305	3.37E-35	0.038	0.516	0.478	-0.280
-0.9019	0.045	0.201	4.19E-21	0.052	0.390	0.339	-0.156
-0.9029	0.826	0.926	1.27E-32	1.922	2.544	0.622	-0.100
-0.9091	0.318	0.708	2.30E-29	0.479	1.292	0.813	-0.390
-0.9094	0.537	0.841	2.40E-43	0.941	1.932	0.991	-0.304
-0.9163	0.184	0.486	3.13E-07	0.435	1.200	0.765	-0.302
-0.9176	0.075	0.342	7.05E-25	0.080	0.517	0.437	-0.267
-0.9252	0.050	0.184	1.83E-21	0.059	0.353	0.294	-0.134
-0.9259	0.204	0.722	5.65E-53	0.355	1.279	0.923	-0.518
-0.9289	0.060	0.207	4.87E-09	0.148	0.578	0.430	-0.147
-0.9299	0.144	0.513	3.09E-12	0.289	1.033	0.744	-0.369
-0.9309	0.000	0.164	4.12E-08	0.000	0.341	0.341	-0.164
-0.9314	0.174	0.264	5.84E-08	0.161	0.524	0.362	-0.090
-0.9338	0.070	0.161	1.20E-08	0.080	0.421	0.341	-0.091
-0.9361	0.020	0.158	3.04E-25	0.035	0.346	0.310	-0.138
-0.9436	0.184	0.328	5.48E-12	0.230	0.696	0.466	-0.144
-0.9451	0.060	0.219	2.53E-08	0.073	0.387	0.314	-0.159
-0.9470	0.080	0.306	7.63E-32	0.097	0.523	0.426	-0.226
-0.9499	0.169	0.520	6.74E-23	0.163	0.734	0.571	-0.351
-0.9531	0.159	0.614	7.09E-41	0.234	1.032	0.798	-0.455
-0.9651	0.204	0.692	1.05E-45	0.267	1.103	0.836	-0.488
-0.9658	0.149	0.585	4.63E-33	0.184	0.908	0.723	-0.436
-0.9693	0.154	0.628	8.44E-47	0.255	1.068	0.813	-0.474
-0.9718	0.085	0.358	5.10E-16	0.115	0.622	0.507	-0.273
-0.9755	0.015	0.150	5.77E-03	0.033	0.352	0.319	-0.135
-0.9770	0.189	0.547	5.89E-23	0.235	0.900	0.666	-0.358
-0.9815	0.199	0.418	5.45E-11	0.200	0.710	0.510	-0.219
-0.9815	0.154	0.385	1.39E-19	0.167	0.665	0.498	-0.231
-0.9873	0.169	0.641	1.21E-45	0.240	1.059	0.819	-0.472
-0.9932	0.124	0.532	2.08E-28	0.127	0.800	0.673	-0.408
-0.9991	0.184	0.565	1.05E-28	0.271	1.031	0.760	-0.381
-1.0003	0.055	0.341	3.54E-34	0.086	0.611	0.525	-0.286

-1.0021	0.134	0.490	1.79E-10	0.196	0.857	0.661	-0.356
-1.0097	0.080	0.222	6.28E-05	0.191	0.680	0.489	-0.142
-1.0143	0.194	0.521	1.52E-15	0.534	1.429	0.895	-0.327
-1.0162	0.164	0.471	3.41E-09	0.280	0.869	0.589	-0.307
-1.0166	0.149	0.554	4.01E-22	0.195	0.914	0.719	-0.405
-1.0185	0.204	0.716	8.66E-47	0.392	1.414	1.021	-0.512
-1.0242	0.189	0.660	2.04E-43	0.509	1.712	1.203	-0.471
-1.0246	0.090	0.470	6.45E-25	0.097	0.739	0.641	-0.380
-1.0289	0.090	0.229	3.31E-07	0.213	0.736	0.523	-0.139
-1.0346	0.209	0.272	4.57E-08	0.604	1.479	0.875	-0.063
-1.0420	0.328	0.655	6.53E-20	0.437	1.267	0.830	-0.327
-1.0503	0.398	0.685	6.87E-25	0.694	1.587	0.893	-0.287
-1.0572	0.910	0.990	1.60E-113	3.027	4.518	1.490	-0.080
-1.0573	0.124	0.355	2.84E-17	0.224	0.796	0.572	-0.231
-1.0580	0.179	0.657	1.00E-48	0.299	1.184	0.884	-0.478
-1.0602	0.005	0.141	3.31E-05	0.003	0.342	0.339	-0.136
-1.0610	0.512	0.795	1.83E-34	0.843	1.735	0.891	-0.283
-1.0631	0.582	0.946	3.45E-85	1.218	2.713	1.495	-0.364
-1.0810	0.418	0.271	3.68E-07	0.722	0.868	0.147	0.147
-1.0909	0.070	0.361	1.00E-33	0.151	0.860	0.709	-0.291
-1.0946	0.000	0.107	1.33E-11	0.000	0.290	0.290	-0.107
-1.0988	0.010	0.181	2.27E-06	0.007	0.393	0.386	-0.171
-1.0990	0.189	0.616	6.19E-42	0.317	1.229	0.912	-0.427
-1.0995	0.010	0.130	4.01E-05	0.008	0.313	0.306	-0.120
-1.1034	0.179	0.673	1.66E-59	0.296	1.295	0.999	-0.494
-1.1186	0.338	0.701	1.19E-35	0.538	1.535	0.997	-0.363
-1.1252	0.219	0.788	3.56E-65	0.398	1.586	1.187	-0.569
-1.1276	0.045	0.195	1.18E-22	0.083	0.496	0.413	-0.150
-1.1439	0.194	0.583	1.57E-31	0.303	1.091	0.789	-0.389
-1.1616	0.299	0.730	2.00E-54	0.473	1.586	1.114	-0.431
-1.1666	0.174	0.514	1.64E-33	0.258	1.016	0.758	-0.340
-1.1687	0.055	0.226	4.88E-15	0.103	0.556	0.454	-0.171
-1.1689	0.199	0.656	1.28E-41	0.320	1.284	0.964	-0.457
-1.1689	0.085	0.276	1.24E-12	0.140	0.700	0.561	-0.191
-1.1692	0.095	0.226	1.59E-12	0.124	0.554	0.430	-0.131
-1.1788	0.104	0.277	4.31E-31	0.149	0.667	0.518	-0.173
-1.1823	0.025	0.229	9.85E-47	0.040	0.469	0.429	-0.204
-1.1882	0.070	0.210	8.91E-08	0.138	0.614	0.476	-0.140
-1.1921	0.204	0.645	3.81E-45	0.336	1.317	0.981	-0.441
-1.2001	0.065	0.247	1.69E-06	0.083	0.491	0.408	-0.182
-1.2032	0.821	0.961	4.52E-60	1.960	3.106	1.146	-0.140
-1.2283	0.368	0.598	1.21E-17	0.693	1.683	0.990	-0.230
-1.2338	0.000	0.147	4.81E-08	0.000	0.386	0.386	-0.147
-1.2354	0.174	0.368	1.17E-14	0.193	0.725	0.532	-0.194
-1.2400	0.672	0.945	3.32E-80	1.425	2.920	1.494	-0.273
-1.2416	0.358	0.627	9.09E-20	0.469	1.234	0.765	-0.269
-1.2427	0.085	0.284	2.45E-29	0.170	0.718	0.548	-0.199

-1.2485	0.209	0.306	1.32E-15	0.227	0.690	0.463	-0.097
-1.2534	0.189	0.689	2.26E-62	0.405	1.651	1.245	-0.500
-1.2579	0.214	0.734	7.97E-48	0.321	1.319	0.998	-0.520
-1.2581	0.035	0.207	9.67E-06	0.040	0.493	0.453	-0.172
-1.2599	0.055	0.213	5.82E-04	0.079	0.533	0.454	-0.158
-1.2600	0.114	0.445	4.39E-41	0.183	0.903	0.720	-0.331
-1.2658	0.149	0.212	2.51E-14	0.236	0.680	0.444	-0.063
-1.2797	0.149	0.665	9.09E-54	0.192	1.127	0.935	-0.516
-1.2809	0.204	0.679	1.25E-51	0.471	1.725	1.254	-0.475
-1.2815	0.104	0.221	1.55E-10	0.204	0.741	0.536	-0.117
-1.2856	0.075	0.351	2.57E-10	0.080	0.687	0.607	-0.276
-1.2952	0.139	0.251	1.09E-23	0.299	0.964	0.665	-0.112
-1.3004	0.114	0.492	1.42E-40	0.155	0.888	0.733	-0.378
-1.3109	0.169	0.265	8.76E-17	0.375	1.152	0.777	-0.096
-1.3133	0.109	0.256	1.11E-09	0.233	0.809	0.576	-0.147
-1.3133	0.184	0.264	1.37E-12	0.447	1.274	0.828	-0.080
-1.3258	0.085	0.393	8.26E-15	0.085	0.699	0.614	-0.308
-1.3381	0.070	0.353	3.51E-14	0.076	0.657	0.580	-0.283
-1.3387	0.284	0.468	1.62E-22	0.393	1.119	0.726	-0.184
-1.3513	0.328	0.508	1.51E-14	0.490	1.236	0.747	-0.180
-1.3525	0.114	0.399	1.41E-29	0.181	0.895	0.714	-0.285
-1.3690	0.065	0.438	1.65E-38	0.100	0.920	0.820	-0.373
-1.3702	0.219	0.408	2.63E-17	0.323	0.935	0.612	-0.189
-1.3787	0.104	0.271	9.17E-18	0.141	0.709	0.568	-0.167
-1.4051	0.224	0.320	1.39E-25	0.552	1.377	0.825	-0.096
-1.4130	0.085	0.240	8.47E-10	0.205	0.851	0.646	-0.155
-1.4423	0.209	0.334	2.57E-24	0.286	0.881	0.595	-0.125
-1.4594	0.264	0.644	4.89E-29	0.311	1.262	0.951	-0.380
-1.5044	0.468	0.802	1.64E-36	0.714	1.772	1.058	-0.334
-1.5311	0.234	0.756	1.19E-59	0.373	1.743	1.370	-0.522
-1.5437	0.333	0.255	1.09E-07	0.533	0.587	0.054	0.078
-1.5821	0.070	0.463	8.26E-20	0.089	0.821	0.732	-0.393
-1.6214	0.134	0.255	3.79E-27	0.353	1.095	0.742	-0.121
-1.6342	0.095	0.254	4.32E-13	0.250	1.015	0.765	-0.159
-1.7474	0.100	0.324	1.43E-37	0.253	1.169	0.916	-0.224
-1.8194	0.015	0.275	1.36E-17	0.029	0.773	0.744	-0.260
-1.8330	0.249	0.487	5.89E-22	0.477	1.840	1.364	-0.238
-1.8830	0.035	0.245	1.49E-14	0.045	0.644	0.599	-0.210
-2.1794	0.194	0.555	1.93E-31	0.210	1.229	1.018	-0.361
-2.3327	0.035	0.237	4.69E-07	0.044	0.734	0.690	-0.202
-2.4709	0.045	0.211	3.10E-04	0.061	0.675	0.614	-0.166
-2.5084	0.010	0.185	1.71E-09	0.017	0.529	0.512	-0.175

Category	Term	Count
GOTERM_BP_DIRECT	GO:0070125~mitochondrial translational elongation	38
GOTERM_BP_DIRECT	GO:0032981~mitochondrial respiratory chain complex I a	33
GOTERM_BP_DIRECT	GO:0006120~mitochondrial electron transport, NADH to	29
GOTERM_BP_DIRECT	GO:0070126~mitochondrial translational termination	36
GOTERM_BP_DIRECT	GO:0098609~cell-cell adhesion	42
GOTERM_BP_DIRECT	GO:0006412~translation	38
GOTERM_BP_DIRECT	GO:0055114~oxidation-reduction process	66
GOTERM_BP_DIRECT	GO:1902600~hydrogen ion transmembrane transport	16
GOTERM_BP_DIRECT	GO:0006626~protein targeting to mitochondrion	11
GOTERM_BP_DIRECT	GO:0032543~mitochondrial translation	11
GOTERM_BP_DIRECT	GO:0006521~regulation of cellular amino acid metabolic	13
GOTERM_BP_DIRECT	GO:0006457~protein folding	26
GOTERM_BP_DIRECT	GO:0006123~mitochondrial electron transport, cytochro	8
GOTERM_BP_DIRECT	GO:0006122~mitochondrial electron transport, ubiquino	7
GOTERM_BP_DIRECT	GO:0033209~tumor necrosis factor-mediated signaling p	19
GOTERM_BP_DIRECT	GO:0051436~negative regulation of ubiquitin-protein liga	14
GOTERM_BP_DIRECT	GO:0000302~response to reactive oxygen species	10
GOTERM_BP_DIRECT	GO:0051603~proteolysis involved in cellular protein cata	11
GOTERM_BP_DIRECT	GO:0051437~positive regulation of ubiquitin-protein liga	14
GOTERM_BP_DIRECT	GO:0050999~regulation of nitric-oxide synthase activity	8
GOTERM_BP_DIRECT	GO:0034975~protein folding in endoplasmic reticulum	6
GOTERM_BP_DIRECT	GO:0006183~GTP biosynthetic process	6
GOTERM_BP_DIRECT	GO:0031145~anaphase-promoting complex-dependent c	14
GOTERM_BP_DIRECT	GO:0030855~epithelial cell differentiation	13
GOTERM_BP_DIRECT	GO:0000413~protein peptidyl-prolyl isomerization	10
GOTERM_BP_DIRECT	GO:0043488~regulation of mRNA stability	16
GOTERM_BP_DIRECT	GO:0036500~ATF6-mediated unfolded protein response	5
GOTERM_BP_DIRECT	GO:0006099~tricarboxylic acid cycle	8
GOTERM_BP_DIRECT	GO:0034599~cellular response to oxidative stress	12
GOTERM_BP_DIRECT	GO:0038061~NIK/NF-kappaB signaling	12
GOTERM_BP_DIRECT	GO:0090090~negative regulation of canonical Wnt signal	21
GOTERM_BP_DIRECT	GO:0033617~mitochondrial respiratory chain complex IV	6
GOTERM_BP_DIRECT	GO:0006983~ER overload response	5
GOTERM_BP_DIRECT	GO:0006163~purine nucleotide metabolic process	5
GOTERM_BP_DIRECT	GO:0090263~positive regulation of canonical Wnt signali	17
GOTERM_BP_DIRECT	GO:0036498~IRE1-mediated unfolded protein response	11
GOTERM_BP_DIRECT	GO:0009060~aerobic respiration	8
GOTERM_BP_DIRECT	GO:0015949~nucleobase-containing small molecule inter	7
GOTERM_BP_DIRECT	GO:0043066~negative regulation of apoptotic process	43

%	PValue	Genes
3.386809	2.38E-23	MRPS15, MRPS16, MRPS36, MRPS14, MRPS11, MRPS34, MRPL19, MRPS12, MR[illegible]
2.941176	5.65E-23	NDUFB9, NDUFA13, NDUFB7, NDUFB10, NDUFA11, NDUFB11, NDUFB5, NDUFB[illegible]
2.58467	3.87E-22	NDUFB9, NDUFA13, NDUFB7, NDUFB10, NDUFA11, NDUFB11, NDUFB5, NDUFB[illegible]
3.208556	5.48E-21	MRPS15, MRPS16, MRPS36, MRPS14, MRPS11, MRPS34, MRPL19, MRPS12, MR[illegible]
3.743316	3.30E-08	YWHAE, LAD1, HSP90AB1, TACSTD2, HDLBP, TWF1, CAPG, ENO1, RTN4, CNN3, S[illegible]
3.386809	3.67E-07	MRPS15, SLC25A3, MRPS16, MRPS36, MRPS14, MRPS11, RPLP1, MRPL19, MRPS[illegible]
5.882353	1.26E-06	KDM5B, TECR, NDUFA10, ALKBH7, TXNDC17, CYP4Z1, SPR, PHYHD1, QSOX1, PH[illegible]
1.426025	2.01E-06	COX8A, CYB5A, COX7B, ATP6V1G1, COX7A2, UQCR11, UQCR10, COX6C, COX6A1[illegible]
0.980392	1.89E-05	TIMM8B, DNAJC19, FIS1, MTX1, MTX2, TIMM13, TIMM17A, TIMM17B, TRNT1, T[illegible]
0.980392	3.29E-05	MRPS15, MRPL51, MRPS16, MRPS11, MRPS12, MRPS34, MRPS18B, MRPS18A, M[illegible]
1.158645	3.40E-05	NQO1, PSMD14, PSMA7, OAZ3, PSMD8, PSMB6, PSMB7, PSMC5, PSMB4, PSMA[illegible]
2.317291	6.68E-05	LRPAP1, FKBP2, HSP90AB1, TXN, HSPBP1, HSP90B1, MLEC, SIL1, CCT5, CCT3, PD[illegible]
0.713012	1.02E-04	COX8A, COX7B, COX7A2L, COX5B, COX6C, COX6A1, COX5A, COX7C
0.623886	1.38E-04	UQCC3, UQCRQ, UQCRFS1, UQCR11, CYC1, UQCR10, UQCRC2
1.693405	2.09E-04	EDARADD, TNFRSF12A, PSMD14, KRT8, PSMA7, TXNDC17, PSMD8, PYCARD, PSM[illegible]
1.247772	2.53E-04	PSMD14, PSMA7, ANAPC11, PSMD8, PSMB6, PSMB7, PSMC5, PSMB4, PSMA4, P[illegible]
0.891266	3.80E-04	PRDX2, PRDX5, HYAL2, GSTP1, PRDX1, NUDT2, APOD, P4HB, TXN, SOD1
0.980392	4.41E-04	PSMB6, PSMB7, PSMB4, TINAGL1, PSMA4, PSMB5, HSPA5, PSMA2, CLPP, CTSF, [illegible]
1.247772	5.05E-04	PSMD14, PSMA7, ANAPC11, PSMD8, PSMB6, PSMB7, PSMC5, PSMB4, PSMA4, P[illegible]
0.713012	6.29E-04	GCHFR, LYPLA1, SPR, DDAH2, NOSTRIN, WASL, CALM2, PTS
0.534759	6.38E-04	PDIA3, HSPA5, CANX, EMC6, CALR, HSP90B1
0.534759	6.38E-04	NME6, IMPDH2, NME2, NME3, NME4, NME1
1.247772	7.39E-04	PSMD14, PSMA7, ANAPC11, PSMD8, PSMB6, PSMB7, PSMC5, PSMB4, PSMA4, P[illegible]
1.158645	8.03E-04	CBR1, ACADVL, EHF, ANXA4, SIX1, DNPH1, CNN3, VEGFA, LGALS3, MUC1, ELF3, I[illegible]
0.891266	8.17E-04	PTPA, FKBP2, PIN1, PPIH, PIN4, PPIG, FKBP4, PPIB, PPIA, PPIC
1.426025	1.12E-03	PSMD14, HSPB1, PSMA7, ZFP36L1, PSMD8, PSMB6, PSMB7, PSMC5, PSMB4, PSM[illegible]
0.445633	1.24E-03	XBP1, HSPA5, DDIT3, CALR, HSP90B1
0.713012	1.28E-03	FH, PDHA1, MDH2, IDH2, SUCLG1, SDHC, PDHB, SDHB
1.069519	1.28E-03	PRDX2, XBP1, SELENOS, STAU1, PARP1, MGMT, LRRK2, ZC3H12A, NME2, PYCR1,
1.069519	1.66E-03	PSMD8, PSMB6, PSMB7, PSMC5, PSMB4, PSMA4, PSMB5, PSMD14, PSMD4, PSM[illegible]
1.871658	1.68E-03	EGR1, WWTR1, PSMD14, CSNK1A1, IGFBP4, CTNND1, IGFBP2, PSMA7, SOX2, PS[illegible]
0.534759	1.86E-03	COA4, CHCHD5, COA3, COX17, COX14, COX20
0.445633	1.96E-03	SELENOS, HSPA5, DDIT3, CCDC47, TMCO1
0.445633	1.96E-03	GUK1, NME2, NME3, NME4, NME1
1.515152	2.01E-03	PSMD14, JUP, LRRK2, DIXDC1, PSMA7, PSMD8, PSMB6, PSMB7, PSMC5, PSMB4,
0.980392	2.34E-03	MYDGF, ACADVL, XBP1, HSPA5, SEC61G, LMNA, DNAJB11, SEC61B, SEC62, PDIA[illegible]
0.713012	2.84E-03	CHCHD5, SDHC, UQCR10, UQCRC2, COX6A1, NDUFV1, SDHB, COX20
0.623886	2.92E-03	GUK1, AK2, NME2, NME4, TXN, AK6, NME1
3.832442	3.21E-03	GSTP1, HSPB1, CIB1, HIGD1A, NOL3, HSPD1, HSP90B1, MYDGF, PRDX2, PRDX5, [illegible]

List Total	Pop Hits	Pop Total	Fold Enrichment	Bonferroni	Benjamini	FDR
1001	85	16792	7.50	7.93E-20	7.93E-20	7.88E-20
1001	63	16792	8.79	1.88E-19	9.40E-20	9.34E-20
1001	49	16792	9.93	1.29E-18	4.29E-19	4.27E-19
1001	86	16792	7.02	1.82E-17	4.56E-18	4.53E-18
1001	271	16792	2.60	1.10E-04	2.20E-05	2.18E-05
1001	253	16792	2.52	1.22E-03	2.04E-04	2.02E-04
1001	592	16792	1.87	4.17E-03	5.97E-04	5.93E-04
1001	61	16792	4.40	6.66E-03	8.35E-04	8.30E-04
1001	34	16792	5.43	6.10E-02	6.99E-03	6.95E-03
1001	36	16792	5.13	1.04E-01	1.03E-02	1.02E-02
1001	51	16792	4.28	1.07E-01	1.03E-02	1.02E-02
1001	180	16792	2.42	1.99E-01	1.85E-02	1.84E-02
1001	20	16792	6.71	2.87E-01	2.60E-02	2.58E-02
1001	15	16792	7.83	3.68E-01	3.28E-02	3.26E-02
1001	118	16792	2.70	5.01E-01	4.63E-02	4.60E-02
1001	71	16792	3.31	5.69E-01	5.26E-02	5.22E-02
1001	39	16792	4.30	7.18E-01	7.44E-02	7.39E-02
1001	48	16792	3.84	7.70E-01	8.15E-02	8.10E-02
1001	76	16792	3.09	8.13E-01	8.83E-02	8.78E-02
1001	26	16792	5.16	8.77E-01	9.65E-02	9.59E-02
1001	13	16792	7.74	8.80E-01	9.65E-02	9.59E-02
1001	13	16792	7.74	8.80E-01	9.65E-02	9.59E-02
1001	79	16792	2.97	9.15E-01	1.07E-01	1.06E-01
1001	70	16792	3.12	9.31E-01	1.09E-01	1.08E-01
1001	43	16792	3.90	9.34E-01	1.09E-01	1.08E-01
1001	103	16792	2.61	9.76E-01	1.43E-01	1.42E-01
1001	9	16792	9.32	9.84E-01	1.47E-01	1.46E-01
1001	29	16792	4.63	9.86E-01	1.47E-01	1.46E-01
1001	64	16792	3.15	9.86E-01	1.47E-01	1.46E-01
1001	66	16792	3.05	9.96E-01	1.80E-01	1.79E-01
1001	163	16792	2.16	9.96E-01	1.80E-01	1.79E-01
1001	16	16792	6.29	9.98E-01	1.91E-01	1.90E-01
1001	10	16792	8.39	9.99E-01	1.91E-01	1.90E-01
1001	10	16792	8.39	9.99E-01	1.91E-01	1.90E-01
1001	120	16792	2.38	9.99E-01	1.91E-01	1.90E-01
1001	59	16792	3.13	1.00E+00	2.17E-01	2.15E-01
1001	33	16792	4.07	1.00E+00	2.55E-01	2.54E-01
1001	25	16792	4.70	1.00E+00	2.56E-01	2.54E-01
1001	455	16792	1.59	1.00E+00	2.74E-01	2.72E-01

Category	Term	Count	%	PValue
KEGG_PATHWAY	hsa00190:Oxidative phosphorylation	54	4.81	4.45E-27
KEGG_PATHWAY	hsa05012:Parkinson's disease	52	4.63	1.28E-23
KEGG_PATHWAY	hsa05016:Huntington's disease	60	5.35	3.46E-23
KEGG_PATHWAY	hsa05010:Alzheimer's disease	53	4.72	1.10E-20
KEGG_PATHWAY	hsa04932:Non-alcoholic fatty liver disease (N/	50	4.46	1.57E-20
KEGG_PATHWAY	hsa01100:Metabolic pathways	156	13.90	1.63E-14
KEGG_PATHWAY	hsa01130:Biosynthesis of antibiotics	42	3.74	3.29E-09
KEGG_PATHWAY	hsa03060:Protein export	11	0.98	1.69E-06
KEGG_PATHWAY	hsa04260:Cardiac muscle contraction	18	1.60	1.71E-05
KEGG_PATHWAY	hsa04141:Protein processing in endoplasmic r	29	2.58	2.45E-05
KEGG_PATHWAY	hsa03010:Ribosome	25	2.23	3.25E-05
KEGG_PATHWAY	hsa03050:Proteasome	11	0.98	9.21E-04
KEGG_PATHWAY	hsa00020:Citrate cycle (TCA cycle)	8	0.71	4.60E-03
KEGG_PATHWAY	hsa01200:Carbon metabolism	17	1.52	6.78E-03
KEGG_PATHWAY	hsa00620:Pyruvate metabolism	9	0.80	6.80E-03
KEGG_PATHWAY	hsa00010:Glycolysis / Gluconeogenesis	12	1.07	7.84E-03
KEGG_PATHWAY	hsa00071:Fatty acid degradation	9	0.80	9.19E-03
KEGG_PATHWAY	hsa05110:Vibrio cholerae infection	10	0.89	1.11E-02
KEGG_PATHWAY	hsa00240:Pyrimidine metabolism	15	1.34	1.31E-02
KEGG_PATHWAY	hsa04146:Peroxisome	13	1.16	1.52E-02

Genes	List Total	Pop Hits	Pop Total	Fold Enrichment	Bonferroni	Benjamini
NDUFA13, COX7B, NDUFA11, NDUFA1	497	133	6879	5.62	1.19E-24	1.19E-24
NDUFA13, COX7B, NDUFA11, LRRK2, N	497	142	6879	5.07	3.43E-21	1.72E-21
NDUFA13, COX7B, NDUFA11, NDUFA1	497	192	6879	4.33	9.27E-21	3.09E-21
NDUFA13, COX7B, NDUFA11, NDUFA1	497	168	6879	4.37	2.95E-18	7.38E-19
NDUFA13, COX7B, NDUFA11, NDUFA1	497	151	6879	4.58	4.21E-18	8.43E-19
NDUFA13, NDUFA11, NDUFA10, ENO1	497	1219	6879	1.77	4.37E-12	7.27E-13
GPI, FH, OAT, ECHS1, SHMT2, AK2, UA	497	212	6879	2.74	8.82E-07	1.26E-07
SPCS2, SPCS1, SRP72, HSPA5, SEC61G,	497	23	6879	6.62	4.52E-04	5.65E-05
COX8A, COX7B, COX7A2, UQCR11, UQ	497	75	6879	3.32	0.00457996	5.10E-04
HSP90AB1, UBE2D4, RPN2, SAR1A, SA	497	169	6879	2.38	0.00654727	6.57E-04
MRPS15, MRPS16, MRPS14, MRPS11,	497	136	6879	2.54	0.00867695	7.92E-04
PSMD8, PSMB6, PSMB7, PSMC5, PSM	497	44	6879	3.46	0.21881094	0.0205687
FH, PDHA1, MDH2, IDH2, SUCLG1, SDH	497	30	6879	3.69	0.7092044	0.09479176
GPI, FH, ECHS1, PDHA1, TPI1, SHMT2,	497	113	6879	2.08	0.83836431	0.12143232
GRHPR, FH, PDHA1, ALDH2, MDH2, HA	497	40	6879	3.11	0.83921753	0.12143232
GPI, ALDH3B2, PDHA1, TPI1, ALDH2, A	497	67	6879	2.48	0.87884432	0.13139933
HADHB, ACADVL, ECHS1, ALDH2, ECI1,	497	42	6879	2.97	0.91580257	0.1448942
SLC12A2, ATP6V0B, ATP6V1G1, ATP6A	497	52	6879	2.66	0.94956886	0.16503115
DUT, CANT1, NME2, NME3, NUDT2, N	497	101	6879	2.06	0.97069278	0.18456717
PEX16, ECH1, IDH2, HSD17B4, ACSL3, S	497	83	6879	2.17	0.98355182	0.20381115

FDR
1.13E-24
1.63E-21
2.93E-21
6.99E-19
7.99E-19
6.89E-13
1.19E-07
5.35E-05
4.83E-04
6.23E-04
7.51E-04
0.01949421
0.08983995
0.11508884
0.11508884
0.12453518
0.1373251
0.15641012
0.1749256
0.1931643
